# Comparative genomics and metabolic profiling of the genus *Lysobacter*

**DOI:** 10.1186/s12864-015-2191-z

**Published:** 2015-11-23

**Authors:** Irene de Bruijn, Xu Cheng, Victor de Jager, Ruth Gómez Expósito, Jeramie Watrous, Nrupali Patel, Joeke Postma, Pieter C. Dorrestein, Donald Kobayashi, Jos M. Raaijmakers

**Affiliations:** Department of Microbial Ecology, Netherlands Institute of Ecology, P.O. Box 50, Wageningen, 6700 AB The Netherlands; Wageningen University and Research Centre, Laboratory of Phytopathology, P.O. Box 8025, Wageningen, 6700 EE The Netherlands; Departments of Pharmacology, Chemistry and Biochemistry; Center for Marine Biotechnology and Biomedicine, Scripps Institution of Oceanography; Skaggs School of Pharmacy and Pharmaceutical Sciences, University of California at San Diego, La Jolla, San Diego, USA; Department of Plant Biology & Pathology, Cook College, Rutgers, The State University of New Jersey, New Brunswick, NJ 08901-8520 USA; Wageningen University and Research Centre, Plant Research International, PO Box 16, Wageningen, 6700 AA The Netherlands

**Keywords:** Lysobacter, Microbial interactions, Comparative genomics, Mass spectrometry imaging, Nonribosomal peptide synthesis

## Abstract

**Background:**

*Lysobacter* species are Gram-negative bacteria widely distributed in soil, plant and freshwater habitats. *Lysobacter* owes its name to the lytic effects on other microorganisms. To better understand their ecology and interactions with other (micro)organisms, five *Lysobacter* strains representing the four species *L. enzymogenes, L. capsici*, *L. gummosus* and *L. antibioticus* were subjected to genomics and metabolomics analyses.

**Results:**

Comparative genomics revealed a diverse genome content among the *Lysobacter* species with a core genome of 2,891 and a pangenome of 10,028 coding sequences. Genes encoding type I, II, III, IV, V secretion systems and type IV pili were highly conserved in all five genomes, whereas type VI secretion systems were only found in *L. enzymogenes* and *L. gummosus*. Genes encoding components of the flagellar apparatus were absent in the two sequenced *L. antibioticus* strains*.* The genomes contained a large number of genes encoding extracellular enzymes including chitinases, glucanases and peptidases. Various nonribosomal peptide synthase (NRPS) and polyketide synthase (PKS) gene clusters encoding putative bioactive metabolites were identified but only few of these clusters were shared between the different species. Metabolic profiling by imaging mass spectrometry complemented, in part, the *in silico* genome analyses and allowed visualisation of the spatial distribution patterns of several secondary metabolites produced by or induced in *Lysobacter* species during interactions with the soil-borne fungus *Rhizoctonia solani*.

**Conclusions:**

Our work shows that mining the genomes of *Lysobacter* species in combination with metabolic profiling provides novel insights into the genomic and metabolic potential of this widely distributed but understudied and versatile bacterial genus.

**Electronic supplementary material:**

The online version of this article (doi:10.1186/s12864-015-2191-z) contains supplementary material, which is available to authorized users.

## Background

*Lysobacter* species are Gram-negative bacteria commonly found in diverse ecosystems, including soil, rhizosphere and freshwater habitats [1, 2]. *Lysobacter* cells are thin, gliding and mucilaginous on agar medium [1]. The genus *Lysobacter* belongs to the family Xanthomonadaceae and was first described in 1978 by Christensen and Cook (Christensen and Cook 1978) and initially included four species that were isolated from Canadian soil or lake water: *L. antibioticus*, *L. brunescens*, *L. enzymogenes*, and *L. gummosus*. Prior to the description of *Lysobacter* as a separate genus, isolates were often confused with the myxobacteria *Polyangium* and *Sorangium* [3] or misidentified as *Xanthomonas* and *Stenotrophomonas.* In the last decade, many more species have been identified, with the majority of isolates coming from soils of Asian origin [2]. Approximately 25 species of *Lysobacter* are now described (International Journal of Systematic and Evolutionary Microbiology, IJSEM) but only two incomplete genome sequences, consisting of multiple contigs [4, 5], are publically available.

*Lysobacter* is named after its lytic effects on many other (micro)organisms including fungi, oomycetes, nematodes, unicellular algae, Gram-negative and Gram-positive bacteria [1]. Originally, isolations were performed by enrichment with chitin or ground mushrooms, followed by plating on agar media containing yeast or bacterial cells as a nutrient source [3]. Their broad-spectrum lytic activity has been attributed to the production of extracellular enzymes like proteases and endopeptidases [6–9], glucanases [10, 11], lipases [12–14], chitinases [15, 16], secondary metabolites [17, 18] and other yet unknown bioactive compounds. Several metabolites produced by *Lysobacter* species, mostly *L. enzymogenes*, have attracted considerable interest for their activities against methicillin-resistant *Staphylococcus aureus* (MRSA) and vancomycin-resistant enterococci (VRE) [17]. These metabolites include tripropeptins, lysobactin (also known as katanosin B) and WAP-8294A [18–22]. Antifungal metabolites produced by *Lysobacter* species are the maltophilins and derivatives thereof, including dihydromaltophillin and xanthobaccin A [18, 23–25]. Other compounds discovered for *Lysobacter* include the antibacterial cephabacins [26, 27], lactivicin and myxin [1, 18]. Recently, a 2,5-diketopiperazine (cyclo(L-Pro-L-Tyr)) was identified in *L. capsici* AZ78 with strong activity against the oomycete plant pathogens *Phytophthora infestans* and *Plasmopara viticola* [28]. The majority of these compounds are encoded by nonribosomal peptide synthetase (NRPS) or polyketide synthase (PKS) genes [18]. For the antimicrobial activities of *Lysobacter* species other than *L. enzymogenes*, however, only few corresponding metabolites and their biosynthetic genes are known and many more bioactive compounds remain to be discovered. Hence, *Lysobacter* species are considered as a neglected resource for novel antibiotics [17].

To begin to unravel the genomic diversity and metabolic potential of the *Lysobacter* genus, we sequenced and closed the genomes of five *Lysobacter* strains representing four species: *L. enzymogenes, L. capsici*, *L. gummosus* and *L. antibioticus*. Comparative genomics was performed to refine the phylogenetic delineation of the *Lysobacter* genus and to identify the core and pan genomes. We then focused specifically on common and unique genes potentially involved in the secretion, regulation and biosynthesis of known and unknown bioactive compounds. Metabolic fingerprinting was performed by dry droplet colony mass spectrometry and matrix-assisted laser desorption/ionization (MALDI) mass spectrometry imaging [29] to complement the *in silico* genome analyses and to elucidate the spatial distribution patterns of these bioactive compounds in interactions between *Lysobacter* species and *Rhizoctonia solani*, an economically important fungal pathogen of multiple crops including wheat, rice and sugar beet.

## Methods

### Strains and culture conditions

*Lysobacter* strains were grown on R2A (Merck) medium at 20–25 °C, unless specified otherwise. All pre-cultures were performed in Luria-Burti (LB) or Tryptic Soy Broth (TSB) and incubated for 2–3 days at 25 °C. All fungi and oomycetes were pre-cultured on potato dextrose agar (PDA; Difco) at 20–25 °C. *Xanthomonas campestris* pv. *campestris* and *Pectobacterium atrosepticum* were pre-cultured in LB medium.

### Isolation of genomic DNA

Strains *L. antibioticus* 55, *L. capsici* 76 and *L. gummosus* 3.2.11 were cultured in TSB medium for 3 days at 25 °C, the cells were washed three times with 0.9 % NaCl and subsequently genomic DNA was extracted using the Master Pure kit from Epicentre according to manufacturer’s protocol, excluding the beat beating step.

### Genomic DNA sequencing

Genomic DNA of strains *L. antibioticus* 55, *L. capsici* 76 and *L. gummosus* 3.2.11 were sequenced using the Pacific Biosciences (PacBio) *RS* sequencing platform. From the genomic DNA, 20 Kb insert size libraries were prepared and size selected using Blue Pippin™ and sequenced using C3 in combination with P5 polymerase chemistry for 2 SMRT cells per genome with 180 min or longer movie time and stage start. The 20-kb continuous-long-read (CLR) data were de novo assembled using the PacBio hierarchical genome assembly process (HGAP3)/Quiver software package version 2.2.0 [30]. Annotation was performed by RAST [31] and CloVR-Microbe [32]. Further annotation was performed by InterProScan [33], KEGG [34] and the NR database. The genome sequences of *L. enzymogenes* C3 and *L. antibioticus* ATCC29479 were obtained from Kobayshi (personal communication) and *L. antibioticus* 13–6 and *L. capsici* AZ78 were downloaded from NCBI.

### Bioinformatic analysis

Whole genome phylogeny was performed using Gegenees [35]. This program fragments the genome sequences in 200 bp fragment using overlap of 100 bp and performs all-against-all BLASTn comparison. From the obtained matrix a phylogenetic tree was generated. Phylogenetic analyses were also performed by concatenated alignments of eight core housekeeping genes: *cys*, *dnaX*, *gly*, *recA*, *recN*, *rpoB*, *rpoD*, *uvrC* and separately, the 16S rRNA gene. Prior to alignment of the concatenated sequence, the sequence of each housekeeping gene was aligned separately and trimmed where necessary. The trees were generated using the best fitted model for Maximum Likelihood with 1000 bootstrap repetitions in MEGA [36]. The seven *Lysobacter* genomes were compared to each other using an all-against-all protein BLASTp [37] of all proteins with a minimum length of 20 residues followed by orthology prediction using orthAgogue with at least 40 % overlap and an inflation value of 1.5 [38]. The Markov cluster (MCL) algorithm [39] was used to extract the orthologous groups. The resulting orthologous groups were used to deduce the core-, variable- and pan-genome of the corresponding dataset. Core- and pan-genome size evolution graphs were generated by calculating the median core- and pan-genome size for each number of combinations in the given species set. The standard deviation is calculated for the number of species involved and plotted with the median core- or pan-genome size. Core- and pan-genome plots are also calculated based on the CDS number ordering of the genomes with the largest genome first. Syntheny analyses were performed using Progressive MAUVE [40]. Gene clusters encoding bioactive compounds were identified using BLASTp analysis of reference gene clusters, using E-value cut-off of 10^−5^ and a identity >60 % and a identity of >70 % in case of NRPS/PKS biosysnthesis clusters. NRPS/PKS gene clusters were also identified by AntiSmash analysis [41], subsequent substrate selection prediction by PKS/NPRS prediction [42], NRPSpredictor2 [43] and phylogenetic analyses. For the phylogenetic analyses, alignments of the adenylation and condensation domains were were made with CLUSTALX (version 1.81). Trees were inferred by Neighbour Joining in CLUSTALX using 1000 bootstrap replicates.

Glycoside hydrolase (GH) domain analysis of the CDSs encoding chitinases and glucanases was performed by first extracting the GH domain by PFAM analysis [44] of CDSs of reference strains described in the CAZy database [45]. The PFAM database did not contain the glycoside hydrolase GH23 domain, but the reference CDSs indicated as GH23 family in the CAZy database gave a significant hit with “SLT” domain. Therefore, those CDSs that contained a significant hit with “SLT” domain were considered as containing a GH23 domain. The GH domain sequences were used for BLASTp analysis against the *Lysobacter* genome sequences with and E-value cut-off of 10^−5^. Blast hits were subjected to PFAM analysis and only those that contained a significant hit with a GH domain were considered as CDSs containing a GH with chitinase of glucanase activity.

To identify CDSs encoding peptidases, the protein sequences of all five strains were blasted against the MEROPS database with default E-value cut-off of 10^−4^ [46]. Subsequently, the CDSs were grouped based on peptidase family and number of CDSs per family counted.

### Exoenzyme activity

The cells from pre-cultures were washed 3x with 0.9 % NaCl and the cell density was adjusted to OD600 of 1. Subsequently, 2–5 μl droplets were spotted on different media containing 1.5-2 % agar. For chitinase activity, R2A and 1/10^th^ strength TSB agar plates were used containing 0.2 % colloidal chitin prepared form crab shell chitin (Sigma) according to [47]. After incubation for 3–7 days at 25 °C, clearing zones surrounding the colonies could be observed. For glucanase activity, R2A medium containing 0.5 % laminarin was used. After growth for 3 days at 25 °C, the colonies were removed by washing with water and the medium was stained with 1 % congo red. After destaining, coloration of the medium was determined. R2A with and without laminarin was included as controls. For lipase activity, the strains were inoculated on R2A medium supplemented with 0.01 % (w/w) CaCl_2_ and 1 % (w/v) Tween 80 and after 3–5 days of incubation at 25 °C, plates were investigated for opaque halos surrounding the colonies, which indicates lipase activity [48]. For protease activity, a medium was prepared containing 15 g/l skimmed milk powder, 4 g/l blood agar base, 0.5 g/l yeast extract. After incubation for 3–7 days at 25 °C, clearing zones surrounding the colonies could be observed.

### Antimicrobial activity

Antimicrobial activity against fungal/oomycete pathogens was determined by spotting suspension of washed cells (see above) on R2A, TSA, PDA, 1/5^th^ strength PDA or 1/10^th^ strength TSA medium at the border of the plate and inoculating a plug of the fungus or oomycete in the middle. After incubation for 3–7 days at 25 °C, hyphal growth inhibition could be determined. Fungal isolates were grown in potato dextrose agar (PDA, Oxoid) or vegetable juice agar plates (vegetable juice (V8) solified with 1.5 % agar) until spore production at 25 °C. To enhance sporulation, *Cercospora* and *Stemphylium* were grown on V8 agar plates under 16 h photoperiod, and spores of *Verticillium* and *Aspergillus* were collected from mycelium and streaked on PDA plates. Fungal spores were collected as described in Trifonova et. al. (2009) with slight modifications. In brief, spores were released from the mycelium by adding 10 ml of 0.9 % NaCl and scratching the surface with a sterile spatula, collected, 10-fold diluted and added to the culture media (PDA, 1/5 PDA and R2A cooled at 48-55 °C at a final concentration of 5 % (v/v). Four 5 μl droplets of each bacterial suspension were spotted at the edges of 90 mm spore inoculated plates. Non spore-inoculated plates were used as control. Plates were incubated at 25 °C for one week and subsequently inhibitory halos were monitored. Antimicrobial activity against bacterial pathogens was determined by preparing R2A, PDA, TSA and 1/5^th^ strength PDA plates with an overlay of 1 % water agar cooled down to 50 °C to which washed cells of a culture of the bacterial pathogens was added. Subsequently, 2–5 μl washed cells from the *Lysobacter* strains were spotted on top of the plates. After incubation for 3–7 days at 25 °C, clearing zones surrounding the colonies could be observed.

Viability staining of *R. solani* AG2-2IIIB was performed comparable to [49] by inoculating small mycelial plugs (2 mm) in 1 ml 1/5 PDB in 24-wells plate (Nunc). The plates were incubated for 4 days at 25 °C, non-shaking. *Lysobacter* strains were grown in R2B for four days at 25 °C, 200 rpm and the cultures were centrifuged for 20 min at maximum speed and the supernatant was collected and filter-sterilised by 0.2 μm (Whatman) The mycelium of *R. solani* was washed twice with 0.9 % NaCl and 1 ml of *Lysobacter* supernatant was added. The samples were incubated for 1, 2, 4, or 6 h. For each timepoint, 100 uM 2,3-Bis-(2-Methoxy-4-Nitro-5-Sulfophenyl)-2H-Tetrazolium-5-Carboxanilide (XTT) + 60 uM menadione was added and after 1 h incubation 200 ul of each sample was transferred into 96-well plate (Nunc) and measured the absorbance at 490 nm.

### Dried droplet mass spectrometry

*Lysobacter* strains, *L. antibioticus* L08, *L. antibioticus* ATCC29479, *L. capsici* L14, *L. gummosus* L15, *L. enzymogenes* C3 and *L. enzymogenes* DCA were grown in 5 ml LB broth overnight at 25 °C. Five μl of a bacterial suspension with an optical density of 1.0 at 600 nm was spotted on R2A agar plates and incubated at 25 °C. After two days of incubation, a plug of 2-mm diameter of the bacterial colony was placed into 100 ul methanol and the sample was vortexed and centrifuged briefly. The supernatant was collected and 0.7 μl of supernatant was mixed 1:1 with saturated Universal MALDI matrix (1:1 mixture of 2,5-dihydroxybenzoic acid and α-cyano-4-hydroxy-cinnamic Acid, Sigma-Aldrich) and 1.0 μl was deposited onto the MALDI target and allowed to dry. The sample plates were subjected to MALDI-TOF mass spectrometry (Microflex from Bruker Daltonics, Billerica, MA, USA) for MS acquisition, and were run in positive reflector mode with a mass range of 0–4 kDa. The data of two technical replicates (two MALDI-TOF runs) of two biological replicates per strain was analysed using FlexAnalysis 3.3 (Bruker Daltonics) and ClinProTool 3.0 software (Bruker Daltonics).

### MALDI-IMS

Cultures were prepared as described above and 5 μl cell suspensions were spot-inoculated on thin R2A agar plates (1–1.5 mm thick; 10 ml in petridish with 9 cm diameter). After 2 days of incubation at 25 °C, regions of agar containing *Lysobacter* colony and surrounding area were cut and placed on top of a MALDI MSP 96 anchor plate (Bruker Daltonics, Billerica, MA, USA). A photograph was taken and a layer of Universal MALDI matrix (1:1 mixture of 2,5-dihydroxybenzoic acid and α-cyano-4-hydroxy-cinnamic Acid, Sigma-Aldrich) was applied to the sample using a 53 μm sieve. Samples were dried at 37 °C for a minimum of 6 h until they were completely dry and adhered to the MALDI plate. The sample plates were subjected to MALDI-TOF mass spectrometry (Autoflex from Bruker Daltonics, Billerica, MA, USA) for IMS acquisition, and were run in positive linear mode, with 500 μm laser intervals in XY and a mass range of 0–4 kDa. Data of two biological replicates per strain was analysed. To study the interaction with *R. solani*: after 2 days of incubation of the *Lysobacter* strains on thin agar plates, a mycelial plug of 2-mm diameter of *R.solani* was placed next to *Lysobacter* colony and incubated for another 2 days at 25 °C. The mycelial plug was removed and the agar region containing both the *Lysobacter* spp. and *R. solani* were cut and placed on top of a MALDI MSP 96 anchor plate and further processed as described above. Data of one sample per interaction was obtained. The data was analysed using FlexImaging 3.0 software (Bruker Daltonics).

## Results and discussion

### Genome sequencing

**Fig. 1:**
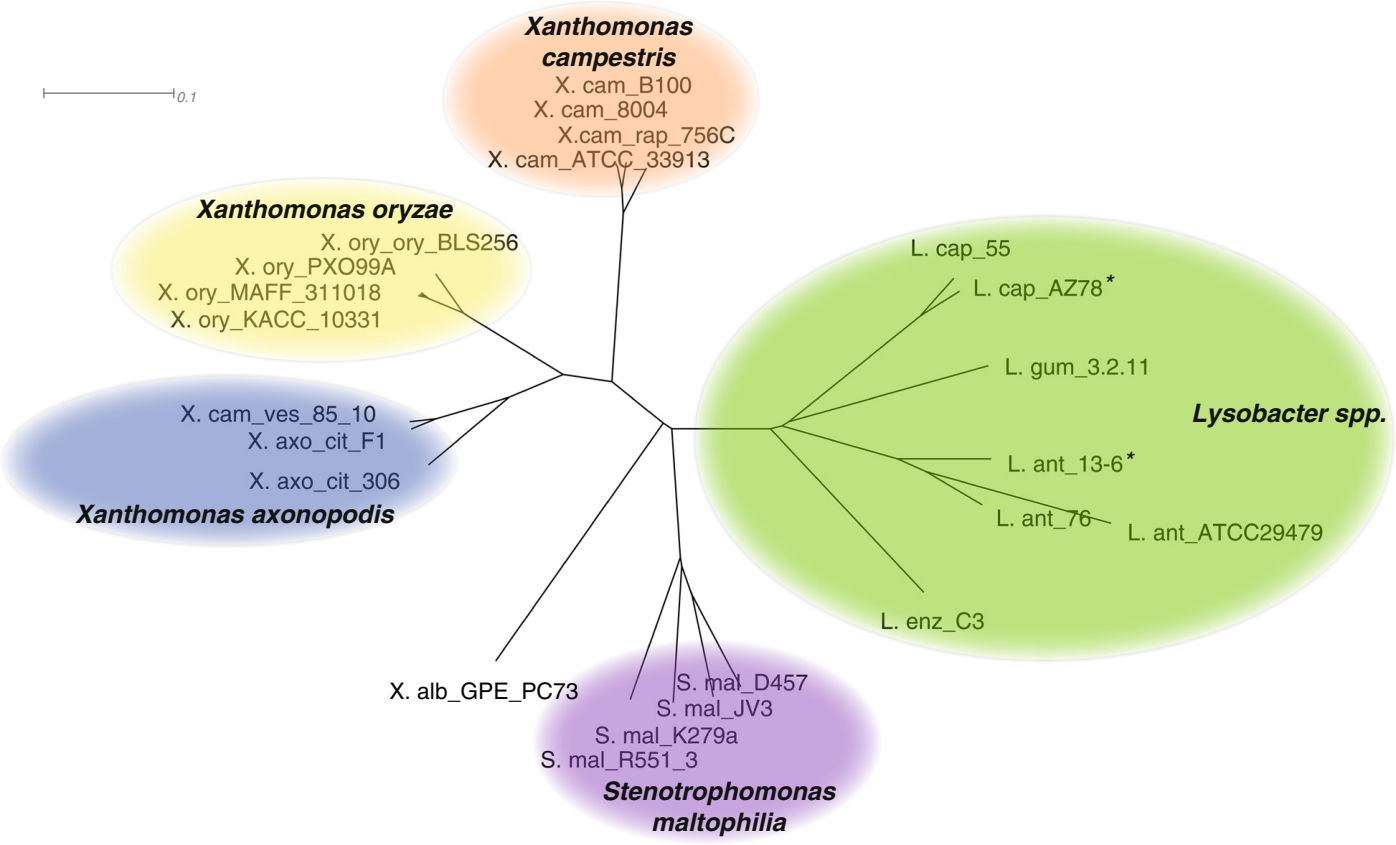
Whole genome phylogeny of *Lysobacter* species. The tree is based on the total nucleotide genome sequences using the Gegenees software. Asterisks indicate the previously published and publically available *Lysobacter* genome sequences. Genome sequences of *Stenotrophomonas maltophilia* and *Xanthomonas* were used as outgroups. S. mal: *Stenotrophomonas maltophilia*; X. alb: *Xanthomonas albilineans*; X. axo_cit_F1: *Xanthomonas axonoponis citrumelo* F1; X. axo_cit 306: *X. axonoponis citri 306*; X. cam: *Xanthomonas campestris*; X. cam_rap: *X. campestris raphani*; X.ory: *Xanthomonas oryzae* pv. *oryzae*; X.ory_ory: *X. oryzae* pv. *oryzicola*

*L. antibioticus* strain 76, *L. capsici* strain 55 and *L. gummosus* strain 3.2.11 (Table 1) were selected for PacBio genome sequencing of size-selected 20-kb-insert libraries. Subsequent *de novo* assembly resulted in closed genome sequences. Comparative genomics of these three genomes including two genomes provided by D. Kobayashi and the two recently deposited, partial *Lysobacter* genome sequences [4, 5], showed that the genome sizes ranged from 5.8–6.4 Mb with a GC content of 66–70 % (Table 2). The number of Coding DNA Sequences (CDSs) in the closed genomes ranged from 5,146 in *L. antibioticus* 76 to 5,686 in *L. capsici* 55 (Table 2). Whole genome phylogeny showed that the *Lysobacter* genomes were distinct from the *Stenotrophomonas* and *Xanthomonas* genomes (Fig. 1). The three *L. antibioticus* and the two *L. capsici* strains clustered closely together and more distant from the other *Lysobacter* species (Fig. 1). Phylogenetic analysis based on multi-locus sequence analysis (MLSA) and 16S rDNA showed similar results (Additional file 1: Figure S1). The *L. antibioticus* 13–6 genome sequence was not included for further analyses, since no predicted protein sequences were available [5].

**Table 1:** *Lysobacter* strains used for comparative genomics

Label	Strain	Isolation source with references	Genome sequence
			accession number and reference
*L. ant* ATCC29479	*L. antibioticu*s ATCC29479	soil Central Experimental Farm, Ontario, Canada [1]	CP013141; this study
*L. ant* 76	*L. antibioticus* 76	bulk soil, Zwaagdijk, The Netherlands [78, 79]	CP011129; this study
*L. ant* 13-6	*L. antibioticus* 13–6	rhizosphere Chinese cabbage [5]	JMTZ00000000; [5]
*L. cap* 55	*L. capsici* 55	bulk soil, Zwaagdijk, The Netherlands [79, 80]	CP011130; this study
*L. cap* AZ78	*L. capsici* AZ78	tobacco rhizosphere [4]	JAJA01000000; [4]
*L. enz* C3	*L. enzymogenes* C3	Kentucky bluegrass leave, Nebraska [81]	CP013140; this study
*L. gum* 3.2.11	*L. gummosus* 3.2.11	bulk soil, Pietersbierum, The Netherlands [79, 82]	CP011131; this study

**Table 2:** Assembly statisitics and features of the *Lysobacter* genome sequences

	*L. ant* ATCC29479	*L. ant* 76	*L. ant 13-6* ^*a*^	*L. cap* 55	*L. cap AZ78* ^*a*^	*L. gum* 3.2.11	*L. enz* C3
*Sequencing platform*	HiSeq 2000	PacBio P5C3	HiSeq 2500	PacBio P5C3	*Illumina GAIIx*	PacBio P5C3	HiSeq 2000
*Asssembly statistics*							
Genome size (Mb)	5.77	5.91	5.53	6.39	6.32	6.06	6.16
# scaffolds	1	1	-	1	-	1	1
# contigs	1	1	168	1	142	1	1
Number of Reads	-	106 778	11 524 134	114 536	7 512 266	112 495	-
N50 Read Length	-	15 230	60 432	14 389	139 986	14 221	-
Mean Read Length	-	11 242	-	10 633	-	10 363	-
GC%	67.0	66.8	67.1	66.6	66.4	66.5	69.8
*Genomic features*							
Coding DNA sequences (CDSs)	5178	5143	4056	5685	5448	5322	5547
# hypothetical proteins	1338	1832	-	2123	-	1845	2088
rRNA number	9	6	-	6	7	6	6
tRNA number	55	51	-	52	85	51	60

^*a*^ These genomes are publically available and consisting of multiple contigs. Statistics were taken from [4, 5]. - indicates unknown.

**Fig. 2:**
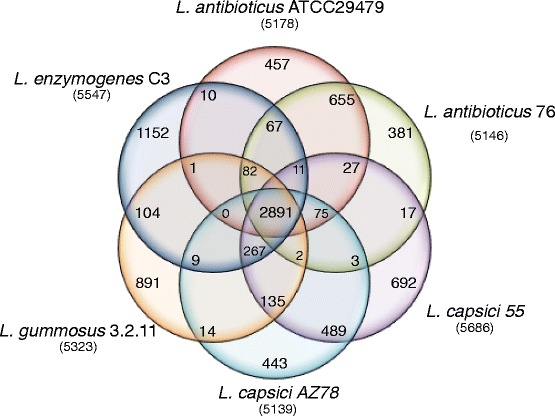
Genomic diversity of *Lysobacter*. The number of unique coding sequences (CDSs) shared by the *Lysobacter* strains representing the core genome is shown in the centre. The total number of CDSs in the analysis was 32019. Overlapping regions show the number of CDSs conserved only within the specified genomes. Numbers in non-overlapping portions of each oval show the number of CDSs unique to each strain. The total number of protein CDSs within each genome is listed below the strain name

All-against-all BLASTp analyses and subsequent orthology clustering of the predicted protein sequences of all five strains and *L. capsici* AZ78 [4] resulted in a pangenome of 10,028 orthologous groups. The core genome consisted of 2,891 CDSs, representing 54 % of the CDSs of each strain (Fig. 2, Additional file 1: Figure S2, Additional file 2: Table S1). This number is larger than the core genome of Xanthomonads (±30 % of the total genome [50]) but comparable to the highly diverse *Pseudomonas fluorescens* clade (±50 %, [51]). Based on the Kyoto Encyclopedia of Genes and Genomes (KEGG) annotation [34], the core genome consists, for a large part, of CDSs for enzymes, transporters and transcription factors (Additional file 1: Figure S3). For the two *L. antibioticus* strains 76 and ATCC29479 and the two *L. capsici* strains, approximately 90 % and 85 % of the CDSs were shared between the strains, indicating that within these species there is a high level of similarity. However, genome alignment of *L. antibioticus* ATCC29479 with *L. antibioticus* 76 showed many rearrangements between the two genomes (Additional file 1: Figure S4). CDSs unique for each strain ranged from 7.5 % for *L. antibioticus* 76 to 22.4 % for *L. enzymogenes* C3 (Additional file 2: Table S2). Approximately 13 % and 9 % of the CDSs were unique for the *L. antibioticus* and *L. capsici* species, respectively (Additional file 2: Table S3). Several of these unique CDSs gene clusters will be discussed in further detail below, focussing primarily on the gene clusters from the strains/species with closed genome sequences.

### Gene/gene clusters encoding bioactive compounds

**Fig. 3:**
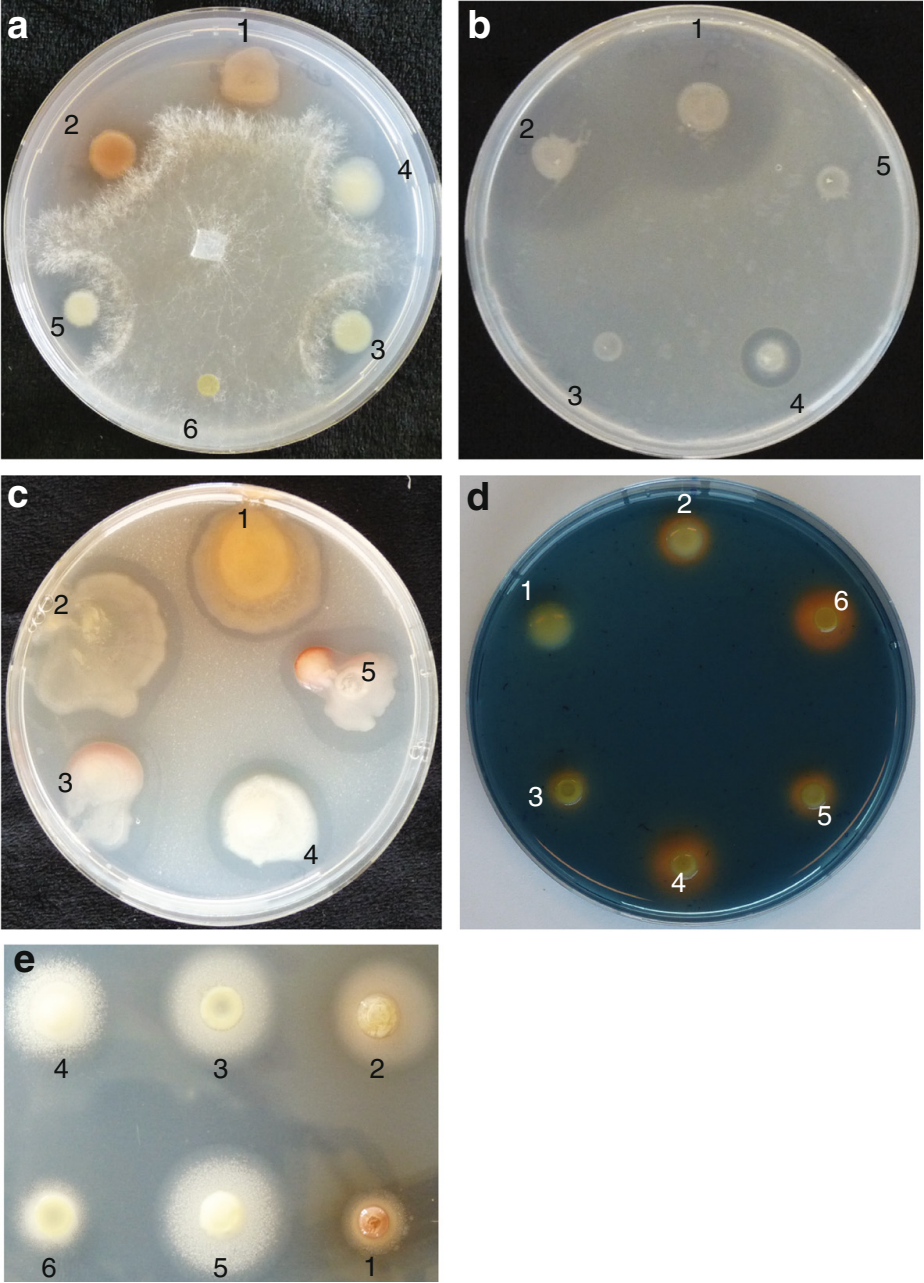
Antimicrobial and extracellular enzyme activity of *Lysobacter*. Antimicrobial activity against **a**) *Rhizoctonia solani* on R2A medium, and **b**) *Xanthomonas campestris* pv *campestris* (Xcc) on 1/5th strength Potato Dextrose Agar. The *Lysobacter* strains were spot inoculated on medium containing Xcc cells. **c** Chitinase activity on R2A medium supplemented with 0.2 % colloidal chitin. **d** siderophore production on CAS medium. When iron is chelated, the medium turns from blue to orange. **e** lipase activity on R2A medium supplemented with 0.01 % CaCl2 and 1 % Tween 80. Lipase activity is visible as a precipitation of calcium crystals surrounding the colony. 1: *L. antibioticus* ATCC29479; 2: *L. antibioticus* 76; 3: *L. capsici* 55; 4: *L. gummosus* 3.2.11; 5: *L. enzymogenes* C3; 6: *L. enzymogenes* DCA is a mutant of *L. enzymogenes* strain C3 with a Tn5 insertion in the gene encoding the global regulator Clp

The *Lysobacter* strains had a different activity spectrum against fungi, oomycetes and bacteria (Fig. 3; Additional file 3: Table S4), suggesting that they harbour or express different or multiple sets of genes coding for antimicrobial compounds. *Lysobacter* owes its name to the lytic activity against bacteria, fungi and algae. This activity has been partially attributed to the production of lytic enzymes such as chitinases, endopeptidases and proteases [1]. All *Lysobacter* strains showed chitinase activity (Fig. 3) and 1,3-β-glucanase activity (data not shown) and their genomes contained multiple CDSs encoding glycoside hydrolase domains conferring to chinase and glucanase activity (Additional file 3: Table S5-S8) according to the Carbohydrate-Active enZYmes (CAZy) database [52]. Almost all strains showed extracellular protease activity, except the *L. gummosus* strain under the condition tested (Additional file 2: Table S4) and 5.7-7.2 % of the total CDSs were predicted to encode peptidases with the majority belonging to the serine proteases followed by the metalloproteases (Additional file 1: Figure S5; Additional file 3: Table S9). Several of these peptidases are secreted in outer membrane vesicles, which are proposed to deliver the lytic enzymes at high concentrations at the site of interaction with other microbes [8, 9, 53–55]. Several peptidases were shown to have potential biofilm degrading activity in *L. gummosus* DSMZ6980 [56]. Only the α-lytic protease (accession number KF738068) had a high identity (99 %) in our strain *L. gummosus* 3.2.11 (Additional file 3: Table S10), indicating that this enzyme might be specific for the *L. gummosus* lineage.

In literature, several antibiotics and their gene clusters have been described for *Lysobacter* species (Additional file 3: Table S11). In our strains, several phenazine-related genes were predicted only for the *L. antibioticus* strains (Table 3). The organization of these putative phenazine biosynthesis genes is different from those in *Pseudomonas aeruginosa* (Additional file 1: Figure S6) and in other bacterial genera [57].

**Table 3:** Selection of genes or gene clusters putatively encoding bioactive metabolites by *Lysobacter* species. The geneID numbers are indicated and for a gene cluster the first gene and the last gene of the cluster is indicated separated by a hyphen

		*L. ant* ATCC29479	*L. ant* 76	*L. cap* 55	*L. gum* 3.2.11	*L. enz* C3
*Exoenzymes*						
Chitinases						
	*chiA*	LA29479_0768	LA76x_1507	LC55x_1254	LG3211_1156	LEC3_1126
		LA29479_4426	LA76x_0490	LC55x_4660	LG3211_1152	
Glucanase						
	*gluA*	-	*-*	LC55x_4859	LG3211_2492	LEC3_2518
	*gluB*	LA29479_937	LA76x_3779	LC55x_3780	LG3211_1435	LEC3_1403
	*gluC*	-	*-*	LC55x_1557	*-*	LEC3_0490
Endopeptidase						
	*alpA*	LA29479_4711	L76x_3467	LC55x_3409	LG3211_1771	LEC3_1730
	*alpB*	-	-	LC55x_3406	-	-
*Antibiotics*						
HSAF/dihydromaltophilin		-		LC55x_2005	LG3211_3209	LEC3_3211-3221
lysobactin		-	-	-	LG3211_2475-76	-
WAP-8294A2		-	-	LC55x_2722-23	-	LEC3_3675-76
phenazine		LA29479_2362-68	LA76x_3329-34	-	-	-
*Number of genes encoding other putative antimicrobial compounds*						
# bacteriocins		2	4	5	2	2
# lantipeptides		2	2	4	2	5
# terpene		-	-	-	1	-
# siderophore		-	-	-	1	-
# other		1	1	-	1	-
# unknown NRPS clusters		6	7	3	3	9

Note: *L. antibioticus* strains contain only a partial gene cluster encoding phenazine

Many of the bioactive compounds that are produced by *Lysobacter* spp. are encoded by NRPS/PKS genes [18]. Several of the known NRPS/PKS gene clusters are present in either one or more of the *Lysobacter* genomes, including those encoding dihydromaltophilin, lysobactin and WAP-8294A biosynthesis (Table 3). The dihydromaltophilin biosynthesis cluster is present in *L. capsici*, *L. gummosus* and *L. enzymogenes,* but not in the two *L. antibioticus* strains. Recently, a partial PKS gene cluster was identified in *L. capsici* AZ78 [28], which had 85 % identity with the predicted proteins encoding Heat-Stable Antifungal Factor (HSAF) in *L. enzymogenes* C3 (LEC3_3215) and 100 % and 91 % identity to the predicted proteins that are HSAF-encoding homologs in our *L. capsici* 55 (LA55_2005) and *L. gummosus* 3.2.11 (LG3211_3209), respectively. The lysobactin biosynthesis cluster is present only in *L. gummosus* 3.2.11. The WAP-8294A biosynthesis cluster is present in *L. capsici* 76 and *L. enzymogenes* C3. The two *L. antibioticus* strains have an NRPS gene cluster which seems partly similar to the WAP-8294A gene cluster of *L. enzymogenes* OH11 [22], but these gene clusters most likely encode a novel compound based on *in silico* structure prediction [42, 58] (Additional file 1: Figure S7). Subsequent Liquid Chromatography- tandem Mass Spectrometry (LC-MS/MS) and Nuclear Magnetic Resonance (NMR) analyses will be required for further structural identification of the metabolites, as well as random or site-directed mutagenesis to identify the corresponding genes. Multiple attempts and approaches to generate mutants, however, have not been successful for the differerent *Lysobacter* species, except for *L. enzymogenes* [15, 59]. Cloning and heterologous expression of several of the identified gene clusters will be an alternative strategy to explore in future experiments to resolve the structures and functions of the encoded metabolites.

Siderophores play an important role in iron acquisition and in competition with other microorganisms. Many siderophores are encoded by NRPS gene clusters. In the *Lysobacter* genomes, however, gene clusters encoding pyoverdin, pyochelin, pseudomonin, yersiniabactin, enterobactin, aerobactin, achromobactin, vibroferrin, staphyloferrin, rhizobactin and Xss were absent or only partially present (Additional file 3: Table S12). Even though iron availability did have an effect on antibacterial activity, *L. capsici* PG4 did not produce siderophores on CAS medium [60], indicating that our *Lysobacter* strains potentially do not possess the siderophores commonly found in other bacterial genera. However, all our strains, except *L. antibioticus* ATCC29479, exhibit iron-chelating capacity on CAS medium (Fig. 3).

Besides the NRPS/PKS gene clusters encoding known antibiotics, several unknown NRPS gene clusters were identified in the *Lysobacter* genomes. These gene clusters could be a new resource for novel antibiotics (Additional file 3: Table S13) but in-depth chemical and functional analyses are required to further investigate this.

### Gene/gene clusters involved in bacterial movement

In *Lysobacter* species, gliding motility is a common form of movement while flagellar motility is not. No flagellar genes were identified in the genomes of either *L. antibioticus* strain (Additional file 3: Table S14). However, components of the flagellar apparatus were found in *L. enzymogenes* C3; the 22 genes coding for a flagellar apparatus (including basal body, hook and regulatory genes) are also conserved in the genomes of *L. capsici* 55 and *L. gummosus* 3.2.11 (Additional file 3: Table S14). The flagellar gene clusters in the genomes of *L. enzymogenes* C3 and *L. gummosus* 3.2.11, but not of *L. capsici* 55, contained an additional gene encoding a transcription factor TCP domain protein downstream of the Sigma-70 regulatory factor gene. The presence of flagellar biosynthesis genes that appear non-functional within bacterial genomes has been observed previously [61] and is suggestive of evolved mechanisms for evasion of host detection as flagellar filament proteins serve as antigenic triggers of host immune responses [62, 63].

The type IV pilus genes were found in 5 different clusters in all five *Lysobacter* genomes and the genetic organization is mostly conserved (Additional file 3: Table S14). *L. capsici* 55, *L. gummosus* 3.2.11 and *L. enzymogenes* C3 contain duplicate regions of cluster IV consisting of *pilE,Y1,X,W,V* and pre-pilin genes.

### Gene/gene clusters involved in regulation and secretion

**Fig. 4:**
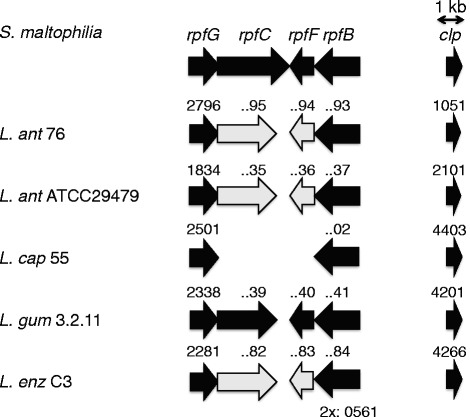
*Lysobacter* genes encoding the diffusible signal factor (DSF)-dependent system or the CRP-like protein Clp. The gene clusters were identified by BLASTp analysis using the reference gene cluster from *Stenotrophomonas maltophilia*. Black arrows indicate the CDSs with an identity >60 %; the grey arrows indicate the CDSs with an identity >40-60 %

Comparable to other Xanthomonads, the production of several bioactive compounds in *Lysobacter* have been shown to be regulated by intercellular signalling or quorum sensing mediated by the diffusible signal factor (DSF)-dependent system and Clp (cyclic AMP receptor (CRP)-like protein) regulator [59, 64–69]. Most components of the DSF-dependent system are present in the *Lysobacter* genomes, although some components had a lower identity to the DSF system described in *Stenotrophomonas* (Fig. 4). In *L. capsici* 55, the *rpfC* and *rpfF* genes were absent (Fig. 4). The Clp protein was present in all genomes with 99 % identity (Fig. 4), indicating that the Clp regulator is highly conserved among *Lysobacter* species.

We observed that the cell-free supernatant of the *Lysobacter* strains could inhibit viability of *R. solani* hyphae (Additional file 1: Figure S8), indicating that several bioactive compounds are secreted. *L. antibioticus* 76 supernant does not reduce cell viability, indicating that its bioactive compounds are cell-bound or not produced in liquid medium. Multiple secretion systems have been identified in Gram-negative bacteria [70], and most are represented in the *Lysobacter* genomes. All strains contain a number of genes associated with Type I (ABC transport) and Type V (autotransport) systems (data not shown). All strains investigated here also contain full complements of genes encoding Type II secretion (T2S) systems involving both the general secretory pathway (GSP) and the twin-arginine translocation (TAT) pathway (Additional file 2: Table S15). GSP consists of one gene cluster consisting of the genes *gspD-N*, and *yajC* and *sec*A,B,D,E,F, and Y genes dispersed throughout the genome. The TAT pathway genes, consisting of *tatA/E*, *tatB*, *tatC* and an RDD family protein predicted as a transmembrane protein associated with transport, were organized in an operon structure. Absent from the gene cluster is the exonuclease gene *tatD*, which is not required for pathway function [71]. However, two *tatD* gene homologues were located distally from the TAT gene cluster within the genomes of all strains.

**Fig. 5:**
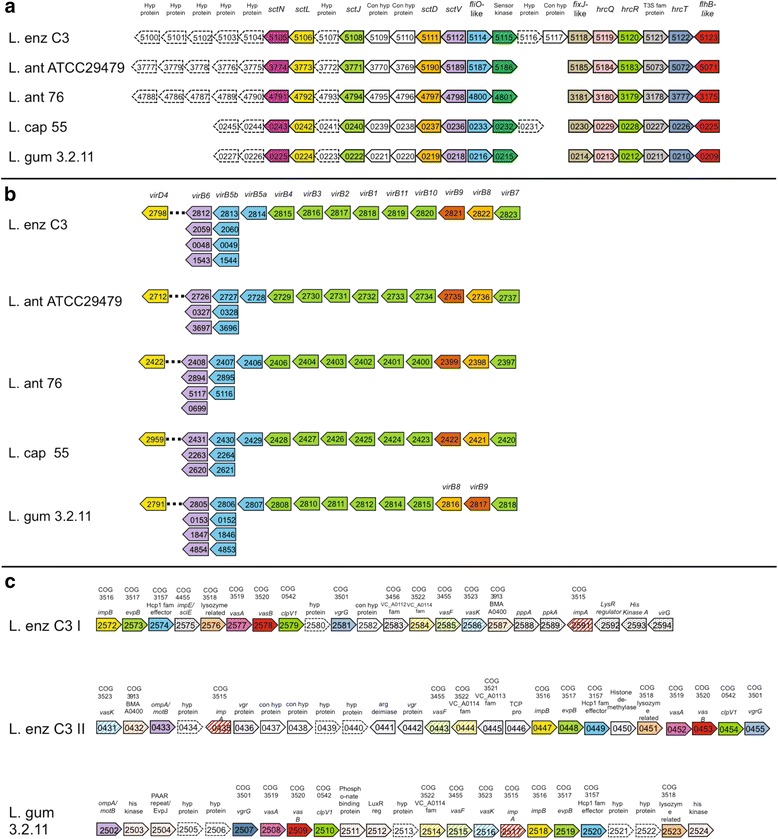
Genetic organization of Type III, Type IV and Type VI secretion pathways in the *Lysobacter* genomes. Arrows represent relative position and transcriptional direction for each gene. Gene/COG calls are indicated above the arrows. Similar coloured boxes between strains represent homologues and numbers within arrows represent GeneIDs. Gene sizes are not drawn to scale. Coloured boxes represent known core type III secretion homologues; closed boxes represent conserved hypothetical proteins; dashed boxes represent non-homologous hypothetical proteins (between strains). **a** Type III secretion. **b** Type IV secretion; *virB5a* and *virB5b* represent duplicate genes. Arrows below *virB6* and *virB5b* represent duplicate gene pairs located throughout the genomes. The dashed line between *virD4* and *virB6* indicates that genes are distally located. **c** Type VI secretion with each cluster represented as a contiguous gene set as indicated by gene identification number. Two gene clusters are identified in *L. enzymogenes* clusters and one in *L. gummosus* 3.2.11. Stippled boxes represent type VI secretion genes unique to each cluster

The genetic organization of the core genes encoding for Type III secretion (T3S) are highly conserved in all five *Lysobacter* strains (Fig. 5a). Conspicuously absent within the T3S pathways for all *Lysobacter* strains is a gene encoding the T3S secretin, a protein necessary for pore formation and transport across the outer membrane and essential for T3S apparatus function [72, 73]. No effector-encoding genes (related to those characterized in animal and plant pathogens) were identified. However, the presence of hypothetical proteins embedded in the core T3S genes suggests their putative effector function. All five strains possess only two secretin homologues, those belonging to T2S and Type IV Pilus (Additional file 3: Table S14). Also the *virD4 - virB1-11* required for Type IV secretion (T4S) are present in conserved genetic organization in all *Lysobacter* genomes with *virD4* located distally from the *virB1-11* gene complex; only the order of *virB8* and *virB9* in *L. gummosus* 3.2.11 was reversed. There are two copies of *virB5* (designated virB5a and b) within the *virB* gene cluster, as well as multiple predicted *virB5-6* gene pairs distributed throughout the genomes of all three strains (Fig. 5b). In contrast to the T3S and T4S pathways, genes encoding for T6S were found only in *L. enzymogenes* strain C3 and *L. gummosus* strain 3.2.11 and with different organization (Fig. 5C). *L. enzymogenes* harbors two distinct Type VI secretion (T6S) pathways encoded by genes located in separate clusters, but only one cluster consisting of 13 genes was identified in *L. gummosus* 3.2.11. The lack of conservation suggests that T6S represents a more recent acquisition among and within *Lysobacter* species.

Taken together, the presence of T3S, T4S, T6S, the flagellar components and type IV pilus suggests that *Lysobacter* species can establish pathogenic interactions with microbial eukaryotic hosts like plant pathogenic fungi (Kobayashi, personal communication).

### Metabolic profiling

#### Live colony MALDI imaging

**Fig. 6:**
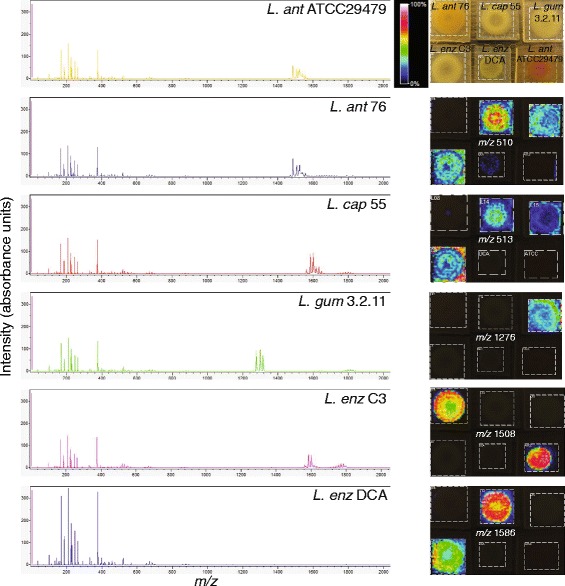
MALDI imaging mass spectrometry (IMS) of *Lysobacter* species. At the top right, the positions of the *Lysobacter* strains grown on R2A medium on the MALDI slide are shown. *Lysobacter enzymogenes* DCA is a mutant of *L. enzymogenes* strain C3 with a Tn5 insertion in the gene encoding the global regulator Clp. The mass spectra are shown on the left and the images at specific m/z values are shown on the right. The colour gradient bar shows the % intensity based on absorbance units after normalisation to the total ion count

Using MALDI-Imaging Mass Spectrometry (MALDI-IMS), the spatial distribution of extracellular metabolites produced by *Lysobacter* colonies was investigated. Several peaks ranging from 0–2000 mass-to-charge ratios (*m/z*) were detected (Fig. 6). Similar masses were detected in methanol extracts of plate cultures (Additional file 1: Figure S9-S10; Additional file 4). Most peaks with *m/z* 0–350 were considered as background since they were present in the matrix and R2A medium control (Additional file 1: Figure S10). The two *L. antibioticus* strains appear to produce similar compounds (Fig. 6). The other strains show different metabolic profiles, although *L. capsici* 55 and *L. enzymogenes* C3 have specific peaks in common (Fig. 6, Additional file 1: Figure S9-S10). In the metabolic profile of the *clp* mutant of *L. enzymogenes* C3, designated mutant strain DCA, most of the compounds produced by wild type strain C3 were absent (Additional file 1: Figure S9-10), confirming that the Clp transcription factor is indeed required for the production of several of these extracellular compounds. The *m/z* values described previously [21, 22, 74] for the dihydromaltophilin, lysobactin, and WAP-8294A2 were 513, 1276, 1562, respectively. The mass spectra show that a compound with an *m/z* value similar to dihydromaltophilin (*m/z* 513) is present in *L. capsici* 55, *L. gummosus* 3.2.11 and *L. enzymogenes* C3 (Additional file 1: Figure S10). This compound was secreted by *L. enzymogenes* C3, but was mostly retained in the colony of *L. capsici* 55 (Fig. 6). A putative structural analogue of dihydromaltophilin with *m/z* 510 was detected at high intensity in *L. capsici* 55 (Fig. 6).

A compound with a similar *m/z* value to lysobactin (*m/z* 1276) is only found in *L. gummosus* 3.2.11 and detected inside and surrounding the colony indicating that this compound is secreted into the medium. Multiple peaks were detected with slightly different *m/z* values (Fig. 6, Additional file 1: Figure S9-S10). It is well-known that the adenylation domains in the NRPS proteins encoding for these types of compounds can have relaxed substrate selectivity [75], leading to the biosynthesis of structural analogues. For *L. capsici* 55 and *L. enzymogenes* C3, a compound with a *m/z* value similar to WAP-8294A2 (*m/z 1562*) is present (Additional file 1: Figure S9-S10), but hardly detected with MALDI-IMS (data not shown). The putative structural analogue with *m/z* 1586 was readily detected inside the colonies of *L. capsici* 55 and *L. enzymogenes* C3 (Fig. 6).

Collectively, the metabolomics data obtained by MALDI matched well with the gene clusters identified in the genomes of these five strains (Table 3). For both *L. antibioticus* strains, a gene cluster partly similar to WAP-8294A2 was identified, but with 11 instead of 12 NRPS modules (Additional file 1: Figure S7). The mass spectra of these strains show peaks with a different *m/z* value (*m/z* 1508), which potentially could correspond to the 11 module gene cluster. This unknown compound was only detected inside the colonies of *L. antibioticus*.

#### *Lysobacter*-fungus interactions

**Fig. 7:**
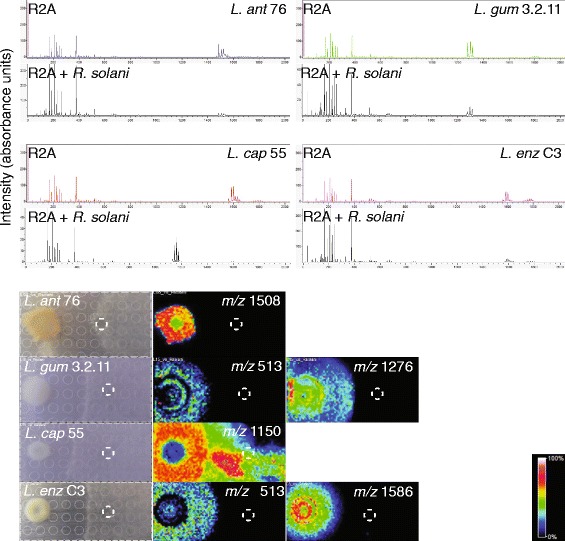
MALDI imaging mass spectrometry of *Lysobacter*-fungus interactions. At the top, the mass spectra of the *Lysobacter* strains grown on R2A medium in absence and presence of the fungus *Rhizoctonia solani* are shown. At the bottom the images of metabolites with specific m/z values are shown. The colour gradient bar shows the % intensity based on aborbance units after normalisation to the total ion count. The dashed circle depicts the point where the fungus *R. solani* was inoculated

All strains inhibited hyphal growth of the soil-borne, plant pathogenic fungus *R. solani* (Fig. 3). In interaction with *R. solani*, we observed that the dihydromaltophilin-like compound (*m/z* 513) was mainly secreted by *L. gummosus* 3.2.11 and *L. enzymogenes* C3 and diffused radially from the bacterial colony (Fig. 7). Since the diffusion area of this compound does not match well with the inhibition zone, it is doubtful if this compound is the main contributor to hyphal growth inhibition of *R. solani*. The lysobactin-like compound (*m/z* 1276) was closely surrounding the colony and is diffusing radially in the medium (Fig. 7). Since its distribution matches better with the hyphal inhibition zone, this compound could be a major contributor to the antifungal activity of *L. capsici 55*. For *L. antibioticus* 76, the unknown compound with *m/z* 1508 stays localised within the colony and most likely is not responsible for the growth inhibition (Fig 7). The compounds with *m/z* 1562–1586 similar to WAP-8294A and derivatives thereof were detected at high levels within and closely surrounding the colony of *L. enzymogenes* C3.

Strikingly, in *L. capsici* 55 these compounds were not detected anymore in interaction with *R. solani*. Instead, a group of compounds with *m/z* values ranging from 1135–1181 was detected (Fig. 7). The compound with a mass of *m/z* 1150 was detected at high concentrations in the inhibition zone, diffusing even underneath the *R. solani* mycelial mat. This suggests that this compound is involved in the *R. solani* inhibition. *R. solani* by itself did not produce compounds with masses of *m/z* 1135–1181 (Additional file 1: Figure S11), suggesting that the production of these compounds by *L. capsici* 55 is induced upon interaction with the fungus. We identified an NRPS/PKS gene cluster that is unique to *L. capsici* 55 (Additional file 1: Figure S12), but based on the number of NRPS modules this gene cluster is most likely not encoding these compounds.

Conclusions Collectively, these results showed that microbial interactions can induce or suppress the expression of cryptic gene clusters and can be used as a tool to trigger cryptic bioactive metabolites [76, 77]. Several other *Lysobacter* metabolites are also interesting for further analysis, including those with *m/z* ranging from 1720–1840. Also several novel NRPS gene clusters identified in the genome sequences (Additional file 2: Table S13) are worth to look into for their ecological functions. In conclusion, mining the genomes of *Lysobacter* species in combination with metabolic profiling provides novel insights into the genomic and metabolic potential of this widely distributed, versatile but understudied bacterial genus.

### Availability of supporting data

The genome sequences are available at the National Center for Biotechnology Information (NCBI) and the accession numbers are mentioned in Table 1. The mass spectrometry imaging data is available on http://hdl.handle.net/10411/20585. All other supporting data are included as additional files.

## Additional files

Additional file 1: Figures S1 to S12. (PDF 2839 kb) Additional file 2: Tables S1 to S3. (PDF 1962 kb) Additional file 3: Tables S4 to S15. (PDF 877 kb) Additional file 4: Mass lists of the dried droplet mass spectrometry analyses. (XLSX 610 kb)
