# Supplementary material for: Comparative genomics and metabolic profiling of the genus Lysobacter
Source: BMC Genomics. 2015 Nov 23;16:991. doi: 10.1186/s12864-015-2191-z (PMC4657364; doi:10.1186/s12864-015-2191-z)
Supplement: Additional file 1: Figures S1 to S12. — (PDF 2839 kb) [file 12864_2015_2191_MOESM1_ESM.pdf]

Supplementary Figures S1-S6

Comparative genomics and metabolic profiling of the genus *Lysobacter*

Irene de Bruijn*, Xu Cheng, Victor de Jager, Ruth Gómez Expósito, Jeramie Watrous, Nrupali Patel, Joeke Postma, Pieter C. Dorrestein, Donald Kobayashi and Jos M. Raaijmakers.

*Correspondence: **Dr. Irene de Bruijn: i.debruijn@nioo.knaw.nl**

Figure S1: Phylogenetic trees showing the relationship of *Lysobacter, Stenotrophomonas* and *Xanthomonas* spp.

Figure S2: Core-pan genome plot of *Lysobacter* strains.

Figure S3: Summary of predicted gene product function of the *Lysobacter* unique core genome (2.891 CDS) using KEGG gene ontology terms.

Figure S4: Whole genome alignment of the *Lysobacter antibioticus* strains.

Figure S5: Percentage of number of CDSs encoding peptidases in the *Lysobacter antibioticus 76* genome sequence by BLASTp analysis in the MEROPS database.

Figure S6: Gene cluster in *Lysobacter antibioticus* strains with similarity to phenazine biosynthesis cluster in *Pseudomonas aeruginosa* PA01.

Figure S7: Gene clusters in *Lysobacter* genome sequences potentially encoding for WAP8294A2 and WAP8294A2-like biosynthesis.

Figure S8: Effect of cell-free supernatant of the *Lysobacter* strains on XTT-formazan production by *Rhizoctonia solani* as a measure of fungal cell viability.

Figure S9: Heat plot of the MALDI mass spectra of metabolites produced by *Lysobacter* species.

Figure S10: Matrix-assisted laser desorption/ionization (MALDI) mass spectra of a dried droplet of a methanol extract of *Lysobacter* strains grown on R2A medium.

Figure S11: Matrix-assisted laser desorption/ionization (MALDI) imaging mass spectrometry (IMS) of *Rhizoctonia solani* incubated on R2A medium.

Figure S12: Putative NRPS/PKS gene cluster unique to *Lysobacter capsici* 55.

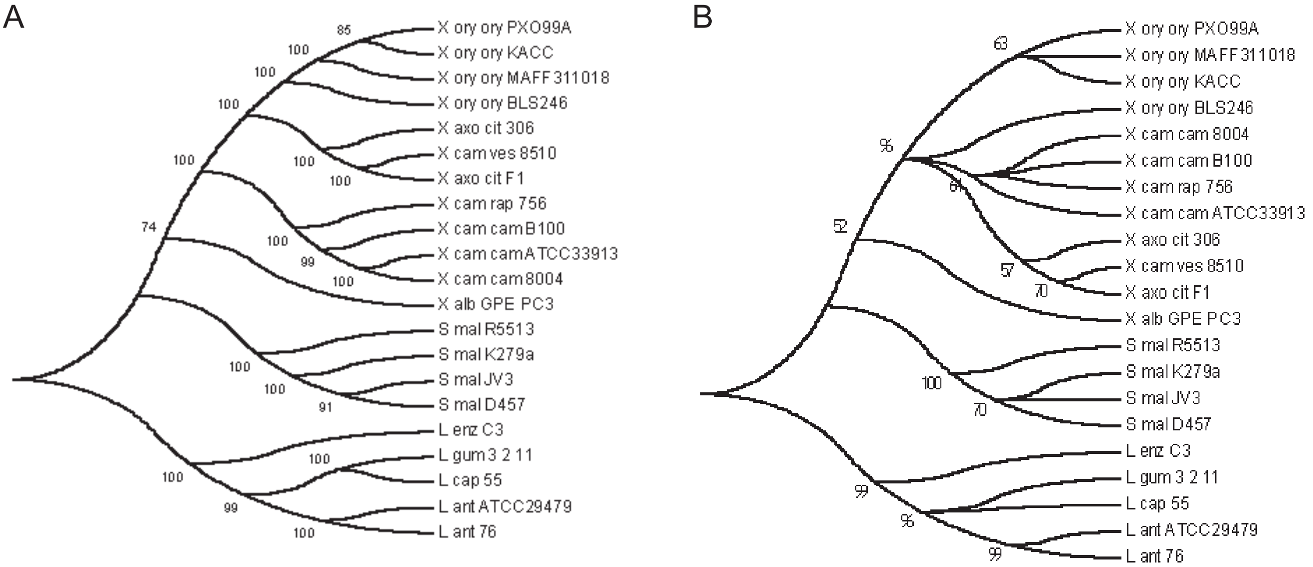

Figure S1. Phylogenetic trees showing the relationship of *Lysobacter, Stenotrophomonas* and *Xanthomonas* spp. The trees are based on A) concatenated alignments of eight core housekeeping genes: *cys*, *dnaX*, *gly*, *recA*, *recN*, *rpoB*, *rpoD*, *uvrC* and B) 16S rRNA gene. The trees were generated using the best fitted model for Maximum Likelihood with 1000 bootstrap repetitions in MEGA. The interior node values of the tree represent the percentage of bootstrap support. L. ant: *Lysobacter antibioticus*; L. cap: *Lysobacter capsici*; L. gum: *Lysobacter gummosus*; L. enz: *Lysobacter enzymogenes* C3; S. mal: *Stenotrophomonas maltophilia*; X. alb: *Xanthomonas albilineans*; X. axo cit F1: *Xanthomonas axonoponis citrumelo* F1; X. axo_cit 306: *X. axonoponis citri 306*; X. cam cam: *Xanthomonas campestris* pv *campestris*; X. cam rap: *X. campestris raphani*; X.ory: *Xanthomonas oryzae* pv. *oryzae*; X.ory_ory: *X. oryzae* pv. *oryzicola*.

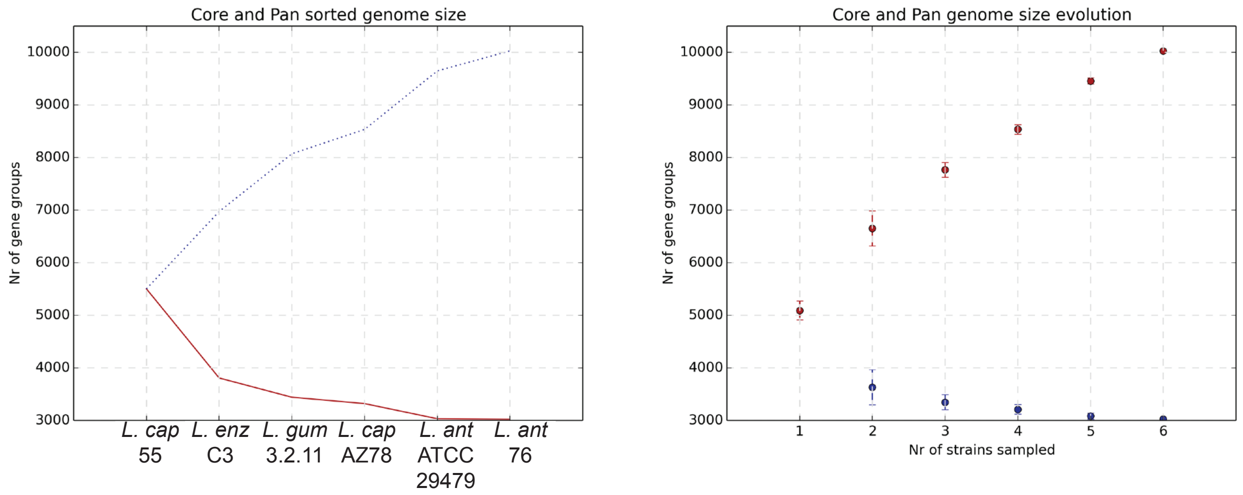

Figure S2. Core-pan genome plot of *Lysobacter* strains. Closed genomes of *L. antibioticus* (L. ant) ATCC29479, *L. antibioticus* 76, *L. capsici* (L. cap) 55, *L. gummosus* (L. gum) 3.2.11, *L. enzymogenes* (L. enz) C3 and incomplete genome sequence of *L. capsici* AZ78 are included. The left panel shows core- and pan-genome plots calculated based on the CDS number ordering of the genomes with the largest genome first. The blue line indicates pan genome, the red line the core genome. The right panel shows the core- and pan-genome size evolution graph generated by calculating the median core- and pan-genome size for each number of combinations in the given species set. The standard deviation is calculated for the number of species involved and plotted with the median core- or pan-genome size. Red dots indicates the pan genome size evolution and the blue dots indicate core genome size evolution.

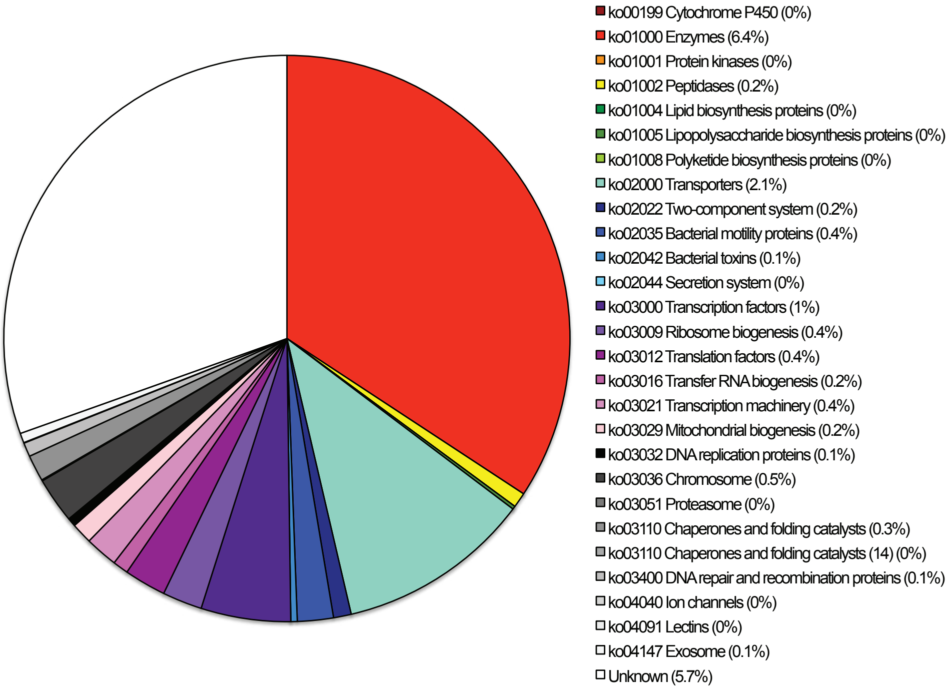

Figure S3. Summary of predicted gene product function of the *Lysobacter* unique core genome (2.891 CDS) using KEGG gene ontology terms. KEGG orthology terms were assigned to the unique core CDSs and, if assigned, grouped according to Brite hierarchy.

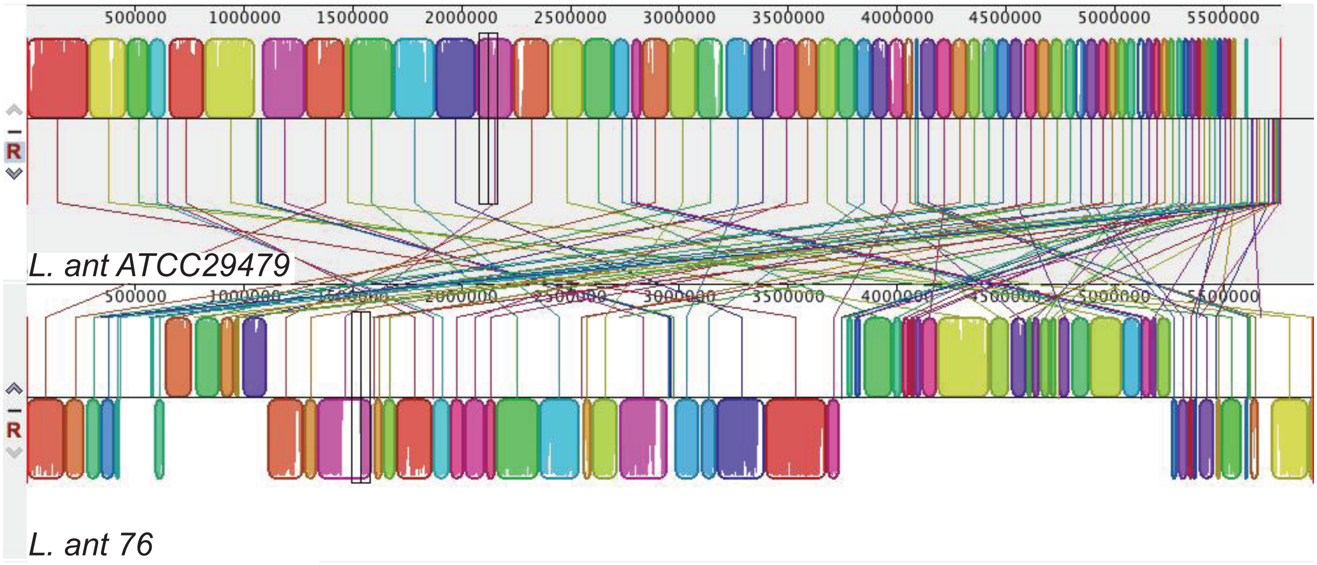

Figure S4. Whole genome alignment of the *Lysobacter antibioticus* strains. The genome sequence of *Lysobacter antibioticus* ATCC29479 was aligned to *L. antibioticus* 76 by Progressive MAUVE.

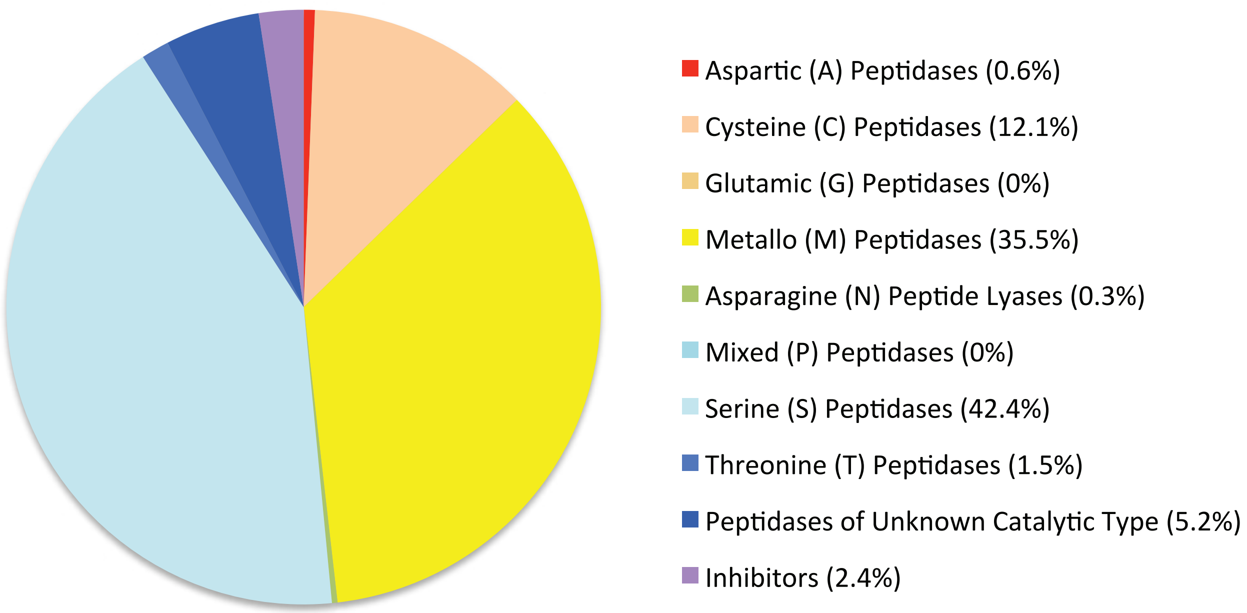

Figure S5. Percentage of number of CDSs encoding peptidases in the *Lysobacter antibioticus 76* genome sequence by BLASTp analysis in the MEROPS database. The pie charts for the other *Lysobacter* strains are comparable. The peptidase protein inhibitors as provided in the database were included in the analysis.

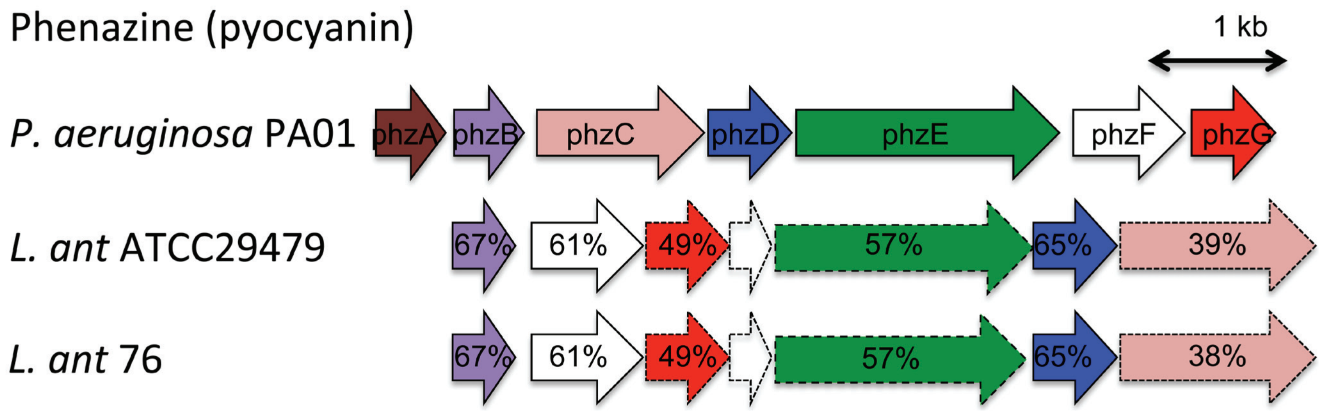

Figure S6. Gene cluster in *Lysobacter antibioticus* strains with similarity to phenazine biosynthesis cluster in *Pseudomonas aeruginosa* PA01. Each gene is colour coded and percentages indicate protein identity compared with *P. aeruginosa* phenazine proteins. L. ant: *Lysobacter antibioticus*.

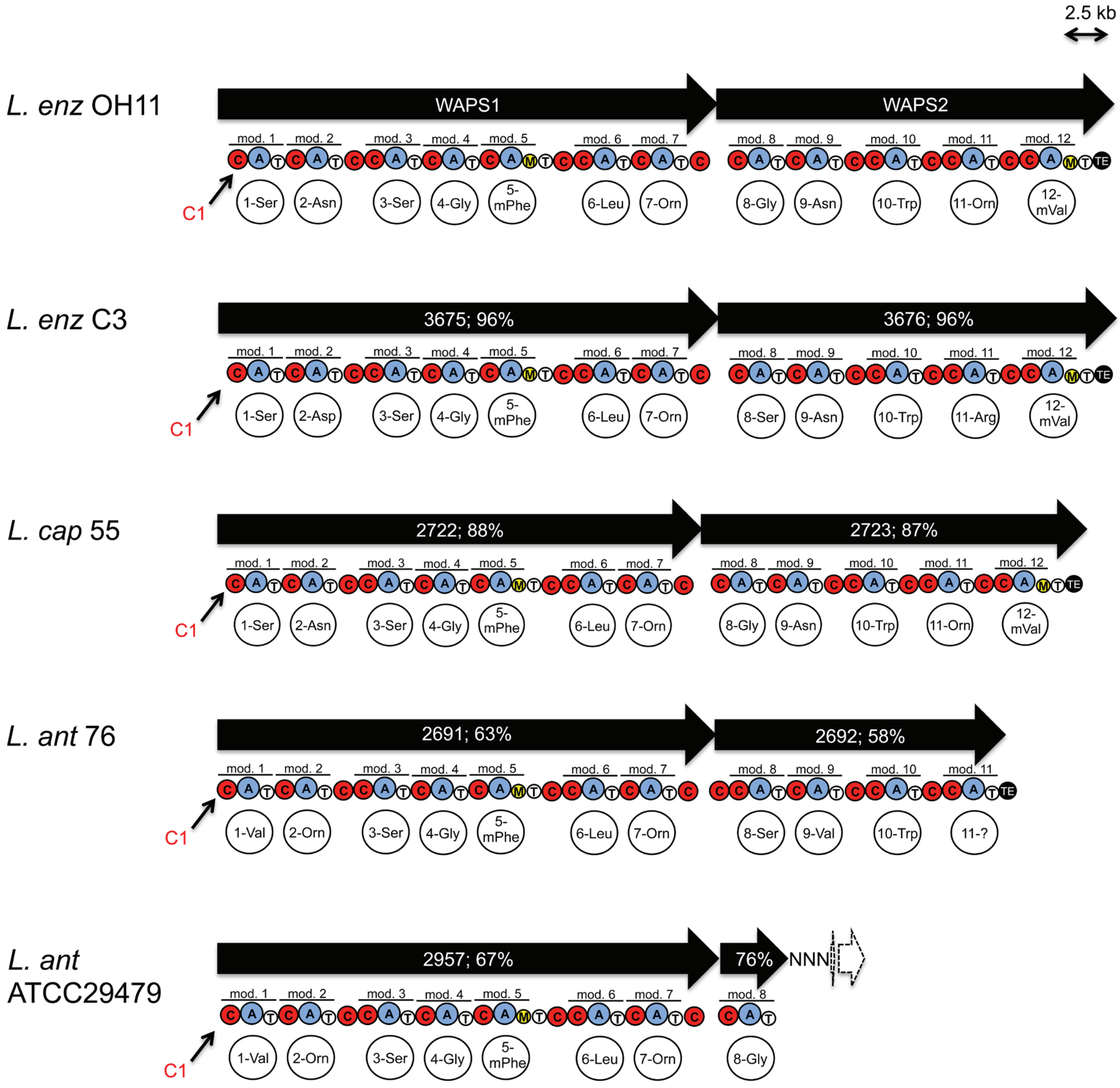

Figure S7. Gene clusters in *Lysobacter* genome sequences potentially encoding for WAP8294A2 and WAP8294A2-like biosynthesis. Indicated are the gene ID numbers and percentages of protein identity compared to the reference *L. enzymogenes* OH11. Underneath the genes the module and domain organization of the NRPSs are shown. The domains are labelled by: C, condensation; A, adenylation; T, thiolation; M, methylation and TE, thioesterification. Underneath the domains are the amino acids predicted to be incorporated into the CLP peptide moiety based on specific signature sequences in each A-domain and subsequent phylogenetic analysis. The number associated with the amino acid refers to the position of the amino acid in the predicted CLP peptide chain. The two *L. antibioticus* strains seem to contain a gene cluster in which only the WAPS1 gene is reasonably identical, indicating that these strains produce a potential novel compound. L. ant: *Lysobacter antibioticus*; L. cap: *L. capsici*; L. enz: *L. enzymogenes*.

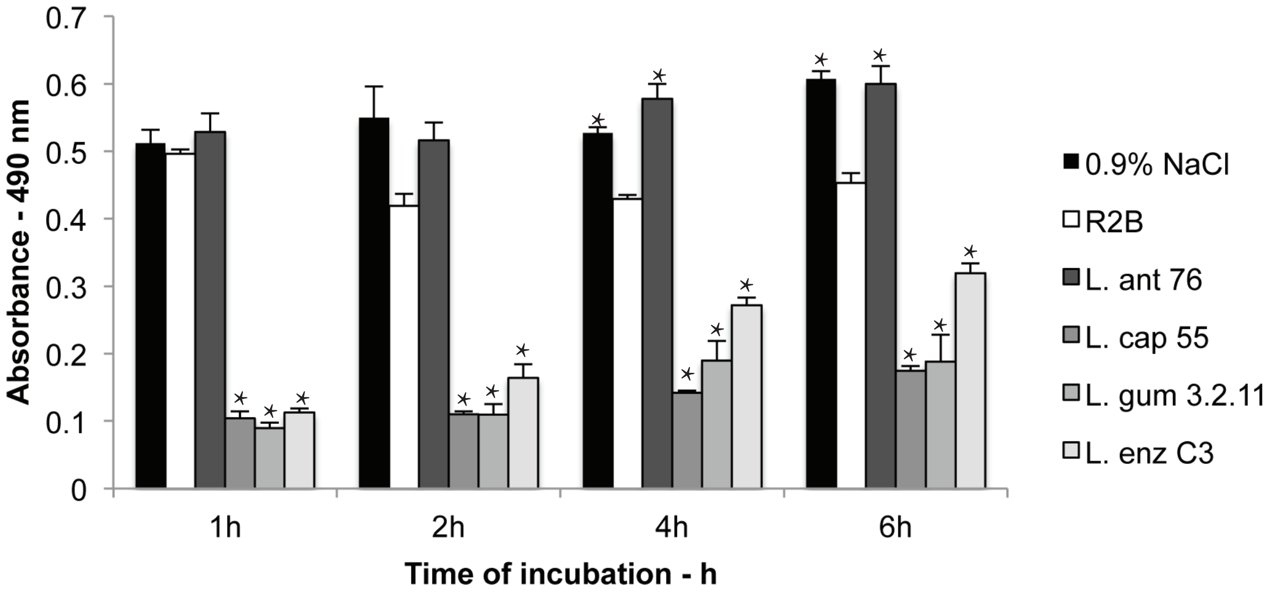

Figure S8. Effect of cell-free supernatant of the *Lysobacter* strains on XTT-formazan production by *Rhizoctonia solani* as a measure of fungal cell viability. Enzymatic activity of *R. solani* was measured by the conversion of XTT (2,3-bis-(2-methoxy-4-nitro-5-sulfophenyl)-2H-tetrazolium-5-carboxanilide) into the orange formazan dye which can be quantified by measuring absorbance at 490 nm. L. ant: *Lysobacter antibioticus*; L. cap: *L. capsici*; L. gum: *L. gummosus*; L. enz: *L. enzymogenes*. Significant differences ($p<0.05$) with the medium control R2B are indicated by asterisks and were calculated using analysis of variance and Dunnet's post-hoc analysis.

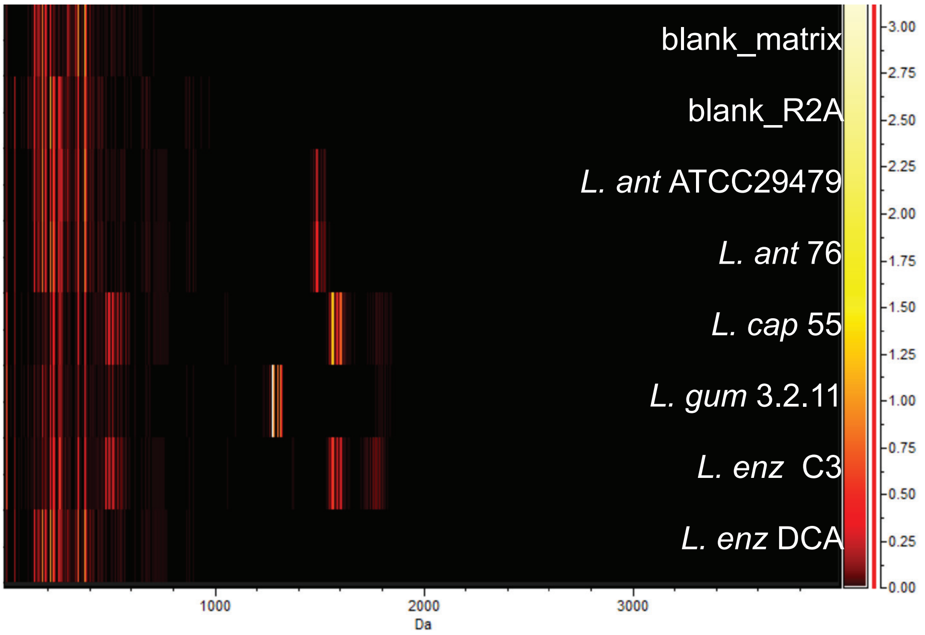

Figure S9. Heat plot of the MALDI mass spectra of metabolites produced by *Lysobacter* species. Metabolites present in ethanol extracts of *Lysobacter* strains grown on R2A medium. Each vertical line represents a peak in the mass spectra. The bar on the right represents the peak intensity (absorbance units) after normalization to the total ion count. L. ant: *Lysobacter antibioticus*; L. cap: *L. capsici*; L. gum: *L. gummosus*; L. enz: *L. enzymogenes*.

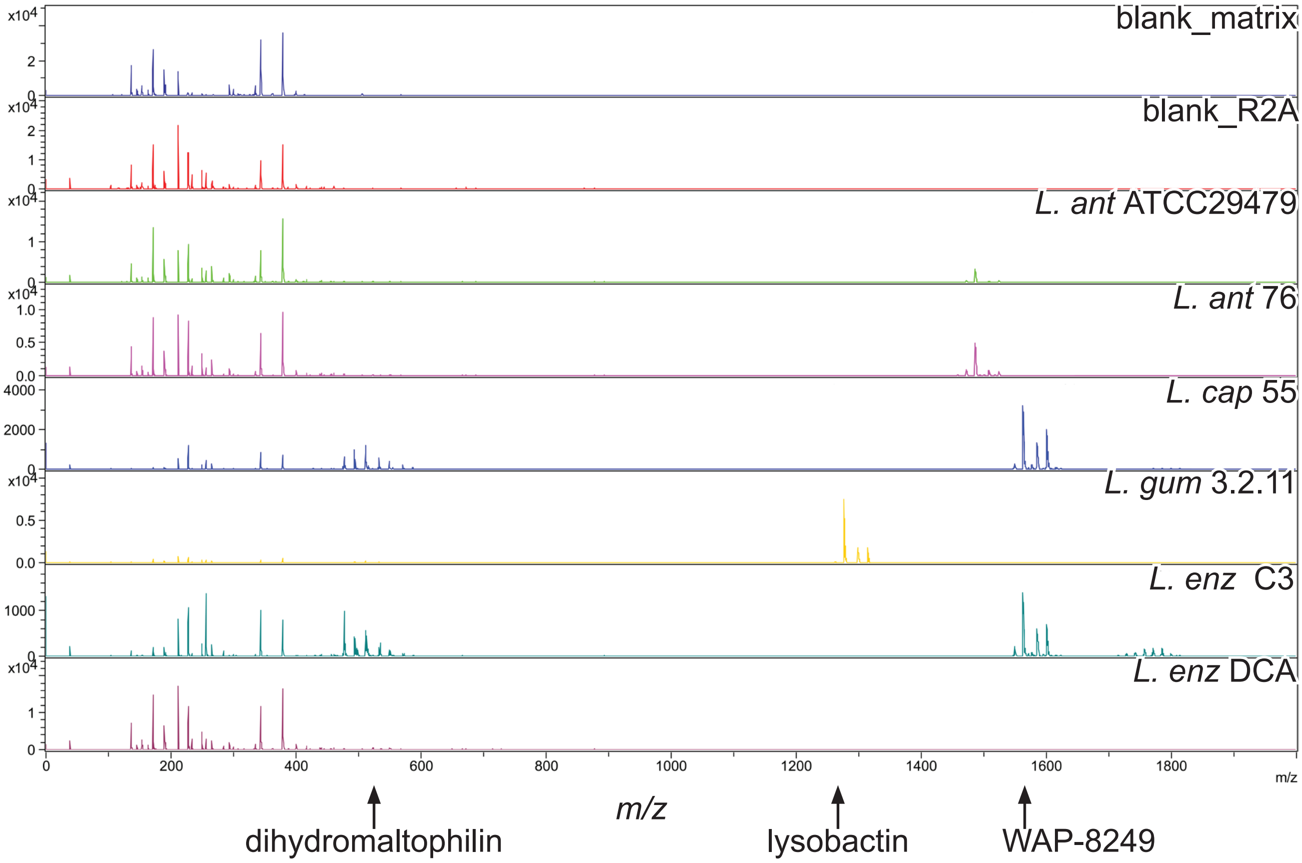

Figure S10. Matrix-assisted laser desorption/ionization (MALDI) mass spectra of a dried droplet of a methanol extract of *Lysobacter* strains grown on R2A medium. L. ant: *Lysobacter antibioticus*; L. cap: *L. capsici*; L. gum: *L. gummosus*; L. enz: *L. enzymogenes*.

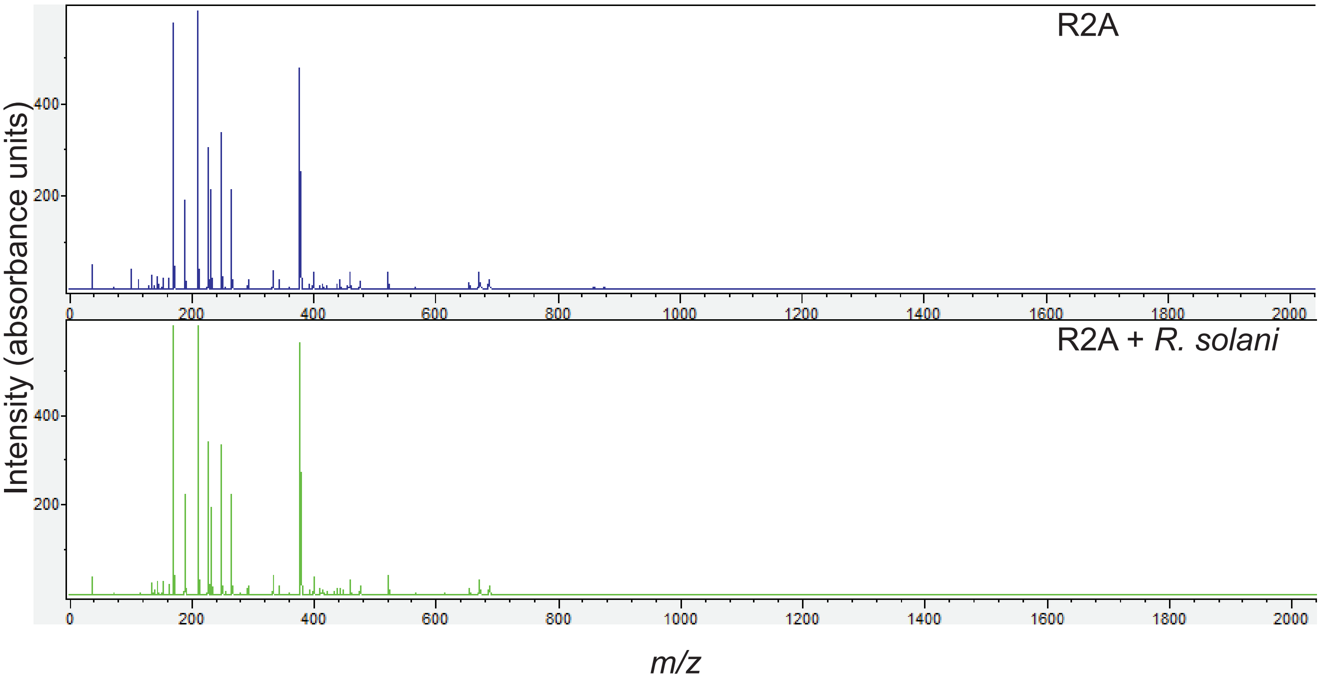

Figure S11. Matrix-assisted laser desorption/ionization (MALDI) imaging mass spectrometry (IMS) of *Rhizoctonia solani* incubated on R2A medium.

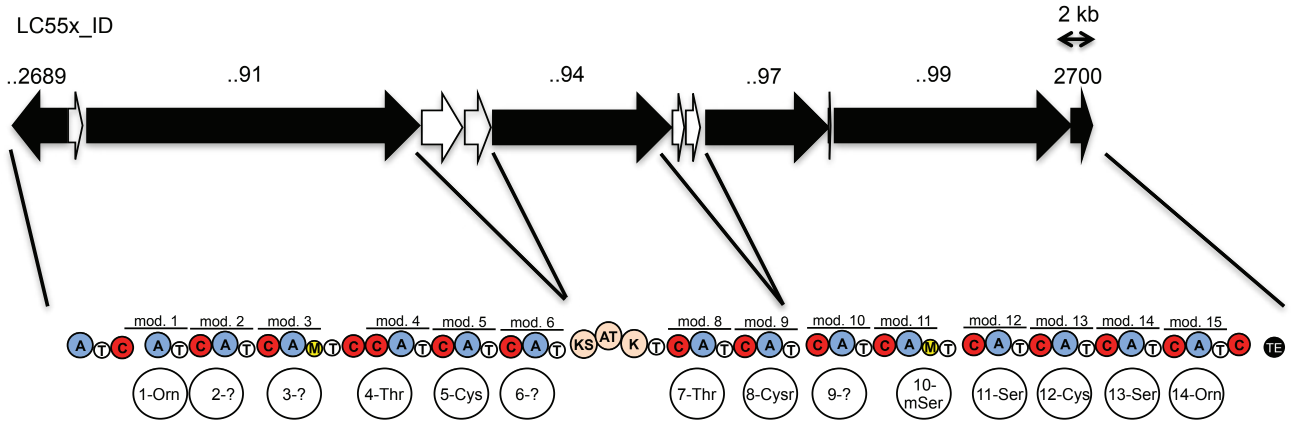

Figure S12. Putative NRPS/PKS gene cluster unique to *Lysobacter capsici* 55. Underneath the genes the module and domain organization of the NRPSs are shown. The domains are labelled by: C, condensation; A, adenylation; T, thiolation; M; methylation and TE, thioesterification. Underneath the domains are the amino acids predicted to be incorporated into the CLP peptide moiety based on specific signature sequences in each A-domain and subsequent phylogenetic analysis. The number associated with the amino acid refers to the position of the amino acid in the predicted CLP peptide chain.
