# Supplementary material for: Comparative genomics and metabolic profiling of the genus Lysobacter
Source: BMC Genomics. 2015 Nov 23;16:991. doi: 10.1186/s12864-015-2191-z (PMC4657364; doi:10.1186/s12864-015-2191-z)
Supplement: Additional file 2: Tables S1 to S3. — (PDF 1962 kb) [file 12864_2015_2191_MOESM2_ESM.pdf]

Supplementary tables S1-S3

Comparative genomics and metabolic profiling of the genus *Lysobacter*

Irene de Bruijn*, Xu Cheng, Victor de Jager, Ruth Gómez Expósito, Jeramie Watrous, Nrupali Patel, Joeke Postma, Pieter C. Dorrestein, Donald Kobayashi and Jos M. Raaijmakers.

*Correspondence: **Dr. Irene de Bruijn: i.debruijn@nioo.knaw.nl**

Table S1: pp 2-109. CDSs in unique core genome of the *Lysobacter*.

Table S2: pp. 110-145. CDSs unique to each *Lysobacter strain*.

Table S3: pp. 146-161. CDSs unique to either *Lysobacter antibioticus or L. capsici* species.

Table S1: CDSs in unique core genome of the *Lysobacter*.

Orthologous group	*L. ant* ATCC29479 GeneID (#3170) 55.8% of genome	Gene description
OG_00172	LA29479_3551	putative acetyltransferase
OG_00176	LA29479_3186	ABC transporter family protein
OG_00177	LA29479_4830	phosphoglycolate phosphatase, bacterial
OG_00178	LA29479_1130	FKBP-type peptidyl-prolyl cis-trans isomerase family protein
OG_00179	LA29479_0960	putative uncharacterized protein
OG_00180	LA29479_1980	cobQ/CobB/MinD/ParA nucleotide binding domain protein
OG_00181	LA29479_3773	sctL
OG_00182	LA29479_3997	tat (twin-arginine translocation) pathway signal sequence domain prot
OG_00183	LA29479_1212	tetratricopeptide repeat family protein
OG_00184	LA29479_0293	amidohydrolase family protein
OG_00185	LA29479_4378	beta-lactamase family protein
OG_00186	LA29479_1303	vacJ like lipofamily protein
OG_00187	LA29479_0984	major Facilitator Superfamily protein
OG_00188	LA29479_4295	orotidine 5'-phosphate decarboxylase
OG_00189	LA29479_0102	peptidase S41 family protein
OG_00190	LA29479_0915	putative transmembrane protein
OG_00191	LA29479_3478	efflux transporter, RND family, MFP subunit
OG_00192	LA29479_2217	multicopper oxidase family protein
OG_00193	LA29479_1150	cupin superfamily protein
OG_00194	LA29479_2104	indole-3-glycerol phosphate synthase family protein
OG_00195	LA29479_0684	dnaA regulatory inactivator Hda
OG_00196	LA29479_2341	conserved hypothetical protein
OG_00197	LA29479_1731	major Facilitator Superfamily protein
OG_00198	LA29479_3647	ATP phosphoribosyltransferase
OG_00199	LA29479_2707	putative uncharacterized protein
OG_00200	LA29479_1775	phosphotransferase
OG_00201	LA29479_2915	putative membrane protein
OG_00202	LA29479_3422	thiamine-phosphate pyrophosphorylase
OG_00203	LA29479_2916	cupin domain protein
OG_00204	LA29479_0658	acetyltransferase family protein
OG_00205	LA29479_5217	scpA/B family protein
OG_00206	LA29479_3325	KNTase C-terminal domain protein
OG_00207	LA29479_4171	ATP-dependent DNA helicase RecG
OG_00208	LA29479_4825	peptidase M23 family protein
OG_00209	LA29479_3074	alpha/beta hydrolase fold family protein
OG_00210	LA29479_1133	bacterial regulatory s, gntR family protein
OG_00211	LA29479_1914	large conductance mechanosensitive channel protein
OG_00212	LA29479_4116	transaldolase
OG_00213	LA29479_4205	ribose 5-phosphate isomerase A
OG_00214	LA29479_4258	threonyl-tRNA synthetase
OG_00215	LA29479_4078	putative dehydratase
OG_00216	LA29479_1886	conserved hypothetical protein
OG_00217	LA29479_3992	metalloendopeptidase, , glycoprotease family protein
OG_00218	LA29479_4752	beta-lactamase family protein
OG_00219	LA29479_2831	acrB/AcrD/AcrF family protein
OG_00220	LA29479_4024	putative uncharacterized protein
OG_00221	LA29479_4715	cold-shock domain protein
OG_00222	LA29479_3600	proline iminopeptidase
OG_00223	LA29479_1413	kinase A inhibitor
OG_00224	LA29479_0885	nlpC/P60 family protein
OG_00225	LA29479_3968	putative membrane protein
OG_00226	LA29479_1317	putative uncharacterized domain protein
OG_00227	LA29479_0623	putative uncharacterized protein
OG_00228	LA29479_1238	hypothetical protein
OG_00229	LA29479_0753	uroporphyrinogen decarboxylase
OG_00230	LA29479_0275	conserved hypothetical protein
OG_00231	LA29479_4957	dihydrofolate reductase type 3
OG_00232	LA29479_1068	replicative DNA helicase
OG_00233	LA29479_3377	disulfide bond formation DsbB family protein
OG_00234	LA29479_1234	tRNA 2-thiocytidine biosynthesis protein TtcA
OG_00235	LA29479_2413	polysaccharide biosynthesis/export family protein
OG_00236	LA29479_0663	putative uncharacterized protein
OG_00237	LA29479_2872	PGAP1-like family protein
OG_00238	LA29479_2909	GMC oxidoreductase family protein
OG_00239	LA29479_3100	glutathione S-transferase, N-terminal domain protein
OG_00240	LA29479_3937	response regulator
OG_00241	LA29479_0916	tonB-dependent Receptor Plug domain protein
OG_00242	LA29479_0312	cytochrome c-555 domain protein
OG_00243	LA29479_3921	beta-lactamase family protein
OG_00244	LA29479_4927	ATP-dependent protease subunit HslV
OG_00245	LA29479_1128	nucleotide sugar dehydrogenase family protein
OG_00246	LA29479_1660	patatin-like phospholipase family protein
OG_00247	LA29479_1869	polyamine ABC transporter, ATP-binding family protein
OG_00248	LA29479_0620	polyprenyl synthetase family protein
OG_00249	LA29479_3848	na+/H+ antiporter family protein
OG_00250	LA29479_4993	thioesterase , putative domain protein

Orthologous group	*L. ant* 76 GeneID (#3214) 56.2% of genome	Gene description
OG_00172	LA76x_3961	LA76x_3961; putative acetyltransferase
OG_00176	LA76x_1837	LA76x_1837; ABC transporter family protein
OG_00177	LA76x_3293	gph; phosphoglycolate phosphatase, bacterial
OG_00178	LA76x_2514	LA76x_2514; FKBP-type peptidyl-prolyl cis-trans isomerase family pr
OG_00179	LA76x_3803	LA76x_3803; curli production assembly/transport component CsgG fa
OG_00180	LA76x_1221	LA76x_1221; cobQ/CobB/MinD/ParA nucleotide binding domain prote
OG_00181	LA76x_4792	LA76x_4792; flagellar assembly FliH family protein
OG_00182	LA76x_4584	LA76x_4584; membrane dipeptidase family protein
OG_00183	LA76x_154	LA76x_154; lipopolysaccharide kinase family protein
OG_00184	LA76x_5151	LA76x_5151; amidohydrolase family protein
OG_00185	LA76x_1460	LA76x_1460; beta-lactamase family protein
OG_00186	LA76x_70	LA76x_70; vacJ like lipofamily protein
OG_00187	LA76x_3828	LA76x_3828; major Facilitator Superfamily protein
OG_00188	LA76x_1140	pyrF; orotidine 5'-phosphate decarboxylase
OG_00189	LA76x_3085	LA76x_3085; peptidase S41 family protein
OG_00190	LA76x_3755	LA76x_3755; conserved hypothetical protein
OG_00191	LA76x_4262	LA76x_4262; efflux transporter, RND family, MFP subunit
OG_00192	LA76x_4283	LA76x_4283; multicopper oxidase family protein
OG_00193	LA76x_2494	LA76x_2494; araC-like ligand binding domain protein
OG_00194	LA76x_1048	LA76x_1048; indole-3-glycerol phosphate synthase family protein
OG_00195	LA76x_1589	hda; dnaA regulatory inactivator Hda
OG_00196	LA76x_4405	LA76x_4405; conserved hypothetical protein
OG_00197	LA76x_2103	LA76x_2103; major Facilitator Superfamily protein
OG_00198	LA76x_3228	hisG; ATP phosphoribosyltransferase
OG_00199	LA76x_2428	LA76x_2428; conserved hypothetical protein
OG_00200	LA76x_2854	LA76x_2854; putative phosphotransferase
OG_00201	LA76x_717	LA76x_717; conserved hypothetical protein
OG_00202	LA76x_4208	thiE; thiamine-phosphate pyrophosphorylase
OG_00203	LA76x_718	LA76x_718; cupin domain protein
OG_00204	LA76x_1617	LA76x_1617; acetyltransferase family protein
OG_00205	LA76x_2434	LA76x_2434; scpA/B family protein
OG_00206	LA76x_162	LA76x_162; KNTase C-terminal domain protein
OG_00207	LA76x_4165	recG; ATP-dependent DNA helicase RecG
OG_00208	LA76x_3288	LA76x_3288; conserved hypothetical protein
OG_00209	LA76x_888	LA76x_888; alpha/beta hydrolase fold family protein
OG_00210	LA76x_2511	LA76x_2511; bacterial regulatory, gntR family protein
OG_00211	LA76x_1379	mscL; large conductance mechanosensitive channel protein
OG_00212	LA76x_338	tal; transaldolase
OG_00213	LA76x_4196	rpiA; ribose 5-phosphate isomerase A
OG_00214	LA76x_1744	thrS; threonine--tRNA ligase
OG_00215	LA76x_253	LA76x_253; fabA-like domain protein
OG_00216	LA76x_2745	LA76x_2745; low affinity iron permease family protein
OG_00217	LA76x_4579	LA76x_4579; putative peptidase
OG_00218	LA76x_2279	LA76x_2279; beta-lactamase family protein
OG_00219	LA76x_2302	LA76x_2302; acrB/AcrD/AcrF family protein
OG_00220	LA76x_4612	LA76x_4612; conserved hypothetical protein
OG_00221	LA76x_3471	LA76x_3471; cold-shock domain protein
OG_00222	LA76x_4008	pip; prolyl aminopeptidase
OG_00223	LA76x_2091	kipI; kinase A inhibitor
OG_00224	LA76x_3725	LA76x_3725; nlpC/P60 family protein
OG_00225	LA76x_800	LA76x_800; hypothetical protein
OG_00226	LA76x_55	LA76x_55; cytochrome oxidase complex assembly 1 family protein
OG_00227	LA76x_1651	LA76x_1651; conserved hypothetical protein
OG_00228	LA76x_130	LA76x_130; hypothetical protein
OG_00229	LA76x_1523	hemE; uroporphyrinogen decarboxylase
OG_00230	LA76x_5170	LA76x_5170; pseudouridine synthase family protein
OG_00231	LA76x_4045	dhfrIII; dihydrofolate reductase type 3
OG_00232	LA76x_2571	dnaB; replicative DNA helicase
OG_00233	LA76x_3902	LA76x_3902; disulfide bond formation DsbB family protein
OG_00234	LA76x_134	ttcA; tRNA 2-thiocytidine biosynthesis protein TtcA
OG_00235	LA76x_3383	LA76x_3383; polysaccharide biosynthesis/export family protein
OG_00236	LA76x_1612	LA76x_1612; conserved hypothetical protein
OG_00237	LA76x_677	LA76x_677; alpha/beta hydrolase family protein
OG_00238	LA76x_711	LA76x_711; pyridine nucleotide-disulfide oxidoreductase family protei
OG_00239	LA76x_912	LA76x_912; glutathione S-transferase, N-terminal domain protein
OG_00240	LA76x_768	LA76x_768; bacterial regulatory, luxR family protein
OG_00241	LA76x_3756	LA76x_3756; tonB dependent receptor family protein
OG_00242	LA76x_5132	LA76x_5132; cytochrome c-555 domain protein
OG_00243	LA76x_751	LA76x_751; beta-lactamase family protein
OG_00244	LA76x_814	hslV; ATP-dependent protease HslVU, peptidase subunit
OG_00245	LA76x_2516	LA76x_2516; nucleotide sugar dehydrogenase family protein
OG_00246	LA76x_2175	LA76x_2175; patatin-like phospholipase family protein
OG_00247	LA76x_2760	LA76x_2760; polyamine ABC transporter, ATP-binding family protein
OG_00248	LA76x_1654	LA76x_1654; polyprenyl synthetase family protein
OG_00249	LA76x_3548	LA76x_3548; na+/H+ antiporter family protein
OG_00250	LA76x_4725	LA76x_4725; putative thioesterase domain protein

OG_00251	LA29479_1602	short chain dehydrogenase family protein	OG_00251	LA76x_2232	LA76x_2232; short chain dehydrogenase family protein
OG_00252	LA29479_1686	putative uncharacterized protein	OG_00252	LA76x_2147	LA76x_2147; conserved hypothetical protein
OG_00253	LA29479_1484	MATE efflux family protein	OG_00253	LA76x_2020	LA76x_2020; MATE efflux family protein
OG_00254	LA29479_4285	riboflavin biosynthesis protein RibD	OG_00254	LA76x_1150	ribD; riboflavin biosynthesis protein RibD
OG_00255	LA29479_4602	ATP-dependent helicase HrpA	OG_00255	LA76x_4097	hrpA; ATP-dependent helicase HrpA
OG_00256	LA29479_0674	sulfite reductase (NADPH) hemoprotein, beta-component	OG_00256	LA76x_1600	cysI; sulfite reductase (NADPH) hemoprotein, beta-component
OG_00257	LA29479_4001	sodium Bile acid symporter family protein	OG_00257	LA76x_4588	LA76x_4588; sodium Bile acid symporter family protein
OG_00258	LA29479_3210	hemolysin	OG_00258	LA76x_1815	tlyC; hemolysin
OG_00259	LA29479_4885	RNase H family protein	OG_00259	LA76x_3543	LA76x_3543; RNase H family protein
OG_00260	LA29479_3323	ABC transporter family protein	OG_00260	LA76x_164	LA76x_164; ABC transporter family protein
OG_00261	LA29479_0641	putative lipoprotein	OG_00261	LA76x_1634	LA76x_1634; hypothetical protein
OG_00262	LA29479_2833	cytochrome c family protein	OG_00262	LA76x_2299	LA76x_2299; cytochrome c family protein
OG_00263	LA29479_3677	biotin-requiring enzyme family protein	OG_00263	LA76x_3200	LA76x_3200; ATP-grasp domain protein
OG_00264	LA29479_3825	arsenical resistance protein ArsH	OG_00264	LA76x_4745	arsH; arsenical resistance protein ArsH
OG_00265	LA29479_2350	cyclic nucleotide-binding domain protein	OG_00265	LA76x_4414	LA76x_4414; cyclic nucleotide-binding domain protein
OG_00266	LA29479_2327	ATP synthase F0, A subunit	OG_00266	LA76x_4390	atpB; ATP synthase F0, A subunit
OG_00267	LA29479_3621	ubiquinone biosynthesis hydroxylase, UbiH/UbiF/VisC/COQ6 family p	OG_00267	LA76x_4030	LA76x_4030; ubiquinone biosynthesis hydroxylase, UbiH/UbiF/VisC/(
OG_00268	LA29479_0705	conserved hypothetical protein	OG_00268	LA76x_1570	LA76x_1570; lipopolysaccharide-assembly, LptC-related family protei
OG_00269	LA29479_3984	tonB-dependent Receptor Plug domain protein	OG_00269	LA76x_4573	LA76x_4573; tonB dependent receptor family protein
OG_00270	LA29479_1764	heat-inducible transcription repressor HrcA	OG_00270	LA76x_2865	hrcA; heat-inducible transcription repressor HrcA
OG_00271	LA29479_0815	putative uncharacterized protein	OG_00271	LA76x_3657	LA76x_3657; conserved hypothetical protein
OG_00272	LA29479_4002	xaa-Pro dipeptidase, putative	OG_00272	LA76x_4589	LA76x_4589; xaa-Pro dipeptidase, putative
OG_00273	LA29479_4117	pyridine nucleotide-disulphide oxidoreductase family protein	OG_00273	LA76x_337	LA76x_337; FAD binding domain protein
OG_00274	LA29479_2953	L-Ala-D/L-Glu epimerase	OG_00274	LA76x_744	ykfB; L-Ala-D/L-Glu epimerase
OG_00275	LA29479_3769	putative uncharacterized protein	OG_00275	LA76x_4796	LA76x_4796; conserved hypothetical protein
OG_00276	LA29479_2418	tyrosine-protein kinase etk	OG_00276	LA76x_3387	etk; tyrosine-protein kinase etk
OG_00277	LA29479_2198	hypothetical protein	OG_00277	LA76x_959	LA76x_959; hypothetical protein
OG_00278	LA29479_3409	DNA-directed RNA polymerase, beta' subunit	OG_00278	LA76x_3935	rpoC; DNA-directed RNA polymerase, beta' subunit
OG_00280	LA29479_0838	hflC protein	OG_00280	LA76x_3678	hflC; hflC protein
OG_00281	LA29479_1192	ABC transporter family protein	OG_00281	LA76x_2451	LA76x_2451; ABC transporter family protein
OG_00282	LA29479_4513	cation diffusion facilitator transporter family protein	OG_00282	LA76x_1921	LA76x_1921; cation diffusion facilitator transporter family protein
OG_00283	LA29479_1433	DNA mismatch repair MutL family protein	OG_00283	LA76x_2071	mutL; DNA mismatch repair MutL family protein
OG_00284	LA29479_3488	glycosyl transferases group 1 family protein	OG_00284	LA76x_4271	LA76x_4271; glycosyl transferases group 1 family protein
OG_00285	LA29479_4003	protoheme IX farnesyltransferase	OG_00285	LA76x_4590	cyoE; protoheme IX farnesyltransferase
OG_00286	LA29479_3125	amino acid permease family protein	OG_00286	LA76x_937	LA76x_937; amino acid permease family protein
OG_00287	LA29479_1060	DNA photolyase family protein	OG_00287	LA76x_2582	LA76x_2582; FAD binding domain of DNA photolyase family protein
OG_00288	LA29479_4896	aminodeoxychorismate lyase	OG_00288	LA76x_3497	pabC; aminodeoxychorismate lyase
OG_00289	LA29479_4972	fructokinase-1	OG_00289	LA76x_379	FRK1; fructokinase-1
OG_00290	LA29479_1384	putative membrane protein	OG_00290	LA76x_5194	LA76x_5194; ABC-2 transporter family protein
OG_00291	LA29479_3628	glycosyl transferase 2 family protein	OG_00291	LA76x_3241	LA76x_3241; glycosyl transferase 2 family protein
OG_00292	LA29479_1996	conserved hypothetical protein	OG_00292	LA76x_1206	yfiO; outer membrane assembly lipoYfiO family protein
OG_00293	LA29479_2496	N-acetylmuramoyl-L-alanine amidase A domain protein	OG_00293	LA76x_4450	cwhA; N-acetylmuramoyl-L-alanine amidase A domain protein
OG_00294	LA29479_3652	phosphoribosylformimino-5-aminoimidazole carboxamide ribotide isor	OG_00294	LA76x_3223	hisA; 1-(5-phosphoribosyl)-5-[(5- phosphoribosylamino)methylidenear
OG_00295	LA29479_3567	putative lipoprotein	OG_00295	LA76x_3975	LA76x_3975; hypothetical protein
OG_00296	LA29479_1085	N-terminal double-transmembrane domain protein	OG_00296	LA76x_2555	LA76x_2555; N-terminal double-transmembrane domain protein
OG_00297	LA29479_4587	acetyltransferase family protein	OG_00297	LA76x_1411	LA76x_1411; acetyltransferase family protein
OG_00298	LA29479_2103	haloacid dehalogenase-like hydrolase family protein	OG_00298	LA76x_1049	LA76x_1049; HAD phosphoserine phosphatase-like hydrolase, IB far
OG_00299	LA29479_0139	outer membrane autotransporter barrel domain protein	OG_00299	LA76x_3048	LA76x_3048; outer membrane autotransporter barrel domain protein
OG_00300	LA29479_0657	cysteine desulfurases, SufS subfamily protein	OG_00300	LA76x_1618	sufS; cysteine desulfurase, SufS family protein
OG_00301	LA29479_0463	ABC transporter family protein	OG_00301	LA76x_4896	LA76x_4896; ABC transporter family protein
OG_00302	LA29479_1598	hypothetical protein	OG_00302	LA76x_2236	LA76x_2236; hypothetical protein
OG_00303	LA29479_2105	uncharacterized protein yqjZ	OG_00303	LA76x_1047	LA76x_1047; conserved hypothetical protein
OG_00304	LA29479_0500	phospholipase D Active site motif family protein	OG_00304	LA76x_4860	LA76x_4860; phospholipase D family protein
OG_00305	LA29479_0237	short chain dehydrogenase family protein	OG_00305	LA76x_2952	LA76x_2952; short chain dehydrogenase family protein
OG_00306	LA29479_1055	copper resistance protein B	OG_00306	LA76x_2587	copB; copper resistance protein B
OG_00307	LA29479_0726	methyladenine glycosylase family protein	OG_00307	LA76x_1549	LA76x_1549; methyladenine glycosylase family protein
OG_00308	LA29479_4147	tautomerase enzyme family protein	OG_00308	LA76x_307	LA76x_307; tautomerase enzyme family protein
OG_00309	LA29479_1261	acetyltransferase family protein	OG_00309	LA76x_110	LA76x_110; acetyltransferase family protein
OG_00310	LA29479_0857	bacterial regulatory s, luxR family protein	OG_00310	LA76x_3696	LA76x_3696; bacterial regulatory, luxR family protein
OG_00311	LA29479_0523	hydrolase of the alpha/beta fold superfamily	OG_00311	LA76x_4838	LA76x_4838; alpha/beta hydrolase fold family protein
OG_00312	LA29479_1116	ATP-NAD kinase family protein	OG_00312	LA76x_2528	LA76x_2528; ATP-NAD kinase family protein
OG_00313	LA29479_3330	fatty acid desaturase family protein	OG_00313	LA76x_158	LA76x_158; fatty acid desaturase family protein
OG_00314	LA29479_4903	3-oxoacyl-[acyl-carrier-protein] synthase 3	OG_00314	LA76x_3503	fabH; 3-oxoacyl-[acyl-carrier-] synthase III family protein
OG_00315	LA29479_0976	formiminoglutamate deiminase	OG_00315	LA76x_3820	hutF; formiminoglutamate deiminase
OG_00316	LA29479_3651	imidazole glycerol phosphate synthase, glutamine amidotransferase s	OG_00316	LA76x_3224	hisH; imidazole glycerol phosphate synthase, glutamine amidotransfe
OG_00317	LA29479_3413	50S ribosomal protein L10	OG_00317	LA76x_3939	rplJ; 50S ribosomal subunit protein L10
OG_00318	LA29479_3534	his Kinase A domain protein	OG_00318	LA76x_2702	LA76x_2702; histidine kinase-, DNA gyrase B-, and HSP90-like ATPa
OG_00319	LA29479_3490	putative quercetin 2,3-dioxygenase yhhW	OG_00319	LA76x_4273	yhhW; pirin-like protein
OG_00320	LA29479_3507	isoamylase	OG_00320	LA76x_2730	iam; isoamylase
OG_00321	LA29479_2891	putative uncharacterized protein	OG_00321	LA76x_695	LA76x_695; conserved hypothetical protein
OG_00322	LA29479_3155	conserved hypothetical protein	OG_00322	LA76x_1865	LA76x_1865; inhibitor of apoptosis-promoting Bax1 family protein
OG_00323	LA29479_4057	uncharacterized , UPF0114 family protein	OG_00323	LA76x_271	LA76x_271; conserved hypothetical protein
OG_00324	LA29479_3489	putative uncharacterized protein	OG_00324	LA76x_4272	LA76x_4272; zinc-finger domain protein
OG_00325	LA29479_4497	putative nitroreductase	OG_00325	LA76x_4662	LA76x_4662; sagB-type dehydrogenase domain protein
OG_00326	LA29479_4141	his Kinase A domain protein	OG_00326	LA76x_313	LA76x_313; his Kinase A domain protein
OG_00327	LA29479_3541	sugar (and other) transporter family protein	OG_00327	LA76x_2696	LA76x_2696; sugar (and other) transporter family protein
OG_00328	LA29479_5196	peptidase M48 family protein	OG_00328	LA76x_3299	htpX; heat shock , integral membrane protein
OG_00329	LA29479_4175	bacterial regulatory helix-turn-helix s, AraC family protein	OG_00329	LA76x_4169	LA76x_4169; bacterial regulatory helix-turn-helix, AraC family protein
OG_00330	LA29479_3602	F5/8 type C domain protein	OG_00330	LA76x_4010	LA76x_4010; F5/8 type C domain protein
OG_00331	LA29479_2079	putative ATP binding component of ABC-transporter	OG_00331	LA76x_1073	LA76x_1073; putative ATP binding component of ABC-transporter
OG_00332	LA29479_1315	putative aspartate/glutamate racemase	OG_00332	LA76x_57	LA76x_57; putative aspartate/glutamate racemase

OG_00334	LA29479_1912	putative uncharacterized domain protein
OG_00335	LA29479_2270	RNA polymerase sigma factor, sigma-70 family protein
OG_00336	LA29479_4451	phosphomannomutase/phosphoglucomutase domain protein
OG_00337	LA29479_1870	putrescine-binding periplasmic protein
OG_00338	LA29479_2156	arginase
OG_00339	LA29479_5202	GTP-dependent nucleic acid-binding protein engD
OG_00340	LA29479_1323	histidine kinase-, DNA gyrase B-, and HSP90-like ATPase family prot
OG_00341	LA29479_4533	chromate reductase
OG_00342	LA29479_4379	putative uncharacterized protein
OG_00343	LA29479_4911	conserved hypothetical protein
OG_00344	LA29479_1673	glutathione peroxidase family protein
OG_00345	LA29479_1063	rickettsia 17 kDa surface antigen family protein
OG_00346	LA29479_2408	enoyl-CoA hydratase/isomerase family protein
OG_00347	LA29479_3459	bifunctional aspartokinase/homoserine dehydrogenase 1, chloroplasti
OG_00348	LA29479_4808	N-acetyl-gamma-glutamyl-phosphate reductase
OG_00349	LA29479_1960	acetyltransferase family protein
OG_00350	LA29479_2347	permease family protein
OG_00351	LA29479_1908	thiazole synthase
OG_00352	LA29479_0818	putative uncharacterized domain protein
OG_00353	LA29479_0288	yecA family protein
OG_00354	LA29479_4012	cytochrome c oxidase, subunit II
OG_00355	LA29479_0717	phosphocarrier, HPr family protein
OG_00356	LA29479_1006	L-threonine 3-dehydrogenase
OG_00357	LA29479_4204	conserved hypothetical protein
OG_00358	LA29479_5072	type III secretion apparatus protein SpaR/YscT/HrcT
OG_00359	LA29479_3836	putative acetyltransferase
OG_00360	LA29479_2273	iron-sulfur cluster insertion protein erpA
OG_00361	LA29479_2219	putative uncharacterized protein
OG_00362	LA29479_5148	HTH-type transcriptional repressor nemR
OG_00363	LA29479_3775	putative uncharacterized protein
OG_00364	LA29479_5131	3-phosphoshikimate 1-carboxyvinyltransferase
OG_00365	LA29479_2751	cysteine dioxygenase type I family protein
OG_00366	LA29479_2527	relaxation protein
OG_00367	LA29479_3333	2-methylcitrate dehydratase
OG_00368	LA29479_4186	hypothetical protein
OG_00369	LA29479_1418	conserved hypothetical protein
OG_00370	LA29479_4177	guanosine-3',5'-bis(diphosphate) 3'-pyrophosphohydrolase
OG_00371	LA29479_3359	phosphate-selective porin O and P family protein
OG_00372	LA29479_4197	aminopeptidase P, N-terminal domain protein
OG_00373	LA29479_2824	tetR-family transcriptional regulator
OG_00374	LA29479_2186	RNA-splicing ligase RtcB
OG_00375	LA29479_4447	DNA repair RadC family protein
OG_00376	LA29479_4681	adenosylmethionine-8-amino-7-oxononanoate transaminase
OG_00377	LA29479_4629	putative uncharacterized protein
OG_00378	LA29479_3896	putative lipoprotein
OG_00379	LA29479_2291	putative AfsR-like transcriptional regulator
OG_00380	LA29479_0861	2C-methyl-D-erythritol 2,4-cyclodiphosphate synthase
OG_00381	LA29479_2237	acetyl-CoA carboxylase, biotin carboxylase
OG_00382	LA29479_0530	hypothetical protein
OG_00383	LA29479_1190	relaxation domain protein
OG_00384	LA29479_5163	rod shape-determining protein RodA
OG_00385	LA29479_3201	putative membrane protein
OG_00386	LA29479_0457	V4R domain protein
OG_00387	LA29479_0700	putative secreted protein
OG_00388	LA29479_1308	similar to SAM-dependent methyltransferase
OG_00389	LA29479_3971	HTH domain protein
OG_00390	LA29479_2793	molybdenum cofactor synthesis domain protein
OG_00391	LA29479_3212	putative diguanylate cyclase/phosphodiesterase
OG_00392	LA29479_0736	proteobacterial lipase chaperone family protein
OG_00393	LA29479_0685	conserved hypothetical protein
OG_00394	LA29479_0067	alpha/beta hydrolase fold family protein
OG_00395	LA29479_3482	type I secretion outer membrane , TolC family protein
OG_00396	LA29479_2794	putative molybdopterin-guanine dinucleotide biosynthesis protein A
OG_00397	LA29479_3675	hydroxymethylglutaryl-CoA lyase
OG_00398	LA29479_3877	conserved hypothetical protein
OG_00399	LA29479_2559	bacterial extracellular solute-binding family protein
OG_00400	LA29479_2076	GDP-mannose 4,6-dehydratase
OG_00401	LA29479_3187	putative membrane protein
OG_00402	LA29479_1086	conserved hypothetical protein
OG_00403	LA29479_4216	ferredoxin
OG_00404	LA29479_3360	his Kinase A domain protein
OG_00405	LA29479_4068	beta-ketoacyl synthase, C-terminal domain protein
OG_00406	LA29479_2045	lppC superfamily
OG_00407	LA29479_1626	3-deoxy-D-manno-octulosonate cytidylyltransferase
OG_00408	LA29479_3252	putative membrane protein
OG_00409	LA29479_0969	bacterial regulatory s, tetR family protein
OG_00410	LA29479_0141	putative uncharacterized protein
OG_00411	LA29479_3214	RNA methyltransferase, TrmH , group 1 family protein
OG_00412	LA29479_4673	response regulator
OG_00413	LA29479_0213	NADH-quinone oxidoreductase, chain I family protein
OG_00414	LA29479_3561	acylphosphatase

OG_00334	LA76x_1381	LA76x_1381; putative membrane domain protein
OG_00335	LA76x_4335	LA76x_4335; RNA polymerase sigma factor, sigma-70 family protein
OG_00336	LA76x_4631	LA76x_4631; phosphoglucomutase/phosphomannomutase, C-termin
OG_00337	LA76x_2759	potF; potF
OG_00338	LA76x_997	rocF; arginase
OG_00339	LA76x_3950	LA76x_3950; 50S ribosome-binding GTPase family protein
OG_00340	LA76x_50	LA76x_50; histidine kinase-, DNA gyrase B-, and HSP90-like ATPase
OG_00341	LA76x_1901	chrR; chromate reductase
OG_00342	LA76x_1459	LA76x_1459; polyketide cyclase / dehydrase and lipid transport famil
OG_00343	LA76x_3510	LA76x_3510; conserved hypothetical protein
OG_00344	LA76x_2160	LA76x_2160; ahpC/TSA family protein
OG_00345	LA76x_2579	LA76x_2579; glycine-zipper containing OmpA-like membrane domai
OG_00346	LA76x_3378	LA76x_3378; enoyl-CoA hydratase/isomerase family protein
OG_00347	LA76x_4244	AKHSDH1; bifunctional aspartokinase/homoserine dehydrogenase 1,
OG_00348	LA76x_3271	argC; N-acetyl-gamma-glutamyl-phosphate reductase
OG_00349	LA76x_1240	LA76x_1240; acetyltransferase family protein
OG_00350	LA76x_4411	LA76x_4411; ftsX-like permease family protein
OG_00351	LA76x_1385	LA76x_1385; thiazole biosynthesis ThiG family protein
OG_00352	LA76x_3660	LA76x_3660; conserved hypothetical protein
OG_00353	LA76x_5156	LA76x_5156; yecA family protein
OG_00354	LA76x_4599	coxB; cytochrome c oxidase, subunit II
OG_00355	LA76x_1558	LA76x_1558; phosphocarrier, HPr family protein
OG_00356	LA76x_3849	tdh; L-threonine 3-dehydrogenase
OG_00357	LA76x_4195	LA76x_4195; EVE domain protein
OG_00358	LA76x_4806	epaR; type III secretion apparatus protein SpaR/YscT/HrcT
OG_00359	LA76x_4734	LA76x_4734; putative acetyltransferase
OG_00360	LA76x_4338	LA76x_4338; essential respiratory protein A
OG_00361	LA76x_4286	LA76x_4286; conserved hypothetical protein
OG_00362	LA76x_635	ydhM; nemR DNA binding transcriptional repressor
OG_00363	LA76x_4790	LA76x_4790; conserved hypothetical protein
OG_00364	LA76x_2260	aroA; 3-phosphoshikimate 1-carboxyvinyltransferase
OG_00365	LA76x_2383	LA76x_2383; cysteine dioxygenase type I family protein
OG_00366	LA76x_4482	LA76x_4482; putative relaxation protein
OG_00367	LA76x_3858	prpD; 2-methylcitrate dehydratase
OG_00368	LA76x_4180	LA76x_4180; hypothetical protein
OG_00369	LA76x_2086	LA76x_2086; conserved hypothetical protein
OG_00370	LA76x_4171	spoT; guanosine-3',5'-bis(diphosphate) 3'-pyrophosphohydrolase
OG_00371	LA76x_3885	LA76x_3885; phosphate-selective porin O and P family protein
OG_00372	LA76x_4189	LA76x_4189; aminopeptidase P, N-terminal domain protein
OG_00373	LA76x_2309	LA76x_2309; bacterial regulatory, tetR family protein
OG_00374	LA76x_969	rtcB; conserved protein
OG_00375	LA76x_4635	LA76x_4635; DNA repair RadC family protein
OG_00376	LA76x_4066	bioA; adenosylmethionine-8-amino-7-oxononanoate transaminase
OG_00377	LA76x_3439	LA76x_3439; hypothetical protein
OG_00378	LA76x_3597	LA76x_3597; hypothetical protein
OG_00379	LA76x_4354	LA76x_4354; putative afsR-like transcriptional regulator
OG_00380	LA76x_3700	ispF; 2-C-methyl-D-erythritol 2,4-cyclodiphosphate synthase
OG_00381	LA76x_4304	accC; acetyl-CoA carboxylase, biotin carboxylase subunit
OG_00382	LA76x_4831	LA76x_4831; conserved hypothetical protein
OG_00383	LA76x_2453	LA76x_2453; relaxation domain protein
OG_00384	LA76x_833	mrdB; rod shape-determining protein RodA
OG_00385	LA76x_1823	LA76x_1823; putative membrane protein
OG_00386	LA76x_4902	LA76x_4902; V4R domain protein
OG_00387	LA76x_1575	LA76x_1575; conserved hypothetical protein
OG_00388	LA76x_65	LA76x_65; ubiE/COQ5 methyltransferase family protein
OG_00389	LA76x_803	LA76x_803; WYL domain protein
OG_00390	LA76x_2340	LA76x_2340; moeA C-terminal region family protein
OG_00391	LA76x_1813	LA76x_1813; putative diguanylate cyclase/phosphodiesterase
OG_00392	LA76x_1540	LA76x_1540; proteobacterial lipase chaperone family protein
OG_00393	LA76x_1588	LA76x_1588; conserved hypothetical protein
OG_00394	LA76x_3121	LA76x_3121; prolyl oligopeptidase family protein
OG_00395	LA76x_4265	LA76x_4265; type I secretion outer membrane , TolC family protein
OG_00396	LA76x_2339	LA76x_2339; mobA-like NTP transferase domain protein
OG_00397	LA76x_3202	HMGCL; hydroxymethylglutaryl-CoA lyase
OG_00398	LA76x_3578	LA76x_3578; conserved hypothetical protein
OG_00399	LA76x_4511	LA76x_4511; bacterial extracellular solute-binding family protein
OG_00400	LA76x_1076	gmd; GDP-mannose 4,6-dehydratase
OG_00401	LA76x_1836	LA76x_1836; putative transmembrane domain protein
OG_00402	LA76x_2554	LA76x_2554; von Willebrand factor type A domain protein
OG_00403	LA76x_1787	fdx1; ferredoxin
OG_00404	LA76x_3886	LA76x_3886; response regulator
OG_00405	LA76x_263	LA76x_263; beta-ketoacyl synthase, C-terminal domain protein
OG_00406	LA76x_1162	LA76x_1162; lppC lipofamily protein
OG_00407	LA76x_2209	kdsB; 3-deoxy-D-manno-octulosonate cytidylyltransferase
OG_00408	LA76x_228	LA76x_228; putative transmembrane protein
OG_00409	LA76x_3812	LA76x_3812; bacterial regulatory, tetR family protein
OG_00410	LA76x_3047	LA76x_3047; conserved hypothetical protein
OG_00411	LA76x_1811	LA76x_1811; RNA methyltransferase, TrmH , group 1 family protein
OG_00412	LA76x_4058	LA76x_4058; response regulator
OG_00413	LA76x_2978	LA76x_2978; NADH-quinone oxidoreductase, chain I family protein
OG_00414	LA76x_3969	LA76x_3969; acylphosphatase family protein

OG_00416	LA29479_4519	outer membrane assembly complex, YaeT protein
OG_00417	LA29479_1661	hydro-lyases, Fe-S type, tartrate/fumarate subfamily, beta region dom
OG_00418	LA29479_5136	ferric reductase like transmembrane component family protein
OG_00419	LA29479_4805	glutamate 5-kinase
OG_00420	LA29479_0581	prolyl oligopeptidase family protein
OG_00421	LA29479_4142	putative protease
OG_00422	LA29479_2225	cupin 2, conserved barrel domain protein
OG_00423	LA29479_1181	uncharacterized protein YjeA
OG_00424	LA29479_3973	putative membrane protein
OG_00425	LA29479_1458	conserved hypothetical protein
OG_00426	LA29479_2040	mraZ family protein
OG_00427	LA29479_3649	histidinol-phosphate aminotransferase
OG_00428	LA29479_2323	ATP synthase F1, alpha subunit
OG_00429	LA29479_3092	HTH-type transcriptional repressor fabR
OG_00430	LA29479_2487	outer membrane protein oprM
OG_00431	LA29479_1410	RNA modification enzyme, MiaB family protein
OG_00432	LA29479_2022	preprotein translocase, SecA subunit
OG_00433	LA29479_4870	SMP-30/Gluconolaconase/LRE-like region-containing protein
OG_00434	LA29479_3465	ketol-acid reductoisomerase
OG_00435	LA29479_2708	bacterial regulatory s, tetR family protein
OG_00436	LA29479_0735	putative lipoprotein
OG_00437	LA29479_3884	pirin C-terminal cupin domain protein
OG_00438	LA29479_4564	pfkB carbohydrate kinase family protein
OG_00439	LA29479_4646	PAP2 superfamily protein
OG_00440	LA29479_3774	putative ATP synthase YscN
OG_00441	LA29479_2732	transglycosylase SLT domain protein
OG_00442	LA29479_2747	thiol:disulfide interchange protein DsbE
OG_00443	LA29479_1888	ku protein
OG_00444	LA29479_0248	subtilase family protein
OG_00445	LA29479_1649	rickettsia 17 kDa surface antigen family protein
OG_00446	LA29479_3354	propionate--CoA ligase
OG_00447	LA29479_2520	glyoxalase/Bleomycin resistance /Dioxygenase superfamily protein
OG_00448	LA29479_2169	response regulator
OG_00449	LA29479_1409	pyridine nucleotide-disulphide oxidoreductase family protein
OG_00450	LA29479_4765	NADPH-dependent FMN reductase family protein
OG_00451	LA29479_4493	diguanylate cyclase domain protein
OG_00452	LA29479_0702	bolA superfamily transcriptional regulator
OG_00453	LA29479_3918	tonB-dependent Receptor Plug domain protein
OG_00454	LA29479_3814	isoaspartyl peptidase
OG_00455	LA29479_1721	cyclic peptide transporter family protein
OG_00456	LA29479_2119	S-adenosylmethionine:2-demethylmenaquinone methyltransferase
OG_00457	LA29479_4905	ribosomal protein L32
OG_00458	LA29479_3994	yqey-like family protein
OG_00459	LA29479_0536	nitrogen regulatory P-II family protein
OG_00460	LA29479_2272	NUDIX domain protein
OG_00461	LA29479_2308	ABC transporter family protein
OG_00462	LA29479_0763	alpha/beta hydrolase fold family protein
OG_00463	LA29479_1776	single-stranded-DNA-specific exonuclease RecJ
OG_00464	LA29479_1676	amidohydrolase family protein
OG_00465	LA29479_3215	inositol monophosphatase family protein
OG_00466	LA29479_0011	methylmalonyl-CoA mutase family protein
OG_00467	LA29479_2864	bacterial regulatory helix-turn-helix , lysR family protein
OG_00468	LA29479_1248	pili assembly chaperone
OG_00469	LA29479_1466	carbon storage regulator
OG_00470	LA29479_0845	dihydropteroate synthase
OG_00471	LA29479_1565	WD40-like Beta Propeller Repeat family protein
OG_00472	LA29479_3960	tonB family C-terminal domain protein
OG_00473	LA29479_1825	PA14 domain protein
OG_00474	LA29479_4738	pseudouridine synthase, RluA family protein
OG_00475	LA29479_3951	2Fe-2S iron-sulfur cluster binding domain protein
OG_00476	LA29479_4283	riboflavin synthase, alpha subunit
OG_00477	LA29479_4467	pfkB carbohydrate kinase family protein
OG_00478	LA29479_0676	bacterial regulatory helix-turn-helix , lysR family protein
OG_00479	LA29479_2064	mannose-1-phosphate guanylyltransferase/mannose-6-phosphate isc
OG_00480	LA29479_1289	short chain dehydrogenase family protein
OG_00481	LA29479_0524	tetratricopeptide repeat family protein
OG_00482	LA29479_2094	acetyltransferase family protein
OG_00483	LA29479_0805	putative uncharacterized protein
OG_00484	LA29479_4772	ribonucleotide reductase, small chain family protein
OG_00485	LA29479_1288	efflux transporter, RND family, MFP subunit
OG_00486	LA29479_2023	peptidase M23 family protein
OG_00487	LA29479_0503	cobQ/CobB/MinD/ParA nucleotide binding domain protein
OG_00488	LA29479_4981	bacterial membrane flanked domain protein
OG_00489	LA29479_3425	HIT domain protein
OG_00490	LA29479_1075	asparaginyl-tRNA synthetase
OG_00491	LA29479_0264	conserved hypothetical protein
OG_00492	LA29479_3863	carbon-nitrogen hydrolase family protein
OG_00493	LA29479_2775	mechanosensitive ion channel family protein
OG_00494	LA29479_1935	exsB family protein
OG_00495	LA29479_2313	his Kinase A domain protein
OG_00496	LA29479_3044	efflux transporter, RND family, MFP subunit

OG_00416	LA76x_1915	yaeT; outer membrane assembly complex, YaeT protein
OG_00417	LA76x_2174	LA76x_2174; hydrolyase, tartrate alpha subunit/fumarate, Fe-S type c
OG_00418	LA76x_2255	yedZ; conserved inner membrane protein
OG_00419	LA76x_3268	proB; glutamate 5-kinase
OG_00420	LA76x_1685	LA76x_1685; prolyl oligopeptidase family protein
OG_00421	LA76x_312	LA76x_312; ecsC family protein
OG_00422	LA76x_4292	LA76x_4292; cupin 2, conserved barrel domain protein
OG_00423	LA76x_2466	LA76x_2466; EF-P lysine aminoacylase GenX
OG_00424	LA76x_805	LA76x_805; putative transmembrane protein
OG_00425	LA76x_2047	LA76x_2047; conserved hypothetical family protein
OG_00426	LA76x_1165	LA76x_1165; mraZ family protein
OG_00427	LA76x_3226	hisC; histidinol-phosphate transaminase
OG_00428	LA76x_4386	atpA; ATP synthase F1, alpha subunit
OG_00429	LA76x_903	fabR; fabR transcriptional repressor FabR transcriptional dual regulat
OG_00430	LA76x_4440	LA76x_4440; putative efflux pump outer membrane protein ttgC
OG_00431	LA76x_2094	rimO; ribosomal protein S12 methylthiotransferase RimO
OG_00432	LA76x_1183	secA; preprotein translocase, SecA subunit
OG_00433	LA76x_3529	LA76x_3529; lactonase, 7-bladed beta-propeller family protein
OG_00434	LA76x_4249	ilvC; ketol-acid reductoisomerase
OG_00435	LA76x_2427	LA76x_2427; bacterial regulatory, tetR family protein
OG_00436	LA76x_1541	LA76x_1541; hypothetical protein
OG_00437	LA76x_3585	LA76x_3585; pirin family protein
OG_00438	LA76x_1435	LA76x_1435; pfkB carbohydrate kinase family protein
OG_00439	LA76x_3456	LA76x_3456; PAP2 superfamily protein
OG_00440	LA76x_4791	LA76x_4791; putative ATP synthase yscN
OG_00441	LA76x_2402	LA76x_2402; transglycosylase SLT domain protein
OG_00442	LA76x_2387	dsbE; disulfide oxidoreductases, DsbE subfamily protein
OG_00443	LA76x_2743	LA76x_2743; ku protein
OG_00444	LA76x_2941	LA76x_2941; subtilase family protein
OG_00445	LA76x_2186	LA76x_2186; glycine zipper 2TM domain protein
OG_00446	LA76x_3879	prpE; propionate--CoA ligase
OG_00447	LA76x_4475	LA76x_4475; glyoxalase/Bleomycin resistance /Dioxygenase superfa
OG_00448	LA76x_986	LA76x_986; transcriptional regulatory , C terminal family protein
OG_00449	LA76x_2095	LA76x_2095; flavo, HI0933 family protein
OG_00450	LA76x_2265	LA76x_2265; NADPH-dependent FMN reductase family protein
OG_00451	LA76x_4666	LA76x_4666; diguanylate cyclase domain protein
OG_00452	LA76x_1573	LA76x_1573; bolA superfamily transcriptional regulator
OG_00453	LA76x_748	LA76x_748; tonB-dependent Receptor Plug domain protein
OG_00454	LA76x_4755	LA76x_4755; iaaA
OG_00455	LA76x_2113	LA76x_2113; cyclic peptide transporter family protein
OG_00456	LA76x_1032	rraA; regulator of ribonuclease activity A
OG_00457	LA76x_3504	rpmF; ribosomal protein L32
OG_00458	LA76x_4581	LA76x_4581; yqey-like family protein
OG_00459	LA76x_4826	LA76x_4826; nitrogen regulatory P-II family protein
OG_00460	LA76x_4337	nudC; NADH pyrophosphatase
OG_00461	LA76x_4371	LA76x_4371; ABC transporter family protein
OG_00462	LA76x_1513	LA76x_1513; alpha/beta hydrolase fold family protein
OG_00463	LA76x_2853	recJ; single-stranded-DNA-specific exonuclease RecJ
OG_00464	LA76x_2157	LA76x_2157; amidohydrolase family protein
OG_00465	LA76x_1810	LA76x_1810; inositol monophosphatase family protein
OG_00466	LA76x_3173	LA76x_3173; methylmalonyl-CoA mutase N-terminal domain protein
OG_00467	LA76x_669	LA76x_669; bacterial regulatory helix-turn-helix , lysR family protein
OG_00468	LA76x_123	LA76x_123; gram-negative pili assembly chaperone, N-terminal doma
OG_00469	LA76x_2037	csrA; carbon storage regulator
OG_00470	LA76x_3684	folP; dihydropteroate synthase
OG_00471	LA76x_1942	LA76x_1942; WD40-like Beta Propeller Repeat family protein
OG_00472	LA76x_791	LA76x_791; tonB family C-terminal domain protein
OG_00473	LA76x_2802	LA76x_2802; PA14 domain protein
OG_00474	LA76x_2293	yceC; 23S rRNA pseudouridine synthase
OG_00475	LA76x_782	LA76x_782; 2Fe-2S iron-sulfur cluster binding domain protein
OG_00476	LA76x_1152	ribE; riboflavin synthase, alpha subunit
OG_00477	LA76x_4691	LA76x_4691; pfkB carbohydrate kinase family protein
OG_00478	LA76x_1598	LA76x_1598; bacterial regulatory helix-turn-helix , lysR family protein
OG_00479	LA76x_1088	LA76x_1088; mannose-1-phosphate guanylyltransferase/mannose-6-
OG_00480	LA76x_82	LA76x_82; short chain dehydrogenase family protein
OG_00481	LA76x_4837	LA76x_4837; tetratricopeptide repeat family protein
OG_00482	LA76x_1058	LA76x_1058; acetyltransferase family protein
OG_00483	LA76x_3649	LA76x_3649; conserved hypothetical protein
OG_00484	LA76x_4117	LA76x_4117; ribonucleotide reductase, small chain family protein
OG_00485	LA76x_83	LA76x_83; efflux transporter, RND family, MFP subunit
OG_00486	LA76x_1182	LA76x_1182; peptidase M23 family protein
OG_00487	LA76x_4857	LA76x_4857; cobQ/CobB/MinD/ParA nucleotide binding domain prote
OG_00488	LA76x_371	LA76x_371; bacterial PH domain protein
OG_00489	LA76x_4211	LA76x_4211; HIT domain protein
OG_00490	LA76x_2564	asnS; asparagine--tRNA ligase
OG_00491	LA76x_2924	LA76x_2924; conserved hypothetical protein
OG_00492	LA76x_3563	LA76x_3563; carbon-nitrogen hydrolase family protein
OG_00493	LA76x_2358	LA76x_2358; mechanosensitive ion channel family protein
OG_00494	LA76x_1264	queC; queuosine biosynthesis protein QueC
OG_00495	LA76x_4376	LA76x_4376; his Kinase A domain protein
OG_00496	LA76x_860	LA76x_860; efflux transporter, RND family, MFP subunit

OG_00497	LA29479_2577	putative uncharacterized protein
OG_00498	LA29479_2055	putative uncharacterized domain protein
OG_00499	LA29479_1447	ferredoxin 1
OG_00500	LA29479_0644	bacterial regulatory helix-turn-helix s, AraC family protein
OG_00501	LA29479_1084	putative transmembrane protein
OG_00502	LA29479_1575	protein YffB
OG_00503	LA29479_1259	cog3541, nucleotidyltransferase protein
OG_00504	LA29479_5021	bacterial extracellular solute-binding s, 5 Middle family protein
OG_00505	LA29479_3658	dehydroquinase class II family protein
OG_00506	LA29479_1674	tonB-dependent Receptor Plug domain protein
OG_00507	LA29479_3300	response regulator
OG_00508	LA29479_4688	cheW-like domain protein
OG_00509	LA29479_3303	FHA domain protein
OG_00510	LA29479_3282	putative uncharacterized protein
OG_00511	LA29479_0811	bacterial regulatory s, gntR family protein
OG_00512	LA29479_1550	putative uncharacterized protein
OG_00513	LA29479_1857	DNA translocase ftsK
OG_00514	LA29479_2555	small Multidrug Resistance family protein
OG_00515	LA29479_4628	putative uncharacterized protein
OG_00516	LA29479_2185	RNA 3'-phosphate cyclase
OG_00517	LA29479_3669	elongation factor P (EF-P) OB domain protein
OG_00518	LA29479_2822	carbon-nitrogen hydrolase family protein
OG_00519	LA29479_5020	glutamate--cysteine ligase
OG_00520	LA29479_3833	his Kinase A domain protein
OG_00521	LA29479_2054	mce related family protein
OG_00522	LA29479_0448	gluconate periplasmic binding protein with phosphoribosyltransferase
OG_00523	LA29479_1385	ABC transporter family protein
OG_00524	LA29479_5017	bacterial regulatory helix-turn-helix , lysR family protein
OG_00525	LA29479_2587	putative uncharacterized protein
OG_00526	LA29479_4615	phosphoglycerate kinase
OG_00527	LA29479_1435	uncharacterised P-loop hydrolase UPF0079 family protein
OG_00528	LA29479_0776	hypothetical protein
OG_00529	LA29479_0542	putative uncharacterized protein
OG_00530	LA29479_1969	inner membrane protein ybaL
OG_00531	LA29479_0879	FAD dependent oxidoreductase family protein
OG_00532	LA29479_4718	beta-hexosaminidase
OG_00533	LA29479_3144	ATP-dependent Clp protease, ATP-binding subunit ClpX
OG_00534	LA29479_0666	flavodoxin family protein
OG_00535	LA29479_2733	P-type DNA transfer ATPase VirB11
OG_00536	LA29479_3574	hypothetical protein
OG_00537	LA29479_3660	2,3-diketo-5-methylthio-1-phosphopentane phosphatase
OG_00538	LA29479_2429	conserved hypothetical protein
OG_00539	LA29479_2229	bacterial regulatory helix-turn-helix , lysR family protein
OG_00540	LA29479_0930	cyclase family protein
OG_00541	LA29479_1685	cyclopropane-fatty-acyl-phospholipid synthase
OG_00542	LA29479_0074	beta-lytic metalloendopeptidase
OG_00543	LA29479_4705	diguanylate cyclase domain protein
OG_00544	LA29479_0905	methyltransferase domain protein
OG_00545	LA29479_0987	glutamyl-tRNA synthetase
OG_00546	LA29479_4195	bacterial regulatory helix-turn-helix , lysR family protein
OG_00547	LA29479_3661	acireductone dioxygenase
OG_00548	LA29479_4108	putative membrane protein
OG_00549	LA29479_4385	glycine cleavage system T protein
OG_00550	LA29479_4314	protein disulfide isomerase
OG_00551	LA29479_4092	SMF family protein
OG_00552	LA29479_0739	periplasmic binding family protein
OG_00553	LA29479_3428	peptidase M20/M25/M40 family protein
OG_00554	LA29479_2042	ribosomal RNA small subunit methyltransferase I
OG_00555	LA29479_2517	putative uncharacterized protein
OG_00556	LA29479_0602	glycosyl hydrolases 31 family protein
OG_00557	LA29479_4300	general secretion pathway protein D
OG_00558	LA29479_0004	outer membrane autotransporter barrel domain protein
OG_00559	LA29479_2473	asnC family protein
OG_00560	LA29479_3480	bacterial regulatory s, tetR family protein
OG_00561	LA29479_4492	isocitrate lyase
OG_00562	LA29479_4157	ATPase associated with various cellular activities family protein
OG_00563	LA29479_5008	GHMP kinases N terminal domain protein
OG_00564	LA29479_1137	putative exported domain protein
OG_00565	LA29479_2309	permease family protein
OG_00566	LA29479_3890	N-acetylmuramoyl-L-alanine amidase family protein
OG_00567	LA29479_4562	diguanylate cyclase domain protein
OG_00568	LA29479_0505	ATPase associated with various cellular activities family protein
OG_00569	LA29479_1944	tol-pal system-associated acyl-CoA thioesterase
OG_00570	LA29479_2645	methyltransferase domain protein
OG_00571	LA29479_0974	bacterial regulatory helix-turn-helix s, AraC family protein
OG_00572	LA29479_1490	signal recognition particle protein
OG_00573	LA29479_1836	rpfF protein
OG_00574	LA29479_4223	putative Fe(2+)-trafficking protein
OG_00575	LA29479_2048	prolyl-tRNA synthetase
OG_00576	LA29479_4740	response regulator
OG_00577	LA29479_4590	tetratricopeptide repeat family protein

OG_00497	LA76x_4528	LA76x_4528; conserved hypothetical protein
OG_00498	LA76x_1097	LA76x_1097; conserved hypothetical protein
OG_00499	LA76x_2059	LA76x_2059; 4Fe-4S binding domain protein
OG_00500	LA76x_1631	LA76x_1631; bacterial regulatory helix-turn-helix, AraC family protein
OG_00501	LA76x_2556	LA76x_2556; putative transmembrane protein
OG_00502	LA76x_1933	LA76x_1933; transcriptional regulator, Spx/MgsR family protein
OG_00503	LA76x_111	LA76x_111; putative nucleotidyltransferase family protein
OG_00504	LA76x_4550	LA76x_4550; bacterial extracellular solute-binding, 5 Middle family pr
OG_00505	LA76x_3216	LA76x_3216; dehydroquinase class II family protein
OG_00506	LA76x_2159	LA76x_2159; tonB dependent receptor family protein
OG_00507	LA76x_184	LA76x_184; bacterial regulatory, luxR family protein
OG_00508	LA76x_4073	LA76x_4073; cheW-like domain protein
OG_00509	LA76x_181	LA76x_181; FHA domain protein
OG_00510	LA76x_198	LA76x_198; conserved hypothetical protein
OG_00511	LA76x_3654	LA76x_3654; bacterial regulatory, gntR family protein
OG_00512	LA76x_1954	LA76x_1954; conserved hypothetical protein
OG_00513	LA76x_2773	ftsK; DNA translocase ftsK
OG_00514	LA76x_4507	LA76x_4507; small Multidrug Resistance family protein
OG_00515	LA76x_3438	LA76x_3438; pectinacetylesterase family protein
OG_00516	LA76x_970	rtcA; RNA 3'-phosphate cyclase
OG_00517	LA76x_3206	LA76x_3206; elongation factor P (EF-P) OB domain protein
OG_00518	LA76x_2311	LA76x_2311; carbon-nitrogen hydrolase family protein
OG_00519	LA76x_4549	LA76x_4549; glutamate--cysteine ligase
OG_00520	LA76x_4737	LA76x_4737; his Kinase A domain protein
OG_00521	LA76x_1098	LA76x_1098; mce related family protein
OG_00522	LA76x_4911	LA76x_4911; phosphoribosyl transferase domain protein
OG_00523	LA76x_5193	LA76x_5193; ABC transporter family protein
OG_00524	LA76x_4545	LA76x_4545; bacterial regulatory helix-turn-helix , lysR family protein
OG_00525	LA76x_4538	LA76x_4538; conserved hypothetical protein
OG_00526	LA76x_4111	pgk; phosphoglycerate kinase
OG_00527	LA76x_2069	LA76x_2069; conserved hypothetical protein
OG_00528	LA76x_3621	LA76x_3621; hypothetical protein
OG_00529	LA76x_1724	LA76x_1724; conserved hypothetical protein
OG_00530	LA76x_1231	LA76x_1231; proton antiporter-2 family protein
OG_00531	LA76x_3718	LA76x_3718; FAD binding domain protein
OG_00532	LA76x_3474	nagZ; beta-hexosaminidase
OG_00533	LA76x_1875	clpX; ATP-dependent Clp protease, ATP-binding subunit ClpX
OG_00534	LA76x_1609	LA76x_1609; flavodoxin family protein
OG_00535	LA76x_2401	virB11; P-type DNA transfer ATPase VirB11
OG_00536	LA76x_3982	LA76x_3982; hypothetical protein
OG_00537	LA76x_3214	mtnC; 2,3-diketo-5-methylthio-1-phosphopentane phosphatase
OG_00538	LA76x_3398	LA76x_3398; conserved hypothetical protein
OG_00539	LA76x_4296	LA76x_4296; bacterial regulatory helix-turn-helix , lysR family protein
OG_00540	LA76x_3770	LA76x_3770; cyclase family protein
OG_00541	LA76x_2148	cfa; cfa
OG_00542	LA76x_3114	LA76x_3114; beta-lytic metalloendopeptidase
OG_00543	LA76x_3461	LA76x_3461; diguanylate cyclase domain protein
OG_00544	LA76x_3745	LA76x_3745; methyltransferase domain protein
OG_00545	LA76x_3830	gltX; glutamate--tRNA ligase
OG_00546	LA76x_4188	LA76x_4188; bacterial regulatory helix-turn-helix , lysR family protein
OG_00547	LA76x_3213	mtnD; acireductone dioxygenase
OG_00548	LA76x_345	LA76x_345; conserved hypothetical protein
OG_00549	LA76x_1452	gcvT; glycine cleavage system T protein
OG_00550	LA76x_1121	LA76x_1121; thioredoxin family protein
OG_00551	LA76x_361	dprA; DNA protecting protein DprA
OG_00552	LA76x_1536	LA76x_1536; periplasmic binding family protein
OG_00553	LA76x_4213	LA76x_4213; peptidase M20/M25/M40 family protein
OG_00554	LA76x_1164	yraL; 16S rRNA 2'-O-ribose C1402 methyltransferase
OG_00555	LA76x_4472	LA76x_4472; conserved hypothetical protein
OG_00556	LA76x_1665	LA76x_1665; glycosyl hydrolases 31 family protein
OG_00557	LA76x_1135	gspD; type II secretion system protein D
OG_00558	LA76x_3180	LA76x_3180; autotransporter beta-domain protein
OG_00559	LA76x_4427	LA76x_4427; asnC family protein
OG_00560	LA76x_4263	LA76x_4263; bacterial regulatory, tetR family protein
OG_00561	LA76x_4667	aceA; isocitrate lyase
OG_00562	LA76x_4150	LA76x_4150; AAA domain family protein
OG_00563	LA76x_4710	LA76x_4710; GHMP kinase N terminal domain protein
OG_00564	LA76x_2507	LA76x_2507; conserved hypothetical protein
OG_00565	LA76x_4372	LA76x_4372; ftsX-like permease family protein
OG_00566	LA76x_3591	LA76x_3591; N-acetylmuramoyl-L-alanine amidase family protein
OG_00567	LA76x_1437	LA76x_1437; diguanylate cyclase domain protein
OG_00568	LA76x_4854	LA76x_4854; ATPase associated with various cellular activities family
OG_00569	LA76x_1255	ybgC; tol-pal system-associated acyl-CoA thioesterase
OG_00570	LA76x_570	LA76x_570; methyltransferase domain protein
OG_00571	LA76x_3818	LA76x_3818; alkA N-terminal domain protein
OG_00572	LA76x_2012	ffh; signal recognition particle protein
OG_00573	LA76x_2794	LA76x_2794; enoyl-CoA hydratase/isomerase family protein
OG_00574	LA76x_1780	yggX; that protects iron-sulfur proteins against oxidative damage
OG_00575	LA76x_1104	proS; proline--tRNA ligase
OG_00576	LA76x_2291	LA76x_2291; bacterial regulatory, luxR family protein
OG_00577	LA76x_4086	LA76x_4086; putative secreted protein

OG_00578	LA29479_3852	peptidase M48 family protein
OG_00579	LA29479_3399	ribosomal protein S19
OG_00580	LA29479_4389	RNA polymerase sigma factor, sigma-70 family protein
OG_00581	LA29479_1483	hypothetical protein
OG_00582	LA29479_1856	alanine dehydrogenase
OG_00583	LA29479_0551	tRNA-i(6)A37 thiotransferase enzyme MiaB
OG_00584	LA29479_4180	guanylate kinase
OG_00585	LA29479_1853	conserved hypothetical protein
OG_00586	LA29479_0606	deoR-like helix-turn-helix domain protein
OG_00587	LA29479_1424	ATPase involved in chromosome partitioning
OG_00588	LA29479_0172	uncharacterized protein yjgR
OG_00589	LA29479_3552	putative uncharacterized protein
OG_00590	LA29479_3414	ribosomal protein L1
OG_00591	LA29479_0155	alpha-2-macroglobulin N-terminal region family protein
OG_00592	LA29479_3195	peptidase M13 family protein
OG_00593	LA29479_2801	nucleoside diphosphate kinase
OG_00594	LA29479_3041	permease family protein
OG_00595	LA29479_1462	sensor protein KdpD
OG_00596	LA29479_2336	conserved hypothetical protein
OG_00597	LA29479_4191	xaa-Pro dipeptidase
OG_00598	LA29479_2032	undecaprenyldiphospho-muramoylpentapeptide beta-N-acetylglucosa
OG_00599	LA29479_4895	aminodeoxychorismate lyase family protein
OG_00600	LA29479_0005	acyl-CoA dehydrogenase, N-terminal domain protein
OG_00601	LA29479_1609	hypothetical protein
OG_00602	LA29479_5161	rod shape-determining protein MreD
OG_00603	LA29479_3156	uncharacterized ybeB domain protein
OG_00604	LA29479_1995	ribosomal large subunit pseudouridine synthase D
OG_00605	LA29479_0925	glyoxalase/Bleomycin resistance /Dioxygenase superfamily protein
OG_00606	LA29479_3788	DGPF domain protein
OG_00607	LA29479_2003	his Kinase A domain protein
OG_00608	LA29479_2978	osmC-like family protein
OG_00609	LA29479_0762	putative uncharacterized protein
OG_00611	LA29479_4270	oar protein
OG_00612	LA29479_1436	uncharacterized protein yjeF
OG_00613	LA29479_4503	glyoxalase/Bleomycin resistance /Dioxygenase superfamily protein
OG_00614	LA29479_4112	putative uncharacterized protein
OG_00615	LA29479_0136	phage Tail Collar domain protein
OG_00616	LA29479_4987	ribosomal RNA small subunit methyltransferase B
OG_00617	LA29479_1217	surface antigen family protein
OG_00618	LA29479_4834	acetoacetyl-CoA reductase
OG_00619	LA29479_4687	methyl-accepting chemotaxis (MCP) signaling domain protein
OG_00620	LA29479_3645	diguanylate cyclase domain protein
OG_00621	LA29479_4960	prolipoprotein diacylglyceryl transferase
OG_00622	LA29479_1059	acyltransferase, WS/DGAT/MGAT family protein
OG_00623	LA29479_1761	ferric uptake regulation protein
OG_00624	LA29479_3382	30S ribosomal protein S13
OG_00625	LA29479_2253	hypothetical protein
OG_00626	LA29479_4984	glycosyl transferase 2 family protein
OG_00627	LA29479_3616	ribosomal RNA large subunit methyltransferase M
OG_00628	LA29479_3809	inner membrane protein alx
OG_00629	LA29479_3122	hypothetical protein
OG_00630	LA29479_2783	GMP synthase [glutamine-hydrolyzing]
OG_00631	LA29479_1363	motA/TolQ/ExbB proton channel family protein
OG_00632	LA29479_2310	permease family protein
OG_00633	LA29479_5016	tetratricopeptide repeat family protein
OG_00634	LA29479_4120	hypothetical protein
OG_00635	LA29479_2089	maleylacetoacetate isomerase
OG_00636	LA29479_4553	putative uncharacterized protein
OG_00637	LA29479_4018	outer membrane transport family protein
OG_00638	LA29479_5067	tat (twin-arginine translocation) pathway signal sequence domain prot
OG_00639	LA29479_1628	lipid A export permease/ATP-binding protein MsbA
OG_00640	LA29479_2294	putative uncharacterized protein
OG_00641	LA29479_0582	oligopeptide transporter, OPT family
OG_00642	LA29479_0502	phosphoglycerate mutase family protein
OG_00643	LA29479_1767	chaperone protein DnaK
OG_00644	LA29479_4988	methionyl-tRNA formyltransferase
OG_00645	LA29479_0630	hypothetical protein
OG_00646	LA29479_4510	magnesium chelatase, subunit ChlI family protein
OG_00647	LA29479_3372	coenzyme PQQ biosynthesis protein E
OG_00648	LA29479_3874	acetyltransferase family protein
OG_00649	LA29479_0201	triose-phosphate isomerase
OG_00650	LA29479_1062	alanine racemase
OG_00651	LA29479_2768	radical SAM domain protein
OG_00652	LA29479_1180	putative uncharacterized protein
OG_00653	LA29479_4697	ATP-dependent DNA helicase protein
OG_00654	LA29479_1468	regulatory recX domain protein
OG_00655	LA29479_0099	PAAR motif family protein
OG_00656	LA29479_2691	hypothetical protein
OG_00657	LA29479_2865	3-oxoadipate enol-lactonase
OG_00658	LA29479_2765	calU3
OG_00659	LA29479_4429	uncharacterized protein yqiC

OG_00578	LA76x_3553	LA76x_3553; peptidase M48 family protein
OG_00579	LA76x_3924	rpsS; ribosomal protein S19
OG_00580	LA76x_1448	LA76x_1448; RNA polymerase sigma factor, sigma-70 family protein
OG_00581	LA76x_2019	LA76x_2019; hypothetical protein
OG_00582	LA76x_2774	ald; alanine dehydrogenase
OG_00583	LA76x_1714	miaB; tRNA-i(6)A37 thiotransferase enzyme MiaB
OG_00584	LA76x_4174	gmk; guanylate kinase
OG_00585	LA76x_2777	LA76x_2777; acetyltransferase domain protein
OG_00586	LA76x_1661	LA76x_1661; deoR-like helix-turn-helix domain protein
OG_00587	LA76x_2080	LA76x_2080; cobQ/CobB/MinD/ParA nucleotide binding domain prote
OG_00588	LA76x_3018	LA76x_3018; putative ATPase
OG_00589	LA76x_3962	LA76x_3962; conserved hypothetical protein
OG_00590	LA76x_3940	rplA; ribosomal protein L1
OG_00591	LA76x_3032	LA76x_3032; alpha-2-macroglobulin MG1 domain protein
OG_00592	LA76x_1829	LA76x_1829; peptidase M13 family protein
OG_00593	LA76x_2332	LA76x_2332; nucleoside diphosphate kinase family protein
OG_00594	LA76x_857	LA76x_857; ftsX-like permease family protein
OG_00595	LA76x_2042	kdpD; sensor protein KdpD
OG_00596	LA76x_4401	LA76x_4401; eamA-like transporter family protein
OG_00597	LA76x_4184	pepQ; xaa-Pro dipeptidase
OG_00598	LA76x_1173	murG; undecaprenyldiphospho-muramoylpentapeptide beta-N-acetylg
OG_00599	LA76x_3496	LA76x_3496; yceG-like family protein
OG_00600	LA76x_3179	LA76x_3179; acyl-CoA dehydrogenase, N-terminal domain protein
OG_00601	LA76x_2226	LA76x_2226; hypothetical protein
OG_00602	LA76x_831	mreD; rod shape-determining protein MreD
OG_00603	LA76x_1864	LA76x_1864; oligomerisation domain protein
OG_00604	LA76x_1207	LA76x_1207; 23S rRNA pseudouridine synthase
OG_00605	LA76x_3765	LA76x_3765; glyoxalase/Bleomycin resistance /Dioxygenase superfa
OG_00606	LA76x_4776	LA76x_4776; YCII-related domain protein
OG_00607	LA76x_1200	LA76x_1200; his Kinase A domain protein
OG_00608	LA76x_2669	LA76x_2669; osmC-like family protein
OG_00609	LA76x_1514	LA76x_1514; conserved hypothetical protein
OG_00611	LA76x_1733	LA76x_1733; cna B-type domain protein
OG_00612	LA76x_2068	LA76x_2068; putative carbohydrate kinase
OG_00613	LA76x_4655	LA76x_4655; glyoxalase/Bleomycin resistance /Dioxygenase superfa
OG_00614	LA76x_341	LA76x_341; hypothetical protein
OG_00615	LA76x_3051	LA76x_3051; phage Tail Collar domain protein
OG_00616	LA76x_365	sun; ribosomal RNA small subunit methyltransferase B
OG_00617	LA76x_150	LA76x_150; surface antigen family protein
OG_00618	LA76x_3301	phbB; acetoacetyl-CoA reductase family protein
OG_00619	LA76x_4072	LA76x_4072; methyl-accepting chemotaxis (MCP) signalling domain
OG_00620	LA76x_3230	LA76x_3230; diguanylate cyclase domain protein
OG_00621	LA76x_4047	lgt; prolipoprotein diacylglyceryl transferase
OG_00622	LA76x_2583	LA76x_2583; acyltransferase, WS/DGAT/MGAT family protein
OG_00623	LA76x_2868	fur; fur
OG_00624	LA76x_3907	rpsM; 30S ribosomal protein S13
OG_00625	LA76x_4320	LA76x_4320; hypothetical protein
OG_00626	LA76x_368	LA76x_368; glycosyl transferase 2 family protein
OG_00627	LA76x_4024	mtfA; 23S rRNA 2'-O-ribose C2498 methyltransferase
OG_00628	LA76x_4760	LA76x_4760; integral membrane , TerC family protein
OG_00629	LA76x_934	LA76x_934; hypothetical protein
OG_00630	LA76x_2349	guaA; guaA GMP synthetase
OG_00631	LA76x_9	LA76x_9; motA/TolQ/ExbB proton channel family protein
OG_00632	LA76x_4373	LA76x_4373; ftsX-like permease family protein
OG_00633	LA76x_4544	LA76x_4544; tetratricopeptide repeat family protein
OG_00634	LA76x_334	LA76x_334; hypothetical protein
OG_00635	LA76x_1063	maiA; maleylacetoacetate isomerase
OG_00636	LA76x_1445	LA76x_1445; conserved hypothetical protein
OG_00637	LA76x_4605	LA76x_4605; outer membrane beta-barrel domain protein
OG_00638	LA76x_4811	LA76x_4811; ahpC/TSA family protein
OG_00639	LA76x_2207	msbA; lipid A export permease/ATP-binding protein MsbA
OG_00640	LA76x_4357	LA76x_4357; conserved hypothetical protein
OG_00641	LA76x_1684	LA76x_1684; oligopeptide transporter, OPT family
OG_00642	LA76x_4858	LA76x_4858; histidine phosphatase super family protein
OG_00643	LA76x_2863	dnaK; chaperone protein DnaK
OG_00644	LA76x_364	fmt; methionyl-tRNA formyltransferase
OG_00645	LA76x_1645	LA76x_1645; alpha/beta hydrolase family protein
OG_00646	LA76x_4648	LA76x_4648; AAA domain family protein
OG_00647	LA76x_3898	pqqE; coenzyme PQQ biosynthesis enzyme PqqE
OG_00648	LA76x_3575	LA76x_3575; acetyltransferase family protein
OG_00649	LA76x_2989	tpiA; triose-phosphate isomerase
OG_00650	LA76x_2580	alr; alanine racemase
OG_00651	LA76x_2366	LA76x_2366; putative calU5
OG_00652	LA76x_2467	LA76x_2467; conserved hypothetical protein
OG_00653	LA76x_4082	LA76x_4082; DEAD/DEAH box helicase family protein
OG_00654	LA76x_2035	recX; regulatory recX domain protein
OG_00655	LA76x_3089	LA76x_3089; PAAR motif family protein
OG_00656	LA76x_618	LA76x_618; hypothetical protein
OG_00657	LA76x_670	pcaD; 3-oxoadipate enol-lactonase
OG_00658	LA76x_2369	LA76x_2369; putative calU3
OG_00659	LA76x_4647	LA76x_4647; membrane fusogenic activity family protein

OG_00660	LA29479_3054	ribonucleoside-diphosphate reductase
OG_00661	LA29479_3132	asuH1
OG_00662	LA29479_0115	inner membrane metabolite transport protein yhjE
OG_00663	LA29479_1732	deoR-like helix-turn-helix domain protein
OG_00664	LA29479_3843	tetratricopeptide repeat family protein
OG_00665	LA29479_2698	bacterial regulatory helix-turn-helix s, AraC family protein
OG_00666	LA29479_2516	uncharacterised UPF0066 family protein
OG_00667	LA29479_4146	glycine cleavage system transcriptional activator
OG_00668	LA29479_4920	prolyl oligopeptidase family protein
OG_00669	LA29479_2251	inner membrane protein ynbA
OG_00670	LA29479_3648	histidinol dehydrogenase
OG_00671	LA29479_1782	conserved hypothetical protein
OG_00672	LA29479_0561	hlyD secretion family protein
OG_00673	LA29479_0625	aspartate kinase domain protein
OG_00674	LA29479_0631	peroxiredoxin osmC
OG_00675	LA29479_1760	outer membrane protein
OG_00676	LA29479_3439	glyoxalase/Bleomycin resistance /Dioxygenase superfamily protein
OG_00677	LA29479_2914	putative transmembrane domain protein
OG_00678	LA29479_1861	acetyltransferase family protein
OG_00679	LA29479_1658	conserved hypothetical protein
OG_00680	LA29479_4153	cell division ATP-binding protein FtsE
OG_00681	LA29479_4648	exodeoxyribonuclease VII, large subunit
OG_00682	LA29479_1820	N(4)-(beta-N-acetylglucosaminyl)-L-asparaginase
OG_00683	LA29479_4174	putative uncharacterized protein
OG_00684	LA29479_4045	coproporphyrinogen-III oxidase, aerobic
OG_00686	LA29479_4444	putative membrane protein
OG_00687	LA29479_3473	3-isopropylmalate dehydrogenase
OG_00688	LA29479_1083	putative uncharacterized protein
OG_00689	LA29479_4139	PAP2 superfamily protein
OG_00690	LA29479_5010	phosphoribosylglycinamide synthetase, ATP-grasp domain protein
OG_00691	LA29479_0208	NADH-quinone oxidoreductase, E subunit
OG_00692	LA29479_2567	uncharacterized aminotransferase YfbQ
OG_00693	LA29479_0521	glucose/galactose transporter WARNING family protein
OG_00694	LA29479_0491	phosphogluconate dehydratase
OG_00695	LA29479_0714	hypothetical protein
OG_00696	LA29479_4347	monofunctional biosynthetic peptidoglycan transglycosylase
OG_00697	LA29479_4260	excinuclease ABC, B subunit
OG_00698	LA29479_2245	acyltransferase family protein
OG_00699	LA29479_3443	V-type H(+)-translocating pyrophosphatase
OG_00700	LA29479_2461	glutaminyl-peptide cyclotransferase
OG_00701	LA29479_2075	NAD dependent epimerase/dehydratase family protein
OG_00702	LA29479_3880	pirin C-terminal cupin domain protein
OG_00703	LA29479_1029	glycoside hydrolase family 9
OG_00704	LA29479_3915	acetyltransferase family protein
OG_00705	LA29479_3949	cytidylyltransferase-like enzyme
OG_00706	LA29479_1160	putative quinone oxidoreductase YhfP
OG_00707	LA29479_2044	hypothetical protein
OG_00708	LA29479_1976	cobinamide kinase / cobinamide phosphate guanyltransferase family
OG_00709	LA29479_4550	conserved hypothetical protein
OG_00710	LA29479_4470	tonB-dependent Receptor Plug domain protein
OG_00711	LA29479_3900	ADP-ribosylglycohydrolase
OG_00712	LA29479_4810	acetylglutamate kinase
OG_00713	LA29479_4122	glutathione S-transferase, C-terminal domain protein
OG_00714	LA29479_3461	threonine synthase
OG_00715	LA29479_3266	cobQ/CobB/MinD/ParA nucleotide binding domain protein
OG_00716	LA29479_0180	phosphatidylserine decarboxylase
OG_00717	LA29479_1452	bacterioferritin comigratory protein
OG_00718	LA29479_0546	putative stringent starvation protein A
OG_00719	LA29479_2523	peptidase propeptide and YPEB domain protein
OG_00720	LA29479_0933	bacterial regulatory helix-turn-helix , lysR family protein
OG_00721	LA29479_3533	response regulator
OG_00722	LA29479_2061	3-oxoacid CoA-transferase, B subunit
OG_00723	LA29479_5209	putative uncharacterized protein
OG_00724	LA29479_1486	tRNA (guanine-N1)-methyltransferase
OG_00725	LA29479_3810	putative uncharacterized protein
OG_00726	LA29479_1537	3-hydroxypropionyl-coenzyme A dehydratase
OG_00727	LA29479_0983	marR family protein
OG_00728	LA29479_1430	electron transport complex, RnfABCDGE type, B subunit
OG_00729	LA29479_2017	type IV-A pilus assembly ATPase PilB
OG_00730	LA29479_3348	cell Wall Hydrolase family protein
OG_00731	LA29479_1445	poly(A) polymerase
OG_00732	LA29479_3858	putative membrane protein
OG_00733	LA29479_4233	acyl-CoA dehydrogenase, middle domain protein
OG_00734	LA29479_0912	putative secreted peptidyl prolyl cis-trans isomerase, cyclophilin type
OG_00735	LA29479_4809	acetyltransferase protein
OG_00736	LA29479_3381	30S ribosomal protein S11
OG_00737	LA29479_2954	nlpC/P60 family protein
OG_00738	LA29479_1728	conserved hypothetical protein
OG_00739	LA29479_3319	uracil DNA glycosylase superfamily protein
OG_00740	LA29479_4076	fatty acyl-CoA synthetase
OG_00741	LA29479_4202	cell division ZapA family protein

OG_00660	LA76x_870	LA76x_870; TSCPD domain protein
OG_00661	LA76x_945	LA76x_945; conserved hypothetical protein
OG_00662	LA76x_3071	LA76x_3071; H+ symporter family protein
OG_00663	LA76x_2102	LA76x_2102; deoR-like helix-turn-helix domain protein
OG_00664	LA76x_4728	LA76x_4728; hemY family protein
OG_00665	LA76x_625	LA76x_625; bacterial regulatory helix-turn-helix, AraC family protein
OG_00666	LA76x_4471	LA76x_4471; conserved hypothetical protein
OG_00667	LA76x_308	gcvA; gcvA transcriptional dual regulator
OG_00668	LA76x_806	LA76x_806; putative PEPTIDASE
OG_00669	LA76x_4318	LA76x_4318; CDP-alcohol phosphatidyltransferase family protein
OG_00670	LA76x_3227	LA76x_3227; hisD
OG_00671	LA76x_2847	LA76x_2847; conserved hypothetical protein
OG_00672	LA76x_1704	LA76x_1704; hlyD secretion family protein
OG_00673	LA76x_1650	LA76x_1650; aspartate kinase domain protein
OG_00674	LA76x_1644	LA76x_1644; peroxiredoxin, OsmC subfamily protein
OG_00675	LA76x_2869	smpA; outer membrane protein
OG_00676	LA76x_4223	LA76x_4223; glyoxalase/Bleomycin resistance /Dioxygenase superfa
OG_00677	LA76x_716	LA76x_716; putative transmembrane domain protein
OG_00678	LA76x_2769	LA76x_2769; acetyltransferase family protein
OG_00679	LA76x_2177	LA76x_2177; conserved hypothetical protein
OG_00680	LA76x_301	ftsE; cell division ATP-binding protein FtsE
OG_00681	LA76x_3458	xseA; exodeoxyribonuclease VII, large subunit
OG_00682	LA76x_2807	AGA; N(4)-(beta-N-acetylglucosaminyl)-L-asparaginase
OG_00683	LA76x_4168	LA76x_4168; right handed beta helix region family protein
OG_00684	LA76x_283	LA76x_283; coproporphyrinogen III oxidase family protein
OG_00686	LA76x_4638	LA76x_4638; hypothetical protein
OG_00687	LA76x_4257	leuB; 3-isopropylmalate dehydrogenase
OG_00688	LA76x_2557	LA76x_2557; conserved hypothetical protein
OG_00689	LA76x_315	LA76x_315; PAP2 superfamily protein
OG_00690	LA76x_4708	LA76x_4708; carbamoyl-phosphate synthase L chain, ATP binding dc
OG_00691	LA76x_2982	nuoE; NADH-quinone oxidoreductase, E subunit
OG_00692	LA76x_4519	LA76x_4519; putative aminotransferase
OG_00693	LA76x_4840	gluP; glucose/galactose transporter WARNING family protein
OG_00694	LA76x_4867	edd; phosphogluconate dehydratase
OG_00695	LA76x_1561	LA76x_1561; EF hand family protein
OG_00696	LA76x_1488	mtgA; monofunctional biosynthetic peptidoglycan transglycosylase
OG_00697	LA76x_1742	uvrB; excinuclease ABC subunit B
OG_00698	LA76x_4312	LA76x_4312; acyltransferase family protein
OG_00699	LA76x_4227	LA76x_4227; V-type H(+)-translocating pyrophosphatase
OG_00700	LA76x_3425	QCT; glutaminyl-peptide cyclotransferase
OG_00701	LA76x_1077	LA76x_1077; NAD dependent epimerase/dehydratase family protein
OG_00702	LA76x_3581	LA76x_3581; pirin family protein
OG_00703	LA76x_2615	LA76x_2615; endoglucanase-related protein
OG_00704	LA76x_3614	LA76x_3614; acetyltransferase family protein
OG_00705	LA76x_780	LA76x_780; mobA-like NTP transferase domain protein
OG_00706	LA76x_2485	LA76x_2485; quinone oxidoreductase, YhdH/YhfP family protein
OG_00707	LA76x_1107	LA76x_1107; hypothetical protein
OG_00708	LA76x_1225	LA76x_1225; cobinamide kinase / cobinamide phosphate guanyltrans
OG_00709	LA76x_1884	rlmH; rRNA large subunit m3Psi methyltransferase RlmH
OG_00710	LA76x_4689	LA76x_4689; tonB dependent receptor family protein
OG_00711	LA76x_3600	LA76x_3600; putative aDP-ribosylglycohydrolase
OG_00712	LA76x_3273	argB; acetylglutamate kinase
OG_00713	LA76x_332	LA76x_332; glutathione S-transferase, C-terminal domain protein
OG_00714	LA76x_4246	thrC; threonine synthase
OG_00715	LA76x_214	LA76x_214; cobQ/CobB/MinD/ParA nucleotide binding domain protei
OG_00716	LA76x_3010	psd; phosphatidylserine decarboxylase
OG_00717	LA76x_2053	LA76x_2053; bacterioferritin comigratory protein
OG_00718	LA76x_1720	sspA; stringent starvation protein A
OG_00719	LA76x_4478	LA76x_4478; peptidase propeptide and YPEB domain protein
OG_00720	LA76x_3773	LA76x_3773; bacterial regulatory helix-turn-helix , lysR family protein
OG_00721	LA76x_2703	LA76x_2703; bacterial regulatory , Fis family protein
OG_00722	LA76x_1091	LA76x_1091; 3-oxoacid CoA-transferase, B subunit
OG_00723	LA76x_4149	LA76x_4149; conserved hypothetical protein
OG_00724	LA76x_2016	trmD; tRNA (guanine(37)-N(1))-methyltransferase
OG_00725	LA76x_4759	LA76x_4759; DNA alkylation repair enzyme family protein
OG_00726	LA76x_1967	LA76x_1967; enoyl-CoA hydratase/isomerase family protein
OG_00727	LA76x_3827	LA76x_3827; marR family protein
OG_00728	LA76x_2074	LA76x_2074; electron transport complex, RnfABCDGE type, B subun
OG_00729	LA76x_1188	pilB; type IV-A pilus assembly ATPase PilB
OG_00730	LA76x_3873	LA76x_3873; cell Wall Hydrolase family protein
OG_00731	LA76x_2061	pcnB; poly(A) polymerase I
OG_00732	LA76x_3558	LA76x_3558; glycosyl transferase 2 family protein
OG_00733	LA76x_1769	LA76x_1769; acyl-CoA dehydrogenase, N-terminal domain protein
OG_00734	LA76x_3752	LA76x_3752; cyclophilin type peptidyl-prolyl cis-trans isomerase/CLD
OG_00735	LA76x_3272	LA76x_3272; acetyltransferase domain protein
OG_00736	LA76x_3906	rpsK; 30S ribosomal protein S11
OG_00737	LA76x_745	LA76x_745; SH3 domain of the SH3b1 type family protein
OG_00738	LA76x_2106	LA76x_2106; conserved hypothetical protein
OG_00739	LA76x_167	LA76x_167; uracil DNA glycosylase superfamily protein
OG_00740	LA76x_255	LA76x_255; putative fatty acyl-CoA synthetase
OG_00741	LA76x_4193	LA76x_4193; cell division ZapA family protein

OG_00742	LA29479_0517	glycosyl hydrolase family 20, catalytic domain protein	OG_00742	LA76x_4843	LA76x_4843; chitobiase/beta-hexosaminidase C-terminal domain pro
OG_00743	LA29479_0378	putative uncharacterized protein	OG_00743	LA76x_5069	LA76x_5069; conserved hypothetical protein
OG_00744	LA29479_4271	UPF0056 inner membrane protein yhgN	OG_00744	LA76x_1731	LA76x_1731; marC integral membrane family protein
OG_00745	LA29479_4145	FMN-dependent NADH-azoreductase 1	OG_00745	LA76x_309	azoR1; FMN-dependent NADH-azoreductase 1
OG_00746	LA29479_4275	inner membrane protein ybcI	OG_00746	LA76x_1160	LA76x_1160; conserved hypothetical protein
OG_00747	LA29479_1296	exodeoxyribonuclease V, gamma subunit	OG_00747	LA76x_77	recC; exodeoxyribonuclease V, gamma subunit
OG_00748	LA29479_2736	virB8 family protein	OG_00748	LA76x_2398	LA76x_2398; virB8 family protein
OG_00749	LA29479_2134	hypothetical protein	OG_00749	LA76x_1018	LA76x_1018; hypothetical protein
OG_00750	LA29479_4100	putative secreted protein	OG_00750	LA76x_353	LA76x_353; putative secreted protein
OG_00751	LA29479_2121	dnaJ domain protein	OG_00751	LA76x_1030	LA76x_1030; dnaJ domain protein
OG_00752	LA29479_4448	flavofamily protein	OG_00752	LA76x_4634	LA76x_4634; flavofamily protein
OG_00753	LA29479_1482	S4 domain protein	OG_00753	LA76x_2021	LA76x_2021; S4 domain protein
OG_00754	LA29479_3625	hypothetical protein	OG_00754	LA76x_3245	LA76x_3245; hypothetical protein
OG_00755	LA29479_1774	transcription elongation factor greA	OG_00755	LA76x_2855	greA; transcription elongation factor GreA
OG_00756	LA29479_3501	putative uncharacterized protein	OG_00756	LA76x_2735	LA76x_2735; conserved hypothetical protein
OG_00757	LA29479_4479	SCP-2 sterol transfer family protein	OG_00757	LA76x_4680	LA76x_4680; SCP-2 sterol transfer family protein
OG_00758	LA29479_2888	histidine kinase-, DNA gyrase B-, and HSP90-like ATPase family prot	OG_00758	LA76x_692	LA76x_692; response regulator
OG_00759	LA29479_4672	his Kinase A domain protein	OG_00759	LA76x_4057	LA76x_4057; his Kinase A domain protein
OG_00760	LA29479_3103	putative uncharacterized protein	OG_00760	LA76x_915	LA76x_915; polyketide cyclase / dehydrase and lipid transport family
OG_00761	LA29479_5135	oxidoreductase molybdopterin binding domain protein	OG_00761	LA76x_2256	LA76x_2256; oxidoreductase molybdopterin binding domain protein
OG_00762	LA29479_0458	threo-3-hydroxyaspartate ammonia-lyase	OG_00762	LA76x_4901	SRY1; threo-3-hydroxyaspartate ammonia-lyase
OG_00763	LA29479_0289	protein YchJ	OG_00763	LA76x_5155	LA76x_5155; putative protein YchJ
OG_00764	LA29479_4953	dimethyladenosine transferase	OG_00764	LA76x_4041	ksgA; dimethyladenosine transferase
OG_00765	LA29479_4945	putative membrane protein	OG_00765	LA76x_4033	LA76x_4033; putative transmembrane protein
OG_00766	LA29479_4106	signal peptide peptidase SppA, 67K type	OG_00766	LA76x_347	sppA; signal peptide peptidase SppA, 67K type
OG_00767	LA29479_3481	methyltransferase domain protein	OG_00767	LA76x_4264	LA76x_4264; methyltransferase domain protein
OG_00768	LA29479_3597	NUDIX domain protein	OG_00768	LA76x_4006	LA76x_4006; NUDIX domain protein
OG_00769	LA29479_2790	endoribonuclease L-PSP family protein	OG_00769	LA76x_2343	LA76x_2343; endoribonuclease L-PSP family protein
OG_00770	LA29479_3929	putative uncharacterized protein	OG_00770	LA76x_760	LA76x_760; conserved hypothetical protein
OG_00771	LA29479_3919	beta-lactamase family protein	OG_00771	LA76x_749	LA76x_749; beta-lactamase family protein
OG_00772	LA29479_4794	putative acetyl-CoA hydrolase	OG_00772	LA76x_4140	LA76x_4140; acetyl-CoA hydrolase/transferase C-terminal domain pr
OG_00773	LA29479_0760	putative uncharacterized protein	OG_00773	LA76x_1516	LA76x_1516; conserved hypothetical protein
OG_00774	LA29479_4334	peptide chain release factor 3	OG_00774	LA76x_1500	prfC; peptide chain release factor 3
OG_00775	LA29479_0655	feS assembly ATPase SufC	OG_00775	LA76x_1620	sufC; feS assembly ATPase SufC
OG_00776	LA29479_3579	putative membrane protein	OG_00776	LA76x_3987	LA76x_3987; putative transmembrane protein
OG_00777	LA29479_1556	quaternary ammonium compound-resistance protein sugE	OG_00777	LA76x_1950	LA76x_1950; small Multidrug Resistance family protein
OG_00778	LA29479_1182	NAD-dependent DNA ligase OB-fold domain protein	OG_00778	LA76x_2465	ligA; DNA ligase, NAD-dependent
OG_00779	LA29479_2730	type IV secretory pathway, VirB3-like family protein	OG_00779	LA76x_2404	LA76x_2404; type IV secretory pathway, VirB3-like family protein
OG_00780	LA29479_2441	protein-tyrosine-phosphatase	OG_00780	LA76x_3407	PPI; protein-tyrosine-phosphatase
OG_00781	LA29479_3344	putative uncharacterized protein	OG_00781	LA76x_3869	LA76x_3869; conserved hypothetical protein
OG_00782	LA29479_1185	bacterial regulatory s, lacI family protein	OG_00782	LA76x_2459	LA76x_2459; periplasmic binding and sugar binding domain of LacI fa
OG_00783	LA29479_1197	6-phosphogluconolactonase	OG_00783	LA76x_2447	LA76x_2447; 6-phosphogluconolactonase
OG_00784	LA29479_3171	conserved hypothetical protein	OG_00784	LA76x_1851	LA76x_1851; conserved hypothetical protein
OG_00785	LA29479_2312	response regulator	OG_00785	LA76x_4375	LA76x_4375; response regulator
OG_00786	LA29479_3158	DNA polymerase III, delta subunit	OG_00786	LA76x_1862	holA; DNA polymerase III, delta subunit
OG_00787	LA29479_4402	O-methyltransferase family protein	OG_00787	LA76x_514	LA76x_514; methyltransferase domain protein
OG_00788	LA29479_0616	single-stranded DNA-binding protein	OG_00788	LA76x_1658	ssb; single-stranded DNA-binding family protein
OG_00789	LA29479_1973	cobalamin 5'-phosphate synthase	OG_00789	LA76x_1228	cobS; cobalamin 5'-phosphate synthase
OG_00790	LA29479_0661	PKHD-type hydroxylase Sbal_3634	OG_00790	LA76x_1614	LA76x_1614; PKHD-type hydroxylase Sbal_3634
OG_00791	LA29479_0686	putative uncharacterized protein	OG_00791	LA76x_1587	LA76x_1587; conserved hypothetical protein
OG_00792	LA29479_3942	glyoxalase/Bleomycin resistance /Dioxygenase superfamily protein	OG_00792	LA76x_773	LA76x_773; glyoxalase/Bleomycin resistance /Dioxygenase superfam
OG_00793	LA29479_4301	general secretion pathway protein N	OG_00793	LA76x_1134	xpsN; general secretion pathway protein N
OG_00794	LA29479_3849	isocitrate/isopropylmalate dehydrogenase family protein	OG_00794	LA76x_3549	LA76x_3549; isocitrate/isopropylmalate dehydrogenase family protein
OG_00795	LA29479_4130	HAMP domain protein	OG_00795	LA76x_324	LA76x_324; HAMP domain protein
OG_00796	LA29479_4982	bacterial membrane flanked domain protein	OG_00796	LA76x_370	LA76x_370; bacterial PH domain protein
OG_00797	LA29479_2712	virD4 protein	OG_00797	LA76x_2422	LA76x_2422; type IV secretory system Conjugative DNA transfer fam
OG_00798	LA29479_0501	yceI-like domain protein	OG_00798	LA76x_4859	LA76x_4859; yceI-like domain protein
OG_00799	LA29479_1249	fimbrial Usher family protein	OG_00799	LA76x_122	LA76x_122; type VII secretion system (T7SS), usher family protein
OG_00800	LA29479_4052	hypothetical protein	OG_00800	LA76x_275	LA76x_275; hypothetical protein
OG_00801	LA29479_4099	putative uncharacterized protein	OG_00801	LA76x_354	LA76x_354; conserved hypothetical protein
OG_00802	LA29479_1695	glutathione S-transferase, C-terminal domain protein	OG_00802	LA76x_2137	LA76x_2137; glutathione S-transferase, C-terminal domain protein
OG_00803	LA29479_1345	sensory box protein	OG_00803	LA76x_26	LA76x_26; sensory box protein
OG_00804	LA29479_2630	putative uncharacterized protein	OG_00804	LA76x_557	LA76x_557; conserved hypothetical protein
OG_00805	LA29479_0849	putative uncharacterized protein	OG_00805	LA76x_3688	LA76x_3688; conserved hypothetical protein
OG_00806	LA29479_4251	integration host factor, alpha subunit	OG_00806	LA76x_1751	ihfA; integration host factor, alpha subunit
OG_00807	LA29479_0244	his Kinase A domain protein	OG_00807	LA76x_2945	LA76x_2945; his Kinase A domain protein
OG_00808	LA29479_1758	polyketide cyclase / dehydrase and lipid transport family protein	OG_00808	LA76x_2871	LA76x_2871; polyketide cyclase / dehydrase and lipid transport family
OG_00809	LA29479_3051	protease Do family protein	OG_00809	LA76x_867	LA76x_867; peptidase Do family protein
OG_00810	LA29479_1766	protein grpE	OG_00810	LA76x_2864	LA76x_2864; grpE family protein
OG_00811	LA29479_4170	putative uncharacterized protein	OG_00811	LA76x_4164	LA76x_4164; conserved hypothetical protein
OG_00812	LA29479_1979	cobalamin biosynthesis protein CobD	OG_00812	LA76x_1222	cobD; cobalamin biosynthesis protein CobD
OG_00813	LA29479_1223	RDD family protein	OG_00813	LA76x_144	LA76x_144; RDD family protein
OG_00814	LA29479_2818	ribosomal protein L36	OG_00814	LA76x_2315	rpmJ; ribosomal protein L36
OG_00815	LA29479_0294	amidohydrolase family protein	OG_00815	LA76x_5150	LA76x_5150; amidohydrolase family protein
OG_00816	LA29479_0716	PTS system fructose IIA component family protein	OG_00816	LA76x_1559	LA76x_1559; PTS system fructose IIA component family protein
OG_00817	LA29479_0781	cupin domain protein	OG_00817	LA76x_3626	LA76x_3626; bacterial regulatory helix-turn-helix, AraC family protein
OG_00818	LA29479_3046	HAD-superhydrolase, subIA, variant 1 family protein	OG_00818	LA76x_862	LA76x_862; HAD hydrolase, IA, variant 1 family protein
OG_00819	LA29479_0948	zinc-binding dehydrogenase family protein	OG_00819	LA76x_3791	LA76x_3791; zinc-binding dehydrogenase family protein
OG_00820	LA29479_0954	RNA methylase UPF0020 family protein	OG_00820	LA76x_3797	LA76x_3797; DNA methylase family protein
OG_00821	LA29479_1225	twin arginine-targeting protein translocase TatB	OG_00821	LA76x_142	tatB; twin arginine-targeting protein translocase TatB
OG_00822	LA29479_3389	30S ribosomal protein S8	OG_00822	LA76x_3914	rpsH; 30S ribosomal subunit protein S8

OG_00823	LA29479_2136	transcriptional regulator marR/emrR family
OG_00824	LA29479_3401	50S ribosomal protein L23
OG_00825	LA29479_3484	3-Deoxy-D-manno-octulosonic-acid transferase family protein
OG_00826	LA29479_4780	5-methyltetrahydropteroyltriglutamate-- homocysteine S-methyltransfe
OG_00827	LA29479_2140	tryptophan 2,3-dioxygenase
OG_00828	LA29479_1690	hypothetical protein
OG_00829	LA29479_2941	bacterial regulatory helix-turn-helix s, AraC family protein
OG_00830	LA29479_5041	pspA/IM30 family protein
OG_00831	LA29479_1178	methylthioribose-1-phosphate isomerase
OG_00832	LA29479_0209	NADH oxidoreductase (quinone), F subunit
OG_00833	LA29479_1639	succinate dehydrogenase, flavoprotein subunit
OG_00834	LA29479_1865	hypothetical protein
OG_00835	LA29479_1376	ribonuclease P protein component
OG_00836	LA29479_1964	regulatory , FmdB family domain protein
OG_00837	LA29479_1184	chromosome segregation protein SMC
OG_00838	LA29479_4036	transporter, monovalent cation:proton antiporter-2 family protein
OG_00839	LA29479_4017	MOSC domain protein
OG_00840	LA29479_2905	bacterial regulatory helix-turn-helix s, AraC family protein
OG_00841	LA29479_4034	hypothetical protein
OG_00842	LA29479_4477	AMP-binding enzyme family protein
OG_00843	LA29479_2572	glutaredoxin family protein
OG_00844	LA29479_1235	conserved hypothetical protein
OG_00845	LA29479_4026	putative membrane protein
OG_00846	LA29479_0998	phosphoglycerate mutase family protein
OG_00847	LA29479_1974	phosphoglycerate mutase family protein
OG_00848	LA29479_4125	possible transcriptional regulator, ArsR family protein
OG_00849	LA29479_2144	pyruvate dehydrogenase E1 component subunit beta
OG_00850	LA29479_0049	peptidase M13 family protein
OG_00851	LA29479_4040	cytochrome d ubiquinol oxidase, subunit II
OG_00852	LA29479_3998	prolyl oligopeptidase family protein
OG_00853	LA29479_5138	FHA domain protein
OG_00854	LA29479_4617	putative transmembrane protein
OG_00855	LA29479_0239	tat (twin-arginine translocation) pathway signal sequence domain prot
OG_00856	LA29479_0950	putative transmembrane protein
OG_00857	LA29479_1058	ompA family protein
OG_00858	LA29479_2951	putative uncharacterized protein
OG_00859	LA29479_0111	glutamine amidotransferase class-I family protein
OG_00860	LA29479_3474	FAD dependent oxidoreductase family protein
OG_00861	LA29479_3430	short chain dehydrogenase
OG_00862	LA29479_4059	dipeptidyl peptidase IV
OG_00863	LA29479_0492	HAD-superhydrolase, subIA, variant 3 family protein
OG_00864	LA29479_5139	polyhydroxyalkanoic acid system family protein
OG_00865	LA29479_1240	putative membrane protein
OG_00866	LA29479_2426	zinc-binding dehydrogenase family protein
OG_00867	LA29479_2897	hypothetical protein
OG_00868	LA29479_3999	hypothetical protein
OG_00869	LA29479_2797	putative uncharacterized protein
OG_00870	LA29479_0441	endoribonuclease L-PSP family protein
OG_00871	LA29479_3606	hydrogen peroxide-inducible genes activator
OG_00872	LA29479_4200	sensory box protein
OG_00873	LA29479_0496	putative esterase HI_1161
OG_00874	LA29479_4488	bacterial regulatory s, gntR family protein
OG_00875	LA29479_1090	modulator of DNA gyrase family protein
OG_00876	LA29479_3581	bacterial regulatory s, gntR family protein
OG_00877	LA29479_4514	ribosome recycling factor
OG_00878	LA29479_0751	putative uncharacterized protein
OG_00879	LA29479_3907	hypothetical protein
OG_00880	LA29479_3301	histidine kinase-, DNA gyrase B-, and HSP90-like ATPase family prote
OG_00881	LA29479_2566	conserved hypothetical protein
OG_00882	LA29479_5227	hemX family protein
OG_00883	LA29479_1645	transketolase, C-terminal domain protein
OG_00884	LA29479_3232	putative uncharacterized protein
OG_00885	LA29479_2172	tRNA dihydrouridine synthase A family protein
OG_00886	LA29479_1253	acetyltransferase family protein
OG_00887	LA29479_2911	HTH domain protein
OG_00888	LA29479_4494	tetratricopeptide repeat family protein
OG_00889	LA29479_1548	hypothetical protein
OG_00890	LA29479_4485	hypothetical protein
OG_00891	LA29479_2353	hypothetical protein
OG_00892	LA29479_1901	GDSL-like Lipase/Acylhydrolase family protein
OG_00893	LA29479_3112	guanosine-3',5'-bis(diphosphate) 3'-pyrophosphohydrolase MESH1
OG_00894	LA29479_4917	UPF0133 protein ybaB
OG_00895	LA29479_2233	ahpC/TSA family protein
OG_00896	LA29479_4313	phosphoribosylformylglycinamidine synthase
OG_00897	LA29479_0703	putative isomerase
OG_00898	LA29479_0192	tryptophan synthase, beta subunit
OG_00899	LA29479_0635	mechanosensitive ion channel family protein
OG_00900	LA29479_3394	30S ribosomal protein S17
OG_00901	LA29479_0797	drug resistance transporter, Bcr/CflA subfamily protein
OG_00902	LA29479_1990	lactoylglutathione lyase
OG_00903	LA29479_4549	tonB family C-terminal domain protein

OG_00823	LA76x_1016	LA76x_1016; transcriptional regulator marR/emrR family
OG_00824	LA76x_3926	rplW; 50S ribosomal subunit protein L23
OG_00825	LA76x_4267	kdtA; 3-deoxy-D-manno-octulosonate(Kdo)-lipid A transferase
OG_00826	LA76x_4125	metE; 5-methyltetrahydropteroyltriglutamate-- homocysteine S-methy
OG_00827	LA76x_1012	kynA; tryptophan 2,3-dioxygenase
OG_00828	LA76x_2143	LA76x_2143; hypothetical protein
OG_00829	LA76x_732	LA76x_732; bacterial regulatory helix-turn-helix, AraC family protein
OG_00830	LA76x_4699	LA76x_4699; pspA/IM30 family protein
OG_00831	LA76x_2469	mtnA; S-methyl-5-thioribose-1-phosphate isomerase
OG_00832	LA76x_2981	nuoF; NADH oxidoreductase (quinone), F subunit
OG_00833	LA76x_2196	sdhA; succinate dehydrogenase, flavoprotein subunit
OG_00834	LA76x_2765	LA76x_2765; hypothetical protein
OG_00835	LA76x_5202	rnpA; ribonuclease P protein component
OG_00836	LA76x_1236	LA76x_1236; regulatory , FmdB family domain protein
OG_00837	LA76x_2461	smc; chromosome segregation protein SMC
OG_00838	LA76x_291	LA76x_291; proton antiporter-2 family protein
OG_00839	LA76x_4604	LA76x_4604; conserved protein
OG_00840	LA76x_707	LA76x_707; bacterial regulatory helix-turn-helix, AraC family protein
OG_00841	LA76x_295	LA76x_295; A pre-toxin domain with the TG motif family protein
OG_00842	LA76x_4682	LA76x_4682; AMP-binding enzyme family protein
OG_00843	LA76x_4523	grxD; monothiol glutaredoxin, Grx4 family
OG_00844	LA76x_133	LA76x_133; conserved hypothetical protein
OG_00845	LA76x_4614	LA76x_4614; MAPEG family protein
OG_00846	LA76x_3840	LA76x_3840; histidine phosphatase super family protein
OG_00847	LA76x_1227	LA76x_1227; histidine phosphatase super family protein
OG_00848	LA76x_329	LA76x_329; putative transcriptional regulator, ArsR family protein
OG_00849	LA76x_1008	LA76x_1008; transketolase, pyrimidine binding domain protein
OG_00850	LA76x_3138	LA76x_3138; endopeptidase, peptidase family M13
OG_00851	LA76x_288	cydB; cytochrome d ubiquinol oxidase, subunit II
OG_00852	LA76x_4585	LA76x_4585; dienelactone hydrolase family protein
OG_00853	LA76x_2253	LA76x_2253; FHA domain protein
OG_00854	LA76x_4113	LA76x_4113; conserved hypothetical protein
OG_00855	LA76x_2950	LA76x_2950; tat (twin-arginine translocation) pathway signal sequenc
OG_00856	LA76x_3793	LA76x_3793; putative transmembrane protein
OG_00857	LA76x_2584	LA76x_2584; glycine zipper family protein
OG_00858	LA76x_742	LA76x_742; conserved hypothetical protein
OG_00859	LA76x_3075	LA76x_3075; glutamine amidotransferase class-I family protein
OG_00860	LA76x_4258	LA76x_4258; FAD binding domain protein
OG_00861	LA76x_4214	LA76x_4214; short chain dehydrogenase
OG_00862	LA76x_269	LA76x_269; dipeptidyl peptidase IV
OG_00863	LA76x_4866	LA76x_4866; HAD hydrolase, IA, variant 1 family protein
OG_00864	LA76x_2252	LA76x_2252; polyhydroxyalkanoic acid system family protein
OG_00865	LA76x_129	LA76x_129; hypothetical protein
OG_00866	LA76x_3395	LA76x_3395; oxidoreductase , NAD-binding Rossmann fold family pr
OG_00867	LA76x_700	LA76x_700; conserved hypothetical protein
OG_00868	LA76x_4586	LA76x_4586; DNA/RNA non-specific endonuclease family protein
OG_00869	LA76x_2336	LA76x_2336; conserved hypothetical protein
OG_00870	LA76x_4916	LA76x_4916; endoribonuclease L-PSP family protein
OG_00871	LA76x_4014	LA76x_4014; bacterial regulatory helix-turn-helix , lysR family protein
OG_00872	LA76x_4191	LA76x_4191; diguanylate cyclase domain protein
OG_00873	LA76x_4863	LA76x_4863; putative esterase HI_1161
OG_00874	LA76x_4671	LA76x_4671; bacterial regulatory, gntR family protein
OG_00875	LA76x_2550	LA76x_2550; modulator of DNA gyrase family protein
OG_00876	LA76x_3989	LA76x_3989; bacterial regulatory, gntR family protein
OG_00877	LA76x_1920	frr; ribosome recycling factor
OG_00878	LA76x_1524	LA76x_1524; conserved hypothetical protein
OG_00879	LA76x_3607	LA76x_3607; conserved hypothetical protein
OG_00880	LA76x_183	LA76x_183; histidine kinase-, DNA gyrase B-, and HSP90-like ATPas
OG_00881	LA76x_4518	rsgA; ribosome small subunit-dependent GTPase A
OG_00882	LA76x_4727	LA76x_4727; hemX family protein
OG_00883	LA76x_2190	LA76x_2190; transketolase, pyrimidine binding domain protein
OG_00884	LA76x_1794	LA76x_1794; conserved hypothetical protein
OG_00885	LA76x_983	dusA; tRNA dihydrouridine synthase A family protein
OG_00886	LA76x_118	LA76x_118; acetyltransferase family protein
OG_00887	LA76x_713	LA76x_713; deoR-like helix-turn-helix domain protein
OG_00888	LA76x_4665	LA76x_4665; TPR repeat family protein
OG_00889	LA76x_1956	LA76x_1956; hypothetical protein
OG_00890	LA76x_4674	LA76x_4674; hypothetical protein
OG_00891	LA76x_4417	LA76x_4417; hypothetical protein
OG_00892	LA76x_1393	LA76x_1393; GDSL-like Lipase/Acylhydrolase family protein
OG_00893	LA76x_924	HDDC3; HD domain-containing protein 3
OG_00894	LA76x_3516	LA76x_3516; DNA-binding protein, YbaB/EbfC family
OG_00895	LA76x_4300	LA76x_4300; thioredoxin family protein
OG_00896	LA76x_1122	purL; phosphoribosylformylglycinamidine synthase
OG_00897	LA76x_1572	LA76x_1572; putative isomerase
OG_00898	LA76x_2998	trpB; tryptophan synthase, beta subunit
OG_00899	LA76x_1640	LA76x_1640; mechanosensitive ion channel family protein
OG_00900	LA76x_3919	rpsQ; 30S ribosomal protein S17
OG_00901	LA76x_3642	LA76x_3642; drug resistance transporter, Bcr/CflA subfamily protein
OG_00902	LA76x_1212	gloA; lactoylglutathione lyase
OG_00903	LA76x_1885	LA76x_1885; tonB family C-terminal domain protein

OG_00904	LA29479_1516	integral membrane protein MviN
OG_00905	LA29479_3370	coenzyme PQQ biosynthesis protein C
OG_00906	LA29479_0225	tRNA pseudouridine synthase B
OG_00907	LA29479_2825	hydrolase
OG_00908	LA29479_1095	2-aminomuconate deaminase
OG_00909	LA29479_1893	apolipoprotein N-acyltransferase
OG_00910	LA29479_0207	NADH dehydrogenase (quinone), D subunit
OG_00911	LA29479_3045	cytosol aminopeptidase
OG_00912	LA29479_3408	ribosomal protein S12
OG_00913	LA29479_2226	helix-turn-helix family protein
OG_00914	LA29479_0780	amidohydrolase family protein
OG_00915	LA29479_4698	tetratricopeptide repeat family protein
OG_00916	LA29479_0718	phosphoenolpyruvate-protein phosphotransferase
OG_00917	LA29479_3049	putative transmembrane protein
OG_00918	LA29479_0285	isocitrate dehydrogenase, NADP-dependent
OG_00919	LA29479_3037	acetyltransferase family protein
OG_00920	LA29479_2235	acetyl-CoA carboxylase, biotin carboxyl carrier protein
OG_00921	LA29479_4900	malonyl CoA-acyl carrier protein transacylase
OG_00922	LA29479_4908	septum formation protein Maf
OG_00923	LA29479_0543	nicotinate-nucleotide diphosphorylase
OG_00924	LA29479_3503	fosmidomycin resistance protein
OG_00925	LA29479_0455	putative uncharacterized protein
OG_00926	LA29479_2830	acrB/AcrD/AcrF family protein
OG_00927	LA29479_3536	cytochrome o ubiquinol oxidase subunit IV
OG_00928	LA29479_3071	peptidase M23 family protein
OG_00929	LA29479_4923	putative lipoprotein
OG_00930	LA29479_4932	cytidine and deoxycytidylate deaminase zinc-binding region family pro
OG_00931	LA29479_2271	putative membrane protein
OG_00932	LA29479_4525	ribonuclease HII family protein
OG_00933	LA29479_1135	putative membrane protein
OG_00935	LA29479_3352	methylisocitrate lyase
OG_00936	LA29479_3203	rhomboid family protein
OG_00937	LA29479_3349	glutathione S-transferase GST-4.5
OG_00938	LA29479_2019	type IV leader peptidase family protein
OG_00939	LA29479_1696	zinc carboxypeptidase family protein
OG_00940	LA29479_1938	peptidoglycan-associated lipoprotein
OG_00941	LA29479_3445	hemolysin-type calcium-binding repeat family protein
OG_00942	LA29479_5162	penicillin-binding protein 2
OG_00943	LA29479_0166	helix-turn-helix family protein
OG_00944	LA29479_1504	hypothetical protein
OG_00945	LA29479_1041	cytochrome c family protein
OG_00946	LA29479_0621	dienelactone hydrolase family protein
OG_00947	LA29479_2145	2-oxoacid dehydrogenases acyltransferase family protein
OG_00948	LA29479_1951	crossover junction endodeoxyribonuclease RuvC
OG_00949	LA29479_3892	putative transmembrane protein, similar to that required for pathogeni
OG_00950	LA29479_4937	cold-active aminopeptidase
OG_00951	LA29479_3235	conserved hypothetical protein
OG_00952	LA29479_1949	holliday junction DNA helicase RuvA
OG_00953	LA29479_4066	helix-turn-helix family protein
OG_00954	LA29479_1166	penicillin-binding protein 1C
OG_00955	LA29479_1326	putative uncharacterized protein
OG_00956	LA29479_3387	ribosomal protein L18
OG_00957	LA29479_4096	DNA topoisomerase I
OG_00958	LA29479_2315	similar to endo-beta-N-acetylglucosaminidase
OG_00959	LA29479_3654	phosphoribosyl-ATP diphosphatase
OG_00960	LA29479_4474	hpcH/HpaI aldolase/citrate lyase family protein
OG_00961	LA29479_2247	putative uncharacterized protein
OG_00962	LA29479_1449	putative secreted protein
OG_00963	LA29479_0310	conserved enzyme
OG_00964	LA29479_1461	K+-transporting ATPase, C subunit
OG_00965	LA29479_1196	glucokinase
OG_00966	LA29479_2499	putative uncharacterized protein
OG_00967	LA29479_0924	glutathione-dependent formaldehyde-activating enzyme family protein
OG_00968	LA29479_0513	sensory box protein
OG_00969	LA29479_1647	acetyltransferase family protein
OG_00970	LA29479_0638	lysE type translocator family protein
OG_00971	LA29479_3883	hypothetical protein
OG_00972	LA29479_3799	outer membrane protein MIP
OG_00973	LA29479_1851	translation initiation factor IF-1
OG_00974	LA29479_1759	uncharacterised family protein
OG_00975	LA29479_2098	4Fe-4S binding domain protein
OG_00976	LA29479_4554	putative uncharacterized protein
OG_00977	LA29479_0238	bacterial regulatory helix-turn-helix , lysR family protein
OG_00978	LA29479_2796	outer membrane assembly lipoprotein YfgL
OG_00979	LA29479_1841	aconitate hydratase 2
OG_00980	LA29479_0095	hypothetical protein
OG_00981	LA29479_0277	bacterial regulatory helix-turn-helix , lysR family protein
OG_00982	LA29479_2575	hypothetical protein
OG_00983	LA29479_1233	recombination-associated protein rdgC
OG_00984	LA29479_2259	uncharacterized protein
OG_00985	LA29479_3693	carboxyl transferase domain protein

OG_00904	LA76x_1987	mviN; integral membrane protein MviN
OG_00905	LA76x_3896	pqqC; coenzyme PQQ biosynthesis protein C
OG_00906	LA76x_2965	truB; tRNA pseudouridine(55) synthase
OG_00907	LA76x_2308	LA76x_2308; alpha/beta hydrolase fold family protein
OG_00908	LA76x_2546	amnD; 2-aminomuconate deaminase
OG_00909	LA76x_1401	lnt; apolipoprotein N-acyltransferase
OG_00910	LA76x_2983	nuoD; NADH dehydrogenase (quinone), D subunit
OG_00911	LA76x_861	LA76x_861; cytosol aminopeptidase
OG_00912	LA76x_3934	rpsL; ribosomal protein S12
OG_00913	LA76x_4293	LA76x_4293; helix-turn-helix family protein
OG_00914	LA76x_3625	LA76x_3625; amidohydrolase family protein
OG_00915	LA76x_4083	LA76x_4083; TPR repeat family protein
OG_00916	LA76x_1557	ptsP; phosphoenolpyruvate-protein phosphotransferase
OG_00917	LA76x_865	LA76x_865; conserved hypothetical protein
OG_00918	LA76x_5159	icd; isocitrate dehydrogenase, NADP-dependent
OG_00919	LA76x_853	LA76x_853; acetyltransferase family protein
OG_00920	LA76x_4302	accB; acetyl-CoA carboxylase, biotin carboxyl carrier protein
OG_00921	LA76x_3501	fabD; malonyl CoA-acyl carrier protein transacylase
OG_00922	LA76x_3507	maf; septum formation protein Maf
OG_00923	LA76x_1723	nadC; nicotinate-nucleotide diphosphorylase
OG_00924	LA76x_2733	LA76x_2733; major Facilitator Superfamily protein
OG_00925	LA76x_4904	LA76x_4904; conserved hypothetical protein
OG_00926	LA76x_2303	LA76x_2303; MMPL family protein
OG_00927	LA76x_2700	cyoD; cytochrome o ubiquinol oxidase subunit IV
OG_00928	LA76x_885	LA76x_885; peptidase M23 family protein
OG_00929	LA76x_809	LA76x_809; prokaryotic lipoprotein-attachment site family protein
OG_00930	LA76x_819	LA76x_819; cytidine and deoxycytidylate deaminase zinc-binding reg
OG_00931	LA76x_4336	LA76x_4336; putative uvs125
OG_00932	LA76x_1908	LA76x_1908; ribonuclease HII family protein
OG_00933	LA76x_2509	LA76x_2509; ABC-2 transporter family protein
OG_00935	LA76x_3877	prpB; methylisocitrate lyase
OG_00936	LA76x_1821	LA76x_1821; rhomboid family protein
OG_00937	LA76x_3874	GST; glutathione S-transferase
OG_00938	LA76x_1186	LA76x_1186; type IV leader peptidase family protein
OG_00939	LA76x_2136	LA76x_2136; zinc carboxypeptidase family protein
OG_00940	LA76x_1261	pal; peptidoglycan-associated lipoprotein
OG_00941	LA76x_4229	LA76x_4229; hemolysin-type calcium-binding repeat family protein
OG_00942	LA76x_832	mrdA; penicillin-binding protein 2
OG_00943	LA76x_3024	LA76x_3024; helix-turn-helix family protein
OG_00944	LA76x_1998	LA76x_1998; hypothetical protein
OG_00945	LA76x_2602	LA76x_2602; cytochrome C oxidase, cbb3-type, subunit III family pro
OG_00946	LA76x_1653	LA76x_1653; phospholipase/Carboxylesterase family protein
OG_00947	LA76x_1007	LA76x_1007; e3 binding domain protein
OG_00948	LA76x_1248	ruvC; crossover junction endodeoxyribonuclease RuvC
OG_00949	LA76x_3593	LA76x_3593; conserved hypothetical protein
OG_00950	LA76x_824	LA76x_824; cold-active aminopeptidase
OG_00951	LA76x_1791	LA76x_1791; conserved hypothetical protein
OG_00952	LA76x_1250	ruvA; holliday junction DNA helicase RuvA
OG_00953	LA76x_265	LA76x_265; helix-turn-helix family protein
OG_00954	LA76x_2479	pbpC; penicillin-binding protein 1C
OG_00955	LA76x_45	LA76x_45; conserved hypothetical protein
OG_00956	LA76x_3912	rplR; ribosomal protein L18
OG_00957	LA76x_357	topA; DNA topoisomerase I
OG_00958	LA76x_4378	LA76x_4378; glycosyl hydrolases 18 family protein
OG_00959	LA76x_3221	hisE; phosphoribosyl-ATP diphosphatase
OG_00960	LA76x_4685	LA76x_4685; hpcH/HpaI aldolase/citrate lyase family protein
OG_00961	LA76x_4314	LA76x_4314; conserved hypothetical protein
OG_00962	LA76x_2057	LA76x_2057; putative secreted protein
OG_00963	LA76x_5134	LA76x_5134; conserved enzyme
OG_00964	LA76x_2044	kdpC; K+-transporting ATPase, C subunit
OG_00965	LA76x_2448	glk; glucokinase
OG_00966	LA76x_4453	LA76x_4453; conserved hypothetical protein
OG_00967	LA76x_3764	LA76x_3764; glutathione-dependent formaldehyde-activating enzyme
OG_00968	LA76x_4846	LA76x_4846; sensory box protein
OG_00969	LA76x_2188	LA76x_2188; acetyltransferase domain protein
OG_00970	LA76x_1637	LA76x_1637; lysE type translocator family protein
OG_00971	LA76x_3584	LA76x_3584; hypothetical protein
OG_00972	LA76x_4766	LA76x_4766; FKBP-type peptidyl-prolyl cis-trans isomerase family pr
OG_00973	LA76x_2779	infA; translation initiation factor IF-1
OG_00974	LA76x_2870	LA76x_2870; rnfH Ubiquitin family protein
OG_00975	LA76x_1054	LA76x_1054; 4Fe-4S binding domain protein
OG_00976	LA76x_1444	LA76x_1444; conserved hypothetical protein
OG_00977	LA76x_2951	LA76x_2951; bacterial regulatory helix-turn-helix , lysR family protein
OG_00978	LA76x_2337	yfgL; outer membrane assembly lipoprotein YfgL
OG_00979	LA76x_2789	acnB; aconitate hydratase 2
OG_00980	LA76x_3093	LA76x_3093; conserved hypothetical protein
OG_00981	LA76x_5168	LA76x_5168; bacterial regulatory helix-turn-helix , lysR family protein
OG_00982	LA76x_4526	LA76x_4526; conserved hypothetical protein
OG_00983	LA76x_135	rdgC; recombination-associated protein rdgC
OG_00984	LA76x_4325	LA76x_4325; conserved hypothetical protein
OG_00985	LA76x_3184	LA76x_3184; carboxyl transferase domain protein

OG_00986	LA29479_4933	putative uncharacterized protein	OG_00986	LA76x_820	LA76x_820; conserved hypothetical protein
OG_00987	LA29479_2944	sarcosine oxidase alpha subunit	OG_00987	LA76x_735	LA76x_735; 2Fe-2S iron-sulfur cluster binding domain protein
OG_00988	LA29479_0907	polyhydroxyalkanoate depolymerase, intracellular family protein	OG_00988	LA76x_3747	phaZ; polyhydroxyalkanoate depolymerase, intracellular family protei
OG_00989	LA29479_0185	D-isomer specific 2-hydroxyacid dehydrogenase, NAD binding domai	OG_00989	LA76x_3004	LA76x_3004; NAD binding domain of 6-phosphogluconate dehydroge
OG_00990	LA29479_0322	4'-phosphopantetheinyl transferase superfamily protein	OG_00990	LA76x_5122	LA76x_5122; 4'-phosphopantetheinyl transferase superfamily protein
OG_00991	LA29479_4306	prepilin-type N-terminal cleavage/methylation domain protein	OG_00991	LA76x_1129	LA76x_1129; prepilin-type N-terminal cleavage/methylation domain p
OG_00992	LA29479_4098	tRNA threonylcarbamoyladenosine biosynthesis protein RimN	OG_00992	LA76x_355	LA76x_355; protein involved in synthesis of threonylcarbamoyladeno
OG_00993	LA29479_0298	bacterial regulatory helix-turn-helix , lysR family protein	OG_00993	LA76x_5146	LA76x_5146; bacterial regulatory helix-turn-helix , lysR family protein
OG_00994	LA29479_0537	glutaredoxin-4	OG_00994	LA76x_1729	grxD; monothiol glutaredoxin, Grx4 family
OG_00995	LA29479_2279	bacterioferritin	OG_00995	LA76x_4343	bfr; bacterioferritin
OG_00996	LA29479_3385	ribosomal protein L30	OG_00996	LA76x_3910	rpmD; ribosomal protein L30
OG_00997	LA29479_4123	metallo-beta-lactamase family protein	OG_00997	LA76x_331	ymaE; metallo-beta-lactamase family protein
OG_00998	LA29479_4054	DNA helicase II	OG_00998	LA76x_273	uvrD; DNA helicase II
OG_00999	LA29479_3231	aminotransferase class-III family protein	OG_00999	LA76x_1795	LA76x_1795; aminotransferase class-III family protein
OG_01000	LA29479_1757	ssrA-binding protein	OG_01000	LA76x_2872	smpB; ssrA-binding protein
OG_01001	LA29479_2719	putative uncharacterized protein	OG_01001	LA76x_2416	LA76x_2416; conserved hypothetical protein
OG_01002	LA29479_2135	4-hydroxyphenylpyruvate dioxygenase	OG_01002	LA76x_1017	hppD; 4-hydroxyphenylpyruvate dioxygenase
OG_01003	LA29479_0840	GTP-binding proten HflX	OG_01003	LA76x_3680	hflX; GTP-binding protein HflX
OG_01004	LA29479_4547	hypothetical protein	OG_01004	LA76x_1886	LA76x_1886; hypothetical protein
OG_01005	LA29479_3373	peptide amidase	OG_01005	LA76x_3899	pam; peptide amidase
OG_01006	LA29479_4236	1-deoxy-D-xylulose-5-phosphate synthase	OG_01006	LA76x_1766	dxs; 1-deoxy-D-xylulose-5-phosphate synthase
OG_01007	LA29479_4610	putative uncharacterized protein	OG_01007	LA76x_4105	LA76x_4105; putative membrane protein
OG_01008	LA29479_2889	response regulator	OG_01008	LA76x_693	LA76x_693; cheB methylesterase family protein
OG_01009	LA29479_1540	phosphate-selective porin O and P family protein	OG_01009	LA76x_1964	LA76x_1964; phosphate-selective porin O and P family protein
OG_01010	LA29479_4952	4-hydroxythreonine-4-phosphate dehydrogenase	OG_01010	LA76x_4040	pdxA; 4-hydroxythreonine-4-phosphate dehydrogenase
OG_01011	LA29479_4069	putative uncharacterized protein	OG_01011	LA76x_262	LA76x_262; beta-ketoacyl synthase, N-terminal domain protein
OG_01012	LA29479_3794	bacterial regulatory s, tetR family protein	OG_01012	LA76x_4771	LA76x_4771; bacterial regulatory, tetR family protein
OG_01013	LA29479_1096	3-hydroxyanthranilate 3,4-dioxygenase	OG_01013	LA76x_2545	nbaC; 3-hydroxyanthranilate 3,4-dioxygenase
OG_01014	LA29479_3305	subtilase family protein	OG_01014	LA76x_179	LA76x_179; conserved hypothetical protein
OG_01015	LA29479_1272	putative uncharacterized protein	OG_01015	LA76x_101	LA76x_101; peptidase inhibitor I78 family protein
OG_01016	LA29479_2709	FAD binding domain protein	OG_01016	LA76x_2426	LA76x_2426; FAD binding domain protein
OG_01017	LA29479_3676	hypothetical protein	OG_01017	LA76x_3201	LA76x_3201; hypothetical protein
OG_01018	LA29479_3871	uncharacterized ywqG domain protein	OG_01018	LA76x_3571	LA76x_3571; conserved hypothetical protein
OG_01019	LA29479_4237	putative membrane protein	OG_01019	LA76x_1764	LA76x_1764; conserved hypothetical protein
OG_01020	LA29479_3039	protein rarD	OG_01020	LA76x_855	LA76x_855; eamA-like transporter family protein
OG_01021	LA29479_0886	nlpC/P60 family protein	OG_01021	LA76x_3726	LA76x_3726; nlpC/P60 family protein
OG_01022	LA29479_0473	cytochrome b561 family protein	OG_01022	LA76x_4885	LA76x_4885; prokaryotic cytochrome b561 family protein
OG_01023	LA29479_1875	short chain dehydrogenase family protein	OG_01023	LA76x_2755	LA76x_2755; short chain dehydrogenase family protein
OG_01024	LA29479_0980	hypothetical protein	OG_01024	LA76x_3824	LA76x_3824; hypothetical protein
OG_01025	LA29479_4317	RDD family protein	OG_01025	LA76x_1118	LA76x_1118; RDD family protein
OG_01026	LA29479_4088	hypothetical protein	OG_01026	LA76x_243	LA76x_243; conserved hypothetical protein
OG_01027	LA29479_0318	putative uncharacterized protein	OG_01027	LA76x_5126	LA76x_5126; conserved hypothetical protein
OG_01028	LA29479_0205	NADH-quinone oxidoreductase, B subunit	OG_01028	LA76x_2985	LA76x_2985; NADH-quinone oxidoreductase, B subunit
OG_01029	LA29479_2757	conserved hypothetical protein	OG_01029	LA76x_2377	LA76x_2377; NRDE family protein
OG_01030	LA29479_0544	putative uncharacterized protein	OG_01030	LA76x_1722	LA76x_1722; conserved hypothetical protein
OG_01031	LA29479_1123	putative uncharacterized protein	OG_01031	LA76x_2521	LA76x_2521; conserved hypothetical protein
OG_01032	LA29479_3454	ion channel family protein	OG_01032	LA76x_4237	LA76x_4237; ion channel family protein
OG_01033	LA29479_1471	lexA repressor	OG_01033	LA76x_2032	lexA; repressor LexA
OG_01034	LA29479_0777	angiotensin-converting enzyme	OG_01034	LA76x_3622	ACE; angiotensin-converting enzyme
OG_01035	LA29479_2917	ADP-ribosylglycohydrolase	OG_01035	LA76x_719	LA76x_719; ADP-ribosylglycohydrolase
OG_01036	LA29479_3042	permease family protein	OG_01036	LA76x_858	LA76x_858; ftsX-like permease family protein
OG_01037	LA29479_2298	hypothetical protein	OG_01037	LA76x_4361	LA76x_4361; hypothetical protein
OG_01038	LA29479_4707	RNA polymerase sigma factor, sigma-70 family protein	OG_01038	LA76x_3463	LA76x_3463; RNA polymerase sigma factor, sigma-70 family protein
OG_01039	LA29479_4387	nfeD-like family protein	OG_01039	LA76x_1450	LA76x_1450; nfeD-like C-terminal, partner-binding family protein
OG_01040	LA29479_2407	acyl-CoA dehydrogenase, N-terminal domain protein	OG_01040	LA76x_3377	LA76x_3377; acyl-CoA dehydrogenase, N-terminal domain protein
OG_01041	LA29479_4815	putative uncharacterized protein	OG_01041	LA76x_3278	LA76x_3278; conserved hypothetical protein
OG_01042	LA29479_1868	putrescine transport system permease protein PotH	OG_01042	LA76x_2761	LA76x_2761; potH
OG_01043	LA29479_2165	conserved hypothetical protein	OG_01043	LA76x_988	LA76x_988; phosphoenolpyruvate:glucose-phosphotransferase regul
OG_01044	LA29479_0724	putative Holliday junction resolvase	OG_01044	LA76x_1551	LA76x_1551; conserved hypothetical protein
OG_01045	LA29479_0943	putative membrane protein	OG_01045	LA76x_3786	LA76x_3786; polyketide cyclase / dehydrase and lipid transport famil
OG_01046	LA29479_3220	#NAME?	OG_01046	LA76x_1806	secF; protein-export membrane protein SecF
OG_01047	LA29479_4075	putative membrane protein	OG_01047	LA76x_256	LA76x_256; MMPL family protein
OG_01048	LA29479_4048	DNA polymerase I family protein	OG_01048	LA76x_279	polA; DNA polymerase I family protein
OG_01049	LA29479_4804	glutamate-5-semialdehyde dehydrogenase	OG_01049	LA76x_3267	proA; glutamate-5-semialdehyde dehydrogenase
OG_01050	LA29479_3388	50S ribosomal protein L6	OG_01050	LA76x_3913	rplF; ribosomal protein L6
OG_01051	LA29479_2112	response regulator	OG_01051	LA76x_1040	LA76x_1040; response regulator
OG_01052	LA29479_3564	NAD(P)H:quinone oxidoreductase, type IV	OG_01052	LA76x_3972	wrbA; quinone oxidoreductase, type IV
OG_01053	LA29479_3623	alpha-1,2-mannosidase, family protein	OG_01053	LA76x_3247	LA76x_3247; alpha-1,2-mannosidase family protein
OG_01054	LA29479_0977	imidazolonepropionase	OG_01054	LA76x_3821	hutI; imidazolonepropionase
OG_01055	LA29479_1903	thiamine biosynthesis protein ThiS	OG_01055	LA76x_1391	thiS; thiamine biosynthesis protein ThiS
OG_01056	LA29479_4594	putative uncharacterized protein	OG_01056	LA76x_4090	LA76x_4090; conserved hypothetical protein
OG_01057	LA29479_4386	hypothetical protein	OG_01057	LA76x_1451	LA76x_1451; hypothetical protein
OG_01058	LA29479_5002	putative peptidoglycan binding domain protein	OG_01058	LA76x_4716	LA76x_4716; conserved hypothetical protein
OG_01059	LA29479_1377	60Kd inner membrane family protein	OG_01059	LA76x_5201	yidC; membrane protein insertase, YidC/Oxa1 family, N-terminal dom
OG_01060	LA29479_3237	hypothetical protein	OG_01060	LA76x_1789	LA76x_1789; hypothetical protein
OG_01061	LA29479_4979	bacterial lipid A biosynthesis acyltransferase family protein	OG_01061	LA76x_373	LA76x_373; bacterial lipid A biosynthesis acyltransferase family prote
OG_01062	LA29479_0504	membrane transport family protein	OG_01062	LA76x_4855	LA76x_4855; membrane transport family protein
OG_01063	LA29479_1146	metallo-beta-lactamase L1	OG_01063	LA76x_2498	LA76x_2498; metallo-beta-lactamase L1
OG_01064	LA29479_5132	histidinol-phosphate aminotransferase	OG_01064	LA76x_2259	hisC; histidinol-phosphate transaminase
OG_01065	LA29479_1399	glutamine amidotransferase class-I family protein	OG_01065	LA76x_2100	LA76x_2100; glutamine amidotransferase class-I family protein
OG_01066	LA29479_2036	UDP-N-acetylmuramyl-tripeptide synthetases family protein	OG_01066	LA76x_1169	murE; UDP-N-acetylmuramyl-tripeptide synthetase family protein

OG_01067	LA29479_3957	outer membrane efflux family protein
OG_01068	LA29479_1343	putative uncharacterized protein
OG_01069	LA29479_4218	pantetheine-phosphate adenylyltransferase
OG_01070	LA29479_0091	bacterial regulatory s, tetR family protein
OG_01071	LA29479_4021	C-terminal processing peptidase family protein
OG_01072	LA29479_2275	conserved hypothetical protein
OG_01073	LA29479_0649	3-hydroxybutyrate dehydrogenase family protein
OG_01074	LA29479_1109	5'-nucleotidase family protein
OG_01075	LA29479_4163	penicillin-binding , 1A family protein
OG_01076	LA29479_2148	glyoxalase/Bleomycin resistance /Dioxygenase superfamily protein
OG_01077	LA29479_1211	RNA polymerase sigma factor, TIGR02999 family protein
OG_01078	LA29479_4611	phenazine biosynthesis PhzF family protein
OG_01079	LA29479_3614	glutaminyl-tRNA synthetase
OG_01080	LA29479_3955	cobalt-zinc-cadmium resistance protein CzcA
OG_01081	LA29479_3121	hypothetical protein
OG_01082	LA29479_2306	efflux transporter, RND family, MFP subunit
OG_01083	LA29479_0410	conserved hypothetical protein
OG_01084	LA29479_4907	hypothetical protein
OG_01085	LA29479_0096	putative uncharacterized protein
OG_01086	LA29479_0970	hypothetical protein
OG_01087	LA29479_3956	efflux transporter, RND family, MFP subunit
OG_01088	LA29479_3895	arginine-tRNA-transferase, C terminus family protein
OG_01089	LA29479_3192	metallo-beta-lactamase superfamily protein
OG_01090	LA29479_3692	hsp70 family protein
OG_01091	LA29479_1329	NIPSNAP family protein
OG_01092	LA29479_2785	bifunctional protein FolD
OG_01093	LA29479_0985	glucose-6-phosphate isomerase
OG_01094	LA29479_4716	phosphorylase 2 family protein
OG_01095	LA29479_4327	ribosomal-protein-alanine acetyltransferase
OG_01096	LA29479_0144	putative lipoprotein
OG_01097	LA29479_1347	dctM-like transporters family protein
OG_01098	LA29479_4914	HIT domain protein
OG_01099	LA29479_5032	cytochrome c4 domain protein
OG_01100	LA29479_3440	putative uncharacterized domain protein
OG_01101	LA29479_3591	hypothetical protein
OG_01102	LA29479_2528	putative uncharacterized protein
OG_01103	LA29479_1982	putative membrane protein
OG_01104	LA29479_1923	lysE type translocator family protein
OG_01105	LA29479_0152	L-sorbosone dehydrogenase
OG_01106	LA29479_1513	signal peptidase II
OG_01107	LA29479_4070	glycosyl transferase 2 family protein
OG_01108	LA29479_0217	proton-translocating NADH-quinone oxidoreductase, chain M family p
OG_01109	LA29479_0243	hypothetical protein
OG_01110	LA29479_3838	acetyl-CoA acetyltransferases family protein
OG_01111	LA29479_4118	putative uncharacterized protein
OG_01112	LA29479_2051	conserved hypothetical protein
OG_01113	LA29479_1073	iron-binding protein iscA
OG_01114	LA29479_2026	cell division protein FtsZ
OG_01115	LA29479_3438	bacterial regulatory helix-turn-helix s, AraC family protein
OG_01116	LA29479_4959	thymidylate synthase
OG_01118	LA29479_4956	conserved hypothetical protein
OG_01119	LA29479_1129	putative FKBP-type peptidyl-prolyl cis-trans isomerase fkpA
OG_01120	LA29479_1142	lyase family protein
OG_01121	LA29479_3227	histidine kinase-, DNA gyrase B-, and HSP90-like ATPase family prote
OG_01122	LA29479_3032	lipoyltransferase
OG_01123	LA29479_2746	cytochrome c-type biogenesis protein CcmF
OG_01124	LA29479_5190	FHA domain protein
OG_01125	LA29479_0176	yhhN-like family protein
OG_01126	LA29479_2149	metallo-beta-lactamase superfamily protein
OG_01127	LA29479_1074	conserved uncharacterized protein
OG_01128	LA29479_3859	periplasmic glucan biosynthesis , MdoG family protein
OG_01129	LA29479_1202	putative uncharacterized protein
OG_01130	LA29479_2805	acetyl-CoA acetyltransferases family protein
OG_01131	LA29479_4331	NHL repeat family protein
OG_01132	LA29479_0773	cell division topological specificity factor MinE
OG_01133	LA29479_1444	2-amino-4-hydroxy-6- hydroxymethyldihydropteridine pyrophosphokin
OG_01134	LA29479_3790	putative uncharacterized protein
OG_01135	LA29479_4723	hypothetical protein
OG_01136	LA29479_0923	putative uncharacterized protein
OG_01137	LA29479_0640	putative lipoprotein
OG_01138	LA29479_4289	serine hydroxymethyltransferase 2
OG_01139	LA29479_0181	SCO1/SenC family protein
OG_01140	LA29479_1558	ribonuclease R
OG_01141	LA29479_0454	putative membrane protein
OG_01142	LA29479_3197	hypothetical protein
OG_01143	LA29479_0171	lipoNlpE domain protein
OG_01144	LA29479_4136	glycosyltransferase family 28 N-terminal domain protein
OG_01145	LA29479_1622	excinuclease ABC, C subunit
OG_01146	LA29479_2563	citrate transporter
OG_01147	LA29479_0733	UPF0001 protein yggS
OG_01148	LA29479_4158	type IV pilus secretin PilQ family protein

OG_01067	LA76x_788	LA76x_788; outer membrane efflux family protein
OG_01068	LA76x_28	LA76x_28; conserved hypothetical protein
OG_01069	LA76x_1785	coaD; pantetheine-phosphate adenylyltransferase
OG_01070	LA76x_3097	LA76x_3097; bacterial regulatory, tetR family protein
OG_01071	LA76x_4608	LA76x_4608; C-terminal processing peptidase family protein
OG_01072	LA76x_4340	LA76x_4340; polymer-forming cytoskeletal family protein
OG_01073	LA76x_1626	LA76x_1626; 3-hydroxybutyrate dehydrogenase family protein
OG_01074	LA76x_2534	LA76x_2534; 5'-nucleotidase family protein
OG_01075	LA76x_4157	mrcA; penicillin-binding protein 1A
OG_01076	LA76x_1004	LA76x_1004; glyoxalase/Bleomycin resistance /Dioxygenase superfa
OG_01077	LA76x_155	LA76x_155; RNA polymerase sigma factor, TIGR02999 family protein
OG_01078	LA76x_4106	LA76x_4106; phenazine biosynthesis , PhzF family protein
OG_01079	LA76x_4022	glnS; glutamine--tRNA ligase
OG_01080	LA76x_786	czcA; cobalt-zinc-cadmium resistance protein czcA
OG_01081	LA76x_933	LA76x_933; hypothetical protein
OG_01082	LA76x_4369	LA76x_4369; efflux transporter, RND family, MFP subunit
OG_01083	LA76x_5042	LA76x_5042; conserved hypothetical family protein
OG_01084	LA76x_3506	LA76x_3506; hypothetical protein
OG_01085	LA76x_3092	LA76x_3092; conserved hypothetical protein
OG_01086	LA76x_3813	LA76x_3813; hypothetical protein
OG_01087	LA76x_787	LA76x_787; efflux transporter, RND family, MFP subunit
OG_01088	LA76x_3596	LA76x_3596; arginine-tRNA-transferase, C terminus family protein
OG_01089	LA76x_1832	LA76x_1832; metallo-beta-lactamase superfamily protein
OG_01090	LA76x_3185	LA76x_3185; hsc62, Hsp70 family chaperone, binds to RpoD and inh
OG_01091	LA76x_42	LA76x_42; NIPSNAP family protein
OG_01092	LA76x_2347	folD; bifunctional protein folD
OG_01093	LA76x_3829	zur; zur transcriptional repressor
OG_01094	LA76x_3472	LA76x_3472; phosphorylase superfamily protein
OG_01095	LA76x_1108	rimI; ribosomal-protein-alanine acetyltransferase
OG_01096	LA76x_3044	LA76x_3044; hypothetical protein
OG_01097	LA76x_24	LA76x_24; citrate transporter family protein
OG_01098	LA76x_3513	ycfF; purine nucleoside phosphoramidase
OG_01099	LA76x_4560	cc4; cytochrome c4 domain protein
OG_01100	LA76x_4224	LA76x_4224; conserved hypothetical protein
OG_01101	LA76x_4000	LA76x_4000; hypothetical protein
OG_01102	LA76x_4483	LA76x_4483; lipase family protein
OG_01103	LA76x_1219	LA76x_1219; putative membrane protein
OG_01104	LA76x_1369	LA76x_1369; lysE type translocator family protein
OG_01105	LA76x_3035	LA76x_3035; glucose / Sorbosone dehydrogenase family protein
OG_01106	LA76x_1991	lspA; signal peptidase II
OG_01107	LA76x_261	LA76x_261; glycosyl transferase 2 family protein
OG_01108	LA76x_2974	LA76x_2974; proton-translocating NADH-quinone oxidoreductase, ch
OG_01109	LA76x_2946	LA76x_2946; hypothetical protein
OG_01110	LA76x_4732	LA76x_4732; putative ACETYL-COA ACYLTRANSFERASE FADA2
OG_01111	LA76x_336	LA76x_336; conserved hypothetical protein
OG_01112	LA76x_1101	LA76x_1101; conserved hypothetical family protein
OG_01113	LA76x_2566	iscA; iron-sulfur cluster assembly protein
OG_01114	LA76x_1179	ftsZ; cell division protein FtsZ
OG_01115	LA76x_4222	LA76x_4222; bacterial regulatory helix-turn-helix, AraC family protein
OG_01116	LA76x_4046	thyA; thymidylate synthase
OG_01118	LA76x_4044	LA76x_4044; putative amidase domain protein
OG_01119	LA76x_2515	LA76x_2515; FKBP-type peptidyl-prolyl cis-trans isomerase family pr
OG_01120	LA76x_2502	LA76x_2502; lyase family protein
OG_01121	LA76x_1799	LA76x_1799; histidine kinase-, DNA gyrase B-, and HSP90-like ATPa
OG_01122	LA76x_848	lipB; lipoyl(octanoyl) transferase
OG_01123	LA76x_2388	ccmF; cytochrome c-type biogenesis protein CcmF
OG_01124	LA76x_4797	LA76x_4797; type III secretion apparatus protein, YscD/HrpQ family
OG_01125	LA76x_3014	LA76x_3014; yhhN-like family protein
OG_01126	LA76x_1003	LA76x_1003; metallo-beta-lactamase superfamily protein
OG_01127	LA76x_2565	LA76x_2565; conserved hypothetical protein
OG_01128	LA76x_3559	LA76x_3559; periplasmic glucan biosynthesis , MdoG family protein
OG_01129	LA76x_2442	LA76x_2442; glucoamylase family protein
OG_01130	LA76x_2328	LA76x_2328; acetyl-CoA C-acetyltransferase family protein
OG_01131	LA76x_1503	LA76x_1503; NHL repeat family protein
OG_01132	LA76x_3617	minE; cell division topological specificity factor MinE
OG_01133	LA76x_2062	folK; 2-amino-4-hydroxy-6- hydroxymethyldihydropteridine diphospho
OG_01134	LA76x_4775	LA76x_4775; conserved hypothetical protein
OG_01135	LA76x_3479	LA76x_3479; hypothetical protein
OG_01136	LA76x_3763	LA76x_3763; conserved hypothetical protein
OG_01137	LA76x_1635	LA76x_1635; hypothetical protein
OG_01138	LA76x_1146	glyA; glyA
OG_01139	LA76x_3009	LA76x_3009; SCO1/SenC family protein
OG_01140	LA76x_1948	rnr; ribonuclease R
OG_01141	LA76x_4905	LA76x_4905; multidrug resistance efflux transporter family protein
OG_01142	LA76x_1827	LA76x_1827; hypothetical protein
OG_01143	LA76x_3019	LA76x_3019; nlpE N-terminal domain protein
OG_01144	LA76x_318	LA76x_318; UDP-glucoronosyl and UDP-glucosyl transferase family p
OG_01145	LA76x_2212	uvrC; excinuclease ABC subunit C
OG_01146	LA76x_4515	LA76x_4515; citrate transporter family protein
OG_01147	LA76x_1543	LA76x_1543; alanine racemase, N-terminal domain protein
OG_01148	LA76x_4152	pilQ; type IV pilus secretin PilQ family protein

OG_01149	LA29479_4198	yecA family protein
OG_01150	LA29479_1667	ABC transporter family protein
OG_01151	LA29479_2284	phasin family domain protein
OG_01152	LA29479_4926	tyrosine recombinase XerC
OG_01153	LA29479_4074	3-Oxoacyl-[acyl-carrier-protein] reductase
OG_01154	LA29479_0636	thioredoxin
OG_01155	LA29479_2329	putative uncharacterized protein
OG_01156	LA29479_4440	spermidine synthase
OG_01157	LA29479_3424	tonB-dependent siderophore receptor family protein
OG_01158	LA29479_3080	bacterial regulatory helix-turn-helix , lysR family protein
OG_01159	LA29479_0946	UPF0213 protein BH0048
OG_01160	LA29479_0198	oxidoreductase iron/ascorbate family
OG_01161	LA29479_3472	3-isopropylmalate dehydratase, small subunit
OG_01162	LA29479_2210	conserved hypothetical protein
OG_01163	LA29479_4886	putative uncharacterized domain protein
OG_01164	LA29479_0825	ankyrin repeat family protein
OG_01165	LA29479_3954	cation diffusion facilitator transporter family protein
OG_01166	LA29479_3179	lysM domain protein
OG_01167	LA29479_0450	8-amino-7-oxononanoate synthase
OG_01168	LA29479_1971	UPF0502 protein yceH
OG_01169	LA29479_1755	hypothetical protein
OG_01170	LA29479_3590	RNA polymerase sigma factor, sigma-70 family protein
OG_01171	LA29479_0204	NADH-ubiquinone/plastoquinone oxidoreductase, chain 3 family prote
OG_01172	LA29479_2756	hypothetical protein
OG_01173	LA29479_4516	cytidylyltransferase family protein
OG_01174	LA29479_1641	succinate dehydrogenase, cytochrome b556 subunit
OG_01175	LA29479_4888	RNA polymerase sigma factor RpoE
OG_01176	LA29479_1318	phenylalanine-4-hydroxylase
OG_01177	LA29479_2234	3-dehydroquinate dehydratase, type II
OG_01178	LA29479_2343	ribosomal small subunit pseudouridine synthase A
OG_01179	LA29479_1528	putative lipoprotein
OG_01180	LA29479_4955	bis
OG_01181	LA29479_2197	putative uncharacterized protein
OG_01182	LA29479_3221	#NAME?
OG_01183	LA29479_2265	major Facilitator Superfamily protein
OG_01184	LA29479_1941	protein TolA
OG_01185	LA29479_0227	ribosomal protein S15
OG_01186	LA29479_3817	RNA polymerase sigma factor, sigma-70 family protein
OG_01187	LA29479_3400	ribosomal protein L2
OG_01188	LA29479_3566	asparaginase
OG_01189	LA29479_0117	conserved hypothetical protein
OG_01190	LA29479_1491	cytochrome C assembly family protein
OG_01191	LA29479_2602	catalase
OG_01192	LA29479_5143	hypothetical protein
OG_01193	LA29479_1300	mce related family protein
OG_01194	LA29479_4432	UPF0324 inner membrane protein yeiH
OG_01195	LA29479_1033	major Facilitator Superfamily protein
OG_01196	LA29479_0245	response regulator
OG_01197	LA29479_4798	sodium:dicarboxylate symporter family protein
OG_01198	LA29479_0153	hypothetical protein
OG_01199	LA29479_1894	hypothetical protein
OG_01200	LA29479_0772	septum site-determining protein MinD
OG_01201	LA29479_0545	stringent starvation protein B
OG_01202	LA29479_0475	RNA polymerase sigma factor, sigma-70 family protein
OG_01203	LA29479_3357	sugar (and other) transporter family protein
OG_01204	LA29479_4431	bacterial regulatory helix-turn-helix , lysR family protein
OG_01205	LA29479_2427	heparinase II/III-like family protein
OG_01206	LA29479_4971	glucose/galactose transporter WARNING family protein
OG_01207	LA29479_0292	7-cyano-7-deazaguanine reductase
OG_01208	LA29479_4934	ubiquinone/menaquinone biosynthesis methyltransferase ubiE
OG_01209	LA29479_2738	aminotransferase YbdL
OG_01210	LA29479_1236	hypothetical protein
OG_01211	LA29479_2819	putative uncharacterized protein
OG_01212	LA29479_2249	uncharacterized protein ynbD
OG_01214	LA29479_2788	putative uncharacterized protein
OG_01215	LA29479_2779	tRNA-specific adenosine deaminase
OG_01216	LA29479_0876	N-formylglutamate deformylase
OG_01217	LA29479_4832	amidohydrolase family Protein OLEI01672_1_465
OG_01218	LA29479_5004	ABC transporter family protein
OG_01219	LA29479_3202	tetratricopeptide repeat family protein
OG_01220	LA29479_4234	HNH endonuclease family protein
OG_01221	LA29479_1945	holliday junction DNA helicase RuvB
OG_01222	LA29479_0884	peptidyl-prolyl cis-trans isomerase
OG_01223	LA29479_1879	short chain dehydrogenase family protein
OG_01224	LA29479_0789	putative uncharacterized protein
OG_01225	LA29479_1830	peptide chain release factor 2
OG_01226	LA29479_4833	glutamate--tRNA ligase
OG_01227	LA29479_1992	saccharopine dehydrogenase
OG_01228	LA29479_0552	acetyltransferase family protein
OG_01229	LA29479_3371	coenzyme PQQ synthesis protein D
OG_01230	LA29479_4683	putative membrane protein

OG_01149	LA76x_4190	LA76x_4190; conserved hypothetical protein
OG_01150	LA76x_2168	LA76x_2168; ABC transporter family protein
OG_01151	LA76x_4348	LA76x_4348; phasin family protein
OG_01152	LA76x_812	xerC; tyrosine recombinase XerC
OG_01153	LA76x_257	LA76x_257; putative 3-Oxoacyl-[acyl-carrier-protein] reductase
OG_01154	LA76x_1639	LA76x_1639; putative thioredoxin
OG_01155	LA76x_4393	LA76x_4393; conserved hypothetical protein
OG_01156	LA76x_4642	speE; spermidine synthase
OG_01157	LA76x_4210	LA76x_4210; tonB-dependent siderophore receptor family protein
OG_01158	LA76x_893	LA76x_893; bacterial regulatory helix-turn-helix , lysR family protein
OG_01159	LA76x_3789	LA76x_3789; conserved hypothetical protein
OG_01160	LA76x_2992	LA76x_2992; 2OG-Fe(II) oxygenase superfamily protein
OG_01161	LA76x_4256	leuD; 3-isopropylmalate dehydratase, small subunit
OG_01162	LA76x_4276	LA76x_4276; mitochondrial fission ELM1 family protein
OG_01163	LA76x_3544	LA76x_3544; methyltransferase domain protein
OG_01164	LA76x_3667	LA76x_3667; ankyrin repeat family protein
OG_01165	LA76x_785	LA76x_785; cation diffusion facilitator transporter family protein
OG_01166	LA76x_1843	LA76x_1843; fecR family protein
OG_01167	LA76x_4909	bioF; 8-amino-7-oxononanoate synthase
OG_01168	LA76x_1230	LA76x_1230; conserved hypothetical protein
OG_01169	LA76x_2874	LA76x_2874; hypothetical protein
OG_01170	LA76x_3999	LA76x_3999; RNA polymerase sigma factor, sigma-70 family protein
OG_01171	LA76x_2986	LA76x_2986; NADH-ubiquinone/plastoquinone oxidoreductase, chain
OG_01172	LA76x_2378	LA76x_2378; hypothetical protein
OG_01173	LA76x_1918	LA76x_1918; cytidylyltransferase family protein
OG_01174	LA76x_2194	sdhC; succinate dehydrogenase, cytochrome b556 subunit
OG_01175	LA76x_3490	rpoE; RNA polymerase sigma factor RpoE
OG_01176	LA76x_54	phhA; phenylalanine-4-hydroxylase
OG_01177	LA76x_4301	aroQ; 3-dehydroquinate dehydratase, type II
OG_01178	LA76x_4407	rsuA; 16S rRNA pseudouridine 516 synthase
OG_01179	LA76x_1975	LA76x_1975; conserved hypothetical protein
OG_01180	LA76x_4043	apaH; bis
OG_01181	LA76x_960	LA76x_960; conserved hypothetical protein
OG_01182	LA76x_1805	secD; protein-export membrane protein SecD
OG_01183	LA76x_4330	LA76x_4330; major Facilitator Superfamily protein
OG_01184	LA76x_1258	tolA; protein TolA
OG_01185	LA76x_2963	rpsO; ribosomal protein S15
OG_01186	LA76x_4753	LA76x_4753; RNA polymerase sigma factor, sigma-70 family protein
OG_01187	LA76x_3925	rplB; ribosomal protein L2
OG_01188	LA76x_3974	LA76x_3974; asparaginase family protein
OG_01189	LA76x_3069	LA76x_3069; conserved hypothetical protein
OG_01190	LA76x_2011	LA76x_2011; cytochrome C assembly family protein
OG_01191	LA76x_529	katB; catalase
OG_01192	LA76x_2248	LA76x_2248; restriction endonuclease family protein
OG_01193	LA76x_73	LA76x_73; mce related family protein
OG_01194	LA76x_4644	LA76x_4644; conserved hypothetical family protein
OG_01195	LA76x_2618	LA76x_2618; major Facilitator Superfamily protein
OG_01196	LA76x_2944	LA76x_2944; bacterial regulatory , Fis family protein
OG_01197	LA76x_4144	LA76x_4144; sodium:dicarboxylate symporter family protein
OG_01198	LA76x_3034	LA76x_3034; hypothetical protein
OG_01199	LA76x_1400	LA76x_1400; hypothetical protein
OG_01200	LA76x_3616	minD; septum site-determining protein MinD
OG_01201	LA76x_1721	LA76x_1721; stringent starvation B family protein
OG_01202	LA76x_4883	LA76x_4883; RNA polymerase sigma factor, sigma-70 family protein
OG_01203	LA76x_3883	LA76x_3883; sugar (and other) transporter family protein
OG_01204	LA76x_4645	LA76x_4645; bacterial regulatory helix-turn-helix , lysR family protein
OG_01205	LA76x_3396	LA76x_3396; heparinase II/III-like family protein
OG_01206	LA76x_380	fucP; H+ symporter permease
OG_01207	LA76x_5152	queF; queuine synthase
OG_01208	LA76x_821	ubiE; 2-OCTAPRENYL-METHOXY-BENZOQ-METH bifunctional 2-oc
OG_01209	LA76x_2396	LA76x_2396; methionine aminotransferase, PLP-dependent
OG_01210	LA76x_132	LA76x_132; hypothetical protein
OG_01211	LA76x_2314	LA76x_2314; conserved hypothetical protein
OG_01212	LA76x_4316	LA76x_4316; putative phosphatase, inner membrane protein
OG_01214	LA76x_2345	LA76x_2345; conserved hypothetical protein
OG_01215	LA76x_2354	LA76x_2354; putative Cytosine/adenosine deaminase
OG_01216	LA76x_3715	hutG; N-formylglutamate deformylase
OG_01217	LA76x_3295	LA76x_3295; amidohydrolase family Protein OLEI01672_1_465
OG_01218	LA76x_4714	LA76x_4714; ABC transporter family protein
OG_01219	LA76x_1822	LA76x_1822; TPR repeat family protein
OG_01220	LA76x_1768	LA76x_1768; HNH endonuclease family protein
OG_01221	LA76x_1254	ruvB; holliday junction DNA helicase RuvB
OG_01222	LA76x_3724	LA76x_3724; FKBP-type peptidyl-prolyl cis-trans isomerase family pr
OG_01223	LA76x_2752	LA76x_2752; short chain dehydrogenase family protein
OG_01224	LA76x_3634	LA76x_3634; conserved hypothetical protein
OG_01225	LA76x_2798	prfB; peptide chain release factor 2
OG_01226	LA76x_3300	gluQ; glutamyl-queuosine tRNA(Asp) synthetase
OG_01227	LA76x_1210	LA76x_1210; conserved hypothetical protein
OG_01228	LA76x_1713	LA76x_1713; acetyltransferase family protein
OG_01229	LA76x_3897	pqqD; coenzyme PQQ biosynthesis protein PqqD
OG_01230	LA76x_4068	LA76x_4068; conserved hypothetical protein

OG_01231	LA29479_0055	putative uncharacterized protein XCC2022	OG_01231	LA76x_3132	LA76x_3132; zinc carboxypeptidase family protein
OG_01232	LA29479_3935	conserved hypothetical protein	OG_01232	LA76x_766	LA76x_766; conserved hypothetical protein
OG_01233	LA29479_4542	modulator of DNA gyrase family protein	OG_01233	LA76x_1892	LA76x_1892; modulator of DNA gyrase family protein
OG_01234	LA29479_1406	RNA pseudouridylate synthase family protein	OG_01234	LA76x_2098	LA76x_2098; RNA pseudouridylate synthase family protein
OG_01235	LA29479_3396	ribosomal protein L16	OG_01235	LA76x_3921	rplP; ribosomal protein L16
OG_01236	LA29479_4589	peptidyl-Asp metalloendopeptidase domain protein	OG_01236	LA76x_1409	LA76x_1409; peptidyl-Asp metalloendopeptidase domain protein
OG_01237	LA29479_0212	NADH dehydrogenase family protein	OG_01237	LA76x_2979	LA76x_2979; NADH dehydrogenase family protein
OG_01238	LA29479_2726	trbL/VirB6 plasmid conjugal transfer family protein	OG_01238	LA76x_2409	LA76x_2409; trbL/VirB6 plasmid conjugal transfer family protein
OG_01239	LA29479_2659	putative uncharacterized protein	OG_01239	LA76x_583	LA76x_583; conserved hypothetical protein
OG_01240	LA29479_0158	putative uncharacterized protein	OG_01240	LA76x_3030	LA76x_3030; conserved hypothetical protein
OG_01241	LA29479_3417	preprotein translocase, SecE subunit	OG_01241	LA76x_3943	secE; preprotein translocase, SecE subunit
OG_01242	LA29479_1271	putative uncharacterized protein	OG_01242	LA76x_102	LA76x_102; RNase_H superfamily protein
OG_01243	LA29479_4384	DSBA-like thioredoxin domain protein	OG_01243	LA76x_1453	LA76x_1453; DSBA-like thioredoxin domain protein
OG_01244	LA29479_4631	conserved domain protein	OG_01244	LA76x_3441	LA76x_3441; conserved domain protein
OG_01245	LA29479_4921	prolyl oligopeptidase family protein	OG_01245	LA76x_807	LA76x_807; prolyl oligopeptidase family protein
OG_01246	LA29479_1183	cell division protein ZipA	OG_01246	LA76x_2462	zipA; cell division protein ZipA
OG_01247	LA29479_4164	putative uncharacterized protein	OG_01247	LA76x_4158	LA76x_4158; conserved hypothetical protein
OG_01248	LA29479_1821	copper homeostasis protein cutC homolog	OG_01248	LA76x_2806	LA76x_2806; conserved hypothetical protein
OG_01249	LA29479_2096	30S ribosomal protein S9	OG_01249	LA76x_1056	rpsI; 30S ribosomal subunit protein S9
OG_01250	LA29479_4008	cytochrome c oxidase subunit III family protein	OG_01250	LA76x_4595	LA76x_4595; cytochrome c oxidase subunit III family protein
OG_01251	LA29479_1957	putative uncharacterized domain protein	OG_01251	LA76x_1242	LA76x_1242; concanavalin A-like lectin/glucanases superfamily prote
OG_01252	LA29479_0981	putative membrane protein	OG_01252	LA76x_3825	LA76x_3825; merC mercury resistance family protein
OG_01253	LA29479_1679	hypothetical protein	OG_01253	LA76x_2154	LA76x_2154; response regulator
OG_01254	LA29479_1280	GXGXG motif family protein	OG_01254	LA76x_92	LA76x_92; FMN-dependent dehydrogenase family protein
OG_01255	LA29479_0746	3-dehydroquinate synthase	OG_01255	LA76x_1529	aroB; 3-dehydroquinate synthase
OG_01256	LA29479_0471	M61 glycyl aminopeptidase family protein	OG_01256	LA76x_4887	LA76x_4887; M61 glycyl aminopeptidase family protein
OG_01257	LA29479_1053	hypothetical protein	OG_01257	LA76x_2589	LA76x_2589; hypothetical protein
OG_01258	LA29479_4189	conserved hypothetical protein	OG_01258	LA76x_4182	LA76x_4182; conserved hypothetical protein
OG_01259	LA29479_2662	putative uncharacterized protein	OG_01259	LA76x_586	LA76x_586; conserved hypothetical protein
OG_01260	LA29479_2269	fecR family protein	OG_01260	LA76x_4334	LA76x_4334; fecR family protein
OG_01261	LA29479_1349	hypothetical protein	OG_01261	LA76x_22	LA76x_22; hypothetical protein
OG_01262	LA29479_3487	O-Antigen Polymerase family protein	OG_01262	LA76x_4270	LA76x_4270; O-Antigen ligase family protein
OG_01263	LA29479_1395	deoxyribonuclease tatD	OG_01263	LA76x_5183	LA76x_5183; magnesium-dependent DNase
OG_01264	LA29479_2252	bifunctional purine biosynthesis protein PurH	OG_01264	LA76x_4319	purH; phosphoribosylaminoimidazolecarboxamide formyltransferase/I
OG_01265	LA29479_2106	anthranilate phosphoribosyltransferase	OG_01265	LA76x_1046	trpD; anthranilate phosphoribosyltransferase
OG_01266	LA29479_2088	twitching motility family protein	OG_01266	LA76x_1064	LA76x_1064; twitching motility family protein
OG_01267	LA29479_4319	conserved hypothetical protein	OG_01267	LA76x_1117	LA76x_1117; putative permease YjgP/YjgQ family protein
OG_01268	LA29479_0583	amino acid/peptide transporter family protein	OG_01268	LA76x_1683	LA76x_1683; H+ symporter) family protein
OG_01269	LA29479_3882	aquaporin Z	OG_01269	LA76x_3583	aqpZ; aqpZ - water MIP channel
OG_01270	LA29479_0195	acetyl-CoA carboxylase, carboxyl transferase, beta subunit	OG_01270	LA76x_2995	accD; acetyl-CoA carboxylase, carboxyl transferase, beta subunit
OG_01271	LA29479_2243	bacterial regulatory , Fis family protein	OG_01271	LA76x_4310	LA76x_4310; bacterial regulatory , Fis family protein
OG_01272	LA29479_1221	glutamine amidotransferase class-I family protein	OG_01272	LA76x_146	LA76x_146; glutamine amidotransferase class-I family protein
OG_01273	LA29479_0236	short chain dehydrogenase family protein	OG_01273	LA76x_2953	LA76x_2953; short chain dehydrogenase family protein
OG_01274	LA29479_3379	DNA-directed RNA polymerase, alpha subunit	OG_01274	LA76x_3904	rpoA; DNA-directed RNA polymerase, alpha subunit
OG_01275	LA29479_4899	3-oxoacyl-(acyl-carrier-protein) reductase	OG_01275	LA76x_3500	fabG; 3-oxoacyl-[acyl-carrier-protein] reductase
OG_01276	LA29479_2392	conserved hypothetical protein	OG_01276	LA76x_3361	LA76x_3361; conserved hypothetical protein
OG_01277	LA29479_4686	histidine kinase-, DNA gyrase B-, and HSP90-like ATPase family prote	OG_01277	LA76x_4071	LA76x_4071; response regulator
OG_01278	LA29479_1670	ferredoxin--NADP reductase	OG_01278	LA76x_2163	fpr; ferredoxin--NADP+ reductase
OG_01279	LA29479_3897	endonuclease/Exonuclease/phosphatase family protein	OG_01279	LA76x_3598	LA76x_3598; endonuclease/Exonuclease/phosphatase family protein
OG_01280	LA29479_4368	conserved hypothetical protein	OG_01280	LA76x_1469	LA76x_1469; conserved hypothetical protein
OG_01281	LA29479_3268	hypothetical protein	OG_01281	LA76x_212	LA76x_212; hypothetical protein
OG_01282	LA29479_0664	nickel transport complex, NikM subunit, transmembrane	OG_01282	LA76x_1611	LA76x_1611; conserved hypothetical protein
OG_01283	LA29479_3264	4'-phosphopantetheinyl transferase superfamily protein	OG_01283	LA76x_216	LA76x_216; 4'-phosphopantetheinyl transferase superfamily protein
OG_01284	LA29479_2348	ABC transporter family protein	OG_01284	LA76x_4412	LA76x_4412; ABC transporter family protein
OG_01285	LA29479_4324	DNA polymerase III chi subunit, HolC family protein	OG_01285	LA76x_1112	LA76x_1112; DNA polymerase III chi subunit, HolC family protein
OG_01286	LA29479_3476	glycerol kinase	OG_01286	LA76x_4260	glpK; glycerol kinase
OG_01287	LA29479_0824	phospholipase A1	OG_01287	LA76x_3666	LA76x_3666; conserved hypothetical protein
OG_01288	LA29479_3650	histidine biosynthesis bifunctional protein hisB	OG_01288	LA76x_3225	LA76x_3225; hisB
OG_01289	LA29479_5040	putative uncharacterized protein	OG_01289	LA76x_4700	LA76x_4700; conserved hypothetical protein
OG_01290	LA29479_3123	N-acetylmuramoyl-L-alanine amidase family protein	OG_01290	LA76x_935	LA76x_935; N-acetylmuramoyl-L-alanine amidase family protein
OG_01291	LA29479_4150	uracil-DNA glycosylase	OG_01291	LA76x_304	ung; uracil-DNA glycosylase
OG_01292	LA29479_4364	major Facilitator Superfamily protein	OG_01292	LA76x_1473	LA76x_1473; sugar (and other) transporter family protein
OG_01293	LA29479_1440	glucose-6-phosphate isomerase	OG_01293	LA76x_2066	pgi; glucose-6-phosphate isomerase
OG_01294	LA29479_4322	putative cytosol aminopeptidase	OG_01294	LA76x_1114	LA76x_1114; putative cytosol aminopeptidase
OG_01295	LA29479_0007	leucine dehydrogenase	OG_01295	LA76x_3177	ldh; leucine dehydrogenase
OG_01296	LA29479_3134	putative uncharacterized protein	OG_01296	LA76x_947	LA76x_947; EF hand family protein
OG_01297	LA29479_0723	aspartate carbamoyltransferase	OG_01297	LA76x_1552	pyrB; aspartate carbamoyltransferase
OG_01298	LA29479_0669	succinyl-diaminopimelate desuccinylase	OG_01298	LA76x_1606	LA76x_1606; peptidase M20/M25/M40 family protein
OG_01299	LA29479_4284	methyltransferase domain protein	OG_01299	LA76x_1151	LA76x_1151; methyltransferase domain protein
OG_01300	LA29479_1532	rhodanese-like domain protein	OG_01300	LA76x_1972	LA76x_1972; rhodanese-like domain protein
OG_01301	LA29479_1205	bolA-like family protein	OG_01301	LA76x_2439	Bola1; bolA-like protein 1
OG_01302	LA29479_3989	putative membrane protein	OG_01302	LA76x_4577	LA76x_4577; conserved hypothetical protein
OG_01303	LA29479_3903	putative integral membrane protein	OG_01303	LA76x_3603	LA76x_3603; putative integral membrane protein
OG_01304	LA29479_4735	fe/S biogenesis protein nfuA	OG_01304	LA76x_2296	gntY; iron-sulfur cluster scaffold protein
OG_01305	LA29479_3966	DMSO reductase anchor subunit family protein	OG_01305	LA76x_798	LA76x_798; DMSO reductase anchor subunit family protein
OG_01306	LA29479_1328	HTH domain protein	OG_01306	LA76x_43	LA76x_43; WYL domain protein
OG_01307	LA29479_1637	conserved hypothetical protein	OG_01307	LA76x_2198	LA76x_2198; MAPEG family protein
OG_01308	LA29479_2766	calU4	OG_01308	LA76x_2368	LA76x_2368; sulfatase family protein
OG_01309	LA29479_2124	conserved hypothetical protein	OG_01309	LA76x_1027	LA76x_1027; conserved hypothetical protein
OG_01310	LA29479_2800	ribosomal RNA large subunit methyltransferase N	OG_01310	LA76x_2333	rlmN; 23S rRNA m2A2503 methyltransferase
OG_01311	LA29479_2331	putative uncharacterized protein	OG_01311	LA76x_4395	LA76x_4395; conserved hypothetical protein

OG_01312	LA29479_2731	trbC/VIRB2 family protein
OG_01313	LA29479_4430	nitrogen regulatory P-II family protein
OG_01314	LA29479_1571	asparagine synthase
OG_01315	LA29479_4563	nucleoside recognition family protein
OG_01316	LA29479_1078	putative FMN-binding domain protein
OG_01317	LA29479_5149	zinc-binding dehydrogenase family protein
OG_01318	LA29479_0531	bacitracin resistance BacA family protein
OG_01319	LA29479_4819	major Facilitator Superfamily protein
OG_01320	LA29479_1187	tonB-dependent Receptor Plug domain protein
OG_01321	LA29479_1943	protein TolQ
OG_01322	LA29479_3881	hypothetical protein
OG_01323	LA29479_2886	cheR methyltransferase, all-alpha domain protein
OG_01324	LA29479_4329	O-succinylhomoserine (thiol)-lyase
OG_01325	LA29479_0032	peptidase, S8 (Subtilisin) family
OG_01326	LA29479_3027	D-alanyl-D-alanine carboxypeptidase dacC
OG_01327	LA29479_2759	pspC domain protein
OG_01328	LA29479_0953	putative uncharacterized protein
OG_01329	LA29479_3563	conserved hypothetical protein
OG_01330	LA29479_1417	UPF0271 protein PH0986
OG_01331	LA29479_0949	uncharacterized protein ybcJ
OG_01332	LA29479_4964	integral membrane TerC family protein
OG_01333	LA29479_2574	putative uncharacterized protein
OG_01334	LA29479_5120	tetratricopeptide repeat family protein
OG_01335	LA29479_0899	sodium:dicarboxylate symporter family protein
OG_01336	LA29479_2160	transcriptional activator, , Baf family protein
OG_01337	LA29479_4691	glutathione synthase
OG_01338	LA29479_0695	hypothetical protein
OG_01339	LA29479_3690	dnaJ domain protein
OG_01340	LA29479_4818	fe-S metabolism associated domain protein
OG_01341	LA29479_4604	ferritin-like domain protein
OG_01342	LA29479_1909	tRNA (guanine-N(7)-)-methyltransferase
OG_01343	LA29479_3547	tetratricopeptide repeat family protein
OG_01344	LA29479_1828	glycosyl hydrolases family 2, TIM barrel domain protein
OG_01345	LA29479_4050	acetyltransferase family protein
OG_01346	LA29479_5140	putative uncharacterized protein
OG_01347	LA29479_0364	HAMP domain protein
OG_01348	LA29479_2132	putative exported lectin/glucanase
OG_01349	LA29479_0914	putative uncharacterized protein
OG_01350	LA29479_3958	hypothetical protein
OG_01351	LA29479_3243	delta-aminolevulinic acid dehydratase
OG_01352	LA29479_4557	hemin uptake hemP domain protein
OG_01353	LA29479_3922	transcriptional regulator OhrR
OG_01354	LA29479_5049	ammonium transporter
OG_01355	LA29479_4576	methionine adenosyltransferase
OG_01356	LA29479_0896	NUDIX domain protein
OG_01357	LA29479_3147	trigger factor
OG_01358	LA29479_2125	putative membrane protein
OG_01359	LA29479_4221	hypothetical protein
OG_01360	LA29479_4512	penicillinase repressor family protein
OG_01361	LA29479_3267	putative chromosome-partitioning protein parB
OG_01362	LA29479_4732	protease Do family protein
OG_01363	LA29479_0874	putative membrane protein
OG_01364	LA29479_3613	putative uncharacterized protein
OG_01365	LA29479_4339	channel , hemolysin III family protein
OG_01366	LA29479_0159	luciferase-like monooxygenase family protein
OG_01367	LA29479_4023	2,3-bisphosphoglycerate-independent phosphoglycerate mutase
OG_01368	LA29479_1071	ribosomal protein S6
OG_01369	LA29479_2337	putative membrane protein
OG_01370	LA29479_2786	putative uncharacterized domain protein
OG_01371	LA29479_0841	RNA chaperone Hfq
OG_01372	LA29479_5001	putative uncharacterized protein
OG_01373	LA29479_2020	dephospho-CoA kinase
OG_01374	LA29479_5133	chorismate mutase
OG_01375	LA29479_4777	putative secreted protein
OG_01376	LA29479_3384	ribosomal protein L15
OG_01377	LA29479_4703	thiamine pyrophosphate enzyme, C-terminal TPP binding domain pro
OG_01378	LA29479_0706	lipopolysaccharide transport periplasmic protein LptA
OG_01379	LA29479_4303	fimbrial assembly family protein
OG_01380	LA29479_3173	conserved hypothetical protein
OG_01381	LA29479_4535	exodeoxyribonuclease VII, small subunit
OG_01382	LA29479_2564	acetoacetyl-CoA reductase
OG_01383	LA29479_3255	RDD family protein
OG_01384	LA29479_1301	toluene tolerance, Ttg2 family protein
OG_01385	LA29479_1132	hypothetical protein
OG_01386	LA29479_1437	iron-sulfur cluster binding protein, putative
OG_01387	LA29479_1339	fructose-1-6-bisphosphatase family protein
OG_01388	LA29479_2780	conserved hypothetical protein
OG_01389	LA29479_0481	acetyltransferase family protein
OG_01390	LA29479_3104	sir2 family protein
OG_01391	LA29479_5232	thiol:disulfide interchange protein DsbA
OG_01392	LA29479_2152	putative uncharacterized protein

OG_01312	LA76x_2403	LA76x_2403; trbC/VIRB2 family protein
OG_01313	LA76x_4646	LA76x_4646; nitrogen regulatory P-II family protein
OG_01314	LA76x_1936	asnB; asparagine synthase
OG_01315	LA76x_1436	LA76x_1436; na+ dependent nucleoside transporter family protein
OG_01316	LA76x_2560	LA76x_2560; putative FMN-binding domain protein
OG_01317	LA76x_636	LA76x_636; zinc-binding dehydrogenase family protein
OG_01318	LA76x_4830	LA76x_4830; bacitracin resistance BacA family protein
OG_01319	LA76x_3282	LA76x_3282; major Facilitator Superfamily protein
OG_01320	LA76x_2457	LA76x_2457; tonB dependent receptor family protein
OG_01321	LA76x_1256	tolQ; protein TolQ
OG_01322	LA76x_3582	LA76x_3582; hypothetical protein
OG_01323	LA76x_690	LA76x_690; cheR methyltransferase, SAM binding domain protein
OG_01324	LA76x_1505	metB; O-succinylhomoserine (thiol)-lyase
OG_01325	LA76x_3155	LA76x_3155; subtilase family protein
OG_01326	LA76x_843	dacC; penicillin-binding protein 6
OG_01327	LA76x_2375	LA76x_2375; pspC domain protein
OG_01328	LA76x_3796	LA76x_3796; conserved hypothetical protein
OG_01329	LA76x_3971	yihY; yihY family inner membrane domain protein
OG_01330	LA76x_2087	LA76x_2087; conserved hypothetical protein
OG_01331	LA76x_3792	LA76x_3792; S4 domain protein
OG_01332	LA76x_4051	LA76x_4051; integral membrane TerC family protein
OG_01333	LA76x_4525	LA76x_4525; conserved hypothetical protein
OG_01334	LA76x_3249	pgaA; poly-beta-1,6 N-acetyl-D-glucosamine export porin PgaA
OG_01335	LA76x_3739	LA76x_3739; sodium:dicarboxylate symporter family protein
OG_01336	LA76x_993	coaX; pantothenate kinase, type III family protein
OG_01337	LA76x_4076	gshB; glutathione synthase
OG_01338	LA76x_1579	LA76x_1579; hypothetical protein
OG_01339	LA76x_3187	LA76x_3187; dnaJ domain protein
OG_01340	LA76x_3281	LA76x_3281; fe-S metabolism associated domain protein
OG_01341	LA76x_4098	LA76x_4098; ferritin-like domain protein
OG_01342	LA76x_1384	trmB; tRNA (guanine-N(7)-)-methyltransferase
OG_01343	LA76x_3957	LA76x_3957; TPR repeat family protein
OG_01344	LA76x_2800	LA76x_2800; glycosyl hydrolases family 2, sugar binding domain prot
OG_01345	LA76x_277	LA76x_277; acetyltransferase family protein
OG_01346	LA76x_2251	LA76x_2251; conserved hypothetical protein
OG_01347	LA76x_5080	LA76x_5080; HAMP domain protein
OG_01348	LA76x_1020	LA76x_1020; putative exported lectin/glucanase
OG_01349	LA76x_3754	LA76x_3754; conserved hypothetical protein
OG_01350	LA76x_789	LA76x_789; hypothetical protein
OG_01351	LA76x_236	LA76x_236; delta-aminolevulinic acid dehydratase family protein
OG_01352	LA76x_1441	LA76x_1441; hemin uptake hemP family protein
OG_01353	LA76x_752	ohrR; transcriptional regulator OhrR
OG_01354	LA76x_4825	amt; ammonium transporter family protein
OG_01355	LA76x_1424	metK; methionine adenosyltransferase
OG_01356	LA76x_3736	LA76x_3736; NUDIX domain protein
OG_01357	LA76x_1873	tig; trigger factor
OG_01358	LA76x_1026	LA76x_1026; hypothetical protein
OG_01359	LA76x_1782	LA76x_1782; hypothetical protein
OG_01360	LA76x_1922	LA76x_1922; penicillinase repressor family protein
OG_01361	LA76x_213	LA76x_213; putative chromosome-partitioning protein parB
OG_01362	LA76x_3488	LA76x_3488; peptidase Do family protein
OG_01363	LA76x_3713	LA76x_3713; hypothetical protein
OG_01364	LA76x_4021	LA76x_4021; conserved hypothetical protein
OG_01365	LA76x_1495	LA76x_1495; channel , hemolysin III family protein
OG_01366	LA76x_3029	LA76x_3029; luciferase oxidoreductase, group 1 family protein
OG_01367	LA76x_4611	gpmI; 2,3-bisphosphoglycerate-independent phosphoglycerate mutas
OG_01368	LA76x_2568	rpsF; ribosomal protein S6
OG_01369	LA76x_4402	LA76x_4402; conserved hypothetical protein
OG_01370	LA76x_2346	LA76x_2346; conserved hypothetical protein
OG_01371	LA76x_3681	hfq; RNA chaperone Hfq
OG_01372	LA76x_4717	LA76x_4717; conserved hypothetical protein
OG_01373	LA76x_1185	coaE; dephospho-CoA kinase
OG_01374	LA76x_2258	pheA; chorismate mutase
OG_01375	LA76x_4122	LA76x_4122; conserved hypothetical protein
OG_01376	LA76x_3909	rplO; ribosomal protein L15
OG_01377	LA76x_3459	LA76x_3459; thiamine pyrophosphate enzyme, C-terminal TPP bindi
OG_01378	LA76x_1569	lptA; lipopolysaccharide transport periplasmic protein LptA
OG_01379	LA76x_1132	LA76x_1132; fimbrial assembly family protein
OG_01380	LA76x_1849	LA76x_1849; conserved hypothetical integral membrane family protei
OG_01381	LA76x_1899	xseB; exodeoxyribonuclease VII, small subunit
OG_01382	LA76x_4516	phbB; acetoacetyl-CoA reductase family protein
OG_01383	LA76x_225	LA76x_225; RDD family protein
OG_01384	LA76x_72	LA76x_72; toluene tolerance, Ttg2 family protein
OG_01385	LA76x_2512	LA76x_2512; hypothetical protein
OG_01386	LA76x_2067	LA76x_2067; putative iron-sulfur cluster-binding protein
OG_01387	LA76x_32	LA76x_32; fructose-1-6-bisphosphatase family protein
OG_01388	LA76x_2353	LA76x_2353; conserved hypothetical protein
OG_01389	LA76x_4877	LA76x_4877; acetyltransferase family protein
OG_01390	LA76x_916	LA76x_916; sir2 family protein
OG_01391	LA76x_4561	LA76x_4561; disulfide oxidoreductase
OG_01392	LA76x_1000	LA76x_1000; conserved hypothetical protein

OG_01393	LA29479_3251	putative uncharacterized protein	OG_01393	LA76x_229	LA76x_229; conserved hypothetical protein
OG_01394	LA29479_2556	glutathione S-transferase	OG_01394	LA76x_4508	bphK; glutathione S-transferase
OG_01395	LA29479_4676	putative transmembrane protein	OG_01395	LA76x_4061	LA76x_4061; conserved hypothetical protein
OG_01396	LA29479_4880	putative uncharacterized protein	OG_01396	LA76x_3538	LA76x_3538; conserved hypothetical protein
OG_01397	LA29479_2934	putative D-serine deaminase (D-serine dehydratase) protein	OG_01397	LA76x_725	LA76x_725; putative D-serine deaminase (D-serine dehydratase) pro
OG_01398	LA29479_3604	alkyl hydroperoxide reductase AhpD	OG_01398	LA76x_4012	ahpD; alkyl hydroperoxide reductase AhpD
OG_01399	LA29479_2689	putative uncharacterized protein	OG_01399	LA76x_616	LA76x_616; conserved hypothetical protein
OG_01400	LA29479_4279	transcription antitermination factor NusB	OG_01400	LA76x_1156	nusB; transcription antitermination factor NusB
OG_01401	LA29479_2735	conjugal transfer family protein	OG_01401	LA76x_2399	LA76x_2399; conjugal transfer family protein
OG_01402	LA29479_2912	glutathione-dependent formaldehyde-activating enzyme family protein	OG_01402	LA76x_714	LA76x_714; glutathione-dependent formaldehyde-activating enzyme
OG_01403	LA29479_3824	bacterial regulatory , arsR family protein	OG_01403	LA76x_4746	LA76x_4746; bacterial regulatory , arsR family protein
OG_01404	LA29479_3783	hypothetical protein	OG_01404	LA76x_4780	LA76x_4780; hypothetical protein
OG_01405	LA29479_4523	hypothetical protein	OG_01405	LA76x_1910	LA76x_1910; hypothetical protein
OG_01406	LA29479_2311	conserved hypothetical protein	OG_01406	LA76x_4374	LA76x_4374; conserved hypothetical protein
OG_01407	LA29479_4345	CBS domain	OG_01407	LA76x_1490	LA76x_1490; CBS domain
OG_01408	LA29479_5023	binding--dependent transport system inner membrane component fan	OG_01408	LA76x_4552	LA76x_4552; binding-protein-dependent transport system inner mem
OG_01409	LA29479_1013	putative uncharacterized protein XOO3489	OG_01409	LA76x_3855	LA76x_3855; conserved hypothetical protein
OG_01410	LA29479_1206	protein ycil	OG_01410	LA76x_2438	LA76x_2438; YCII-related domain protein
OG_01411	LA29479_3095	glutathione S-transferase, C-terminal domain protein	OG_01411	LA76x_906	LA76x_906; glutathione S-transferase, C-terminal domain protein
OG_01412	LA29479_0587	glucose-1-phosphate thymidylyltransferase	OG_01412	LA76x_1679	rfbA; glucose-1-phosphate thymidylyltransferase
OG_01413	LA29479_4011	cytochrome c oxidase, subunit I	OG_01413	LA76x_4598	ctaD; cytochrome c oxidase, subunit I
OG_01414	LA29479_0154	penicillin-binding protein 1C	OG_01414	LA76x_3033	pbpC; penicillin-binding protein 1C
OG_01415	LA29479_2500	cold shock protein CapB	OG_01415	LA76x_4454	LA76x_4454; 'Cold-shock' DNA-binding domain protein
OG_01416	LA29479_2340	UPF0234 protein yajQ	OG_01416	LA76x_4404	yajQ; nucleotide binding protein
OG_01417	LA29479_0678	siroheme synthase	OG_01417	LA76x_1596	cysG; cysG
OG_01418	LA29479_0648	patatin-like phospholipase family protein	OG_01418	LA76x_1627	LA76x_1627; patatin-like phospholipase family protein
OG_01419	LA29479_2117	lipid kinase YegS	OG_01419	LA76x_1035	yegS; lipid kinase YegS
OG_01420	LA29479_3471	3-isopropylmalate dehydratase, large subunit	OG_01420	LA76x_4255	leuC; 3-isopropylmalate dehydratase, large subunit
OG_01421	LA29479_0355	putative membrane protein	OG_01421	LA76x_5089	LA76x_5089; putative transmembrane protein
OG_01422	LA29479_3940	lysE type translocator family protein	OG_01422	LA76x_771	LA76x_771; lysE type translocator family protein
OG_01423	LA29479_4811	acetylornithine deacetylase	OG_01423	LA76x_3274	argE; acetylornithine deacetylase
OG_01424	LA29479_1477	DNA mismatch repair protein MutS	OG_01424	LA76x_2026	mutS; DNA mismatch repair protein MutS
OG_01425	LA29479_4457	putative uncharacterized domain protein	OG_01425	LA76x_4625	LA76x_4625; conserved hypothetical protein
OG_01426	LA29479_4545	septum formation protein Maf	OG_01426	LA76x_1888	maf; septum formation protein Maf
OG_01427	LA29479_3941	putative uncharacterized protein	OG_01427	LA76x_772	LA76x_772; conserved hypothetical protein
OG_01428	LA29479_0526	RDD family protein	OG_01428	LA76x_4835	LA76x_4835; RDD family protein
OG_01429	LA29479_1997	glutamine-dependent NAD(+) synthetase	OG_01429	LA76x_1205	nadE; NAD+ synthetase
OG_01430	LA29479_1448	hypothetical protein	OG_01430	LA76x_2058	LA76x_2058; hypothetical protein
OG_01431	LA29479_3592	putative secreted protein	OG_01431	LA76x_4001	LA76x_4001; conserved hypothetical protein
OG_01432	LA29479_2502	bacterial SH3 domain protein	OG_01432	LA76x_4457	LA76x_4457; peptidase M23 family protein
OG_01433	LA29479_4742	putative carboxyvinyl-carboxyphosphonate phosphorylmutase	OG_01433	LA76x_2289	LA76x_2289; phosphoenolpyruvate phosphomutase family protein
OG_01434	LA29479_1842	conserved hypothetical protein	OG_01434	LA76x_2788	LA76x_2788; conserved hypothetical protein
OG_01435	LA29479_3646	trp repressor family protein	OG_01435	LA76x_3229	LA76x_3229; trp repressor family protein
OG_01436	LA29479_0866	3-deoxy-8-phosphooctulonate synthase	OG_01436	LA76x_3705	kdsA; 3-deoxy-8-phosphooctulonate synthase
OG_01437	LA29479_4828	conserved hypothetical protein	OG_01437	LA76x_3291	LA76x_3291; type I GTP cyclohydrolase folE2 family protein
OG_01438	LA29479_3335	bacterial regulatory helix-turn-helix , lysR family protein	OG_01438	LA76x_3860	LA76x_3860; bacterial regulatory helix-turn-helix , lysR family protein
OG_01439	LA29479_3562	ahpC/TSA family protein	OG_01439	LA76x_3970	LA76x_3970; thioredoxin family protein
OG_01440	LA29479_4925	conserved hypothetical protein	OG_01440	LA76x_811	LA76x_811; conserved hypothetical protein
OG_01441	LA29479_2000	succinyl-CoA ligase [ADP-forming] subunit alpha	OG_01441	LA76x_1202	sucD; succinyl-CoA synthetase, alpha subunit
OG_01442	LA29479_3950	xdhC and CoxI family protein	OG_01442	LA76x_781	LA76x_781; xdhC and CoxI family protein
OG_01443	LA29479_4530	DNA polymerase III subunit alpha	OG_01443	LA76x_1904	LA76x_1904; DNA polymerase III, &alpha subunit
OG_01444	LA29479_1672	putative membrane protein	OG_01444	LA76x_2161	LA76x_2161; acyltransferase family protein
OG_01445	LA29479_1168	hypothetical protein	OG_01445	LA76x_2477	LA76x_2477; hypothetical protein
OG_01446	LA29479_1163	peroxiredoxin-2E-2, chloroplastic	OG_01446	LA76x_2482	PRXIIE-2; peroxiredoxin-2E-2, chloroplastic
OG_01447	LA29479_2405	putative uncharacterized domain protein	OG_01447	LA76x_3374	LA76x_3374; conserved hypothetical protein
OG_01448	LA29479_1479	catalase/peroxidase HPI	OG_01448	LA76x_2024	katG; catalase/peroxidase HPI
OG_01449	LA29479_4456	putative membrane protein	OG_01449	LA76x_4626	LA76x_4626; putative beta-lactamase induction signal transducer Am
OG_01450	LA29479_0792	putative uncharacterized protein	OG_01450	LA76x_3637	LA76x_3637; phosphatase 2C family protein
OG_01451	LA29479_4536	farnesyl diphosphate synthase	OG_01451	LA76x_1898	ispA; geranyltranstransferase
OG_01452	LA29479_1892	hypothetical protein	OG_01452	LA76x_1402	LA76x_1402; hypothetical protein
OG_01453	LA29479_1098	kynureninase	OG_01453	LA76x_2543	kynU; kynureninase
OG_01454	LA29479_3397	ribosomal protein S3	OG_01454	LA76x_3922	rpsC; ribosomal protein S3
OG_01455	LA29479_3495	mgtC family protein	OG_01455	LA76x_2741	LA76x_2741; mgtC family protein
OG_01456	LA29479_2743	heme exporter CcmC family protein	OG_01456	LA76x_2391	ccmC; heme exporter CcmC family protein
OG_01457	LA29479_3364	aminotransferase class I and II family protein	OG_01457	LA76x_3890	LA76x_3890; aminotransferase class I and II family protein
OG_01458	LA29479_1204	bacterial regulatory s, lacI family protein	OG_01458	LA76x_2440	LA76x_2440; periplasmic binding and sugar binding domain of LacI fa
OG_01459	LA29479_2813	tetratricopeptide repeat family protein	OG_01459	LA76x_2321	LA76x_2321; TPR repeat family protein
OG_01460	LA29479_1169	alpha-2-macroglobulin N-terminal region family protein	OG_01460	LA76x_2476	LA76x_2476; alpha-2-macroglobulin N-terminal region family protein
OG_01461	LA29479_0939	DEAD/DEAH box helicase family protein	OG_01461	LA76x_3781	LA76x_3781; helicase conserved C-terminal domain protein
OG_01462	LA29479_1151	acetyltransferase family protein	OG_01462	LA76x_2493	LA76x_2493; acetyltransferase family protein
OG_01463	LA29479_4461	tyrosyl-tRNA synthetase	OG_01463	LA76x_4622	tyrS; tyrosine--tRNA ligase
OG_01464	LA29479_0259	putative uncharacterized protein	OG_01464	LA76x_2929	LA76x_2929; marR family protein
OG_01465	LA29479_3996	DNA primase	OG_01465	LA76x_4583	dnaG; DNA primase
OG_01466	LA29479_2588	S-formylglutathione hydrolase	OG_01466	LA76x_4539	fghA; S-formylglutathione hydrolase
OG_01467	LA29479_1724	ABC transporter family protein	OG_01467	LA76x_2110	ccmA; heme ABC exporter, ATP-binding protein CcmA
OG_01468	LA29479_4483	RNA pseudouridylate synthase family protein	OG_01468	LA76x_4676	LA76x_4676; RNA pseudouridylate synthase family protein
OG_01469	LA29479_2155	entericidin EcnA/B family protein	OG_01469	LA76x_998	LA76x_998; entericidin EcnA/B family protein
OG_01470	LA29479_2476	uncharacterized isochorismatase family protein HVO_2328	OG_01470	LA76x_4429	LA76x_4429; conserved hypothetical protein
OG_01471	LA29479_4450	deoxyuridine 5'-triphosphate nucleotidohydrolase	OG_01471	LA76x_4632	dut; dut
OG_01472	LA29479_4090	sulfate ABC transporter, sulfate-binding family protein	OG_01472	LA76x_241	LA76x_241; sulfate ABC transporter, sulfate-binding family protein
OG_01473	LA29479_3432	PAP2 superfamily protein	OG_01473	LA76x_4216	LA76x_4216; conserved hypothetical protein

OG_01474	LA29479_1273	putative secreted protein
OG_01475	LA29479_0440	4-hydroxybenzoate polyprenyl transferase
OG_01476	LA29479_0978	membrane dipeptidase family protein
OG_01477	LA29479_5193	kamA family protein
OG_01478	LA29479_1629	biopolymer transport ExbD/TolR family protein
OG_01479	LA29479_3254	RDD family protein
OG_01480	LA29479_4257	translation initiation factor IF-3
OG_01481	LA29479_0232	bacterial regulatory helix-turn-helix s, AraC family protein
OG_01482	LA29479_1578	protein-P-II uridylyltransferase
OG_01483	LA29479_4454	acetyltransferase family protein
OG_01484	LA29479_4949	methionine-R-sulfoxide reductase
OG_01485	LA29479_2773	conserved hypothetical protein
OG_01486	LA29479_2489	acriflavine resistance protein A
OG_01487	LA29479_0786	transcriptional regulatory , C terminal family protein
OG_01488	LA29479_0261	NAD dependent epimerase/dehydratase family protein
OG_01489	LA29479_5005	putative membrane protein
OG_01490	LA29479_1843	tRNA (5-methylaminomethyl-2-thiouridylate)-methyltransferase
OG_01491	LA29479_0305	aminoglycoside/hydroxyurea antibiotic resistance kinase family protei
OG_01492	LA29479_0384	hypothetical protein
OG_01493	LA29479_4376	alcohol dehydrogenase [acceptor]
OG_01494	LA29479_0003	acetyl-CoA acetyltransferases family protein
OG_01495	LA29479_4455	exodeoxyribonuclease III
OG_01496	LA29479_3991	dihydroneopterin aldolase
OG_01497	LA29479_2024	putative uncharacterized protein
OG_01498	LA29479_1864	protein CrcB
OG_01499	LA29479_3224	S-adenosylmethionine:tRNA ribosyltransferase-isomerase
OG_01500	LA29479_3256	protein YrdA
OG_01501	LA29479_4445	sporulation related domain protein
OG_01502	LA29479_1136	putative secreted protein
OG_01503	LA29479_4871	moaE family protein
OG_01504	LA29479_4459	peptidase M23 family protein
OG_01505	LA29479_0854	putative membrane protein
OG_01506	LA29479_1895	putative uncharacterized protein
OG_01507	LA29479_4555	tonB-dependent heme/hemoglobin receptor family protein
OG_01508	LA29479_4009	cytochrome c oxidase assembly CtaG / Cox11 family protein
OG_01509	LA29479_4786	S1/P1 Nuclease family protein
OG_01510	LA29479_3850	putative uncharacterized domain protein
OG_01511	LA29479_2491	DSBA-like thioredoxin domain protein
OG_01512	LA29479_4232	putative uncharacterized protein
OG_01513	LA29479_4072	short chain dehydrogenase family protein
OG_01514	LA29479_1867	binding--dependent transport system inner membrane component fan
OG_01515	LA29479_4033	transcription termination factor Rho
OG_01516	LA29479_2035	UDP-N-acetylmuramoyl-tripeptide--D-alanyl-D- alanine ligase
OG_01517	LA29479_3878	phosphoglycerate mutase family protein
OG_01518	LA29479_3353	2-deoxystreptamine pathway aminotransferase protein
OG_01519	LA29479_0135	phage Tail Collar domain protein
OG_01521	LA29479_4593	putative uncharacterized protein
OG_01522	LA29479_1263	conserved hypothetical protein
OG_01523	LA29479_2099	2-nonaprenyl-3-methyl-6-methoxy-1,4-benzoquinol hydroxylase
OG_01524	LA29479_1237	hypothetical protein
OG_01525	LA29479_1519	ribosomal protein L27
OG_01526	LA29479_4534	tRNA(Ile)-lysidine synthetase
OG_01527	LA29479_2812	glycosyl transferase 4 family protein
OG_01528	LA29479_3366	GTP-binding protein TypA/BipA
OG_01529	LA29479_1682	glycerophosphoryl diester phosphodiesterase family protein
OG_01530	LA29479_0266	putative uncharacterized protein
OG_01531	LA29479_4951	PPIC-type PPIASE domain protein
OG_01532	LA29479_3052	ribonucleoside-diphosphate reductase, adenosylcobalamin-depender
OG_01533	LA29479_1210	formamidopyrimidine-DNA glycosylase
OG_01534	LA29479_1597	nucleotide sugar dehydrogenase family protein
OG_01535	LA29479_0573	putative uncharacterized protein
OG_01536	LA29479_0302	major Facilitator Superfamily protein
OG_01537	LA29479_4879	glycosyltransferase 9 family protein
OG_01538	LA29479_1891	conserved hypothetical protein
OG_01539	LA29479_2324	ATP synthase F1, delta subunit
OG_01540	LA29479_2480	SPFH domain / Band 7 family protein
OG_01541	LA29479_4684	cheW-like domain protein
OG_01542	LA29479_0793	von Willebrand factor type A domain protein
OG_01543	LA29479_4540	modulator of DNA gyrase family protein
OG_01544	LA29479_2937	alpha-N-acetylgalactosaminidase
OG_01545	LA29479_3642	histidyl-tRNA synthetase
OG_01546	LA29479_3893	RNA polymerase sigma factor, sigma-70 family protein
OG_01547	LA29479_0668	uncharacterized protein HI_1008
OG_01548	LA29479_2913	bacterial regulatory s, gntR family protein
OG_01549	LA29479_4983	CDP-Glycerol:Poly(glycerophosphate) glycerophosphotransferase far
OG_01550	LA29479_0993	sporulation related domain protein
OG_01551	LA29479_0461	hypothetical protein
OG_01552	LA29479_0842	tRNA delta(2)-isopentenylpyrophosphate transferase
OG_01553	LA29479_4361	putative uncharacterized protein
OG_01554	LA29479_2342	HAD-superhydrolase, sublA, variant 3 family protein
OG_01555	LA29479_4206	impB/mucB/samB family protein

OG_01474	LA76x_100	LA76x_100; peptidase inhibitor I78 family protein
OG_01475	LA76x_4917	ubiA; 4-hydroxybenzoate polyprenyl transferase
OG_01476	LA76x_3822	LA76x_3822; membrane dipeptidase family protein
OG_01477	LA76x_3297	LA76x_3297; kamA family protein
OG_01478	LA76x_2206	LA76x_2206; biopolymer transport ExbD/TolR family protein
OG_01479	LA76x_226	LA76x_226; RDD family protein
OG_01480	LA76x_1745	infC; translation initiation factor IF-3
OG_01481	LA76x_2958	LA76x_2958; bacterial regulatory helix-turn-helix, AraC family protein
OG_01482	LA76x_1930	glnD; protein-P-II uridylyltransferase
OG_01483	LA76x_4628	LA76x_4628; acetyltransferase family protein
OG_01484	LA76x_4037	msrB; methionine-R-sulfoxide reductase
OG_01485	LA76x_2360	LA76x_2360; kinase/pyrophosphorylase family protein
OG_01486	LA76x_4442	acrA; acrA Membrane Fusion Protein
OG_01487	LA76x_3631	LA76x_3631; transcriptional regulatory , C terminal family protein
OG_01488	LA76x_2927	LA76x_2927; NAD dependent epimerase/dehydratase family protein
OG_01489	LA76x_4713	LA76x_4713; ABC-2 transporter family protein
OG_01490	LA76x_2787	trmU; tRNA (5-methylaminomethyl-2-thiouridylate)-methyltransferase
OG_01491	LA76x_5138	LA76x_5138; aminoglycoside/hydroxyurea antibiotic resistance kinas
OG_01492	LA76x_5063	LA76x_5063; hypothetical protein
OG_01493	LA76x_1461	alkJ; alcohol dehydrogenase [acceptor]
OG_01494	LA76x_3181	LA76x_3181; acetyl-CoA C-acetyltransferase family protein
OG_01495	LA76x_4627	xth; exodeoxyribonuclease III
OG_01496	LA76x_4578	folB; dihydroneopterin aldolase
OG_01497	LA76x_1181	LA76x_1181; conserved hypothetical protein
OG_01498	LA76x_2766	LA76x_2766; crcB-like family protein
OG_01499	LA76x_1802	queA; tRNA ribosyltransferase-isomerase
OG_01500	LA76x_224	LA76x_224; bacterial transferase hexapeptide family protein
OG_01501	LA76x_4637	LA76x_4637; sporulation related domain protein
OG_01502	LA76x_2508	LA76x_2508; conserved hypothetical protein
OG_01503	LA76x_3530	LA76x_3530; moaE family protein
OG_01504	LA76x_4623	LA76x_4623; peptidase M23 family protein
OG_01505	LA76x_3693	LA76x_3693; putative transmembrane protein
OG_01506	LA76x_1399	LA76x_1399; GDSL-like Lipase/Acylhydrolase family protein
OG_01507	LA76x_1443	LA76x_1443; tonB-dependent hemoglobin/transferrin/lactoferrin recep
OG_01508	LA76x_4596	LA76x_4596; cytochrome c oxidase assembly CtaG/Cox11 family pro
OG_01509	LA76x_4132	LA76x_4132; S1/P1 Nuclease family protein
OG_01510	LA76x_3550	LA76x_3550; conserved hypothetical protein
OG_01511	LA76x_4444	LA76x_4444; DSBA-like thioredoxin domain protein
OG_01512	LA76x_1770	LA76x_1770; late embryogenesis abundant family protein
OG_01513	LA76x_259	LA76x_259; short chain dehydrogenase family protein
OG_01514	LA76x_2762	potI; potI
OG_01515	LA76x_297	rho; transcription termination factor Rho
OG_01516	LA76x_1170	LA76x_1170; UDP
OG_01517	LA76x_3579	LA76x_3579; histidine phosphatase super family protein
OG_01518	LA76x_3878	LA76x_3878; suppressor of fused family protein
OG_01519	LA76x_3052	LA76x_3052; phage Tail Collar domain protein
OG_01521	LA76x_4089	LA76x_4089; conserved hypothetical protein
OG_01522	LA76x_108	LA76x_108; methyltransferase domain protein
OG_01523	LA76x_1053	LA76x_1053; ubiquinone biosynthesis COQ7 family protein
OG_01524	LA76x_131	LA76x_131; hypothetical protein
OG_01525	LA76x_1984	rpmA; ribosomal protein L27
OG_01526	LA76x_1900	tilS; tRNA(Ile)-lysidine synthetase
OG_01527	LA76x_2322	LA76x_2322; glycosyl transferase 4 family protein
OG_01528	LA76x_3892	typA; GTP-binding protein TypA/BipA
OG_01529	LA76x_2150	LA76x_2150; glycerophosphoryl diester phosphodiesterase family pro
OG_01530	LA76x_5179	LA76x_5179; conserved hypothetical protein
OG_01531	LA76x_4039	LA76x_4039; surA N-terminal domain protein
OG_01532	LA76x_868	LA76x_868; ribonucleoside-diphosphate reductase, adenosylcobalam
OG_01533	LA76x_156	mutM; formamidopyrimidine-DNA glycosylase
OG_01534	LA76x_2237	LA76x_2237; nucleotide sugar dehydrogenase family protein
OG_01535	LA76x_1691	LA76x_1691; conserved hypothetical protein
OG_01536	LA76x_5141	LA76x_5141; major Facilitator Superfamily protein
OG_01537	LA76x_3537	LA76x_3537; glycosyltransferase 9 family protein
OG_01538	LA76x_1403	LA76x_1403; ATP-dependant zinc protease family protein
OG_01539	LA76x_4387	atpH; ATP synthase F1, delta subunit
OG_01540	LA76x_4433	LA76x_4433; SPFH domain / Band 7 family protein
OG_01541	LA76x_4069	LA76x_4069; cheW-like domain protein
OG_01542	LA76x_3638	LA76x_3638; von Willebrand factor type A domain protein
OG_01543	LA76x_1894	LA76x_1894; modulator of DNA gyrase family protein
OG_01544	LA76x_728	nagA; alpha-N-acetylgalactosaminidase
OG_01545	LA76x_3234	hisS; histidine--tRNA ligase
OG_01546	LA76x_3594	LA76x_3594; RNA polymerase sigma factor, sigma-70 family protein
OG_01547	LA76x_1607	LA76x_1607; conserved hypothetical protein
OG_01548	LA76x_715	LA76x_715; bacterial regulatory, gntR family protein
OG_01549	LA76x_369	LA76x_369; CDP-Glycerol:Poly(glycerophosphate) glycerophosphotra
OG_01550	LA76x_3836	LA76x_3836; sporulation related domain protein
OG_01551	LA76x_4898	LA76x_4898; hlyD secretion family protein
OG_01552	LA76x_3682	miaA; tRNA dimethylallyltransferase
OG_01553	LA76x_1475	LA76x_1475; conserved hypothetical protein
OG_01554	LA76x_4406	LA76x_4406; HAD hydrolase, IA, variant 3 family protein
OG_01555	LA76x_4197	LA76x_4197; impB/mucB/samB family protein

OG_01556	LA29479_4469	beta-lactamase family protein	OG_01556	LA76x_4690	LA76x_4690; beta-lactamase family protein
OG_01557	LA29479_4709	putative uncharacterized protein TTHA1053	OG_01557	LA76x_3465	LA76x_3465; conserved hypothetical protein
OG_01558	LA29479_0707	ABC transporter family protein	OG_01558	LA76x_1568	LA76x_1568; ABC transporter family protein
OG_01559	LA29479_2811	short chain dehydrogenase family protein	OG_01559	LA76x_2323	LA76x_2323; short chain dehydrogenase family protein
OG_01560	LA29479_3679	enoyl-CoA hydratase/isomerase family protein	OG_01560	LA76x_3197	LA76x_3197; enoyl-CoA hydratase/isomerase family protein
OG_01561	LA29479_1681	NAD-dependent ADP-ribosyltransferase sirtuin-4	OG_01561	LA76x_2152	SIRT4; NAD-dependent ADP-ribosyltransferase sirtuin-4
OG_01562	LA29479_4097	chitin binding domain protein	OG_01562	LA76x_356	LA76x_356; chitin binding domain protein
OG_01563	LA29479_2804	enoyl-CoA hydratase/isomerase family protein	OG_01563	LA76x_2329	LA76x_2329; enoyl-CoA hydratase/isomerase family protein
OG_01564	LA29479_2789	putative uncharacterized protein	OG_01564	LA76x_2344	LA76x_2344; alpha/beta hydrolase fold family protein
OG_01565	LA29479_3241	sulfate ABC transporter, permease protein CysW	OG_01565	LA76x_238	cysW; sulfate ABC transporter, permease protein CysW
OG_01566	LA29479_0904	uncharacterised family protein	OG_01566	LA76x_3744	LA76x_3744; conserved hypothetical protein
OG_01567	LA29479_0309	AFG1-like ATPase family protein	OG_01567	LA76x_5135	LA76x_5135; AFG1-like ATPase family protein
OG_01568	LA29479_2772	conserved hypothetical protein	OG_01568	LA76x_2361	LA76x_2361; conserved hypothetical protein
OG_01569	LA29479_4671	efflux transporter, RND family, MFP subunit	OG_01569	LA76x_4056	LA76x_4056; efflux transporter, RND family, MFP subunit
OG_01570	LA29479_3183	ATP-dependent RNA helicase rhlE	OG_01570	LA76x_1840	LA76x_1840; DEAD-box-containing ATP-dependent RNA helicase fa
OG_01571	LA29479_1097	2-amino-3-carboxymuconate-6-semialdehyde decarboxylase	OG_01571	LA76x_2544	Acmsd; 2-amino-3-carboxymuconate-6-semialdehyde decarboxylase
OG_01572	LA29479_3102	fatty acid hydroxylase superfamily protein	OG_01572	LA76x_914	LA76x_914; fatty acid hydroxylase superfamily protein
OG_01573	LA29479_1546	phosphate transport system regulatory protein PhoU	OG_01573	LA76x_1958	phoU; phosphate transport system regulatory protein PhoU
OG_01574	LA29479_4071	conserved hypothetical protein	OG_01574	LA76x_260	LA76x_260; conserved hypothetical protein
OG_01575	LA29479_4138	sulfatase family protein	OG_01575	LA76x_316	LA76x_316; sulfatase family protein
OG_01576	LA29479_0858	smr domain protein	OG_01576	LA76x_3697	LA76x_3697; smr domain protein
OG_01577	LA29479_2741	heme ABC exporter, ATP-binding protein CcmA	OG_01577	LA76x_2393	ccmA; heme ABC exporter, ATP-binding protein CcmA
OG_01578	LA29479_0665	apbE family protein	OG_01578	LA76x_1610	LA76x_1610; apbE family protein
OG_01579	LA29479_0672	sulfate adenylyltransferase, small subunit	OG_01579	LA76x_1603	cysD; sulfate adenylyltransferase, small subunit
OG_01580	LA29479_1216	conserved hypothetical protein	OG_01580	LA76x_151	LA76x_151; conserved hypothetical protein
OG_01581	LA29479_4332	peptidase M23 family protein	OG_01581	LA76x_1502	LA76x_1502; conserved hypothetical protein
OG_01582	LA29479_3626	major Facilitator Superfamily protein	OG_01582	LA76x_3244	LA76x_3244; transmembrane secretion effector family protein
OG_01583	LA29479_4121	bacterial regulatory s, gntR family protein	OG_01583	LA76x_333	LA76x_333; bacterial regulatory, gntR family protein
OG_01584	LA29479_2949	conserved hypothetical protein	OG_01584	LA76x_740	LA76x_740; conserved hypothetical protein
OG_01585	LA29479_0890	thioesterase superfamily protein	OG_01585	LA76x_3729	LA76x_3729; thioesterase superfamily protein
OG_01586	LA29479_2958	multidrug resistance ABC transporter ATP-binding/permease protein	OG_01586	LA76x_2690	bmrA; multidrug resistance ABC transporter ATP-binding/permease p
OG_01587	LA29479_2469	hypothetical protein	OG_01587	LA76x_4423	LA76x_4423; hypothetical protein
OG_01588	LA29479_3433	ATP-dependent protease La domain protein	OG_01588	LA76x_4217	LA76x_4217; ATP-dependent protease La domain protein
OG_01589	LA29479_5013	tRNA (cytidine/uridine-2'-O-)-methyltransferase TrmL	OG_01589	LA76x_4705	trmL; tRNA (cytidine(34)-2'-O)-methyltransferase
OG_01590	LA29479_3407	ribosomal protein S7	OG_01590	LA76x_3933	rpsG; ribosomal protein S7
OG_01591	LA29479_2777	putative uncharacterized protein	OG_01591	LA76x_2356	LA76x_2356; conserved hypothetical protein
OG_01592	LA29479_3560	putative uncharacterized protein XOO4025	OG_01592	LA76x_3968	LA76x_3968; conserved hypothetical protein
OG_01593	LA29479_2749	tetratricopeptide repeat family protein	OG_01593	LA76x_2385	LA76x_2385; TPR repeat family protein
OG_01594	LA29479_4356	hypothetical protein	OG_01594	LA76x_1481	LA76x_1481; hypothetical protein
OG_01595	LA29479_1290	hypothetical protein	OG_01595	LA76x_81	LA76x_81; conserved hypothetical protein
OG_01596	LA29479_3274	ribosomal protein L33	OG_01596	LA76x_206	rpmG; ribosomal protein L33
OG_01597	LA29479_0220	ribosome maturation factor rimP domain protein	OG_01597	LA76x_2970	rimP; ribosome maturation domain protein
OG_01598	LA29479_3464	dehydratase family protein	OG_01598	LA76x_4248	ilvD; dihydroxy-acid dehydratase
OG_01600	LA29479_4821	RNA polymerase-binding protein DksA	OG_01600	LA76x_3284	dksA; RNA polymerase-binding protein DksA
OG_01601	LA29479_0327	trbL/VirB6 plasmid conjugal transfer family protein	OG_01601	LA76x_5117	LA76x_5117; trbL/VirB6 plasmid conjugal transfer family protein
OG_01602	LA29479_2276	putative transmembrane protein	OG_01602	LA76x_4341	LA76x_4341; putative transmembrane protein
OG_01603	LA29479_1568	polysaccharide deacetylase family protein	OG_01603	LA76x_1939	LA76x_1939; polysaccharide deacetylase family protein
OG_01604	LA29479_2025	UDP-3-O-[3-hydroxymyristoyl] N-acetylglucosamine deacetylase	OG_01604	LA76x_1180	lpxC; UDP-3-O-[3-hydroxymyristoyl] N-acetylglucosamine deacetylas
OG_01605	LA29479_1920	efflux transporter, RND family, MFP subunit	OG_01605	LA76x_1372	LA76x_1372; efflux transporter, RND family, MFP subunit
OG_01606	LA29479_2943	FAD dependent oxidoreductase family protein	OG_01606	LA76x_734	LA76x_734; FAD binding domain protein
OG_01607	LA29479_2097	ribosomal protein L13	OG_01607	LA76x_1055	rplM; ribosomal protein L13
OG_01608	LA29479_0870	putative uncharacterized protein	OG_01608	LA76x_3709	LA76x_3709; conserved hypothetical protein
OG_01609	LA29479_4727	GTP-binding protein Era	OG_01609	LA76x_3483	era; GTP-binding protein Era
OG_01610	LA29479_0766	cytochrome c family protein	OG_01610	LA76x_1510	LA76x_1510; cytochrome c family protein
OG_01611	LA29479_0659	benzene 1,2-dioxygenase system ferredoxin subunit	OG_01611	LA76x_1616	bnzC; benzene 1,2-dioxygenase system ferredoxin subunit
OG_01612	LA29479_4374	calcineurin-like phosphoesterase family protein	OG_01612	LA76x_1463	LA76x_1463; calcineurin-like phosphoesterase family protein
OG_01613	LA29479_5173	rare lipoA family protein	OG_01613	LA76x_842	rlpA; rare lipoA family protein
OG_01614	LA29479_4876	metallo-beta-lactamase superfamily protein	OG_01614	LA76x_3534	LA76x_3534; metallo-beta-lactamase superfamily protein
OG_01615	LA29479_0076	hypothetical protein	OG_01615	LA76x_3112	LA76x_3112; hypothetical protein
OG_01616	LA29479_1494	efflux transporter, outer membrane factor (OMF) lipo, NodT family pro	OG_01616	LA76x_2008	LA76x_2008; efflux transporter, outer membrane factor (OMF) lipo, N
OG_01617	LA29479_4693	putative uncharacterized domain protein	OG_01617	LA76x_4078	LA76x_4078; conserved hypothetical protein
OG_01618	LA29479_4441	arginine decarboxylase	OG_01618	LA76x_4641	speA; arginine decarboxylase
OG_01619	LA29479_2720	putative lipoprotein	OG_01619	LA76x_2415	LA76x_2415; hypothetical protein
OG_01620	LA29479_3343	recF/RecN/SMC N terminal domain protein	OG_01620	LA76x_3868	LA76x_3868; recF/RecN/SMC N terminal domain protein
OG_01621	LA29479_3559	putative uncharacterized protein	OG_01621	LA76x_3967	LA76x_3967; conserved hypothetical protein
OG_01622	LA29479_0697	PAS fold family protein	OG_01622	LA76x_1578	LA76x_1578; sensory box protein
OG_01623	LA29479_1936	radical SAM superfamily protein	OG_01623	LA76x_1263	LA76x_1263; radical SAM superfamily protein
OG_01624	LA29479_4893	DNA polymerase III, delta' subunit	OG_01624	LA76x_3494	holB; DNA polymerase III, delta' subunit
OG_01625	LA29479_2901	hypothetical protein	OG_01625	LA76x_703	LA76x_703; hypothetical protein
OG_01626	LA29479_1381	DNA binding , excisionase family domain protein	OG_01626	LA76x_5197	LA76x_5197; DNA binding , excisionase family domain protein
OG_01627	LA29479_3236	metallo-beta-lactamase superfamily protein	OG_01627	LA76x_1790	LA76x_1790; metallo-beta-lactamase superfamily protein
OG_01628	LA29479_1905	putative uncharacterized protein	OG_01628	LA76x_1389	LA76x_1389; conserved hypothetical protein
OG_01629	LA29479_0541	N5-carboxyaminoimidazole ribonucleotide mutase	OG_01629	LA76x_1725	purE; purE
OG_01630	LA29479_0847	ribosomal RNA large subunit methyltransferase E	OG_01630	LA76x_3686	rrmJ; 23S rRNA 2'-O-ribose U2552 methyltransferase
OG_01631	LA29479_1643	putative glycine cleavage system t (Aminomethyltransferase) protein	OG_01631	LA76x_2192	LA76x_2192; aminomethyltransferase folate-binding domain protein
OG_01632	LA29479_4010	putative membrane protein	OG_01632	LA76x_4597	LA76x_4597; hypothetical protein
OG_01633	LA29479_1010	2-amino-3-ketobutyrate coenzyme A ligase	OG_01633	LA76x_3852	kbl; 2-amino-3-ketobutyrate coenzyme A ligase
OG_01634	LA29479_4375	putative membrane protein	OG_01634	LA76x_1462	LA76x_1462; TLC ATP/ADP transporter family protein
OG_01635	LA29479_0495	bacterial regulatory s, lacI family protein	OG_01635	LA76x_4864	LA76x_4864; periplasmic binding and sugar binding domain of LacI fa
OG_01636	LA29479_3993	ribosomal protein S21	OG_01636	LA76x_4580	rpsU; ribosomal protein S21
OG_01637	LA29479_4722	putative uncharacterized protein	OG_01637	LA76x_3478	LA76x_3478; conserved hypothetical protein

OG_01638	LA29479_3500	putative uncharacterized protein
OG_01639	LA29479_3861	glycosyl transferases group 1 family protein
OG_01640	LA29479_1863	replication-associated recombination protein A
OG_01641	LA29479_1918	acrB/AcrD/AcrF family protein
OG_01642	LA29479_4574	acyltransferase family protein
OG_01643	LA29479_0770	homoserine dehydrogenase family protein
OG_01644	LA29479_2049	H-NS histone family protein
OG_01645	LA29479_1924	fructose-bisphosphate aldolase
OG_01646	LA29479_5134	phosphoserine aminotransferase
OG_01647	LA29479_4518	RIP metalloprotease RseP
OG_01648	LA29479_2232	thioredoxin family protein
OG_01649	LA29479_1076	putative transmembrane protein
OG_01650	LA29479_0675	phosphoadenosine phosphosulfate reductase
OG_01651	LA29479_4909	putative membrane protein
OG_01652	LA29479_0487	bacterial regulatory s, gntR family protein
OG_01653	LA29479_2163	degV family protein
OG_01654	LA29479_3622	putative 2-octaprenyl-6-methoxyphenol hydroxylase domain protein
OG_01655	LA29479_2345	putative membrane protein
OG_01656	LA29479_4915	recombination protein RecR
OG_01657	LA29479_0601	tonB-dependent Receptor Plug domain protein
OG_01658	LA29479_3925	outer membrane autotransporter barrel domain protein
OG_01659	LA29479_0509	gamma-glutamyltransferase
OG_01660	LA29479_4274	uncharacterised UPF0102 family protein
OG_01661	LA29479_5012	major Facilitator Superfamily protein
OG_01662	LA29479_2334	ompW family protein
OG_01663	LA29479_0129	zinc-binding dehydrogenase family protein
OG_01664	LA29479_0991	amidophosphoribosyltransferase
OG_01665	LA29479_1962	aspartyl-tRNA synthetase
OG_01666	LA29479_1678	peptidase M3 family protein
OG_01667	LA29479_4750	hypothetical protein
OG_01668	LA29479_3803	hypothetical protein
OG_01669	LA29479_1450	dihydrodipicolinate synthase
OG_01670	LA29479_1756	sensory box protein
OG_01671	LA29479_4292	ABC transporter family protein
OG_01672	LA29479_0656	feS assembly protein SufD
OG_01673	LA29479_0643	putative uncharacterized protein
OG_01674	LA29479_2456	heavy metal sensor kinase family protein
OG_01675	LA29479_4824	dihydroorotase
OG_01676	LA29479_0357	putative uncharacterized protein
OG_01677	LA29479_2126	conserved hypothetical protein
OG_01678	LA29479_3586	NAD(P) transhydrogenase beta subunit
OG_01679	LA29479_1542	phosphate ABC transporter, phosphate-binding protein PstS
OG_01680	LA29479_4442	putative uncharacterized protein yjpA
OG_01681	LA29479_4791	ABC transporter family protein
OG_01682	LA29479_3582	bacterial regulatory helix-turn-helix s, AraC family protein
OG_01683	LA29479_4173	putative uncharacterized protein
OG_01684	LA29479_0803	histidine kinase-, DNA gyrase B-, and HSP90-like ATPase family prot
OG_01685	LA29479_1266	heat shock protein
OG_01686	LA29479_4572	aspartate racemase family protein
OG_01687	LA29479_3909	hhH-GPD superbase excision DNA repair family protein
OG_01688	LA29479_1543	phosphate ABC transporter, permease protein PstC
OG_01689	LA29479_1230	ferrochelatase
OG_01690	LA29479_1625	low molecular weight phosphotyrosine phosphatase family protein
OG_01691	LA29479_4812	argininosuccinate synthase
OG_01692	LA29479_5204	ribosomal protein L25, Ctc-form
OG_01693	LA29479_4940	glycosyl hydrolases 25 family protein
OG_01694	LA29479_4967	permease family protein
OG_01695	LA29479_2781	bacterial regulatory s, tetR family protein
OG_01696	LA29479_1834	response regulator
OG_01697	LA29479_4095	RDD family protein
OG_01698	LA29479_2774	phosphoenolpyruvate synthase
OG_01699	LA29479_1054	copper resistance protein A
OG_01700	LA29479_3350	putative uncharacterized protein
OG_01701	LA29479_4473	transporter, solute:sodium symporter family protein
OG_01702	LA29479_4954	protein ApaG
OG_01703	LA29479_4588	5,10-methylenetetrahydrofolate reductase
OG_01704	LA29479_4626	UDP-N-acetylenolpyruvoylglucosamine reductase
OG_01705	LA29479_2115	anthranilate synthase component I
OG_01706	LA29479_3539	ubiquinol oxidase, subunit II
OG_01707	LA29479_3926	peptidase propeptide and YPEB domain protein
OG_01708	LA29479_4089	phosphate-selective porin O and P family protein
OG_01709	LA29479_3435	adenylate kinase
OG_01710	LA29479_0170	transcription elongation factor GreB
OG_01711	LA29479_3891	acyl-CoA thioesterase II
OG_01712	LA29479_3864	acetyltransferase, GNAT family
OG_01713	LA29479_3670	short chain dehydrogenase family protein
OG_01714	LA29479_3605	alkyl hydroperoxide reductase subunit C
OG_01715	LA29479_2807	FAD dependent oxidoreductase family protein
OG_01716	LA29479_2767	radical SAM superfamily protein
OG_01717	LA29479_5205	ribose-phosphate pyrophosphokinase
OG_01718	LA29479_0578	conserved hypothetical protein

OG_01638	LA76x_2736	LA76x_2736; T5orf172 domain protein
OG_01639	LA76x_3561	LA76x_3561; glycosyl transferases group 1 family protein
OG_01640	LA76x_2767	ycaJ; recombination factor
OG_01641	LA76x_1374	LA76x_1374; MMPL family protein
OG_01642	LA76x_1426	LA76x_1426; acyltransferase family protein
OG_01643	LA76x_1506	LA76x_1506; homoserine dehydrogenase family protein
OG_01644	LA76x_1103	LA76x_1103; H-NS histone family protein
OG_01645	LA76x_1368	fbab; fructose-bisphosphate aldolase
OG_01646	LA76x_2257	serC; phosphoserine transaminase
OG_01647	LA76x_1916	rseP; RIP metalloprotease RseP
OG_01648	LA76x_4299	LA76x_4299; thioredoxin family protein
OG_01649	LA76x_2562	LA76x_2562; putative transmembrane protein
OG_01650	LA76x_1599	cysH; phosphoadenosine phosphosulfate reductase
OG_01651	LA76x_3508	LA76x_3508; dolichyl-phosphate-mannose-mannosyltransferase fam
OG_01652	LA76x_4871	LA76x_4871; bacterial regulatory, gntR family protein
OG_01653	LA76x_990	LA76x_990; EDD, DegV family domain protein
OG_01654	LA76x_4031	LA76x_4031; ubiquinone biosynthesis hydroxylase, UbiH/UbiF/VisC/(
OG_01655	LA76x_4409	LA76x_4409; conserved hypothetical protein
OG_01656	LA76x_3514	recR; recombination protein RecR
OG_01657	LA76x_1666	LA76x_1666; tonB dependent receptor family protein
OG_01658	LA76x_756	LA76x_756; autotransporter beta-domain protein
OG_01659	LA76x_4850	ggt; gamma-glutamyltransferase
OG_01660	LA76x_1161	LA76x_1161; conserved hypothetical protein
OG_01661	LA76x_4706	LA76x_4706; major Facilitator Superfamily protein
OG_01662	LA76x_4399	LA76x_4399; outer membrane beta-barrel domain protein
OG_01663	LA76x_3058	LA76x_3058; zinc-binding dehydrogenase family protein
OG_01664	LA76x_3834	purF; amidophosphoribosyltransferase
OG_01665	LA76x_1238	aspS; aspartate--tRNA ligase
OG_01666	LA76x_2155	LA76x_2155; peptidase M3 family protein
OG_01667	LA76x_2283	LA76x_2283; hypothetical protein
OG_01668	LA76x_4764	LA76x_4764; hypothetical protein
OG_01669	LA76x_2056	dapA; dihydrodipicolinate synthase
OG_01670	LA76x_2873	LA76x_2873; sensory box protein
OG_01671	LA76x_1143	LA76x_1143; ATP-binding cassette , ChvD family protein
OG_01672	LA76x_1619	sufD; feS assembly protein SufD
OG_01673	LA76x_1632	LA76x_1632; toxin-antitoxin system toxin component, PIN family
OG_01674	LA76x_3420	LA76x_3420; heavy metal sensor kinase family protein
OG_01675	LA76x_3287	pyrC; dihydroorotase
OG_01676	LA76x_5087	LA76x_5087; conserved hypothetical protein
OG_01677	LA76x_1025	LA76x_1025; eamA-like transporter family protein
OG_01678	LA76x_3995	pntB; NAD(P) transhydrogenase subunit beta
OG_01679	LA76x_1962	pstS; phosphate ABC transporter, phosphate-binding protein PstS
OG_01680	LA76x_4640	LA76x_4640; YCII-related domain protein
OG_01681	LA76x_4137	LA76x_4137; ABC transporter family protein
OG_01682	LA76x_3990	LA76x_3990; bacterial regulatory helix-turn-helix, AraC family protein
OG_01683	LA76x_4167	LA76x_4167; cytochrome c family protein
OG_01684	LA76x_3647	LA76x_3647; histidine kinase-, DNA gyrase B-, and HSP90-like ATPa
OG_01685	LA76x_106	LA76x_106; hsp70 family protein
OG_01686	LA76x_1428	LA76x_1428; aspartate racemase family protein
OG_01687	LA76x_3609	LA76x_3609; hhH-GPD superbase excision DNA repair family protein
OG_01688	LA76x_1961	pstC; phosphate ABC transporter, permease protein PstC
OG_01689	LA76x_138	hemH; ferrochelatase
OG_01690	LA76x_2210	LA76x_2210; low molecular weight phosphotyrosine phosphatase far
OG_01691	LA76x_3275	LA76x_3275; asparagine synthase family protein
OG_01692	LA76x_3952	LA76x_3952; ribosomal protein L25, Ctc-form
OG_01693	LA76x_827	LA76x_827; glycosyl hydrolases 25 family protein
OG_01694	LA76x_4054	LA76x_4054; ftsX-like permease family protein
OG_01695	LA76x_2352	LA76x_2352; bacterial regulatory, tetR family protein
OG_01696	LA76x_2796	LA76x_2796; response regulator
OG_01697	LA76x_358	LA76x_358; RDD family protein
OG_01698	LA76x_2359	ppsA; phosphoenolpyruvate synthase
OG_01699	LA76x_2588	copA; copper resistance protein A
OG_01700	LA76x_3875	LA76x_3875; conserved hypothetical protein
OG_01701	LA76x_4686	LA76x_4686; sodium symporter family protein
OG_01702	LA76x_4042	apaG; protein ApaG
OG_01703	LA76x_1410	metF; 5,10-methylenetetrahydrofolate reductase
OG_01704	LA76x_3436	murB; UDP-N-acetylenolpyruvoylglucosamine reductase
OG_01705	LA76x_1037	trpE; anthranilate synthase component I
OG_01706	LA76x_2697	cyoA; ubiquinol oxidase, subunit II
OG_01707	LA76x_757	LA76x_757; pepSY-associated TM helix family protein
OG_01708	LA76x_242	LA76x_242; conserved hypothetical protein
OG_01709	LA76x_4219	adk; adenylate kinase
OG_01710	LA76x_3020	greB; transcription elongation factor GreB
OG_01711	LA76x_3592	tesB; acyl-CoA thioesterase II
OG_01712	LA76x_3564	LA76x_3564; acetyltransferase family protein
OG_01713	LA76x_3205	LA76x_3205; short chain dehydrogenase family protein
OG_01714	LA76x_4013	ahpC; alkyl hydroperoxide reductase subunit C
OG_01715	LA76x_2326	LA76x_2326; pyridine nucleotide-disulfide oxidoreductase family prote
OG_01716	LA76x_2367	LA76x_2367; radical SAM superfamily protein
OG_01717	LA76x_3953	prs; ribose-phosphate pyrophosphokinase
OG_01718	LA76x_1687	LA76x_1687; conserved hypothetical protein

OG_01719	LA29479_1659	phospholipase A1 family protein
OG_01720	LA29479_1634	putative uncharacterized domain protein
OG_01721	LA29479_3865	inner membrane CreD family protein
OG_01722	LA29479_2349	GDSL-like Lipase/Acylhydrolase family protein
OG_01723	LA29479_1291	bacterial regulatory helix-turn-helix , lysR family protein
OG_01724	LA29479_3550	peptide chain release factor 1
OG_01725	LA29479_0130	redox-sensitive transcriptional activator SoxR
OG_01726	LA29479_1590	bacterial regulatory s, tetR family protein
OG_01727	LA29479_0868	CTP synthase
OG_01728	LA29479_0645	inner membrane protein ytfF
OG_01729	LA29479_4226	acetyltransferase family protein
OG_01730	LA29479_1654	rickettsia 17 kDa surface antigen family protein
OG_01731	LA29479_2533	nucleoprotein/polynucleotide-associated enzyme
OG_01732	LA29479_4771	ribonucleoside-diphosphate reductase, alpha subunit
OG_01733	LA29479_2161	biotin-[acetyl-CoA-carboxylase] ligase
OG_01734	LA29479_3468	branched-chain amino acid aminotransferase
OG_01735	LA29479_0711	phosphoenolpyruvate-dependent sugar phosphotransferase system,
OG_01736	LA29479_0872	DNA topoisomerase IV, B subunit
OG_01737	LA29479_2823	acetyltransferase family protein
OG_01738	LA29479_0197	sulfatase family protein
OG_01739	LA29479_1651	tatD related DNase family protein
OG_01740	LA29479_4109	putative uncharacterized protein
OG_01741	LA29479_4335	hypothetical protein
OG_01742	LA29479_1463	response regulator
OG_01743	LA29479_0952	nicotinamide phosphoribosyltransferase
OG_01744	LA29479_0255	DNA polymerase III, alpha subunit
OG_01745	LA29479_1541	phosphate ABC transporter, phosphate-binding protein PstS
OG_01746	LA29479_3818	fecR family protein
OG_01747	LA29479_0959	ATPase
OG_01748	LA29479_3452	transcriptional regulatory , C terminal family protein
OG_01749	LA29479_4712	hypothetical protein
OG_01750	LA29479_3475	major intrinsic family protein
OG_01751	LA29479_2576	AMP nucleosidase, putative
OG_01752	LA29479_3078	drug resistance MFS transporter, drug:H+ antiporter-1 family protein
OG_01753	LA29479_2222	chaperonin GroL
OG_01754	LA29479_4941	pfkB carbohydrate kinase family protein
OG_01755	LA29479_4807	prolyl-tRNA synthetase
OG_01756	LA29479_0532	glutamine synthetase, type I
OG_01757	LA29479_1593	putative uncharacterized protein
OG_01758	LA29479_1596	conserved region in glutamate synthase family protein
OG_01759	LA29479_4449	DNA / pantothenate metabolism flavofamily protein
OG_01760	LA29479_0485	N-acetylglucosamine-6-phosphate deacetylase
OG_01761	LA29479_1511	sodium/hydrogen exchanger family protein
OG_01762	LA29479_1360	biopolymer transport ExbD/TolR family protein
OG_01763	LA29479_0660	tonB-dependent siderophore receptor family protein
OG_01764	LA29479_3446	F5/8 type C domain protein
OG_01765	LA29479_2814	putative membrane protein
OG_01766	LA29479_2561	response regulator
OG_01767	LA29479_3398	ribosomal protein L22
OG_01768	LA29479_1386	alpha/beta hydrolase fold family protein
OG_01769	LA29479_4929	NAD dependent epimerase/dehydratase family protein
OG_01770	LA29479_1646	possible glutathione S-transferase
OG_01771	LA29479_1953	cob(I)alamin adenosyltransferase
OG_01772	LA29479_0594	metallo-beta-lactamase superfamily protein
OG_01773	LA29479_3358	aerobic C4-dicarboxylate transport protein
OG_01774	LA29479_4004	cytochrome oxidase assembly family protein
OG_01775	LA29479_5022	conserved hypothetical protein
OG_01776	LA29479_5061	bacterial regulatory s, lacI family protein
OG_01777	LA29479_0286	uncharacterised family protein
OG_01778	LA29479_1392	putative uncharacterized domain protein
OG_01779	LA29479_1121	homocysteine S-methyltransferase family protein
OG_01780	LA29479_4201	putative uncharacterized protein
OG_01781	LA29479_2111	HAMP domain protein
OG_01782	LA29479_4383	glycine cleavage system H protein
OG_01783	LA29479_1729	glutathione S-transferase, N-terminal domain protein
OG_01784	LA29479_4541	conserved hypothetical protein
OG_01785	LA29479_3912	putative uncharacterized protein
OG_01786	LA29479_4019	rhomboid family protein
OG_01787	LA29479_2834	cytochrome c554 domain protein
OG_01788	LA29479_3466	acetolactate synthase, large subunit, biosynthetic type
OG_01789	LA29479_0062	PHB depolymerase PhaZ7
OG_01790	LA29479_4838	conserved hypothetical protein
OG_01791	LA29479_1993	putative uncharacterized domain protein
OG_01792	LA29479_4996	protein-export chaperone SecB
OG_01793	LA29479_2021	mutator mutT family protein
OG_01794	LA29479_4962	conserved hypothetical protein
OG_01795	LA29479_2230	conserved hypothetical protein
OG_01796	LA29479_0489	6-phosphogluconolactonase
OG_01797	LA29479_2570	methyltransferase domain protein
OG_01798	LA29479_1310	hypothetical protein
OG_01799	LA29479_3455	thiamine biosynthesis protein ThiC

OG_01719	LA76x_2176	LA76x_2176; phospholipase A1 family protein
OG_01720	LA76x_2201	LA76x_2201; hypothetical protein
OG_01721	LA76x_3565	LA76x_3565; inner membrane CreD family protein
OG_01722	LA76x_4413	LA76x_4413; GDSL-like Lipase/Acylhydrolase family protein
OG_01723	LA76x_80	LA76x_80; bacterial regulatory helix-turn-helix , lysR family protein
OG_01724	LA76x_3960	prfA; peptide chain release factor 1
OG_01725	LA76x_3057	soxR; redox-sensitive transcriptional activator SoxR
OG_01726	LA76x_2245	LA76x_2245; bacterial regulatory, tetR family protein
OG_01727	LA76x_3707	pyrG; CTP synthase
OG_01728	LA76x_1630	LA76x_1630; eamA-like transporter family protein
OG_01729	LA76x_1777	LA76x_1777; acetyltransferase family protein
OG_01730	LA76x_2181	LA76x_2181; glycine zipper 2TM domain protein
OG_01731	LA76x_4488	LA76x_4488; conserved hypothetical protein
OG_01732	LA76x_4116	LA76x_4116; ribonucleoside-diphosphate reductase, alpha subunit
OG_01733	LA76x_992	LA76x_992; biotin--[acetyl-CoA-carboxylase] ligase
OG_01734	LA76x_4252	LA76x_4252; branched-chain amino acid aminotransferase
OG_01735	LA76x_1564	LA76x_1564; phosphoenolpyruvate-dependent sugar phosphotransfe
OG_01736	LA76x_3711	parE; DNA topoisomerase IV, B subunit
OG_01737	LA76x_2310	LA76x_2310; acetyltransferase family protein
OG_01738	LA76x_2993	LA76x_2993; type I phosphodiesterase / nucleotide pyrophosphatase
OG_01739	LA76x_2184	LA76x_2184; tatD related DNase family protein
OG_01740	LA76x_344	LA76x_344; conserved hypothetical protein
OG_01741	LA76x_1499	LA76x_1499; hypothetical protein
OG_01742	LA76x_2041	LA76x_2041; transcriptional regulatory , C terminal family protein
OG_01743	LA76x_3795	NAMPT; nicotinamide phosphoribosyltransferase
OG_01744	LA76x_2933	dnaE; DNA polymerase III, alpha subunit
OG_01745	LA76x_1963	pstS; phosphate ABC transporter, phosphate-binding protein PstS
OG_01746	LA76x_4752	LA76x_4752; fecR family protein
OG_01747	LA76x_3802	LA76x_3802; recF/RecN/SMC N terminal domain protein
OG_01748	LA76x_4236	LA76x_4236; transcriptional regulatory , C terminal family protein
OG_01749	LA76x_3468	LA76x_3468; hypothetical protein
OG_01750	LA76x_4259	LA76x_4259; major intrinsic family protein
OG_01751	LA76x_4527	LA76x_4527; AMP nucleosidase, putative
OG_01752	LA76x_891	LA76x_891; major Facilitator Superfamily protein
OG_01753	LA76x_4289	groL; chaperonin GroL
OG_01754	LA76x_828	LA76x_828; pfkB carbohydrate kinase family protein
OG_01755	LA76x_3270	proS; proline--tRNA ligase
OG_01756	LA76x_4829	glnA; glutamine synthetase, type I
OG_01757	LA76x_2242	LA76x_2242; tetratricopeptide repeat family protein
OG_01758	LA76x_2238	LA76x_2238; conserved region in glutamate synthase family protein
OG_01759	LA76x_4633	coaBC; phosphopantothenoylcysteine decarboxylase / phosphopantc
OG_01760	LA76x_4873	nagA; N-acetylglucosamine-6-phosphate deacetylase
OG_01761	LA76x_1993	LA76x_1993; sodium/hydrogen exchanger family protein
OG_01762	LA76x_12	LA76x_12; biopolymer transport ExbD/TolR family protein
OG_01763	LA76x_1615	LA76x_1615; tonB-dependent siderophore receptor family protein
OG_01764	LA76x_4230	LA76x_4230; F5/8 type C domain protein
OG_01765	LA76x_2320	LA76x_2320; conserved hypothetical protein
OG_01766	LA76x_4513	LA76x_4513; response regulator
OG_01767	LA76x_3923	rplV; ribosomal protein L22
OG_01768	LA76x_5192	LA76x_5192; alpha/beta hydrolase fold family protein
OG_01769	LA76x_816	LA76x_816; NAD dependent epimerase/dehydratase family protein
OG_01770	LA76x_2189	LA76x_2189; putative glutathione S-transferase
OG_01771	LA76x_1246	LA76x_1246; alpha/beta hydrolase fold family protein
OG_01772	LA76x_1673	LA76x_1673; metallo-beta-lactamase superfamily protein
OG_01773	LA76x_3884	dctA; dctA dicarboxylate transporter
OG_01774	LA76x_4591	LA76x_4591; cytochrome oxidase assembly family protein
OG_01775	LA76x_4551	LA76x_4551; VIT family protein
OG_01776	LA76x_4816	LA76x_4816; periplasmic binding and sugar binding domain of LacI fa
OG_01777	LA76x_5158	LA76x_5158; flagellin N-methylase family protein
OG_01778	LA76x_5186	LA76x_5186; conserved hypothetical protein
OG_01779	LA76x_2523	LA76x_2523; homocysteine S-methyltransferase family protein
OG_01780	LA76x_4192	LA76x_4192; conserved hypothetical protein
OG_01781	LA76x_1041	LA76x_1041; HAMP domain protein
OG_01782	LA76x_1454	gcvH; glycine cleavage system H protein
OG_01783	LA76x_2105	LA76x_2105; glutathione S-transferase, C-terminal domain protein
OG_01784	LA76x_1893	LA76x_1893; conserved hypothetical protein
OG_01785	LA76x_3611	LA76x_3611; polyketide cyclase / dehydrase and lipid transport family
OG_01786	LA76x_4606	LA76x_4606; rhomboid family protein
OG_01787	LA76x_2298	LA76x_2298; cytochrome c554 domain protein
OG_01788	LA76x_4250	ilvB; acetolactate synthase, large subunit, biosynthetic type
OG_01789	LA76x_3126	phaZ7; PHB depolymerase PhaZ7
OG_01790	LA76x_3306	LA76x_3306; formylglycine-generating sulfatase enzyme family prote
OG_01791	LA76x_1209	LA76x_1209; conserved hypothetical protein
OG_01792	LA76x_4722	secB; protein-export chaperone SecB
OG_01793	LA76x_1184	LA76x_1184; mutator mutT family protein
OG_01794	LA76x_4049	LA76x_4049; repair family protein
OG_01795	LA76x_4297	LA76x_4297; conserved hypothetical protein
OG_01796	LA76x_4869	pgl; 6-phosphogluconolactonase
OG_01797	LA76x_4521	LA76x_4521; methyltransferase domain protein
OG_01798	LA76x_62	LA76x_62; hypothetical protein
OG_01799	LA76x_4239	thiC; thiamine biosynthesis protein ThiC

OG_01800	LA29479_2513	ABC-2 type transporter family protein
OG_01801	LA29479_4989	peptide deformylase
OG_01802	LA29479_3544	aminotransferase class-V family protein
OG_01803	LA29479_4296	hypothetical protein
OG_01804	LA29479_2948	metallopeptidase M24 family protein
OG_01805	LA29479_1583	conserved hypothetical protein
OG_01806	LA29479_1153	oxoglutarate dehydrogenase (succinyl-transferring), E1 component
OG_01807	LA29479_3978	putative membrane protein
OG_01808	LA29479_2047	putative transmembrane anchor protein
OG_01809	LA29479_3117	alpha/beta hydrolase fold family protein
OG_01810	LA29479_0186	aspartate-semialdehyde dehydrogenase
OG_01811	LA29479_5119	poly-beta-1,6-N-acetyl-D-glucosamine N-deacetylase
OG_01812	LA29479_3617	putative uncharacterized domain protein
OG_01813	LA29479_2737	virB7-like protein
OG_01814	LA29479_1648	putative glutaryl-CoA dehydrogenase
OG_01815	LA29479_3908	D-alanyl-D-alanine carboxypeptidase family protein
OG_01816	LA29479_1383	tonB family C-terminal domain protein
OG_01817	LA29479_0316	putative uncharacterized protein
OG_01818	LA29479_4817	cysteinyl-tRNA synthetase
OG_01819	LA29479_0734	pyrroline-5-carboxylate reductase
OG_01820	LA29479_1365	tetratricopeptide repeat family protein
OG_01821	LA29479_3674	sensory box protein
OG_01822	LA29479_3964	porphobilinogen deaminase
OG_01823	LA29479_0681	nucleotidyl transferase family protein
OG_01824	LA29479_4797	transketolase
OG_01825	LA29479_4517	1-deoxy-D-xylulose 5-phosphate reductoisomerase
OG_01826	LA29479_2483	hypothetical protein
OG_01827	LA29479_0336	chromate transporter, chromate ion transporter family protein
OG_01828	LA29479_1884	DEAD/DEAH box helicase family protein
OG_01829	LA29479_1650	putative uncharacterized protein XOO1406
OG_01830	LA29479_5223	HAMP domain protein
OG_01831	LA29479_1340	sigma-54 interaction domain protein
OG_01832	LA29479_1284	diguanylate cyclase domain protein
OG_01833	LA29479_2287	tfoX C-terminal domain protein
OG_01834	LA29479_3047	conserved hypothetical protein
OG_01835	LA29479_1627	tetraacyldisaccharide 4'-kinase
OG_01836	LA29479_2810	UTP-glucose-1-phosphate uridylyltransferase
OG_01837	LA29479_4160	pilO protein
OG_01838	LA29479_0147	HI0933-like family protein
OG_01839	LA29479_5154	acyltransferase family protein
OG_01840	LA29479_1552	ribonuclease T
OG_01841	LA29479_2033	cell division protein FtsW
OG_01842	LA29479_2470	RNA polymerase sigma factor, sigma-70 family protein
OG_01843	LA29479_1441	aspartate 1-decarboxylase
OG_01844	LA29479_0270	aldo/keto reductase family protein
OG_01845	LA29479_4239	pepSY-associated TM helix family protein
OG_01846	LA29479_4302	general secretion pathway protein M
OG_01847	LA29479_3601	protein-(glutamine-N5) methyltransferase, release factor-specific
OG_01848	LA29479_4080	acyl carrier protein
OG_01849	LA29479_1244	acyltransferase family protein
OG_01850	LA29479_4222	A/G-specific adenine glycosylase
OG_01851	LA29479_4304	general secretion pathway K family protein
OG_01852	LA29479_1421	hypothetical protein
OG_01853	LA29479_3253	conserved hypothetical protein
OG_01854	LA29479_1917	acetyltransferase family protein
OG_01855	LA29479_0167	putative membrane protein
OG_01856	LA29479_3240	sulfate ABC transporter, permease protein CysT
OG_01857	LA29479_2228	zinc-binding dehydrogenase family protein
OG_01858	LA29479_0828	adenylosuccinate synthetase
OG_01859	LA29479_4726	DNA repair protein RecO
OG_01860	LA29479_4056	hypothetical protein
OG_01861	LA29479_1773	carbamoyl-phosphate synthase, large subunit
OG_01862	LA29479_4647	4-hydroxy-3-methylbut-2-en-1-yl diphosphate synthase
OG_01863	LA29479_1072	conserved hypothetical protein
OG_01864	LA29479_1488	ribosomal protein S16
OG_01865	LA29479_0128	major Facilitator Superfamily protein
OG_01866	LA29479_1070	ribosomal protein S18
OG_01867	LA29479_0549	ubiquinol-cytochrome c reductase, iron-sulfur subunit
OG_01868	LA29479_0257	cell division inhibitor protein
OG_01869	LA29479_0966	putative uncharacterized protein
OG_01870	LA29479_0947	putative transmembrane protein
OG_01871	LA29479_4498	2-nitropropane dioxygenase family protein
OG_01872	LA29479_0560	ABC transporter family protein
OG_01873	LA29479_5044	putative uncharacterized protein
OG_01874	LA29479_4625	dihydroorotate oxidase
OG_01875	LA29479_0263	RES domain protein
OG_01876	LA29479_2457	response regulator
OG_01877	LA29479_3383	preprotein translocase subunit SecY
OG_01878	LA29479_0968	urocanate hydratase
OG_01879	LA29479_2325	ATP synthase F0, B subunit
OG_01880	LA29479_3073	msr3035 protein

OG_01800	LA76x_4468	LA76x_4468; ABC-2 type transporter family protein
OG_01801	LA76x_363	def; peptide deformylase
OG_01802	LA76x_2694	LA76x_2694; putative sphingosine-1-phosphate lyase
OG_01803	LA76x_1139	LA76x_1139; conserved hypothetical protein
OG_01804	LA76x_739	LA76x_739; metallopeptidase M24 family protein
OG_01805	LA76x_1925	LA76x_1925; mitochondrial biogenesis AIM24 family protein
OG_01806	LA76x_2491	sucA; oxoglutarate dehydrogenase (succinyl-transferring), E1 compor
OG_01807	LA76x_4567	LA76x_4567; sulfite exporter TauE/SafE family protein
OG_01808	LA76x_1105	LA76x_1105; conserved hypothetical protein
OG_01809	LA76x_929	LA76x_929; alpha/beta hydrolase fold family protein
OG_01810	LA76x_3003	asd; aspartate-semialdehyde dehydrogenase
OG_01811	LA76x_3250	pgaB; poly-beta-1,6-N-acetyl-D-glucosamine N-deacetylase PgaB
OG_01812	LA76x_4025	LA76x_4025; EF hand family protein
OG_01813	LA76x_2397	LA76x_2397; toxin co-regulated pilus biosynthesis Q family protein
OG_01814	LA76x_2187	fadE7; ACYL-CoA DEHYDROGENASE FADE7
OG_01815	LA76x_3608	LA76x_3608; D-alanyl-D-alanine carboxypeptidase family protein
OG_01816	LA76x_5195	LA76x_5195; tonB family C-terminal domain protein
OG_01817	LA76x_5128	LA76x_5128; conserved hypothetical protein
OG_01818	LA76x_3280	cysS; cysteine--tRNA ligase
OG_01819	LA76x_1542	proC; pyrroline-5-carboxylate reductase
OG_01820	LA76x_7	LA76x_7; tetratricopeptide repeat family protein
OG_01821	LA76x_3203	LA76x_3203; diguanylate cyclase domain protein
OG_01822	LA76x_795	hemC; porphobilinogen deaminase
OG_01823	LA76x_1592	LA76x_1592; mobA-like NTP transferase domain protein
OG_01824	LA76x_4143	tkt; transketolase
OG_01825	LA76x_1917	dxr; 1-deoxy-D-xylulose 5-phosphate reductoisomerase
OG_01826	LA76x_4436	LA76x_4436; hypothetical protein
OG_01827	LA76x_5108	chr; chromate transporter, chromate ion transporter family protein
OG_01828	LA76x_2747	LA76x_2747; DEAD/DEAH box helicase family protein
OG_01829	LA76x_2185	LA76x_2185; conserved hypothetical protein
OG_01830	LA76x_1406	LA76x_1406; HAMP domain protein
OG_01831	LA76x_31	LA76x_31; bacterial regulatory , Fis family protein
OG_01832	LA76x_87	LA76x_87; diguanylate cyclase domain protein
OG_01833	LA76x_4351	LA76x_4351; tfoX C-terminal domain protein
OG_01834	LA76x_863	LA76x_863; conserved hypothetical protein
OG_01835	LA76x_2208	lpxK; tetraacyldisaccharide 4'-kinase
OG_01836	LA76x_2324	galU; UTP-glucose-1-phosphate uridylyltransferase
OG_01837	LA76x_4154	pilO; pilO protein
OG_01838	LA76x_3041	LA76x_3041; flavo, HI0933 family protein
OG_01839	LA76x_641	LA76x_641; acyltransferase family protein
OG_01840	LA76x_1952	rnt; ribonuclease T
OG_01841	LA76x_1172	ftsW; cell division protein FtsW
OG_01842	LA76x_4424	LA76x_4424; RNA polymerase sigma factor, sigma-70 family protein
OG_01843	LA76x_2065	panD; aspartate 1-decarboxylase
OG_01844	LA76x_5175	LA76x_5175; aldo/keto reductase family protein
OG_01845	LA76x_1763	LA76x_1763; pepSY-associated TM helix family protein
OG_01846	LA76x_1133	LA76x_1133; type II secretion system (T2SS), M subtype b family pro
OG_01847	LA76x_4009	prmC; protein-(glutamine-N5) methyltransferase, release factor-speci
OG_01848	LA76x_251	LA76x_251; phosphopantetheine attachment site family protein
OG_01849	LA76x_127	plsB; glycerol-3-phosphate O-acyltransferase
OG_01850	LA76x_1781	mutY; A/G-specific adenine glycosylase
OG_01851	LA76x_1131	LA76x_1131; type II secretion system (T2SS), K family protein
OG_01852	LA76x_2083	LA76x_2083; hypothetical protein
OG_01853	LA76x_227	LA76x_227; conserved hypothetical protein
OG_01854	LA76x_1375	LA76x_1375; acetyltransferase domain protein
OG_01855	LA76x_3023	LA76x_3023; conserved hypothetical protein
OG_01856	LA76x_239	cysT; sulfate ABC transporter, permease protein CysT
OG_01857	LA76x_4295	LA76x_4295; alcohol dehydrogenase
OG_01858	LA76x_3670	purA; adenylosuccinate synthase
OG_01859	LA76x_3482	recO; DNA repair protein RecO
OG_01860	LA76x_272	LA76x_272; M61 glycyl aminopeptidase family protein
OG_01861	LA76x_2856	carB; carbamoyl-phosphate synthase, large subunit
OG_01862	LA76x_3457	ispG; 4-hydroxy-3-methylbut-2-en-1-yl diphosphate synthase
OG_01863	LA76x_2567	LA76x_2567; conserved hypothetical protein
OG_01864	LA76x_2014	rpsP; ribosomal protein S16
OG_01865	LA76x_3059	LA76x_3059; sugar (and other) transporter family protein
OG_01866	LA76x_2569	rpsR; ribosomal protein S18
OG_01867	LA76x_1717	petA; ubiquinol-cytochrome c reductase, iron-sulfur subunit
OG_01868	LA76x_2931	LA76x_2931; putative cell division inhibitor protein
OG_01869	LA76x_3809	LA76x_3809; conserved hypothetical protein
OG_01870	LA76x_3790	LA76x_3790; MAPEG family protein
OG_01871	LA76x_4661	LA76x_4661; nitronate monooxygenase family protein
OG_01872	LA76x_1705	LA76x_1705; ABC transporter family protein
OG_01873	LA76x_4696	LA76x_4696; AAA domain family protein
OG_01874	LA76x_3435	pyrD; dihydroorotate dehydrogenase
OG_01875	LA76x_2925	LA76x_2925; RES domain protein
OG_01876	LA76x_3421	LA76x_3421; response regulator
OG_01877	LA76x_3908	secY; preprotein translocase, SecY subunit
OG_01878	LA76x_3811	hutU; urocanate hydratase
OG_01879	LA76x_4388	atpF; ATP synthase F0, B subunit
OG_01880	LA76x_887	LA76x_887; putative msr3035 protein

OG_01881	LA29479_2241	ribosomal protein L11 methyltransferase
OG_01882	LA29479_1319	pyridine nucleotide-disulphide oxidoreductase family protein
OG_01883	LA29479_4728	ribonuclease III
OG_01884	LA29479_3426	acetylornithine and succinylornithine aminotransferases family protei
OG_01885	LA29479_3386	ribosomal protein S5
OG_01886	LA29479_4480	2-polyprenylphenol 6-hydroxylase
OG_01887	LA29479_1655	peptidyl-Lys metalloendopeptidase
OG_01888	LA29479_2510	tetratricopeptide repeat family protein
OG_01889	LA29479_2734	bacterial conjugation TrbI-like family protein
OG_01890	LA29479_2568	GDSL-like Lipase/Acylhydrolase family protein
OG_01891	LA29479_1215	dinB family protein
OG_01892	LA29479_5054	subtilase family protein
OG_01893	LA29479_2826	traB family protein
OG_01894	LA29479_1907	putative uncharacterized protein
OG_01895	LA29479_1224	twin arginine-targeting protein translocase TatC
OG_01896	LA29479_3835	superoxide dismutase [Cu-Zn] domain protein
OG_01897	LA29479_4711	alpha-lytic protease
OG_01898	LA29479_0291	lysM domain protein
OG_01899	LA29479_2981	bacterial regulatory helix-turn-helix , lysR family protein
OG_01900	LA29479_4359	bacterial regulatory s, tetR family protein
OG_01901	LA29479_0163	peptidyl-prolyl cis-trans isomerase domain protein
OG_01902	LA29479_3888	putative uncharacterized protein
OG_01903	LA29479_4528	hypothetical protein
OG_01904	LA29479_5069	hypothetical protein
OG_01905	LA29479_3781	hypothetical protein
OG_01906	LA29479_1845	putative uncharacterized protein
OG_01907	LA29479_0988	UDP-2,3-diacylglucosamine hydrolase
OG_01908	LA29479_0253	D-(-)-3-hydroxybutyrate oligomer hydrolase
OG_01909	LA29479_1043	protein
OG_01910	LA29479_0935	tonB-dependent Receptor Plug domain protein
OG_01911	LA29479_1472	marR family protein
OG_01912	LA29479_3340	zinc-binding alcohol dehydrogenase family protein
OG_01913	LA29479_1994	putative uncharacterized protein STY2850
OG_01914	LA29479_5046	coA-transferase III family protein
OG_01915	LA29479_5182	alpha-1,2-mannosidase, family protein
OG_01916	LA29479_3188	bacterial low temperature requirement A family protein
OG_01917	LA29479_0878	glutathione reductase
OG_01918	LA29479_3983	multifunctional CCA protein
OG_01919	LA29479_0577	inner membrane protein YjiY
OG_01920	LA29479_3334	catalytic LigB subunit of aromatic ring-opening dioxygenase family pro
OG_01921	LA29479_4570	TIM-barrel , nifR3 family protein
OG_01922	LA29479_2571	polysaccharide deacetylase family protein
OG_01923	LA29479_0112	uncharacterized enzyme family protein
OG_01924	LA29479_2352	rebB protein
OG_01925	LA29479_0467	tRNA uridine 5-carboxymethylaminomethyl modification enzyme GidA
OG_01926	LA29479_0585	dTDP-4-dehydrorhamnose reductase
OG_01927	LA29479_4281	6,7-dimethyl-8-ribityllumazine synthase
OG_01928	LA29479_2744	hypothetical protein
OG_01929	LA29479_1282	GTP cyclohydrolase I
OG_01930	LA29479_0944	bacterial regulatory , arsR family protein
OG_01931	LA29479_3043	ABC transporter family protein
OG_01932	LA29479_0942	fatty acid desaturase family protein
OG_01933	LA29479_4290	aspartyl/Asparaginyl beta-hydroxylase family protein
OG_01934	LA29479_3680	putative ferrous iron transport protein A
OG_01935	LA29479_3271	glycosyl transferase 2 family protein
OG_01936	LA29479_1200	binding--dependent transport system inner membrane component fa
OG_01938	LA29479_3415	ribosomal protein L11
OG_01939	LA29479_4000	putative uncharacterized protein
OG_01940	LA29479_4977	RNA polymerase sigma factor RpoD
OG_01941	LA29479_1564	conserved hypothetical protein
OG_01942	LA29479_0229	polyribonucleotide nucleotidyltransferase
OG_01943	LA29479_4288	transcriptional regulator NrdR
OG_01944	LA29479_3165	xaa-Pro dipeptidase, putative
OG_01945	LA29479_3196	putative uncharacterized protein
OG_01946	LA29479_2685	AIG2-like family protein
OG_01947	LA29479_1915	peptidase M28
OG_01948	LA29479_3265	methyltransferase GidB
OG_01949	LA29479_4877	lipopolysaccharide kinase family protein
OG_01950	LA29479_1538	hypothetical protein
OG_01951	LA29479_2326	ATP synthase F0, C subunit
OG_01952	LA29479_1302	putative uncharacterized protein
OG_01953	LA29479_2421	nucleotide sugar dehydrogenase family protein
OG_01954	LA29479_3198	putative uncharacterized protein
OG_01955	LA29479_3331	hypothetical protein
OG_01956	LA29479_1493	marR family protein
OG_01957	LA29479_3131	putative uncharacterized protein
OG_01958	LA29479_1101	conserved hypothetical protein
OG_01959	LA29479_3162	leucyl-tRNA synthetase
OG_01960	LA29479_5073	type III secretion , HrpO family protein
OG_01961	LA29479_3887	ribosomal RNA large subunit methyltransferase L
OG_01962	LA29479_3411	ribosomal protein L7/L12

OG_01881	LA76x_4308	prmA; ribosomal protein L11 methyltransferase
OG_01882	LA76x_53	LA76x_53; FAD-NAD(P)-binding family protein
OG_01883	LA76x_3484	rnc; ribonuclease III
OG_01884	LA76x_4212	LA76x_4212; astC
OG_01885	LA76x_3911	rpsE; ribosomal protein S5
OG_01886	LA76x_4679	ubiB; 2-polyprenylphenol 6-hydroxylase
OG_01887	LA76x_2180	MEP; peptidyl-Lys metalloendopeptidase
OG_01888	LA76x_4465	LA76x_4465; TPR repeat family protein
OG_01889	LA76x_2400	LA76x_2400; bacterial conjugation TrbI-like family protein
OG_01890	LA76x_4520	LA76x_4520; GDSL-like Lipase/Acylhydrolase family protein
OG_01891	LA76x_152	LA76x_152; dinB family protein
OG_01892	LA76x_4821	LA76x_4821; subtilase family protein
OG_01893	LA76x_2307	LA76x_2307; traB family protein
OG_01894	LA76x_1387	LA76x_1387; conserved hypothetical protein
OG_01895	LA76x_143	tatC; twin arginine-targeting protein translocase TatC
OG_01896	LA76x_4735	SOD1; superoxide dismutase [Cu-Zn] domain protein
OG_01897	LA76x_3467	alpha-LP; alpha-lytic protease
OG_01898	LA76x_5153	LA76x_5153; lysM domain protein
OG_01899	LA76x_2666	LA76x_2666; bacterial regulatory helix-turn-helix , lysR family protein
OG_01900	LA76x_1478	LA76x_1478; bacterial regulatory, tetR family protein
OG_01901	LA76x_3027	ytfC; peptidyl-prolyl cis-trans isomerase
OG_01902	LA76x_3589	LA76x_3589; conserved hypothetical protein
OG_01903	LA76x_1906	LA76x_1906; conserved hypothetical protein
OG_01904	LA76x_4809	LA76x_4809; hypothetical protein
OG_01905	LA76x_4782	LA76x_4782; hypothetical protein
OG_01906	LA76x_2785	LA76x_2785; conserved hypothetical protein
OG_01907	LA76x_3831	lpxH; UDP-2,3-diacylglucosamine hydrolase
OG_01908	LA76x_2936	LA76x_2936; 3HB-oligomer hydrolase family protein
OG_01909	LA76x_2599	LA76x_2599; putative predicted protein
OG_01910	LA76x_3776	LA76x_3776; tonB dependent receptor family protein
OG_01911	LA76x_2031	LA76x_2031; acetyltransferase family protein
OG_01912	LA76x_3865	LA76x_3865; zinc-binding alcohol dehydrogenase family protein
OG_01913	LA76x_1208	LA76x_1208; conserved hypothetical protein
OG_01914	LA76x_4694	LA76x_4694; coA-transferase III family protein
OG_01915	LA76x_382	LA76x_382; alpha-1,2-mannosidase family protein
OG_01916	LA76x_1835	LA76x_1835; bacterial low temperature requirement A family protein
OG_01917	LA76x_3717	LA76x_3717; glutathione amide reductase
OG_01918	LA76x_4572	cca; tRNA nucleotidyltransferase
OG_01919	LA76x_1688	LA76x_1688; carbon starvation CstA family protein
OG_01920	LA76x_3859	LA76x_3859; catalytic LigB subunit of aromatic ring-opening dioxyger
OG_01921	LA76x_1430	LA76x_1430; TIM-barrel , nifR3 family protein
OG_01922	LA76x_4522	LA76x_4522; polysaccharide deacetylase family protein
OG_01923	LA76x_3074	LA76x_3074; carboxylate-amine ligase, YbdK family protein
OG_01924	LA76x_4416	LA76x_4416; killing trait family protein
OG_01925	LA76x_4891	gidA; tRNA uridine 5-carboxymethylaminomethyl modification enzyme
OG_01926	LA76x_1681	rfbD; dTDP-4-dehydrorhamnose reductase
OG_01927	LA76x_1154	ribH; 6,7-dimethyl-8-ribityllumazine synthase
OG_01928	LA76x_2390	LA76x_2390; hypothetical protein
OG_01929	LA76x_89	folE; GTP cyclohydrolase I
OG_01930	LA76x_3787	LA76x_3787; bacterial regulatory , arsR family protein
OG_01931	LA76x_859	LA76x_859; ABC transporter family protein
OG_01932	LA76x_3785	LA76x_3785; putative fatty acid desaturase protein
OG_01933	LA76x_1145	LA76x_1145; aspartyl/Asparaginyl beta-hydroxylase family protein
OG_01934	LA76x_3196	LA76x_3196; feoA domain protein
OG_01935	LA76x_209	LA76x_209; glycosyl transferase 2 family protein
OG_01936	LA76x_2444	LA76x_2444; binding-protein-dependent transport system inner mem
OG_01938	LA76x_3941	rplK; ribosomal protein L11
OG_01939	LA76x_4587	LA76x_4587; conserved hypothetical protein
OG_01940	LA76x_375	rpoD; RNA polymerase sigma factor RpoD
OG_01941	LA76x_1943	LA76x_1943; conserved hypothetical protein
OG_01942	LA76x_2961	pnp; polyribonucleotide nucleotidyltransferase
OG_01943	LA76x_1147	nrdR; transcriptional regulator NrdR
OG_01944	LA76x_1857	LA76x_1857; xaa-Pro dipeptidase, putative
OG_01945	LA76x_1828	LA76x_1828; conserved hypothetical protein
OG_01946	LA76x_612	LA76x_612; AIG2-like family protein
OG_01947	LA76x_1378	LA76x_1378; peptidase M28
OG_01948	LA76x_215	gidB; 16S rRNA (guanine(527)-N(7))-methyltransferase GidB
OG_01949	LA76x_3535	LA76x_3535; lipopolysaccharide kinase family protein
OG_01950	LA76x_1966	LA76x_1966; hypothetical protein
OG_01951	LA76x_4389	atpE; ATP synthase F0, C subunit
OG_01952	LA76x_71	LA76x_71; STAS domain protein
OG_01953	LA76x_3390	LA76x_3390; nucleotide sugar dehydrogenase family protein
OG_01954	LA76x_1826	LA76x_1826; conserved hypothetical protein
OG_01955	LA76x_157	LA76x_157; CHAD domain protein
OG_01956	LA76x_2009	LA76x_2009; marR family protein
OG_01957	LA76x_944	LA76x_944; cysteine-rich CPXCG family protein
OG_01958	LA76x_2540	LA76x_2540; conserved hypothetical protein
OG_01959	LA76x_1859	leuS; leucine--tRNA ligase
OG_01960	LA76x_4805	LA76x_4805; type III secretion , HrpO family protein
OG_01961	LA76x_3588	rlmL; 23S rRNA m2G2445 methyltransferase
OG_01962	LA76x_3937	rplL; ribosomal protein L7/L12

OG_01963	LA29479_0701	UDP-N-acetylglucosamine 1-carboxyvinyltransferase
OG_01964	LA29479_0759	yceI-like domain protein
OG_01965	LA29479_1247	spore Coat Protein U domain protein
OG_01966	LA29479_4680	ADP compounds hydrolase nudE
OG_01967	LA29479_0662	pepSY-associated TM helix family protein
OG_01968	LA29479_2553	pyruvate dehydrogenase (acetyl-transferring), homodimeric type
OG_01969	LA29479_5024	bacterial regulatory s, lacI family protein
OG_01970	LA29479_2792	moeZ/MoeB domain protein
OG_01971	LA29479_3662	methylthioribulose-1-phosphate dehydratase
OG_01972	LA29479_4129	response regulator
OG_01973	LA29479_4875	molybdenum cofactor biosynthesis protein A
OG_01974	LA29479_4784	glyceraldehyde-3-phosphate dehydrogenase, type I
OG_01975	LA29479_4950	organic solvent tolerance family protein
OG_01976	LA29479_3159	rare lipoB family protein
OG_01977	LA29479_4453	orotate phosphoribosyltransferase
OG_01978	LA29479_2907	putative uncharacterized protein
OG_01979	LA29479_3961	phospholipase/Carboxylesterase family protein
OG_01980	LA29479_2153	UPF0337 protein yjbJ
OG_01981	LA29479_0775	putative uncharacterized protein
OG_01982	LA29479_2039	S-adenosyl-methyltransferase MraW
OG_01983	LA29479_0258	lexA repressor
OG_01984	LA29479_2330	dihydrolipoyl dehydrogenase
OG_01985	LA29479_3304	hypothetical protein
OG_01986	LA29479_4897	3-oxoacyl-[acyl-carrier-protein] synthase 2
OG_01987	LA29479_3200	ATPase associated with various cellular activities family protein
OG_01988	LA29479_0211	NADH dehydrogenase (quinone), G subunit
OG_01989	LA29479_1498	enoyl-CoA hydratase/isomerase family protein
OG_01990	LA29479_0254	CDP-diacylglycerol--serine O-phosphatidyltransferase
OG_01991	LA29479_0362	ahpC/TSA family protein
OG_01992	LA29479_1208	putative uncharacterized protein
OG_01993	LA29479_3447	fatty acid hydroxylase superfamily protein
OG_01994	LA29479_0564	short chain dehydrogenase family protein
OG_01995	LA29479_3930	putative uncharacterized protein
OG_01996	LA29479_0725	conserved hypothetical protein
OG_01997	LA29479_3502	DNA-N1-methyladenine dioxygenase
OG_01998	LA29479_4264	prepilin-type N-terminal cleavage/methylation domain protein
OG_01999	LA29479_5222	response regulator
OG_02000	LA29479_2515	activator of Hsp90 ATPase homolog 1-like family protein
OG_02001	LA29479_4443	NAD binding domain of 6-phosphogluconate dehydrogenase family p
OG_02002	LA29479_2001	succinyl-CoA ligase [ADP-forming] subunit beta
OG_02003	LA29479_1161	response regulator
OG_02004	LA29479_1657	cold shock protein CapB
OG_02005	LA29479_1279	major Facilitator Superfamily protein
OG_02006	LA29479_3856	ppx/GppA phosphatase family protein
OG_02007	LA29479_2201	hydrolase, haloacid delahogenase-like family
OG_02008	LA29479_3141	DNA-binding protein HU
OG_02009	LA29479_3416	transcription termination/antitermination factor NusG
OG_02010	LA29479_1220	glycyl-tRNA synthetase, alpha subunit
OG_02011	LA29479_1226	twin arginine-targeting translocase, TatA/E family protein
OG_02012	LA29479_1389	inner membrane transport permease yadH
OG_02013	LA29479_3320	UPF0126 inner membrane protein yicG
OG_02014	LA29479_3318	sugar (and other) transporter family protein
OG_02015	LA29479_3404	ribosomal protein S10
OG_02016	LA29479_3953	putative uncharacterized protein
OG_02017	LA29479_0919	conserved hypothetical protein
OG_02018	LA29479_3546	outer-membrane lipololB domain protein
OG_02019	LA29479_2558	malic enzyme, NAD binding domain protein
OG_02020	LA29479_4624	putative membrane protein
OG_02021	LA29479_1359	pyridoxal phosphate biosynthesis PdxJ family protein
OG_02022	LA29479_3199	ATPase associated with various cellular activities family protein
OG_02023	LA29479_1355	conserved hypothetical protein
OG_02024	LA29479_1526	putative thioesterase domain protein
OG_02025	LA29479_2764	calU1
OG_02026	LA29479_4093	conserved hypothetical protein
OG_02027	LA29479_3362	malate dehydrogenase
OG_02028	LA29479_1882	cu(I)-responsive transcriptional regulator
OG_02029	LA29479_1250	spore Coat Protein U domain protein
OG_02030	LA29479_2328	putative uncharacterized protein
OG_02031	LA29479_0856	5'/3'-nucleotidase SurE
OG_02032	LA29479_0804	response regulator
OG_02033	LA29479_5211	von Willebrand factor type A domain protein
OG_02034	LA29479_2339	NADP-dependent alcohol dehydrogenase C 1
OG_02035	LA29479_2815	integration host factor, beta subunit
OG_02036	LA29479_0992	colicin V production family protein
OG_02037	LA29479_0921	formate dehydrogenase family accessory protein FdhD
OG_02038	LA29479_2053	CAAX amino terminal protease family protein
OG_02039	LA29479_4151	response regulator
OG_02040	LA29479_0629	rickettsia 17 kDa surface antigen family protein
OG_02041	LA29479_0894	insulinase family protein
OG_02042	LA29479_2286	GAF domain protein
OG_02043	LA29479_2248	hypothetical protein

OG_01963	LA76x_1574	murA; UDP-N-acetylglucosamine 1-carboxyvinyltransferase
OG_01964	LA76x_1517	LA76x_1517; yceI-like domain protein
OG_01965	LA76x_124	LA76x_124; spore Coat Protein U domain protein
OG_01966	LA76x_4065	LA76x_4065; NUDIX domain protein
OG_01967	LA76x_1613	LA76x_1613; pepSY-associated TM helix family protein
OG_01968	LA76x_4505	aceE; pyruvate dehydrogenase (acetyl-transferring), homodimeric typ
OG_01969	LA76x_4553	LA76x_4553; periplasmic binding and sugar binding domain of LacI fa
OG_01970	LA76x_2341	LA76x_2341; moeZ/MoeB domain protein
OG_01971	LA76x_3212	mtnB; methylthioribulose-1-phosphate dehydratase
OG_01972	LA76x_325	LA76x_325; response regulator
OG_01973	LA76x_3533	moaA; molybdenum cofactor biosynthesis protein A
OG_01974	LA76x_4130	gap; glyceraldehyde-3-phosphate dehydrogenase, type I
OG_01975	LA76x_4038	LA76x_4038; ostA-like family protein
OG_01976	LA76x_1861	LA76x_1861; lipopolysaccharide-assembly family protein
OG_01977	LA76x_4629	pyrE; orotate phosphoribosyltransferase
OG_01978	LA76x_709	LA76x_709; conserved hypothetical protein
OG_01979	LA76x_792	LA76x_792; dienelactone hydrolase family protein
OG_01980	LA76x_999	LA76x_999; putative stress response protein
OG_01981	LA76x_3619	LA76x_3619; conserved hypothetical protein
OG_01982	LA76x_1166	mraW; S-adenosyl-methyltransferase MraW
OG_01983	LA76x_2930	lexA; repressor LexA
OG_01984	LA76x_4394	lpdA; dihydrolipoyl dehydrogenase
OG_01985	LA76x_180	LA76x_180; hypothetical protein
OG_01986	LA76x_3498	fabF; beta-ketoacyl-acyl-carrier-protein synthase II
OG_01987	LA76x_1824	LA76x_1824; ATPase associated with various cellular activities family
OG_01988	LA76x_2980	nuoG; NADH dehydrogenase (quinone), G subunit
OG_01989	LA76x_2004	LA76x_2004; enoyl-CoA hydratase/isomerase family protein
OG_01990	LA76x_2934	pssA; CDP-diacylglycerol-serine O-phosphatidyltransferase
OG_01991	LA76x_5082	LA76x_5082; ahpC/TSA family protein
OG_01992	LA76x_2435	LA76x_2435; conserved hypothetical protein
OG_01993	LA76x_4231	LA76x_4231; fatty acid hydroxylase superfamily protein
OG_01994	LA76x_1699	LA76x_1699; saccharopine dehydrogenase family protein
OG_01995	LA76x_761	LA76x_761; conserved hypothetical protein
OG_01996	LA76x_1550	LA76x_1550; conserved hypothetical protein
OG_01997	LA76x_2734	LA76x_2734; 2OG-Fe(II) oxygenase superfamily protein
OG_01998	LA76x_1738	LA76x_1738; prepilin-type N-terminal cleavage/methylation domain p
OG_01999	LA76x_1405	LA76x_1405; transcriptional regulatory , C terminal family protein
OG_02000	LA76x_4470	LA76x_4470; conserved hypothetical protein
OG_02001	LA76x_4639	LA76x_4639; NAD binding domain of 6-phosphogluconate dehydroge
OG_02002	LA76x_1201	sucC; succinyl-CoA synthetase, &beta subunit
OG_02003	LA76x_2484	LA76x_2484; response regulator
OG_02004	LA76x_2178	LA76x_2178; 'Cold-shock' DNA-binding domain protein
OG_02005	LA76x_93	LA76x_93; sugar (and other) transporter family protein
OG_02006	LA76x_3557	LA76x_3557; ppx/GppA phosphatase family protein
OG_02007	LA76x_955	LA76x_955; hydrolase, haloacid delahogenase-like family
OG_02008	LA76x_1877	LA76x_1877; bacterial DNA-binding family protein
OG_02009	LA76x_3942	nusG; transcription termination/antitermination factor NusG
OG_02010	LA76x_147	glyQ; glycine--tRNA ligase, alpha subunit
OG_02011	LA76x_141	tatA; twin arginine-targeting translocase, TatA/E family protein
OG_02012	LA76x_5189	LA76x_5189; ABC-2 type transporter family protein
OG_02013	LA76x_166	LA76x_166; conserved hypothetical protein
OG_02014	LA76x_169	LA76x_169; sugar (and other) transporter family protein
OG_02015	LA76x_3929	rpsJ; ribosomal protein S10
OG_02016	LA76x_784	LA76x_784; conserved hypothetical protein
OG_02017	LA76x_3759	LA76x_3759; conserved hypothetical protein
OG_02018	LA76x_3956	lolB; outer membrane lipoprotein LolB
OG_02019	LA76x_4510	LA76x_4510; malic enzyme, NAD binding domain protein
OG_02020	LA76x_3434	LA76x_3434; conserved hypothetical protein
OG_02021	LA76x_13	pdxJ; pyridoxine 5'-phosphate synthase
OG_02022	LA76x_1825	LA76x_1825; AAA domain family protein
OG_02023	LA76x_16	LA76x_16; conserved hypothetical protein
OG_02024	LA76x_1977	LA76x_1977; conserved hypothetical protein
OG_02025	LA76x_2370	LA76x_2370; conserved hypothetical protein
OG_02026	LA76x_360	LA76x_360; conserved hypothetical protein
OG_02027	LA76x_3888	mdh; malate dehydrogenase
OG_02028	LA76x_2749	cueR; cu(I)-responsive transcriptional regulator
OG_02029	LA76x_121	LA76x_121; spore Coat Protein U domain protein
OG_02030	LA76x_4392	LA76x_4392; conserved hypothetical protein
OG_02031	LA76x_3695	surE; 5'/3'-nucleotidase SurE
OG_02032	LA76x_3648	LA76x_3648; response regulator
OG_02033	LA76x_4147	LA76x_4147; conserved hypothetical protein
OG_02034	LA76x_4403	adhC2; NADP-dependent alcohol dehydrogenase C 2
OG_02035	LA76x_2319	ihfB; integration host factor, beta subunit
OG_02036	LA76x_3835	LA76x_3835; colicin V production family protein
OG_02037	LA76x_3761	LA76x_3761; formate dehydrogenase family accessory protein FdhD
OG_02038	LA76x_1099	LA76x_1099; CAAX protease self-immunity family protein
OG_02039	LA76x_303	LA76x_303; response regulator
OG_02040	LA76x_1646	LA76x_1646; putative secreted protein
OG_02041	LA76x_3733	LA76x_3733; peptidase M16 inactive domain protein
OG_02042	LA76x_4350	LA76x_4350; GAF domain protein
OG_02043	LA76x_4315	LA76x_4315; hypothetical protein

OG_02044	LA29479_2277	putative transmembrane protein	OG_02044	LA76x_4342	LA76x_4342; conserved hypothetical protein
OG_02045	LA29479_3867	response regulator	OG_02045	LA76x_3567	LA76x_3567; transcriptional regulatory , C terminal family protein
OG_02046	LA29479_5226	DEAD/DEAH box helicase family protein	OG_02046	LA76x_299	rhlB; EG10844 domain protein
OG_02047	LA29479_1839	aconitate hydratase 1	OG_02047	LA76x_2791	acnA; aconitate hydratase 1
OG_02048	LA29479_4241	gumN family protein	OG_02048	LA76x_1761	LA76x_1761; traB family protein
OG_02049	LA29479_4207	ABC transporter family protein	OG_02049	LA76x_4198	LA76x_4198; ABC transporter family protein
OG_02050	LA29479_0525	phosphatidylethanolamine-binding family protein	OG_02050	LA76x_4836	LA76x_4836; phosphatidylethanolamine-binding family protein
OG_02051	LA29479_3361	response regulator	OG_02051	LA76x_3887	LA76x_3887; bacterial regulatory, luxR family protein
OG_02052	LA29479_2227	extracellular ribonuclease domain protein	OG_02052	LA76x_4294	bsn; extracellular ribonuclease domain protein
OG_02053	LA29479_5026	alpha amylase, catalytic domain protein	OG_02053	LA76x_4554	LA76x_4554; alpha amylase, catalytic domain protein
OG_02054	LA29479_5064	response regulator	OG_02054	LA76x_4813	LA76x_4813; bacterial regulatory , Fis family protein
OG_02055	LA29479_3242	sulfate ABC transporter, ATP-binding family protein	OG_02055	LA76x_237	cysA; sulfate ABC transporter, ATP-binding family protein
OG_02056	LA29479_2189	sigma-54 interaction domain protein	OG_02056	LA76x_967	LA76x_967; rtcR transcriptional regulator RtcR transcriptional activato
OG_02057	LA29479_1555	chitinase class I family protein	OG_02057	LA76x_1951	LA76x_1951; chitinase class I family protein
OG_02058	LA29479_4307	putative general secretion pathway protein h	OG_02058	LA76x_1128	LA76x_1128; type II transport GspH family protein
OG_02059	LA29479_1278	putative lipoprotein	OG_02059	LA76x_94	LA76x_94; hypothetical protein
OG_02060	LA29479_0222	translation initiation factor IF-2	OG_02060	LA76x_2968	infB; translation initiation factor IF-2
OG_02061	LA29479_1298	uncharacterized ABC transporter ATP-binding protein HI_1087	OG_02061	LA76x_75	LA76x_75; ABC transporter family protein
OG_02062	LA29479_4521	beta-hydroxyacyl-(acyl-carrier-protein) dehydratase FabZ	OG_02062	LA76x_1912	fabZ; beta-hydroxyacyl-(acyl-carrier-protein) dehydratase FabZ
OG_02063	LA29479_3967	nrfC protein	OG_02063	LA76x_799	nrfC; nrfC protein
OG_02064	LA29479_4297	tat (twin-arginine translocation) pathway signal sequence domain prot	OG_02064	LA76x_1138	LA76x_1138; metallo-beta-lactamase superfamily protein
OG_02065	LA29479_5129	tonB family C-terminal domain protein	OG_02065	LA76x_2262	LA76x_2262; tonB family C-terminal domain protein
OG_02066	LA29479_0507	AIG2-like family protein	OG_02066	LA76x_4852	LA76x_4852; AIG2-like family protein
OG_02067	LA29479_3145	ATP-dependent Clp protease, proteolytic subunit ClpP	OG_02067	LA76x_1874	clpP; ATP-dependent Clp endopeptidase, proteolytic subunit ClpP
OG_02068	LA29479_4390	putative uncharacterized protein	OG_02068	LA76x_1447	LA76x_1447; hypothetical protein
OG_02069	LA29479_4077	putative xanthomonadin biosynthesis acyltransferase protein	OG_02069	LA76x_254	LA76x_254; bacterial lipid A biosynthesis acyltransferase family prote
OG_02070	LA29479_1431	gumN family protein	OG_02070	LA76x_2073	LA76x_2073; traB family protein
OG_02071	LA29479_4573	calcineurin-like phosphoesterase family protein	OG_02071	LA76x_1427	LA76x_1427; calcineurin-like phosphoesterase family protein
OG_02072	LA29479_1633	liporeleasing system, transmembrane , LolC/E family protein	OG_02072	LA76x_2202	LA76x_2202; liporeleasing system, transmembrane , LolC/E family pr
OG_02073	LA29479_2065	aldose 1-epimerase family protein	OG_02073	LA76x_1087	LA76x_1087; aldose 1-epimerase family protein
OG_02074	LA29479_2299	hypothetical protein	OG_02074	LA76x_4362	LA76x_4362; hypothetical protein
OG_02075	LA29479_0627	phenazine biosynthesis PhzF family protein	OG_02075	LA76x_1648	LA76x_1648; phenazine biosynthesis , PhzF family protein
OG_02076	LA29479_2952	helix-turn-helix domain, rpiR family protein	OG_02076	LA76x_743	LA76x_743; helix-turn-helix domain, rpiR family protein
OG_02077	LA29479_1584	UMP kinase	OG_02077	LA76x_1924	pyrH; UMP kinase
OG_02078	LA29479_4176	endoribonuclease L-PSP, putative	OG_02078	LA76x_4170	LA76x_4170; endoribonuclease L-PSP, putative
OG_02079	LA29479_5212	tetratricopeptide repeat family protein	OG_02079	LA76x_4146	LA76x_4146; von Willebrand factor type A domain protein
OG_02080	LA29479_2758	putative uncharacterized protein	OG_02080	LA76x_2376	LA76x_2376; conserved hypothetical protein
OG_02081	LA29479_1599	diguanylate cyclase domain protein	OG_02081	LA76x_2235	LA76x_2235; diguanylate cyclase domain protein
OG_02082	LA29479_4487	acyltransferase family protein	OG_02082	LA76x_4672	LA76x_4672; acyltransferase family protein
OG_02083	LA29479_0278	hypothetical protein	OG_02083	LA76x_5166	LA76x_5166; hypothetical protein
OG_02084	LA29479_0515	putative uncharacterized protein XALc	OG_02084	LA76x_4844	LA76x_4844; conserved hypothetical protein
OG_02085	LA29479_0306	hypothetical protein	OG_02085	LA76x_5137	LA76x_5137; hypothetical protein
OG_02086	LA29479_4790	molybdate ABC transporter, permease protein	OG_02086	LA76x_4136	modB; molybdate ABC transporter, permease protein
OG_02087	LA29479_5066	conserved hypothetical protein	OG_02087	LA76x_4812	LA76x_4812; conserved hypothetical protein
OG_02088	LA29479_3497	putative phospholipid-binding domain protein	OG_02088	LA76x_2739	LA76x_2739; BON domain protein
OG_02089	LA29479_3980	transglycosylase SLT domain protein	OG_02089	LA76x_4569	LA76x_4569; transglycosylase SLT domain protein
OG_02090	LA29479_4305	prepilin-type N-terminal cleavage/methylation domain protein	OG_02090	LA76x_1130	LA76x_1130; prepilin-type N-terminal cleavage/methylation domain p
OG_02091	LA29479_2714	hypothetical protein	OG_02091	LA76x_2420	LA76x_2420; hypothetical protein
OG_02092	LA29479_2107	anthranilate synthase component II	OG_02092	LA76x_1045	trpG; anthranilate synthase component II
OG_02093	LA29479_2583	amidohydrolase family protein	OG_02093	LA76x_4534	LA76x_4534; amidohydrolase family protein
OG_02094	LA29479_4739	ribonuclease, Rne/Rng family domain protein	OG_02094	LA76x_2292	LA76x_2292; ribonuclease, Rne/Rng family domain protein
OG_02095	LA29479_4016	putative integron gene cassette protein	OG_02095	LA76x_4603	LA76x_4603; conserved hypothetical protein
OG_02096	LA29479_3901	hypothetical protein	OG_02096	LA76x_3601	LA76x_3601; hypothetical protein
OG_02097	LA29479_2462	DNA topoisomerase IV, A subunit	OG_02097	LA76x_3426	parC; DNA topoisomerase IV, A subunit
OG_02098	LA29479_4764	bacterial regulatory helix-turn-helix , lysR family protein	OG_02098	LA76x_2266	LA76x_2266; bacterial regulatory helix-turn-helix , lysR family protein
OG_02099	LA29479_2670	hypothetical protein	OG_02099	LA76x_594	LA76x_594; HNH/ENDO VII supernuclease with conserved GHE resi
OG_02100	LA29479_4789	molybdate ABC transporter, periplasmic molybdate-binding protein	OG_02100	LA76x_4135	modA; molybdate ABC transporter, periplasmic molybdate-binding pro
OG_02101	LA29479_3248	uncharacterised family protein	OG_02101	LA76x_232	LA76x_232; flagellin N-methylase family protein
OG_02102	LA29479_3222	preprotein translocase, YajC subunit	OG_02102	LA76x_1804	yajC; preprotein translocase, YajC subunit
OG_02103	LA29479_3315	acetate--CoA ligase	OG_02103	LA76x_171	acs; acetate--CoA ligase
OG_02104	LA29479_3031	conserved hypothetical protein	OG_02104	LA76x_847	LA76x_847; conserved hypothetical protein
OG_02105	LA29479_4524	lipid-A-disaccharide synthase	OG_02105	LA76x_1909	lpxB; lipid-A-disaccharide synthase
OG_02106	LA29479_4690	response regulator	OG_02106	LA76x_4075	LA76x_4075; response regulator
OG_02108	LA29479_1539	endonuclease III	OG_02108	LA76x_1965	nth; endonuclease III
OG_02109	LA29479_4168	ribosomal protein L31	OG_02109	LA76x_4162	rpmE; ribosomal protein L31
OG_02110	LA29479_1521	ompA family protein	OG_02110	LA76x_1982	LA76x_1982; ompA family protein
OG_02111	LA29479_1872	conserved hypothetical protein	OG_02111	LA76x_2758	LA76x_2758; conserved hypothetical family protein
OG_02112	LA29479_0199	putative uncharacterized protein	OG_02112	LA76x_2991	LA76x_2991; trypsin family protein
OG_02113	LA29479_3553	molybdenum cofactor biosynthesis protein B	OG_02113	LA76x_3963	moaB; molybdenum cofactor biosynthesis protein B
OG_02114	LA29479_0646	tonB-dependent Receptor Plug domain protein	OG_02114	LA76x_1629	LA76x_1629; tonB dependent receptor family protein
OG_02115	LA29479_3558	helix-turn-helix family protein	OG_02115	LA76x_3966	LA76x_3966; helix-turn-helix family protein
OG_02116	LA29479_4608	ATP-dependent DNA helicase RecQ	OG_02116	LA76x_4102	recQ; ATP-dependent DNA helicase RecQ
OG_02117	LA29479_5043	SPFH domain / Band 7 family protein	OG_02117	LA76x_4697	LA76x_4697; conserved hypothetical protein
OG_02118	LA29479_0215	NADH-quinone oxidoreductase subunit 11	OG_02118	LA76x_2976	nuoK; NADH-quinone oxidoreductase subunit K
OG_02119	LA29479_1958	hypothetical protein	OG_02119	LA76x_1241	LA76x_1241; hypothetical protein
OG_02120	LA29479_3659	hypothetical protein	OG_02120	LA76x_3215	LA76x_3215; hypothetical protein
OG_02121	LA29479_4717	phosphoribosyl transferase domain protein	OG_02121	LA76x_3473	LA76x_3473; phosphoribosyl transferase domain protein
OG_02122	LA29479_2164	hypothetical protein	OG_02122	LA76x_989	LA76x_989; hypothetical protein
OG_02123	LA29479_0006	eukaryotic-type carbonic anhydrase family protein	OG_02123	LA76x_3178	LA76x_3178; eukaryotic-type carbonic anhydrase family protein
OG_02124	LA29479_2471	putative membrane protein	OG_02124	LA76x_4425	LA76x_4425; putative transmembrane protein
OG_02125	LA29479_4478	short chain dehydrogenase family protein	OG_02125	LA76x_4681	LA76x_4681; polysaccharide biosynthesis family protein

OG_02126	LA29479_0194	tryptophan synthase, alpha subunit	OG_02126	LA76x_2996	trpA; tryptophan synthase, alpha subunit
OG_02127	LA29479_1387	oxidoreductase FAD-binding domain protein	OG_02127	LA76x_5191	LA76x_5191; oxidoreductase NAD-binding domain protein
OG_02128	LA29479_1754	hypothetical protein	OG_02128	LA76x_2875	LA76x_2875; hypothetical protein
OG_02129	LA29479_2297	hypothetical protein	OG_02129	LA76x_4359	LA76x_4359; hypothetical protein
OG_02130	LA29479_1127	protein slyX homolog	OG_02130	LA76x_2517	LA76x_2517; conserved hypothetical protein
OG_02131	LA29479_1487	16S rRNA processing protein RimM	OG_02131	LA76x_2015	rimM; 16S rRNA processing protein RimM
OG_02132	LA29479_4476	alpha/beta hydrolase fold family protein	OG_02132	LA76x_4683	LA76x_4683; alpha/beta hydrolase fold family protein
OG_02133	LA29479_1972	bacterial regulatory helix-turn-helix s, AraC family protein	OG_02133	LA76x_1229	LA76x_1229; bacterial regulatory helix-turn-helix, AraC family protein
OG_02134	LA29479_4063	beta-lactamase family protein	OG_02134	LA76x_266	LA76x_266; beta-lactamase family protein
OG_02135	LA29479_3105	amidohydrolase family protein	OG_02135	LA76x_917	LA76x_917; amidohydrolase family protein
OG_02136	LA29479_1147	glyoxalase/Bleomycin resistance /Dioxygenase superfamily protein	OG_02136	LA76x_2497	LA76x_2497; glyoxalase/Bleomycin resistance /Dioxygenase superfa
OG_02137	LA29479_1544	phosphate ABC transporter, permease protein PstA	OG_02137	LA76x_1960	pstA; phosphate ABC transporter, permease protein PstA
OG_02138	LA29479_1574	succinyl-diaminopimelate desuccinylase	OG_02138	LA76x_1934	dapE; succinyl-diaminopimelate desuccinylase
OG_02139	LA29479_2028	cell division FtsQ family protein	OG_02139	LA76x_1177	LA76x_1177; cell division FtsQ family protein
OG_02140	LA29479_1939	tol-Pal system beta propeller repeat protein TolB	OG_02140	LA76x_1260	tolB; tol-Pal system beta propeller repeat protein TolB
OG_02141	LA29479_1952	conserved hypothetical protein	OG_02141	LA76x_1247	LA76x_1247; DNA-binding regulatory , YebC/PmpR family protein
OG_02142	LA29479_3826	arsenate reductase	OG_02142	LA76x_4744	arsC; arsenate reductase
OG_02143	LA29479_1415	conserved hypothetical protein	OG_02143	LA76x_2089	LA76x_2089; conserved hypothetical protein
OG_02144	LA29479_4161	fimbrial assembly family protein	OG_02144	LA76x_4155	LA76x_4155; fimbrial assembly family protein
OG_02145	LA29479_3050	putative uncharacterized protein	OG_02145	LA76x_866	LA76x_866; conserved hypothetical protein
OG_02146	LA29479_2565	conserved hypothetical protein	OG_02146	LA76x_4517	LA76x_4517; conserved hypothetical protein
OG_02147	LA29479_0588	dTDP-glucose 4,6-dehydratase	OG_02147	LA76x_1678	rfbB; dTDP-glucose 4,6-dehydratase
OG_02148	LA29479_5192	translation elongation factor P	OG_02148	LA76x_3296	efp; translation elongation factor P
OG_02149	LA29479_2750	homoserine O-acetyltransferase	OG_02149	LA76x_2384	metX; homoserine O-acetyltransferase
OG_02150	LA29479_4975	putative uncharacterized protein	OG_02150	LA76x_376	LA76x_376; snoaL-like domain protein
OG_02151	LA29479_0910	transcriptional regulator PadR-like family protein	OG_02151	LA76x_3750	LA76x_3750; transcriptional regulator PadR-like family protein
OG_02152	LA29479_1432	conserved hypothetical protein	OG_02152	LA76x_2072	LA76x_2072; conserved hypothetical protein
OG_02153	LA29479_3985	vanZ like family protein	OG_02153	LA76x_4574	LA76x_4574; vanZ like family protein
OG_02154	LA29479_5007	tonB dependent receptor family protein	OG_02154	LA76x_4711	LA76x_4711; tonB dependent receptor family protein
OG_02155	LA29479_2908	putative uncharacterized protein	OG_02155	LA76x_710	LA76x_710; conserved hypothetical protein
OG_02156	LA29479_1520	ribosomal protein L21	OG_02156	LA76x_1983	rplU; ribosomal protein L21
OG_02157	LA29479_3988	putative uncharacterized domain protein	OG_02157	LA76x_4576	LA76x_4576; hypothetical protein
OG_02158	LA29479_3029	hypothetical protein	OG_02158	LA76x_845	LA76x_845; GDSL-like Lipase/Acylhydrolase family protein
OG_02159	LA29479_3339	aldo/keto reductase family protein	OG_02159	LA76x_3864	LA76x_3864; aldo/keto reductase family protein
OG_02160	LA29479_5187	fliO-like	OG_02160	LA76x_4800	LA76x_4800; flagellar biosynthesis , FliO family protein
OG_02161	LA29479_2244	cytidylyltransferase family protein	OG_02161	LA76x_4311	LA76x_4311; cytidylyltransferase family protein
OG_02162	LA29479_2451	proline iminopeptidase	OG_02162	LA76x_3414	pip; prolyl aminopeptidase
OG_02163	LA29479_0547	cytochrome C1 family protein	OG_02163	LA76x_1719	LA76x_1719; cytochrome C1 family protein
OG_02164	LA29479_0667	conserved hypothetical protein	OG_02164	LA76x_1608	LA76x_1608; hutD family protein
OG_02165	LA29479_1295	exodeoxyribonuclease V, beta subunit	OG_02165	LA76x_78	recB; exodeoxyribonuclease V, beta subunit
OG_02166	LA29479_4217	secreted protein	OG_02166	LA76x_1786	LA76x_1786; conserved hypothetical protein
OG_02167	LA29479_1530	hypothetical protein	OG_02167	LA76x_1973	LA76x_1973; PBP superfamily domain protein
OG_02168	LA29479_3345	putative uncharacterized protein	OG_02168	LA76x_3870	LA76x_3870; conserved hypothetical protein
OG_02169	LA29479_4961	conserved hypothetical protein	OG_02169	LA76x_4048	LA76x_4048; repair family protein
OG_02170	LA29479_0848	RNA-binding protein YhbY	OG_02170	LA76x_3687	LA76x_3687; CRS1 / YhbY domain protein
OG_02171	LA29479_4278	thiamine-monophosphate kinase	OG_02171	LA76x_1157	thiL; thiamine-monophosphate kinase
OG_02172	LA29479_1592	hypothetical protein	OG_02172	LA76x_2243	LA76x_2243; hypothetical protein
OG_02173	LA29479_1306	hypothetical protein	OG_02173	LA76x_67	LA76x_67; hypothetical protein
OG_02174	LA29479_1910	citrate transporter family protein	OG_02174	LA76x_1383	LA76x_1383; citrate transporter family protein
OG_02175	LA29479_1175	uncharacterized protein ybaA	OG_02175	LA76x_2472	LA76x_2472; conserved hypothetical protein
OG_02176	LA29479_3772	putative uncharacterized protein	OG_02176	LA76x_4793	LA76x_4793; conserved hypothetical protein
OG_02177	LA29479_3312	response regulator	OG_02177	LA76x_173	LA76x_173; bacterial regulatory, luxR family protein
OG_02178	LA29479_4320	conserved hypothetical protein	OG_02178	LA76x_1116	LA76x_1116; putative permease YjgP/YjgQ family protein
OG_02179	LA29479_2468	RNA polymerase sigma factor, sigma-70 family protein	OG_02179	LA76x_4422	LA76x_4422; sigma-70, region 4 family protein
OG_02180	LA29479_2713	hypothetical protein	OG_02180	LA76x_2421	LA76x_2421; hypothetical protein
OG_02181	LA29479_3204	6-O-methylguanine DNA methyltransferase, DNA binding domain prot	OG_02181	LA76x_1820	LA76x_1820; 6-O-methylguanine DNA methyltransferase, DNA bindir
OG_02182	LA29479_2890	response regulator	OG_02182	LA76x_694	wspR; response regulator
OG_02183	LA29479_1911	rieske [2Fe-2S] domain protein	OG_02183	LA76x_1382	LA76x_1382; rieske [2Fe-2S] domain protein
OG_02184	LA29479_2739	putative uncharacterized protein	OG_02184	LA76x_2395	LA76x_2395; conserved hypothetical protein
OG_02185	LA29479_3036	asnC family protein	OG_02185	LA76x_852	LA76x_852; asnC family protein
OG_02186	LA29479_2239	putative exported protein	OG_02186	LA76x_4306	LA76x_4306; conserved hypothetical protein
OG_02187	LA29479_1079	short chain dehydrogenase family protein	OG_02187	LA76x_2559	LA76x_2559; short chain dehydrogenase family protein
OG_02188	LA29479_0469	putative uncharacterized protein VP0956	OG_02188	LA76x_4889	LA76x_4889; conserved hypothetical protein
OG_02189	LA29479_5216	segregation and condensation protein B	OG_02189	LA76x_2433	scpB; segregation and condensation protein B
OG_02190	LA29479_4346	glycosyl transferase 2 family protein	OG_02190	LA76x_1489	LA76x_1489; glycosyl transferase 2 family protein
OG_02191	LA29479_3167	putative membrane protein	OG_02191	LA76x_1855	LA76x_1855; conserved hypothetical protein
OG_02192	LA29479_4388	protein QmcA	OG_02192	LA76x_1449	LA76x_1449; SPFH domain / Band 7 family protein
OG_02193	LA29479_5006	putative membrane protein	OG_02193	LA76x_4712	LA76x_4712; ABC-2 transporter family protein
OG_02194	LA29479_1442	pantoate--beta-alanine ligase	OG_02194	LA76x_2064	panC; pantoate--beta-alanine ligase
OG_02195	LA29479_2129	NAD dependent epimerase/dehydratase family protein	OG_02195	LA76x_1023	LA76x_1023; NAD dependent epimerase/dehydratase family protein
OG_02196	LA29479_0680	phosphotransferase enzyme family protein	OG_02196	LA76x_1593	LA76x_1593; phosphotransferase enzyme family protein
OG_02197	LA29479_3917	transglutaminase-like superfamily protein	OG_02197	LA76x_747	LA76x_747; transglutaminase-like superfamily protein
OG_02198	LA29479_0727	putative membrane protein	OG_02198	LA76x_1548	LA76x_1548; conserved hypothetical protein
OG_02199	LA29479_3963	response regulator	OG_02199	LA76x_794	LA76x_794; response regulator
OG_02200	LA29479_1378	tRNA modification GTPase TrmE	OG_02200	LA76x_5200	trmE; tRNA modification GTPase TrmE
OG_02201	LA29479_2274	putative transmembrane protein	OG_02201	LA76x_4339	LA76x_4339; putative transmembrane protein
OG_02202	LA29479_4190	pilZ domain protein	OG_02202	LA76x_4183	LA76x_4183; pilZ domain protein
OG_02203	LA29479_0865	phosphopyruvate hydratase	OG_02203	LA76x_3704	eno; phosphopyruvate hydratase
OG_02204	LA29479_4022	peptidase M23 family protein	OG_02204	LA76x_4610	LA76x_4610; peptidase M23 family protein
OG_02205	LA29479_3213	phosphate-binding protein	OG_02205	LA76x_1812	LA76x_1812; ABC transporter, phosphonate, periplasmic substrate-bi
OG_02206	LA29479_0303	hlyD secretion family protein	OG_02206	LA76x_5140	LA76x_5140; efflux transporter, RND family, MFP subunit

OG_02207	LA29479_2293	putative uncharacterized protein	OG_02207	LA76x_4356	LA76x_4356; conserved hypothetical protein
OG_02208	LA29479_0941	prokaryotic transcription elongation factor, GreA/GreB, C-terminal dor	OG_02208	LA76x_3783	LA76x_3783; transcription elongation factor, GreA/GreB, C-term fami
OG_02209	LA29479_4179	DNA-directed RNA polymerase, omega subunit	OG_02209	LA76x_4173	rpoZ; DNA-directed RNA polymerase, omega subunit
OG_02210	LA29479_0262	hypothetical protein	OG_02210	LA76x_2926	LA76x_2926; conserved hypothetical protein
OG_02211	LA29479_2322	ATP synthase F1, gamma subunit	OG_02211	LA76x_4385	atpG; ATP synthase F1, gamma subunit
OG_02212	LA29479_3657	putative uncharacterized protein orf556	OG_02212	LA76x_3218	LA76x_3218; bacteriocin-protection, YdeI/OmpD-Associated family pi
OG_02213	LA29479_1286	efflux transporter, outer membrane factor (OMF) lipo, NodT family pro	OG_02213	LA76x_85	LA76x_85; efflux transporter, outer membrane factor (OMF) lipo, Nod
OG_02214	LA29479_3946	putative uncharacterized protein	OG_02214	LA76x_777	LA76x_777; conserved hypothetical protein
OG_02215	LA29479_4898	acyl carrier protein	OG_02215	LA76x_3499	acpP; acyl carrier protein
OG_02216	LA29479_4835	polyhydroxyalkanoate synthesis repressor PhaR	OG_02216	LA76x_3302	phaR; polyhydroxyalkanoate synthesis repressor PhaR
OG_02217	LA29479_2138	amino acid/peptide transporter family protein	OG_02217	LA76x_1014	LA76x_1014; H+ symporter) family protein
OG_02218	LA29479_4999	putative uncharacterized protein	OG_02218	LA76x_4719	LA76x_4719; outer membrane beta-barrel domain protein
OG_02219	LA29479_4015	putative lipoprotein	OG_02219	LA76x_4602	LA76x_4602; conserved hypothetical protein
OG_02220	LA29479_4970	bacterial regulatory s, lacI family protein	OG_02220	LA76x_381	LA76x_381; bacterial regulatory, lacI family protein
OG_02221	LA29479_4049	putative secreted protein	OG_02221	LA76x_278	LA76x_278; conserved hypothetical protein
OG_02222	LA29479_1989	ATP-dependent chaperone ClpB	OG_02222	LA76x_1213	clpB; ATP-dependent chaperone protein ClpB
OG_02223	LA29479_0474	catalase family protein	OG_02223	LA76x_4884	LA76x_4884; catalase family protein
OG_02224	LA29479_4403	putative transcriptional regulator	OG_02224	LA76x_513	LA76x_513; putative transcriptional regulator
OG_02225	LA29479_0895	insulinase family protein	OG_02225	LA76x_3734	LA76x_3734; peptidase M16 inactive domain protein
OG_02226	LA29479_1416	putative membrane protein	OG_02226	LA76x_2088	LA76x_2088; conserved hypothetical protein
OG_02227	LA29479_1443	3-methyl-2-oxobutanoate hydroxymethyltransferase	OG_02227	LA76x_2063	panB; 3-methyl-2-oxobutanoate hydroxymethyltransferase
OG_02228	LA29479_1195	glucose-6-phosphate dehydrogenase	OG_02228	LA76x_2449	zwf; glucose-6-phosphate dehydrogenase
OG_02229	LA29479_0196	phosphoglucosamine mutase	OG_02229	LA76x_2994	glmM; phosphoglucosamine mutase
OG_02230	LA29479_4491	malate synthase A	OG_02230	LA76x_4668	aceB; malate synthase A
OG_02231	LA29479_3456	putative uncharacterized protein	OG_02231	LA76x_4240	LA76x_4240; conserved hypothetical protein
OG_02232	LA29479_3687	hypothetical protein	OG_02232	LA76x_3191	LA76x_3191; hypothetical protein
OG_02233	LA29479_4947	histone deacetylase domain protein	OG_02233	LA76x_4035	LA76x_4035; histone deacetylase domain protein
OG_02234	LA29479_2718	putative peptidoglycan binding domain protein	OG_02234	LA76x_2417	LA76x_2417; putative peptidoglycan binding domain protein
OG_02235	LA29479_2406	methylmalonate-semialdehyde dehydrogenase	OG_02235	LA76x_3376	mmsA; methylmalonate-semialdehyde dehydrogenase
OG_02236	LA29479_5184	conserved hypothetical protein	OG_02236	LA76x_4803	epaO; type III secretion apparatus protein, YscQ/HrcQ family
OG_02237	LA29479_4391	PDZ domain family protein	OG_02237	LA76x_1446	LA76x_1446; PDZ domain family protein
OG_02238	LA29479_1663	putative membrane protein	OG_02238	LA76x_2172	LA76x_2172; hypothetical protein
OG_02239	LA29479_3093	oxidoreductase FAD-binding domain protein	OG_02239	LA76x_904	LA76x_904; 2Fe-2S iron-sulfur cluster binding domain protein
OG_02240	LA29479_3595	nitroreductase family protein	OG_02240	LA76x_4004	LA76x_4004; nitroreductase family protein
OG_02241	LA29479_1705	thioesterase domain protein	OG_02241	LA76x_2127	LA76x_2127; alpha/beta hydrolase family protein
OG_02242	LA29479_3421	rubredoxin-2	OG_02242	LA76x_4207	rubA; rubredoxin
OG_02243	LA29479_1638	succinate dehydrogenase and fumarate reductase iron-sulfur family p	OG_02243	LA76x_2197	LA76x_2197; succinate dehydrogenase and fumarate reductase iron-
OG_02244	LA29479_3875	transcription-repair coupling factor	OG_02244	LA76x_3576	mfd; transcription-repair coupling factor
OG_02245	LA29479_2004	response regulator	OG_02245	LA76x_1199	LA76x_1199; bacterial regulatory , Fis family protein
OG_02246	LA29479_3172	putative L-sorbosone dehydrogenase	OG_02246	LA76x_1850	LA76x_1850; glucose / Sorbosone dehydrogenase family protein
OG_02247	LA29479_5039	putative uncharacterized protein	OG_02247	LA76x_4701	LA76x_4701; conserved hypothetical protein
OG_02248	LA29479_0719	magnesium transporter	OG_02248	LA76x_1556	mgtE; magnesium transporter
OG_02249	LA29479_0190	N-(5'phosphoribosyl)anthranilate (PRA) isomerase family protein	OG_02249	LA76x_3000	LA76x_3000; N-(5'phosphoribosyl)anthranilate (PRA) isomerase fami
OG_02250	LA29479_3420	conserved hypothetical protein	OG_02250	LA76x_4206	LA76x_4206; conserved hypothetical protein
OG_02251	LA29479_4924	diaminopimelate epimerase	OG_02251	LA76x_810	dapF; diaminopimelate epimerase
OG_02252	LA29479_4006	putative transmembrane protein	OG_02252	LA76x_4593	LA76x_4593; SURF1 family protein
OG_02253	LA29479_3120	glycosyl hydrolases 8 family protein	OG_02253	LA76x_932	LA76x_932; glycosyl hydrolases 8 family protein
OG_02254	LA29479_4725	response regulator	OG_02254	LA76x_3481	LA76x_3481; response regulator
OG_02255	LA29479_3681	ferrous iron transport protein B	OG_02255	LA76x_3195	feoB; ferrous iron transport protein B
OG_02256	LA29479_4349	putative uncharacterized protein	OG_02256	LA76x_1486	LA76x_1486; conserved hypothetical protein
OG_02257	LA29479_1854	thioredoxin-disulfide reductase	OG_02257	LA76x_2776	trxB; thioredoxin-disulfide reductase
OG_02258	LA29479_0313	putative uncharacterized protein	OG_02258	LA76x_5131	LA76x_5131; conserved hypothetical protein
OG_02259	LA29479_4931	putative membrane protein	OG_02259	LA76x_817	LA76x_817; conserved hypothetical protein
OG_02260	LA29479_4737	tonB family C-terminal domain protein	OG_02260	LA76x_2294	LA76x_2294; tonB family C-terminal domain protein
OG_02261	LA29479_4558	putative voltage-gated potassium channel subunit beta	OG_02261	LA76x_1440	Kcnab2; voltage-gated potassium channel subunit beta-2
OG_02262	LA29479_0311	endonuclease/Exonuclease/phosphatase family protein	OG_02262	LA76x_5133	LA76x_5133; endonuclease/Exonuclease/phosphatase family protein
OG_02263	LA29479_5045	putative uncharacterized protein	OG_02263	LA76x_4695	LA76x_4695; PLD-like domain protein
OG_02264	LA29479_3889	MOSC domain protein	OG_02264	LA76x_3590	LA76x_3590; MOSC domain protein
OG_02265	LA29479_1916	RNA 2'-O ribose methyltransferase substrate binding family protein	OG_02265	LA76x_1376	LA76x_1376; RNA 2'-O ribose methyltransferase substrate binding fa
OG_02266	LA29479_1694	putative uncharacterized domain protein	OG_02266	LA76x_2138	LA76x_2138; conserved hypothetical protein
OG_02267	LA29479_2029	D-alanine--D-alanine ligase B	OG_02267	LA76x_1176	ddl; D-alanine--D-alanine ligase
OG_02268	LA29479_0958	bacterial regulatory helix-turn-helix , lysR family protein	OG_02268	LA76x_3801	LA76x_3801; bacterial regulatory helix-turn-helix , lysR family protein
OG_02269	LA29479_3249	conserved hypothetical protein	OG_02269	LA76x_231	LA76x_231; conserved hypothetical protein
OG_02270	LA29479_4330	alpha/beta hydrolase fold family protein	OG_02270	LA76x_1504	LA76x_1504; alpha/beta hydrolase fold family protein
OG_02271	LA29479_0652	feS assembly SUF system regulator	OG_02271	LA76x_1623	LA76x_1623; feS assembly SUF system regulator
OG_02272	LA29479_4137	patatin-like phospholipase family protein	OG_02272	LA76x_317	LA76x_317; patatin-like phospholipase family protein
OG_02273	LA29479_2465	outer membrane autotransporter barrel domain protein	OG_02273	LA76x_3429	LA76x_3429; autotransporter beta-domain protein
OG_02274	LA29479_1587	fatty acyl-CoA reductase domain protein	OG_02274	LA76x_2247	acrA1; oxidoreductase, short-chain dehydrogenase/reductase family
OG_02275	LA29479_1632	liporeleasing system, ATP-binding protein	OG_02275	LA76x_2203	lolD; liporeleasing system, ATP-binding protein
OG_02276	LA29479_1032	pfkB carbohydrate kinase family protein	OG_02276	LA76x_2617	LA76x_2617; pfkB carbohydrate kinase family protein
OG_02277	LA29479_0216	proton-translocating NADH-quinone oxidoreductase, chain L family pr	OG_02277	LA76x_2975	LA76x_2975; proton-translocating NADH-quinone oxidoreductase, ch
OG_02278	LA29479_3827	acetyltransferase family protein	OG_02278	LA76x_4743	LA76x_4743; acetyltransferase family protein
OG_02279	LA29479_3257	aldehyde dehydrogenase family protein	OG_02279	LA76x_223	LA76x_223; aldehyde dehydrogenase family protein
OG_02280	LA29479_2980	pirin family protein	OG_02280	LA76x_2667	LA76x_2667; cupin domain protein
OG_02281	LA29479_1453	phoH-like family protein	OG_02281	LA76x_2052	LA76x_2052; AAA domain protein
OG_02282	LA29479_1312	putative uncharacterized protein	OG_02282	LA76x_60	LA76x_60; metA-pathway of phenol degradation family protein
OG_02283	LA29479_4181	UPF0701 protein yicC	OG_02283	LA76x_4175	yicC; conserved protein
OG_02285	LA29479_2238	putative uncharacterized domain protein	OG_02285	LA76x_4305	LA76x_4305; conserved hypothetical protein
OG_02286	LA29479_3627	hypothetical protein	OG_02286	LA76x_3243	LA76x_3243; hypothetical protein
OG_02287	LA29479_0830	metallo-beta-lactamase superfamily protein	OG_02287	LA76x_3672	LA76x_3672; metallo-beta-lactamase superfamily protein
OG_02288	LA29479_5195	sensory box protein	OG_02288	LA76x_3298	LA76x_3298; diguanylate cyclase domain protein

OG_02289	LA29479_0671	adenylylsulfate kinase
OG_02290	LA29479_2798	putative transmembrane protein
OG_02291	LA29479_3142	ATP-dependent protease La
OG_02292	LA29479_2947	aldehyde dehydrogenase family protein
OG_02293	LA29479_3491	yceI-like domain protein
OG_02294	LA29479_3392	ribosomal protein L24
OG_02295	LA29479_3886	methyltransferase domain protein
OG_02296	LA29479_3118	6-phosphogluconate dehydrogenase
OG_02297	LA29479_2580	putative membrane protein
OG_02298	LA29479_4316	tyrosine recombinase XerD
OG_02299	LA29479_1876	phosphotransferase enzyme family protein
OG_02300	LA29479_2050	conserved hypothetical protein
OG_02301	LA29479_0875	uncharacterized protein ycaQ
OG_02302	LA29479_4039	putative uncharacterized protein
OG_02303	LA29479_1768	chaperone protein DnaJ
OG_02304	LA29479_1333	response regulator
OG_02305	LA29479_4990	lysM domain protein
OG_02306	LA29479_4729	possible transmembrane protein
OG_02307	LA29479_2562	phosphate-selective porin O and P family protein
OG_02308	LA29479_5009	pyridoxal-phosphate dependent enzyme family protein
OG_02309	LA29479_3246	shikimate 5-dehydrogenase
OG_02310	LA29479_4946	ATP:cob(I)alamin adenosyltransferase, putative
OG_02311	LA29479_4994	putative uncharacterized protein
OG_02312	LA29479_3923	organic hydroperoxide resistance protein
OG_02313	LA29479_1057	FAD dependent oxidoreductase family protein
OG_02314	LA29479_1517	ribosomal protein S20
OG_02315	LA29479_4182	ribonuclease PH
OG_02316	LA29479_4767	seryl-tRNA synthetase
OG_02317	LA29479_4355	hypothetical protein
OG_02318	LA29479_4942	rod shape-determining protein mreB
OG_02319	LA29479_0883	conserved hypothetical protein
OG_02320	LA29479_5141	patatin-like phospholipase family protein
OG_02321	LA29479_3492	putative malonic semialdehyde reductase RutE
OG_02322	LA29479_4159	pilus assembly , PilP family protein
OG_02323	LA29479_0191	bacterial regulatory helix-turn-helix , lysR family protein
OG_02324	LA29479_3098	asnC family protein
OG_02325	LA29479_1422	RDD family protein
OG_02326	LA29479_0462	hypothetical protein
OG_02327	LA29479_4685	cheB methylesterase family protein
OG_02328	LA29479_1987	amidohydrolase family protein
OG_02329	LA29479_3969	molydopterin dinucleotide binding domain protein
OG_02330	LA29479_3226	response regulator
OG_02331	LA29479_0200	uracil DNA glycosylase superfamily protein
OG_02332	LA29479_1309	rhomboid family protein
OG_02333	LA29479_1120	bacterial regulatory , arsR family protein
OG_02334	LA29479_1332	HAMP domain protein
OG_02335	LA29479_4184	glyoxalase/Bleomycin resistance /Dioxygenase superfamily protein
OG_02336	LA29479_5130	tonB family C-terminal domain protein
OG_02337	LA29479_0761	L-serine ammonia-lyase
OG_02338	LA29479_4369	tat (twin-arginine translocation) pathway signal sequence domain prot
OG_02339	LA29479_1485	ribosomal protein L19
OG_02340	LA29479_0934	tfoX N-terminal domain protein
OG_02341	LA29479_1375	ribosomal protein L34
OG_02342	LA29479_0110	endoribonuclease L-PSP family protein
OG_02343	LA29479_1394	hypothetical protein
OG_02344	LA29479_3287	glutathione S-transferase
OG_02345	LA29479_4235	hypothetical protein
OG_02346	LA29479_3486	lipid A biosynthesis lauroyl (or palmitoleoyl) acyltransferase family pro
OG_02347	LA29479_2066	electron transfer flavodomain protein
OG_02348	LA29479_0570	uncharacterized UPF0054 family protein
OG_02349	LA29479_3904	SPFH domain / Band 7 family protein
OG_02350	LA29479_2425	hypothetical protein
OG_02351	LA29479_1512	4-hydroxy-3-methylbut-2-enyl diphosphate reductase
OG_02352	LA29479_3841	X-Pro dipeptidyl-peptidase C-terminal non-catalytic domain protein
OG_02353	LA29479_0256	nucleotidyltransferase/DNA polymerase involved in DNA repair
OG_02354	LA29479_1688	putative uncharacterized protein
OG_02355	LA29479_1687	conserved hypothetical protein
OG_02356	LA29479_1975	nicotinate-nucleotide--dimethylbenzimidazole phosphoribosyltransfera
OG_02357	LA29479_0913	spermidine N(1)-acetyltransferase
OG_02358	LA29479_3113	oxidoreductase YdhF
OG_02359	LA29479_2143	pyruvate dehydrogenase (acetyl-transferring) E1 component, alpha su
OG_02360	LA29479_3902	phosphoribosylglycinamide formyltransferase 2
OG_02361	LA29479_4559	hypothetical protein
OG_02362	LA29479_1134	ABC transporter family protein
OG_02363	LA29479_2745	cytochrome c-type biogenesis protein CcmE
OG_02364	LA29479_0782	conserved hypothetical protein
OG_02365	LA29479_4194	lin1944 protein
OG_02366	LA29479_3081	peptidase M28 family protein
OG_02367	LA29479_4887	anti sigma-E RseA, N-terminal domain protein
OG_02368	LA29479_3469	pyridoxal-phosphate dependent enzyme family protein
OG_02369	LA29479_4358	phosphoenolpyruvate carboxykinase family protein

OG_02289	LA76x_1604	cysN/C; sulfate adenylate transferase, subunit 1/adenylylsulfate kinas
OG_02290	LA76x_2335	LA76x_2335; helix-turn-helix family protein
OG_02291	LA76x_1876	lon; ATP-dependent protease La
OG_02292	LA76x_738	LA76x_738; aldehyde dehydrogenase family protein
OG_02293	LA76x_4274	LA76x_4274; yceI-like domain protein
OG_02294	LA76x_3917	rplX; ribosomal protein L24
OG_02295	LA76x_3587	LA76x_3587; methyltransferase domain protein
OG_02296	LA76x_930	gnd; 6-phosphogluconate dehydrogenase
OG_02297	LA76x_4531	LA76x_4531; conserved hypothetical protein
OG_02298	LA76x_1119	xerD; tyrosine recombinase XerD
OG_02299	LA76x_2754	LA76x_2754; phosphotransferase enzyme family protein
OG_02300	LA76x_1102	LA76x_1102; conserved hypothetical protein
OG_02301	LA76x_3714	ycaQ; conserved protein
OG_02302	LA76x_289	LA76x_289; ycgL domain protein
OG_02303	LA76x_2861	dnaJ; chaperone protein DnaJ
OG_02304	LA76x_39	LA76x_39; transcriptional regulatory , C terminal family protein
OG_02305	LA76x_362	LA76x_362; lysM domain protein
OG_02306	LA76x_3485	LA76x_3485; putative transmembrane protein
OG_02307	LA76x_4514	LA76x_4514; phosphate-selective porin O and P family protein
OG_02308	LA76x_4709	LA76x_4709; pyridoxal-phosphate dependent enzyme family protein
OG_02309	LA76x_234	aroE; shikimate 5-dehydrogenase
OG_02310	LA76x_4034	LA76x_4034; ATP:cob(I)alamin adenosyltransferase, putative
OG_02311	LA76x_4724	LA76x_4724; conserved hypothetical protein
OG_02312	LA76x_753	ohr; organic hydroperoxide resistance protein
OG_02313	LA76x_2585	LA76x_2585; pyridine nucleotide-disulfide oxidoreductase family prot
OG_02314	LA76x_1986	rpsT; ribosomal protein S20
OG_02315	LA76x_4176	rph; ribonuclease PH
OG_02316	LA76x_2263	serS; serine--tRNA ligase
OG_02317	LA76x_1482	LA76x_1482; conserved hypothetical protein
OG_02318	LA76x_829	mreB; mreB
OG_02319	LA76x_3723	LA76x_3723; conserved hypothetical protein
OG_02320	LA76x_2250	LA76x_2250; patatin-like phospholipase family protein
OG_02321	LA76x_4275	LA76x_4275; nitroreductase family protein
OG_02322	LA76x_4153	LA76x_4153; pilus assembly , PilP family protein
OG_02323	LA76x_2999	LA76x_2999; bacterial regulatory helix-turn-helix , lysR family protein
OG_02324	LA76x_909	LA76x_909; asnC family protein
OG_02325	LA76x_2082	LA76x_2082; RDD family protein
OG_02326	LA76x_4897	LA76x_4897; hlyD secretion family protein
OG_02327	LA76x_4070	LA76x_4070; cheB methylesterase family protein
OG_02328	LA76x_1214	LA76x_1214; amidohydrolase family protein
OG_02329	LA76x_801	LA76x_801; molydopterin dinucleotide binding domain protein
OG_02330	LA76x_1800	LA76x_1800; bacterial regulatory, luxR family protein
OG_02331	LA76x_2990	LA76x_2990; DNA-deoxyinosine glycosylase
OG_02332	LA76x_64	LA76x_64; rhomboid family protein
OG_02333	LA76x_2524	LA76x_2524; methyltransferase domain protein
OG_02334	LA76x_40	LA76x_40; HAMP domain protein
OG_02335	LA76x_4178	LA76x_4178; glyoxalase/Bleomycin resistance /Dioxygenase superfa
OG_02336	LA76x_2261	LA76x_2261; tonB family C-terminal domain protein
OG_02337	LA76x_1515	LA76x_1515; L-serine ammonia-lyase
OG_02338	LA76x_1468	LA76x_1468; NAD dependent epimerase/dehydratase family protein
OG_02339	LA76x_2017	rplS; ribosomal protein L19
OG_02340	LA76x_3775	LA76x_3775; tfoX N-terminal domain protein
OG_02341	LA76x_5203	rpmH; ribosomal protein L34
OG_02342	LA76x_3076	LA76x_3076; endoribonuclease L-PSP family protein
OG_02343	LA76x_5184	LA76x_5184; hypothetical protein
OG_02344	LA76x_196	LA76x_196; glutathione S-transferase
OG_02345	LA76x_1767	LA76x_1767; hypothetical protein
OG_02346	LA76x_4269	lpxL; lipid A biosynthesis lauroyl (or palmitoleoyl) acyltransferase fami
OG_02347	LA76x_1086	LA76x_1086; electron transfer flavodomain protein
OG_02348	LA76x_1693	LA76x_1693; conserved hypothetical protein
OG_02349	LA76x_3604	LA76x_3604; SPFH domain / Band 7 family protein
OG_02350	LA76x_3394	LA76x_3394; glycosyl transferases group 1 family protein
OG_02351	LA76x_1992	ispH; 4-hydroxy-3-methylbut-2-enyl diphosphate reductase
OG_02352	LA76x_4730	LA76x_4730; hydrolase CocE/NonD family protein
OG_02353	LA76x_2932	LA76x_2932; impB/mucB/samB family protein
OG_02354	LA76x_2145	LA76x_2145; conserved hypothetical protein
OG_02355	LA76x_2146	LA76x_2146; pseudouridine synthase family protein
OG_02356	LA76x_1226	cobT; nicotinate-nucleotide--dimethylbenzimidazole phosphoribosyltra
OG_02357	LA76x_3753	LA76x_3753; spermidine n1-acetyltransferase
OG_02358	LA76x_925	LA76x_925; aldo/keto reductase family protein
OG_02359	LA76x_1009	pdhA; pyruvate dehydrogenase (acetyl-transferring) E1 component, a
OG_02360	LA76x_3602	purT; phosphoribosylglycinamide formyltransferase 2
OG_02361	LA76x_1439	LA76x_1439; hypothetical protein
OG_02362	LA76x_2510	LA76x_2510; ABC transporter family protein
OG_02363	LA76x_2389	LA76x_2389; ccmE family protein
OG_02364	LA76x_3627	LA76x_3627; triose-phosphate Transporter family protein
OG_02365	LA76x_4186	LA76x_4186; conserved hypothetical protein
OG_02366	LA76x_894	LA76x_894; peptidase M20/M25/M40 family protein
OG_02367	LA76x_3489	LA76x_3489; anti sigma-E RseA, N-terminal domain protein
OG_02368	LA76x_4253	LA76x_4253; pyridoxal-phosphate dependent enzyme family protein
OG_02369	LA76x_1479	LA76x_1479; phosphoenolpyruvate carboxykinase family protein

OG_02370	LA29479_4543	putative secreted protein	OG_02370	LA76x_1890	LA76x_1890; conserved hypothetical protein
OG_02371	LA29479_2507	6-phosphogluconolactonase	OG_02371	LA76x_4462	LA76x_4462; lactonase, 7-bladed beta-propeller family protein
OG_02372	LA29479_5071	conserved hypothetical protein	OG_02372	LA76x_4807	LA76x_4807; type III secretion , YscU/HrpY family protein
OG_02373	LA29479_2063	putative uncharacterized protein dcsG	OG_02373	LA76x_1089	LA76x_1089; ATP-grasp domain protein
OG_02374	LA29479_2518	excalibur calcium-binding domain protein	OG_02374	LA76x_4473	LA76x_4473; excalibur calcium-binding domain protein
OG_02375	LA29479_2760	putative uncharacterized protein	OG_02375	LA76x_2374	LA76x_2374; conserved hypothetical protein
OG_02376	LA29479_2803	bacterial regulatory s, tetR family protein	OG_02376	LA76x_2330	LA76x_2330; bacterial regulatory, tetR family protein
OG_02377	LA29479_0682	RNA 2'-phosphotransferase, Tpt1 / KptA family protein	OG_02377	LA76x_1591	LA76x_1591; RNA 2'-phosphotransferase, Tpt1 / KptA family protein
OG_02378	LA29479_1327	RNA polymerase sigma factor, sigma-70 family protein	OG_02378	LA76x_44	LA76x_44; RNA polymerase sigma factor, sigma-70 family protein
OG_02379	LA29479_0453	ubiE/COQ5 methyltransferase family protein	OG_02379	LA76x_4906	LA76x_4906; methyltransferase domain protein
OG_02380	LA29479_3653	imidazole glycerol phosphate synthase subunit hisF	OG_02380	LA76x_3222	hisF; imidazole glycerol phosphate synthase, HisF subunit
OG_02381	LA29479_4753	isochorismatase family protein	OG_02381	LA76x_2278	LA76x_2278; isochorismatase family protein
OG_02382	LA29479_2046	CDP-diacylglycerol--serine O-phosphatidyltransferase	OG_02382	LA76x_1106	pssA; CDP-diacylglycerol-serine O-phosphatidyltransferase
OG_02383	LA29479_3868	conserved hypothetical protein	OG_02383	LA76x_3568	LA76x_3568; conserved hypothetical protein
OG_02384	LA29479_4083	putative pteridine-dependent dioxygenase	OG_02384	LA76x_248	LA76x_248; putative pteridine-dependent dioxygenase
OG_02385	LA29479_1201	bacterial extracellular solute-binding family protein	OG_02385	LA76x_2443	LA76x_2443; bacterial extracellular solute-binding family protein
OG_02386	LA29479_2486	bacterial regulatory s, tetR family protein	OG_02386	LA76x_4439	LA76x_4439; bacterial regulatory, tetR family protein
OG_02387	LA29479_4836	acetoacetyl-CoA reductase	OG_02387	LA76x_3304	phbB; acetoacetyl-CoA reductase family protein
OG_02389	LA29479_4839	ribonuclease D	OG_02389	LA76x_3307	rnd; ribonuclease D
OG_02390	LA29479_1832	lysyl-tRNA synthetase	OG_02390	LA76x_2797	lysS; lysine--tRNA ligase
OG_02391	LA29479_1966	ABC transporter family protein	OG_02391	LA76x_1235	LA76x_1235; ABC transporter family protein
OG_02392	LA29479_0787	putative uncharacterized protein	OG_02392	LA76x_3632	LA76x_3632; conserved hypothetical protein
OG_02393	LA29479_2887	cheW-like domain protein	OG_02393	LA76x_691	LA76x_691; cheW-like domain protein
OG_02394	LA29479_2799	type IV pilus biogenesis/stability protein PilW	OG_02394	LA76x_2334	pilF; type IV pilus biogenesis/stability protein PilW
OG_02395	LA29479_0442	short chain fatty acid transporter family protein	OG_02395	LA76x_4915	LA76x_4915; C4-dicarboxylate anaerobic carrier family protein
OG_02396	LA29479_1940	protein tolA-like	OG_02396	LA76x_1259	LA76x_1259; tonB C terminal family protein
OG_02397	LA29479_0249	citrate transporter family protein	OG_02397	LA76x_2940	LA76x_2940; citrate transporter family protein
OG_02398	LA29479_3962	histidine kinase family protein	OG_02398	LA76x_793	LA76x_793; histidine kinase family protein
OG_02399	LA29479_0713	UPF0042 nucleotide-binding protein yhbJ	OG_02399	LA76x_1562	LA76x_1562; putative P-loop containing ATPase
OG_02400	LA29479_4678	nucleoside triphosphate pyrophosphohydrolase	OG_02400	LA76x_4063	mazG; nucleoside triphosphate pyrophosphohydrolase
OG_02401	LA29479_4255	ribosomal protein L20	OG_02401	LA76x_1747	rplT; ribosomal protein L20
OG_02402	LA29479_3451	bacterial regulatory helix-turn-helix s, AraC family protein	OG_02402	LA76x_4235	LA76x_4235; bacterial regulatory helix-turn-helix, AraC family protein
OG_02403	LA29479_3423	glutamate-1-semialdehyde-2,1-aminomutase	OG_02403	LA76x_4209	hemL; glutamate-1-semialdehyde-2,1-aminomutase
OG_02404	LA29479_3573	leupeptin-inactivating enzyme 1	OG_02404	LA76x_3981	lieA; leupeptin-inactivating enzyme 1
OG_02405	LA29479_3612	conserved hypothetical protein	OG_02405	LA76x_4020	LA76x_4020; eamA-like transporter family protein
OG_02406	LA29479_1733	DEAD/DEAH box helicase family protein	OG_02406	LA76x_2101	LA76x_2101; helicase conserved C-terminal domain protein
OG_02407	LA29479_2321	ATP synthase F1, beta subunit	OG_02407	LA76x_4384	atpD; ATP synthase F1, beta subunit
OG_02408	LA29479_0252	response regulator	OG_02408	LA76x_2937	LA76x_2937; bacterial regulatory, luxR family protein
OG_02409	LA29479_0522	acyltransferase family protein	OG_02409	LA76x_4839	LA76x_4839; acyltransferase family protein
OG_02410	LA29479_0982	tonB-dependent Receptor Plug domain protein	OG_02410	LA76x_3826	LA76x_3826; tonB dependent receptor family protein
OG_02411	LA29479_2283	queuosine biosynthesis protein QueD	OG_02411	LA76x_4347	queD; queuosine biosynthesis protein QueD
OG_02412	LA29479_4916	putative membrane protein	OG_02412	LA76x_3515	LA76x_3515; hypothetical protein
OG_02413	LA29479_5047	conserved hypothetical protein	OG_02413	LA76x_4693	LA76x_4693; conserved hypothetical protein
OG_02414	LA29479_0709	RNA polymerase sigma-54 factor	OG_02414	LA76x_1566	rpoN; RNA polymerase sigma-54 factor
OG_02415	LA29479_4696	glyoxalase	OG_02415	LA76x_4081	LA76x_4081; glyoxalase-like domain protein
OG_02416	LA29479_3470	2-isopropylmalate synthase	OG_02416	LA76x_4254	leuA; 2-isopropylmalate synthase
OG_02417	LA29479_4544	ribonuclease G	OG_02417	LA76x_1889	cafA; ribonuclease G
OG_02419	LA29479_4490	bacterial regulatory helix-turn-helix , lysR family protein	OG_02419	LA76x_4669	LA76x_4669; bacterial regulatory helix-turn-helix , lysR family protein
OG_02420	LA29479_2159	sporulation related domain protein	OG_02420	LA76x_994	LA76x_994; sporulation related domain protein
OG_02421	LA29479_1281	glutamate synthase [NADPH] small chain	OG_02421	LA76x_90	gltD; glutamate synthase (NADPH) small chain glutamate synthase, s
OG_02422	LA29479_4446	arginyl-tRNA synthetase	OG_02422	LA76x_4636	argS; arginine--tRNA ligase
OG_02423	LA29479_2018	bacterial type II secretion system F domain protein	OG_02423	LA76x_1187	LA76x_1187; type II secretion system (T2SS), F family protein
OG_02424	LA29479_3682	putative uncharacterized protein	OG_02424	LA76x_3194	LA76x_3194; conserved hypothetical protein
OG_02425	LA29479_3995	conserved hypothetical protein	OG_02425	LA76x_4582	yihY; yihY family inner membrane domain protein
OG_02426	LA29479_0214	NADH-ubiquinone/plastoquinone oxidoreductase chain 6 family prote	OG_02426	LA76x_2977	LA76x_2977; NADH-ubiquinone/plastoquinone oxidoreductase chain
OG_02427	LA29479_1105	putative secreted protein	OG_02427	LA76x_2537	LA76x_2537; conserved hypothetical protein
OG_02428	LA29479_4582	adenosylhomocysteinase	OG_02428	LA76x_1416	ahcY; adenosylhomocysteinase
OG_02429	LA29479_0218	proton-translocating NADH-quinone oxidoreductase, chain N family p	OG_02429	LA76x_2973	LA76x_2973; proton-translocating NADH-quinone oxidoreductase, ch
OG_02430	LA29479_1324	response regulator	OG_02430	LA76x_48	LA76x_48; bacterial regulatory, luxR family protein
OG_02431	LA29479_0085	conserved hypothetical protein	OG_02431	LA76x_3103	LA76x_3103; conserved hypothetical protein
OG_02432	LA29479_1937	tol-pal system protein YbgF	OG_02432	LA76x_1262	ygbF; tol-pal system protein YbgF
OG_02433	LA29479_3945	O-methyltransferase N-terminus family protein	OG_02433	LA76x_776	LA76x_776; S-adenosyl methyltransferase family protein
OG_02434	LA29479_5068	putative uncharacterized protein	OG_02434	LA76x_4810	LA76x_4810; hypothetical protein
OG_02435	LA29479_4829	putative uncharacterized protein	OG_02435	LA76x_3292	LA76x_3292; conserved hypothetical protein
OG_02436	LA29479_4309	bacterial type II secretion system F domain protein	OG_02436	LA76x_1126	LA76x_1126; type II secretion system (T2SS), F family protein
OG_02437	LA29479_0276	methyltransferase	OG_02437	LA76x_5169	LA76x_5169; methyltransferase domain protein
OG_02438	LA29479_3324	putative membrane protein	OG_02438	LA76x_163	LA76x_163; hypothetical protein
OG_02439	LA29479_4014	delta-1-pyrroline-5-carboxylate dehydrogenase	OG_02439	LA76x_4601	LA76x_4601; delta-1-pyrroline-5-carboxylate dehydrogenase
OG_02440	LA29479_4282	3,4-dihydroxy-2-butanone 4-phosphate synthase	OG_02440	LA76x_1153	ribB; 3,4-dihydroxy-2-butanone-4-phosphate synthase
OG_02441	LA29479_4913	outer membrane lipoSlp family protein	OG_02441	LA76x_3512	LA76x_3512; outer membrane lipoSlp family protein
OG_02442	LA29479_4695	putative molecular chaperone	OG_02442	LA76x_4080	LA76x_4080; putative peptidase
OG_02443	LA29479_1350	thermolysin metallopeptidase, catalytic domain protein	OG_02443	LA76x_21	LA76x_21; thermolysin metallopeptidase, catalytic domain protein
OG_02444	LA29479_2582	uncharacterised , DegV COG1307 family protein	OG_02444	LA76x_4533	LA76x_4533; EDD, DegV family domain protein
OG_02445	LA29479_3038	conserved hypothetical protein	OG_02445	LA76x_854	LA76x_854; eamA-like transporter family protein
OG_02446	LA29479_4997	NAD-dependent glycerol-3-phosphate dehydrogenase N-terminus far	OG_02446	LA76x_4721	gpsA; gpsA
OG_02447	LA29479_0827	flavodoxin-like fold family protein	OG_02447	LA76x_3669	NQO2; ribosyldihydronicotinamide dehydrogenase [quinone]
OG_02448	LA29479_2495	major Facilitator Superfamily protein	OG_02448	LA76x_4448	LA76x_4448; major Facilitator Superfamily protein
OG_02449	LA29479_2170	putative uncharacterized protein	OG_02449	LA76x_985	LA76x_985; conserved hypothetical protein
OG_02450	LA29479_2081	glycosyl transferases group 1 family protein	OG_02450	LA76x_1071	LA76x_1071; glycosyl transferases group 1 family protein
OG_02451	LA29479_3365	cyclophilin type peptidyl-prolyl cis-trans isomerase/CLD family protein	OG_02451	LA76x_3891	LA76x_3891; cyclophilin type peptidyl-prolyl cis-trans isomerase/CLD
OG_02452	LA29479_0798	glyoxalase/Bleomycin resistance /Dioxygenase superfamily protein	OG_02452	LA76x_3643	LA76x_3643; glyoxalase/Bleomycin resistance /Dioxygenase superfa

OG_02453	LA29479_4627	conserved hypothetical protein	OG_02453	LA76x_3437	LA76x_3437; eamA-like transporter family protein
OG_02454	LA29479_0043	hypothetical protein	OG_02454	LA76x_3144	LA76x_3144; hypothetical protein
OG_02455	LA29479_4126	activator of Hsp90 ATPase homolog 1-like family protein	OG_02455	LA76x_328	LA76x_328; conserved hypothetical protein
OG_02456	LA29479_2806	secretion chaperone	OG_02456	LA76x_2327	csaA; secretion chaperone
OG_02457	LA29479_0134	acetyltransferase family protein	OG_02457	LA76x_3053	LA76x_3053; acetyltransferase family protein
OG_02458	LA29479_1352	bacterial regulatory helix-turn-helix , lysR family protein	OG_02458	LA76x_19	LA76x_19; bacterial regulatory helix-turn-helix , lysR family protein
OG_02459	LA29479_2171	amidinotransferase family protein	OG_02459	LA76x_984	LA76x_984; amidinotransferase family protein
OG_02460	LA29479_0853	lysM domain protein	OG_02460	LA76x_3692	LA76x_3692; lysM domain protein
OG_02461	LA29479_1056	putative uncharacterized protein	OG_02461	LA76x_2586	LA76x_2586; conserved hypothetical protein
OG_02462	LA29479_1846	ATP-dependent Clp protease adapter protein ClpS	OG_02462	LA76x_2784	clpS; ATP-dependent Clp protease adapter protein ClpS
OG_02463	LA29479_4963	lemA family protein	OG_02463	LA76x_4050	LA76x_4050; lemA family protein
OG_02464	LA29479_1473	protein YgaD	OG_02464	LA76x_2030	LA76x_2030; competence/damage-inducible CinA C-terminal domain
OG_02465	LA29479_1549	putative uncharacterized protein	OG_02465	LA76x_1955	LA76x_1955; conserved hypothetical protein
OG_02466	LA29479_0452	putative pimeloyl-BioC--CoA transferase BioH	OG_02466	LA76x_4907	bioH; pimelyl-[acyl-carrier protein] methyl ester esterase
OG_02467	LA29479_5171	putative uncharacterized protein	OG_02467	LA76x_840	LA76x_840; conserved hypothetical protein
OG_02468	LA29479_1640	succinate dehydrogenase, hydrophobic membrane anchor protein	OG_02468	LA76x_2195	sdhD; succinate dehydrogenase, hydrophobic membrane anchor prot
OG_02469	LA29479_3974	endonuclease/Exonuclease/phosphatase family protein	OG_02469	LA76x_4563	LA76x_4563; endonuclease/Exonuclease/phosphatase family protein
OG_02470	LA29479_2261	putative uncharacterized protein	OG_02470	LA76x_4327	LA76x_4327; mechanosensitive ion channel family protein
OG_02471	LA29479_4185	non-canonical purine NTP pyrophosphatase, rdgB/HAM1 family	OG_02471	LA76x_4179	rdgB; non-canonical purine NTP pyrophosphatase, RdgB/HAM1 famil
OG_02472	LA29479_3205	hypothetical protein	OG_02472	LA76x_1819	LA76x_1819; hypothetical protein
OG_02473	LA29479_1144	adenylosuccinate lyase	OG_02473	LA76x_2500	purB; adenylosuccinate lyase
OG_02474	LA29479_1535	FKBP-type peptidyl-prolyl cis-trans isomerase family protein	OG_02474	LA76x_1969	LA76x_1969; FKBP-type peptidyl-prolyl cis-trans isomerase family pr
OG_02475	LA29479_2423	bacterial transferase hexapeptide family protein	OG_02475	LA76x_3392	LA76x_3392; bacterial transferase hexapeptide family protein
OG_02476	LA29479_0315	ion channel family protein	OG_02476	LA76x_5129	LA76x_5129; ion channel family protein
OG_02477	LA29479_0535	conserved hypothetical protein	OG_02477	LA76x_4827	LA76x_4827; conserved hypothetical family protein
OG_02478	LA29479_4005	putative membrane protein	OG_02478	LA76x_4592	LA76x_4592; conserved hypothetical protein
OG_02479	LA29479_4131	tetratricopeptide repeat family protein	OG_02479	LA76x_323	LA76x_323; TPR repeat family protein
OG_02480	LA29479_4552	PPIC-type PPIASE domain protein	OG_02480	LA76x_1881	LA76x_1881; PPIC-type PPIASE domain protein
OG_02481	LA29479_1176	yceI-like domain protein	OG_02481	LA76x_2471	LA76x_2471; yceI-like domain protein
OG_02482	LA29479_4094	pilin family protein	OG_02482	LA76x_359	LA76x_359; pilin family protein
OG_02483	LA29479_4935	short chain dehydrogenase family protein	OG_02483	LA76x_822	LA76x_822; short chain dehydrogenase family protein
OG_02484	LA29479_0188	fimV protein	OG_02484	LA76x_3002	fimV; fimV N-terminal domain
OG_02485	LA29479_0184	chorismate synthase	OG_02485	LA76x_3005	aroC; chorismate synthase
OG_02486	LA29479_0121	putative uncharacterized protein	OG_02486	LA76x_3065	LA76x_3065; PLD-like domain protein
OG_02487	LA29479_1177	DNA gyrase, A subunit	OG_02487	LA76x_2470	gyrA; DNA gyrase, A subunit
OG_02488	LA29479_0800	glycine dehydrogenase	OG_02488	LA76x_3644	gcvP; glycine dehydrogenase
OG_02489	LA29479_2497	condensation domain protein	OG_02489	LA76x_4451	LA76x_4451; condensation domain protein
OG_02490	LA29479_3615	cytidine and deoxycytidylate deaminase zinc-binding region	OG_02490	LA76x_4023	LA76x_4023; cytidine and deoxycytidylate deaminase zinc-binding re
OG_02491	LA29479_3853	phosphate regulon transcriptional regulatory protein PhoB	OG_02491	LA76x_3554	phoB; phosphate regulon transcriptional regulatory protein PhoB
OG_02492	LA29479_0994	bifunctional protein folC	OG_02492	LA76x_3837	folC; FOLC
OG_02493	LA29479_1156	possible lysine decarboxylase family protein	OG_02493	LA76x_2488	LA76x_2488; putative lysine decarboxylase family protein
OG_02494	LA29479_5074	fliP family protein	OG_02494	LA76x_4804	epaP; type III secretion apparatus protein, YscR/HrcR family
OG_02495	LA29479_2628	putative uncharacterized protein	OG_02495	LA76x_555	LA76x_555; lumazine-binding family protein
OG_02496	LA29479_0575	magnesium and cobalt transport protein CorA	OG_02496	LA76x_1689	corA; magnesium and cobalt transport protein CorA
OG_02497	LA29479_1162	dnaJ domain protein	OG_02497	LA76x_2483	LA76x_2483; dnaJ C terminal domain protein
OG_02499	LA29479_3130	putative uncharacterized protein	OG_02499	LA76x_942	LA76x_942; conserved hypothetical protein
OG_02500	LA29479_1983	putative membrane protein	OG_02500	LA76x_1218	LA76x_1218; putative membrane protein
OG_02501	LA29479_4928	heat shock protein HslVU, ATPase subunit HslU	OG_02501	LA76x_815	hslU; ATP-dependent protease HslVU, ATPase subunit
OG_02502	LA29479_4298	glycosyl transferase 2 family protein	OG_02502	LA76x_1137	LA76x_1137; glycosyl transferase 2 family protein
OG_02503	LA29479_3391	ribosomal L5 family protein	OG_02503	LA76x_3916	LA76x_3916; ribosomal L5 family protein
OG_02504	LA29479_1251	5'-nucleotidase, lipoprotein e(P4) family	OG_02504	LA76x_120	LA76x_120; HAD super, subIIIB family protein
OG_02505	LA29479_1455	phosphomethylpyrimidine kinase	OG_02505	LA76x_2050	thiD; phosphomethylpyrimidine kinase
OG_02506	LA29479_0999	phosphoglycerate dehydrogenase	OG_02506	LA76x_3841	serA; phosphoglycerate dehydrogenase
OG_02507	LA29479_1198	F5/8 type C domain protein	OG_02507	LA76x_2446	LA76x_2446; F5/8 type C domain protein
OG_02508	LA29479_1771	carbamoyl-phosphate synthase, small subunit	OG_02508	LA76x_2858	carA; carbamoyl-phosphate synthase, small subunit
OG_02509	LA29479_4699	penicillin-binding protein 1B	OG_02509	LA76x_4084	mrcB; penicillin-binding protein 1B
OG_02510	LA29479_3441	putative secreted protein	OG_02510	LA76x_4225	LA76x_4225; putative secreted protein
OG_02511	LA29479_2945	pyridine nucleotide-disulphide oxidoreductase family protein	OG_02511	LA76x_736	LA76x_736; FAD binding domain protein
OG_02512	LA29479_4067	conserved hypothetical protein	OG_02512	LA76x_264	LA76x_264; conserved hypothetical protein
OG_02513	LA29479_2319	gtrA-like family protein	OG_02513	LA76x_4382	LA76x_4382; gtrA-like family protein
OG_02514	LA29479_0179	putative uncharacterized protein	OG_02514	LA76x_3011	LA76x_3011; hypothetical protein
OG_02515	LA29479_0926	conserved hypothetical protein	OG_02515	LA76x_3766	LA76x_3766; conserved hypothetical protein
OG_02516	LA29479_0732	twitching mobility protein	OG_02516	LA76x_1544	LA76x_1544; twitching motility family protein
OG_02517	LA29479_4857	DNA polymerase III, subunits gamma and tau	OG_02517	LA76x_3517	dnaX; DNA polymerase III, subunit gamma and tau
OG_02518	LA29479_3410	DNA-directed RNA polymerase, beta subunit	OG_02518	LA76x_3936	rpoB; DNA-directed RNA polymerase, beta subunit
OG_02519	LA29479_4087	rrf2 family protein	OG_02519	LA76x_244	LA76x_244; rrf2 family protein
OG_02520	LA29479_3972	patatin-like phospholipase family protein	OG_02520	LA76x_804	LA76x_804; patatin-like phospholipase family protein
OG_02521	LA29479_1222	putative membrane protein	OG_02521	LA76x_145	LA76x_145; conserved hypothetical protein
OG_02522	LA29479_3609	peptide methionine sulfoxide reductase	OG_02522	LA76x_4017	MSRA; peptide methionine sulfoxide reductase
OG_02523	LA29479_0317	UPF0719 inner membrane protein yjfL	OG_02523	LA76x_5127	LA76x_5127; conserved hypothetical protein
OG_02524	LA29479_4561	uncharacterized UPF0016 family protein	OG_02524	LA76x_1438	LA76x_1438; conserved hypothetical protein
OG_02525	LA29479_2084	conserved hypothetical protein	OG_02525	LA76x_1068	LA76x_1068; conserved hypothetical protein
OG_02526	LA29479_1545	phosphate ABC transporter, ATP-binding protein	OG_02526	LA76x_1959	pstB; phosphate ABC transporter, ATP-binding protein
OG_02527	LA29479_2498	glycosyl transferase 2 family protein	OG_02527	LA76x_4452	LA76x_4452; glycosyl transferase 2 family protein
OG_02528	LA29479_2512	amino acid permease family protein	OG_02528	LA76x_4467	LA76x_4467; amino acid permease family protein
OG_02529	LA29479_1722	bacterial regulatory helix-turn-helix s, AraC family protein	OG_02529	LA76x_2112	LA76x_2112; bacterial regulatory helix-turn-helix, AraC family protein
OG_02530	LA29479_1751	putative exported protein	OG_02530	LA76x_2877	LA76x_2877; putative membrane protein
OG_02531	LA29479_1077	putative uncharacterized protein	OG_02531	LA76x_2561	LA76x_2561; conserved hypothetical protein
OG_02532	LA29479_3668	acetyltransferase family protein	OG_02532	LA76x_3207	LA76x_3207; acetyltransferase family protein
OG_02533	LA29479_3792	RNA polymerase sigma factor, sigma-70 family protein	OG_02533	LA76x_4773	LA76x_4773; RNA polymerase sigma factor, sigma-70 family protein
OG_02534	LA29479_3914	histidine kinase-, DNA gyrase B-, and HSP90-like ATPase family prot	OG_02534	LA76x_3613	LA76x_3613; histidine kinase-, DNA gyrase B-, and HSP90-like ATPa

OG_02535	LA29479_3393	ribosomal protein L14
OG_02536	LA29479_2514	ABC transporter family protein
OG_02537	LA29479_1255	uvrD/REP helicase family protein
OG_02538	LA29479_4906	conserved hypothetical protein
OG_02539	LA29479_3380	ribosomal protein S4
OG_02540	LA29479_3982	putative uncharacterized protein
OG_02541	LA29479_1126	translation initiation factor IF-2
OG_02542	LA29479_4674	conserved hypothetical protein
OG_02543	LA29479_3402	50S ribosomal protein L4
OG_02544	LA29479_2030	UDP-N-acetylmuramate--alanine ligase
OG_02545	LA29479_0538	superoxide dismutase [Fe]
OG_02546	LA29479_5011	putative uncharacterized protein
OG_02547	LA29479_4362	putative membrane protein
OG_02548	LA29479_0931	conserved hypothetical protein
OG_02549	LA29479_3565	putative membrane protein
OG_02550	LA29479_2100	S-adenosylmethionine decarboxylase proenzyme
OG_02551	LA29479_2946	dihydrodipicolinate synthetase family protein
OG_02553	LA29479_4930	acetyltransferase family protein
OG_02554	LA29479_0839	hflK protein
OG_02555	LA29479_0622	UDP-N-acetylmuramoylalanine--D-glutamate ligase
OG_02556	LA29479_0704	hydrolase, HAD super, subfamily III A
OG_02557	LA29479_3448	inorganic pyrophosphatase
OG_02558	LA29479_4132	bacterial regulatory s, tetR family protein
OG_02559	LA29479_4119	alternative sigma factor RpoH
OG_02560	LA29479_2332	dihydrolipoyllysine-residue acetyltransferase E2 component of pyruva
OG_02561	LA29479_1977	cobyric acid synthase CobQ
OG_02562	LA29479_0143	rhodanese-like domain protein
OG_02563	LA29479_4592	GTP pyrophosphokinase
OG_02564	LA29479_4679	3'(2'),5'-bisphosphate nucleotidase
OG_02565	LA29479_1635	conserved hypothetical protein
OG_02566	LA29479_0937	tat (twin-arginine translocation) pathway signal sequence domain prot
OG_02567	LA29479_2581	DSBA-like thioredoxin domain protein
OG_02568	LA29479_0846	ATP-dependent zinc metalloprotease FtsH
OG_02569	LA29479_4240	putative membrane protein
OG_02570	LA29479_0586	dTDP-4-dehydrorhamnose 3,5-epimerase
OG_02571	LA29479_0559	ABC-2 type transporter family protein
OG_02572	LA29479_4310	general secretory pathway protein E
OG_02573	LA29479_3338	GDSL-like Lipase/Acylhydrolase family protein
OG_02574	LA29479_2742	heme exporter protein CcmB
OG_02575	LA29479_0922	molydopterin dinucleotide binding domain protein
OG_02576	LA29479_1199	binding--dependent transport system inner membrane component fan
OG_02577	LA29479_4966	hypothetical protein
OG_02578	LA29479_1304	rmuC family protein
OG_02579	LA29479_2809	hypothetical protein
OG_02580	LA29479_2885	cheW-like domain protein
OG_02581	LA29479_0859	putative lipoprotein
OG_02582	LA29479_5150	peptidase M28 family protein
OG_02583	LA29479_0860	tRNA pseudouridine synthase D family protein
OG_02584	LA29479_3317	porin
OG_02585	LA29479_1388	ABC transporter family protein
OG_02586	LA29479_1269	DEAD/DEAH box helicase family protein
OG_02587	LA29479_4614	5'-nucleotidase
OG_02588	LA29479_4618	putative membrane protein
OG_02589	LA29479_3905	putative membrane protein
OG_02590	LA29479_4230	putative uncharacterized protein
OG_02591	LA29479_3363	glutaredoxin family protein
OG_02592	LA29479_4104	uncharacterized protein
OG_02593	LA29479_2820	putative uncharacterized protein
OG_02594	LA29479_2832	efflux transporter, RND family, MFP subunit
OG_02595	LA29479_0468	glucans biosynthesis protein D
OG_02596	LA29479_2266	putative uncharacterized protein
OG_02597	LA29479_4773	putative uncharacterized protein TTHB207
OG_02598	LA29479_2316	UDP-N-acetylglucosamine diphosphorylase/glucosamine-1-phosphat
OG_02599	LA29479_4140	response regulator
OG_02600	LA29479_4107	MATE efflux family protein
OG_02601	LA29479_3048	putative uncharacterized protein
OG_02602	LA29479_0632	small GTP-binding domain protein
OG_02603	LA29479_1117	bacterial NAD-glutamate dehydrogenase family protein
OG_02604	LA29479_3549	glutamyl-tRNA reductase
OG_02605	LA29479_4713	conserved hypothetical protein
OG_02606	LA29479_0967	hypothetical protein
OG_02607	LA29479_3206	phosphate transporter family protein
OG_02608	LA29479_0764	tat (twin-arginine translocation) pathway signal sequence domain prot
OG_02609	LA29479_1478	ATPase associated with various cellular activities family protein
OG_02610	LA29479_2884	HAMP domain protein
OG_02611	LA29479_3347	NADH:flavin oxidoreductase / NADH oxidase family protein
OG_02612	LA29479_4452	hypothetical protein
OG_02613	LA29479_0269	response regulator
OG_02614	LA29479_0651	SET domain protein
OG_02615	LA29479_1252	ribonuclease UK114 domain protein
OG_02616	LA29479_1113	amino acid permease family protein

OG_02535	LA76x_3918	rplN; ribosomal protein L14
OG_02536	LA76x_4469	LA76x_4469; ABC transporter family protein
OG_02537	LA76x_115	LA76x_115; uvrD/REP helicase N-terminal domain protein
OG_02538	LA76x_3505	LA76x_3505; conserved hypothetical protein
OG_02539	LA76x_3905	rpsD; ribosomal protein S4
OG_02540	LA76x_4571	LA76x_4571; conserved hypothetical protein
OG_02541	LA76x_2518	LA76x_2518; conserved hypothetical protein
OG_02542	LA76x_4059	LA76x_4059; conserved hypothetical protein
OG_02543	LA76x_3927	rplD; 50S ribosomal protein L4
OG_02544	LA76x_1175	murC; UDP-N-acetylmuramate--alanine ligase
OG_02545	LA76x_1728	sodB; sodB
OG_02546	LA76x_4707	LA76x_4707; conserved hypothetical protein
OG_02547	LA76x_1474	LA76x_1474; conserved hypothetical protein
OG_02548	LA76x_3771	LA76x_3771; eamA-like transporter family protein
OG_02549	LA76x_3973	LA76x_3973; conserved hypothetical protein
OG_02550	LA76x_1052	speD; S-adenosylmethionine decarboxylase proenzyme
OG_02551	LA76x_737	LA76x_737; dihydrodipicolinate synthetase family protein
OG_02553	LA76x_818	LA76x_818; acetyltransferase family protein
OG_02554	LA76x_3679	hflK; hflK protein
OG_02555	LA76x_1652	murD; UDP-N-acetylmuramoylalanine--D-glutamate ligase
OG_02556	LA76x_1571	LA76x_1571; hydrolase, HAD super, subfamily III A
OG_02557	LA76x_4232	ppa; ppa
OG_02558	LA76x_322	LA76x_322; bacterial regulatory, tetR family protein
OG_02559	LA76x_335	rpoH; alternative sigma factor RpoH
OG_02560	LA76x_4396	aceF; dihydrolipoyllysine-residue acetyltransferase
OG_02561	LA76x_1224	cobQ; cobyric acid synthase CobQ
OG_02562	LA76x_3045	LA76x_3045; rhodanese-like domain protein
OG_02563	LA76x_4088	LA76x_4088; relA/SpoT family protein
OG_02564	LA76x_4064	cysQ; 3'(2'),5'-bisphosphate nucleotidase
OG_02565	LA76x_2200	LA76x_2200; flavinator of succinate dehydrogenase family protein
OG_02566	LA76x_3779	LA76x_3779; putative glucan endo-1,3-beta-D-glucosidase
OG_02567	LA76x_4532	LA76x_4532; DSBA-like thioredoxin domain protein
OG_02568	LA76x_3685	hflB; ftsH HflB
OG_02569	LA76x_1762	LA76x_1762; conserved hypothetical protein
OG_02570	LA76x_1680	rfbC; dTDP-4-dehydrorhamnose 3,5-epimerase
OG_02571	LA76x_1706	LA76x_1706; ABC-2 type transporter family protein
OG_02572	LA76x_1125	gspE; type II secretion system protein E
OG_02573	LA76x_3863	LA76x_3863; GDSL-like Lipase/Acylhydrolase family protein
OG_02574	LA76x_2392	ccmB; heme exporter protein CcmB
OG_02575	LA76x_3762	LA76x_3762; molydopterin dinucleotide binding domain protein
OG_02576	LA76x_2445	LA76x_2445; binding-protein-dependent transport system inner mem
OG_02577	LA76x_4053	LA76x_4053; hypothetical protein
OG_02578	LA76x_69	LA76x_69; rmuC family protein
OG_02579	LA76x_2325	LA76x_2325; hypothetical protein
OG_02580	LA76x_689	LA76x_689; cheW-like domain protein
OG_02581	LA76x_3698	LA76x_3698; hypothetical protein
OG_02582	LA76x_637	LA76x_637; peptidase M20/M25/M40 family protein
OG_02583	LA76x_3699	LA76x_3699; tRNA pseudouridine synthase, TruD family protein
OG_02584	LA76x_170	LA76x_170; conserved hypothetical protein
OG_02585	LA76x_5190	LA76x_5190; ABC transporter family protein
OG_02586	LA76x_103	LA76x_103; DEAD/DEAH box helicase family protein
OG_02587	LA76x_4110	LA76x_4110; 5'-nucleotidase
OG_02588	LA76x_4114	LA76x_4114; conserved hypothetical protein
OG_02589	LA76x_3605	LA76x_3605; hypothetical protein
OG_02590	LA76x_1772	LA76x_1772; conserved hypothetical protein
OG_02591	LA76x_3889	LA76x_3889; glutaredoxin family protein
OG_02592	LA76x_349	LA76x_349; short chain dehydrogenase family protein
OG_02593	LA76x_2313	LA76x_2313; conserved hypothetical protein
OG_02594	LA76x_2301	LA76x_2301; efflux transporter, RND family, MFP subunit
OG_02595	LA76x_4890	LA76x_4890; glucan biosynthesis protein D
OG_02596	LA76x_4331	LA76x_4331; interferon-induced transmembrane family protein
OG_02597	LA76x_4118	LA76x_4118; conserved hypothetical protein
OG_02598	LA76x_4379	glmU; UDP-N-acetylglucosamine diphosphorylase/glucosamine-1-pho
OG_02599	LA76x_314	LA76x_314; response regulator
OG_02600	LA76x_346	LA76x_346; MATE efflux family protein
OG_02601	LA76x_864	LA76x_864; conserved hypothetical protein
OG_02602	LA76x_1643	LA76x_1643; small GTP-binding domain protein
OG_02603	LA76x_2527	gdhB; NAD-specific glutamate dehydrogenase
OG_02604	LA76x_3959	hemA; glutamyl-tRNA reductase
OG_02605	LA76x_3469	LA76x_3469; L,D-transpeptidase catalytic domain protein
OG_02606	LA76x_3810	LA76x_3810; hypothetical protein
OG_02607	LA76x_1818	LA76x_1818; phosphate transporter family protein
OG_02608	LA76x_1512	LA76x_1512; tat (twin-arginine translocation) pathway signal sequenc
OG_02609	LA76x_2025	LA76x_2025; sigma-54 interaction domain protein
OG_02610	LA76x_688	LA76x_688; methyl-accepting chemotaxis (MCP) signalling domain p
OG_02611	LA76x_3872	LA76x_3872; NADH:flavin oxidoreductase / NADH oxidase family pro
OG_02612	LA76x_4630	LA76x_4630; hypothetical protein
OG_02613	LA76x_5176	LA76x_5176; his Kinase A domain protein
OG_02614	LA76x_1624	LA76x_1624; SET domain protein
OG_02615	LA76x_119	HRSP12; ribonuclease UK114 domain protein
OG_02616	LA76x_2530	LA76x_2530; amino acid permease family protein

OG_02617	LA29479_1460	K+-transporting ATPase, B subunit
OG_02618	LA29479_1697	endonuclease/Exonuclease/phosphatase family protein
OG_02619	LA29479_2195	acetyltransferase family protein
OG_02620	LA29479_1763	DNA repair protein RecN
OG_02621	LA29479_0568	phoH-like family protein
OG_02623	LA29479_1671	lytTr DNA-binding domain protein
OG_02624	LA29479_2333	putative uncharacterized protein
OG_02625	LA29479_4162	type IV pilus assembly PilM family protein
OG_02626	LA29479_4872	molybdopterin converting factor, subunit 1
OG_02627	LA29479_1034	tonB-dependent Receptor Plug domain protein
OG_02628	LA29479_2192	penicillin binding transpeptidase domain protein
OG_02629	LA29479_0486	glutamine-fructose-6-phosphate transaminase
OG_02630	LA29479_2281	NUDIX domain protein
OG_02631	LA29479_2410	3-hydroxyisobutyrate dehydrogenase
OG_02632	LA29479_3094	fatty acid desaturase family protein
OG_02633	LA29479_3770	putative uncharacterized protein
OG_02634	LA29479_4169	inosine-uridine preferring nucleoside hydrolase family protein
OG_02635	LA29479_1527	putative uncharacterized TTHA1943 domain protein
OG_02636	LA29479_1595	arsenate reductase
OG_02637	LA29479_0737	lactonizing lipase
OG_02638	LA29479_2816	ribosomal protein S1
OG_02639	LA29479_4991	uroporphyrinogen-III synthase
OG_02640	LA29479_1621	CDP-diacylglycerol--glycerol-3-phosphate 3-phosphatidyltransferase
OG_02642	LA29479_0962	glyoxalase/Bleomycin resistance /Dioxygenase superfamily protein
OG_02643	LA29479_2218	putative exported protein
OG_02644	LA29479_2416	low molecular weight protein-tyrosine-phosphatase wzb
OG_02645	LA29479_0957	zinc-binding dehydrogenase family protein
OG_02646	LA29479_2090	fumarylacetoacetate (FAA) hydrolase family protein
OG_02647	LA29479_4020	conserved hypothetical protein
OG_02648	LA29479_0712	HPr Serine kinase N terminus family protein
OG_02649	LA29479_1299	putative phospholipid ABC transporter permease protein mlaE
OG_02650	LA29479_2661	ompA family protein
OG_02651	LA29479_3936	HAMP domain protein
OG_02652	LA29479_3129	glucose / Sorbosone dehydrogenase family protein
OG_02653	LA29479_0279	isocitrate dehydrogenase kinase/phosphatase family protein
OG_02654	LA29479_3277	leupeptin-inactivating enzyme 1
OG_02655	LA29479_3034	C-terminal processing peptidase family protein
OG_02656	LA29479_2062	3-oxoacid CoA-transferase, A subunit
OG_02657	LA29479_2778	putative uncharacterized protein
OG_02658	LA29479_1547	RNA polymerase sigma factor, sigma-70 family protein
OG_02659	LA29479_3390	ribosomal S14p/S29e family protein
OG_02660	LA29479_0449	biotin synthase
OG_02661	LA29479_1031	ROK family protein
OG_02662	LA29479_4203	5-formyltetrahydrofolate cyclo-ligase
OG_02663	LA29479_0189	tRNA pseudouridine synthase A
OG_02664	LA29479_1942	protein TolR
OG_02665	LA29479_4520	UDP-3-O-[3-hydroxymyristoyl] glucosamine N-acyltransferase
OG_02666	LA29479_4086	oligopeptide transport system permease protein oppC
OG_02667	LA29479_0990	conserved hypothetical protein
OG_02668	LA29479_1427	putative secreted protein
OG_02669	LA29479_3033	lipoic acid synthetase
OG_02670	LA29479_0903	intestinal-type alkaline phosphatase
OG_02671	LA29479_2422	polysaccharide biosynthesis family protein
OG_02672	LA29479_4038	ankyrin repeat family protein
OG_02673	LA29479_0742	pyridoxine/pyridoxamine 5'-phosphate oxidase
OG_02674	LA29479_4152	permease family protein
OG_02675	LA29479_4115	putative uncharacterized protein
OG_02676	LA29479_3378	ribosomal protein L17
OG_02677	LA29479_0683	macro domain protein
OG_02678	LA29479_3656	calcineurin-like phosphoesterase family protein
OG_02679	LA29479_1737	putative uncharacterized protein
OG_02680	LA29479_4253	phenylalanyl-tRNA synthetase, alpha subunit
OG_02681	LA29479_3776	putative uncharacterized protein orf5
OG_02682	LA29479_4308	general secretion pathway protein G
OG_02683	LA29479_0131	conserved hypothetical protein
OG_02684	LA29479_0765	4-hydroxybenzoyl-CoA reductase subunit gamma
OG_02685	LA29479_4354	aspartyl/Asparaginyl beta-hydroxylase family protein
OG_02686	LA29479_1155	dihydrolipoyl dehydrogenase
OG_02687	LA29479_3583	putative membrane protein
OG_02688	LA29479_1408	putative uncharacterized protein
OG_02689	LA29479_3786	putative transcriptional regulator, ArsR family protein
OG_02690	LA29479_1668	soluble aldose sugar dehydrogenase yliI
OG_02691	LA29479_4571	conserved hypothetical protein
OG_02692	LA29479_1873	bacterial regulatory helix-turn-helix , lysR family protein
OG_02693	LA29479_2267	putative membrane protein
OG_02694	LA29479_3128	transglycosylase SLT domain protein
OG_02695	LA29479_1579	methionine aminopeptidase, type I
OG_02696	LA29479_0478	pyruvate ferredoxin/flavodoxin oxidoreductase family protein
OG_02697	LA29479_1140	putative lipoprotein
OG_02698	LA29479_0693	hypothetical protein
OG_02699	LA29479_2753	putative membrane protein

OG_02617	LA76x_2045	kdpB; K+-transporting ATPase, B subunit
OG_02618	LA76x_2135	LA76x_2135; endonuclease/Exonuclease/phosphatase family protein
OG_02619	LA76x_962	LA76x_962; acetyltransferase family protein
OG_02620	LA76x_2866	recN; DNA repair protein RecN
OG_02621	LA76x_1695	LA76x_1695; putative protein with nucleoside triphosphate hydrolase
OG_02623	LA76x_2162	LA76x_2162; lytTr DNA-binding domain protein
OG_02624	LA76x_4397	LA76x_4397; conserved hypothetical protein
OG_02625	LA76x_4156	LA76x_4156; type IV pilus assembly PilM family protein
OG_02626	LA76x_3531	moaD; molybdopterin converting factor, subunit 1
OG_02627	LA76x_2619	LA76x_2619; tonB dependent receptor family protein
OG_02628	LA76x_964	LA76x_964; penicillin binding transpeptidase domain protein
OG_02629	LA76x_4872	LA76x_4872; glutamine-fructose-6-phosphate transaminase
OG_02630	LA76x_4345	LA76x_4345; NUDIX domain protein
OG_02631	LA76x_3380	mmsB; 3-hydroxyisobutyrate dehydrogenase
OG_02632	LA76x_905	LA76x_905; fatty acid desaturase family protein
OG_02633	LA76x_4795	LA76x_4795; conserved hypothetical protein
OG_02634	LA76x_4163	LA76x_4163; inosine-uridine preferring nucleoside hydrolase family p
OG_02635	LA76x_1976	LA76x_1976; conserved hypothetical protein
OG_02636	LA76x_2239	arsC; arsenate reductase
OG_02637	LA76x_1539	lipL; lactonizing lipase
OG_02638	LA76x_2318	rpsA; ribosomal protein S1
OG_02639	LA76x_4726	LA76x_4726; uroporphyrinogen-III synthase HemD family protein
OG_02640	LA76x_2213	pgsA; CDP-diacylglycerol--glycerol-3-phosphate 3-phosphatidyltransf
OG_02642	LA76x_3805	LA76x_3805; glyoxalase/Bleomycin resistance /Dioxygenase superfa
OG_02643	LA76x_4285	LA76x_4285; alpha/beta hydrolase family protein
OG_02644	LA76x_3386	LA76x_3386; putative acid phosphatase Wzb
OG_02645	LA76x_3800	LA76x_3800; zinc-binding dehydrogenase family protein
OG_02646	LA76x_1062	LA76x_1062; fumarylacetoacetate (FAA) hydrolase family protein
OG_02647	LA76x_4607	LA76x_4607; conserved hypothetical protein
OG_02648	LA76x_1563	LA76x_1563; HPr Serine kinase N terminus family protein
OG_02649	LA76x_74	yrbE; mlaE
OG_02650	LA76x_585	LA76x_585; ompA family protein
OG_02651	LA76x_767	LA76x_767; histidine kinase-, DNA gyrase B-, and HSP90-like ATPas
OG_02652	LA76x_941	LA76x_941; glucose / Sorbosone dehydrogenase family protein
OG_02653	LA76x_5165	LA76x_5165; isocitrate dehydrogenase kinase/phosphatase family pr
OG_02654	LA76x_203	lieA; leupeptin-inactivating enzyme 1 domain protein
OG_02655	LA76x_850	LA76x_850; C-terminal processing peptidase family protein
OG_02656	LA76x_1090	LA76x_1090; 3-oxoacid CoA-transferase, A subunit
OG_02657	LA76x_2355	LA76x_2355; conserved hypothetical protein
OG_02658	LA76x_1957	LA76x_1957; RNA polymerase sigma factor, sigma-70 family protein
OG_02659	LA76x_3915	LA76x_3915; ribosomal S14p/S29e family protein
OG_02660	LA76x_4910	bioB; biotin synthase
OG_02661	LA76x_2616	LA76x_2616; ROK family protein
OG_02662	LA76x_4194	LA76x_4194; 5-formyltetrahydrofolate cyclo-ligase
OG_02663	LA76x_3001	truA; tRNA pseudouridine(38-40) synthase
OG_02664	LA76x_1257	tolR; protein TolR
OG_02665	LA76x_1914	lpxD; UDP-3-O-[3-hydroxymyristoyl] glucosamine N-acyltransferase
OG_02666	LA76x_245	LA76x_245; binding-protein-dependent transport system inner memb
OG_02667	LA76x_3833	LA76x_3833; conserved hypothetical protein
OG_02668	LA76x_2077	LA76x_2077; conserved hypothetical protein
OG_02669	LA76x_849	lipA; lipoyl synthase
OG_02670	LA76x_3743	Akp3; alkaline phosphatase 3, intestine, not Mn requiring
OG_02671	LA76x_3391	LA76x_3391; matE family protein
OG_02672	LA76x_290	LA76x_290; ankyrin repeat family protein
OG_02673	LA76x_1533	pdxH; pyridoxamine 5'-phosphate oxidase
OG_02674	LA76x_302	LA76x_302; ftsX-like permease family protein
OG_02675	LA76x_339	LA76x_339; conserved hypothetical protein
OG_02676	LA76x_3903	rplQ; ribosomal protein L17
OG_02677	LA76x_1590	LA76x_1590; macro domain protein
OG_02678	LA76x_3220	LA76x_3220; calcineurin-like phosphoesterase family protein
OG_02679	LA76x_2920	LA76x_2920; conserved hypothetical protein
OG_02680	LA76x_1749	pheS; phenylalanine--tRNA ligase, alpha subunit
OG_02681	LA76x_4789	LA76x_4789; conserved hypothetical protein
OG_02682	LA76x_1127	gspG; type II secretion system protein G
OG_02683	LA76x_3056	LA76x_3056; conserved hypothetical protein
OG_02684	LA76x_1511	hcrC; 4-hydroxybenzoyl-CoA reductase subunit gamma
OG_02685	LA76x_1483	LA76x_1483; aspartyl/Asparaginyl beta-hydroxylase family protein
OG_02686	LA76x_2489	lpdA; dihydrolipoyl dehydrogenase
OG_02687	LA76x_3991	LA76x_3991; conserved hypothetical protein
OG_02688	LA76x_2096	LA76x_2096; conserved hypothetical protein
OG_02689	LA76x_4778	LA76x_4778; putative transcriptional regulator, ArsR family protein
OG_02690	LA76x_2167	yliI; aldose sugar dehydrogenase
OG_02691	LA76x_1429	LA76x_1429; conserved hypothetical protein
OG_02692	LA76x_2757	LA76x_2757; bacterial regulatory helix-turn-helix , lysR family protein
OG_02693	LA76x_4332	LA76x_4332; conserved hypothetical protein
OG_02694	LA76x_940	LA76x_940; transglycosylase SLT domain protein
OG_02695	LA76x_1929	map; methionine aminopeptidase, type I
OG_02696	LA76x_4880	LA76x_4880; thiamine pyrophosphate enzyme, C-terminal TPP bindi
OG_02697	LA76x_2504	LA76x_2504; hypothetical protein
OG_02698	LA76x_1581	LA76x_1581; conserved hypothetical protein
OG_02699	LA76x_2381	LA76x_2381; conserved hypothetical protein

OG_02700	LA29479_3846	lysM domain protein
OG_02701	LA29479_0936	peptidase M1 family protein
OG_02702	LA29479_3185	putative membrane protein
OG_02703	LA29479_1991	putative membrane protein
OG_02704	LA29479_1119	acyl-CoA dehydrogenase, N-terminal domain protein
OG_02705	LA29479_2578	exported amidohydrolase
OG_02706	LA29479_1481	putative uncharacterized protein
OG_02707	LA29479_2242	putative uncharacterized protein
OG_02708	LA29479_0580	conserved hypothetical protein
OG_02709	LA29479_1926	pyruvate kinase
OG_02710	LA29479_4111	putative secreted protein
OG_02711	LA29479_1631	DNA internalization-related competence protein ComEC/Rec2
OG_02712	LA29479_0628	sodium Bile acid symporter family protein
OG_02713	LA29479_4027	putative uncharacterized protein
OG_02714	LA29479_1630	motA/TolQ/ExbB proton channel family protein
OG_02715	LA29479_2729	type IV secretion system protein virB4
OG_02716	LA29479_0221	transcription elongation protein nusA
OG_02717	LA29479_2501	hypothetical protein
OG_02718	LA29479_4831	3-demethylubiquinone-9 3-O-methyltransferase
OG_02719	LA29479_5203	peptidyl-tRNA hydrolase
OG_02720	LA29479_0157	putative membrane protein
OG_02721	LA29479_2484	conserved hypothetical protein
OG_02722	LA29479_3965	hypothetical protein
OG_02723	LA29479_0493	2-dehydro-3-deoxyphosphogluconate aldolase/4-hydroxy-2-oxoglutar
OG_02724	LA29479_4539	conserved hypothetical protein
OG_02725	LA29479_3395	ribosomal protein L29
OG_02726	LA29479_4486	peptidyl-tRNA hydrolase domain protein
OG_02727	LA29479_4978	D-tyrosyl-tRNA(Tyr) deacylase
OG_02728	LA29479_3072	bacterial regulatory helix-turn-helix s, AraC family protein
OG_02729	LA29479_5018	conserved hypothetical protein
OG_02730	LA29479_0745	shikimate kinase family protein
OG_02731	LA29479_3223	queuine tRNA-ribosyltransferase
OG_02732	LA29479_1515	riboflavin biosynthesis protein RibF
OG_02733	LA29479_1124	hydrolase
OG_02734	LA29479_3906	putative uncharacterized protein
OG_02735	LA29479_1848	hypothetical protein
OG_02736	LA29479_1978	threonine-phosphate decarboxylase
OG_02737	LA29479_3302	tetratricopeptide repeat family protein
OG_02738	LA29479_1496	multidrug resistance protein B
OG_02739	LA29479_5225	thioredoxin
OG_02740	LA29479_1451	ACT domain protein
OG_02741	LA29479_2557	bacterial regulatory helix-turn-helix , lysR family protein
OG_02742	LA29479_1838	putative uncharacterized protein
OG_02743	LA29479_3913	response regulator
OG_02744	LA29479_1700	2'-5' RNA ligase
OG_02745	LA29479_4041	bacterial Cytochrome Ubiquinol Oxidase family protein
OG_02746	LA29479_3771	conserved hypothetical protein
OG_02747	LA29479_3665	NUDIX domain protein
OG_02748	LA29479_3460	homoserine kinase
OG_02749	LA29479_1753	hypothetical protein
OG_02750	LA29479_2903	efflux transporter, RND family, MFP subunit
OG_02751	LA29479_4785	outer-membrane channel-forming protein VI
OG_02752	LA29479_0548	cytochrome b(C-terminal)/b6/petD family protein
OG_02753	LA29479_0297	aldo/keto reductase family protein
OG_02754	LA29479_2196	putative uncharacterized protein
OG_02755	LA29479_4294	methionine aminopeptidase, type I
OG_02756	LA29479_0476	transmembrane regulator PrtR
OG_02757	LA29479_4806	argininosuccinate lyase
OG_02758	LA29479_5186	his Kinase A domain protein
OG_02759	LA29479_2037	penicillin-binding protein 3
OG_02760	LA29479_1859	outer membrane lipocarrier protein LolA
OG_02761	LA29479_1414	biotin-dependent carboxylase uncharacterized domain protein
OG_02762	LA29479_1428	methionyl-tRNA synthetase
OG_02763	LA29479_0358	putative uncharacterized protein
OG_02764	LA29479_4426	chitinase D
OG_02765	LA29479_2212	glutamate-ammonia ligase adenylyltransferase family protein
OG_02766	LA29479_1603	hypothetical protein
OG_02767	LA29479_4689	response regulator
OG_02768	LA29479_0137	phage Tail Collar domain protein
OG_02769	LA29479_2027	cell division protein FtsA
OG_02770	LA29479_4299	putative uncharacterized protein
OG_02771	LA29479_1094	carbonic anhydrase family protein
OG_02772	LA29479_4458	uncharacterised family protein
OG_02773	LA29479_3124	NADPH-dependent FMN reductase family protein
OG_02774	LA29479_0084	lipase family protein
OG_02775	LA29479_0497	hypothetical protein
OG_02776	LA29479_4128	tat (twin-arginine translocation) pathway signal sequence domain prot
OG_02777	LA29479_0363	response regulator
OG_02778	LA29479_3434	UDP-N-acetylmuramate:L-alanyl-gamma-D-glutamyl- meso-diaminop
OG_02779	LA29479_4736	pterin 4 alpha carbinolamine dehydratase family protein
OG_02780	LA29479_2346	putative uncharacterized protein

OG_02700	LA76x_3546	LA76x_3546; lysM domain protein
OG_02701	LA76x_3777	LA76x_3777; peptidase M1 family protein
OG_02702	LA76x_1838	LA76x_1838; peptidase M1 family protein
OG_02703	LA76x_1211	LA76x_1211; putative transmembrane protein
OG_02704	LA76x_2525	LA76x_2525; acyl-CoA dehydrogenase, N-terminal domain protein
OG_02705	LA76x_4529	LA76x_4529; amidohydrolase family protein
OG_02706	LA76x_2022	LA76x_2022; conserved hypothetical protein
OG_02707	LA76x_4309	LA76x_4309; conserved hypothetical protein
OG_02708	LA76x_1686	LA76x_1686; conserved hypothetical protein
OG_02709	LA76x_1366	pyk; pyruvate kinase
OG_02710	LA76x_342	LA76x_342; conserved hypothetical protein
OG_02711	LA76x_2204	LA76x_2204; DNA internalization-related competence protein ComEC
OG_02712	LA76x_1647	LA76x_1647; sodium Bile acid symporter family protein
OG_02713	LA76x_4615	LA76x_4615; biofilm formation and stress response factor family prote
OG_02714	LA76x_2205	LA76x_2205; motA/TolQ/ExbB proton channel family protein
OG_02715	LA76x_2406	virB4; type IV secretion system protein virB4
OG_02716	LA76x_2969	nusA; transcription termination/antitermination L factor
OG_02717	LA76x_4456	LA76x_4456; HEAT repeats family protein
OG_02718	LA76x_3294	ubiG; 3-demethylubiquinone-9 3-O-methyltransferase
OG_02719	LA76x_3951	pth; peptidyl-tRNA hydrolase
OG_02720	LA76x_3031	LA76x_3031; hypothetical protein
OG_02721	LA76x_4437	LA76x_4437; eamA-like transporter family protein
OG_02722	LA76x_797	LA76x_797; conserved hypothetical protein
OG_02723	LA76x_4865	eda; 2-dehydro-3-deoxyphosphogluconate aldolase/4-hydroxy-2-oxog
OG_02724	LA76x_1895	LA76x_1895; conserved hypothetical protein
OG_02725	LA76x_3920	rpmC; ribosomal protein L29
OG_02726	LA76x_4673	LA76x_4673; RF-1 domain protein
OG_02727	LA76x_374	dtd; D-tyrosyl-tRNA(Tyr) deacylase
OG_02728	LA76x_886	LA76x_886; bacterial regulatory helix-turn-helix, AraC family protein
OG_02729	LA76x_4546	LA76x_4546; eamA-like transporter family protein
OG_02730	LA76x_1530	LA76x_1530; shikimate kinase family protein
OG_02731	LA76x_1803	tgt; queuine tRNA-ribosyltransferase
OG_02732	LA76x_1989	ribF; riboflavin biosynthesis protein RibF
OG_02733	LA76x_2520	LA76x_2520; alpha/beta hydrolase fold family protein
OG_02734	LA76x_3606	LA76x_3606; conserved hypothetical protein
OG_02735	LA76x_2782	LA76x_2782; hypothetical protein
OG_02736	LA76x_1223	LA76x_1223; threonine-phosphate decarboxylase
OG_02737	LA76x_182	LA76x_182; TPR repeat family protein
OG_02738	LA76x_2005	LA76x_2005; H+ antiporter-2 family protein
OG_02739	LA76x_298	trxA; thioredoxin
OG_02740	LA76x_2055	LA76x_2055; ACT domain protein
OG_02741	LA76x_4509	LA76x_4509; bacterial regulatory helix-turn-helix , lysR family protein
OG_02742	LA76x_2792	LA76x_2792; conserved hypothetical protein
OG_02743	LA76x_3612	LA76x_3612; bacterial regulatory, luxR family protein
OG_02744	LA76x_2132	LA76x_2132; 2'-5' RNA ligase
OG_02745	LA76x_287	LA76x_287; bacterial Cytochrome Ubiquinol Oxidase family protein
OG_02746	LA76x_4794	LA76x_4794; type III secretion apparatus lipoprotein, YscJ/HrcJ famil
OG_02747	LA76x_3209	LA76x_3209; NUDIX domain protein
OG_02748	LA76x_4245	thrB; homoserine kinase
OG_02749	LA76x_2876	LA76x_2876; bacteriophage CI repressor helix-turn-helix domain prot
OG_02750	LA76x_705	LA76x_705; efflux transporter, RND family, MFP subunit
OG_02751	LA76x_4131	LA76x_4131; outer-membrane channel-forming protein VI
OG_02752	LA76x_1718	LA76x_1718; cytochrome b(C-terminal)/b6/petD family protein
OG_02753	LA76x_5147	LA76x_5147; aldo/keto reductase family protein
OG_02754	LA76x_961	LA76x_961; conserved hypothetical protein
OG_02755	LA76x_1141	map; methionine aminopeptidase, type I
OG_02756	LA76x_4882	LA76x_4882; putative transmembrane regulator PrtR
OG_02757	LA76x_3269	argH; argininosuccinate lyase
OG_02758	LA76x_4801	LA76x_4801; his Kinase A domain protein
OG_02759	LA76x_1168	LA76x_1168; penicillin binding transpeptidase domain protein
OG_02760	LA76x_2771	lolA; outer membrane lipocarrier protein LolA
OG_02761	LA76x_2090	LA76x_2090; conserved hypothetical protein
OG_02762	LA76x_2076	metG; methionyl-tRNA synthetase
OG_02763	LA76x_5086	LA76x_5086; EF-hand domain protein
OG_02764	LA76x_490	chiD; chitinase D
OG_02765	LA76x_4278	LA76x_4278; glnD PII-uridylyltransferase family protein
OG_02766	LA76x_2231	LA76x_2231; hypothetical protein
OG_02767	LA76x_4074	LA76x_4074; response regulator
OG_02768	LA76x_3050	LA76x_3050; phage Tail Collar domain protein
OG_02769	LA76x_1178	ftsA; cell division protein FtsA
OG_02770	LA76x_1136	LA76x_1136; conserved hypothetical protein
OG_02771	LA76x_2547	LA76x_2547; carbonic anhydrase family protein
OG_02772	LA76x_4624	anmK; anhydro-N-acetylmuramic acid kinase
OG_02773	LA76x_936	LA76x_936; NADPH-dependent FMN reductase family protein
OG_02774	LA76x_3104	LA76x_3104; lipase family protein
OG_02775	LA76x_4862	LA76x_4862; hypothetical protein
OG_02776	LA76x_326	LA76x_326; alpha/beta hydrolase fold family protein
OG_02777	LA76x_5081	LA76x_5081; transcriptional regulatory , C terminal family protein
OG_02778	LA76x_4218	mpl; L-alanyl-gamma-D-glutamyl-meso-diaminopimelate ligase
OG_02779	LA76x_2295	LA76x_2295; pterin 4 alpha carbinolamine dehydratase family protein
OG_02780	LA76x_4410	LA76x_4410; conserved hypothetical protein

OG_02781	LA29479_3431	serine/threonine protein kinase
OG_02782	LA29479_1358	hypothetical protein
OG_02783	LA29479_1411	dienelactone hydrolase family protein
OG_02784	LA29479_4148	bacterial regulatory helix-turn-helix , lysR family protein
OG_02785	LA29479_0132	hypothetical protein
OG_02786	LA29479_2485	tat (twin-arginine translocation) pathway signal sequence domain prot
OG_02787	LA29479_3180	diguanylate cyclase domain protein
OG_02788	LA29479_3689	conserved hypothetical protein
OG_02789	LA29479_1874	acyl-CoA dehydrogenase, middle domain protein
OG_02790	LA29479_4731	GTP-binding protein LepA
OG_02791	LA29479_2482	RNA-splicing ligase RtcB
OG_02792	LA29479_4167	citrate (Si)-synthase
OG_02793	LA29479_4165	putative uncharacterized protein
OG_02794	LA29479_1420	pyroglutamyl-peptidase I
OG_02795	LA29479_2052	ABC transporter family protein
OG_02796	LA29479_3593	alanine dehydrogenase/pyridine nucleotide transhydrogenase
OG_02797	LA29479_4272	short chain dehydrogenase family protein
OG_02798	LA29479_3578	conserved hypothetical protein
OG_02799	LA29479_1566	transporter, monovalent cation:proton antiporter-2 family protein
OG_02800	LA29479_1495	hlyD secretion family protein
OG_02801	LA29479_3837	putative uncharacterized protein
OG_02802	LA29479_1502	DNA repair protein RadA
OG_02803	LA29479_1093	aldehyde dehydrogenase family 8 member A1
OG_02804	LA29479_4511	blaR1 peptidase M56 family protein
OG_02805	LA29479_0314	pspA/IM30 family protein
OG_02806	LA29479_2763	poly(R)-hydroxyalkanoic acid synthase, class III, PhaE subunit
OG_02807	LA29479_2354	hypothetical protein
OG_02808	LA29479_1232	conserved hypothetical protein
OG_02809	LA29479_3619	sugar (and other) transporter family protein
OG_02810	LA29479_4938	alpha/beta hydrolase fold family protein
OG_02811	LA29479_1245	putative uncharacterized protein
OG_02812	LA29479_1669	putative uncharacterized protein
OG_02813	LA29479_5042	putative transmembrane protein
OG_02814	LA29479_1338	hypothetical protein
OG_02815	LA29479_5142	poly(hydroxyalkanoate) granule-associated domain protein
OG_02816	LA29479_0653	feS assembly protein SufB
OG_02817	LA29479_5035	3-oxoacyl-synthase III
OG_02818	LA29479_0871	hypothetical protein
OG_02819	LA29479_1434	N-acetylmuramoyl-L-alanine amidase family protein
OG_02820	LA29479_4188	radical SAM superfamily protein
OG_02821	LA29479_0206	NADH (or F420H2) dehydrogenase, subunit C family protein
OG_02822	LA29479_0757	ycel-like domain protein
OG_02823	LA29479_3535	SURF1 family protein
OG_02824	LA29479_4058	putative disulfide bond reductase yfcG
OG_02825	LA29479_0173	lysM domain protein
OG_02826	LA29479_4922	conserved hypothetical protein
OG_02827	LA29479_4965	diacylglycerol kinase
OG_02828	LA29479_3820	methyltransferase domain protein
OG_02829	LA29479_3270	short chain dehydrogenase family protein
OG_02830	LA29479_3688	putative uncharacterized protein
OG_02831	LA29479_1314	peptidase M28 family protein
OG_02832	LA29479_4912	transglutaminase-like superfamily protein
OG_02833	LA29479_4682	RNA methyltransferase family protein
OG_02834	LA29479_2231	divalent-cation tolerance protein CutA
OG_02835	LA29479_0972	phosphodiesterase-nucleotide pyrophosphatase
OG_02836	LA29479_0891	NADPH-dependent FMN reductase family protein
OG_02837	LA29479_4124	cupin domain protein
OG_02838	LA29479_0673	sulfite reductase [NADPH] flavoprotein, alpha-component
OG_02839	LA29479_0699	putative uncharacterized protein XOO1194
OG_02840	LA29479_0855	protein-L-isoaspartate O-methyltransferase
OG_02841	LA29479_0979	putative uncharacterized protein
OG_02842	LA29479_3920	D-alanyl-D-alanine dipeptidase
OG_02843	LA29479_0133	putative uncharacterized protein
OG_02844	LA29479_0460	putative lipoprotein
OG_02845	LA29479_3336	major Facilitator Superfamily protein
OG_02846	LA29479_0048	putative uncharacterized protein
OG_02847	LA29479_2776	oligoribonuclease
OG_02848	LA29479_0260	putative uncharacterized protein
OG_02849	LA29479_4538	conserved hypothetical protein
OG_02850	LA29479_4985	O-Antigen Polymerase family protein
OG_02851	LA29479_5118	poly-beta-1,6-N-acetyl-D-glucosamine synthase
OG_02852	LA29479_3643	histidine ammonia-lyase
OG_02853	LA29479_2250	uncharacterized protein ynbC
OG_02854	LA29479_3537	cytochrome o ubiquinol oxidase, subunit III
OG_02855	LA29479_1525	excinuclease ABC, A subunit
OG_02856	LA29479_1099	kynurenine 3-monooxygenase
OG_02857	LA29479_2591	transcriptional repressor frmR
OG_02858	LA29479_2038	cell division protein FtsL
OG_02859	LA29479_3211	exopolysaccharide synthesis, ExoD family protein
OG_02860	LA29479_0304	efflux transporter, outer membrane factor (OMF) lipo, NodT family pro
OG_02861	LA29479_1069	ribosomal protein L9

OG_02781	LA76x_4215	LA76x_4215; phosphotransferase enzyme family protein
OG_02782	LA76x_14	LA76x_14; hypothetical protein
OG_02783	LA76x_2093	LA76x_2093; dienelactone hydrolase family protein
OG_02784	LA76x_306	LA76x_306; bacterial regulatory helix-turn-helix , lysR family protein
OG_02785	LA76x_3055	LA76x_3055; hypothetical protein
OG_02786	LA76x_4438	LA76x_4438; bacterial regulatory, gntR family protein
OG_02787	LA76x_1842	LA76x_1842; diguanylate cyclase domain protein
OG_02788	LA76x_3189	LA76x_3189; polymer-forming cytoskeletal family protein
OG_02789	LA76x_2756	LA76x_2756; acyl-CoA dehydrogenase, N-terminal domain protein
OG_02790	LA76x_3487	lepA; GTP-binding protein LepA
OG_02791	LA76x_4435	rtcB; conserved protein
OG_02792	LA76x_4161	gltA; citrate (Si)-synthase
OG_02793	LA76x_4159	LA76x_4159; conserved hypothetical protein
OG_02794	LA76x_2084	pcp; pyroglutamyl-peptidase I
OG_02795	LA76x_1100	LA76x_1100; ABC transporter family protein
OG_02796	LA76x_4002	LA76x_4002; putative NAD
OG_02797	LA76x_1730	LA76x_1730; short chain dehydrogenase family protein
OG_02798	LA76x_3986	LA76x_3986; conserved hypothetical protein
OG_02799	LA76x_1941	LA76x_1941; proton antiporter-2 family protein
OG_02800	LA76x_2007	LA76x_2007; efflux transporter, RND family, MFP subunit
OG_02801	LA76x_4733	LA76x_4733; conserved hypothetical protein
OG_02802	LA76x_1999	radA; DNA repair protein RadA
OG_02803	LA76x_2548	ALDH8A1; aldehyde dehydrogenase family 8 member A1
OG_02804	LA76x_1923	LA76x_1923; blaR1 peptidase M56 family protein
OG_02805	LA76x_5130	LA76x_5130; pspA/IM30 family protein
OG_02806	LA76x_2371	phaE; poly(R)-hydroxyalkanoic acid synthase, class III, PhaE subunit
OG_02807	LA76x_4418	LA76x_4418; hypothetical protein
OG_02808	LA76x_136	LA76x_136; conserved hypothetical protein
OG_02809	LA76x_4028	LA76x_4028; sugar (and other) transporter family protein
OG_02810	LA76x_825	LA76x_825; dienelactone hydrolase family protein
OG_02811	LA76x_126	LA76x_126; conserved hypothetical protein
OG_02812	LA76x_2164	LA76x_2164; conserved hypothetical protein
OG_02813	LA76x_4698	LA76x_4698; putative transmembrane protein
OG_02814	LA76x_33	LA76x_33; hypothetical protein
OG_02815	LA76x_2249	LA76x_2249; poly(hydroxyalkanoate) granule-associated domain prot
OG_02816	LA76x_1622	sufB; feS assembly protein SufB
OG_02817	LA76x_4702	fabH; 3-oxoacyl-synthase III
OG_02818	LA76x_3710	LA76x_3710; hypothetical protein
OG_02819	LA76x_2070	LA76x_2070; N-acetylmuramoyl-L-alanine amidase family protein
OG_02820	LA76x_4181	LA76x_4181; radical SAM superfamily protein
OG_02821	LA76x_2984	nuoC; NADH (or F420H2) dehydrogenase, subunit C family protein
OG_02822	LA76x_1519	LA76x_1519; ycel-like domain protein
OG_02823	LA76x_2701	LA76x_2701; SURF1 family protein
OG_02824	LA76x_270	LA76x_270; disulfide bond reductase
OG_02825	LA76x_3017	LA76x_3017; lysM domain protein
OG_02826	LA76x_808	LA76x_808; conserved hypothetical protein
OG_02827	LA76x_4052	dgkA; dgkA
OG_02828	LA76x_4750	LA76x_4750; putative trans-aconitate 2-methyltransferase
OG_02829	LA76x_211	LA76x_211; polysaccharide biosynthesis family protein
OG_02830	LA76x_3190	LA76x_3190; conserved hypothetical protein
OG_02831	LA76x_58	LA76x_58; M42 glutamyl aminopeptidase family protein
OG_02832	LA76x_3511	LA76x_3511; transglutaminase-like superfamily protein
OG_02833	LA76x_4067	LA76x_4067; RNA methyltransferase, RsmE family protein
OG_02834	LA76x_4298	cutA; divalent-cation tolerance protein cutA
OG_02835	LA76x_3815	LA76x_3815; phosphodiesterase-nucleotide pyrophosphatase
OG_02836	LA76x_3730	LA76x_3730; NADPH-dependent FMN reductase family protein
OG_02837	LA76x_330	LA76x_330; cupin domain protein
OG_02838	LA76x_1601	LA76x_1601; sulfite reductase [NADPH] flavoprotein, alpha-compone
OG_02839	LA76x_1576	LA76x_1576; conserved hypothetical protein
OG_02840	LA76x_3694	pcm; protein-L-isoaspartate O-methyltransferase
OG_02841	LA76x_3823	LA76x_3823; outer membrane beta-barrel domain protein
OG_02842	LA76x_750	LA76x_750; D-ala-D-ala dipeptidase family protein
OG_02843	LA76x_3054	LA76x_3054; conserved hypothetical protein
OG_02844	LA76x_4899	LA76x_4899; hlyD secretion family protein
OG_02845	LA76x_3861	LA76x_3861; sugar (and other) transporter family protein
OG_02846	LA76x_3139	LA76x_3139; methyltransferase domain protein
OG_02847	LA76x_2357	orn; oligoribonuclease
OG_02848	LA76x_2928	LA76x_2928; conserved hypothetical protein
OG_02849	LA76x_1896	LA76x_1896; conserved hypothetical protein
OG_02850	LA76x_367	LA76x_367; O-Antigen ligase family protein
OG_02851	LA76x_3251	pgaC; poly-beta-1,6 N-acetyl-D-glucosamine synthase
OG_02852	LA76x_3233	LA76x_3233; aromatic amino acid lyase family protein
OG_02853	LA76x_4317	LA76x_4317; putative hydrolase
OG_02854	LA76x_2699	cyoC; cytochrome o ubiquinol oxidase, subunit III
OG_02855	LA76x_1978	uvrA; excinuclease ABC subunit A
OG_02856	LA76x_2542	kmo; kynurenine 3-monooxygenase
OG_02857	LA76x_4541	LA76x_4541; metal-sensitive transcriptional repressor family protein
OG_02858	LA76x_1167	ftsL; cell division protein FtsL
OG_02859	LA76x_1814	LA76x_1814; exopolysaccharide synthesis, ExoD family protein
OG_02860	LA76x_5139	LA76x_5139; efflux transporter, outer membrane factor (OMF) lipo, N
OG_02861	LA76x_2570	rplI; ribosomal protein L9

OG_02862	LA29479_4085	ABC transporter family protein
OG_02863	LA29479_4884	DNA polymerase III, epsilon subunit
OG_02864	LA29479_4219	ribosomal RNA small subunit methyltransferase D
OG_02865	LA29479_4351	tonB-dependent Receptor Plug domain protein
OG_02866	LA29479_1518	GTP-binding protein Obg/CgtA
OG_02867	LA29479_4677	putative uncharacterized domain protein
OG_02868	LA29479_4062	conserved hypothetical protein
OG_02869	LA29479_2590	S-(hydroxymethyl)glutathione dehydrogenase/class III alcohol dehydr
OG_02870	LA29479_1589	acyl-CoA-binding protein
OG_02871	LA29479_3866	sensor protein CreC
OG_02872	LA29479_3504	bacterial regulatory helix-turn-helix s, AraC family protein
OG_02873	LA29479_1179	putative uncharacterized protein
OG_02874	LA29479_1219	glycyl-tRNA synthetase, beta subunit
OG_02875	LA29479_1600	hypothetical protein
OG_02876	LA29479_4276	FAD linked oxidases, C-terminal domain protein
OG_02877	LA29479_5031	putative GTP-binding protein EngB
OG_02878	LA29479_5189	FHIPEP family protein
OG_02879	LA29479_0240	conserved hypothetical protein
OG_02880	LA29479_0148	beta-lactamase family protein
OG_02881	LA29479_0851	hypothetical protein
OG_02882	LA29479_2285	dethiobiotin synthase
OG_02883	LA29479_0929	bacterial regulatory helix-turn-helix , lysR family protein
OG_02884	LA29479_0951	bifunctional NMN adenylyltransferase/Nudix hydrolase
OG_02885	LA29479_2420	UDP-N-acetylglucosamine 2-epimerase
OG_02886	LA29479_3250	ATPase associated with various cellular activities family protein
OG_02887	LA29479_3629	polysaccharide deacetylase family protein
OG_02888	LA29479_4714	acyl-CoA dehydrogenase, middle domain protein
OG_02889	LA29479_3496	plasmid stabilization system protein
OG_02890	LA29479_4360	acyl-CoA dehydrogenase, C-terminal domain protein
OG_02891	LA29479_0114	uncharacterized oxidoreductase ydgJ
OG_02892	LA29479_3273	50S ribosomal protein L28
OG_02893	LA29479_1877	phosphoglycerate mutase family protein
OG_02894	LA29479_0520	glucokinase family protein
OG_02895	LA29479_4890	enoyl-CoA hydratase/isomerase family protein
OG_02896	LA29479_0125	glycosyl transferases group 1 family protein
OG_02897	LA29479_4939	putative uncharacterized protein
OG_02898	LA29479_4220	signal recognition particle-docking protein FtsY
OG_02899	LA29479_3035	methylated-DNA-[]-cysteine S-methyltransferase family protein
OG_02900	LA29479_3367	GTP-binding TypA domain protein
OG_02901	LA29479_3457	UPF0098 protein ybhB
OG_02902	LA29479_4515	di-trans,poly-cis-decaprenylcistransferase
OG_02903	LA29479_0710	ribosomal subunit interface protein
OG_02904	LA29479_4609	POTRA domain, ShlB-type family protein
OG_02905	LA29479_0359	doxX family protein
OG_02906	LA29479_5028	tonB-dependent Receptor Plug domain protein
OG_02907	LA29479_3952	tat (twin-arginine translocation) pathway signal sequence domain prot
OG_02908	LA29479_0837	putative membrane protein
OG_02909	LA29479_5034	putative secreted protein
OG_02910	LA29479_4704	aldehyde dehydrogenase family protein
OG_02911	LA29479_4357	RNA polymerase sigma factor, sigma-70 family protein
OG_02912	LA29479_0971	hypothetical protein
OG_02913	LA29479_5185	transcriptional regulatory protein fixJ
OG_02914	LA29479_4710	ABC transporter transmembrane region family protein
OG_02915	LA29479_3784	glycosyl transferases group 1 family protein
OG_02916	LA29479_3644	2OG-Fe(II) oxygenase superfamily protein
OG_02917	LA29479_3834	nitrogen regulation protein NR
OG_02918	LA29479_3234	uracil phosphoribosyltransferase
OG_02919	LA29479_3596	5'-3' exonuclease, C-terminal SAM fold family protein
OG_02920	LA29479_3075	beta-lactamase L2
OG_02921	LA29479_3096	bacterial regulatory helix-turn-helix , lysR family protein
OG_02922	LA29479_1691	rhomboid family protein
OG_02923	LA29479_4546	conserved hypothetical protein
OG_02924	LA29479_2080	ABC-2 type transporter family protein
OG_02925	LA29479_4172	tonB-dependent Receptor Plug domain protein
OG_02926	LA29479_3855	polyphosphate kinase family protein
OG_02927	LA29479_3948	hypothetical protein
OG_02928	LA29479_0508	putative uncharacterized protein
OG_02929	LA29479_2857	short-chain alcohol dehydrogenase family protein
OG_02930	LA29479_4537	putative membrane protein
OG_02931	LA29479_0574	putative membrane protein
OG_02932	LA29479_0863	2-C-methyl-D-erythritol 4-phosphate cytidylyltransferase
OG_02933	LA29479_4973	N-acylglucosamine 2-epimerase
OG_02934	LA29479_3260	exodeoxyribonuclease III
OG_02935	LA29479_2034	phospho-N-acetylmuramoyl-pentapeptide-transferas e
OG_02936	LA29479_0307	bacterial DNA-binding family protein
OG_02937	LA29479_1186	beta-phosphoglucomutase
OG_02938	LA29479_1469	protein RecA
OG_02939	LA29479_1275	ribonuclease
OG_02940	LA29479_0283	putative uncharacterized protein
OG_02941	LA29479_3815	peptidase S51 dipeptidase E
OG_02942	LA29479_2817	cytidylate kinase

OG_02862	LA76x_246	nikE; nickel import ATP-binding protein NikE
OG_02863	LA76x_3542	dnaQ; DNA polymerase III, epsilon subunit
OG_02864	LA76x_1784	LA76x_1784; RNA methyltransferase, RsmD family
OG_02865	LA76x_1484	LA76x_1484; tonB dependent receptor family protein
OG_02866	LA76x_1985	cgtA; obg family GTPase CgtA
OG_02867	LA76x_4062	LA76x_4062; conserved hypothetical protein
OG_02868	LA76x_267	LA76x_267; conserved hypothetical protein
OG_02869	LA76x_4540	LA76x_4540; S-(hydroxymethyl)glutathione dehydrogenase/class III a
OG_02870	LA76x_2246	DBI; acyl-CoA-binding protein
OG_02871	LA76x_3566	LA76x_3566; HAMP domain protein
OG_02872	LA76x_2732	LA76x_2732; bacterial regulatory helix-turn-helix, AraC family protein
OG_02873	LA76x_2468	LA76x_2468; conserved hypothetical protein
OG_02874	LA76x_148	glyS; glycine--tRNA ligase, beta subunit
OG_02875	LA76x_2234	LA76x_2234; hypothetical protein
OG_02876	LA76x_1159	LA76x_1159; FAD linked oxidase, C-terminal domain protein
OG_02877	LA76x_4559	ysxC; ribosome biogenesis GTP-binding protein YsxC
OG_02878	LA76x_4798	LA76x_4798; type III secretion , HrcV family protein
OG_02879	LA76x_2949	LA76x_2949; conserved hypothetical protein
OG_02880	LA76x_3039	LA76x_3039; beta-lactamase family protein
OG_02881	LA76x_3690	LA76x_3690; TOBE domain protein
OG_02882	LA76x_4349	bioD; dethiobiotin synthase
OG_02883	LA76x_3769	LA76x_3769; bacterial regulatory helix-turn-helix , lysR family protein
OG_02884	LA76x_3794	LA76x_3794; bifunctional NMN adenylyltransferase/Nudix hydrolase
OG_02885	LA76x_3389	LA76x_3389; UDP-N-acetylglucosamine 2-epimerase
OG_02886	LA76x_230	moxR2; magnesium chelatase, putative
OG_02887	LA76x_3240	LA76x_3240; polysaccharide deacetylase family protein
OG_02888	LA76x_3470	fadE8; acyl-CoA dehydrogenase, putative
OG_02889	LA76x_2740	LA76x_2740; putative plasmid stabilization system protein
OG_02890	LA76x_1477	LA76x_1477; acyl-CoA dehydrogenase, N-terminal domain protein
OG_02891	LA76x_3072	LA76x_3072; putative oxidoreductase
OG_02892	LA76x_207	rpmB; ribosomal protein L28
OG_02893	LA76x_2753	LA76x_2753; histidine phosphatase super family protein
OG_02894	LA76x_4841	LA76x_4841; glucokinase family protein
OG_02895	LA76x_3491	LA76x_3491; enoyl-CoA hydratase/isomerase family protein
OG_02896	LA76x_3061	LA76x_3061; glycosyl transferases group 1 family protein
OG_02897	LA76x_826	LA76x_826; conserved hypothetical protein
OG_02898	LA76x_1783	ftsY; signal recognition particle-docking protein FtsY
OG_02899	LA76x_851	LA76x_851; methylated-DNA-[]-cysteine S-methyltransferase family p
OG_02900	LA76x_3893	LA76x_3893; conserved hypothetical protein
OG_02901	LA76x_4241	LA76x_4241; phosphatidylethanolamine-binding family protein
OG_02902	LA76x_1919	uppS; di-trans,poly-cis-decaprenylcistransferase
OG_02903	LA76x_1565	raiA; ribosomal subunit interface protein
OG_02904	LA76x_4104	LA76x_4104; POTRA domain, ShlB-type family protein
OG_02905	LA76x_5085	LA76x_5085; doxX family protein
OG_02906	LA76x_4556	LA76x_4556; tonB-dependent Receptor Plug domain protein
OG_02907	LA76x_783	LA76x_783; tat (twin-arginine translocation) pathway signal sequence
OG_02908	LA76x_3677	LA76x_3677; conserved hypothetical protein
OG_02909	LA76x_4703	LA76x_4703; conserved hypothetical protein
OG_02910	LA76x_3460	LA76x_3460; aldehyde dehydrogenase family protein
OG_02911	LA76x_1480	LA76x_1480; RNA polymerase sigma factor, sigma-70 family protein
OG_02912	LA76x_3814	LA76x_3814; snoaL-like domain protein
OG_02913	LA76x_4802	fixJ; transcriptional regulatory protein fixJ
OG_02914	LA76x_3466	LA76x_3466; ABC transporter family protein
OG_02915	LA76x_4779	LA76x_4779; glycosyl transferases group 1 family protein
OG_02916	LA76x_3232	LA76x_3232; 2OG-Fe(II) oxygenase superfamily protein
OG_02917	LA76x_4736	ntrC; nitrogen regulation protein NR
OG_02918	LA76x_1792	upp; uracil phosphoribosyltransferase
OG_02919	LA76x_4005	LA76x_4005; 5'-3' exonuclease, C-terminal SAM fold family protein
OG_02920	LA76x_889	blaL2; beta-lactamase L2
OG_02921	LA76x_907	LA76x_907; bacterial regulatory helix-turn-helix , lysR family protein
OG_02922	LA76x_2142	LA76x_2142; rhomboid family protein
OG_02923	LA76x_1887	LA76x_1887; conserved hypothetical protein
OG_02924	LA76x_1072	LA76x_1072; ABC-2 type transporter family protein
OG_02925	LA76x_4166	LA76x_4166; tonB dependent receptor family protein
OG_02926	LA76x_3556	ppk1; polyphosphate kinase 1
OG_02927	LA76x_779	LA76x_779; hypothetical protein
OG_02928	LA76x_4851	LA76x_4851; ATP-grasp domain protein
OG_02929	LA76x_662	LA76x_662; NAD(P)H binding domain of trans-2-enoyl-CoA reductase
OG_02930	LA76x_1897	LA76x_1897; tic20-like family protein
OG_02931	LA76x_1690	LA76x_1690; conserved hypothetical protein
OG_02932	LA76x_3702	ispD; 4-diphosphocytidyl-2C-methyl-D-erythritol synthetase
OG_02933	LA76x_378	LA76x_378; N-acylglucosamine 2-epimerase family protein
OG_02934	LA76x_220	xth; exodeoxyribonuclease III
OG_02935	LA76x_1171	mraY; phospho-N-acetylmuramoyl-pentapeptide- transferase
OG_02936	LA76x_5136	LA76x_5136; bacterial DNA-binding family protein
OG_02937	LA76x_2458	pgmB; beta-phosphoglucomutase
OG_02938	LA76x_2034	recA; protein RecA
OG_02939	LA76x_97	ribonuclease; ribonuclease
OG_02940	LA76x_5161	LA76x_5161; conserved hypothetical protein
OG_02941	LA76x_4754	LA76x_4754; peptidase S51 family protein
OG_02942	LA76x_2317	cmk; cytidylate kinase

OG_02943	LA29479_0563	metallo-beta-lactamase superfamily protein
OG_02944	LA29479_4692	tonB family C-terminal domain protein
OG_02945	LA29479_4892	type IV fimbriae assembly protein
OG_02946	LA29479_1258	thymidine kinase family protein
OG_02947	LA29479_0550	transglycosylase SLT domain protein
OG_02948	LA29479_0299	amino acid permease family protein
OG_02949	LA29479_1844	NUDIX hydrolase
OG_02950	LA29479_1824	alpha-1,2-mannosidase, family protein
OG_02951	LA29479_2344	methyltransferase domain protein
OG_02952	LA29479_1122	methionine synthase
OG_02953	LA29479_3177	conserved hypothetical protein
OG_02954	LA29479_1203	tonB dependent receptor family protein
OG_02955	LA29479_2246	CDP-alcohol phosphatidyltransferase family protein
OG_02956	LA29479_0290	putative uncharacterized protein
OG_02957	LA29479_1581	translation elongation factor Ts
OG_02958	LA29479_3879	hypothetical protein
OG_02959	LA29479_0488	glucose-6-phosphate dehydrogenase
OG_02960	LA29479_2151	tryptophanyl-tRNA synthetase
OG_02961	LA29479_2711	hydrolase
OG_02962	LA29479_4580	prolyl oligopeptidase family protein
OG_02963	LA29479_4472	bacterial regulatory helix-turn-helix , lysR family protein
OG_02964	LA29479_1577	2,3,4,5-tetrahydropyridine-2,6-dicarboxylate N-succinyltransferase
OG_02965	LA29479_1335	impB/mucB/samB family protein
OG_02966	LA29479_1769	hypothetical protein
OG_02967	LA29479_2067	electron transfer flavodomain protein
OG_02968	LA29479_4701	glycosyl transferase 2 family protein
OG_02969	LA29479_0319	glutathionylspermidine synthase family protein
OG_02970	LA29479_2120	ribulose-phosphate 3-epimerase
OG_02971	LA29479_1423	deoxycytidine triphosphate deaminase
OG_02972	LA29479_4113	primosomal protein N'
OG_02973	LA29479_5234	branched-chain amino acid aminotransferase
OG_02974	LA29479_2762	poly(R)-hydroxyalkanoic acid synthase, class III, PhaC subunit
OG_02975	LA29479_4522	acyl-[acyl-carrier-protein]-UDP-N- acetylglucosamine O-acyltransfera
OG_02976	LA29479_3671	acetyltransferase family protein
OG_02977	LA29479_0698	phosphoribosylglycinamide formyltransferase
OG_02978	LA29479_2122	phosphoribosylaminoimidazole-succinocarboxamide synthase
OG_02979	LA29479_2660	conserved hypothetical protein
OG_02980	LA29479_1835	sensory/regulatory protein rpfC
OG_02981	LA29479_5030	hypothetical protein
OG_02982	LA29479_4719	CYTH domain protein
OG_02983	LA29479_0758	cytochrome b561 family protein
OG_02984	LA29479_1890	DNA ligase D, 3'-phosphoesterase domain
OG_02985	LA29479_1334	hypothetical protein
OG_02986	LA29479_4837	peptidase M48 family protein
OG_02987	LA29479_4365	NAD(P)H:quinone oxidoreductase, type IV
OG_02988	LA29479_1913	fumarylacetoacetate (FAA) hydrolase family protein
OG_02989	LA29479_1419	conserved hypothetical protein
OG_02990	LA29479_4043	prolyl oligopeptidase family protein
OG_02992	LA29479_3555	polyphosphate kinase 2 family protein
OG_02993	LA29479_2472	polysaccharide deacetylase family protein
OG_02994	LA29479_1088	putative uncharacterized protein
OG_02995	LA29479_1612	hypothetical protein
OG_02996	LA29479_0928	NAD-dependent epimerase/dehydratase
OG_02997	LA29479_1100	exonuclease family protein
OG_02998	LA29479_0090	HTH-type transcriptional activator AmpR
OG_02999	LA29479_2150	putative membrane protein
OG_03000	LA29479_1580	ribosomal protein S2
OG_03001	LA29479_1231	alpha/beta hydrolase fold family protein
OG_03002	LA29479_1981	cob(I)yrinic acid a,c-diamide adenosyltransferase
OG_03003	LA29479_1720	hypothetical protein
OG_03004	LA29479_3207	putative pit accessory protein
OG_03005	LA29479_2428	glycosyl transferases group 1 family protein
OG_03006	LA29479_3272	lipid A Biosynthesis N-terminal domain protein
OG_03007	LA29479_3986	conserved hypothetical protein
OG_03008	LA29479_4730	signal peptidase I
OG_03009	LA29479_2168	HAMP domain protein
OG_03010	LA29479_3845	hydroxyacylglutathione hydrolase
OG_03011	LA29479_2904	acrB/AcrD/AcrF family protein
OG_03012	LA29479_1514	isoleucyl-tRNA synthetase
OG_03013	LA29479_2255	phosphoribosylamine--glycine ligase
OG_03014	LA29479_1653	putative uncharacterized protein
OG_03015	LA29479_2162	conserved hypothetical protein
OG_03016	LA29479_0688	phosphoribosylformylglycinamidine cyclo-ligase
OG_03017	LA29479_3467	putative acetolactate synthase small subunit
OG_03018	LA29479_5048	conserved hypothetical protein
OG_03019	LA29479_2769	hypothetical protein
OG_03020	LA29479_4894	thymidylate kinase
OG_03021	LA29479_4968	ABC transporter family protein
OG_03022	LA29479_1889	alpha-L-glutamate ligases, RimK family protein
OG_03023	LA29479_2223	10 kDa chaperonin
OG_03024	LA29479_0459	permease family protein

OG_02943	LA76x_1700	LA76x_1700; metallo-beta-lactamase superfamily protein
OG_02944	LA76x_4077	LA76x_4077; tonB family C-terminal domain protein
OG_02945	LA76x_3493	pilZ; type IV fimbriae assembly protein
OG_02946	LA76x_112	LA76x_112; thymidine kinase family protein
OG_02947	LA76x_1715	LA76x_1715; transglycosylase SLT domain protein
OG_02948	LA76x_5144	LA76x_5144; spore germination family protein
OG_02949	LA76x_2786	LA76x_2786; NUDIX hydrolase
OG_02950	LA76x_2803	LA76x_2803; alpha-1,2-mannosidase family protein
OG_02951	LA76x_4408	LA76x_4408; methyltransferase small domain protein
OG_02952	LA76x_2522	metH; methionine synthase
OG_02953	LA76x_1845	LA76x_1845; conserved hypothetical protein
OG_02954	LA76x_2441	LA76x_2441; carboxypeptidase regulatory-like domain protein
OG_02955	LA76x_4313	LA76x_4313; CDP-alcohol phosphatidyltransferase family protein
OG_02956	LA76x_5154	LA76x_5154; conserved hypothetical protein
OG_02957	LA76x_1927	tsf; translation elongation factor Ts
OG_02958	LA76x_3580	LA76x_3580; hypothetical protein
OG_02959	LA76x_4870	zwf; glucose-6-phosphate dehydrogenase
OG_02960	LA76x_1001	trpS; tryptophan--tRNA ligase
OG_02961	LA76x_2423	LA76x_2423; hydrolase
OG_02962	LA76x_1418	LA76x_1418; prolyl oligopeptidase family protein
OG_02963	LA76x_4687	LA76x_4687; bacterial regulatory helix-turn-helix , lysR family protein
OG_02964	LA76x_1931	dapD; 2,3,4,5-tetrahydropyridine-2,6-dicarboxylate N-succinyltransfer
OG_02965	LA76x_37	LA76x_37; impB/mucB/samB family protein
OG_02966	LA76x_2860	LA76x_2860; transglutaminase-like superfamily protein
OG_02967	LA76x_1085	LA76x_1085; electron transfer flavodomain protein
OG_02968	LA76x_4085	LA76x_4085; glycosyl transferase 2 family protein
OG_02969	LA76x_5124	LA76x_5124; glutathionylspermidine synthase preATP-grasp family p
OG_02970	LA76x_1031	rpe; ribulose-phosphate 3-epimerase
OG_02971	LA76x_2081	dcd; deoxycytidine triphosphate deaminase
OG_02972	LA76x_340	priA; primosomal protein N'
OG_02973	LA76x_1407	ilvE; branched-chain amino acid aminotransferase
OG_02974	LA76x_2372	phaC; poly(R)-hydroxyalkanoic acid synthase, class III, PhaC subunit
OG_02975	LA76x_1911	lpxA; acyl-[acyl-carrier-protein]-UDP-N- acetylglucosamine O-acyltran
OG_02976	LA76x_3204	LA76x_3204; acetyltransferase family protein
OG_02977	LA76x_1577	purN; phosphoribosylglycinamide formyltransferase
OG_02978	LA76x_1029	purC; phosphoribosylaminoimidazolesuccinocarboxamide synthase
OG_02979	LA76x_584	LA76x_584; conserved hypothetical protein
OG_02980	LA76x_2795	rpfC; sensory/regulatory protein rpfC
OG_02981	LA76x_4558	LA76x_4558; tetratricopeptide repeat family protein
OG_02982	LA76x_3475	LA76x_3475; CYTH domain protein
OG_02983	LA76x_1518	LA76x_1518; prokaryotic cytochrome b561 family protein
OG_02984	LA76x_2742	ligD; DNA ligase D
OG_02985	LA76x_38	LA76x_38; acetyltransferase domain protein
OG_02986	LA76x_3305	LA76x_3305; peptidase M48 family protein
OG_02987	LA76x_1472	wrbA; quinone oxidoreductase, type IV
OG_02988	LA76x_1380	LA76x_1380; fumarylacetoacetate (FAA) hydrolase family protein
OG_02989	LA76x_2085	LA76x_2085; conserved hypothetical protein
OG_02990	LA76x_285	LA76x_285; prolyl oligopeptidase family protein
OG_02992	LA76x_3965	ppk2; polyphosphate kinase 2
OG_02993	LA76x_4426	LA76x_4426; polysaccharide deacetylase family protein
OG_02994	LA76x_2552	LA76x_2552; conserved hypothetical protein
OG_02995	LA76x_2223	LA76x_2223; hypothetical protein
OG_02996	LA76x_3768	LA76x_3768; NAD-dependent epimerase/dehydratase
OG_02997	LA76x_2541	LA76x_2541; exonuclease family protein
OG_02998	LA76x_3098	ampR; HTH-type transcriptional activator AmpR
OG_02999	LA76x_1002	LA76x_1002; putative transmembrane protein
OG_03000	LA76x_1928	rpsB; ribosomal protein S2
OG_03001	LA76x_137	LA76x_137; alpha/beta hydrolase fold family protein
OG_03002	LA76x_1220	cobO; cob(I)yrinic acid a,c-diamide adenosyltransferase
OG_03003	LA76x_2114	LA76x_2114; hypothetical protein
OG_03004	LA76x_1817	LA76x_1817; conserved hypothetical protein
OG_03005	LA76x_3397	LA76x_3397; glycosyl transferases group 1 family protein
OG_03006	LA76x_208	LA76x_208; lipid A Biosynthesis N-terminal domain protein
OG_03007	LA76x_4575	LA76x_4575; S-adenosyl-L-methionine-dependent methyltransferase
OG_03008	LA76x_3486	lepB; signal peptidase I
OG_03009	LA76x_987	LA76x_987; histidine kinase-, DNA gyrase B-, and HSP90-like ATPas
OG_03010	LA76x_3545	gloB; hydroxyacylglutathione hydrolase
OG_03011	LA76x_706	LA76x_706; acrB/AcrD/AcrF family protein
OG_03012	LA76x_1990	ileS; isoleucine--tRNA ligase
OG_03013	LA76x_4322	purD; phosphoribosylamine--glycine ligase
OG_03014	LA76x_2182	LA76x_2182; cytochrome C' family protein
OG_03015	LA76x_991	plsY; acyl-phosphate glycerol 3-phosphate acyltransferase
OG_03016	LA76x_1586	purM; phosphoribosylformylglycinamidine cyclo-ligase
OG_03017	LA76x_4251	LA76x_4251; ACT domain protein
OG_03018	LA76x_4692	LA76x_4692; formylglycine-generating sulfatase enzyme family prote
OG_03019	LA76x_2364	LA76x_2364; hypothetical protein
OG_03020	LA76x_3495	tmk; thymidylate kinase
OG_03021	LA76x_4055	LA76x_4055; ABC transporter family protein
OG_03022	LA76x_1404	LA76x_1404; alpha-L-glutamate ligases, RimK family protein
OG_03023	LA76x_4290	groES; 10 kDa chaperonin
OG_03024	LA76x_4900	LA76x_4900; ftsX-like permease family protein

OG_03025	LA29479_1087	ATPase associated with various cellular activities family protein
OG_03026	LA29479_1131	glutathione peroxidase family protein
OG_03027	LA29479_1459	K+-transporting ATPase, A subunit
OG_03028	LA29479_4873	molybdenum cofactor biosynthesis protein C
OG_03029	LA29479_2058	electron transfer flavoprotein-ubiquinone oxidoreductase, mitochondri
OG_03030	LA29479_3169	translation elongation factor P
OG_03031	LA29479_4567	pepSY-associated TM helix family protein
OG_03032	LA29479_4721	23S rRNA (uracil-5-)-methyltransferase RumA
OG_03033	LA29479_4995	rhodanese-like domain protein
OG_03034	LA29479_2057	2OG-Fe(II) oxygenase superfamily protein
OG_03035	LA29479_2784	inosine-5'-monophosphate dehydrogenase
OG_03036	LA29479_3854	phosphate regulon sensor kinase PhoR
OG_03037	LA29479_3910	conserved hypothetical protein
OG_03038	LA29479_1011	conserved hypothetical protein
OG_03039	LA29479_1467	alanyl-tRNA synthetase
OG_03040	LA29479_0956	acetyltransferase family protein
OG_03041	LA29479_4079	putative membrane protein
OG_03042	LA29479_3436	pyrophosphate-dependent phosphofructokinase
OG_03043	LA29479_1605	short chain dehydrogenase family protein
OG_03044	LA29479_2942	4-hydroxyproline epimerase
OG_03045	LA29479_4800	hydrolase CocE/NonD family protein
OG_03046	LA29479_4252	phenylalanyl-tRNA synthetase, beta subunit
OG_03047	LA29479_4759	hypothetical protein
OG_03048	LA29479_1227	conserved hypothetical protein
OG_03049	LA29479_0108	chitinase C
OG_03050	LA29479_2320	ATP synthase F1, epsilon subunit
OG_03051	LA29479_0527	domain of unknown function family protein
OG_03052	LA29479_0202	preprotein translocase, SecG subunit
OG_03053	LA29479_2467	rebB protein
OG_03054	LA29479_2761	GDSL-like Lipase/Acylhydrolase family protein
OG_03056	LA29479_0009	hypothetical protein
OG_03057	LA29479_0731	type II/IV secretion system family protein
OG_03058	LA29479_0512	ankyrin repeat family protein
OG_03059	LA29479_1529	enoyl-CoA hydratase/isomerase family protein
OG_03060	LA29479_3351	2-methylcitrate synthase/citrate synthase II family protein
OG_03061	LA29479_3588	putative membrane protein
OG_03062	LA29479_0744	conserved hypothetical protein
OG_03063	LA29479_1852	leucyl/phenylalanyl-tRNA--protein transferase
OG_03064	LA29479_0975	methylated-DNA-[]-cysteine S-methyltransferase family protein
OG_03065	LA29479_4250	merR regulatory family protein
OG_03066	LA29479_0670	putative membrane protein
OG_03067	LA29479_0514	response regulator
OG_03068	LA29479_1154	dihydrolipoyllysine-residue succinyltransferase, E2 component of oxo
OG_03069	LA29479_4757	putative uncharacterized protein
OG_03070	LA29479_3947	bacterial regulatory helix-turn-helix , lysR family protein
OG_03071	LA29479_2409	enoyl-CoA hydratase/isomerase family protein
OG_03072	LA29479_2086	outer membrane domain protein
OG_03073	LA29479_4531	acetyl-CoA carboxylase, carboxyl transferase, alpha subunit
OG_03074	LA29479_1393	putative membrane protein
OG_03075	LA29479_2087	putative lipoprotein
OG_03076	LA29479_0109	hypothetical protein
OG_03077	LA29479_1500	glutaredoxin family protein
OG_03078	LA29479_1474	tonB-dependent Receptor Plug domain protein
OG_03079	LA29479_2082	cystathionine beta-lyase
OG_03080	LA29479_4621	alpha-aminoadipic semialdehyde dehydrogenase
OG_03081	LA29479_1412	histidine triad (HIT) protein
OG_03082	LA29479_4910	ATPase associated with various cellular activities family protein
OG_03083	LA29479_3157	nicotinate (nicotinamide) nucleotide adenylyltransferase
OG_03084	LA29479_4340	asmA family protein
OG_03085	LA29479_3403	ribosomal L3 family protein
OG_03086	LA29479_1524	putative lipoprotein

OG_03025	LA76x_2553	LA76x_2553; AAA domain family protein
OG_03026	LA76x_2513	LA76x_2513; glutathione peroxidase family protein
OG_03027	LA76x_2046	kdpA; K+-transporting ATPase, A subunit
OG_03028	LA76x_3532	moaC; molybdenum cofactor biosynthesis protein C
OG_03029	LA76x_1094	ETFDH; electron transfer flavoprotein-ubiquinone oxidoreductase, mit
OG_03030	LA76x_1853	LA76x_1853; putative translation elongation factor P
OG_03031	LA76x_1433	LA76x_1433; pepSY-associated TM helix family protein
OG_03032	LA76x_3477	rumA; 23S rRNA (uracil-5-)-methyltransferase RumA
OG_03033	LA76x_4723	LA76x_4723; rhodanese-like domain protein
OG_03034	LA76x_1095	LA76x_1095; 2OG-Fe(II) oxygenase superfamily protein
OG_03035	LA76x_2348	guaB; inosine-5'-monophosphate dehydrogenase
OG_03036	LA76x_3555	phoR; phosphate regulon sensor kinase PhoR
OG_03037	LA76x_3610	LA76x_3610; conserved hypothetical protein
OG_03038	LA76x_3853	LA76x_3853; pseudouridine synthase family protein
OG_03039	LA76x_2036	alaS; alanine--tRNA ligase
OG_03040	LA76x_3799	LA76x_3799; acetyltransferase family protein
OG_03041	LA76x_252	LA76x_252; putative xanthomonadin biosynthesis membrane protein
OG_03042	LA76x_4220	LA76x_4220; pyrophosphate-dependent phosphofructokinase
OG_03043	LA76x_2229	LA76x_2229; NAD dependent epimerase/dehydratase family protein
OG_03044	LA76x_733	LA76x_733; 4-hydroxyproline epimerase
OG_03045	LA76x_3266	LA76x_3266; hydrolase CocE/NonD family protein
OG_03046	LA76x_1750	pheT; phenylalanine--tRNA ligase, beta subunit
OG_03047	LA76x_2272	LA76x_2272; gram-negative bacterial tonB family protein
OG_03048	LA76x_140	LA76x_140; conserved hypothetical protein
OG_03049	LA76x_3079	chiG; secreted chitinase domain protein
OG_03050	LA76x_4383	atpC; ATP synthase F1, epsilon subunit
OG_03051	LA76x_4834	LA76x_4834; META domain protein
OG_03052	LA76x_2988	secG; preprotein translocase, SecG subunit
OG_03053	LA76x_4421	LA76x_4421; killing trait family protein
OG_03054	LA76x_2373	LA76x_2373; GDSL-like Lipase/Acylhydrolase family protein
OG_03056	LA76x_3175	LA76x_3175; YXWGXW repeat family protein
OG_03057	LA76x_1545	LA76x_1545; twitching motility family protein
OG_03058	LA76x_4847	ANK1; ankyrin-1
OG_03059	LA76x_1974	LA76x_1974; enoyl-CoA hydratase/isomerase family protein
OG_03060	LA76x_3876	LA76x_3876; conserved hypothetical protein
OG_03061	LA76x_3997	LA76x_3997; conserved hypothetical protein
OG_03062	LA76x_1531	LA76x_1531; dodecin family protein
OG_03063	LA76x_2778	aat; leucyl/phenylalanyl-tRNA--protein transferase
OG_03064	LA76x_3819	LA76x_3819; methylated-DNA-[]-cysteine S-methyltransferase family
OG_03065	LA76x_1752	LA76x_1752; merR regulatory family protein
OG_03066	LA76x_1605	LA76x_1605; transglycosylase associated family protein
OG_03067	LA76x_4845	LA76x_4845; response regulator
OG_03068	LA76x_2490	sucB; dihydrolipoyllysine-residue succinyltransferase, E2 component
OG_03069	LA76x_2275	LA76x_2275; peptidase MA superfamily protein
OG_03070	LA76x_778	LA76x_778; bacterial regulatory helix-turn-helix , lysR family protein
OG_03071	LA76x_3379	LA76x_3379; enoyl-CoA hydratase/isomerase family protein
OG_03072	LA76x_1066	uptE; outer membrane domain protein
OG_03073	LA76x_1903	accA; acetyl-CoA carboxylase, carboxyl transferase, alpha subunit
OG_03074	LA76x_5185	LA76x_5185; hypothetical protein
OG_03075	LA76x_1065	LA76x_1065; conserved hypothetical protein
OG_03076	LA76x_3077	LA76x_3077; hypothetical protein
OG_03077	LA76x_2001	LA76x_2001; glutaredoxin family protein
OG_03078	LA76x_2029	LA76x_2029; tonB dependent receptor family protein
OG_03079	LA76x_1070	cysA; cystathionine gamma-lyase
OG_03080	LA76x_3431	ALDH7A1; alpha-aminoadipic semialdehyde dehydrogenase
OG_03081	LA76x_2092	LA76x_2092; histidine triad (HIT) protein
OG_03082	LA76x_3509	LA76x_3509; AAA domain family protein
OG_03083	LA76x_1863	nadD; nicotinate (nicotinamide) nucleotide adenylyltransferase
OG_03084	LA76x_1494	LA76x_1494; asmA family protein
OG_03085	LA76x_3928	rplC; 50S ribosomal protein L3
OG_03086	LA76x_1979	LA76x_1979; ompA family protein

Orthologous group	*L. cap* 55<br>GeneID (#3178)<br>50.8% of genome	Gene description
OG_00172	LC55x_3984	LC55x_3984; putative acetyltransferase
OG_00176	LC55x_1763	LC55x_1763; ABC transporter family protein
OG_00177	LC55x_3325	gph; phosphoglycolate phosphatase, bacterial
OG_00178	LC55x_2836	LC55x_2836; FKBP-type peptidyl-prolyl cis-trans isomerase family pr
OG_00179	LC55x_3812	LC55x_3812; curli production assembly/transport component CsgG fa
OG_00180	LC55x_4229	LC55x_4229; cobQ/CobB/MinD/ParA nucleotide binding domain prote
OG_00181	LC55x_242	LC55x_242; flagellar assembly FliH family protein
OG_00182	LC55x_496	LC55x_496; membrane dipeptidase family protein
OG_00183	LC55x_5172	LC55x_5172; TPR repeat family protein
OG_00184	LC55x_5678	LC55x_5678; amidohydrolase family protein
OG_00185	LC55x_1198	LC55x_1198; beta-lactamase family protein
OG_00186	LC55x_93	LC55x_93; vacJ like lipofamily protein
OG_00187	LC55x_3836	LC55x_3836; major Facilitator Superfamily protein
OG_00188	LC55x_4309	pyrF; orotidine 5'-phosphate decarboxylase
OG_00189	LC55x_3121	LC55x_3121; peptidase S41 family protein
OG_00190	LC55x_3753	LC55x_3753; conserved hypothetical protein
OG_00191	LC55x_921	LC55x_921; efflux transporter, RND family, MFP subunit
OG_00192	LC55x_901	LC55x_901; multicopper oxidase family protein
OG_00193	LC55x_2867	LC55x_2867; araC-like ligand binding domain protein
OG_00194	LC55x_4406	LC55x_4406; indole-3-glycerol phosphate synthase family protein
OG_00195	LC55x_1358	hda; dnaA regulatory inactivator Hda
OG_00196	LC55x_760	LC55x_760; conserved hypothetical protein
OG_00197	LC55x_2092	LC55x_2092; major Facilitator Superfamily protein
OG_00198	LC55x_3272	hisG; ATP phosphoribosyltransferase
OG_00199	LC55x_2953	LC55x_2953; conserved hypothetical protein
OG_00200	LC55x_2484	LC55x_2484; putative phosphotransferase
OG_00201	LC55x_569	LC55x_569; conserved hypothetical protein
OG_00202	LC55x_980	thiE; thiamine-phosphate pyrophosphorylase
OG_00203	LC55x_571	LC55x_571; cupin domain protein
OG_00204	LC55x_1398	LC55x_1398; acetyltransferase family protein
OG_00205	LC55x_2948	LC55x_2948; scpA/B family protein
OG_00206	LC55x_5164	LC55x_5164; KNTase C-terminal domain protein
OG_00207	LC55x_1025	recG; ATP-dependent DNA helicase RecG
OG_00208	LC55x_3320	LC55x_3320; peptidase M23 family protein
OG_00209	LC55x_385	LC55x_385; alpha/beta hydrolase fold family protein
OG_00210	LC55x_2839	LC55x_2839; bacterial regulatory, gntR family protein
OG_00211	LC55x_1109	mscL; large conductance mechanosensitive channel protein
OG_00212	LC55x_4952	tal; transaldolase
OG_00213	LC55x_992	rpiA; ribose 5-phosphate isomerase A
OG_00214	LC55x_1663	thrS; threonine--tRNA ligase
OG_00215	LC55x_5091	LC55x_5091; fabA-like domain protein
OG_00216	LC55x_2571	LC55x_2571; low affinity iron permease family protein
OG_00217	LC55x_503	LC55x_503; putative peptidase
OG_00218	LC55x_5591	LC55x_5591; beta-lactamase family protein
OG_00219	LC55x_2298	LC55x_2298; export membrane family protein
OG_00220	LC55x_460	LC55x_460; conserved hypothetical protein
OG_00221	LC55x_3419	LC55x_3419; cold-shock domain protein
OG_00222	LC55x_4062	pip; prolyl aminopeptidase
OG_00223	LC55x_2080	LC55x_2080; conserved hypothetical protein
OG_00224	LC55x_3725	LC55x_3725; nlpC/P60 family protein
OG_00225	LC55x_4701	LC55x_4701; hypothetical protein
OG_00226	LC55x_74	LC55x_74; cytochrome oxidase complex assembly 1 family protein
OG_00227	LC55x_1439	LC55x_1439; conserved hypothetical protein
OG_00228	LC55x_5196	LC55x_5196; hypothetical protein
OG_00229	LC55x_1275	hemE; uroporphyrinogen decarboxylase
OG_00230	LC55x_5703	LC55x_5703; pseudouridine synthase family protein
OG_00231	LC55x_4100	dhfrIII; dihydrofolate reductase type 3
OG_00232	LC55x_2767	dnaB; replicative DNA helicase
OG_00233	LC55x_3927	LC55x_3927; disulfide bond formation DsbB family protein
OG_00234	LC55x_5192	LC55x_5192; PP-loop family protein
OG_00235	LC55x_2660	LC55x_2660; polysaccharide biosynthesis/export family protein
OG_00236	LC55x_1393	LC55x_1393; conserved hypothetical protein
OG_00237	LC55x_5635	LC55x_5635; alpha/beta hydrolase family protein
OG_00238	LC55x_563	LC55x_563; GMC oxidoreductase family protein
OG_00239	LC55x_4533	LC55x_4533; glutathione S-transferase, N-terminal domain protein
OG_00240	LC55x_4729	LC55x_4729; bacterial regulatory, luxR family protein
OG_00241	LC55x_3754	LC55x_3754; tonB dependent receptor family protein
OG_00242	LC55x_5653	LC55x_5653; cytochrome c family protein
OG_00243	LC55x_4746	LC55x_4746; beta-lactamase family protein
OG_00244	LC55x_4684	hslV; ATP-dependent protease HslVU, peptidase subunit
OG_00245	LC55x_2834	LC55x_2834; nucleotide sugar dehydrogenase family protein
OG_00246	LC55x_2152	LC55x_2152; patatin-like phospholipase family protein
OG_00247	LC55x_2558	LC55x_2558; polyamine ABC transporter, ATP-binding family protein
OG_00248	LC55x_1443	LC55x_1443; polyprenyl synthetase family protein
OG_00249	LC55x_3501	LC55x_3501; na+/H+ antiporter family protein
OG_00250	LC55x_318	LC55x_318; putative thioesterase domain protein

Orthologous group	*L. cap* AZ78<br>Acc. no. (#3178)<br>56.3% of genome	Gene description
OG_00172	EYR66714.1	acetyltransferase [Lysobacter capsici AZ78]
OG_00176	EYR65687.1	multidrug ABC transporter ATPase [Lysobacter capsici AZ78]
OG_00177	EYR70077.1	phosphoglycolate phosphatase [Lysobacter capsici AZ78]
OG_00178	EYR69397.1	peptidyl-prolyl cis-trans isomerase [Lysobacter capsici AZ78]
OG_00179	EYR67116.1	peptidoglycan-binding protein [Lysobacter capsici AZ78]
OG_00180	EYR69090.1	cobyrinic acid a,c-diamide synthase [Lysobacter capsici AZ78]
OG_00181	EYR66179.1	hypothetical protein AZ78_22685 [Lysobacter capsici AZ78]
OG_00182	EYR66447.1	peptidase M19 [Lysobacter capsici AZ78]
OG_00183	EYR67998.1	protein kinase [Lysobacter capsici AZ78]
OG_00184	EYR65876.1	N-acyl-L-amino acid amidohydrolase [Lysobacter capsici AZ78]
OG_00185	EYR65786.1	beta-lactamase [Lysobacter capsici AZ78]
OG_00186	EYR69757.1	intercellular spreading VacJ lipoprotein [Lysobacter capsici AZ78]
OG_00187	EYR65743.1	major facilitator transporter [Lysobacter capsici AZ78]
OG_00188	EYR69157.1	orotidine 5'-phosphate decarboxylase [Lysobacter capsici AZ78]
OG_00189	EYR66973.1	hypothetical protein AZ78_18555 [Lysobacter capsici AZ78]
OG_00190	EYR67065.1	hypothetical protein AZ78_17540 [Lysobacter capsici AZ78]
OG_00191	EYR66838.1	hypothetical protein AZ78_18840 [Lysobacter capsici AZ78]
OG_00192	EYR66857.1	hypothetical protein AZ78_18935 [Lysobacter capsici AZ78]
OG_00193	EYR69425.1	transcriptional regulator [Lysobacter capsici AZ78]
OG_00194	EYR69234.1	indole-3-glycerol-phosphate synthase [Lysobacter capsici AZ78]
OG_00195	EYR69891.1	hypothetical protein AZ78_01620 [Lysobacter capsici AZ78]
OG_00196	EYR67629.1	hypothetical protein AZ78_14735 [Lysobacter capsici AZ78]
OG_00197	EYR66413.1	MFS transporter [Lysobacter capsici AZ78]
OG_00198	EYR70043.1	ATP phosphoribosyltransferase [Lysobacter capsici AZ78]
OG_00199	EYR66209.1	50S ribosomal protein L21 [Lysobacter capsici AZ78]
OG_00200	EYR69530.1	regulatory protein [Lysobacter capsici AZ78]
OG_00201	EYR70028.1	membrane protein [Lysobacter capsici AZ78]
OG_00202	EYR65389.1	thiamine-phosphate pyrophosphorylase [Lysobacter capsici AZ78]
OG_00203	EYR70027.1	cupin [Lysobacter capsici AZ78]
OG_00204	EYR67170.1	hypothetical protein AZ78_17255 [Lysobacter capsici AZ78]
OG_00205	EYR66212.1	rifampin ADP-ribosyl transferase [Lysobacter capsici AZ78]
OG_00206	EYR68003.1	kanamycin nucleotidyltransferase [Lysobacter capsici AZ78]
OG_00207	EYR67755.1	ATP-dependent DNA helicase RecG [Lysobacter capsici AZ78]
OG_00208	EYR70074.1	peptidase [Lysobacter capsici AZ78]
OG_00209	EYR65627.1	hydrolase [Lysobacter capsici AZ78]
OG_00210	EYR69400.1	GntR family transcriptional regulator [Lysobacter capsici AZ78]
OG_00211	EYR68472.1	large conductance mechanosensitive channel protein MscL [Lysobac
OG_00212	EYR67269.1	transaldolase [Lysobacter capsici AZ78]
OG_00213	EYR67781.1	ribose 5-phosphate isomerase [Lysobacter capsici AZ78]
OG_00214	EYR70445.1	threonyl-tRNA synthetase, partial [Lysobacter capsici AZ78]
OG_00215	EYR68057.1	dehydratase [Lysobacter capsici AZ78]
OG_00216	EYR68322.1	hypothetical protein AZ78_11695 [Lysobacter capsici AZ78]
OG_00217	EYR66441.1	protein kinase [Lysobacter capsici AZ78]
OG_00218	EYR65421.1	beta-lactamase [Lysobacter capsici AZ78]
OG_00219	EYR69661.1	transporter [Lysobacter capsici AZ78]
OG_00220	EYR66476.1	hypothetical protein AZ78_21070 [Lysobacter capsici AZ78]
OG_00221	EYR66509.1	cold-shock protein [Lysobacter capsici AZ78]
OG_00222	EYR65840.1	proline iminopeptidase [Lysobacter capsici AZ78]
OG_00223	EYR66423.1	hypothetical protein AZ78_21420 [Lysobacter capsici AZ78]
OG_00224	EYR65900.1	hypothetical protein AZ78_23615 [Lysobacter capsici AZ78]
OG_00225	EYR65663.1	hypothetical protein AZ78_25010 [Lysobacter capsici AZ78]
OG_00226	EYR69748.1	hypothetical protein AZ78_04780 [Lysobacter capsici AZ78]
OG_00227	EYR67137.1	hypothetical protein AZ78_17090 [Lysobacter capsici AZ78]
OG_00228	EYR68993.1	hypothetical protein AZ78_07330 [Lysobacter capsici AZ78]
OG_00229	EYR67674.1	uroporphyrinogen decarboxylase [Lysobacter capsici AZ78]
OG_00230	EYR65855.1	ribosomal large subunit pseudouridine synthase E [Lysobacter capsi
OG_00231	EYR65501.1	diacylglycerol kinase [Lysobacter capsici AZ78]
OG_00232	EYR69335.1	DNA helicase [Lysobacter capsici AZ78]
OG_00233	EYR66678.1	dihydrolipoamide acetyltransferase [Lysobacter capsici AZ78]
OG_00234	EYR68864.1	tRNA 2-thiocytidine biosynthesis protein TtcA [Lysobacter capsici AZ
OG_00235	EYR68238.1	capsular polysaccharide biosynthesis protein [Lysobacter capsici AZ
OG_00236	EYR67175.1	hypothetical protein AZ78_17280 [Lysobacter capsici AZ78]
OG_00237	EYR66273.1	hypothetical protein AZ78_22145 [Lysobacter capsici AZ78]
OG_00238	EYR67920.1	glucose-methanol-choline oxidoreductase [Lysobacter capsici AZ78]
OG_00239	EYR68391.1	glutathione S-transferase [Lysobacter capsici AZ78]
OG_00240	EYR66115.1	LuxR family transcriptional regulator [Lysobacter capsici AZ78]
OG_00241	EYR67066.1	TonB-dependent receptor [Lysobacter capsici AZ78]
OG_00242	EYR66289.1	cytochrome C [Lysobacter capsici AZ78]
OG_00243	EYR66100.1	beta-lactamase [Lysobacter capsici AZ78]
OG_00244	EYR70182.1	peptidase [Lysobacter capsici AZ78]
OG_00245	EYR69395.1	UDP-glucose 6-dehydrogenase [Lysobacter capsici AZ78]
OG_00246	EYR67373.1	patatin [Lysobacter capsici AZ78]
OG_00247	EYR65537.1	ABC transporter ATP-binding protein [Lysobacter capsici AZ78]
OG_00248	EYR67133.1	octaprenyl-diphosphate synthase [Lysobacter capsici AZ78]
OG_00249	EYR66630.1	sodium:proton antiporter [Lysobacter capsici AZ78]
OG_00250	EYR68101.1	thioesterase [Lysobacter capsici AZ78]

OG_00251	LC55x_2229	LC55x_2229; short chain dehydrogenase family protein	OG_00251	EYR67424.1	pteridine reductase [Lysobacter capsici AZ78]
OG_00252	LC55x_2120	LC55x_2120; conserved hypothetical protein	OG_00252	EYR66386.1	hypothetical protein AZ78_21230 [Lysobacter capsici AZ78]
OG_00253	LC55x_1978	LC55x_1978; MATE efflux family protein	OG_00253	EYR66768.1	multidrug transporter MatE [Lysobacter capsici AZ78]
OG_00254	LC55x_4297	ribD; riboflavin biosynthesis protein RibD	OG_00254	EYR69151.1	diaminohydroxyphosphoribosylaminopyrimidine deaminase [Lysobac
OG_00255	LC55x_4164	hrpA; ATP-dependent helicase HrpA	OG_00255	EYR68173.1	DEAD/DEAH box helicase [Lysobacter capsici AZ78]
OG_00256	LC55x_1367	cysI; sulfite reductase (NADPH) hemoprotein, beta-component	OG_00256	EYR67198.1	sulfite reductase [Lysobacter capsici AZ78]
OG_00257	LC55x_491	LC55x_491; sodium Bile acid symporter family protein	OG_00257	EYR66449.1	membrane protein [Lysobacter capsici AZ78]
OG_00258	LC55x_1736	tlyC; hemolysin	OG_00258	EYR70384.1	hemolysin [Lysobacter capsici AZ78]
OG_00259	LC55x_3497	LC55x_3497; RNase H family protein	OG_00259	EYR66626.1	ribonuclease H [Lysobacter capsici AZ78]
OG_00260	LC55x_5161	LC55x_5161; ABC transporter family protein	OG_00260	EYR68004.1	copper ABC transporter ATP-binding protein [Lysobacter capsici AZ7
OG_00261	LC55x_1418	LC55x_1418; hypothetical protein	OG_00261	EYR67153.1	hypothetical protein AZ78_17170 [Lysobacter capsici AZ78]
OG_00262	LC55x_2296	LC55x_2296; cytochrome c-552 domain protein	OG_00262	EYR69663.1	cytochrome C [Lysobacter capsici AZ78]
OG_00263	LC55x_3210	LC55x_3210; ATP-grasp domain protein	OG_00263	EYR67978.1	3-methylcrotonyl-CoA carboxylase subunit alpha [Lysobacter capsici
OG_00264	LC55x_5558	arsH; arsenical resistance protein ArsH	OG_00264	EYR68986.1	NADPH-dependent FMN reductase [Lysobacter capsici AZ78]
OG_00265	LC55x_750	LC55x_750; cyclic nucleotide-binding domain protein	OG_00265	EYR67638.1	cyclic nucleotide-binding protein [Lysobacter capsici AZ78]
OG_00266	LC55x_775	atpB; ATP synthase F0, A subunit	OG_00266	EYR67615.1	F0F1 ATP synthase subunit A [Lysobacter capsici AZ78]
OG_00267	LC55x_4085	LC55x_4085; ubiquinone biosynthesis hydroxylase, UbiH/UbiF/VisC/(	OG_00267	EYR65437.1	2-octaprenyl-3-methyl-6-methoxy-1,4-benzoquinol hydroxylase [Lyso
OG_00268	LC55x_1326	LC55x_1326; lipopolysaccharide-assembly, LptC-related family protei	OG_00268	EYR69910.1	hypothetical protein AZ78_01755 [Lysobacter capsici AZ78]
OG_00269	LC55x_509	LC55x_509; tonB dependent receptor family protein	OG_00269	EYR66435.1	ligand-gated channel protein [Lysobacter capsici AZ78]
OG_00270	LC55x_2471	hrcA; heat-inducible transcription repressor HrcA	OG_00270	EYR69540.1	HrcA family transcriptional regulator [Lysobacter capsici AZ78]
OG_00271	LC55x_3134	LC55x_3134; conserved hypothetical protein	OG_00271	EYR66960.1	hypothetical protein AZ78_18480 [Lysobacter capsici AZ78]
OG_00272	LC55x_490	LC55x_490; xaa-Pro dipeptidase, putative	OG_00272	EYR66450.1	Xaa-Pro dipeptidase [Lysobacter capsici AZ78]
OG_00273	LC55x_4953	LC55x_4953; FAD binding domain protein	OG_00273	EYR67268.1	NADH dehydrogenase [Lysobacter capsici AZ78]
OG_00274	LC55x_4752	ykfB; L-Ala-D/L-Glu epimerase	OG_00274	EYR66094.1	L-alanine-DL-glutamate epimerase [Lysobacter capsici AZ78]
OG_00275	LC55x_238	LC55x_238; conserved hypothetical protein	OG_00275	EYR66175.1	hypothetical protein AZ78_22665 [Lysobacter capsici AZ78]
OG_00276	LC55x_2658	etk; tyrosine-protein kinase etk	OG_00276	EYR68241.1	tyrosine protein kinase [Lysobacter capsici AZ78]
OG_00277	LC55x_4490	LC55x_4490; hypothetical protein	OG_00277	EYR68588.1	hypothetical protein AZ78_10170 [Lysobacter capsici AZ78]
OG_00278	LC55x_3959	rpoC; DNA-directed RNA polymerase, beta' subunit	OG_00278	EYR65488.1	DNA-directed RNA polymerase subunit beta' [Lysobacter capsici AZ7
OG_00280	LC55x_3667	hflC; hflC protein	OG_00280	EYR65949.1	membane protease HflC [Lysobacter capsici AZ78]
OG_00281	LC55x_2932	LC55x_2932; ABC transporter family protein	OG_00281	EYR66228.1	glycerol-3-phosphate ABC transporter ATP-binding protein [Lysobact
OG_00282	LC55x_1863	LC55x_1863; cation diffusion facilitator transporter family protein	OG_00282	EYR66055.1	hypothetical protein AZ78_23130 [Lysobacter capsici AZ78]
OG_00283	LC55x_2054	mutL; DNA mismatch repair MutL family protein	OG_00283	EYR65605.1	DNA mismatch repair protein MutL [Lysobacter capsici AZ78]
OG_00284	LC55x_913	LC55x_913; glycosyl transferases group 1 family protein	OG_00284	EYR66846.1	glycosyl transferase [Lysobacter capsici AZ78]
OG_00285	LC55x_489	cyoE; protoheme IX farnesyltransferase	OG_00285	EYR66451.1	protoheme IX farnesyltransferase [Lysobacter capsici AZ78]
OG_00286	LC55x_4500	LC55x_4500; amino acid permease family protein	OG_00286	EYR68412.1	amino acid transporter [Lysobacter capsici AZ78]
OG_00287	LC55x_2753	LC55x_2753; FAD binding domain of DNA photolyase family protein	OG_00287	EYR69321.1	deoxyribodipyrimidine photo-lyase [Lysobacter capsici AZ78]
OG_00288	LC55x_3445	pabC; aminodeoxychorismate lyase	OG_00288	EYR66592.1	4-amino-4-deoxychorismate lyase [Lysobacter capsici AZ78]
OG_00289	LC55x_4905	FRK1; fructokinase-1	OG_00289	EYR68708.1	fructokinase [Lysobacter capsici AZ78]
OG_00290	LC55x_5726	LC55x_5726; ABC-2 transporter family protein	OG_00290	EYR69694.1	sodium ABC transporter permease [Lysobacter capsici AZ78]
OG_00291	LC55x_3282	LC55x_3282; glycosyl transferase 2 family protein	OG_00291	EYR70049.1	chitin synthase [Lysobacter capsici AZ78]
OG_00292	LC55x_4249	yfiO; outer membrane assembly lipoYfiO family protein	OG_00292	EYR69104.1	competence protein [Lysobacter capsici AZ78]
OG_00293	LC55x_712	cwhA; N-acetylmuramoyl-L-alanine amidase A domain protein	OG_00293	EYR70096.1	hypothetical protein AZ78_01945 [Lysobacter capsici AZ78]
OG_00294	LC55x_3267	hisA; 1-(5-phosphoribosyl)-5-[(5- phosphoribosylamino)methylidenea	OG_00294	EYR70038.1	1-(5-phosphoribosyl)-5-[(5-phosphoribosylamino)methylideneamino]
OG_00295	LC55x_4000	LC55x_4000; hypothetical protein	OG_00295	EYR66729.1	lectin [Lysobacter capsici AZ78]
OG_00296	LC55x_2786	LC55x_2786; N-terminal double-transmembrane domain protein	OG_00296	EYR69349.1	hypothetical protein AZ78_05505 [Lysobacter capsici AZ78]
OG_00297	LC55x_1145	LC55x_1145; acetyltransferase family protein	OG_00297	EYR68518.1	GNAT family acetyltransferase [Lysobacter capsici AZ78]
OG_00298	LC55x_4405	LC55x_4405; HAD phosphoserine phosphatase-like hydrolase, IB far	OG_00298	EYR69233.1	haloacid dehalogenase [Lysobacter capsici AZ78]
OG_00299	LC55x_3079	LC55x_3079; outer membrane autotransporter barrel domain protein	OG_00299	EYR67855.1	hemagglutinin [Lysobacter capsici AZ78]
OG_00300	LC55x_1399	sufS; cysteine desulfurase, SufS family protein	OG_00300	EYR67169.1	cysteine desulfurase [Lysobacter capsici AZ78]
OG_00301	LC55x_118	LC55x_118; ABC transporter family protein	OG_00301	EYR69775.1	ABC transporter [Lysobacter capsici AZ78]
OG_00302	LC55x_2234	LC55x_2234; hypothetical protein	OG_00302	EYR67464.1	hypothetical protein AZ78_15985 [Lysobacter capsici AZ78]
OG_00303	LC55x_4411	LC55x_4411; conserved hypothetical protein	OG_00303	EYR69239.1	hypothetical protein AZ78_07200 [Lysobacter capsici AZ78]
OG_00304	LC55x_162	LC55x_162; phospholipase D family protein	OG_00304	EYR65366.1	cardiolipin synthase [Lysobacter capsici AZ78]
OG_00305	LC55x_2979	LC55x_2979; short chain dehydrogenase family protein	OG_00305	EYR65352.1	3-ketoacyl-ACP reductase [Lysobacter capsici AZ78]
OG_00306	LC55x_2744	copB; copper resistance protein B	OG_00306	EYR69314.1	hypothetical protein AZ78_05325 [Lysobacter capsici AZ78]
OG_00307	LC55x_1303	LC55x_1303; methyladenine glycosylase family protein	OG_00307	EYR69929.1	DNA-3-methyladenine glycosylase [Lysobacter capsici AZ78]
OG_00308	LC55x_5017	LC55x_5017; tautomerase enzyme family protein	OG_00308	EYR67213.1	4-oxalocrotonate tautomerase [Lysobacter capsici AZ78]
OG_00309	LC55x_5220	LC55x_5220; acetyltransferase family protein	OG_00309	EYR68882.1	N-acetyltransferase GCN5 [Lysobacter capsici AZ78]
OG_00310	LC55x_3688	LC55x_3688; bacterial regulatory, luxR family protein	OG_00310	EYR65930.1	hypothetical protein AZ78_23770 [Lysobacter capsici AZ78]
OG_00311	LC55x_191	LC55x_191; dienelactone hydrolase family protein	OG_00311	EYR66131.1	alpha/beta hydrolase [Lysobacter capsici AZ78]
OG_00312	LC55x_2816	LC55x_2816; ATP-NAD kinase family protein	OG_00312	EYR69378.1	inorganic polyphosphate/ATP-NAD kinase [Lysobacter capsici AZ78]
OG_00313	LC55x_5167	LC55x_5167; fatty acid desaturase family protein	OG_00313	EYR68001.1	aminotransferase [Lysobacter capsici AZ78]
OG_00314	LC55x_3452	fabH; 3-oxoacyl-[acyl-carrier-] synthase III family protein	OG_00314	EYR66598.1	3-oxoacyl-ACP synthase [Lysobacter capsici AZ78]
OG_00315	LC55x_3827	hutF; formiminoglutamate deiminase	OG_00315	EYR65752.1	N-formimino-L-glutamate deiminase [Lysobacter capsici AZ78]
OG_00316	LC55x_3268	hisH; imidazole glycerol phosphate synthase, glutamine amidotransfe	OG_00316	EYR70039.1	imidazole glycerol phosphate synthase [Lysobacter capsici AZ78]
OG_00317	LC55x_3962	rplJ; 50S ribosomal subunit protein L10	OG_00317	EYR65485.1	50S ribosomal protein L10 [Lysobacter capsici AZ78]
OG_00318	LC55x_2624	LC55x_2624; histidine kinase-, DNA gyrase B-, and HSP90-like ATPa	OG_00318	EYR68272.1	histidine kinase [Lysobacter capsici AZ78]
OG_00319	LC55x_911	LC55x_911; cupin domain protein	OG_00319	EYR66848.1	hypothetical protein AZ78_18890 [Lysobacter capsici AZ78]
OG_00320	LC55x_2586	iam; isoamylase	OG_00320	EYR68309.1	isoamylase [Lysobacter capsici AZ78]
OG_00321	LC55x_3135	LC55x_3135; conserved hypothetical protein	OG_00321	EYR66959.1	hypothetical protein AZ78_18475 [Lysobacter capsici AZ78]
OG_00322	LC55x_1800	LC55x_1800; inhibitor of apoptosis-promoting Bax1 family protein	OG_00322	EYR67289.1	membrane protein [Lysobacter capsici AZ78]
OG_00323	LC55x_5057	LC55x_5057; conserved hypothetical protein	OG_00323	EYR65584.1	hypothetical protein AZ78_25550 [Lysobacter capsici AZ78]
OG_00324	LC55x_912	LC55x_912; zinc-finger domain protein	OG_00324	EYR66847.1	hypothetical protein AZ78_18885 [Lysobacter capsici AZ78]
OG_00325	LC55x_382	LC55x_382; sagB-type dehydrogenase domain protein	OG_00325	EYR65630.1	processing protein [Lysobacter capsici AZ78]
OG_00326	LC55x_5011	LC55x_5011; his Kinase A domain protein	OG_00326	EYR67219.1	psensor histidine kinase [Lysobacter capsici AZ78]
OG_00327	LC55x_2630	LC55x_2630; sugar (and other) transporter family protein	OG_00327	EYR68266.1	arabinose ABC transporter permease [Lysobacter capsici AZ78]
OG_00328	LC55x_3333	LC55x_3333; peptidase M48 family protein	OG_00328	EYR66555.1	heat shock protein HtpX [Lysobacter capsici AZ78]
OG_00329	LC55x_1021	LC55x_1021; bacterial regulatory helix-turn-helix, AraC family protein	OG_00329	EYR67759.1	AraC family transcriptional regulator [Lysobacter capsici AZ78]
OG_00330	LC55x_4066	LC55x_4066; F5/8 type C domain protein	OG_00330	EYR65842.1	lysyl endopeptidase [Lysobacter capsici AZ78]
OG_00331	LC55x_4382	LC55x_4382; putative ATP binding component of ABC-transporter	OG_00331	EYR69216.1	sugar ABC transporter ATP-binding protein [Lysobacter capsici AZ78
OG_00332	LC55x_75	LC55x_75; putative aspartate/glutamate racemase	OG_00332	EYR69749.1	aspartate racemase [Lysobacter capsici AZ78]

OG_00334	LC55x_1111	LC55x_1111; hypothetical protein	OG_00334	EYR68474.1	membrane protein [Lysobacter capsici AZ78]
OG_00335	LC55x_841	LC55x_841; RNA polymerase sigma factor, sigma-70 family protein	OG_00335	EYR67572.1	RNA polymerase subunit sigma-24 [Lysobacter capsici AZ78]
OG_00336	LC55x_435	LC55x_435; phosphoglucomutase/phosphomannomutase, C-termina	OG_00336	EYR65521.1	phosphomannomutase [Lysobacter capsici AZ78]
OG_00337	LC55x_2559	potF; potF	OG_00337	EYR65538.1	hypothetical protein AZ78_25795 [Lysobacter capsici AZ78]
OG_00338	LC55x_4460	rocF; arginase	OG_00338	EYR68439.1	arginase [Lysobacter capsici AZ78]
OG_00339	LC55x_3973	LC55x_3973; 50S ribosome-binding GTPase family protein	OG_00339	EYR66706.1	GTP-binding protein YchF [Lysobacter capsici AZ78]
OG_00340	LC55x_65	LC55x_65; histidine kinase-, DNA gyrase B-, and HSP90-like ATPase	OG_00340	EYR69742.1	sensor histidine kinase [Lysobacter capsici AZ78]
OG_00341	LC55x_1844	chrR; chromate reductase	OG_00341	EYR66038.1	oxidoreductase [Lysobacter capsici AZ78]
OG_00342	LC55x_1197	LC55x_1197; polyketide cyclase / dehydrase and lipid transport famil	OG_00342	EYR65787.1	hypothetical protein AZ78_24520 [Lysobacter capsici AZ78]
OG_00343	LC55x_3459	LC55x_3459; conserved hypothetical protein	OG_00343	EYR66604.1	membrane protein [Lysobacter capsici AZ78]
OG_00344	LC55x_2137	LC55x_2137; ahpC/TSA family protein	OG_00344	EYR66372.1	Gpo [Lysobacter capsici AZ78]
OG_00345	LC55x_2756	LC55x_2756; glycine-zipper containing OmpA-like membrane domair	OG_00345	EYR69324.1	hypothetical protein AZ78_05380 [Lysobacter capsici AZ78]
OG_00346	LC55x_2448	LC55x_2448; enoyl-CoA hydratase/isomerase family protein	OG_00346	EYR69555.1	enoyl-CoA hydratase [Lysobacter capsici AZ78]
OG_00347	LC55x_942	AKHSDH1; bifunctional aspartokinase/homoserine dehydrogenase 1,	OG_00347	EYR66819.1	aspartokinase [Lysobacter capsici AZ78]
OG_00348	LC55x_3304	argC; N-acetyl-gamma-glutamyl-phosphate reductase	OG_00348	EYR70061.1	N-acetyl-gamma-glutamyl-phosphate reductase [Lysobacter capsici A
OG_00349	LC55x_4208	LC55x_4208; acetyltransferase family protein	OG_00349	EYR69074.1	acetyltransferase [Lysobacter capsici AZ78]
OG_00350	LC55x_753	LC55x_753; ftsX-like permease family protein	OG_00350	EYR67635.1	inner membrane transport permease [Lysobacter capsici AZ78]
OG_00351	LC55x_1115	LC55x_1115; thiazole biosynthesis ThiG family protein	OG_00351	EYR68478.1	thiazole synthase [Lysobacter capsici AZ78]
OG_00352	LC55x_3644	LC55x_3644; conserved hypothetical protein	OG_00352	EYR68652.1	hypothetical protein AZ78_08445 [Lysobacter capsici AZ78]
OG_00353	LC55x_5687	LC55x_5687; yecA family protein	OG_00353	EYR65871.1	hypothetical protein AZ78_24045 [Lysobacter capsici AZ78]
OG_00354	LC55x_480	coxB; cytochrome c oxidase, subunit II	OG_00354	EYR66460.1	cytochrome B5 [Lysobacter capsici AZ78]
OG_00355	LC55x_1314	LC55x_1314; phosphocarrier, HPr family protein	OG_00355	EYR69920.1	phosphocarrier protein HPr [Lysobacter capsici AZ78]
OG_00356	LC55x_3860	tdh; L-threonine 3-dehydrogenase	OG_00356	EYR65548.1	L-threonine 3-dehydrogenase [Lysobacter capsici AZ78]
OG_00357	LC55x_993	LC55x_993; conserved hypothetical protein	OG_00357	EYR67780.1	EVE domain-containing protein [Lysobacter capsici AZ78]
OG_00358	LC55x_226	epaR; type III secretion apparatus protein SpaR/YscT/HrcT	OG_00358	EYR66165.1	hypothetical protein AZ78_22615 [Lysobacter capsici AZ78]
OG_00359	LC55x_306	LC55x_306; acetyltransferase family protein	OG_00359	EYR67036.1	hypothetical protein AZ78_18145 [Lysobacter capsici AZ78]
OG_00360	LC55x_838	LC55x_838; essential respiratory protein A	OG_00360	EYR67575.1	iron--sulfur cluster insertion protein ErpA [Lysobacter capsici AZ78]
OG_00361	LC55x_899	LC55x_899; hypothetical protein	OG_00361	EYR66859.1	hypothetical protein AZ78_18945 [Lysobacter capsici AZ78]
OG_00362	LC55x_2308	ydhM; nemR DNA binding transcriptional repressor	OG_00362	EYR69656.1	TetR family transcriptional regulator [Lysobacter capsici AZ78]
OG_00363	LC55x_244	LC55x_244; conserved hypothetical protein	OG_00363	EYR66181.1	hypothetical protein AZ78_22695 [Lysobacter capsici AZ78]
OG_00364	LC55x_2259	aroA; 3-phosphoshikimate 1-carboxyvinyltransferase	OG_00364	EYR67446.1	3-phosphoshikimate 1-carboxyvinyltransferase [Lysobacter capsici A
OG_00365	LC55x_2406	LC55x_2406; cysteine dioxygenase type I family protein	OG_00365	EYR69590.1	cysteine dioxygenase [Lysobacter capsici AZ78]
OG_00366	LC55x_4801	LC55x_4801; putative relaxation protein	OG_00366	EYR68777.1	relaxation protein [Lysobacter capsici AZ78]
OG_00367	LC55x_3868	prpD; 2-methylcitrate dehydratase	OG_00367	EYR65541.1	2-methylcitrate dehydratase [Lysobacter capsici AZ78]
OG_00368	LC55x_1009	LC55x_1009; hypothetical protein	OG_00368	EYR67789.1	hypothetical protein AZ78_14220 [Lysobacter capsici AZ78]
OG_00369	LC55x_2071	LC55x_2071; conserved hypothetical protein	OG_00369	EYR65397.1	membrane protein [Lysobacter capsici AZ78]
OG_00370	LC55x_1019	spoT; guanosine-3',5'-bis(diphosphate) 3'-pyrophosphohydrolase	OG_00370	EYR67761.1	bifunctional (p)ppGpp synthetase II/ guanosine-3',5'-bis pyrophospha
OG_00371	LC55x_3902	LC55x_3902; phosphate-selective porin O and P family protein	OG_00371	EYR66660.1	porin [Lysobacter capsici AZ78]
OG_00372	LC55x_1000	LC55x_1000; aminopeptidase P, N-terminal domain protein	OG_00372	EYR67774.1	proline aminopeptidase P II [Lysobacter capsici AZ78]
OG_00373	LC55x_2307	LC55x_2307; bacterial regulatory, tetR family protein	OG_00373	EYR69657.1	TetR family transcriptional regulator [Lysobacter capsici AZ78]
OG_00374	LC55x_4477	rtcB; conserved protein	OG_00374	EYR68426.1	RTCB protein [Lysobacter capsici AZ78]
OG_00375	LC55x_430	LC55x_430; DNA repair RadC family protein	OG_00375	EYR65516.1	hypothetical protein AZ78_25820 [Lysobacter capsici AZ78]
OG_00376	LC55x_4130	bioA; adenosylmethionine-8-amino-7-oxononanoate transaminase	OG_00376	EYR66350.1	adenosylmethionine-8-amino-7-oxononanoate aminotransferase [Lys
OG_00377	LC55x_3387	LC55x_3387; conserved hypothetical protein	OG_00377	EYR66528.1	hypothetical protein AZ78_20490 [Lysobacter capsici AZ78]
OG_00378	LC55x_3557	LC55x_3557; hypothetical protein	OG_00378	EYR67508.1	hypothetical protein AZ78_15290 [Lysobacter capsici AZ78]
OG_00379	LC55x_819	LC55x_819; putative afsR-like transcriptional regulator	OG_00379	EYR67684.1	hypothetical protein AZ78_14460 [Lysobacter capsici AZ78]
OG_00380	LC55x_3693	ispF; 2-C-methyl-D-erythritol 2,4-cyclodiphosphate synthase	OG_00380	EYR65925.1	2-C-methyl-D-erythritol 2,4-cyclodiphosphate synthase [Lysobacter c
OG_00381	LC55x_875	accC; acetyl-CoA carboxylase, biotin carboxylase subunit	OG_00381	EYR66882.1	acetyl-CoA carboxylase biotin carboxylase subunit [Lysobacter capsi
OG_00382	LC55x_202	LC55x_202; conserved hypothetical protein	OG_00382	EYR66142.1	hypothetical protein AZ78_22500 [Lysobacter capsici AZ78]
OG_00383	LC55x_2928	LC55x_2928; hypothetical protein	OG_00383	EYR66232.1	hypothetical protein AZ78_21930 [Lysobacter capsici AZ78]
OG_00384	LC55x_4664	mrdB; rod shape-determining protein RodA	OG_00384	EYR70165.1	rod shape-determining protein RodA [Lysobacter capsici AZ78]
OG_00385	LC55x_1743	LC55x_1743; putative membrane protein	OG_00385	EYR65706.1	hypothetical protein AZ78_24965 [Lysobacter capsici AZ78]
OG_00386	LC55x_112	LC55x_112; V4R domain protein	OG_00386	EYR69881.1	hypothetical protein AZ78_04955 [Lysobacter capsici AZ78]
OG_00387	LC55x_1332	LC55x_1332; conserved hypothetical protein	OG_00387	EYR69905.1	hypothetical protein AZ78_01730 [Lysobacter capsici AZ78]
OG_00388	LC55x_86	LC55x_86; methyltransferase domain protein	OG_00388	EYR69754.1	oxidoreductase [Lysobacter capsici AZ78]
OG_00389	LC55x_4697	LC55x_4697; WYL domain protein	OG_00389	EYR65659.1	transcriptional regulator [Lysobacter capsici AZ78]
OG_00390	LC55x_2341	LC55x_2341; moeA C-terminal region family protein	OG_00390	EYR69629.1	molybdopterin biosynthesis protein [Lysobacter capsici AZ78]
OG_00391	LC55x_1734	LC55x_1734; putative diguanylate cyclase/phosphodiesterase	OG_00391	EYR70382.1	DeoR family transcriptional regulator [Lysobacter capsici AZ78]
OG_00392	LC55x_1290	LC55x_1290; proteobacterial lipase chaperone family protein	OG_00392	EYR67706.1	hypothetical protein AZ78_14980 [Lysobacter capsici AZ78]
OG_00393	LC55x_1357	LC55x_1357; conserved hypothetical protein	OG_00393	EYR69892.1	membrane protein [Lysobacter capsici AZ78]
OG_00394	LC55x_3147	LC55x_3147; prolyl oligopeptidase family protein	OG_00394	EYR66951.1	hypothetical protein AZ78_18435 [Lysobacter capsici AZ78]
OG_00395	LC55x_918	LC55x_918; type I secretion outer membrane , TolC family protein	OG_00395	EYR66842.1	membrane protein [Lysobacter capsici AZ78]
OG_00396	LC55x_2340	LC55x_2340; mobA-like NTP transferase domain protein	OG_00396	EYR69630.1	molybdopterin-guanine dinucleotide biosynthesis protein MobA [Lyso
OG_00397	LC55x_3214	HMGCL; hydroxymethylglutaryl-CoA lyase	OG_00397	EYR67973.1	hydroxymethylglutaryl-CoA lyase [Lysobacter capsici AZ78]
OG_00398	LC55x_3535	LC55x_3535; conserved hypothetical protein	OG_00398	EYR67487.1	hypothetical protein AZ78_15185 [Lysobacter capsici AZ78]
OG_00399	LC55x_601	LC55x_601; bacterial extracellular solute-binding family protein	OG_00399	EYR70004.1	ABC transporter substrate-binding protein [Lysobacter capsici AZ78]
OG_00400	LC55x_4379	gmd; GDP-mannose 4,6-dehydratase	OG_00400	EYR69213.1	GDP-mannose 4,6-dehydratase [Lysobacter capsici AZ78]
OG_00401	LC55x_1759	LC55x_1759; putative transmembrane protein	OG_00401	EYR65691.1	membrane protein [Lysobacter capsici AZ78]
OG_00402	LC55x_2787	LC55x_2787; von Willebrand factor type A domain protein	OG_00402	EYR69350.1	von Willebrand factor A [Lysobacter capsici AZ78]
OG_00403	LC55x_1709	fdx1; ferredoxin	OG_00403	EYR70367.1	ferredoxin [Lysobacter capsici AZ78]
OG_00404	LC55x_3904	LC55x_3904; response regulator	OG_00404	EYR66661.1	sensor histidine kinase [Lysobacter capsici AZ78]
OG_00405	LC55x_5073	LC55x_5073; beta-ketoacyl synthase, C-terminal domain protein	OG_00405	EYR68066.1	3-oxoacyl-ACP synthase [Lysobacter capsici AZ78]
OG_00406	LC55x_4284	LC55x_4284; lppC lipofamily protein	OG_00406	EYR69140.1	lppc lipoprotein [Lysobacter capsici AZ78]
OG_00407	LC55x_2186	kdsB; 3-deoxy-D-manno-octulosonate cytidylyltransferase	OG_00407	EYR67403.1	3-deoxy-manno-octulosonate cytidylyltransferase [Lysobacter capsic
OG_00408	LC55x_5124	LC55x_5124; putative transmembrane protein	OG_00408	EYR68032.1	membrane protein [Lysobacter capsici AZ78]
OG_00409	LC55x_3818	LC55x_3818; bacterial regulatory, tetR family protein	OG_00409	EYR67122.1	hypothetical protein AZ78_17830 [Lysobacter capsici AZ78]
OG_00410	LC55x_3077	LC55x_3077; conserved hypothetical protein	OG_00410	EYR67854.1	hypothetical protein AZ78_13820 [Lysobacter capsici AZ78]
OG_00411	LC55x_1731	LC55x_1731; RNA methyltransferase, TrmH , group 1 family protein	OG_00411	EYR70380.1	RNA methyltransferase [Lysobacter capsici AZ78]
OG_00412	LC55x_4119	LC55x_4119; transcriptional regulatory , C terminal family protein	OG_00412	EYR66361.1	XRE family transcriptional regulator [Lysobacter capsici AZ78]
OG_00413	LC55x_3008	LC55x_3008; NADH-quinone oxidoreductase, chain I family protein	OG_00413	EYR67806.1	NADH dehydrogenase subunit I [Lysobacter capsici AZ78]
OG_00414	LC55x_3993	acyP; acylphosphatase	OG_00414	EYR66723.1	acylphosphatase [Lysobacter capsici AZ78]

OG_00416	LC55x_1857	yaeT; outer membrane assembly complex, YaeT protein
OG_00417	LC55x_2151	LC55x_2151; hydrolyase, tartrate alpha subunit/fumarate, Fe-S type (
OG_00418	LC55x_2253	yedZ; conserved inner membrane protein
OG_00419	LC55x_3301	proB; glutamate 5-kinase
OG_00420	LC55x_1568	LC55x_1568; prolyl oligopeptidase family protein
OG_00421	LC55x_5012	LC55x_5012; ecsC family protein
OG_00422	LC55x_891	LC55x_891; cupin 2, conserved barrel domain protein
OG_00423	LC55x_2914	LC55x_2914; EF-P lysine aminoacylase GenX
OG_00424	LC55x_4695	LC55x_4695; putative transmembrane protein
OG_00425	LC55x_2028	LC55x_2028; conserved hypothetical family protein
OG_00426	LC55x_4279	LC55x_4279; mraZ family protein
OG_00427	LC55x_3270	hisC; histidinol-phosphate transaminase
OG_00428	LC55x_779	atpA; ATP synthase F1, alpha subunit
OG_00429	LC55x_4541	fabR; fabR transcriptional repressor FabR transcriptional dual regulat
OG_00430	LC55x_724	LC55x_724; putative efflux pump outer membrane protein ttgC
OG_00431	LC55x_2083	rimO; ribosomal protein S12 methylthiotransferase RimO
OG_00432	LC55x_4262	secA; preprotein translocase, SecA subunit
OG_00433	LC55x_3482	LC55x_3482; lactonase, 7-bladed beta-propeller family protein
OG_00434	LC55x_936	ilvC; ketol-acid reductoisomerase
OG_00435	LC55x_2954	LC55x_2954; bacterial regulatory, tetR family protein
OG_00436	LC55x_1291	LC55x_1291; hypothetical protein
OG_00437	LC55x_3542	LC55x_3542; pirin family protein
OG_00438	LC55x_1171	LC55x_1171; pfkB carbohydrate kinase family protein
OG_00439	LC55x_3394	LC55x_3394; PAP2 superfamily protein
OG_00440	LC55x_243	LC55x_243; putative ATP synthase yscN
OG_00441	LC55x_2425	LC55x_2425; transglycosylase SLT domain protein
OG_00442	LC55x_2410	dsbE; disulfide oxidoreductases, DsbE subfamily protein
OG_00443	LC55x_2572	LC55x_2572; ku protein
OG_00444	LC55x_3109	LC55x_3109; subtilase family protein
OG_00445	LC55x_2163	LC55x_2163; glycine zipper 2TM domain protein
OG_00446	LC55x_3895	prpE; propionate--CoA ligase
OG_00447	LC55x_683	LC55x_683; glyoxalase-like domain protein
OG_00448	LC55x_4471	LC55x_4471; transcriptional regulatory , C terminal family protein
OG_00449	LC55x_2084	LC55x_2084; flavo, HI0933 family protein
OG_00450	LC55x_2264	LC55x_2264; NADPH-dependent FMN reductase family protein
OG_00451	LC55x_379	LC55x_379; diguanylate cyclase domain protein
OG_00452	LC55x_1330	LC55x_1330; bolA superfamily transcriptional regulator
OG_00453	LC55x_4749	LC55x_4749; tonB-dependent Receptor Plug domain protein
OG_00454	LC55x_283	LC55x_283; iaaA
OG_00455	LC55x_2693	LC55x_2693; cyclic peptide transporter family protein
OG_00456	LC55x_4423	rraA; regulator of ribonuclease activity A
OG_00457	LC55x_3453	rpmF; ribosomal protein L32
OG_00458	LC55x_501	LC55x_501; yqey-like family protein
OG_00459	LC55x_208	LC55x_208; nitrogen regulatory P-II family protein
OG_00460	LC55x_839	nudC; NADH pyrophosphatase
OG_00461	LC55x_796	LC55x_796; ABC transporter family protein
OG_00462	LC55x_1260	LC55x_1260; alpha/beta hydrolase fold family protein
OG_00463	LC55x_2485	recJ; single-stranded-DNA-specific exonuclease RecJ
OG_00464	LC55x_2134	LC55x_2134; amidohydrolase family protein
OG_00465	LC55x_1730	LC55x_1730; inositol monophosphatase family protein
OG_00466	LC55x_3186	LC55x_3186; methylmalonyl-CoA mutase N-terminal domain protein
OG_00467	LC55x_2535	LC55x_2535; bacterial regulatory helix-turn-helix , lysR family protein
OG_00468	LC55x_5204	LC55x_5204; gram-negative pili assembly chaperone, N-terminal dor
OG_00469	LC55x_2018	csrA; carbon storage regulator
OG_00470	LC55x_3673	folP; dihydropteroate synthase
OG_00471	LC55x_1894	LC55x_1894; WD40-like Beta Propeller Repeat family protein
OG_00472	LC55x_4709	LC55x_4709; tonB family C-terminal domain protein
OG_00473	LC55x_2494	LC55x_2494; PA14 domain protein
OG_00474	LC55x_2289	yceC; 23S rRNA pseudouridine synthase
OG_00475	LC55x_4717	LC55x_4717; 2Fe-2S iron-sulfur cluster binding domain protein
OG_00476	LC55x_4295	ribE; riboflavin synthase, alpha subunit
OG_00477	LC55x_351	LC55x_351; pfkB carbohydrate kinase family protein
OG_00478	LC55x_1365	LC55x_1365; bacterial regulatory helix-turn-helix , lysR family protein
OG_00479	LC55x_4363	LC55x_4363; mannose-1-phosphate guanylyltransferase/mannose-6
OG_00480	LC55x_106	LC55x_106; short chain dehydrogenase family protein
OG_00481	LC55x_193	LC55x_193; tetratricopeptide repeat family protein
OG_00482	LC55x_4396	LC55x_4396; acetyltransferase family protein
OG_00483	LC55x_3633	LC55x_3633; putative secreted protein
OG_00484	LC55x_1082	LC55x_1082; ribonucleotide reductase, small chain family protein
OG_00485	LC55x_107	LC55x_107; efflux transporter, RND family, MFP subunit
OG_00486	LC55x_4263	LC55x_4263; peptidase M23 family protein
OG_00487	LC55x_165	LC55x_165; cobQ/CobB/MinD/ParA nucleotide binding domain protei
OG_00488	LC55x_4914	LC55x_4914; bacterial PH domain protein
OG_00489	LC55x_977	LC55x_977; HIT domain protein
OG_00490	LC55x_2778	asnS; asparagine--tRNA ligase
OG_00491	LC55x_2442	LC55x_2442; conserved hypothetical protein
OG_00492	LC55x_3520	LC55x_3520; carbon-nitrogen hydrolase family protein
OG_00493	LC55x_2365	LC55x_2365; mechanosensitive ion channel family protein
OG_00494	LC55x_4184	queC; queuosine biosynthesis protein QueC
OG_00495	LC55x_791	LC55x_791; his Kinase A domain protein
OG_00496	LC55x_4614	LC55x_4614; efflux transporter, RND family, MFP subunit

OG_00416	EYR66049.1	membrane protein [Lysobacter capsici AZ78]
OG_00417	EYR67372.1	fumarate hydratase [Lysobacter capsici AZ78]
OG_00418	EYR67441.1	sulfite oxidase [Lysobacter capsici AZ78]
OG_00419	EYR70058.1	glutamate 5-kinase [Lysobacter capsici AZ78]
OG_00420	EYR70287.1	peptidase S9 [Lysobacter capsici AZ78]
OG_00421	EYR67218.1	peptidase [Lysobacter capsici AZ78]
OG_00422	EYR66866.1	cupin [Lysobacter capsici AZ78]
OG_00423	EYR69470.1	lysyl-tRNA synthetase [Lysobacter capsici AZ78]
OG_00424	EYR70192.1	membrane protein [Lysobacter capsici AZ78]
OG_00425	EYR65375.1	hypothetical protein AZ78_26680 [Lysobacter capsici AZ78]
OG_00426	EYR69137.1	cell division protein MraZ [Lysobacter capsici AZ78]
OG_00427	EYR70041.1	histidinol-phosphate aminotransferase [Lysobacter capsici AZ78]
OG_00428	EYR67611.1	F0F1 ATP synthase subunit alpha [Lysobacter capsici AZ78]
OG_00429	EYR68383.1	TetR family transcriptional regulator [Lysobacter capsici AZ78]
OG_00430	EYR67655.1	multidrug transporter [Lysobacter capsici AZ78]
OG_00431	EYR66420.1	ribosomal protein S12 methylthiotransferase [Lysobacter capsici AZ7
OG_00432	EYR69120.1	preprotein translocase subunit SecA [Lysobacter capsici AZ78]
OG_00433	EYR66653.1	hypothetical protein AZ78_20075 [Lysobacter capsici AZ78]
OG_00434	EYR66825.1	ketol-acid reductoisomerase [Lysobacter capsici AZ78]
OG_00435	EYR66208.1	TetR family transcriptional regulator [Lysobacter capsici AZ78]
OG_00436	EYR67705.1	hypothetical protein AZ78_14975 [Lysobacter capsici AZ78]
OG_00437	EYR67494.1	pirin [Lysobacter capsici AZ78]
OG_00438	EYR68537.1	ribokinase [Lysobacter capsici AZ78]
OG_00439	EYR66525.1	membrane protein [Lysobacter capsici AZ78]
OG_00440	EYR66180.1	ATP synthase [Lysobacter capsici AZ78]
OG_00441	EYR69573.1	type VI secretion protein [Lysobacter capsici AZ78]
OG_00442	EYR69586.1	thiol:disulfide interchange protein [Lysobacter capsici AZ78]
OG_00443	EYR68321.1	Ku domain-containing protein [Lysobacter capsici AZ78]
OG_00444	EYR65455.1	hypothetical protein AZ78_26275 [Lysobacter capsici AZ78]
OG_00445	EYR67382.1	membrane protein [Lysobacter capsici AZ78]
OG_00446	EYR65727.1	acetyl-CoA synthetase [Lysobacter capsici AZ78]
OG_00447	EYR69954.1	extradiol dioxygenase [Lysobacter capsici AZ78]
OG_00448	EYR68431.1	transcriptional regulator [Lysobacter capsici AZ78]
OG_00449	EYR66419.1	membrane protein [Lysobacter capsici AZ78]
OG_00450	EYR69687.1	azoreductase [Lysobacter capsici AZ78]
OG_00451	EYR65633.1	cyclic nucleotide-binding protein [Lysobacter capsici AZ78]
OG_00452	EYR69907.1	BolA family transcriptional regulator [Lysobacter capsici AZ78]
OG_00453	EYR66097.1	TonB-denpendent receptor [Lysobacter capsici AZ78]
OG_00454	EYR67010.1	asparaginase [Lysobacter capsici AZ78]
OG_00455	EYR65459.1	hypothetical protein AZ78_26180 [Lysobacter capsici AZ78]
OG_00456	EYR65464.1	ribonuclease [Lysobacter capsici AZ78]
OG_00457	EYR66599.1	50S ribosomal protein L32 [Lysobacter capsici AZ78]
OG_00458	EYR66443.1	GatB/Yqey [Lysobacter capsici AZ78]
OG_00459	EYR66148.1	nitrogen regulatory protein P-II 1 [Lysobacter capsici AZ78]
OG_00460	EYR67574.1	NADH pyrophosphatase [Lysobacter capsici AZ78]
OG_00461	EYR67595.1	phosphonate ABC transporter ATP-binding protein [Lysobacter capsi
OG_00462	EYR65649.1	alpha/beta hydrolase [Lysobacter capsici AZ78]
OG_00463	EYR69529.1	ssDNA exonuclease RecJ [Lysobacter capsici AZ78]
OG_00464	EYR66374.1	hypothetical protein AZ78_21170 [Lysobacter capsici AZ78]
OG_00465	EYR70379.1	inositol monophosphatase [Lysobacter capsici AZ78]
OG_00466	EYR66911.1	methylmalonyl-CoA mutase [Lysobacter capsici AZ78]
OG_00467	EYR69489.1	transcriptional regulator [Lysobacter capsici AZ78]
OG_00468	EYR68869.1	pilus assembly protein [Lysobacter capsici AZ78]
OG_00469	EYR66740.1	carbon storage regulator CsrA [Lysobacter capsici AZ78]
OG_00470	EYR65944.1	dihydropteroate synthase [Lysobacter capsici AZ78]
OG_00471	EYR65998.1	hypothetical protein AZ78_23520 [Lysobacter capsici AZ78]
OG_00472	EYR65671.1	hypothetical protein AZ78_25050 [Lysobacter capsici AZ78]
OG_00473	EYR69523.1	beta-N-acetylhexosaminidase [Lysobacter capsici AZ78]
OG_00474	EYR69668.1	23S rRNA pseudouridylate synthase C [Lysobacter capsici AZ78]
OG_00475	EYR65680.1	(2Fe-2S)-binding protein [Lysobacter capsici AZ78]
OG_00476	EYR69149.1	riboflavin synthase subunit alpha [Lysobacter capsici AZ78]
OG_00477	EYR68127.1	ribokinase [Lysobacter capsici AZ78]
OG_00478	EYR67200.1	LysR family transcriptional regulator [Lysobacter capsici AZ78]
OG_00479	EYR69200.1	mannose-1-phosphate guanylyltransferase [Lysobacter capsici AZ78]
OG_00480	EYR69766.1	short-chain dehydrogenase [Lysobacter capsici AZ78]
OG_00481	EYR66133.1	hypothetical protein AZ78_22455 [Lysobacter capsici AZ78]
OG_00482	EYR69227.1	GNAT family acetyltransferase [Lysobacter capsici AZ78]
OG_00483	EYR68659.1	hypothetical protein AZ78_08500 [Lysobacter capsici AZ78]
OG_00484	EYR67710.1	ribonucleoside-diphosphate reductase [Lysobacter capsici AZ78]
OG_00485	EYR69767.1	AcrA [Lysobacter capsici AZ78]
OG_00486	EYR69121.1	membrane protein [Lysobacter capsici AZ78]
OG_00487	EYR65363.1	CMP-binding protein [Lysobacter capsici AZ78]
OG_00488	EYR68702.1	membrane protein [Lysobacter capsici AZ78]
OG_00489	EYR65392.1	HIT family hydrolase [Lysobacter capsici AZ78]
OG_00490	EYR69342.1	asparaginyl-tRNA synthetase [Lysobacter capsici AZ78]
OG_00491	EYR69559.1	hypothetical protein AZ78_03510 [Lysobacter capsici AZ78]
OG_00492	EYR67470.1	nitrilase [Lysobacter capsici AZ78]
OG_00493	EYR69614.1	small conductance mechanosensitive channel protein MscS [Lysobac
OG_00494	EYR68163.1	7-cyano-7-deazaguanine synthase [Lysobacter capsici AZ78]
OG_00495	EYR67600.1	histidine kinase [Lysobacter capsici AZ78]
OG_00496	EYR68346.1	acriflavin resistance protein [Lysobacter capsici AZ78]

OG_00497	LC55x_581	LC55x_581; conserved hypothetical protein	OG_00497	EYR70018.1	amidohydrolase [Lysobacter capsici AZ78]
OG_00498	LC55x_4354	LC55x_4354; conserved hypothetical protein	OG_00498	EYR69194.1	ABC transporter [Lysobacter capsici AZ78]
OG_00499	LC55x_2039	LC55x_2039; 4Fe-4S binding domain protein	OG_00499	EYR65594.1	ferredoxin [Lysobacter capsici AZ78]
OG_00500	LC55x_1415	LC55x_1415; bacterial regulatory helix-turn-helix, AraC family protein	OG_00500	EYR67156.1	AraC family transcriptional regulator [Lysobacter capsici AZ78]
OG_00501	LC55x_2785	LC55x_2785; putative transmembrane protein	OG_00501	EYR69348.1	hypothetical protein AZ78_05500 [Lysobacter capsici AZ78]
OG_00502	LC55x_1880	LC55x_1880; transcriptional regulator, Spx/MgsR family protein	OG_00502	EYR66072.1	arsenate reductase [Lysobacter capsici AZ78]
OG_00503	LC55x_5219	LC55x_5219; putative nucleotidyltransferase family protein	OG_00503	EYR68881.1	nucleotidyltransferase [Lysobacter capsici AZ78]
OG_00504	LC55x_538	LC55x_538; bacterial extracellular solute-binding, 5 Middle family pro	OG_00504	EYR67898.1	hypothetical protein AZ78_12890 [Lysobacter capsici AZ78]
OG_00505	LC55x_3262	LC55x_3262; dehydroquinase class II family protein	OG_00505	EYR70033.1	3-dehydroquinate dehydratase [Lysobacter capsici AZ78]
OG_00506	LC55x_2136	LC55x_2136; tonB dependent receptor family protein	OG_00506	EYR66373.1	TonB-denpendent receptor [Lysobacter capsici AZ78]
OG_00507	LC55x_1689	LC55x_1689; bacterial regulatory, luxR family protein	OG_00507	EYR70353.1	LuxR family transcriptional regulator [Lysobacter capsici AZ78]
OG_00508	LC55x_4143	LC55x_4143; cheW-like domain protein	OG_00508	EYR66338.1	pilus biogenesis protein [Lysobacter capsici AZ78]
OG_00509	LC55x_1692	LC55x_1692; FHA domain protein	OG_00509	EYR70356.1	diguanylate cyclase [Lysobacter capsici AZ78]
OG_00510	LC55x_5538	LC55x_5538; conserved hypothetical protein	OG_00510	EYR68970.1	acetyltransferase [Lysobacter capsici AZ78]
OG_00511	LC55x_4778	LC55x_4778; bacterial regulatory, gntR family protein	OG_00511	EYR68790.1	GntR family transcriptional regulator [Lysobacter capsici AZ78]
OG_00512	LC55x_1907	LC55x_1907; conserved hypothetical protein	OG_00512	EYR65988.1	hypothetical protein AZ78_23460 [Lysobacter capsici AZ78]
OG_00513	LC55x_2532	ftsK; DNA translocase ftsK	OG_00513	EYR69492.1	cell division protein FtsK [Lysobacter capsici AZ78]
OG_00514	LC55x_608	LC55x_608; small Multidrug Resistance family protein	OG_00514	EYR69997.1	multidrug transporter [Lysobacter capsici AZ78]
OG_00515	LC55x_3386	LC55x_3386; pectinacetylesterase family protein	OG_00515	EYR66529.1	hypothetical protein AZ78_20495 [Lysobacter capsici AZ78]
OG_00516	LC55x_4476	rtcA; RNA 3'-phosphate cyclase	OG_00516	EYR68427.1	RNA 3'-terminal-phosphate cyclase [Lysobacter capsici AZ78]
OG_00517	LC55x_3244	LC55x_3244; elongation factor P (EF-P) OB domain protein	OG_00517	EYR67937.1	elongation factor P [Lysobacter capsici AZ78]
OG_00518	LC55x_2311	LC55x_2311; carbon-nitrogen hydrolase family protein	OG_00518	EYR69653.1	apolipoprotein acyltransferase [Lysobacter capsici AZ78]
OG_00519	LC55x_539	LC55x_539; glutamate--cysteine ligase	OG_00519	EYR67899.1	glutamate--cysteine ligase [Lysobacter capsici AZ78]
OG_00520	LC55x_302	LC55x_302; his Kinase A domain protein	OG_00520	EYR67033.1	nitrogen regulation protein NR(II) [Lysobacter capsici AZ78]
OG_00521	LC55x_4353	LC55x_4353; mce related family protein	OG_00521	EYR69193.1	ABC transporter permease [Lysobacter capsici AZ78]
OG_00522	LC55x_5247	LC55x_5247; phosphoribosyl transferase domain protein	OG_00522	EYR68902.1	competence protein ComF [Lysobacter capsici AZ78]
OG_00523	LC55x_5725	LC55x_5725; ABC transporter family protein	OG_00523	EYR69693.1	ABC transporter ATP-binding protein [Lysobacter capsici AZ78]
OG_00524	LC55x_542	LC55x_542; bacterial regulatory helix-turn-helix , lysR family protein	OG_00524	EYR67902.1	hypothetical protein AZ78_12910 [Lysobacter capsici AZ78]
OG_00525	LC55x_573	LC55x_573; conserved hypothetical protein	OG_00525	EYR70025.1	hypothetical protein AZ78_02590 [Lysobacter capsici AZ78]
OG_00526	LC55x_1087	pgk; phosphoglycerate kinase	OG_00526	EYR68458.1	phosphoglycerate kinase [Lysobacter capsici AZ78]
OG_00527	LC55x_2050	yjeE; essential with weak ATPase activity domain protein	OG_00527	EYR65603.1	ATP-binding protein [Lysobacter capsici AZ78]
OG_00528	LC55x_3589	LC55x_3589; hypothetical protein	OG_00528	EYR67535.1	hypothetical protein AZ78_15425 [Lysobacter capsici AZ78]
OG_00529	LC55x_1616	LC55x_1616; conserved hypothetical protein	OG_00529	EYR70321.1	hypothetical protein AZ78_01135 [Lysobacter capsici AZ78]
OG_00530	LC55x_4219	LC55x_4219; proton antiporter-2 family protein	OG_00530	EYR69080.1	cation:proton antiport protein [Lysobacter capsici AZ78]
OG_00531	LC55x_3716	LC55x_3716; NAD(P)-binding Rossmann-like domain protein	OG_00531	EYR65906.1	D-amino acid dehydrogenase [Lysobacter capsici AZ78]
OG_00532	LC55x_3422	nagZ; beta-hexosaminidase	OG_00532	EYR66506.1	beta-hexosaminidase [Lysobacter capsici AZ78]
OG_00533	LC55x_1819	clpX; ATP-dependent Clp protease, ATP-binding subunit ClpX	OG_00533	EYR67306.1	ATP-dependent protease [Lysobacter capsici AZ78]
OG_00534	LC55x_1390	LC55x_1390; flavodoxin family protein	OG_00534	EYR67178.1	hypothetical protein AZ78_17295 [Lysobacter capsici AZ78]
OG_00535	LC55x_2424	virB11; P-type DNA transfer ATPase VirB11	OG_00535	EYR69574.1	hypothetical protein AZ78_03610 [Lysobacter capsici AZ78]
OG_00536	LC55x_4010	LC55x_4010; hypothetical protein	OG_00536	EYR65565.1	hypothetical protein AZ78_25620 [Lysobacter capsici AZ78]
OG_00537	LC55x_3260	mtnC; 2,3-diketo-5-methylthio-1-phosphopentane phosphatase	OG_00537	EYR67923.1	haloacid dehalogenase [Lysobacter capsici AZ78]
OG_00538	LC55x_2646	LC55x_2646; conserved hypothetical protein	OG_00538	EYR68252.1	hypothetical protein AZ78_11340 [Lysobacter capsici AZ78]
OG_00539	LC55x_887	LC55x_887; bacterial regulatory helix-turn-helix , lysR family protein	OG_00539	EYR66871.1	hypothetical protein AZ78_19005 [Lysobacter capsici AZ78]
OG_00540	LC55x_3772	LC55x_3772; cyclase family protein	OG_00540	EYR67082.1	cyclase [Lysobacter capsici AZ78]
OG_00541	LC55x_2121	cfa; cfa	OG_00541	EYR66385.1	cyclopropane fatty acyl phospholipid synthase [Lysobacter capsici AZ
OG_00542	LC55x_945	LC55x_945; beta-lytic metalloendopeptidase	OG_00542	EYR66817.1	hypothetical protein AZ78_18735 [Lysobacter capsici AZ78]
OG_00543	LC55x_3399	LC55x_3399; diguanylate cyclase domain protein	OG_00543	EYR66519.1	membrane protein [Lysobacter capsici AZ78]
OG_00544	LC55x_3741	LC55x_3741; methyltransferase domain protein	OG_00544	EYR67054.1	methyltransferase [Lysobacter capsici AZ78]
OG_00545	LC55x_3838	gltX; glutamate--tRNA ligase	OG_00545	EYR65741.1	glutaminyl-tRNA synthetase [Lysobacter capsici AZ78]
OG_00546	LC55x_1001	LC55x_1001; bacterial regulatory helix-turn-helix , lysR family protein	OG_00546	EYR67773.1	LysR family transcriptional regulator [Lysobacter capsici AZ78]
OG_00547	LC55x_3258	mtnD; acireductone dioxygenase	OG_00547	EYR67925.1	acireductone dioxygenase [Lysobacter capsici AZ78]
OG_00548	LC55x_4943	LC55x_4943; conserved hypothetical protein	OG_00548	EYR68807.1	hypothetical protein AZ78_08640 [Lysobacter capsici AZ78]
OG_00549	LC55x_1190	gcvT; glycine cleavage system T protein	OG_00549	EYR65793.1	glycine cleavage system aminomethyltransferase T [Lysobacter caps
OG_00550	LC55x_4330	LC55x_4330; DSBA-like thioredoxin domain protein	OG_00550	EYR69174.1	chitinase [Lysobacter capsici AZ78]
OG_00551	LC55x_4924	dprA; DNA protecting protein DprA	OG_00551	EYR68692.1	DNA processing protein DprA [Lysobacter capsici AZ78]
OG_00552	LC55x_1287	LC55x_1287; periplasmic binding family protein	OG_00552	EYR67664.1	ABC transporter substrate-binding protein [Lysobacter capsici AZ78]
OG_00553	LC55x_975	LC55x_975; peptidase M20/M25/M40 family protein	OG_00553	EYR65394.1	peptidase M20 [Lysobacter capsici AZ78]
OG_00554	LC55x_4283	yraL; 16S rRNA 2'-O-ribose C1402 methyltransferase	OG_00554	EYR69139.1	16S rRNA methyltransferase [Lysobacter capsici AZ78]
OG_00555	LC55x_687	LC55x_687; conserved hypothetical protein	OG_00555	EYR69952.1	dehydrogenase [Lysobacter capsici AZ78]
OG_00556	LC55x_1552	LC55x_1552; glycosyl hydrolases 31 family protein	OG_00556	EYR70274.1	glycosyl hydrolase [Lysobacter capsici AZ78]
OG_00557	LC55x_4315	gspD; type II secretion system protein D	OG_00557	EYR69162.1	general secretion pathway protein D [Lysobacter capsici AZ78]
OG_00558	LC55x_3192	LC55x_3192; autotransporter beta-domain protein	OG_00558	EYR66906.1	hypothetical protein AZ78_18205 [Lysobacter capsici AZ78]
OG_00559	LC55x_737	LC55x_737; asnC family protein	OG_00559	EYR67647.1	AsnC family transcriptional regulator [Lysobacter capsici AZ78]
OG_00560	LC55x_920	LC55x_920; bacterial regulatory, tetR family protein	OG_00560	EYR66840.1	hypothetical protein AZ78_18850 [Lysobacter capsici AZ78]
OG_00561	LC55x_376	aceA; isocitrate lyase	OG_00561	EYR68146.1	isocitrate lyase [Lysobacter capsici AZ78]
OG_00562	LC55x_1042	LC55x_1042; AAA domain family protein	OG_00562	EYR67742.1	ATPase AAA [Lysobacter capsici AZ78]
OG_00563	LC55x_332	LC55x_332; GHMP kinase N terminal domain protein	OG_00563	EYR68177.1	hypothetical protein AZ78_11830 [Lysobacter capsici AZ78]
OG_00564	LC55x_2843	LC55x_2843; conserved hypothetical protein	OG_00564	EYR69404.1	hypothetical protein AZ78_05785 [Lysobacter capsici AZ78]
OG_00565	LC55x_795	LC55x_795; ftsX-like permease family protein	OG_00565	EYR67596.1	ABC transporter ATP-binding protein [Lysobacter capsici AZ78]
OG_00566	LC55x_3547	LC55x_3547; N-acetylmuramoyl-L-alanine amidase family protein	OG_00566	EYR67499.1	N-acetylmuramoyl-L-alanine amidase [Lysobacter capsici AZ78]
OG_00567	LC55x_1173	LC55x_1173; diguanylate cyclase domain protein	OG_00567	EYR68539.1	diguanylate cyclase [Lysobacter capsici AZ78]
OG_00568	LC55x_169	LC55x_169; ATPase associated with various cellular activities family	OG_00568	EYR65410.1	ATPase [Lysobacter capsici AZ78]
OG_00569	LC55x_4194	ybgC; tol-pal system-associated acyl-CoA thioesterase	OG_00569	EYR68155.1	tol-pal system-associated acyl-CoA thioesterase [Lysobacter capsici
OG_00570	LC55x_4840	LC55x_4840; methyltransferase domain protein	OG_00570	EYR68754.1	methyltransferase [Lysobacter capsici AZ78]
OG_00571	LC55x_3825	LC55x_3825; alkA N-terminal domain protein	OG_00571	EYR65754.1	DNA methylase [Lysobacter capsici AZ78]
OG_00572	LC55x_1971	ffh; signal recognition particle protein	OG_00572	EYR66774.1	signal recognition particle [Lysobacter capsici AZ78]
OG_00573	LC55x_5419	LC55x_5419; enoyl-CoA hydratase/isomerase family protein	OG_00573	EYR68909.1	enoyl-CoA hydratase [Lysobacter capsici AZ78]
OG_00574	LC55x_1702	yggX; that protects iron-sulfur proteins against oxidative damage	OG_00574	EYR70361.1	iron transporter [Lysobacter capsici AZ78]
OG_00575	LC55x_4347	proS; proline--tRNA ligase	OG_00575	EYR69187.1	prolyl-tRNA synthetase [Lysobacter capsici AZ78]
OG_00576	LC55x_2287	LC55x_2287; bacterial regulatory, luxR family protein	OG_00576	EYR69670.1	response regulator [Lysobacter capsici AZ78]
OG_00577	LC55x_4157	LC55x_4157; putative secreted protein	OG_00577	EYR66324.1	hypothetical protein AZ78_21475 [Lysobacter capsici AZ78]

OG_00578	LC55x_3507	LC55x_3507; peptidase M48 family protein	OG_00578	EYR66633.1	peptidase [Lysobacter capsici AZ78]
OG_00579	LC55x_3949	rpsS; ribosomal protein S19	OG_00579	EYR66700.1	30S ribosomal protein S19 [Lysobacter capsici AZ78]
OG_00580	LC55x_1186	LC55x_1186; RNA polymerase sigma factor, sigma-70 family protein	OG_00580	EYR65797.1	hypothetical protein AZ78_24570 [Lysobacter capsici AZ78]
OG_00581	LC55x_1977	LC55x_1977; hypothetical protein	OG_00581	EYR66787.1	hypothetical protein AZ78_19375 [Lysobacter capsici AZ78]
OG_00582	LC55x_2531	ald; alanine dehydrogenase	OG_00582	EYR69493.1	alanine dehydrogenase [Lysobacter capsici AZ78]
OG_00583	LC55x_1605	miaB; tRNA-i(6)A37 thiotransferase enzyme MiaB	OG_00583	EYR70312.1	(dimethylallyl)adenosine tRNA methylthiotransferase [Lysobacter cap
OG_00584	LC55x_1015	gmk; guanylate kinase	OG_00584	EYR67763.1	guanylate kinase [Lysobacter capsici AZ78]
OG_00585	LC55x_2528	LC55x_2528; acetyltransferase domain protein	OG_00585	EYR69496.1	hypothetical protein AZ78_03095 [Lysobacter capsici AZ78]
OG_00586	LC55x_1548	LC55x_1548; deoR-like helix-turn-helix domain protein	OG_00586	EYR70270.1	DeoR family transcriptional regulator [Lysobacter capsici AZ78]
OG_00587	LC55x_2065	LC55x_2065; cobQ/CobB/MinD/ParA nucleotide binding domain prote	OG_00587	EYR65403.1	hypothetical protein AZ78_26505 [Lysobacter capsici AZ78]
OG_00588	LC55x_3047	LC55x_3047; putative ATPase	OG_00588	EYR67833.1	ATP-binding protein [Lysobacter capsici AZ78]
OG_00589	LC55x_3985	LC55x_3985; conserved hypothetical protein	OG_00589	EYR66715.1	hypothetical protein AZ78_19795 [Lysobacter capsici AZ78]
OG_00590	LC55x_3963	rplA; ribosomal protein L1	OG_00590	EYR65484.1	50S ribosomal protein L1 [Lysobacter capsici AZ78]
OG_00591	LC55x_3063	LC55x_3063; alpha-2-macroglobulin N-terminal region family protein	OG_00591	EYR67845.1	alpha-2-macroglobulin [Lysobacter capsici AZ78]
OG_00592	LC55x_1749	LC55x_1749; peptidase M13 family protein	OG_00592	EYR65700.1	peptidase M13 [Lysobacter capsici AZ78]
OG_00593	LC55x_2332	LC55x_2332; nucleoside diphosphate kinase family protein	OG_00593	EYR69636.1	nucleoside diphosphate kinase [Lysobacter capsici AZ78]
OG_00594	LC55x_4617	LC55x_4617; ftsX-like permease family protein	OG_00594	EYR68343.1	membrane protein [Lysobacter capsici AZ78]
OG_00595	LC55x_2024	kdpD; sensor protein KdpD	OG_00595	EYR66737.1	osmosensitive K channel His kinase sensor [Lysobacter capsici AZ7
OG_00596	LC55x_764	LC55x_764; eamA-like transporter family protein	OG_00596	EYR67625.1	membrane protein [Lysobacter capsici AZ78]
OG_00597	LC55x_1005	pepQ; xaa-Pro dipeptidase	OG_00597	EYR67771.1	proline dipeptidase [Lysobacter capsici AZ78]
OG_00598	LC55x_4271	murG; undecaprenyldiphospho-muramoylpentapeptide beta-N-acetyl	OG_00598	EYR69129.1	UDP-diphospho-muramoylpentapeptide beta-N- acetylglucosaminylt
OG_00599	LC55x_3444	LC55x_3444; yceG-like family protein	OG_00599	EYR66591.1	aminodeoxychorismate lyase [Lysobacter capsici AZ78]
OG_00600	LC55x_3191	LC55x_3191; acyl-CoA dehydrogenase, N-terminal domain protein	OG_00600	EYR66907.1	isovaleryl-CoA dehydrogenase [Lysobacter capsici AZ78]
OG_00601	LC55x_4838	LC55x_4838; conserved hypothetical protein	OG_00601	EYR68756.1	hypothetical protein AZ78_09160 [Lysobacter capsici AZ78]
OG_00602	LC55x_4666	mreD; rod shape-determining protein MreD	OG_00602	EYR70167.1	rod shape-determining protein MreD [Lysobacter capsici AZ78]
OG_00603	LC55x_1799	LC55x_1799; oligomerisation domain protein	OG_00603	EYR67288.1	iojap family protein [Lysobacter capsici AZ78]
OG_00604	LC55x_4248	LC55x_4248; 23S rRNA pseudouridine synthase	OG_00604	EYR69103.1	23S rRNA pseudouridine synthase D [Lysobacter capsici AZ78]
OG_00605	LC55x_3767	LC55x_3767; conserved hypothetical protein	OG_00605	EYR67076.1	glyoxalase [Lysobacter capsici AZ78]
OG_00606	LC55x_259	LC55x_259; YCII-related domain protein	OG_00606	EYR66988.1	hypothetical protein AZ78_17900 [Lysobacter capsici AZ78]
OG_00607	LC55x_4254	LC55x_4254; his Kinase A domain protein	OG_00607	EYR69109.1	ATPase [Lysobacter capsici AZ78]
OG_00608	LC55x_676	LC55x_676; osmC-like family protein	OG_00608	EYR69959.1	peroxiredoxin [Lysobacter capsici AZ78]
OG_00609	LC55x_1261	LC55x_1261; conserved hypothetical protein	OG_00609	EYR65650.1	hypothetical protein AZ78_25225 [Lysobacter capsici AZ78]
OG_00611	LC55x_1625	LC55x_1625; carboxypeptidase regulatory-like domain protein	OG_00611	EYR70328.1	Oar protein [Lysobacter capsici AZ78]
OG_00612	LC55x_2049	LC55x_2049; putative carbohydrate kinase	OG_00612	EYR65602.1	carbohydrate kinase [Lysobacter capsici AZ78]
OG_00613	LC55x_2209	LC55x_2209; glyoxalase/Bleomycin resistance /Dioxygenase superfa	OG_00613	EYR67411.1	glyoxalase [Lysobacter capsici AZ78]
OG_00614	LC55x_4947	LC55x_4947; sigma-70, region 4 family protein	OG_00614	EYR68674.1	membrane protein [Lysobacter capsici AZ78]
OG_00615	LC55x_3081	LC55x_3081; phage Tail Collar domain protein	OG_00615	EYR67857.1	microcystin dependent protein [Lysobacter capsici AZ78]
OG_00616	LC55x_4920	sun; ribosomal RNA small subunit methyltransferase B	OG_00616	EYR68696.1	16S rRNA methyltransferase [Lysobacter capsici AZ78]
OG_00617	LC55x_5175	LC55x_5175; surface antigen family protein	OG_00617	EYR67995.1	membrane protein [Lysobacter capsici AZ78]
OG_00618	LC55x_3335	phbB; acetoacetyl-CoA reductase family protein	OG_00618	EYR66553.1	3-ketoacyl-ACP reductase [Lysobacter capsici AZ78]
OG_00619	LC55x_4142	LC55x_4142; methyl-accepting chemotaxis (MCP) signalling domain	OG_00619	EYR66339.1	pilus biogenesis protein [Lysobacter capsici AZ78]
OG_00620	LC55x_3274	LC55x_3274; diguanylate cyclase domain protein	OG_00620	EYR70136.1	hypothetical protein AZ78_02695 [Lysobacter capsici AZ78]
OG_00621	LC55x_4103	lgt; prolipoprotein diacylglyceryl transferase	OG_00621	EYR65504.1	prolipoprotein diacylglyceryl transferase [Lysobacter capsici AZ78]
OG_00622	LC55x_2752	LC55x_2752; acyltransferase, WS/DGAT/MGAT family protein	OG_00622	EYR69320.1	hypothetical protein AZ78_05360 [Lysobacter capsici AZ78]
OG_00623	LC55x_2468	fur; fur	OG_00623	EYR69542.1	Fur family transcriptional regulator [Lysobacter capsici AZ78]
OG_00624	LC55x_3932	rpsM; 30S ribosomal protein S13	OG_00624	EYR66683.1	30S ribosomal protein S13 [Lysobacter capsici AZ78]
OG_00625	LC55x_856	LC55x_856; hypothetical protein	OG_00625	EYR66900.1	hypothetical protein AZ78_19155 [Lysobacter capsici AZ78]
OG_00626	LC55x_4917	LC55x_4917; glycosyl transferase 2 family protein	OG_00626	EYR68699.1	beta 1,4 glucosyltransferase [Lysobacter capsici AZ78]
OG_00627	LC55x_4080	mtfA; 23S rRNA 2'-O-ribose C2498 methyltransferase	OG_00627	EYR65432.1	23S rRNA methyltransferase [Lysobacter capsici AZ78]
OG_00628	LC55x_279	LC55x_279; integral membrane , TerC family protein	OG_00628	EYR67005.1	membrane protein [Lysobacter capsici AZ78]
OG_00629	LC55x_4504	LC55x_4504; hypothetical protein	OG_00629	EYR68586.1	hypothetical protein AZ78_10105 [Lysobacter capsici AZ78]
OG_00630	LC55x_2351	guaA; guaA GMP synthetase	OG_00630	EYR69621.1	GMP synthase [Lysobacter capsici AZ78]
OG_00631	LC55x_10	LC55x_10; motA/TolQ/ExbB proton channel family protein	OG_00631	EYR69713.1	biopolymer transporter ExbB [Lysobacter capsici AZ78]
OG_00632	LC55x_794	LC55x_794; ftsX-like permease family protein	OG_00632	EYR67597.1	ABC transporter permease [Lysobacter capsici AZ78]
OG_00633	LC55x_543	LC55x_543; tetratricopeptide repeat family protein	OG_00633	EYR67903.1	hypothetical protein AZ78_12915 [Lysobacter capsici AZ78]
OG_00634	LC55x_4983	LC55x_4983; hypothetical protein	OG_00634	EYR67245.1	hypothetical protein AZ78_16875 [Lysobacter capsici AZ78]
OG_00635	LC55x_4391	maiA; maleylacetoacetate isomerase	OG_00635	EYR69224.1	maleylacetoacetate isomerase [Lysobacter capsici AZ78]
OG_00636	LC55x_1182	LC55x_1182; conserved hypothetical protein	OG_00636	EYR65800.1	hypothetical protein AZ78_24585 [Lysobacter capsici AZ78]
OG_00637	LC55x_468	LC55x_468; outer membrane beta-barrel domain protein	OG_00637	EYR66488.1	hypothetical protein AZ78_21035 [Lysobacter capsici AZ78]
OG_00638	LC55x_220	LC55x_220; ahpC/TSA family protein	OG_00638	EYR66161.1	photosynthetic protein synthase I [Lysobacter capsici AZ78]
OG_00639	LC55x_2184	msbA; lipid A export permease/ATP-binding protein MsbA	OG_00639	EYR67401.1	ATP-binding protein [Lysobacter capsici AZ78]
OG_00640	LC55x_816	LC55x_816; conserved hypothetical protein	OG_00640	EYR67686.1	hypothetical protein AZ78_14475 [Lysobacter capsici AZ78]
OG_00641	LC55x_1567	LC55x_1567; oligopeptide transporter, OPT family	OG_00641	EYR70286.1	peptide transporter [Lysobacter capsici AZ78]
OG_00642	LC55x_164	LC55x_164; histidine phosphatase super family protein	OG_00642	EYR65364.1	phosphoglycerate mutase [Lysobacter capsici AZ78]
OG_00643	LC55x_2473	dnaK; chaperone protein DnaK	OG_00643	EYR69538.1	molecular chaperone DnaK [Lysobacter capsici AZ78]
OG_00644	LC55x_4921	fmt; methionyl-tRNA formyltransferase	OG_00644	EYR68695.1	methionyl-tRNA formyltransferase [Lysobacter capsici AZ78]
OG_00645	LC55x_1431	LC55x_1431; alpha/beta hydrolase family protein	OG_00645	EYR67144.1	hypothetical protein AZ78_17125 [Lysobacter capsici AZ78]
OG_00646	LC55x_416	LC55x_416; sigma-54 interaction domain protein	OG_00646	EYR65622.1	Fis family transcriptional regulator [Lysobacter capsici AZ78]
OG_00647	LC55x_3921	pqqE; coenzyme PQQ biosynthesis enzyme PqqE	OG_00647	EYR66673.1	pyrroloquinoline quinone biosynthesis protein PqqE [Lysobacter caps
OG_00648	LC55x_3531	LC55x_3531; acetyltransferase family protein	OG_00648	EYR67483.1	acyl-CoA synthetase [Lysobacter capsici AZ78]
OG_00649	LC55x_3019	tpiA; triose-phosphate isomerase	OG_00649	EYR67816.1	triosephosphate isomerase [Lysobacter capsici AZ78]
OG_00650	LC55x_2755	alr; alanine racemase	OG_00650	EYR69323.1	alanine racemase [Lysobacter capsici AZ78]
OG_00651	LC55x_2375	LC55x_2375; putative calU5	OG_00651	EYR69609.1	radical SAM protein [Lysobacter capsici AZ78]
OG_00652	LC55x_2913	LC55x_2913; conserved hypothetical protein	OG_00652	EYR69469.1	hypothetical protein AZ78_06110 [Lysobacter capsici AZ78]
OG_00653	LC55x_4152	LC55x_4152; DEAD/DEAH box helicase family protein	OG_00653	EYR66329.1	helicase [Lysobacter capsici AZ78]
OG_00654	LC55x_2016	recX; regulatory recX domain protein	OG_00654	EYR66742.1	recombination protein RecX [Lysobacter capsici AZ78]
OG_00655	LC55x_3123	LC55x_3123; PAAR motif family protein	OG_00655	EYR66971.1	hypothetical protein AZ78_18545 [Lysobacter capsici AZ78]
OG_00656	LC55x_653	LC55x_653; hypothetical protein	OG_00656	EYR70117.1	hypothetical protein AZ78_02220 [Lysobacter capsici AZ78]
OG_00657	LC55x_2536	LC55x_2536; alpha/beta hydrolase fold family protein	OG_00657	EYR69488.1	3-oxoadipate enol-lactonase [Lysobacter capsici AZ78]
OG_00658	LC55x_2378	LC55x_2378; hypothetical protein	OG_00658	EYR69834.1	hypothetical protein AZ78_03830 [Lysobacter capsici AZ78]
OG_00659	LC55x_417	LC55x_417; membrane fusogenic activity family protein	OG_00659	EYR65621.1	hypothetical protein AZ78_25285 [Lysobacter capsici AZ78]

OG_00660	LC55x_4598	LC55x_4598; TSCPD domain protein	OG_00660	EYR68641.1	NrdJb [Lysobacter capsici AZ78]
OG_00661	LC55x_4493	LC55x_4493; conserved hypothetical protein	OG_00661	EYR68417.1	hypothetical protein AZ78_10155 [Lysobacter capsici AZ78]
OG_00662	LC55x_3102	LC55x_3102; sugar (and other) transporter family protein	OG_00662	EYR65451.1	MFS transporter [Lysobacter capsici AZ78]
OG_00663	LC55x_2091	LC55x_2091; deoR-like helix-turn-helix domain protein	OG_00663	EYR66414.1	DeoR faimly transcriptional regulator [Lysobacter capsici AZ78]
OG_00664	LC55x_315	LC55x_315; hemY family protein	OG_00664	EYR68098.1	porphyrin biosynthesis protein [Lysobacter capsici AZ78]
OG_00665	LC55x_5208	LC55x_5208; bacterial regulatory helix-turn-helix, AraC family protein	OG_00665	EYR68873.1	AraC family transcriptional regulator [Lysobacter capsici AZ78]
OG_00666	LC55x_688	LC55x_688; conserved hypothetical protein	OG_00666	EYR69951.1	SAM-binding protein [Lysobacter capsici AZ78]
OG_00667	LC55x_5016	gcvA; gcvA transcriptional dual regulator	OG_00667	EYR67214.1	LysR family transcriptional regulator [Lysobacter capsici AZ78]
OG_00668	LC55x_4694	LC55x_4694; putative PEPTIDASE	OG_00668	EYR70191.1	prolyl oligopeptidase [Lysobacter capsici AZ78]
OG_00669	LC55x_859	LC55x_859; CDP-alcohol phosphatidyltransferase family protein	OG_00669	EYR66898.1	CDP-alcohol phosphatidyltransferase [Lysobacter capsici AZ78]
OG_00670	LC55x_3271	LC55x_3271; hisD	OG_00670	EYR70042.1	bifunctional histidinal dehydrogenase/ histidinol dehydrogenase [Lyso
OG_00671	LC55x_4039	LC55x_4039; conserved hypothetical protein	OG_00671	EYR65818.1	hypothetical protein AZ78_24210 [Lysobacter capsici AZ78]
OG_00672	LC55x_1596	LC55x_1596; hlyD secretion family protein	OG_00672	EYR70306.1	hemolysin secretion protein D [Lysobacter capsici AZ78]
OG_00673	LC55x_1438	LC55x_1438; aspartate kinase domain protein	OG_00673	EYR67138.1	aspartate kinase [Lysobacter capsici AZ78]
OG_00674	LC55x_1430	LC55x_1430; peroxiredoxin, OsmC subfamily protein	OG_00674	EYR67145.1	peroxiredoxin [Lysobacter capsici AZ78]
OG_00675	LC55x_2467	smpA; outer membrane protein	OG_00675	EYR69543.1	membrane protein SmpA [Lysobacter capsici AZ78]
OG_00676	LC55x_966	LC55x_966; glyoxalase/Bleomycin resistance /Dioxygenase superfan	OG_00676	EYR66799.1	glyxoylase [Lysobacter capsici AZ78]
OG_00677	LC55x_568	LC55x_568; putative transmembrane domain protein	OG_00677	EYR70029.1	membrane protein [Lysobacter capsici AZ78]
OG_00678	LC55x_2534	LC55x_2534; acetyltransferase family protein	OG_00678	EYR69490.1	GNAT family acetyltraansferase [Lysobacter capsici AZ78]
OG_00679	LC55x_2155	LC55x_2155; conserved hypothetical protein	OG_00679	EYR67375.1	membrane protein [Lysobacter capsici AZ78]
OG_00680	LC55x_5027	ftsE; cell division ATP-binding protein FtsE	OG_00680	EYR66314.1	cell division protein FtsE [Lysobacter capsici AZ78]
OG_00681	LC55x_3396	xseA; exodeoxyribonuclease VII, large subunit	OG_00681	EYR66523.1	exodeoxyribonuclease VII large subunit [Lysobacter capsici AZ78]
OG_00682	LC55x_2487	LC55x_2487; N(4)-(Beta-N-acetylglucosaminyl)-L-asparaginase	OG_00682	EYR69527.1	N(4)-(beta-N-acetylglucosaminyl)-L-asparaginase [Lysobacter capsic
OG_00683	LC55x_1022	LC55x_1022; right handed beta helix region family protein	OG_00683	EYR67758.1	hypothetical protein AZ78_14170 [Lysobacter capsici AZ78]
OG_00684	LC55x_5044	LC55x_5044; coproporphyrinogen III oxidase family protein	OG_00684	EYR65572.1	coproporphyrinogen III oxidase [Lysobacter capsici AZ78]
OG_00686	LC55x_426	LC55x_426; hypothetical protein	OG_00686	EYR65513.1	hypothetical protein AZ78_25805 [Lysobacter capsici AZ78]
OG_00687	LC55x_927	leuB; 3-isopropylmalate dehydrogenase	OG_00687	EYR66833.1	3-isopropylmalate dehydrogenase [Lysobacter capsici AZ78]
OG_00688	LC55x_2783	LC55x_2783; putative transmembrane protein	OG_00688	EYR69347.1	hypothetical protein AZ78_05495 [Lysobacter capsici AZ78]
OG_00689	LC55x_5009	LC55x_5009; PAP2 superfamily protein	OG_00689	EYR67221.1	hypothetical protein AZ78_16755 [Lysobacter capsici AZ78]
OG_00690	LC55x_334	LC55x_334; carbamoyl-phosphate synthase L chain, ATP binding dor	OG_00690	EYR68112.1	carboxylate--amine ligase [Lysobacter capsici AZ78]
OG_00691	LC55x_3012	nuoE; NADH-quinone oxidoreductase, E subunit	OG_00691	EYR67810.1	NADH dehydrogenase subunit E [Lysobacter capsici AZ78]
OG_00692	LC55x_590	LC55x_590; putative aminotransferase	OG_00692	EYR70011.1	aminotransferase [Lysobacter capsici AZ78]
OG_00693	LC55x_189	gluP; glucose/galactose transporter WARNING family protein	OG_00693	EYR66129.1	major facilitator transporter [Lysobacter capsici AZ78]
OG_00694	LC55x_154	edd; phosphogluconate dehydratase	OG_00694	EYR69797.1	phosphogluconate dehydratase [Lysobacter capsici AZ78]
OG_00695	LC55x_1317	LC55x_1317; EF hand family protein	OG_00695	EYR69918.1	hypothetical protein AZ78_01795 [Lysobacter capsici AZ78]
OG_00696	LC55x_1232	mtgA; monofunctional biosynthetic peptidoglycan transglycosylase	OG_00696	EYR65385.1	peptidoglycan transglycosylase [Lysobacter capsici AZ78]
OG_00697	LC55x_1657	uvrB; excinuclease ABC subunit B	OG_00697	EYR70334.1	excinuclease ABC subunit B [Lysobacter capsici AZ78]
OG_00698	LC55x_865	LC55x_865; acyltransferase family protein	OG_00698	EYR66892.1	1-acyl-sn-glycerol-3-phosphate acyltransferase [Lysobacter capsici A
OG_00699	LC55x_963	LC55x_963; V-type H(+)-translocating pyrophosphatase	OG_00699	EYR66802.1	pyrophosphatase [Lysobacter capsici AZ78]
OG_00700	LC55x_3356	LC55x_3356; glutamine cyclotransferase	OG_00700	EYR66539.1	glutamine cyclotransferase [Lysobacter capsici AZ78]
OG_00701	LC55x_4380	LC55x_4380; polysaccharide biosynthesis family protein	OG_00701	EYR69214.1	NAD-dependent dehydratase [Lysobacter capsici AZ78]
OG_00702	LC55x_3538	LC55x_3538; pirin family protein	OG_00702	EYR67490.1	pirin [Lysobacter capsici AZ78]
OG_00703	LC55x_2706	LC55x_2706; endoglucanase-related protein	OG_00703	EYR69292.1	glycosyl hydrolase [Lysobacter capsici AZ78]
OG_00704	LC55x_3582	LC55x_3582; acetyltransferase family protein	OG_00704	EYR67529.1	acetyltransferase [Lysobacter capsici AZ78]
OG_00705	LC55x_4720	LC55x_4720; conserved hypothetical protein	OG_00705	EYR65682.1	hypothetical protein AZ78_25105 [Lysobacter capsici AZ78]
OG_00706	LC55x_2874	LC55x_2874; quinone oxidoreductase, YhdH/YhfP family protein	OG_00706	EYR69433.1	quinone oxidoreductase [Lysobacter capsici AZ78]
OG_00707	LC55x_4343	LC55x_4343; hypothetical protein	OG_00707	EYR69184.1	alanine acetyltransferase [Lysobacter capsici AZ78]
OG_00708	LC55x_4225	LC55x_4225; cobinamide kinase / cobinamide phosphate guanyltrans	OG_00708	EYR69086.1	adenosylcobinamide kinase [Lysobacter capsici AZ78]
OG_00709	LC55x_1827	rlmH; rRNA large subunit m3Psi methyltransferase RlmH	OG_00709	EYR67310.1	50S rRNA methyltransferase [Lysobacter capsici AZ78]
OG_00710	LC55x_353	LC55x_353; tonB dependent receptor family protein	OG_00710	EYR68180.1	hypothetical protein AZ78_11935 [Lysobacter capsici AZ78]
OG_00711	LC55x_3563	LC55x_3563; putative aDP-ribosylglycohydrolase	OG_00711	EYR67513.1	ADP-ribosylation/crystallin J1 [Lysobacter capsici AZ78]
OG_00712	LC55x_3306	argB; acetylglutamate kinase	OG_00712	EYR70063.1	acetylglutamate kinase [Lysobacter capsici AZ78]
OG_00713	LC55x_4985	LC55x_4985; disulfide bond reductase	OG_00713	EYR67243.1	glutathione S-transferase [Lysobacter capsici AZ78]
OG_00714	LC55x_940	thrC; threonine synthase	OG_00714	EYR66821.1	threonine synthase [Lysobacter capsici AZ78]
OG_00715	LC55x_5138	LC55x_5138; cobQ/CobB/MinD/ParA nucleotide binding domain prote	OG_00715	EYR68022.1	chromosome partitioning protein ParA [Lysobacter capsici AZ78]
OG_00716	LC55x_3040	psd; phosphatidylserine decarboxylase	OG_00716	EYR67829.1	phosphatidylserine decarboxylase [Lysobacter capsici AZ78]
OG_00717	LC55x_2034	LC55x_2034; bacterioferritin comigratory protein	OG_00717	EYR65590.1	peroxiredoxin [Lysobacter capsici AZ78]
OG_00718	LC55x_1611	sspA; stringent starvation protein A	OG_00718	EYR70317.1	stringent starvation protein A [Lysobacter capsici AZ78]
OG_00719	LC55x_681	LC55x_681; peptidase propeptide and YPEB domain protein	OG_00719	EYR69956.1	hypothetical protein AZ78_02085 [Lysobacter capsici AZ78]
OG_00720	LC55x_3774	LC55x_3774; bacterial regulatory helix-turn-helix , lysR family protein	OG_00720	EYR67084.1	LysR family transcriptional regulator [Lysobacter capsici AZ78]
OG_00721	LC55x_2623	LC55x_2623; bacterial regulatory , Fis family protein	OG_00721	EYR68273.1	chemotaxis protein CheY [Lysobacter capsici AZ78]
OG_00722	LC55x_4360	LC55x_4360; 3-oxoacid CoA-transferase, B subunit	OG_00722	EYR69197.1	succinyl-CoA:3-ketoacid-CoA transferase [Lysobacter capsici AZ78]
OG_00723	LC55x_1043	LC55x_1043; conserved hypothetical protein	OG_00723	EYR67741.1	ATPase [Lysobacter capsici AZ78]
OG_00724	LC55x_1974	trmD; tRNA (guanine(37)-N(1))-methyltransferase	OG_00724	EYR66770.1	tRNA (guanine-N1)-methyltransferase [Lysobacter capsici AZ78]
OG_00725	LC55x_281	LC55x_281; DNA alkylation repair enzyme family protein	OG_00725	EYR67007.1	hypothetical protein AZ78_18000 [Lysobacter capsici AZ78]
OG_00726	LC55x_1924	LC55x_1924; enoyl-CoA hydratase/isomerase family protein	OG_00726	EYR65975.1	enoyl-CoA hydratase [Lysobacter capsici AZ78]
OG_00727	LC55x_3835	LC55x_3835; bacterial regulatory , arsR family protein	OG_00727	EYR65744.1	hypothetical protein AZ78_24695 [Lysobacter capsici AZ78]
OG_00728	LC55x_2060	LC55x_2060; electron transport complex, RnfABCDGE type, B subur	OG_00728	EYR65407.1	ferredoxin [Lysobacter capsici AZ78]
OG_00729	LC55x_4257	pilB; type IV-A pilus assembly ATPase PilB	OG_00729	EYR69115.1	type II secretory protein GspE [Lysobacter capsici AZ78]
OG_00730	LC55x_3888	LC55x_3888; cell Wall Hydrolase family protein	OG_00730	EYR65721.1	cell wall hydrolase [Lysobacter capsici AZ78]
OG_00731	LC55x_2041	pcnB; poly(A) polymerase family protein	OG_00731	EYR65595.1	polynucleotide adenylyltransferase [Lysobacter capsici AZ78]
OG_00732	LC55x_3516	LC55x_3516; glycosyl transferase 2 family protein	OG_00732	EYR66640.1	glucosyltransferase [Lysobacter capsici AZ78]
OG_00733	LC55x_1687	LC55x_1687; acyl-CoA dehydrogenase, N-terminal domain protein	OG_00733	EYR70351.1	acyl-CoA dehydrogenase [Lysobacter capsici AZ78]
OG_00734	LC55x_3750	LC55x_3750; cyclophilin type peptidyl-prolyl cis-trans isomerase/CLD	OG_00734	EYR67062.1	peptidylprolyl isomerase [Lysobacter capsici AZ78]
OG_00735	LC55x_3305	LC55x_3305; acetyltransferase family protein	OG_00735	EYR70062.1	acetyltransferase [Lysobacter capsici AZ78]
OG_00736	LC55x_3931	rpsK; 30S ribosomal protein S11	OG_00736	EYR66682.1	30S ribosomal protein S11 [Lysobacter capsici AZ78]
OG_00737	LC55x_4751	LC55x_4751; SH3 domain of the SH3b1 type family protein	OG_00737	EYR66095.1	hypothetical protein AZ78_22825 [Lysobacter capsici AZ78]
OG_00738	LC55x_2095	LC55x_2095; conserved hypothetical protein	OG_00738	EYR66410.1	hypothetical protein AZ78_21355 [Lysobacter capsici AZ78]
OG_00739	LC55x_5159	LC55x_5159; uracil DNA glycosylase superfamily protein	OG_00739	EYR68006.1	IclR family transcriptional regulator [Lysobacter capsici AZ78]
OG_00740	LC55x_5089	LC55x_5089; putative fatty acyl-CoA synthetase	OG_00740	EYR68059.1	fatty acyl CoA synthetase [Lysobacter capsici AZ78]
OG_00741	LC55x_996	LC55x_996; cell division ZapA family protein	OG_00741	EYR67778.1	cell division ZapA family protein [Lysobacter capsici AZ78]

OG_00742	LC55x_185	LC55x_185; glycosyl hydrolase family 20, catalytic domain protein	OG_00742	EYR66126.1	hypothetical protein AZ78_22420 [Lysobacter capsici AZ78]
OG_00743	LC55x_4839	LC55x_4839; cupin domain protein	OG_00743	EYR68755.1	cupin [Lysobacter capsici AZ78]
OG_00744	LC55x_1624	LC55x_1624; marC integral membrane family protein	OG_00744	EYR70327.1	membrane protein [Lysobacter capsici AZ78]
OG_00745	LC55x_5015	LC55x_5015; NADPH-dependent FMN reductase family protein	OG_00745	EYR67215.1	NAD(P)H dehydrogenase (quinone) [Lysobacter capsici AZ78]
OG_00746	LC55x_4287	LC55x_4287; conserved hypothetical protein	OG_00746	EYR69142.1	membrane protein [Lysobacter capsici AZ78]
OG_00747	LC55x_100	recC; exodeoxyribonuclease V, gamma subunit	OG_00747	EYR69763.1	exodeoxyribonuclease V subunit gamma [Lysobacter capsici AZ78]
OG_00748	LC55x_2421	LC55x_2421; virB8 family protein	OG_00748	EYR69577.1	conjugative transfer protein [Lysobacter capsici AZ78]
OG_00749	LC55x_4436	LC55x_4436; hypothetical protein	OG_00749	EYR65477.1	hypothetical protein AZ78_26150 [Lysobacter capsici AZ78]
OG_00750	LC55x_4936	LC55x_4936; putative secreted protein	OG_00750	EYR68682.1	hypothetical protein AZ78_08675 [Lysobacter capsici AZ78]
OG_00751	LC55x_4425	LC55x_4425; dnaJ domain protein	OG_00751	EYR65466.1	membrane protein [Lysobacter capsici AZ78]
OG_00752	LC55x_431	LC55x_431; flavofamily protein	OG_00752	EYR65517.1	hypothetical protein AZ78_25825 [Lysobacter capsici AZ78]
OG_00753	LC55x_1982	LC55x_1982; S4 domain protein	OG_00753	EYR66767.1	ribosome-associated heat shock protein Hsp15 [Lysobacter capsici A
OG_00754	LC55x_3284	LC55x_3284; hypothetical protein	OG_00754	EYR70050.1	hypothetical protein AZ78_02750 [Lysobacter capsici AZ78]
OG_00755	LC55x_2483	greA; transcription elongation factor GreA	OG_00755	EYR69531.1	transcription elongation factor GreA [Lysobacter capsici AZ78]
OG_00756	LC55x_2580	LC55x_2580; conserved hypothetical protein	OG_00756	EYR68314.1	hypothetical protein AZ78_11655 [Lysobacter capsici AZ78]
OG_00757	LC55x_363	LC55x_363; SCP-2 sterol transfer family protein	OG_00757	EYR68136.1	sterol-binding protein [Lysobacter capsici AZ78]
OG_00758	LC55x_2666	LC55x_2666; response regulator	OG_00758	EYR68232.1	hypothetical protein AZ78_11240 [Lysobacter capsici AZ78]
OG_00759	LC55x_4118	LC55x_4118; his Kinase A domain protein	OG_00759	EYR66362.1	hypothetical protein AZ78_21665 [Lysobacter capsici AZ78]
OG_00760	LC55x_4531	LC55x_4531; polyketide cyclase / dehydrase and lipid transport famil	OG_00760	EYR68577.1	hypothetical protein AZ78_09970 [Lysobacter capsici AZ78]
OG_00761	LC55x_2254	LC55x_2254; oxidoreductase molybdopterin binding domain protein	OG_00761	EYR67442.1	sulfoxide reductase catalytic subunit YedY [Lysobacter capsici AZ78]
OG_00762	LC55x_113	SRY1; threo-3-hydroxyaspartate ammonia-lyase	OG_00762	EYR69772.1	serine/threonine dehydratase [Lysobacter capsici AZ78]
OG_00763	LC55x_5686	LC55x_5686; putative protein YchJ	OG_00763	EYR65872.1	hypothetical protein AZ78_24050 [Lysobacter capsici AZ78]
OG_00764	LC55x_4095	ksgA; dimethyladenosine transferase	OG_00764	EYR65496.1	16S rRNA methyltransferase [Lysobacter capsici AZ78]
OG_00765	LC55x_4087	LC55x_4087; putative transmembrane protein	OG_00765	EYR65439.1	membrane protein [Lysobacter capsici AZ78]
OG_00766	LC55x_4941	sppA; signal peptide peptidase SppA, 67K type	OG_00766	EYR68679.1	endopeptidase IV [Lysobacter capsici AZ78]
OG_00767	LC55x_919	LC55x_919; methyltransferase domain protein	OG_00767	EYR66841.1	protein-L-isoaspartate O-methyltransferase [Lysobacter capsici AZ78
OG_00768	LC55x_4052	LC55x_4052; NUDIX domain protein	OG_00768	EYR65831.1	DNA mismatch repair protein MutT [Lysobacter capsici AZ78]
OG_00769	LC55x_2343	LC55x_2343; endoribonuclease L-PSP family protein	OG_00769	EYR69627.1	translation initiation inhibitor [Lysobacter capsici AZ78]
OG_00770	LC55x_4738	LC55x_4738; conserved hypothetical protein	OG_00770	EYR66106.1	hypothetical protein AZ78_22880 [Lysobacter capsici AZ78]
OG_00771	LC55x_4748	LC55x_4748; beta-lactamase family protein	OG_00771	EYR66098.1	penicillin-binding protein 4 [Lysobacter capsici AZ78]
OG_00772	LC55x_1066	LC55x_1066; acetyl-CoA hydrolase/transferase C-terminal domain pr	OG_00772	EYR67723.1	acetyl-CoA hydrolase [Lysobacter capsici AZ78]
OG_00773	LC55x_1263	LC55x_1263; conserved hypothetical protein	OG_00773	EYR65652.1	glutaredoxin [Lysobacter capsici AZ78]
OG_00774	LC55x_1241	prfC; peptide chain release factor 3	OG_00774	EYR65637.1	peptide chain release factor 3 [Lysobacter capsici AZ78]
OG_00775	LC55x_1401	sufC; feS assembly ATPase SufC	OG_00775	EYR67167.1	transporter [Lysobacter capsici AZ78]
OG_00776	LC55x_4033	LC55x_4033; putative membrane protein	OG_00776	EYR65812.1	hypothetical protein AZ78_24180 [Lysobacter capsici AZ78]
OG_00777	LC55x_1902	LC55x_1902; small Multidrug Resistance family protein	OG_00777	EYR65992.1	multidrug transporter [Lysobacter capsici AZ78]
OG_00778	LC55x_2915	ligA; DNA ligase, NAD-dependent	OG_00778	EYR69471.1	NAD-dependent DNA ligase LigA [Lysobacter capsici AZ78]
OG_00779	LC55x_2427	LC55x_2427; type IV secretory pathway, VirB3-like family protein	OG_00779	EYR69571.1	hypothetical protein AZ78_03595 [Lysobacter capsici AZ78]
OG_00780	LC55x_4807	PPI; protein-tyrosine-phosphatase	OG_00780	EYR68774.1	metallophosphoesterase [Lysobacter capsici AZ78]
OG_00781	LC55x_3885	LC55x_3885; conserved hypothetical protein	OG_00781	EYR65718.1	hypothetical protein AZ78_24800 [Lysobacter capsici AZ78]
OG_00782	LC55x_2922	LC55x_2922; periplasmic binding and sugar binding domain of LacI f	OG_00782	EYR69477.1	LacI family transcriptional regulator [Lysobacter capsici AZ78]
OG_00783	LC55x_2936	LC55x_2936; 6-phosphogluconolactonase	OG_00783	EYR66224.1	hypothetical protein AZ78_21890 [Lysobacter capsici AZ78]
OG_00784	LC55x_1780	LC55x_1780; conserved hypothetical protein	OG_00784	EYR67277.1	membrane protein [Lysobacter capsici AZ78]
OG_00785	LC55x_792	LC55x_792; AAA domain family protein	OG_00785	EYR67599.1	histidine kinase [Lysobacter capsici AZ78]
OG_00786	LC55x_1797	holA; DNA polymerase III, delta subunit	OG_00786	EYR67286.1	DNA polymerase III subunit delta [Lysobacter capsici AZ78]
OG_00787	LC55x_805	LC55x_805; O-methyltransferase family protein	OG_00787	EYR67591.1	O-methyltransferase [Lysobacter capsici AZ78]
OG_00788	LC55x_1483	ssb; single-stranded DNA-binding family protein	OG_00788	EYR70212.1	single-stranded DNA-binding protein [Lysobacter capsici AZ78]
OG_00789	LC55x_4222	cobS; cobalamin 5'-phosphate synthase	OG_00789	EYR69083.1	cobalamin synthase [Lysobacter capsici AZ78]
OG_00790	LC55x_1395	LC55x_1395; 2OG-Fe(II) oxygenase superfamily protein	OG_00790	EYR67173.1	Fe(II)-dependent oxygenase [Lysobacter capsici AZ78]
OG_00791	LC55x_1356	LC55x_1356; conserved hypothetical protein	OG_00791	EYR69893.1	hypothetical protein AZ78_01630 [Lysobacter capsici AZ78]
OG_00792	LC55x_4725	LC55x_4725; glyoxalase/Bleomycin resistance /Dioxygenase superfa	OG_00792	EYR66119.1	hypothetical protein AZ78_22945 [Lysobacter capsici AZ78]
OG_00793	LC55x_4316	xpsN; general secretion pathway protein N	OG_00793	EYR69163.1	general secretion pathway protein N [Lysobacter capsici AZ78]
OG_00794	LC55x_3503	LC55x_3503; isocitrate/isopropylmalate dehydrogenase family protei	OG_00794	EYR66631.1	isocitrate dehydrogenase [Lysobacter capsici AZ78]
OG_00795	LC55x_5001	LC55x_5001; HAMP domain protein	OG_00795	EYR67228.1	hypothetical protein AZ78_16790 [Lysobacter capsici AZ78]
OG_00796	LC55x_4915	LC55x_4915; bacterial PH domain protein	OG_00796	EYR68701.1	membrane protein [Lysobacter capsici AZ78]
OG_00797	LC55x_2959	LC55x_2959; type IV secretory system Conjugative DNA transfer fam	OG_00797	EYR66204.1	hypothetical protein AZ78_21785 [Lysobacter capsici AZ78]
OG_00798	LC55x_163	LC55x_163; yceI-like domain protein	OG_00798	EYR65365.1	hypothetical protein AZ78_26785 [Lysobacter capsici AZ78]
OG_00799	LC55x_5205	LC55x_5205; type VII secretion system (T7SS), usher family protein	OG_00799	EYR68870.1	fimbriae usher protein [Lysobacter capsici AZ78]
OG_00800	LC55x_5052	LC55x_5052; hypothetical protein	OG_00800	EYR65579.1	hypothetical protein AZ78_25520 [Lysobacter capsici AZ78]
OG_00801	LC55x_4935	LC55x_4935; conserved hypothetical protein	OG_00801	EYR68683.1	hypothetical protein AZ78_08680 [Lysobacter capsici AZ78]
OG_00802	LC55x_2111	LC55x_2111; glutathione S-transferase, C-terminal domain protein	OG_00802	EYR66395.1	hypothetical protein AZ78_21280 [Lysobacter capsici AZ78]
OG_00803	LC55x_34	LC55x_34; sensory box protein	OG_00803	EYR69728.1	hisitidine kinase [Lysobacter capsici AZ78]
OG_00804	LC55x_623	LC55x_623; conserved hypothetical protein	OG_00804	EYR69991.1	3-demethylubiquinone-9 3-methyltransferase [Lysobacter capsici AZ7
OG_00805	LC55x_3677	LC55x_3677; conserved hypothetical protein	OG_00805	EYR65940.1	hypothetical protein AZ78_23820 [Lysobacter capsici AZ78]
OG_00806	LC55x_1671	ihfA; integration host factor, alpha subunit	OG_00806	EYR70342.1	integration host factor subunit alpha [Lysobacter capsici AZ78]
OG_00807	LC55x_2971	LC55x_2971; his Kinase A domain protein	OG_00807	EYR66191.1	hypothetical protein AZ78_21720 [Lysobacter capsici AZ78]
OG_00808	LC55x_2465	LC55x_2465; polyketide cyclase / dehydrase and lipid transport famil	OG_00808	EYR69545.1	oligoketide cyclase [Lysobacter capsici AZ78]
OG_00809	LC55x_4602	LC55x_4602; peptidase Do family protein	OG_00809	EYR68355.1	heat-shock protein [Lysobacter capsici AZ78]
OG_00810	LC55x_2472	grpE; protein grpE	OG_00810	EYR69539.1	heat shock protein GrpE [Lysobacter capsici AZ78]
OG_00811	LC55x_1027	LC55x_1027; conserved hypothetical protein	OG_00811	EYR67787.1	hypothetical protein AZ78_14145 [Lysobacter capsici AZ78]
OG_00812	LC55x_4228	cobD; cobalamin biosynthesis protein CobD	OG_00812	EYR69089.1	cobalamin biosynthesis protein CobD [Lysobacter capsici AZ78]
OG_00813	LC55x_5183	LC55x_5183; RDD family protein	OG_00813	EYR68855.1	hypothetical protein AZ78_07265 [Lysobacter capsici AZ78]
OG_00814	LC55x_2314	rpmJ; ribosomal protein L36	OG_00814	EYR69650.1	50S ribosomal protein L36 [Lysobacter capsici AZ78]
OG_00815	LC55x_5676	LC55x_5676; amidohydrolase family protein	OG_00815	EYR65878.1	N-acyl-L-amino acid amidohydrolase [Lysobacter capsici AZ78]
OG_00816	LC55x_1315	LC55x_1315; PTS system fructose IIA component family protein	OG_00816	EYR69919.1	PTS system sugar transporter subunit IIA [Lysobacter capsici AZ78]
OG_00817	LC55x_3595	LC55x_3595; cupin domain protein	OG_00817	EYR67539.1	AraC family transcriptional regulator [Lysobacter capsici AZ78]
OG_00818	LC55x_4608	LC55x_4608; HAD hydrolase, IA, variant 1 family protein	OG_00818	EYR68349.1	haloacid dehalogenase [Lysobacter capsici AZ78]
OG_00819	LC55x_3796	LC55x_3796; zinc-binding dehydrogenase family protein	OG_00819	EYR67103.1	alcohol dehydrogenase [Lysobacter capsici AZ78]
OG_00820	LC55x_3804	LC55x_3804; DNA methylase family protein	OG_00820	EYR67110.1	hypothetical protein AZ78_17770 [Lysobacter capsici AZ78]
OG_00821	LC55x_5185	tatB; twin arginine-targeting protein translocase TatB	OG_00821	EYR68857.1	translocase [Lysobacter capsici AZ78]
OG_00822	LC55x_3939	rpsH; 30S ribosomal subunit protein S8	OG_00822	EYR66690.1	30S ribosomal protein S8 [Lysobacter capsici AZ78]

OG_00823	LC55x_4438	LC55x_4438; transcriptional regulator marR/emrR family	OG_00823	EYR65479.1	MarR family transcriptional regulator [Lysobacter capsici AZ78]
OG_00824	LC55x_3951	rplW; 50S ribosomal subunit protein L23	OG_00824	EYR66702.1	50S ribosomal protein L23 [Lysobacter capsici AZ78]
OG_00825	LC55x_917	kdtA; 3-deoxy-D-manno-octulosonate(Kdo)-lipid A transferase	OG_00825	EYR66843.1	3-deoxy-D-manno-octulosonic acid transferase [Lysobacter capsici A
OG_00826	LC55x_1077	metE; 5-methyltetrahydropteroyltriglutamate-- homocysteine S-methy	OG_00826	EYR67714.1	5-methyltetrahydropteroyltriglutamate--homocysteine methyltransfera
OG_00827	LC55x_4441	kynA; tryptophan 2,3-dioxygenase	OG_00827	EYR68456.1	tryptophan 2,3-dioxygenase [Lysobacter capsici AZ78]
OG_00828	LC55x_2116	LC55x_2116; hypothetical protein	OG_00828	EYR66390.1	hypothetical protein AZ78_21250 [Lysobacter capsici AZ78]
OG_00829	LC55x_4767	LC55x_4767; bacterial regulatory helix-turn-helix, AraC family protein	OG_00829	EYR66080.1	AraC family transcriptional regulator [Lysobacter capsici AZ78]
OG_00830	LC55x_343	LC55x_343; pspA/IM30 family protein	OG_00830	EYR68121.1	hypothetical protein AZ78_11885 [Lysobacter capsici AZ78]
OG_00831	LC55x_2911	mtnA; S-methyl-5-thioribose-1-phosphate isomerase	OG_00831	EYR69467.1	methylthioribose-1-phosphate isomerase [Lysobacter capsici AZ78]
OG_00832	LC55x_3011	nuoF; NADH oxidoreductase (quinone), F subunit	OG_00832	EYR67809.1	NADH dehydrogenase [Lysobacter capsici AZ78]
OG_00833	LC55x_2172	sdhA; succinate dehydrogenase, flavoprotein subunit	OG_00833	EYR67390.1	succinate dehydrogenase [Lysobacter capsici AZ78]
OG_00834	LC55x_2540	LC55x_2540; hypothetical protein	OG_00834	EYR69803.1	hypothetical protein AZ78_03035 [Lysobacter capsici AZ78]
OG_00835	LC55x_5743	rnpA; ribonuclease P protein component	OG_00835	EYR69704.1	ribonuclease P [Lysobacter capsici AZ78]
OG_00836	LC55x_4212	LC55x_4212; regulatory , FmdB family domain protein	OG_00836	EYR69076.1	regulatory protein, FmdB family [Lysobacter capsici AZ78]
OG_00837	LC55x_2918	smc; chromosome segregation protein SMC	OG_00837	EYR69473.1	chromosome partitioning protein Smc [Lysobacter capsici AZ78]
OG_00838	LC55x_5035	LC55x_5035; proton antiporter-2 family protein	OG_00838	EYR66304.1	potassium transporter [Lysobacter capsici AZ78]
OG_00839	LC55x_476	LC55x_476; MOSC domain protein	OG_00839	EYR66464.1	molybdenum cofactor sulfurase [Lysobacter capsici AZ78]
OG_00840	LC55x_554	LC55x_554; bacterial regulatory helix-turn-helix, AraC family protein	OG_00840	EYR67914.1	hypothetical protein AZ78_12975 [Lysobacter capsici AZ78]
OG_00841	LC55x_5032	LC55x_5032; hypothetical protein	OG_00841	EYR66308.1	hypothetical protein AZ78_22320 [Lysobacter capsici AZ78]
OG_00842	LC55x_359	LC55x_359; AMP-binding enzyme family protein	OG_00842	EYR68133.1	peptide synthase [Lysobacter capsici AZ78]
OG_00843	LC55x_586	LC55x_586; glutaredoxin family protein	OG_00843	EYR70015.1	glutaredoxin [Lysobacter capsici AZ78]
OG_00844	LC55x_5193	LC55x_5193; conserved hypothetical protein	OG_00844	EYR68865.1	hypothetical protein AZ78_07315 [Lysobacter capsici AZ78]
OG_00845	LC55x_458	LC55x_458; MAPEG family protein	OG_00845	EYR66477.1	membrane protein [Lysobacter capsici AZ78]
OG_00846	LC55x_3851	LC55x_3851; histidine phosphatase super family protein	OG_00846	EYR65557.1	phosphoglycerate mutase [Lysobacter capsici AZ78]
OG_00847	LC55x_4223	LC55x_4223; histidine phosphatase super family protein	OG_00847	EYR69084.1	fructose-2,6-bisphosphatase [Lysobacter capsici AZ78]
OG_00848	LC55x_4989	LC55x_4989; bacterial regulatory , arsR family protein	OG_00848	EYR67239.1	ArsR family transcriptional regulator [Lysobacter capsici AZ78]
OG_00849	LC55x_4445	LC55x_4445; transketolase, pyrimidine binding domain protein	OG_00849	EYR68452.1	2-oxoisovalerate dehydrogenase subunit beta [Lysobacter capsici AZ
OG_00850	LC55x_3163	LC55x_3163; peptidase M13 family protein	OG_00850	EYR66936.1	peptidase M13 [Lysobacter capsici AZ78]
OG_00851	LC55x_5038	cydB; cytochrome d ubiquinol oxidase, subunit II	OG_00851	EYR66301.1	cytochrome D ubiquinol oxidase subunit II [Lysobacter capsici AZ78]
OG_00852	LC55x_495	LC55x_495; dienelactone hydrolase family protein	OG_00852	EYR66448.1	peptidase S9 [Lysobacter capsici AZ78]
OG_00853	LC55x_2252	LC55x_2252; FHA domain protein	OG_00853	EYR67440.1	signal peptide protein [Lysobacter capsici AZ78]
OG_00854	LC55x_1086	LC55x_1086; conserved hypothetical protein	OG_00854	EYR65358.1	hypothetical protein AZ78_26825 [Lysobacter capsici AZ78]
OG_00855	LC55x_2977	LC55x_2977; tat (twin-arginine translocation) pathway signal sequenc	OG_00855	EYR66186.1	hypothetical protein AZ78_21695 [Lysobacter capsici AZ78]
OG_00856	LC55x_3800	LC55x_3800; putative transmembrane protein	OG_00856	EYR67106.1	hypothetical protein AZ78_17750 [Lysobacter capsici AZ78]
OG_00857	LC55x_2750	LC55x_2750; glycine-zipper containing OmpA-like membrane domai	OG_00857	EYR69319.1	cell envelope biogenesis protein OmpA [Lysobacter capsici AZ78]
OG_00858	LC55x_4754	LC55x_4754; putative secreted protein	OG_00858	EYR66092.1	hypothetical protein AZ78_22810 [Lysobacter capsici AZ78]
OG_00859	LC55x_3105	LC55x_3105; glutamine amidotransferase class-I family protein	OG_00859	EYR65454.1	hypothetical protein AZ78_26270 [Lysobacter capsici AZ78]
OG_00860	LC55x_926	LC55x_926; FAD binding domain protein	OG_00860	EYR66834.1	glycerol-3-phosphate dehydrogenase [Lysobacter capsici AZ78]
OG_00861	LC55x_974	LC55x_974; short chain dehydrogenase	OG_00861	EYR66791.1	short-chain dehydrogenase [Lysobacter capsici AZ78]
OG_00862	LC55x_5059	LC55x_5059; dipeptidyl peptidase IV	OG_00862	EYR65586.1	peptidase S9 [Lysobacter capsici AZ78]
OG_00863	LC55x_155	LC55x_155; HAD hydrolase, IA, variant 1 family protein	OG_00863	EYR69798.1	HAD family hydrolase [Lysobacter capsici AZ78]
OG_00864	LC55x_2251	LC55x_2251; polyhydroxyalkanoic acid system family protein	OG_00864	EYR67439.1	polyhydroxyalkanoic acid synthase [Lysobacter capsici AZ78]
OG_00865	LC55x_5197	LC55x_5197; hypothetical protein	OG_00865	EYR68994.1	hypothetical protein AZ78_07335 [Lysobacter capsici AZ78]
OG_00866	LC55x_2649	LC55x_2649; oxidoreductase , NAD-binding Rossmann fold family pr	OG_00866	EYR68249.1	dehydrogenase [Lysobacter capsici AZ78]
OG_00867	LC55x_2432	LC55x_2432; conserved hypothetical protein	OG_00867	EYR69568.1	hypothetical protein AZ78_03560 [Lysobacter capsici AZ78]
OG_00868	LC55x_493	LC55x_493; DNA/RNA non-specific endonuclease family protein	OG_00868	EYR66483.1	hypothetical protein AZ78_20900 [Lysobacter capsici AZ78]
OG_00869	LC55x_2336	LC55x_2336; conserved hypothetical protein	OG_00869	EYR69632.1	membrane protein [Lysobacter capsici AZ78]
OG_00870	LC55x_5253	LC55x_5253; endoribonuclease L-PSP family protein	OG_00870	EYR68904.1	endoribonuclease L-PSP [Lysobacter capsici AZ78]
OG_00871	LC55x_4069	LC55x_4069; bacterial regulatory helix-turn-helix , lysR family protein	OG_00871	EYR65379.1	LysR family transcriptional regulator [Lysobacter capsici AZ78]
OG_00872	LC55x_998	LC55x_998; diguanylate cyclase domain protein	OG_00872	EYR67776.1	sensor protein [Lysobacter capsici AZ78]
OG_00873	LC55x_158	LC55x_158; putative esterase HI_1161	OG_00873	EYR69801.1	esterase [Lysobacter capsici AZ78]
OG_00874	LC55x_372	LC55x_372; bacterial regulatory, gntR family protein	OG_00874	EYR68142.1	GntR family transcriptional regulator [Lysobacter capsici AZ78]
OG_00875	LC55x_2791	LC55x_2791; modulator of DNA gyrase family protein	OG_00875	EYR69355.1	TldD protein [Lysobacter capsici AZ78]
OG_00876	LC55x_4034	LC55x_4034; bacterial regulatory, gntR family protein	OG_00876	EYR65813.1	hypothetical protein AZ78_24185 [Lysobacter capsici AZ78]
OG_00877	LC55x_1862	frr; ribosome recycling factor	OG_00877	EYR66054.1	ribosome recycling factor [Lysobacter capsici AZ78]
OG_00878	LC55x_1276	LC55x_1276; WGR domain protein	OG_00878	EYR67673.1	hypothetical protein AZ78_15040 [Lysobacter capsici AZ78]
OG_00879	LC55x_3571	LC55x_3571; conserved hypothetical protein	OG_00879	EYR67520.1	hypothetical protein AZ78_15350 [Lysobacter capsici AZ78]
OG_00880	LC55x_1690	LC55x_1690; histidine kinase-, DNA gyrase B-, and HSP90-like ATPa	OG_00880	EYR70354.1	histidine kinase [Lysobacter capsici AZ78]
OG_00881	LC55x_592	rsgA; ribosome small subunit-dependent GTPase A	OG_00881	EYR70010.1	GTPase RsgA [Lysobacter capsici AZ78]
OG_00882	LC55x_316	LC55x_316; hemX family protein	OG_00882	EYR68099.1	uroporphyrin-III methyltransferase [Lysobacter capsici AZ78]
OG_00883	LC55x_2167	LC55x_2167; transketolase, pyrimidine binding domain protein	OG_00883	EYR67386.1	MFS transporter [Lysobacter capsici AZ78]
OG_00884	LC55x_1716	LC55x_1716; conserved hypothetical protein	OG_00884	EYR70458.1	hypothetical protein AZ78_01505 [Lysobacter capsici AZ78]
OG_00885	LC55x_4474	dusA; tRNA dihydrouridine synthase A family protein	OG_00885	EYR68428.1	tRNA-dihydrouridine synthase A [Lysobacter capsici AZ78]
OG_00886	LC55x_5213	LC55x_5213; acetyltransferase family protein	OG_00886	EYR68877.1	GCN5 family acetyltransferase [Lysobacter capsici AZ78]
OG_00887	LC55x_564	LC55x_564; deoR-like helix-turn-helix domain protein	OG_00887	EYR67921.1	hypothetical protein AZ78_13010 [Lysobacter capsici AZ78]
OG_00888	LC55x_381	LC55x_381; TPR repeat family protein	OG_00888	EYR65632.1	hypothetical protein AZ78_25340 [Lysobacter capsici AZ78]
OG_00889	LC55x_1909	LC55x_1909; hypothetical protein	OG_00889	EYR65986.1	hypothetical protein AZ78_23450 [Lysobacter capsici AZ78]
OG_00890	LC55x_368	LC55x_368; hypothetical protein	OG_00890	EYR68182.1	hypothetical protein AZ78_12000 [Lysobacter capsici AZ78]
OG_00891	LC55x_747	LC55x_747; hypothetical protein	OG_00891	EYR67697.1	hypothetical protein AZ78_14800 [Lysobacter capsici AZ78]
OG_00892	LC55x_1119	LC55x_1119; GDSL-like Lipase/Acylhydrolase family protein	OG_00892	EYR68481.1	lipase/esterase [Lysobacter capsici AZ78]
OG_00893	LC55x_4518	HDDC3; HD domain-containing protein 3	OG_00893	EYR68403.1	phosphohydrolase [Lysobacter capsici AZ78]
OG_00894	LC55x_3468	LC55x_3468; DNA-binding protein, YbaB/EbfC family	OG_00894	EYR66611.1	hypothetical protein AZ78_20015 [Lysobacter capsici AZ78]
OG_00895	LC55x_880	LC55x_880; ahpC/TSA family protein	OG_00895	EYR66877.1	hypothetical protein AZ78_19035 [Lysobacter capsici AZ78]
OG_00896	LC55x_4328	purL; phosphoribosylformylglycinamidine synthase	OG_00896	EYR69173.1	phosphoribosylformylglycinamidine synthase [Lysobacter capsici AZ7
OG_00897	LC55x_1328	LC55x_1328; putative isomerase	OG_00897	EYR69908.1	D-arabinose 5-phosphate isomerase [Lysobacter capsici AZ78]
OG_00898	LC55x_3029	trpB; tryptophan synthase, beta subunit	OG_00898	EYR65370.1	tryptophan synthase subunit beta [Lysobacter capsici AZ78]
OG_00899	LC55x_1427	LC55x_1427; mechanosensitive ion channel family protein	OG_00899	EYR67148.1	hypothetical protein AZ78_17145 [Lysobacter capsici AZ78]
OG_00900	LC55x_3944	rpsQ; 30S ribosomal protein S17	OG_00900	EYR66695.1	30S ribosomal protein S17 [Lysobacter capsici AZ78]
OG_00901	LC55x_3611	LC55x_3611; drug resistance transporter, Bcr/CflA subfamily protein	OG_00901	EYR67553.1	MFS transporter [Lysobacter capsici AZ78]
OG_00902	LC55x_4242	gloA; lactoylglutathione lyase	OG_00902	EYR69098.1	lactoylglutathione lyase [Lysobacter capsici AZ78]
OG_00903	LC55x_1828	LC55x_1828; tonB family C-terminal domain protein	OG_00903	EYR66021.1	hypothetical protein AZ78_22960 [Lysobacter capsici AZ78]

OG_00904	LC55x_1943	mviN; integral membrane protein MviN
OG_00905	LC55x_3919	pqqC; coenzyme PQQ biosynthesis protein C
OG_00906	LC55x_2996	truB; tRNA pseudouridine(55) synthase
OG_00907	LC55x_2306	LC55x_2306; alpha/beta hydrolase fold family protein
OG_00908	LC55x_2796	amnD; 2-aminomuconate deaminase
OG_00909	LC55x_1995	lnt; apolipoprotein N-acyltransferase
OG_00910	LC55x_3013	nuoD; NADH dehydrogenase (quinone), D subunit
OG_00911	LC55x_4609	LC55x_4609; cytosol aminopeptidase
OG_00912	LC55x_3958	rpsL; ribosomal protein S12
OG_00913	LC55x_890	LC55x_890; helix-turn-helix family protein
OG_00914	LC55x_3594	LC55x_3594; amidohydrolase family protein
OG_00915	LC55x_4153	LC55x_4153; TPR repeat family protein
OG_00916	LC55x_1313	ptsP; phosphoenolpyruvate-protein phosphotransferase
OG_00917	LC55x_4604	LC55x_4604; conserved hypothetical protein
OG_00918	LC55x_5693	icd; isocitrate dehydrogenase, NADP-dependent
OG_00919	LC55x_4621	LC55x_4621; acetyltransferase family protein
OG_00920	LC55x_877	accB; acetyl-CoA carboxylase, biotin carboxyl carrier protein
OG_00921	LC55x_3451	fabD; malonyl CoA-acyl carrier protein transacylase
OG_00922	LC55x_3456	maf; septum formation protein Maf
OG_00923	LC55x_1615	nadC; nicotinate-nucleotide diphosphorylase
OG_00924	LC55x_2582	LC55x_2582; major Facilitator Superfamily protein
OG_00925	LC55x_110	LC55x_110; conserved hypothetical protein
OG_00926	LC55x_2299	LC55x_2299; MMPL family protein
OG_00927	LC55x_2626	cyoD; cytochrome o ubiquinol oxidase subunit IV
OG_00928	LC55x_2642	LC55x_2642; peptidase M23 family protein
OG_00929	LC55x_4689	LC55x_4689; hypothetical protein
OG_00930	LC55x_4679	LC55x_4679; cytidine and deoxycytidylate deaminase zinc-binding re
OG_00931	LC55x_840	LC55x_840; putative uvs125
OG_00932	LC55x_1851	LC55x_1851; ribonuclease HII family protein
OG_00933	LC55x_2841	LC55x_2841; ABC-2 transporter family protein
OG_00935	LC55x_3893	prpB; methylisocitrate lyase
OG_00936	LC55x_1741	LC55x_1741; rhomboid family protein
OG_00937	LC55x_3890	GST; glutathione S-transferase
OG_00938	LC55x_4259	LC55x_4259; type IV leader peptidase family protein
OG_00939	LC55x_2109	LC55x_2109; zinc carboxypeptidase family protein
OG_00940	LC55x_4187	pal; peptidoglycan-associated lipoprotein
OG_00941	LC55x_957	LC55x_957; hemolysin-type calcium-binding repeat family protein
OG_00942	LC55x_4665	mrdA; penicillin-binding protein 2
OG_00943	LC55x_3052	LC55x_3052; helix-turn-helix family protein
OG_00944	LC55x_1954	LC55x_1954; hypothetical protein
OG_00945	LC55x_5569	LC55x_5569; cytochrome C oxidase, cbb3-type, subunit III family pro
OG_00946	LC55x_1441	LC55x_1441; dienelactone hydrolase family protein
OG_00947	LC55x_4447	LC55x_4447; 2-oxoacid dehydrogenases acyltransferase family prote
OG_00948	LC55x_4201	ruvC; crossover junction endodeoxyribonuclease RuvC
OG_00949	LC55x_3549	LC55x_3549; conserved hypothetical protein
OG_00950	LC55x_4675	LC55x_4675; cold-active aminopeptidase
OG_00951	LC55x_1713	LC55x_1713; conserved hypothetical protein
OG_00952	LC55x_4199	ruvA; holliday junction DNA helicase RuvA
OG_00953	LC55x_5065	LC55x_5065; helix-turn-helix family protein
OG_00954	LC55x_2881	pbpC; penicillin-binding protein 1C
OG_00955	LC55x_63	LC55x_63; conserved hypothetical protein
OG_00956	LC55x_3937	rplR; ribosomal protein L18
OG_00957	LC55x_4929	topA; DNA topoisomerase I
OG_00958	LC55x_788	LC55x_788; glycosyl hydrolases 18 family protein
OG_00959	LC55x_3265	hisE; phosphoribosyl-ATP diphosphatase
OG_00960	LC55x_357	LC55x_357; hpcH/HpaI aldolase/citrate lyase family protein
OG_00961	LC55x_863	LC55x_863; conserved hypothetical protein
OG_00962	LC55x_2037	LC55x_2037; putative secreted protein
OG_00963	LC55x_5656	LC55x_5656; conserved enzyme
OG_00964	LC55x_2025	kdpC; K+-transporting ATPase, C subunit
OG_00965	LC55x_2935	glk; glucokinase
OG_00966	LC55x_709	LC55x_709; conserved hypothetical protein
OG_00967	LC55x_3766	LC55x_3766; glutathione-dependent formaldehyde-activating enzyme
OG_00968	LC55x_182	LC55x_182; sensory box protein
OG_00969	LC55x_2165	LC55x_2165; acetyltransferase domain protein
OG_00970	LC55x_1423	LC55x_1423; lysE type translocator family protein
OG_00971	LC55x_3541	LC55x_3541; hypothetical protein
OG_00972	LC55x_274	LC55x_274; FKBP-type peptidyl-prolyl cis-trans isomerase family pro
OG_00973	LC55x_2526	infA; translation initiation factor IF-1
OG_00974	LC55x_2466	LC55x_2466; rnfH Ubiquitin family protein
OG_00975	LC55x_4400	LC55x_4400; 4Fe-4S binding domain protein
OG_00976	LC55x_1181	LC55x_1181; conserved hypothetical protein
OG_00977	LC55x_2978	LC55x_2978; bacterial regulatory helix-turn-helix , lysR family protein
OG_00978	LC55x_2337	yfgL; outer membrane assembly lipoprotein YfgL
OG_00979	LC55x_2513	acnB; aconitate hydratase 2
OG_00980	LC55x_3127	LC55x_3127; conserved hypothetical protein
OG_00981	LC55x_5700	LC55x_5700; bacterial regulatory helix-turn-helix , lysR family protein
OG_00982	LC55x_583	LC55x_583; conserved hypothetical protein
OG_00983	LC55x_5191	LC55x_5191; exonuclease, RdgC family protein
OG_00984	LC55x_851	LC55x_851; conserved hypothetical protein
OG_00985	LC55x_3197	LC55x_3197; carboxyl transferase domain protein

OG_00904	EYR65964.1	membrane protein [Lysobacter capsici AZ78]
OG_00905	EYR66671.1	pyrroloquinoline quinone biosynthesis protein PqqC [Lysobacter caps
OG_00906	EYR67796.1	tRNA pseudouridine synthase B [Lysobacter capsici AZ78]
OG_00907	EYR69658.1	hydrolase [Lysobacter capsici AZ78]
OG_00908	EYR69360.1	2-aminomuconate deaminase [Lysobacter capsici AZ78]
OG_00909	EYR66760.1	apolipoprotein N-acyltransferase [Lysobacter capsici AZ78]
OG_00910	EYR67811.1	NADH dehydrogenase subunit D [Lysobacter capsici AZ78]
OG_00911	EYR68348.1	cytosol aminopeptidase [Lysobacter capsici AZ78]
OG_00912	EYR65489.1	30S ribosomal protein S12 [Lysobacter capsici AZ78]
OG_00913	EYR66867.1	hypothetical protein AZ78_18985 [Lysobacter capsici AZ78]
OG_00914	EYR67538.1	amidohydrolase [Lysobacter capsici AZ78]
OG_00915	EYR66328.1	hypothetical protein AZ78_21495 [Lysobacter capsici AZ78]
OG_00916	EYR69921.1	phosphoenolpyruvate-protein phosphotransferase [Lysobacter capsic
OG_00917	EYR68353.1	membrane protein [Lysobacter capsici AZ78]
OG_00918	EYR65865.1	isocitrate dehydrogenase [Lysobacter capsici AZ78]
OG_00919	EYR68340.1	N-acetyltransferase GCN5 [Lysobacter capsici AZ78]
OG_00920	EYR66880.1	acetyl-CoA carboxylase biotin carboxyl carrier protein [Lysobacter ca
OG_00921	EYR66597.1	malonyl CoA-ACP transacylase [Lysobacter capsici AZ78]
OG_00922	EYR66601.1	septum formation protein Maf [Lysobacter capsici AZ78]
OG_00923	EYR70320.1	nicotinate-nucleotide pyrophosphorylase [Lysobacter capsici AZ78]
OG_00924	EYR68312.1	Fosmidomycin resistance protein [Lysobacter capsici AZ78]
OG_00925	EYR69770.1	hypothetical protein AZ78_04945 [Lysobacter capsici AZ78]
OG_00926	EYR69660.1	acriflavin resistance protein [Lysobacter capsici AZ78]
OG_00927	EYR68270.1	hypothetical protein AZ78_11430 [Lysobacter capsici AZ78]
OG_00928	EYR68255.1	hypothetical protein AZ78_11355 [Lysobacter capsici AZ78]
OG_00929	EYR70187.1	hypothetical protein AZ78_00290 [Lysobacter capsici AZ78]
OG_00930	EYR70177.1	cytosine deaminase [Lysobacter capsici AZ78]
OG_00931	EYR67573.1	membrane protein [Lysobacter capsici AZ78]
OG_00932	EYR66043.1	ribonuclease HII [Lysobacter capsici AZ78]
OG_00933	EYR69402.1	hypothetical protein AZ78_05775 [Lysobacter capsici AZ78]
OG_00935	EYR65725.1	2-methylisocitrate lyase [Lysobacter capsici AZ78]
OG_00936	EYR70389.1	membrane protein [Lysobacter capsici AZ78]
OG_00937	EYR65722.1	glutathione S-transferase [Lysobacter capsici AZ78]
OG_00938	EYR69117.1	N-methyltransferase [Lysobacter capsici AZ78]
OG_00939	EYR66397.1	peptidase M14 carboxypeptidase A [Lysobacter capsici AZ78]
OG_00940	EYR68160.1	membrane protein [Lysobacter capsici AZ78]
OG_00941	EYR66807.1	hypothetical protein AZ78_18680 [Lysobacter capsici AZ78]
OG_00942	EYR70166.1	penicillin-binding protein 2 [Lysobacter capsici AZ78]
OG_00943	EYR67838.1	XRE family transcriptional regulator [Lysobacter capsici AZ78]
OG_00944	EYR66007.1	hypothetical protein AZ78_23260 [Lysobacter capsici AZ78]
OG_00945	EYR68666.1	cytochrome C [Lysobacter capsici AZ78]
OG_00946	EYR67135.1	DeoR family transcriptional regulator [Lysobacter capsici AZ78]
OG_00947	EYR68450.1	branched-chain alpha-keto acid dehydrogenase subunit E2 [Lysobac
OG_00948	EYR68149.1	Holliday junction resolvase [Lysobacter capsici AZ78]
OG_00949	EYR67502.1	hypothetical protein AZ78_15260 [Lysobacter capsici AZ78]
OG_00950	EYR70173.1	aminopeptidase [Lysobacter capsici AZ78]
OG_00951	EYR70456.1	hypothetical protein AZ78_01490 [Lysobacter capsici AZ78]
OG_00952	EYR68150.1	Holliday junction DNA helicase RuvA [Lysobacter capsici AZ78]
OG_00953	EYR68071.1	XRE family transcriptional regulator [Lysobacter capsici AZ78]
OG_00954	EYR69440.1	penicillin-binding protein 1C [Lysobacter capsici AZ78]
OG_00955	EYR69740.1	alkylhydroperoxidase [Lysobacter capsici AZ78]
OG_00956	EYR66688.1	50S ribosomal protein L18 [Lysobacter capsici AZ78]
OG_00957	EYR68688.1	DNA topoisomerase I [Lysobacter capsici AZ78]
OG_00958	EYR67603.1	chitinase [Lysobacter capsici AZ78]
OG_00959	EYR70036.1	phosphoribosyl-AMP cyclohydrolase [Lysobacter capsici AZ78]
OG_00960	EYR68131.1	citryl-CoA lyase [Lysobacter capsici AZ78]
OG_00961	EYR66894.1	hypothetical protein AZ78_19125 [Lysobacter capsici AZ78]
OG_00962	EYR65593.1	hypothetical protein AZ78_25395 [Lysobacter capsici AZ78]
OG_00963	EYR66291.1	alpha/beta hydrolase [Lysobacter capsici AZ78]
OG_00964	EYR65371.1	ATPase [Lysobacter capsici AZ78]
OG_00965	EYR66225.1	glucokinase [Lysobacter capsici AZ78]
OG_00966	EYR69940.1	hypothetical protein AZ78_01960 [Lysobacter capsici AZ78]
OG_00967	EYR67075.1	aldehyde-activating protein [Lysobacter capsici AZ78]
OG_00968	EYR66123.1	hypothetical protein AZ78_22405 [Lysobacter capsici AZ78]
OG_00969	EYR67384.1	acetyltransferase [Lysobacter capsici AZ78]
OG_00970	EYR67150.1	hypothetical protein AZ78_17155 [Lysobacter capsici AZ78]
OG_00971	EYR67493.1	hypothetical protein AZ78_15215 [Lysobacter capsici AZ78]
OG_00972	EYR67000.1	hypothetical protein AZ78_17965 [Lysobacter capsici AZ78]
OG_00973	EYR69498.1	translation initiation factor IF-1 [Lysobacter capsici AZ78]
OG_00974	EYR69544.1	hypothetical protein AZ78_03400 [Lysobacter capsici AZ78]
OG_00975	EYR69282.1	hypothetical protein AZ78_07145 [Lysobacter capsici AZ78]
OG_00976	EYR65801.1	hypothetical protein AZ78_24590 [Lysobacter capsici AZ78]
OG_00977	EYR65353.1	LysR family transcriptional regulator [Lysobacter capsici AZ78]
OG_00978	EYR69631.1	PQQ containing lipoprotein [Lysobacter capsici AZ78]
OG_00979	EYR69505.1	bifunctional aconitate hydratase 2/2-methylisocitrate dehydratase [Ly
OG_00980	EYR66967.1	hypothetical protein AZ78_18525 [Lysobacter capsici AZ78]
OG_00981	EYR65858.1	biotin transporter BioY [Lysobacter capsici AZ78]
OG_00982	EYR70133.1	hypothetical protein AZ78_02540 [Lysobacter capsici AZ78]
OG_00983	EYR68863.1	recombinase RdgC [Lysobacter capsici AZ78]
OG_00984	EYR67565.1	hypothetical protein AZ78_14320 [Lysobacter capsici AZ78]
OG_00985	EYR67992.1	methylcrotonoyl-CoA carboxylase [Lysobacter capsici AZ78]

OG_00986	LC55x_4678	LC55x_4678; acetyltransferase domain protein	OG_00986	EYR70176.1	GCN5 family N-acetyltransferase [Lysobacter capsici AZ78]
OG_00987	LC55x_4764	LC55x_4764; 2Fe-2S iron-sulfur cluster binding domain protein	OG_00987	EYR66083.1	(2Fe-2S)-binding protein [Lysobacter capsici AZ78]
OG_00988	LC55x_3742	phaZ; polyhydroxyalkanoate depolymerase, intracellular family protei	OG_00988	EYR67055.1	esterase [Lysobacter capsici AZ78]
OG_00989	LC55x_3036	gyaR; glyoxylate reductase	OG_00989	EYR67826.1	2-hydroxyacid dehydrogenase [Lysobacter capsici AZ78]
OG_00990	LC55x_5640	LC55x_5640; 4'-phosphopantetheinyl transferase superfamily protein	OG_00990	EYR66278.1	hypothetical protein AZ78_22170 [Lysobacter capsici AZ78]
OG_00991	LC55x_4321	LC55x_4321; prepilin-type N-terminal cleavage/methylation domain p	OG_00991	EYR69168.1	general secretion pathway protein I [Lysobacter capsici AZ78]
OG_00992	LC55x_4933	LC55x_4933; protein involved in synthesis of threonylcarbamoyladen	OG_00992	EYR68685.1	tRNA threonylcarbamoyladenosine biosynthesis protein RimN [Lysob
OG_00993	LC55x_5669	LC55x_5669; bacterial regulatory helix-turn-helix , lysR family protein	OG_00993	EYR65885.1	transcriptional regulator [Lysobacter capsici AZ78]
OG_00994	LC55x_1622	grxD; monothiol glutaredoxin, Grx4 family	OG_00994	EYR70325.1	glutaredoxin [Lysobacter capsici AZ78]
OG_00995	LC55x_832	bfr; bacterioferritin	OG_00995	EYR67580.1	bacterioferritin [Lysobacter capsici AZ78]
OG_00996	LC55x_3935	rpmD; ribosomal protein L30	OG_00996	EYR66686.1	50S ribosomal protein L30 [Lysobacter capsici AZ78]
OG_00997	LC55x_4986	ymaE; metallo-beta-lactamase family protein	OG_00997	EYR67242.1	hypothetical protein AZ78_16860 [Lysobacter capsici AZ78]
OG_00998	LC55x_5055	uvrD; DNA helicase II	OG_00998	EYR65582.1	DNA helicase II [Lysobacter capsici AZ78]
OG_00999	LC55x_1717	LC55x_1717; aminotransferase class-III family protein	OG_00999	EYR70370.1	lysine 6-aminotransferase [Lysobacter capsici AZ78]
OG_01000	LC55x_2464	smpB; ssrA-binding protein	OG_01000	EYR69546.1	SsrA-binding protein [Lysobacter capsici AZ78]
OG_01001	LC55x_2964	LC55x_2964; conserved hypothetical protein	OG_01001	EYR66199.1	hypothetical protein AZ78_21760 [Lysobacter capsici AZ78]
OG_01002	LC55x_4437	hppD; 4-hydroxyphenylpyruvate dioxygenase	OG_01002	EYR65478.1	4-hydroxyphenylpyruvate dioxygenase [Lysobacter capsici AZ78]
OG_01003	LC55x_3669	hflX; GTP-binding protein HflX	OG_01003	EYR65947.1	GTPase HflX [Lysobacter capsici AZ78]
OG_01004	LC55x_1829	LC55x_1829; hypothetical protein	OG_01004	EYR66022.1	hypothetical protein AZ78_22965 [Lysobacter capsici AZ78]
OG_01005	LC55x_3924	pam; peptide amidase	OG_01005	EYR66675.1	amidase [Lysobacter capsici AZ78]
OG_01006	LC55x_1683	dxs; 1-deoxy-D-xylulose-5-phosphate synthase	OG_01006	EYR70349.1	1-deoxy-D-xylulose-5-phosphate synthase [Lysobacter capsici AZ78]
OG_01007	LC55x_4169	LC55x_4169; hypothetical protein	OG_01007	EYR68202.1	hypothetical protein AZ78_12265 [Lysobacter capsici AZ78]
OG_01008	LC55x_2667	LC55x_2667; cheB methylesterase family protein	OG_01008	EYR68231.1	chemotaxis protein CheY [Lysobacter capsici AZ78]
OG_01009	LC55x_1921	LC55x_1921; phosphate-selective porin O and P family protein	OG_01009	EYR65978.1	porin [Lysobacter capsici AZ78]
OG_01010	LC55x_4094	pdxA; 4-hydroxythreonine-4-phosphate dehydrogenase	OG_01010	EYR65495.1	4-hydroxythreonine-4-phosphate dehydrogenase [Lysobacter capsici
OG_01011	LC55x_5074	LC55x_5074; beta-ketoacyl synthase, N-terminal domain protein	OG_01011	EYR68065.1	hypothetical protein AZ78_12770 [Lysobacter capsici AZ78]
OG_01012	LC55x_268	LC55x_268; bacterial regulatory, tetR family protein	OG_01012	EYR66995.1	TetR family transcriptional regulator [Lysobacter capsici AZ78]
OG_01013	LC55x_2797	nbaC; 3-hydroxyanthranilate 3,4-dioxygenase	OG_01013	EYR69361.1	3-hydroxyanthranilate 3,4-dioxygenase [Lysobacter capsici AZ78]
OG_01014	LC55x_1695	LC55x_1695; subtilase family protein	OG_01014	EYR70358.1	peptidase S8 [Lysobacter capsici AZ78]
OG_01015	LC55x_5227	LC55x_5227; peptidase inhibitor I78 family protein	OG_01015	EYR68888.1	starvation-inducible outer membrane lipoprotein [Lysobacter capsici /
OG_01016	LC55x_2955	LC55x_2955; FAD binding domain protein	OG_01016	EYR66207.1	FAD binding protein [Lysobacter capsici AZ78]
OG_01017	LC55x_3213	LC55x_3213; hypothetical protein	OG_01017	EYR67974.1	hypothetical protein AZ78_13280 [Lysobacter capsici AZ78]
OG_01018	LC55x_3529	LC55x_3529; conserved hypothetical protein	OG_01018	EYR67481.1	hypothetical protein AZ78_15150 [Lysobacter capsici AZ78]
OG_01019	LC55x_1681	LC55x_1681; conserved hypothetical protein	OG_01019	EYR70348.1	iron transporter [Lysobacter capsici AZ78]
OG_01020	LC55x_4619	LC55x_4619; eamA-like transporter family protein	OG_01020	EYR68342.1	membrane protein [Lysobacter capsici AZ78]
OG_01021	LC55x_3726	LC55x_3726; nlpC/P60 family protein	OG_01021	EYR65899.1	hypothetical protein AZ78_23610 [Lysobacter capsici AZ78]
OG_01022	LC55x_133	LC55x_133; prokaryotic cytochrome b561 family protein	OG_01022	EYR69784.1	cytochrome B561 [Lysobacter capsici AZ78]
OG_01023	LC55x_2563	LC55x_2563; short chain dehydrogenase family protein	OG_01023	EYR68330.1	short-chain dehydrogenase [Lysobacter capsici AZ78]
OG_01024	LC55x_3832	LC55x_3832; hypothetical protein	OG_01024	EYR65747.1	SSU ribosomal protein S31P [Lysobacter capsici AZ78]
OG_01025	LC55x_4332	LC55x_4332; RDD family protein	OG_01025	EYR69176.1	membrane protein [Lysobacter capsici AZ78]
OG_01026	LC55x_5104	LC55x_5104; hypothetical protein	OG_01026	EYR68090.1	hypothetical protein AZ78_12655 [Lysobacter capsici AZ78]
OG_01027	LC55x_5646	LC55x_5646; conserved hypothetical protein	OG_01027	EYR66283.1	hypothetical protein AZ78_22195 [Lysobacter capsici AZ78]
OG_01028	LC55x_3015	LC55x_3015; NADH-ubiquinone oxidoreductase 20 kDa subunit, mitc	OG_01028	EYR67813.1	NADH dehydrogenase subunit B [Lysobacter capsici AZ78]
OG_01029	LC55x_2397	LC55x_2397; NRDE family protein	OG_01029	EYR69593.1	hypothetical protein AZ78_03750 [Lysobacter capsici AZ78]
OG_01030	LC55x_1614	LC55x_1614; conserved hypothetical protein	OG_01030	EYR70319.1	membrane protein [Lysobacter capsici AZ78]
OG_01031	LC55x_2825	LC55x_2825; conserved hypothetical protein	OG_01031	EYR69386.1	hypothetical protein AZ78_05690 [Lysobacter capsici AZ78]
OG_01032	LC55x_948	LC55x_948; ion channel family protein	OG_01032	EYR66814.1	transmembrane ion channel [Lysobacter capsici AZ78]
OG_01033	LC55x_2013	lexA; repressor LexA	OG_01033	EYR66744.1	LexA family transcriptional regulator [Lysobacter capsici AZ78]
OG_01034	LC55x_3591	ACE; angiotensin-converting enzyme	OG_01034	EYR67536.1	peptidase M20 [Lysobacter capsici AZ78]
OG_01035	LC55x_572	LC55x_572; ADP-ribosylglycohydrolase	OG_01035	EYR70026.1	ADP-ribosylglycohydrolase [Lysobacter capsici AZ78]
OG_01036	LC55x_4616	LC55x_4616; ftsX-like permease family protein	OG_01036	EYR68344.1	membrane protein [Lysobacter capsici AZ78]
OG_01037	LC55x_814	LC55x_814; putative pdmP3	OG_01037	EYR67688.1	hypothetical protein AZ78_14485 [Lysobacter capsici AZ78]
OG_01038	LC55x_3401	LC55x_3401; RNA polymerase sigma factor, sigma-70 family protein	OG_01038	EYR66518.1	RNA polymerase sigma factor [Lysobacter capsici AZ78]
OG_01039	LC55x_1188	LC55x_1188; nfeD-like C-terminal, partner-binding family protein	OG_01039	EYR65795.1	membrane protein [Lysobacter capsici AZ78]
OG_01040	LC55x_2447	LC55x_2447; acyl-CoA dehydrogenase, N-terminal domain protein	OG_01040	EYR69556.1	acyl-CoA dehydrogenase [Lysobacter capsici AZ78]
OG_01041	LC55x_3310	LC55x_3310; conserved hypothetical protein	OG_01041	EYR70066.1	hypothetical protein AZ78_02880 [Lysobacter capsici AZ78]
OG_01042	LC55x_2557	LC55x_2557; potH	OG_01042	EYR65536.1	putrescine/spermidine ABC transporter permease [Lysobacter capsic
OG_01043	LC55x_4469	LC55x_4469; phosphoenolpyruvate:glucose-phosphotransferase regu	OG_01043	EYR68433.1	zinc-dependent peptidase (M family) [Lysobacter capsici AZ78]
OG_01044	LC55x_1305	LC55x_1305; conserved hypothetical protein	OG_01044	EYR69927.1	Holliday junction resolvase [Lysobacter capsici AZ78]
OG_01045	LC55x_3789	LC55x_3789; polyketide cyclase / dehydrase and lipid transport famil	OG_01045	EYR67096.1	hypothetical protein AZ78_17700 [Lysobacter capsici AZ78]
OG_01046	LC55x_1727	secF; protein-export membrane protein SecF	OG_01046	EYR70377.1	preprotein translocase subunit SecF [Lysobacter capsici AZ78]
OG_01047	LC55x_5088	LC55x_5088; MMPL family protein	OG_01047	EYR68060.1	membrane protein [Lysobacter capsici AZ78]
OG_01048	LC55x_5047	polA; DNA polymerase I, 3' -- 5' polymerase, 5' -- 3' and 3' -- 5' exonu	OG_01048	EYR65575.1	DNA polymerase I [Lysobacter capsici AZ78]
OG_01049	LC55x_3300	proA; glutamate-5-semialdehyde dehydrogenase	OG_01049	EYR70057.1	gamma-glutamyl phosphate reductase [Lysobacter capsici AZ78]
OG_01050	LC55x_3938	rplF; ribosomal protein L6	OG_01050	EYR66689.1	50S ribosomal protein L6 [Lysobacter capsici AZ78]
OG_01051	LC55x_4416	LC55x_4416; response regulator	OG_01051	EYR69243.1	XRE family transcriptional regulator [Lysobacter capsici AZ78]
OG_01052	LC55x_3996	wrbA; quinone oxidoreductase, type IV	OG_01052	EYR66726.1	NAD(P)H quinone oxidoreductase [Lysobacter capsici AZ78]
OG_01053	LC55x_3286	LC55x_3286; alpha-1,2-mannosidase family protein	OG_01053	EYR70051.1	alpha-1 2-mannosidase [Lysobacter capsici AZ78]
OG_01054	LC55x_3828	hutI; imidazolonepropionase	OG_01054	EYR65751.1	imidazolonepropionase [Lysobacter capsici AZ78]
OG_01055	LC55x_1118	thiS; thiamine biosynthesis protein ThiS	OG_01055	EYR68480.1	sulfur carrier protein ThiS [Lysobacter capsici AZ78]
OG_01056	LC55x_4162	LC55x_4162; putative secreted protein	OG_01056	EYR66319.1	hypothetical protein AZ78_21445 [Lysobacter capsici AZ78]
OG_01057	LC55x_1189	LC55x_1189; hypothetical protein	OG_01057	EYR65794.1	hypothetical protein AZ78_24555 [Lysobacter capsici AZ78]
OG_01058	LC55x_325	LC55x_325; putative peptidoglycan binding domain protein	OG_01058	EYR68174.1	hypothetical protein AZ78_11800 [Lysobacter capsici AZ78]
OG_01059	LC55x_5742	yidC; membrane protein insertase, YidC/Oxa1 family, N-terminal dom	OG_01059	EYR69703.1	insertase [Lysobacter capsici AZ78]
OG_01060	LC55x_1711	LC55x_1711; hypothetical protein	OG_01060	EYR70455.1	hypothetical protein AZ78_01480 [Lysobacter capsici AZ78]
OG_01061	LC55x_4913	LC55x_4913; bacterial lipid A biosynthesis acyltransferase family prot	OG_01061	EYR68703.1	lauroyl acyltransferase [Lysobacter capsici AZ78]
OG_01062	LC55x_168	LC55x_168; membrane transport family protein	OG_01062	EYR65409.1	malate permease [Lysobacter capsici AZ78]
OG_01063	LC55x_2864	LC55x_2864; metallo-beta-lactamase superfamily protein	OG_01063	EYR69422.1	hypothetical protein AZ78_05875 [Lysobacter capsici AZ78]
OG_01064	LC55x_2258	hisC; histidinol-phosphate transaminase	OG_01064	EYR67445.1	histidinol-phosphate aminotransferase [Lysobacter capsici AZ78]
OG_01065	LC55x_2088	LC55x_2088; glutamine amidotransferase class-I family protein	OG_01065	EYR66416.1	hypothetical protein AZ78_21385 [Lysobacter capsici AZ78]
OG_01066	LC55x_4275	murE; UDP-N-acetylmuramyl-tripeptide synthetase family protein	OG_01066	EYR69133.1	UDP-N-acetylmuramoylalanyl-D-glutamate--2,6-diaminopimelate liga

OG_01067	LC55x_4711	LC55x_4711; outer membrane efflux family protein	OG_01067	EYR65673.1	cation transporter [Lysobacter capsici AZ78]
OG_01068	LC55x_36	LC55x_36; conserved hypothetical protein	OG_01068	EYR69730.1	hypothetical protein AZ78_04650 [Lysobacter capsici AZ78]
OG_01069	LC55x_1707	coaD; pantetheine-phosphate adenylyltransferase	OG_01069	EYR70365.1	phosphopantetheine adenylyltransferase [Lysobacter capsici AZ78]
OG_01070	LC55x_3130	LC55x_3130; bacterial regulatory, tetR family protein	OG_01070	EYR66964.1	TetR family transcriptional regulator [Lysobacter capsici AZ78]
OG_01071	LC55x_463	LC55x_463; C-terminal processing peptidase family protein	OG_01071	EYR66473.1	peptidase S41 [Lysobacter capsici AZ78]
OG_01072	LC55x_836	LC55x_836; polymer-forming cytoskeletal family protein	OG_01072	EYR67577.1	cell shape determination protein CcmA [Lysobacter capsici AZ78]
OG_01073	LC55x_1407	LC55x_1407; 3-hydroxybutyrate dehydrogenase family protein	OG_01073	EYR67162.1	3-hydroxybutyrate dehydrogenase [Lysobacter capsici AZ78]
OG_01074	LC55x_2813	LC55x_2813; 5'-nucleotidase family protein	OG_01074	EYR69374.1	5'-nucleotidase [Lysobacter capsici AZ78]
OG_01075	LC55x_1035	LC55x_1035; penicillin-binding , 1A family protein	OG_01075	EYR67748.1	penicillin-binding protein 1A [Lysobacter capsici AZ78]
OG_01076	LC55x_4451	LC55x_4451; glyoxalase-like domain protein	OG_01076	EYR68447.1	glyoxalase [Lysobacter capsici AZ78]
OG_01077	LC55x_5170	LC55x_5170; RNA polymerase sigma factor, TIGR02999 family prote	OG_01077	EYR67999.1	RNA polymerase sigma factor [Lysobacter capsici AZ78]
OG_01078	LC55x_4170	LC55x_4170; phenazine biosynthesis , PhzF family protein	OG_01078	EYR68169.1	phenazine biosynthesis protein [Lysobacter capsici AZ78]
OG_01079	LC55x_4078	glnS; glutamine--tRNA ligase	OG_01079	EYR65430.1	glutaminyl-tRNA synthetase [Lysobacter capsici AZ78]
OG_01080	LC55x_4713	czcA; cobalt-zinc-cadmium resistance protein czcA	OG_01080	EYR65675.1	cation transporter [Lysobacter capsici AZ78]
OG_01081	LC55x_4505	LC55x_4505; hypothetical protein	OG_01081	EYR68585.1	hypothetical protein AZ78_10100 [Lysobacter capsici AZ78]
OG_01082	LC55x_798	LC55x_798; efflux transporter, RND family, MFP subunit	OG_01082	EYR67594.1	ABC transporter permease [Lysobacter capsici AZ78]
OG_01083	LC55x_4574	LC55x_4574; conserved hypothetical family protein	OG_01083	EYR68372.1	hypothetical protein AZ78_09745 [Lysobacter capsici AZ78]
OG_01084	LC55x_3455	LC55x_3455; hypothetical protein	OG_01084	EYR66644.1	hypothetical protein AZ78_19955 [Lysobacter capsici AZ78]
OG_01085	LC55x_3126	LC55x_3126; conserved hypothetical protein	OG_01085	EYR66968.1	hypothetical protein AZ78_18530 [Lysobacter capsici AZ78]
OG_01086	LC55x_3820	LC55x_3820; hypothetical protein	OG_01086	EYR67124.1	hypothetical protein AZ78_17840 [Lysobacter capsici AZ78]
OG_01087	LC55x_4712	LC55x_4712; efflux transporter, RND family, MFP subunit	OG_01087	EYR65674.1	cobalt-zinc-cadmium resistance protein [Lysobacter capsici AZ78]
OG_01088	LC55x_3556	LC55x_3556; arginine-tRNA-transferase, C terminus family protein	OG_01088	EYR67507.1	hypothetical protein AZ78_15285 [Lysobacter capsici AZ78]
OG_01089	LC55x_1754	LC55x_1754; metallo-beta-lactamase superfamily protein	OG_01089	EYR65697.1	beta-lactamase [Lysobacter capsici AZ78]
OG_01090	LC55x_3198	LC55x_3198; hsc62, Hsp70 family chaperone, binds to RpoD and inh	OG_01090	EYR67991.1	chaperone heat shock Hsp70 protein [Lysobacter capsici AZ78]
OG_01091	LC55x_59	LC55x_59; NIPSNAP family protein	OG_01091	EYR69737.1	hypothetical protein AZ78_04715 [Lysobacter capsici AZ78]
OG_01092	LC55x_2347	folD; bifunctional protein folD	OG_01092	EYR69623.1	methenyltetrahydrofolate cyclohydrolase [Lysobacter capsici AZ78]
OG_01093	LC55x_3837	zur; zur transcriptional repressor	OG_01093	EYR65742.1	Fur family transcriptional regulator [Lysobacter capsici AZ78]
OG_01094	LC55x_3420	LC55x_3420; phosphorylase superfamily protein	OG_01094	EYR66508.1	5'-methylthioadenosine phosphorylase [Lysobacter capsici AZ78]
OG_01095	LC55x_4342	rimI; ribosomal-protein-alanine acetyltransferase	OG_01095	EYR69183.1	alanine acetyltransferase [Lysobacter capsici AZ78]
OG_01096	LC55x_3073	LC55x_3073; hypothetical protein	OG_01096	EYR67882.1	hypothetical protein AZ78_13795 [Lysobacter capsici AZ78]
OG_01097	LC55x_32	LC55x_32; citrate transporter family protein	OG_01097	EYR69727.1	sulfur deprivation response regulator [Lysobacter capsici AZ78]
OG_01098	LC55x_3465	ycfF; purine nucleoside phosphoramidase	OG_01098	EYR66608.1	histidine triad (HIT) protein [Lysobacter capsici AZ78]
OG_01099	LC55x_525	cc4; cytochrome c4 domain protein	OG_01099	EYR67886.1	cytochrome C, partial [Lysobacter capsici AZ78]
OG_01100	LC55x_965	LC55x_965; conserved hypothetical protein	OG_01100	EYR66800.1	hypothetical protein AZ78_18645 [Lysobacter capsici AZ78]
OG_01101	LC55x_4045	LC55x_4045; hypothetical protein	OG_01101	EYR65824.1	hypothetical protein AZ78_24240 [Lysobacter capsici AZ78]
OG_01102	LC55x_5620	LC55x_5620; putative lipase	OG_01102	EYR66260.1	hypothetical protein AZ78_22075 [Lysobacter capsici AZ78]
OG_01103	LC55x_4231	LC55x_4231; conserved hypothetical protein	OG_01103	EYR69092.1	cobalamin transporter [Lysobacter capsici AZ78]
OG_01104	LC55x_1096	LC55x_1096; lysE type translocator family protein	OG_01104	EYR68463.1	threonine transporter [Lysobacter capsici AZ78]
OG_01105	LC55x_3067	LC55x_3067; glucose / Sorbosone dehydrogenase family protein	OG_01105	EYR67847.1	sorbosone dehydrogenase [Lysobacter capsici AZ78]
OG_01106	LC55x_1947	lspA; signal peptidase II	OG_01106	EYR65961.1	peptidase A8 [Lysobacter capsici AZ78]
OG_01107	LC55x_5075	LC55x_5075; glycosyl transferase 2 family protein	OG_01107	EYR68064.1	dolichyl-phosphate mannose synthase [Lysobacter capsici AZ78]
OG_01108	LC55x_3004	LC55x_3004; proton-translocating NADH-quinone oxidoreductase, ch	OG_01108	EYR67802.1	NADH:ubiquinone oxidoreductase subunit M [Lysobacter capsici AZ7
OG_01109	LC55x_2973	LC55x_2973; hypothetical protein	OG_01109	EYR66189.1	hypothetical protein AZ78_21710 [Lysobacter capsici AZ78]
OG_01110	LC55x_309	LC55x_309; putative ACETYL-COA ACYLTRANSFERASE FADA2	OG_01110	EYR67039.1	acetyl-CoA acetyltransferase [Lysobacter capsici AZ78]
OG_01111	LC55x_4955	LC55x_4955; conserved hypothetical protein	OG_01111	EYR67266.1	hypothetical protein AZ78_16980 [Lysobacter capsici AZ78]
OG_01112	LC55x_4350	LC55x_4350; conserved hypothetical family protein	OG_01112	EYR69190.1	ABC transporter permease [Lysobacter capsici AZ78]
OG_01113	LC55x_2775	ydiC; fe-S cluster assembly protein	OG_01113	EYR69340.1	iron-sulfur cluster assembly protein [Lysobacter capsici AZ78]
OG_01114	LC55x_4266	ftsZ; cell division protein FtsZ	OG_01114	EYR69124.1	peptidase M23 [Lysobacter capsici AZ78]
OG_01115	LC55x_967	LC55x_967; bacterial regulatory helix-turn-helix, AraC family protein	OG_01115	EYR66798.1	hypothetical protein AZ78_18635 [Lysobacter capsici AZ78]
OG_01116	LC55x_4102	thyA; thymidylate synthase	OG_01116	EYR65503.1	thymidylate synthase [Lysobacter capsici AZ78]
OG_01118	LC55x_4098	LC55x_4098; putative amidase domain protein	OG_01118	EYR65499.1	hypothetical protein AZ78_25935 [Lysobacter capsici AZ78]
OG_01119	LC55x_2835	LC55x_2835; FKBP-type peptidyl-prolyl cis-trans isomerase family pr	OG_01119	EYR69396.1	hypothetical protein AZ78_05745 [Lysobacter capsici AZ78]
OG_01120	LC55x_2860	LC55x_2860; fumarase C family protein	OG_01120	EYR69420.1	fumarate hydratase [Lysobacter capsici AZ78]
OG_01121	LC55x_1720	LC55x_1720; histidine kinase-, DNA gyrase B-, and HSP90-like ATPa	OG_01121	EYR70371.1	histidine kinase [Lysobacter capsici AZ78]
OG_01122	LC55x_4630	lipB; lipoyl(octanoyl) transferase	OG_01122	EYR68335.1	lipoate-protein ligase B [Lysobacter capsici AZ78]
OG_01123	LC55x_2411	ccmF; cytochrome c-type biogenesis protein CcmF	OG_01123	EYR69585.1	cytochrome C biogenesis protein [Lysobacter capsici AZ78]
OG_01124	LC55x_237	LC55x_237; FHA domain protein	OG_01124	EYR66174.1	hypothetical protein AZ78_22660 [Lysobacter capsici AZ78]
OG_01125	LC55x_3044	LC55x_3044; yhhN-like family protein	OG_01125	EYR67831.1	hypothetical protein AZ78_13650 [Lysobacter capsici AZ78]
OG_01126	LC55x_4453	LC55x_4453; metallo-beta-lactamase superfamily protein	OG_01126	EYR68445.1	beta-lactamase [Lysobacter capsici AZ78]
OG_01127	LC55x_2777	LC55x_2777; conserved hypothetical protein	OG_01127	EYR69341.1	hypothetical protein AZ78_05465 [Lysobacter capsici AZ78]
OG_01128	LC55x_3517	LC55x_3517; periplasmic glucan biosynthesis , MdoG family protein	OG_01128	EYR66641.1	glucan biosynthesis protein D [Lysobacter capsici AZ78]
OG_01129	LC55x_2941	LC55x_2941; glucoamylase family protein	OG_01129	EYR66219.1	hypothetical protein AZ78_21865 [Lysobacter capsici AZ78]
OG_01130	LC55x_2329	LC55x_2329; acetyl-CoA C-acetyltransferase family protein	OG_01130	EYR69639.1	acetyl-CoA acetyltransferase [Lysobacter capsici AZ78]
OG_01131	LC55x_1244	LC55x_1244; NHL repeat family protein	OG_01131	EYR65639.1	phytase [Lysobacter capsici AZ78]
OG_01132	LC55x_3585	minE; cell division topological specificity factor MinE	OG_01132	EYR67532.1	cell division topological specificity factor [Lysobacter capsici AZ78]
OG_01133	LC55x_2042	folK; 2-amino-4-hydroxy-6- hydroxymethyldihydropteridine diphospho	OG_01133	EYR65596.1	2-amino-4-hydroxy-6-hydroxymethyldihydropteridine pyrophosphokin
OG_01134	LC55x_261	LC55x_261; conserved hypothetical protein	OG_01134	EYR66989.1	3-demethylubiquinone-9 3-methyltransferase [Lysobacter capsici AZ7
OG_01135	LC55x_3427	LC55x_3427; hypothetical protein	OG_01135	EYR66557.1	hypothetical protein AZ78_20315 [Lysobacter capsici AZ78]
OG_01136	LC55x_3765	LC55x_3765; conserved hypothetical protein	OG_01136	EYR67074.1	hypothetical protein AZ78_17585 [Lysobacter capsici AZ78]
OG_01137	LC55x_1420	LC55x_1420; hypothetical protein	OG_01137	EYR67152.1	hypothetical protein AZ78_17165 [Lysobacter capsici AZ78]
OG_01138	LC55x_4303	LC55x_4303; serine hydroxymethyltransferase family protein	OG_01138	EYR69153.1	serine hydroxymethyltransferase [Lysobacter capsici AZ78]
OG_01139	LC55x_3039	LC55x_3039; SCO1/SenC family protein	OG_01139	EYR67828.1	hypothetical protein AZ78_13630 [Lysobacter capsici AZ78]
OG_01140	LC55x_1898	rnr; ribonuclease R	OG_01140	EYR65994.1	exoribonuclease R [Lysobacter capsici AZ78]
OG_01141	LC55x_5240	LC55x_5240; multidrug resistance efflux transporter family protein	OG_01141	EYR68896.1	membrane protein [Lysobacter capsici AZ78]
OG_01142	LC55x_1747	LC55x_1747; hypothetical protein	OG_01142	EYR65702.1	hypothetical protein AZ78_24945 [Lysobacter capsici AZ78]
OG_01143	LC55x_3048	LC55x_3048; nlpE N-terminal domain protein	OG_01143	EYR67834.1	hypothetical protein AZ78_13670 [Lysobacter capsici AZ78]
OG_01144	LC55x_5005	LC55x_5005; UDP-glucoronosyl and UDP-glucosyl transferase family	OG_01144	EYR67224.1	hypothetical protein AZ78_16770 [Lysobacter capsici AZ78]
OG_01145	LC55x_2189	uvrC; excinuclease ABC subunit C	OG_01145	EYR67405.1	excinuclease ABC subunit C [Lysobacter capsici AZ78]
OG_01146	LC55x_596	LC55x_596; citrate transporter family protein	OG_01146	EYR70007.1	citrate transporter [Lysobacter capsici AZ78]
OG_01147	LC55x_1294	LC55x_1294; alanine racemase, N-terminal domain protein	OG_01147	EYR67660.1	pyridoxal phosphate biosynthesis protein [Lysobacter capsici AZ78]
OG_01148	LC55x_1041	pilQ; type IV pilus secretin PilQ family protein	OG_01148	EYR67743.1	fimbrial protein [Lysobacter capsici AZ78]

OG_01149	LC55x_999	LC55x_999; conserved hypothetical protein	OG_01149	EYR67775.1	hypothetical protein AZ78_14265 [Lysobacter capsici AZ78]
OG_01150	LC55x_2147	LC55x_2147; ABC transporter family protein	OG_01150	EYR67369.1	ABC transporter ATP-binding protein [Lysobacter capsici AZ78]
OG_01151	LC55x_825	LC55x_825; phasin family protein	OG_01151	EYR67583.1	hypothetical protein AZ78_14435 [Lysobacter capsici AZ78]
OG_01152	LC55x_4685	xerC; tyrosine recombinase XerC	OG_01152	EYR70183.1	tyrosine recombinase XerC [Lysobacter capsici AZ78]
OG_01153	LC55x_5086	LC55x_5086; putative 3-oxoacyl-[acyl-carrier protein] reductase	OG_01153	EYR68061.1	phosphotransferase [Lysobacter capsici AZ78]
OG_01154	LC55x_1424	LC55x_1424; putative thioredoxin	OG_01154	EYR67149.1	hypothetical protein AZ78_17150 [Lysobacter capsici AZ78]
OG_01155	LC55x_773	LC55x_773; outer membrane beta-barrel domain protein	OG_01155	EYR67618.1	hypothetical protein AZ78_14675 [Lysobacter capsici AZ78]
OG_01156	LC55x_422	speE; spermidine synthase	OG_01156	EYR65615.1	spermidine synthase [Lysobacter capsici AZ78]
OG_01157	LC55x_978	LC55x_978; tonB-dependent siderophore receptor family protein	OG_01157	EYR65391.1	ferrisiderophore receptor [Lysobacter capsici AZ78]
OG_01158	LC55x_4564	LC55x_4564; bacterial regulatory helix-turn-helix , lysR family protein	OG_01158	EYR68376.1	LysR family transcriptional regulator [Lysobacter capsici AZ78]
OG_01159	LC55x_3794	LC55x_3794; conserved hypothetical protein	OG_01159	EYR67101.1	hypothetical protein AZ78_17725 [Lysobacter capsici AZ78]
OG_01160	LC55x_3024	LC55x_3024; 2OG-Fe(II) oxygenase superfamily protein	OG_01160	EYR67820.1	flavonol synthase [Lysobacter capsici AZ78]
OG_01161	LC55x_928	leuD; 3-isopropylmalate dehydratase, small subunit	OG_01161	EYR66832.1	isopropylmalate isomerase [Lysobacter capsici AZ78]
OG_01162	LC55x_908	LC55x_908; mitochondrial fission ELM1 family protein	OG_01162	EYR66851.1	nucleoside-diphosphate-sugar epimerase [Lysobacter capsici AZ78]
OG_01163	LC55x_3498	LC55x_3498; methyltransferase domain protein	OG_01163	EYR66627.1	hypothetical protein AZ78_20155 [Lysobacter capsici AZ78]
OG_01164	LC55x_3655	LC55x_3655; ankyrin repeat family protein	OG_01164	EYR68643.1	ABC transporter permease [Lysobacter capsici AZ78]
OG_01165	LC55x_4714	LC55x_4714; cation diffusion facilitator transporter family protein	OG_01165	EYR65676.1	cation diffusion facilitator family transporter [Lysobacter capsici AZ78
OG_01166	LC55x_1772	LC55x_1772; fecR family protein	OG_01166	EYR67273.1	peptidoglycan-binding protein LysM [Lysobacter capsici AZ78]
OG_01167	LC55x_5244	bioF; 8-amino-7-oxononanoate synthase	OG_01167	EYR68900.1	8-amino-7-oxononanoate synthase [Lysobacter capsici AZ78]
OG_01168	LC55x_4220	LC55x_4220; conserved hypothetical protein	OG_01168	EYR69081.1	hypothetical protein AZ78_06205 [Lysobacter capsici AZ78]
OG_01169	LC55x_2462	LC55x_2462; hypothetical protein	OG_01169	EYR69815.1	hypothetical protein AZ78_03420 [Lysobacter capsici AZ78]
OG_01170	LC55x_4044	LC55x_4044; RNA polymerase sigma factor, sigma-70 family protein	OG_01170	EYR65823.1	hypothetical protein AZ78_24235 [Lysobacter capsici AZ78]
OG_01171	LC55x_3016	LC55x_3016; NADH-ubiquinone/plastoquinone oxidoreductase, chain	OG_01171	EYR67814.1	NADH:ubiquinone oxidoreductase subunit A [Lysobacter capsici AZ7
OG_01172	LC55x_2398	LC55x_2398; hypothetical protein	OG_01172	EYR69831.1	hypothetical protein AZ78_03745 [Lysobacter capsici AZ78]
OG_01173	LC55x_1860	LC55x_1860; cytidylyltransferase family protein	OG_01173	EYR66052.1	phosphatidate cytidylyltransferase [Lysobacter capsici AZ78]
OG_01174	LC55x_2170	sdhC; succinate dehydrogenase, cytochrome b556 subunit	OG_01174	EYR67388.1	succinate dehydrogenase [Lysobacter capsici AZ78]
OG_01175	LC55x_3438	rpoE; RNA polymerase sigma factor RpoE	OG_01175	EYR66493.1	RNA polymerase sigma factor RpoE [Lysobacter capsici AZ78]
OG_01176	LC55x_72	phhA; phenylalanine-4-hydroxylase	OG_01176	EYR69747.1	phenylalanine 4-monooxygenase [Lysobacter capsici AZ78]
OG_01177	LC55x_878	aroQ; 3-dehydroquinate dehydratase, type II	OG_01177	EYR66879.1	3-dehydroquinate dehydratase [Lysobacter capsici AZ78]
OG_01178	LC55x_758	rsuA; 16S rRNA pseudouridine 516 synthase	OG_01178	EYR67631.1	pseudouridine synthase [Lysobacter capsici AZ78]
OG_01179	LC55x_1932	LC55x_1932; conserved hypothetical protein	OG_01179	EYR66013.1	hypothetical protein AZ78_23365 [Lysobacter capsici AZ78]
OG_01180	LC55x_4097	apaH; bis	OG_01180	EYR65498.1	diadenosine tetraphosphatase [Lysobacter capsici AZ78]
OG_01181	LC55x_4489	LC55x_4489; conserved hypothetical protein	OG_01181	EYR68420.1	hypothetical protein AZ78_10175 [Lysobacter capsici AZ78]
OG_01182	LC55x_1726	secD; secD	OG_01182	EYR70376.1	preprotein translocase subunit SecD [Lysobacter capsici AZ78]
OG_01183	LC55x_846	LC55x_846; major Facilitator Superfamily protein	OG_01183	EYR67568.1	glycerol acyltransferase [Lysobacter capsici AZ78]
OG_01184	LC55x_4191	tolA; protein TolA	OG_01184	EYR68158.1	membrane protein TolA [Lysobacter capsici AZ78]
OG_01185	LC55x_2995	rpsO; ribosomal protein S15	OG_01185	EYR67795.1	30S ribosomal protein S15 [Lysobacter capsici AZ78]
OG_01186	LC55x_285	LC55x_285; RNA polymerase sigma factor, sigma-70 family protein	OG_01186	EYR67012.1	transcriptional regulator [Lysobacter capsici AZ78]
OG_01187	LC55x_3950	rplB; ribosomal protein L2	OG_01187	EYR66701.1	50S ribosomal protein L2 [Lysobacter capsici AZ78]
OG_01188	LC55x_3999	LC55x_3999; asparaginase family protein	OG_01188	EYR66728.1	asparaginase [Lysobacter capsici AZ78]
OG_01189	LC55x_3100	LC55x_3100; conserved hypothetical protein	OG_01189	EYR65449.1	metal-dependent hydrolase [Lysobacter capsici AZ78]
OG_01190	LC55x_1970	LC55x_1970; cytochrome C assembly family protein	OG_01190	EYR66775.1	membrane protein [Lysobacter capsici AZ78]
OG_01191	LC55x_4341	katB; catalase	OG_01191	EYR69182.1	catalase [Lysobacter capsici AZ78]
OG_01192	LC55x_2247	LC55x_2247; restriction endonuclease family protein	OG_01192	EYR67467.1	hypothetical protein AZ78_16045 [Lysobacter capsici AZ78]
OG_01193	LC55x_96	LC55x_96; mce related family protein	OG_01193	EYR69760.1	mammalian cell entry protein [Lysobacter capsici AZ78]
OG_01194	LC55x_420	LC55x_420; conserved hypothetical family protein	OG_01194	EYR65618.1	membrane protein [Lysobacter capsici AZ78]
OG_01195	LC55x_2703	LC55x_2703; major Facilitator Superfamily protein	OG_01195	EYR69289.1	major facilitator transporter [Lysobacter capsici AZ78]
OG_01196	LC55x_2970	LC55x_2970; bacterial regulatory , Fis family protein	OG_01196	EYR66192.1	chemotaxis protein CheY [Lysobacter capsici AZ78]
OG_01197	LC55x_1049	LC55x_1049; sodium:dicarboxylate symporter family protein	OG_01197	EYR67737.1	proton glutamate symport protein [Lysobacter capsici AZ78]
OG_01198	LC55x_3066	LC55x_3066; hypothetical protein	OG_01198	EYR67881.1	hypothetical protein AZ78_13760 [Lysobacter capsici AZ78]
OG_01199	LC55x_1994	LC55x_1994; hypothetical protein	OG_01199	EYR66761.1	hypothetical protein AZ78_19305 [Lysobacter capsici AZ78]
OG_01200	LC55x_3584	minD; septum site-determining protein MinD	OG_01200	EYR67531.1	cell division inhibitor MinD [Lysobacter capsici AZ78]
OG_01201	LC55x_1613	LC55x_1613; stringent starvation B family protein	OG_01201	EYR70318.1	peptidase [Lysobacter capsici AZ78]
OG_01202	LC55x_135	LC55x_135; RNA polymerase sigma factor, sigma-70 family protein	OG_01202	EYR69786.1	RNA polymerase subunit sigma-24 [Lysobacter capsici AZ78]
OG_01203	LC55x_3900	LC55x_3900; sugar (and other) transporter family protein	OG_01203	EYR66658.1	MFS transporter [Lysobacter capsici AZ78]
OG_01204	LC55x_419	LC55x_419; bacterial regulatory helix-turn-helix , lysR family protein	OG_01204	EYR65619.1	LysR family transcriptional regulator [Lysobacter capsici AZ78]
OG_01205	LC55x_2648	LC55x_2648; heparinase II/III-like family protein	OG_01205	EYR68250.1	hypothetical protein AZ78_11330 [Lysobacter capsici AZ78]
OG_01206	LC55x_4904	gluP; glucose/galactose transporter WARNING family protein	OG_01206	EYR68709.1	MFS transporter [Lysobacter capsici AZ78]
OG_01207	LC55x_5683	queF; queuine synthase	OG_01207	EYR65875.1	7-cyano-7-deazaguanine reductase [Lysobacter capsici AZ78]
OG_01208	LC55x_4677	ubiE; 2-OCTAPRENYL-METHOXY-BENZOQ-METH bifunctional 2-oc	OG_01208	EYR70175.1	ubiquinone/menaquinone biosynthesis methyltransferase [Lysobacte
OG_01209	LC55x_2419	LC55x_2419; methionine aminotransferase, PLP-dependent	OG_01209	EYR69579.1	aminotransferase [Lysobacter capsici AZ78]
OG_01210	LC55x_5194	LC55x_5194; hypothetical protein	OG_01210	EYR68991.1	hypothetical protein AZ78_07320 [Lysobacter capsici AZ78]
OG_01211	LC55x_2313	LC55x_2313; conserved hypothetical protein	OG_01211	EYR69651.1	hypothetical protein AZ78_04135 [Lysobacter capsici AZ78]
OG_01212	LC55x_861	LC55x_861; putative phosphatase, inner membrane protein	OG_01212	EYR66896.1	ser/threonine protein phosphatase [Lysobacter capsici AZ78]
OG_01214	LC55x_2345	LC55x_2345; conserved hypothetical protein	OG_01214	EYR69625.1	hypothetical protein AZ78_03980 [Lysobacter capsici AZ78]
OG_01215	LC55x_2359	LC55x_2359; tRNA-specific adenosine deaminase monomer	OG_01215	EYR69618.1	deoxycytidylate deaminase [Lysobacter capsici AZ78]
OG_01216	LC55x_3713	hutG; N-formylglutamate deformylase	OG_01216	EYR65909.1	N-formylglutamate amidohydrolase [Lysobacter capsici AZ78]
OG_01217	LC55x_3328	LC55x_3328; amidohydrolase family Protein OLEI01672_1_465	OG_01217	EYR70079.1	N-ethylammeline chlorohydrolase [Lysobacter capsici AZ78]
OG_01218	LC55x_328	LC55x_328; ABC transporter family protein	OG_01218	EYR68109.1	ABC transporter ATP-binding protein [Lysobacter capsici AZ78]
OG_01219	LC55x_1742	LC55x_1742; TPR repeat family protein	OG_01219	EYR65707.1	hypothetical protein AZ78_24970, partial [Lysobacter capsici AZ78]
OG_01220	LC55x_1685	LC55x_1685; HNH endonuclease family protein	OG_01220	EYR70350.1	HNH endonuclease [Lysobacter capsici AZ78]
OG_01221	LC55x_4195	ruvB; holliday junction DNA helicase RuvB	OG_01221	EYR68154.1	Holliday junction DNA helicase RuvB [Lysobacter capsici AZ78]
OG_01222	LC55x_3724	slyD; FKBP-type peptidyl-prolyl cis-trans isomerase slyD	OG_01222	EYR65901.1	peptidyl-prolyl cis-trans isomerase [Lysobacter capsici AZ78]
OG_01223	LC55x_2566	LC55x_2566; short chain dehydrogenase family protein	OG_01223	EYR68327.1	short-chain dehydrogenase [Lysobacter capsici AZ78]
OG_01224	LC55x_3603	LC55x_3603; conserved hypothetical protein	OG_01224	EYR67545.1	hypothetical protein AZ78_15475 [Lysobacter capsici AZ78]
OG_01225	LC55x_2499	prfB; peptide chain release factor 2	OG_01225	EYR69519.1	peptide chain release factor 2 [Lysobacter capsici AZ78]
OG_01226	LC55x_3334	gluQ; glutamyl-queuosine tRNA(Asp) synthetase	OG_01226	EYR66554.1	glutamyl-Q tRNA(Asp) ligase [Lysobacter capsici AZ78]
OG_01227	LC55x_4245	LC55x_4245; conserved hypothetical protein	OG_01227	EYR69100.1	hypothetical protein AZ78_06315 [Lysobacter capsici AZ78]
OG_01228	LC55x_1604	LC55x_1604; acetyltransferase family protein	OG_01228	EYR70311.1	N-acetyltransferase [Lysobacter capsici AZ78]
OG_01229	LC55x_3920	pqqD; coenzyme PQQ biosynthesis protein PqqD	OG_01229	EYR66672.1	coenzyme PQQ biosynthesis protein D [Lysobacter capsici AZ78]
OG_01230	LC55x_4138	LC55x_4138; conserved hypothetical protein	OG_01230	EYR66343.1	membrane protein [Lysobacter capsici AZ78]

OG_01231	LC55x_3159	LC55x_3159; zinc carboxypeptidase family protein	OG_01231	EYR66940.1	peptidase [Lysobacter capsici AZ78]
OG_01232	LC55x_4732	LC55x_4732; conserved hypothetical protein	OG_01232	EYR66112.1	hypothetical protein AZ78_22910 [Lysobacter capsici AZ78]
OG_01233	LC55x_1835	LC55x_1835; modulator of DNA gyrase family protein	OG_01233	EYR66027.1	protease TldD [Lysobacter capsici AZ78]
OG_01234	LC55x_2087	LC55x_2087; RNA pseudouridylate synthase family protein	OG_01234	EYR66417.1	pseudouridine synthase [Lysobacter capsici AZ78]
OG_01235	LC55x_3946	rplP; ribosomal protein L16	OG_01235	EYR66697.1	50S ribosomal protein L16 [Lysobacter capsici AZ78]
OG_01236	LC55x_1143	LC55x_1143; peptidyl-Asp metalloendopeptidase domain protein	OG_01236	EYR68516.1	peptidase [Lysobacter capsici AZ78]
OG_01237	LC55x_3009	LC55x_3009; NADH dehydrogenase family protein	OG_01237	EYR67807.1	NADH:ubiquinone oxidoreductase subunit H [Lysobacter capsici AZ7
OG_01238	LC55x_3345	LC55x_3345; trbL/VirB6 plasmid conjugal transfer family protein	OG_01238	EYR66547.1	type VI secretion protein [Lysobacter capsici AZ78]
OG_01239	LC55x_634	LC55x_634; conserved hypothetical protein	OG_01239	EYR69984.1	hypothetical protein AZ78_02310 [Lysobacter capsici AZ78]
OG_01240	LC55x_3061	LC55x_3061; conserved hypothetical protein	OG_01240	EYR67877.1	hypothetical protein AZ78_13730 [Lysobacter capsici AZ78]
OG_01241	LC55x_3966	secE; preprotein translocase, SecE subunit	OG_01241	EYR65481.1	preprotein translocase subunit SecE [Lysobacter capsici AZ78]
OG_01242	LC55x_5226	LC55x_5226; RNase_H superfamily protein	OG_01242	EYR68887.1	exonuclease [Lysobacter capsici AZ78]
OG_01243	LC55x_1191	LC55x_1191; DSBA-like thioredoxin domain protein	OG_01243	EYR65792.1	hypothetical protein AZ78_24545 [Lysobacter capsici AZ78]
OG_01244	LC55x_3389	LC55x_3389; hypothetical protein	OG_01244	EYR66526.1	hypothetical protein AZ78_20480 [Lysobacter capsici AZ78]
OG_01245	LC55x_4693	LC55x_4693; prolyl oligopeptidase family protein	OG_01245	EYR70190.1	peptidase [Lysobacter capsici AZ78]
OG_01246	LC55x_2917	zipA; cell division protein ZipA	OG_01246	EYR69472.1	cell division protein ZipA [Lysobacter capsici AZ78]
OG_01247	LC55x_1034	LC55x_1034; conserved hypothetical protein	OG_01247	EYR67749.1	hypothetical protein AZ78_14110 [Lysobacter capsici AZ78]
OG_01248	LC55x_2488	LC55x_2488; conserved hypothetical protein	OG_01248	EYR69526.1	copper homeostasis protein CutC [Lysobacter capsici AZ78]
OG_01249	LC55x_4398	rpsI; 30S ribosomal subunit protein S9	OG_01249	EYR69228.1	30S ribosomal protein S9 [Lysobacter capsici AZ78]
OG_01250	LC55x_484	LC55x_484; cytochrome c oxidase subunit III family protein	OG_01250	EYR66456.1	MFS transporter [Lysobacter capsici AZ78]
OG_01251	LC55x_4206	LC55x_4206; concanavalin A-like lectin/glucanases superfamily prote	OG_01251	EYR65346.1	hypothetical protein AZ78_26920 [Lysobacter capsici AZ78]
OG_01252	LC55x_3833	LC55x_3833; merC mercury resistance family protein	OG_01252	EYR65746.1	membrane protein [Lysobacter capsici AZ78]
OG_01253	LC55x_2131	LC55x_2131; transcriptional regulatory , C terminal family protein	OG_01253	EYR66377.1	hypothetical protein AZ78_21185 [Lysobacter capsici AZ78]
OG_01254	LC55x_5235	LC55x_5235; FMN-dependent dehydrogenase family protein	OG_01254	EYR68892.1	glutamate synthase subunit alpha [Lysobacter capsici AZ78]
OG_01255	LC55x_1280	aroB; 3-dehydroquinate synthase	OG_01255	EYR67670.1	3-dehydroquinate synthase [Lysobacter capsici AZ78]
OG_01256	LC55x_129	LC55x_129; M61 glycyl aminopeptidase family protein	OG_01256	EYR69781.1	peptidase M61 [Lysobacter capsici AZ78]
OG_01257	LC55x_2742	LC55x_2742; hypothetical protein	OG_01257	EYR69312.1	hypothetical protein AZ78_05315 [Lysobacter capsici AZ78]
OG_01258	LC55x_1007	LC55x_1007; conserved hypothetical protein	OG_01258	EYR67769.1	hypothetical protein AZ78_14230 [Lysobacter capsici AZ78]
OG_01259	LC55x_637	LC55x_637; conserved hypothetical protein	OG_01259	EYR69981.1	hypothetical protein AZ78_02295 [Lysobacter capsici AZ78]
OG_01260	LC55x_842	LC55x_842; fecR family protein	OG_01260	EYR67571.1	membrane protein [Lysobacter capsici AZ78]
OG_01261	LC55x_30	LC55x_30; hypothetical protein	OG_01261	EYR69864.1	hypothetical protein AZ78_04630 [Lysobacter capsici AZ78]
OG_01262	LC55x_914	LC55x_914; O-Antigen ligase family protein	OG_01262	EYR66845.1	membrane protein [Lysobacter capsici AZ78]
OG_01263	LC55x_5713	LC55x_5713; magnesium-dependent DNase	OG_01263	EYR65845.1	preprotein translocase subunit TatD [Lysobacter capsici AZ78]
OG_01264	LC55x_857	purH; phosphoribosylaminoimidazolecarboxamide formyltransferase/I	OG_01264	EYR66899.1	purine biosynthesis protein purH [Lysobacter capsici AZ78]
OG_01265	LC55x_4412	trpD; anthranilate phosphoribosyltransferase	OG_01265	EYR69240.1	anthranilate phosphoribosyltransferase [Lysobacter capsici AZ78]
OG_01266	LC55x_4390	LC55x_4390; twitching motility family protein	OG_01266	EYR69223.1	twitching motility protein PilT [Lysobacter capsici AZ78]
OG_01267	LC55x_4333	LC55x_4333; putative permease YjgP/YjgQ family protein	OG_01267	EYR69177.1	membrane protein [Lysobacter capsici AZ78]
OG_01268	LC55x_1565	LC55x_1565; H+ symporter) family protein	OG_01268	EYR70285.1	MFS transporter [Lysobacter capsici AZ78]
OG_01269	LC55x_3540	aqpZ; aqpZ - water MIP channel	OG_01269	EYR67492.1	aquaporin Z [Lysobacter capsici AZ78]
OG_01270	LC55x_3027	accD; acetyl-CoA carboxylase, carboxyl transferase, beta subunit	OG_01270	EYR65368.1	acetyl-CoA carboxylase carboxyl transferase subunit beta [Lysobacte
OG_01271	LC55x_869	LC55x_869; bacterial regulatory , Fis family protein	OG_01271	EYR66888.1	Fis family transcriptional regulator [Lysobacter capsici AZ78]
OG_01272	LC55x_5181	LC55x_5181; glutamine amidotransferase class-I family protein	OG_01272	EYR68854.1	glutamine amidotransferase [Lysobacter capsici AZ78]
OG_01273	LC55x_2980	LC55x_2980; short chain dehydrogenase family protein	OG_01273	EYR65351.1	oxidoreductase [Lysobacter capsici AZ78]
OG_01274	LC55x_3929	rpoA; DNA-directed RNA polymerase, alpha subunit	OG_01274	EYR66680.1	DNA-directed RNA polymerase subunit alpha [Lysobacter capsici AZ
OG_01275	LC55x_3448	fabG; 3-oxoacyl-[acyl-carrier-protein] reductase	OG_01275	EYR66595.1	3-ketoacyl-ACP reductase [Lysobacter capsici AZ78]
OG_01276	LC55x_362	LC55x_362; conserved hypothetical protein	OG_01276	EYR68135.1	membrane protein [Lysobacter capsici AZ78]
OG_01277	LC55x_4141	LC55x_4141; response regulator	OG_01277	EYR66340.1	hypothetical protein AZ78_21555 [Lysobacter capsici AZ78]
OG_01278	LC55x_2141	fpr; ferredoxin--NADP+ reductase	OG_01278	EYR66368.1	ferredoxin--NADP reductase [Lysobacter capsici AZ78]
OG_01279	LC55x_3559	LC55x_3559; endonuclease/Exonuclease/phosphatase family protein	OG_01279	EYR67509.1	endonuclease [Lysobacter capsici AZ78]
OG_01280	LC55x_1204	LC55x_1204; conserved hypothetical protein	OG_01280	EYR65781.1	membrane protein [Lysobacter capsici AZ78]
OG_01281	LC55x_5140	LC55x_5140; hypothetical protein	OG_01281	EYR68081.1	hypothetical protein AZ78_12470 [Lysobacter capsici AZ78]
OG_01282	LC55x_1392	LC55x_1392; conserved hypothetical protein	OG_01282	EYR67176.1	ABC transporter permease [Lysobacter capsici AZ78]
OG_01283	LC55x_5136	LC55x_5136; 4'-phosphopantetheinyl transferase superfamily protein	OG_01283	EYR68024.1	4-phosphopantetheinyl transferase [Lysobacter capsici AZ78]
OG_01284	LC55x_752	LC55x_752; ABC transporter family protein	OG_01284	EYR67636.1	ABC transporter ATP-binding protein [Lysobacter capsici AZ78]
OG_01285	LC55x_4337	LC55x_4337; DNA polymerase III chi subunit, HolC family protein	OG_01285	EYR69180.1	DNA polymerase III subunit chi [Lysobacter capsici AZ78]
OG_01286	LC55x_923	glpK; glycerol kinase	OG_01286	EYR66836.1	glycerol kinase [Lysobacter capsici AZ78]
OG_01287	LC55x_3654	LC55x_3654; conserved hypothetical protein	OG_01287	EYR68644.1	phospholipase [Lysobacter capsici AZ78]
OG_01288	LC55x_3269	LC55x_3269; hisB	OG_01288	EYR70040.1	imidazoleglycerol-phosphate dehydratase [Lysobacter capsici AZ78]
OG_01289	LC55x_342	LC55x_342; conserved hypothetical protein	OG_01289	EYR68120.1	hypothetical protein AZ78_11880 [Lysobacter capsici AZ78]
OG_01290	LC55x_4503	LC55x_4503; N-acetylmuramoyl-L-alanine amidase family protein	OG_01290	EYR68410.1	N-acetylmuramoyl-L-alanine amidase [Lysobacter capsici AZ78]
OG_01291	LC55x_5023	ung; uracil-DNA glycosylase	OG_01291	EYR66317.1	uracil-DNA glycosylase [Lysobacter capsici AZ78]
OG_01292	LC55x_1213	LC55x_1213; sugar (and other) transporter family protein	OG_01292	EYR65773.1	MFS transporter [Lysobacter capsici AZ78]
OG_01293	LC55x_2046	pgi; glucose-6-phosphate isomerase	OG_01293	EYR65600.1	glucose-6-phosphate isomerase [Lysobacter capsici AZ78]
OG_01294	LC55x_4336	LC55x_4336; putative cytosol aminopeptidase	OG_01294	EYR69179.1	multifunctional aminopeptidase A [Lysobacter capsici AZ78]
OG_01295	LC55x_3189	ldh; leucine dehydrogenase	OG_01295	EYR66909.1	leucine dehydrogenase [Lysobacter capsici AZ78]
OG_01296	LC55x_4492	LC55x_4492; EF hand family protein	OG_01296	EYR68418.1	hypothetical protein AZ78_10160 [Lysobacter capsici AZ78]
OG_01297	LC55x_1306	pyrB; aspartate carbamoyltransferase	OG_01297	EYR69926.1	aspartate carbamoyltransferase [Lysobacter capsici AZ78]
OG_01298	LC55x_1382	LC55x_1382; peptidase M20/M25/M40 family protein	OG_01298	EYR67182.1	peptidase M20 [Lysobacter capsici AZ78]
OG_01299	LC55x_4296	LC55x_4296; methyltransferase domain protein	OG_01299	EYR69150.1	SAM-dependent methyltransferase [Lysobacter capsici AZ78]
OG_01300	LC55x_1928	LC55x_1928; rhodanese-like domain protein	OG_01300	EYR65973.1	sulfurtransferase [Lysobacter capsici AZ78]
OG_01301	LC55x_2944	LC55x_2944; bolA-like family protein	OG_01301	EYR66216.1	cell division protein BolA [Lysobacter capsici AZ78]
OG_01302	LC55x_505	LC55x_505; conserved hypothetical protein	OG_01302	EYR66439.1	hypothetical protein AZ78_20840 [Lysobacter capsici AZ78]
OG_01303	LC55x_3567	LC55x_3567; putative integral membrane protein	OG_01303	EYR67516.1	hypothetical protein AZ78_15330 [Lysobacter capsici AZ78]
OG_01304	LC55x_2293	gntY; iron-sulfur cluster scaffold protein	OG_01304	EYR69665.1	Fe/S biogenesis protein NfuA [Lysobacter capsici AZ78]
OG_01305	LC55x_4703	LC55x_4703; DMSO reductase anchor subunit family protein	OG_01305	EYR65665.1	hypothetical protein AZ78_25020 [Lysobacter capsici AZ78]
OG_01306	LC55x_60	LC55x_60; WYL domain protein	OG_01306	EYR69738.1	DeoR faimly transcriptional regulator [Lysobacter capsici AZ78]
OG_01307	LC55x_2175	LC55x_2175; MAPEG family protein	OG_01307	EYR67393.1	hypothetical protein AZ78_15720 [Lysobacter capsici AZ78]
OG_01308	LC55x_2377	LC55x_2377; type I phosphodiesterase / nucleotide pyrophosphatase	OG_01308	EYR69607.1	membrane-associated metal-dependent hydrolase [Lysobacter capsi
OG_01309	LC55x_4428	LC55x_4428; conserved hypothetical protein	OG_01309	EYR65469.1	membrane protein [Lysobacter capsici AZ78]
OG_01310	LC55x_2333	rlmN; 23S rRNA m2A2503 methyltransferase	OG_01310	EYR69635.1	50S rRNA methyltransferase [Lysobacter capsici AZ78]
OG_01311	LC55x_770	LC55x_770; conserved hypothetical protein	OG_01311	EYR67620.1	hypothetical protein AZ78_14685 [Lysobacter capsici AZ78]

OG_01312	LC55x_2426	LC55x_2426; trbC/VIRB2 family protein
OG_01313	LC55x_418	LC55x_418; nitrogen regulatory P-II family protein
OG_01314	LC55x_1885	asnB; asparagine synthase
OG_01315	LC55x_1172	LC55x_1172; na+ dependent nucleoside transporter family protein
OG_01316	LC55x_2781	LC55x_2781; putative FMN-binding domain protein
OG_01317	LC55x_2309	LC55x_2309; zinc-binding dehydrogenase family protein
OG_01318	LC55x_203	LC55x_203; bacitracin resistance BacA family protein
OG_01319	LC55x_3313	LC55x_3313; major Facilitator Superfamily protein
OG_01320	LC55x_2924	LC55x_2924; tonB dependent receptor family protein
OG_01321	LC55x_4193	tolQ; protein TolQ
OG_01322	LC55x_3539	LC55x_3539; hypothetical protein
OG_01323	LC55x_2664	LC55x_2664; cheR methyltransferase, SAM binding domain protein
OG_01324	LC55x_1247	metB; O-succinylhomoserine (thiol)-lyase
OG_01325	LC55x_5629	LC55x_5629; subtilase family protein
OG_01326	LC55x_4637	dacC; penicillin-binding protein 6
OG_01327	LC55x_2393	LC55x_2393; pspC domain protein
OG_01328	LC55x_3803	LC55x_3803; conserved hypothetical protein
OG_01329	LC55x_3995	yihY; yihY family inner membrane domain protein
OG_01330	LC55x_2072	LC55x_2072; conserved hypothetical protein
OG_01331	LC55x_3797	LC55x_3797; S4 domain protein
OG_01332	LC55x_4109	LC55x_4109; integral membrane TerC family protein
OG_01333	LC55x_584	LC55x_584; conserved hypothetical protein
OG_01334	LC55x_3287	pgaA; poly-beta-1,6 N-acetyl-D-glucosamine export porin PgaA
OG_01335	LC55x_3738	LC55x_3738; sodium:dicarboxylate symporter family protein
OG_01336	LC55x_4463	coaX; pantothenate kinase, type III family protein
OG_01337	LC55x_4146	gshB; glutathione synthase
OG_01338	LC55x_1341	LC55x_1341; hypothetical protein
OG_01339	LC55x_3201	LC55x_3201; dnaJ domain protein
OG_01340	LC55x_3312	LC55x_3312; fe-S metabolism associated domain protein
OG_01341	LC55x_4165	LC55x_4165; ferritin-like domain protein
OG_01342	LC55x_1114	trmB; tRNA (guanine-N(7)-)-methyltransferase
OG_01343	LC55x_3980	LC55x_3980; TPR repeat family protein
OG_01344	LC55x_2497	LC55x_2497; glycosyl hydrolases family 2, sugar binding domain pro
OG_01345	LC55x_5049	LC55x_5049; acetyltransferase family protein
OG_01346	LC55x_2250	LC55x_2250; conserved hypothetical protein
OG_01347	LC55x_5609	LC55x_5609; HAMP domain protein
OG_01348	LC55x_4434	LC55x_4434; putative exported lectin/glucanase
OG_01349	LC55x_3752	LC55x_3752; conserved hypothetical protein
OG_01350	LC55x_4710	LC55x_4710; hypothetical protein
OG_01351	LC55x_5113	LC55x_5113; delta-aminolevulinic acid dehydratase family protein
OG_01352	LC55x_1179	LC55x_1179; hemin uptake hemP family protein
OG_01353	LC55x_4745	ohrR; transcriptional regulator OhrR
OG_01354	LC55x_209	amt; ammonium transporter family protein
OG_01355	LC55x_1159	metK; methionine adenosyltransferase
OG_01356	LC55x_3731	LC55x_3731; NUDIX domain protein
OG_01357	LC55x_1817	tig; trigger factor
OG_01358	LC55x_4429	LC55x_4429; hypothetical protein
OG_01359	LC55x_1704	LC55x_1704; hypothetical protein
OG_01360	LC55x_1864	LC55x_1864; penicillinase repressor family protein
OG_01361	LC55x_5139	LC55x_5139; putative chromosome-partitioning protein parB
OG_01362	LC55x_3436	LC55x_3436; peptidase Do family protein
OG_01363	LC55x_3710	LC55x_3710; hypothetical protein
OG_01364	LC55x_4076	LC55x_4076; conserved hypothetical protein
OG_01365	LC55x_1239	LC55x_1239; channel , hemolysin III family protein
OG_01366	LC55x_3060	LC55x_3060; luciferase oxidoreductase, group 1 family protein
OG_01367	LC55x_461	gpmI; 2,3-bisphosphoglycerate-independent phosphoglycerate mutas
OG_01368	LC55x_2772	rpsF; ribosomal protein S6
OG_01369	LC55x_763	LC55x_763; conserved hypothetical protein
OG_01370	LC55x_2346	LC55x_2346; conserved hypothetical protein
OG_01371	LC55x_3670	hfq; RNA chaperone Hfq
OG_01372	LC55x_324	LC55x_324; 2OG-Fe(II) oxygenase superfamily protein
OG_01373	LC55x_4260	coaE; dephospho-CoA kinase
OG_01374	LC55x_2257	pheA; chorismate mutase
OG_01375	LC55x_1079	LC55x_1079; conserved hypothetical protein
OG_01376	LC55x_3934	rplO; ribosomal protein L15
OG_01377	LC55x_3397	LC55x_3397; thiamine pyrophosphate enzyme, C-terminal TPP bindi
OG_01378	LC55x_1325	lptA; lipopolysaccharide transport periplasmic protein LptA
OG_01379	LC55x_4318	LC55x_4318; fimbrial assembly family protein
OG_01380	LC55x_1778	LC55x_1778; conserved hypothetical integral membrane family prote
OG_01381	LC55x_1842	xseB; exodeoxyribonuclease VII, small subunit
OG_01382	LC55x_595	phbB; acetoacetyl-CoA reductase family protein
OG_01383	LC55x_5127	LC55x_5127; RDD family protein
OG_01384	LC55x_95	LC55x_95; toluene tolerance, Ttg2 family protein
OG_01385	LC55x_2838	LC55x_2838; hypothetical protein
OG_01386	LC55x_2048	LC55x_2048; putative iron-sulfur cluster-binding protein
OG_01387	LC55x_48	LC55x_48; fructose-1-6-bisphosphatase family protein
OG_01388	LC55x_2357	LC55x_2357; conserved hypothetical protein
OG_01389	LC55x_1092	LC55x_1092; acetyltransferase family protein
OG_01390	LC55x_4529	LC55x_4529; sir2 family protein
OG_01391	LC55x_524	LC55x_524; disulfide oxidoreductase
OG_01392	LC55x_4456	LC55x_4456; conserved hypothetical protein

OG_01312	EYR69572.1	type VI secretion protein [Lysobacter capsici AZ78]
OG_01313	EYR65620.1	nitrogen regulatory protein P-II 1 [Lysobacter capsici AZ78]
OG_01314	EYR66001.1	asparagine synthetase B [Lysobacter capsici AZ78]
OG_01315	EYR68538.1	nucleoside transporter NupC [Lysobacter capsici AZ78]
OG_01316	EYR69345.1	regulatory protein [Lysobacter capsici AZ78]
OG_01317	EYR69655.1	2-alkenal reductase [Lysobacter capsici AZ78]
OG_01318	EYR66143.1	UDP pyrophosphate phosphatase [Lysobacter capsici AZ78]
OG_01319	EYR70069.1	multidrug transporter [Lysobacter capsici AZ78]
OG_01320	EYR66235.1	TonB-denpendent receptor [Lysobacter capsici AZ78]
OG_01321	EYR68156.1	protein tolQ [Lysobacter capsici AZ78]
OG_01322	EYR67491.1	hypothetical protein AZ78_15205 [Lysobacter capsici AZ78]
OG_01323	EYR68234.1	hypothetical protein AZ78_11250 [Lysobacter capsici AZ78]
OG_01324	EYR65641.1	cystathionine gamma-synthase [Lysobacter capsici AZ78]
OG_01325	EYR66268.1	hypothetical protein AZ78_22120 [Lysobacter capsici AZ78]
OG_01326	EYR70153.1	D-alanyl-D-alanine carboxypeptidase [Lysobacter capsici AZ78]
OG_01327	EYR69595.1	stress-responsive transcriptional regulator [Lysobacter capsici AZ78]
OG_01328	EYR67109.1	hypothetical protein AZ78_17765 [Lysobacter capsici AZ78]
OG_01329	EYR66725.1	ribonuclease BN [Lysobacter capsici AZ78]
OG_01330	EYR65396.1	hypothetical protein AZ78_26470 [Lysobacter capsici AZ78]
OG_01331	EYR67104.1	RNA-binding protein [Lysobacter capsici AZ78]
OG_01332	EYR65511.1	membrane protein [Lysobacter capsici AZ78]
OG_01333	EYR70016.1	SpoVR like family protein [Lysobacter capsici AZ78]
OG_01334	EYR70052.1	hemin storage protein [Lysobacter capsici AZ78]
OG_01335	EYR67050.1	amino acid:proton symporter [Lysobacter capsici AZ78]
OG_01336	EYR68437.1	pantothenate kinase [Lysobacter capsici AZ78]
OG_01337	EYR66335.1	glutathione synthetase [Lysobacter capsici AZ78]
OG_01338	EYR70086.1	hypothetical protein AZ78_01690 [Lysobacter capsici AZ78]
OG_01339	EYR67988.1	hypothetical protein AZ78_13350 [Lysobacter capsici AZ78]
OG_01340	EYR70068.1	hypothetical protein AZ78_02890 [Lysobacter capsici AZ78]
OG_01341	EYR68172.1	DNA-binding protein [Lysobacter capsici AZ78]
OG_01342	EYR68477.1	tRNA (guanine-N(7)-)-methyltransferase [Lysobacter capsici AZ78]
OG_01343	EYR66711.1	membrane protein [Lysobacter capsici AZ78]
OG_01344	EYR69521.1	beta-mannosidase [Lysobacter capsici AZ78]
OG_01345	EYR65577.1	hypothetical protein AZ78_25510 [Lysobacter capsici AZ78]
OG_01346	EYR67438.1	hypothetical protein AZ78_16060 [Lysobacter capsici AZ78]
OG_01347	EYR66247.1	ATPase [Lysobacter capsici AZ78]
OG_01348	EYR65475.1	hypothetical protein AZ78_26140 [Lysobacter capsici AZ78]
OG_01349	EYR67064.1	hypothetical protein AZ78_17535 [Lysobacter capsici AZ78]
OG_01350	EYR65672.1	hypothetical protein AZ78_25055 [Lysobacter capsici AZ78]
OG_01351	EYR68040.1	delta-aminolevulinic acid dehydratase [Lysobacter capsici AZ78]
OG_01352	EYR65803.1	hypothetical protein AZ78_24600 [Lysobacter capsici AZ78]
OG_01353	EYR66101.1	MarR family transcriptional regulator [Lysobacter capsici AZ78]
OG_01354	EYR66149.1	ammonia channel protein [Lysobacter capsici AZ78]
OG_01355	EYR68527.1	S-adenosylmethionine synthetase [Lysobacter capsici AZ78]
OG_01356	EYR65894.1	7,8-dihydro-8-oxoguanine-triphosphatase [Lysobacter capsici AZ78]
OG_01357	EYR67304.1	trigger factor [Lysobacter capsici AZ78]
OG_01358	EYR65470.1	hypothetical protein AZ78_26110 [Lysobacter capsici AZ78]
OG_01359	EYR70454.1	hypothetical protein AZ78_01445 [Lysobacter capsici AZ78]
OG_01360	EYR66056.1	methicillin resistance protein [Lysobacter capsici AZ78]
OG_01361	EYR68021.1	chromosome partitioning protein ParB [Lysobacter capsici AZ78]
OG_01362	EYR66495.1	peptidase S1 [Lysobacter capsici AZ78]
OG_01363	EYR65912.1	hypothetical protein AZ78_23675 [Lysobacter capsici AZ78]
OG_01364	EYR65429.1	hypothetical protein AZ78_26295 [Lysobacter capsici AZ78]
OG_01365	EYR65636.1	hemolysin D [Lysobacter capsici AZ78]
OG_01366	EYR67844.1	alkane 1-monooxygenase [Lysobacter capsici AZ78]
OG_01367	EYR66475.1	phosphoglyceromutase [Lysobacter capsici AZ78]
OG_01368	EYR69338.1	30S ribosomal protein S6 [Lysobacter capsici AZ78]
OG_01369	EYR67626.1	membrane protein [Lysobacter capsici AZ78]
OG_01370	EYR69624.1	cell division protein DedD [Lysobacter capsici AZ78]
OG_01371	EYR65946.1	RNA-binding protein [Lysobacter capsici AZ78]
OG_01372	EYR68107.1	prolyl 4-hydroxylase [Lysobacter capsici AZ78]
OG_01373	EYR69118.1	dephospho-CoA kinase [Lysobacter capsici AZ78]
OG_01374	EYR67444.1	prephenate dehydratase [Lysobacter capsici AZ78]
OG_01375	EYR67713.1	hypothetical protein AZ78_13915 [Lysobacter capsici AZ78]
OG_01376	EYR66685.1	50S ribosomal protein L15 [Lysobacter capsici AZ78]
OG_01377	EYR66522.1	acetolactate synthase [Lysobacter capsici AZ78]
OG_01378	EYR69911.1	hypothetical protein AZ78_01760 [Lysobacter capsici AZ78]
OG_01379	EYR69165.1	general secretion pathway protein L [Lysobacter capsici AZ78]
OG_01380	EYR67275.1	membrane protein [Lysobacter capsici AZ78]
OG_01381	EYR66035.1	exodeoxyribonuclease VII small subunit [Lysobacter capsici AZ78]
OG_01382	EYR70008.1	3-ketoacyl-ACP reductase [Lysobacter capsici AZ78]
OG_01383	EYR68029.1	hypothetical protein AZ78_12535 [Lysobacter capsici AZ78]
OG_01384	EYR69759.1	organic solvent ABC transporter [Lysobacter capsici AZ78]
OG_01385	EYR69399.1	hypothetical protein AZ78_05760 [Lysobacter capsici AZ78]
OG_01386	EYR65601.1	Epoxyqueuosine reductase [Lysobacter capsici AZ78]
OG_01387	EYR69732.1	fructose-1 6-bisphosphatase [Lysobacter capsici AZ78]
OG_01388	EYR69619.1	hypothetical protein AZ78_03935 [Lysobacter capsici AZ78]
OG_01389	EYR68460.1	N-acetyltransferase GCN5 [Lysobacter capsici AZ78]
OG_01390	EYR68393.1	NAD-dependent deacetylase [Lysobacter capsici AZ78]
OG_01391	EYR66427.1	dihydroneopterin aldolase [Lysobacter capsici AZ78]
OG_01392	EYR68442.1	hypothetical protein AZ78_10315 [Lysobacter capsici AZ78]

OG_01393	LC55x_5123	LC55x_5123; conserved hypothetical protein
OG_01394	LC55x_607	bphK; glutathione S-transferase
OG_01395	LC55x_4123	LC55x_4123; conserved hypothetical protein
OG_01396	LC55x_3493	LC55x_3493; conserved hypothetical protein
OG_01397	LC55x_4773	LC55x_4773; putative D-serine deaminase (D-serine dehydratase) pr
OG_01398	LC55x_4067	ahpD; alkyl hydroperoxide reductase AhpD
OG_01399	LC55x_5592	LC55x_5592; conserved hypothetical protein
OG_01400	LC55x_4291	nusB; transcription antitermination factor NusB
OG_01401	LC55x_2422	LC55x_2422; conjugal transfer family protein
OG_01402	LC55x_565	LC55x_565; glutathione-dependent formaldehyde-activating enzyme
OG_01403	LC55x_5557	LC55x_5557; bacterial regulatory , arsR family protein
OG_01404	LC55x_253	LC55x_253; hypothetical protein
OG_01405	LC55x_1853	LC55x_1853; hypothetical protein
OG_01406	LC55x_793	LC55x_793; conserved hypothetical protein
OG_01407	LC55x_1234	LC55x_1234; CBS domain
OG_01408	LC55x_536	LC55x_536; binding-protein-dependent transport system inner memb
OG_01409	LC55x_3865	LC55x_3865; conserved hypothetical protein
OG_01410	LC55x_2945	LC55x_2945; YCII-related domain protein
OG_01411	LC55x_4538	LC55x_4538; glutathione S-transferase, C-terminal domain protein
OG_01412	LC55x_1561	rfbA; glucose-1-phosphate thymidylyltransferase
OG_01413	LC55x_481	ctaD; cytochrome c oxidase, subunit I
OG_01414	LC55x_3064	pbpC; penicillin-binding protein 1C
OG_01415	LC55x_708	tapA; temperature acclimation protein A
OG_01416	LC55x_761	yajQ; nucleotide binding protein
OG_01417	LC55x_1364	cysG; cysG
OG_01418	LC55x_1408	LC55x_1408; patatin-like phospholipase family protein
OG_01419	LC55x_4421	yegS; lipid kinase YegS
OG_01420	LC55x_929	leuC; 3-isopropylmalate dehydratase large subunit
OG_01421	LC55x_5612	LC55x_5612; ferric reductase like transmembrane component family
OG_01422	LC55x_4727	LC55x_4727; lysE type translocator family protein
OG_01423	LC55x_3307	argE; acetylornithine deacetylase
OG_01424	LC55x_1989	mutS; DNA mismatch repair protein MutS
OG_01425	LC55x_443	LC55x_443; conserved hypothetical protein
OG_01426	LC55x_1831	maf; septum formation protein Maf
OG_01427	LC55x_4726	LC55x_4726; conserved hypothetical protein
OG_01428	LC55x_196	LC55x_196; RDD family protein
OG_01429	LC55x_4250	nadE; NAD+ synthetase
OG_01430	LC55x_2038	LC55x_2038; hypothetical protein
OG_01431	LC55x_4046	LC55x_4046; conserved hypothetical protein
OG_01432	LC55x_706	LC55x_706; peptidase M23 family protein
OG_01433	LC55x_2286	LC55x_2286; phosphoenolpyruvate phosphomutase family protein
OG_01434	LC55x_2515	LC55x_2515; conserved hypothetical protein
OG_01435	LC55x_3273	LC55x_3273; trp repressor family protein
OG_01436	LC55x_3698	kdsA; 3-deoxy-8-phosphooctulonate synthase
OG_01437	LC55x_3323	LC55x_3323; type I GTP cyclohydrolase folE2 family protein
OG_01438	LC55x_3874	LC55x_3874; bacterial regulatory helix-turn-helix , lysR family protein
OG_01439	LC55x_3994	LC55x_3994; ahpC/TSA family protein
OG_01440	LC55x_4687	LC55x_4687; conserved hypothetical protein
OG_01441	LC55x_4252	sucD; succinyl-CoA synthetase, alpha subunit
OG_01442	LC55x_4719	LC55x_4719; xdhC and CoxI family protein
OG_01443	LC55x_1847	LC55x_1847; DNA polymerase III, &alpha subunit
OG_01444	LC55x_2138	LC55x_2138; acyltransferase family protein
OG_01445	LC55x_2884	LC55x_2884; hypothetical protein
OG_01446	LC55x_2878	LC55x_2878; conserved hypothetical protein
OG_01447	LC55x_5596	LC55x_5596; conserved hypothetical protein
OG_01448	LC55x_1984	katG; catalase/peroxidase HPI
OG_01449	LC55x_442	LC55x_442; putative beta-lactamase induction signal transducer Amp
OG_01450	LC55x_3606	LC55x_3606; phosphatase 2C family protein
OG_01451	LC55x_1841	LC55x_1841; polyprenyl synthetase family protein
OG_01452	LC55x_1136	LC55x_1136; hypothetical protein
OG_01453	LC55x_2800	kynU; kynureninase
OG_01454	LC55x_3947	rpsC; ribosomal protein S3
OG_01455	LC55x_2574	LC55x_2574; mgtC family protein
OG_01456	LC55x_2415	ccmC; heme exporter CcmC family protein
OG_01457	LC55x_3908	LC55x_3908; aminotransferase class I and II family protein
OG_01458	LC55x_2943	LC55x_2943; periplasmic binding and sugar binding domain of LacI fa
OG_01459	LC55x_2319	LC55x_2319; TPR repeat family protein
OG_01460	LC55x_2885	LC55x_2885; alpha-2-macroglobulin N-terminal region family protein
OG_01461	LC55x_3784	LC55x_3784; ATP-dependent RNA helicase, specific for 23S rRNA
OG_01462	LC55x_2868	LC55x_2868; acetyltransferase family protein
OG_01463	LC55x_446	tyrS; tyrosine--tRNA ligase
OG_01464	LC55x_2437	LC55x_2437; marR family protein
OG_01465	LC55x_497	dnaG; DNA primase
OG_01466	LC55x_548	fghA; S-formylglutathione hydrolase
OG_01467	LC55x_2099	ccmA; heme ABC exporter, ATP-binding protein CcmA
OG_01468	LC55x_366	LC55x_366; RNA pseudouridylate synthase family protein
OG_01469	LC55x_4459	LC55x_4459; entericidin EcnA/B family protein
OG_01470	LC55x_735	LC55x_735; conserved hypothetical protein
OG_01471	LC55x_434	dut; dut
OG_01472	LC55x_5106	LC55x_5106; sulfate ABC transporter, sulfate-binding family protein
OG_01473	LC55x_972	LC55x_972; conserved hypothetical protein

OG_01393	EYR68033.1	hypothetical protein AZ78_12555 [Lysobacter capsici AZ78]
OG_01394	EYR69998.1	Gst [Lysobacter capsici AZ78]
OG_01395	EYR66357.1	membrane protein [Lysobacter capsici AZ78]
OG_01396	EYR66624.1	hypothetical protein AZ78_20130 [Lysobacter capsici AZ78]
OG_01397	EYR66075.1	hypothetical protein AZ78_22725 [Lysobacter capsici AZ78]
OG_01398	EYR65381.1	alkyl hydroperoxide reductase [Lysobacter capsici AZ78]
OG_01399	EYR65422.1	hypothetical protein AZ78_26395 [Lysobacter capsici AZ78]
OG_01400	EYR69145.1	transcription antitermination protein NusB [Lysobacter capsici AZ78]
OG_01401	EYR69576.1	hypothetical protein AZ78_03620 [Lysobacter capsici AZ78]
OG_01402	EYR67922.1	aldehyde-activating protein [Lysobacter capsici AZ78]
OG_01403	EYR68985.1	ArsR family transcriptional regulator [Lysobacter capsici AZ78]
OG_01404	EYR66983.1	hypothetical protein AZ78_17875 [Lysobacter capsici AZ78]
OG_01405	EYR66045.1	hypothetical protein AZ78_23080 [Lysobacter capsici AZ78]
OG_01406	EYR67598.1	hypothetical protein AZ78_14570 [Lysobacter capsici AZ78]
OG_01407	EYR65383.1	histidine kinase [Lysobacter capsici AZ78]
OG_01408	EYR67896.1	hypothetical protein AZ78_12880 [Lysobacter capsici AZ78]
OG_01409	EYR65542.1	hypothetical protein AZ78_25680 [Lysobacter capsici AZ78]
OG_01410	EYR66215.1	YciI enzymatic domain-containing protein [Lysobacter capsici AZ78]
OG_01411	EYR68386.1	glutathione S-transferase [Lysobacter capsici AZ78]
OG_01412	EYR70281.1	glucose-1-phosphate thymidylyltransferase [Lysobacter capsici AZ78
OG_01413	EYR66459.1	cytochrome oxidase subunit I [Lysobacter capsici AZ78]
OG_01414	EYR67846.1	penicillin-binding protein 1C [Lysobacter capsici AZ78]
OG_01415	EYR69941.1	cold-shock protein [Lysobacter capsici AZ78]
OG_01416	EYR67628.1	nucleotide-binding protein [Lysobacter capsici AZ78]
OG_01417	EYR67201.1	sirohydrochlorin ferrochelatase [Lysobacter capsici AZ78]
OG_01418	EYR67161.1	hypothetical protein AZ78_17210 [Lysobacter capsici AZ78]
OG_01419	EYR69246.1	lipid kinase [Lysobacter capsici AZ78]
OG_01420	EYR66831.1	isopropylmalate isomerase [Lysobacter capsici AZ78]
OG_01421	EYR66252.1	membrane protein [Lysobacter capsici AZ78]
OG_01422	EYR66117.1	lysine transporter LysE [Lysobacter capsici AZ78]
OG_01423	EYR70064.1	acetylornithine deacetylase [Lysobacter capsici AZ78]
OG_01424	EYR66764.1	DNA mismatch repair protein MutS [Lysobacter capsici AZ78]
OG_01425	EYR65528.1	hypothetical protein AZ78_25880 [Lysobacter capsici AZ78]
OG_01426	EYR66024.1	hypothetical protein AZ78_22975 [Lysobacter capsici AZ78]
OG_01427	EYR66118.1	hypothetical protein AZ78_22940 [Lysobacter capsici AZ78]
OG_01428	EYR66136.1	hypothetical protein AZ78_22470 [Lysobacter capsici AZ78]
OG_01429	EYR69105.1	NAD synthetase [Lysobacter capsici AZ78]
OG_01430	EYR65610.1	hypothetical protein AZ78_25400 [Lysobacter capsici AZ78]
OG_01431	EYR65825.1	hypothetical protein AZ78_24245 [Lysobacter capsici AZ78]
OG_01432	EYR69942.1	metalloendopeptidase [Lysobacter capsici AZ78]
OG_01433	EYR69671.1	PEP phosphonomutase-like enzyme [Lysobacter capsici AZ78]
OG_01434	EYR69503.1	lysogenization protein HflD [Lysobacter capsici AZ78]
OG_01435	EYR70044.1	TrpR [Lysobacter capsici AZ78]
OG_01436	EYR65921.1	2-dehydro-3-deoxyphosphooctonate aldolase [Lysobacter capsici AZ
OG_01437	EYR70075.1	GTP cyclohydrolase [Lysobacter capsici AZ78]
OG_01438	EYR65708.1	LysR family transcriptional regulator [Lysobacter capsici AZ78]
OG_01439	EYR66724.1	thioredoxin [Lysobacter capsici AZ78]
OG_01440	EYR70185.1	hypothetical protein AZ78_00280 [Lysobacter capsici AZ78]
OG_01441	EYR69107.1	succinyl-CoA synthetase subunit alpha [Lysobacter capsici AZ78]
OG_01442	EYR65681.1	hypothetical protein AZ78_25100 [Lysobacter capsici AZ78]
OG_01443	EYR66041.1	DNA polymerase III subunit alpha [Lysobacter capsici AZ78]
OG_01444	EYR66371.1	membrane protein [Lysobacter capsici AZ78]
OG_01445	EYR69443.1	hypothetical protein AZ78_05980 [Lysobacter capsici AZ78]
OG_01446	EYR69437.1	peroxiredoxin [Lysobacter capsici AZ78]
OG_01447	EYR65427.1	hypothetical protein AZ78_26420 [Lysobacter capsici AZ78]
OG_01448	EYR66765.1	catalase/hydroperoxidase HPI(I) [Lysobacter capsici AZ78]
OG_01449	EYR65527.1	beta-lactamase [Lysobacter capsici AZ78]
OG_01450	EYR67550.1	hypothetical protein AZ78_15500 [Lysobacter capsici AZ78]
OG_01451	EYR66034.1	farnesyl-diphosphate synthase [Lysobacter capsici AZ78]
OG_01452	EYR68633.1	hypothetical protein AZ78_10865 [Lysobacter capsici AZ78]
OG_01453	EYR69364.1	kynureninase [Lysobacter capsici AZ78]
OG_01454	EYR66698.1	30S ribosomal protein S3 [Lysobacter capsici AZ78]
OG_01455	EYR68319.1	hypothetical protein AZ78_11680 [Lysobacter capsici AZ78]
OG_01456	EYR69582.1	heme ABC transporter permease [Lysobacter capsici AZ78]
OG_01457	EYR66665.1	aminotransferase [Lysobacter capsici AZ78]
OG_01458	EYR66217.1	LacI family transcription regulator [Lysobacter capsici AZ78]
OG_01459	EYR69645.1	hypothetical protein AZ78_04105 [Lysobacter capsici AZ78]
OG_01460	EYR69444.1	hypothetical protein AZ78_05985 [Lysobacter capsici AZ78]
OG_01461	EYR67091.1	RNA helicase [Lysobacter capsici AZ78]
OG_01462	EYR69426.1	hypothetical protein AZ78_05895 [Lysobacter capsici AZ78]
OG_01463	EYR65531.1	tyrosyl-tRNA synthetase [Lysobacter capsici AZ78]
OG_01464	EYR69563.1	MarR family transcriptional regulator [Lysobacter capsici AZ78]
OG_01465	EYR66446.1	DNA primase [Lysobacter capsici AZ78]
OG_01466	EYR67909.1	S-formylglutathione hydrolase [Lysobacter capsici AZ78]
OG_01467	EYR66406.1	heme ABC transporter ATPase [Lysobacter capsici AZ78]
OG_01468	EYR68139.1	pseudouridylate synthase [Lysobacter capsici AZ78]
OG_01469	EYR68440.1	entericidin [Lysobacter capsici AZ78]
OG_01470	EYR67649.1	isochorismatase [Lysobacter capsici AZ78]
OG_01471	EYR65520.1	deoxyuridine 5'-triphosphate nucleotidohydrolase [Lysobacter capsic
OG_01472	EYR68045.1	sulfate transporter subunit [Lysobacter capsici AZ78]
OG_01473	EYR66793.1	membrane protein [Lysobacter capsici AZ78]

OG_01474	LC55x_5228	LC55x_5228; peptidase inhibitor I78 family protein	OG_01474	EYR68889.1	hypothetical protein AZ78_07475 [Lysobacter capsici AZ78]
OG_01475	LC55x_5255	ubiA; 4-hydroxybenzoate polyprenyl transferase	OG_01475	EYR68905.1	4-hydroxybenzoate polyprenyltransferase [Lysobacter capsici AZ78]
OG_01476	LC55x_3829	LC55x_3829; membrane dipeptidase family protein	OG_01476	EYR65750.1	hypothetical protein AZ78_24725 [Lysobacter capsici AZ78]
OG_01477	LC55x_3330	LC55x_3330; kamA family protein	OG_01477	EYR70081.1	lysine 2,3-aminomutase [Lysobacter capsici AZ78]
OG_01478	LC55x_2183	LC55x_2183; biopolymer transport ExbD/TolR family protein	OG_01478	EYR67400.1	biopolymer transporter [Lysobacter capsici AZ78]
OG_01479	LC55x_5126	LC55x_5126; RDD family protein	OG_01479	EYR68030.1	membrane protein [Lysobacter capsici AZ78]
OG_01480	LC55x_1664	infC; translation initiation factor IF-3	OG_01480	EYR70337.1	translation initiation factor IF-3 [Lysobacter capsici AZ78]
OG_01481	LC55x_2210	LC55x_2210; bacterial regulatory helix-turn-helix, AraC family protein	OG_01481	EYR67412.1	AraC family transcriptional regulator [Lysobacter capsici AZ78]
OG_01482	LC55x_1874	glnD; protein-P-II uridylyltransferase	OG_01482	EYR66067.1	PII uridylyl-transferase [Lysobacter capsici AZ78]
OG_01483	LC55x_440	LC55x_440; acetyltransferase family protein	OG_01483	EYR65525.1	hypothetical protein AZ78_25865 [Lysobacter capsici AZ78]
OG_01484	LC55x_4091	msrB; methionine-R-sulfoxide reductase	OG_01484	EYR65492.1	hypothetical protein AZ78_25900 [Lysobacter capsici AZ78]
OG_01485	LC55x_2368	LC55x_2368; kinase/pyrophosphorylase family protein	OG_01485	EYR69612.1	PEP synthetase regulatory protein [Lysobacter capsici AZ78]
OG_01486	LC55x_722	acrA; acrA Membrane Fusion Protein	OG_01486	EYR67657.1	hemolysin D [Lysobacter capsici AZ78]
OG_01487	LC55x_3599	LC55x_3599; transcriptional regulatory , C terminal family protein	OG_01487	EYR67542.1	transcriptional regulator [Lysobacter capsici AZ78]
OG_01488	LC55x_2439	LC55x_2439; NAD dependent epimerase/dehydratase family protein	OG_01488	EYR69561.1	NAD dependent epimerase/dehydratase [Lysobacter capsici AZ78]
OG_01489	LC55x_329	LC55x_329; ABC-2 transporter family protein	OG_01489	EYR68175.1	hypothetical protein AZ78_11815 [Lysobacter capsici AZ78]
OG_01490	LC55x_2516	trmU; tRNA (5-methylaminomethyl-2-thiouridylate)-methyltransferase	OG_01490	EYR69502.1	thiouridylase [Lysobacter capsici AZ78]
OG_01491	LC55x_5662	LC55x_5662; aminoglycoside/hydroxyurea antibiotic resistance kinas	OG_01491	EYR66296.1	streptomycin 3''-kinase [Lysobacter capsici AZ78]
OG_01492	LC55x_624	LC55x_624; hypothetical protein	OG_01492	EYR70122.1	hypothetical protein AZ78_02350 [Lysobacter capsici AZ78]
OG_01493	LC55x_1199	alkJ; alcohol dehydrogenase [acceptor]	OG_01493	EYR65785.1	choline dehydrogenase [Lysobacter capsici AZ78]
OG_01494	LC55x_3194	LC55x_3194; acetyl-CoA C-acetyltransferase family protein	OG_01494	EYR66905.1	acetyl-CoA acetyltransferase [Lysobacter capsici AZ78]
OG_01495	LC55x_441	xth; exodeoxyribonuclease III	OG_01495	EYR65526.1	catabolite repression control protein [Lysobacter capsici AZ78]
OG_01496	LC55x_504	folB; dihydroneopterin aldolase	OG_01496	EYR66440.1	dihydroneopterin triphosphate 2'-epimerase [Lysobacter capsici AZ78
OG_01497	LC55x_4264	LC55x_4264; conserved hypothetical protein	OG_01497	EYR69122.1	hypothetical protein AZ78_06460 [Lysobacter capsici AZ78]
OG_01498	LC55x_2539	LC55x_2539; crcB-like family protein	OG_01498	EYR69485.1	camphor resistance protein CrcB [Lysobacter capsici AZ78]
OG_01499	LC55x_1723	queA; tRNA ribosyltransferase-isomerase	OG_01499	EYR70373.1	S-adenosylmethionine tRNA ribosyltransferase [Lysobacter capsici A
OG_01500	LC55x_5128	LC55x_5128; bacterial transferase hexapeptide family protein	OG_01500	EYR68028.1	carbonic anhydrase [Lysobacter capsici AZ78]
OG_01501	LC55x_428	LC55x_428; sporulation related domain protein	OG_01501	EYR65514.1	hypothetical protein AZ78_25810 [Lysobacter capsici AZ78]
OG_01502	LC55x_2842	LC55x_2842; conserved hypothetical protein	OG_01502	EYR69403.1	hypothetical protein AZ78_05780 [Lysobacter capsici AZ78]
OG_01503	LC55x_3483	LC55x_3483; moaE family protein	OG_01503	EYR66615.1	molybdopterin-converting factor chain 2 [Lysobacter capsici AZ78]
OG_01504	LC55x_445	LC55x_445; peptidase M23 family protein	OG_01504	EYR65530.1	membrane protein [Lysobacter capsici AZ78]
OG_01505	LC55x_3685	LC55x_3685; putative transmembrane protein	OG_01505	EYR65933.1	membrane protein [Lysobacter capsici AZ78]
OG_01506	LC55x_1993	LC55x_1993; GDSL-like Lipase/Acylhydrolase family protein	OG_01506	EYR66762.1	hypothetical protein AZ78_19310 [Lysobacter capsici AZ78]
OG_01507	LC55x_1180	LC55x_1180; tonB-dependent hemoglobin/transferrin/lactoferrin recep	OG_01507	EYR65802.1	sugar transporter [Lysobacter capsici AZ78]
OG_01508	LC55x_483	LC55x_483; cytochrome c oxidase assembly CtaG/Cox11 family prot	OG_01508	EYR66457.1	cysteine ABC transporter substrate-binding protein [Lysobacter capsi
OG_01509	LC55x_1071	LC55x_1071; S1/P1 Nuclease family protein	OG_01509	EYR67718.1	endonuclease [Lysobacter capsici AZ78]
OG_01510	LC55x_3504	LC55x_3504; conserved hypothetical protein	OG_01510	EYR66632.1	alkylhydroperoxidase [Lysobacter capsici AZ78]
OG_01511	LC55x_720	LC55x_720; DSBA-like thioredoxin domain protein	OG_01511	EYR69934.1	DSBA oxidoreductase [Lysobacter capsici AZ78]
OG_01512	LC55x_1688	LC55x_1688; late embryogenesis abundant family protein	OG_01512	EYR70352.1	hypothetical protein AZ78_01380 [Lysobacter capsici AZ78]
OG_01513	LC55x_5084	LC55x_5084; short chain dehydrogenase family protein	OG_01513	EYR68062.1	3-ketoacyl-ACP reductase [Lysobacter capsici AZ78]
OG_01514	LC55x_2556	potI; potI	OG_01514	EYR65535.1	putrescine/spermidine ABC transporter permease [Lysobacter capsic
OG_01515	LC55x_5031	rho; transcription termination factor Rho	OG_01515	EYR66309.1	transcription termination factor Rho [Lysobacter capsici AZ78]
OG_01516	LC55x_4274	LC55x_4274; UDP	OG_01516	EYR69132.1	UDP-N-acetylmuramoylalanyl-D-glutamyl-2, 6-diaminopimelate--D-al
OG_01517	LC55x_3536	LC55x_3536; histidine phosphatase super family protein	OG_01517	EYR67488.1	hypothetical protein AZ78_15190 [Lysobacter capsici AZ78]
OG_01518	LC55x_3894	LC55x_3894; suppressor of fused family protein	OG_01518	EYR65726.1	hypothetical protein AZ78_24840 [Lysobacter capsici AZ78]
OG_01519	LC55x_3082	LC55x_3082; phage Tail Collar domain protein	OG_01519	EYR67858.1	microcystin dependent protein [Lysobacter capsici AZ78]
OG_01521	LC55x_4161	LC55x_4161; conserved hypothetical protein	OG_01521	EYR66320.1	hypothetical protein AZ78_21450 [Lysobacter capsici AZ78]
OG_01522	LC55x_5222	LC55x_5222; conserved hypothetical protein	OG_01522	EYR68884.1	hypothetical protein AZ78_07445 [Lysobacter capsici AZ78]
OG_01523	LC55x_4401	LC55x_4401; ubiquinone biosynthesis COQ7 family protein	OG_01523	EYR69230.1	2-nonaprenyl-3-methyl-6-methoxy-1,4-benzoquinol hydroxylase [Lys
OG_01524	LC55x_5195	LC55x_5195; hypothetical protein	OG_01524	EYR68992.1	hypothetical protein AZ78_07325 [Lysobacter capsici AZ78]
OG_01525	LC55x_1940	rpmA; ribosomal protein L27	OG_01525	EYR65967.1	50S ribosomal protein L27 [Lysobacter capsici AZ78]
OG_01526	LC55x_1843	LC55x_1843; conserved hypothetical protein	OG_01526	EYR66037.1	hypothetical protein AZ78_23040 [Lysobacter capsici AZ78]
OG_01527	LC55x_2320	LC55x_2320; glycosyl transferase 4 family protein	OG_01527	EYR69644.1	lipopolysaccharide biosynthesis protein [Lysobacter capsici AZ78]
OG_01528	LC55x_3914	typA; GTP-binding protein TypA/BipA	OG_01528	EYR66667.1	GTP-binding protein [Lysobacter capsici AZ78]
OG_01529	LC55x_2125	LC55x_2125; glycerophosphoryl diester phosphodiesterase family pr	OG_01529	EYR66381.1	hypothetical protein AZ78_21205 [Lysobacter capsici AZ78]
OG_01530	LC55x_5710	LC55x_5710; hypothetical protein	OG_01530	EYR65848.1	hypothetical protein AZ78_23925 [Lysobacter capsici AZ78]
OG_01531	LC55x_4093	LC55x_4093; surA N-terminal domain protein	OG_01531	EYR65494.1	molecular chaperone SurA [Lysobacter capsici AZ78]
OG_01532	LC55x_4600	LC55x_4600; ribonucleoside-diphosphate reductase, adenosylcobala	OG_01532	EYR68356.1	NrdJa [Lysobacter capsici AZ78]
OG_01533	LC55x_5169	mutM; formamidopyrimidine-DNA glycosylase	OG_01533	EYR68000.1	5-hydroxymethyluracil DNA glycosylase [Lysobacter capsici AZ78]
OG_01534	LC55x_2235	LC55x_2235; nucleotide sugar dehydrogenase family protein	OG_01534	EYR67428.1	Vi polysaccharide biosynthesis protein vipA/tviB [Lysobacter capsici /
OG_01535	LC55x_1574	LC55x_1574; conserved hypothetical protein	OG_01535	EYR70293.1	magnesium transporter [Lysobacter capsici AZ78]
OG_01536	LC55x_5666	LC55x_5666; major Facilitator Superfamily protein	OG_01536	EYR65889.1	hypothetical protein AZ78_24135 [Lysobacter capsici AZ78]
OG_01537	LC55x_3490	LC55x_3490; glycosyltransferase 9 family protein	OG_01537	EYR66622.1	ADP-heptose--LPS heptosyltransferase [Lysobacter capsici AZ78]
OG_01538	LC55x_1137	LC55x_1137; ATP-dependant zinc protease family protein	OG_01538	EYR68511.1	ribosomal protein S6 modification protein [Lysobacter capsici AZ78]
OG_01539	LC55x_778	atpH; ATP synthase F1, delta subunit	OG_01539	EYR67612.1	F0F1 ATP synthase subunit delta [Lysobacter capsici AZ78]
OG_01540	LC55x_731	LC55x_731; SPFH domain / Band 7 family protein	OG_01540	EYR67650.1	hypothetical protein AZ78_14885 [Lysobacter capsici AZ78]
OG_01541	LC55x_4139	LC55x_4139; cheW-like domain protein	OG_01541	EYR66342.1	chemotaxis protein CheW [Lysobacter capsici AZ78]
OG_01542	LC55x_3607	LC55x_3607; von Willebrand factor type A domain protein	OG_01542	EYR67551.1	hypothetical protein AZ78_15505 [Lysobacter capsici AZ78]
OG_01543	LC55x_1837	LC55x_1837; modulator of DNA gyrase family protein	OG_01543	EYR66029.1	peptidase C69 [Lysobacter capsici AZ78]
OG_01544	LC55x_4772	nagA; alpha-N-acetylgalactosaminidase	OG_01544	EYR66076.1	glycosyl hydrolase family 109 [Lysobacter capsici AZ78]
OG_01545	LC55x_3277	hisS; histidine--tRNA ligase	OG_01545	EYR70047.1	histidyl-tRNA synthase [Lysobacter capsici AZ78]
OG_01546	LC55x_3552	LC55x_3552; RNA polymerase sigma factor, sigma-70 family protein	OG_01546	EYR67504.1	hypothetical protein AZ78_15270 [Lysobacter capsici AZ78]
OG_01547	LC55x_1383	LC55x_1383; competence ComEA helix-hairpin-helix repeat region d	OG_01547	EYR67181.1	DNA transport competence protein [Lysobacter capsici AZ78]
OG_01548	LC55x_566	LC55x_566; bacterial regulatory, gntR family protein	OG_01548	EYR70030.1	GntR family transcriptional regulator [Lysobacter capsici AZ78]
OG_01549	LC55x_4916	LC55x_4916; UDP-N-acetylglucosamine 2-epimerase family protein	OG_01549	EYR68700.1	CDP-glycerol:glycerophosphate glycerophosphotransferase [Lysobac
OG_01550	LC55x_3848	LC55x_3848; sporulation related domain protein	OG_01550	EYR65730.1	sporulation protein [Lysobacter capsici AZ78]
OG_01551	LC55x_116	LC55x_116; hlyD secretion family protein	OG_01551	EYR69882.1	hypothetical protein AZ78_04975 [Lysobacter capsici AZ78]
OG_01552	LC55x_3671	miaA; tRNA dimethylallyltransferase	OG_01552	EYR65945.1	tRNA delta(2)-isopentenylpyrophosphate transferase [Lysobacter cap
OG_01553	LC55x_1217	LC55x_1217; conserved hypothetical protein	OG_01553	EYR65763.1	hypothetical protein AZ78_24385 [Lysobacter capsici AZ78]
OG_01554	LC55x_759	LC55x_759; HAD hydrolase, IA, variant 3 family protein	OG_01554	EYR67630.1	hydrolase [Lysobacter capsici AZ78]
OG_01555	LC55x_991	LC55x_991; impB/mucB/samB family protein	OG_01555	EYR67782.1	DNA-directed DNA polymerase [Lysobacter capsici AZ78]

OG_01556	LC55x_352	LC55x_352; beta-lactamase family protein	OG_01556	EYR68128.1	beta-lactamase [Lysobacter capsici AZ78]
OG_01557	LC55x_3403	LC55x_3403; conserved hypothetical protein	OG_01557	EYR66517.1	thymidylate synthase [Lysobacter capsici AZ78]
OG_01558	LC55x_1324	yhbG; lptB	OG_01558	EYR69912.1	lipopolysaccharide ABC transporter ATP-binding protein [Lysobacter
OG_01559	LC55x_2321	LC55x_2321; short chain dehydrogenase family protein	OG_01559	EYR69643.1	multidrug MFS transporter [Lysobacter capsici AZ78]
OG_01560	LC55x_3208	LC55x_3208; enoyl-CoA hydratase/isomerase family protein	OG_01560	EYR67980.1	enoyl-CoA hydratase [Lysobacter capsici AZ78]
OG_01561	LC55x_2127	SIRT4; NAD-dependent ADP-ribosyltransferase sirtuin-4	OG_01561	EYR66380.1	NAD-dependent deacetylase [Lysobacter capsici AZ78]
OG_01562	LC55x_4931	LC55x_4931; chitin binding domain protein	OG_01562	EYR68687.1	cellulose-binding protein [Lysobacter capsici AZ78]
OG_01563	LC55x_2330	LC55x_2330; enoyl-CoA hydratase/isomerase family protein	OG_01563	EYR69638.1	3-hydroxyacyl-CoA dehydrogenase [Lysobacter capsici AZ78]
OG_01564	LC55x_2344	LC55x_2344; alpha/beta hydrolase family protein	OG_01564	EYR69626.1	PhoP [Lysobacter capsici AZ78]
OG_01565	LC55x_5111	cysW; sulfate ABC transporter, permease protein CysW	OG_01565	EYR68042.1	sulfate ABC transporter permease [Lysobacter capsici AZ78]
OG_01566	LC55x_3740	LC55x_3740; conserved hypothetical protein	OG_01566	EYR67053.1	membrane protein [Lysobacter capsici AZ78]
OG_01567	LC55x_5658	LC55x_5658; AFG1-like ATPase family protein	OG_01567	EYR66293.1	ATPase [Lysobacter capsici AZ78]
OG_01568	LC55x_2369	LC55x_2369; conserved hypothetical protein	OG_01568	EYR69611.1	hypothetical protein AZ78_03875 [Lysobacter capsici AZ78]
OG_01569	LC55x_4115	LC55x_4115; efflux transporter, RND family, MFP subunit	OG_01569	EYR66364.1	hypothetical protein AZ78_21675 [Lysobacter capsici AZ78]
OG_01570	LC55x_1767	LC55x_1767; DEAD-box-containing ATP-dependent RNA helicase fa	OG_01570	EYR65347.1	RNA helicase [Lysobacter capsici AZ78]
OG_01571	LC55x_2798	Acmsd; 2-amino-3-carboxymuconate-6-semialdehyde decarboxylase	OG_01571	EYR69362.1	2-amino-3-carboxymuconate-6-semialdehyde decarboxylase [Lysoba
OG_01572	LC55x_4532	LC55x_4532; fatty acid hydroxylase superfamily protein	OG_01572	EYR68392.1	sterol desaturase [Lysobacter capsici AZ78]
OG_01573	LC55x_1912	phoU; phosphate transport system regulatory protein PhoU	OG_01573	EYR65984.1	PhoU family transcriptional regulator [Lysobacter capsici AZ78]
OG_01574	LC55x_5081	LC55x_5081; conserved hypothetical protein	OG_01574	EYR68063.1	hypothetical protein AZ78_12760 [Lysobacter capsici AZ78]
OG_01575	LC55x_5008	LC55x_5008; sulfatase family protein	OG_01575	EYR67222.1	membrane protein [Lysobacter capsici AZ78]
OG_01576	LC55x_3690	LC55x_3690; smr domain protein	OG_01576	EYR65929.1	Smr DNA repair family protein [Lysobacter capsici AZ78]
OG_01577	LC55x_2417	ccmA; heme ABC exporter, ATP-binding protein CcmA	OG_01577	EYR69580.1	cytochrome C biogenesis protein CcmA [Lysobacter capsici AZ78]
OG_01578	LC55x_1391	LC55x_1391; apbE family protein	OG_01578	EYR67177.1	hypothetical protein AZ78_17290 [Lysobacter capsici AZ78]
OG_01579	LC55x_1369	cysD; sulfate adenylyltransferase, small subunit	OG_01579	EYR67195.1	sulfate adenylyltransferase subunit 2 [Lysobacter capsici AZ78]
OG_01580	LC55x_5174	LC55x_5174; conserved hypothetical protein	OG_01580	EYR67996.1	pathogenicity protein [Lysobacter capsici AZ78]
OG_01581	LC55x_1242	LC55x_1242; conserved hypothetical protein	OG_01581	EYR65638.1	peptidase [Lysobacter capsici AZ78]
OG_01582	LC55x_3285	LC55x_3285; major Facilitator Superfamily protein	OG_01582	EYR70140.1	hypothetical protein AZ78_02745 [Lysobacter capsici AZ78]
OG_01583	LC55x_4984	LC55x_4984; bacterial regulatory, gntR family protein	OG_01583	EYR67244.1	GntR family transcriptional regulator [Lysobacter capsici AZ78]
OG_01584	LC55x_4755	LC55x_4755; conserved hypothetical protein	OG_01584	EYR66091.1	hypothetical protein AZ78_22805 [Lysobacter capsici AZ78]
OG_01585	LC55x_3727	LC55x_3727; thioesterase superfamily protein	OG_01585	EYR65898.1	acyl-CoA hydrolase [Lysobacter capsici AZ78]
OG_01586	LC55x_2720	bmrA; multidrug resistance ABC transporter ATP-binding/permease p	OG_01586	EYR69295.1	hypothetical protein AZ78_05215 [Lysobacter capsici AZ78]
OG_01587	LC55x_741	LC55x_741; hypothetical protein	OG_01587	EYR67643.1	hypothetical protein AZ78_14830 [Lysobacter capsici AZ78]
OG_01588	LC55x_971	LC55x_971; ATP-dependent protease La domain protein	OG_01588	EYR66794.1	ATP-dependent protease [Lysobacter capsici AZ78]
OG_01589	LC55x_337	trmL; tRNA (cytidine(34)-2'-O)-methyltransferase	OG_01589	EYR68115.1	rRNA methyltransferase [Lysobacter capsici AZ78]
OG_01590	LC55x_3957	rpsG; ribosomal protein S7	OG_01590	EYR65490.1	30S ribosomal protein S7 [Lysobacter capsici AZ78]
OG_01591	LC55x_2363	LC55x_2363; conserved hypothetical protein	OG_01591	EYR69837.1	hypothetical protein AZ78_03900 [Lysobacter capsici AZ78]
OG_01592	LC55x_3991	LC55x_3991; conserved hypothetical protein	OG_01592	EYR66721.1	membrane protein [Lysobacter capsici AZ78]
OG_01593	LC55x_2408	LC55x_2408; tetratricopeptide repeat family protein	OG_01593	EYR69588.1	cytochrome C biogenesis protein [Lysobacter capsici AZ78]
OG_01594	LC55x_1223	LC55x_1223; hypothetical protein	OG_01594	EYR65758.1	hypothetical protein AZ78_24360 [Lysobacter capsici AZ78]
OG_01595	LC55x_105	LC55x_105; conserved hypothetical protein	OG_01595	EYR69880.1	hypothetical protein AZ78_04920 [Lysobacter capsici AZ78]
OG_01596	LC55x_5146	rpmG; ribosomal protein L33	OG_01596	EYR68015.1	50S ribosomal protein L33 [Lysobacter capsici AZ78]
OG_01597	LC55x_3001	rimP; ribosome maturation domain protein	OG_01597	EYR67800.1	ribosome maturation protein RimP [Lysobacter capsici AZ78]
OG_01598	LC55x_937	ilvD; dihydroxy-acid dehydratase	OG_01598	EYR66824.1	dihydroxy-acid dehydratase [Lysobacter capsici AZ78]
OG_01600	LC55x_3316	dksA; RNA polymerase-binding protein DksA	OG_01600	EYR70070.1	molecular chaperone DnaK [Lysobacter capsici AZ78]
OG_01601	LC55x_2620	LC55x_2620; trbL/VirB6 plasmid conjugal transfer family protein	OG_01601	EYR68276.1	hypothetical protein AZ78_11460 [Lysobacter capsici AZ78]
OG_01602	LC55x_835	LC55x_835; putative transmembrane protein	OG_01602	EYR67578.1	membrane protein [Lysobacter capsici AZ78]
OG_01603	LC55x_1889	LC55x_1889; polysaccharide deacetylase family protein	OG_01603	EYR66000.1	polysaccharide deacetylase [Lysobacter capsici AZ78]
OG_01604	LC55x_4265	lpxC; UDP-3-O-[3-hydroxymyristoyl] N-acetylglucosamine deacetylas	OG_01604	EYR69123.1	UDP-3-O-(3-hydroxymyristoyl) glucosamine N-acyltransferase [Lysob
OG_01605	LC55x_1100	LC55x_1100; efflux transporter, RND family, MFP subunit	OG_01605	EYR68465.1	RND transporter [Lysobacter capsici AZ78]
OG_01606	LC55x_4765	LC55x_4765; FAD binding domain protein	OG_01606	EYR66082.1	D-amino acid oxidase [Lysobacter capsici AZ78]
OG_01607	LC55x_4399	rplM; ribosomal protein L13	OG_01607	EYR69229.1	50S ribosomal protein L13 [Lysobacter capsici AZ78]
OG_01608	LC55x_3702	LC55x_3702; conserved hypothetical protein	OG_01608	EYR65917.1	hypothetical protein AZ78_23700 [Lysobacter capsici AZ78]
OG_01609	LC55x_3431	era; GTP-binding protein Era	OG_01609	EYR66500.1	GTP-binding protein Era [Lysobacter capsici AZ78]
OG_01610	LC55x_1257	LC55x_1257; cytochrome c family protein	OG_01610	EYR65646.1	cytochrome CBB3 [Lysobacter capsici AZ78]
OG_01611	LC55x_1397	bnzC; benzene 1,2-dioxygenase system ferredoxin subunit	OG_01611	EYR67171.1	benzene 1,2-dioxygenase ferredoxin [Lysobacter capsici AZ78]
OG_01612	LC55x_1201	LC55x_1201; calcineurin-like phosphoesterase family protein	OG_01612	EYR65783.1	hypothetical protein AZ78_24500 [Lysobacter capsici AZ78]
OG_01613	LC55x_4638	rlpA; rare lipoA family protein	OG_01613	EYR70154.1	hypothetical protein AZ78_00015 [Lysobacter capsici AZ78]
OG_01614	LC55x_3488	LC55x_3488; metallo-beta-lactamase superfamily protein	OG_01614	EYR66620.1	beta-lactamase [Lysobacter capsici AZ78]
OG_01615	LC55x_3145	LC55x_3145; hypothetical protein	OG_01615	EYR66953.1	hypothetical protein AZ78_18445 [Lysobacter capsici AZ78]
OG_01616	LC55x_1968	LC55x_1968; efflux transporter, outer membrane factor (OMF) lipo, N	OG_01616	EYR66777.1	multidrug RND transporter [Lysobacter capsici AZ78]
OG_01617	LC55x_4149	LC55x_4149; hypothetical protein	OG_01617	EYR66332.1	hypothetical protein AZ78_21515 [Lysobacter capsici AZ78]
OG_01618	LC55x_423	speA; arginine decarboxylase	OG_01618	EYR65614.1	arginine decarboxylase [Lysobacter capsici AZ78]
OG_01619	LC55x_2965	LC55x_2965; hypothetical protein	OG_01619	EYR66198.1	hypothetical protein AZ78_21755 [Lysobacter capsici AZ78]
OG_01620	LC55x_3884	LC55x_3884; recF/RecN/SMC N terminal domain protein	OG_01620	EYR65717.1	hypothetical protein AZ78_24795 [Lysobacter capsici AZ78]
OG_01621	LC55x_3990	LC55x_3990; hypothetical protein	OG_01621	EYR66720.1	hypothetical protein AZ78_19820 [Lysobacter capsici AZ78]
OG_01622	LC55x_1340	LC55x_1340; TPR repeat family protein	OG_01622	EYR70087.1	hypothetical protein AZ78_01695 [Lysobacter capsici AZ78]
OG_01623	LC55x_4185	LC55x_4185; radical SAM superfamily protein	OG_01623	EYR68162.1	7-carboxy-7-deazaguanine synthase [Lysobacter capsici AZ78]
OG_01624	LC55x_3442	holB; DNA polymerase III, delta' subunit	OG_01624	EYR66589.1	DNA polymerase III subunit delta' [Lysobacter capsici AZ78]
OG_01625	LC55x_4776	LC55x_4776; hypothetical protein	OG_01625	EYR68851.1	hypothetical protein AZ78_09465 [Lysobacter capsici AZ78]
OG_01626	LC55x_5730	LC55x_5730; DNA binding , excisionase family domain protein	OG_01626	EYR69696.1	antifreeze protein, type I [Lysobacter capsici AZ78]
OG_01627	LC55x_1712	LC55x_1712; metallo-beta-lactamase superfamily protein	OG_01627	EYR70368.1	metallo-beta-lactamase [Lysobacter capsici AZ78]
OG_01628	LC55x_1116	LC55x_1116; conserved hypothetical protein	OG_01628	EYR68479.1	acid phosphatase [Lysobacter capsici AZ78]
OG_01629	LC55x_1617	purE; phosphoribosylaminoimidazole carboxylase, catalytic subunit	OG_01629	EYR70322.1	N5-carboxyaminoimidazole ribonucleotide mutase [Lysobacter capsic
OG_01630	LC55x_3675	rrmJ; 23S rRNA 2'-O-ribose U2552 methyltransferase	OG_01630	EYR65942.1	23S rRNA methyltransferase [Lysobacter capsici AZ78]
OG_01631	LC55x_2168	LC55x_2168; aminomethyltransferase folate-binding domain protein	OG_01631	EYR67387.1	aminomethyl transferase [Lysobacter capsici AZ78]
OG_01632	LC55x_482	LC55x_482; hypothetical protein	OG_01632	EYR66458.1	hypothetical protein AZ78_20960 [Lysobacter capsici AZ78]
OG_01633	LC55x_3861	kbl; 2-amino-3-ketobutyrate coenzyme A ligase	OG_01633	EYR65547.1	2-amino-3-ketobutyrate CoA ligase [Lysobacter capsici AZ78]
OG_01634	LC55x_1200	LC55x_1200; major Facilitator Superfamily protein	OG_01634	EYR65784.1	translocase [Lysobacter capsici AZ78]
OG_01635	LC55x_157	LC55x_157; periplasmic binding and sugar binding domain of LacI fa	OG_01635	EYR69800.1	LacI family transcription regulator [Lysobacter capsici AZ78]
OG_01636	LC55x_502	rpsU; ribosomal protein S21	OG_01636	EYR66442.1	30S ribosomal protein S21 [Lysobacter capsici AZ78]
OG_01637	LC55x_3426	LC55x_3426; conserved hypothetical protein	OG_01637	EYR66503.1	hypothetical protein AZ78_20320 [Lysobacter capsici AZ78]

OG_01638	LC55x_2579	LC55x_2579; T5orf172 domain protein
OG_01639	LC55x_3518	LC55x_3518; glycosyl transferases group 1 family protein
OG_01640	LC55x_2538	LC55x_2538; sigma-54 interaction domain protein
OG_01641	LC55x_1102	LC55x_1102; MMPL family protein
OG_01642	LC55x_1160	LC55x_1160; acyltransferase family protein
OG_01643	LC55x_1248	LC55x_1248; homoserine dehydrogenase family protein
OG_01644	LC55x_4348	LC55x_4348; H-NS histone family protein
OG_01645	LC55x_1095	fbab; fructose-bisphosphate aldolase
OG_01646	LC55x_2256	serC; phosphoserine transaminase
OG_01647	LC55x_1858	rseP; RIP metalloprotease RseP
OG_01648	LC55x_881	LC55x_881; thioredoxin family protein
OG_01649	LC55x_2779	LC55x_2779; putative transmembrane protein
OG_01650	LC55x_1366	cysH; phosphoadenosine phosphosulfate reductase
OG_01651	LC55x_3457	LC55x_3457; dolichyl-phosphate-mannose-mannosyltransferase fam
OG_01652	LC55x_150	LC55x_150; bacterial regulatory, gntR family protein
OG_01653	LC55x_4467	LC55x_4467; EDD, DegV family domain protein
OG_01654	LC55x_4086	LC55x_4086; ubiquinone biosynthesis hydroxylase, UbiH/UbiF/VisC/(
OG_01655	LC55x_756	LC55x_756; conserved hypothetical protein
OG_01656	LC55x_3466	recR; recombination protein RecR
OG_01657	LC55x_1553	LC55x_1553; tonB dependent receptor family protein
OG_01658	LC55x_4742	LC55x_4742; autotransporter-associated beta strand repeat family pr
OG_01659	LC55x_173	ggt; gamma-glutamyltransferase
OG_01660	LC55x_4286	LC55x_4286; conserved hypothetical protein
OG_01661	LC55x_336	LC55x_336; major Facilitator Superfamily protein
OG_01662	LC55x_766	LC55x_766; ompW family protein
OG_01663	LC55x_3090	LC55x_3090; zinc-binding dehydrogenase family protein
OG_01664	LC55x_3846	purF; amidophosphoribosyltransferase
OG_01665	LC55x_4210	aspS; aspartate--tRNA ligase
OG_01666	LC55x_2132	LC55x_2132; peptidase M3 family protein
OG_01667	LC55x_3237	LC55x_3237; conserved hypothetical protein
OG_01668	LC55x_277	LC55x_277; hypothetical protein
OG_01669	LC55x_2036	dapA; dihydrodipicolinate synthase
OG_01670	LC55x_2463	LC55x_2463; sensory box protein
OG_01671	LC55x_4306	ccmA; heme ABC exporter, ATP-binding protein CcmA
OG_01672	LC55x_1400	sufD; feS assembly protein SufD
OG_01673	LC55x_1416	LC55x_1416; toxin-antitoxin system toxin component, PIN family
OG_01674	LC55x_3354	LC55x_3354; heavy metal sensor kinase family protein
OG_01675	LC55x_3319	pyrC; dihydroorotase
OG_01676	LC55x_5614	LC55x_5614; conserved hypothetical protein
OG_01677	LC55x_4430	LC55x_4430; eamA-like transporter family protein
OG_01678	LC55x_4042	pntB; NAD(P) transhydrogenase subunit beta
OG_01679	LC55x_1917	pstS; phosphate ABC transporter, phosphate-binding protein PstS
OG_01680	LC55x_424	LC55x_424; YCII-related domain protein
OG_01681	LC55x_1067	LC55x_1067; ABC transporter family protein
OG_01682	LC55x_4036	LC55x_4036; bacterial regulatory helix-turn-helix, AraC family protein
OG_01683	LC55x_1023	LC55x_1023; cytochrome c family protein
OG_01684	LC55x_3631	LC55x_3631; histidine kinase-, DNA gyrase B-, and HSP90-like ATPa
OG_01685	LC55x_5223	LC55x_5223; hsp70 family protein
OG_01686	LC55x_1162	LC55x_1162; aspartate racemase family protein
OG_01687	LC55x_3576	LC55x_3576; hhH-GPD superbase excision DNA repair family proteir
OG_01688	LC55x_1915	pstC; phosphate ABC transporter, permease protein PstC
OG_01689	LC55x_5188	hemH; ferrochelatase
OG_01690	LC55x_2187	LC55x_2187; low molecular weight phosphotyrosine phosphatase far
OG_01691	LC55x_3308	argG; argininosuccinate synthase
OG_01692	LC55x_3975	LC55x_3975; ribosomal protein L25, Ctc-form
OG_01693	LC55x_4671	LC55x_4671; glycosyl hydrolases 25 family protein
OG_01694	LC55x_4113	LC55x_4113; ftsX-like permease family protein
OG_01695	LC55x_2355	LC55x_2355; bacterial regulatory, tetR family protein
OG_01696	LC55x_2501	rpfG; response regulator
OG_01697	LC55x_4928	LC55x_4928; RDD family protein
OG_01698	LC55x_2366	ppsA; phosphoenolpyruvate synthase
OG_01699	LC55x_2743	copA; copper resistance protein A
OG_01700	LC55x_3891	LC55x_3891; conserved hypothetical protein
OG_01701	LC55x_356	LC55x_356; sodium symporter family protein
OG_01702	LC55x_4096	apaG; protein ApaG
OG_01703	LC55x_1144	metF; 5,10-methylenetetrahydrofolate reductase
OG_01704	LC55x_3382	murB; UDP-N-acetylenolpyruvoylglucosamine reductase
OG_01705	LC55x_4419	trpE; anthranilate synthase component I
OG_01706	LC55x_2629	cyoA; ubiquinol oxidase, subunit II
OG_01707	LC55x_4741	LC55x_4741; pepSY-associated TM helix family protein
OG_01708	LC55x_5105	LC55x_5105; phosphate-selective porin O and P family protein
OG_01709	LC55x_969	adk; adenylate kinase
OG_01710	LC55x_3049	greB; transcription elongation factor GreB
OG_01711	LC55x_3548	tesB; acyl-CoA thioesterase II
OG_01712	LC55x_3521	LC55x_3521; acetyltransferase family protein
OG_01713	LC55x_3243	LC55x_3243; short chain dehydrogenase family protein
OG_01714	LC55x_4068	ahpC; alkyl hydroperoxide reductase subunit C
OG_01715	LC55x_2324	LC55x_2324; FAD binding domain protein
OG_01716	LC55x_2376	LC55x_2376; radical SAM superfamily protein
OG_01717	LC55x_3976	prs; ribose-phosphate pyrophosphokinase
OG_01718	LC55x_1570	LC55x_1570; conserved hypothetical protein

OG_01638	EYR68315.1	hypothetical protein AZ78_11660 [Lysobacter capsici AZ78]
OG_01639	EYR66642.1	glycosyl transferase [Lysobacter capsici AZ78]
OG_01640	EYR69486.1	ATPase AAA [Lysobacter capsici AZ78]
OG_01641	EYR68466.1	multidrug transporter [Lysobacter capsici AZ78]
OG_01642	EYR68528.1	acyltransferase [Lysobacter capsici AZ78]
OG_01643	EYR65642.1	homoserine dehydrogenase [Lysobacter capsici AZ78]
OG_01644	EYR69188.1	DNA-binding protein [Lysobacter capsici AZ78]
OG_01645	EYR68462.1	fructose-bisphosphate aldolase [Lysobacter capsici AZ78]
OG_01646	EYR67443.1	MFS transporter [Lysobacter capsici AZ78]
OG_01647	EYR66050.1	zinc metalloprotease [Lysobacter capsici AZ78]
OG_01648	EYR66876.1	hypothetical protein AZ78_19030 [Lysobacter capsici AZ78]
OG_01649	EYR69343.1	membrane protein [Lysobacter capsici AZ78]
OG_01650	EYR67199.1	phosphoadenosine phosphosulfate reductase [Lysobacter capsici AZ
OG_01651	EYR66602.1	membrane protein [Lysobacter capsici AZ78]
OG_01652	EYR69794.1	GntR family transcriptional regulator [Lysobacter capsici AZ78]
OG_01653	EYR68593.1	hypothetical protein AZ78_10270 [Lysobacter capsici AZ78]
OG_01654	EYR65438.1	2-octaprenyl-6-methoxyphenyl hydroxylase [Lysobacter capsici AZ78
OG_01655	EYR67633.1	hypothetical protein AZ78_14755 [Lysobacter capsici AZ78]
OG_01656	EYR66609.1	recombinase RecR [Lysobacter capsici AZ78]
OG_01657	EYR70275.1	TonB-dependent receptor [Lysobacter capsici AZ78]
OG_01658	EYR66103.1	hypothetical protein AZ78_22865 [Lysobacter capsici AZ78]
OG_01659	EYR65413.1	gamma-glutamyltransferase [Lysobacter capsici AZ78]
OG_01660	EYR69141.1	hypothetical protein AZ78_06565 [Lysobacter capsici AZ78]
OG_01661	EYR68114.1	MFS transporter [Lysobacter capsici AZ78]
OG_01662	EYR67623.1	membrane protein [Lysobacter capsici AZ78]
OG_01663	EYR67863.1	alcohol dehydrogenase [Lysobacter capsici AZ78]
OG_01664	EYR65732.1	amidophosphoribosyltransferase [Lysobacter capsici AZ78]
OG_01665	EYR69075.1	aspartyl-tRNA synthetase [Lysobacter capsici AZ78]
OG_01666	EYR66376.1	dipeptidyl carboxypeptidase II [Lysobacter capsici AZ78]
OG_01667	EYR67954.1	hypothetical protein AZ78_13180 [Lysobacter capsici AZ78]
OG_01668	EYR67002.1	hypothetical protein AZ78_17975 [Lysobacter capsici AZ78]
OG_01669	EYR65592.1	dihydrodipicolinate synthase [Lysobacter capsici AZ78]
OG_01670	EYR69547.1	histidine kinase [Lysobacter capsici AZ78]
OG_01671	EYR69155.1	ABC transporter ATP-binding protein [Lysobacter capsici AZ78]
OG_01672	EYR67168.1	ABC transporter permease [Lysobacter capsici AZ78]
OG_01673	EYR67155.1	hypothetical protein AZ78_17180 [Lysobacter capsici AZ78]
OG_01674	EYR66541.1	sensor histidine kinase [Lysobacter capsici AZ78]
OG_01675	EYR70073.1	dihydroorotase [Lysobacter capsici AZ78]
OG_01676	EYR66254.1	hypothetical protein AZ78_22045 [Lysobacter capsici AZ78]
OG_01677	EYR65471.1	hypothetical protein AZ78_26115 [Lysobacter capsici AZ78]
OG_01678	EYR65821.1	NADP transhydrogenase subunit alpha [Lysobacter capsici AZ78]
OG_01679	EYR65980.1	phosphate-binding protein [Lysobacter capsici AZ78]
OG_01680	EYR65613.1	hypothetical protein AZ78_25245 [Lysobacter capsici AZ78]
OG_01681	EYR67722.1	molybdate ABC transporter ATP-binding protein [Lysobacter capsici /
OG_01682	EYR65814.1	hypothetical protein AZ78_24190 [Lysobacter capsici AZ78]
OG_01683	EYR67757.1	hypothetical protein AZ78_14165 [Lysobacter capsici AZ78]
OG_01684	EYR68661.1	histidine kinase [Lysobacter capsici AZ78]
OG_01685	EYR68885.1	heat shock protein Hsp70 [Lysobacter capsici AZ78]
OG_01686	EYR68530.1	aspartate racemase [Lysobacter capsici AZ78]
OG_01687	EYR67524.1	DNA-3-methyladenine glycosylase [Lysobacter capsici AZ78]
OG_01688	EYR65981.1	phosphate ABC transporter permease [Lysobacter capsici AZ78]
OG_01689	EYR68860.1	ferrochelatase [Lysobacter capsici AZ78]
OG_01690	EYR67404.1	phosphotyrosine protein phosphatase [Lysobacter capsici AZ78]
OG_01691	EYR70065.1	argininosuccinate synthase [Lysobacter capsici AZ78]
OG_01692	EYR66708.1	50S ribosomal protein L25 [Lysobacter capsici AZ78]
OG_01693	EYR70170.1	lysozyme [Lysobacter capsici AZ78]
OG_01694	EYR66366.1	hypothetical protein AZ78_21685 [Lysobacter capsici AZ78]
OG_01695	EYR69620.1	TetR family transcriptional regulator [Lysobacter capsici AZ78]
OG_01696	EYR69517.1	divalent ion tolerance protein CutA [Lysobacter capsici AZ78]
OG_01697	EYR68689.1	transporter [Lysobacter capsici AZ78]
OG_01698	EYR69613.1	phosphoenolpyruvate synthase [Lysobacter capsici AZ78]
OG_01699	EYR69313.1	hypothetical protein AZ78_05320 [Lysobacter capsici AZ78]
OG_01700	EYR65723.1	hypothetical protein AZ78_24825 [Lysobacter capsici AZ78]
OG_01701	EYR68130.1	sodium:solute symporter [Lysobacter capsici AZ78]
OG_01702	EYR65497.1	magnesium transporter ApaG [Lysobacter capsici AZ78]
OG_01703	EYR68517.1	5,10-methylenetetrahydrofolate reductase [Lysobacter capsici AZ78]
OG_01704	EYR66530.1	UDP-N-acetylenolpyruvoylglucosamine reductase [Lysobacter capsic
OG_01705	EYR69245.1	anthranilate synthase subunit I [Lysobacter capsici AZ78]
OG_01706	EYR68267.1	cytochrome O ubiquinol oxidase [Lysobacter capsici AZ78]
OG_01707	EYR66104.1	peptidase M4 [Lysobacter capsici AZ78]
OG_01708	EYR68046.1	porin [Lysobacter capsici AZ78]
OG_01709	EYR66796.1	adenylate kinase [Lysobacter capsici AZ78]
OG_01710	EYR67835.1	transcription elongation factor GreB [Lysobacter capsici AZ78]
OG_01711	EYR67501.1	acyl-CoA thioesterase [Lysobacter capsici AZ78]
OG_01712	EYR67471.1	hypothetical protein AZ78_15100 [Lysobacter capsici AZ78]
OG_01713	EYR67938.1	3-hydroxy-2-methylbutyryl-CoA dehydrogenase [Lysobacter capsici /
OG_01714	EYR65380.1	alkyl hydroperoxide reductase [Lysobacter capsici AZ78]
OG_01715	EYR69641.1	FAD-dependent oxidoreductase [Lysobacter capsici AZ78]
OG_01716	EYR69608.1	coproporphyrinogen III oxidase [Lysobacter capsici AZ78]
OG_01717	EYR66709.1	ribose-phosphate pyrophosphokinase [Lysobacter capsici AZ78]
OG_01718	EYR70289.1	hypothetical protein AZ78_00940 [Lysobacter capsici AZ78]

OG_01719	LC55x_2154	LC55x_2154; putative phospholipase A1	OG_01719	EYR67374.1	phospholipase [Lysobacter capsici AZ78]
OG_01720	LC55x_2178	LC55x_2178; putative membrane protein	OG_01720	EYR67395.1	membrane protein [Lysobacter capsici AZ78]
OG_01721	LC55x_3522	LC55x_3522; inner membrane CreD family protein	OG_01721	EYR67472.1	hypothetical protein AZ78_15105 [Lysobacter capsici AZ78]
OG_01722	LC55x_751	LC55x_751; GDSL-like Lipase/Acylhydrolase family protein	OG_01722	EYR67637.1	arylesterase [Lysobacter capsici AZ78]
OG_01723	LC55x_104	LC55x_104; bacterial regulatory helix-turn-helix , lysR family protein	OG_01723	EYR69765.1	LysR family transcriptional regulator [Lysobacter capsici AZ78]
OG_01724	LC55x_3983	prfA; peptide chain release factor 1	OG_01724	EYR66713.1	peptide chain release factor 1 [Lysobacter capsici AZ78]
OG_01725	LC55x_3089	soxR; redox-sensitive transcriptional activator SoxR	OG_01725	EYR67862.1	MerR family transcriptional regulator [Lysobacter capsici AZ78]
OG_01726	LC55x_2243	LC55x_2243; bacterial regulatory, tetR family protein	OG_01726	EYR67433.1	transcriptional regulator [Lysobacter capsici AZ78]
OG_01727	LC55x_3700	pyrG; CTP synthase	OG_01727	EYR65919.1	CTP synthetase [Lysobacter capsici AZ78]
OG_01728	LC55x_1414	LC55x_1414; eamA-like transporter family protein	OG_01728	EYR67157.1	membrane protein [Lysobacter capsici AZ78]
OG_01729	LC55x_1700	LC55x_1700; acetyltransferase family protein	OG_01729	EYR70360.1	GCN5 family N-acetyltransferase [Lysobacter capsici AZ78]
OG_01730	LC55x_2158	LC55x_2158; glycine zipper 2TM domain protein	OG_01730	EYR67378.1	hypothetical protein AZ78_15640 [Lysobacter capsici AZ78]
OG_01731	LC55x_4799	LC55x_4799; conserved hypothetical protein	OG_01731	EYR68778.1	nucleoprotein/polynucleotide-associated enzyme [Lysobacter capsici
OG_01732	LC55x_1083	LC55x_1083; ribonucleoside-diphosphate reductase, alpha subunit	OG_01732	EYR67709.1	ribonucleoside-diphosphate reductase [Lysobacter capsici AZ78]
OG_01733	LC55x_4464	LC55x_4464; biotin--[acetyl-CoA-carboxylase] ligase	OG_01733	EYR68436.1	biotin-protein ligase [Lysobacter capsici AZ78]
OG_01734	LC55x_933	LC55x_933; branched-chain amino acid aminotransferase	OG_01734	EYR66828.1	hypothetical protein AZ78_18790 [Lysobacter capsici AZ78]
OG_01735	LC55x_1320	LC55x_1320; phosphoenolpyruvate-dependent sugar phosphotransfe	OG_01735	EYR69915.1	PTS fructose transporter subunit IIA [Lysobacter capsici AZ78]
OG_01736	LC55x_3707	parE; DNA topoisomerase IV, B subunit	OG_01736	EYR65913.1	DNA topoisomerase IV subunit B [Lysobacter capsici AZ78]
OG_01737	LC55x_2310	LC55x_2310; acetyltransferase family protein	OG_01737	EYR69654.1	phosphinothricin N-acetyltransferase [Lysobacter capsici AZ78]
OG_01738	LC55x_3025	LC55x_3025; type I phosphodiesterase / nucleotide pyrophosphatase	OG_01738	EYR67821.1	membrane protein [Lysobacter capsici AZ78]
OG_01739	LC55x_2161	LC55x_2161; tatD related DNase family protein	OG_01739	EYR67380.1	DNAase [Lysobacter capsici AZ78]
OG_01740	LC55x_4944	LC55x_4944; conserved hypothetical protein	OG_01740	EYR68677.1	hypothetical protein AZ78_08635 [Lysobacter capsici AZ78]
OG_01741	LC55x_1240	LC55x_1240; hypothetical protein	OG_01741	EYR65653.1	hypothetical protein AZ78_25135 [Lysobacter capsici AZ78]
OG_01742	LC55x_2023	LC55x_2023; response regulator	OG_01742	EYR66738.1	Fis family transcriptional regulator [Lysobacter capsici AZ78]
OG_01743	LC55x_3802	NAMPT; nicotinamide phosphoribosyltransferase	OG_01743	EYR67108.1	nicotinate phosphoribosyltransferase [Lysobacter capsici AZ78]
OG_01744	LC55x_2433	dnaE; DNA polymerase III, alpha subunit	OG_01744	EYR69567.1	DNA polymerase [Lysobacter capsici AZ78]
OG_01745	LC55x_1919	pstS; phosphate ABC transporter, phosphate-binding protein PstS	OG_01745	EYR65979.1	phosphate-binding protein [Lysobacter capsici AZ78]
OG_01746	LC55x_286	LC55x_286; fecR family protein	OG_01746	EYR67013.1	hypothetical protein AZ78_18030 [Lysobacter capsici AZ78]
OG_01747	LC55x_3811	LC55x_3811; recF/RecN/SMC N terminal domain protein	OG_01747	EYR67115.1	ATP-binding protein [Lysobacter capsici AZ78]
OG_01748	LC55x_949	LC55x_949; transcriptional regulatory , C terminal family protein	OG_01748	EYR66813.1	hypothetical protein AZ78_18715 [Lysobacter capsici AZ78]
OG_01749	LC55x_3412	LC55x_3412; hypothetical protein	OG_01749	EYR66561.1	hypothetical protein AZ78_20385 [Lysobacter capsici AZ78]
OG_01750	LC55x_924	LC55x_924; major intrinsic family protein	OG_01750	EYR66835.1	glycerol transporter [Lysobacter capsici AZ78]
OG_01751	LC55x_582	LC55x_582; AMP nucleosidase, putative	OG_01751	EYR70017.1	AMP nucleosidase [Lysobacter capsici AZ78]
OG_01752	LC55x_4565	LC55x_4565; H+ antiporter-2 family protein	OG_01752	EYR68375.1	MFS transporter [Lysobacter capsici AZ78]
OG_01753	LC55x_895	groL; chaperonin GroL	OG_01753	EYR66862.1	molecular chaperone GroEL [Lysobacter capsici AZ78]
OG_01754	LC55x_4669	LC55x_4669; pfkB carbohydrate kinase family protein	OG_01754	EYR70169.1	sugar kinase [Lysobacter capsici AZ78]
OG_01755	LC55x_3303	proS; proline--tRNA ligase	OG_01755	EYR70060.1	prolyl-tRNA synthetase [Lysobacter capsici AZ78]
OG_01756	LC55x_205	glnA; glutamine synthetase, type I	OG_01756	EYR66145.1	glutamine synthetase [Lysobacter capsici AZ78]
OG_01757	LC55x_2239	LC55x_2239; tetratricopeptide repeat family protein	OG_01757	EYR67431.1	hypothetical protein AZ78_16010 [Lysobacter capsici AZ78]
OG_01758	LC55x_2236	LC55x_2236; conserved region in glutamate synthase family protein	OG_01758	EYR67429.1	glutamate synthase [Lysobacter capsici AZ78]
OG_01759	LC55x_432	coaBC; phosphopantothenoylcysteine decarboxylase / phosphopantc	OG_01759	EYR65518.1	hypothetical protein AZ78_25830 [Lysobacter capsici AZ78]
OG_01760	LC55x_148	nagA; N-acetylglucosamine-6-phosphate deacetylase	OG_01760	EYR69792.1	N-acetylglucosamine 6-phosphate deacetylase [Lysobacter capsici A
OG_01761	LC55x_2220	LC55x_2220; sodium/hydrogen exchanger family protein	OG_01761	EYR67416.1	sodium:proton antiporter [Lysobacter capsici AZ78]
OG_01762	LC55x_13	LC55x_13; biopolymer transport ExbD/TolR family protein	OG_01762	EYR69716.1	biopolymer transporter ExbD [Lysobacter capsici AZ78]
OG_01763	LC55x_1396	LC55x_1396; tonB-dependent siderophore receptor family protein	OG_01763	EYR67172.1	TonB-denpendent receptor [Lysobacter capsici AZ78]
OG_01764	LC55x_956	LC55x_956; F5/8 type C domain protein	OG_01764	EYR66808.1	hypothetical protein AZ78_18685 [Lysobacter capsici AZ78]
OG_01765	LC55x_2318	LC55x_2318; conserved hypothetical protein	OG_01765	EYR69646.1	membrane protein [Lysobacter capsici AZ78]
OG_01766	LC55x_598	LC55x_598; response regulator	OG_01766	EYR70005.1	transcriptional regulator [Lysobacter capsici AZ78]
OG_01767	LC55x_3948	rplV; ribosomal protein L22	OG_01767	EYR66699.1	50S ribosomal protein L22 [Lysobacter capsici AZ78]
OG_01768	LC55x_5724	LC55x_5724; alpha/beta hydrolase fold family protein	OG_01768	EYR69692.1	cysteine proteinase [Lysobacter capsici AZ78]
OG_01769	LC55x_4682	LC55x_4682; NAD dependent epimerase/dehydratase family protein	OG_01769	EYR70180.1	epimerase [Lysobacter capsici AZ78]
OG_01770	LC55x_2166	LC55x_2166; putative glutathione S-transferase	OG_01770	EYR67385.1	glutathione S-transferase [Lysobacter capsici AZ78]
OG_01771	LC55x_4203	LC55x_4203; alpha/beta hydrolase fold family protein	OG_01771	EYR68147.1	hypothetical protein AZ78_12060 [Lysobacter capsici AZ78]
OG_01772	LC55x_5510	LC55x_5510; metallo-beta-lactamase superfamily protein	OG_01772	EYR68951.1	lactamase [Lysobacter capsici AZ78]
OG_01773	LC55x_3901	dctA; dctA dicarboxylate transporter	OG_01773	EYR66659.1	C4-dicarboxylate ABC transporter [Lysobacter capsici AZ78]
OG_01774	LC55x_488	LC55x_488; cytochrome oxidase assembly family protein	OG_01774	EYR66452.1	cytochrome oxidase assembly protein [Lysobacter capsici AZ78]
OG_01775	LC55x_537	LC55x_537; VIT family protein	OG_01775	EYR67897.1	hypothetical protein AZ78_12885 [Lysobacter capsici AZ78]
OG_01776	LC55x_214	LC55x_214; periplasmic binding and sugar binding domain of LacI fa	OG_01776	EYR66157.1	LacI family transcriptional regulator [Lysobacter capsici AZ78]
OG_01777	LC55x_5688	LC55x_5688; flagellin N-methylase family protein	OG_01777	EYR65870.1	hypothetical protein AZ78_24040 [Lysobacter capsici AZ78]
OG_01778	LC55x_5717	LC55x_5717; conserved hypothetical protein	OG_01778	EYR69856.1	hypothetical protein AZ78_04395 [Lysobacter capsici AZ78]
OG_01779	LC55x_2823	LC55x_2823; homocysteine S-methyltransferase family protein	OG_01779	EYR69384.1	5-methyltetrahydrofolate--homocysteine methyltransferase [Lysobact
OG_01780	LC55x_997	LC55x_997; conserved hypothetical protein	OG_01780	EYR67777.1	hypothetical protein AZ78_14275 [Lysobacter capsici AZ78]
OG_01781	LC55x_4415	LC55x_4415; HAMP domain protein	OG_01781	EYR69242.1	ATPase [Lysobacter capsici AZ78]
OG_01782	LC55x_1193	gcvH; glycine cleavage system H protein	OG_01782	EYR65791.1	glycine cleavage system protein H [Lysobacter capsici AZ78]
OG_01783	LC55x_2094	LC55x_2094; glutathione S-transferase, C-terminal domain protein	OG_01783	EYR66411.1	glutathione S-transferase [Lysobacter capsici AZ78]
OG_01784	LC55x_1836	LC55x_1836; conserved hypothetical protein	OG_01784	EYR66028.1	hypothetical protein AZ78_22995 [Lysobacter capsici AZ78]
OG_01785	LC55x_3578	LC55x_3578; polyketide cyclase / dehydrase and lipid transport famil	OG_01785	EYR67526.1	polyketide cyclase [Lysobacter capsici AZ78]
OG_01786	LC55x_467	LC55x_467; rhomboid family protein	OG_01786	EYR66471.1	membrane protein [Lysobacter capsici AZ78]
OG_01787	LC55x_2295	LC55x_2295; cytochrome c-552 domain protein	OG_01787	EYR69664.1	cytochrome C biogenesis protein CcsA [Lysobacter capsici AZ78]
OG_01788	LC55x_935	ilvB; acetolactate synthase, large subunit, biosynthetic type	OG_01788	EYR66826.1	acetolactate synthase [Lysobacter capsici AZ78]
OG_01789	LC55x_3153	phaZ7; PHB depolymerase PhaZ7	OG_01789	EYR66945.1	hypothetical protein AZ78_18405 [Lysobacter capsici AZ78]
OG_01790	LC55x_3340	LC55x_3340; formylglycine-generating sulfatase enzyme family prote	OG_01790	EYR66549.1	hypothetical protein AZ78_20705 [Lysobacter capsici AZ78]
OG_01791	LC55x_4246	LC55x_4246; conserved hypothetical protein	OG_01791	EYR69101.1	hypothetical protein AZ78_06320 [Lysobacter capsici AZ78]
OG_01792	LC55x_321	secB; protein-export chaperone SecB	OG_01792	EYR68104.1	preprotein translocase subunit SecB [Lysobacter capsici AZ78]
OG_01793	LC55x_4261	LC55x_4261; mutator mutT family protein	OG_01793	EYR69119.1	mutator mutT protein [Lysobacter capsici AZ78]
OG_01794	LC55x_4105	LC55x_4105; repair family protein	OG_01794	EYR65506.1	hypothetical protein AZ78_25970 [Lysobacter capsici AZ78]
OG_01795	LC55x_885	LC55x_885; conserved hypothetical protein	OG_01795	EYR66872.1	membrane protein [Lysobacter capsici AZ78]
OG_01796	LC55x_152	pgl; 6-phosphogluconolactonase	OG_01796	EYR69796.1	6-phosphogluconolactonase [Lysobacter capsici AZ78]
OG_01797	LC55x_588	LC55x_588; methyltransferase domain protein	OG_01797	EYR70013.1	methyltransferase [Lysobacter capsici AZ78]
OG_01798	LC55x_84	LC55x_84; hypothetical protein	OG_01798	EYR69875.1	hypothetical protein AZ78_04825 [Lysobacter capsici AZ78]
OG_01799	LC55x_947	thiC; thiamine biosynthesis protein ThiC	OG_01799	EYR66815.1	phosphomethylpyrimidine synthase ThiC [Lysobacter capsici AZ78]

OG_01800	LC55x_691	LC55x_691; ABC-2 type transporter family protein
OG_01801	LC55x_4922	def; peptide deformylase
OG_01802	LC55x_2632	LC55x_2632; aminotransferase class-V family protein
OG_01803	LC55x_4311	LC55x_4311; conserved hypothetical protein
OG_01804	LC55x_4760	LC55x_4760; metallopeptidase M24 family protein
OG_01805	LC55x_1867	LC55x_1867; mitochondrial biogenesis AIM24 family protein
OG_01806	LC55x_2869	sucA; oxoglutarate dehydrogenase (succinyl-transferring), E1 compo
OG_01807	LC55x_517	LC55x_517; sulfite exporter TauE/SafE family protein
OG_01808	LC55x_4345	LC55x_4345; conserved hypothetical protein
OG_01809	LC55x_4513	LC55x_4513; alpha/beta hydrolase fold family protein
OG_01810	LC55x_3035	asd; aspartate-semialdehyde dehydrogenase
OG_01811	LC55x_3288	pgaB; poly-beta-1,6-N-acetyl-D-glucosamine N-deacetylase PgaB
OG_01812	LC55x_4081	LC55x_4081; EF hand family protein
OG_01813	LC55x_2420	LC55x_2420; conserved hypothetical protein
OG_01814	LC55x_2164	fadE7; ACYL-CoA DEHYDROGENASE FADE7
OG_01815	LC55x_3573	LC55x_3573; D-alanyl-D-alanine carboxypeptidase family protein
OG_01816	LC55x_5729	LC55x_5729; tonB family C-terminal domain protein
OG_01817	LC55x_5648	LC55x_5648; conserved hypothetical protein
OG_01818	LC55x_3311	cysS; cysteine--tRNA ligase
OG_01819	LC55x_1292	proC; pyrroline-5-carboxylate reductase
OG_01820	LC55x_8	LC55x_8; tetratricopeptide repeat family protein
OG_01821	LC55x_3215	LC55x_3215; diguanylate cyclase domain protein
OG_01822	LC55x_4705	hemC; porphobilinogen deaminase
OG_01823	LC55x_1361	LC55x_1361; mobA-like NTP transferase domain protein
OG_01824	LC55x_1063	tkt; transketolase
OG_01825	LC55x_1859	dxr; 1-deoxy-D-xylulose 5-phosphate reductoisomerase
OG_01826	LC55x_728	LC55x_728; hypothetical protein
OG_01827	LC55x_642	chr; chromate transporter, chromate ion transporter family protein
OG_01828	LC55x_2569	LC55x_2569; DEAD/DEAH box helicase family protein
OG_01829	LC55x_2162	LC55x_2162; putative transmembrane protein
OG_01830	LC55x_1140	LC55x_1140; HAMP domain protein
OG_01831	LC55x_47	LC55x_47; bacterial regulatory , Fis family protein
OG_01832	LC55x_5239	LC55x_5239; diguanylate cyclase domain protein
OG_01833	LC55x_822	LC55x_822; tfoX C-terminal domain protein
OG_01834	LC55x_4606	LC55x_4606; conserved hypothetical protein
OG_01835	LC55x_2185	lpxK; tetraacyldisaccharide 4'-kinase
OG_01836	LC55x_2322	galU; UTP-glucose-1-phosphate uridylyltransferase
OG_01837	LC55x_1039	pilO; pilO protein
OG_01838	LC55x_3071	LC55x_3071; flavo, HI0933 family protein
OG_01839	LC55x_4790	LC55x_4790; acyltransferase family protein
OG_01840	LC55x_1906	rnt; ribonuclease T
OG_01841	LC55x_4272	ftsW; cell division protein FtsW
OG_01842	LC55x_740	LC55x_740; RNA polymerase sigma factor, sigma-70 family protein
OG_01843	LC55x_2045	panD; aspartate 1-decarboxylase
OG_01844	LC55x_5708	LC55x_5708; aldo/keto reductase family protein
OG_01845	LC55x_1680	LC55x_1680; pepSY-associated TM helix family protein
OG_01846	LC55x_4317	LC55x_4317; type II secretion system (T2SS), M subtype b family pro
OG_01847	LC55x_4063	prmC; protein-(glutamine-N5) methyltransferase, release factor-speci
OG_01848	LC55x_5093	LC55x_5093; phosphopantetheine attachment site family protein
OG_01849	LC55x_5199	plsB; glycerol-3-phosphate O-acyltransferase
OG_01850	LC55x_1703	mutY; A/G-specific adenine glycosylase
OG_01851	LC55x_4319	LC55x_4319; type II secretion system (T2SS), K family protein
OG_01852	LC55x_2068	LC55x_2068; hypothetical protein
OG_01853	LC55x_5125	LC55x_5125; conserved hypothetical protein
OG_01854	LC55x_1105	LC55x_1105; acetyltransferase family protein
OG_01855	LC55x_3051	LC55x_3051; conserved hypothetical protein
OG_01856	LC55x_5110	cysT; sulfate ABC transporter, permease protein CysT
OG_01857	LC55x_888	LC55x_888; zinc-binding dehydrogenase family protein
OG_01858	LC55x_3663	purA; adenylosuccinate synthase
OG_01859	LC55x_3429	recO; DNA repair protein RecO
OG_01860	LC55x_5056	LC55x_5056; peptidase MA superfamily protein
OG_01861	LC55x_2482	carB; carbamoyl-phosphate synthase, large subunit
OG_01862	LC55x_3395	ispG; 4-hydroxy-3-methylbut-2-en-1-yl diphosphate synthase
OG_01863	LC55x_2773	LC55x_2773; conserved hypothetical protein
OG_01864	LC55x_1972	rpsP; ribosomal protein S16
OG_01865	LC55x_3092	LC55x_3092; sugar (and other) transporter family protein
OG_01866	LC55x_2771	rpsR; ribosomal protein S18
OG_01867	LC55x_1608	petA; ubiquinol-cytochrome c reductase, iron-sulfur subunit
OG_01868	LC55x_2435	LC55x_2435; putative cell division inhibitor protein
OG_01869	LC55x_1811	LC55x_1811; conserved hypothetical protein
OG_01870	LC55x_3795	LC55x_3795; MAPEG family protein
OG_01871	LC55x_383	LC55x_383; nitronate monooxygenase family protein
OG_01872	LC55x_1597	LC55x_1597; ABC transporter family protein
OG_01873	LC55x_346	LC55x_346; AAA domain family protein
OG_01874	LC55x_3381	pyrD; dihydroorotate dehydrogenase
OG_01875	LC55x_2441	LC55x_2441; RES domain protein
OG_01876	LC55x_3355	LC55x_3355; response regulator
OG_01877	LC55x_3933	secY; preprotein translocase, SecY subunit
OG_01878	LC55x_3817	hutU; urocanate hydratase
OG_01879	LC55x_777	atpF; ATP synthase F0, B subunit
OG_01880	LC55x_386	LC55x_386; putative msr3035 protein

OG_01800	EYR69948.1	sugar ABC transporter permease [Lysobacter capsici AZ78]
OG_01801	EYR68694.1	peptide deformylase [Lysobacter capsici AZ78]
OG_01802	EYR68263.1	hypothetical protein AZ78_11395 [Lysobacter capsici AZ78]
OG_01803	EYR69266.1	hypothetical protein AZ78_06695 [Lysobacter capsici AZ78]
OG_01804	EYR66087.1	X-Pro dipeptidase [Lysobacter capsici AZ78]
OG_01805	EYR66060.1	hypothetical protein AZ78_23155 [Lysobacter capsici AZ78]
OG_01806	EYR69428.1	2-oxoglutarate dehydrogenase E1 [Lysobacter capsici AZ78]
OG_01807	EYR66431.1	membrane protein [Lysobacter capsici AZ78]
OG_01808	EYR69186.1	hypothetical protein AZ78_06865 [Lysobacter capsici AZ78]
OG_01809	EYR68406.1	alpha/beta hydrolase [Lysobacter capsici AZ78]
OG_01810	EYR67825.1	semialdehyde dehydrogenase [Lysobacter capsici AZ78]
OG_01811	EYR70053.1	hemin storage protein [Lysobacter capsici AZ78]
OG_01812	EYR65433.1	hypothetical protein AZ78_26315 [Lysobacter capsici AZ78]
OG_01813	EYR69578.1	hypothetical protein AZ78_03630 [Lysobacter capsici AZ78]
OG_01814	EYR67383.1	acyl-CoA dehydrogenase [Lysobacter capsici AZ78]
OG_01815	EYR67521.1	peptidase [Lysobacter capsici AZ78]
OG_01816	EYR69695.1	cell envelope biogenesis protein TonB [Lysobacter capsici AZ78]
OG_01817	EYR66285.1	hypothetical protein AZ78_22205 [Lysobacter capsici AZ78]
OG_01818	EYR70067.1	cysteinyl-tRNA synthetase [Lysobacter capsici AZ78]
OG_01819	EYR67662.1	pyrroline-5-carboxylate reductase [Lysobacter capsici AZ78]
OG_01820	EYR69712.1	hypothetical protein AZ78_04530 [Lysobacter capsici AZ78]
OG_01821	EYR67972.1	hypothetical protein AZ78_13270 [Lysobacter capsici AZ78]
OG_01822	EYR65667.1	porphobilinogen deaminase [Lysobacter capsici AZ78]
OG_01823	EYR67204.1	mannose-1-phosphate guanylyltransferase [Lysobacter capsici AZ78
OG_01824	EYR67725.1	transketolase [Lysobacter capsici AZ78]
OG_01825	EYR66051.1	1-deoxy-D-xylulose 5-phosphate reductoisomerase [Lysobacter caps
OG_01826	EYR67703.1	hypothetical protein AZ78_14900 [Lysobacter capsici AZ78]
OG_01827	EYR69979.1	ChrA protein [Lysobacter capsici AZ78]
OG_01828	EYR68324.1	ATP-dependent DNA helicase [Lysobacter capsici AZ78]
OG_01829	EYR67381.1	membrane protein [Lysobacter capsici AZ78]
OG_01830	EYR68514.1	psensor histidine kinase [Lysobacter capsici AZ78]
OG_01831	EYR69731.1	Fis family transcriptional regulator [Lysobacter capsici AZ78]
OG_01832	EYR68999.1	hypothetical protein AZ78_07520 [Lysobacter capsici AZ78]
OG_01833	EYR67586.1	transcriptional regulator [Lysobacter capsici AZ78]
OG_01834	EYR68351.1	transporter [Lysobacter capsici AZ78]
OG_01835	EYR67402.1	tetraacyldisaccharide 4'-kinase [Lysobacter capsici AZ78]
OG_01836	EYR69642.1	UTP--glucose-1-phosphate uridylyltransferase [Lysobacter capsici A
OG_01837	EYR67745.1	fimbrial protein [Lysobacter capsici AZ78]
OG_01838	EYR67851.1	NAD(FAD)-utilizing dehydrogenase [Lysobacter capsici AZ78]
OG_01839	EYR68848.1	hypothetical protein AZ78_09415 [Lysobacter capsici AZ78]
OG_01840	EYR65989.1	ribonuclease T [Lysobacter capsici AZ78]
OG_01841	EYR69130.1	cell division protein FtsW [Lysobacter capsici AZ78]
OG_01842	EYR67644.1	RNA polymerase subunit sigma-24 [Lysobacter capsici AZ78]
OG_01843	EYR65599.1	aspartate decarboxylase [Lysobacter capsici AZ78]
OG_01844	EYR65851.1	NADP-dependent aryl-alcohol dehydrogenase [Lysobacter capsici A
OG_01845	EYR70347.1	membrane protein [Lysobacter capsici AZ78]
OG_01846	EYR69164.1	general secretion pathway protein M [Lysobacter capsici AZ78]
OG_01847	EYR65841.1	N5-glutamine S-adenosyl-L-methionine-dependent methyltransferase
OG_01848	EYR68055.1	acyl carrier protein [Lysobacter capsici AZ78]
OG_01849	EYR68866.1	glycerol-3-phosphate acyltransferase [Lysobacter capsici AZ78]
OG_01850	EYR70362.1	DNA glycosylase [Lysobacter capsici AZ78]
OG_01851	EYR69166.1	general secretion pathway protein K [Lysobacter capsici AZ78]
OG_01852	EYR65400.1	hypothetical protein AZ78_26490 [Lysobacter capsici AZ78]
OG_01853	EYR68031.1	membrane protein [Lysobacter capsici AZ78]
OG_01854	EYR68468.1	acetyltransferase [Lysobacter capsici AZ78]
OG_01855	EYR67837.1	membrane protein [Lysobacter capsici AZ78]
OG_01856	EYR68043.1	sulfate/thiosulfate transporter subunit [Lysobacter capsici AZ78]
OG_01857	EYR66870.1	hypothetical protein AZ78_19000 [Lysobacter capsici AZ78]
OG_01858	EYR65952.1	adenylosuccinate synthetase [Lysobacter capsici AZ78]
OG_01859	EYR66501.1	DNA repair protein RecO [Lysobacter capsici AZ78]
OG_01860	EYR65583.1	hypothetical protein AZ78_25545 [Lysobacter capsici AZ78]
OG_01861	EYR69532.1	carbamoyl phosphate synthase large subunit [Lysobacter capsici AZ7
OG_01862	EYR66524.1	4-hydroxy-3-methylbut-2-en-1-yl diphosphate synthase [Lysobacter c
OG_01863	EYR69339.1	hypothetical protein AZ78_05455 [Lysobacter capsici AZ78]
OG_01864	EYR66773.1	30S ribosomal protein S16 [Lysobacter capsici AZ78]
OG_01865	EYR67864.1	MFS transporter [Lysobacter capsici AZ78]
OG_01866	EYR69337.1	30S ribosomal protein S18 [Lysobacter capsici AZ78]
OG_01867	EYR70314.1	ubiquinol-cytochrome C reductase [Lysobacter capsici AZ78]
OG_01868	EYR69565.1	CDP-6-deoxy-delta-3,4-glucoseen reductase [Lysobacter capsici AZ7
OG_01869	EYR67290.1	activator of HSP90 ATPase [Lysobacter capsici AZ78]
OG_01870	EYR67102.1	membrane protein [Lysobacter capsici AZ78]
OG_01871	EYR65629.1	2-nitropropane dioxygenase [Lysobacter capsici AZ78]
OG_01872	EYR70307.1	ABC transporter ATP-binding protein [Lysobacter capsici AZ78]
OG_01873	EYR68123.1	DNA repair ATPase [Lysobacter capsici AZ78]
OG_01874	EYR66531.1	dihydroorotate dehydrogenase [Lysobacter capsici AZ78]
OG_01875	EYR69560.1	hypothetical protein AZ78_03515 [Lysobacter capsici AZ78]
OG_01876	EYR66540.1	transcriptional regulator [Lysobacter capsici AZ78]
OG_01877	EYR66684.1	preprotein translocase subunit SecY [Lysobacter capsici AZ78]
OG_01878	EYR67121.1	urocanate hydratase [Lysobacter capsici AZ78]
OG_01879	EYR67613.1	F0F1 ATP synthase subunit B [Lysobacter capsici AZ78]
OG_01880	EYR65626.1	hypothetical protein AZ78_25310 [Lysobacter capsici AZ78]

OG_01881	LC55x_871	prmA; ribosomal protein L11 methyltransferase	OG_01881	EYR66886.1	ribosomal protein L11 methyltransferase [Lysobacter capsici AZ78]
OG_01882	LC55x_69	LC55x_69; pyridine nucleotide-disulfide oxidoreductase family protein	OG_01882	EYR69744.1	pyridine nucleotide-disulfide oxidoreductase [Lysobacter capsici AZ7
OG_01883	LC55x_3432	rnc; ribonuclease III	OG_01883	EYR66499.1	ribonuclease III [Lysobacter capsici AZ78]
OG_01884	LC55x_976	LC55x_976; astC	OG_01884	EYR65393.1	acetylornithine aminotransferase [Lysobacter capsici AZ78]
OG_01885	LC55x_3936	rpsE; ribosomal protein S5	OG_01885	EYR66687.1	30S ribosomal protein S5 [Lysobacter capsici AZ78]
OG_01886	LC55x_364	ubiB; 2-polyprenylphenol 6-hydroxylase	OG_01886	EYR68137.1	ubiquinone biosynthesis protein UbiB [Lysobacter capsici AZ78]
OG_01887	LC55x_2157	MEP; peptidyl-Lys metalloendopeptidase	OG_01887	EYR67377.1	peptidase M35 [Lysobacter capsici AZ78]
OG_01888	LC55x_695	LC55x_695; tetratricopeptide repeat family protein	OG_01888	EYR70104.1	hypothetical protein AZ78_02020 [Lysobacter capsici AZ78]
OG_01889	LC55x_2423	LC55x_2423; bacterial conjugation TrbI-like family protein	OG_01889	EYR69575.1	secretion protein [Lysobacter capsici AZ78]
OG_01890	LC55x_589	LC55x_589; GDSL-like Lipase/Acylhydrolase family protein	OG_01890	EYR70012.1	lysophospholipase [Lysobacter capsici AZ78]
OG_01891	LC55x_5173	LC55x_5173; dinB family protein	OG_01891	EYR67997.1	diguanylate cyclase [Lysobacter capsici AZ78]
OG_01892	LC55x_211	LC55x_211; subtilase family protein	OG_01892	EYR66155.1	hypothetical protein AZ78_22565 [Lysobacter capsici AZ78]
OG_01893	LC55x_2304	LC55x_2304; traB family protein	OG_01893	EYR69659.1	pheromone shutdown protein [Lysobacter capsici AZ78]
OG_01894	LC55x_2455	LC55x_2455; conserved hypothetical protein	OG_01894	EYR69819.1	hypothetical protein AZ78_03455 [Lysobacter capsici AZ78]
OG_01895	LC55x_5184	tatC; twin arginine-targeting protein translocase TatC	OG_01895	EYR68856.1	preprotein translocase subunit TatC [Lysobacter capsici AZ78]
OG_01896	LC55x_305	sod-1; superoxide dismutase [Cu-Zn]	OG_01896	EYR67035.1	superoxide dismutase [Lysobacter capsici AZ78]
OG_01897	LC55x_3409	alpha-LP; alpha-lytic protease	OG_01897	EYR66514.1	serine protease [Lysobacter capsici AZ78]
OG_01898	LC55x_5684	LC55x_5684; lysM domain protein	OG_01898	EYR65874.1	peptidoglycan-binding protein LysM [Lysobacter capsici AZ78]
OG_01899	LC55x_5606	LC55x_5606; bacterial regulatory helix-turn-helix , lysR family protein	OG_01899	EYR66246.1	LysR family transcriptional regulator [Lysobacter capsici AZ78]
OG_01900	LC55x_1220	LC55x_1220; bacterial regulatory, tetR family protein	OG_01900	EYR65761.1	TetR family transcriptional regulator [Lysobacter capsici AZ78]
OG_01901	LC55x_3054	fbp; peptidyl-prolyl cis-trans isomerase domain protein	OG_01901	EYR67839.1	peptidyl-prolyl cis-trans isomerase [Lysobacter capsici AZ78]
OG_01902	LC55x_3545	LC55x_3545; conserved hypothetical protein	OG_01902	EYR67497.1	hypothetical protein AZ78_15235 [Lysobacter capsici AZ78]
OG_01903	LC55x_1850	LC55x_1850; hypothetical protein	OG_01903	EYR66042.1	hypothetical protein AZ78_23065 [Lysobacter capsici AZ78]
OG_01904	LC55x_223	LC55x_223; hypothetical protein	OG_01904	EYR66163.1	hypothetical protein AZ78_22605 [Lysobacter capsici AZ78]
OG_01905	LC55x_250	LC55x_250; hypothetical protein	OG_01905	EYR66981.1	hypothetical protein AZ78_17865 [Lysobacter capsici AZ78]
OG_01906	LC55x_2518	LC55x_2518; bacterial sensory transduction regulator family protein	OG_01906	EYR69500.1	hypothetical protein AZ78_03150 [Lysobacter capsici AZ78]
OG_01907	LC55x_3839	lpxH; UDP-2,3-diacylglucosamine hydrolase	OG_01907	EYR65740.1	UDP-2,3-diacylglucosamine hydrolase [Lysobacter capsici AZ78]
OG_01908	LC55x_2386	LC55x_2386; 3HB-oligomer hydrolase family protein	OG_01908	EYR69601.1	hydrogenase [Lysobacter capsici AZ78]
OG_01909	LC55x_2733	LC55x_2733; putative domain protein	OG_01909	EYR69303.1	hypothetical protein AZ78_05270 [Lysobacter capsici AZ78]
OG_01910	LC55x_3776	LC55x_3776; tonB dependent receptor family protein	OG_01910	EYR67086.1	hypothetical protein AZ78_17650 [Lysobacter capsici AZ78]
OG_01911	LC55x_2012	LC55x_2012; acetyltransferase family protein	OG_01911	EYR66745.1	MarR family transcriptional regulator [Lysobacter capsici AZ78]
OG_01912	LC55x_3878	LC55x_3878; zinc-binding alcohol dehydrogenase family protein	OG_01912	EYR65712.1	NADPH:quinone reductase [Lysobacter capsici AZ78]
OG_01913	LC55x_4247	LC55x_4247; conserved hypothetical protein	OG_01913	EYR69102.1	laccase [Lysobacter capsici AZ78]
OG_01914	LC55x_348	LC55x_348; coA-transferase III family protein	OG_01914	EYR68125.1	formyl-CoA transferase [Lysobacter capsici AZ78]
OG_01915	LC55x_4902	LC55x_4902; alpha-1,2-mannosidase family protein	OG_01915	EYR68711.1	sugar hydrolase [Lysobacter capsici AZ78]
OG_01916	LC55x_1758	LC55x_1758; bacterial low temperature requirement A family protein	OG_01916	EYR65692.1	membrane protein [Lysobacter capsici AZ78]
OG_01917	LC55x_3715	LC55x_3715; glutathione amide reductase	OG_01917	EYR65907.1	glutathione reductase [Lysobacter capsici AZ78]
OG_01918	LC55x_512	cca; tRNA nucleotidyltransferase	OG_01918	EYR66434.1	hypothetical protein AZ78_20805 [Lysobacter capsici AZ78]
OG_01919	LC55x_1571	LC55x_1571; carbon starvation CstA family protein	OG_01919	EYR70290.1	carbon starvation protein A [Lysobacter capsici AZ78]
OG_01920	LC55x_3870	LC55x_3870; catalytic LigB subunit of aromatic ring-opening dioxyge	OG_01920	EYR65540.1	hypothetical protein AZ78_25665 [Lysobacter capsici AZ78]
OG_01921	LC55x_1165	LC55x_1165; TIM-barrel , nifR3 family protein	OG_01921	EYR68533.1	tRNA-dihydrouridine synthase B [Lysobacter capsici AZ78]
OG_01922	LC55x_587	LC55x_587; polysaccharide deacetylase family protein	OG_01922	EYR70014.1	acetylxylan esterase [Lysobacter capsici AZ78]
OG_01923	LC55x_3104	LC55x_3104; carboxylate-amine ligase, YbdK family protein	OG_01923	EYR65453.1	hypothetical protein AZ78_26265 [Lysobacter capsici AZ78]
OG_01924	LC55x_748	LC55x_748; killing trait family protein	OG_01924	EYR67640.1	RebB protein [Lysobacter capsici AZ78]
OG_01925	LC55x_122	gidA; tRNA uridine 5-carboxymethylaminomethyl modification enzyme	OG_01925	EYR69777.1	tRNA uridine 5-carboxymethylaminomethyl modification protein [Lyso
OG_01926	LC55x_1563	rfbD; dTDP-4-dehydrorhamnose reductase	OG_01926	EYR70283.1	dTDP-4-dehydrorhamnose reductase [Lysobacter capsici AZ78]
OG_01927	LC55x_4292	ribH; 6,7-dimethyl-8-ribityllumazine synthase	OG_01927	EYR69146.1	6,7-dimethyl-8-ribityllumazine synthase [Lysobacter capsici AZ78]
OG_01928	LC55x_2414	LC55x_2414; hypothetical protein	OG_01928	EYR69583.1	heme exporter protein CcmD [Lysobacter capsici AZ78]
OG_01929	LC55x_5237	folE; GTP cyclohydrolase I	OG_01929	EYR68894.1	GTP cyclohydrolase [Lysobacter capsici AZ78]
OG_01930	LC55x_3792	LC55x_3792; bacterial regulatory , arsR family protein	OG_01930	EYR67099.1	ArsR family transcriptional regulator [Lysobacter capsici AZ78]
OG_01931	LC55x_4615	LC55x_4615; ABC transporter family protein	OG_01931	EYR68345.1	ABC transporter ATP-binding protein [Lysobacter capsici AZ78]
OG_01932	LC55x_3788	LC55x_3788; putative fatty acid desaturase protein	OG_01932	EYR67095.1	hypothetical protein AZ78_17695 [Lysobacter capsici AZ78]
OG_01933	LC55x_4305	LC55x_4305; aspartyl/Asparaginyl beta-hydroxylase family protein	OG_01933	EYR69154.1	aspartyl/asparaginyl beta-hydroxylase [Lysobacter capsici AZ78]
OG_01934	LC55x_3207	LC55x_3207; feoA domain protein	OG_01934	EYR67981.1	iron transporter [Lysobacter capsici AZ78]
OG_01935	LC55x_5142	LC55x_5142; glycosyl transferase 2 family protein	OG_01935	EYR68019.1	dolichol-phosphate mannosyltransferase [Lysobacter capsici AZ78]
OG_01936	LC55x_2939	LC55x_2939; binding-protein-dependent transport system inner mem	OG_01936	EYR66221.1	sugar ABC transporter permease [Lysobacter capsici AZ78]
OG_01938	LC55x_3964	rplK; ribosomal protein L11	OG_01938	EYR65483.1	50S ribosomal protein L11 [Lysobacter capsici AZ78]
OG_01939	LC55x_492	LC55x_492; hypothetical protein	OG_01939	EYR66484.1	hypothetical protein AZ78_20905 [Lysobacter capsici AZ78]
OG_01940	LC55x_4911	rpoD; RNA polymerase sigma factor RpoD	OG_01940	EYR68705.1	RNA polymerase sigma factor RpoD [Lysobacter capsici AZ78]
OG_01941	LC55x_1895	LC55x_1895; conserved hypothetical protein	OG_01941	EYR65997.1	hypothetical protein AZ78_23515 [Lysobacter capsici AZ78]
OG_01942	LC55x_2994	pnp; polyribonucleotide nucleotidyltransferase	OG_01942	EYR67794.1	polynucleotide phosphorylase/polyadenylase [Lysobacter capsici AZ
OG_01943	LC55x_4300	nrdR; transcriptional regulator NrdR	OG_01943	EYR69152.1	NrdR family transcriptional regulator [Lysobacter capsici AZ78]
OG_01944	LC55x_1788	LC55x_1788; xaa-Pro dipeptidase, putative	OG_01944	EYR67281.1	amidohydrolase [Lysobacter capsici AZ78]
OG_01945	LC55x_1748	LC55x_1748; conserved hypothetical protein	OG_01945	EYR65701.1	hypothetical protein AZ78_24940 [Lysobacter capsici AZ78]
OG_01946	LC55x_4832	LC55x_4832; AIG2-like family protein	OG_01946	EYR68761.1	hypothetical protein AZ78_09195 [Lysobacter capsici AZ78]
OG_01947	LC55x_1108	LC55x_1108; peptidase M28	OG_01947	EYR68471.1	aminopeptidase [Lysobacter capsici AZ78]
OG_01948	LC55x_5137	gidB; 16S rRNA (guanine(527)-N(7))-methyltransferase GidB	OG_01948	EYR68023.1	16S rRNA methyltransferase [Lysobacter capsici AZ78]
OG_01949	LC55x_3489	LC55x_3489; lipopolysaccharide kinase family protein	OG_01949	EYR66621.1	3-deoxy-D-manno-octulosonic acid kinase [Lysobacter capsici AZ78]
OG_01950	LC55x_1923	LC55x_1923; hypothetical protein	OG_01950	EYR65976.1	hypothetical protein AZ78_23400 [Lysobacter capsici AZ78]
OG_01951	LC55x_776	atpE; ATP synthase F0, C subunit	OG_01951	EYR67614.1	F0F1 ATP synthase subunit C [Lysobacter capsici AZ78]
OG_01952	LC55x_94	LC55x_94; STAS domain protein	OG_01952	EYR69758.1	anti-sigma B factor antagonist [Lysobacter capsici AZ78]
OG_01953	LC55x_2655	LC55x_2655; nucleotide sugar dehydrogenase family protein	OG_01953	EYR68243.1	UDP-N-acetyl-D-mannosamine dehydrogenase [Lysobacter capsici A
OG_01954	LC55x_1746	LC55x_1746; conserved hypothetical protein	OG_01954	EYR65703.1	hypothetical protein AZ78_24950 [Lysobacter capsici AZ78]
OG_01955	LC55x_5168	LC55x_5168; CHAD domain protein	OG_01955	EYR68074.1	hypothetical protein AZ78_12335 [Lysobacter capsici AZ78]
OG_01956	LC55x_1969	LC55x_1969; marR family protein	OG_01956	EYR66776.1	MarR family transcriptional regulator [Lysobacter capsici AZ78]
OG_01957	LC55x_4494	LC55x_4494; cysteine-rich CPXCG family protein	OG_01957	EYR68416.1	hypothetical protein AZ78_10150 [Lysobacter capsici AZ78]
OG_01958	LC55x_2805	LC55x_2805; conserved hypothetical protein	OG_01958	EYR69368.1	hypothetical protein AZ78_05600 [Lysobacter capsici AZ78]
OG_01959	LC55x_1793	leuS; leucine--tRNA ligase	OG_01959	EYR67283.1	leucyl-tRNA synthetase [Lysobacter capsici AZ78]
OG_01960	LC55x_227	LC55x_227; type III secretion , HrpO family protein	OG_01960	EYR66166.1	HrpO family type III secretion protein [Lysobacter capsici AZ78]
OG_01961	LC55x_3544	LC55x_3544; conserved hypothetical protein	OG_01961	EYR67496.1	23S rRNA methyltransferase [Lysobacter capsici AZ78]
OG_01962	LC55x_3961	rplL; ribosomal protein L7/L12	OG_01962	EYR65486.1	50S ribosomal protein L7 [Lysobacter capsici AZ78]

OG_01963	LC55x_1331	murA; UDP-N-acetylglucosamine 1-carboxyvinyltransferase	OG_01963	EYR69906.1	UDP-N-acetylglucosamine 1-carboxyvinyltransferase [Lysobacter ca
OG_01964	LC55x_1264	LC55x_1264; yceI-like domain protein	OG_01964	EYR67680.1	hypothetical protein AZ78_15090 [Lysobacter capsici AZ78]
OG_01965	LC55x_5203	LC55x_5203; spore Coat Protein U domain protein	OG_01965	EYR68868.1	protein U [Lysobacter capsici AZ78]
OG_01966	LC55x_4129	LC55x_4129; NUDIX domain protein	OG_01966	EYR66351.1	ADP-ribose diphosphatase [Lysobacter capsici AZ78]
OG_01967	LC55x_1394	LC55x_1394; pepSY-associated TM helix family protein	OG_01967	EYR67174.1	membrane protein [Lysobacter capsici AZ78]
OG_01968	LC55x_610	aceE; pyruvate dehydrogenase (acetyl-transferring), homodimeric typ	OG_01968	EYR69995.1	pyruvate dehydrogenase [Lysobacter capsici AZ78]
OG_01969	LC55x_535	LC55x_535; periplasmic binding and sugar binding domain of LacI fa	OG_01969	EYR67895.1	LacI family transcriptional regulator [Lysobacter capsici AZ78]
OG_01970	LC55x_2342	LC55x_2342; thiF family protein	OG_01970	EYR69628.1	molybdopterin biosynthesis protein MoeB [Lysobacter capsici AZ78]
OG_01971	LC55x_3257	mtnB; methylthioribulose-1-phosphate dehydratase	OG_01971	EYR67926.1	methylthioribulose-1-phosphate dehydratase [Lysobacter capsici AZ
OG_01972	LC55x_5000	LC55x_5000; response regulator	OG_01972	EYR67229.1	transcriptional regulator [Lysobacter capsici AZ78]
OG_01973	LC55x_3487	moaA; molybdenum cofactor biosynthesis protein A	OG_01973	EYR66619.1	molybdenum cofactor biosynthesis protein MoeA [Lysobacter capsici
OG_01974	LC55x_1073	gap; glyceraldehyde-3-phosphate dehydrogenase, type I	OG_01974	EYR67716.1	glyceraldehyde-3-phosphate dehydrogenase [Lysobacter capsici AZ
OG_01975	LC55x_4092	LC55x_4092; ostA-like family protein	OG_01975	EYR65493.1	organic solvent tolerance protein [Lysobacter capsici AZ78]
OG_01976	LC55x_1796	LC55x_1796; lipopolysaccharide-assembly family protein	OG_01976	EYR67285.1	hypothetical protein AZ78_16245 [Lysobacter capsici AZ78]
OG_01977	LC55x_439	pyrE; orotate phosphoribosyltransferase	OG_01977	EYR65524.1	orotate phosphoribosyltransferase [Lysobacter capsici AZ78]
OG_01978	LC55x_561	LC55x_561; conserved hypothetical protein	OG_01978	EYR67918.1	hypothetical protein AZ78_12995 [Lysobacter capsici AZ78]
OG_01979	LC55x_4708	LC55x_4708; alpha/beta hydrolase fold family protein	OG_01979	EYR65670.1	carboxylesterase [Lysobacter capsici AZ78]
OG_01980	LC55x_4457	LC55x_4457; putative stress response protein	OG_01980	EYR68441.1	hypothetical protein AZ78_10310 [Lysobacter capsici AZ78]
OG_01981	LC55x_3586	LC55x_3586; conserved hypothetical protein	OG_01981	EYR67533.1	hypothetical protein AZ78_15415 [Lysobacter capsici AZ78]
OG_01982	LC55x_4278	mraW; S-adenosyl-methyltransferase MraW	OG_01982	EYR69136.1	16S rRNA methyltransferase [Lysobacter capsici AZ78]
OG_01983	LC55x_2436	lexA; repressor LexA	OG_01983	EYR69564.1	LexA family transcriptional regulator [Lysobacter capsici AZ78]
OG_01984	LC55x_771	lpdA; dihydrolipoyl dehydrogenase	OG_01984	EYR67619.1	dihydrolipoamide dehydrogenase [Lysobacter capsici AZ78]
OG_01985	LC55x_1693	LC55x_1693; conserved hypothetical protein	OG_01985	EYR70357.1	hypothetical protein AZ78_01405 [Lysobacter capsici AZ78]
OG_01986	LC55x_3446	fabF; beta-ketoacyl-acyl-carrier-protein synthase II	OG_01986	EYR66593.1	3-oxoacyl-ACP synthase [Lysobacter capsici AZ78]
OG_01987	LC55x_1744	LC55x_1744; ATPase associated with various cellular activities family	OG_01987	EYR65705.1	cell division protein [Lysobacter capsici AZ78]
OG_01988	LC55x_3010	nuoG; NADH dehydrogenase (quinone), G subunit	OG_01988	EYR67808.1	NADH dehydrogenase subunit G [Lysobacter capsici AZ78]
OG_01989	LC55x_1963	LC55x_1963; enoyl-CoA hydratase/isomerase family protein	OG_01989	EYR65956.1	hydrogenase maturation protein [Lysobacter capsici AZ78]
OG_01990	LC55x_2388	pssA; CDP-diacylglycerol-serine O-phosphatidyltransferase	OG_01990	EYR69600.1	CDP-diacylglycerol--serine O-phosphatidyltransferase [Lysobacter ca
OG_01991	LC55x_5611	LC55x_5611; ahpC/TSA family protein	OG_01991	EYR66249.1	thioredoxin [Lysobacter capsici AZ78]
OG_01992	LC55x_2947	LC55x_2947; conserved hypothetical protein	OG_01992	EYR66213.1	hypothetical protein AZ78_21835 [Lysobacter capsici AZ78]
OG_01993	LC55x_955	LC55x_955; fatty acid hydroxylase superfamily protein	OG_01993	EYR66809.1	fatty acid hydroxylase [Lysobacter capsici AZ78]
OG_01994	LC55x_1593	LC55x_1593; saccharopine dehydrogenase family protein	OG_01994	EYR70304.1	Sterol-4-alpha-carboxylate 3-dehydrogenase [Lysobacter capsici AZ
OG_01995	LC55x_4737	LC55x_4737; conserved hypothetical protein	OG_01995	EYR66107.1	membrane protein [Lysobacter capsici AZ78]
OG_01996	LC55x_1304	LC55x_1304; conserved hypothetical protein	OG_01996	EYR69928.1	hypothetical protein AZ78_01865 [Lysobacter capsici AZ78]
OG_01997	LC55x_2581	LC55x_2581; 2OG-Fe(II) oxygenase superfamily protein	OG_01997	EYR68313.1	hypothetical protein AZ78_11650 [Lysobacter capsici AZ78]
OG_01998	LC55x_1653	LC55x_1653; putative pilW	OG_01998	EYR70330.1	pilus assembly protein PilW [Lysobacter capsici AZ78]
OG_01999	LC55x_1139	LC55x_1139; transcriptional regulatory , C terminal family protein	OG_01999	EYR68513.1	XRE family transcriptional regulator [Lysobacter capsici AZ78]
OG_02000	LC55x_689	LC55x_689; conserved hypothetical protein	OG_02000	EYR69950.1	activator of HSP90 ATPase [Lysobacter capsici AZ78]
OG_02001	LC55x_425	LC55x_425; NAD binding domain of 6-phosphogluconate dehydroger	OG_02001	EYR65612.1	hypothetical protein AZ78_25240 [Lysobacter capsici AZ78]
OG_02002	LC55x_4253	sucC; succinyl-CoA synthetase, &beta subunit	OG_02002	EYR69108.1	malate--CoA ligase subunit beta [Lysobacter capsici AZ78]
OG_02003	LC55x_2875	LC55x_2875; response regulator	OG_02003	EYR69434.1	chemotaxis protein CheY [Lysobacter capsici AZ78]
OG_02004	LC55x_2156	LC55x_2156; 'Cold-shock' DNA-binding domain protein	OG_02004	EYR67376.1	cold-shock protein [Lysobacter capsici AZ78]
OG_02005	LC55x_5233	LC55x_5233; sugar (and other) transporter family protein	OG_02005	EYR68891.1	MFS transporter [Lysobacter capsici AZ78]
OG_02006	LC55x_3511	LC55x_3511; ppx/GppA phosphatase family protein	OG_02006	EYR66637.1	exopolyphosphatase [Lysobacter capsici AZ78]
OG_02007	LC55x_4491	LC55x_4491; hydrolase, haloacid delahogenase-like family	OG_02007	EYR68419.1	haloacid dehalogenase [Lysobacter capsici AZ78]
OG_02008	LC55x_1821	LC55x_1821; bacterial DNA-binding family protein	OG_02008	EYR67308.1	transcriptional regulator [Lysobacter capsici AZ78]
OG_02009	LC55x_3965	nusG; transcription termination/antitermination factor NusG	OG_02009	EYR65482.1	transcription antitermination protein NusG [Lysobacter capsici AZ78]
OG_02010	LC55x_5179	glyQ; glycine--tRNA ligase, alpha subunit	OG_02010	EYR68853.1	glycyl-tRNA synthetase [Lysobacter capsici AZ78]
OG_02011	LC55x_5186	tatA; twin arginine-targeting translocase, TatA/E family protein	OG_02011	EYR68858.1	preprotein translocase subunit TatA [Lysobacter capsici AZ78]
OG_02012	LC55x_5718	LC55x_5718; ABC-2 type transporter family protein	OG_02012	EYR69689.1	membrane protein [Lysobacter capsici AZ78]
OG_02013	LC55x_5160	LC55x_5160; conserved hypothetical protein	OG_02013	EYR68005.1	membrane protein [Lysobacter capsici AZ78]
OG_02014	LC55x_5158	LC55x_5158; sugar (and other) transporter family protein	OG_02014	EYR68007.1	major facilitator transporter [Lysobacter capsici AZ78]
OG_02015	LC55x_3954	rpsJ; ribosomal protein S10	OG_02015	EYR66705.1	30S ribosomal protein S10 [Lysobacter capsici AZ78]
OG_02016	LC55x_4715	LC55x_4715; conserved hypothetical protein	OG_02016	EYR65677.1	hypothetical protein AZ78_25080 [Lysobacter capsici AZ78]
OG_02017	LC55x_3758	LC55x_3758; conserved hypothetical protein	OG_02017	EYR67068.1	hypothetical protein AZ78_17555 [Lysobacter capsici AZ78]
OG_02018	LC55x_3979	lolB; outer membrane lipoprotein LolB	OG_02018	EYR66710.1	membrane protein [Lysobacter capsici AZ78]
OG_02019	LC55x_602	LC55x_602; malic enzyme, NAD binding domain protein	OG_02019	EYR70003.1	malic enzyme [Lysobacter capsici AZ78]
OG_02020	LC55x_3374	LC55x_3374; conserved hypothetical protein	OG_02020	EYR66532.1	membrane protein [Lysobacter capsici AZ78]
OG_02021	LC55x_14	pdxJ; pyridoxine 5'-phosphate synthase	OG_02021	EYR69717.1	pyridoxamine 5'-phosphate oxidase [Lysobacter capsici AZ78]
OG_02022	LC55x_1745	LC55x_1745; AAA domain family protein	OG_02022	EYR65704.1	ATPase AAA [Lysobacter capsici AZ78]
OG_02023	LC55x_17	LC55x_17; conserved hypothetical protein	OG_02023	EYR69860.1	hypothetical protein AZ78_04575 [Lysobacter capsici AZ78]
OG_02024	LC55x_1934	LC55x_1934; conserved hypothetical protein	OG_02024	EYR65970.1	thioesterase [Lysobacter capsici AZ78]
OG_02025	LC55x_2379	LC55x_2379; putative calU2	OG_02025	EYR69606.1	hypothetical protein AZ78_03825 [Lysobacter capsici AZ78]
OG_02026	LC55x_4925	LC55x_4925; conserved hypothetical protein	OG_02026	EYR68691.1	hypothetical protein AZ78_08720 [Lysobacter capsici AZ78]
OG_02027	LC55x_3906	mdh; malate dehydrogenase	OG_02027	EYR66663.1	malate dehydrogenase [Lysobacter capsici AZ78]
OG_02028	LC55x_2567	cueR; cu(I)-responsive transcriptional regulator	OG_02028	EYR68326.1	hypothetical protein AZ78_11715 [Lysobacter capsici AZ78]
OG_02029	LC55x_5206	LC55x_5206; spore Coat Protein U domain protein	OG_02029	EYR68871.1	hypothetical protein AZ78_07370 [Lysobacter capsici AZ78]
OG_02030	LC55x_774	LC55x_774; conserved hypothetical protein	OG_02030	EYR67617.1	membrane protein [Lysobacter capsici AZ78]
OG_02031	LC55x_3687	surE; 5'/3'-nucleotidase SurE	OG_02031	EYR65931.1	stationary phase survival protein SurE [Lysobacter capsici AZ78]
OG_02032	LC55x_3632	LC55x_3632; response regulator	OG_02032	EYR68660.1	transcriptional regulator [Lysobacter capsici AZ78]
OG_02033	LC55x_1045	LC55x_1045; von Willebrand factor type A domain protein	OG_02033	EYR67739.1	hypothetical protein AZ78_14060 [Lysobacter capsici AZ78]
OG_02034	LC55x_762	adhC2; NADP-dependent alcohol dehydrogenase C 2	OG_02034	EYR67627.1	alcohol dehydrogenase [Lysobacter capsici AZ78]
OG_02035	LC55x_2317	ihfB; integration host factor, beta subunit	OG_02035	EYR69647.1	integration host factor subunit beta [Lysobacter capsici AZ78]
OG_02036	LC55x_3847	LC55x_3847; colicin V production family protein	OG_02036	EYR65731.1	hypothetical protein AZ78_24630 [Lysobacter capsici AZ78]
OG_02037	LC55x_3763	LC55x_3763; formate dehydrogenase family accessory protein FdhD	OG_02037	EYR67072.1	formate dehydrogenase accessory protein FdhD [Lysobacter capsici
OG_02038	LC55x_4352	LC55x_4352; CAAX protease self-immunity family protein	OG_02038	EYR69192.1	CAAX amino terminal protease [Lysobacter capsici AZ78]
OG_02039	LC55x_5024	LC55x_5024; response regulator	OG_02039	EYR66316.1	hypothetical protein AZ78_22360 [Lysobacter capsici AZ78]
OG_02040	LC55x_1432	LC55x_1432; putative secreted protein	OG_02040	EYR67143.1	hypothetical protein AZ78_17120 [Lysobacter capsici AZ78]
OG_02041	LC55x_3729	LC55x_3729; peptidase M16 inactive domain protein	OG_02041	EYR65896.1	hypothetical protein AZ78_23595 [Lysobacter capsici AZ78]
OG_02042	LC55x_823	LC55x_823; GAF domain protein	OG_02042	EYR67585.1	diguanylate cyclase [Lysobacter capsici AZ78]
OG_02043	LC55x_862	LC55x_862; conserved hypothetical protein	OG_02043	EYR66895.1	hypothetical protein AZ78_19130 [Lysobacter capsici AZ78]

OG_02044	LC55x_834	LC55x_834; conserved hypothetical protein	OG_02044	EYR67579.1	membrane protein [Lysobacter capsici AZ78]
OG_02045	LC55x_3524	LC55x_3524; transcriptional regulatory , C terminal family protein	OG_02045	EYR67475.1	transcriptional regulator [Lysobacter capsici AZ78]
OG_02046	LC55x_5029	rhlB; EG10844	OG_02046	EYR66311.1	RNA helicase [Lysobacter capsici AZ78]
OG_02047	LC55x_2504	acnA; aconitate hydratase 1	OG_02047	EYR69514.1	aconitate hydratase [Lysobacter capsici AZ78]
OG_02048	LC55x_1678	LC55x_1678; traB family protein	OG_02048	EYR70345.1	GumN protein [Lysobacter capsici AZ78]
OG_02049	LC55x_990	LC55x_990; ABC transporter family protein	OG_02049	EYR67783.1	multidrug ABC transporter ATP-binding protein [Lysobacter capsici AZ
OG_02050	LC55x_194	LC55x_194; phosphatidylethanolamine-binding family protein	OG_02050	EYR66134.1	phosphatidylethanolamine-binding protein [Lysobacter capsici AZ78]
OG_02051	LC55x_3905	LC55x_3905; bacterial regulatory, luxR family protein	OG_02051	EYR66662.1	LuxR family transcriptional regulator [Lysobacter capsici AZ78]
OG_02052	LC55x_889	bsn; extracellular ribonuclease domain protein	OG_02052	EYR66868.1	ribonuclease [Lysobacter capsici AZ78]
OG_02053	LC55x_534	LC55x_534; alpha amylase, catalytic domain protein	OG_02053	EYR67894.1	cyclomaltodextrin glucanotransferase [Lysobacter capsici AZ78]
OG_02054	LC55x_217	LC55x_217; bacterial regulatory , Fis family protein	OG_02054	EYR66159.1	chemotaxis protein CheY [Lysobacter capsici AZ78]
OG_02055	LC55x_5112	cysA; sulfate ABC transporter, ATP-binding family protein	OG_02055	EYR68041.1	sulfate ABC transporter ATP-binding protein [Lysobacter capsici AZ7
OG_02056	LC55x_4480	LC55x_4480; rtcR transcriptional regulator RtcR transcriptional activa	OG_02056	EYR68424.1	Fis family transcriptional regulator [Lysobacter capsici AZ78]
OG_02057	LC55x_1904	LC55x_1904; chitinase class I family protein	OG_02057	EYR65990.1	pyocin R, lytic enzyme [Lysobacter capsici AZ78]
OG_02058	LC55x_4322	LC55x_4322; type II transport GspH family protein	OG_02058	EYR69169.1	general secretion pathway protein H [Lysobacter capsici AZ78]
OG_02059	LC55x_5232	LC55x_5232; hypothetical protein	OG_02059	EYR68998.1	hypothetical protein AZ78_07490 [Lysobacter capsici AZ78]
OG_02060	LC55x_2999	infB; translation initiation factor IF-2	OG_02060	EYR67798.1	translation initiation factor IF-2 [Lysobacter capsici AZ78]
OG_02061	LC55x_98	LC55x_98; ABC transporter family protein	OG_02061	EYR69762.1	ABC transporter ATP-binding protein [Lysobacter capsici AZ78]
OG_02062	LC55x_1855	fabZ; beta-hydroxyacyl-(acyl-carrier-protein) dehydratase FabZ	OG_02062	EYR66047.1	3-hydroxyacyl-ACP dehydratase [Lysobacter capsici AZ78]
OG_02063	LC55x_4702	nrfC; nrfC protein	OG_02063	EYR65664.1	hypothetical protein AZ78_25015 [Lysobacter capsici AZ78]
OG_02064	LC55x_4312	LC55x_4312; metallo-beta-lactamase superfamily protein	OG_02064	EYR69159.1	beta-lactamase [Lysobacter capsici AZ78]
OG_02065	LC55x_2262	LC55x_2262; tonB family C-terminal domain protein	OG_02065	EYR67448.1	energy transducer TonB [Lysobacter capsici AZ78]
OG_02066	LC55x_170	LC55x_170; AIG2-like family protein	OG_02066	EYR65411.1	hypothetical protein AZ78_26440 [Lysobacter capsici AZ78]
OG_02067	LC55x_1818	clpP; ATP-dependent Clp endopeptidase, proteolytic subunit ClpP	OG_02067	EYR67305.1	ATP-dependent Clp protease proteolytic subunit [Lysobacter capsici A
OG_02068	LC55x_1185	LC55x_1185; hypothetical protein	OG_02068	EYR65798.1	hypothetical protein AZ78_24575 [Lysobacter capsici AZ78]
OG_02069	LC55x_5090	LC55x_5090; bacterial lipid A biosynthesis acyltransferase family prot	OG_02069	EYR68058.1	acyltransferase [Lysobacter capsici AZ78]
OG_02070	LC55x_2056	LC55x_2056; traB family protein	OG_02070	EYR65607.1	hypothetical protein AZ78_25480 [Lysobacter capsici AZ78]
OG_02071	LC55x_1161	LC55x_1161; calcineurin-like phosphoesterase family protein	OG_02071	EYR68529.1	metallophosphoesterase [Lysobacter capsici AZ78]
OG_02072	LC55x_2179	LC55x_2179; liporeleasing system, transmembrane , LolC/E family pr	OG_02072	EYR67396.1	cell division protein FtsX [Lysobacter capsici AZ78]
OG_02073	LC55x_4364	LC55x_4364; aldose 1-epimerase family protein	OG_02073	EYR69201.1	aldose 1-epimerase [Lysobacter capsici AZ78]
OG_02074	LC55x_813	LC55x_813; hypothetical protein	OG_02074	EYR67689.1	hypothetical protein AZ78_14490 [Lysobacter capsici AZ78]
OG_02075	LC55x_1437	LC55x_1437; phenazine biosynthesis , PhzF family protein	OG_02075	EYR67139.1	phenazine antibiotic biosynthesis protein [Lysobacter capsici AZ78]
OG_02076	LC55x_4753	LC55x_4753; helix-turn-helix domain, rpiR family protein	OG_02076	EYR66093.1	transcriptional regulator [Lysobacter capsici AZ78]
OG_02077	LC55x_1866	pyrH; UMP kinase	OG_02077	EYR66058.1	uridylate kinase [Lysobacter capsici AZ78]
OG_02078	LC55x_1020	LC55x_1020; endoribonuclease L-PSP, putative	OG_02078	EYR67760.1	endoribonuclease [Lysobacter capsici AZ78]
OG_02079	LC55x_1046	LC55x_1046; von Willebrand factor type A domain protein	OG_02079	EYR67738.1	membrane protein [Lysobacter capsici AZ78]
OG_02080	LC55x_2394	LC55x_2394; conserved hypothetical protein	OG_02080	EYR69594.1	hypothetical protein AZ78_03755 [Lysobacter capsici AZ78]
OG_02081	LC55x_2233	LC55x_2233; diguanylate cyclase domain protein	OG_02081	EYR67427.1	histidine kinase [Lysobacter capsici AZ78]
OG_02082	LC55x_370	LC55x_370; acyltransferase family protein	OG_02082	EYR68141.1	acyltransferase [Lysobacter capsici AZ78]
OG_02083	LC55x_5699	LC55x_5699; hypothetical protein	OG_02083	EYR65859.1	hypothetical protein AZ78_23985 [Lysobacter capsici AZ78]
OG_02084	LC55x_184	LC55x_184; tetratricopeptide repeat family protein	OG_02084	EYR66125.1	hypothetical protein AZ78_22415 [Lysobacter capsici AZ78]
OG_02085	LC55x_5661	LC55x_5661; putative integron gene cassette domain protein	OG_02085	EYR66295.1	hypothetical protein AZ78_22255 [Lysobacter capsici AZ78]
OG_02086	LC55x_1068	modB; molybdate ABC transporter, permease protein	OG_02086	EYR67721.1	molybdate ABC transporter permease [Lysobacter capsici AZ78]
OG_02087	LC55x_219	LC55x_219; conserved hypothetical protein	OG_02087	EYR66160.1	membrane protein [Lysobacter capsici AZ78]
OG_02088	LC55x_2576	LC55x_2576; BON domain protein	OG_02088	EYR68317.1	hypothetical protein AZ78_11670 [Lysobacter capsici AZ78]
OG_02089	LC55x_515	LC55x_515; transglycosylase SLT domain protein	OG_02089	EYR66432.1	lytic murein transglycosylase [Lysobacter capsici AZ78]
OG_02090	LC55x_4320	LC55x_4320; prepilin-type N-terminal cleavage/methylation domain p	OG_02090	EYR69167.1	general secretion pathway protein J [Lysobacter capsici AZ78]
OG_02091	LC55x_2961	LC55x_2961; hypothetical protein	OG_02091	EYR66202.1	hypothetical protein AZ78_21775 [Lysobacter capsici AZ78]
OG_02092	LC55x_4413	trpG; anthranilate synthase component II	OG_02092	EYR69241.1	anthranilate synthase subunit II [Lysobacter capsici AZ78]
OG_02093	LC55x_574	LC55x_574; amidohydrolase family protein	OG_02093	EYR70024.1	amidohydrolase [Lysobacter capsici AZ78]
OG_02094	LC55x_2288	LC55x_2288; ribonuclease, Rne/Rng family domain protein	OG_02094	EYR69669.1	ribonuclease E [Lysobacter capsici AZ78]
OG_02095	LC55x_477	LC55x_477; conserved hypothetical protein	OG_02095	EYR66463.1	hypothetical protein AZ78_20985 [Lysobacter capsici AZ78]
OG_02096	LC55x_3564	LC55x_3564; hypothetical protein	OG_02096	EYR67514.1	hypothetical protein AZ78_15320 [Lysobacter capsici AZ78]
OG_02097	LC55x_3358	parC; DNA topoisomerase IV, A subunit	OG_02097	EYR66538.1	DNA topoisomerase IV subunit A [Lysobacter capsici AZ78]
OG_02098	LC55x_2265	LC55x_2265; bacterial regulatory helix-turn-helix , lysR family protein	OG_02098	EYR69686.1	LysR family transcriptional regulator [Lysobacter capsici AZ78]
OG_02099	LC55x_2670	LC55x_2670; HNH/ENDO VII supernuclease with conserved GHE res	OG_02099	EYR68229.1	hypothetical protein AZ78_11225 [Lysobacter capsici AZ78]
OG_02100	LC55x_1069	modA; molybdate ABC transporter, periplasmic molybdate-binding pro	OG_02100	EYR67720.1	molybdenum ABC transporter substrate-binding protein [Lysobacter c
OG_02101	LC55x_5120	LC55x_5120; flagellin N-methylase family protein	OG_02101	EYR68036.1	proteinase inhibitor [Lysobacter capsici AZ78]
OG_02102	LC55x_1725	yajC; preprotein translocase, YajC subunit	OG_02102	EYR70375.1	preprotein translocase subunit YajC [Lysobacter capsici AZ78]
OG_02103	LC55x_5156	acs; acetate--CoA ligase	OG_02103	EYR68009.1	acetyl-CoA synthetase [Lysobacter capsici AZ78]
OG_02104	LC55x_4631	LC55x_4631; conserved hypothetical protein	OG_02104	EYR68334.1	hypothetical protein AZ78_09490 [Lysobacter capsici AZ78]
OG_02105	LC55x_1852	lpxB; lipid-A-disaccharide synthase	OG_02105	EYR66044.1	lipid-A-disaccharide synthase [Lysobacter capsici AZ78]
OG_02106	LC55x_4145	LC55x_4145; response regulator	OG_02106	EYR66336.1	pilus response regulator PilG [Lysobacter capsici AZ78]
OG_02108	LC55x_1922	nth; endonuclease III	OG_02108	EYR65977.1	endonuclease III [Lysobacter capsici AZ78]
OG_02109	LC55x_1029	rpmE; ribosomal protein L31	OG_02109	EYR67753.1	50S ribosomal protein L31 [Lysobacter capsici AZ78]
OG_02110	LC55x_4241	LC55x_4241; ompA family protein	OG_02110	EYR69250.1	hypothetical protein AZ78_06300 [Lysobacter capsici AZ78]
OG_02111	LC55x_2560	LC55x_2560; conserved hypothetical family protein	OG_02111	EYR65539.1	hypothetical protein AZ78_25800 [Lysobacter capsici AZ78]
OG_02112	LC55x_3022	LC55x_3022; conserved hypothetical protein	OG_02112	EYR67818.1	hypothetical protein AZ78_13575 [Lysobacter capsici AZ78]
OG_02113	LC55x_3986	moaB; molybdenum cofactor biosynthesis protein B	OG_02113	EYR66716.1	molybdopterin biosynthesis protein B [Lysobacter capsici AZ78]
OG_02114	LC55x_1413	LC55x_1413; tonB dependent receptor family protein	OG_02114	EYR67158.1	hypothetical protein AZ78_17195 [Lysobacter capsici AZ78]
OG_02115	LC55x_3989	LC55x_3989; helix-turn-helix family protein	OG_02115	EYR66719.1	transcriptional regulator [Lysobacter capsici AZ78]
OG_02116	LC55x_4166	recQ; ATP-dependent DNA helicase RecQ	OG_02116	EYR68171.1	ATP-dependent DNA helicase RecQ [Lysobacter capsici AZ78]
OG_02117	LC55x_345	LC55x_345; conserved hypothetical protein	OG_02117	EYR68122.1	hypothetical protein AZ78_11895 [Lysobacter capsici AZ78]
OG_02118	LC55x_3006	nuoK; NADH-quinone oxidoreductase subunit K	OG_02118	EYR67804.1	NADH-quinone oxidoreductase subunit K [Lysobacter capsici AZ78]
OG_02119	LC55x_4207	LC55x_4207; hypothetical protein	OG_02119	EYR65345.1	hypothetical protein AZ78_26915 [Lysobacter capsici AZ78]
OG_02120	LC55x_3261	LC55x_3261; conserved hypothetical protein	OG_02120	EYR70032.1	hypothetical protein AZ78_02630 [Lysobacter capsici AZ78]
OG_02121	LC55x_3421	LC55x_3421; phosphoribosyl transferase domain protein	OG_02121	EYR66507.1	hypoxanthine phosphoribosyltransferase [Lysobacter capsici AZ78]
OG_02122	LC55x_4468	LC55x_4468; hypothetical protein	OG_02122	EYR68434.1	hypothetical protein AZ78_10265 [Lysobacter capsici AZ78]
OG_02123	LC55x_3190	LC55x_3190; eukaryotic-type carbonic anhydrase family protein	OG_02123	EYR66908.1	hypothetical protein AZ78_18215 [Lysobacter capsici AZ78]
OG_02124	LC55x_739	LC55x_739; putative transmembrane protein	OG_02124	EYR67645.1	hypothetical protein AZ78_14840 [Lysobacter capsici AZ78]
OG_02125	LC55x_361	LC55x_361; polysaccharide biosynthesis family protein	OG_02125	EYR68134.1	3-beta hydroxysteroid dehydrogenase [Lysobacter capsici AZ78]

OG_02126	LC55x_3028	trpA; tryptophan synthase, alpha subunit
OG_02127	LC55x_5721	LC55x_5721; oxidoreductase NAD-binding domain protein
OG_02128	LC55x_2461	LC55x_2461; hypothetical protein
OG_02129	LC55x_815	LC55x_815; hypothetical protein
OG_02130	LC55x_2833	LC55x_2833; conserved hypothetical protein
OG_02131	LC55x_1973	rimM; 16S rRNA processing protein RimM
OG_02132	LC55x_358	LC55x_358; alpha/beta hydrolase fold family protein
OG_02133	LC55x_4221	LC55x_4221; bacterial regulatory helix-turn-helix, AraC family protein
OG_02134	LC55x_5062	LC55x_5062; beta-lactamase family protein
OG_02135	LC55x_4527	LC55x_4527; amidohydrolase family protein
OG_02136	LC55x_2865	LC55x_2865; glyoxalase/Bleomycin resistance /Dioxygenase superfa
OG_02137	LC55x_1914	pstA; phosphate ABC transporter, permease protein PstA
OG_02138	LC55x_1881	dapE; succinyl-diaminopimelate desuccinylase
OG_02139	LC55x_4268	LC55x_4268; cell division FtsQ family protein
OG_02140	LC55x_4188	tolB; tol-Pal system beta propeller repeat protein TolB
OG_02141	LC55x_4202	LC55x_4202; DNA-binding regulatory , YebC/PmpR family protein
OG_02142	LC55x_5559	arsC; arsenate reductase
OG_02143	LC55x_2075	LC55x_2075; conserved hypothetical protein
OG_02144	LC55x_1038	LC55x_1038; fimbrial assembly family protein
OG_02145	LC55x_4603	LC55x_4603; conserved hypothetical protein
OG_02146	LC55x_594	LC55x_594; conserved hypothetical protein
OG_02147	LC55x_1560	rfbB; dTDP-glucose 4,6-dehydratase
OG_02148	LC55x_3329	efp; translation elongation factor P
OG_02149	LC55x_2407	metX; homoserine O-acetyltransferase
OG_02150	LC55x_4908	LC55x_4908; snoaL-like domain protein
OG_02151	LC55x_3749	LC55x_3749; transcriptional regulator PadR-like family protein
OG_02152	LC55x_2055	LC55x_2055; conserved hypothetical protein
OG_02153	LC55x_508	LC55x_508; vanZ like family protein
OG_02154	LC55x_331	LC55x_331; tonB dependent receptor family protein
OG_02155	LC55x_562	LC55x_562; conserved hypothetical protein
OG_02156	LC55x_1939	rplU; ribosomal protein L21
OG_02157	LC55x_506	LC55x_506; hypothetical protein
OG_02158	LC55x_4633	LC55x_4633; hypothetical protein
OG_02159	LC55x_700	LC55x_700; aldo/keto reductase family protein
OG_02160	LC55x_233	LC55x_233; flagellar biosynthesis , FliO family protein
OG_02161	LC55x_866	LC55x_866; cytidylyltransferase family protein
OG_02162	LC55x_1815	pip; prolyl aminopeptidase
OG_02163	LC55x_1610	LC55x_1610; cytochrome C1 family protein
OG_02164	LC55x_1388	LC55x_1388; hutD family protein
OG_02165	LC55x_101	recB; exodeoxyribonuclease V, beta subunit
OG_02166	LC55x_1708	LC55x_1708; putative secreted protein
OG_02167	LC55x_1930	LC55x_1930; PBP superfamily domain protein
OG_02168	LC55x_3886	LC55x_3886; conserved hypothetical protein
OG_02169	LC55x_4104	LC55x_4104; repair family protein
OG_02170	LC55x_3676	LC55x_3676; CRS1 / YhbY domain protein
OG_02171	LC55x_4290	thiL; thiamine-monophosphate kinase
OG_02172	LC55x_2240	LC55x_2240; hypothetical protein
OG_02173	LC55x_89	LC55x_89; hypothetical protein
OG_02174	LC55x_1113	LC55x_1113; citrate transporter family protein
OG_02175	LC55x_2893	LC55x_2893; conserved hypothetical protein
OG_02176	LC55x_241	LC55x_241; conserved hypothetical protein
OG_02177	LC55x_5154	LC55x_5154; bacterial regulatory, luxR family protein
OG_02178	LC55x_4334	LC55x_4334; putative permease YjgP/YjgQ family protein
OG_02179	LC55x_742	LC55x_742; sigma-70, region 4 family protein
OG_02180	LC55x_2960	LC55x_2960; hypothetical protein
OG_02181	LC55x_1740	LC55x_1740; 6-O-methylguanine DNA methyltransferase, DNA bindir
OG_02182	LC55x_2669	wspR; response regulator
OG_02183	LC55x_1112	LC55x_1112; rieske [2Fe-2S] domain protein
OG_02184	LC55x_2418	LC55x_2418; conserved hypothetical protein
OG_02185	LC55x_4623	LC55x_4623; asnC family protein
OG_02186	LC55x_873	LC55x_873; putative secreted protein
OG_02187	LC55x_2782	LC55x_2782; short chain dehydrogenase family protein
OG_02188	LC55x_126	LC55x_126; conserved hypothetical protein
OG_02189	LC55x_2950	scpB; segregation and condensation protein B
OG_02190	LC55x_1233	LC55x_1233; glycosyl transferase 2 family protein
OG_02191	LC55x_1784	LC55x_1784; conserved hypothetical protein
OG_02192	LC55x_1187	LC55x_1187; SPFH domain / Band 7 family protein
OG_02193	LC55x_330	LC55x_330; ABC-2 transporter family protein
OG_02194	LC55x_2044	panC; pantoate--beta-alanine ligase
OG_02195	LC55x_4431	LC55x_4431; NAD dependent epimerase/dehydratase family protein
OG_02196	LC55x_1362	LC55x_1362; phosphotransferase enzyme family protein
OG_02197	LC55x_4750	LC55x_4750; transglutaminase-like superfamily protein
OG_02198	LC55x_1302	LC55x_1302; conserved hypothetical protein
OG_02199	LC55x_4706	LC55x_4706; response regulator
OG_02200	LC55x_5741	trmE; tRNA modification GTPase TrmE
OG_02201	LC55x_837	LC55x_837; conserved hypothetical protein
OG_02202	LC55x_1006	LC55x_1006; pilZ domain protein
OG_02203	LC55x_3696	eno; phosphopyruvate hydratase
OG_02204	LC55x_462	LC55x_462; peptidase M23 family protein
OG_02205	LC55x_1733	LC55x_1733; ABC transporter, phosphonate, periplasmic substrate-b
OG_02206	LC55x_5665	LC55x_5665; efflux transporter, RND family, MFP subunit

OG_02126	EYR65369.1	tryptophan synthase subunit alpha [Lysobacter capsici AZ78]
OG_02127	EYR69691.1	phenol hydroxylase [Lysobacter capsici AZ78]
OG_02128	EYR69816.1	hypothetical protein AZ78_03425 [Lysobacter capsici AZ78]
OG_02129	EYR67687.1	hypothetical protein AZ78_14480 [Lysobacter capsici AZ78]
OG_02130	EYR69394.1	hypothetical protein AZ78_05735 [Lysobacter capsici AZ78]
OG_02131	EYR66772.1	16S rRNA-processing protein RimM [Lysobacter capsici AZ78]
OG_02132	EYR68132.1	alpha/beta hydrolase [Lysobacter capsici AZ78]
OG_02133	EYR69082.1	AraC family transcriptional regulator [Lysobacter capsici AZ78]
OG_02134	EYR68072.1	beta-lactamase [Lysobacter capsici AZ78]
OG_02135	EYR68580.1	hypothetical protein AZ78_09995 [Lysobacter capsici AZ78]
OG_02136	EYR69423.1	glyoxalase [Lysobacter capsici AZ78]
OG_02137	EYR65982.1	phosphate transporter permease subunit PtsA [Lysobacter capsici AZ
OG_02138	EYR66073.1	succinyl-diaminopimelate desuccinylase [Lysobacter capsici AZ78]
OG_02139	EYR69126.1	cell division protein FtsQ [Lysobacter capsici AZ78]
OG_02140	EYR68159.1	translocation protein TolB [Lysobacter capsici AZ78]
OG_02141	EYR68148.1	transcriptional regulator [Lysobacter capsici AZ78]
OG_02142	EYR68987.1	arsenate reductase [Lysobacter capsici AZ78]
OG_02143	EYR65360.1	hypothetical protein AZ78_26815 [Lysobacter capsici AZ78]
OG_02144	EYR67746.1	fimbrial protein [Lysobacter capsici AZ78]
OG_02145	EYR68354.1	hypothetical protein AZ78_09610 [Lysobacter capsici AZ78]
OG_02146	EYR70009.1	hypothetical protein AZ78_02495 [Lysobacter capsici AZ78]
OG_02147	EYR70280.1	dTDP-glucose 4,6-dehydratase [Lysobacter capsici AZ78]
OG_02148	EYR70080.1	elongation factor P [Lysobacter capsici AZ78]
OG_02149	EYR69589.1	homoserine O-acetyltransferase [Lysobacter capsici AZ78]
OG_02150	EYR68706.1	hypothetical protein AZ78_08805 [Lysobacter capsici AZ78]
OG_02151	EYR67061.1	PadR family transcriptional regulator [Lysobacter capsici AZ78]
OG_02152	EYR65606.1	hypothetical protein AZ78_25475 [Lysobacter capsici AZ78]
OG_02153	EYR66436.1	membrane protein [Lysobacter capsici AZ78]
OG_02154	EYR68110.1	TonB-denpendent receptor [Lysobacter capsici AZ78]
OG_02155	EYR67919.1	hypothetical protein AZ78_13000 [Lysobacter capsici AZ78]
OG_02156	EYR65968.1	50S ribosomal protein L21 [Lysobacter capsici AZ78]
OG_02157	EYR66438.1	hypothetical protein AZ78_20835 [Lysobacter capsici AZ78]
OG_02158	EYR68543.1	hypothetical protein AZ78_09480 [Lysobacter capsici AZ78]
OG_02159	EYR69943.1	aldo/keto reductase [Lysobacter capsici AZ78]
OG_02160	EYR66172.1	hypothetical protein AZ78_22650 [Lysobacter capsici AZ78]
OG_02161	EYR66891.1	phosphatidate cytidylyltransferase [Lysobacter capsici AZ78]
OG_02162	EYR67291.1	proline iminopeptidase [Lysobacter capsici AZ78]
OG_02163	EYR70316.1	cytochrome C [Lysobacter capsici AZ78]
OG_02164	EYR67180.1	HutD-family protein [Lysobacter capsici AZ78]
OG_02165	EYR69764.1	exodeoxyribonuclease V subunit beta [Lysobacter capsici AZ78]
OG_02166	EYR70366.1	hypothetical protein AZ78_01465 [Lysobacter capsici AZ78]
OG_02167	EYR66014.1	hypothetical protein AZ78_23375 [Lysobacter capsici AZ78]
OG_02168	EYR65719.1	hypothetical protein AZ78_24805 [Lysobacter capsici AZ78]
OG_02169	EYR65505.1	hypothetical protein AZ78_25965 [Lysobacter capsici AZ78]
OG_02170	EYR65941.1	RNA-binding protein [Lysobacter capsici AZ78]
OG_02171	EYR69144.1	thiamine monophosphate kinase [Lysobacter capsici AZ78]
OG_02172	EYR67432.1	hypothetical protein AZ78_16015 [Lysobacter capsici AZ78]
OG_02173	EYR69878.1	hypothetical protein AZ78_04850 [Lysobacter capsici AZ78]
OG_02174	EYR68476.1	sulfur deprivation response regulator [Lysobacter capsici AZ78]
OG_02175	EYR69449.1	RNA signal recognition particle 4.5S RNA [Lysobacter capsici AZ78]
OG_02176	EYR66178.1	hypothetical protein AZ78_22680 [Lysobacter capsici AZ78]
OG_02177	EYR68011.1	LuxR family transcriptional regulator [Lysobacter capsici AZ78]
OG_02178	EYR69178.1	membrane protein [Lysobacter capsici AZ78]
OG_02179	EYR67642.1	RNA polymerase sigma70 [Lysobacter capsici AZ78]
OG_02180	EYR66203.1	hypothetical protein AZ78_21780 [Lysobacter capsici AZ78]
OG_02181	EYR70388.1	methylated-DNA--protein-cysteine methyltransferase [Lysobacter cap
OG_02182	EYR68230.1	diguanylate cyclase [Lysobacter capsici AZ78]
OG_02183	EYR68475.1	ferredoxin [Lysobacter capsici AZ78]
OG_02184	EYR69824.1	hypothetical protein AZ78_03640 [Lysobacter capsici AZ78]
OG_02185	EYR68339.1	AsnC family transcriptional regulator [Lysobacter capsici AZ78]
OG_02186	EYR66884.1	hypothetical protein AZ78_19070 [Lysobacter capsici AZ78]
OG_02187	EYR69346.1	short-chain dehydrogenase [Lysobacter capsici AZ78]
OG_02188	EYR69779.1	hypothetical protein AZ78_05010 [Lysobacter capsici AZ78]
OG_02189	EYR66211.1	hypothetical protein AZ78_21825 [Lysobacter capsici AZ78]
OG_02190	EYR65384.1	glycosyl transferase family 2 [Lysobacter capsici AZ78]
OG_02191	EYR67280.1	membrane protein [Lysobacter capsici AZ78]
OG_02192	EYR65796.1	membrane protein [Lysobacter capsici AZ78]
OG_02193	EYR68176.1	hypothetical protein AZ78_11820 [Lysobacter capsici AZ78]
OG_02194	EYR65598.1	pantoate--beta-alanine ligase [Lysobacter capsici AZ78]
OG_02195	EYR65472.1	nucleoside-diphosphate sugar epimerase [Lysobacter capsici AZ78]
OG_02196	EYR67203.1	aminoglycoside phosphotransferase [Lysobacter capsici AZ78]
OG_02197	EYR66096.1	transglutaminase [Lysobacter capsici AZ78]
OG_02198	EYR69930.1	membrane protein [Lysobacter capsici AZ78]
OG_02199	EYR65668.1	transcriptional regulator [Lysobacter capsici AZ78]
OG_02200	EYR69702.1	tRNA modification GTPase TrmE [Lysobacter capsici AZ78]
OG_02201	EYR67576.1	membrane protein [Lysobacter capsici AZ78]
OG_02202	EYR67770.1	pilZ family protein [Lysobacter capsici AZ78]
OG_02203	EYR65923.1	enolase [Lysobacter capsici AZ78]
OG_02204	EYR66474.1	peptidase M23 [Lysobacter capsici AZ78]
OG_02205	EYR70381.1	anion binding protein [Lysobacter capsici AZ78]
OG_02206	EYR65890.1	hypothetical protein AZ78_24140 [Lysobacter capsici AZ78]

OG_02207	LC55x_817	LC55x_817; conserved hypothetical protein
OG_02208	LC55x_3786	LC55x_3786; transcription elongation factor, GreA/GreB, C-term fami
OG_02209	LC55x_1017	rpoZ; DNA-directed RNA polymerase, omega subunit
OG_02210	LC55x_2440	LC55x_2440; conserved hypothetical protein
OG_02211	LC55x_780	atpG; ATP synthase F1, gamma subunit
OG_02212	LC55x_3263	LC55x_3263; bacteriocin-protection, YdeI/OmpD-Associated family p
OG_02213	LC55x_109	LC55x_109; efflux transporter, outer membrane factor (OMF) lipo, No
OG_02214	LC55x_4723	LC55x_4723; alkylhydroperoxidase AhpD family core domain protein
OG_02215	LC55x_3447	acpP; acyl carrier protein
OG_02216	LC55x_3337	phaR; polyhydroxyalkanoate synthesis repressor PhaR
OG_02217	LC55x_4439	LC55x_4439; H+ symporter) family protein
OG_02218	LC55x_323	LC55x_323; outer membrane beta-barrel domain protein
OG_02219	LC55x_478	LC55x_478; conserved hypothetical protein
OG_02220	LC55x_4903	LC55x_4903; bacterial regulatory, lacI family protein
OG_02221	LC55x_5048	LC55x_5048; conserved hypothetical protein
OG_02222	LC55x_4240	clpB; ATP-dependent chaperone protein ClpB
OG_02223	LC55x_134	LC55x_134; catalase family protein
OG_02224	LC55x_806	LC55x_806; putative transcriptional regulator
OG_02225	LC55x_3730	LC55x_3730; peptidase M16 inactive domain protein
OG_02226	LC55x_2073	LC55x_2073; conserved hypothetical protein
OG_02227	LC55x_2043	panB; 3-methyl-2-oxobutanoate hydroxymethyltransferase
OG_02228	LC55x_2934	zwf; glucose-6-phosphate dehydrogenase
OG_02229	LC55x_3026	glmM; phosphoglucosamine mutase
OG_02230	LC55x_375	aceB; malate synthase A
OG_02231	LC55x_946	LC55x_946; conserved hypothetical protein
OG_02232	LC55x_3204	LC55x_3204; hypothetical protein
OG_02233	LC55x_4089	LC55x_4089; histone deacetylase domain protein
OG_02234	LC55x_2963	LC55x_2963; putative peptidoglycan binding domain protein
OG_02235	LC55x_2446	mmsA; methylmalonate-semialdehyde dehydrogenase
OG_02236	LC55x_229	epaO; type III secretion apparatus protein, YscQ/HrcQ family
OG_02237	LC55x_1184	LC55x_1184; PDZ domain family protein
OG_02238	LC55x_2150	LC55x_2150; hypothetical protein
OG_02239	LC55x_4540	LC55x_4540; 2Fe-2S iron-sulfur cluster binding domain protein
OG_02240	LC55x_4050	LC55x_4050; nitroreductase family protein
OG_02241	LC55x_1555	LC55x_1555; thioesterase domain protein
OG_02242	LC55x_981	rubA; rubredoxin
OG_02243	LC55x_2173	LC55x_2173; succinate dehydrogenase and fumarate reductase iron-
OG_02244	LC55x_3534	mfd; transcription-repair coupling factor
OG_02245	LC55x_4255	LC55x_4255; bacterial regulatory , Fis family protein
OG_02246	LC55x_1779	LC55x_1779; glucose / Sorbosone dehydrogenase family protein
OG_02247	LC55x_341	LC55x_341; conserved hypothetical protein
OG_02248	LC55x_1311	mgtE; magnesium transporter
OG_02249	LC55x_3031	LC55x_3031; N-(5'phosphoribosyl)anthranilate (PRA) isomerase fami
OG_02250	LC55x_982	LC55x_982; conserved hypothetical protein
OG_02251	LC55x_4688	dapF; diaminopimelate epimerase
OG_02252	LC55x_486	LC55x_486; SURF1 family protein
OG_02253	LC55x_4508	LC55x_4508; endoglucanase
OG_02254	LC55x_3428	LC55x_3428; response regulator
OG_02255	LC55x_3206	feoB; ferrous iron transport protein B
OG_02256	LC55x_1230	LC55x_1230; conserved hypothetical protein
OG_02257	LC55x_2530	trxB; thioredoxin-disulfide reductase
OG_02258	LC55x_5651	LC55x_5651; conserved hypothetical protein
OG_02259	LC55x_4681	LC55x_4681; conserved hypothetical protein
OG_02260	LC55x_2291	LC55x_2291; tonB family C-terminal domain protein
OG_02261	LC55x_1178	LC55x_1178; aldo/keto reductase family protein
OG_02262	LC55x_5654	LC55x_5654; endonuclease/Exonuclease/phosphatase family protein
OG_02263	LC55x_347	LC55x_347; PLD-like domain protein
OG_02264	LC55x_3546	LC55x_3546; MOSC domain protein
OG_02265	LC55x_1106	LC55x_1106; RNA 2'-O ribose methyltransferase substrate binding fa
OG_02266	LC55x_2112	LC55x_2112; hypothetical protein
OG_02267	LC55x_4269	ddl; D-alanine--D-alanine ligase
OG_02268	LC55x_3809	LC55x_3809; bacterial regulatory helix-turn-helix , lysR family protein
OG_02269	LC55x_5121	LC55x_5121; conserved hypothetical protein
OG_02270	LC55x_1246	LC55x_1246; alpha/beta hydrolase fold family protein
OG_02271	LC55x_1404	LC55x_1404; feS assembly SUF system regulator
OG_02272	LC55x_5007	LC55x_5007; patatin-like phospholipase family protein
OG_02273	LC55x_3366	LC55x_3366; autotransporter beta-domain protein
OG_02274	LC55x_2246	acrA1; oxidoreductase, short-chain dehydrogenase/reductase family
OG_02275	LC55x_2180	lolD; liporeleasing system, ATP-binding protein
OG_02276	LC55x_2704	LC55x_2704; pfkB carbohydrate kinase family protein
OG_02277	LC55x_3005	LC55x_3005; proton-translocating NADH-quinone oxidoreductase, ch
OG_02278	LC55x_5480	LC55x_5480; acetyltransferase family protein
OG_02279	LC55x_5130	LC55x_5130; aldehyde dehydrogenase family protein
OG_02280	LC55x_5605	LC55x_5605; cupin domain protein
OG_02281	LC55x_2033	LC55x_2033; AAA domain protein
OG_02282	LC55x_83	LC55x_83; metA-pathway of phenol degradation family protein
OG_02283	LC55x_1014	yicC; conserved protein
OG_02285	LC55x_874	LC55x_874; conserved hypothetical protein
OG_02286	LC55x_3283	LC55x_3283; methyltransferase domain protein
OG_02287	LC55x_4834	LC55x_4834; metallo-beta-lactamase superfamily protein
OG_02288	LC55x_3332	LC55x_3332; diguanylate cyclase domain protein

OG_02207	EYR67685.1	hypothetical protein AZ78_14470 [Lysobacter capsici AZ78]
OG_02208	EYR67093.1	hypothetical protein AZ78_17685 [Lysobacter capsici AZ78]
OG_02209	EYR67762.1	DNA-directed RNA polymerase subunit omega [Lysobacter capsici A
OG_02210	EYR69821.1	hypothetical protein AZ78_03520 [Lysobacter capsici AZ78]
OG_02211	EYR67610.1	F0F1 ATP synthase subunit gamma [Lysobacter capsici AZ78]
OG_02212	EYR70034.1	hypothetical protein AZ78_02640 [Lysobacter capsici AZ78]
OG_02213	EYR69769.1	RND transporter [Lysobacter capsici AZ78]
OG_02214	EYR65685.1	hypothetical protein AZ78_25120 [Lysobacter capsici AZ78]
OG_02215	EYR66594.1	acyl carrier protein [Lysobacter capsici AZ78]
OG_02216	EYR66552.1	polyhydroxyalkanoate synthesis repressor PhaR [Lysobacter capsici
OG_02217	EYR68457.1	dihydroorotate dehydrogenase [Lysobacter capsici AZ78]
OG_02218	EYR68106.1	hypothetical protein AZ78_11790 [Lysobacter capsici AZ78]
OG_02219	EYR66462.1	hypothetical protein AZ78_20980 [Lysobacter capsici AZ78]
OG_02220	EYR68710.1	transcriptional regulator [Lysobacter capsici AZ78]
OG_02221	EYR65576.1	hypothetical protein AZ78_25505 [Lysobacter capsici AZ78]
OG_02222	EYR69097.1	protein disaggregation chaperone [Lysobacter capsici AZ78]
OG_02223	EYR69785.1	catalase [Lysobacter capsici AZ78]
OG_02224	EYR67590.1	TetR family transcriptional regulator [Lysobacter capsici AZ78]
OG_02225	EYR65895.1	hypothetical protein AZ78_23590 [Lysobacter capsici AZ78]
OG_02226	EYR65395.1	membrane protein [Lysobacter capsici AZ78]
OG_02227	EYR65597.1	3-methyl-2-oxobutanoate hydroxymethyltransferase [Lysobacter caps
OG_02228	EYR66226.1	glucose-6-phosphate 1-dehydrogenase [Lysobacter capsici AZ78]
OG_02229	EYR65367.1	phosphoglucosamine mutase [Lysobacter capsici AZ78]
OG_02230	EYR68145.1	malate synthase [Lysobacter capsici AZ78]
OG_02231	EYR66816.1	hypothetical protein AZ78_18730 [Lysobacter capsici AZ78]
OG_02232	EYR67984.1	hypothetical protein AZ78_13330 [Lysobacter capsici AZ78]
OG_02233	EYR65441.1	acetoin utilization protein [Lysobacter capsici AZ78]
OG_02234	EYR66200.1	hypothetical protein AZ78_21765 [Lysobacter capsici AZ78]
OG_02235	EYR69557.1	methylmalonate-semialdehyde dehydrogenase [Lysobacter capsici A
OG_02236	EYR66168.1	hypothetical protein AZ78_22630 [Lysobacter capsici AZ78]
OG_02237	EYR65799.1	hypothetical protein AZ78_24580 [Lysobacter capsici AZ78]
OG_02238	EYR67451.1	hypothetical protein AZ78_15600 [Lysobacter capsici AZ78]
OG_02239	EYR68384.1	oxidoreductase [Lysobacter capsici AZ78]
OG_02240	EYR65829.1	nitroreductase [Lysobacter capsici AZ78]
OG_02241	EYR70277.1	protein PvdG [Lysobacter capsici AZ78]
OG_02242	EYR65388.1	rubredoxin [Lysobacter capsici AZ78]
OG_02243	EYR67391.1	succinate dehydrogenase iron-sulfur subunit [Lysobacter capsici AZ7
OG_02244	EYR67486.1	transcription-repair coupling factor [Lysobacter capsici AZ78]
OG_02245	EYR69110.1	chemotaxis protein CheY [Lysobacter capsici AZ78]
OG_02246	EYR67276.1	sorbosone dehydrogenase [Lysobacter capsici AZ78]
OG_02247	EYR68119.1	hypothetical protein AZ78_11875 [Lysobacter capsici AZ78]
OG_02248	EYR69923.1	magnesium transporter [Lysobacter capsici AZ78]
OG_02249	EYR67822.1	N-(5'-phosphoribosyl)anthranilate isomerase [Lysobacter capsici AZ7
OG_02250	EYR65387.1	ACR family protein [Lysobacter capsici AZ78]
OG_02251	EYR70186.1	diaminopimelate epimerase [Lysobacter capsici AZ78]
OG_02252	EYR66454.1	membrane protein [Lysobacter capsici AZ78]
OG_02253	EYR68409.1	beta-glucanase [Lysobacter capsici AZ78]
OG_02254	EYR66502.1	transcriptional regulator [Lysobacter capsici AZ78]
OG_02255	EYR67982.1	iron transporter FeoB [Lysobacter capsici AZ78]
OG_02256	EYR65386.1	hypothetical protein AZ78_26615 [Lysobacter capsici AZ78]
OG_02257	EYR69494.1	thioredoxin reductase [Lysobacter capsici AZ78]
OG_02258	EYR66288.1	hypothetical protein AZ78_22220 [Lysobacter capsici AZ78]
OG_02259	EYR70179.1	membrane protein [Lysobacter capsici AZ78]
OG_02260	EYR69859.1	hypothetical protein AZ78_04535 [Lysobacter capsici AZ78]
OG_02261	EYR65804.1	NADP-dependent aryl-alcohol dehydrogenase [Lysobacter capsici AZ
OG_02262	EYR66290.1	hypothetical protein AZ78_22230 [Lysobacter capsici AZ78]
OG_02263	EYR68124.1	nuclease [Lysobacter capsici AZ78]
OG_02264	EYR67498.1	molybdenum cofactor sulfurase [Lysobacter capsici AZ78]
OG_02265	EYR68469.1	rRNA methyltransferase [Lysobacter capsici AZ78]
OG_02266	EYR66394.1	hypothetical protein AZ78_21275 [Lysobacter capsici AZ78]
OG_02267	EYR69127.1	D-alanine--D-alanine ligase [Lysobacter capsici AZ78]
OG_02268	EYR67114.1	LysR family transcriptional regulator [Lysobacter capsici AZ78]
OG_02269	EYR68035.1	hypothetical protein AZ78_12565 [Lysobacter capsici AZ78]
OG_02270	EYR65640.1	homoserine O-acetyltransferase [Lysobacter capsici AZ78]
OG_02271	EYR67164.1	Rrf2 family transcriptional regulator [Lysobacter capsici AZ78]
OG_02272	EYR67223.1	hypothetical protein AZ78_16765 [Lysobacter capsici AZ78]
OG_02273	EYR66578.1	hypothetical protein AZ78_20590, partial [Lysobacter capsici AZ78]
OG_02274	EYR67435.1	short-chain dehydrogenase [Lysobacter capsici AZ78]
OG_02275	EYR67397.1	ABC transporter ATP-binding protein [Lysobacter capsici AZ78]
OG_02276	EYR69290.1	hypothetical protein AZ78_05190 [Lysobacter capsici AZ78]
OG_02277	EYR67803.1	NADH:ubiquinone oxidoreductase subunit L [Lysobacter capsici AZ7
OG_02278	EYR68938.1	N-acetyltransferase GCN5 [Lysobacter capsici AZ78]
OG_02279	EYR68027.1	aldehyde dehydrogenase [Lysobacter capsici AZ78]
OG_02280	EYR66245.1	pirin [Lysobacter capsici AZ78]
OG_02281	EYR65589.1	PhoH family protein [Lysobacter capsici AZ78]
OG_02282	EYR69752.1	hypothetical protein AZ78_04820 [Lysobacter capsici AZ78]
OG_02283	EYR67764.1	hypothetical protein AZ78_14200 [Lysobacter capsici AZ78]
OG_02285	EYR66883.1	hypothetical protein AZ78_19065 [Lysobacter capsici AZ78]
OG_02286	EYR70139.1	hypothetical protein AZ78_02740 [Lysobacter capsici AZ78]
OG_02287	EYR68760.1	beta-lactamase [Lysobacter capsici AZ78]
OG_02288	EYR70082.1	membrane protein [Lysobacter capsici AZ78]

OG_02289	LC55x_1370	cysC; adenylylsulfate kinase	OG_02289	EYR67194.1	adenylylsulfate kinase [Lysobacter capsici AZ78]
OG_02290	LC55x_2335	LC55x_2335; helix-turn-helix family protein	OG_02290	EYR69633.1	membrane protein [Lysobacter capsici AZ78]
OG_02291	LC55x_1820	lon; ATP-dependent protease La	OG_02291	EYR67307.1	peptidase [Lysobacter capsici AZ78]
OG_02292	LC55x_4761	LC55x_4761; aldehyde dehydrogenase family protein	OG_02292	EYR66086.1	ketoglutarate semialdehyde dehydrogenase [Lysobacter capsici AZ7
OG_02293	LC55x_910	LC55x_910; yceI-like domain protein	OG_02293	EYR66849.1	polyisoprenoid-binding protein [Lysobacter capsici AZ78]
OG_02294	LC55x_3942	rplX; ribosomal protein L24	OG_02294	EYR66693.1	50S ribosomal protein L24 [Lysobacter capsici AZ78]
OG_02295	LC55x_3543	LC55x_3543; methyltransferase domain protein	OG_02295	EYR67495.1	hypothetical protein AZ78_15225 [Lysobacter capsici AZ78]
OG_02296	LC55x_4511	gnd; 6-phosphogluconate dehydrogenase	OG_02296	EYR68407.1	6-phosphogluconate dehydrogenase [Lysobacter capsici AZ78]
OG_02297	LC55x_577	LC55x_577; conserved hypothetical protein	OG_02297	EYR70021.1	hypothetical protein AZ78_02570 [Lysobacter capsici AZ78]
OG_02298	LC55x_4331	xerD; tyrosine recombinase XerD	OG_02298	EYR69175.1	tyrosine recombinase XerD [Lysobacter capsici AZ78]
OG_02299	LC55x_2564	LC55x_2564; phosphotransferase enzyme family protein	OG_02299	EYR68329.1	aminoglycoside phosphotransferase [Lysobacter capsici AZ78]
OG_02300	LC55x_4349	LC55x_4349; conserved hypothetical protein	OG_02300	EYR69189.1	membrane protein [Lysobacter capsici AZ78]
OG_02301	LC55x_3712	ycaQ; conserved protein	OG_02301	EYR65910.1	hypothetical protein AZ78_23665 [Lysobacter capsici AZ78]
OG_02302	LC55x_5037	LC55x_5037; ycgL domain protein	OG_02302	EYR66302.1	membrane protein [Lysobacter capsici AZ78]
OG_02303	LC55x_2474	dnaJ; chaperone protein DnaJ	OG_02303	EYR69537.1	molecular chaperone DnaJ [Lysobacter capsici AZ78]
OG_02304	LC55x_56	LC55x_56; transcriptional regulatory , C terminal family protein	OG_02304	EYR69734.1	transcriptional regulator [Lysobacter capsici AZ78]
OG_02305	LC55x_4923	LC55x_4923; lysM domain protein	OG_02305	EYR68693.1	peptidoglycan-binding protein LysM [Lysobacter capsici AZ78]
OG_02306	LC55x_3433	LC55x_3433; conserved hypothetical protein	OG_02306	EYR66498.1	hypothetical protein AZ78_20285 [Lysobacter capsici AZ78]
OG_02307	LC55x_597	LC55x_597; phosphate-selective porin O and P family protein	OG_02307	EYR70006.1	porin [Lysobacter capsici AZ78]
OG_02308	LC55x_333	LC55x_333; pyridoxal-phosphate dependent enzyme family protein	OG_02308	EYR68111.1	cysteine synthase [Lysobacter capsici AZ78]
OG_02309	LC55x_5117	aroE; shikimate 5-dehydrogenase	OG_02309	EYR68038.1	shikimate 5-dehydrogenase [Lysobacter capsici AZ78]
OG_02310	LC55x_4088	LC55x_4088; ATP:cob(I)alamin adenosyltransferase, putative	OG_02310	EYR65440.1	ATP--cobalamin adenosyltransferase [Lysobacter capsici AZ78]
OG_02311	LC55x_319	LC55x_319; conserved hypothetical protein	OG_02311	EYR68102.1	hypothetical protein AZ78_11770 [Lysobacter capsici AZ78]
OG_02312	LC55x_4744	ohr; organic hydroperoxide resistance protein	OG_02312	EYR66102.1	Organic hydroperoxide resistance protein [Lysobacter capsici AZ78]
OG_02313	LC55x_2746	LC55x_2746; FAD binding domain protein	OG_02313	EYR69316.1	glycine/D-amino acid oxidase [Lysobacter capsici AZ78]
OG_02314	LC55x_1942	rpsT; ribosomal protein S20	OG_02314	EYR65965.1	30S ribosomal protein S20 [Lysobacter capsici AZ78]
OG_02315	LC55x_1013	rph; ribonuclease PH	OG_02315	EYR67765.1	ribonuclease PH [Lysobacter capsici AZ78]
OG_02316	LC55x_2263	serS; serine--tRNA ligase	OG_02316	EYR67449.1	seryl-tRNA synthetase [Lysobacter capsici AZ78]
OG_02317	LC55x_1224	LC55x_1224; conserved hypothetical protein	OG_02317	EYR65757.1	hypothetical protein AZ78_24355 [Lysobacter capsici AZ78]
OG_02318	LC55x_4668	mreB; mreB	OG_02318	EYR70168.1	rod shape-determining protein Mbl [Lysobacter capsici AZ78]
OG_02319	LC55x_3721	LC55x_3721; conserved hypothetical protein	OG_02319	EYR65903.1	hypothetical protein AZ78_23630 [Lysobacter capsici AZ78]
OG_02320	LC55x_2249	LC55x_2249; patatin-like phospholipase family protein	OG_02320	EYR67437.1	lectin subunit Beta [Lysobacter capsici AZ78]
OG_02321	LC55x_909	LC55x_909; nitroreductase family protein	OG_02321	EYR66850.1	malonic semialdehyde reductase [Lysobacter capsici AZ78]
OG_02322	LC55x_1040	LC55x_1040; pilus assembly , PilP family protein	OG_02322	EYR67744.1	pilus assembly protein PilP [Lysobacter capsici AZ78]
OG_02323	LC55x_2274	LC55x_2274; bacterial regulatory helix-turn-helix , lysR family protein	OG_02323	EYR69679.1	LysR family transcriptional regulator [Lysobacter capsici AZ78]
OG_02324	LC55x_4535	LC55x_4535; asnC family protein	OG_02324	EYR68389.1	ArsR family transcriptional regulator [Lysobacter capsici AZ78]
OG_02325	LC55x_2067	LC55x_2067; RDD family protein	OG_02325	EYR65401.1	hypothetical protein AZ78_26495 [Lysobacter capsici AZ78]
OG_02326	LC55x_117	LC55x_117; hlyD secretion family protein	OG_02326	EYR69883.1	hypothetical protein AZ78_04980 [Lysobacter capsici AZ78]
OG_02327	LC55x_4140	LC55x_4140; cheB methylesterase family protein	OG_02327	EYR66341.1	hypothetical protein AZ78_21560 [Lysobacter capsici AZ78]
OG_02328	LC55x_4239	LC55x_4239; amidohydrolase family protein	OG_02328	EYR69249.1	hypothetical protein AZ78_06290 [Lysobacter capsici AZ78]
OG_02329	LC55x_4700	LC55x_4700; molydopterin dinucleotide binding domain protein	OG_02329	EYR65662.1	formate dehydrogenase [Lysobacter capsici AZ78]
OG_02330	LC55x_1721	LC55x_1721; bacterial regulatory, luxR family protein	OG_02330	EYR70372.1	transcriptional regulator [Lysobacter capsici AZ78]
OG_02331	LC55x_3020	LC55x_3020; DNA-deoxyinosine glycosylase	OG_02331	EYR67817.1	DNA glycosylase [Lysobacter capsici AZ78]
OG_02332	LC55x_85	LC55x_85; rhomboid family protein	OG_02332	EYR69753.1	membrane protein [Lysobacter capsici AZ78]
OG_02333	LC55x_2822	LC55x_2822; methyltransferase domain protein	OG_02333	EYR69383.1	ArsR family transcriptional regulator [Lysobacter capsici AZ78]
OG_02334	LC55x_57	LC55x_57; his Kinase A domain protein	OG_02334	EYR69735.1	histidine kinase [Lysobacter capsici AZ78]
OG_02335	LC55x_1011	LC55x_1011; glyoxalase/Bleomycin resistance /Dioxygenase superfa	OG_02335	EYR67766.1	hypothetical protein AZ78_14210 [Lysobacter capsici AZ78]
OG_02336	LC55x_2261	LC55x_2261; tonB family C-terminal domain protein	OG_02336	EYR67447.1	energry transducer TonB [Lysobacter capsici AZ78]
OG_02337	LC55x_1262	LC55x_1262; L-serine ammonia-lyase	OG_02337	EYR65651.1	serine dehydratase [Lysobacter capsici AZ78]
OG_02338	LC55x_1203	LC55x_1203; tat (twin-arginine translocation) pathway signal sequen	OG_02338	EYR65782.1	hypothetical protein AZ78_24490 [Lysobacter capsici AZ78]
OG_02339	LC55x_1975	rplS; ribosomal protein L19	OG_02339	EYR66769.1	50S ribosomal protein L19 [Lysobacter capsici AZ78]
OG_02340	LC55x_3775	LC55x_3775; tfoX N-terminal domain protein	OG_02340	EYR67085.1	hypothetical protein AZ78_17645 [Lysobacter capsici AZ78]
OG_02341	LC55x_5744	rpmH; ribosomal protein L34	OG_02341	EYR69705.1	50S ribosomal protein L34 [Lysobacter capsici AZ78]
OG_02342	LC55x_3113	LC55x_3113; endoribonuclease L-PSP family protein	OG_02342	EYR66980.1	aminoacrylate peracid reductase [Lysobacter capsici AZ78]
OG_02343	LC55x_5714	LC55x_5714; hypothetical protein	OG_02343	EYR65844.1	hypothetical protein AZ78_23905 [Lysobacter capsici AZ78]
OG_02344	LC55x_5535	LC55x_5535; glutathione S-transferase	OG_02344	EYR68967.1	glutathione S-transferase [Lysobacter capsici AZ78]
OG_02345	LC55x_1684	LC55x_1684; hypothetical protein	OG_02345	EYR70452.1	hypothetical protein AZ78_01365 [Lysobacter capsici AZ78]
OG_02346	LC55x_915	lpxL; lipid A biosynthesis lauroyl (or palmitoleoyl) acyltransferase fami	OG_02346	EYR66844.1	lauroyl acyltransferase [Lysobacter capsici AZ78]
OG_02347	LC55x_4369	LC55x_4369; electron transfer flavodomain protein	OG_02347	EYR69203.1	electron transfer flavoprotein subunit beta [Lysobacter capsici AZ78]
OG_02348	LC55x_1585	ybeY; conserved protein involved in translation	OG_02348	EYR70301.1	rRNA maturation factor [Lysobacter capsici AZ78]
OG_02349	LC55x_3568	LC55x_3568; SPFH domain / Band 7 family protein	OG_02349	EYR67517.1	membrane protein [Lysobacter capsici AZ78]
OG_02350	LC55x_2651	LC55x_2651; glycosyl transferases group 1 family protein	OG_02350	EYR68247.1	hypothetical protein AZ78_11315 [Lysobacter capsici AZ78]
OG_02351	LC55x_1948	ispH; 4-hydroxy-3-methylbut-2-enyl diphosphate reductase	OG_02351	EYR65960.1	4-hydroxy-3-methylbut-2-enyl diphosphate reductase [Lysobacter ca
OG_02352	LC55x_314	LC55x_314; hydrolase CocE/NonD family protein	OG_02352	EYR67044.1	X-Pro dipeptidyl-peptidase [Lysobacter capsici AZ78]
OG_02353	LC55x_2434	LC55x_2434; impB/mucB/samB family protein	OG_02353	EYR69566.1	DNA repair nucleotidyltransferase [Lysobacter capsici AZ78]
OG_02354	LC55x_2118	LC55x_2118; conserved hypothetical protein	OG_02354	EYR66388.1	acetyltransferase [Lysobacter capsici AZ78]
OG_02355	LC55x_2119	LC55x_2119; pseudouridine synthase family protein	OG_02355	EYR66387.1	RNA pseudouridine synthase [Lysobacter capsici AZ78]
OG_02356	LC55x_4224	cobT; nicotinate-nucleotide--dimethylbenzimidazole phosphoribosyltra	OG_02356	EYR69085.1	nicotinate-nucleotide--dimethylbenzimidazole phosphoribosyltransfer
OG_02357	LC55x_3751	LC55x_3751; spermidine n1-acetyltransferase	OG_02357	EYR67063.1	spermidine N1-acetyltransferase [Lysobacter capsici AZ78]
OG_02358	LC55x_4517	LC55x_4517; aldo/keto reductase family protein	OG_02358	EYR68404.1	oxidoreductase [Lysobacter capsici AZ78]
OG_02359	LC55x_4444	pdhA; pyruvate dehydrogenase (acetyl-transferring) E1 component, a	OG_02359	EYR68453.1	ABC transporter permease [Lysobacter capsici AZ78]
OG_02360	LC55x_3565	purT; phosphoribosylglycinamide formyltransferase 2	OG_02360	EYR67515.1	phosphoribosylglycinamide formyltransferase [Lysobacter capsici AZ
OG_02361	LC55x_1177	LC55x_1177; hypothetical protein	OG_02361	EYR65805.1	hypothetical protein AZ78_24610 [Lysobacter capsici AZ78]
OG_02362	LC55x_2840	LC55x_2840; ABC transporter family protein	OG_02362	EYR69401.1	multidrug ABC transporter ATP-binding protein [Lysobacter capsici A
OG_02363	LC55x_2413	LC55x_2413; ccmE family protein	OG_02363	EYR69584.1	cytochrome C biogenesis protein CcmC [Lysobacter capsici AZ78]
OG_02364	LC55x_3596	LC55x_3596; triose-phosphate Transporter family protein	OG_02364	EYR67540.1	multidrug DMT transporter [Lysobacter capsici AZ78]
OG_02365	LC55x_1002	LC55x_1002; conserved hypothetical protein	OG_02365	EYR67772.1	short-chain dehydrogenase [Lysobacter capsici AZ78]
OG_02366	LC55x_4563	LC55x_4563; peptidase M20/M25/M40 family protein	OG_02366	EYR68377.1	peptidase M20 [Lysobacter capsici AZ78]
OG_02367	LC55x_3437	LC55x_3437; anti sigma-E RseA, N-terminal domain protein	OG_02367	EYR66494.1	regulatory protein [Lysobacter capsici AZ78]
OG_02368	LC55x_932	LC55x_932; pyridoxal-phosphate dependent enzyme family protein	OG_02368	EYR66829.1	threonine dehydratase [Lysobacter capsici AZ78]
OG_02369	LC55x_1221	LC55x_1221; phosphoenolpyruvate carboxykinase family protein	OG_02369	EYR65760.1	phosphoenolpyruvate carboxykinase [Lysobacter capsici AZ78]

OG_02370	LC55x_1834	LC55x_1834; conserved hypothetical protein
OG_02371	LC55x_698	LC55x_698; lactonase, 7-bladed beta-propeller family protein
OG_02372	LC55x_225	LC55x_225; type III secretion , YscU/HrpY family protein
OG_02373	LC55x_4362	LC55x_4362; ATP-grasp domain protein
OG_02374	LC55x_685	LC55x_685; excalibur calcium-binding domain protein
OG_02375	LC55x_2392	LC55x_2392; transmembrane domain protein
OG_02376	LC55x_2331	LC55x_2331; bacterial regulatory, tetR family protein
OG_02377	LC55x_1360	LC55x_1360; RNA 2'-phosphotransferase, Tpt1 / KptA family protein
OG_02378	LC55x_62	LC55x_62; RNA polymerase sigma factor, sigma-70 family protein
OG_02379	LC55x_5241	LC55x_5241; methyltransferase domain protein
OG_02380	LC55x_3266	hisF; imidazole glycerol phosphate synthase, HisF subunit
OG_02381	LC55x_4647	LC55x_4647; isochorismatase family protein
OG_02382	LC55x_4344	pssA; CDP-diacylglycerol-serine O-phosphatidyltransferase
OG_02383	LC55x_3525	LC55x_3525; conserved hypothetical protein
OG_02384	LC55x_5095	LC55x_5095; putative pteridine-dependent dioxygenase
OG_02385	LC55x_2940	LC55x_2940; bacterial extracellular solute-binding family protein
OG_02386	LC55x_725	LC55x_725; bacterial regulatory, tetR family protein
OG_02387	LC55x_3338	phbB; acetoacetyl-CoA reductase family protein
OG_02389	LC55x_3341	rnd; ribonuclease D
OG_02390	LC55x_2500	lysS; lysine--tRNA ligase
OG_02391	LC55x_4214	LC55x_4214; ABC transporter family protein
OG_02392	LC55x_3600	LC55x_3600; conserved hypothetical protein
OG_02393	LC55x_2665	LC55x_2665; cheW-like domain protein
OG_02394	LC55x_2334	pilF; type IV pilus biogenesis/stability protein PilW
OG_02395	LC55x_5252	LC55x_5252; C4-dicarboxylate anaerobic carrier family protein
OG_02396	LC55x_4189	LC55x_4189; hypothetical protein
OG_02397	LC55x_2380	LC55x_2380; citrate transporter family protein
OG_02398	LC55x_4707	LC55x_4707; histidine kinase family protein
OG_02399	LC55x_1318	LC55x_1318; putative P-loop containing ATPase
OG_02400	LC55x_4127	mazG; nucleoside triphosphate pyrophosphohydrolase
OG_02401	LC55x_1667	rplT; ribosomal protein L20
OG_02402	LC55x_950	LC55x_950; bacterial regulatory helix-turn-helix, AraC family protein
OG_02403	LC55x_979	hemL; glutamate-1-semialdehyde-2,1-aminomutase
OG_02404	LC55x_4009	lieA; leupeptin-inactivating enzyme 1
OG_02405	LC55x_4074	LC55x_4074; eamA-like transporter family protein
OG_02406	LC55x_2089	LC55x_2089; conserved hypothetical protein
OG_02407	LC55x_781	atpD; ATP synthase F1, beta subunit
OG_02408	LC55x_2385	LC55x_2385; bacterial regulatory, luxR family protein
OG_02409	LC55x_190	LC55x_190; acyltransferase family protein
OG_02410	LC55x_3834	LC55x_3834; tonB dependent receptor family protein
OG_02411	LC55x_827	queD; queuosine biosynthesis protein QueD
OG_02412	LC55x_3467	LC55x_3467; hypothetical protein
OG_02413	LC55x_349	LC55x_349; prenyltransferase-like family protein
OG_02414	LC55x_1323	rpoN; RNA polymerase sigma-54 factor
OG_02415	LC55x_4151	LC55x_4151; glyoxalase-like domain protein
OG_02416	LC55x_931	leuA; 2-isopropylmalate synthase
OG_02417	LC55x_1832	cafA; ribonuclease G
OG_02419	LC55x_374	LC55x_374; lysR substrate binding domain protein
OG_02420	LC55x_4462	LC55x_4462; sporulation related domain protein
OG_02421	LC55x_5236	gltD; glutamate synthase (NADPH) small chain glutamate synthase, s
OG_02422	LC55x_429	argS; arginine--tRNA ligase
OG_02423	LC55x_4258	LC55x_4258; type II secretion system (T2SS), F family protein
OG_02424	LC55x_3205	LC55x_3205; hypothetical protein
OG_02425	LC55x_499	yihY; yihY family inner membrane domain protein
OG_02426	LC55x_3007	LC55x_3007; NADH-ubiquinone/plastoquinone oxidoreductase chain
OG_02427	LC55x_2810	LC55x_2810; conserved hypothetical protein
OG_02428	LC55x_1153	ahcY; adenosylhomocysteinase
OG_02429	LC55x_3003	LC55x_3003; proton-translocating NADH-quinone oxidoreductase, ch
OG_02430	LC55x_64	LC55x_64; bacterial regulatory, luxR family protein
OG_02431	LC55x_3137	LC55x_3137; conserved hypothetical protein
OG_02432	LC55x_4186	ygbF; tol-pal system protein YbgF
OG_02433	LC55x_4724	LC55x_4724; leucine carboxyl methyltransferase family protein
OG_02434	LC55x_222	LC55x_222; hypothetical protein
OG_02435	LC55x_3324	LC55x_3324; conserved hypothetical protein
OG_02436	LC55x_4325	LC55x_4325; type II secretion system (T2SS), F family protein
OG_02437	LC55x_5701	LC55x_5701; methyltransferase domain protein
OG_02438	LC55x_5162	LC55x_5162; hypothetical protein
OG_02439	LC55x_479	LC55x_479; delta-1-pyrroline-5-carboxylate dehydrogenase
OG_02440	LC55x_4294	ribB; 3,4-dihydroxy-2-butanone-4-phosphate synthase
OG_02441	LC55x_3464	LC55x_3464; outer membrane lipoSlp family protein
OG_02442	LC55x_4150	LC55x_4150; putative peptidase
OG_02443	LC55x_29	LC55x_29; thermolysin metallopeptidase, catalytic domain protein
OG_02444	LC55x_575	LC55x_575; EDD, DegV family domain protein
OG_02445	LC55x_4620	LC55x_4620; eamA-like transporter family protein
OG_02446	LC55x_322	gpsA; gpsA
OG_02447	LC55x_5621	LC55x_5621; NADPH-dependent FMN reductase family protein
OG_02448	LC55x_713	LC55x_713; major Facilitator Superfamily protein
OG_02449	LC55x_4472	LC55x_4472; conserved hypothetical protein
OG_02450	LC55x_4384	LC55x_4384; glycosyl transferases group 1 family protein
OG_02451	LC55x_3913	LC55x_3913; cyclophilin type peptidyl-prolyl cis-trans isomerase/CLD
OG_02452	LC55x_3612	LC55x_3612; glyoxalase/Bleomycin resistance /Dioxygenase superfa

OG_02370	EYR66026.1	hypothetical protein AZ78_22985 [Lysobacter capsici AZ78]
OG_02371	EYR69944.1	3-carboxymuconate cyclase [Lysobacter capsici AZ78]
OG_02372	EYR66164.1	hypothetical protein AZ78_22610 [Lysobacter capsici AZ78]
OG_02373	EYR69199.1	hypothetical protein AZ78_06935 [Lysobacter capsici AZ78]
OG_02374	EYR69953.1	calcium-binding protein [Lysobacter capsici AZ78]
OG_02375	EYR69596.1	hypothetical protein AZ78_03765 [Lysobacter capsici AZ78]
OG_02376	EYR69637.1	TetR family transcriptional regulator [Lysobacter capsici AZ78]
OG_02377	EYR67205.1	RNA 2'-phosphotransferase [Lysobacter capsici AZ78]
OG_02378	EYR69739.1	RNA polymerase subunit sigma24 [Lysobacter capsici AZ78]
OG_02379	EYR68897.1	malonyl-CoA O-methyltransferase [Lysobacter capsici AZ78]
OG_02380	EYR70037.1	imidazole glycerol phosphate synthase [Lysobacter capsici AZ78]
OG_02381	EYR70158.1	cysteine hydrolase [Lysobacter capsici AZ78]
OG_02382	EYR69185.1	CDP-diacylglycerol--serine O-phosphatidyltransferase [Lysobacter ca
OG_02383	EYR67476.1	protein involved in catabolism of external DNA [Lysobacter capsici AZ
OG_02384	EYR68053.1	pteridine-dependent deoxygenase [Lysobacter capsici AZ78]
OG_02385	EYR66220.1	ABC transporter substrate-binding protein [Lysobacter capsici AZ78]
OG_02386	EYR67654.1	TetR family transcriptional regulator [Lysobacter capsici AZ78]
OG_02387	EYR66551.1	3-ketoacyl-ACP reductase [Lysobacter capsici AZ78]
OG_02389	EYR66548.1	ribonuclease D [Lysobacter capsici AZ78]
OG_02390	EYR69518.1	lysyl-tRNA synthetase [Lysobacter capsici AZ78]
OG_02391	EYR69077.1	glutathione ABC transporter ATP-binding protein [Lysobacter capsici
OG_02392	EYR67543.1	hypothetical protein AZ78_15465 [Lysobacter capsici AZ78]
OG_02393	EYR68233.1	hypothetical protein AZ78_11245 [Lysobacter capsici AZ78]
OG_02394	EYR69634.1	fimbrial protein [Lysobacter capsici AZ78]
OG_02395	EYR68903.1	short chain fatty acid transporter [Lysobacter capsici AZ78]
OG_02396	EYR68188.1	hypothetical protein AZ78_12125 [Lysobacter capsici AZ78]
OG_02397	EYR69605.1	transporter [Lysobacter capsici AZ78]
OG_02398	EYR65669.1	histidine kinase [Lysobacter capsici AZ78]
OG_02399	EYR69917.1	glmZ(sRNA)-inactivating NTPase [Lysobacter capsici AZ78]
OG_02400	EYR66353.1	nucleoside triphosphate pyrophosphohydrolase [Lysobacter capsici A
OG_02401	EYR70339.1	50S ribosomal protein L20 [Lysobacter capsici AZ78]
OG_02402	EYR66812.1	AraC family transcriptional regulator [Lysobacter capsici AZ78]
OG_02403	EYR65390.1	glutamate-1-semialdehyde aminotransferase [Lysobacter capsici AZ7
OG_02404	EYR65564.1	hypothetical protein AZ78_25615 [Lysobacter capsici AZ78]
OG_02405	EYR65428.1	hypothetical protein AZ78_26290 [Lysobacter capsici AZ78]
OG_02406	EYR66415.1	RNA helicase [Lysobacter capsici AZ78]
OG_02407	EYR67609.1	F0F1 ATP synthase subunit beta [Lysobacter capsici AZ78]
OG_02408	EYR69602.1	LuxR family transcriptional regulator [Lysobacter capsici AZ78]
OG_02409	EYR66130.1	acetyltransferase [Lysobacter capsici AZ78]
OG_02410	EYR65745.1	ligand-gated channel [Lysobacter capsici AZ78]
OG_02411	EYR67582.1	6-carboxy-5,6,7,8-tetrahydropterin synthase [Lysobacter capsici AZ7
OG_02412	EYR66610.1	hypothetical protein AZ78_20010 [Lysobacter capsici AZ78]
OG_02413	EYR68179.1	hypothetical protein AZ78_11915 [Lysobacter capsici AZ78]
OG_02414	EYR69913.1	RNA polymerase sigma54 factor [Lysobacter capsici AZ78]
OG_02415	EYR66330.1	hypothetical protein AZ78_21505 [Lysobacter capsici AZ78]
OG_02416	EYR66830.1	2-isopropylmalate synthase [Lysobacter capsici AZ78]
OG_02417	EYR66025.1	ribonuclease G [Lysobacter capsici AZ78]
OG_02419	EYR68144.1	LysR family transcriptional regulator [Lysobacter capsici AZ78]
OG_02420	EYR68438.1	sporulation protein [Lysobacter capsici AZ78]
OG_02421	EYR68893.1	glutamate synthase subunit beta [Lysobacter capsici AZ78]
OG_02422	EYR65515.1	arginyl-tRNA synthetase [Lysobacter capsici AZ78]
OG_02423	EYR69116.1	type II secretion system protein F [Lysobacter capsici AZ78]
OG_02424	EYR67983.1	hypothetical protein AZ78_13325 [Lysobacter capsici AZ78]
OG_02425	EYR66444.1	BrkB protein [Lysobacter capsici AZ78]
OG_02426	EYR67805.1	NADH:ubiquinone oxidoreductase subunit J [Lysobacter capsici AZ7
OG_02427	EYR69372.1	hypothetical protein AZ78_05620 [Lysobacter capsici AZ78]
OG_02428	EYR68523.1	S-adenosyl-L-homocysteine hydrolase [Lysobacter capsici AZ78]
OG_02429	EYR67801.1	NADH:ubiquinone oxidoreductase subunit N [Lysobacter capsici AZ7
OG_02430	EYR69741.1	LuxR family transcriptional regulator [Lysobacter capsici AZ78]
OG_02431	EYR66958.1	hypothetical protein AZ78_18470 [Lysobacter capsici AZ78]
OG_02432	EYR68161.1	hypothetical protein AZ78_12140 [Lysobacter capsici AZ78]
OG_02433	EYR66120.1	hypothetical protein AZ78_22950 [Lysobacter capsici AZ78]
OG_02434	EYR66162.1	hypothetical protein AZ78_22600 [Lysobacter capsici AZ78]
OG_02435	EYR70076.1	phytoene synthase [Lysobacter capsici AZ78]
OG_02436	EYR69171.1	general secretion pathway protein F [Lysobacter capsici AZ78]
OG_02437	EYR65857.1	hypothetical protein AZ78_23975 [Lysobacter capsici AZ78]
OG_02438	EYR68077.1	hypothetical protein AZ78_12365 [Lysobacter capsici AZ78]
OG_02439	EYR66461.1	pyrroline-5-carboxylate dehydrogenase [Lysobacter capsici AZ78]
OG_02440	EYR69148.1	3,4-dihydroxy-2-butanone 4-phosphate synthase [Lysobacter capsici
OG_02441	EYR66607.1	membrane protein [Lysobacter capsici AZ78]
OG_02442	EYR66331.1	glycoprotease [Lysobacter capsici AZ78]
OG_02443	EYR69726.1	peptidase M4 [Lysobacter capsici AZ78]
OG_02444	EYR70023.1	hypothetical protein AZ78_02580 [Lysobacter capsici AZ78]
OG_02445	EYR68341.1	multidrug transporter [Lysobacter capsici AZ78]
OG_02446	EYR68105.1	NAD(P)H-dependent glycerol-3-phosphate dehydrogenase [Lysobac
OG_02447	EYR66261.1	NAD(P)H dehydrogenase [Lysobacter capsici AZ78]
OG_02448	EYR69938.1	MFS transporter [Lysobacter capsici AZ78]
OG_02449	EYR68430.1	hypothetical protein AZ78_10245 [Lysobacter capsici AZ78]
OG_02450	EYR69218.1	glycosyl transferase [Lysobacter capsici AZ78]
OG_02451	EYR66666.1	peptidylprolyl isomerase [Lysobacter capsici AZ78]
OG_02452	EYR67554.1	lactoylglutathione lyase [Lysobacter capsici AZ78]

OG_02453	LC55x_3383	LC55x_3383; eamA-like transporter family protein	OG_02453	EYR66587.1	membrane protein [Lysobacter capsici AZ78]
OG_02454	LC55x_3167	LC55x_3167; hypothetical protein	OG_02454	EYR66934.1	hypothetical protein AZ78_18350 [Lysobacter capsici AZ78]
OG_02455	LC55x_4990	LC55x_4990; conserved hypothetical protein	OG_02455	EYR67238.1	vanillate O-demethylase oxidoreductase VanB [Lysobacter capsici AZ
OG_02456	LC55x_2325	csaA; secretion chaperone	OG_02456	EYR69640.1	tRNA-binding protein [Lysobacter capsici AZ78]
OG_02457	LC55x_3083	LC55x_3083; acetyltransferase family protein	OG_02457	EYR67859.1	acetyltransferase [Lysobacter capsici AZ78]
OG_02458	LC55x_23	LC55x_23; bacterial regulatory helix-turn-helix , lysR family protein	OG_02458	EYR69722.1	LysR family transcriptional regulator [Lysobacter capsici AZ78]
OG_02459	LC55x_4473	LC55x_4473; amidinotransferase family protein	OG_02459	EYR68429.1	amidinotransferase [Lysobacter capsici AZ78]
OG_02460	LC55x_3684	LC55x_3684; lysM domain protein	OG_02460	EYR65934.1	membrane protein [Lysobacter capsici AZ78]
OG_02461	LC55x_2745	LC55x_2745; conserved hypothetical protein	OG_02461	EYR69315.1	hypothetical protein AZ78_05330 [Lysobacter capsici AZ78]
OG_02462	LC55x_2519	clpS; ATP-dependent Clp protease adapter protein ClpS	OG_02462	EYR69499.1	ATP-dependent Clp protease ClpS [Lysobacter capsici AZ78]
OG_02463	LC55x_4108	LC55x_4108; lemA family protein	OG_02463	EYR65510.1	LemA family protein [Lysobacter capsici AZ78]
OG_02464	LC55x_2011	LC55x_2011; competence/damage-inducible CinA C-terminal domain	OG_02464	EYR66746.1	competence damage-inducible protein A [Lysobacter capsici AZ78]
OG_02465	LC55x_1908	LC55x_1908; conserved hypothetical protein	OG_02465	EYR65987.1	hypothetical protein AZ78_23455 [Lysobacter capsici AZ78]
OG_02466	LC55x_5242	bioH; pimelyl-[acyl-carrier protein] methyl ester esterase	OG_02466	EYR68898.1	pimelyl-ACP methyl ester esterase [Lysobacter capsici AZ78]
OG_02467	LC55x_4643	LC55x_4643; conserved hypothetical protein	OG_02467	EYR70391.1	hypothetical protein AZ78_00035 [Lysobacter capsici AZ78]
OG_02468	LC55x_2171	sdhD; succinate dehydrogenase, hydrophobic membrane anchor prot	OG_02468	EYR67389.1	succinate dehydrogenase [Lysobacter capsici AZ78]
OG_02469	LC55x_522	LC55x_522; endonuclease/Exonuclease/phosphatase family protein	OG_02469	EYR66428.1	endonuclease [Lysobacter capsici AZ78]
OG_02470	LC55x_848	LC55x_848; mechanosensitive ion channel family protein	OG_02470	EYR67567.1	membrane protein [Lysobacter capsici AZ78]
OG_02471	LC55x_1010	rdgB; non-canonical purine NTP pyrophosphatase, RdgB/HAM1 famil	OG_02471	EYR67767.1	nucleoside-triphosphate diphosphatase [Lysobacter capsici AZ78]
OG_02472	LC55x_1739	LC55x_1739; hypothetical protein	OG_02472	EYR70387.1	hypothetical protein AZ78_01605 [Lysobacter capsici AZ78]
OG_02473	LC55x_2862	purB; adenylosuccinate lyase	OG_02473	EYR69421.1	adenylosuccinate lyase [Lysobacter capsici AZ78]
OG_02474	LC55x_1925	LC55x_1925; FKBP-type peptidyl-prolyl cis-trans isomerase family pr	OG_02474	EYR65974.1	peptidylprolyl isomerase [Lysobacter capsici AZ78]
OG_02475	LC55x_2653	LC55x_2653; bacterial transferase hexapeptide family protein	OG_02475	EYR68245.1	hypothetical protein AZ78_11305 [Lysobacter capsici AZ78]
OG_02476	LC55x_5649	LC55x_5649; ion channel family protein	OG_02476	EYR66286.1	potassium channel protein [Lysobacter capsici AZ78]
OG_02477	LC55x_207	LC55x_207; conserved hypothetical family protein	OG_02477	EYR66147.1	hypothetical protein AZ78_22525 [Lysobacter capsici AZ78]
OG_02478	LC55x_487	LC55x_487; conserved hypothetical protein	OG_02478	EYR66453.1	hypothetical protein AZ78_20930 [Lysobacter capsici AZ78]
OG_02479	LC55x_5002	LC55x_5002; TPR repeat family protein	OG_02479	EYR67227.1	hypothetical protein AZ78_16785 [Lysobacter capsici AZ78]
OG_02480	LC55x_1825	LC55x_1825; PPIC-type PPIASE domain protein	OG_02480	EYR67309.1	peptidylprolyl isomerase [Lysobacter capsici AZ78]
OG_02481	LC55x_2897	LC55x_2897; yceI-like domain protein	OG_02481	EYR69453.1	polyisoprenoid-binding protein [Lysobacter capsici AZ78]
OG_02482	LC55x_4927	LC55x_4927; pilin family protein	OG_02482	EYR68690.1	PilA [Lysobacter capsici AZ78]
OG_02483	LC55x_4676	LC55x_4676; short chain dehydrogenase family protein	OG_02483	EYR70174.1	3-ketoacyl-ACP reductase [Lysobacter capsici AZ78]
OG_02484	LC55x_3033	fimV; fimV N-terminal domain	OG_02484	EYR67824.1	fimV protein [Lysobacter capsici AZ78]
OG_02485	LC55x_3037	aroC; chorismate synthase	OG_02485	EYR67827.1	chorismate synthase [Lysobacter capsici AZ78]
OG_02486	LC55x_3096	LC55x_3096; PLD-like domain protein	OG_02486	EYR65443.1	phospholipase D [Lysobacter capsici AZ78]
OG_02487	LC55x_2910	gyrA; DNA gyrase, A subunit	OG_02487	EYR69466.1	DNA gyrase subunit A [Lysobacter capsici AZ78]
OG_02488	LC55x_3614	gcvP; glycine dehydrogenase	OG_02488	EYR67555.1	glycine dehydrogenase [Lysobacter capsici AZ78]
OG_02489	LC55x_711	LC55x_711; condensation domain protein	OG_02489	EYR70097.1	hypothetical protein AZ78_01950 [Lysobacter capsici AZ78]
OG_02490	LC55x_4079	LC55x_4079; cytidine and deoxycytidylate deaminase zinc-binding re	OG_02490	EYR65431.1	nucleoside deaminase [Lysobacter capsici AZ78]
OG_02491	LC55x_3508	phoB; phosphate regulon transcriptional regulatory protein PhoB	OG_02491	EYR66634.1	PhoB family transcriptional regulator [Lysobacter capsici AZ78]
OG_02492	LC55x_3849	folC; FOLC	OG_02492	EYR65729.1	folylpolyglutamate synthase [Lysobacter capsici AZ78]
OG_02493	LC55x_2872	LC55x_2872; putative lysine decarboxylase family protein	OG_02493	EYR69431.1	lysine decarboxylase [Lysobacter capsici AZ78]
OG_02494	LC55x_228	epaP; type III secretion apparatus protein, YscR/HrcR family	OG_02494	EYR66167.1	hypothetical protein AZ78_22625 [Lysobacter capsici AZ78]
OG_02495	LC55x_5470	LC55x_5470; lumazine-binding family protein	OG_02495	EYR68936.1	hypothetical protein AZ78_07925 [Lysobacter capsici AZ78]
OG_02496	LC55x_1572	corA; magnesium and cobalt transport protein CorA	OG_02496	EYR70291.1	magnesium transporter CorA [Lysobacter capsici AZ78]
OG_02497	LC55x_2877	LC55x_2877; dnaJ C terminal domain protein	OG_02497	EYR69436.1	cytochrome C biogenesis protein [Lysobacter capsici AZ78]
OG_02499	LC55x_4495	LC55x_4495; conserved hypothetical protein	OG_02499	EYR68415.1	hypothetical protein AZ78_10145 [Lysobacter capsici AZ78]
OG_02500	LC55x_4232	LC55x_4232; putative membrane protein	OG_02500	EYR69093.1	membrane protein [Lysobacter capsici AZ78]
OG_02501	LC55x_4683	hslU; ATP-dependent protease HslVU, ATPase subunit	OG_02501	EYR70181.1	ATP-dependent protease [Lysobacter capsici AZ78]
OG_02502	LC55x_4313	LC55x_4313; glycosyl transferase 2 family protein	OG_02502	EYR69160.1	glycosyl transferase [Lysobacter capsici AZ78]
OG_02503	LC55x_3941	LC55x_3941; ribosomal L5 family protein	OG_02503	EYR66692.1	50S ribosomal protein L5 [Lysobacter capsici AZ78]
OG_02504	LC55x_5209	LC55x_5209; HAD super, subIIIB family protein	OG_02504	EYR68874.1	acid phosphatase [Lysobacter capsici AZ78]
OG_02505	LC55x_2031	thiD; phosphomethylpyrimidine kinase	OG_02505	EYR65588.1	phosphomethylpyrimidine kinase [Lysobacter capsici AZ78]
OG_02506	LC55x_3852	serA; phosphoglycerate dehydrogenase	OG_02506	EYR65556.1	hypothetical protein AZ78_25750 [Lysobacter capsici AZ78]
OG_02507	LC55x_2937	LC55x_2937; F5/8 type C domain protein	OG_02507	EYR66223.1	hypothetical protein AZ78_21885 [Lysobacter capsici AZ78]
OG_02508	LC55x_2479	carA; carbamoyl-phosphate synthase, small subunit	OG_02508	EYR69534.1	carbamoyl phosphate synthase small subunit [Lysobacter capsici AZ7
OG_02509	LC55x_4154	mrcB; penicillin-binding protein 1B	OG_02509	EYR66327.1	penicillin-binding protein 1B [Lysobacter capsici AZ78]
OG_02510	LC55x_964	LC55x_964; putative secreted protein	OG_02510	EYR66801.1	hypothetical protein AZ78_18650 [Lysobacter capsici AZ78]
OG_02511	LC55x_4763	LC55x_4763; FAD binding domain protein	OG_02511	EYR66084.1	oxidoreductase [Lysobacter capsici AZ78]
OG_02512	LC55x_5071	LC55x_5071; conserved hypothetical protein	OG_02512	EYR68067.1	hypothetical protein AZ78_12780 [Lysobacter capsici AZ78]
OG_02513	LC55x_783	LC55x_783; gtrA-like family protein	OG_02513	EYR67607.1	membrane protein [Lysobacter capsici AZ78]
OG_02514	LC55x_3041	LC55x_3041; hypothetical protein	OG_02514	EYR67874.1	hypothetical protein AZ78_13640 [Lysobacter capsici AZ78]
OG_02515	LC55x_3768	LC55x_3768; conserved hypothetical protein	OG_02515	EYR67077.1	hypothetical protein AZ78_17600 [Lysobacter capsici AZ78]
OG_02516	LC55x_1295	LC55x_1295; twitching motility family protein	OG_02516	EYR67659.1	twitching motility protein PilT [Lysobacter capsici AZ78]
OG_02517	LC55x_3469	dnaX; DNA polymerase III, subunit gamma and tau	OG_02517	EYR66612.1	DNA polymerase III subunit gamma/tau [Lysobacter capsici AZ78]
OG_02518	LC55x_3960	rpoB; DNA-directed RNA polymerase, beta subunit	OG_02518	EYR65487.1	DNA-directed RNA polymerase subunit beta [Lysobacter capsici AZ7
OG_02519	LC55x_5103	LC55x_5103; rrf2 family protein	OG_02519	EYR68047.1	hypothetical protein AZ78_12660 [Lysobacter capsici AZ78]
OG_02520	LC55x_4696	LC55x_4696; patatin-like phospholipase family protein	OG_02520	EYR65658.1	hypothetical protein AZ78_24985 [Lysobacter capsici AZ78]
OG_02521	LC55x_5182	LC55x_5182; hypothetical protein	OG_02521	EYR68990.1	hypothetical protein AZ78_07260 [Lysobacter capsici AZ78]
OG_02522	LC55x_4071	MSRA; peptide methionine sulfoxide reductase	OG_02522	EYR65377.1	methionine sulfoxide reductase A [Lysobacter capsici AZ78]
OG_02523	LC55x_5647	LC55x_5647; conserved hypothetical protein	OG_02523	EYR66284.1	membrane protein [Lysobacter capsici AZ78]
OG_02524	LC55x_1174	LC55x_1174; conserved hypothetical protein	OG_02524	EYR68540.1	membrane protein [Lysobacter capsici AZ78]
OG_02525	LC55x_4387	LC55x_4387; conserved hypothetical protein	OG_02525	EYR69220.1	UptF protein [Lysobacter capsici AZ78]
OG_02526	LC55x_1913	pstB; phosphate ABC transporter, ATP-binding protein	OG_02526	EYR65983.1	phosphate ABC transporter ATP-binding protein [Lysobacter capsici A
OG_02527	LC55x_710	LC55x_710; glycosyl transferase 2 family protein	OG_02527	EYR69939.1	rhamnosyltransferase [Lysobacter capsici AZ78]
OG_02528	LC55x_692	LC55x_692; amino acid permease family protein	OG_02528	EYR69947.1	alanine glycine permease [Lysobacter capsici AZ78]
OG_02529	LC55x_2101	LC55x_2101; bacterial regulatory helix-turn-helix, AraC family protein	OG_02529	EYR66404.1	hypothetical protein AZ78_21325 [Lysobacter capsici AZ78]
OG_02530	LC55x_2445	LC55x_2445; putative membrane protein	OG_02530	EYR69558.1	hypothetical protein AZ78_03500 [Lysobacter capsici AZ78]
OG_02531	LC55x_2780	LC55x_2780; conserved hypothetical protein	OG_02531	EYR69344.1	hypothetical protein AZ78_05480 [Lysobacter capsici AZ78]
OG_02532	LC55x_3246	LC55x_3246; acetyltransferase family protein	OG_02532	EYR67935.1	hypothetical protein AZ78_13080 [Lysobacter capsici AZ78]
OG_02533	LC55x_262	LC55x_262; RNA polymerase sigma factor, sigma-70 family protein	OG_02533	EYR66990.1	RNA polymerase subunit sigma-24 [Lysobacter capsici AZ78]
OG_02534	LC55x_3580	LC55x_3580; histidine kinase-, DNA gyrase B-, and HSP90-like ATPa	OG_02534	EYR67528.1	psensor histidine kinase [Lysobacter capsici AZ78]

OG_02535	LC55x_3943	rplN; ribosomal protein L14	OG_02535	EYR66694.1	50S ribosomal protein L14 [Lysobacter capsici AZ78]
OG_02536	LC55x_690	LC55x_690; ABC transporter family protein	OG_02536	EYR69949.1	multidrug ABC transporter ATP-binding protein [Lysobacter capsici AZ
OG_02537	LC55x_5215	LC55x_5215; uvrD/REP helicase N-terminal domain protein	OG_02537	EYR68878.1	ATP-dependent DNA helicase Rep [Lysobacter capsici AZ78]
OG_02538	LC55x_3454	LC55x_3454; conserved hypothetical protein	OG_02538	EYR66600.1	characterized ACR protein [Lysobacter capsici AZ78]
OG_02539	LC55x_3930	rpsD; ribosomal protein S4	OG_02539	EYR66681.1	30S ribosomal protein S4 [Lysobacter capsici AZ78]
OG_02540	LC55x_513	LC55x_513; conserved hypothetical protein	OG_02540	EYR66433.1	hypothetical protein AZ78_20800 [Lysobacter capsici AZ78]
OG_02541	LC55x_2831	LC55x_2831; conserved hypothetical protein	OG_02541	EYR69393.1	nucleoprotein/polynucleotide-associated enzyme [Lysobacter capsici
OG_02542	LC55x_4120	LC55x_4120; conserved hypothetical protein	OG_02542	EYR66360.1	transmembrane signal peptide protein [Lysobacter capsici AZ78]
OG_02543	LC55x_3952	rplD; 50S ribosomal protein L4	OG_02543	EYR66703.1	50S ribosomal protein L4 [Lysobacter capsici AZ78]
OG_02544	LC55x_4270	murC; UDP-N-acetylmuramate--alanine ligase	OG_02544	EYR69128.1	UDP-N-acetylmuramate--alanine ligase [Lysobacter capsici AZ78]
OG_02545	LC55x_1621	sodB; sodB	OG_02545	EYR70324.1	superoxide dismutase [Lysobacter capsici AZ78]
OG_02546	LC55x_335	LC55x_335; conserved hypothetical protein	OG_02546	EYR68113.1	hypothetical protein AZ78_11845 [Lysobacter capsici AZ78]
OG_02547	LC55x_1216	LC55x_1216; conserved hypothetical protein	OG_02547	EYR65764.1	hypothetical protein AZ78_24390 [Lysobacter capsici AZ78]
OG_02548	LC55x_3773	LC55x_3773; eamA-like transporter family protein	OG_02548	EYR67083.1	drug/metabolite transporter permease [Lysobacter capsici AZ78]
OG_02549	LC55x_3997	LC55x_3997; conserved hypothetical protein	OG_02549	EYR66727.1	membrane protein [Lysobacter capsici AZ78]
OG_02550	LC55x_4402	speD; S-adenosylmethionine decarboxylase proenzyme	OG_02550	EYR69231.1	S-adenosylmethionine decarboxylase [Lysobacter capsici AZ78]
OG_02551	LC55x_4762	LC55x_4762; dihydrodipicolinate synthetase family protein	OG_02551	EYR66085.1	dihydrodipicolinate synthetase [Lysobacter capsici AZ78]
OG_02553	LC55x_4680	LC55x_4680; acetyltransferase family protein	OG_02553	EYR70178.1	histone acetyltransferase HPA2-like acetyltransferase [Lysobacter ca
OG_02554	LC55x_3668	hflK; hflK protein	OG_02554	EYR65948.1	membrane protein [Lysobacter capsici AZ78]
OG_02555	LC55x_1440	murD; UDP-N-acetylmuramoylalanine--D-glutamate ligase	OG_02555	EYR67136.1	UDP-N-acetylmuramoyl-L-alanyl-D-glutamate synthetase [Lysobacte
OG_02556	LC55x_1327	LC55x_1327; phosphatase, YrbI family	OG_02556	EYR69909.1	HAD family hydrolase [Lysobacter capsici AZ78]
OG_02557	LC55x_954	ppa; ppa	OG_02557	EYR66810.1	inorganic pyrophosphatase [Lysobacter capsici AZ78]
OG_02558	LC55x_5003	LC55x_5003; bacterial regulatory, tetR family protein	OG_02558	EYR67226.1	hypothetical protein AZ78_16780 [Lysobacter capsici AZ78]
OG_02559	LC55x_4960	rpoH; alternative sigma factor RpoH	OG_02559	EYR67265.1	RNA polymerase sigma 70 [Lysobacter capsici AZ78]
OG_02560	LC55x_769	aceF; dihydrolipoyllysine-residue acetyltransferase	OG_02560	EYR67621.1	dihydrolipoamide acetyltransferase [Lysobacter capsici AZ78]
OG_02561	LC55x_4226	cobQ; cobyric acid synthase CobQ	OG_02561	EYR69087.1	cobyric acid synthase [Lysobacter capsici AZ78]
OG_02562	LC55x_3074	LC55x_3074; rhodanese-like domain protein	OG_02562	EYR67852.1	hypothetical protein AZ78_13800 [Lysobacter capsici AZ78]
OG_02563	LC55x_4160	LC55x_4160; relA/SpoT family protein	OG_02563	EYR66321.1	ATP:GTP 3'-pyrophosphotransferase [Lysobacter capsici AZ78]
OG_02564	LC55x_4128	cysQ; 3'(2'),5'-bisphosphate nucleotidase	OG_02564	EYR66352.1	3'-5'-bisphosphate nucleotidase [Lysobacter capsici AZ78]
OG_02565	LC55x_2177	LC55x_2177; flavinator of succinate dehydrogenase family protein	OG_02565	EYR67394.1	hypothetical protein AZ78_15730 [Lysobacter capsici AZ78]
OG_02566	LC55x_3780	LC55x_3780; tat (twin-arginine translocation) pathway signal sequen	OG_02566	EYR67090.1	hypothetical protein AZ78_17670 [Lysobacter capsici AZ78]
OG_02567	LC55x_576	LC55x_576; DSBA-like thioredoxin domain protein	OG_02567	EYR70022.1	thioredoxin oxidoreductase [Lysobacter capsici AZ78]
OG_02568	LC55x_3674	hflB; ftsH HflB	OG_02568	EYR65943.1	ATP-dependent metalloprotease [Lysobacter capsici AZ78]
OG_02569	LC55x_1679	LC55x_1679; conserved hypothetical protein	OG_02569	EYR70346.1	iron transporter [Lysobacter capsici AZ78]
OG_02570	LC55x_1562	rfbC; dTDP-4-dehydrorhamnose 3,5-epimerase	OG_02570	EYR70282.1	dTDP-4-dehydrorhamnose 3,5-epimerase [Lysobacter capsici AZ78]
OG_02571	LC55x_1598	LC55x_1598; ABC-2 type transporter family protein	OG_02571	EYR70308.1	ABC transporter [Lysobacter capsici AZ78]
OG_02572	LC55x_4326	gspE; type II secretion system protein E	OG_02572	EYR69172.1	general secretion pathway protein E [Lysobacter capsici AZ78]
OG_02573	LC55x_3877	LC55x_3877; GDSL-like Lipase/Acylhydrolase family protein	OG_02573	EYR65711.1	lipase [Lysobacter capsici AZ78]
OG_02574	LC55x_2416	ccmB; heme exporter protein CcmB	OG_02574	EYR69581.1	heme ABC transporter permease [Lysobacter capsici AZ78]
OG_02575	LC55x_3764	LC55x_3764; molydopterin dinucleotide binding domain protein	OG_02575	EYR67073.1	CbbBc protein [Lysobacter capsici AZ78]
OG_02576	LC55x_2938	LC55x_2938; binding-protein-dependent transport system inner mem	OG_02576	EYR66222.1	sugar ABC transporter permease [Lysobacter capsici AZ78]
OG_02577	LC55x_5483	LC55x_5483; hypothetical protein	OG_02577	EYR68939.1	hypothetical protein AZ78_07985 [Lysobacter capsici AZ78]
OG_02578	LC55x_90	LC55x_90; rmuC family protein	OG_02578	EYR69755.1	recombinase RmuC [Lysobacter capsici AZ78]
OG_02579	LC55x_2323	LC55x_2323; hypothetical protein	OG_02579	EYR69843.1	hypothetical protein AZ78_04085 [Lysobacter capsici AZ78]
OG_02580	LC55x_2663	LC55x_2663; cheW-like domain protein	OG_02580	EYR68235.1	hypothetical protein AZ78_11255 [Lysobacter capsici AZ78]
OG_02581	LC55x_3691	LC55x_3691; hypothetical protein	OG_02581	EYR65928.1	hypothetical protein AZ78_23760 [Lysobacter capsici AZ78]
OG_02582	LC55x_5624	LC55x_5624; peptidase M20/M25/M40 family protein	OG_02582	EYR66264.1	peptidase M28 [Lysobacter capsici AZ78]
OG_02583	LC55x_3692	LC55x_3692; tRNA pseudouridine synthase, TruD family protein	OG_02583	EYR65926.1	tRNA pseudouridine synthase D [Lysobacter capsici AZ78]
OG_02584	LC55x_5157	LC55x_5157; conserved hypothetical protein	OG_02584	EYR68008.1	hypothetical protein AZ78_12390 [Lysobacter capsici AZ78]
OG_02585	LC55x_5719	LC55x_5719; ABC transporter family protein	OG_02585	EYR69690.1	ABC transporter [Lysobacter capsici AZ78]
OG_02586	LC55x_5225	LC55x_5225; DEAD/DEAH box helicase family protein	OG_02586	EYR68886.1	helicase [Lysobacter capsici AZ78]
OG_02587	LC55x_1088	LC55x_1088; 5'-nucleotidase	OG_02587	EYR68459.1	HAD family hydrolase [Lysobacter capsici AZ78]
OG_02588	LC55x_1085	LC55x_1085; conserved hypothetical protein	OG_02588	EYR65359.1	membrane protein, partial [Lysobacter capsici AZ78]
OG_02589	LC55x_3569	LC55x_3569; hypothetical protein	OG_02589	EYR67518.1	hypothetical protein AZ78_15340 [Lysobacter capsici AZ78]
OG_02590	LC55x_1697	LC55x_1697; conserved hypothetical protein	OG_02590	EYR70359.1	hypothetical protein AZ78_01415 [Lysobacter capsici AZ78]
OG_02591	LC55x_3907	LC55x_3907; glutaredoxin family protein	OG_02591	EYR66664.1	glutaredoxin [Lysobacter capsici AZ78]
OG_02592	LC55x_4939	LC55x_4939; short chain dehydrogenase family protein	OG_02592	EYR68681.1	tropinone reductase [Lysobacter capsici AZ78]
OG_02593	LC55x_2312	LC55x_2312; conserved hypothetical protein	OG_02593	EYR69652.1	agmatine deiminase [Lysobacter capsici AZ78]
OG_02594	LC55x_2297	LC55x_2297; efflux transporter, RND family, MFP subunit	OG_02594	EYR69662.1	hemolysin D [Lysobacter capsici AZ78]
OG_02595	LC55x_124	LC55x_124; glucan biosynthesis protein D	OG_02595	EYR69778.1	glucan biosynthesis protein D [Lysobacter capsici AZ78]
OG_02596	LC55x_845	LC55x_845; interferon-induced transmembrane family protein	OG_02596	EYR67569.1	membrane protein [Lysobacter capsici AZ78]
OG_02597	LC55x_1081	LC55x_1081; conserved hypothetical protein	OG_02597	EYR67711.1	thioesterase [Lysobacter capsici AZ78]
OG_02598	LC55x_787	glmU; UDP-N-acetylglucosamine diphosphorylase/glucosamine-1-ph	OG_02598	EYR67604.1	bifunctional N-acetylglucosamine-1-phosphate uridyltransferase/gluc
OG_02599	LC55x_5010	LC55x_5010; response regulator	OG_02599	EYR67220.1	chemotaxis protein CheY [Lysobacter capsici AZ78]
OG_02600	LC55x_4942	LC55x_4942; MATE efflux family protein	OG_02600	EYR68678.1	multidrug transporter MatE [Lysobacter capsici AZ78]
OG_02601	LC55x_4605	LC55x_4605; conserved hypothetical protein	OG_02601	EYR68352.1	hypothetical protein AZ78_09600 [Lysobacter capsici AZ78]
OG_02602	LC55x_1429	LC55x_1429; small GTP-binding domain protein	OG_02602	EYR67146.1	elongation factor G [Lysobacter capsici AZ78]
OG_02603	LC55x_2818	LC55x_2818; bacterial NAD-glutamate dehydrogenase family protein	OG_02603	EYR69380.1	glutamate dehydrogenase [Lysobacter capsici AZ78]
OG_02604	LC55x_3982	hemA; glutamyl-tRNA reductase	OG_02604	EYR66712.1	glutamyl-tRNA reductase [Lysobacter capsici AZ78]
OG_02605	LC55x_3416	LC55x_3416; L,D-transpeptidase catalytic domain protein	OG_02605	EYR66511.1	hypothetical protein AZ78_20365 [Lysobacter capsici AZ78]
OG_02606	LC55x_3816	LC55x_3816; hypothetical protein	OG_02606	EYR67120.1	hypothetical protein AZ78_17820 [Lysobacter capsici AZ78]
OG_02607	LC55x_1738	LC55x_1738; phosphate transporter family protein	OG_02607	EYR70386.1	inorganic phosphate transporter [Lysobacter capsici AZ78]
OG_02608	LC55x_1259	LC55x_1259; molybdopterin-binding domain of aldehyde dehydrogen	OG_02608	EYR65648.1	aldehyde oxidase [Lysobacter capsici AZ78]
OG_02609	LC55x_1988	LC55x_1988; sigma-54 interaction domain protein	OG_02609	EYR66783.1	hypothetical protein AZ78_19335 [Lysobacter capsici AZ78]
OG_02610	LC55x_2662	LC55x_2662; methyl-accepting chemotaxis (MCP) signalling domain	OG_02610	EYR68236.1	chemotaxis protein [Lysobacter capsici AZ78]
OG_02611	LC55x_3887	LC55x_3887; NADH:flavin oxidoreductase / NADH oxidase family pro	OG_02611	EYR65720.1	2,4-dienoyl-CoA reductase [Lysobacter capsici AZ78]
OG_02612	LC55x_438	LC55x_438; conserved hypothetical protein	OG_02612	EYR65523.1	hypothetical protein AZ78_25855 [Lysobacter capsici AZ78]
OG_02613	LC55x_5709	LC55x_5709; his Kinase A domain protein	OG_02613	EYR65850.1	hypothetical protein AZ78_23935 [Lysobacter capsici AZ78]
OG_02614	LC55x_1405	LC55x_1405; SET domain protein	OG_02614	EYR67163.1	nuclear protein SET [Lysobacter capsici AZ78]
OG_02615	LC55x_5211	HRSP12; ribonuclease UK114 domain protein	OG_02615	EYR68875.1	endoribonuclease L-PSP [Lysobacter capsici AZ78]
OG_02616	LC55x_2814	LC55x_2814; amino acid permease family protein	OG_02616	EYR69375.1	amino acid transporter [Lysobacter capsici AZ78]

OG_02617	LC55x_2026	kdpB; K+-transporting ATPase, B subunit
OG_02618	LC55x_2107	LC55x_2107; endonuclease/Exonuclease/phosphatase family protein
OG_02619	LC55x_4486	LC55x_4486; acetyltransferase family protein
OG_02620	LC55x_2470	recN; DNA repair protein RecN
OG_02621	LC55x_1587	LC55x_1587; putative protein with nucleoside triphosphate hydrolase
OG_02623	LC55x_2139	LC55x_2139; lytTr DNA-binding domain protein
OG_02624	LC55x_768	LC55x_768; conserved hypothetical protein
OG_02625	LC55x_1037	LC55x_1037; type IV pilus assembly PilM family protein
OG_02626	LC55x_3484	LC55x_3484; molybdopterin converting factor, subunit 1
OG_02627	LC55x_2702	LC55x_2702; tonB dependent receptor family protein
OG_02628	LC55x_4483	LC55x_4483; penicillin binding transpeptidase domain protein
OG_02629	LC55x_149	LC55x_149; glutamine-fructose-6-phosphate transaminase
OG_02630	LC55x_829	LC55x_829; NUDIX domain protein
OG_02631	LC55x_2450	mmsB; 3-hydroxyisobutyrate dehydrogenase
OG_02632	LC55x_4539	LC55x_4539; stearoyl-CoA 9-desaturase
OG_02633	LC55x_239	LC55x_239; conserved hypothetical protein
OG_02634	LC55x_1028	LC55x_1028; inosine-uridine preferring nucleoside hydrolase family p
OG_02635	LC55x_1933	LC55x_1933; conserved hypothetical protein
OG_02636	LC55x_2237	arsC; arsenate reductase
OG_02637	LC55x_1289	lipL; lactonizing lipase
OG_02638	LC55x_2316	rpsA; ribosomal protein S1
OG_02639	LC55x_317	LC55x_317; uroporphyrinogen-III synthase HemD family protein
OG_02640	LC55x_2190	pgsA; CDP-diacylglycerol--glycerol-3-phosphate 3-phosphatidyltransf
OG_02642	LC55x_3814	LC55x_3814; glyoxalase/Bleomycin resistance /Dioxygenase superfa
OG_02643	LC55x_900	LC55x_900; alpha/beta hydrolase family protein
OG_02644	LC55x_2659	LC55x_2659; putative acid phosphatase Wzb
OG_02645	LC55x_3808	LC55x_3808; alcohol dehydrogenase
OG_02646	LC55x_4392	LC55x_4392; fumarylacetoacetate (FAA) hydrolase family protein
OG_02647	LC55x_466	LC55x_466; conserved hypothetical protein
OG_02648	LC55x_1319	LC55x_1319; HPr Serine kinase N terminus family protein
OG_02649	LC55x_97	yrbE; mlaE
OG_02650	LC55x_636	LC55x_636; ompA family protein
OG_02651	LC55x_4730	LC55x_4730; histidine kinase-, DNA gyrase B-, and HSP90-like ATPa
OG_02652	LC55x_4496	LC55x_4496; glucose / Sorbosone dehydrogenase family protein
OG_02653	LC55x_5698	LC55x_5698; isocitrate dehydrogenase kinase/phosphatase family pr
OG_02654	LC55x_5147	LC55x_5147; trypsin-like peptidase domain protein
OG_02655	LC55x_4627	LC55x_4627; C-terminal processing peptidase family protein
OG_02656	LC55x_4361	LC55x_4361; 3-oxoacid CoA-transferase, A subunit
OG_02657	LC55x_2362	LC55x_2362; conserved hypothetical protein
OG_02658	LC55x_1911	LC55x_1911; RNA polymerase sigma factor, sigma-70 family protein
OG_02659	LC55x_3940	rpsN; 30S ribosomal subunit protein S14
OG_02660	LC55x_5245	bioB; biotin synthase
OG_02661	LC55x_2705	LC55x_2705; ROK family protein
OG_02662	LC55x_994	LC55x_994; 5-formyltetrahydrofolate cyclo-ligase
OG_02663	LC55x_3032	truA; tRNA pseudouridine(38-40) synthase
OG_02664	LC55x_4192	tolR; protein TolR
OG_02665	LC55x_1856	lpxD; UDP-3-O-[3-hydroxymyristoyl] glucosamine N-acyltransferase
OG_02666	LC55x_5102	LC55x_5102; binding-protein-dependent transport system inner mem
OG_02667	LC55x_3845	LC55x_3845; conserved hypothetical protein
OG_02668	LC55x_2062	LC55x_2062; conserved hypothetical protein
OG_02669	LC55x_4629	lipA; lipoyl synthase
OG_02670	LC55x_3739	Akp3; alkaline phosphatase 3, intestine, not Mn requiring
OG_02671	LC55x_2654	LC55x_2654; polysaccharide biosynthesis family protein
OG_02672	LC55x_5036	LC55x_5036; ankyrin repeat family protein
OG_02673	LC55x_1284	pdxH; pyridoxamine 5'-phosphate oxidase
OG_02674	LC55x_5026	LC55x_5026; ftsX-like permease family protein
OG_02675	LC55x_4951	LC55x_4951; conserved hypothetical protein
OG_02676	LC55x_3928	rplQ; ribosomal protein L17
OG_02677	LC55x_1359	LC55x_1359; macro domain protein
OG_02678	LC55x_3264	LC55x_3264; calcineurin-like phosphoesterase family protein
OG_02679	LC55x_4585	LC55x_4585; conserved hypothetical protein
OG_02680	LC55x_1669	pheS; phenylalanine--tRNA ligase, alpha subunit
OG_02681	LC55x_245	LC55x_245; conserved hypothetical protein
OG_02682	LC55x_4323	gspG; type II secretion system protein G
OG_02683	LC55x_3088	LC55x_3088; conserved hypothetical protein
OG_02684	LC55x_1258	hcrC; 4-hydroxybenzoyl-CoA reductase subunit gamma domain prote
OG_02685	LC55x_1225	LC55x_1225; aspartyl/Asparaginyl beta-hydroxylase family protein
OG_02686	LC55x_2871	lpdA; dihydrolipoyl dehydrogenase
OG_02687	LC55x_4037	LC55x_4037; conserved hypothetical protein
OG_02688	LC55x_2085	LC55x_2085; conserved hypothetical protein
OG_02689	LC55x_256	LC55x_256; putative transcriptional regulator, ArsR family protein
OG_02690	LC55x_2145	yliI; aldose sugar dehydrogenase
OG_02691	LC55x_1163	LC55x_1163; conserved hypothetical protein
OG_02692	LC55x_2561	LC55x_2561; bacterial regulatory helix-turn-helix , lysR family protein
OG_02693	LC55x_844	LC55x_844; conserved hypothetical protein
OG_02694	LC55x_4497	LC55x_4497; transglycosylase SLT domain protein
OG_02695	LC55x_1873	map; methionine aminopeptidase, type I
OG_02696	LC55x_139	LC55x_139; thiamine pyrophosphate enzyme, C-terminal TPP bindin
OG_02697	LC55x_2859	LC55x_2859; hypothetical protein
OG_02698	LC55x_1348	LC55x_1348; conserved hypothetical protein
OG_02699	LC55x_2405	LC55x_2405; conserved hypothetical protein

OG_02617	EYR65372.1	potassium-transporting ATPase subunit B [Lysobacter capsici AZ78]
OG_02618	EYR66399.1	hypothetical protein AZ78_21300 [Lysobacter capsici AZ78]
OG_02619	EYR68422.1	GNAT family acetyltransferase [Lysobacter capsici AZ78]
OG_02620	EYR69541.1	DNA repair protein RecN [Lysobacter capsici AZ78]
OG_02621	EYR70302.1	ATP-binding protein [Lysobacter capsici AZ78]
OG_02623	EYR66370.1	LytTR family transcriptional regulator [Lysobacter capsici AZ78]
OG_02624	EYR67622.1	hypothetical protein AZ78_14695 [Lysobacter capsici AZ78]
OG_02625	EYR67747.1	fimbrial assembly protein [Lysobacter capsici AZ78]
OG_02626	EYR66616.1	molybdopterin-converting factor chain 1 [Lysobacter capsici AZ78]
OG_02627	EYR69288.1	hypothetical protein AZ78_05180 [Lysobacter capsici AZ78]
OG_02628	EYR68589.1	hypothetical protein AZ78_10195 [Lysobacter capsici AZ78]
OG_02629	EYR69793.1	sigma factor regulator FecR [Lysobacter capsici AZ78]
OG_02630	EYR67581.1	RNA pyrophosphohydrolase [Lysobacter capsici AZ78]
OG_02631	EYR69553.1	3-hydroxyisobutyrate dehydrogenase [Lysobacter capsici AZ78]
OG_02632	EYR68385.1	acyl-CoA desaturase [Lysobacter capsici AZ78]
OG_02633	EYR66176.1	hypothetical protein AZ78_22670 [Lysobacter capsici AZ78]
OG_02634	EYR67754.1	inosine-uridine preferring nucleoside hydrolase [Lysobacter capsici A
OG_02635	EYR65971.1	copper(I)-binding protein [Lysobacter capsici AZ78]
OG_02636	EYR67430.1	arsenate reductase [Lysobacter capsici AZ78]
OG_02637	EYR67663.1	lactonizing lipase [Lysobacter capsici AZ78]
OG_02638	EYR69648.1	30S ribosomal protein S1 [Lysobacter capsici AZ78]
OG_02639	EYR68100.1	uroporphyrinogen-III synthase [Lysobacter capsici AZ78]
OG_02640	EYR67406.1	CDP-diacylglycerol--glycerol-3-phosphate 3-phosphatidyltransferase
OG_02642	EYR67118.1	bleomycin resistance protein [Lysobacter capsici AZ78]
OG_02643	EYR66858.1	hypothetical protein AZ78_18940 [Lysobacter capsici AZ78]
OG_02644	EYR68240.1	hypothetical protein AZ78_11280 [Lysobacter capsici AZ78]
OG_02645	EYR67113.1	NADPH:quinone oxidoreductase [Lysobacter capsici AZ78]
OG_02646	EYR69225.1	fumarylacetoacetate hydrolase [Lysobacter capsici AZ78]
OG_02647	EYR66472.1	hypothetical protein AZ78_21045 [Lysobacter capsici AZ78]
OG_02648	EYR69916.1	HPr kinase [Lysobacter capsici AZ78]
OG_02649	EYR69761.1	ABC transporter permease [Lysobacter capsici AZ78]
OG_02650	EYR69982.1	membrane protein [Lysobacter capsici AZ78]
OG_02651	EYR66114.1	hypothetical protein AZ78_22920 [Lysobacter capsici AZ78]
OG_02652	EYR68414.1	dehydrogenase [Lysobacter capsici AZ78]
OG_02653	EYR65860.1	isocitrate dehydrogenase [Lysobacter capsici AZ78]
OG_02654	EYR68014.1	hypothetical protein AZ78_12435 [Lysobacter capsici AZ78]
OG_02655	EYR68337.1	peptidase S41 [Lysobacter capsici AZ78]
OG_02656	EYR69198.1	succinyl-CoA:3-ketoacid-CoA transferase [Lysobacter capsici AZ78]
OG_02657	EYR69838.1	hypothetical protein AZ78_03905 [Lysobacter capsici AZ78]
OG_02658	EYR65985.1	RNA polymerase subunit sigma-24 [Lysobacter capsici AZ78]
OG_02659	EYR66691.1	30S ribosomal protein S14 [Lysobacter capsici AZ78]
OG_02660	EYR68901.1	biotin synthase [Lysobacter capsici AZ78]
OG_02661	EYR69291.1	hypothetical protein AZ78_05195 [Lysobacter capsici AZ78]
OG_02662	EYR67779.1	5-formyltetrahydrofolate cyclo-ligase [Lysobacter capsici AZ78]
OG_02663	EYR67823.1	tRNA pseudouridine synthase A [Lysobacter capsici AZ78]
OG_02664	EYR68157.1	biopolymer transporter TolR [Lysobacter capsici AZ78]
OG_02665	EYR66048.1	UDP-3-O-(3-hydroxymyristoyl) glucosamine N-acyltransferase [Lysob
OG_02666	EYR68048.1	peptide ABC transporter permease [Lysobacter capsici AZ78]
OG_02667	EYR65733.1	anaerobic ribonucleoside triphosphate reductase [Lysobacter capsici
OG_02668	EYR65405.1	hypothetical protein AZ78_26515 [Lysobacter capsici AZ78]
OG_02669	EYR68336.1	lipoyl synthase [Lysobacter capsici AZ78]
OG_02670	EYR67052.1	alkaline phosphatase [Lysobacter capsici AZ78]
OG_02671	EYR68244.1	hypothetical protein AZ78_11300 [Lysobacter capsici AZ78]
OG_02672	EYR66303.1	ankyrin [Lysobacter capsici AZ78]
OG_02673	EYR67667.1	pyridoxamine 5'-phosphate oxidase [Lysobacter capsici AZ78]
OG_02674	EYR66315.1	cell division protein FtsX [Lysobacter capsici AZ78]
OG_02675	EYR67270.1	hypothetical protein AZ78_17000 [Lysobacter capsici AZ78]
OG_02676	EYR66679.1	50S ribosomal protein L17 [Lysobacter capsici AZ78]
OG_02677	EYR67206.1	hypothetical protein AZ78_17435 [Lysobacter capsici AZ78]
OG_02678	EYR70035.1	calcineurin phosphoesterase [Lysobacter capsici AZ78]
OG_02679	EYR68363.1	hypothetical protein AZ78_09695 [Lysobacter capsici AZ78]
OG_02680	EYR70340.1	phenylalanyl-tRNA synthetase [Lysobacter capsici AZ78]
OG_02681	EYR66182.1	hypothetical protein AZ78_22700 [Lysobacter capsici AZ78]
OG_02682	EYR69170.1	general secretion pathway protein G [Lysobacter capsici AZ78]
OG_02683	EYR67861.1	hypothetical protein AZ78_13870 [Lysobacter capsici AZ78]
OG_02684	EYR65647.1	(2Fe-2S)-binding protein [Lysobacter capsici AZ78]
OG_02685	EYR65756.1	hypothetical protein AZ78_24350 [Lysobacter capsici AZ78]
OG_02686	EYR69430.1	dihydrolipoamide dehydrogenase [Lysobacter capsici AZ78]
OG_02687	EYR65816.1	hypothetical protein AZ78_24200 [Lysobacter capsici AZ78]
OG_02688	EYR66418.1	hypothetical protein AZ78_21395 [Lysobacter capsici AZ78]
OG_02689	EYR66985.1	ArsR family transcriptional regulator [Lysobacter capsici AZ78]
OG_02690	EYR67368.1	aldose sugar dehydrogenase YliI [Lysobacter capsici AZ78]
OG_02691	EYR68531.1	membrane protein [Lysobacter capsici AZ78]
OG_02692	EYR68332.1	hypothetical protein AZ78_11745 [Lysobacter capsici AZ78]
OG_02693	EYR67570.1	membrane protein [Lysobacter capsici AZ78]
OG_02694	EYR68413.1	transglycosylase [Lysobacter capsici AZ78]
OG_02695	EYR66066.1	Map [Lysobacter capsici AZ78]
OG_02696	EYR69788.1	MFS transporter [Lysobacter capsici AZ78]
OG_02697	EYR69419.1	hypothetical protein AZ78_05860 [Lysobacter capsici AZ78]
OG_02698	EYR69897.1	hypothetical protein AZ78_01665 [Lysobacter capsici AZ78]
OG_02699	EYR69591.1	membrane protein [Lysobacter capsici AZ78]

OG_02700	LC55x_3500	LC55x_3500; lysM domain protein	OG_02700	EYR66629.1	lytic transglycosylase [Lysobacter capsici AZ78]
OG_02701	LC55x_3777	LC55x_3777; peptidase M1 family protein	OG_02701	EYR67087.1	hypothetical protein AZ78_17655 [Lysobacter capsici AZ78]
OG_02702	LC55x_1764	LC55x_1764; peptidase M1 family protein	OG_02702	EYR65686.1	membrane protein [Lysobacter capsici AZ78]
OG_02703	LC55x_4243	LC55x_4243; putative transmembrane protein	OG_02703	EYR69099.1	membrane protein [Lysobacter capsici AZ78]
OG_02704	LC55x_2820	LC55x_2820; acyl-CoA dehydrogenase, N-terminal domain protein	OG_02704	EYR69382.1	acyl-CoA dehydrogenase [Lysobacter capsici AZ78]
OG_02705	LC55x_580	LC55x_580; amidohydrolase family protein	OG_02705	EYR70019.1	amidohydrolase [Lysobacter capsici AZ78]
OG_02706	LC55x_1983	LC55x_1983; conserved hypothetical protein	OG_02706	EYR66766.1	calcium-binding protein [Lysobacter capsici AZ78]
OG_02707	LC55x_870	LC55x_870; conserved hypothetical protein	OG_02707	EYR66887.1	hypothetical protein AZ78_19085 [Lysobacter capsici AZ78]
OG_02708	LC55x_1569	LC55x_1569; conserved hypothetical protein	OG_02708	EYR70288.1	membrane protein [Lysobacter capsici AZ78]
OG_02709	LC55x_1093	pyk; pyruvate kinase	OG_02709	EYR68461.1	pyruvate kinase [Lysobacter capsici AZ78]
OG_02710	LC55x_4946	LC55x_4946; conserved hypothetical protein	OG_02710	EYR68675.1	hypothetical protein AZ78_08625 [Lysobacter capsici AZ78]
OG_02711	LC55x_2181	LC55x_2181; DNA internalization-related competence protein ComE(	OG_02711	EYR67398.1	transporter [Lysobacter capsici AZ78]
OG_02712	LC55x_1435	LC55x_1435; sodium Bile acid symporter family protein	OG_02712	EYR67140.1	hypothetical protein AZ78_17105 [Lysobacter capsici AZ78]
OG_02713	LC55x_457	LC55x_457; biofilm formation and stress response factor family prote	OG_02713	EYR66478.1	hypothetical protein AZ78_21085 [Lysobacter capsici AZ78]
OG_02714	LC55x_2182	LC55x_2182; motA/TolQ/ExbB proton channel family protein	OG_02714	EYR67399.1	biopolymer transporter ExbB [Lysobacter capsici AZ78]
OG_02715	LC55x_2428	virB4; type IV secretion system protein virB4	OG_02715	EYR69570.1	hypothetical protein AZ78_03590 [Lysobacter capsici AZ78]
OG_02716	LC55x_3000	nusA; transcription termination/antitermination L factor	OG_02716	EYR67799.1	peptidase M54 [Lysobacter capsici AZ78]
OG_02717	LC55x_707	LC55x_707; HEAT repeats family protein	OG_02717	EYR70098.1	hypothetical protein AZ78_01970 [Lysobacter capsici AZ78]
OG_02718	LC55x_3326	ubiG; 3-demethylubiquinone-9 3-O-methyltransferase	OG_02718	EYR70078.1	3-demethylubiquinone-9 3-methyltransferase [Lysobacter capsici AZ7
OG_02719	LC55x_3974	LC55x_3974; peptidyl-tRNA hydrolase family protein	OG_02719	EYR66707.1	peptidyl-tRNA hydrolase [Lysobacter capsici AZ78]
OG_02720	LC55x_3062	LC55x_3062; hypothetical protein	OG_02720	EYR67878.1	hypothetical protein AZ78_13735 [Lysobacter capsici AZ78]
OG_02721	LC55x_727	LC55x_727; eamA-like transporter family protein	OG_02721	EYR67652.1	permease [Lysobacter capsici AZ78]
OG_02722	LC55x_4704	LC55x_4704; conserved hypothetical protein	OG_02722	EYR65666.1	hypothetical protein AZ78_25025 [Lysobacter capsici AZ78]
OG_02723	LC55x_156	eda; 2-dehydro-3-deoxyphosphogluconate aldolase/4-hydroxy-2-oxog	OG_02723	EYR69799.1	ketohydroxyglutarate aldolase [Lysobacter capsici AZ78]
OG_02724	LC55x_1838	LC55x_1838; conserved hypothetical protein	OG_02724	EYR66030.1	hypothetical protein AZ78_23005 [Lysobacter capsici AZ78]
OG_02725	LC55x_3945	rpmC; ribosomal protein L29	OG_02725	EYR66696.1	50S ribosomal protein L29 [Lysobacter capsici AZ78]
OG_02726	LC55x_369	LC55x_369; RF-1 domain protein	OG_02726	EYR68140.1	peptidyl-tRNA hydrolase [Lysobacter capsici AZ78]
OG_02727	LC55x_4912	dtd; D-tyrosyl-tRNA(Tyr) deacylase	OG_02727	EYR68704.1	D-tyrosyl-tRNA(Tyr) deacylase [Lysobacter capsici AZ78]
OG_02728	LC55x_387	LC55x_387; bacterial regulatory helix-turn-helix, AraC family protein	OG_02728	EYR65625.1	AraC family transcriptional regulator [Lysobacter capsici AZ78]
OG_02729	LC55x_541	LC55x_541; eamA-like transporter family protein	OG_02729	EYR67901.1	hypothetical protein AZ78_12905 [Lysobacter capsici AZ78]
OG_02730	LC55x_1281	LC55x_1281; shikimate kinase family protein	OG_02730	EYR67669.1	shikimate kinase [Lysobacter capsici AZ78]
OG_02731	LC55x_1724	tgt; queuine tRNA-ribosyltransferase	OG_02731	EYR70374.1	queuine tRNA-ribosyltransferase [Lysobacter capsici AZ78]
OG_02732	LC55x_1945	ribF; riboflavin biosynthesis protein RibF	OG_02732	EYR65963.1	riboflavin kinase [Lysobacter capsici AZ78]
OG_02733	LC55x_2830	LC55x_2830; alpha/beta hydrolase fold family protein	OG_02733	EYR69392.1	hypothetical protein AZ78_05725 [Lysobacter capsici AZ78]
OG_02734	LC55x_3570	LC55x_3570; conserved hypothetical protein	OG_02734	EYR67519.1	hypothetical protein AZ78_15345 [Lysobacter capsici AZ78]
OG_02735	LC55x_2523	LC55x_2523; hypothetical protein	OG_02735	EYR69807.1	hypothetical protein AZ78_03125 [Lysobacter capsici AZ78]
OG_02736	LC55x_4227	LC55x_4227; threonine-phosphate decarboxylase	OG_02736	EYR69088.1	threonine-phosphate decarboxylase [Lysobacter capsici AZ78]
OG_02737	LC55x_1691	LC55x_1691; tetratricopeptide repeat family protein	OG_02737	EYR70355.1	serine/threonine protein kinase [Lysobacter capsici AZ78]
OG_02738	LC55x_1965	LC55x_1965; H+ antiporter-2 family protein	OG_02738	EYR66779.1	multidrug resistance protein B [Lysobacter capsici AZ78]
OG_02739	LC55x_5030	trxA; thioredoxin	OG_02739	EYR66310.1	thioredoxin [Lysobacter capsici AZ78]
OG_02740	LC55x_2035	LC55x_2035; ACT domain protein	OG_02740	EYR65591.1	glycine cleavage system regulatory protein [Lysobacter capsici AZ78
OG_02741	LC55x_606	LC55x_606; bacterial regulatory helix-turn-helix , lysR family protein	OG_02741	EYR69999.1	LysR family transcriptional regulator [Lysobacter capsici AZ78]
OG_02742	LC55x_2503	LC55x_2503; conserved hypothetical protein	OG_02742	EYR69515.1	hypothetical protein AZ78_03230 [Lysobacter capsici AZ78]
OG_02743	LC55x_3579	LC55x_3579; bacterial regulatory, luxR family protein	OG_02743	EYR67527.1	LuxR family transcriptional regulator [Lysobacter capsici AZ78]
OG_02744	LC55x_2104	LC55x_2104; 2'-5' RNA ligase	OG_02744	EYR66402.1	hypothetical protein AZ78_21315 [Lysobacter capsici AZ78]
OG_02745	LC55x_5040	LC55x_5040; bacterial Cytochrome Ubiquinol Oxidase family protein	OG_02745	EYR66299.1	cytochrome D ubiquinol oxidase subunit I [Lysobacter capsici AZ78]
OG_02746	LC55x_240	LC55x_240; type III secretion apparatus lipoprotein, YscJ/HrcJ family	OG_02746	EYR66177.1	hypothetical protein AZ78_22675 [Lysobacter capsici AZ78]
OG_02747	LC55x_3254	LC55x_3254; NUDIX domain protein	OG_02747	EYR67929.1	NUDIX hydrolase [Lysobacter capsici AZ78]
OG_02748	LC55x_941	thrB; homoserine kinase	OG_02748	EYR66820.1	homoserine kinase [Lysobacter capsici AZ78]
OG_02749	LC55x_2460	LC55x_2460; bacteriophage CI repressor helix-turn-helix domain prot	OG_02749	EYR69548.1	DNA-binding protein [Lysobacter capsici AZ78]
OG_02750	LC55x_549	LC55x_549; efflux transporter, RND family, MFP subunit	OG_02750	EYR67910.1	hypothetical protein AZ78_12950 [Lysobacter capsici AZ78]
OG_02751	LC55x_1072	LC55x_1072; ompW family protein	OG_02751	EYR67717.1	membrane protein [Lysobacter capsici AZ78]
OG_02752	LC55x_1609	LC55x_1609; cytochrome b(C-terminal)/b6/petD family protein	OG_02752	EYR70315.1	cytochrome B [Lysobacter capsici AZ78]
OG_02753	LC55x_5670	LC55x_5670; aldo/keto reductase family protein	OG_02753	EYR65884.1	aldo/keto reductase [Lysobacter capsici AZ78]
OG_02754	LC55x_4487	LC55x_4487; conserved hypothetical protein	OG_02754	EYR68421.1	hypothetical protein AZ78_10180 [Lysobacter capsici AZ78]
OG_02755	LC55x_4308	map; methionine aminopeptidase, type I	OG_02755	EYR69156.1	methionine aminopeptidase [Lysobacter capsici AZ78]
OG_02756	LC55x_136	LC55x_136; putative transmembrane regulator	OG_02756	EYR69787.1	transmembrane anti-sigma factor [Lysobacter capsici AZ78]
OG_02757	LC55x_3302	argH; argininosuccinate lyase	OG_02757	EYR70059.1	argininosuccinate lyase [Lysobacter capsici AZ78]
OG_02758	LC55x_231	LC55x_231; his Kinase A domain protein	OG_02758	EYR66171.1	hypothetical protein AZ78_22645 [Lysobacter capsici AZ78]
OG_02759	LC55x_4276	ftsI; essential cell division FtsI; penicillin-binding protein 3	OG_02759	EYR69134.1	cell division protein [Lysobacter capsici AZ78]
OG_02760	LC55x_2533	lolA; outer membrane lipocarrier protein LolA	OG_02760	EYR69491.1	membrane protein [Lysobacter capsici AZ78]
OG_02761	LC55x_2079	LC55x_2079; conserved hypothetical protein	OG_02761	EYR66424.1	hypothetical protein AZ78_21425 [Lysobacter capsici AZ78]
OG_02762	LC55x_2061	metG; methionyl-tRNA synthetase	OG_02762	EYR65406.1	methionyl-tRNA synthetase [Lysobacter capsici AZ78]
OG_02763	LC55x_5615	LC55x_5615; EF hand family protein	OG_02763	EYR66255.1	hypothetical protein AZ78_22050 [Lysobacter capsici AZ78]
OG_02764	LC55x_4660	chiD; chitinase D	OG_02764	EYR70163.1	glycosyl hydrolase [Lysobacter capsici AZ78]
OG_02765	LC55x_906	LC55x_906; glnD PII-uridylyltransferase family protein	OG_02765	EYR66853.1	glutamate-ammonia ligase adenylyltransferase [Lysobacter capsici A
OG_02766	LC55x_2228	LC55x_2228; 7, 8-dihydro-6-hydroxymethylpterin-pyrophosphokinase	OG_02766	EYR67462.1	hypothetical protein AZ78_15955 [Lysobacter capsici AZ78]
OG_02767	LC55x_4144	LC55x_4144; response regulator	OG_02767	EYR66337.1	chemotaxis protein CheY [Lysobacter capsici AZ78]
OG_02768	LC55x_3080	LC55x_3080; phage Tail Collar domain protein	OG_02768	EYR67856.1	microcystin dependent protein [Lysobacter capsici AZ78]
OG_02769	LC55x_4267	ftsA; cell division protein FtsA	OG_02769	EYR69125.1	cell division protein FtsA [Lysobacter capsici AZ78]
OG_02770	LC55x_4314	LC55x_4314; glycosyl transferase 2 family protein	OG_02770	EYR69161.1	polysaccharide production-like protein [Lysobacter capsici AZ78]
OG_02771	LC55x_2795	LC55x_2795; carbonic anhydrase family protein	OG_02771	EYR69359.1	carbonate dehydratase [Lysobacter capsici AZ78]
OG_02772	LC55x_444	LC55x_444; conserved hypothetical protein	OG_02772	EYR65529.1	anhydro-N-acetylmuramic acid kinase [Lysobacter capsici AZ78]
OG_02773	LC55x_4501	LC55x_4501; NADPH-dependent FMN reductase family protein	OG_02773	EYR68411.1	FMN reductase [Lysobacter capsici AZ78]
OG_02774	LC55x_3140	LC55x_3140; lipase family protein	OG_02774	EYR66955.1	hypothetical protein AZ78_18455 [Lysobacter capsici AZ78]
OG_02775	LC55x_159	LC55x_159; hypothetical protein	OG_02775	EYR69890.1	hypothetical protein AZ78_05150 [Lysobacter capsici AZ78]
OG_02776	LC55x_384	LC55x_384; alpha/beta hydrolase fold family protein	OG_02776	EYR65628.1	alpha/beta hydrolase [Lysobacter capsici AZ78]
OG_02777	LC55x_5610	LC55x_5610; transcriptional regulatory , C terminal family protein	OG_02777	EYR66248.1	chemotaxis protein CheY [Lysobacter capsici AZ78]
OG_02778	LC55x_970	mpl; L-alanyl-gamma-D-glutamyl-meso-diaminopimelate ligase	OG_02778	EYR66795.1	UDP-N-acetylmuramate:L-alanyl-gamma-D-glutamyl-meso-diaminop
OG_02779	LC55x_2292	LC55x_2292; pterin 4 alpha carbinolamine dehydratase family protein	OG_02779	EYR69666.1	pterin-4-alpha-carbinolamine dehydratase [Lysobacter capsici AZ78]
OG_02780	LC55x_755	LC55x_755; conserved hypothetical protein	OG_02780	EYR67634.1	hypothetical protein AZ78_14760 [Lysobacter capsici AZ78]

OG_02781	LC55x_973	LC55x_973; putative serine/threonine protein kinase
OG_02782	LC55x_15	LC55x_15; hypothetical protein
OG_02783	LC55x_2082	LC55x_2082; dienelactone hydrolase family protein
OG_02784	LC55x_5018	LC55x_5018; bacterial regulatory helix-turn-helix , lysR family protein
OG_02785	LC55x_3086	LC55x_3086; hypothetical protein
OG_02786	LC55x_726	LC55x_726; bacterial regulatory, gntR family protein
OG_02787	LC55x_1771	LC55x_1771; diguanylate cyclase domain protein
OG_02788	LC55x_3202	LC55x_3202; polymer-forming cytoskeletal family protein
OG_02789	LC55x_2562	LC55x_2562; acyl-CoA dehydrogenase, C-terminal domain protein
OG_02790	LC55x_3435	lepA; GTP-binding protein LepA
OG_02791	LC55x_729	rtcB; conserved protein
OG_02792	LC55x_1030	gltA; citrate (Si)-synthase
OG_02793	LC55x_1033	LC55x_1033; conserved hypothetical protein
OG_02794	LC55x_2069	pcp; pyroglutamyl-peptidase I
OG_02795	LC55x_4351	LC55x_4351; ABC transporter family protein
OG_02796	LC55x_4048	LC55x_4048; putative NAD
OG_02797	LC55x_1623	LC55x_1623; oxidoreductase, short chain dehydrogenase/reductase
OG_02798	LC55x_4032	LC55x_4032; conserved hypothetical protein
OG_02799	LC55x_1892	LC55x_1892; proton antiporter-2 family protein
OG_02800	LC55x_1967	LC55x_1967; efflux transporter, RND family, MFP subunit
OG_02801	LC55x_308	LC55x_308; conserved hypothetical protein
OG_02802	LC55x_1955	radA; DNA repair protein RadA
OG_02803	LC55x_2794	ALDH8A1; aldehyde dehydrogenase family 8 member A1
OG_02804	LC55x_1865	LC55x_1865; blaR1 peptidase M56 family protein
OG_02805	LC55x_5650	LC55x_5650; pspA/IM30 family protein
OG_02806	LC55x_2389	phaE; poly(R)-hydroxyalkanoic acid synthase, class III, PhaE subunit
OG_02807	LC55x_746	LC55x_746; hypothetical protein
OG_02808	LC55x_5190	LC55x_5190; conserved hypothetical protein
OG_02809	LC55x_4082	LC55x_4082; sugar (and other) transporter family protein
OG_02810	LC55x_4674	LC55x_4674; dienelactone hydrolase family protein
OG_02811	LC55x_5201	LC55x_5201; conserved hypothetical protein
OG_02812	LC55x_2143	LC55x_2143; conserved hypothetical protein
OG_02813	LC55x_344	LC55x_344; conserved hypothetical protein
OG_02814	LC55x_51	LC55x_51; hypothetical protein
OG_02815	LC55x_2248	LC55x_2248; poly(hydroxyalkanoate) granule-associated domain pro
OG_02816	LC55x_1403	sufB; feS assembly protein SufB
OG_02817	LC55x_340	fabH; 3-oxoacyl-synthase III
OG_02818	LC55x_3704	LC55x_3704; hypothetical protein
OG_02819	LC55x_2051	LC55x_2051; N-acetylmuramoyl-L-alanine amidase family protein
OG_02820	LC55x_1008	LC55x_1008; radical SAM superfamily protein
OG_02821	LC55x_3014	nuoC; NADH (or F420H2) dehydrogenase, subunit C family protein
OG_02822	LC55x_1266	LC55x_1266; ycel-like domain protein
OG_02823	LC55x_2625	LC55x_2625; SURF1 family protein
OG_02824	LC55x_5058	LC55x_5058; disulfide bond reductase
OG_02825	LC55x_3046	LC55x_3046; lysM domain protein
OG_02826	LC55x_4691	LC55x_4691; conserved hypothetical protein
OG_02827	LC55x_4110	dgkA; dgkA
OG_02828	LC55x_288	LC55x_288; putative trans-aconitate 2-methyltransferase
OG_02829	LC55x_5141	LC55x_5141; polysaccharide biosynthesis family protein
OG_02830	LC55x_3203	LC55x_3203; zinc-ribbon domain protein
OG_02831	LC55x_76	LC55x_76; M42 glutamyl aminopeptidase family protein
OG_02832	LC55x_3463	LC55x_3463; transglutaminase-like superfamily protein
OG_02833	LC55x_4131	LC55x_4131; RNA methyltransferase, RsmE family protein
OG_02834	LC55x_882	cutA; divalent-cation tolerance protein cutA
OG_02835	LC55x_3822	LC55x_3822; phosphodiesterase-nucleotide pyrophosphatase
OG_02836	LC55x_3728	LC55x_3728; NADPH-dependent FMN reductase family protein
OG_02837	LC55x_4987	LC55x_4987; cupin domain protein
OG_02838	LC55x_1368	LC55x_1368; sulfite reductase [NADPH] flavoprotein, alpha-compone
OG_02839	LC55x_1333	LC55x_1333; conserved hypothetical protein
OG_02840	LC55x_3686	pcm; protein-L-isoaspartate O-methyltransferase
OG_02841	LC55x_3831	LC55x_3831; outer membrane autotransporter barrel domain protein
OG_02842	LC55x_4747	LC55x_4747; D-ala-D-ala dipeptidase family protein
OG_02843	LC55x_3085	LC55x_3085; conserved hypothetical protein
OG_02844	LC55x_115	LC55x_115; hlyD secretion family protein
OG_02845	LC55x_3875	LC55x_3875; sugar (and other) transporter family protein
OG_02846	LC55x_3166	LC55x_3166; methyltransferase domain protein
OG_02847	LC55x_2364	orn; oligoribonuclease
OG_02848	LC55x_2438	LC55x_2438; conserved hypothetical protein
OG_02849	LC55x_1839	LC55x_1839; conserved hypothetical protein
OG_02850	LC55x_4918	LC55x_4918; O-Antigen ligase family protein
OG_02851	LC55x_3289	pgaC; poly-beta-1,6 N-acetyl-D-glucosamine synthase
OG_02852	LC55x_3276	LC55x_3276; aromatic amino acid lyase family protein
OG_02853	LC55x_860	LC55x_860; putative hydrolase
OG_02854	LC55x_2627	cyoC; cytochrome o ubiquinol oxidase, subunit III
OG_02855	LC55x_1935	uvrA; excinuclease ABC subunit A
OG_02856	LC55x_2802	kmo; kynurenine 3-monooxygenase
OG_02857	LC55x_546	LC55x_546; metal-sensitive transcriptional repressor family protein
OG_02858	LC55x_4277	ftsL; cell division protein FtsL
OG_02859	LC55x_1735	LC55x_1735; exopolysaccharide synthesis, ExoD family protein
OG_02860	LC55x_5664	LC55x_5664; efflux transporter, outer membrane factor (OMF) lipo, N
OG_02861	LC55x_2770	rplI; ribosomal protein L9

OG_02781	EYR66792.1	serine/threonine protein kinase [Lysobacter capsici AZ78]
OG_02782	EYR69718.1	hypothetical protein AZ78_04565 [Lysobacter capsici AZ78]
OG_02783	EYR66421.1	carboxymethylenebutenolidase [Lysobacter capsici AZ78]
OG_02784	EYR67212.1	hypothetical protein AZ78_16710 [Lysobacter capsici AZ78]
OG_02785	EYR67885.1	hypothetical protein AZ78_13865 [Lysobacter capsici AZ78]
OG_02786	EYR67653.1	aspartate aminotransferase [Lysobacter capsici AZ78]
OG_02787	EYR67272.1	diguanylate cyclase [Lysobacter capsici AZ78]
OG_02788	EYR67986.1	hypothetical protein AZ78_13340 [Lysobacter capsici AZ78]
OG_02789	EYR68331.1	acyl-CoA dehydrogenase [Lysobacter capsici AZ78]
OG_02790	EYR66496.1	elongation factor 4 [Lysobacter capsici AZ78]
OG_02791	EYR67651.1	RTCB protein [Lysobacter capsici AZ78]
OG_02792	EYR67752.1	type II citrate synthase [Lysobacter capsici AZ78]
OG_02793	EYR67786.1	hypothetical protein AZ78_14115 [Lysobacter capsici AZ78]
OG_02794	EYR65399.1	pyrrolidone-carboxylate peptidase [Lysobacter capsici AZ78]
OG_02795	EYR69191.1	iron ABC transporter ATP-binding protein [Lysobacter capsici AZ78]
OG_02796	EYR65827.1	NAD(P) transhydrogenase subunit alpha [Lysobacter capsici AZ78]
OG_02797	EYR70326.1	short-chain dehydrogenase [Lysobacter capsici AZ78]
OG_02798	EYR65811.1	FeS assembly SUF system protein SufT [Lysobacter capsici AZ78]
OG_02799	EYR65999.1	potassium transporter [Lysobacter capsici AZ78]
OG_02800	EYR66778.1	multidrug transporter [Lysobacter capsici AZ78]
OG_02801	EYR67038.1	hypothetical protein AZ78_18155 [Lysobacter capsici AZ78]
OG_02802	EYR65958.1	DNA repair protein RadA [Lysobacter capsici AZ78]
OG_02803	EYR69358.1	2-hydroxymuconic semialdehyde dehydrogenase [Lysobacter capsic
OG_02804	EYR66057.1	hypothetical protein AZ78_23140 [Lysobacter capsici AZ78]
OG_02805	EYR66287.1	hypothetical protein AZ78_22215 [Lysobacter capsici AZ78]
OG_02806	EYR69599.1	PHA synthase subunit [Lysobacter capsici AZ78]
OG_02807	EYR67698.1	hypothetical protein AZ78_14805 [Lysobacter capsici AZ78]
OG_02808	EYR68862.1	hypothetical protein AZ78_07300 [Lysobacter capsici AZ78]
OG_02809	EYR65434.1	MFS transporter [Lysobacter capsici AZ78]
OG_02810	EYR70172.1	alpha/beta hydrolase [Lysobacter capsici AZ78]
OG_02811	EYR68867.1	hypothetical protein AZ78_07350 [Lysobacter capsici AZ78]
OG_02812	EYR66367.1	transcription accessory protein [Lysobacter capsici AZ78]
OG_02813	EYR68178.1	hypothetical protein AZ78_11890 [Lysobacter capsici AZ78]
OG_02814	EYR69867.1	hypothetical protein AZ78_04675 [Lysobacter capsici AZ78]
OG_02815	EYR67436.1	poly granule associated protein [Lysobacter capsici AZ78]
OG_02816	EYR67165.1	cysteine desulfurase activator complex subunit SufB [Lysobacter cap
OG_02817	EYR68118.1	3-oxoacyl-ACP synthase [Lysobacter capsici AZ78]
OG_02818	EYR65915.1	hypothetical protein AZ78_23690 [Lysobacter capsici AZ78]
OG_02819	EYR65604.1	N-acetylmuramoyl-L-alanine amidase [Lysobacter capsici AZ78]
OG_02820	EYR67768.1	coproporphyrinogen III oxidase [Lysobacter capsici AZ78]
OG_02821	EYR67812.1	NADH dehydrogenase subunit C [Lysobacter capsici AZ78]
OG_02822	EYR67678.1	hypothetical protein AZ78_15080 [Lysobacter capsici AZ78]
OG_02823	EYR68271.1	hypothetical protein AZ78_11435 [Lysobacter capsici AZ78]
OG_02824	EYR65585.1	glutathione S-transferase [Lysobacter capsici AZ78]
OG_02825	EYR67832.1	murein transglycosylase [Lysobacter capsici AZ78]
OG_02826	EYR70189.1	membrane protein [Lysobacter capsici AZ78]
OG_02827	EYR65512.1	DeoR family transcriptional regulator [Lysobacter capsici AZ78]
OG_02828	EYR67015.1	trans-aconitate 2-methyltransferase [Lysobacter capsici AZ78]
OG_02829	EYR68020.1	NAD dependent epimerase/dehydratase [Lysobacter capsici AZ78]
OG_02830	EYR67985.1	hypothetical protein AZ78_13335 [Lysobacter capsici AZ78]
OG_02831	EYR69750.1	peptidase M28 [Lysobacter capsici AZ78]
OG_02832	EYR66606.1	membrane protein [Lysobacter capsici AZ78]
OG_02833	EYR66349.1	16S rRNA methyltransferase [Lysobacter capsici AZ78]
OG_02834	EYR66875.1	dihydroorotate dehydrogenase [Lysobacter capsici AZ78]
OG_02835	EYR67127.1	phosphodiesterase-nucleotide pyrophosphatase [Lysobacter capsici
OG_02836	EYR65897.1	hypothetical protein AZ78_23600 [Lysobacter capsici AZ78]
OG_02837	EYR67241.1	cupin [Lysobacter capsici AZ78]
OG_02838	EYR67196.1	NADP oxidoreductase [Lysobacter capsici AZ78]
OG_02839	EYR69904.1	hypothetical protein AZ78_01725 [Lysobacter capsici AZ78]
OG_02840	EYR65932.1	protein-L-isoaspartate O-methyltransferase [Lysobacter capsici AZ78
OG_02841	EYR65748.1	hypothetical protein AZ78_24715 [Lysobacter capsici AZ78]
OG_02842	EYR66099.1	D-alanyl-D-alanine dipeptidase [Lysobacter capsici AZ78]
OG_02843	EYR67860.1	hypothetical protein AZ78_13855 [Lysobacter capsici AZ78]
OG_02844	EYR69774.1	membrane protein [Lysobacter capsici AZ78]
OG_02845	EYR65709.1	DHA1 family MFS transporter [Lysobacter capsici AZ78]
OG_02846	EYR66935.1	methyltransferase [Lysobacter capsici AZ78]
OG_02847	EYR69615.1	oligoribonuclease [Lysobacter capsici AZ78]
OG_02848	EYR69562.1	thiol-disulfide oxidoreductase [Lysobacter capsici AZ78]
OG_02849	EYR66031.1	hypothetical protein AZ78_23010 [Lysobacter capsici AZ78]
OG_02850	EYR68698.1	polymerase [Lysobacter capsici AZ78]
OG_02851	EYR70054.1	N-glycosyltransferase [Lysobacter capsici AZ78]
OG_02852	EYR70046.1	histidine ammonia-lyase [Lysobacter capsici AZ78]
OG_02853	EYR66897.1	hypothetical protein AZ78_19140 [Lysobacter capsici AZ78]
OG_02854	EYR68269.1	cytochrome O ubiquinol oxidase [Lysobacter capsici AZ78]
OG_02855	EYR65969.1	excinuclease ABC subunit A [Lysobacter capsici AZ78]
OG_02856	EYR69366.1	kynurenine 3-monooxygenase [Lysobacter capsici AZ78]
OG_02857	EYR67906.1	hypothetical protein AZ78_12930 [Lysobacter capsici AZ78]
OG_02858	EYR69135.1	cell division protein FtsL [Lysobacter capsici AZ78]
OG_02859	EYR70383.1	exod protein [Lysobacter capsici AZ78]
OG_02860	EYR65891.1	hypothetical protein AZ78_24145 [Lysobacter capsici AZ78]
OG_02861	EYR69336.1	50S ribosomal protein L9 [Lysobacter capsici AZ78]

OG_02862	LC55x_5101	nikE; nickel import ATP-binding protein NikE	OG_02862	EYR68049.1	glutathione ABC transporter ATP-binding protein [Lysobacter capsici
OG_02863	LC55x_3496	dnaQ; DNA polymerase III, epsilon subunit	OG_02863	EYR66625.1	DNA polymerase III subunit epsilon [Lysobacter capsici AZ78]
OG_02864	LC55x_1706	LC55x_1706; RNA methyltransferase, RsmD family	OG_02864	EYR70364.1	methyltransferase [Lysobacter capsici AZ78]
OG_02865	LC55x_1226	LC55x_1226; tonB dependent receptor family protein	OG_02865	EYR65755.1	hypothetical protein AZ78_24345 [Lysobacter capsici AZ78]
OG_02866	LC55x_1941	cgtA; obg family GTPase CgtA	OG_02866	EYR65966.1	GTPase CgtA [Lysobacter capsici AZ78]
OG_02867	LC55x_4125	LC55x_4125; conserved hypothetical protein	OG_02867	EYR66355.1	hypothetical protein AZ78_21630 [Lysobacter capsici AZ78]
OG_02868	LC55x_5060	LC55x_5060; conserved hypothetical protein	OG_02868	EYR65587.1	hypothetical protein AZ78_25565 [Lysobacter capsici AZ78]
OG_02869	LC55x_547	LC55x_547; S-(hydroxymethyl)glutathione dehydrogenase/class III al	OG_02869	EYR67907.1	S-(hydroxymethyl)glutathione dehydrogenase [Lysobacter capsici AZ
OG_02870	LC55x_2245	DBI; acyl-CoA-binding protein	OG_02870	EYR67434.1	acyl-CoA-binding protein [Lysobacter capsici AZ78]
OG_02871	LC55x_3523	LC55x_3523; HAMP domain protein	OG_02871	EYR67474.1	hypothetical protein AZ78_15115 [Lysobacter capsici AZ78]
OG_02872	LC55x_2583	LC55x_2583; bacterial regulatory helix-turn-helix, AraC family protein	OG_02872	EYR68311.1	hypothetical protein AZ78_11640 [Lysobacter capsici AZ78]
OG_02873	LC55x_2912	LC55x_2912; conserved hypothetical protein	OG_02873	EYR69468.1	hypothetical protein AZ78_06105 [Lysobacter capsici AZ78]
OG_02874	LC55x_5178	glyS; glycine--tRNA ligase, beta subunit	OG_02874	EYR68852.1	glycyl-tRNA synthetase subunit beta [Lysobacter capsici AZ78]
OG_02875	LC55x_2232	LC55x_2232; hypothetical protein	OG_02875	EYR67463.1	hypothetical protein AZ78_15975 [Lysobacter capsici AZ78]
OG_02876	LC55x_4288	LC55x_4288; FAD linked oxidase, C-terminal domain protein	OG_02876	EYR69143.1	dimethylmenaquinone methyltransferase [Lysobacter capsici AZ78]
OG_02877	LC55x_526	ysxC; ribosome biogenesis GTP-binding protein YsxC	OG_02877	EYR67887.1	GTP-binding protein YsxC [Lysobacter capsici AZ78]
OG_02878	LC55x_236	LC55x_236; type III secretion , HrcV family protein	OG_02878	EYR66173.1	hypothetical protein AZ78_22655 [Lysobacter capsici AZ78]
OG_02879	LC55x_2976	LC55x_2976; conserved hypothetical protein	OG_02879	EYR66187.1	hypothetical protein AZ78_21700 [Lysobacter capsici AZ78]
OG_02880	LC55x_3069	LC55x_3069; beta-lactamase family protein	OG_02880	EYR67850.1	beta-lactamase [Lysobacter capsici AZ78]
OG_02881	LC55x_3679	LC55x_3679; hypothetical protein	OG_02881	EYR65937.1	hypothetical protein AZ78_23805 [Lysobacter capsici AZ78]
OG_02882	LC55x_824	bioD; dethiobiotin synthase	OG_02882	EYR67584.1	dithiobiotin synthetase [Lysobacter capsici AZ78]
OG_02883	LC55x_3771	LC55x_3771; bacterial regulatory helix-turn-helix , lysR family protein	OG_02883	EYR67081.1	hypothetical protein AZ78_17620 [Lysobacter capsici AZ78]
OG_02884	LC55x_3801	LC55x_3801; bifunctional NMN adenylyltransferase/Nudix hydrolase	OG_02884	EYR67107.1	ADP-ribose pyrophosphatase [Lysobacter capsici AZ78]
OG_02885	LC55x_2656	LC55x_2656; UDP-N-acetylglucosamine 2-epimerase	OG_02885	EYR68242.1	hypothetical protein AZ78_11290 [Lysobacter capsici AZ78]
OG_02886	LC55x_5122	moxR2; magnesium chelatase, putative	OG_02886	EYR68034.1	magnesium chelatase [Lysobacter capsici AZ78]
OG_02887	LC55x_3281	LC55x_3281; polysaccharide deacetylase family protein	OG_02887	EYR70048.1	chitooligosaccharide deacetylase [Lysobacter capsici AZ78]
OG_02888	LC55x_3417	LC55x_3417; acyl-CoA dehydrogenase, C-terminal domain protein	OG_02888	EYR66510.1	acyl-CoA dehydrogenase [Lysobacter capsici AZ78]
OG_02889	LC55x_2575	LC55x_2575; putative osmotically inducible lipoprotein B	OG_02889	EYR68318.1	transcriptional regulator [Lysobacter capsici AZ78]
OG_02890	LC55x_1219	LC55x_1219; acyl-CoA dehydrogenase, N-terminal domain protein	OG_02890	EYR65762.1	acyl-CoA dehydrogenase [Lysobacter capsici AZ78]
OG_02891	LC55x_3103	LC55x_3103; putative oxidoreductase	OG_02891	EYR65452.1	oxidoreductase [Lysobacter capsici AZ78]
OG_02892	LC55x_5145	rpmB; ribosomal protein L28	OG_02892	EYR68016.1	50S ribosomal protein L28 [Lysobacter capsici AZ78]
OG_02893	LC55x_2565	LC55x_2565; histidine phosphatase super family protein	OG_02893	EYR68328.1	hypothetical protein AZ78_11725 [Lysobacter capsici AZ78]
OG_02894	LC55x_187	LC55x_187; glucokinase family protein	OG_02894	EYR66128.1	hypothetical protein AZ78_22430 [Lysobacter capsici AZ78]
OG_02895	LC55x_3439	LC55x_3439; enoyl-CoA hydratase/isomerase family protein	OG_02895	EYR66492.1	3-hydroxyacyl-CoA dehydrogenase [Lysobacter capsici AZ78]
OG_02896	LC55x_3094	LC55x_3094; glycosyl transferases group 1 family protein	OG_02896	EYR65442.1	hypothetical protein AZ78_26210 [Lysobacter capsici AZ78]
OG_02897	LC55x_4673	LC55x_4673; conserved hypothetical protein	OG_02897	EYR70171.1	hypothetical protein AZ78_00210 [Lysobacter capsici AZ78]
OG_02898	LC55x_1705	ftsY; signal recognition particle-docking protein FtsY	OG_02898	EYR70363.1	cell division protein FtsY [Lysobacter capsici AZ78]
OG_02899	LC55x_4626	LC55x_4626; methylated-DNA-[]-cysteine S-methyltransferase family	OG_02899	EYR68338.1	AraC family transcriptional regulator [Lysobacter capsici AZ78]
OG_02900	LC55x_3916	LC55x_3916; conserved hypothetical protein	OG_02900	EYR66668.1	membrane protein [Lysobacter capsici AZ78]
OG_02901	LC55x_944	LC55x_944; phosphatidylethanolamine-binding family protein	OG_02901	EYR66818.1	hypothetical protein AZ78_18740 [Lysobacter capsici AZ78]
OG_02902	LC55x_1861	uppS; di-trans,poly-cis-decaprenylcistransferase	OG_02902	EYR66053.1	UDP pyrophosphate synthase [Lysobacter capsici AZ78]
OG_02903	LC55x_1321	raiA; ribosomal subunit interface protein	OG_02903	EYR69914.1	ribosome hibernation promoting factor HPF [Lysobacter capsici AZ78
OG_02904	LC55x_4168	LC55x_4168; POTRA domain, ShlB-type family protein	OG_02904	EYR68170.1	sugar transporter [Lysobacter capsici AZ78]
OG_02905	LC55x_5616	LC55x_5616; doxX family protein	OG_02905	EYR66256.1	hypothetical protein AZ78_22055 [Lysobacter capsici AZ78]
OG_02906	LC55x_531	LC55x_531; tonB dependent receptor family protein	OG_02906	EYR67892.1	hypothetical protein AZ78_12860 [Lysobacter capsici AZ78]
OG_02907	LC55x_4716	LC55x_4716; molybdopterin-binding domain of aldehyde dehydrogen	OG_02907	EYR65678.1	twin-arginine translocation pathway signal protein [Lysobacter capsic
OG_02908	LC55x_3666	LC55x_3666; conserved hypothetical protein	OG_02908	EYR65950.1	membrane protein [Lysobacter capsici AZ78]
OG_02909	LC55x_339	LC55x_339; conserved hypothetical protein	OG_02909	EYR68117.1	hypothetical protein AZ78_11865 [Lysobacter capsici AZ78]
OG_02910	LC55x_3398	LC55x_3398; aldehyde dehydrogenase family protein	OG_02910	EYR66521.1	aldehyde dehydrogenase [Lysobacter capsici AZ78]
OG_02911	LC55x_1222	LC55x_1222; RNA polymerase sigma factor, sigma-70 family protein	OG_02911	EYR65759.1	RNA polymerase sigma24 factor [Lysobacter capsici AZ78]
OG_02912	LC55x_3821	LC55x_3821; hypothetical protein	OG_02912	EYR67125.1	hypothetical protein AZ78_17845 [Lysobacter capsici AZ78]
OG_02913	LC55x_230	LC55x_230; bacterial regulatory, luxR family protein	OG_02913	EYR66169.1	hypothetical protein AZ78_22635 [Lysobacter capsici AZ78]
OG_02914	LC55x_3404	LC55x_3404; ABC transporter family protein	OG_02914	EYR66516.1	ABC transporter ATP-binding protein [Lysobacter capsici AZ78]
OG_02915	LC55x_254	LC55x_254; glycosyl transferases group 1 family protein	OG_02915	EYR66984.1	hypothetical protein AZ78_17880 [Lysobacter capsici AZ78]
OG_02916	LC55x_3275	LC55x_3275; 2OG-Fe(II) oxygenase superfamily protein	OG_02916	EYR70045.1	hypothetical protein AZ78_02700 [Lysobacter capsici AZ78]
OG_02917	LC55x_303	ntrC; nitrogen regulation protein NR	OG_02917	EYR67034.1	nitrogen regulation protein NR(I) [Lysobacter capsici AZ78]
OG_02918	LC55x_1714	upp; uracil phosphoribosyltransferase	OG_02918	EYR70369.1	uracil phosphoribosyltransferase [Lysobacter capsici AZ78]
OG_02919	LC55x_4051	LC55x_4051; 5'-3' exonuclease, C-terminal SAM fold family protein	OG_02919	EYR65830.1	exodeoxyribonuclease IX [Lysobacter capsici AZ78]
OG_02920	LC55x_4567	blaCTX-M-27; beta-lactamase CTX-M-27	OG_02920	EYR68374.1	beta-lactamase [Lysobacter capsici AZ78]
OG_02921	LC55x_4537	LC55x_4537; bacterial regulatory helix-turn-helix , lysR family protein	OG_02921	EYR68387.1	LysR family transcriptional regulator [Lysobacter capsici AZ78]
OG_02922	LC55x_2115	LC55x_2115; rhomboid family protein	OG_02922	EYR66391.1	membrane protein [Lysobacter capsici AZ78]
OG_02923	LC55x_1830	LC55x_1830; conserved hypothetical protein	OG_02923	EYR66023.1	membrane protein [Lysobacter capsici AZ78]
OG_02924	LC55x_4383	LC55x_4383; ABC-2 type transporter family protein	OG_02924	EYR69217.1	Wzm [Lysobacter capsici AZ78]
OG_02925	LC55x_1024	LC55x_1024; tonB dependent receptor family protein	OG_02925	EYR67756.1	TonB-denpendent receptor [Lysobacter capsici AZ78]
OG_02926	LC55x_3510	ppk1; polyphosphate kinase 1	OG_02926	EYR66636.1	polyphosphate kinase [Lysobacter capsici AZ78]
OG_02927	LC55x_4721	LC55x_4721; hypothetical protein	OG_02927	EYR65683.1	hypothetical protein AZ78_25110 [Lysobacter capsici AZ78]
OG_02928	LC55x_171	LC55x_171; conserved hypothetical protein	OG_02928	EYR65412.1	hypothetical protein AZ78_26445 [Lysobacter capsici AZ78]
OG_02929	LC55x_2269	LC55x_2269; NAD(P)H binding domain of trans-2-enoyl-CoA reducta	OG_02929	EYR69685.1	trans-2-enoyl-CoA reductase [Lysobacter capsici AZ78]
OG_02930	LC55x_1840	LC55x_1840; tic20-like family protein	OG_02930	EYR66033.1	membrane protein [Lysobacter capsici AZ78]
OG_02931	LC55x_1573	LC55x_1573; conserved hypothetical protein	OG_02931	EYR70292.1	membrane protein [Lysobacter capsici AZ78]
OG_02932	LC55x_3694	ispD; 4-diphosphocytidyl-2C-methyl-D-erythritol synthetase	OG_02932	EYR65924.1	2-C-methyl-D-erythritol 4-phosphate cytidylyltransferase [Lysobacter
OG_02933	LC55x_4906	LC55x_4906; N-acylglucosamine 2-epimerase family protein	OG_02933	EYR68707.1	N-acylglucosamine 2-epimerase [Lysobacter capsici AZ78]
OG_02934	LC55x_5133	xth; exodeoxyribonuclease III	OG_02934	EYR68026.1	exodeoxyribonuclease III [Lysobacter capsici AZ78]
OG_02935	LC55x_4273	mraY; phospho-N-acetylmuramoyl-pentapeptide- transferase	OG_02935	EYR69131.1	phospho-N-acetylmuramoyl-pentapeptide-transferase [Lysobacter ca
OG_02936	LC55x_5660	LC55x_5660; bacterial DNA-binding family protein	OG_02936	EYR66294.1	DNA-binding protein [Lysobacter capsici AZ78]
OG_02937	LC55x_2923	pgmB; beta-phosphoglucomutase	OG_02937	EYR66236.1	beta-phosphoglucomutase [Lysobacter capsici AZ78]
OG_02938	LC55x_2015	recA; protein RecA	OG_02938	EYR66743.1	recombinase RecA [Lysobacter capsici AZ78]
OG_02939	LC55x_5229	ribonuclease; ribonuclease	OG_02939	EYR68890.1	ribonuclease [Lysobacter capsici AZ78]
OG_02940	LC55x_5694	LC55x_5694; conserved hypothetical protein	OG_02940	EYR65864.1	hypothetical protein AZ78_24010 [Lysobacter capsici AZ78]
OG_02941	LC55x_284	LC55x_284; peptidase S51 family protein	OG_02941	EYR67011.1	hypothetical protein AZ78_18020 [Lysobacter capsici AZ78]
OG_02942	LC55x_2315	cmk; cytidylate kinase	OG_02942	EYR69649.1	cytidylate kinase [Lysobacter capsici AZ78]

OG_02943	LC55x_1594	LC55x_1594; metallo-beta-lactamase superfamily protein	OG_02943	EYR70305.1	lactamase [Lysobacter capsici AZ78]
OG_02944	LC55x_4147	LC55x_4147; tonB family C-terminal domain protein	OG_02944	EYR66334.1	cell envelope biogenesis protein TonB [Lysobacter capsici AZ78]
OG_02945	LC55x_3441	pilZ; type IV fimbriae assembly protein	OG_02945	EYR66588.1	pilus biogenesis protein PilZ [Lysobacter capsici AZ78]
OG_02946	LC55x_5218	LC55x_5218; thymidine kinase family protein	OG_02946	EYR68880.1	thymidine kinase [Lysobacter capsici AZ78]
OG_02947	LC55x_1606	LC55x_1606; transglycosylase SLT domain protein	OG_02947	EYR70313.1	transglycosylase [Lysobacter capsici AZ78]
OG_02948	LC55x_5668	LC55x_5668; amino acid permease family protein	OG_02948	EYR65886.1	hypothetical protein AZ78_24120 [Lysobacter capsici AZ78]
OG_02949	LC55x_2517	LC55x_2517; NUDIX hydrolase	OG_02949	EYR69501.1	7,8-dihydro-8-oxoguanine-triphosphatase [Lysobacter capsici AZ78]
OG_02950	LC55x_2493	LC55x_2493; alpha-1,2-mannosidase family protein	OG_02950	EYR69524.1	alpha-1 2-mannosidase [Lysobacter capsici AZ78]
OG_02951	LC55x_757	LC55x_757; methyltransferase small domain protein	OG_02951	EYR67632.1	16S rRNA methyltransferase [Lysobacter capsici AZ78]
OG_02952	LC55x_2824	metH; methionine synthase	OG_02952	EYR69385.1	5-methyltetrahydrofolate--homocysteine methyltransferase [Lysobact
OG_02953	LC55x_1774	LC55x_1774; conserved hypothetical protein	OG_02953	EYR67274.1	hypothetical protein AZ78_16140 [Lysobacter capsici AZ78]
OG_02954	LC55x_2942	LC55x_2942; carboxypeptidase regulatory-like domain protein	OG_02954	EYR66218.1	membrane protein [Lysobacter capsici AZ78]
OG_02955	LC55x_864	LC55x_864; CDP-alcohol phosphatidyltransferase family protein	OG_02955	EYR66893.1	hypothetical protein AZ78_19120 [Lysobacter capsici AZ78]
OG_02956	LC55x_5685	LC55x_5685; conserved hypothetical protein	OG_02956	EYR65873.1	hypothetical protein AZ78_24055 [Lysobacter capsici AZ78]
OG_02957	LC55x_1870	tsf; translation elongation factor Ts	OG_02957	EYR66063.1	endo-1,4-D-glucanase [Lysobacter capsici AZ78]
OG_02958	LC55x_3537	LC55x_3537; hypothetical protein	OG_02958	EYR67489.1	hypothetical protein AZ78_15195 [Lysobacter capsici AZ78]
OG_02959	LC55x_151	zwf; glucose-6-phosphate dehydrogenase	OG_02959	EYR69795.1	glucose-6-phosphate 1-dehydrogenase [Lysobacter capsici AZ78]
OG_02960	LC55x_4455	trpS; tryptophan--tRNA ligase	OG_02960	EYR68443.1	tryptophanyl-tRNA synthetase [Lysobacter capsici AZ78]
OG_02961	LC55x_2957	LC55x_2957; hydrolase	OG_02961	EYR66205.1	carbon-nitrogen hydrolase [Lysobacter capsici AZ78]
OG_02962	LC55x_1154	LC55x_1154; prolyl oligopeptidase family protein	OG_02962	EYR68524.1	glutamyl peptidase [Lysobacter capsici AZ78]
OG_02963	LC55x_354	LC55x_354; bacterial regulatory helix-turn-helix , lysR family protein	OG_02963	EYR68129.1	LysR family transcriptional regulator [Lysobacter capsici AZ78]
OG_02964	LC55x_1875	dapD; 2,3,4,5-tetrahydropyridine-2,6-dicarboxylate N-succinyltransfer	OG_02964	EYR66068.1	2,3,4,5-tetrahydropyridine-2,6-carboxylate N-succinyltransferase [Lys
OG_02965	LC55x_54	LC55x_54; impB/mucB/samB family protein	OG_02965	EYR69733.1	DNA polymerase IV [Lysobacter capsici AZ78]
OG_02966	LC55x_2475	LC55x_2475; transglutaminase-like superfamily protein	OG_02966	EYR69814.1	hypothetical protein AZ78_03360 [Lysobacter capsici AZ78]
OG_02967	LC55x_4370	LC55x_4370; electron transfer flavodomain protein	OG_02967	EYR69204.1	electron transfer flavoprotein subunit beta [Lysobacter capsici AZ78]
OG_02968	LC55x_4155	LC55x_4155; glycosyl transferase 2 family protein	OG_02968	EYR66326.1	hypothetical protein AZ78_21485 [Lysobacter capsici AZ78]
OG_02969	LC55x_5645	LC55x_5645; glutathionylspermidine synthase preATP-grasp family p	OG_02969	EYR66282.1	hypothetical protein AZ78_22190 [Lysobacter capsici AZ78]
OG_02970	LC55x_4424	rpe; ribulose-phosphate 3-epimerase	OG_02970	EYR65465.1	ribulose-phosphate 3-epimerase [Lysobacter capsici AZ78]
OG_02971	LC55x_2066	dcd; deoxycytidine triphosphate deaminase	OG_02971	EYR65402.1	deoxycytidine triphosphate deaminase [Lysobacter capsici AZ78]
OG_02972	LC55x_4948	priA; primosomal protein N'	OG_02972	EYR68673.1	primosome assembly protein PriA [Lysobacter capsici AZ78]
OG_02973	LC55x_1141	ilvE; branched-chain amino acid aminotransferase	OG_02973	EYR68515.1	branched-chain amino acid aminotransferase [Lysobacter capsici AZ
OG_02974	LC55x_2390	phaC; poly(R)-hydroxyalkanoic acid synthase, class III, PhaC subunit	OG_02974	EYR69598.1	poly-beta-hydroxybutyrate polymerase [Lysobacter capsici AZ78]
OG_02975	LC55x_1854	lpxA; acyl-[acyl-carrier-protein]-UDP-N- acetylglucosamine O-acyltrar	OG_02975	EYR66046.1	UDP-N-acetylglucosamine acyltransferase [Lysobacter capsici AZ78]
OG_02976	LC55x_3240	LC55x_3240; acetyltransferase family protein	OG_02976	EYR67941.1	acetyltransferase [Lysobacter capsici AZ78]
OG_02977	LC55x_1334	purN; phosphoribosylglycinamide formyltransferase	OG_02977	EYR69903.1	phosphoribosylglycinamide formyltransferase [Lysobacter capsici AZ
OG_02978	LC55x_4426	purC; phosphoribosylaminoimidazolesuccinocarboxamide synthase	OG_02978	EYR65467.1	phosphoribosylaminoimidazole-succinocarboxamide synthase [Lysob
OG_02979	LC55x_635	LC55x_635; conserved hypothetical protein	OG_02979	EYR69983.1	membrane protein [Lysobacter capsici AZ78]
OG_02980	LC55x_5418	LC55x_5418; response regulator	OG_02980	EYR69016.1	hypothetical protein AZ78_07680 [Lysobacter capsici AZ78]
OG_02981	LC55x_527	LC55x_527; tetratricopeptide repeat family protein	OG_02981	EYR67888.1	hypothetical protein AZ78_12840 [Lysobacter capsici AZ78]
OG_02982	LC55x_3423	LC55x_3423; CYTH domain protein	OG_02982	EYR66505.1	adenylate cyclase [Lysobacter capsici AZ78]
OG_02983	LC55x_1265	LC55x_1265; prokaryotic cytochrome b561 family protein	OG_02983	EYR67679.1	cytochrome B561 [Lysobacter capsici AZ78]
OG_02984	LC55x_2573	ligD; DNA ligase D	OG_02984	EYR68320.1	hypothetical protein AZ78_11685 [Lysobacter capsici AZ78]
OG_02985	LC55x_55	LC55x_55; acetyltransferase domain protein	OG_02985	EYR69870.1	hypothetical protein AZ78_04695 [Lysobacter capsici AZ78]
OG_02986	LC55x_3339	LC55x_3339; peptidase M48 family protein	OG_02986	EYR66550.1	peptidase [Lysobacter capsici AZ78]
OG_02987	LC55x_1210	wrbA; quinone oxidoreductase, type IV	OG_02987	EYR65775.1	NAD(P)H quinone oxidoreductase [Lysobacter capsici AZ78]
OG_02988	LC55x_1110	LC55x_1110; fumarylacetoacetate (FAA) hydrolase family protein	OG_02988	EYR68473.1	fumarylacetoacetase [Lysobacter capsici AZ78]
OG_02989	LC55x_2070	LC55x_2070; conserved hypothetical protein	OG_02989	EYR65398.1	hypothetical protein AZ78_26480 [Lysobacter capsici AZ78]
OG_02990	LC55x_5042	LC55x_5042; prolyl oligopeptidase family protein	OG_02990	EYR66298.1	peptidase S9 [Lysobacter capsici AZ78]
OG_02992	LC55x_3988	ppk2; polyphosphate kinase 2	OG_02992	EYR66718.1	polyphosphate kinase [Lysobacter capsici AZ78]
OG_02993	LC55x_738	LC55x_738; polysaccharide deacetylase family protein	OG_02993	EYR67646.1	polysaccharide deacetylase [Lysobacter capsici AZ78]
OG_02994	LC55x_2789	LC55x_2789; conserved hypothetical protein	OG_02994	EYR69353.1	hypothetical protein AZ78_05525 [Lysobacter capsici AZ78]
OG_02995	LC55x_2404	LC55x_2404; hypothetical protein	OG_02995	EYR69827.1	hypothetical protein AZ78_03715 [Lysobacter capsici AZ78]
OG_02996	LC55x_3770	LC55x_3770; NAD-dependent epimerase/dehydratase	OG_02996	EYR67080.1	3-beta hydroxysteroid dehydrogenase [Lysobacter capsici AZ78]
OG_02997	LC55x_2803	LC55x_2803; exonuclease family protein	OG_02997	EYR69367.1	exonuclease I [Lysobacter capsici AZ78]
OG_02998	LC55x_3132	ampR; HTH-type transcriptional activator AmpR	OG_02998	EYR66961.1	LysR family transcriptional regulator [Lysobacter capsici AZ78]
OG_02999	LC55x_4454	LC55x_4454; putative transmembrane protein	OG_02999	EYR68444.1	membrane protein [Lysobacter capsici AZ78]
OG_03000	LC55x_1871	rpsB; ribosomal protein S2	OG_03000	EYR66064.1	30S ribosomal protein S2 [Lysobacter capsici AZ78]
OG_03001	LC55x_5189	LC55x_5189; alpha/beta hydrolase fold family protein	OG_03001	EYR68861.1	hydrolase [Lysobacter capsici AZ78]
OG_03002	LC55x_4230	cobO; cob(I)yrinic acid a,c-diamide adenosyltransferase	OG_03002	EYR69091.1	cob(I)alamin adenolsyltransferase/cobinamide ATP-dependent adeno
OG_03003	LC55x_2102	LC55x_2102; hypothetical protein	OG_03003	EYR66403.1	hypothetical protein AZ78_21320 [Lysobacter capsici AZ78]
OG_03004	LC55x_1737	LC55x_1737; conserved hypothetical protein	OG_03004	EYR70385.1	pit accessory protein [Lysobacter capsici AZ78]
OG_03005	LC55x_2647	LC55x_2647; glycosyl transferases group 1 family protein	OG_03005	EYR68251.1	glycosyl transferase [Lysobacter capsici AZ78]
OG_03006	LC55x_5143	LC55x_5143; lipid A Biosynthesis N-terminal domain protein	OG_03006	EYR68018.1	membrane protein [Lysobacter capsici AZ78]
OG_03007	LC55x_507	LC55x_507; S-adenosyl-L-methionine-dependent methyltransferase f	OG_03007	EYR66437.1	hypothetical protein AZ78_20830 [Lysobacter capsici AZ78]
OG_03008	LC55x_3434	lepB; signal peptidase I	OG_03008	EYR66497.1	signal peptidase [Lysobacter capsici AZ78]
OG_03009	LC55x_4470	LC55x_4470; HAMP domain protein	OG_03009	EYR68432.1	histidine kinase [Lysobacter capsici AZ78]
OG_03010	LC55x_3499	gloB; hydroxyacylglutathione hydrolase	OG_03010	EYR66628.1	hydroxyacylglutathione hydrolase [Lysobacter capsici AZ78]
OG_03011	LC55x_550	LC55x_550; acrB/AcrD/AcrF family protein	OG_03011	EYR67911.1	multidrug transporter [Lysobacter capsici AZ78]
OG_03012	LC55x_1946	ileS; isoleucine--tRNA ligase	OG_03012	EYR65962.1	isoleucyl-tRNA synthetase [Lysobacter capsici AZ78]
OG_03013	LC55x_854	purD; phosphoribosylamine--glycine ligase	OG_03013	EYR66902.1	phosphoribosylamine--glycine ligase [Lysobacter capsici AZ78]
OG_03014	LC55x_2159	LC55x_2159; cytochrome C' family protein	OG_03014	EYR67379.1	hypothetical protein AZ78_15645 [Lysobacter capsici AZ78]
OG_03015	LC55x_4465	plsY; acyl-phosphate glycerol 3-phosphate acyltransferase	OG_03015	EYR68435.1	membrane protein [Lysobacter capsici AZ78]
OG_03016	LC55x_1354	purM; phosphoribosylformylglycinamidine cyclo-ligase	OG_03016	EYR69894.1	phosphoribosylaminoimidazole synthetase [Lysobacter capsici AZ78]
OG_03017	LC55x_934	LC55x_934; ACT domain protein	OG_03017	EYR66827.1	hypothetical protein AZ78_18785 [Lysobacter capsici AZ78]
OG_03018	LC55x_350	LC55x_350; conserved hypothetical protein	OG_03018	EYR68126.1	sulfatase-modifying factor protein [Lysobacter capsici AZ78]
OG_03019	LC55x_2374	LC55x_2374; hypothetical protein	OG_03019	EYR69835.1	hypothetical protein AZ78_03850 [Lysobacter capsici AZ78]
OG_03020	LC55x_3443	tmk; thymidylate kinase	OG_03020	EYR66590.1	thymidylate kinase [Lysobacter capsici AZ78]
OG_03021	LC55x_4114	LC55x_4114; ABC transporter family protein	OG_03021	EYR66365.1	macrolide ABC transporter ATP-binding protein [Lysobacter capsici A
OG_03022	LC55x_1138	LC55x_1138; alpha-L-glutamate ligases, RimK family protein	OG_03022	EYR68512.1	ribosomal protein S6 modification protein [Lysobacter capsici AZ78]
OG_03023	LC55x_894	groES; 10 kDa chaperonin	OG_03023	EYR66863.1	molecular chaperone GroES [Lysobacter capsici AZ78]
OG_03024	LC55x_114	LC55x_114; ftsX-like permease family protein	OG_03024	EYR69773.1	ABC transporter permease [Lysobacter capsici AZ78]

OG_03025	LC55x_2788	LC55x_2788; AAA domain family protein
OG_03026	LC55x_2837	LC55x_2837; glutathione peroxidase family protein
OG_03027	LC55x_2027	kdpA; K+-transporting ATPase, A subunit
OG_03028	LC55x_3485	moaC; molybdenum cofactor biosynthesis protein C
OG_03029	LC55x_4358	LC55x_4358; pyridine nucleotide-disulfide oxidoreductase family prot
OG_03030	LC55x_1782	LC55x_1782; putative translation elongation factor P
OG_03031	LC55x_1168	LC55x_1168; pepSY-associated TM helix family protein
OG_03032	LC55x_3425	rumA; 23S rRNA (uracil-5-)-methyltransferase RumA
OG_03033	LC55x_320	LC55x_320; rhodanese-like domain protein
OG_03034	LC55x_4357	LC55x_4357; 2OG-Fe(II) oxygenase superfamily protein
OG_03035	LC55x_2350	guaB; inosine-5'-monophosphate dehydrogenase
OG_03036	LC55x_3509	phoR; phosphate regulon sensor kinase PhoR
OG_03037	LC55x_3577	LC55x_3577; conserved hypothetical protein
OG_03038	LC55x_3864	LC55x_3864; pseudouridine synthase family protein
OG_03039	LC55x_2017	alaS; alanine--tRNA ligase
OG_03040	LC55x_3807	LC55x_3807; acetyltransferase family protein
OG_03041	LC55x_5092	LC55x_5092; putative ketosynthase
OG_03042	LC55x_968	LC55x_968; pyrophosphate-dependent phosphofructokinase
OG_03043	LC55x_2226	LC55x_2226; polysaccharide biosynthesis family protein
OG_03044	LC55x_4766	LC55x_4766; 4-hydroxyproline epimerase
OG_03045	LC55x_3291	LC55x_3291; hydrolase CocE/NonD family protein
OG_03046	LC55x_1670	pheT; phenylalanine--tRNA ligase, beta subunit
OG_03047	LC55x_5034	LC55x_5034; tonB family C-terminal domain protein
OG_03048	LC55x_5187	LC55x_5187; conserved hypothetical protein
OG_03049	LC55x_3117	chiG; secreted chitinase domain protein
OG_03050	LC55x_782	atpC; ATP synthase F1, epsilon subunit
OG_03051	LC55x_201	LC55x_201; META domain protein
OG_03052	LC55x_3018	secG; preprotein translocase, SecG subunit
OG_03053	LC55x_743	LC55x_743; killing trait family protein
OG_03054	LC55x_2391	LC55x_2391; GDSL-like Lipase/Acylhydrolase family protein
OG_03056	LC55x_3187	LC55x_3187; YXWGXW repeat family protein
OG_03057	LC55x_1296	LC55x_1296; twitching motility family protein
OG_03058	LC55x_180	LC55x_180; ankyrin repeat family protein
OG_03059	LC55x_1931	LC55x_1931; enoyl-CoA hydratase/isomerase family protein
OG_03060	LC55x_3892	LC55x_3892; conserved hypothetical protein
OG_03061	LC55x_4043	LC55x_4043; conserved hypothetical protein
OG_03062	LC55x_1282	LC55x_1282; dodecin family protein
OG_03063	LC55x_2527	aat; leucyl/phenylalanyl-tRNA--protein transferase
OG_03064	LC55x_3826	LC55x_3826; methylated-DNA-[]-cysteine S-methyltransferase family
OG_03065	LC55x_1672	LC55x_1672; merR regulatory family protein
OG_03066	LC55x_1371	LC55x_1371; transglycosylase associated family protein
OG_03067	LC55x_183	LC55x_183; response regulator
OG_03068	LC55x_2870	sucB; dihydrolipoyllysine-residue succinyltransferase, E2 component
OG_03069	LC55x_5550	LC55x_5550; conserved hypothetical protein
OG_03070	LC55x_4722	LC55x_4722; bacterial regulatory helix-turn-helix , lysR family protein
OG_03071	LC55x_2449	LC55x_2449; enoyl-CoA hydratase/isomerase family protein
OG_03072	LC55x_4388	uptE; outer membrane domain protein
OG_03073	LC55x_1846	accA; acetyl-CoA carboxylase, carboxyl transferase, alpha subunit
OG_03074	LC55x_5716	LC55x_5716; hypothetical protein
OG_03075	LC55x_4389	LC55x_4389; conserved hypothetical protein
OG_03076	LC55x_3114	LC55x_3114; hypothetical protein
OG_03077	LC55x_1957	LC55x_1957; glutaredoxin family protein
OG_03078	LC55x_2010	LC55x_2010; tonB dependent receptor family protein
OG_03079	LC55x_4385	cysA; cystathionine gamma-lyase
OG_03080	LC55x_3369	ALDH7A1; alpha-aminoadipic semialdehyde dehydrogenase
OG_03081	LC55x_2081	LC55x_2081; conserved hypothetical protein
OG_03082	LC55x_3458	LC55x_3458; AAA domain family protein
OG_03083	LC55x_1798	nadD; nicotinate (nicotinamide) nucleotide adenylyltransferase
OG_03084	LC55x_1238	LC55x_1238; asmA family protein
OG_03085	LC55x_3953	rplC; 50S ribosomal protein L3
OG_03086	LC55x_1936	LC55x_1936; ompA family protein

OG_03025	EYR69351.1	ATPase AAA [Lysobacter capsici AZ78]
OG_03026	EYR69398.1	vitamin B12 ABC transporter permease [Lysobacter capsici AZ78]
OG_03027	EYR65373.1	potassium-transporting ATPase subunit A [Lysobacter capsici AZ78]
OG_03028	EYR66617.1	molybdenum cofactor biosynthesis protein MoaC [Lysobacter capsici
OG_03029	EYR69196.1	electron transfer flavoprotein [Lysobacter capsici AZ78]
OG_03030	EYR67278.1	translation elongation factor P (EF-P) [Lysobacter capsici AZ78]
OG_03031	EYR68536.1	peptidase [Lysobacter capsici AZ78]
OG_03032	EYR66504.1	23S rRNA methyltransferase [Lysobacter capsici AZ78]
OG_03033	EYR68103.1	membrane protein [Lysobacter capsici AZ78]
OG_03034	EYR69195.1	DNA methylase [Lysobacter capsici AZ78]
OG_03035	EYR69622.1	inosine 5'-monophosphate dehydrogenase [Lysobacter capsici AZ78]
OG_03036	EYR66635.1	histidine kinase [Lysobacter capsici AZ78]
OG_03037	EYR67525.1	hypothetical protein AZ78_15375 [Lysobacter capsici AZ78]
OG_03038	EYR65543.1	hypothetical protein AZ78_25685 [Lysobacter capsici AZ78]
OG_03039	EYR66741.1	alanyl-tRNA synthetase [Lysobacter capsici AZ78]
OG_03040	EYR67112.1	GCN5 family acetyltransferase [Lysobacter capsici AZ78]
OG_03041	EYR68056.1	ketosynthase [Lysobacter capsici AZ78]
OG_03042	EYR66797.1	6-phosphofructokinase [Lysobacter capsici AZ78]
OG_03043	EYR67422.1	NAD-dependent epimerase [Lysobacter capsici AZ78]
OG_03044	EYR66081.1	hydroxyproline-2-epimerase [Lysobacter capsici AZ78]
OG_03045	EYR70055.1	peptidase S15 [Lysobacter capsici AZ78]
OG_03046	EYR70341.1	phenylalanyl-tRNA synthetase subunit beta [Lysobacter capsici AZ78
OG_03047	EYR66305.1	hypothetical protein AZ78_22305 [Lysobacter capsici AZ78]
OG_03048	EYR68859.1	hypothetical protein AZ78_07285 [Lysobacter capsici AZ78]
OG_03049	EYR66977.1	chitinase [Lysobacter capsici AZ78]
OG_03050	EYR67608.1	F0F1 ATP synthase subunit epsilon [Lysobacter capsici AZ78]
OG_03051	EYR66141.1	hypothetical protein AZ78_22495 [Lysobacter capsici AZ78]
OG_03052	EYR67815.1	preprotein translocase subunit SecG [Lysobacter capsici AZ78]
OG_03053	EYR67641.1	RebB like protein [Lysobacter capsici AZ78]
OG_03054	EYR69597.1	lipase/acylhydrolase [Lysobacter capsici AZ78]
OG_03056	EYR66910.1	hypothetical protein AZ78_18225 [Lysobacter capsici AZ78]
OG_03057	EYR67658.1	twitching motility protein PilT [Lysobacter capsici AZ78]
OG_03058	EYR66122.1	hypothetical protein AZ78_22400 [Lysobacter capsici AZ78]
OG_03059	EYR65972.1	enoyl-CoA hydratase [Lysobacter capsici AZ78]
OG_03060	EYR65724.1	methylcitrate synthase [Lysobacter capsici AZ78]
OG_03061	EYR65822.1	NAD(P) transhydrogenase [Lysobacter capsici AZ78]
OG_03062	EYR67668.1	hypothetical protein AZ78_15015 [Lysobacter capsici AZ78]
OG_03063	EYR69497.1	leucyl/phenylalanyl-tRNA--protein transferase [Lysobacter capsici AZ
OG_03064	EYR65753.1	methylated-DNA--protein-cysteine methyltransferase [Lysobacter cap
OG_03065	EYR70343.1	MerR family transcriptional regulator [Lysobacter capsici AZ78]
OG_03066	EYR67193.1	transglycosylase [Lysobacter capsici AZ78]
OG_03067	EYR66124.1	hypothetical protein AZ78_22410 [Lysobacter capsici AZ78]
OG_03068	EYR69429.1	dihydrolipoamide succinyltransferase [Lysobacter capsici AZ78]
OG_03069	EYR68979.1	Membrane-bound lytic murein transglycosylase [Lysobacter capsici A
OG_03070	EYR65684.1	hypothetical protein AZ78_25115 [Lysobacter capsici AZ78]
OG_03071	EYR69554.1	enoyl-CoA hydratase [Lysobacter capsici AZ78]
OG_03072	EYR69221.1	membrane protein [Lysobacter capsici AZ78]
OG_03073	EYR66040.1	acetyl-CoA carboxylase subunit alpha [Lysobacter capsici AZ78]
OG_03074	EYR69855.1	hypothetical protein AZ78_04390 [Lysobacter capsici AZ78]
OG_03075	EYR69222.1	membrane protein [Lysobacter capsici AZ78]
OG_03076	EYR66979.1	hypothetical protein AZ78_18585 [Lysobacter capsici AZ78]
OG_03077	EYR65957.1	glutaredoxin [Lysobacter capsici AZ78]
OG_03078	EYR66748.1	TonB-denpendent receptor [Lysobacter capsici AZ78]
OG_03079	EYR69219.1	cystathionine beta-lyase [Lysobacter capsici AZ78]
OG_03080	EYR66533.1	aldehyde dehydrogenase [Lysobacter capsici AZ78]
OG_03081	EYR66422.1	histidine triad (HIT) protein [Lysobacter capsici AZ78]
OG_03082	EYR66603.1	ATPase AAA [Lysobacter capsici AZ78]
OG_03083	EYR67287.1	nicotinic acid mononucleotide adenylyltransferase [Lysobacter capsic
OG_03084	EYR65635.1	membrane protein [Lysobacter capsici AZ78]
OG_03085	EYR66704.1	50S ribosomal protein L3 [Lysobacter capsici AZ78]
OG_03086	EYR66011.1	hypothetical protein AZ78_23340 [Lysobacter capsici AZ78]

Orthologous group	*L. gum* 3.2.11<br>GeneID (#3185)<br>54.3% of genome	Gene description
OG_00172	LG3211_3795	LG3211_3795; putative acetyltransferase
OG_00176	LG3211_3411	LG3211_3411; ABC transporter family protein
OG_00177	LG3211_1873	gph; phosphoglycolate phosphatase, bacterial
OG_00178	LG3211_2704	LG3211_2704; FKBP-type peptidyl-prolyl cis-trans isomerase family p
OG_00179	LG3211_1409	LG3211_1409; curli production assembly/transport component CsgG
OG_00180	LG3211_4020	LG3211_4020; cobQ/CobB/MinD/ParA nucleotide binding domain pro
OG_00181	LG3211_224	LG3211_224; flagellar assembly FliH family protein
OG_00182	LG3211_493	LG3211_493; membrane dipeptidase family protein
OG_00183	LG3211_5148	LG3211_5148; TPR repeat family protein
OG_00184	LG3211_5331	LG3211_5331; amidohydrolase family protein
OG_00185	LG3211_1107	LG3211_1107; beta-lactamase family protein
OG_00186	LG3211_84	LG3211_84; vacJ like lipofamily protein
OG_00187	LG3211_1382	LG3211_1382; major Facilitator Superfamily protein
OG_00188	LG3211_4109	pyrF; orotidine 5'-phosphate decarboxylase
OG_00189	LG3211_2109	LG3211_2109; peptidase S41 family protein
OG_00190	LG3211_1461	LG3211_1461; conserved hypothetical protein
OG_00191	LG3211_860	LG3211_860; efflux transporter, RND family, MFP subunit
OG_00192	LG3211_842	LG3211_842; multicopper oxidase family protein
OG_00193	LG3211_2719	LG3211_2719; araC-like ligand binding domain protein
OG_00194	LG3211_4213	LG3211_4213; indole-3-glycerol phosphate synthase family protein
OG_00195	LG3211_1267	hda; dnaA regulatory inactivator Hda
OG_00196	LG3211_697	LG3211_697; conserved hypothetical protein
OG_00197	LG3211_3125	LG3211_3125; major Facilitator Superfamily protein
OG_00198	LG3211_1945	hisG; ATP phosphoribosyltransferase
OG_00199	LG3211_2787	LG3211_2787; conserved hypothetical protein
OG_00200	LG3211_2318	LG3211_2318; putative phosphotransferase
OG_00201	LG3211_557	LG3211_557; conserved hypothetical protein
OG_00202	LG3211_921	thiE; thiamine-phosphate pyrophosphorylase
OG_00203	LG3211_558	LG3211_558; cupin domain protein
OG_00204	LG3211_1295	LG3211_1295; acetyltransferase family protein
OG_00205	LG3211_2783	LG3211_2783; scpA/B family protein
OG_00206	LG3211_5139	LG3211_5139; KNTase C-terminal domain protein
OG_00207	LG3211_964	recG; ATP-dependent DNA helicase RecG
OG_00208	LG3211_1878	LG3211_1878; peptidase M23 family protein
OG_00209	LG3211_407	LG3211_407; alpha/beta hydrolase fold family protein
OG_00210	LG3211_2707	LG3211_2707; bacterial regulatory, gntR family protein
OG_00211	LG3211_1034	mscL; large conductance mechanosensitive channel protein
OG_00212	LG3211_4963	tal; transaldolase
OG_00213	LG3211_932	rpiA; ribose 5-phosphate isomerase A
OG_00214	LG3211_3541	thrS; threonine--tRNA ligase
OG_00215	LG3211_5060	LG3211_5060; fabA-like domain protein
OG_00216	LG3211_2414	LG3211_2414; low affinity iron permease family protein
OG_00217	LG3211_500	LG3211_500; putative peptidase
OG_00218	LG3211_778	LG3211_778; beta-lactamase family protein
OG_00219	LG3211_2926	LG3211_2926; MMPL family protein
OG_00220	LG3211_463	LG3211_463; conserved hypothetical protein
OG_00221	LG3211_1765	LG3211_1765; cold-shock domain protein
OG_00222	LG3211_3862	pip; prolyl aminopeptidase
OG_00223	LG3211_3136	LG3211_3136; conserved hypothetical protein
OG_00224	LG3211_1484	LG3211_1484; nlpC/P60 family protein
OG_00225	LG3211_4494	LG3211_4494; hypothetical protein
OG_00226	LG3211_67	LG3211_67; cytochrome oxidase complex assembly 1 family protein
OG_00227	LG3211_3706	LG3211_3706; conserved hypothetical protein
OG_00228	LG3211_5179	LG3211_5179; hypothetical protein
OG_00229	LG3211_1173	hemE; uroporphyrinogen decarboxylase
OG_00230	LG3211_5348	LG3211_5348; pseudouridine synthase family protein
OG_00231	LG3211_3899	dhfrIII; dihydrofolate reductase type 3
OG_00232	LG3211_2641	dnaB; replicative DNA helicase
OG_00233	LG3211_3737	LG3211_3737; disulfide bond formation DsbB family protein
OG_00234	LG3211_5174	LG3211_5174; PP-loop family protein
OG_00235	LG3211_4628	LG3211_4628; polysaccharide biosynthesis/export family protein
OG_00236	LG3211_1290	LG3211_1290; conserved hypothetical protein
OG_00237	LG3211_1966	LG3211_1966; alpha/beta hydrolase family protein
OG_00238	LG3211_551	LG3211_551; GMC oxidoreductase family protein
OG_00239	LG3211_4343	LG3211_4343; glutathione S-transferase, N-terminal domain protein
OG_00240	LG3211_4522	LG3211_4522; bacterial regulatory, luxR family protein
OG_00241	LG3211_1460	LG3211_1460; tonB dependent receptor family protein
OG_00242	LG3211_5311	LG3211_5311; cytochrome c family protein
OG_00243	LG3211_4540	LG3211_4540; beta-lactamase family protein
OG_00244	LG3211_4479	hslV; ATP-dependent protease HslVU, peptidase subunit
OG_00245	LG3211_2702	LG3211_2702; nucleotide sugar dehydrogenase family protein
OG_00246	LG3211_3075	LG3211_3075; patatin-like phospholipase family protein
OG_00247	LG3211_2397	LG3211_2397; polyamine ABC transporter, ATP-binding family protei
OG_00248	LG3211_3703	LG3211_3703; polyprenyl synthetase family protein
OG_00249	LG3211_1690	LG3211_1690; na+/H+ antiporter family protein
OG_00250	LG3211_337	LG3211_337; putative thioesterase domain protein

Orthologous group	*L. enz* C3<br>GeneID (#3170)<br>52.1% of genome	Gene description
OG_00172	LEC3_3816	acetyltransferase (GNAT) family
OG_00176	LEC3_3471	ABC transporter, ATP-binding protein
OG_00177	LEC3_1818	phosphoglycolate phosphatase
OG_00178	LEC3_2705	domain amino terminal to FKBP-type peptidyl-prolyl isomerase/peptid
OG_00179	LEC3_1374	conserved hypothetical protein
OG_00180	LEC3_4088	cobyrinic Acid a,c-diamide synthase
OG_00181	LEC3_5106	type III secretion apparatus protein
OG_00182	LEC3_0305	renal dipeptidase
OG_00183	LEC3_0140	serine/threonine-protein kinase Pkn5
OG_00184	LEC3_5397	thermostable carboxypeptidase 1
OG_00185	LEC3_1080	beta-lactamase
OG_00186	LEC3_5238	lipoprotein VacJ precusor
OG_00187	LEC3_1351	transporter, major facilitator family
OG_00188	LEC3_4166	orotidine 5'-phosphate decarboxylase
OG_00189	LEC3_2048	peptidase, S41 family
OG_00190	LEC3_1427	lipoprotein
OG_00191	LEC3_0810	efflux transporter, RND family, MFP subunit
OG_00192	LEC3_0790	multicopper oxidase
OG_00193	LEC3_2719	cupin superfamily protein
OG_00194	LEC3_4270	indole-3-glycerol phosphate synthase
OG_00195	LEC3_1223	DnaA regulatory inactivator Hda
OG_00196	LEC3_0665	conserved hypothetical protein
OG_00197	LEC3_3129	transporter, major facilitator family
OG_00198	LEC3_1897	ATP phosphoribosyltransferase
OG_00199	LEC3_2793	response regulator receiver domain protein
OG_00200	LEC3_2241	regulatory protein
OG_00201	LEC3_4822	conserved hypothetical protein
OG_00202	LEC3_0874	thiamine-phosphate pyrophosphorylase
OG_00203	LEC3_4821	cupin superfamily protein
OG_00204	LEC3_1255	acetyltransferase (GNAT) family
OG_00205	LEC3_2790	segregation and condensation protein A
OG_00206	LEC3_2537	aminoglycoside 4'-O-nucleotidyltransferase, putative
OG_00207	LEC3_0921	ATP-dependent DNA helicase
OG_00208	LEC3_1823	peptidase
OG_00209	LEC3_0232	hydrolase, alpha/beta hydrolase fold family protein
OG_00210	LEC3_2708	transcriptional regulator, GntR family
OG_00211	LEC3_1000	large conductance mechanosensitive channel protein
OG_00212	LEC3_4873	transaldolase
OG_00213	LEC3_0882	ribose 5-phosphate isomerase A
OG_00214	LEC3_3570	threonyl-tRNA synthetase
OG_00215	LEC3_4976	conserved hypothetical protein
OG_00216	LEC3_2359	conserved hypothetical protein
OG_00217	LEC3_0310	O-sialoglycoprotein endopeptidase
OG_00218	LEC3_2442	beta-lactamase
OG_00219	LEC3_2937	acriflavin resistance protein (frameshift)
OG_00220	LEC3_0271	conserved hypothetical protein
OG_00221	LEC3_1721	'Cold-shock' DNA-binding domain protein
OG_00222	LEC3_3881	proline iminopeptidase
OG_00223	LEC3_3140	conserved hypothetical protein
OG_00224	LEC3_1460	lipoprotein
OG_00225	LEC3_4546	hypothetical protein
OG_00226	LEC3_5273	conserved hypothetical protein
OG_00227	LEC3_3731	conserved hypothetical protein
OG_00228	LEC3_0110	hypothetical protein
OG_00229	LEC3_1147	uroporphyrinogen decarboxylase
OG_00230	LEC3_5373	ribosomal large subunit pseudouridine synthase E
OG_00231	LEC3_3918	dihydrofolate reductase
OG_00232	LEC3_2647	replicative DNA helicase
OG_00233	LEC3_3764	disulfide bond formation protein DsbB
OG_00234	LEC3_0114	conserved hypothetical protein
OG_00235	LEC3_2261	polysaccharide biosynthesis/export protein
OG_00236	LEC3_1246	conserved hypothetical protein
OG_00237	LEC3_1918	PGAP1 family protein
OG_00238	LEC3_4831	FAD dependent oxidoreductase
OG_00239	LEC3_4387	glutathione S-transferase, N- domain
OG_00240	LEC3_4581	transcriptional regulator, LuxR family
OG_00241	LEC3_1426	TonB-dependent receptor
OG_00242	LEC3_5427	cytochrome C5
OG_00243	LEC3_4595	beta-lactamase
OG_00244	LEC3_4531	ATP-dependent protease hslV
OG_00245	LEC3_2703	udp-glucose 6-dehydrogenase
OG_00246	LEC3_3066	patatin
OG_00247	LEC3_2342	putrescine transport ATP-binding protein potG
OG_00248	LEC3_3728	polyprenyl synthetase
OG_00249	LEC3_1656	Na+/H+ antiporter family
OG_00250	LEC3_5034	thioesterase domain

OG_00251	LG3211_3018	LG3211_3018; short chain dehydrogenase family protein	OG_00251	LEC3_3011	conserved hypothetical protein
OG_00252	LG3211_3101	LG3211_3101; conserved hypothetical protein	OG_00252	LEC3_3103	conserved hypothetical protein
OG_00253	LG3211_3224	LG3211_3224; MATE efflux family protein	OG_00253	LEC3_3248	MATE efflux family protein
OG_00254	LG3211_4100	ribD; riboflavin biosynthesis protein RibD	OG_00254	LEC3_4156	riboflavin biosynthesis protein RibD
OG_00255	LG3211_3959	hrpA; ATP-dependent helicase HrpA	OG_00255	LEC3_3982	ATP dependent RNA helicase
OG_00256	LG3211_1278	cysI; sulfite reductase (NADPH) hemoprotein, beta-component	OG_00256	LEC3_1234	sulfite reductase (NADPH) hemoprotein, beta-component
OG_00257	LG3211_489	LG3211_489; sodium Bile acid symporter family protein	OG_00257	LEC3_0301	sodium bile acid symporter family
OG_00258	LG3211_3440	tlyC; hemolysin	OG_00258	LEC3_3503	hemolysin
OG_00259	LG3211_1694	LG3211_1694; RNase H family protein	OG_00259	LEC3_1660	Ribonuclease H
OG_00260	LG3211_5136	LG3211_5136; ABC transporter family protein	OG_00260	LEC3_0149	ABC transporter, ATP-binding protein
OG_00261	LG3211_1319	LG3211_1319; hypothetical protein	OG_00261	LEC3_1274	lipoprotein
OG_00262	LG3211_2928	LG3211_2928; cytochrome c family protein	OG_00262	LEC3_2939	cytochrome C family protein
OG_00263	LG3211_2020	LG3211_2020; ATP-grasp domain protein	OG_00263	LEC3_1950	methylcrotonoyl-CoA carboxylase subunit alpha
OG_00264	LG3211_312	arsH; arsenical resistance protein ArsH	OG_00264	LEC3_3688	arsenical resistance protein ArsH
OG_00265	LG3211_688	LG3211_688; cyclic nucleotide-binding domain protein	OG_00265	LEC3_4661	cyclic nucleotide-binding domain
OG_00266	LG3211_712	atpB; ATP synthase F0, A subunit	OG_00266	LEC3_0683	ATP synthase F0, A subunit
OG_00267	LG3211_3885	LG3211_3885; ubiquinone biosynthesis hydroxylase, UbiH/UbiF/VisC	OG_00267	LEC3_3903	2-octaprenyl-3-methyl-5-hydroxy-6-methoxy-1,4-be nzoquinol
OG_00268	LG3211_1240	LG3211_1240; lipopolysaccharide-assembly, LptC-related family prot	OG_00268	LEC3_1195	conserved hypothetical protein
OG_00269	LG3211_506	LG3211_506; tonB dependent receptor family protein	OG_00269	LEC3_0319	TonB-dependent receptor
OG_00270	LG3211_2304	hrcA; heat-inducible transcription repressor HrcA	OG_00270	LEC3_2230	heat-inducible transcription repressor HrcA
OG_00271	LG3211_2095	LG3211_2095; conserved hypothetical protein	OG_00271	LEC3_2033	conserved hypothetical protein
OG_00272	LG3211_488	LG3211_488; xaa-Pro dipeptidase, putative	OG_00272	LEC3_0297	amidohydrolase family protein
OG_00273	LG3211_4964	LG3211_4964; FAD binding domain protein	OG_00273	LEC3_4874	NADH dehydrogenase
OG_00274	LG3211_4546	ykfB; L-Ala-D/L-Glu epimerase	OG_00274	LEC3_4601	chloromuconate cycloisomerase
OG_00275	LG3211_220	LG3211_220; conserved hypothetical protein	OG_00275	LEC3_5110	conserved hypothetical protein
OG_00276	LG3211_4630	etk; tyrosine-protein kinase etk	OG_00276	LEC3_2258	tyrosine-protein kinase
OG_00277	LG3211_4297	LG3211_4297; hypothetical protein	OG_00277	LEC3_4357	hypothetical protein
OG_00278	LG3211_3769	rpoC; DNA-directed RNA polymerase, beta' subunit	OG_00278	LEC3_3796	DNA-directed RNA polymerase, beta' subunit
OG_00280	LG3211_1538	hflC; hflC protein	OG_00280	LEC3_1513	HflC protein
OG_00281	LG3211_2766	LG3211_2766; ABC transporter family protein	OG_00281	LEC3_2773	ABC transporter, ATP-binding protein
OG_00282	LG3211_3321	LG3211_3321; cation diffusion facilitator transporter family protein	OG_00282	LEC3_3373	cation diffusion facilitator family transporter
OG_00283	LG3211_3158	mutL; DNA mismatch repair MutL family protein	OG_00283	LEC3_3165	DNA mismatch repair protein
OG_00284	LG3211_853	LG3211_853; glycosyl transferases group 1 family protein	OG_00284	LEC3_0799	glycosyl transferase, group 1 family protein
OG_00285	LG3211_487	cyoE; protoheme IX farnesyltransferase	OG_00285	LEC3_0295	protoheme IX farnesyltransferase
OG_00286	LG3211_4308	LG3211_4308; amino acid permease family protein	OG_00286	LEC3_4368	amino acid permease
OG_00287	LG3211_2628	LG3211_2628; FAD binding domain of DNA photolyase family protein	OG_00287	LEC3_2636	deoxyribodipyrimidine photolyase
OG_00288	LG3211_1740	pabC; aminodeoxychorismate lyase	OG_00288	LEC3_1695	aminotransferase, class IV
OG_00289	LG3211_4919	FRK1; fructokinase-1	OG_00289	LEC3_0375	fructokinase-1
OG_00290	LG3211_5367	LG3211_5367; ABC-2 transporter family protein	OG_00290	LEC3_5352	ABC transporter sodium permease, putative
OG_00291	LG3211_1926	LG3211_1926; glycosyl transferase 2 family protein	OG_00291	LEC3_1876	glycosyl transferase, group 2 family protein
OG_00292	LG3211_4037	yfiO; outer membrane assembly lipoYfiO family protein	OG_00292	LEC3_4105	outer membrane assembly lipoprotein YfiO
OG_00293	LG3211_649	cwhA; N-acetylmuramoyl-L-alanine amidase A domain protein	OG_00293	LEC3_4698	N-acetylmuramoyl-L-alanine amidase
OG_00294	LG3211_1950	hisA; 1-(5-phosphoribosyl)-5-[(5- phosphoribosylamino)methylidenea	OG_00294	LEC3_1902	phosphoribosylformimino-5-aminoimidazole carboxamide ribotide iso
OG_00295	LG3211_3811	LG3211_3811; hypothetical protein	OG_00295	LEC3_3834	lipoprotein
OG_00296	LG3211_2658	LG3211_2658; N-terminal double-transmembrane domain protein	OG_00296	LEC3_2664	conserved domain protein
OG_00297	LG3211_1061	LG3211_1061; acetyltransferase family protein	OG_00297	LEC3_1030	acetyltransferase (GNAT) family
OG_00298	LG3211_4212	LG3211_4212; HAD phosphoserine phosphatase-like hydrolase, IB fa	OG_00298	LEC3_4269	HAD-superfamily hydrolase, subfamily IB
OG_00299	LG3211_2145	LG3211_2145; outer membrane autotransporter barrel domain protei	OG_00299	LEC3_2086	hemagglutinin
OG_00300	LG3211_1296	sufS; cysteine desulfurase, SufS family protein	OG_00300	LEC3_1256	cysteine desulfurase
OG_00301	LG3211_109	LG3211_109; ABC transporter family protein	OG_00301	LEC3_5215	ABC transporter, ATP-binding protein
OG_00302	LG3211_3014	LG3211_3014; hypothetical protein	OG_00302	LEC3_3006	hypothetical protein
OG_00303	LG3211_4218	LG3211_4218; conserved hypothetical protein	OG_00303	LEC3_4271	Antibiotic biosynthesis monooxygenase
OG_00304	LG3211_142	LG3211_142; phospholipase D family protein	OG_00304	LEC3_5182	phospholipase D Active site motif
OG_00305	LG3211_2237	LG3211_2237; short chain dehydrogenase family protein	OG_00305	LEC3_2191	oxidoreductase, short chain dehydrogenase/reductase family protein
OG_00306	LG3211_2618	copB; copper resistance protein B	OG_00306	LEC3_2629	copper resistance protein B
OG_00307	LG3211_1221	LG3211_1221; methyladenine glycosylase family protein	OG_00307	LEC3_1176	DNA-3-methyladenine glycosylase I
OG_00308	LG3211_4998	LG3211_4998; tautomerase enzyme family protein	OG_00308	LEC3_4908	4-oxalocrotonate tautomerase
OG_00309	LG3211_5202	LG3211_5202; acetyltransferase family protein	OG_00309	LEC3_0087	acetyltransferase, including N-acetylases of ribosomal protein
OG_00310	LG3211_1514	LG3211_1514; bacterial regulatory, luxR family protein	OG_00310	LEC3_1495	two-component transcriptional regulator, LuxR family
OG_00311	LG3211_177	LG3211_177; dienelactone hydrolase family protein	OG_00311	LEC3_5153	hydrolase, alpha/beta fold family protein
OG_00312	LG3211_2688	LG3211_2688; ATP-NAD kinase family protein	OG_00312	LEC3_2690	inorganic polyphosphate/ATP-NAD kinase
OG_00313	LG3211_5143	LG3211_5143; fatty acid desaturase family protein	OG_00313	LEC3_0144	fatty acid desaturase
OG_00314	LG3211_1733	fabH; 3-oxoacyl-[acyl-carrier-] synthase III family protein	OG_00314	LEC3_1689	3-oxoacyl-(acyl-carrier-protein) synthase III
OG_00315	LG3211_1392	hutF; formiminoglutamate deiminase	OG_00315	LEC3_1360	formiminoglutamate deiminase
OG_00316	LG3211_1949	hisH; imidazole glycerol phosphate synthase, glutamine amidotransfe	OG_00316	LEC3_1901	imidazole glycerol phosphate synthase, glutamine amidotransferase s
OG_00317	LG3211_3773	rplJ; 50S ribosomal subunit protein L10	OG_00317	LEC3_3799	ribosomal protein L10
OG_00318	LG3211_2430	LG3211_2430; histidine kinase-, DNA gyrase B-, and HSP90-like ATF	OG_00318	LEC3_2372	histidine kinase
OG_00319	LG3211_851	LG3211_851; cupin domain protein	OG_00319	LEC3_0797	pirin domain protein
OG_00320	LG3211_2427	iam; isoamylase	OG_00320	LEC3_2370	isoamylase precursor
OG_00321	LG3211_2090	LG3211_2090; conserved hypothetical protein	OG_00321	LEC3_2029	conserved hypothetical protein
OG_00322	LG3211_3375	LG3211_3375; inhibitor of apoptosis-promoting Bax1 family protein	OG_00322	LEC3_3430	membrane protein
OG_00323	LG3211_5038	LG3211_5038; conserved hypothetical protein	OG_00323	LEC3_4948	UPF0114 protein
OG_00324	LG3211_852	LG3211_852; zinc-finger domain protein	OG_00324	LEC3_0798	conserved hypothetical protein
OG_00325	LG3211_404	LG3211_404; sagB-type dehydrogenase domain protein	OG_00325	LEC3_0229	conserved hypothetical protein
OG_00326	LG3211_4991	LG3211_4991; HAMP domain protein	OG_00326	LEC3_4903	sensor histidine kinase
OG_00327	LG3211_2436	LG3211_2436; major Facilitator Superfamily protein	OG_00327	LEC3_2378	transporter, major facilitator superfamily
OG_00328	LG3211_1865	htpX; heat shock , integral membrane protein	OG_00328	LEC3_1811	HtpX N-terminus/M48 family peptidase
OG_00329	LG3211_960	LG3211_960; bacterial regulatory helix-turn-helix, AraC family protein	OG_00329	LEC3_0916	transcriptional regulator, AraC family
OG_00330	LG3211_3868	LG3211_3868; F5/8 type C domain protein	OG_00330	LEC3_3884	protease 1
OG_00331	LG3211_4187	LG3211_4187; putative ATP binding component of ABC-transporter	OG_00331	LEC3_4245	ABC transporter, ATP-binding protein
OG_00332	LG3211_69	LG3211_69; putative aspartate/glutamate racemase	OG_00332	LEC3_5274	aspartate racemase

OG_00334	LG3211_1036	LG3211_1036; conserved hypothetical protein	OG_00334	LEC3_1002	conserved hypothetical protein
OG_00335	LG3211_770	LG3211_770; RNA polymerase sigma factor, sigma-70 family protein	OG_00335	LEC3_0731	RNA polymerase sigma factor, sigma-70 family
OG_00336	LG3211_444	LG3211_444; phosphoglucomutase/phosphomannomutase, C-termin	OG_00336	LEC3_0254	phosphomannomutase
OG_00337	LG3211_2398	potF; potF	OG_00337	LEC3_2343	bacterial extracellular solute-binding protein
OG_00338	LG3211_4264	rocF; arginase	OG_00338	LEC3_4333	arginase
OG_00339	LG3211_3784	LG3211_3784; 50S ribosome-binding GTPase family protein	OG_00339	LEC3_3806	GTP-binding protein YchF
OG_00340	LG3211_54	LG3211_54; histidine kinase-, DNA gyrase B-, and HSP90-like ATPas	OG_00340	LEC3_5267	two-component system sensor protein
OG_00341	LG3211_3338	chrR; chromate reductase	OG_00341	LEC3_3393	FAD dependent oxidoreductase
OG_00342	LG3211_1106	LG3211_1106; polyketide cyclase / dehydrase and lipid transport fam	OG_00342	LEC3_1079	DoxX family protein
OG_00343	LG3211_1726	LG3211_1726; conserved hypothetical protein	OG_00343	LEC3_1682	conserved hypothetical protein
OG_00344	LG3211_3088	LG3211_3088; ahpC/TSA family protein	OG_00344	LEC3_3082	peroxiredoxin Hyr1
OG_00345	LG3211_2636	LG3211_2636; glycine zipper family protein	OG_00345	LEC3_2639	rickettsia 17 kDa surface antigen family
OG_00346	LG3211_2267	LG3211_2267; enoyl-CoA hydratase/isomerase family protein	OG_00346	LEC3_0577	3-hydroxybutyryl-CoA dehydratase
OG_00347	LG3211_881	AKHSDH1; bifunctional aspartokinase/homoserine dehydrogenase 1,	OG_00347	LEC3_0829	aspartate kinase/homoserine dehydrogenase
OG_00348	LG3211_1892	argC; N-acetyl-gamma-glutamyl-phosphate reductase	OG_00348	LEC3_1841	N-acetyl-gamma-glutamyl-phosphate reductase
OG_00349	LG3211_4003	LG3211_4003; acetyltransferase family protein	OG_00349	LEC3_4068	acetyltransferase (GNAT) family
OG_00350	LG3211_691	LG3211_691; ftsX-like permease family protein	OG_00350	LEC3_0656	efflux ABC transporter, permease protein
OG_00351	LG3211_1040	LG3211_1040; thiazole biosynthesis ThiG family protein	OG_00351	LEC3_1006	thiazole biosynthesis protein ThiG
OG_00352	LG3211_1559	LG3211_1559; conserved hypothetical protein	OG_00352	LEC3_1525	hypothetical protein
OG_00353	LG3211_5336	LG3211_5336; yecA family protein	OG_00353	LEC3_5392	YecA family protein
OG_00354	LG3211_479	coxB; cytochrome c oxidase, subunit II	OG_00354	LEC3_0286	cytochrome c oxidase, subunit II
OG_00355	LG3211_1229	LG3211_1229; phosphocarrier, HPr family protein	OG_00355	LEC3_1184	phosphocarrier protein HPr (Histidine-containing protein)
OG_00356	LG3211_1361	tdh; L-threonine 3-dehydrogenase	OG_00356	LEC3_1319	L-threonine 3-dehydrogenase
OG_00357	LG3211_933	LG3211_933; EVE domain protein	OG_00357	LEC3_0883	conserved hypothetical protein
OG_00358	LG3211_210	epaR; type III secretion apparatus protein SpaR/YscT/HrcT	OG_00358	LEC3_5122	type III secretion apparatus protein
OG_00359	LG3211_324	LG3211_324; putative acetyltransferase	OG_00359	LEC3_5045	acetyltransferase (GNAT) family
OG_00360	LG3211_766	LG3211_766; essential respiratory protein A	OG_00360	LEC3_0728	iron-sulfur cluster assembly accessory protein
OG_00361	LG3211_837	LG3211_837; conserved hypothetical protein	OG_00361	LEC3_0785	hypothetical protein
OG_00362	LG3211_178	LG3211_178; bacterial regulatory, tetR family protein	OG_00362	LEC3_1038	transcriptional regulator, TetR family
OG_00363	LG3211_226	LG3211_226; conserved hypothetical protein	OG_00363	LEC3_5104	conserved hypothetical protein
OG_00364	LG3211_2990	aroA; 3-phosphoshikimate 1-carboxyvinyltransferase	OG_00364	LEC3_2981	3-phosphoshikimate 1-carboxyvinyltransferase
OG_00365	LG3211_2831	LG3211_2831; cysteine dioxygenase type I family protein	OG_00365	LEC3_2837	cysteine dioxygenase type I
OG_00366	LG3211_4339	LG3211_4339; putative relaxation protein	OG_00366	LEC3_5183	conserved hypothetical protein
OG_00367	LG3211_1352	prpD; 2-methylcitrate dehydratase	OG_00367	LEC3_1308	2-methylcitrate dehydratase
OG_00368	LG3211_950	LG3211_950; hypothetical protein	OG_00368	LEC3_0905	hypothetical protein
OG_00369	LG3211_3143	LG3211_3143; conserved hypothetical protein	OG_00369	LEC3_3151	membrane protein
OG_00370	LG3211_958	spoT; guanosine-3',5'-bis(diphosphate) 3'-pyrophosphohydrolase	OG_00370	LEC3_0912	RelA/SpoT family protein
OG_00371	LG3211_3717	LG3211_3717; phosphate-selective porin O and P family protein	OG_00371	LEC3_3741	phosphate-selective porin O and P
OG_00372	LG3211_940	LG3211_940; aminopeptidase P, N-terminal domain protein	OG_00372	LEC3_0896	Xaa-Pro aminopeptidase
OG_00373	LG3211_2921	LG3211_2921; bacterial regulatory, tetR family protein	OG_00373	LEC3_2933	transcriptional regulator, TetR family
OG_00374	LG3211_4287	rtcB; conserved protein	OG_00374	LEC3_4348	conserved hypothetical protein
OG_00375	LG3211_438	LG3211_438; DNA repair RadC family protein	OG_00375	LEC3_0250	DNA repair protein RadC
OG_00376	LG3211_3922	bioA; adenosylmethionine-8-amino-7-oxononanoate transaminase	OG_00376	LEC3_3946	adenosylmethionine-8-amino-7-oxononanoate transaminase
OG_00377	LG3211_1791	LG3211_1791; conserved hypothetical protein	OG_00377	LEC3_1752	conserved hypothetical protein
OG_00378	LG3211_1623	LG3211_1623; hypothetical protein	OG_00378	LEC3_1596	lipoprotein
OG_00379	LG3211_749	LG3211_749; putative afsR-like transcriptional regulator	OG_00379	LEC3_0713	hypothetical protein
OG_00380	LG3211_1510	ispF; 2-C-methyl-D-erythritol 2,4-cyclodiphosphate synthase	OG_00380	LEC3_1491	2C-methyl-D-erythritol 2,4-cyclodiphosphate synthase
OG_00381	LG3211_815	accC; acetyl-CoA carboxylase, biotin carboxylase subunit	OG_00381	LEC3_0767	acetyl-CoA carboxylase, biotin carboxylase
OG_00382	LG3211_186	LG3211_186; conserved hypothetical protein	OG_00382	LEC3_5145	hypothetical protein
OG_00383	LG3211_2763	LG3211_2763; hypothetical protein	OG_00383	LEC3_2769	conserved hypothetical protein
OG_00384	LG3211_4459	mrdB; rod shape-determining protein RodA	OG_00384	LEC3_4509	rod shape-determining protein RodA
OG_00385	LG3211_3431	LG3211_3431; putative membrane protein	OG_00385	LEC3_3495	hypothetical protein
OG_00386	LG3211_103	LG3211_103; V4R domain protein	OG_00386	LEC3_5221	V4R domain
OG_00387	LG3211_1245	LG3211_1245; conserved hypothetical protein	OG_00387	LEC3_1200	conserved hypothetical protein
OG_00388	LG3211_78	LG3211_78; methyltransferase domain protein	OG_00388	LEC3_5244	oxidoreductase
OG_00389	LG3211_4490	LG3211_4490; WYL domain protein	OG_00389	LEC3_4542	transcriptional regulatory protein
OG_00390	LG3211_2889	LG3211_2889; moeA C-terminal region family protein	OG_00390	LEC3_2903	molybdopterin biosynthesis protein
OG_00391	LG3211_3442	LG3211_3442; putative diguanylate cyclase/phosphodiesterase	OG_00391	LEC3_3505	cyclic diguanylate phosphodiesterase/diguanylate cyclase
OG_00392	LG3211_1207	LG3211_1207; proteobacterial lipase chaperone family protein	OG_00392	LEC3_1158	lipase chaperone
OG_00393	LG3211_1266	LG3211_1266; conserved hypothetical protein	OG_00393	LEC3_1222	permease
OG_00394	LG3211_2084	LG3211_2084; prolyl oligopeptidase family protein	OG_00394	LEC3_2023	hydrolase, alpha/beta fold family protein
OG_00395	LG3211_857	LG3211_857; type I secretion outer membrane , TolC family protein	OG_00395	LEC3_0807	protein TolC
OG_00396	LG3211_2890	LG3211_2890; mobA-like NTP transferase domain protein	OG_00396	LEC3_2904	conserved hypothetical protein
OG_00397	LG3211_2016	HMGCL; hydroxymethylglutaryl-CoA lyase	OG_00397	LEC3_1948	Hydroxymethylglutaryl-CoA lyase
OG_00398	LG3211_1646	LG3211_1646; conserved hypothetical protein	OG_00398	LEC3_1618	GlcG protein
OG_00399	LG3211_588	LG3211_588; bacterial extracellular solute-binding family protein	OG_00399	LEC3_4778	periplasmic iron-binding protein
OG_00400	LG3211_4184	gmd; GDP-mannose 4,6-dehydratase	OG_00400	LEC3_4242	GDP-mannose 4,6-dehydratase
OG_00401	LG3211_3412	LG3211_3412; putative transmembrane protein	OG_00401	LEC3_3475	conserved hypothetical protein
OG_00402	LG3211_2659	LG3211_2659; von Willebrand factor type A domain protein	OG_00402	LEC3_2665	conserved hypothetical protein
OG_00403	LG3211_3473	fdx1; ferredoxin	OG_00403	LEC3_3530	ferredoxin
OG_00404	LG3211_3718	LG3211_3718; response regulator	OG_00404	LEC3_3743	sensory box histidine kinase
OG_00405	LG3211_5049	LG3211_5049; beta-ketoacyl synthase, C-terminal domain protein	OG_00405	LEC3_4967	beta-ketoacyl synthase
OG_00406	LG3211_4089	LG3211_4089; lppC lipofamily protein	OG_00406	LEC3_4143	lipoprotein, LppC family
OG_00407	LG3211_3040	kdsB; 3-deoxy-D-manno-octulosonate cytidylyltransferase	OG_00407	LEC3_3031	3-deoxy-D-manno-octulosonate cytidylyltransferase
OG_00408	LG3211_5093	LG3211_5093; putative transmembrane protein	OG_00408	LEC3_5001	conserved hypothetical protein
OG_00409	LG3211_1402	LG3211_1402; bacterial regulatory, tetR family protein	OG_00409	LEC3_1367	transcriptional regulator, TetR family
OG_00410	LG3211_2146	LG3211_2146; conserved hypothetical protein	OG_00410	LEC3_2087	conserved hypothetical protein
OG_00411	LG3211_3444	LG3211_3444; RNA methyltransferase, TrmH , group 1 family protein	OG_00411	LEC3_3507	RNA methyltransferase, TrmH family, group 1
OG_00412	LG3211_3913	LG3211_3913; transcriptional regulatory , C terminal family protein	OG_00412	LEC3_3935	Transcriptional regulatory protein, C terminal - DNA-binding response
OG_00413	LG3211_2207	LG3211_2207; NADH-quinone oxidoreductase, chain I family protein	OG_00413	LEC3_2167	NADH-quinone oxidoreductase, chain I family protein
OG_00414	LG3211_3804	LG3211_3804; acylphosphatase family protein	OG_00414	LEC3_3828	acylphosphatase

OG_00416	LG3211_3327	yaeT; outer membrane assembly complex, YaeT protein	OG_00416	LEC3_3379	outer membrane protein assembly complex, YaeT protein
OG_00417	LG3211_3076	LG3211_3076; hydrolyase, tartrate alpha subunit/fumarate, Fe-S type	OG_00417	LEC3_3068	fumarate hydratase, class I
OG_00418	LG3211_2995	LG3211_2995; ferric reductase like transmembrane component family	OG_00418	LEC3_2986	Ferric reductase like transmembrane component protein
OG_00419	LG3211_1895	proB; glutamate 5-kinase	OG_00419	LEC3_1844	glutamate 5-kinase
OG_00420	LG3211_3648	LG3211_3648; prolyl oligopeptidase family protein	OG_00420	LEC3_3636	prolyl oligopeptidase family
OG_00421	LG3211_4993	LG3211_4993; ecsC family protein	OG_00421	LEC3_4904	conserved hypothetical protein
OG_00422	LG3211_830	LG3211_830; cupin 2, conserved barrel domain protein	OG_00422	LEC3_0780	cupin domain protein
OG_00423	LG3211_2750	LG3211_2750; EF-P lysine aminoacylase GenX	OG_00423	LEC3_2755	putative lysyl-tRNA synthetase
OG_00424	LG3211_4487	LG3211_4487; conserved hypothetical protein	OG_00424	LEC3_4540	conserved hypothetical protein
OG_00425	LG3211_3184	LG3211_3184; conserved hypothetical family protein	OG_00425	LEC3_3191	conserved hypothetical protein
OG_00426	LG3211_4085	LG3211_4085; mraZ family protein	OG_00426	LEC3_4139	MraZ protein
OG_00427	LG3211_1947	hisC; histidinol-phosphate transaminase	OG_00427	LEC3_1899	histidinol-phosphate aminotransferase
OG_00428	LG3211_716	atpA; ATP synthase F1, alpha subunit	OG_00428	LEC3_0687	ATP synthase F1, alpha subunit
OG_00429	LG3211_4351	fabR; fabR transcriptional repressor FabR transcriptional dual regulat	OG_00429	LEC3_4393	transcriptional regulator, TetR family
OG_00430	LG3211_660	LG3211_660; putative efflux pump outer membrane protein ttgC	OG_00430	LEC3_4688	efflux transporter, outer membrane factor (OMF) lipoprotein, NodT far
OG_00431	LG3211_3133	rimO; ribosomal protein S12 methylthiotransferase RimO	OG_00431	LEC3_3137	MiaB-like tRNA modifying enzyme YliG
OG_00432	LG3211_4066	secA; preprotein translocase, SecA subunit	OG_00432	LEC3_4122	preprotein translocase, SecA subunit
OG_00433	LG3211_1712	LG3211_1712; cytochrome D1 heme domain protein	OG_00433	LEC3_1673	40-residue yvtn family beta-propeller repeat protein
OG_00434	LG3211_875	ilvC; ketol-acid reductoisomerase	OG_00434	LEC3_0824	ketol-acid reductoisomerase
OG_00435	LG3211_2788	LG3211_2788; bacterial regulatory, tetR family protein	OG_00435	LEC3_2794	transcriptional regulator, TetR family
OG_00436	LG3211_1208	LG3211_1208; hypothetical protein	OG_00436	LEC3_1159	lipoprotein
OG_00437	LG3211_1638	LG3211_1638; pirin family protein	OG_00437	LEC3_1608	pirin-like protein
OG_00438	LG3211_1080	LG3211_1080; pfkB carbohydrate kinase family protein	OG_00438	LEC3_1054	kinase, pfkB family protein
OG_00439	LG3211_1784	LG3211_1784; PAP2 superfamily protein	OG_00439	LEC3_1747	phosphatidylglycerophosphatase B-related protein
OG_00440	LG3211_225	LG3211_225; putative ATP synthase yscN	OG_00440	LEC3_5105	ATPase FliI/YscN family
OG_00441	LG3211_2812	LG3211_2812; transglycosylase SLT domain protein	OG_00441	LEC3_2818	VirB1 protein
OG_00442	LG3211_2827	dsbE; disulfide oxidoreductases, DsbE subfamily protein	OG_00442	LEC3_2832	thiol:disulfide interchange protein dsbE
OG_00443	LG3211_2415	LG3211_2415; ku protein	OG_00443	LEC3_3697	Ku protein
OG_00444	LG3211_2118	LG3211_2118; subtilase family protein	OG_00444	LEC3_2063	peptidase, families S8 and S53
OG_00445	LG3211_3062	LG3211_3062; glycine zipper 2TM domain protein	OG_00445	LEC3_3056	rickettsia 17 kDa surface antigen family
OG_00446	LG3211_1331	prpE; propionate--CoA ligase	OG_00446	LEC3_1286	propionate--CoA ligase
OG_00447	LG3211_620	LG3211_620; glyoxalase-like domain protein	OG_00447	LEC3_4730	glyoxalase
OG_00448	LG3211_4275	LG3211_4275; transcriptional regulatory , C terminal family protein	OG_00448	LEC3_4342	Transcriptional regulatory proteins, C terminal - transcriptional regulat
OG_00449	LG3211_3132	LG3211_3132; flavo, HI0933 family protein	OG_00449	LEC3_3136	FAD dependent oxidoreductase
OG_00450	LG3211_2986	LG3211_2986; NADPH-dependent FMN reductase family protein	OG_00450	LEC3_2976	NADPH-dependent FMN reductase domain protein
OG_00451	LG3211_400	LG3211_400; diguanylate cyclase domain protein	OG_00451	LEC3_0226	diguanylate cyclase (GGDEF) domain
OG_00452	LG3211_1243	LG3211_1243; bolA superfamily transcriptional regulator	OG_00452	LEC3_1198	BolA family protein
OG_00453	LG3211_4543	LG3211_4543; tonB-dependent Receptor Plug domain protein	OG_00453	LEC3_4598	TonB-dependent receptor
OG_00454	LG3211_266	LG3211_266; iaaA	OG_00454	LEC3_5061	L-asparagineamidohydrolase precursor
OG_00455	LG3211_3696	LG3211_3696; cyclic peptide transporter family protein	OG_00455	LEC3_2561	ABC transporter ATP-binding protein yojI
OG_00456	LG3211_4227	rraA; regulator of ribonuclease activity A	OG_00456	LEC3_4287	RraA family
OG_00457	LG3211_1732	rpmF; ribosomal protein L32	OG_00457	LEC3_1688	ribosomal protein L32
OG_00458	LG3211_498	LG3211_498; yqey-like family protein	OG_00458	LEC3_0308	GatB/Yqey domain protein
OG_00459	LG3211_192	LG3211_192; nitrogen regulatory P-II family protein	OG_00459	LEC3_5139	nitrogen regulatory protein P-II
OG_00460	LG3211_767	nudC; NADH pyrophosphatase	OG_00460	LEC3_0729	NADH pyrophosphatase-like rudimentary NUDIX domain protein
OG_00461	LG3211_734	LG3211_734; ABC transporter family protein	OG_00461	LEC3_0699	ABC transporter
OG_00462	LG3211_1161	LG3211_1161; alpha/beta hydrolase fold family protein	OG_00462	LEC3_1133	alpha/beta hydrolase fold
OG_00463	LG3211_2319	recJ; single-stranded-DNA-specific exonuclease RecJ	OG_00463	LEC3_2242	single-stranded-DNA-specific exonuclease
OG_00464	LG3211_3091	LG3211_3091; amidohydrolase family protein	OG_00464	LEC3_3087	Atrazine chlorohydrolase
OG_00465	LG3211_3445	LG3211_3445; inositol monophosphatase family protein	OG_00465	LEC3_3508	Inositol-1-monophosphatase
OG_00466	LG3211_2045	LG3211_2045; methylmalonyl-CoA mutase N-terminal domain protein	OG_00466	LEC3_1980	methylmalonyl-CoA mutase, alpha subunit
OG_00467	LG3211_4614	LG3211_4614; bacterial regulatory helix-turn-helix , lysR family protei	OG_00467	LEC3_0649	probable transcriptional regulator
OG_00468	LG3211_5185	LG3211_5185; gram-negative pili assembly chaperone, N-terminal dc	OG_00468	LEC3_0102	type I pili usher pathway
OG_00469	LG3211_3193	csrA; carbon storage regulator	OG_00469	LEC3_3199	carbon storage regulator
OG_00470	LG3211_1532	folP; dihydropteroate synthase	OG_00470	LEC3_1508	dihydropteroate synthase
OG_00471	LG3211_3301	LG3211_3301; WD40-like Beta Propeller Repeat family protein	OG_00471	LEC3_3343	lipoprotein
OG_00472	LG3211_4502	LG3211_4502; tonB family C-terminal domain protein	OG_00472	LEC3_4556	TonB family C-terminal domain (possible frameshift)
OG_00473	LG3211_2331	LG3211_2331; PA14 domain protein	OG_00473	LEC3_2271	beta-hexosaminidase
OG_00474	LG3211_2935	yceC; 23S rRNA pseudouridine synthase	OG_00474	LEC3_2948	ribosomal large subunit pseudouridine synthase C
OG_00475	LG3211_4510	LG3211_4510; 2Fe-2S iron-sulfur cluster binding domain protein	OG_00475	LEC3_4565	2Fe-2S iron-sulfur cluster binding domain
OG_00476	LG3211_4098	ribE; riboflavin synthase, alpha subunit	OG_00476	LEC3_4153	riboflavin synthase, alpha subunit
OG_00477	LG3211_374	LG3211_374; pfkB carbohydrate kinase family protein	OG_00477	LEC3_0192	ribokinase
OG_00478	LG3211_1276	LG3211_1276; bacterial regulatory helix-turn-helix , lysR family protei	OG_00478	LEC3_1232	HTH-type transcriptional regulator CysB
OG_00479	LG3211_4164	LG3211_4164; mannose-1-phosphate guanylyltransferase/mannose-	OG_00479	LEC3_4230	mannose-6-phosphate isomerase
OG_00480	LG3211_96	LG3211_96; short chain dehydrogenase family protein	OG_00480	LEC3_5227	oxidoreductase, short chain dehydrogenase/reductase family
OG_00481	LG3211_180	LG3211_180; TPR repeat family protein	OG_00481	LEC3_5152	tetratricopeptide repeat domain protein
OG_00482	LG3211_4203	LG3211_4203; acetyltransferase family protein	OG_00482	LEC3_4258	acetyltransferase (GNAT) family
OG_00483	LG3211_1561	LG3211_1561; hypothetical protein	OG_00483	LEC3_1538	lipoprotein
OG_00484	LG3211_1008	LG3211_1008; ribonucleotide reductase, small chain family protein	OG_00484	LEC3_0971	ribonucleotide reductase, small chain
OG_00485	LG3211_97	LG3211_97; efflux transporter, RND family, MFP subunit	OG_00485	LEC3_5226	efflux transporter, RND family, MFP subunit
OG_00486	LG3211_4067	LG3211_4067; peptidase M23 family protein	OG_00486	LEC3_4123	M23 peptidase domain protein
OG_00487	LG3211_145	LG3211_145; cobQ/CobB/MinD/ParA nucleotide binding domain prote	OG_00487	LEC3_5179	CobQ/CobB/MinD/ParA nucleotide binding domain
OG_00488	LG3211_4930	LG3211_4930; bacterial PH domain protein	OG_00488	LEC3_0365	bacterial membrane flanked domain
OG_00489	LG3211_918	LG3211_918; HIT domain protein	OG_00489	LEC3_0871	histidine triad (HIT) protein
OG_00490	LG3211_2652	asnS; asparagine--tRNA ligase	OG_00490	LEC3_2656	asparaginyl-tRNA synthetase
OG_00491	LG3211_2260	LG3211_2260; conserved hypothetical protein	OG_00491	LEC3_2213	conserved hypothetical protein
OG_00492	LG3211_1673	LG3211_1673; carbon-nitrogen hydrolase family protein	OG_00492	LEC3_1635	nitrilase
OG_00493	LG3211_2866	LG3211_2866; mechanosensitive ion channel family protein	OG_00493	LEC3_2878	transporter, small conductance mechanosensitive ion channel (MscS)
OG_00494	LG3211_3979	queC; queuosine biosynthesis protein QueC	OG_00494	LEC3_4045	ExsB protein
OG_00495	LG3211_728	LG3211_728; his Kinase A domain protein	OG_00495	LEC3_0693	histidine kinase
OG_00496	LG3211_4418	LG3211_4418; efflux transporter, RND family, MFP subunit	OG_00496	LEC3_4462	efflux transporter, RND family, MFP subunit

OG_00497	LG3211_567	LG3211_567; amidohydrolase family protein	OG_00497	LEC3_4803	amidohydrolase family protein
OG_00498	LG3211_4154	LG3211_4154; conserved hypothetical protein	OG_00498	LEC3_4220	conserved hypothetical protein
OG_00499	LG3211_3173	LG3211_3173; 4Fe-4S binding domain protein	OG_00499	LEC3_3180	ferredoxin domain protein
OG_00500	LG3211_1311	LG3211_1311; bacterial regulatory helix-turn-helix, AraC family protei	OG_00500	LEC3_1271	transcriptional regulator, AraC family
OG_00501	LG3211_2657	LG3211_2657; putative transmembrane protein	OG_00501	LEC3_2663	conserved hypothetical protein
OG_00502	LG3211_3309	LG3211_3309; transcriptional regulator, Spx/MgsR family protein	OG_00502	LEC3_3356	ArsC family protein
OG_00503	LG3211_5201	LG3211_5201; putative nucleotidyltransferase family protein	OG_00503	LEC3_0088	conserved hypothetical protein
OG_00504	LG3211_534	LG3211_534; bacterial extracellular solute-binding, 5 Middle family pr	OG_00504	LEC3_0346	bacterial extracellular solute-binding proteins, family 5
OG_00505	LG3211_1957	LG3211_1957; dehydroquinase class II family protein	OG_00505	LEC3_1908	dehydroquinase class II
OG_00506	LG3211_3089	LG3211_3089; tonB dependent receptor family protein	OG_00506	LEC3_3084	TonB-dependent receptor
OG_00507	LG3211_3518	LG3211_3518; bacterial regulatory, luxR family protein	OG_00507	LEC3_3550	two component transcriptional regulator, LuxR family
OG_00508	LG3211_3936	LG3211_3936; cheW-like domain protein	OG_00508	LEC3_3960	Type IV pilus assembly protein
OG_00509	LG3211_3515	LG3211_3515; FHA domain protein	OG_00509	LEC3_3547	adenylate/guanylate cyclase
OG_00510	LG3211_782	LG3211_782; conserved hypothetical protein	OG_00510	LEC3_2444	acetyltransferase family
OG_00511	LG3211_4608	LG3211_4608; bacterial regulatory, gntR family protein	OG_00511	LEC3_4654	transcriptional regulator, GntR family
OG_00512	LG3211_3290	LG3211_3290; conserved hypothetical protein	OG_00512	LEC3_3322	conserved hypothetical protein
OG_00513	LG3211_2372	ftsK; DNA translocase ftsK	OG_00513	LEC3_2319	FtsK/SpoIIIE family
OG_00514	LG3211_596	LG3211_596; small Multidrug Resistance family protein	OG_00514	LEC3_4771	multidrug resistance protein, SMR family
OG_00515	LG3211_1792	LG3211_1792; pectinacetylesterase family protein	OG_00515	LEC3_1753	conserved hypothetical protein
OG_00516	LG3211_4286	rtcA; RNA 3'-phosphate cyclase	OG_00516	LEC3_4347	RNA 3'-phosphate cyclase
OG_00517	LG3211_1972	LG3211_1972; elongation factor P (EF-P) OB domain protein	OG_00517	LEC3_1927	elongation factor P-like protein
OG_00518	LG3211_2919	LG3211_2919; carbon-nitrogen hydrolase family protein	OG_00518	LEC3_2931	beta-alanine synthetase
OG_00519	LG3211_535	LG3211_535; glutamate--cysteine ligase	OG_00519	LEC3_0347	glutamate-cysteine ligase
OG_00520	LG3211_321	LG3211_321; his Kinase A domain protein	OG_00520	LEC3_5049	nitrogen regulation protein NtrB
OG_00521	LG3211_4153	LG3211_4153; mce related family protein	OG_00521	LEC3_4219	mce related protein
OG_00522	LG3211_5228	LG3211_5228; phosphoribosyl transferase domain protein	OG_00522	LEC3_0060	putative comF family protein
OG_00523	LG3211_5366	LG3211_5366; ABC transporter family protein	OG_00523	LEC3_5353	ATP-binding transport protein natA
OG_00524	LG3211_537	LG3211_537; bacterial regulatory helix-turn-helix , lysR family protein	OG_00524	LEC3_4848	transcriptional regulator, LysR family
OG_00525	LG3211_560	LG3211_560; conserved hypothetical protein	OG_00525	LEC3_4819	conserved hypothetical protein
OG_00526	LG3211_1014	pgk; phosphoglycerate kinase	OG_00526	LEC3_0977	phosphoglycerate kinase
OG_00527	LG3211_3161	LG3211_3161; conserved hypothetical protein	OG_00527	LEC3_3169	conserved hypothetical protein
OG_00528	LG3211_1596	LG3211_1596; hypothetical protein	OG_00528	LEC3_1571	hypothetical protein
OG_00529	LG3211_3601	LG3211_3601; conserved hypothetical protein	OG_00529	LEC3_3593	conserved hypothetical protein
OG_00530	LG3211_4010	LG3211_4010; proton antiporter-2 family protein	OG_00530	LEC3_4078	inner membrane protein YbaL
OG_00531	LG3211_1492	LG3211_1492; FAD binding domain protein	OG_00531	LEC3_1467	FAD dependent oxidoreductase
OG_00532	LG3211_1762	nagZ; beta-hexosaminidase	OG_00532	LEC3_1718	beta-hexosaminidase
OG_00533	LG3211_3363	clpX; ATP-dependent Clp protease, ATP-binding subunit ClpX	OG_00533	LEC3_3418	ATP-dependent Clp protease, ATP-binding subunit ClpX
OG_00534	LG3211_1287	LG3211_1287; flavodoxin family protein	OG_00534	LEC3_1243	flavodoxin/oxidoreductase NAD-binding domain
OG_00535	LG3211_2814	virB11; P-type DNA transfer ATPase VirB11	OG_00535	LEC3_2819	VirB11 protein
OG_00536	LG3211_3822	LG3211_3822; hypothetical protein	OG_00536	LEC3_3846	conserved hypothetical protein
OG_00537	LG3211_1959	mtnC; 2,3-diketo-5-methylthio-1-phosphopentane phosphatase	OG_00537	LEC3_1910	2,3-diketo-5-methylthio-1-phosphopentane phosphatase
OG_00538	LG3211_4643	LG3211_4643; conserved hypothetical protein	OG_00538	LEC3_2245	conserved hypothetical protein
OG_00539	LG3211_826	LG3211_826; bacterial regulatory helix-turn-helix , lysR family protein	OG_00539	LEC3_0775	transcriptional regulator, LysR family
OG_00540	LG3211_1442	LG3211_1442; cyclase family protein	OG_00540	LEC3_1411	cyclase
OG_00541	LG3211_3100	cfa; cfa	OG_00541	LEC3_3101	cyclopropane-fatty-acyl-phospholipid synthase
OG_00542	LG3211_883	LG3211_883; beta-lytic metalloendopeptidase	OG_00542	LEC3_0833	beta-lytic metalloendopeptidase
OG_00543	LG3211_1777	LG3211_1777; diguanylate cyclase domain protein	OG_00543	LEC3_1739	sensory box-containing diguanylate cyclase/cyclic diguanylate phospl
OG_00544	LG3211_1468	LG3211_1468; methyltransferase domain protein	OG_00544	LEC3_1436	methyltransferase domain family
OG_00545	LG3211_1380	gltX; glutamate--tRNA ligase	OG_00545	LEC3_1348	glutamyl-tRNA synthetase
OG_00546	LG3211_941	LG3211_941; bacterial regulatory helix-turn-helix , lysR family protein	OG_00546	LEC3_0897	transcriptional regulator, LysR family
OG_00547	LG3211_1960	mtnD; acireductone dioxygenase	OG_00547	LEC3_1911	dioxygenase
OG_00548	LG3211_4955	LG3211_4955; conserved hypothetical protein	OG_00548	LEC3_4861	conserved hypothetical protein
OG_00549	LG3211_1099	gcvT; glycine cleavage system T protein	OG_00549	LEC3_1072	glycine cleavage system T protein
OG_00550	LG3211_4128	LG3211_4128; DSBA-like thioredoxin domain protein	OG_00550	LEC3_4187	conserved hypothetical protein
OG_00551	LG3211_4940	dprA; DNA protecting protein DprA	OG_00551	LEC3_0353	DNA protecting protein DprA
OG_00552	LG3211_1189	LG3211_1189; periplasmic binding family protein	OG_00552	LEC3_1156	periplasmic binding protein SEQUENCING GAP
OG_00553	LG3211_915	LG3211_915; peptidase M20/M25/M40 family protein	OG_00553	LEC3_0868	peptidase family protein
OG_00554	LG3211_4088	yraL; 16S rRNA 2'-O-ribose C1402 methyltransferase	OG_00554	LEC3_4142	conserved hypothetical protein
OG_00555	LG3211_623	LG3211_623; conserved hypothetical protein	OG_00555	LEC3_4726	Dgpf domain protein
OG_00556	LG3211_4564	LG3211_4564; glycosyl hydrolases 31 family protein	OG_00556	LEC3_3654	glycosyl hydrolase, family 31
OG_00557	LG3211_4115	gspD; type II secretion system protein D	OG_00557	LEC3_4172	general secretion pathway protein D
OG_00558	LG3211_2038	LG3211_2038; autotransporter beta-domain protein	OG_00558	LEC3_1973	serine protease
OG_00559	LG3211_675	LG3211_675; asnC family protein	OG_00559	LEC3_4674	transcriptional regulator, AsnC family
OG_00560	LG3211_859	LG3211_859; bacterial regulatory, tetR family protein	OG_00560	LEC3_0809	transcriptional regulator, TetR family
OG_00561	LG3211_399	aceA; isocitrate lyase	OG_00561	LEC3_0223	isocitrate lyase
OG_00562	LG3211_979	LG3211_979; AAA domain family protein	OG_00562	LEC3_0937	methanol dehydrogenase regulatory protein
OG_00563	LG3211_352	LG3211_352; GHMP kinase N terminal domain protein	OG_00563	LEC3_5021	GHMP kinases putative ATP-binding protein
OG_00564	LG3211_2711	LG3211_2711; conserved hypothetical protein	OG_00564	LEC3_2712	lipoprotein
OG_00565	LG3211_733	LG3211_733; ftsX-like permease family protein	OG_00565	LEC3_0698	efflux ABC transporter, permease protein
OG_00566	LG3211_1633	LG3211_1633; N-acetylmuramoyl-L-alanine amidase family protein	OG_00566	LEC3_1602	N-acetylmuramoyl-L-alanine amidase
OG_00567	LG3211_1082	LG3211_1082; diguanylate cyclase domain protein	OG_00567	LEC3_1056	diguanylate cyclase
OG_00568	LG3211_150	LG3211_150; ATPase associated with various cellular activities family	OG_00568	LEC3_5175	ATPase family associated with various cellular activities (AAA)
OG_00569	LG3211_3989	ybgC; tol-pal system-associated acyl-CoA thioesterase	OG_00569	LEC3_4057	tol-pal system-associated acyl-CoA thioesterase
OG_00570	LG3211_1991	LG3211_1991; ubiE/COQ5 methyltransferase family protein	OG_00570	LEC3_0492	methyltransferase type 12
OG_00571	LG3211_1394	LG3211_1394; alkA N-terminal domain protein	OG_00571	LEC3_1362	DNA-3-methyladenine glycosylase II
OG_00572	LG3211_3232	ffh; signal recognition particle protein	OG_00572	LEC3_3257	signal recognition particle protein
OG_00573	LG3211_2340	rpfF; enoyl-CoA hydratase/isomerase family protein	OG_00573	LEC3_2283	Enoyl-CoA hydratase/isomerase family RpfF
OG_00574	LG3211_3480	yggX; that protects iron-sulfur proteins against oxidative damage	OG_00574	LEC3_3537	Fe(2+)-trafficking protein
OG_00575	LG3211_4146	proS; proline--tRNA ligase	OG_00575	LEC3_4202	prolyl-tRNA synthetase
OG_00576	LG3211_2938	LG3211_2938; bacterial regulatory, luxR family protein	OG_00576	LEC3_2951	two-component system response regulator, LuxR family
OG_00577	LG3211_3954	LG3211_3954; putative secreted protein	OG_00577	LEC3_3977	conserved hypothetical protein

OG_00578	LG3211_1683	LG3211_1683; peptidase M48 family protein	OG_00578	LEC3_1651	peptidase, M48 family
OG_00579	LG3211_3759	rpsS; ribosomal protein S19	OG_00579	LEC3_3786	ribosomal protein S19
OG_00580	LG3211_1094	LG3211_1094; RNA polymerase sigma factor, sigma-70 family protei	OG_00580	LEC3_1068	RNA polymerase sigma-E factor
OG_00581	LG3211_3226	LG3211_3226; hypothetical protein	OG_00581	LEC3_3249	conserved hypothetical protein
OG_00582	LG3211_2370	ald; alanine dehydrogenase	OG_00582	LEC3_2317	alanine dehydrogenase
OG_00583	LG3211_3611	miaB; tRNA-i(6)A37 thiotransferase enzyme MiaB	OG_00583	LEC3_3604	tRNA-I(6)A37 thiotransferase enzyme MiaB
OG_00584	LG3211_955	gmk; guanylate kinase	OG_00584	LEC3_0910	guanylate kinase
OG_00585	LG3211_2367	LG3211_2367; acetyltransferase domain protein	OG_00585	LEC3_2313	conserved hypothetical protein
OG_00586	LG3211_2482	LG3211_2482; deoR C terminal sensor domain protein	OG_00586	LEC3_3659	aga operon transcriptional repressor
OG_00587	LG3211_3150	LG3211_3150; cobQ/CobB/MinD/ParA nucleotide binding domain pro	OG_00587	LEC3_3157	conserved hypothetical protein
OG_00588	LG3211_2172	LG3211_2172; putative ATPase	OG_00588	LEC3_2128	conserved hypothetical protein
OG_00589	LG3211_3796	LG3211_3796; conserved hypothetical protein	OG_00589	LEC3_3817	tetratricopeptide repeat family protein
OG_00590	LG3211_3774	rplA; ribosomal protein L1	OG_00590	LEC3_3800	ribosomal protein L1
OG_00591	LG3211_2161	LG3211_2161; alpha-2-macroglobulin N-terminal region family protei	OG_00591	LEC3_2115	alpha-2-macroglobulin family N- region
OG_00592	LG3211_3425	LG3211_3425; peptidase M13 family protein	OG_00592	LEC3_3488	peptidase, family M13
OG_00593	LG3211_2897	LG3211_2897; nucleoside diphosphate kinase family protein	OG_00593	LEC3_2911	nucleoside diphosphate kinase
OG_00594	LG3211_4421	LG3211_4421; ftsX-like permease family protein	OG_00594	LEC3_4465	efflux ABC transporter, permease protein
OG_00595	LG3211_3188	kdpD; sensor protein KdpD	OG_00595	LEC3_3196	two-component system sensor kinase KdpD
OG_00596	LG3211_701	LG3211_701; eamA-like transporter family protein	OG_00596	LEC3_0669	integral membrane protein DUF6
OG_00597	LG3211_946	pepQ; xaa-Pro dipeptidase	OG_00597	LEC3_0900	Xaa-Pro dipeptidase
OG_00598	LG3211_4077	murG; undecaprenyldiphospho-muramoylpentapeptide beta-N-acetylg	OG_00598	LEC3_4131	undecaprenyldiphospho-muramoylpentapeptide beta-N-acetylglucosa
OG_00599	LG3211_1741	LG3211_1741; yceG-like family protein	OG_00599	LEC3_1696	conserved hypothetical protein
OG_00600	LG3211_2039	LG3211_2039; acyl-CoA dehydrogenase, N-terminal domain protein	OG_00600	LEC3_1974	Acyl-CoA dehydrogenase, C-terminal domain
OG_00601	LG3211_893	LG3211_893; conserved hypothetical protein	OG_00601	LEC3_1035	conserved hypothetical protein
OG_00602	LG3211_4461	mreD; rod shape-determining protein MreD	OG_00602	LEC3_4511	rod shape-determining protein MreD
OG_00603	LG3211_3376	LG3211_3376; oligomerisation domain protein	OG_00603	LEC3_3433	iojap homolog protein
OG_00604	LG3211_4036	LG3211_4036; 23S rRNA pseudouridine synthase	OG_00604	LEC3_4104	ribosomal large subunit pseudouridine synthase D
OG_00605	LG3211_1447	LG3211_1447; glyoxalase/Bleomycin resistance /Dioxygenase superf	OG_00605	LEC3_1415	glyoxalase/bleomycin resistance protein/dioxygenase
OG_00606	LG3211_244	LG3211_244; YCII-related domain protein	OG_00606	LEC3_5086	DGPF domain protein
OG_00607	LG3211_4044	LG3211_4044; his Kinase A domain protein	OG_00607	LEC3_4112	Type IV pilus sensor protein
OG_00608	LG3211_4381	LG3211_4381; osmC-like family protein	OG_00608	LEC3_4419	OsmC-like protein
OG_00609	LG3211_1162	LG3211_1162; conserved hypothetical protein	OG_00609	LEC3_1134	conserved hypothetical protein
OG_00611	LG3211_3592	LG3211_3592; carboxypeptidase regulatory-like domain protein	OG_00611	LEC3_3584	Oar protein
OG_00612	LG3211_3162	LG3211_3162; putative carbohydrate kinase	OG_00612	LEC3_3170	carbohydrate kinase, YjeF related protein
OG_00613	LG3211_1815	LG3211_1815; glyoxalase/Bleomycin resistance /Dioxygenase superf	OG_00613	LEC3_0513	glyoxalase family protein
OG_00614	LG3211_4958	LG3211_4958; sigma-70, region 4 family protein	OG_00614	LEC3_4864	conserved hypothetical protein
OG_00615	LG3211_2143	LG3211_2143; phage Tail Collar domain protein	OG_00615	LEC3_2084	microcystin dependent protein
OG_00616	LG3211_4936	sun; ribosomal RNA small subunit methyltransferase B	OG_00616	LEC3_0358	ribosomal RNA small subunit methyltransferase B
OG_00617	LG3211_5155	LG3211_5155; surface antigen family protein	OG_00617	LEC3_0132	pathogenicity outer membrane protein
OG_00618	LG3211_1863	phbB; acetoacetyl-CoA reductase family protein	OG_00618	LEC3_1809	acetoacetyl-CoA reductase
OG_00619	LG3211_3935	LG3211_3935; methyl-accepting chemotaxis (MCP) signalling domai	OG_00619	LEC3_3959	Type IV pilus assembly protein
OG_00620	LG3211_1943	LG3211_1943; diguanylate cyclase domain protein	OG_00620	LEC3_1895	diguanylate cyclase
OG_00621	LG3211_3902	lgt; prolipoprotein diacylglyceryl transferase	OG_00621	LEC3_3921	prolipoprotein diacylglyceryl transferase
OG_00622	LG3211_2626	LG3211_2626; acyltransferase, WS/DGAT/MGAT family protein	OG_00622	LEC3_2635	acyltransferase, ws/dgat/mgat subfamily
OG_00623	LG3211_2301	fur; fur	OG_00623	LEC3_2228	ferric uptake regulation protein
OG_00624	LG3211_3742	rpsM; 30S ribosomal subunit protein S13	OG_00624	LEC3_3769	ribosomal protein S13
OG_00625	LG3211_797	LG3211_797; hypothetical protein	OG_00625	LEC3_0748	hypothetical protein
OG_00626	LG3211_4933	LG3211_4933; glycosyl transferase 2 family protein	OG_00626	LEC3_0361	lipopolysaccharide core biosynthesis glycosyl transferase
OG_00627	LG3211_3879	mtfA; 23S rRNA 2'-O-ribose C2498 methyltransferase	OG_00627	LEC3_3897	RNA 2'-O-ribose methyltransferase MtfA
OG_00628	LG3211_264	LG3211_264; integral membrane , TerC family protein	OG_00628	LEC3_5065	integral membrane protein, TerC family
OG_00629	LG3211_4311	LG3211_4311; hypothetical protein	OG_00629	LEC3_4372	hypothetical protein
OG_00630	LG3211_2878	guaA; guaA GMP synthetase	OG_00630	LEC3_2893	GMP synthase
OG_00631	LG3211_9	LG3211_9; motA/TolQ/ExbB proton channel family protein	OG_00631	LEC3_5330	transporter, MotA/TolQ/ExbB proton channel family
OG_00632	LG3211_732	LG3211_732; ftsX-like permease family protein	OG_00632	LEC3_0697	efflux ABC transporter, permease protein
OG_00633	LG3211_538	LG3211_538; TPR repeat family protein	OG_00633	LEC3_4847	tetratricopeptide repeat domain protein
OG_00634	LG3211_4970	LG3211_4970; hypothetical protein	OG_00634	LEC3_4881	conserved hypothetical protein
OG_00635	LG3211_4197	maiA; maleylacetoacetate isomerase	OG_00635	LEC3_4256	maleylacetoacetate isomerase
OG_00636	LG3211_1091	LG3211_1091; conserved hypothetical protein	OG_00636	LEC3_1065	conserved hypothetical protein
OG_00637	LG3211_469	LG3211_469; outer membrane beta-barrel domain protein	OG_00637	LEC3_0279	outer membrane protein, FadL family
OG_00638	LG3211_206	LG3211_206; ahpC/TSA family protein	OG_00638	LEC3_5126	SCO1/SenC family protein
OG_00639	LG3211_3042	msbA; lipid A export permease/ATP-binding protein MsbA	OG_00639	LEC3_3033	lipid A export permease/ATP-binding protein MsbA
OG_00640	LG3211_746	LG3211_746; conserved hypothetical protein	OG_00640	LEC3_0710	hypothetical protein
OG_00641	LG3211_3649	LG3211_3649; oligopeptide transporter, OPT family	OG_00641	LEC3_3637	oligopeptide transporter, OPT family
OG_00642	LG3211_144	LG3211_144; histidine phosphatase super family protein	OG_00642	LEC3_5180	phosphohistidine phosphatase SixA
OG_00643	LG3211_2306	dnaK; chaperone protein DnaK	OG_00643	LEC3_2232	chaperone protein dnaK
OG_00644	LG3211_4937	fmt; methionyl-tRNA formyltransferase	OG_00644	LEC3_0357	methionyl-tRNA formyltransferase
OG_00645	LG3211_1329	LG3211_1329; alpha/beta hydrolase family protein	OG_00645	LEC3_1284	hypothetical protein
OG_00646	LG3211_421	LG3211_421; sigma-54 interaction domain protein	OG_00646	LEC3_0236	Mg chelatase family protein
OG_00647	LG3211_3732	pqqE; coenzyme PQQ biosynthesis enzyme PqqE	OG_00647	LEC3_3759	coenzyme PQQ biosynthesis protein E
OG_00648	LG3211_1649	LG3211_1649; acetyltransferase family protein	OG_00648	LEC3_1623	N-acetyltransferase family protein
OG_00649	LG3211_2196	tpiA; triose-phosphate isomerase	OG_00649	LEC3_2157	triose-phosphate isomerase
OG_00650	LG3211_2632	alr; alanine racemase	OG_00650	LEC3_2638	alanine racemase
OG_00651	LG3211_2859	LG3211_2859; putative calU5	OG_00651	LEC3_2871	CalU5
OG_00652	LG3211_2749	LG3211_2749; conserved hypothetical protein	OG_00652	LEC3_2754	conserved hypothetical protein
OG_00653	LG3211_3948	LG3211_3948; DEAD/DEAH box helicase family protein	OG_00653	LEC3_3970	ATP-dependent helicase
OG_00654	LG3211_3195	recX; regulatory recX domain protein	OG_00654	LEC3_3202	regulatory protein RecX
OG_00655	LG3211_2107	LG3211_2107; PAAR motif family protein	OG_00655	LEC3_2046	PAAR motif family protein
OG_00656	LG3211_4654	LG3211_4654; hypothetical protein	OG_00656	LEC3_2218	hypothetical protein
OG_00657	LG3211_4613	pcaD; 3-oxoadipate enol-lactonase	OG_00657	LEC3_0651	3-oxoadipate enol-lactonase
OG_00658	LG3211_2856	LG3211_2856; putative calU3	OG_00658	LEC3_2868	CalU3
OG_00659	LG3211_422	LG3211_422; membrane fusogenic activity family protein	OG_00659	LEC3_0237	conserved hypothetical protein

OG_00660	LG3211_4400	LG3211_4400; TSCPD domain protein	OG_00660	LEC3_4447	ribonucleotide reductase
OG_00661	LG3211_4301	LG3211_4301; conserved hypothetical protein	OG_00661	LEC3_4360	conserved hypothetical protein
OG_00662	LG3211_2126	LG3211_2126; H+ symporter family protein	OG_00662	LEC3_2070	transporter, major facilitator superfamily
OG_00663	LG3211_3126	LG3211_3126; deoR-like helix-turn-helix domain protein	OG_00663	LEC3_3130	transcriptional regulator, DeoR family
OG_00664	LG3211_334	LG3211_334; hemY family protein	OG_00664	LEC3_5037	hemY-like protein
OG_00665	LG3211_4682	LG3211_4682; bacterial regulatory helix-turn-helix, AraC family protei	OG_00665	LEC3_1769	transcriptional regulator, AraC family
OG_00666	LG3211_625	LG3211_625; conserved hypothetical protein	OG_00666	LEC3_4725	conserved hypothetical protein
OG_00667	LG3211_4997	gcvA; gcvA transcriptional dual regulator	OG_00667	LEC3_4907	transcriptional regulator, LysR family
OG_00668	LG3211_4486	LG3211_4486; putative PEPTIDASE	OG_00668	LEC3_4539	prolyl oligopeptidase/S9A/B/C peptidase domain protein
OG_00669	LG3211_800	LG3211_800; CDP-alcohol phosphatidyltransferase family protein	OG_00669	LEC3_0751	CDP-alcohol phosphatidyltransferase
OG_00670	LG3211_1946	LG3211_1946; hisD	OG_00670	LEC3_1898	histidinol dehydrogenase
OG_00671	LG3211_3840	LG3211_3840; conserved hypothetical protein	OG_00671	LEC3_3863	conserved hypothetical protein
OG_00672	LG3211_3619	LG3211_3619; hlyD secretion family protein	OG_00672	LEC3_3610	secretion protein HlyD family protein
OG_00673	LG3211_3707	LG3211_3707; aspartate kinase domain protein	OG_00673	LEC3_3732	aspartate kinase/diaminopimelate decarboxylase
OG_00674	LG3211_1328	LG3211_1328; peroxiredoxin, OsmC subfamily protein	OG_00674	LEC3_1283	OsmC/Ohr family protein
OG_00675	LG3211_2300	smpA; outer membrane protein	OG_00675	LEC3_2227	outer membrane protein
OG_00676	LG3211_906	LG3211_906; glyoxalase/Bleomycin resistance /Dioxygenase superfa	OG_00676	LEC3_0853	glyoxalase/bleomycin resistance protein/dioxygenase
OG_00677	LG3211_556	LG3211_556; putative transmembrane domain protein	OG_00677	LEC3_4823	heavy metal transporter family protein
OG_00678	LG3211_2374	LG3211_2374; acetyltransferase family protein	OG_00678	LEC3_2321	acetyltransferase (GNAT) family
OG_00679	LG3211_3073	LG3211_3073; conserved hypothetical protein	OG_00679	LEC3_3064	membrane protein
OG_00680	LG3211_5007	ftsE; cell division ATP-binding protein FtsE	OG_00680	LEC3_4915	cell division ATP-binding protein FtsE
OG_00681	LG3211_1782	xseA; exodeoxyribonuclease VII, large subunit	OG_00681	LEC3_1745	exodeoxyribonuclease VII, large subunit
OG_00682	LG3211_2324	LG3211_2324; N(4)-(Beta-N-acetylglucosaminyl)-L-asparaginase	OG_00682	LEC3_2264	N(4)-(Beta-N-acetylglucosaminyl)-L-asparaginase
OG_00683	LG3211_961	LG3211_961; right handed beta helix region family protein	OG_00683	LEC3_0917	parallel beta-helix repeat protein
OG_00684	LG3211_5025	LG3211_5025; coproporphyrinogen III oxidase family protein	OG_00684	LEC3_4939	coproporphyrinogen III oxidase, aerobic
OG_00686	LG3211_434	LG3211_434; hypothetical protein	OG_00686	LEC3_0247	conserved hypothetical protein
OG_00687	LG3211_866	leuB; 3-isopropylmalate dehydrogenase	OG_00687	LEC3_0816	3-isopropylmalate dehydrogenase
OG_00688	LG3211_2656	LG3211_2656; putative transmembrane protein	OG_00688	LEC3_2662	conserved hypothetical protein
OG_00689	LG3211_4989	LG3211_4989; PAP2 superfamily protein	OG_00689	LEC3_4900	transmembrane protein
OG_00690	LG3211_354	LG3211_354; phosphoribosylglycinamide synthetase, ATP-grasp dom	OG_00690	LEC3_5019	conserved hypothetical protein
OG_00691	LG3211_2203	nuoE; NADH-quinone oxidoreductase, E subunit	OG_00691	LEC3_2163	NADH-quinone oxidoreductase, E subunit family protein
OG_00692	LG3211_577	LG3211_577; putative aminotransferase	OG_00692	LEC3_4788	aminotransferase class I and II/beta-eliminating lyase
OG_00693	LG3211_175	gluP; glucose/galactose transporter WARNING family protein	OG_00693	LEC3_5156	glucose/galactose transporter
OG_00694	LG3211_133	edd; phosphogluconate dehydratase	OG_00694	LEC3_5191	phosphogluconate dehydratase
OG_00695	LG3211_1232	LG3211_1232; EF hand family protein	OG_00695	LEC3_1187	calcium-binding EF-hand-containing protein
OG_00696	LG3211_1132	mtgA; monofunctional biosynthetic peptidoglycan transglycosylase	OG_00696	LEC3_1109	monofunctional biosynthetic peptidoglycan transglycosylase
OG_00697	LG3211_3547	uvrB; excinuclease ABC subunit B	OG_00697	LEC3_3574	excinuclease ABC, B subunit
OG_00698	LG3211_806	LG3211_806; acyltransferase family protein	OG_00698	LEC3_0757	acyltransferase
OG_00699	LG3211_902	LG3211_902; V-type H(+)-translocating pyrophosphatase	OG_00699	LEC3_0850	V-type H(+)-translocating pyrophosphatase
OG_00700	LG3211_1819	LG3211_1819; glutamine cyclotransferase	OG_00700	LEC3_1778	glutamine cyclotransferase
OG_00701	LG3211_4185	LG3211_4185; NAD dependent epimerase/dehydratase family protein	OG_00701	LEC3_4243	NAD dependent epimerase/dehydratase family protein
OG_00702	LG3211_1643	LG3211_1643; pirin family protein	OG_00702	LEC3_1615	pirin domain protein
OG_00703	LG3211_2552	LG3211_2552; glycosyl hydrolase 9 family protein	OG_00703	LEC3_2536	glycosyl hydrolase, family 9
OG_00704	LG3211_1602	LG3211_1602; acetyltransferase family protein	OG_00704	LEC3_1577	acetyltransferase (GNAT) family
OG_00705	LG3211_4512	LG3211_4512; conserved hypothetical protein	OG_00705	LEC3_4567	molybdopterin-guanine dinucleotide biosynthesis-like protein
OG_00706	LG3211_2726	LG3211_2726; quinone oxidoreductase, YhdH/YhfP family protein	OG_00706	LEC3_2729	quinone oxidoreductase, YhdH/YhfP family
OG_00707	LG3211_4143	LG3211_4143; hypothetical protein	OG_00707	LEC3_4199	hypothetical protein
OG_00708	LG3211_4016	LG3211_4016; cobinamide kinase / cobinamide phosphate guanyltrai	OG_00708	LEC3_4084	cobinamide kinase/cobinamide phosphate guanylyltransferase
OG_00709	LG3211_3355	rlmH; rRNA large subunit m3Psi methyltransferase RlmH	OG_00709	LEC3_3412	conserved hypothetical protein
OG_00710	LG3211_376	LG3211_376; tonB dependent receptor family protein	OG_00710	LEC3_0194	TonB-dependent receptor
OG_00711	LG3211_1618	LG3211_1618; putative aDP-ribosylation/crystallin J1	OG_00711	LEC3_1592	conserved hypothetical protein
OG_00712	LG3211_1890	argB; acetylglutamate kinase	OG_00712	LEC3_1839	acetylglutamate kinase
OG_00713	LG3211_4972	LG3211_4972; glutathione S-transferase, C-terminal domain protein	OG_00713	LEC3_4883	glutathione S-transferase
OG_00714	LG3211_879	thrC; threonine synthase	OG_00714	LEC3_0827	threonine synthase
OG_00715	LG3211_5109	LG3211_5109; cobQ/CobB/MinD/ParA nucleotide binding domain pro	OG_00715	LEC3_0177	CobQ/CobB/MinD/ParA nucleotide binding domain
OG_00716	LG3211_2177	psd; phosphatidylserine decarboxylase	OG_00716	LEC3_2137	phosphatidylserine decarboxylase
OG_00717	LG3211_3179	LG3211_3179; bacterioferritin comigratory protein	OG_00717	LEC3_3185	antioxidant, AhpC/TSA family
OG_00718	LG3211_3605	sspA; stringent starvation protein A	OG_00718	LEC3_3598	stringent starvation protein A
OG_00719	LG3211_616	LG3211_616; peptidase propeptide and YPEB domain protein	OG_00719	LEC3_4732	conserved hypothetical protein
OG_00720	LG3211_1439	LG3211_1439; bacterial regulatory helix-turn-helix , lysR family protei	OG_00720	LEC3_1408	transcriptional regulator, LysR family
OG_00721	LG3211_2429	LG3211_2429; bacterial regulatory , Fis family protein	OG_00721	LEC3_2371	photosynthetic apparatus regulatory protein RegA
OG_00722	LG3211_4161	LG3211_4161; 3-oxoacid CoA-transferase, B subunit	OG_00722	LEC3_4227	succinyl-CoA:3-ketoacid-coenzyme A transferase, subunit B
OG_00723	LG3211_980	LG3211_980; conserved hypothetical protein	OG_00723	LEC3_0939	conserved hypothetical protein
OG_00724	LG3211_3228	trmD; tRNA (guanine(37)-N(1))-methyltransferase	OG_00724	LEC3_3253	tRNA (guanine-N1)-methyltransferase
OG_00725	LG3211_265	LG3211_265; DNA alkylation repair enzyme family protein	OG_00725	LEC3_5063	conserved hypothetical protein
OG_00726	LG3211_3276	LG3211_3276; enoyl-CoA hydratase/isomerase family protein	OG_00726	LEC3_3309	3-hydroxybutyryl-CoA dehydratase
OG_00727	LG3211_1384	LG3211_1384; winged helix DNA-binding domain protein	OG_00727	LEC3_1352	transcriptional regulator, MarR family
OG_00728	LG3211_3155	LG3211_3155; electron transport complex, RnfABCDGE type, B subu	OG_00728	LEC3_3162	electron transport complex, RnfABCDGE type, B subunit protein
OG_00729	LG3211_4061	pilB; type IV-A pilus assembly ATPase PilB	OG_00729	LEC3_4116	type IV pilus assembly protein
OG_00730	LG3211_1338	LG3211_1338; cell Wall Hydrolase family protein	OG_00730	LEC3_1292	cell wall hydrolase
OG_00731	LG3211_3170	pcnB; poly(A) polymerase family protein	OG_00731	LEC3_3179	poly(A) polymerase
OG_00732	LG3211_1677	LG3211_1677; glycosyl transferase 2 family protein	OG_00732	LEC3_1640	conserved hypothetical protein
OG_00733	LG3211_3520	LG3211_3520; acyl-CoA dehydrogenase, N-terminal domain protein	OG_00733	LEC3_3552	acyl-CoA dehydrogenase
OG_00734	LG3211_1464	LG3211_1464; cyclophilin type peptidyl-prolyl cis-trans isomerase/CL	OG_00734	LEC3_1431	conserved hypothetical protein
OG_00735	LG3211_1891	LG3211_1891; acetyltransferase domain protein	OG_00735	LEC3_1840	amino-acid acetyltransferase
OG_00736	LG3211_3741	rpsK; 30S ribosomal protein S11	OG_00736	LEC3_3768	ribosomal protein S11
OG_00737	LG3211_4545	LG3211_4545; SH3 domain of the SH3b1 type family protein	OG_00737	LEC3_4600	conserved hypothetical protein
OG_00738	LG3211_3123	ydiU; conserved protein	OG_00738	LEC3_3122	conserved hypothetical protein
OG_00739	LG3211_5132	LG3211_5132; uracil DNA glycosylase superfamily protein	OG_00739	LEC3_0152	uracil-DNA glycosylase superfamily
OG_00740	LG3211_5058	LG3211_5058; putative fatty acyl-CoA synthetase	OG_00740	LEC3_4974	conserved hypothetical protein
OG_00741	LG3211_935	LG3211_935; cell division ZapA family protein	OG_00741	LEC3_0885	conserved hypothetical protein

OG_00742	LG3211_169	LG3211_169; carbohydrate binding module family protein	OG_00742	LEC3_5161	beta-hexosaminidase
OG_00743	LG3211_5292	LG3211_5292; conserved hypothetical protein	OG_00743	LEC3_1146	cupin domain protein
OG_00744	LG3211_3593	LG3211_3593; marC integral membrane family protein	OG_00744	LEC3_3585	multiple antibiotic resistance
OG_00745	LG3211_4996	azoR1; FMN-dependent NADH-azoreductase 1	OG_00745	LEC3_4905	fmn-dependent NADH-azoreductase 2
OG_00746	LG3211_4091	LG3211_4091; conserved hypothetical protein	OG_00746	LEC3_4145	inner membrane protein Ybcl
OG_00747	LG3211_91	recC; exodeoxyribonuclease V, gamma subunit	OG_00747	LEC3_5232	exodeoxyribonuclease V, gamma subunit
OG_00748	LG3211_2817	LG3211_2817; virB8 family protein	OG_00748	LEC3_2822	VirB8 protein
OG_00749	LG3211_4240	LG3211_4240; hypothetical protein	OG_00749	LEC3_4304	conserved hypothetical protein
OG_00750	LG3211_4949	LG3211_4949; putative secreted protein	OG_00750	LEC3_4856	conserved hypothetical protein
OG_00751	LG3211_4229	LG3211_4229; dnaJ domain protein	OG_00751	LEC3_4289	dnaJ domain
OG_00752	LG3211_439	LG3211_439; flavofamily protein	OG_00752	LEC3_0251	DNA/pantothenate metabolism flavoprotein
OG_00753	LG3211_3223	LG3211_3223; S4 domain protein	OG_00753	LEC3_3246	S4 domain
OG_00754	LG3211_1923	LG3211_1923; hypothetical protein	OG_00754	LEC3_1873	conserved hypothetical protein
OG_00755	LG3211_2317	greA; transcription elongation factor GreA	OG_00755	LEC3_2240	transcription elongation factor GreA
OG_00756	LG3211_2421	LG3211_2421; conserved hypothetical protein	OG_00756	LEC3_2365	conserved hypothetical protein
OG_00757	LG3211_387	LG3211_387; SCP-2 sterol transfer family protein	OG_00757	LEC3_0205	conserved hypothetical protein
OG_00758	LG3211_2459	LG3211_2459; response regulator	OG_00758	LEC3_2449	histidine kinase
OG_00759	LG3211_3912	LG3211_3912; his Kinase A domain protein	OG_00759	LEC3_3934	histidine kinase
OG_00760	LG3211_4341	LG3211_4341; polyketide cyclase / dehydrase and lipid transport fam	OG_00760	LEC3_4384	conserved hypothetical protein
OG_00761	LG3211_2994	LG3211_2994; oxidoreductase molybdopterin binding domain protein	OG_00761	LEC3_2985	UPF0190 protein YedY
OG_00762	LG3211_104	SRY1; threo-3-hydroxyaspartate ammonia-lyase	OG_00762	LEC3_5220	Serine racemase
OG_00763	LG3211_5335	LG3211_5335; putative protein YchJ	OG_00763	LEC3_5393	UPF0225 protein YchJ
OG_00764	LG3211_3894	ksgA; dimethyladenosine transferase	OG_00764	LEC3_3913	dimethyladenosine transferase
OG_00765	LG3211_3887	LG3211_3887; putative transmembrane protein	OG_00765	LEC3_3906	conserved hypothetical protein
OG_00766	LG3211_4952	sppA; signal peptide peptidase SppA, 67K type	OG_00766	LEC3_4859	signal peptide peptidase SppA, 67K type
OG_00767	LG3211_858	LG3211_858; methyltransferase domain protein	OG_00767	LEC3_0808	protein-L-isoaspartate(D-aspartate) O-methyltransferase (PCMT)
OG_00768	LG3211_3852	LG3211_3852; NUDIX domain protein	OG_00768	LEC3_3873	mutT/nudix family protein
OG_00769	LG3211_2886	LG3211_2886; endoribonuclease L-PSP family protein	OG_00769	LEC3_2899	endoribonuclease L-PSP family protein
OG_00770	LG3211_4532	LG3211_4532; conserved hypothetical protein	OG_00770	LEC3_4585	conserved hypothetical protein
OG_00771	LG3211_4542	LG3211_4542; beta-lactamase family protein	OG_00771	LEC3_4597	beta-lactamase
OG_00772	LG3211_990	LG3211_990; acetyl-CoA hydrolase/transferase C-terminal domain pr	OG_00772	LEC3_0950	4-hydroxybutyrate coenzyme A transferase
OG_00773	LG3211_1164	LG3211_1164; conserved hypothetical protein	OG_00773	LEC3_1137	glutaredoxin-like domain
OG_00774	LG3211_1141	prfC; peptide chain release factor 3	OG_00774	LEC3_1118	peptide chain release factor 3
OG_00775	LG3211_1299	sufC; feS assembly ATPase SufC	OG_00775	LEC3_1258	FeS assembly ATPase SufC
OG_00776	LG3211_3834	LG3211_3834; conserved hypothetical protein	OG_00776	LEC3_3857	conserved hypothetical protein
OG_00777	LG3211_3295	LG3211_3295; small Multidrug Resistance family protein	OG_00777	LEC3_3327	multidrug resistance protein, SMR family
OG_00778	LG3211_2751	ligA; DNA ligase, NAD-dependent	OG_00778	LEC3_2756	DNA ligase, NAD-dependent
OG_00779	LG3211_2810	LG3211_2810; type IV secretory pathway, VirB3-like family protein	OG_00779	LEC3_2816	VirB3 protein
OG_00780	LG3211_2470	LG3211_2470; calcineurin-like phosphoesterase family protein	OG_00780	LEC3_4421	protein-tyrosine-phosphatase
OG_00781	LG3211_1342	LG3211_1342; conserved hypothetical protein	OG_00781	LEC3_1295	conserved hypothetical protein
OG_00782	LG3211_2755	LG3211_2755; periplasmic binding and sugar binding domain of LacI	OG_00782	LEC3_2762	bacterial regulatory proteins, lacI family
OG_00783	LG3211_2770	pgl; 6-phosphogluconolactonase	OG_00783	LEC3_2777	6-phosphogluconolactonase
OG_00784	LG3211_3396	LG3211_3396; conserved hypothetical protein	OG_00784	LEC3_3451	conserved hypothetical protein
OG_00785	LG3211_729	LG3211_729; AAA domain family protein	OG_00785	LEC3_0694	C4-dicarboxylate transport transcriptional regulatory protein DctD
OG_00786	LG3211_3378	holA; DNA polymerase III, delta subunit	OG_00786	LEC3_3435	DNA polymerase III, delta subunit
OG_00787	LG3211_4735	LG3211_4735; methyltransferase domain protein	OG_00787	LEC3_0526	O-methyltransferase
OG_00788	LG3211_3679	ssb; single-stranded DNA-binding family protein	OG_00788	LEC3_3716	single-strand binding protein
OG_00789	LG3211_4013	cobS; cobalamin 5'-phosphate synthase	OG_00789	LEC3_4081	cobalamin 5'-phosphate synthase
OG_00790	LG3211_1292	LG3211_1292; PKHD-type hydroxylase Sbal_3634	OG_00790	LEC3_1248	hydroxylase
OG_00791	LG3211_1265	LG3211_1265; conserved hypothetical protein	OG_00791	LEC3_1221	conserved hypothetical protein
OG_00792	LG3211_4518	LG3211_4518; glyoxalase/Bleomycin resistance /Dioxygenase superf	OG_00792	LEC3_4574	glyoxylase family protein
OG_00793	LG3211_4116	xpsN; general secretion pathway protein N	OG_00793	LEC3_4173	general secretion pathway protein N
OG_00794	LG3211_1687	LG3211_1687; isocitrate/isopropylmalate dehydrogenase family prote	OG_00794	LEC3_1654	isocitrate/isopropylmalate dehydrogenase
OG_00795	LG3211_4981	LG3211_4981; HAMP domain protein	OG_00795	LEC3_4890	sensor kinase RpeA
OG_00796	LG3211_4931	LG3211_4931; bacterial PH domain protein	OG_00796	LEC3_0364	bacterial membrane flanked domain
OG_00797	LG3211_2791	LG3211_2791; type IV secretory system Conjugative DNA transfer fa	OG_00797	LEC3_2798	VirD4 protein
OG_00798	LG3211_143	LG3211_143; ycel-like domain protein	OG_00798	LEC3_5181	Ycel like family
OG_00799	LG3211_5186	LG3211_5186; type VII secretion system (T7SS), usher family protein	OG_00799	LEC3_0101	outer membrane usher protein
OG_00800	LG3211_5032	LG3211_5032; hypothetical protein	OG_00800	LEC3_4943	lipoprotein
OG_00801	LG3211_4948	LG3211_4948; conserved hypothetical protein	OG_00801	LEC3_4855	conserved hypothetical protein
OG_00802	LG3211_3109	LG3211_3109; glutathione S-transferase, C-terminal domain protein	OG_00802	LEC3_3112	glutathione S-transferase
OG_00803	LG3211_26	LG3211_26; sensory box protein	OG_00803	LEC3_5310	sensory box histidine kinase
OG_00804	LG3211_606	LG3211_606; conserved hypothetical protein	OG_00804	LEC3_4759	3-demethylubiquinone-9 3-methyltransferase domain protein
OG_00805	LG3211_1528	LG3211_1528; conserved hypothetical protein	OG_00805	LEC3_1504	conserved hypothetical protein
OG_00806	LG3211_3532	ihfA; integration host factor, alpha subunit	OG_00806	LEC3_3563	integration host factor, alpha subunit
OG_00807	LG3211_2249	LG3211_2249; his Kinase A domain protein	OG_00807	LEC3_2201	histidine kinase
OG_00808	LG3211_2298	LG3211_2298; polyketide cyclase / dehydrase and lipid transport fam	OG_00808	LEC3_2225	oligoketide cyclase/lipid transport protein
OG_00809	LG3211_4404	LG3211_4404; peptidase Do family protein	OG_00809	LEC3_4451	periplasmic serine protease, Do/DeqQ family
OG_00810	LG3211_2305	grpE; protein grpE	OG_00810	LEC3_2231	co-chaperone GrpE
OG_00811	LG3211_965	LG3211_965; conserved hypothetical protein	OG_00811	LEC3_0923	conserved hypothetical protein
OG_00812	LG3211_4019	cobD; cobalamin biosynthesis protein CobD	OG_00812	LEC3_4087	cobalamin biosynthesis protein CobD
OG_00813	LG3211_5163	LG3211_5163; RDD family protein	OG_00813	LEC3_0125	RDD family
OG_00814	LG3211_2915	rpmJ; ribosomal protein L36	OG_00814	LEC3_2927	ribosomal protein L36
OG_00815	LG3211_5328	LG3211_5328; amidohydrolase family protein	OG_00815	LEC3_5399	amidohydrolase
OG_00816	LG3211_1230	LG3211_1230; PTS system fructose IIA component family protein	OG_00816	LEC3_1185	PTS system fructose IIA component
OG_00817	LG3211_1590	LG3211_1590; bacterial regulatory helix-turn-helix, AraC family protei	OG_00817	LEC3_1566	transcriptional regulator, AraC family
OG_00818	LG3211_4411	LG3211_4411; HAD hydrolase, IA, variant 1 family protein	OG_00818	LEC3_4457	haloacid dehalogenase-like hydrolase protein
OG_00819	LG3211_1422	LG3211_1422; zinc-binding dehydrogenase family protein	OG_00819	LEC3_1388	alcohol dehydrogenase, zinc-binding
OG_00820	LG3211_1416	LG3211_1416; DNA methylase family protein	OG_00820	LEC3_1381	RNA methylase family
OG_00821	LG3211_5165	tatB; twin arginine-targeting protein translocase TatB	OG_00821	LEC3_0123	twin arginine-targeting protein translocase TatB
OG_00822	LG3211_3749	rpsH; 30S ribosomal subunit protein S8	OG_00822	LEC3_3776	ribosomal protein S8

OG_00823	LG3211_4242	LG3211_4242; transcriptional regulator marR/emrR family	OG_00823	LEC3_4306	transcriptional regulator, MarR family
OG_00824	LG3211_3761	rplW; 50S ribosomal subunit protein L23	OG_00824	LEC3_3788	ribosomal protein L23
OG_00825	LG3211_856	kdtA; 3-deoxy-D-manno-octulosonate(Kdo)-lipid A transferase	OG_00825	LEC3_0803	3-deoxy-D-manno-octulosonic-acid transferase
OG_00826	LG3211_1002	metE; 5-methyltetrahydropteroyltriglutamate-- homocysteine S-methy	OG_00826	LEC3_0965	5-methyltetrahydropteroyltriglutamate-- homocysteine S-methyltransfe
OG_00827	LG3211_4245	kynA; tryptophan 2,3-dioxygenase	OG_00827	LEC3_4310	tryptophan 2,3-dioxygenase
OG_00828	LG3211_3105	LG3211_3105; hypothetical protein	OG_00828	LEC3_3108	hypothetical protein
OG_00829	LG3211_4558	LG3211_4558; bacterial regulatory helix-turn-helix, AraC family protei	OG_00829	LEC3_4613	transcriptional regulator, AraC family
OG_00830	LG3211_366	LG3211_366; pspA/IM30 family protein	OG_00830	LEC3_0183	PspA/IM30 family protein
OG_00831	LG3211_2747	mtnA; S-methyl-5-thioribose-1-phosphate isomerase	OG_00831	LEC3_2752	methylthioribose-1-phosphate isomerase
OG_00832	LG3211_2204	nuoF; NADH oxidoreductase (quinone), F subunit	OG_00832	LEC3_2164	NADH oxidoreductase (quinone), F subunit
OG_00833	LG3211_3053	sdhA; succinate dehydrogenase, flavoprotein subunit	OG_00833	LEC3_3044	succinate dehydrogenase, flavoprotein subunit
OG_00834	LG3211_2378	LG3211_2378; hypothetical protein	OG_00834	LEC3_2324	hypothetical protein
OG_00835	LG3211_5379	rnpA; ribonuclease P protein component	OG_00835	LEC3_5342	ribonuclease P protein component
OG_00836	LG3211_4006	LG3211_4006; regulatory , FmdB family domain protein	OG_00836	LEC3_4071	regulatory protein, FmdB family
OG_00837	LG3211_2753	smc; chromosome segregation protein SMC	OG_00837	LEC3_2758	chromosome segregation protein SMC
OG_00838	LG3211_5015	LG3211_5015; proton antiporter-2 family protein	OG_00838	LEC3_4929	glutathione-regulated potassium-efflux system protein kefB
OG_00839	LG3211_470	LG3211_470; conserved protein	OG_00839	LEC3_0282	mosc domain protein
OG_00840	LG3211_545	LG3211_545; bacterial regulatory helix-turn-helix, AraC family protein	OG_00840	LEC3_4836	transcriptional regulator, AraC family
OG_00841	LG3211_5012	LG3211_5012; A pre-toxin domain with the TG motif family protein	OG_00841	LEC3_4926	conserved hypothetical protein
OG_00842	LG3211_384	LG3211_384; AMP-binding enzyme family protein	OG_00842	LEC3_0202	AMP-binding enzyme
OG_00843	LG3211_573	grxD; monothiol glutaredoxin, Grx4 family	OG_00843	LEC3_4795	glutaredoxin
OG_00844	LG3211_5176	LG3211_5176; conserved hypothetical protein	OG_00844	LEC3_0113	conserved domain protein
OG_00845	LG3211_460	LG3211_460; MAPEG family protein	OG_00845	LEC3_0269	membrane protein
OG_00846	LG3211_1370	LG3211_1370; histidine phosphatase super family protein	OG_00846	LEC3_1333	phosphoglycerate mutase family protein
OG_00847	LG3211_4014	LG3211_4014; histidine phosphatase super family protein	OG_00847	LEC3_4082	phosphoglycerate mutase family protein
OG_00848	LG3211_4975	LG3211_4975; bacterial regulatory , arsR family protein	OG_00848	LEC3_4886	arsenical resistance operon repressor
OG_00849	LG3211_4250	pdhB; pyruvate dehydrogenase E1 component subunit beta	OG_00849	LEC3_4317	pyruvate dehydrogenase E1 component, beta subunit
OG_00850	LG3211_2067	LG3211_2067; peptidase M13 family protein	OG_00850	LEC3_2006	peptidase, family M13
OG_00851	LG3211_5022	cydB; cytochrome d ubiquinol oxidase, subunit II	OG_00851	LEC3_4933	cytochrome d ubiquinol oxidase, subunit II
OG_00852	LG3211_492	LG3211_492; dienelactone hydrolase family protein	OG_00852	LEC3_0304	peptidase, S9A/B/C families, catalytic domain
OG_00853	LG3211_2996	LG3211_2996; FHA domain protein	OG_00853	LEC3_2987	FHA domain
OG_00854	LG3211_1012	LG3211_1012; conserved hypothetical protein	OG_00854	LEC3_0976	conserved hypothetical protein
OG_00855	LG3211_2243	LG3211_2243; tat (twin-arginine translocation) pathway signal sequer	OG_00855	LEC3_2195	Tat (twin-arginine translocation) pathway signal sequence protein
OG_00856	LG3211_1420	LG3211_1420; putative transmembrane protein	OG_00856	LEC3_1385	conserved hypothetical protein
OG_00857	LG3211_2624	LG3211_2624; glycine zipper family protein	OG_00857	LEC3_2634	outer membrane protein, OmpA family
OG_00858	LG3211_4548	LG3211_4548; conserved hypothetical protein	OG_00858	LEC3_4603	conserved hypothetical protein
OG_00859	LG3211_2119	LG3211_2119; glutamine amidotransferase class-I family protein	OG_00859	LEC3_2065	peptidase C26
OG_00860	LG3211_865	LG3211_865; FAD binding domain protein	OG_00860	LEC3_0815	FAD dependent oxidoreductase
OG_00861	LG3211_914	LG3211_914; short chain dehydrogenase	OG_00861	LEC3_0866	oxidoreductase, short chain dehydrogenase/reductase family
OG_00862	LG3211_5040	LG3211_5040; dipeptidyl peptidase IV	OG_00862	LEC3_4950	dipeptidyl peptidase IV
OG_00863	LG3211_135	LG3211_135; HAD hydrolase, IA, variant 1 family protein	OG_00863	LEC3_5190	HAD-superfamily hydrolase, subfamily IA, variant 3
OG_00864	LG3211_2997	LG3211_2997; polyhydroxyalkanoic acid system family protein	OG_00864	LEC3_2988	conserved hypothetical protein
OG_00865	LG3211_5180	LG3211_5180; hypothetical protein	OG_00865	LEC3_0109	membrane protein
OG_00866	LG3211_4640	LG3211_4640; oxidoreductase , NAD-binding Rossmann fold family p	OG_00866	LEC3_2248	oxidoreductase, zinc-binding dehydrogenase family/NAD-binding Ros
OG_00867	LG3211_2803	LG3211_2803; conserved hypothetical protein	OG_00867	LEC3_2811	conserved hypothetical protein
OG_00868	LG3211_491	LG3211_491; DNA/RNA non-specific endonuclease family protein	OG_00868	LEC3_0303	conserved hypothetical protein
OG_00869	LG3211_2893	LG3211_2893; conserved hypothetical protein	OG_00869	LEC3_2907	conserved hypothetical protein
OG_00870	LG3211_5233	LG3211_5233; endoribonuclease L-PSP family protein	OG_00870	LEC3_0057	endoribonuclease L-PSP family protein
OG_00871	LG3211_3872	LG3211_3872; bacterial regulatory helix-turn-helix , lysR family protei	OG_00871	LEC3_3887	transcriptional regulator, LysR family/LysR substrate binding domain
OG_00872	LG3211_937	LG3211_937; diguanylate cyclase domain protein	OG_00872	LEC3_0894	sensory box protein
OG_00873	LG3211_138	LG3211_138; putative esterase HI_1161	OG_00873	LEC3_5187	esterase
OG_00874	LG3211_394	LG3211_394; bacterial regulatory, gntR family protein	OG_00874	LEC3_0218	transcriptional regulator, GntR family
OG_00875	LG3211_2663	LG3211_2663; modulator of DNA gyrase family protein	OG_00875	LEC3_2669	TldD/PmbA family protein
OG_00876	LG3211_3835	LG3211_3835; bacterial regulatory, gntR family protein	OG_00876	LEC3_3858	bacterial regulatory proteins, gntR family/aminotransferase class I an
OG_00877	LG3211_3322	frr; ribosome recycling factor	OG_00877	LEC3_3374	ribosome recycling factor
OG_00878	LG3211_1175	LG3211_1175; WGR domain protein	OG_00878	LEC3_1148	WGR domain protein
OG_00879	LG3211_1611	LG3211_1611; conserved hypothetical protein	OG_00879	LEC3_1585	hypothetical protein
OG_00880	LG3211_3517	LG3211_3517; histidine kinase-, DNA gyrase B-, and HSP90-like ATF	OG_00880	LEC3_3549	histidine kinase
OG_00881	LG3211_579	rsgA; ribosome small subunit-dependent GTPase A	OG_00881	LEC3_4786	ribosome associated GTPase
OG_00882	LG3211_335	LG3211_335; hemX family protein	OG_00882	LEC3_5036	putative uroporphyrin-III C-methyltransferase
OG_00883	LG3211_3058	LG3211_3058; transketolase, pyrimidine binding domain protein	OG_00883	LEC3_3052	transketolase
OG_00884	LG3211_3465	LG3211_3465; conserved hypothetical protein	OG_00884	LEC3_3523	hypothetical protein
OG_00885	LG3211_4278	LG3211_4278; dihydrouridine synthase family protein	OG_00885	LEC3_4345	tRNA-dihydrouridine synthase A
OG_00886	LG3211_5195	LG3211_5195; acetyltransferase family protein	OG_00886	LEC3_0092	acetyltransferase, GNAT family
OG_00887	LG3211_553	LG3211_553; deoR-like helix-turn-helix domain protein	OG_00887	LEC3_4828	transcriptional regulator, DoeR family
OG_00888	LG3211_401	LG3211_401; TPR repeat family protein	OG_00888	LEC3_0227	tetratricopeptide repeat domain protein
OG_00889	LG3211_3288	LG3211_3288; conserved hypothetical protein	OG_00889	LEC3_3320	conserved hypothetical protein
OG_00890	LG3211_391	LG3211_391; hypothetical protein	OG_00890	LEC3_0213	hypothetical protein
OG_00891	LG3211_685	LG3211_685; hypothetical protein	OG_00891	LEC3_4665	conserved hypothetical protein
OG_00892	LG3211_1045	LG3211_1045; GDSL-like Lipase/Acylhydrolase family protein	OG_00892	LEC3_1012	lipase/esterase
OG_00893	LG3211_4327	HDDC3; HD domain-containing protein 3	OG_00893	LEC3_4379	metal dependent phosphohydrolase
OG_00894	LG3211_1720	LG3211_1720; DNA-binding protein, YbaB/EbfC family	OG_00894	LEC3_1676	conserved hypothetical protein
OG_00895	LG3211_820	LG3211_820; ahpC/TSA family protein	OG_00895	LEC3_0770	redoxin/antioxidant, AhpC/TSA family
OG_00896	LG3211_4127	purL; phosphoribosylformylglycinamidine synthase	OG_00896	LEC3_4186	phosphoribosylformylglycinamidine synthase
OG_00897	LG3211_1242	LG3211_1242; putative isomerase	OG_00897	LEC3_1197	arabinose 5-phosphate isomerase
OG_00898	LG3211_2187	trpB; tryptophan synthase, beta subunit	OG_00898	LEC3_2147	tryptophan synthase, beta subunit
OG_00899	LG3211_1324	LG3211_1324; mechanosensitive ion channel family protein	OG_00899	LEC3_1279	transporter, small conductance mechanosensitive ion channel (MscS)
OG_00900	LG3211_3754	rpsQ; 30S ribosomal protein S17	OG_00900	LEC3_3781	ribosomal protein S17
OG_00901	LG3211_1572	LG3211_1572; drug resistance transporter, Bcr/CflA subfamily protein	OG_00901	LEC3_1549	drug resistance transporter, Bcr/CflA subfamily
OG_00902	LG3211_4029	gloA; lactoylglutathione lyase	OG_00902	LEC3_4099	lactoylglutathione lyase
OG_00903	LG3211_3354	LG3211_3354; tonB family C-terminal domain protein	OG_00903	LEC3_3411	TonB family C-terminal domain

OG_00904	LG3211_3257	mviN; integral membrane protein MviN	OG_00904	LEC3_3289	integral membrane protein MviN
OG_00905	LG3211_3730	pqqC; coenzyme PQQ biosynthesis protein C	OG_00905	LEC3_3757	coenzyme PQQ biosynthesis protein C
OG_00906	LG3211_2220	truB; tRNA pseudouridine(55) synthase	OG_00906	LEC3_2178	tRNA pseudouridine synthase B
OG_00907	LG3211_2922	LG3211_2922; alpha/beta hydrolase fold family protein	OG_00907	LEC3_2934	hydrolase
OG_00908	LG3211_2668	amnD; 2-aminomuconate deaminase	OG_00908	LEC3_2674	endoribonuclease L-PSP
OG_00909	LG3211_2606	lnt; apolipoprotein N-acyltransferase	OG_00909	LEC3_3228	apolipoprotein N-acyltransferase
OG_00910	LG3211_2202	nuoD; NADH dehydrogenase (quinone), D subunit	OG_00910	LEC3_2162	NADH dehydrogenase, D subunit
OG_00911	LG3211_4412	LG3211_4412; cytosol aminopeptidase	OG_00911	LEC3_4458	cytosol aminopeptidase family, catalytic domain
OG_00912	LG3211_3768	rpsL; ribosomal protein S12	OG_00912	LEC3_3795	ribosomal protein S12
OG_00913	LG3211_829	LG3211_829; helix-turn-helix family protein	OG_00913	LEC3_0779	HTH type transcriptional regulator
OG_00914	LG3211_1591	LG3211_1591; amidohydrolase family protein	OG_00914	LEC3_1569	amidohydrolase family protein
OG_00915	LG3211_3949	LG3211_3949; TPR repeat family protein	OG_00915	LEC3_3971	tetratricopeptide repeat domain protein
OG_00916	LG3211_1228	ptsP; phosphoenolpyruvate-protein phosphotransferase	OG_00916	LEC3_1183	phosphotransferase system enzyme I
OG_00917	LG3211_4406	LG3211_4406; putative transmembrane protein	OG_00917	LEC3_4453	conserved hypothetical protein
OG_00918	LG3211_5340	icd; isocitrate dehydrogenase, NADP-dependent	OG_00918	LEC3_5386	isocitrate dehydrogenase
OG_00919	LG3211_4426	LG3211_4426; acetyltransferase family protein	OG_00919	LEC3_4469	acetyltransferase (GNAT) family
OG_00920	LG3211_817	accB; acetyl-CoA carboxylase, biotin carboxyl carrier protein	OG_00920	LEC3_0768	acetyl-CoA carboxylase, biotin carboxyl carrier protein
OG_00921	LG3211_1734	fabD; malonyl CoA-acyl carrier protein transacylase	OG_00921	LEC3_1691	malonyl CoA-acyl carrier protein transacylase
OG_00922	LG3211_1729	maf; septum formation protein Maf	OG_00922	LEC3_1685	septum formation protein Maf
OG_00923	LG3211_3602	nadC; nicotinate-nucleotide diphosphorylase	OG_00923	LEC3_3594	nicotinate-nucleotide pyrophosphorylase
OG_00924	LG3211_2423	LG3211_2423; major Facilitator Superfamily protein	OG_00924	LEC3_2367	fosmidomycin resistance protein (major facilitator superfamily)
OG_00925	LG3211_101	LG3211_101; conserved hypothetical protein	OG_00925	LEC3_5223	conserved hypothetical protein
OG_00926	LG3211_2925	LG3211_2925; MMPL family protein	OG_00926	LEC3_2936	RND superfamily protein
OG_00927	LG3211_2432	cyoD; cytochrome o ubiquinol oxidase subunit IV	OG_00927	LEC3_2374	cytochrome o ubiquinol oxidase subunit IV
OG_00928	LG3211_2448	LG3211_2448; peptidase M23 family protein	OG_00928	LEC3_2385	M23 peptidase domain protein
OG_00929	LG3211_4483	LG3211_4483; prokaryotic lipoprotein-attachment site family protein	OG_00929	LEC3_4535	conserved hypothetical protein
OG_00930	LG3211_4474	LG3211_4474; cytidine and deoxycytidylate deaminase zinc-binding r	OG_00930	LEC3_4525	cytidine and deoxycytidylate deaminase zinc-binding region
OG_00931	LG3211_768	LG3211_768; putative uvs125	OG_00931	LEC3_0730	transmembrane protein
OG_00932	LG3211_3333	LG3211_3333; ribonuclease HII family protein	OG_00932	LEC3_3385	ribonuclease HII
OG_00933	LG3211_2709	LG3211_2709; ABC-2 transporter family protein	OG_00933	LEC3_2710	conserved hypothetical protein
OG_00935	LG3211_1333	prpB; methylisocitrate lyase	OG_00935	LEC3_1288	methylisocitrate lyase
OG_00936	LG3211_3434	LG3211_3434; rhomboid family protein	OG_00936	LEC3_3497	peptidase, S54 (rhomboid) family
OG_00937	LG3211_1337	GST; glutathione S-transferase	OG_00937	LEC3_1291	glutathione S-transferase
OG_00938	LG3211_4063	LG3211_4063; type IV leader peptidase family protein	OG_00938	LEC3_4118	Type IV prepilin-like proteins leader peptide-processing enzyme
OG_00939	LG3211_3111	LG3211_3111; zinc carboxypeptidase family protein	OG_00939	LEC3_3113	zinc carboxypeptidase
OG_00940	LG3211_3983	pal; peptidoglycan-associated lipoprotein	OG_00940	LEC3_4051	peptidoglycan-associated lipoprotein
OG_00941	LG3211_899	LG3211_899; hemolysin-type calcium-binding repeat family protein	OG_00941	LEC3_0845	hemolysin-type calcium-binding repeat
OG_00942	LG3211_4460	mrdA; penicillin-binding protein 2	OG_00942	LEC3_4510	penicillin binding protein
OG_00943	LG3211_2167	LG3211_2167; helix-turn-helix family protein	OG_00943	LEC3_2123	HTH type transcriptional regulator
OG_00944	LG3211_3246	LG3211_3246; hypothetical protein	OG_00944	LEC3_3275	conserved hypothetical protein
OG_00945	LG3211_2619	LG3211_2619; cytochrome C oxidase, cbb3-type, subunit III family pr	OG_00945	LEC3_2630	conserved hypothetical protein
OG_00946	LG3211_3704	LG3211_3704; prolyl oligopeptidase family protein	OG_00946	LEC3_3729	dienelactone hydrolase family protein
OG_00947	LG3211_4252	LG3211_4252; 2-oxoacid dehydrogenases acyltransferase family prot	OG_00947	LEC3_4319	2-oxoacid dehydrogenases acyltransferase (catalytic domain)/biotin-r
OG_00948	LG3211_3995	ruvC; crossover junction endodeoxyribonuclease RuvC	OG_00948	LEC3_4062	crossover junction endodeoxyribonuclease
OG_00949	LG3211_1631	LG3211_1631; putative membrane protein	OG_00949	LEC3_1600	pathogenicity-related protein
OG_00950	LG3211_4469	LG3211_4469; cold-active aminopeptidase	OG_00950	LEC3_4519	leukotriene A-4 hydrolase
OG_00951	LG3211_3469	LG3211_3469; conserved hypothetical protein	OG_00951	LEC3_3526	conserved hypothetical protein
OG_00952	LG3211_3994	ruvA; holliday junction DNA helicase RuvA	OG_00952	LEC3_4061	holliday junction DNA helicase
OG_00953	LG3211_5045	LG3211_5045; helix-turn-helix family protein	OG_00953	LEC3_4959	HTH type transcriptional regulator
OG_00954	LG3211_2734	pbpC; penicillin-binding protein 1C	OG_00954	LEC3_2737	penicillin-binding protein 1C
OG_00955	LG3211_52	LG3211_52; conserved hypothetical protein	OG_00955	LEC3_5269	alkylhydroperoxidase AhpD core protein
OG_00956	LG3211_3747	rplR; ribosomal protein L18	OG_00956	LEC3_3774	ribosomal protein L18
OG_00957	LG3211_4944	topA; DNA topoisomerase I	OG_00957	LEC3_0349	DNA topoisomerase I
OG_00958	LG3211_724	LG3211_724; conserved hypothetical protein	OG_00958	LEC3_3649	endo-beta-N-acetylglucosaminidase H
OG_00959	LG3211_1952	hisE; phosphoribosyl-ATP diphosphatase	OG_00959	LEC3_1904	phosphoribosyl-ATP pyrophosphohydrolase
OG_00960	LG3211_381	LG3211_381; hpcH/HpaI aldolase/citrate lyase family protein	OG_00960	LEC3_0200	HpcH/HpaI aldolase/citrate lyase family protein
OG_00961	LG3211_804	LG3211_804; conserved hypothetical protein	OG_00961	LEC3_0755	conserved hypothetical protein
OG_00962	LG3211_3176	LG3211_3176; putative secreted protein	OG_00962	LEC3_3182	lipoprotein
OG_00963	LG3211_5313	LG3211_5313; conserved enzyme	OG_00963	LEC3_5423	conserved hypothetical protein
OG_00964	LG3211_3187	kdpC; K+-transporting ATPase, C subunit	OG_00964	LEC3_3194	K+-transporting ATPase, C subunit
OG_00965	LG3211_2769	glk; glucokinase	OG_00965	LEC3_2776	glucokinase
OG_00966	LG3211_646	LG3211_646; conserved hypothetical protein	OG_00966	LEC3_4701	conserved hypothetical protein
OG_00967	LG3211_1448	LG3211_1448; glutathione-dependent formaldehyde-activating enzym	OG_00967	LEC3_1416	glutathione-dependent formaldehyde-activating protein
OG_00968	LG3211_165	LG3211_165; sensory box protein	OG_00968	LEC3_5165	sensory box histidine kinase
OG_00969	LG3211_3060	LG3211_3060; acetyltransferase domain protein	OG_00969	LEC3_3054	acetyltransferase (GNAT) family
OG_00970	LG3211_1322	LG3211_1322; lysE type translocator family protein	OG_00970	LEC3_1277	translocator protein, LysE family
OG_00971	LG3211_1640	LG3211_1640; hypothetical protein	OG_00971	LEC3_1609	hypothetical protein
OG_00972	LG3211_258	LG3211_258; FKBP-type peptidyl-prolyl cis-trans isomerase family pr	OG_00972	LEC3_5073	peptidyl-prolyl cis-trans isomerase, FKBP-type
OG_00973	LG3211_2365	infA; translation initiation factor IF-1	OG_00973	LEC3_2311	translation initiation factor IF-1
OG_00974	LG3211_2299	LG3211_2299; thiS family protein	OG_00974	LEC3_2226	UPF0125 protein
OG_00975	LG3211_4207	LG3211_4207; 4Fe-4S binding domain protein	OG_00975	LEC3_4262	glycolate oxidase, iron-sulfur subunit protein
OG_00976	LG3211_1090	LG3211_1090; conserved hypothetical protein	OG_00976	LEC3_1063	conserved hypothetical protein
OG_00977	LG3211_2238	LG3211_2238; bacterial regulatory helix-turn-helix , lysR family protei	OG_00977	LEC3_2192	transcriptional regulator, LysR family
OG_00978	LG3211_2892	yfgL; outer membrane assembly lipoprotein YfgL	OG_00978	LEC3_2906	outer membrane assembly lipoprotein YfgL
OG_00979	LG3211_2352	acnB; aconitate hydratase 2	OG_00979	LEC3_2298	aconitate hydratase 2
OG_00980	LG3211_2104	LG3211_2104; conserved hypothetical protein	OG_00980	LEC3_2043	conserved hypothetical protein
OG_00981	LG3211_5346	LG3211_5346; bacterial regulatory helix-turn-helix , lysR family protei	OG_00981	LEC3_5375	transcriptional regulator, LysR family
OG_00982	LG3211_570	LG3211_570; conserved hypothetical protein	OG_00982	LEC3_4800	conserved hypothetical protein
OG_00983	LG3211_5173	LG3211_5173; exonuclease, RdgC family protein	OG_00983	LEC3_0115	recombination-associated protein RdgC
OG_00984	LG3211_794	LG3211_794; conserved hypothetical protein	OG_00984	LEC3_0744	surface layer protein
OG_00985	LG3211_2033	LG3211_2033; carboxyl transferase domain protein	OG_00985	LEC3_1967	methylcrotonoyl-CoA carboxylase beta chain,

OG_00986	LG3211_4473	LG3211_4473; acetyltransferase domain protein	OG_00986	LEC3_4523	conserved hypothetical protein
OG_00987	LG3211_4555	LG3211_4555; 2Fe-2S iron-sulfur cluster binding domain protein	OG_00987	LEC3_4610	ferredoxin
OG_00988	LG3211_1467	phaZ; polyhydroxyalkanoate depolymerase, intracellular family protei	OG_00988	LEC3_1434	polyhydroxyalkanoate depolymerase, intracellular
OG_00989	LG3211_2182	LG3211_2182; D-isomer specific 2-hydroxyacid dehydrogenase, cata	OG_00989	LEC3_2141	D-isomer specific 2-hydroxyacid dehydrogenase
OG_00990	LG3211_5301	LG3211_5301; 4'-phosphopantetheinyl transferase superfamily protei	OG_00990	LEC3_5440	4'-phosphopantetheinyl transferase superfamily
OG_00991	LG3211_4121	LG3211_4121; prepilin-type N-terminal cleavage/methylation domain	OG_00991	LEC3_4178	general secretion pathway protein I
OG_00992	LG3211_4946	LG3211_4946; protein involved in synthesis of threonylcarbamoylade	OG_00992	LEC3_4853	Sua5/YciO/YrdC/YwlC family protein
OG_00993	LG3211_5325	LG3211_5325; bacterial regulatory helix-turn-helix , lysR family protei	OG_00993	LEC3_5406	putative HTH-type transcriptional regulator YcaN
OG_00994	LG3211_3595	grxD; monothiol glutaredoxin, Grx4 family	OG_00994	LEC3_3587	glutaredoxin-4
OG_00995	LG3211_761	bfr; bacterioferritin	OG_00995	LEC3_0723	bacterioferritin
OG_00996	LG3211_3745	rpmD; ribosomal protein L30	OG_00996	LEC3_3772	ribosomal protein L30
OG_00997	LG3211_4973	LG3211_4973; conserved hypothetical protein	OG_00997	LEC3_4884	metallo-beta-lactamase family protein
OG_00998	LG3211_5035	uvrD; DNA helicase II	OG_00998	LEC3_4946	DNA helicase II
OG_00999	LG3211_3464	LG3211_3464; aminotransferase class-III family protein	OG_00999	LEC3_3522	aminotransferase class-III
OG_01000	LG3211_2297	smpB; ssrA-binding protein	OG_01000	LEC3_2224	SsrA-binding protein
OG_01001	LG3211_2795	LG3211_2795; hypothetical protein	OG_01001	LEC3_2802	lipoprotein
OG_01002	LG3211_4241	hppD; 4-hydroxyphenylpyruvate dioxygenase	OG_01002	LEC3_4305	4-hydroxyphenylpyruvate dioxygenase SEQUENCING GAP
OG_01003	LG3211_1536	hflX; GTP-binding protein HflX	OG_01003	LEC3_1511	GTP-binding proten HflX
OG_01004	LG3211_3352	LG3211_3352; hypothetical protein	OG_01004	LEC3_3410	clumping factor B precusor
OG_01005	LG3211_3733	pam; peptide amidase	OG_01005	LEC3_3761	amidase
OG_01006	LG3211_3524	dxs; 1-deoxy-D-xylulose-5-phosphate synthase	OG_01006	LEC3_3556	1-deoxy-D-xylulose-5-phosphate synthase
OG_01007	LG3211_3966	LG3211_3966; hypothetical protein	OG_01007	LEC3_3990	lipoprotein
OG_01008	LG3211_2460	LG3211_2460; cheB methylesterase family protein	OG_01008	LEC3_2448	protein-glutamate methylesterase CheB
OG_01009	LG3211_3279	LG3211_3279; phosphate-selective porin O and P family protein	OG_01009	LEC3_3312	phosphate-selective porin O and P
OG_01010	LG3211_3893	pdxA; 4-hydroxythreonine-4-phosphate dehydrogenase	OG_01010	LEC3_3912	4-hydroxythreonine-4-phosphate dehydrogenase
OG_01011	LG3211_5050	LG3211_5050; beta-ketoacyl synthase, N-terminal domain protein	OG_01011	LEC3_4968	conserved hypothetical protein
OG_01012	LG3211_252	LG3211_252; bacterial regulatory, tetR family protein	OG_01012	LEC3_5077	transcriptional regulator, TetR family
OG_01013	LG3211_2669	nbaC; 3-hydroxyanthranilate 3,4-dioxygenase	OG_01013	LEC3_2675	3-hydroxyanthranilate 3,4-dioxygenase
OG_01014	LG3211_3513	LG3211_3513; subtilase family protein	OG_01014	LEC3_3545	peptidase, families S8 and S53
OG_01015	LG3211_5208	LG3211_5208; peptidase inhibitor I78 family protein	OG_01015	LEC3_0081	conserved hypothetical protein
OG_01016	LG3211_2789	LG3211_2789; FAD binding domain protein	OG_01016	LEC3_2795	FAD dependent oxidoreductase
OG_01017	LG3211_2017	LG3211_2017; hypothetical protein	OG_01017	LEC3_1949	hypothetical protein
OG_01018	LG3211_1662	LG3211_1662; conserved hypothetical protein	OG_01018	LEC3_1625	conserved hypothetical protein
OG_01019	LG3211_3525	LG3211_3525; conserved hypothetical protein	OG_01019	LEC3_3557	conserved hypothetical protein
OG_01020	LG3211_4424	LG3211_4424; triose-phosphate Transporter family protein	OG_01020	LEC3_4467	rarD protein
OG_01021	LG3211_1483	LG3211_1483; nlpC/P60 family protein	OG_01021	LEC3_1459	outer membrane lipoprotein
OG_01022	LG3211_119	LG3211_119; prokaryotic cytochrome b561 family protein	OG_01022	LEC3_5205	cytochrome B561
OG_01023	LG3211_2405	LG3211_2405; short chain dehydrogenase family protein	OG_01023	LEC3_2347	oxidoreductase, short chain dehydrogenase/reductase family
OG_01024	LG3211_1387	LG3211_1387; hypothetical protein	OG_01024	LEC3_1355	ribosomal-like protein
OG_01025	LG3211_4130	LG3211_4130; RDD family protein	OG_01025	LEC3_4190	RDD family
OG_01026	LG3211_5072	LG3211_5072; hypothetical protein	OG_01026	LEC3_4986	hypothetical protein
OG_01027	LG3211_5305	LG3211_5305; conserved hypothetical protein	OG_01027	LEC3_5435	conserved hypothetical protein
OG_01028	LG3211_2200	LG3211_2200; NADH-quinone oxidoreductase, B subunit	OG_01028	LEC3_2160	NADH-quinone oxidoreductase, B subunit family protein
OG_01029	LG3211_2841	LG3211_2841; NRDE family protein	OG_01029	LEC3_2852	conserved hypothetical protein
OG_01030	LG3211_3603	LG3211_3603; conserved hypothetical protein	OG_01030	LEC3_3595	conserved hypothetical protein
OG_01031	LG3211_2695	LG3211_2695; conserved hypothetical protein	OG_01031	LEC3_2696	hypothetical protein
OG_01032	LG3211_886	LG3211_886; ion channel family protein	OG_01032	LEC3_0836	Ion channel family protein
OG_01033	LG3211_3198	lexA; repressor LexA	OG_01033	LEC3_3205	LexA repressor
OG_01034	LG3211_1594	LG3211_1594; angiotensin-converting enzyme family protein	OG_01034	LEC3_1570	dipeptidyl carboxydipeptidase family
OG_01035	LG3211_559	LG3211_559; ADP-ribosylglycohydrolase	OG_01035	LEC3_4820	ADP-ribosylation/Crystallin J1
OG_01036	LG3211_4420	LG3211_4420; ftsX-like permease family protein	OG_01036	LEC3_4464	efflux ABC transporter, permease protein
OG_01037	LG3211_744	LG3211_744; hypothetical protein	OG_01037	LEC3_0708	hypothetical protein
OG_01038	LG3211_1776	LG3211_1776; RNA polymerase sigma factor, sigma-70 family protei	OG_01038	LEC3_1738	RNA polymerase sigma factor, sigma-70 family
OG_01039	LG3211_1096	LG3211_1096; nfeD-like C-terminal, partner-binding family protein	OG_01039	LEC3_1070	Nodulation efficiency protein D
OG_01040	LG3211_2266	LG3211_2266; acyl-CoA dehydrogenase, N-terminal domain protein	OG_01040	LEC3_0576	acyl-coa dehydrogenase
OG_01041	LG3211_1885	LG3211_1885; conserved hypothetical protein	OG_01041	LEC3_1834	conserved hypothetical protein
OG_01042	LG3211_2396	LG3211_2396; potH	OG_01042	LEC3_2341	ABC transporter, permease protein
OG_01043	LG3211_4273	LG3211_4273; phosphoenolpyruvate:glucose-phosphotransferase re	OG_01043	LEC3_4340	conserved hypothetical protein
OG_01044	LG3211_1223	LG3211_1223; conserved hypothetical protein	OG_01044	LEC3_1178	holliday junction resolvase
OG_01045	LG3211_1427	LG3211_1427; polyketide cyclase / dehydrase and lipid transport fam	OG_01045	LEC3_1394	conserved hypothetical protein
OG_01046	LG3211_3453	secF; protein-export membrane protein SecF	OG_01046	LEC3_3512	protein-export membrane protein SecF
OG_01047	LG3211_5057	LG3211_5057; MMPL family protein	OG_01047	LEC3_4973	membrane protein
OG_01048	LG3211_5028	polA; DNA polymerase I family protein	OG_01048	LEC3_4940	DNA polymerase I
OG_01049	LG3211_1896	proA; glutamate-5-semialdehyde dehydrogenase	OG_01049	LEC3_1845	gamma-glutamyl phosphate reductase
OG_01050	LG3211_3748	rplF; ribosomal protein L6	OG_01050	LEC3_3775	ribosomal protein L6
OG_01051	LG3211_4222	LG3211_4222; response regulator	OG_01051	LEC3_4282	Transcriptional regulatory proteins, C terminal - transcriptional regulat
OG_01052	LG3211_3808	wrbA; quinone oxidoreductase, type IV	OG_01052	LEC3_3831	NAD(P)H:quinone oxidoreductase, type IV
OG_01053	LG3211_1922	LG3211_1922; alpha-1,2-mannosidase family protein	OG_01053	LEC3_1872	alpha-1,2-mannosidase
OG_01054	LG3211_1391	hutI; imidazolonepropionase	OG_01054	LEC3_1359	imidazolonepropionase
OG_01055	LG3211_1044	thiS; thiamine biosynthesis protein ThiS	OG_01055	LEC3_1011	thiamine biosynthesis protein ThiS
OG_01056	LG3211_3958	LG3211_3958; conserved hypothetical protein	OG_01056	LEC3_3980	conserved hypothetical protein
OG_01057	LG3211_1098	LG3211_1098; hypothetical protein	OG_01057	LEC3_1071	conserved hypothetical protein
OG_01058	LG3211_345	LG3211_345; putative peptidoglycan binding domain protein	OG_01058	LEC3_5027	peptidoglycan-binding domain protein
OG_01059	LG3211_5378	yidC; membrane protein insertase, YidC/Oxa1 family, N-terminal dom	OG_01059	LEC3_5343	inner membrane protein OxaA
OG_01060	LG3211_3471	LG3211_3471; hypothetical protein	OG_01060	LEC3_3528	nuclease
OG_01061	LG3211_4928	LG3211_4928; bacterial lipid A biosynthesis acyltransferase family pr	OG_01061	LEC3_0366	lipid A biosynthesis (KDO)2-(lauroyl)-lipid IVA acyltransferase
OG_01062	LG3211_149	LG3211_149; membrane transport family protein	OG_01062	LEC3_5176	auxin Efflux Carrier
OG_01063	LG3211_2717	LG3211_2717; metallo-beta-lactamase superfamily protein	OG_01063	LEC3_2717	metallo-beta-lactamase L1 (Beta-lactamase type II) (Penicillinase)
OG_01064	LG3211_2991	hisC; histidinol-phosphate transaminase	OG_01064	LEC3_2982	histidinol-phosphate aminotransferase
OG_01065	LG3211_3129	LG3211_3129; glutamine amidotransferase class-I family protein	OG_01065	LEC3_3132	glutamine amidotransferase class-I
OG_01066	LG3211_4081	murE; UDP-N-acetylmuramyl-tripeptide synthetase family protein	OG_01066	LEC3_4135	UDP-N-acetylmuramyl-tripeptide synthetases

OG_01067	LG3211_4504	LG3211_4504; outer membrane efflux family protein	OG_01067	LEC3_4558	outer membrane efflux protein
OG_01068	LG3211_28	LG3211_28; conserved hypothetical protein	OG_01068	LEC3_5295	prolyl 4-hydroxylase, alpha subunit
OG_01069	LG3211_3475	coaD; pantetheine-phosphate adenylyltransferase	OG_01069	LEC3_3532	pantetheine-phosphate adenylyltransferase
OG_01070	LG3211_2099	LG3211_2099; bacterial regulatory, tetR family protein	OG_01070	LEC3_2037	transcriptional regulator, TetR family
OG_01071	LG3211_466	LG3211_466; C-terminal processing peptidase family protein	OG_01071	LEC3_0275	C-terminal processing peptidase
OG_01072	LG3211_764	LG3211_764; polymer-forming cytoskeletal family protein	OG_01072	LEC3_0726	conserved hypothetical protein
OG_01073	LG3211_1306	LG3211_1306; 3-hydroxybutyrate dehydrogenase family protein	OG_01073	LEC3_1263	D-beta-hydroxybutyrate dehydrogenase
OG_01074	LG3211_2683	LG3211_2683; 5'-nucleotidase family protein	OG_01074	LEC3_2686	5'-nucleotidase
OG_01075	LG3211_972	LG3211_972; peptidoglycan synthetase penicillin-binding protein 1A	OG_01075	LEC3_0931	penicillin-binding protein 1A
OG_01076	LG3211_4256	LG3211_4256; glyoxalase-like domain protein	OG_01076	LEC3_4321	conserved hypothetical protein
OG_01077	LG3211_5146	LG3211_5146; RNA polymerase sigma factor, TIGR02999 family prot	OG_01077	LEC3_0141	RNA polymerase sigma factor, ECF family
OG_01078	LG3211_3967	LG3211_3967; phenazine biosynthesis , PhzF family protein	OG_01078	LEC3_3991	phenazine biosynthesis protein PhzF family
OG_01079	LG3211_3877	glnS; glutamine--tRNA ligase	OG_01079	LEC3_3895	glutaminyl-tRNA synthetase
OG_01080	LG3211_4506	czcA; cobalt-zinc-cadmium resistance protein czcA	OG_01080	LEC3_4560	cation efflux system protein czcA
OG_01081	LG3211_4312	LG3211_4312; hypothetical protein	OG_01081	LEC3_4373	conserved hypothetical protein
OG_01082	LG3211_737	LG3211_737; efflux transporter, RND family, MFP subunit	OG_01082	LEC3_0701	efflux transporter, RND family, MFP subunit
OG_01083	LG3211_2488	LG3211_2488; conserved hypothetical family protein	OG_01083	LEC3_2413	conserved hypothetical protein
OG_01084	LG3211_1730	LG3211_1730; hypothetical protein	OG_01084	LEC3_1686	chromosome segregation protein SEQUENCING GAP
OG_01085	LG3211_2105	LG3211_2105; conserved hypothetical protein	OG_01085	LEC3_2044	conserved hypothetical protein
OG_01086	LG3211_1399	LG3211_1399; hypothetical protein	OG_01086	LEC3_1365	hypothetical protein
OG_01087	LG3211_4505	LG3211_4505; efflux transporter, RND family, MFP subunit	OG_01087	LEC3_4559	efflux transporter, RND family, MFP subunit
OG_01088	LG3211_1624	LG3211_1624; arginine-tRNA-transferase, C terminus family protein	OG_01088	LEC3_1597	arginine-tRNA-protein transferase
OG_01089	LG3211_3415	LG3211_3415; metallo-beta-lactamase superfamily protein	OG_01089	LEC3_3485	metallo-beta-lactamase superfamily protein
OG_01090	LG3211_2032	LG3211_2032; hsc62, Hsp70 family chaperone, binds to RpoD and ir	OG_01090	LEC3_1966	dnaK family protein
OG_01091	LG3211_49	LG3211_49; NIPSNAP family protein	OG_01091	LEC3_5249	nipsnap family protein
OG_01092	LG3211_2881	LG3211_2881; tetrahydrofolate dehydrogenase/cyclohydrolase, catal	OG_01092	LEC3_2895	tetrahydrofolate dehydrogenase/cyclohydrolase, NAD(P)-binding and
OG_01093	LG3211_1381	zur; zur transcriptional repressor	OG_01093	LEC3_1349	transcriptional regulator, Fur family
OG_01094	LG3211_1764	LG3211_1764; phosphorylase superfamily protein	OG_01094	LEC3_1720	methylthioadenosine phosphorylase
OG_01095	LG3211_4142	riml; ribosomal-protein-alanine acetyltransferase	OG_01095	LEC3_4198	ribosomal-protein-alanine acetyltransferase
OG_01096	LG3211_2149	LG3211_2149; hypothetical protein	OG_01096	LEC3_2090	lipoprotein
OG_01097	LG3211_25	LG3211_25; citrate transporter family protein	OG_01097	LEC3_5311	TRAP transporter, DctM-like membrane protein
OG_01098	LG3211_1723	ycfF; purine nucleoside phosphoramidase	OG_01098	LEC3_1679	histidine triad nucleotide-binding protein 2 (hint-2)(hint-3)
OG_01099	LG3211_521	cc4; cytochrome c4 domain protein	OG_01099	LEC3_0334	cytochrome C4
OG_01100	LG3211_905	LG3211_905; tRNA_anti-like family protein	OG_01100	LEC3_0852	conserved hypothetical protein
OG_01101	LG3211_3844	LG3211_3844; hypothetical protein	OG_01101	LEC3_3867	conserved hypothetical protein
OG_01102	LG3211_4338	LG3211_4338; lipase family protein	OG_01102	LEC3_5184	conserved hypothetical protein
OG_01103	LG3211_4022	LG3211_4022; putative membrane protein	OG_01103	LEC3_4090	conserved hypothetical protein
OG_01104	LG3211_1023	LG3211_1023; lysE type translocator family protein	OG_01104	LEC3_0987	translocator protein, LysE family
OG_01105	LG3211_2156	LG3211_2156; glucose / Sorbosone dehydrogenase family protein	OG_01105	LEC3_2096	L-sorbosone dehydrogenase
OG_01106	LG3211_3254	lspA; signal peptidase II	OG_01106	LEC3_3285	signal peptidase II
OG_01107	LG3211_5051	LG3211_5051; glycosyl transferase 2 family protein	OG_01107	LEC3_4969	glycosyl transferase, group 2 family protein
OG_01108	LG3211_2211	LG3211_2211; proton-translocating NADH-quinone oxidoreductase, c	OG_01108	LEC3_2171	proton-translocating NADH-quinone oxidoreductase, chain M family p
OG_01109	LG3211_2248	LG3211_2248; hypothetical protein	OG_01109	LEC3_2200	hypothetical protein
OG_01110	LG3211_328	LG3211_328; putative ACETYL-COA ACYLTRANSFERASE FADA2	OG_01110	LEC3_5043	acetyl-CoA acetyltransferases
OG_01111	LG3211_4965	LG3211_4965; conserved hypothetical protein	OG_01111	LEC3_4877	conserved hypothetical protein
OG_01112	LG3211_4149	LG3211_4149; conserved hypothetical family protein	OG_01112	LEC3_4216	conserved hypothetical protein
OG_01113	LG3211_2650	iscA; iron-sulfur cluster assembly protein	OG_01113	LEC3_2654	iron-sulfur cluster assembly protein IscA
OG_01114	LG3211_4071	ftsZ; cell division protein FtsZ	OG_01114	LEC3_4126	cell division protein FtsZ
OG_01115	LG3211_907	LG3211_907; bacterial regulatory helix-turn-helix, AraC family protein	OG_01115	LEC3_0854	transcriptional regulator, AraC family
OG_01116	LG3211_3901	thyA; thymidylate synthase	OG_01116	LEC3_3920	thymidylate synthase
OG_01118	LG3211_3897	LG3211_3897; putative amidase domain protein	OG_01118	LEC3_3916	conserved hypothetical protein
OG_01119	LG3211_2703	LG3211_2703; FKBP-type peptidyl-prolyl cis-trans isomerase family p	OG_01119	LEC3_2704	domain amino terminal to FKBP-type peptidyl-prolyl isomerase/peptid
OG_01120	LG3211_2714	LG3211_2714; lyase family protein	OG_01120	LEC3_2715	fumarate hydratase, class II
OG_01121	LG3211_3459	LG3211_3459; histidine kinase-, DNA gyrase B-, and HSP90-like ATF	OG_01121	LEC3_3518	histidine kinase
OG_01122	LG3211_4431	lipB; lipoyl(octanoyl) transferase	OG_01122	LEC3_4476	lipoyltransferase
OG_01123	LG3211_2826	ccmF; cytochrome c-type biogenesis protein CcmF	OG_01123	LEC3_2831	cytochrome c-type biogenesis protein
OG_01124	LG3211_219	LG3211_219; type III secretion apparatus protein, YscD/HrpQ family	OG_01124	LEC3_5111	type III secretion apparatus protein
OG_01125	LG3211_2175	LG3211_2175; yhhN-like family protein	OG_01125	LEC3_2134	YhhN-like protein
OG_01126	LG3211_4257	LG3211_4257; metallo-beta-lactamase superfamily protein	OG_01126	LEC3_4326	metallo-beta-lactamase superfamily protein
OG_01127	LG3211_2651	LG3211_2651; conserved hypothetical protein	OG_01127	LEC3_2655	conserved hypothetical protein
OG_01128	LG3211_1676	LG3211_1676; periplasmic glucan biosynthesis , MdoG family protein	OG_01128	LEC3_1639	periplasmic glucan biosynthesis protein, MdoG
OG_01129	LG3211_2776	LG3211_2776; glucoamylase family protein	OG_01129	LEC3_2782	conserved hypothetical protein
OG_01130	LG3211_2900	LG3211_2900; acetyl-CoA C-acetyltransferase family protein	OG_01130	LEC3_2914	3-ketoacyl-CoA thiolase A
OG_01131	LG3211_1144	LG3211_1144; NHL repeat family protein	OG_01131	LEC3_1121	phytase precusor
OG_01132	LG3211_1599	minE; cell division topological specificity factor MinE	OG_01132	LEC3_1574	cell division topological specificity factor MinE
OG_01133	LG3211_3169	folK; 2-amino-4-hydroxy-6- hydroxymethyldihydropteridine diphospho	OG_01133	LEC3_3177	2-amino-4-hydroxy-6- hydroxymethyldihydropteridine pyrophosphokir
OG_01134	LG3211_245	LG3211_245; conserved hypothetical protein	OG_01134	LEC3_5085	3-demethylubiquinone-9 3-methyltransferase domain protein
OG_01135	LG3211_1758	LG3211_1758; conserved hypothetical protein	OG_01135	LEC3_1714	conserved hypothetical protein
OG_01136	LG3211_1449	LG3211_1449; conserved hypothetical protein	OG_01136	LEC3_1419	conserved hypothetical protein
OG_01137	LG3211_1321	LG3211_1321; hypothetical protein	OG_01137	LEC3_1275	lipoprotein
OG_01138	LG3211_4103	LG3211_4103; serine hydroxymethyltransferase family protein	OG_01138	LEC3_4161	serine hydroxymethyltransferase
OG_01139	LG3211_2178	LG3211_2178; SCO1/SenC family protein	OG_01139	LEC3_2138	SCO1/SenC family protein
OG_01140	LG3211_3297	rnr; ribonuclease R	OG_01140	LEC3_3338	ribonuclease R
OG_01141	LG3211_5222	LG3211_5222; multidrug resistance efflux transporter family protein	OG_01141	LEC3_0066	membrane protein
OG_01142	LG3211_3427	LG3211_3427; hypothetical protein	OG_01142	LEC3_3490	conserved hypothetical protein
OG_01143	LG3211_2171	LG3211_2171; nlpE N-terminal domain protein	OG_01143	LEC3_2127	conserved hypothetical protein
OG_01144	LG3211_4986	LG3211_4986; UDP-glucoronosyl and UDP-glucosyl transferase fami	OG_01144	LEC3_4895	UDP-glucoronosyl and UDP-glucosyl transferase
OG_01145	LG3211_3038	uvrC; excinuclease ABC subunit C	OG_01145	LEC3_3028	excinuclease ABC, C subunit
OG_01146	LG3211_584	LG3211_584; citrate transporter family protein	OG_01146	LEC3_4782	citrate transporter
OG_01147	LG3211_1210	LG3211_1210; alanine racemase, N-terminal domain protein	OG_01147	LEC3_1162	conserved hypothetical protein
OG_01148	LG3211_978	pilQ; type IV pilus secretin PilQ family protein	OG_01148	LEC3_0936	Type IV assembly protein

OG_01149	LG3211_939	LG3211_939; conserved hypothetical protein
OG_01150	LG3211_3081	LG3211_3081; ABC transporter family protein
OG_01151	LG3211_756	LG3211_756; phasin family protein
OG_01152	LG3211_4480	xerC; tyrosine recombinase XerC
OG_01153	LG3211_5056	LG3211_5056; putative 3-oxoacyl-[acyl-carrier protein] reductase
OG_01154	LG3211_1323	LG3211_1323; putative thioredoxin
OG_01155	LG3211_709	LG3211_709; outer membrane beta-barrel domain protein
OG_01156	LG3211_427	speE; spermidine synthase
OG_01157	LG3211_919	LG3211_919; tonB-dependent siderophore receptor family protein
OG_01158	LG3211_4369	LG3211_4369; bacterial regulatory helix-turn-helix , lysR family protei
OG_01159	LG3211_1424	LG3211_1424; conserved hypothetical protein
OG_01160	LG3211_2192	LG3211_2192; 2OG-Fe(II) oxygenase superfamily protein
OG_01161	LG3211_867	leuD; 3-isopropylmalate dehydratase, small subunit
OG_01162	LG3211_848	LG3211_848; mitochondrial fission ELM1 family protein
OG_01163	LG3211_1693	LG3211_1693; methyltransferase domain protein
OG_01164	LG3211_1548	LG3211_1548; ankyrin repeat family protein
OG_01165	LG3211_4507	LG3211_4507; cation diffusion facilitator transporter family protein
OG_01166	LG3211_3402	LG3211_3402; fecR family protein
OG_01167	LG3211_5226	bioF; 8-amino-7-oxononanoate synthase
OG_01168	LG3211_4011	LG3211_4011; conserved hypothetical protein
OG_01169	LG3211_2295	LG3211_2295; hypothetical protein
OG_01170	LG3211_3843	LG3211_3843; RNA polymerase sigma factor, sigma-70 family protei
OG_01171	LG3211_2199	LG3211_2199; NADH-ubiquinone/plastoquinone oxidoreductase, cha
OG_01172	LG3211_2840	LG3211_2840; hypothetical protein
OG_01173	LG3211_3324	LG3211_3324; cytidylyltransferase family protein
OG_01174	LG3211_3055	sdhC; succinate dehydrogenase, cytochrome b556 subunit
OG_01175	LG3211_1747	rpoE; RNA polymerase sigma factor RpoE
OG_01176	LG3211_66	phhA; phenylalanine-4-hydroxylase
OG_01177	LG3211_818	aroQ; 3-dehydroquinate dehydratase, type II
OG_01178	LG3211_695	rsuA; 16S rRNA pseudouridine 516 synthase
OG_01179	LG3211_3269	LG3211_3269; conserved hypothetical protein
OG_01180	LG3211_3896	apaH; bis
OG_01181	LG3211_4295	LG3211_4295; conserved hypothetical protein
OG_01182	LG3211_3454	secD; protein-export membrane protein SecD
OG_01183	LG3211_775	LG3211_775; major Facilitator Superfamily protein
OG_01184	LG3211_3986	tolA; protein TolA
OG_01185	LG3211_2221	rpsO; ribosomal protein S15
OG_01186	LG3211_268	LG3211_268; RNA polymerase sigma factor, sigma-70 family protein
OG_01187	LG3211_3760	rplB; ribosomal protein L2
OG_01188	LG3211_3810	LG3211_3810; asparaginase family protein
OG_01189	LG3211_2128	LG3211_2128; conserved hypothetical protein
OG_01190	LG3211_3233	LG3211_3233; cytochrome C assembly family protein
OG_01191	LG3211_4139	katB; catalase
OG_01192	LG3211_3001	LG3211_3001; restriction endonuclease family protein
OG_01193	LG3211_87	LG3211_87; mce related family protein
OG_01194	LG3211_425	LG3211_425; conserved hypothetical family protein
OG_01195	LG3211_2549	LG3211_2549; major Facilitator Superfamily protein
OG_01196	LG3211_2250	LG3211_2250; bacterial regulatory , Fis family protein
OG_01197	LG3211_985	LG3211_985; sodium:dicarboxylate symporter family protein
OG_01198	LG3211_2157	LG3211_2157; hypothetical protein
OG_01199	LG3211_2607	LG3211_2607; hypothetical protein
OG_01200	LG3211_1600	minD; septum site-determining protein MinD
OG_01201	LG3211_3604	LG3211_3604; stringent starvation B family protein
OG_01202	LG3211_121	LG3211_121; RNA polymerase sigma factor, sigma-70 family protein
OG_01203	LG3211_3715	LG3211_3715; sugar (and other) transporter family protein
OG_01204	LG3211_424	LG3211_424; bacterial regulatory helix-turn-helix , lysR family protein
OG_01205	LG3211_4641	LG3211_4641; heparinase II/III-like family protein
OG_01206	LG3211_4918	gluP; glucose/galactose transporter WARNING family protein
OG_01207	LG3211_5332	queF; queuine synthase
OG_01208	LG3211_4472	ubiE; 2-OCTAPRENYL-METHOXY-BENZOQ-METH bifunctional 2-oc
OG_01209	LG3211_2819	LG3211_2819; methionine aminotransferase, PLP-dependent
OG_01210	LG3211_5177	LG3211_5177; hypothetical protein
OG_01211	LG3211_2916	LG3211_2916; conserved hypothetical protein
OG_01212	LG3211_802	LG3211_802; putative phosphatase, inner membrane protein
OG_01214	LG3211_2883	LG3211_2883; conserved hypothetical protein
OG_01215	LG3211_2873	LG3211_2873; putative Cytosine/adenosine deaminase
OG_01216	LG3211_1495	hutG; N-formylglutamate deformylase
OG_01217	LG3211_1871	LG3211_1871; amidohydrolase family Protein OLEI01672_1_465
OG_01218	LG3211_348	LG3211_348; ABC transporter family protein
OG_01219	LG3211_3432	LG3211_3432; TPR repeat family protein
OG_01220	LG3211_3522	LG3211_3522; HNH endonuclease family protein
OG_01221	LG3211_3990	ruvB; holliday junction DNA helicase RuvB
OG_01222	LG3211_1485	LG3211_1485; FKBP-type peptidyl-prolyl cis-trans isomerase family p
OG_01223	LG3211_2408	LG3211_2408; short chain dehydrogenase family protein
OG_01224	LG3211_1581	LG3211_1581; conserved hypothetical protein
OG_01225	LG3211_2336	prfB; peptide chain release factor 2
OG_01226	LG3211_1864	gluQ; glutamyl-queuosine tRNA(Asp) synthetase
OG_01227	LG3211_4033	LG3211_4033; conserved hypothetical protein
OG_01228	LG3211_3612	LG3211_3612; acetyltransferase family protein
OG_01229	LG3211_3731	pqqD; coenzyme PQQ biosynthesis protein PqqD
OG_01230	LG3211_3931	LG3211_3931; conserved hypothetical protein

OG_01149	LEC3_0895	YecA family protein
OG_01150	LEC3_3073	ABC transporter, ATP-binding protein
OG_01151	LEC3_0719	conserved hypothetical protein
OG_01152	LEC3_4532	tyrosine recombinase XerC
OG_01153	LEC3_4972	phosphotransferase family protein
OG_01154	LEC3_1278	conserved hypothetical protein
OG_01155	LEC3_0679	conserved hypothetical protein
OG_01156	LEC3_0243	spermidine synthase
OG_01157	LEC3_0872	TonB-dependent siderophore receptor
OG_01158	LEC3_4413	transcriptional regulator, LysR family
OG_01159	LEC3_1390	excinuclease ABC, C subunit-like
OG_01160	LEC3_2153	oxidoreductase
OG_01161	LEC3_0817	3-isopropylmalate dehydratase, small subunit
OG_01162	LEC3_0794	conserved hypothetical protein
OG_01163	LEC3_1659	conserved hypothetical protein
OG_01164	LEC3_1520	ankyrin repeat protein
OG_01165	LEC3_4561	cation diffusion facilitator family transporter
OG_01166	LEC3_3457	peptidoglycan-binding LysM
OG_01167	LEC3_0062	8-amino-7-oxononanoate synthase
OG_01168	LEC3_4079	Protein of unknown function, DUF480
OG_01169	LEC3_2222	hypothetical protein
OG_01170	LEC3_3866	RNA polymerase sigma factor SigV
OG_01171	LEC3_2159	NADH-quinone oxidoreductase, A subunit
OG_01172	LEC3_2851	hypothetical protein
OG_01173	LEC3_3376	cytidylyltransferase family
OG_01174	LEC3_3046	succinate dehydrogenase, cytochrome b556 subunit
OG_01175	LEC3_1703	RNA polymerase sigma-E factor
OG_01176	LEC3_5254	phenylalanine-4-hydroxylase
OG_01177	LEC3_0769	3-dehydroquinate dehydratase, type II
OG_01178	LEC3_0661	ribosomal small subunit pseudouridine synthase A
OG_01179	LEC3_3301	conserved hypothetical protein
OG_01180	LEC3_3915	bis(5'-nucleosyl)-tetraphosphatase (symmetrical)
OG_01181	LEC3_4356	conserved hypothetical protein
OG_01182	LEC3_3513	protein-export membrane protein SecD
OG_01183	LEC3_0736	2-acylglycerophosphoethanolamine acyltransferase
OG_01184	LEC3_4054	protein TolA
OG_01185	LEC3_2179	ribosomal protein S15
OG_01186	LEC3_5059	RNA polymerase sigma factor, sigma-70 family
OG_01187	LEC3_3787	ribosomal protein L2
OG_01188	LEC3_3833	conserved hypothetical protein
OG_01189	LEC3_2072	conserved hypothetical protein
OG_01190	LEC3_3259	cytochrome C assembly protein
OG_01191	LEC3_3240	catalase
OG_01192	LEC3_2994	conserved hypothetical protein
OG_01193	LEC3_5235	toluene ABC transport system permease protein
OG_01194	LEC3_0241	inner membrane protein
OG_01195	LEC3_2533	transporter, major facilitator family
OG_01196	LEC3_2202	DNA-binding response regulator
OG_01197	LEC3_0944	transporter, dicarboxylate/amino acid:cation (Na+ or H+) symporter (D
OG_01198	LEC3_2097	lipoprotein
OG_01199	LEC3_3229	conserved hypothetical protein
OG_01200	LEC3_1575	septum site-determining protein MinD
OG_01201	LEC3_3596	stringent starvation protein B
OG_01202	LEC3_5203	RNA polymerase factor sigma-70 subfamily
OG_01203	LEC3_3739	transporter, major facilitator superfamily
OG_01204	LEC3_0240	transcriptional regulator, LysR family
OG_01205	LEC3_2247	heparinase II/III family protein
OG_01206	LEC3_0376	glucose/galactose transporter
OG_01207	LEC3_5396	7-cyano-7-deazaguanine reductase
OG_01208	LEC3_4522	ubiquinone/menaquinone biosynthesis methyltransferase UbiE
OG_01209	LEC3_2824	aminotransferase class I and II
OG_01210	LEC3_0112	conserved hypothetical protein
OG_01211	LEC3_2928	conserved hypothetical protein
OG_01212	LEC3_0753	dual specificity phosphatase, catalytic domain protein
OG_01214	LEC3_2897	conserved hypothetical protein
OG_01215	LEC3_2885	tRNA-specific adenosine deaminase
OG_01216	LEC3_1471	N-formylglutamate amidohydrolase
OG_01217	LEC3_1816	N-ethylammeline chlorohydrolase
OG_01218	LEC3_5025	ABC transporter, ATP-binding protein
OG_01219	LEC3_3496	tetratricopeptide repeat domain protein
OG_01220	LEC3_3554	HNH endonuclease domain protein
OG_01221	LEC3_4058	holliday junction DNA helicase
OG_01222	LEC3_1461	peptidyl-prolyl cis-trans isomerase, FKBP-type
OG_01223	LEC3_2350	oxidoreductase, short chain dehydrogenase/reductase family
OG_01224	LEC3_1558	conserved hypothetical protein
OG_01225	LEC3_2276	peptide chain release factor 2
OG_01226	LEC3_1810	glutamyl-Q tRNA(Asp) synthetase
OG_01227	LEC3_4101	conserved hypothetical protein
OG_01228	LEC3_3605	acetyltransferase (GNAT) family
OG_01229	LEC3_3758	coenzyme PQQ synthesis protein D
OG_01230	LEC3_3955	membrane protein

OG_01231	LG3211_2068	LG3211_2068; zinc carboxypeptidase family protein	OG_01231	LEC3_2011	Zn-dependent enzyme from deacylase/carboxypeptidase superfamily
OG_01232	LG3211_4524	LG3211_4524; conserved hypothetical protein	OG_01232	LEC3_4583	membrane/transport protein
OG_01233	LG3211_3347	LG3211_3347; modulator of DNA gyrase family protein	OG_01233	LEC3_3405	TldD/PmbA family protein
OG_01234	LG3211_3130	LG3211_3130; RNA pseudouridylate synthase family protein	OG_01234	LEC3_3133	RNA pseudouridine synthase A
OG_01235	LG3211_3756	rplP; ribosomal protein L16	OG_01235	LEC3_3783	ribosomal protein L16
OG_01236	LG3211_1059	LG3211_1059; peptidyl-Asp metalloendopeptidase domain protein	OG_01236	LEC3_1028	conserved hypothetical protein
OG_01237	LG3211_2206	LG3211_2206; NADH dehydrogenase family protein	OG_01237	LEC3_2166	NADH dehydrogenase, H subunit
OG_01238	LG3211_1847	LG3211_1847; trbL/VirB6 plasmid conjugal transfer family protein	OG_01238	LEC3_1543	TrbL/VirB6 plasmid conjugal transfer protein
OG_01239	LG3211_4705	LG3211_4705; conserved hypothetical protein	OG_01239	LEC3_0521	conserved hypothetical protein
OG_01240	LG3211_2164	LG3211_2164; conserved hypothetical protein	OG_01240	LEC3_2118	conserved hypothetical protein
OG_01241	LG3211_3777	secE; preprotein translocase, SecE subunit	OG_01241	LEC3_3803	preprotein translocase, SecE subunit
OG_01242	LG3211_5207	LG3211_5207; RNase_H superfamily protein	OG_01242	LEC3_0082	conserved hypothetical protein
OG_01243	LG3211_1100	LG3211_1100; DSBA-like thioredoxin domain protein	OG_01243	LEC3_1073	dsba oxidoreductase
OG_01244	LG3211_1790	LG3211_1790; hypothetical protein	OG_01244	LEC3_1750	conserved hypothetical protein
OG_01245	LG3211_4485	LG3211_4485; prolyl oligopeptidase family protein	OG_01245	LEC3_4537	prolyl oligopeptidase, N-terminal beta-propeller domain
OG_01246	LG3211_2752	zipA; cell division protein ZipA	OG_01246	LEC3_2757	cell division protein ZipA
OG_01247	LG3211_971	LG3211_971; conserved hypothetical protein	OG_01247	LEC3_0929	conserved hypothetical protein
OG_01248	LG3211_2325	cutC; copper homeostasis protein	OG_01248	LEC3_2265	copper homeostasis protein cutC
OG_01249	LG3211_4205	rpsI; 30S ribosomal subunit protein S9	OG_01249	LEC3_4259	ribosomal protein S9
OG_01250	LG3211_483	LG3211_483; cytochrome c oxidase subunit III family protein	OG_01250	LEC3_0290	cytochrome c oxidase, subunit III
OG_01251	LG3211_4001	LG3211_4001; concanavalin A-like lectin/glucanases superfamily prot	OG_01251	LEC3_4066	conserved hypothetical protein
OG_01252	LG3211_1386	LG3211_1386; merC mercury resistance family protein	OG_01252	LEC3_1354	conserved hypothetical protein
OG_01253	LG3211_3094	LG3211_3094; transcriptional regulatory , C terminal family protein	OG_01253	LEC3_3091	two component transcriptional regulator, winged helix family
OG_01254	LG3211_5216	LG3211_5216; FMN-dependent dehydrogenase family protein	OG_01254	LEC3_0072	glutamine amidotransferases class-II/glutamate synthase
OG_01255	LG3211_1180	aroB; 3-dehydroquinate synthase	OG_01255	LEC3_1150	3-dehydroquinate synthase
OG_01256	LG3211_117	LG3211_117; M61 glycyl aminopeptidase family protein	OG_01256	LEC3_5208	peptidase, M61 (glycyl aminopeptidase) family
OG_01257	LG3211_2616	LG3211_2616; hypothetical protein	OG_01257	LEC3_2627	conserved hypothetical protein
OG_01258	LG3211_948	LG3211_948; conserved hypothetical protein	OG_01258	LEC3_0902	conserved hypothetical protein
OG_01259	LG3211_4702	LG3211_4702; conserved hypothetical protein	OG_01259	LEC3_0524	conserved hypothetical protein
OG_01260	LG3211_771	LG3211_771; fecR family protein	OG_01260	LEC3_0732	sigma factor regulatory protein, FecR/PupR family
OG_01261	LG3211_24	LG3211_24; hypothetical protein	OG_01261	LEC3_5312	hypothetical protein
OG_01262	LG3211_854	LG3211_854; O-Antigen ligase family protein	OG_01262	LEC3_0800	O-Antigen Polymerase
OG_01263	LG3211_5357	LG3211_5357; magnesium-dependent DNase	OG_01263	LEC3_5364	type V secretory pathway protein
OG_01264	LG3211_798	purH; phosphoribosylaminoimidazolecarboxamide formyltransferase/I	OG_01264	LEC3_0749	bifunctional purine biosynthesis protein PurH
OG_01265	LG3211_4219	trpD; anthranilate phosphoribosyltransferase	OG_01265	LEC3_4272	anthranilate phosphoribosyltransferase
OG_01266	LG3211_4196	LG3211_4196; twitching motility family protein	OG_01266	LEC3_4255	Type IV pilus assembly protein
OG_01267	LG3211_4131	LG3211_4131; putative permease YjgP/YjgQ family protein	OG_01267	LEC3_4191	putative permease, YjgP/YjgQ family
OG_01268	LG3211_3651	LG3211_3651; H+ symporter) family protein	OG_01268	LEC3_3638	amino acid/peptide transporter (Peptide:H+ symporter)
OG_01269	LG3211_1641	aqpZ; aqpZ - water MIP channel	OG_01269	LEC3_1612	major intrinsic protein (MIP) family channel proteins
OG_01270	LG3211_2189	accD; acetyl-CoA carboxylase, carboxyl transferase, beta subunit	OG_01270	LEC3_2149	acetyl-CoA carboxylase, carboxyl transferase, beta subunit
OG_01271	LG3211_810	LG3211_810; bacterial regulatory , Fis family protein	OG_01271	LEC3_0760	DNA-binding protein Fis
OG_01272	LG3211_5161	LG3211_5161; glutamine amidotransferase class-I family protein	OG_01272	LEC3_0127	glutamine amidotransferase class-I
OG_01273	LG3211_2236	LG3211_2236; short chain dehydrogenase family protein	OG_01273	LEC3_2190	oxidoreductase, short chain dehydrogenase/reductase family
OG_01274	LG3211_3739	rpoA; DNA-directed RNA polymerase, alpha subunit	OG_01274	LEC3_3766	DNA-directed RNA polymerase, alpha subunit
OG_01275	LG3211_1737	fabG; 3-oxoacyl-[acyl-carrier-protein] reductase	OG_01275	LEC3_1692	3-oxoacyl-(acyl-carrier-protein) reductase
OG_01276	LG3211_386	LG3211_386; conserved hypothetical protein	OG_01276	LEC3_0648	conserved hypothetical protein
OG_01277	LG3211_3934	LG3211_3934; response regulator	OG_01277	LEC3_3958	Hpt domain/response regulator receiver domain/histidine kinase-, DN
OG_01278	LG3211_3084	fpr; ferredoxin--NADP+ reductase	OG_01278	LEC3_3078	oxidoreductase FAD/NAD(P) binding domain protein
OG_01279	LG3211_1622	LG3211_1622; endonuclease/Exonuclease/phosphatase family prote	OG_01279	LEC3_1595	endonuclease/exonuclease/phosphatase family
OG_01280	LG3211_1115	LG3211_1115; conserved hypothetical protein	OG_01280	LEC3_1090	conserved hypothetical protein
OG_01281	LG3211_5112	LG3211_5112; hypothetical protein	OG_01281	LEC3_0174	conserved hypothetical protein
OG_01282	LG3211_1289	LG3211_1289; conserved hypothetical protein	OG_01282	LEC3_1245	conserved hypothetical protein
OG_01283	LG3211_5107	LG3211_5107; 4'-phosphopantetheinyl transferase superfamily protei	OG_01283	LEC3_0179	4'-phosphopantetheinyl transferase Sfp
OG_01284	LG3211_690	LG3211_690; ABC transporter family protein	OG_01284	LEC3_0655	ABC transporter, ATP-binding protein
OG_01285	LG3211_4136	LG3211_4136; DNA polymerase III chi subunit, HolC family protein	OG_01285	LEC3_4196	DNA polymerase III subunit chi
OG_01286	LG3211_863	glpK; glycerol kinase	OG_01286	LEC3_0812	glycerol kinase
OG_01287	LG3211_1549	phlA; extracellular phospholipase A1	OG_01287	LEC3_1521	phospholipase A1
OG_01288	LG3211_1948	LG3211_1948; hisB	OG_01288	LEC3_1900	histidine biosynthesis bifunctional protein hisB
OG_01289	LG3211_365	LG3211_365; conserved hypothetical protein	OG_01289	LEC3_0182	conserved hypothetical protein
OG_01290	LG3211_4310	LG3211_4310; N-acetylmuramoyl-L-alanine amidase family protein	OG_01290	LEC3_4371	N-acetylmuramoyl-L-alanine amidase
OG_01291	LG3211_5004	ung; uracil-DNA glycosylase	OG_01291	LEC3_4912	uracil-DNA glycosylase
OG_01292	LG3211_1118	LG3211_1118; sugar (and other) transporter family protein	OG_01292	LEC3_1092	transporter, major facilitator family
OG_01293	LG3211_3165	pgi; glucose-6-phosphate isomerase	OG_01293	LEC3_3172	glucose-6-phosphate isomerase
OG_01294	LG3211_4134	LG3211_4134; putative cytosol aminopeptidase	OG_01294	LEC3_4194	cytosol aminopeptidase family
OG_01295	LG3211_2041	ldh; leucine dehydrogenase	OG_01295	LEC3_1976	Glu/Leu/Phe/Val dehydrogenase
OG_01296	LG3211_4299	LG3211_4299; EF hand family protein	OG_01296	LEC3_4359	calcium-binding EF-hand containing protein
OG_01297	LG3211_1224	pyrB; aspartate carbamoyltransferase	OG_01297	LEC3_1179	aspartate carbamoyltransferase
OG_01298	LG3211_1284	LG3211_1284; peptidase M20/M25/M40 family protein	OG_01298	LEC3_1240	peptidase family M20/M25/M40
OG_01299	LG3211_4099	LG3211_4099; methyltransferase domain protein	OG_01299	LEC3_4154	methyltransferase type 11
OG_01300	LG3211_3272	LG3211_3272; rhodanese-like domain protein	OG_01300	LEC3_3305	rhodanese-like domain
OG_01301	LG3211_2779	LG3211_2779; bolA-like family protein	OG_01301	LEC3_2786	morphogene BolA protein
OG_01302	LG3211_502	LG3211_502; conserved hypothetical protein	OG_01302	LEC3_0312	membrane protein
OG_01303	LG3211_1615	LG3211_1615; putative integral membrane domain protein	OG_01303	LEC3_1589	integral membrane protein
OG_01304	LG3211_2931	gntY; iron-sulfur cluster scaffold protein	OG_01304	LEC3_2944	iron-sulphur cluster biosynthesis/NifU-like domain
OG_01305	LG3211_4496	LG3211_4496; DMSO reductase anchor subunit family protein	OG_01305	LEC3_4548	DMSO reductase anchor subunit (dmsC)
OG_01306	LG3211_50	LG3211_50; HTH domain protein	OG_01306	LEC3_5250	bacterial regulatory protein, DeoR family
OG_01307	LG3211_3051	LG3211_3051; MAPEG family protein	OG_01307	LEC3_3042	conserved hypothetical protein
OG_01308	LG3211_2857	LG3211_2857; type I phosphodiesterase / nucleotide pyrophosphatas	OG_01308	LEC3_2869	CalU4
OG_01309	LG3211_4232	LG3211_4232; conserved hypothetical protein	OG_01309	LEC3_4293	conserved hypothetical protein
OG_01310	LG3211_2896	rlmN; 23S rRNA m2A2503 methyltransferase	OG_01310	LEC3_2910	radical SAM enzyme, Cfr family
OG_01311	LG3211_707	LG3211_707; conserved hypothetical protein	OG_01311	LEC3_0676	conserved hypothetical protein

OG_01312	LG3211_2811	LG3211_2811; trbC/VIRB2 family protein	OG_01312	LEC3_2817	VirB2 protein
OG_01313	LG3211_423	LG3211_423; nitrogen regulatory P-II family protein	OG_01313	LEC3_0238	nitrogen regulatory protein P-II
OG_01314	LG3211_3305	asnB; asparagine synthase	OG_01314	LEC3_3352	asparagine synthase
OG_01315	LG3211_1081	LG3211_1081; na+ dependent nucleoside transporter family protein	OG_01315	LEC3_1055	Na+ dependent nucleoside transporter
OG_01316	LG3211_3424	LG3211_3424; putative FMN-binding domain protein	OG_01316	LEC3_2659	transcriptional regulatory protein
OG_01317	LG3211_179	LG3211_179; zinc-binding dehydrogenase family protein	OG_01317	LEC3_1039	NADP-dependent oxidoreductase YncB
OG_01318	LG3211_187	LG3211_187; bacitracin resistance BacA family protein	OG_01318	LEC3_5144	undecaprenol kinase, putative
OG_01319	LG3211_1882	LG3211_1882; major Facilitator Superfamily protein	OG_01319	LEC3_1830	transporter, major facilitator family
OG_01320	LG3211_2759	LG3211_2759; tonB dependent receptor family protein	OG_01320	LEC3_2765	TonB-dependent receptor
OG_01321	LG3211_3988	tolQ; protein TolQ	OG_01321	LEC3_4056	protein TolQ
OG_01322	LG3211_1642	LG3211_1642; hypothetical protein	OG_01322	LEC3_1614	conserved hypothetical protein
OG_01323	LG3211_2457	LG3211_2457; cheR methyltransferase, SAM binding domain protein	OG_01323	LEC3_2451	cheR-type methyltransferase
OG_01324	LG3211_1146	metB; O-succinylhomoserine (thiol)-lyase	OG_01324	LEC3_1123	O-succinylhomoserine (thiol)-lyase
OG_01325	LG3211_4454	LG3211_4454; subtilase family protein	OG_01325	LEC3_1993	peptidase, families S8 and S53
OG_01326	LG3211_4438	dacC; penicillin-binding protein 6	OG_01326	LEC3_4481	penicillin-binding protein 6 precursor
OG_01327	LG3211_2844	LG3211_2844; pspC domain protein	OG_01327	LEC3_2855	conserved domain protein
OG_01328	LG3211_1417	LG3211_1417; conserved hypothetical protein	OG_01328	LEC3_1382	conserved hypothetical protein
OG_01329	LG3211_3806	yihY; yihY family inner membrane domain protein	OG_01329	LEC3_3830	tRNA-processing ribonuclease BN
OG_01330	LG3211_3142	LG3211_3142; conserved hypothetical protein	OG_01330	LEC3_3150	UPF0271 protein
OG_01331	LG3211_1421	LG3211_1421; S4 domain protein	OG_01331	LEC3_1387	conserved hypothetical protein
OG_01332	LG3211_3906	LG3211_3906; integral membrane TerC family protein	OG_01332	LEC3_3927	integral membrane protein, TerC family
OG_01333	LG3211_572	LG3211_572; conserved hypothetical protein	OG_01333	LEC3_4799	conserved hypothetical protein SEQUENCING GAP
OG_01334	LG3211_1920	pgaA; poly-beta-1,6 N-acetyl-D-glucosamine export porin PgaA	OG_01334	LEC3_1871	Biofilm PGA synthesis protein precursor
OG_01335	LG3211_1474	LG3211_1474; sodium:dicarboxylate symporter family protein	OG_01335	LEC3_1444	transporter, dicarboxylate/amino acid:cation (Na+ or H+) symporter (D
OG_01336	LG3211_4268	coaX; pantothenate kinase, type III family protein	OG_01336	LEC3_4335	transcriptional activator, putative, Baf family
OG_01337	LG3211_3939	gshB; glutathione synthase	OG_01337	LEC3_3964	glutathione synthase
OG_01338	LG3211_1253	LG3211_1253; hypothetical protein	OG_01338	LEC3_1210	hypothetical protein
OG_01339	LG3211_2030	LG3211_2030; dnaJ domain protein	OG_01339	LEC3_1964	dnaJ domain
OG_01340	LG3211_1883	LG3211_1883; fe-S metabolism associated domain protein	OG_01340	LEC3_1831	cysteine desulfuration protein SufE
OG_01341	LG3211_3960	LG3211_3960; ferritin-like domain protein	OG_01341	LEC3_3983	DNA protection during starvation protein
OG_01342	LG3211_1039	trmB; tRNA (guanine-N(7)-)-methyltransferase	OG_01342	LEC3_1005	tRNA (guanine-N(7)-)-methyltransferase
OG_01343	LG3211_3791	LG3211_3791; TPR repeat family protein	OG_01343	LEC3_3812	tetratricopeptide repeat family protein
OG_01344	LG3211_2335	LG3211_2335; glycosyl hydrolases family 2, sugar binding domain pr	OG_01344	LEC3_2273	glycosyl hydrolase, family 2
OG_01345	LG3211_5031	LG3211_5031; acetyltransferase family protein	OG_01345	LEC3_4942	acetyltransferase (GNAT) family
OG_01346	LG3211_2998	LG3211_2998; conserved hypothetical protein	OG_01346	LEC3_2990	conserved hypothetical protein
OG_01347	LG3211_2956	LG3211_2956; HAMP domain protein	OG_01347	LEC3_2973	signal transduction histidine kinase
OG_01348	LG3211_4238	LG3211_4238; putative exported lectin/glucanase	OG_01348	LEC3_4301	conserved hypothetical protein
OG_01349	LG3211_1462	LG3211_1462; conserved hypothetical protein	OG_01349	LEC3_1428	conserved hypothetical protein
OG_01350	LG3211_4503	LG3211_4503; hypothetical protein	OG_01350	LEC3_4557	hypothetical protein
OG_01351	LG3211_5078	LG3211_5078; delta-aminolevulinic acid dehydratase family protein	OG_01351	LEC3_4992	delta-aminolevulinic acid dehydratase
OG_01352	LG3211_1087	LG3211_1087; hemin uptake hemP family protein	OG_01352	LEC3_1061	conserved hypothetical protein
OG_01353	LG3211_4538	ohrR; transcriptional regulator OhrR	OG_01353	LEC3_4593	transcriptional regulator, MarR family
OG_01354	LG3211_193	amt; ammonium transporter family protein	OG_01354	LEC3_5138	ammonium transporter
OG_01355	LG3211_1070	metK; methionine adenosyltransferase	OG_01355	LEC3_1041	S-adenosylmethionine synthetase
OG_01356	LG3211_1476	LG3211_1476; NUDIX domain protein	OG_01356	LEC3_1446	mutT/nudix family protein
OG_01357	LG3211_3365	tig; trigger factor	OG_01357	LEC3_3421	trigger factor
OG_01358	LG3211_4233	LG3211_4233; hypothetical protein	OG_01358	LEC3_4296	hypothetical protein
OG_01359	LG3211_3478	LG3211_3478; hypothetical protein	OG_01359	LEC3_3535	hypothetical protein
OG_01360	LG3211_3319	LG3211_3319; penicillinase repressor family protein	OG_01360	LEC3_3371	methicillin resistance protein
OG_01361	LG3211_5110	LG3211_5110; putative chromosome-partitioning protein parB	OG_01361	LEC3_0176	ParB-like partition proteins
OG_01362	LG3211_1749	LG3211_1749; peptidase Do family protein	OG_01362	LEC3_1705	protease DeqQ precursor
OG_01363	LG3211_1499	LG3211_1499; hypothetical protein	OG_01363	LEC3_1473	hypothetical protein
OG_01364	LG3211_3876	LG3211_3876; conserved hypothetical protein	OG_01364	LEC3_3894	conserved hypothetical protein
OG_01365	LG3211_1136	LG3211_1136; channel , hemolysin III family protein	OG_01365	LEC3_1115	channel protein, hemolysin III family
OG_01366	LG3211_2165	LG3211_2165; luciferase oxidoreductase, group 1 family protein	OG_01366	LEC3_2119	luciferase-like monooxygenase family protein
OG_01367	LG3211_464	gpmI; 2,3-bisphosphoglycerate-independent phosphoglycerate mutas	OG_01367	LEC3_0272	2,3-bisphosphoglycerate-independent phosphoglycerate mutase
OG_01368	LG3211_2645	rpsF; ribosomal protein S6	OG_01368	LEC3_2650	ribosomal protein S6
OG_01369	LG3211_700	LG3211_700; conserved hypothetical protein	OG_01369	LEC3_0668	conserved hypothetical protein
OG_01370	LG3211_2882	LG3211_2882; conserved hypothetical protein	OG_01370	LEC3_2896	conserved hypothetical protein
OG_01371	LG3211_1535	hfq; RNA chaperone Hfq	OG_01371	LEC3_1510	RNA-binding protein
OG_01372	LG3211_344	LG3211_344; 2OG-Fe(II) oxygenase superfamily protein	OG_01372	LEC3_5028	conserved hypothetical protein
OG_01373	LG3211_4064	coaE; dephospho-CoA kinase	OG_01373	LEC3_4119	dephospho-CoA kinase
OG_01374	LG3211_2992	pheA; chorismate mutase	OG_01374	LEC3_2983	chorismate mutase/prephenate dehydratase/ACT domain
OG_01375	LG3211_1003	LG3211_1003; conserved hypothetical protein	OG_01375	LEC3_0968	lipoprotein
OG_01376	LG3211_3744	rplO; ribosomal protein L15	OG_01376	LEC3_3771	ribosomal protein L15
OG_01377	LG3211_1780	LG3211_1780; thiamine pyrophosphate enzyme, C-terminal TPP binc	OG_01377	LEC3_1743	thiamine pyrophosphate enzyme
OG_01378	LG3211_1239	lptA; lipopolysaccharide transport periplasmic protein LptA	OG_01378	LEC3_1194	cell envelope biogenesis protein YhbN
OG_01379	LG3211_4118	LG3211_4118; fimbrial assembly family protein	OG_01379	LEC3_4175	fimbrial assembly protein
OG_01380	LG3211_3398	LG3211_3398; conserved hypothetical integral membrane family prot	OG_01380	LEC3_3453	conserved hypothetical integral membrane protein
OG_01381	LG3211_3340	xseB; exodeoxyribonuclease VII, small subunit	OG_01381	LEC3_3396	exodeoxyribonuclease VII, small subunit
OG_01382	LG3211_583	phbB; acetoacetyl-CoA reductase family protein	OG_01382	LEC3_4783	acetoacetyl-CoA reductase
OG_01383	LG3211_5096	LG3211_5096; RDD family protein	OG_01383	LEC3_5004	RDD family
OG_01384	LG3211_86	LG3211_86; toluene tolerance, Ttg2 family protein	OG_01384	LEC3_5236	toluene tolerance protein Ttg2D
OG_01385	LG3211_2706	LG3211_2706; hypothetical protein	OG_01385	LEC3_2707	hypothetical protein
OG_01386	LG3211_3163	LG3211_3163; putative iron-sulfur cluster-binding protein	OG_01386	LEC3_3171	iron-sulfur cluster binding protein
OG_01387	LG3211_40	LG3211_40; fructose-1-6-bisphosphatase family protein	OG_01387	LEC3_5284	fructose-1,6-bisphosphatase
OG_01388	LG3211_2874	LG3211_2874; conserved hypothetical protein	OG_01388	LEC3_2886	conserved hypothetical protein
OG_01389	LG3211_1019	LG3211_1019; acetyltransferase family protein	OG_01389	LEC3_0982	acetyltransferase (GNAT) family
OG_01390	LG3211_4336	LG3211_4336; sir2 family protein	OG_01390	LEC3_4382	transcriptional regulator, Sir2 family
OG_01391	LG3211_520	LG3211_520; disulfide oxidoreductase	OG_01391	LEC3_0333	thiol:disulfide interchange protein
OG_01392	LG3211_4260	LG3211_4260; conserved hypothetical protein	OG_01392	LEC3_4330	lipoprotein

OG_01393	LG3211_5092	LG3211_5092; conserved hypothetical protein	OG_01393	LEC3_5000	conserved hypothetical protein
OG_01394	LG3211_595	bphK; glutathione S-transferase	OG_01394	LEC3_4772	glutathione S-transferase
OG_01395	LG3211_3915	LG3211_3915; conserved hypothetical protein	OG_01395	LEC3_3940	membrane protein
OG_01396	LG3211_1699	LG3211_1699; conserved hypothetical protein	OG_01396	LEC3_1663	conserved hypothetical protein
OG_01397	LG3211_4570	LG3211_4570; putative D-serine deaminase (D-serine dehydratase) p	OG_01397	LEC3_4617	D-amino acid deaminase
OG_01398	LG3211_3870	ahpD; alkyl hydroperoxide reductase AhpD	OG_01398	LEC3_3885	alkylhydroperoxidase, AhpD family
OG_01399	LG3211_2006	LG3211_2006; conserved hypothetical protein	OG_01399	LEC3_5336	conserved hypothetical protein
OG_01400	LG3211_4095	nusB; transcription antitermination factor NusB	OG_01400	LEC3_4150	transcription termination/antitermination protein NusB
OG_01401	LG3211_2816	LG3211_2816; conjugal transfer family protein	OG_01401	LEC3_2821	VirB9 protein
OG_01402	LG3211_554	LG3211_554; glutathione-dependent formaldehyde-activating enzyme	OG_01402	LEC3_4827	glutathione-dependent formaldehyde-activating
OG_01403	LG3211_311	LG3211_311; bacterial regulatory , arsR family protein	OG_01403	LEC3_3687	transcriptional regulator, ArsR-family
OG_01404	LG3211_236	LG3211_236; hypothetical protein	OG_01404	LEC3_5094	hypothetical protein
OG_01405	LG3211_3331	LG3211_3331; hypothetical protein	OG_01405	LEC3_3383	hypothetical protein
OG_01406	LG3211_730	LG3211_730; conserved hypothetical protein	OG_01406	LEC3_0696	conserved hypothetical protein
OG_01407	LG3211_1134	LG3211_1134; CBS domain	OG_01407	LEC3_1111	CBS domain protein
OG_01408	LG3211_532	oppB; oppB	OG_01408	LEC3_0344	ABC transporter, permease protein
OG_01409	LG3211_1355	LG3211_1355; conserved hypothetical protein	OG_01409	LEC3_1313	conserved hypothetical protein
OG_01410	LG3211_2780	LG3211_2780; YCII-related domain protein	OG_01410	LEC3_2787	YcII domain protein
OG_01411	LG3211_4348	LG3211_4348; glutathione S-transferase, C-terminal domain protein	OG_01411	LEC3_4390	glutathione S-transferase, C- domain
OG_01412	LG3211_3656	rfbA; glucose-1-phosphate thymidylyltransferase	OG_01412	LEC3_3642	glucose-1-phosphate thymidylyltransferase
OG_01413	LG3211_480	ctaD; cytochrome c oxidase, subunit I	OG_01413	LEC3_0287	cytochrome c oxidase, subunit I
OG_01414	LG3211_2159	pbpC; penicillin-binding protein 1C	OG_01414	LEC3_2114	penicillin-binding protein 1C
OG_01415	LG3211_645	LG3211_645; 'Cold-shock' DNA-binding domain protein	OG_01415	LEC3_4702	'Cold-shock' DNA-binding domain protein
OG_01416	LG3211_698	LG3211_698; conserved hypothetical protein	OG_01416	LEC3_0666	conserved hypothetical protein
OG_01417	LG3211_1274	cysG; cysG	OG_01417	LEC3_1230	uroporphyrin-III C-methyltransferase
OG_01418	LG3211_1307	LG3211_1307; patatin-like phospholipase family protein	OG_01418	LEC3_1264	patatin- like phospholipase
OG_01419	LG3211_4226	yegS; lipid kinase YegS	OG_01419	LEC3_4286	diacylglycerol kinase catalytic domain
OG_01420	LG3211_868	leuC; 3-isopropylmalate dehydratase, large subunit	OG_01420	LEC3_0818	3-isopropylmalate dehydratase, large subunit
OG_01421	LG3211_2950	LG3211_2950; ferric reductase like transmembrane component famil	OG_01421	LEC3_2969	ferric reductase like transmembrane component protein
OG_01422	LG3211_4520	LG3211_4520; lysE type translocator family protein	OG_01422	LEC3_4576	translocator protein, LysE family
OG_01423	LG3211_1889	argE; acetylornithine deacetylase	OG_01423	LEC3_1838	peptidase family M20/M25/M40
OG_01424	LG3211_3219	mutS; DNA mismatch repair protein MutS	OG_01424	LEC3_3238	DNA mismatch repair protein
OG_01425	LG3211_450	LG3211_450; conserved hypothetical protein	OG_01425	LEC3_0261	conserved hypothetical protein
OG_01426	LG3211_3350	maf; septum formation protein Maf	OG_01426	LEC3_3408	septum formation protein Maf
OG_01427	LG3211_4519	LG3211_4519; conserved hypothetical protein	OG_01427	LEC3_4575	conserved hypothetical protein
OG_01428	LG3211_183	LG3211_183; RDD family protein	OG_01428	LEC3_5148	RDD family
OG_01429	LG3211_4038	nadE; NAD+ synthetase	OG_01429	LEC3_4106	Glutamine-dependent NAD(+) synthetase
OG_01430	LG3211_3175	LG3211_3175; hypothetical protein	OG_01430	LEC3_3181	hypothetical protein
OG_01431	LG3211_3845	LG3211_3845; conserved hypothetical protein	OG_01431	LEC3_3868	conserved hypothetical protein
OG_01432	LG3211_642	LG3211_642; peptidase M23 family protein	OG_01432	LEC3_4705	M23 peptidase/bacterial SH3 domain protein
OG_01433	LG3211_2939	LG3211_2939; phosphoenolpyruvate phosphomutase family protein	OG_01433	LEC3_2953	conserved hypothetical protein
OG_01434	LG3211_2354	LG3211_2354; conserved hypothetical protein	OG_01434	LEC3_2300	conserved hypothetical protein
OG_01435	LG3211_1944	LG3211_1944; trp repressor family protein	OG_01435	LEC3_1896	trp operon repressor
OG_01436	LG3211_1506	kdsA; 3-deoxy-8-phosphooctulonate synthase	OG_01436	LEC3_1485	3-deoxy-8-phosphooctulonate synthase
OG_01437	LG3211_1875	LG3211_1875; type I GTP cyclohydrolase folE2 family protein	OG_01437	LEC3_1820	conserved hypothetical protein
OG_01438	LG3211_1350	LG3211_1350; bacterial regulatory helix-turn-helix , lysR family protei	OG_01438	LEC3_1305	transcriptional regulator, LysR-family
OG_01439	LG3211_3805	LG3211_3805; ahpC/TSA family protein	OG_01439	LEC3_3829	redoxin domain protein
OG_01440	LG3211_4481	LG3211_4481; conserved hypothetical protein	OG_01440	LEC3_4533	conserved hypothetical protein
OG_01441	LG3211_4041	sucD; succinyl-CoA synthetase, alpha subunit	OG_01441	LEC3_4109	succinyl-CoA synthetase, alpha subunit
OG_01442	LG3211_4511	LG3211_4511; xdhC and CoxI family protein	OG_01442	LEC3_4566	xanthine dehydrogenase accessory factor
OG_01443	LG3211_3336	LG3211_3336; DNA polymerase III, &alpha subunit	OG_01443	LEC3_3389	DNA polymerase III, alpha subunit
OG_01444	LG3211_3087	LG3211_3087; acyltransferase family protein	OG_01444	LEC3_3081	acyltransferase family
OG_01445	LG3211_2735	LG3211_2735; hypothetical protein	OG_01445	LEC3_2739	hypothetical protein
OG_01446	LG3211_2729	LG3211_2729; peroxiredoxin	OG_01446	LEC3_2732	Peroxiredoxin-5, mitochondrial
OG_01447	LG3211_4611	LG3211_4611; conserved hypothetical protein	OG_01447	LEC3_0652	conserved hypothetical protein
OG_01448	LG3211_3221	katG; catalase/peroxidase HPI	OG_01448	LEC3_3243	catalase/peroxidase HPI
OG_01449	LG3211_449	LG3211_449; putative beta-lactamase induction signal transducer Am	OG_01449	LEC3_0260	major facilitator superfamily MFS_1, putative
OG_01450	LG3211_1577	LG3211_1577; phosphatase 2C family protein	OG_01450	LEC3_1555	conserved hypothetical protein
OG_01451	LG3211_3341	ispA; geranyltranstransferase	OG_01451	LEC3_3397	polyprenyl synthetase
OG_01452	LG3211_1051	LG3211_1051; hypothetical protein	OG_01452	LEC3_1019	hypothetical protein
OG_01453	LG3211_2671	kynU; kynureninase	OG_01453	LEC3_2678	kynureninase
OG_01454	LG3211_3757	rpsC; ribosomal protein S3	OG_01454	LEC3_3784	ribosomal protein S3
OG_01455	LG3211_2417	LG3211_2417; mgtC family protein	OG_01455	LEC3_2414	putative Mg2+ transporter-C (MgtC) family
OG_01456	LG3211_2823	ccmC; heme exporter CcmC family protein	OG_01456	LEC3_2828	heme exporter protein CcmC
OG_01457	LG3211_3723	LG3211_3723; aminotransferase class I and II family protein	OG_01457	LEC3_3747	aminotransferase class I and II
OG_01458	LG3211_2778	LG3211_2778; periplasmic binding and sugar binding domain of LacI	OG_01458	LEC3_2785	transcriptional regulator, lacI family
OG_01459	LG3211_2909	LG3211_2909; TPR repeat family protein	OG_01459	LEC3_2922	tetratricopeptide repeat
OG_01460	LG3211_2736	LG3211_2736; alpha-2-macroglobulin N-terminal region family protei	OG_01460	LEC3_2740	alpha-2-macroglobulin family N- region
OG_01461	LG3211_1431	LG3211_1431; ATP-dependent RNA helicase, specific for 23S rRNA	OG_01461	LEC3_1400	dead/deah box helicase domain/helicase conserved domain/dbpa rna
OG_01462	LG3211_2720	LG3211_2720; conserved hypothetical protein	OG_01462	LEC3_2720	acetyltransferase (GNAT) family
OG_01463	LG3211_453	tyrS; tyrosine--tRNA ligase	OG_01463	LEC3_0265	tyrosyl-tRNA synthetase
OG_01464	LG3211_2255	LG3211_2255; marR family protein	OG_01464	LEC3_2208	transcriptional regulator, ArsR family
OG_01465	LG3211_496	dnaG; DNA primase	OG_01465	LEC3_0306	DNA primase
OG_01466	LG3211_542	fghA; S-formylglutathione hydrolase	OG_01466	LEC3_4840	S-formylglutathione hydrolase
OG_01467	LG3211_3120	ccmA; heme ABC exporter, ATP-binding protein CcmA	OG_01467	LEC3_3120	ABC transporter ATP-binding protein uup
OG_01468	LG3211_390	LG3211_390; RNA pseudouridylate synthase family protein	OG_01468	LEC3_0212	ribosomal large subunit pseudouridine synthase C
OG_01469	LG3211_4263	LG3211_4263; entericidin EcnA/B family protein	OG_01469	LEC3_4332	Entericidin EcnA/B family
OG_01470	LG3211_673	LG3211_673; conserved hypothetical protein	OG_01470	LEC3_4675	isochorismatase family protein
OG_01471	LG3211_442	dut; dut	OG_01471	LEC3_0253	dUTP diphosphatase
OG_01472	LG3211_5074	LG3211_5074; sulfate ABC transporter, sulfate-binding family protein	OG_01472	LEC3_4988	sulfate ABC transporter, sulfate-binding protein
OG_01473	LG3211_911	LG3211_911; conserved hypothetical protein	OG_01473	LEC3_0864	SNARE associated Golgi protein/PAP2 superfamily protein

OG_01474	LG3211_5209	LG3211_5209; peptidase inhibitor I78 family protein	OG_01474	LEC3_0080	hypothetical protein
OG_01475	LG3211_5234	ubiA; 4-hydroxybenzoate polyprenyl transferase	OG_01475	LEC3_0054	4-hydroxybenzoate polyprenyl transferase
OG_01476	LG3211_1390	LG3211_1390; membrane dipeptidase family protein	OG_01476	LEC3_1358	renal dipeptidase
OG_01477	LG3211_1867	LG3211_1867; kamA family protein	OG_01477	LEC3_1813	lysine 2,3-aminomutase YodO family protein
OG_01478	LG3211_3043	LG3211_3043; biopolymer transport ExbD/TolR family protein	OG_01478	LEC3_3034	transport energizing protein, ExbD/TolR family
OG_01479	LG3211_5095	LG3211_5095; RDD family protein	OG_01479	LEC3_5003	conserved hypothetical protein
OG_01480	LG3211_3540	infC; translation initiation factor IF-3	OG_01480	LEC3_3569	translation initiation factor IF-3
OG_01481	LG3211_1814	LG3211_1814; bacterial regulatory helix-turn-helix, AraC family protei	OG_01481	LEC3_0514	transcriptional regulator, AraC family
OG_01482	LG3211_3312	glnD; protein-P-II uridylyltransferase	OG_01482	LEC3_3359	protein-P-II uridylyltransferase
OG_01483	LG3211_447	LG3211_447; acetyltransferase family protein	OG_01483	LEC3_0258	acetyltransferase (GNAT) family
OG_01484	LG3211_3890	msrB; methionine-R-sulfoxide reductase	OG_01484	LEC3_3909	methionine-R-sulfoxide reductase
OG_01485	LG3211_2864	LG3211_2864; kinase/pyrophosphorylase family protein	OG_01485	LEC3_2876	UPF0085 protein
OG_01486	LG3211_658	acrA; acrA Membrane Fusion Protein	OG_01486	LEC3_4690	acriflavine resistance protein A
OG_01487	LG3211_1586	LG3211_1586; transcriptional regulatory , C terminal family protein	OG_01487	LEC3_1563	transcriptional regulatory protein
OG_01488	LG3211_2257	LG3211_2257; NAD dependent epimerase/dehydratase family protein	OG_01488	LEC3_2210	NAD dependent epimerase/dehydratase family
OG_01489	LG3211_349	LG3211_349; ABC-2 transporter family protein	OG_01489	LEC3_5024	membrane protein
OG_01490	LG3211_2355	trmU; tRNA (5-methylaminomethyl-2-thiouridylate)-methyltransferase	OG_01490	LEC3_2301	tRNA (5-methylaminomethyl-2-thiouridylate)-methyltransferase
OG_01491	LG3211_5319	LG3211_5319; aminoglycoside/hydroxyurea antibiotic resistance kina	OG_01491	LEC3_5415	streptomycin 3''-kinase (Streptomycin 3''-phosphotransferase) (SPH)
OG_01492	LG3211_607	LG3211_607; hypothetical protein	OG_01492	LEC3_4760	hypothetical protein
OG_01493	LG3211_1108	alkJ; alcohol dehydrogenase [acceptor]	OG_01493	LEC3_1081	alcohol dehydrogenase [acceptor]
OG_01494	LG3211_2036	LG3211_2036; acetyl-CoA C-acetyltransferase family protein	OG_01494	LEC3_1971	acetyl-CoA acetyltransferase
OG_01495	LG3211_448	xth; exodeoxyribonuclease III	OG_01495	LEC3_0259	exodeoxyribonuclease III
OG_01496	LG3211_501	folB; dihydroneopterin aldolase	OG_01496	LEC3_0311	dihydroneopterin aldolase
OG_01497	LG3211_4068	LG3211_4068; conserved hypothetical protein	OG_01497	LEC3_4124	conserved hypothetical protein
OG_01498	LG3211_2377	LG3211_2377; crcB-like family protein	OG_01498	LEC3_2625	CrcB protein
OG_01499	LG3211_3457	queA; tRNA ribosyltransferase-isomerase	OG_01499	LEC3_3516	S-adenosylmethionine:tRNA ribosyltransferase-isomerase
OG_01500	LG3211_5097	LG3211_5097; bacterial transferase hexapeptide family protein	OG_01500	LEC3_5005	transferase
OG_01501	LG3211_436	LG3211_436; sporulation related domain protein	OG_01501	LEC3_0248	sporulation and cell division repeat protein
OG_01502	LG3211_2710	LG3211_2710; conserved hypothetical protein	OG_01502	LEC3_2711	conserved hypothetical protein
OG_01503	LG3211_1710	LG3211_1710; moaE family protein	OG_01503	LEC3_1672	molybdopterin converting factor, subunit 2
OG_01504	LG3211_452	LG3211_452; peptidase M23 family protein	OG_01504	LEC3_0263	M23 peptidase domain protein
OG_01505	LG3211_1517	LG3211_1517; putative transmembrane protein	OG_01505	LEC3_1498	lipoprotein
OG_01506	LG3211_2608	LG3211_2608; GDSL-like Lipase/Acylhydrolase family protein	OG_01506	LEC3_3230	conserved hypothetical protein
OG_01507	LG3211_1089	LG3211_1089; tonB-dependent hemoglobin/transferrin/lactoferrin rec	OG_01507	LEC3_1062	TonB-dependent outer membrane receptor
OG_01508	LG3211_482	LG3211_482; cytochrome c oxidase assembly CtaG/Cox11 family pro	OG_01508	LEC3_0289	cytochrome C oxidase assembly protein, CtaG / Cox11
OG_01509	LG3211_996	LG3211_996; S1/P1 Nuclease family protein	OG_01509	LEC3_0960	endonuclease S1
OG_01510	LG3211_1686	LG3211_1686; conserved hypothetical protein	OG_01510	LEC3_1653	gamma-carboxymuconolactone decarboxylase
OG_01511	LG3211_654	LG3211_654; DSBA-like thioredoxin domain protein	OG_01511	LEC3_4695	DsbA oxidoreductase
OG_01512	LG3211_3519	LG3211_3519; conserved hypothetical protein	OG_01512	LEC3_3551	conserved hypothetical protein
OG_01513	LG3211_5054	LG3211_5054; short chain dehydrogenase family protein	OG_01513	LEC3_4971	short chain dehydrogenase/NAD dependent epimerase/dehydratase f
OG_01514	LG3211_2395	potI; potI	OG_01514	LEC3_2340	ABC transporter, permease protein
OG_01515	LG3211_5011	rho; transcription termination factor Rho	OG_01515	LEC3_4918	transcription termination factor Rho
OG_01516	LG3211_4080	LG3211_4080; UDP	OG_01516	LEC3_4134	UDP-N-acetylmuramoyl-tripeptide--D-alanyl-D- alanine ligase
OG_01517	LG3211_1645	LG3211_1645; histidine phosphatase super family protein	OG_01517	LEC3_1617	phosphoglycerate mutase family protein
OG_01518	LG3211_1332	LG3211_1332; suppressor of fused family protein	OG_01518	LEC3_1287	BtrU-like protein
OG_01519	LG3211_2142	LG3211_2142; phage Tail Collar domain protein	OG_01519	LEC3_2083	phage Tail Collar
OG_01521	LG3211_3957	LG3211_3957; putative secreted protein	OG_01521	LEC3_3979	conserved hypothetical protein
OG_01522	LG3211_5204	LG3211_5204; conserved hypothetical protein	OG_01522	LEC3_0085	conserved hypothetical protein
OG_01523	LG3211_4208	LG3211_4208; rubrerythrin family protein	OG_01523	LEC3_4263	ubiquinone biosynthesis protein
OG_01524	LG3211_5178	LG3211_5178; hypothetical protein	OG_01524	LEC3_0111	hypothetical protein
OG_01525	LG3211_3260	rpmA; ribosomal protein L27	OG_01525	LEC3_3292	ribosomal protein L27
OG_01526	LG3211_3339	LG3211_3339; conserved hypothetical protein	OG_01526	LEC3_3394	tRNA(Ile)-lysidine synthetase
OG_01527	LG3211_2908	LG3211_2908; glycosyl transferase 4 family protein	OG_01527	LEC3_2921	glycosyl transferase, group 4 family protein
OG_01528	LG3211_3726	typA; GTP-binding protein TypA/BipA	OG_01528	LEC3_3752	GTP-binding protein TypA/BipA
OG_01529	LG3211_3097	LG3211_3097; glycerophosphoryl diester phosphodiesterase family p	OG_01529	LEC3_3097	glycerophosphoryl diester phosphodiesterase family
OG_01530	LG3211_5354	LG3211_5354; hypothetical protein	OG_01530	LEC3_5366	conserved hypothetical protein
OG_01531	LG3211_3892	LG3211_3892; surA N-terminal domain protein	OG_01531	LEC3_3911	surA N-terminal domain/PPIC-type PPIASE domain protein
OG_01532	LG3211_4402	LG3211_4402; ribonucleoside-diphosphate reductase, adenosylcobal	OG_01532	LEC3_4449	ribonucleoside-diphosphate reductase, adenosylcobalamin-dependen
OG_01533	LG3211_5145	mutM; formamidopyrimidine-DNA glycosylase	OG_01533	LEC3_0142	formamidopyrimidine-DNA glycosylase
OG_01534	LG3211_3013	LG3211_3013; nucleotide sugar dehydrogenase family protein	OG_01534	LEC3_3005	nucleotide sugar dehydrogenase family protein
OG_01535	LG3211_3640	LG3211_3640; conserved hypothetical protein	OG_01535	LEC3_3629	magnesium and cobalt efflux protein CorC
OG_01536	LG3211_5322	LG3211_5322; major Facilitator Superfamily protein	OG_01536	LEC3_5412	transporter, major facilitator family
OG_01537	LG3211_1703	LG3211_1703; glycosyltransferase 9 family protein	OG_01537	LEC3_1665	heptosyltransferase
OG_01538	LG3211_1052	LG3211_1052; ATP-dependant zinc protease family protein	OG_01538	LEC3_1021	ribosomal protein S6 modification protein
OG_01539	LG3211_715	atpH; ATP synthase F1, delta subunit	OG_01539	LEC3_0686	ATP synthase F1, delta subunit
OG_01540	LG3211_667	LG3211_667; SPFH domain / Band 7 family protein	OG_01540	LEC3_4679	band 7 protein family
OG_01541	LG3211_3932	LG3211_3932; cheW-like domain protein	OG_01541	LEC3_3956	conserved hypothetical protein
OG_01542	LG3211_1576	LG3211_1576; von Willebrand factor type A domain protein	OG_01542	LEC3_1554	von Willebrand factor, type A
OG_01543	LG3211_3345	LG3211_3345; modulator of DNA gyrase family protein	OG_01543	LEC3_3402	PmbA protein
OG_01544	LG3211_4566	nagA; alpha-N-acetylgalactosaminidase	OG_01544	LEC3_4614	oxidoreductase family, NAD-binding Rossmann fold
OG_01545	LG3211_1940	hisS; histidine--tRNA ligase	OG_01545	LEC3_1891	histidyl-tRNA synthetase
OG_01546	LG3211_1629	LG3211_1629; RNA polymerase sigma factor, sigma-70 family protein	OG_01546	LEC3_1599	RNA polymerase sigma factor, sigma-70 family
OG_01547	LG3211_1285	LG3211_1285; competence ComEA helix-hairpin-helix repeat region	OG_01547	LEC3_1241	competence protein ComEA helix-hairpin-helix repeat region
OG_01548	LG3211_555	LG3211_555; bacterial regulatory, gntR family protein	OG_01548	LEC3_4824	aminotransferase, classes I and II superfamily
OG_01549	LG3211_4932	LG3211_4932; CDP-Glycerol:Poly(glycerophosphate) glycerophosph	OG_01549	LEC3_0362	CDP-Glycerol:Poly(glycerophosphate) glycerophosphotransferase
OG_01550	LG3211_1373	LG3211_1373; sporulation related domain protein	OG_01550	LEC3_1337	sporulation and cell division repeat protein
OG_01551	LG3211_107	LG3211_107; hlyD secretion family protein	OG_01551	LEC3_5217	HlyD secretion family protein
OG_01552	LG3211_1534	miaA; tRNA dimethylallyltransferase	OG_01552	LEC3_1509	tRNA delta(2)-isopentenylpyrophosphate transferase
OG_01553	LG3211_1120	LG3211_1120; conserved hypothetical protein	OG_01553	LEC3_1095	conserved hypothetical protein
OG_01554	LG3211_696	LG3211_696; HAD hydrolase, IA, variant 3 family protein	OG_01554	LEC3_0662	haloacid dehalogenase-like hydrolase
OG_01555	LG3211_930	LG3211_930; impB/mucB/samB family protein	OG_01555	LEC3_0880	DNA polymerase IV

OG_01556	LG3211_375	LG3211_375; beta-lactamase family protein	OG_01556	LEC3_0193	beta-lactamase
OG_01557	LG3211_1774	LG3211_1774; conserved hypothetical protein	OG_01557	LEC3_1736	conserved hypothetical protein
OG_01558	LG3211_1238	yhbG; lptB	OG_01558	LEC3_1193	ABC transporter, ATP-binding protein
OG_01559	LG3211_2907	LG3211_2907; short chain dehydrogenase family protein	OG_01559	LEC3_2920	epimerase/dehydratase
OG_01560	LG3211_2023	LG3211_2023; enoyl-CoA hydratase/isomerase family protein	OG_01560	LEC3_1954	enoyl-CoA hydratase
OG_01561	LG3211_3096	SIRT4; NAD-dependent ADP-ribosyltransferase sirtuin-4	OG_01561	LEC3_3095	transcriptional regulator, Sir2 family
OG_01562	LG3211_4945	LG3211_4945; chitin binding domain protein	OG_01562	LEC3_4851	chitin binding domain
OG_01563	LG3211_2899	LG3211_2899; enoyl-CoA hydratase/isomerase family protein	OG_01563	LEC3_2913	3-hydroxyacyl-CoA dehydrogenase
OG_01564	LG3211_2885	LG3211_2885; prolyl oligopeptidase family protein	OG_01564	LEC3_2898	hydrolase, alpha/beta fold family protein
OG_01565	LG3211_5076	cysW; sulfate ABC transporter, permease protein CysW	OG_01565	LEC3_4990	sulfate ABC transporter, permease protein cysW
OG_01566	LG3211_1469	LG3211_1469; conserved hypothetical protein	OG_01566	LEC3_1437	membrane protein
OG_01567	LG3211_5315	LG3211_5315; AFG1-like ATPase family protein	OG_01567	LEC3_5421	ATPase, AFG1 family
OG_01568	LG3211_2863	LG3211_2863; conserved hypothetical protein	OG_01568	LEC3_2875	conserved hypothetical protein
OG_01569	LG3211_3911	LG3211_3911; efflux transporter, RND family, MFP subunit	OG_01569	LEC3_3933	efflux transporter, RND family, MFP subunit
OG_01570	LG3211_3407	LG3211_3407; DEAD-box-containing ATP-dependent RNA helicase fa	OG_01570	LEC3_3468	DEAD/DEAH box helicase/helicase conserved C-terminal domain
OG_01571	LG3211_2670	Acmsd; 2-amino-3-carboxymuconate-6-semialdehyde decarboxylase	OG_01571	LEC3_2676	2-amino-3-carboxymuconate 6-semialdehyde decarboxylase
OG_01572	LG3211_4342	LG3211_4342; fatty acid hydroxylase superfamily protein	OG_01572	LEC3_4385	fatty acid hydroxylase superfamily
OG_01573	LG3211_3286	phoU; phosphate transport system regulatory protein PhoU	OG_01573	LEC3_3318	phosphate transport system regulatory protein PhoU
OG_01574	LG3211_5052	LG3211_5052; conserved hypothetical protein	OG_01574	LEC3_4970	lipoprotein
OG_01575	LG3211_4988	LG3211_4988; sulfatase family protein	OG_01575	LEC3_4899	sulfatase
OG_01576	LG3211_1513	LG3211_1513; smr domain protein	OG_01576	LEC3_1494	Smr domain protein
OG_01577	LG3211_2821	ccmA; heme ABC exporter, ATP-binding protein CcmA	OG_01577	LEC3_2826	heme ABC exporter, ATP-binding protein CcmA
OG_01578	LG3211_1288	LG3211_1288; apbE family protein	OG_01578	LEC3_1244	ApbE family lipoprotein
OG_01579	LG3211_1281	cysD; sulfate adenylyltransferase, small subunit	OG_01579	LEC3_1237	sulfate adenylyltransferase, small subunit
OG_01580	LG3211_5154	LG3211_5154; conserved hypothetical protein	OG_01580	LEC3_0133	conserved hypothetical protein
OG_01581	LG3211_1143	LG3211_1143; conserved hypothetical protein	OG_01581	LEC3_1120	peptidase
OG_01582	LG3211_1924	LG3211_1924; hypothetical protein	OG_01582	LEC3_1874	major facilitator superfamily MFS_1, putative
OG_01583	LG3211_4971	LG3211_4971; bacterial regulatory, gntR family protein	OG_01583	LEC3_4882	transcriptional regulator, GntR family
OG_01584	LG3211_4549	LG3211_4549; conserved hypothetical protein	OG_01584	LEC3_4604	conserved hypothetical protein
OG_01585	LG3211_1481	LG3211_1481; thioesterase superfamily protein	OG_01585	LEC3_1451	acyl-CoA hydrolase
OG_01586	LG3211_2478	bmrA; multidrug resistance ABC transporter ATP-binding/permease p	OG_01586	LEC3_3673	ABC transporter, ATP-binding protein
OG_01587	LG3211_679	LG3211_679; hypothetical protein	OG_01587	LEC3_4670	conserved hypothetical protein
OG_01588	LG3211_912	LG3211_912; ATP-dependent protease La domain protein	OG_01588	LEC3_0863	ATP-dependent protease La (LON) domain
OG_01589	LG3211_360	LG3211_360; putative methyltransferase	OG_01589	LEC3_5016	RNA methyltransferase, TrmH family, group 2
OG_01590	LG3211_3767	rpsG; ribosomal protein S7	OG_01590	LEC3_3794	ribosomal protein S7
OG_01591	LG3211_2869	LG3211_2869; conserved hypothetical protein	OG_01591	LEC3_2880	conserved hypothetical protein
OG_01592	LG3211_3803	LG3211_3803; conserved hypothetical protein	OG_01592	LEC3_3826	conserved hypothetical protein
OG_01593	LG3211_2829	LG3211_2829; TPR repeat family protein	OG_01593	LEC3_2834	tetratricopeptide repeat
OG_01594	LG3211_1125	LG3211_1125; hypothetical protein	OG_01594	LEC3_1101	conserved hypothetical protein
OG_01595	LG3211_95	LG3211_95; hypothetical protein	OG_01595	LEC3_5228	hypothetical protein
OG_01596	LG3211_5118	rpmG; ribosomal protein L33	OG_01596	LEC3_0168	ribosomal protein L33
OG_01597	LG3211_2215	rimP; ribosome maturation domain protein	OG_01597	LEC3_2174	UPF0090 protein, YhbC family
OG_01598	LG3211_876	ilvD; dihydroxy-acid dehydratase	OG_01598	LEC3_0825	dehydratase family
OG_01600	LG3211_1881	dksA; RNA polymerase-binding protein DksA	OG_01600	LEC3_1827	RNA polymerase-binding protein DksA
OG_01601	LG3211_153	LG3211_153; trbL/VirB6 plasmid conjugal transfer family protein	OG_01601	LEC3_2059	traH protein
OG_01602	LG3211_763	LG3211_763; putative transmembrane protein	OG_01602	LEC3_0725	conserved hypothetical protein
OG_01603	LG3211_3304	LG3211_3304; polysaccharide deacetylase family protein	OG_01603	LEC3_3348	polysaccharide deacetylase family protein
OG_01604	LG3211_4070	lpxC; UDP-3-O-[3-hydroxymyristoyl] N-acetylglucosamine deacetylas	OG_01604	LEC3_4125	UDP-3-O-[3-hydroxymyristoyl] N-acetylglucosamine deacetylase
OG_01605	LG3211_1026	LG3211_1026; efflux transporter, RND family, MFP subunit	OG_01605	LEC3_0990	RND efflux membrane fusion protein
OG_01606	LG3211_4556	LG3211_4556; FAD binding domain protein	OG_01606	LEC3_4611	FAD dependent oxidoreductase
OG_01607	LG3211_4206	rplM; ribosomal protein L13	OG_01607	LEC3_4260	ribosomal protein L13
OG_01608	LG3211_1503	LG3211_1503; conserved hypothetical protein	OG_01608	LEC3_1481	conserved hypothetical protein
OG_01609	LG3211_1754	era; GTP-binding protein Era	OG_01609	LEC3_1711	GTP-binding protein Era
OG_01610	LG3211_1158	LG3211_1158; cytochrome c family protein	OG_01610	LEC3_1130	gluconate 2-dehydrogenase cytochrome c subunit
OG_01611	LG3211_1294	bnzC; benzene 1,2-dioxygenase system ferredoxin subunit	OG_01611	LEC3_1254	benzene 1,2-dioxygenase, ferredoxin protein
OG_01612	LG3211_1111	LG3211_1111; calcineurin-like phosphoesterase family protein	OG_01612	LEC3_1084	phosphoesterase
OG_01613	LG3211_4439	rlpA; rare lipoA family protein	OG_01613	LEC3_4482	rare lipoprotein A
OG_01614	LG3211_1705	LG3211_1705; metallo-beta-lactamase superfamily protein	OG_01614	LEC3_1667	metallo-beta-lactamase domain protein
OG_01615	LG3211_2085	LG3211_2085; hypothetical protein	OG_01615	LEC3_2025	hypothetical protein
OG_01616	LG3211_3236	LG3211_3236; efflux transporter, outer membrane factor (OMF) lipo,	OG_01616	LEC3_3263	metal ion efflux RND protein family
OG_01617	LG3211_2007	LG3211_2007; conserved hypothetical protein	OG_01617	LEC3_3966	hypothetical protein
OG_01618	LG3211_430	speA; arginine decarboxylase	OG_01618	LEC3_0244	biosynthetic arginine decarboxylase
OG_01619	LG3211_2797	LG3211_2797; hypothetical protein	OG_01619	LEC3_2804	hypothetical protein
OG_01620	LG3211_1343	LG3211_1343; recF/RecN/SMC N terminal domain protein	OG_01620	LEC3_1296	RecF/RecN/SMC N terminal domain
OG_01621	LG3211_3802	LG3211_3802; hypothetical protein	OG_01621	LEC3_3825	conserved hypothetical protein
OG_01622	LG3211_1252	LG3211_1252; TPR repeat family protein	OG_01622	LEC3_1209	tetratricopeptide repeat domain protein
OG_01623	LG3211_3981	LG3211_3981; radical SAM superfamily protein	OG_01623	LEC3_4049	radical SAM domain protein
OG_01624	LG3211_1743	holB; DNA polymerase III, delta' subunit	OG_01624	LEC3_1698	DNA polymerase III subunit delta prime
OG_01625	LG3211_4575	LG3211_4575; hypothetical protein	OG_01625	LEC3_4621	hypothetical protein
OG_01626	LG3211_5369	LG3211_5369; DNA binding , excisionase family domain protein	OG_01626	LEC3_5349	DNA binding domain, excisionase family
OG_01627	LG3211_3470	LG3211_3470; metallo-beta-lactamase superfamily protein	OG_01627	LEC3_3527	metallo-beta-lactamase superfamily protein
OG_01628	LG3211_1042	LG3211_1042; conserved hypothetical protein	OG_01628	LEC3_1009	conserved hypothetical protein
OG_01629	LG3211_3600	purE; phosphoribosylaminoimidazole carboxylase,catalytic subunit	OG_01629	LEC3_3592	phosphoribosylaminoimidazole carboxylase, catalytic subunit
OG_01630	LG3211_1530	rrmJ; 23S rRNA 2'-O-ribose U2552 methyltransferase	OG_01630	LEC3_1506	ribosomal RNA large subunit methyltransferase J
OG_01631	LG3211_3056	LG3211_3056; glycine cleavage T-C-terminal barrel domain protein	OG_01631	LEC3_3048	aminomethyltransferase
OG_01632	LG3211_481	LG3211_481; hypothetical protein	OG_01632	LEC3_0288	hypothetical protein
OG_01633	LG3211_1359	kbl; 2-amino-3-ketobutyrate coenzyme A ligase	OG_01633	LEC3_1318	2-amino-3-ketobutyrate coenzyme A ligase
OG_01634	LG3211_1110	LG3211_1110; TLC ATP/ADP transporter family protein	OG_01634	LEC3_1083	conserved hypothetical protein
OG_01635	LG3211_137	LG3211_137; periplasmic binding and sugar binding domain of LacI fa	OG_01635	LEC3_5188	transcriptional regulator, LacI family
OG_01636	LG3211_499	rpsU; ribosomal protein S21	OG_01636	LEC3_0309	ribosomal protein S21
OG_01637	LG3211_1759	LG3211_1759; conserved hypothetical protein	OG_01637	LEC3_1715	lipoprotein

OG_01638	LG3211_2420	LG3211_2420; T5orf172 domain protein	OG_01638	LEC3_2364	conserved hypothetical protein
OG_01639	LG3211_1675	LG3211_1675; glycosyl transferases group 1 family protein	OG_01639	LEC3_1637	glycosyl transferase, group 1 family protein
OG_01640	LG3211_2376	LG3211_2376; sigma-54 interaction domain protein	OG_01640	LEC3_2323	ATPase, AAA family protein
OG_01641	LG3211_1028	LG3211_1028; MMPL family protein	OG_01641	LEC3_0992	RND superfamily protein
OG_01642	LG3211_1071	LG3211_1071; acyltransferase family protein	OG_01642	LEC3_1043	acyltransferase
OG_01643	LG3211_1147	LG3211_1147; homoserine dehydrogenase family protein	OG_01643	LEC3_1124	homoserine dehydrogenase
OG_01644	LG3211_4147	LG3211_4147; H-NS histone family protein	OG_01644	LEC3_4204	DNA-binding protein H-NS
OG_01645	LG3211_1022	fbab; fructose-bisphosphate aldolase	OG_01645	LEC3_0986	fructose-bisphosphate aldolase
OG_01646	LG3211_2993	serC; phosphoserine transaminase	OG_01646	LEC3_2984	phosphoserine aminotransferase
OG_01647	LG3211_3326	rseP; RIP metalloprotease RseP	OG_01647	LEC3_3378	RIP metalloprotease RseP
OG_01648	LG3211_821	LG3211_821; thioredoxin family protein	OG_01648	LEC3_0771	thioredoxin/cytochrome C biogenesis protein
OG_01649	LG3211_2653	LG3211_2653; putative transmembrane protein	OG_01649	LEC3_2657	conserved hypothetical protein
OG_01650	LG3211_1277	cysH; phosphoadenosine phosphosulfate reductase	OG_01650	LEC3_1233	phosphoadenosine phosphosulfate reductase
OG_01651	LG3211_1728	LG3211_1728; dolichyl-phosphate-mannose-mannosyltransferase far	OG_01651	LEC3_1684	membrane protein
OG_01652	LG3211_130	LG3211_130; bacterial regulatory, gntR family protein	OG_01652	LEC3_5195	transcriptional regulator, GntR family
OG_01653	LG3211_4271	LG3211_4271; EDD, DegV family domain protein	OG_01653	LEC3_4338	DegV family protein
OG_01654	LG3211_3886	LG3211_3886; ubiquinone biosynthesis hydroxylase, UbiH/UbiF/VisC	OG_01654	LEC3_3904	2-polyprenyl-6-methoxyphenol 4-hydroxylase
OG_01655	LG3211_693	LG3211_693; conserved hypothetical protein	OG_01655	LEC3_0659	membrane protein
OG_01656	LG3211_1722	recR; recombination protein RecR	OG_01656	LEC3_1678	recombination protein
OG_01657	LG3211_4562	LG3211_4562; tonB dependent receptor family protein	OG_01657	LEC3_3653	TonB-dependent receptor
OG_01658	LG3211_2577	fimV; fimV N-terminal domain	OG_01658	LEC3_4589	outer membrane autotransporter barrel domain protein
OG_01659	LG3211_158	ggt; gamma-glutamyltransferase	OG_01659	LEC3_5169	gamma-glutamyltransferase
OG_01660	LG3211_4090	LG3211_4090; conserved hypothetical protein	OG_01660	LEC3_4144	conserved hypothetical protein
OG_01661	LG3211_356	LG3211_356; major Facilitator Superfamily protein	OG_01661	LEC3_5017	transporter, major facilitator family
OG_01662	LG3211_703	LG3211_703; ompW family protein	OG_01662	LEC3_0671	outer membrane protein, OmpW family
OG_01663	LG3211_2134	LG3211_2134; zinc-binding dehydrogenase family protein	OG_01663	LEC3_2077	alcohol dehydrogenase, zinc-binding
OG_01664	LG3211_1375	purF; amidophosphoribosyltransferase	OG_01664	LEC3_1339	amidophosphoribosyltransferase
OG_01665	LG3211_4004	aspS; aspartate--tRNA ligase	OG_01665	LEC3_4069	aspartyl-tRNA synthetase
OG_01666	LG3211_3093	LG3211_3093; peptidase M3 family protein	OG_01666	LEC3_3090	peptidyl-dipeptidase dcp
OG_01667	LG3211_1978	LG3211_1978; conserved hypothetical protein	OG_01667	LEC3_1930	conserved hypothetical protein
OG_01668	LG3211_261	LG3211_261; hypothetical protein	OG_01668	LEC3_5069	hypothetical protein
OG_01669	LG3211_3177	dapA; dihydrodipicolinate synthase	OG_01669	LEC3_3183	dihydrodipicolinate synthase
OG_01670	LG3211_2296	LG3211_2296; sensory box protein	OG_01670	LEC3_2223	sensory box histidine kinase
OG_01671	LG3211_4106	ccmA; heme ABC exporter, ATP-binding protein CcmA	OG_01671	LEC3_4163	ABC transporter, ATP-binding protein
OG_01672	LG3211_1298	sufD; feS assembly protein SufD	OG_01672	LEC3_1257	FeS assembly protein SufD
OG_01673	LG3211_1312	LG3211_1312; PIN domain protein	OG_01673	LEC3_1272	conserved hypothetical protein
OG_01674	LG3211_1824	LG3211_1824; heavy metal sensor kinase family protein	OG_01674	LEC3_1781	putative heavy metal histidine sensor kinase
OG_01675	LG3211_1879	pyrC; dihydroorotase	OG_01675	LEC3_1824	dihydroorotase, multifunctional complex type
OG_01676	LG3211_2948	LG3211_2948; conserved hypothetical protein	OG_01676	LEC3_2967	AP endonuclease, family 2
OG_01677	LG3211_4234	LG3211_4234; eamA-like transporter family protein	OG_01677	LEC3_4297	integral membrane protein DUF6
OG_01678	LG3211_3841	pntB; NAD(P) transhydrogenase subunit beta	OG_01678	LEC3_3864	NAD(P) transhydrogenase beta subunit
OG_01679	LG3211_3281	pstS; phosphate ABC transporter, phosphate-binding protein PstS	OG_01679	LEC3_3314	phosphate ABC transporter, phosphate-binding protein PstS
OG_01680	LG3211_431	LG3211_431; YCII-related domain protein	OG_01680	LEC3_0245	conserved hypothetical protein
OG_01681	LG3211_991	LG3211_991; ABC transporter family protein	OG_01681	LEC3_0953	ABC transporter, ATP-binding protein
OG_01682	LG3211_3837	LG3211_3837; bacterial regulatory helix-turn-helix, AraC family protei	OG_01682	LEC3_3860	transcriptional regulator, AraC family
OG_01683	LG3211_962	LG3211_962; planctomycete cytochrome C family protein	OG_01683	LEC3_0918	lipoprotein
OG_01684	LG3211_1563	LG3211_1563; histidine kinase-, DNA gyrase B-, and HSP90-like ATF	OG_01684	LEC3_1540	histidine kinase
OG_01685	LG3211_5205	LG3211_5205; hsp70 family protein	OG_01685	LEC3_0084	conserved hypothetical protein
OG_01686	LG3211_1073	LG3211_1073; aspartate racemase family protein	OG_01686	LEC3_1045	aspartate racemase
OG_01687	LG3211_1607	LG3211_1607; hhH-GPD superbase excision DNA repair family prote	OG_01687	LEC3_1582	DNA-3-methyladenine glycosylase
OG_01688	LG3211_3282	pstC; phosphate ABC transporter, permease protein PstC	OG_01688	LEC3_3315	phosphate ABC transporter, permease protein PstC
OG_01689	LG3211_5170	hemH; ferrochelatase	OG_01689	LEC3_0119	ferrochelatase
OG_01690	LG3211_3039	LG3211_3039; low molecular weight phosphotyrosine phosphatase fa	OG_01690	LEC3_3030	Low molecular weight protein-tyrosine-phosphatase
OG_01691	LG3211_1888	argG; argininosuccinate synthase	OG_01691	LEC3_1837	argininosuccinate synthase
OG_01692	LG3211_3786	LG3211_3786; ribosomal protein L25, Ctc-form	OG_01692	LEC3_3808	ribosomal protein L25
OG_01693	LG3211_4466	LG3211_4466; glycosyl hydrolases 25 family protein	OG_01693	LEC3_4515	glycosyl hydrolases family 25
OG_01694	LG3211_3909	LG3211_3909; ftsX-like permease family protein	OG_01694	LEC3_3931	efflux ABC transporter, permease protein
OG_01695	LG3211_2875	LG3211_2875; bacterial regulatory, tetR family protein	OG_01695	LEC3_2887	transcriptional regulator, TetR family
OG_01696	LG3211_2338	rpfG; response regulator	OG_01696	LEC3_2281	response regulator
OG_01697	LG3211_4943	LG3211_4943; RDD family protein	OG_01697	LEC3_0350	RDD family
OG_01698	LG3211_2865	ppsA; phosphoenolpyruvate synthase	OG_01698	LEC3_2877	phosphoenolpyruvate synthase
OG_01699	LG3211_2617	copA; copper resistance protein A	OG_01699	LEC3_2628	copper resistance protein A
OG_01700	LG3211_1336	LG3211_1336; conserved hypothetical protein	OG_01700	LEC3_1290	conserved hypothetical protein
OG_01701	LG3211_380	LG3211_380; sodium symporter family protein	OG_01701	LEC3_0199	transporter, solute:sodium symporter (SSS) family
OG_01702	LG3211_3895	apaG; protein ApaG	OG_01702	LEC3_3914	protein ApaG
OG_01703	LG3211_1060	metF; 5,10-methylenetetrahydrofolate reductase	OG_01703	LEC3_1029	5,10-methylenetetrahydrofolate reductase
OG_01704	LG3211_1794	murB; UDP-N-acetylenolpyruvoylglucosamine reductase	OG_01704	LEC3_1757	UDP-N-acetylenolpyruvoylglucosamine reductase
OG_01705	LG3211_4224	trpE; anthranilate synthase component I	OG_01705	LEC3_4285	anthranilate synthase component I
OG_01706	LG3211_2435	cyoA; ubiquinol oxidase, subunit II	OG_01706	LEC3_2377	ubiquinol oxidase, subunit II
OG_01707	LG3211_4535	LG3211_4535; pepSY-associated TM helix family protein	OG_01707	LEC3_4587	propeptide, PepSY amd peptidase M4
OG_01708	LG3211_5073	LG3211_5073; phosphate-selective porin O and P family protein	OG_01708	LEC3_4987	phosphate-selective porin O and P
OG_01709	LG3211_909	adk; adenylate kinase	OG_01709	LEC3_0857	adenylate kinase
OG_01710	LG3211_2170	greB; transcription elongation factor GreB	OG_01710	LEC3_2126	transcription elongation factor GreB
OG_01711	LG3211_1632	tesB; acyl-CoA thioesterase II	OG_01711	LEC3_1601	acyl-CoA thioesterase II
OG_01712	LG3211_1672	LG3211_1672; acetyltransferase family protein	OG_01712	LEC3_1634	conserved hypothetical protein
OG_01713	LG3211_1973	LG3211_1973; short chain dehydrogenase family protein	OG_01713	LEC3_1928	3-hydroxyacyl-coa dehydrogenase type II
OG_01714	LG3211_3871	ahpC; alkyl hydroperoxide reductase subunit C	OG_01714	LEC3_3886	peroxiredoxin
OG_01715	LG3211_2904	LG3211_2904; pyridine nucleotide-disulfide oxidoreductase family prc	OG_01715	LEC3_2917	FAD dependent oxidoreductase
OG_01716	LG3211_2858	LG3211_2858; radical SAM superfamily protein	OG_01716	LEC3_2870	coproporphyrinogen III oxidase family protein
OG_01717	LG3211_3787	prs; ribose-phosphate pyrophosphokinase	OG_01717	LEC3_3809	ribose-phosphate pyrophosphokinase
OG_01718	LG3211_3646	LG3211_3646; conserved hypothetical protein	OG_01718	LEC3_3633	conserved domain protein

OG_01719	LG3211_3074	LG3211_3074; phospholipase A1 family protein	OG_01719	LEC3_3065	outer membrane phospholipase A
OG_01720	LG3211_3048	LG3211_3048; conserved hypothetical protein	OG_01720	LEC3_3040	conserved hypothetical protein
OG_01721	LG3211_1671	LG3211_1671; inner membrane CreD family protein	OG_01721	LEC3_1632	inner membrane protein CreD
OG_01722	LG3211_689	LG3211_689; GDSL-like Lipase/Acylhydrolase family protein	OG_01722	LEC3_0654	Lysophospholipase
OG_01723	LG3211_94	LG3211_94; bacterial regulatory helix-turn-helix , lysR family protein	OG_01723	LEC3_5229	transcriptional regulator, LysR family
OG_01724	LG3211_3794	prfA; peptide chain release factor 1	OG_01724	LEC3_3815	peptide chain release factor 1
OG_01725	LG3211_2135	soxR; redox-sensitive transcriptional activator SoxR	OG_01725	LEC3_2078	redox-sensitive transcriptional activator SoxR
OG_01726	LG3211_3004	LG3211_3004; bacterial regulatory, tetR family protein	OG_01726	LEC3_2997	transcriptional regulator, TetR family
OG_01727	LG3211_1505	pyrG; CTP synthase	OG_01727	LEC3_1483	CTP synthase
OG_01728	LG3211_1310	LG3211_1310; eamA-like transporter family protein	OG_01728	LEC3_1270	inner membrane protein YtfF
OG_01729	LG3211_3483	LG3211_3483; acetyltransferase family protein	OG_01729	LEC3_3539	acetyltransferase (GNAT) family
OG_01730	LG3211_3068	LG3211_3068; glycine zipper 2TM domain protein	OG_01730	LEC3_3061	rickettsia 17 kDa surface antigen family
OG_01731	LG3211_611	LG3211_611; conserved hypothetical protein	OG_01731	LEC3_4741	nucleoprotein/polynucleotide-associated enzyme
OG_01732	LG3211_1009	LG3211_1009; ribonucleoside-diphosphate reductase, alpha subunit	OG_01732	LEC3_0973	ribonucleoside-diphosphate reductase, alpha subunit
OG_01733	LG3211_4269	LG3211_4269; biotin--[acetyl-CoA-carboxylase] ligase	OG_01733	LEC3_4336	biotin-[acetyl-CoA-carboxylase] ligase
OG_01734	LG3211_872	LG3211_872; branched-chain amino acid aminotransferase	OG_01734	LEC3_0821	branched-chain amino acid aminotransferase
OG_01735	LG3211_1235	LG3211_1235; phosphoenolpyruvate-dependent sugar phosphotrans	OG_01735	LEC3_1190	phosphoenolpyruvate-dependent sugar phosphotransferase system,
OG_01736	LG3211_1500	parE; DNA topoisomerase IV, B subunit	OG_01736	LEC3_1475	DNA topoisomerase IV, B subunit
OG_01737	LG3211_2920	LG3211_2920; acetyltransferase family protein	OG_01737	LEC3_2932	acetyltransferase (GNAT) family
OG_01738	LG3211_2191	LG3211_2191; type I phosphodiesterase / nucleotide pyrophosphatas	OG_01738	LEC3_2152	sulfatase
OG_01739	LG3211_3065	LG3211_3065; tatD related DNase family protein	OG_01739	LEC3_3058	TatD related DNase
OG_01740	LG3211_4956	LG3211_4956; conserved hypothetical protein	OG_01740	LEC3_4862	conserved hypothetical protein
OG_01741	LG3211_1140	LG3211_1140; hypothetical protein	OG_01741	LEC3_1117	hypothetical protein
OG_01742	LG3211_3189	LG3211_3189; response regulator	OG_01742	LEC3_3197	KDP operon transcriptional regulatory protein KdpE
OG_01743	LG3211_1418	NAMPT; nicotinamide phosphoribosyltransferase	OG_01743	LEC3_1383	Nicotinate phosphoribosyltransferase (NAPRTase) family
OG_01744	LG3211_2251	dnaE; DNA polymerase III, alpha subunit	OG_01744	LEC3_2204	DNA polymerase III, alpha subunit
OG_01745	LG3211_3280	pstS; phosphate ABC transporter, phosphate-binding protein PstS	OG_01745	LEC3_3313	phosphate ABC transporter, phosphate-binding protein PstS
OG_01746	LG3211_269	LG3211_269; fecR family protein	OG_01746	LEC3_5058	sigma factor regulatory protein, FecR/PupR family
OG_01747	LG3211_1410	LG3211_1410; recF/RecN/SMC N terminal domain protein	OG_01747	LEC3_1375	ATP-binding protein
OG_01748	LG3211_887	LG3211_887; transcriptional regulatory , C terminal family protein	OG_01748	LEC3_0837	Transcriptional regulatory proteins, C terminal - transcriptional regulat
OG_01749	LG3211_1769	LG3211_1769; hypothetical protein	OG_01749	LEC3_1727	hypothetical protein
OG_01750	LG3211_864	LG3211_864; major intrinsic family protein	OG_01750	LEC3_0813	transporter, major intrinsic protein (MIP) family
OG_01751	LG3211_568	LG3211_568; AMP nucleosidase, putative	OG_01751	LEC3_4802	AMP nucleosidase
OG_01752	LG3211_4371	LG3211_4371; H+ antiporter-2 family protein	OG_01752	LEC3_4414	drug resistance MFS transporter, drug:H+ antiporter-1 (14 Spanner) (
OG_01753	LG3211_834	groL; chaperonin GroL	OG_01753	LEC3_0784	60 kDa chaperonin
OG_01754	LG3211_4465	LG3211_4465; pfkB carbohydrate kinase family protein	OG_01754	LEC3_4514	carbohydrate kinase, PfkB family
OG_01755	LG3211_1893	proS; proline--tRNA ligase	OG_01755	LEC3_1842	prolyl-tRNA synthetase
OG_01756	LG3211_189	glnA; glutamine synthetase, type I	OG_01756	LEC3_5142	glutamine synthetase, type I
OG_01757	LG3211_3007	LG3211_3007; conserved hypothetical protein	OG_01757	LEC3_3001	tetratricopeptide repeat family protein
OG_01758	LG3211_3011	LG3211_3011; conserved region in glutamate synthase family protein	OG_01758	LEC3_3003	conserved region in glutamate synthase
OG_01759	LG3211_440	coaBC; phosphopantothenoylcysteine decarboxylase / phosphopantc	OG_01759	LEC3_0252	DNA/pantothenate metabolism flavoprotein
OG_01760	LG3211_128	nagA; N-acetylglucosamine-6-phosphate deacetylase	OG_01760	LEC3_5197	N-acetylglucosamine-6-phosphate deacetylase
OG_01761	LG3211_3252	LG3211_3252; sodium/hydrogen exchanger family protein	OG_01761	LEC3_4072	transporter, monovalent cation:proton antiporter-2 (CPA2) family
OG_01762	LG3211_12	LG3211_12; biopolymer transport ExbD/TolR family protein	OG_01762	LEC3_5327	transport energizing protein, ExbD/TolR family
OG_01763	LG3211_1293	LG3211_1293; tonB-dependent siderophore receptor family protein	OG_01763	LEC3_1249	TonB-dependent siderophore receptor
OG_01764	LG3211_898	LG3211_898; F5/8 type C domain protein	OG_01764	LEC3_0844	endo-beta-N-acetylglucosaminidase
OG_01765	LG3211_2910	LG3211_2910; conserved hypothetical protein	OG_01765	LEC3_2923	hypothetical protein
OG_01766	LG3211_586	LG3211_586; response regulator	OG_01766	LEC3_4780	Transcriptional regulatory protein, C terminal - DNA-binding response
OG_01767	LG3211_3758	rplV; ribosomal protein L22	OG_01767	LEC3_3785	ribosomal protein L22
OG_01768	LG3211_5365	LG3211_5365; alpha/beta hydrolase fold family protein	OG_01768	LEC3_5355	hydrolase, alpha/beta fold family/TAP-like protein
OG_01769	LG3211_4477	LG3211_4477; NAD dependent epimerase/dehydratase family protein	OG_01769	LEC3_4529	NADH dehydrogenase
OG_01770	LG3211_3059	LG3211_3059; putative glutathione S-transferase	OG_01770	LEC3_3053	glutathione S-transferase
OG_01771	LG3211_3997	LG3211_3997; alpha/beta hydrolase fold family protein	OG_01771	LEC3_4064	conserved hypothetical protein
OG_01772	LG3211_4716	LG3211_4716; metallo-beta-lactamase superfamily protein	OG_01772	LEC3_5301	metallo-beta-lactamase domain protein
OG_01773	LG3211_3716	dctA; dctA dicarboxylate transporter	OG_01773	LEC3_3740	aerobic C4-dicarboxylate transport protein
OG_01774	LG3211_486	LG3211_486; cytochrome oxidase assembly family protein	OG_01774	LEC3_0294	cytochrome oxidase assembly protein
OG_01775	LG3211_533	LG3211_533; VIT family protein	OG_01775	LEC3_0345	integral membrane protein
OG_01776	LG3211_201	LG3211_201; periplasmic binding and sugar binding domain of LacI f	OG_01776	LEC3_5131	transcriptional regulator, lacI family
OG_01777	LG3211_5338	LG3211_5338; flagellin N-methylase family protein	OG_01777	LEC3_5391	ferredoxin
OG_01778	LG3211_5360	LG3211_5360; conserved hypothetical protein	OG_01778	LEC3_5360	lipoprotein
OG_01779	LG3211_2692	LG3211_2692; homocysteine S-methyltransferase family protein	OG_01779	LEC3_2694	methionine synthase
OG_01780	LG3211_936	LG3211_936; conserved hypothetical protein	OG_01780	LEC3_0886	conserved hypothetical protein
OG_01781	LG3211_4221	LG3211_4221; HAMP domain protein	OG_01781	LEC3_4281	sensor histidine kinase
OG_01782	LG3211_1102	gcvH; glycine cleavage system H protein	OG_01782	LEC3_1074	glycine cleavage system H protein
OG_01783	LG3211_3124	LG3211_3124; glutathione S-transferase, C-terminal domain protein	OG_01783	LEC3_3125	glutathione S-transferase III
OG_01784	LG3211_3346	LG3211_3346; conserved hypothetical protein	OG_01784	LEC3_3403	UPF0307 protein
OG_01785	LG3211_1605	LG3211_1605; polyketide cyclase / dehydrase and lipid transport fam	OG_01785	LEC3_1580	conserved hypothetical protein
OG_01786	LG3211_468	LG3211_468; rhomboid family protein	OG_01786	LEC3_0278	membrane protein, rhomboid family
OG_01787	LG3211_2929	LG3211_2929; cytochrome c554 domain protein	OG_01787	LEC3_2940	cytochrome C family protein
OG_01788	LG3211_874	ilvB; acetolactate synthase, large subunit, biosynthetic type	OG_01788	LEC3_0823	acetolactate synthase, large subunit, biosynthetic type
OG_01789	LG3211_2076	phaZ7; PHB depolymerase PhaZ7	OG_01789	LEC3_2019	lipase family
OG_01790	LG3211_1859	LG3211_1859; formylglycine-generating sulfatase enzyme family prot	OG_01790	LEC3_1803	lipoprotein
OG_01791	LG3211_4034	LG3211_4034; hypothetical protein	OG_01791	LEC3_4102	conserved hypothetical protein
OG_01792	LG3211_340	secB; protein-export chaperone SecB	OG_01792	LEC3_5031	protein-export chaperone SecB
OG_01793	LG3211_4065	LG3211_4065; mutator mutT family protein	OG_01793	LEC3_4120	mutator MutT protein
OG_01794	LG3211_3904	LG3211_3904; repair family protein	OG_01794	LEC3_3923	conserved hypothetical protein
OG_01795	LG3211_825	LG3211_825; conserved hypothetical protein	OG_01795	LEC3_0774	oxacillin resistance-associated protein fmtc
OG_01796	LG3211_132	pgl; 6-phosphogluconolactonase	OG_01796	LEC3_5192	6-phosphogluconolactonase
OG_01797	LG3211_575	LG3211_575; methyltransferase domain protein	OG_01797	LEC3_4790	methyltransferase
OG_01798	LG3211_75	LG3211_75; hypothetical protein	OG_01798	LEC3_5279	conserved hypothetical protein
OG_01799	LG3211_885	thiC; thiamine biosynthesis protein ThiC	OG_01799	LEC3_0835	thiamine biosynthesis protein ThiC

OG_01800	LG3211_628	LG3211_628; ABC-2 type transporter family protein	OG_01800	LEC3_4722	ABC-2 type transporter
OG_01801	LG3211_4938	def; peptide deformylase	OG_01801	LEC3_0356	peptide deformylase
OG_01802	LG3211_2437	LG3211_2437; aminotransferase class-V family protein	OG_01802	LEC3_2379	Sphinganine-1-phosphate aldolase
OG_01803	LG3211_4111	LG3211_4111; conserved hypothetical protein	OG_01803	LEC3_4168	hypothetical protein
OG_01804	LG3211_4551	LG3211_4551; metallopeptidase M24 family protein	OG_01804	LEC3_4606	creatinase/prolidase N-terminal domain/metallopeptidase family M24
OG_01805	LG3211_3316	LG3211_3316; mitochondrial biogenesis AIM24 family protein	OG_01805	LEC3_3368	conserved hypothetical protein
OG_01806	LG3211_2721	sucA; oxoglutarate dehydrogenase (succinyl-transferring), E1 compor	OG_01806	LEC3_2723	oxoglutarate dehydrogenase, E1 component
OG_01807	LG3211_512	LG3211_512; sulfite exporter TauE/SafE family protein	OG_01807	LEC3_0327	conserved hypothetical protein
OG_01808	LG3211_4145	LG3211_4145; conserved hypothetical protein	OG_01808	LEC3_4201	conserved hypothetical protein
OG_01809	LG3211_4323	LG3211_4323; alpha/beta hydrolase fold family protein	OG_01809	LEC3_4376	alpha/beta hydrolase fold family protein
OG_01810	LG3211_2183	asd; aspartate-semialdehyde dehydrogenase	OG_01810	LEC3_2142	aspartate-semialdehyde dehydrogenase
OG_01811	LG3211_1919	pgaB; poly-beta-1,6-N-acetyl-D-glucosamine N-deacetylase PgaB	OG_01811	LEC3_1870	biofilm PGA synthesis lipoprotein PgaB
OG_01812	LG3211_3880	LG3211_3880; EF hand family protein	OG_01812	LEC3_3898	hypothetical protein
OG_01813	LG3211_2818	LG3211_2818; conserved hypothetical protein	OG_01813	LEC3_2823	virB7-like protein
OG_01814	LG3211_3061	fadE7; ACYL-CoA DEHYDROGENASE FADE7	OG_01814	LEC3_3055	glutaryl-CoA dehydrogenase, (GCD)
OG_01815	LG3211_1610	LG3211_1610; D-alanyl-D-alanine carboxypeptidase family protein	OG_01815	LEC3_1584	D-alanyl-D-alanine carboxypeptidase
OG_01816	LG3211_5368	LG3211_5368; tonB family C-terminal domain protein	OG_01816	LEC3_5350	TonB family C-terminal domain
OG_01817	LG3211_5307	LG3211_5307; conserved hypothetical protein	OG_01817	LEC3_5433	conserved hypothetical protein
OG_01818	LG3211_1884	cysS; cysteine--tRNA ligase	OG_01818	LEC3_1832	cysteinyl-tRNA synthetase
OG_01819	LG3211_1209	proC; pyrroline-5-carboxylate reductase	OG_01819	LEC3_1160	pyrroline-5-carboxylate reductase
OG_01820	LG3211_7	LG3211_7; tetratricopeptide repeat family protein	OG_01820	LEC3_5332	tetratricopeptide repeat domain protein
OG_01821	LG3211_2015	LG3211_2015; diguanylate cyclase domain protein	OG_01821	LEC3_1947	PAS domain protein
OG_01822	LG3211_4498	hemC; porphobilinogen deaminase	OG_01822	LEC3_4552	porphobilinogen deaminase
OG_01823	LG3211_1271	LG3211_1271; mobA-like NTP transferase domain protein	OG_01823	LEC3_1227	nucleotidyltransferase family protein
OG_01824	LG3211_986	tkt; transketolase	OG_01824	LEC3_0948	transketolase
OG_01825	LG3211_3325	dxr; 1-deoxy-D-xylulose 5-phosphate reductoisomerase	OG_01825	LEC3_3377	1-deoxy-D-xylulose 5-phosphate reductoisomerase
OG_01826	LG3211_664	LG3211_664; hypothetical protein	OG_01826	LEC3_4682	conserved hypothetical protein
OG_01827	LG3211_3975	chr; chromate transporter, chromate ion transporter family protein	OG_01827	LEC3_4016	chromate transporter, chromate ion transporter (CHR) family
OG_01828	LG3211_2411	LG3211_2411; DEAD/DEAH box helicase family protein	OG_01828	LEC3_2356	dead/H associated family
OG_01829	LG3211_3064	LG3211_3064; putative transmembrane protein	OG_01829	LEC3_3057	conserved hypothetical protein
OG_01830	LG3211_1055	LG3211_1055; HAMP domain protein	OG_01830	LEC3_1024	sensor histidine kinase
OG_01831	LG3211_37	LG3211_37; bacterial regulatory , Fis family protein	OG_01831	LEC3_5286	sigma-54 dependent transcriptional regulator
OG_01832	LG3211_5221	LG3211_5221; diguanylate cyclase domain protein	OG_01832	LEC3_0067	diguanylate cyclase
OG_01833	LG3211_753	LG3211_753; tfoX C-terminal domain protein	OG_01833	LEC3_0716	TfoX C- domain superfamily
OG_01834	LG3211_4409	LG3211_4409; conserved hypothetical protein	OG_01834	LEC3_4455	transport protein
OG_01835	LG3211_3041	lpxK; tetraacyldisaccharide 4'-kinase	OG_01835	LEC3_3032	tetraacyldisaccharide 4'-kinase
OG_01836	LG3211_2906	galU; UTP-glucose-1-phosphate uridylyltransferase	OG_01836	LEC3_2919	UTP-glucose-1-phosphate uridylyltransferase
OG_01837	LG3211_976	pilO; pilO protein	OG_01837	LEC3_0934	Type IV assembly protein
OG_01838	LG3211_2150	LG3211_2150; flavo, HI0933 family protein	OG_01838	LEC3_2091	conserved hypothetical protein
OG_01839	LG3211_4650	LG3211_4650; acyltransferase family protein	OG_01839	LEC3_0534	acyltransferase family
OG_01840	LG3211_3292	rnt; ribonuclease T	OG_01840	LEC3_3323	ribonuclease T
OG_01841	LG3211_4078	ftsW; cell division protein FtsW	OG_01841	LEC3_4132	cell division protein FtsW
OG_01842	LG3211_678	LG3211_678; RNA polymerase sigma factor, sigma-70 family protein	OG_01842	LEC3_4671	RNA polymerase sigma-E factor, ECR family
OG_01843	LG3211_3166	panD; aspartate 1-decarboxylase	OG_01843	LEC3_3173	aspartate 1-decarboxylase
OG_01844	LG3211_5352	LG3211_5352; aldo/keto reductase family protein	OG_01844	LEC3_5369	oxidoreductase, aldo/keto reductase family
OG_01845	LG3211_3526	LG3211_3526; pepSY-associated TM helix family protein	OG_01845	LEC3_3558	aspartate kinase
OG_01846	LG3211_4117	LG3211_4117; type II secretion system (T2SS), M subtype b family pr	OG_01846	LEC3_4174	general secretion pathway protein M
OG_01847	LG3211_3863	prmC; protein-(glutamine-N5) methyltransferase, release factor-speci	OG_01847	LEC3_3882	N5-glutamine methyltransferase
OG_01848	LG3211_5062	LG3211_5062; phosphopantetheine attachment site family protein	OG_01848	LEC3_4978	conserved hypothetical protein
OG_01849	LG3211_5181	plsB; glycerol-3-phosphate O-acyltransferase	OG_01849	LEC3_0106	acyltransferase
OG_01850	LG3211_3479	mutY; A/G-specific adenine glycosylase	OG_01850	LEC3_3536	A/G-specific adenine glycosylase
OG_01851	LG3211_4119	LG3211_4119; type II secretion system (T2SS), K family protein	OG_01851	LEC3_4176	general secretion pathway protein K
OG_01852	LG3211_3146	LG3211_3146; hypothetical protein	OG_01852	LEC3_3154	hypothetical protein
OG_01853	LG3211_5094	LG3211_5094; conserved hypothetical protein	OG_01853	LEC3_5002	integral membrane protein
OG_01854	LG3211_1030	LG3211_1030; acetyltransferase domain protein	OG_01854	LEC3_0995	acetyltransferase (GNAT) family
OG_01855	LG3211_2168	LG3211_2168; hypothetical protein	OG_01855	LEC3_2124	conserved hypothetical protein
OG_01856	LG3211_5075	cysT; sulfate ABC transporter, permease protein CysT	OG_01856	LEC3_4989	sulfate ABC transporter, permease protein CysT
OG_01857	LG3211_827	LG3211_827; zinc-binding dehydrogenase family protein	OG_01857	LEC3_0776	oxidoreductase, zinc-binding dehydrogenase family
OG_01858	LG3211_1542	purA; adenylosuccinate synthase	OG_01858	LEC3_1516	adenylosuccinate synthetase
OG_01859	LG3211_1755	recO; DNA repair protein RecO	OG_01859	LEC3_1712	DNA repair protein
OG_01860	LG3211_5037	LG3211_5037; M61 glycyl aminopeptidase family protein	OG_01860	LEC3_4947	conserved hypothetical protein
OG_01861	LG3211_2315	carB; carbamoyl-phosphate synthase, large subunit	OG_01861	LEC3_2238	carbamoyl-phosphate synthase, large subunit
OG_01862	LG3211_1783	ispG; 4-hydroxy-3-methylbut-2-en-1-yl diphosphate synthase	OG_01862	LEC3_1746	4-hydroxy-3-methylbut-2-en-1-yl diphosphate synthase
OG_01863	LG3211_2646	LG3211_2646; conserved hypothetical protein	OG_01863	LEC3_2652	conserved hypothetical protein
OG_01864	LG3211_3230	rpsP; ribosomal protein S16	OG_01864	LEC3_3255	ribosomal protein S16
OG_01865	LG3211_2132	LG3211_2132; sugar (and other) transporter family protein	OG_01865	LEC3_2076	transporter, major facilitator family
OG_01866	LG3211_2644	rpsR; ribosomal protein S18	OG_01866	LEC3_2649	ribosomal protein S18
OG_01867	LG3211_3608	petA; ubiquinol-cytochrome c reductase, iron-sulfur subunit	OG_01867	LEC3_3601	ubiquinol-cytochrome c reductase, iron-sulfur subunit
OG_01868	LG3211_2253	LG3211_2253; conserved hypothetical protein	OG_01868	LEC3_2206	conserved hypothetical protein
OG_01869	LG3211_1404	LG3211_1404; conserved hypothetical protein	OG_01869	LEC3_1369	activator of Hsp90 ATPase homolog 1-like protein
OG_01870	LG3211_1423	LG3211_1423; MAPEG family protein	OG_01870	LEC3_1389	inner membrane protein
OG_01871	LG3211_406	LG3211_406; nitronate monooxygenase family protein	OG_01871	LEC3_0230	oxidoreductase, 2-nitropropane dioxygenase family
OG_01872	LG3211_3618	LG3211_3618; ABC transporter family protein	OG_01872	LEC3_3609	ABC transporter, ATP-binding protein
OG_01873	LG3211_369	LG3211_369; AAA domain family protein	OG_01873	LEC3_0187	conserved hypothetical protein
OG_01874	LG3211_1795	pyrD; dihydroorotate dehydrogenase	OG_01874	LEC3_1758	dihydroorotate oxidase
OG_01875	LG3211_2259	LG3211_2259; RES domain protein	OG_01875	LEC3_2212	RES domain superfamily
OG_01876	LG3211_1823	LG3211_1823; response regulator	OG_01876	LEC3_1780	Two component heavy metal response transcriptional regulator
OG_01877	LG3211_3743	secY; preprotein translocase, SecY subunit	OG_01877	LEC3_3770	preprotein translocase, SecY subunit
OG_01878	LG3211_1403	hutU; urocanate hydratase	OG_01878	LEC3_1368	urocanate hydratase
OG_01879	LG3211_714	atpF; ATP synthase F0, B subunit	OG_01879	LEC3_0685	ATP synthase F0, B subunit
OG_01880	LG3211_408	LG3211_408; hypothetical protein	OG_01880	LEC3_0233	conserved hypothetical protein

OG_01881	LG3211_812	prmA; ribosomal protein L11 methyltransferase	OG_01881	LEC3_0762	ribosomal protein L11 methyltransferase
OG_01882	LG3211_57	LG3211_57; FAD-NAD(P)-binding family protein	OG_01882	LEC3_5262	pyridine nucleotide-disulphide oxidoreductase
OG_01883	LG3211_1753	rnc; ribonuclease III	OG_01883	LEC3_1710	ribonuclease III
OG_01884	LG3211_917	LG3211_917; argD	OG_01884	LEC3_0870	succinylornithine transaminase
OG_01885	LG3211_3746	rpsE; ribosomal protein S5	OG_01885	LEC3_3773	ribosomal protein S5
OG_01886	LG3211_388	ubiB; 2-polyprenylphenol 6-hydroxylase	OG_01886	LEC3_0206	2-polyprenylphenol 6-hydroxylase
OG_01887	LG3211_3069	MEP; peptidyl-Lys metalloendopeptidase	OG_01887	LEC3_3062	extracellular protease
OG_01888	LG3211_633	LG3211_633; TPR repeat family protein	OG_01888	LEC3_4715	tetratricopeptide repeat domain protein
OG_01889	LG3211_2815	LG3211_2815; bacterial conjugation TrbI-like family protein	OG_01889	LEC3_2820	VirB10 protein
OG_01890	LG3211_576	LG3211_576; GDSL-like Lipase/Acylhydrolase family protein	OG_01890	LEC3_4789	GDSL-like Lipase/Acylhydrolase
OG_01891	LG3211_5149	LG3211_5149; dinB family protein	OG_01891	LEC3_0136	DinB family protein
OG_01892	LG3211_196	LG3211_196; subtilase family protein	OG_01892	LEC3_5135	peptidase, families S8 and S53
OG_01893	LG3211_2924	LG3211_2924; traB family protein	OG_01893	LEC3_2935	traB family protein
OG_01894	LG3211_2272	LG3211_2272; conserved hypothetical protein	OG_01894	LEC3_1036	conserved hypothetical protein
OG_01895	LG3211_5164	tatC; twin arginine-targeting protein translocase TatC	OG_01895	LEC3_0124	twin arginine-targeting protein translocase TatC
OG_01896	LG3211_323	SOD1; superoxide dismutase [Cu-Zn] domain protein	OG_01896	LEC3_5046	superoxide dismutase, Cu-Zn
OG_01897	LG3211_1771	alpha-LP; alpha-lytic protease	OG_01897	LEC3_1730	alpha-lytic protease prodomain/trypsin
OG_01898	LG3211_5333	LG3211_5333; lysM domain protein	OG_01898	LEC3_5395	LysM/phospholipid-binding domain protein
OG_01899	LG3211_4677	LG3211_4677; bacterial regulatory helix-turn-helix , lysR family protei	OG_01899	LEC3_0568	transcriptional regulator, LysR family
OG_01900	LG3211_1122	LG3211_1122; bacterial regulatory, tetR family protein	OG_01900	LEC3_1098	transcriptional regulator, TetR family
OG_01901	LG3211_2166	ytfC; peptidyl-prolyl cis-trans isomerase	OG_01901	LEC3_2120	peptidyl-prolyl cis-trans isomerase, FKBP-type
OG_01902	LG3211_1635	LG3211_1635; conserved hypothetical protein	OG_01902	LEC3_1604	conserved hypothetical protein
OG_01903	LG3211_3334	LG3211_3334; conserved hypothetical protein	OG_01903	LEC3_3386	hypothetical protein
OG_01904	LG3211_208	LG3211_208; hypothetical protein	OG_01904	LEC3_5124	hypothetical protein
OG_01905	LG3211_231	LG3211_231; hypothetical protein	OG_01905	LEC3_5099	hypothetical protein
OG_01906	LG3211_2357	LG3211_2357; conserved hypothetical protein	OG_01906	LEC3_2303	conserved hypothetical protein
OG_01907	LG3211_1379	lpxH; UDP-2,3-diacylglucosamine hydrolase	OG_01907	LEC3_1346	UDP-2,3-diacylglucosamine hydrolase
OG_01908	LG3211_2850	LG3211_2850; 3HB-oligomer hydrolase family protein	OG_01908	LEC3_2861	lipoprotein
OG_01909	LG3211_2613	LG3211_2613; putative predicted protein	OG_01909	LEC3_2618	hypothetical protein
OG_01910	LG3211_1437	LG3211_1437; tonB dependent receptor family protein	OG_01910	LEC3_1406	TonB-dependent receptor
OG_01911	LG3211_3199	LG3211_3199; acetyltransferase family protein	OG_01911	LEC3_3206	transcriptional regulator, MarR family; N-acetyltransferase
OG_01912	LG3211_1346	LG3211_1346; zinc-binding alcohol dehydrogenase family protein	OG_01912	LEC3_1301	zinc-binding alcohol dehydrogenase family protein
OG_01913	LG3211_4035	LG3211_4035; conserved hypothetical protein	OG_01913	LEC3_4103	conserved hypothetical protein
OG_01914	LG3211_371	LG3211_371; coA-transferase III family protein	OG_01914	LEC3_0189	L-carnitine dehydratase/bile acid-inducible protein F
OG_01915	LG3211_4916	LG3211_4916; alpha-1,2-mannosidase family protein	OG_01915	LEC3_0378	alpha-1,2-mannosidase, putative
OG_01916	LG3211_3413	LG3211_3413; bacterial low temperature requirement A family protein	OG_01916	LEC3_3476	low temperature requirement protein LtrA
OG_01917	LG3211_1493	LG3211_1493; glutathione amide reductase	OG_01917	LEC3_1468	glutathione reductase
OG_01918	LG3211_507	cca; tRNA nucleotidyltransferase	OG_01918	LEC3_0323	tRNA nucleotidyltransferase
OG_01919	LG3211_3645	LG3211_3645; carbon starvation CstA family protein	OG_01919	LEC3_3632	carbon starvation family protein
OG_01920	LG3211_1351	LG3211_1351; catalytic LigB subunit of aromatic ring-opening dioxyg	OG_01920	LEC3_1307	4,5-dopa dioxygenase extradiol
OG_01921	LG3211_1076	LG3211_1076; TIM-barrel , nifR3 family protein	OG_01921	LEC3_1048	tRNA-dihydrouridine synthase B
OG_01922	LG3211_574	LG3211_574; polysaccharide deacetylase family protein	OG_01922	LEC3_4794	polysaccharide deacetylase
OG_01923	LG3211_2120	LG3211_2120; carboxylate-amine ligase, YbdK family protein	OG_01923	LEC3_2066	glutamate-cysteine ligase family 2
OG_01924	LG3211_686	LG3211_686; killing trait family protein	OG_01924	LEC3_4664	RebB protein
OG_01925	LG3211_112	gidA; tRNA uridine 5-carboxymethylaminomethyl modification enzyme	OG_01925	LEC3_5211	tRNA uridine 5-carboxymethylaminomethyl modification enzyme GidA
OG_01926	LG3211_3654	rfbD; dTDP-4-dehydrorhamnose reductase	OG_01926	LEC3_3640	dTDP-4-dehydrorhamnose reductase
OG_01927	LG3211_4096	ribH; 6,7-dimethyl-8-ribityllumazine synthase	OG_01927	LEC3_4151	6,7-dimethyl-8-ribityllumazine synthase
OG_01928	LG3211_2824	LG3211_2824; heme exporter D family protein	OG_01928	LEC3_2829	heme exporter protein CcmD
OG_01929	LG3211_5219	folE; GTP cyclohydrolase I	OG_01929	LEC3_0069	GTP cyclohydrolase I
OG_01930	LG3211_1426	LG3211_1426; bacterial regulatory , arsR family protein	OG_01930	LEC3_1391	transcriptional regulator, ArsR family
OG_01931	LG3211_4419	LG3211_4419; ABC transporter family protein	OG_01931	LEC3_4463	ABC transporter, ATP-binding protein
OG_01932	LG3211_1428	LG3211_1428; putative fatty acid desaturase protein	OG_01932	LEC3_1395	fatty acid desaturase
OG_01933	LG3211_4104	LG3211_4104; aspartyl/Asparaginyl beta-hydroxylase family protein	OG_01933	LEC3_4162	aspartyl/asparaginyl beta-hydroxylase family
OG_01934	LG3211_2024	LG3211_2024; feoA domain protein	OG_01934	LEC3_1955	FeoA domain
OG_01935	LG3211_5114	LG3211_5114; glycosyl transferase 2 family protein	OG_01935	LEC3_0172	glycosyl transferase, group 2 family protein
OG_01936	LG3211_2774	LG3211_2774; binding-protein-dependent transport system inner mem	OG_01936	LEC3_2780	ABC transporter, permease protein
OG_01938	LG3211_3775	rplK; ribosomal protein L11	OG_01938	LEC3_3801	ribosomal protein L11
OG_01939	LG3211_490	LG3211_490; hypothetical protein	OG_01939	LEC3_0302	conserved hypothetical protein
OG_01940	LG3211_4926	rpoD; RNA polymerase sigma factor RpoD	OG_01940	LEC3_0368	RNA polymerase primary sigma factor
OG_01941	LG3211_3300	LG3211_3300; conserved hypothetical protein	OG_01941	LEC3_3342	conserved hypothetical protein
OG_01942	LG3211_2223	pnp; polyribonucleotide nucleotidyltransferase	OG_01942	LEC3_2180	Polyribonucleotide nucleotidyltransferase, RNA binding domain
OG_01943	LG3211_4102	nrdR; transcriptional regulator NrdR	OG_01943	LEC3_4159	transcriptional regulator NrdR
OG_01944	LG3211_3386	LG3211_3386; xaa-Pro dipeptidase, putative	OG_01944	LEC3_3443	amidohydrolase family protein
OG_01945	LG3211_3426	LG3211_3426; conserved hypothetical protein	OG_01945	LEC3_3489	conserved hypothetical protein
OG_01946	LG3211_780	LG3211_780; AIG2-like family protein	OG_01946	LEC3_2412	conserved hypothetical protein
OG_01947	LG3211_1033	LG3211_1033; peptidase M28	OG_01947	LEC3_0999	peptidase, M28 family
OG_01948	LG3211_5108	gidB; 16S rRNA (guanine(527)-N(7))-methyltransferase GidB	OG_01948	LEC3_0178	methyltransferase GidB
OG_01949	LG3211_1704	LG3211_1704; lipopolysaccharide kinase family protein	OG_01949	LEC3_1666	lipopolysaccharide kinase (Kdo/WaaP) family
OG_01950	LG3211_3277	LG3211_3277; hypothetical protein	OG_01950	LEC3_3310	hypothetical protein
OG_01951	LG3211_713	atpE; ATP synthase F0, C subunit	OG_01951	LEC3_0684	ATP synthase F0, C subunit
OG_01952	LG3211_85	LG3211_85; STAS domain protein	OG_01952	LEC3_5237	conserved hypothetical protein
OG_01953	LG3211_4634	LG3211_4634; nucleotide sugar dehydrogenase family protein	OG_01953	LEC3_2254	NDP-N-acetyl-D-galactosaminuronic acid dehydrogenase
OG_01954	LG3211_3428	LG3211_3428; conserved hypothetical protein	OG_01954	LEC3_3491	conserved hypothetical protein
OG_01955	LG3211_5144	LG3211_5144; CHAD domain protein	OG_01955	LEC3_0143	hypothetical protein
OG_01956	LG3211_3235	LG3211_3235; marR family protein	OG_01956	LEC3_3262	transcriptional regulator, MarR family
OG_01957	LG3211_4303	LG3211_4303; cysteine-rich CPXCG family protein	OG_01957	LEC3_4362	conserved domain protein
OG_01958	LG3211_2675	LG3211_2675; conserved hypothetical protein	OG_01958	LEC3_2681	conserved hypothetical protein
OG_01959	LG3211_3381	leuS; leucine--tRNA ligase	OG_01959	LEC3_3437	leucyl-tRNA synthetase
OG_01960	LG3211_211	LG3211_211; type III secretion , HrpO family protein	OG_01960	LEC3_5121	type III secretion family protein
OG_01961	LG3211_1636	LG3211_1636; conserved hypothetical protein	OG_01961	LEC3_1605	RNA methylase family
OG_01962	LG3211_3772	rplL; ribosomal protein L7/L12	OG_01962	LEC3_3798	ribosomal protein L12

OG_01963	LG3211_1244	murA; UDP-N-acetylglucosamine 1-carboxyvinyltransferase	OG_01963	LEC3_1199	UDP-N-acetylglucosamine 1-carboxyvinyltransferase
OG_01964	LG3211_1165	LG3211_1165; ycel-like domain protein	OG_01964	LEC3_1139	Ycel family protein
OG_01965	LG3211_5184	LG3211_5184; spore Coat Protein U domain protein	OG_01965	LEC3_0103	protein U domain protein
OG_01966	LG3211_3921	LG3211_3921; NUDIX domain protein	OG_01966	LEC3_3945	ADP compounds hydrolase
OG_01967	LG3211_1291	LG3211_1291; pepSY-associated TM helix family protein	OG_01967	LEC3_1247	PepSY-associated TM helix domain protein
OG_01968	LG3211_597	aceE; pyruvate dehydrogenase (acetyl-transferring), homodimeric typ	OG_01968	LEC3_4769	pyruvate dehydrogenase (acetyl-transferring), homodimeric type
OG_01969	LG3211_531	LG3211_531; periplasmic binding and sugar binding domain of Lacl f	OG_01969	LEC3_0343	transcriptional regulator, LacI family
OG_01970	LG3211_2888	LG3211_2888; thiF family protein	OG_01970	LEC3_2901	molybdopterin biosynthesis protein
OG_01971	LG3211_1961	mtnB; methylthioribulose-1-phosphate dehydratase	OG_01971	LEC3_1912	methylthioribulose-1-phosphate dehydratase
OG_01972	LG3211_4980	LG3211_4980; response regulator	OG_01972	LEC3_4889	DNA-binding response regulator
OG_01973	LG3211_1706	moaA; molybdenum cofactor biosynthesis protein A	OG_01973	LEC3_1668	molybdenum cofactor biosynthesis protein A
OG_01974	LG3211_998	gap; glyceraldehyde-3-phosphate dehydrogenase, type I	OG_01974	LEC3_0962	glyceraldehyde-3-phosphate dehydrogenase, type I
OG_01975	LG3211_3891	LG3211_3891; ostA-like family protein	OG_01975	LEC3_3910	LPS-assembly protein (Organic solvent tolerance protein)
OG_01976	LG3211_3379	LG3211_3379; lipopolysaccharide-assembly family protein	OG_01976	LEC3_3436	rare lipoprotein B
OG_01977	LG3211_446	pyrE; orotate phosphoribosyltransferase	OG_01977	LEC3_0257	orotate phosphoribosyltransferase
OG_01978	LG3211_549	LG3211_549; conserved hypothetical protein	OG_01978	LEC3_4833	conserved hypothetical protein
OG_01979	LG3211_4501	LG3211_4501; alpha/beta hydrolase fold family protein	OG_01979	LEC3_4555	phospholipase/carboxylesterase
OG_01980	LG3211_4261	LG3211_4261; putative stress response protein	OG_01980	LEC3_4331	conserved domain protein
OG_01981	LG3211_1598	LG3211_1598; conserved hypothetical protein	OG_01981	LEC3_1573	conserved hypothetical protein
OG_01982	LG3211_4084	mraW; S-adenosyl-methyltransferase MraW	OG_01982	LEC3_4138	S-adenosyl-methyltransferase MraW
OG_01983	LG3211_2254	lexA; repressor LexA	OG_01983	LEC3_2207	LexA repressor
OG_01984	LG3211_708	lpdA; dihydrolipoyl dehydrogenase	OG_01984	LEC3_0677	dihydrolipoyl dehydrogenase
OG_01985	LG3211_3514	LG3211_3514; hypothetical protein	OG_01985	LEC3_3546	lipoprotein
OG_01986	LG3211_1739	fabF; beta-ketoacyl-acyl-carrier-protein synthase II	OG_01986	LEC3_1694	3-oxoacyl-[acyl-carrier-protein] synthase 2
OG_01987	LG3211_3430	LG3211_3430; ATPase associated with various cellular activities fami	OG_01987	LEC3_3494	ATPase, AAA family protein
OG_01988	LG3211_2205	nuoG; NADH dehydrogenase (quinone), G subunit	OG_01988	LEC3_2165	NADH dehydrogenase, G subunit
OG_01989	LG3211_3240	LG3211_3240; enoyl-CoA hydratase/isomerase family protein	OG_01989	LEC3_3267	formyl transferase/enoyl-CoA hydratase/isomerase family protein
OG_01990	LG3211_2849	pssA; CDP-diacylglycerol-serine O-phosphatidyltransferase	OG_01990	LEC3_2860	CDP-diacylglycerol--serine O-phosphatidyltransferase
OG_01991	LG3211_2954	LG3211_2954; ahpC/TSA family protein	OG_01991	LEC3_2970	redoxin/antioxidant, AhpC/TSA family
OG_01992	LG3211_2782	LG3211_2782; conserved hypothetical protein	OG_01992	LEC3_2789	lipoprotein
OG_01993	LG3211_897	LG3211_897; fatty acid hydroxylase superfamily protein	OG_01993	LEC3_0843	conserved hypothetical protein
OG_01994	LG3211_3622	LG3211_3622; saccharopine dehydrogenase family protein	OG_01994	LEC3_3613	3-beta hydroxysteroid dehydrogenase/isomerase family protein
OG_01995	LG3211_4530	LG3211_4530; conserved hypothetical protein	OG_01995	LEC3_4584	outer membrane protein
OG_01996	LG3211_1222	LG3211_1222; conserved hypothetical protein	OG_01996	LEC3_1177	conserved hypothetical protein
OG_01997	LG3211_2422	LG3211_2422; 2OG-Fe(II) oxygenase superfamily protein	OG_01997	LEC3_2366	conserved hypothetical protein
OG_01998	LG3211_3551	LG3211_3551; putative pilW	OG_01998	LEC3_3578	Type IV pilus assembly protien
OG_01999	LG3211_1054	LG3211_1054; transcriptional regulatory , C terminal family protein	OG_01999	LEC3_1023	Transcriptional regulatory protein, C terminal
OG_02000	LG3211_626	LG3211_626; conserved hypothetical protein	OG_02000	LEC3_4724	activator of Hsp90 ATPase homolog 1-like protein
OG_02001	LG3211_433	LG3211_433; NAD binding domain of 6-phosphogluconate dehydroge	OG_02001	LEC3_0246	Uncharacterized oxidoreductase
OG_02002	LG3211_4042	sucC; succinyl-CoA synthetase, &beta subunit	OG_02002	LEC3_4111	succinyl-CoA synthetase, beta subunit
OG_02003	LG3211_2727	LG3211_2727; response regulator	OG_02003	LEC3_2730	Type IV assembly protein
OG_02004	LG3211_3072	LG3211_3072; 'Cold-shock' DNA-binding domain protein	OG_02004	LEC3_3063	'Cold-shock' DNA-binding domain
OG_02005	LG3211_5215	LG3211_5215; sugar (and other) transporter family protein	OG_02005	LEC3_0075	tetracycline resistance protein, class A (TetA(A))
OG_02006	LG3211_1679	LG3211_1679; ppx/GppA phosphatase family protein	OG_02006	LEC3_1646	Ppx/GppA phosphatase family
OG_02007	LG3211_4298	LG3211_4298; hydrolase, haloacid delahogenase-like family	OG_02007	LEC3_4358	haloacid dehalogenase-like hydrolase
OG_02008	LG3211_3361	LG3211_3361; bacterial DNA-binding family protein	OG_02008	LEC3_3415	DNA-binding protein HU-beta
OG_02009	LG3211_3776	nusG; transcription termination/antitermination factor NusG	OG_02009	LEC3_3802	transcription termination/antitermination protein NusG
OG_02010	LG3211_5160	glyQ; glycine--tRNA ligase, alpha subunit	OG_02010	LEC3_0128	glycyl-tRNA synthetase, alpha subunit
OG_02011	LG3211_5166	tatA; twin arginine-targeting translocase, TatA/E family protein	OG_02011	LEC3_0122	twin arginine-targeting protein translocase, TatA/E family
OG_02012	LG3211_5361	LG3211_5361; ABC-2 type transporter family protein	OG_02012	LEC3_5358	ABC-2 type transporter
OG_02013	LG3211_5133	LG3211_5133; conserved hypothetical protein	OG_02013	LEC3_0151	membrane protein
OG_02014	LG3211_5131	LG3211_5131; sugar (and other) transporter family protein	OG_02014	LEC3_0154	transporter, major facilitator superfamily
OG_02015	LG3211_3764	rpsJ; ribosomal protein S10	OG_02015	LEC3_3791	ribosomal protein S10
OG_02016	LG3211_4508	LG3211_4508; putative lipoprotein	OG_02016	LEC3_4563	conserved hypothetical protein
OG_02017	LG3211_1458	LG3211_1458; conserved hypothetical protein	OG_02017	LEC3_1424	conserved hypothetical protein
OG_02018	LG3211_3790	lolB; outer membrane lipoprotein LolB	OG_02018	LEC3_3811	outer membrane lipoprotein LolB
OG_02019	LG3211_589	LG3211_589; malic enzyme, NAD binding domain protein	OG_02019	LEC3_4777	NADP-dependent malic enzyme
OG_02020	LG3211_1798	LG3211_1798; conserved hypothetical protein	OG_02020	LEC3_1761	conserved hypothetical protein
OG_02021	LG3211_13	pdxJ; pyridoxine 5'-phosphate synthase	OG_02021	LEC3_5323	pyridoxal phosphate biosynthesis protein PdxJ
OG_02022	LG3211_3429	LG3211_3429; AAA domain family protein	OG_02022	LEC3_3492	ATPase RavA
OG_02023	LG3211_17	LG3211_17; conserved hypothetical protein	OG_02023	LEC3_5320	protein of unknown function (DUF541)
OG_02024	LG3211_3267	LG3211_3267; conserved hypothetical protein	OG_02024	LEC3_3298	thioesterase superfamily
OG_02025	LG3211_2855	LG3211_2855; leucine Rich repeat family protein	OG_02025	LEC3_2867	CalU2
OG_02026	LG3211_4941	LG3211_4941; conserved hypothetical protein	OG_02026	LEC3_0352	smg protein
OG_02027	LG3211_3721	mdh; malate dehydrogenase	OG_02027	LEC3_3745	malate dehydrogenase
OG_02028	LG3211_2409	cueR; cu(I)-responsive transcriptional regulator	OG_02028	LEC3_2353	Cu(I)-responsive transcriptional regulator
OG_02029	LG3211_5187	LG3211_5187; spore Coat Protein U domain protein	OG_02029	LEC3_0100	spore coat U domain protein
OG_02030	LG3211_710	LG3211_710; conserved hypothetical protein	OG_02030	LEC3_0681	membrane protein
OG_02031	LG3211_1515	surE; 5'/3'-nucleotidase SurE	OG_02031	LEC3_1496	5'/3'-nucleotidase SurE
OG_02032	LG3211_1562	LG3211_1562; response regulator	OG_02032	LEC3_1539	response regulator receiver/LytTr DNA-binding domain protein
OG_02033	LG3211_982	LG3211_982; von Willebrand factor type A domain protein	OG_02033	LEC3_0940	von Willebrand factor, type A
OG_02034	LG3211_699	adhC2; NADP-dependent alcohol dehydrogenase C 2	OG_02034	LEC3_0667	alcohol dehydrogenase, zinc-containing
OG_02035	LG3211_2911	ihfB; integration host factor, beta subunit	OG_02035	LEC3_2924	integration host factor, beta subunit
OG_02036	LG3211_1374	LG3211_1374; colicin V production family protein	OG_02036	LEC3_1338	colicin V production protein
OG_02037	LG3211_1451	LG3211_1451; formate dehydrogenase family accessory protein FdhI	OG_02037	LEC3_1421	formate dehydrogenase family accessory protein FdhD
OG_02038	LG3211_4152	LG3211_4152; CAAX protease self-immunity family protein	OG_02038	LEC3_4218	CAAX protease family protein
OG_02039	LG3211_5005	LG3211_5005; response regulator	OG_02039	LEC3_4913	response regulator protein
OG_02040	LG3211_1330	LG3211_1330; putative secreted protein	OG_02040	LEC3_1285	rickettsia 17 kDa surface antigen family
OG_02041	LG3211_1478	LG3211_1478; peptidase M16 inactive domain protein	OG_02041	LEC3_1448	peptidase, M16 family
OG_02042	LG3211_754	LG3211_754; GAF domain protein	OG_02042	LEC3_0717	GAF domain protein
OG_02043	LG3211_803	LG3211_803; hypothetical protein	OG_02043	LEC3_0754	conserved hypothetical protein

OG_02044	LG3211_762	LG3211_762; conserved hypothetical protein	OG_02044	LEC3_0724	conserved hypothetical protein
OG_02045	LG3211_1667	LG3211_1667; transcriptional regulatory , C terminal family protein	OG_02045	LEC3_1628	transcriptional regulatory proteins, C terminal - transcriptional regulato
OG_02046	LG3211_5009	rhlB; EG10844	OG_02046	LEC3_4916	ATP-dependent RNA helicase
OG_02047	LG3211_2343	acnA; aconitate hydratase 1	OG_02047	LEC3_2294	aconitate hydratase 1
OG_02048	LG3211_3528	LG3211_3528; traB family protein	OG_02048	LEC3_3560	GumN protein
OG_02049	LG3211_929	LG3211_929; ABC transporter family protein	OG_02049	LEC3_0879	ABC transporter
OG_02050	LG3211_182	LG3211_182; phosphatidylethanolamine-binding family protein	OG_02050	LEC3_5150	conserved hypothetical protein
OG_02051	LG3211_3720	LG3211_3720; bacterial regulatory, luxR family protein	OG_02051	LEC3_3744	transcriptional regulator, LuxR/UhpA family
OG_02052	LG3211_828	bsn; extracellular ribonuclease domain protein	OG_02052	LEC3_0778	extracellular ribonuclease precusor
OG_02053	LG3211_530	LG3211_530; alpha amylase, catalytic domain protein	OG_02053	LEC3_0342	cyclomaltodextrin glucanotransferase
OG_02054	LG3211_204	LG3211_204; bacterial regulatory , Fis family protein	OG_02054	LEC3_5128	photosynthetic apparatus regulatory protein regA
OG_02055	LG3211_5077	cysA; sulfate ABC transporter, ATP-binding family protein	OG_02055	LEC3_4991	sulfate ABC transporter, ATP-binding protein
OG_02056	LG3211_4289	LG3211_4289; rtcR transcriptional regulator RtcR transcriptional activ	OG_02056	LEC3_4350	transcriptional regulatory protein RtcR
OG_02057	LG3211_3294	LG3211_3294; chitinase class I family protein	OG_02057	LEC3_3325	chitinase class I
OG_02058	LG3211_4122	LG3211_4122; putative general secretion pathway protein h	OG_02058	LEC3_4179	general secretion pathway protein H
OG_02059	LG3211_5214	LG3211_5214; hypothetical protein	OG_02059	LEC3_0076	hypothetical protein
OG_02060	LG3211_2217	infB; translation initiation factor IF-2	OG_02060	LEC3_2176	translation initiation factor IF-2
OG_02061	LG3211_89	LG3211_89; ABC transporter family protein	OG_02061	LEC3_5233	toluene ABC transporter
OG_02062	LG3211_3329	fabZ; beta-hydroxyacyl-(acyl-carrier-protein) dehydratase FabZ	OG_02062	LEC3_3381	beta-hydroxyacyl-(acyl-carrier-protein) dehydratase FabZ
OG_02063	LG3211_4495	nrfC; nrfC protein	OG_02063	LEC3_4547	dmso reductase chain B
OG_02064	LG3211_4112	LG3211_4112; metallo-beta-lactamase superfamily protein	OG_02064	LEC3_4169	ribonuclease Z
OG_02065	LG3211_2988	LG3211_2988; tonB family C-terminal domain protein	OG_02065	LEC3_2979	TonB family C-terminal domain
OG_02066	LG3211_155	LG3211_155; AIG2-like family protein	OG_02066	LEC3_5171	conserved hypothetical protein
OG_02067	LG3211_3364	clpP; ATP-dependent Clp endopeptidase, proteolytic subunit ClpP	OG_02067	LEC3_3419	ATP-dependent Clp protease, proteolytic subunit ClpP
OG_02068	LG3211_1093	LG3211_1093; hypothetical protein	OG_02068	LEC3_1067	hypothetical protein
OG_02069	LG3211_5059	LG3211_5059; bacterial lipid A biosynthesis acyltransferase family pr	OG_02069	LEC3_4975	lipid A biosynthesis (KDO)2-(lauroyl)-lipid IVA acyltransferase
OG_02070	LG3211_3156	LG3211_3156; traB family protein	OG_02070	LEC3_3163	GumN protein
OG_02071	LG3211_1072	LG3211_1072; calcineurin-like phosphoesterase superfamily domain	OG_02071	LEC3_1044	Ser/Thr protein phosphatase family protein
OG_02072	LG3211_3047	LG3211_3047; liporeleasing system, transmembrane , LolC/E family	OG_02072	LEC3_3039	lipoprotein releasing system, transmembrane protein, LolC/E family
OG_02073	LG3211_4165	LG3211_4165; aldose 1-epimerase family protein	OG_02073	LEC3_4231	aldose 1-epimerase
OG_02074	LG3211_743	LG3211_743; hypothetical protein	OG_02074	LEC3_0707	hypothetical protein
OG_02075	LG3211_3708	LG3211_3708; phenazine biosynthesis , PhzF family protein	OG_02075	LEC3_3733	phenazine biosynthesis protein PhzF family
OG_02076	LG3211_4547	LG3211_4547; helix-turn-helix domain, rpiR family protein	OG_02076	LEC3_4602	transcriptional regulator, RpiR family
OG_02077	LG3211_3317	pyrH; UMP kinase	OG_02077	LEC3_3369	uridylate kinase
OG_02078	LG3211_959	LG3211_959; endoribonuclease L-PSP, putative	OG_02078	LEC3_0913	endoribonuclease L-PSP family protein
OG_02079	LG3211_983	LG3211_983; von Willebrand factor type A domain protein	OG_02079	LEC3_0941	tetratricopeptide repeat family protein
OG_02080	LG3211_2842	LG3211_2842; conserved hypothetical protein	OG_02080	LEC3_2854	conserved hypothetical protein
OG_02081	LG3211_3015	LG3211_3015; diguanylate cyclase domain protein	OG_02081	LEC3_3007	diguanylate cyclase
OG_02082	LG3211_393	LG3211_393; acyltransferase family protein	OG_02082	LEC3_0215	acyltransferase
OG_02083	LG3211_5345	LG3211_5345; hypothetical protein	OG_02083	LEC3_5376	hypothetical protein
OG_02084	LG3211_168	LG3211_168; conserved hypothetical protein	OG_02084	LEC3_5163	conserved hypothetical protein
OG_02085	LG3211_5318	LG3211_5318; hypothetical protein	OG_02085	LEC3_5416	conserved hypothetical protein
OG_02086	LG3211_992	modB; molybdate ABC transporter, permease protein	OG_02086	LEC3_0954	molybdate ABC transporter, permease protein
OG_02087	LG3211_205	LG3211_205; conserved hypothetical protein	OG_02087	LEC3_5127	conserved hypothetical protein
OG_02088	LG3211_2419	LG3211_2419; BON domain protein	OG_02088	LEC3_2361	transport-associated protein
OG_02089	LG3211_510	LG3211_510; transglycosylase SLT domain protein	OG_02089	LEC3_0326	transglycosylase SLT domain
OG_02090	LG3211_4120	LG3211_4120; prepilin-type N-terminal cleavage/methylation domain	OG_02090	LEC3_4177	general secretion pathway protein J
OG_02091	LG3211_2793	LG3211_2793; hypothetical protein	OG_02091	LEC3_2800	conserved hypothetical protein
OG_02092	LG3211_4220	trpG; anthranilate synthase component II	OG_02092	LEC3_4273	Anthranilate synthase component II
OG_02093	LG3211_561	LG3211_561; amidohydrolase family protein	OG_02093	LEC3_4810	amidohydrolase family protein
OG_02094	LG3211_2937	LG3211_2937; ribonuclease, Rne/Rng family domain protein	OG_02094	LEC3_2949	ribonuclease E
OG_02095	LG3211_471	LG3211_471; conserved hypothetical protein	OG_02095	LEC3_0283	conserved hypothetical protein
OG_02096	LG3211_1617	LG3211_1617; hypothetical protein	OG_02096	LEC3_1591	hypothetical protein
OG_02097	LG3211_1818	parC; DNA topoisomerase IV, A subunit	OG_02097	LEC3_1776	DNA topoisomerase IV, A subunit
OG_02098	LG3211_2985	LG3211_2985; bacterial regulatory helix-turn-helix , lysR family protei	OG_02098	LEC3_2975	transcriptional regulator, LysR family
OG_02099	LG3211_2454	LG3211_2454; HNH/ENDO VII supernuclease with conserved GHE r	OG_02099	LEC3_2431	conserved hypothetical protein
OG_02100	LG3211_993	modA; molybdate ABC transporter, periplasmic molybdate-binding pr	OG_02100	LEC3_0955	molybdate ABC transporter, periplasmic molybdate-binding protein
OG_02101	LG3211_5085	LG3211_5085; flagellin N-methylase family protein	OG_02101	LEC3_4997	UPF0153 protein
OG_02102	LG3211_3455	yajC; preprotein translocase, YajC subunit	OG_02102	LEC3_3514	preprotein translocase, YajC subunit
OG_02103	LG3211_5128	acs; acetate--CoA ligase	OG_02103	LEC3_0157	acetate--CoA ligase
OG_02104	LG3211_4432	LG3211_4432; conserved hypothetical protein	OG_02104	LEC3_4477	UPF0250 protein
OG_02105	LG3211_3332	lpxB; lipid-A-disaccharide synthase	OG_02105	LEC3_3384	lipid-A-disaccharide synthase
OG_02106	LG3211_3938	LG3211_3938; response regulator	OG_02106	LEC3_3962	Type IV pilus assembly protien
OG_02108	LG3211_3278	nth; endonuclease III	OG_02108	LEC3_3311	endonuclease III
OG_02109	LG3211_967	rpmE; ribosomal protein L31	OG_02109	LEC3_0925	ribosomal protein L31
OG_02110	LG3211_2579	LG3211_2579; ompA family protein	OG_02110	LEC3_0433	OmpA/MotB family protein
OG_02111	LG3211_2399	LG3211_2399; conserved hypothetical family protein	OG_02111	LEC3_2344	conserved hypothetical protein
OG_02112	LG3211_2194	LG3211_2194; conserved hypothetical protein	OG_02112	LEC3_2154	conserved hypothetical protein
OG_02113	LG3211_3798	moaB; molybdenum cofactor biosynthesis protein B	OG_02113	LEC3_3820	molybdenum cofactor biosynthesis protein B
OG_02114	LG3211_1309	LG3211_1309; tonB dependent receptor family protein	OG_02114	LEC3_1268	TonB-dependent receptor
OG_02115	LG3211_3801	LG3211_3801; cro/C1-type HTH DNA-binding domain protein	OG_02115	LEC3_3824	HTH type transcriptional regulator
OG_02116	LG3211_3964	recQ; ATP-dependent DNA helicase RecQ	OG_02116	LEC3_3985	ATP-dependent DNA helicase
OG_02117	LG3211_368	LG3211_368; conserved hypothetical protein	OG_02117	LEC3_0185	conserved hypothetical protein
OG_02118	LG3211_2209	nuoK; NADH-quinone oxidoreductase subunit K	OG_02118	LEC3_2169	NADH dehydrogenase, K subunit
OG_02119	LG3211_4002	LG3211_4002; hypothetical protein	OG_02119	LEC3_4067	hypothetical protein
OG_02120	LG3211_1958	LG3211_1958; conserved hypothetical protein	OG_02120	LEC3_1909	conserved hypothetical protein
OG_02121	LG3211_1763	LG3211_1763; phosphoribosyl transferase domain protein	OG_02121	LEC3_1719	hypoxanthine phosphoribosyltransferase
OG_02122	LG3211_4272	LG3211_4272; hypothetical protein	OG_02122	LEC3_4339	hypothetical protein
OG_02123	LG3211_2040	LG3211_2040; eukaryotic-type carbonic anhydrase family protein	OG_02123	LEC3_1975	carbonic anhydrase precusor
OG_02124	LG3211_677	LG3211_677; putative transmembrane protein	OG_02124	LEC3_4672	hypothetical protein
OG_02125	LG3211_385	LG3211_385; polysaccharide biosynthesis family protein	OG_02125	LEC3_0204	NAD(P)H steroid dehydrogenase

OG_02126	LG3211_2188	trpA; tryptophan synthase, alpha subunit	OG_02126	LEC3_2148	tryptophan synthase, alpha subunit
OG_02127	LG3211_5363	LG3211_5363; oxidoreductase NAD-binding domain protein	OG_02127	LEC3_5356	phenol hydroxylase
OG_02128	LG3211_2294	LG3211_2294; hypothetical protein	OG_02128	LEC3_2221	hypothetical protein
OG_02129	LG3211_745	LG3211_745; conserved hypothetical protein	OG_02129	LEC3_0709	hypothetical protein
OG_02130	LG3211_2701	LG3211_2701; slyX family protein	OG_02130	LEC3_2702	slyX family protein
OG_02131	LG3211_3229	rimM; 16S rRNA processing protein RimM	OG_02131	LEC3_3254	16S rRNA processing protein RimM
OG_02132	LG3211_383	LG3211_383; alpha/beta hydrolase fold family protein	OG_02132	LEC3_0201	haloalkane dehalogenase
OG_02133	LG3211_4012	LG3211_4012; bacterial regulatory helix-turn-helix, AraC family protei	OG_02133	LEC3_4080	transcriptional regulator, AraC family
OG_02134	LG3211_5044	LG3211_5044; beta-lactamase family protein	OG_02134	LEC3_4956	beta-lactamase
OG_02135	LG3211_4335	LG3211_4335; amidohydrolase family protein	OG_02135	LEC3_4381	amidohydrolase family protein
OG_02136	LG3211_2718	LG3211_2718; glyoxalase/Bleomycin resistance /Dioxygenase superf	OG_02136	LEC3_2718	lactoylglutathione lyase
OG_02137	LG3211_3283	pstA; phosphate ABC transporter, permease protein PstA	OG_02137	LEC3_3316	phosphate ABC transporter, permease protein PstA
OG_02138	LG3211_3308	dapE; succinyl-diaminopimelate desuccinylase	OG_02138	LEC3_3349	possibly something SEQUENCING GAP
OG_02139	LG3211_4073	LG3211_4073; cell division FtsQ family protein	OG_02139	LEC3_4128	cell division protein FtsQ
OG_02140	LG3211_3984	tolB; tol-Pal system beta propeller repeat protein TolB	OG_02140	LEC3_4052	Tol-Pal system beta propeller repeat protein TolB
OG_02141	LG3211_3996	LG3211_3996; DNA-binding regulatory , YebC/PmpR family protein	OG_02141	LEC3_4063	conserved hypothetical protein
OG_02142	LG3211_313	arsC; arsenate reductase	OG_02142	LEC3_3689	arsenate reductase
OG_02143	LG3211_3140	LG3211_3140; conserved hypothetical protein	OG_02143	LEC3_3145	conserved hypothetical protein
OG_02144	LG3211_975	LG3211_975; fimbrial assembly family protein	OG_02144	LEC3_0933	Type IV pilus assembly protein
OG_02145	LG3211_4405	LG3211_4405; conserved hypothetical protein	OG_02145	LEC3_4452	conserved hypothetical protein
OG_02146	LG3211_580	LG3211_580; conserved hypothetical protein	OG_02146	LEC3_4785	conserved hypothetical protein
OG_02147	LG3211_3657	rfbB; dTDP-glucose 4,6-dehydratase	OG_02147	LEC3_3643	dTDP-glucose 4,6-dehydratase
OG_02148	LG3211_1869	efp; translation elongation factor P	OG_02148	LEC3_1814	translation elongation factor P
OG_02149	LG3211_2830	metX; homoserine O-acetyltransferase	OG_02149	LEC3_2836	homoserine O-acetyltransferase
OG_02150	LG3211_4921	LG3211_4921; snoaL-like domain protein	OG_02150	LEC3_0373	conserved hypothetical protein
OG_02151	LG3211_1465	LG3211_1465; transcriptional regulator PadR-like family protein	OG_02151	LEC3_1432	transcriptional regulator, PadR family
OG_02152	LG3211_3157	LG3211_3157; conserved hypothetical protein	OG_02152	LEC3_3164	conserved hypothetical protein
OG_02153	LG3211_505	LG3211_505; vanZ like family protein	OG_02153	LEC3_0317	VanZ like family
OG_02154	LG3211_351	LG3211_351; tonB dependent receptor family protein	OG_02154	LEC3_5022	TonB-dependent receptor
OG_02155	LG3211_550	LG3211_550; conserved hypothetical protein	OG_02155	LEC3_4832	conserved hypothetical protein
OG_02156	LG3211_3261	rplU; ribosomal protein L21	OG_02156	LEC3_3293	ribosomal protein L21
OG_02157	LG3211_503	LG3211_503; conserved hypothetical protein	OG_02157	LEC3_0314	hypothetical protein
OG_02158	LG3211_4436	LG3211_4436; hypothetical protein	OG_02158	LEC3_4480	hypothetical protein
OG_02159	LG3211_638	LG3211_638; aldo/keto reductase family protein	OG_02159	LEC3_4711	oxidoreductase, aldo/keto reductase family
OG_02160	LG3211_216	LG3211_216; flagellar biosynthesis , FliO family protein	OG_02160	LEC3_5114	type III secretion protein; FliO like
OG_02161	LG3211_807	LG3211_807; cytidylyltransferase family protein	OG_02161	LEC3_0758	cytidylyltransferase family
OG_02162	LG3211_3369	pip; prolyl aminopeptidase	OG_02162	LEC3_3423	proline iminopeptidase
OG_02163	LG3211_3606	LG3211_3606; cytochrome C1 family protein	OG_02163	LEC3_3599	cytochrome C1 family protein
OG_02164	LG3211_1286	LG3211_1286; hutD family protein	OG_02164	LEC3_1242	conserved hypothetical protein
OG_02165	LG3211_92	recB; exodeoxyribonuclease V, beta subunit	OG_02165	LEC3_5231	exodeoxyribonuclease V, beta subunit
OG_02166	LG3211_3474	LG3211_3474; putative secreted protein	OG_02166	LEC3_3531	lipoprotein
OG_02167	LG3211_3271	LG3211_3271; PBP superfamily domain protein	OG_02167	LEC3_3303	OmpA family
OG_02168	LG3211_1341	LG3211_1341; conserved hypothetical protein	OG_02168	LEC3_1294	conserved hypothetical protein
OG_02169	LG3211_3903	LG3211_3903; repair family protein	OG_02169	LEC3_3922	conserved hypothetical protein
OG_02170	LG3211_1529	LG3211_1529; CRS1 / YbhY domain protein	OG_02170	LEC3_1505	conserved hypothetical protein
OG_02171	LG3211_4094	thiL; thiamine-monophosphate kinase	OG_02171	LEC3_4149	thiamine-monophosphate kinase
OG_02172	LG3211_3006	LG3211_3006; hypothetical protein	OG_02172	LEC3_3000	hypothetical protein
OG_02173	LG3211_80	LG3211_80; hypothetical protein	OG_02173	LEC3_5242	hypothetical protein
OG_02174	LG3211_1038	LG3211_1038; citrate transporter family protein	OG_02174	LEC3_1004	citrate transporter
OG_02175	LG3211_2742	LG3211_2742; conserved hypothetical protein	OG_02175	LEC3_2746	conserved hypothetical protein
OG_02176	LG3211_223	LG3211_223; conserved hypothetical protein	OG_02176	LEC3_5107	hypothetical protein
OG_02177	LG3211_5126	LG3211_5126; bacterial regulatory, luxR family protein	OG_02177	LEC3_0159	transcriptional regulator, LuxR family
OG_02178	LG3211_4132	LG3211_4132; putative permease YjgP/YjgQ family protein	OG_02178	LEC3_4192	putative permease, YjgP/YjgQ family
OG_02179	LG3211_680	LG3211_680; sigma-70 region 2 family protein	OG_02179	LEC3_4669	RNA polymerase sigma factor, sigma-70 family
OG_02180	LG3211_2792	LG3211_2792; hypothetical protein	OG_02180	LEC3_2799	conserved hypothetical protein
OG_02181	LG3211_3435	LG3211_3435; 6-O-methylguanine DNA methyltransferase, DNA bind	OG_02181	LEC3_3498	6-O-methylguanine DNA methyltransferase, DNA binding domain prot
OG_02182	LG3211_2461	wspR; response regulator	OG_02182	LEC3_2447	WspR
OG_02183	LG3211_1037	LG3211_1037; rieske [2Fe-2S] domain protein	OG_02183	LEC3_1003	iron-sulfur cluster-binding protein, Rieske family
OG_02184	LG3211_2820	LG3211_2820; conserved hypothetical protein	OG_02184	LEC3_2825	conserved hypothetical protein
OG_02185	LG3211_4427	LG3211_4427; asnC family protein	OG_02185	LEC3_4470	AsnC family
OG_02186	LG3211_813	LG3211_813; conserved hypothetical protein	OG_02186	LEC3_0764	conserved hypothetical protein
OG_02187	LG3211_2655	LG3211_2655; short chain dehydrogenase family protein	OG_02187	LEC3_2660	oxidoreductase, short chain dehydrogenase/reductase family protein
OG_02188	LG3211_115	LG3211_115; conserved hypothetical protein	OG_02188	LEC3_2443	FAD dependent oxidoreductase
OG_02189	LG3211_2784	scpB; segregation and condensation protein B	OG_02189	LEC3_2791	segregation and condensation protein B
OG_02190	LG3211_1133	LG3211_1133; glycosyl transferase 2 family protein	OG_02190	LEC3_1110	glycosyl transferase, group 2 family protein
OG_02191	LG3211_3391	LG3211_3391; conserved hypothetical protein	OG_02191	LEC3_3446	membrane protein
OG_02192	LG3211_1095	LG3211_1095; SPFH domain / Band 7 family protein	OG_02192	LEC3_1069	inner membrane protein
OG_02193	LG3211_350	LG3211_350; ABC-2 transporter family protein	OG_02193	LEC3_5023	membrane protein
OG_02194	LG3211_3167	panC; pantoate--beta-alanine ligase	OG_02194	LEC3_3174	pantoate--beta-alanine ligase
OG_02195	LG3211_4235	LG3211_4235; NAD dependent epimerase/dehydratase family protei	OG_02195	LEC3_4298	NAD-dependent epimerase/dehydratase
OG_02196	LG3211_1272	LG3211_1272; phosphotransferase enzyme family protein	OG_02196	LEC3_1228	phosphotransferase family protein
OG_02197	LG3211_4544	LG3211_4544; transglutaminase-like superfamily protein	OG_02197	LEC3_4599	transglutaminase-like superfamily
OG_02198	LG3211_1220	LG3211_1220; conserved hypothetical protein	OG_02198	LEC3_1174	membrane protein
OG_02199	LG3211_4499	LG3211_4499; response regulator	OG_02199	LEC3_4553	alginate biosynthesis regulatory protein
OG_02200	LG3211_5376	trmE; tRNA modification GTPase TrmE	OG_02200	LEC3_5344	tRNA modification GTPase TrmE
OG_02201	LG3211_765	LG3211_765; putative membrane protein	OG_02201	LEC3_0727	conserved hypothetical protein
OG_02202	LG3211_947	LG3211_947; pilZ domain protein	OG_02202	LEC3_0901	type IV pilus assembly protein PilZ
OG_02203	LG3211_1507	eno; phosphopyruvate hydratase	OG_02203	LEC3_1488	phosphopyruvate hydratase
OG_02204	LG3211_465	LG3211_465; peptidase M23 family protein	OG_02204	LEC3_0273	peptidase, family M23/M37 domain protein
OG_02205	LG3211_3443	LG3211_3443; ABC transporter, phosphonate, periplasmic substrate-	OG_02205	LEC3_3506	conserved hypothetical protein
OG_02206	LG3211_5321	LG3211_5321; efflux transporter, RND family, MFP subunit	OG_02206	LEC3_5413	HlyD secretion family protein

OG_02207	LG3211_747	LG3211_747; conserved hypothetical protein	OG_02207	LEC3_0711	hypothetical protein
OG_02208	LG3211_1429	LG3211_1429; transcription elongation factor, GreA/GreB, C-term fan	OG_02208	LEC3_1396	transcription elongation factor GreB
OG_02209	LG3211_956	rpoZ; DNA-directed RNA polymerase, omega subunit	OG_02209	LEC3_0911	DNA-directed RNA polymerase, omega subunit
OG_02210	LG3211_2258	LG3211_2258; conserved hypothetical protein	OG_02210	LEC3_2211	hypothetical protein
OG_02211	LG3211_717	atpG; ATP synthase F1, gamma subunit	OG_02211	LEC3_0688	ATP synthase F1, gamma subunit
OG_02212	LG3211_1956	LG3211_1956; bacteriocin-protection, YdeI/OmpD-Associated family	OG_02212	LEC3_1906	conserved hypothetical protein
OG_02213	LG3211_99	LG3211_99; efflux transporter, outer membrane factor (OMF) lipo, No	OG_02213	LEC3_5224	metal ion efflux RND protein family
OG_02214	LG3211_4515	LG3211_4515; conserved hypothetical protein	OG_02214	LEC3_4571	carboxymuconolactone decarboxylase family protein
OG_02215	LG3211_1738	acpP; acyl carrier protein	OG_02215	LEC3_1693	acyl carrier protein
OG_02216	LG3211_1862	phaR; polyhydroxyalkanoate synthesis repressor PhaR	OG_02216	LEC3_1807	polyhydroxyalkanoate synthesis repressor PhaR
OG_02217	LG3211_4244	LG3211_4244; H+ symporter) family protein	OG_02217	LEC3_4308	amino acid/peptide transporter (Peptide:H+ symporter)
OG_02218	LG3211_343	LG3211_343; outer membrane beta-barrel domain protein	OG_02218	LEC3_5029	conserved hypothetical protein
OG_02219	LG3211_472	LG3211_472; conserved hypothetical protein	OG_02219	LEC3_0284	lipoprotein
OG_02220	LG3211_4917	LG3211_4917; bacterial regulatory, lacI family protein	OG_02220	LEC3_0377	transcriptional regulator, lacI family
OG_02221	LG3211_5030	LG3211_5030; conserved hypothetical protein	OG_02221	LEC3_4941	lipoprotein
OG_02222	LG3211_4028	clpB; ATP-dependent chaperone protein ClpB	OG_02222	LEC3_4098	ATP-dependent chaperone ClpB
OG_02223	LG3211_120	LG3211_120; catalase family protein	OG_02223	LEC3_5204	catalase
OG_02224	LG3211_4736	LG3211_4736; putative transcriptional regulator	OG_02224	LEC3_0525	transcriptional regulator, TetR family
OG_02225	LG3211_1477	LG3211_1477; peptidase M16 inactive domain protein	OG_02225	LEC3_1447	peptidase, M16 family
OG_02226	LG3211_3141	LG3211_3141; hypothetical protein	OG_02226	LEC3_3149	conserved hypothetical protein
OG_02227	LG3211_3168	panB; 3-methyl-2-oxobutanoate hydroxymethyltransferase	OG_02227	LEC3_3176	3-methyl-2-oxobutanoate hydroxymethyltransferase
OG_02228	LG3211_2768	zwf; glucose-6-phosphate dehydrogenase	OG_02228	LEC3_2775	glucose-6-phosphate 1-dehydrogenase
OG_02229	LG3211_2190	glmM; phosphoglucosamine mutase	OG_02229	LEC3_2150	phosphoglucosamine mutase
OG_02230	LG3211_398	aceB; malate synthase A	OG_02230	LEC3_0222	malate synthase A
OG_02231	LG3211_884	LG3211_884; conserved hypothetical protein	OG_02231	LEC3_0834	conserved hypothetical protein
OG_02232	LG3211_2027	LG3211_2027; hypothetical protein	OG_02232	LEC3_1959	hypothetical protein
OG_02233	LG3211_3889	LG3211_3889; histone deacetylase domain protein	OG_02233	LEC3_3908	histone deacetylase family
OG_02234	LG3211_2794	LG3211_2794; putative peptidoglycan binding domain protein	OG_02234	LEC3_2801	M23 peptidase/putative peptidoglycan binding domain protein
OG_02235	LG3211_2265	mmsA; methylmalonate-semialdehyde dehydrogenase	OG_02235	LEC3_0575	methylmalonate-semialdehyde dehydrogenase (acylating)
OG_02236	LG3211_213	epaO; type III secretion apparatus protein, YscQ/HrcQ family	OG_02236	LEC3_5119	type III secretion apparatus protein
OG_02237	LG3211_1092	LG3211_1092; PDZ domain family protein	OG_02237	LEC3_1066	peptidase
OG_02238	LG3211_3077	LG3211_3077; hypothetical protein	OG_02238	LEC3_3069	hypothetical protein
OG_02239	LG3211_4350	LG3211_4350; 2Fe-2S iron-sulfur cluster binding domain protein	OG_02239	LEC3_4392	oxidoreductase
OG_02240	LG3211_3850	LG3211_3850; nitroreductase family protein	OG_02240	LEC3_3871	conserved hypothetical protein
OG_02241	LG3211_3688	LG3211_3688; alpha/beta hydrolase family protein	OG_02241	LEC3_2111	linear gramicidin dehydrogenase LgrE
OG_02242	LG3211_922	rubA; rubredoxin	OG_02242	LEC3_0875	rubredoxin
OG_02243	LG3211_3052	LG3211_3052; succinate dehydrogenase and fumarate reductase iro	OG_02243	LEC3_3043	succinate dehydrogenase iron-sulfur subunit
OG_02244	LG3211_1647	mfd; transcription-repair coupling factor	OG_02244	LEC3_1619	transcription-repair coupling factor
OG_02245	LG3211_4045	LG3211_4045; bacterial regulatory , Fis family protein	OG_02245	LEC3_4113	Type IV pilus expression regulatory protein
OG_02246	LG3211_3397	LG3211_3397; glucose / Sorbosone dehydrogenase family protein	OG_02246	LEC3_3452	L-sorbosone dehydrogenase
OG_02247	LG3211_364	LG3211_364; conserved hypothetical protein	OG_02247	LEC3_0181	conserved hypothetical protein
OG_02248	LG3211_1226	mgtE; magnesium transporter	OG_02248	LEC3_1182	magnesium transporter
OG_02249	LG3211_2186	LG3211_2186; N-(5'phosphoribosyl)anthranilate (PRA) isomerase far	OG_02249	LEC3_2146	N-(5'phosphoribosyl)anthranilate (PRA) isomerase
OG_02250	LG3211_923	LG3211_923; conserved hypothetical protein	OG_02250	LEC3_0876	conserved hypothetical protein
OG_02251	LG3211_4482	dapF; diaminopimelate epimerase	OG_02251	LEC3_4534	diaminopimelate epimerase
OG_02252	LG3211_484	LG3211_484; SURF1 family protein	OG_02252	LEC3_0292	conserved hypothetical protein
OG_02253	LG3211_4319	csn45; chitosanase	OG_02253	LEC3_4374	beta-glucanase
OG_02254	LG3211_1756	LG3211_1756; response regulator	OG_02254	LEC3_1713	sensor histidine kinase
OG_02255	LG3211_2025	feoB; ferrous iron transport protein B	OG_02255	LEC3_1956	ferrous iron transport protein B
OG_02256	LG3211_1130	LG3211_1130; conserved hypothetical protein	OG_02256	LEC3_1107	conserved hypothetical protein
OG_02257	LG3211_2368	trxB; thioredoxin-disulfide reductase	OG_02257	LEC3_2315	thioredoxin-disulfide reductase
OG_02258	LG3211_5310	LG3211_5310; conserved hypothetical protein	OG_02258	LEC3_5428	conserved hypothetical protein
OG_02259	LG3211_4476	LG3211_4476; conserved hypothetical protein	OG_02259	LEC3_4528	integral membrane protein
OG_02260	LG3211_2933	LG3211_2933; tonB family C-terminal domain protein	OG_02260	LEC3_2946	TonB family C-terminal domain
OG_02261	LG3211_1086	LG3211_1086; aldo/keto reductase family protein	OG_02261	LEC3_1059	oxidoreductase, aldo/keto reductase family
OG_02262	LG3211_5312	LG3211_5312; endonuclease/Exonuclease/phosphatase family prote	OG_02262	LEC3_5425	nuclease
OG_02263	LG3211_370	LG3211_370; PLD-like domain protein	OG_02263	LEC3_0188	conserved hypothetical protein
OG_02264	LG3211_1634	LG3211_1634; MOSC domain protein	OG_02264	LEC3_1603	MOSC domain protein
OG_02265	LG3211_1031	LG3211_1031; RNA 2'-O ribose methyltransferase substrate binding f	OG_02265	LEC3_0996	RNA methyltransferase, TrmH family, group 3
OG_02266	LG3211_3108	LG3211_3108; hypothetical protein	OG_02266	LEC3_3111	conserved hypothetical protein
OG_02267	LG3211_4074	ddlB; ddlB	OG_02267	LEC3_4129	D-alanine-D-alanine ligase
OG_02268	LG3211_1411	LG3211_1411; bacterial regulatory helix-turn-helix , lysR family protei	OG_02268	LEC3_1376	transcriptional regulator, LysR family
OG_02269	LG3211_5090	LG3211_5090; conserved hypothetical protein	OG_02269	LEC3_4998	conserved hypothetical protein
OG_02270	LG3211_1145	LG3211_1145; alpha/beta hydrolase fold family protein	OG_02270	LEC3_1122	alpha/beta hydrolase fold
OG_02271	LG3211_1303	LG3211_1303; feS assembly SUF system regulator	OG_02271	LEC3_1261	FeS assembly SUF system regulator
OG_02272	LG3211_4987	LG3211_4987; patatin-like phospholipase family protein	OG_02272	LEC3_4896	patatin-like phospholipase
OG_02273	LG3211_1457	LG3211_1457; autotransporter beta-domain protein	OG_02273	LEC3_1767	outer membrane autotransporter barrel domain protein
OG_02274	LG3211_3002	acrA1; oxidoreductase, short-chain dehydrogenase/reductase family	OG_02274	LEC3_2995	NAD dependent epimerase/dehydratase family
OG_02275	LG3211_3046	lolD; liporeleasing system, ATP-binding protein	OG_02275	LEC3_3038	lipoprotein releasing system, ATP-binding protein
OG_02276	LG3211_2550	LG3211_2550; pfkB carbohydrate kinase family protein	OG_02276	LEC3_2534	kinase, pfkB family protein
OG_02277	LG3211_2210	LG3211_2210; proton-translocating NADH-quinone oxidoreductase, c	OG_02277	LEC3_2170	proton-translocating NADH-quinone oxidoreductase, chain L family pr
OG_02278	LG3211_2958	LG3211_2958; acetyltransferase family protein	OG_02278	LEC3_4011	acetyltransferase, GNAT family
OG_02279	LG3211_5100	LG3211_5100; aldehyde dehydrogenase family protein	OG_02279	LEC3_5006	aldehyde dehydrogenase (NAD) family protein
OG_02280	LG3211_4678	LG3211_4678; cupin domain protein	OG_02280	LEC3_0566	cupin domain protein
OG_02281	LG3211_3180	LG3211_3180; AAA domain protein	OG_02281	LEC3_3187	PhoH family protein
OG_02282	LG3211_74	LG3211_74; metA-pathway of phenol degradation family protein	OG_02282	LEC3_5278	conserved hypothetical protein
OG_02283	LG3211_954	yicC; conserved protein	OG_02283	LEC3_0909	conserved hypothetical protein
OG_02285	LG3211_814	LG3211_814; conserved hypothetical protein	OG_02285	LEC3_0765	conserved hypothetical protein
OG_02286	LG3211_1925	LG3211_1925; methyltransferase domain protein	OG_02286	LEC3_1875	hypothetical protein
OG_02287	LG3211_4456	LG3211_4456; metallo-beta-lactamase superfamily protein	OG_02287	LEC3_2423	metallo-beta-lactamase superfamily protein
OG_02288	LG3211_1866	LG3211_1866; diguanylate cyclase domain protein	OG_02288	LEC3_1812	sensory box-containing diguanylate cyclase/cyclic diguanylate phospl

OG_02289	LG3211_1282	cysC; adenylylsulfate kinase	OG_02289	LEC3_1238	bifunctional enzyme CysN/cysC
OG_02290	LG3211_2894	LG3211_2894; helix-turn-helix domain protein	OG_02290	LEC3_2908	conserved hypothetical protein
OG_02291	LG3211_3362	lon; ATP-dependent protease La	OG_02291	LEC3_3416	ATP-dependent protease La
OG_02292	LG3211_4552	LG3211_4552; aldehyde dehydrogenase family protein	OG_02292	LEC3_4607	aldehyde dehydrogenase (NAD) family protein
OG_02293	LG3211_850	LG3211_850; ycel-like domain protein	OG_02293	LEC3_0796	Ycel like family
OG_02294	LG3211_3752	rplX; ribosomal protein L24	OG_02294	LEC3_3779	ribosomal protein L24
OG_02295	LG3211_1637	LG3211_1637; methyltransferase domain protein	OG_02295	LEC3_1606	conserved hypothetical protein
OG_02296	LG3211_4322	gnd; 6-phosphogluconate dehydrogenase	OG_02296	LEC3_4375	6-phosphogluconate dehydrogenase (decarboxylating)
OG_02297	LG3211_565	LG3211_565; conserved hypothetical protein	OG_02297	LEC3_4807	conserved hypothetical protein
OG_02298	LG3211_4129	xerD; tyrosine recombinase XerD	OG_02298	LEC3_4189	tyrosine recombinase
OG_02299	LG3211_2406	LG3211_2406; phosphotransferase enzyme family protein	OG_02299	LEC3_2348	Phosphotransferase enzyme family
OG_02300	LG3211_4148	LG3211_4148; conserved hypothetical protein	OG_02300	LEC3_4205	membrane protein
OG_02301	LG3211_1496	ycaQ; conserved protein	OG_02301	LEC3_1472	conserved hypothetical protein
OG_02302	LG3211_5017	LG3211_5017; ycgL domain protein	OG_02302	LEC3_4931	conserved hypothetical protein
OG_02303	LG3211_2307	dnaJ; chaperone protein DnaJ	OG_02303	LEC3_2233	chaperone protein dnaJ
OG_02304	LG3211_46	LG3211_46; qseB transcriptional activator	OG_02304	LEC3_5247	response regulator receiver domain
OG_02305	LG3211_4939	LG3211_4939; lysM domain protein	OG_02305	LEC3_0354	peptidoglycan-binding LysM
OG_02306	LG3211_1752	LG3211_1752; conserved hypothetical protein	OG_02306	LEC3_1709	conserved hypothetical protein
OG_02307	LG3211_585	LG3211_585; phosphate-selective porin O and P family protein	OG_02307	LEC3_4781	phosphate-selective porin O and P
OG_02308	LG3211_353	LG3211_353; pyridoxal-phosphate dependent enzyme family protein	OG_02308	LEC3_5020	pyridoxal-phosphate dependent enzyme
OG_02309	LG3211_5083	aroE; shikimate 5-dehydrogenase	OG_02309	LEC3_4995	shikimate 5-dehydrogenase
OG_02310	LG3211_3888	LG3211_3888; ATP:cob(I)alamin adenosyltransferase, putative	OG_02310	LEC3_3907	ATP:cob(I)alamin adenosyltransferase
OG_02311	LG3211_338	LG3211_338; conserved hypothetical protein	OG_02311	LEC3_5033	lipoprotein
OG_02312	LG3211_4537	ohr; organic hydroperoxide resistance protein	OG_02312	LEC3_4592	OsmC-like protein
OG_02313	LG3211_2621	LG3211_2621; FAD binding domain protein	OG_02313	LEC3_2406	oxidoreductase
OG_02314	LG3211_3258	rpsT; ribosomal protein S20	OG_02314	LEC3_3290	ribosomal protein S20
OG_02315	LG3211_953	rph; ribonuclease PH	OG_02315	LEC3_0908	ribonuclease PH
OG_02316	LG3211_2987	serS; serine--tRNA ligase	OG_02316	LEC3_2978	seryl-tRNA synthetase
OG_02317	LG3211_1126	LG3211_1126; conserved hypothetical protein	OG_02317	LEC3_1102	conserved hypothetical protein
OG_02318	LG3211_4463	mreB; mreB	OG_02318	LEC3_4513	cell shape determining protein, MreB/Mrl family
OG_02319	LG3211_1486	LG3211_1486; conserved hypothetical protein	OG_02319	LEC3_1463	conserved hypothetical protein
OG_02320	LG3211_2999	LG3211_2999; patatin-like phospholipase family protein	OG_02320	LEC3_2991	phospholipase, patatin family
OG_02321	LG3211_849	LG3211_849; nitroreductase family protein	OG_02321	LEC3_0795	nitroreductase family protein
OG_02322	LG3211_977	LG3211_977; pilus assembly , PilP family protein	OG_02322	LEC3_0935	Type IV assembly protein
OG_02323	LG3211_2943	LG3211_2943; bacterial regulatory helix-turn-helix , lysR family protei	OG_02323	LEC3_2960	transcriptional regulator, LysR family
OG_02324	LG3211_4345	LG3211_4345; asnC family protein	OG_02324	LEC3_4388	transcriptional regulator, AsnC family
OG_02325	LG3211_3147	LG3211_3147; RDD family protein	OG_02325	LEC3_3155	RDD family
OG_02326	LG3211_108	LG3211_108; hlyD secretion family protein	OG_02326	LEC3_5216	conserved hypothetical protein
OG_02327	LG3211_3933	LG3211_3933; cheB methylesterase family protein	OG_02327	LEC3_3957	glutamate methyltransferase
OG_02328	LG3211_4027	LG3211_4027; amidohydrolase family protein	OG_02328	LEC3_4096	amidohydrolase family protein
OG_02329	LG3211_4493	LG3211_4493; molydopterin dinucleotide binding domain protein	OG_02329	LEC3_4545	molybdopterin oxidoreductase
OG_02330	LG3211_3458	LG3211_3458; bacterial regulatory, luxR family protein	OG_02330	LEC3_3517	transcriptional regulator, LuxR family
OG_02331	LG3211_2195	LG3211_2195; DNA-deoxyinosine glycosylase	OG_02331	LEC3_2156	conserved hypothetical protein
OG_02332	LG3211_77	LG3211_77; rhomboid family protein	OG_02332	LEC3_5245	peptidase, S54 (rhomboid) family
OG_02333	LG3211_2691	LG3211_2691; methyltransferase domain protein	OG_02333	LEC3_2693	transcriptional regulator, ArsR family
OG_02334	LG3211_47	LG3211_47; his Kinase A domain protein	OG_02334	LEC3_5248	sensor histidine kinase
OG_02335	LG3211_952	LG3211_952; glyoxalase/Bleomycin resistance /Dioxygenase superfa	OG_02335	LEC3_0907	glyoxalase
OG_02336	LG3211_2989	LG3211_2989; tonB family C-terminal domain protein	OG_02336	LEC3_2980	TonB family C-terminal domain
OG_02337	LG3211_1163	LG3211_1163; L-serine ammonia-lyase	OG_02337	LEC3_1135	L-serine ammonia-lyase
OG_02338	LG3211_1114	LG3211_1114; NAD dependent epimerase/dehydratase family protein	OG_02338	LEC3_1089	NAD dependent epimerase/dehydratase family protein
OG_02339	LG3211_3227	rplS; ribosomal protein L19	OG_02339	LEC3_3252	ribosomal protein L19
OG_02340	LG3211_1438	LG3211_1438; tfoX N-terminal domain protein	OG_02340	LEC3_1407	TfoX-like protein
OG_02341	LG3211_5380	rpmH; ribosomal protein L34	OG_02341	LEC3_5340	ribosomal protein L34
OG_02342	LG3211_2116	LG3211_2116; putative translation initiation inhibitor	OG_02342	LEC3_2055	endoribonuclease L-PSP family protein
OG_02343	LG3211_5358	LG3211_5358; hypothetical protein	OG_02343	LEC3_5363	hypothetical protein
OG_02344	LG3211_789	LG3211_789; glutathione S-transferase	OG_02344	LEC3_0418	glutathione S-transferase
OG_02345	LG3211_3523	LG3211_3523; hypothetical protein	OG_02345	LEC3_3555	hypothetical protein
OG_02346	LG3211_855	lpxL; lipid A biosynthesis lauroyl (or palmitoleoyl) acyltransferase fami	OG_02346	LEC3_0801	lipid A biosynthesis lauroyl (or palmitoleoyl) acyltransferase
OG_02347	LG3211_4166	LG3211_4166; electron transfer flavodomain protein	OG_02347	LEC3_4233	electron transfer flavoprotein beta subunit
OG_02348	LG3211_3627	LG3211_3627; conserved hypothetical protein	OG_02348	LEC3_3619	UPF0054 protein
OG_02349	LG3211_1614	LG3211_1614; SPFH domain / Band 7 family protein	OG_02349	LEC3_1588	Spfh domain/band 7 family protein
OG_02350	LG3211_4639	LG3211_4639; hypothetical protein	OG_02350	LEC3_2250	glycosyl transferase
OG_02351	LG3211_3253	ispH; 4-hydroxy-3-methylbut-2-enyl diphosphate reductase	OG_02351	LEC3_3284	4-hydroxy-3-methylbut-2-enyl diphosphate reductase
OG_02352	LG3211_332	LG3211_332; hydrolase CocE/NonD family protein	OG_02352	LEC3_5038	hydrolase, CocE/NonD family
OG_02353	LG3211_2252	LG3211_2252; impB/mucB/samB family protein	OG_02353	LEC3_2205	nucleotidyltransferase/DNA polymerase involved in DNA repair
OG_02354	LG3211_3103	LG3211_3103; conserved hypothetical protein	OG_02354	LEC3_3105	acetyltransferase
OG_02355	LG3211_3102	LG3211_3102; pseudouridine synthase family protein	OG_02355	LEC3_3104	ribosomal large subunit pseudouridine synthase F
OG_02356	LG3211_4015	cobT; nicotinate-nucleotide--dimethylbenzimidazole phosphoribosyltra	OG_02356	LEC3_4083	nicotinate-nucleotide--dimethylbenzimidazole phosphoribosyltransfera
OG_02357	LG3211_1463	LG3211_1463; spermidine n1-acetyltransferase	OG_02357	LEC3_1429	spermidine N(1)-acetyltransferase
OG_02358	LG3211_4326	LG3211_4326; aldo/keto reductase family protein	OG_02358	LEC3_4378	oxidoreductase
OG_02359	LG3211_4249	pdhA; pyruvate dehydrogenase (acetyl-transferring) E1 component, a	OG_02359	LEC3_4316	pyruvate dehydrogenase (acetyl-transferring) E1 component, alpha su
OG_02360	LG3211_1616	purT; phosphoribosylglycinamide formyltransferase 2	OG_02360	LEC3_1590	phosphoribosylglycinamide formyltransferase 2
OG_02361	LG3211_1085	LG3211_1085; hypothetical protein	OG_02361	LEC3_1058	hypothetical protein
OG_02362	LG3211_2708	LG3211_2708; ABC transporter family protein	OG_02362	LEC3_2709	ABC transporter, ATP-binding protein
OG_02363	LG3211_2825	LG3211_2825; ccmE family protein	OG_02363	LEC3_2830	cytochrome c-type biogenesis protein
OG_02364	LG3211_1589	LG3211_1589; triose-phosphate Transporter family protein	OG_02364	LEC3_1565	integral membrane protein DUF6
OG_02365	LG3211_942	LG3211_942; conserved hypothetical protein	OG_02365	LEC3_0898	oxidoreductase, short chain dehydrogenase/reductase family
OG_02366	LG3211_4366	LG3211_4366; peptidase M20/M25/M40 family protein	OG_02366	LEC3_4412	peptidase, M28 family
OG_02367	LG3211_1748	LG3211_1748; anti sigma-E RseA, N-terminal domain protein	OG_02367	LEC3_1704	Anti sigma-E protein
OG_02368	LG3211_871	LG3211_871; pyridoxal-phosphate dependent enzyme family protein	OG_02368	LEC3_0820	threonine dehydratase catabolic
OG_02369	LG3211_1123	LG3211_1123; phosphoenolpyruvate carboxykinase family protein	OG_02369	LEC3_1099	phosphoenolpyruvate carboxykinase

OG_02370	LG3211_3348	LG3211_3348; conserved hypothetical protein	OG_02370	LEC3_3406	conserved hypothetical protein
OG_02371	LG3211_637	LG3211_637; lactonase, 7-bladed beta-propeller family protein	OG_02371	LEC3_4712	conserved hypothetical protein
OG_02372	LG3211_209	LG3211_209; type III secretion , YscU/HrpY family protein	OG_02372	LEC3_5123	type III secretion protein, flhB like
OG_02373	LG3211_4163	LG3211_4163; ATP-grasp domain protein	OG_02373	LEC3_4229	conserved hypothetical protein
OG_02374	LG3211_622	LG3211_622; excalibur calcium-binding domain protein	OG_02374	LEC3_4728	excalibur domain protein
OG_02375	LG3211_2845	LG3211_2845; conserved hypothetical protein	OG_02375	LEC3_2856	conserved hypothetical protein
OG_02376	LG3211_2898	LG3211_2898; bacterial regulatory, tetR family protein	OG_02376	LEC3_2912	transcriptional regulator, TetR family
OG_02377	LG3211_1270	LG3211_1270; RNA 2'-phosphotransferase, Tpt1 / KptA family protein	OG_02377	LEC3_1226	RNA 2'-phosphotransferase
OG_02378	LG3211_51	LG3211_51; RNA polymerase sigma factor, sigma-70 family protein	OG_02378	LEC3_5270	RNA polymerase sigma factor, sigma-70 family
OG_02379	LG3211_5223	LG3211_5223; methyltransferase domain protein	OG_02379	LEC3_0065	biotin biosynthesis protein BioC
OG_02380	LG3211_1951	hisF; imidazole glycerol phosphate synthase, HisF subunit	OG_02380	LEC3_1903	imidazoleglycerol phosphate synthase, cyclase subunit
OG_02381	LG3211_4681	LG3211_4681; isochorismatase family protein	OG_02381	LEC3_1771	isochorismatase family protein
OG_02382	LG3211_4144	pssA; CDP-diacylglycerol-serine O-phosphatidyltransferase	OG_02382	LEC3_4200	CDP-diacylglycerol--serine O-phosphatidyltransferase
OG_02383	LG3211_1665	LG3211_1665; conserved hypothetical protein	OG_02383	LEC3_1627	protein of unknown function DUF519
OG_02384	LG3211_5064	LG3211_5064; putative pteridine-dependent dioxygenase	OG_02384	LEC3_4979	pteridine-dependent deoxygenase like protein
OG_02385	LG3211_2775	LG3211_2775; bacterial extracellular solute-binding family protein	OG_02385	LEC3_2781	bacterial extracellular solute-binding protein
OG_02386	LG3211_661	LG3211_661; bacterial regulatory, tetR family protein	OG_02386	LEC3_4687	bacterial regulatory proteins, tetR family
OG_02387	LG3211_1861	phbB; acetoacetyl-CoA reductase family protein	OG_02387	LEC3_1806	acetoacetyl-CoA reductase
OG_02389	LG3211_1858	rnd; ribonuclease D	OG_02389	LEC3_1802	ribonuclease D
OG_02390	LG3211_2337	lysS; lysine--tRNA ligase	OG_02390	LEC3_2278	lysyl-tRNA synthetase
OG_02391	LG3211_4007	LG3211_4007; ABC transporter family protein	OG_02391	LEC3_4074	ABC transporter, ATP-binding protein
OG_02392	LG3211_1585	LG3211_1585; conserved hypothetical protein	OG_02392	LEC3_1562	conserved hypothetical protein
OG_02393	LG3211_2458	LG3211_2458; cheW-like domain protein	OG_02393	LEC3_2450	CheW domain protein
OG_02394	LG3211_2895	pilF; type IV pilus biogenesis/stability protein PilW	OG_02394	LEC3_2909	type IV pilus assembly protein
OG_02395	LG3211_5232	LG3211_5232; C4-dicarboxylate anaerobic carrier family protein	OG_02395	LEC3_0058	short chain fatty acid transporter, putative
OG_02396	LG3211_3985	LG3211_3985; hypothetical protein	OG_02396	LEC3_4053	protein TolA-like
OG_02397	LG3211_2854	LG3211_2854; citrate transporter family protein	OG_02397	LEC3_2866	citrate transporter
OG_02398	LG3211_4500	LG3211_4500; histidine kinase family protein	OG_02398	LEC3_4554	two-component system sensor protein
OG_02399	LG3211_1233	LG3211_1233; putative P-loop containing ATPase	OG_02399	LEC3_1188	UP0042 protein
OG_02400	LG3211_3919	mazG; nucleoside triphosphate pyrophosphohydrolase	OG_02400	LEC3_3943	MazG family protein
OG_02401	LG3211_3537	rplT; ribosomal protein L20	OG_02401	LEC3_3567	ribosomal protein L20
OG_02402	LG3211_888	LG3211_888; bacterial regulatory helix-turn-helix, AraC family protein	OG_02402	LEC3_0838	transcriptional regulator, AraC family
OG_02403	LG3211_920	hemL; glutamate-1-semialdehyde-2,1-aminomutase	OG_02403	LEC3_0873	glutamate-1-semialdehyde-2,1-aminomutase
OG_02404	LG3211_3821	lieA; leupeptin-inactivating enzyme 1	OG_02404	LEC3_3845	PKD domain/proprotein convertase P-domain protein
OG_02405	LG3211_3875	LG3211_3875; eamA-like transporter family protein	OG_02405	LEC3_3893	integral membrane protein DUF6
OG_02406	LG3211_3128	LG3211_3128; helicase conserved C-terminal domain protein	OG_02406	LEC3_3131	ATP-dependent RNA helicase
OG_02407	LG3211_718	atpD; ATP synthase F1, beta subunit	OG_02407	LEC3_0689	ATP synthase F1, beta subunit
OG_02408	LG3211_2851	LG3211_2851; bacterial regulatory, luxR family protein	OG_02408	LEC3_2862	two component transcriptional regulator, LuxR family
OG_02409	LG3211_176	LG3211_176; acyltransferase family protein	OG_02409	LEC3_5154	acetyltransferase
OG_02410	LG3211_1385	LG3211_1385; tonB dependent receptor family protein	OG_02410	LEC3_1353	TonB-dependent receptor
OG_02411	LG3211_757	queD; queuosine biosynthesis protein QueD	OG_02411	LEC3_0720	queuosine biosynthesis protein QueD
OG_02412	LG3211_1721	LG3211_1721; hypothetical protein	OG_02412	LEC3_1677	hypothetical protein
OG_02413	LG3211_372	LG3211_372; conserved hypothetical protein	OG_02413	LEC3_0190	conserved hypothetical protein
OG_02414	LG3211_1237	rpoN; RNA polymerase sigma-54 factor	OG_02414	LEC3_1192	RNA polymerase sigma-54 factor
OG_02415	LG3211_3947	LG3211_3947; glyoxalase-like domain protein	OG_02415	LEC3_3969	glyoxalase/bleomycin resistance protein/dioxygenase
OG_02416	LG3211_869	leuA; 2-isopropylmalate synthase	OG_02416	LEC3_0819	2-isopropylmalate synthase
OG_02417	LG3211_3349	cafA; ribonuclease G	OG_02417	LEC3_3407	ribonuclease G
OG_02419	LG3211_396	LG3211_396; bacterial regulatory helix-turn-helix , lysR family protein	OG_02419	LEC3_0221	transcriptional regulator, LysR family
OG_02420	LG3211_4267	LG3211_4267; sporulation related domain protein	OG_02420	LEC3_4334	sporulation and cell division repeat protein
OG_02421	LG3211_5218	gltD; glutamate synthase (NADPH) small chain glutamate synthase, s	OG_02421	LEC3_0070	glutamate synthase, small subunit
OG_02422	LG3211_437	argS; arginine--tRNA ligase	OG_02422	LEC3_0249	arginyl-tRNA synthetase
OG_02423	LG3211_4062	LG3211_4062; type II secretion system (T2SS), F family protein	OG_02423	LEC3_4117	Type IV pilus assembly protein
OG_02424	LG3211_2026	LG3211_2026; conserved hypothetical protein	OG_02424	LEC3_1957	conserved hypothetical protein
OG_02425	LG3211_497	yihY; yihY family inner membrane domain protein	OG_02425	LEC3_0307	ribonuclease BN
OG_02426	LG3211_2208	LG3211_2208; NADH-ubiquinone/plastoquinone oxidoreductase chai	OG_02426	LEC3_2168	NADH dehydrogenase, J subunit
OG_02427	LG3211_2681	LG3211_2681; conserved hypothetical protein	OG_02427	LEC3_2684	conserved hypothetical protein
OG_02428	LG3211_1063	ahcY; adenosylhomocysteinase	OG_02428	LEC3_1033	adenosylhomocysteinase
OG_02429	LG3211_2212	LG3211_2212; proton-translocating NADH-quinone oxidoreductase, c	OG_02429	LEC3_2172	proton-translocating NADH-quinone oxidoreductase, chain N family p
OG_02430	LG3211_53	LG3211_53; bacterial regulatory, luxR family protein	OG_02430	LEC3_5268	transcriptional regulator, LuxR family
OG_02431	LG3211_2088	LG3211_2088; conserved hypothetical protein	OG_02431	LEC3_2028	conserved hypothetical protein
OG_02432	LG3211_3982	ygbF; tol-pal system protein YbgF	OG_02432	LEC3_4050	tol-pal system protein YbgF
OG_02433	LG3211_4516	LG3211_4516; S-adenosyl methyltransferase family protein	OG_02433	LEC3_4572	tetracenomycin polyketide synthesis O-methyltransferase TcmP
OG_02434	LG3211_207	LG3211_207; hypothetical protein	OG_02434	LEC3_5125	hypothetical protein
OG_02435	LG3211_1874	LG3211_1874; conserved hypothetical protein	OG_02435	LEC3_1819	conserved hypothetical protein
OG_02436	LG3211_4124	LG3211_4124; type II secretion system (T2SS), F family protein	OG_02436	LEC3_4181	general secretion pathway protein F
OG_02437	LG3211_5347	LG3211_5347; methyltransferase domain protein	OG_02437	LEC3_5374	methyltransferase type 11
OG_02438	LG3211_5137	LG3211_5137; hypothetical protein	OG_02438	LEC3_0148	hypothetical protein
OG_02439	LG3211_477	LG3211_477; delta-1-pyrroline-5-carboxylate dehydrogenase	OG_02439	LEC3_0285	proline dehydrogenase
OG_02440	LG3211_4097	ribB; 3,4-dihydroxy-2-butanone-4-phosphate synthase	OG_02440	LEC3_4152	3,4-dihydroxy-2-butanone 4-phosphate synthase
OG_02441	LG3211_1724	LG3211_1724; outer membrane lipoSlp family protein	OG_02441	LEC3_1680	outer membrane lipoprotein, Slp family
OG_02442	LG3211_3946	LG3211_3946; putative peptidase	OG_02442	LEC3_3968	glycoprotease family
OG_02443	LG3211_23	LG3211_23; thermolysin metallopeptidase, catalytic domain protein	OG_02443	LEC3_5314	fungalysin/thermolysin propeptide/thermolysin metallopeptidase doma
OG_02444	LG3211_563	LG3211_563; EDD, DegV family domain protein	OG_02444	LEC3_4809	DegV family protein
OG_02445	LG3211_4425	LG3211_4425; eamA-like transporter family protein	OG_02445	LEC3_4468	integral membrane protein DUF6
OG_02446	LG3211_341	gpsA; glycerol-3-phosphate dehydrogenase [NAD(P)+]	OG_02446	LEC3_5030	NAD-dependent glycerol-3-phosphate dehydrogenase
OG_02447	LG3211_1543	NQO2; ribosyldihydronicotinamide dehydrogenase [quinone]	OG_02447	LEC3_1517	flavodoxin-like fold family protein
OG_02448	LG3211_651	LG3211_651; major Facilitator Superfamily protein	OG_02448	LEC3_4697	transporter, major facilitator family
OG_02449	LG3211_4276	LG3211_4276; conserved hypothetical protein	OG_02449	LEC3_4343	conserved hypothetical protein
OG_02450	LG3211_4189	LG3211_4189; glycosyl transferases group 1 family protein	OG_02450	LEC3_4247	glycosyl transferase, group 1 family protein
OG_02451	LG3211_3725	LG3211_3725; cyclophilin type peptidyl-prolyl cis-trans isomerase/CL	OG_02451	LEC3_3751	peptidyl-prolyl cis-trans isomerase, cyclophilin-type
OG_02452	LG3211_1571	LG3211_1571; glyoxalase/Bleomycin resistance /Dioxygenase superf	OG_02452	LEC3_1548	glyoxalase/bleomycin resistance protein/dioxygenase

OG_02453	LG3211_1793	LG3211_1793; eamA-like transporter family protein	OG_02453	LEC3_1756	Integral membrane protein DUF6
OG_02454	LG3211_2063	LG3211_2063; hypothetical protein	OG_02454	LEC3_2000	hypothetical protein
OG_02455	LG3211_4976	LG3211_4976; conserved hypothetical protein	OG_02455	LEC3_4887	Aha1 domain superfamily
OG_02456	LG3211_2903	csaA; secretion chaperone	OG_02456	LEC3_2916	export-related chaperone CsaA
OG_02457	LG3211_2141	LG3211_2141; acetyltransferase family protein	OG_02457	LEC3_2082	acetyltransferase (GNAT) family
OG_02458	LG3211_21	LG3211_21; bacterial regulatory helix-turn-helix , lysR family protein	OG_02458	LEC3_5317	transcriptional regulator, LysR family
OG_02459	LG3211_4277	LG3211_4277; amidinotransferase family protein	OG_02459	LEC3_4344	Amidinotransferase
OG_02460	LG3211_1518	LG3211_1518; lysM domain protein	OG_02460	LEC3_1499	outer membrane antigenic lipoprotein
OG_02461	LG3211_2620	LG3211_2620; conserved hypothetical protein	OG_02461	LEC3_2631	conserved hypothetical protein
OG_02462	LG3211_2358	clpS; ATP-dependent Clp protease adapter protein ClpS	OG_02462	LEC3_2304	ATP-dependent Clp protease adaptor protein ClpS
OG_02463	LG3211_3905	LG3211_3905; lemA family protein	OG_02463	LEC3_3926	LemA family protein
OG_02464	LG3211_3200	LG3211_3200; competence/damage-inducible CinA C-terminal doma	OG_02464	LEC3_3207	competence/damage-inducible protein CinA
OG_02465	LG3211_3289	LG3211_3289; conserved hypothetical protein	OG_02465	LEC3_3321	UPF0276 protein
OG_02466	LG3211_5224	bioH; pimelyl-[acyl-carrier protein] methyl ester esterase	OG_02466	LEC3_0063	putative pimeloyl-BioC--CoA transferase BioH
OG_02467	LG3211_4443	LG3211_4443; conserved hypothetical protein	OG_02467	LEC3_4484	conserved hypothetical protein
OG_02468	LG3211_3054	sdhD; succinate dehydrogenase, hydrophobic membrane anchor prot	OG_02468	LEC3_3045	succinate dehydrogenase, hydrophobic membrane anchor protein
OG_02469	LG3211_518	LG3211_518; endonuclease/Exonuclease/phosphatase family protein	OG_02469	LEC3_0331	endonuclease/exonuclease/phosphatase family protein
OG_02470	LG3211_777	LG3211_777; mechanosensitive ion channel family protein	OG_02470	LEC3_0738	conserved hypothetical protein
OG_02471	LG3211_951	rdgB; non-canonical purine NTP pyrophosphatase, RdgB/HAM1 famil	OG_02471	LEC3_0906	non-canonical purine NTP pyrophosphatase, RdgB/HAM1 family
OG_02472	LG3211_3436	LG3211_3436; hypothetical protein	OG_02472	LEC3_3499	hypothetical protein
OG_02473	LG3211_2715	purB; adenylosuccinate lyase	OG_02473	LEC3_2716	adenylosuccinate lyase
OG_02474	LG3211_3275	LG3211_3275; FKBP-type peptidyl-prolyl cis-trans isomerase family p	OG_02474	LEC3_3308	domain amino terminal to FKBP-type peptidyl-prolyl isomerase/peptid
OG_02475	LG3211_4636	LG3211_4636; bacterial transferase hexapeptide family protein	OG_02475	LEC3_2252	nodulation protein L
OG_02476	LG3211_5308	LG3211_5308; ion channel family protein	OG_02476	LEC3_5432	ion channel
OG_02477	LG3211_191	LG3211_191; conserved hypothetical family protein	OG_02477	LEC3_5140	conserved hypothetical protein
OG_02478	LG3211_485	LG3211_485; conserved hypothetical protein	OG_02478	LEC3_0293	conserved hypothetical protein
OG_02479	LG3211_4982	LG3211_4982; TPR repeat family protein	OG_02479	LEC3_4891	tetratricopeptide repeat domain protein
OG_02480	LG3211_3357	LG3211_3357; PPIC-type PPIASE domain protein	OG_02480	LEC3_3413	PPIC-type PPIASE domain
OG_02481	LG3211_2744	LG3211_2744; ycel-like domain protein	OG_02481	LEC3_2747	Ycel like family
OG_02482	LG3211_4942	LG3211_4942; pilin family protein	OG_02482	LEC3_0351	fimbrial protein
OG_02483	LG3211_4471	LG3211_4471; short chain dehydrogenase family protein	OG_02483	LEC3_4521	oxidoreductase, short chain dehydrogenase/reductase family
OG_02484	LG3211_2184	fimV; fimV N-terminal domain	OG_02484	LEC3_2144	Type IV fimbrial biogenesis protein
OG_02485	LG3211_2181	aroC; chorismate synthase	OG_02485	LEC3_2140	chorismate synthase
OG_02486	LG3211_2131	LG3211_2131; PLD-like domain protein	OG_02486	LEC3_2073	phospholipase D/transphosphatidylase
OG_02487	LG3211_2745	gyrA; DNA gyrase, A subunit	OG_02487	LEC3_2749	DNA gyrase, A subunit
OG_02488	LG3211_1570	gcvP; glycine dehydrogenase	OG_02488	LEC3_1547	glycine dehydrogenase
OG_02489	LG3211_648	LG3211_648; condensation domain protein	OG_02489	LEC3_4699	peptide synthetase
OG_02490	LG3211_3878	LG3211_3878; cytidine and deoxycytidylate deaminase zinc-binding r	OG_02490	LEC3_3896	cytidine and deoxycytidylate deaminase zinc-binding domain protein
OG_02491	LG3211_1682	phoB; phosphate regulon transcriptional regulatory protein PhoB	OG_02491	LEC3_1649	phosphate regulon transcriptional regulatory protein PhoB
OG_02492	LG3211_1372	folC; FOLC	OG_02492	LEC3_1336	folylpolyglutamate synthase/dihydrofolate synthase
OG_02493	LG3211_2724	LG3211_2724; putative lysine decarboxylase family protein	OG_02493	LEC3_2726	possible lysine decarboxylase
OG_02494	LG3211_212	epaP; type III secretion apparatus protein, YscR/HrcR family	OG_02494	LEC3_5120	type III secretion apparatus protein
OG_02495	LG3211_2371	LG3211_2371; lumazine-binding family protein	OG_02495	LEC3_5280	conserved hypothetical protein
OG_02496	LG3211_3643	corA; magnesium and cobalt transport protein CorA	OG_02496	LEC3_3631	magnesium and cobalt transport protein corA
OG_02497	LG3211_2728	LG3211_2728; dnaJ C terminal domain protein	OG_02497	LEC3_2731	dnaJ domain
OG_02499	LG3211_4304	LG3211_4304; conserved hypothetical protein	OG_02499	LEC3_4363	conserved hypothetical protein
OG_02500	LG3211_4023	LG3211_4023; putative transmembrane protein	OG_02500	LEC3_4091	membrane protein
OG_02501	LG3211_4478	hslU; ATP-dependent protease HslVU, ATPase subunit	OG_02501	LEC3_4530	heat shock protein HslVU, ATPase subunit HslU
OG_02502	LG3211_4113	LG3211_4113; glycosyl transferase 2 family protein	OG_02502	LEC3_4170	glycosyl transferase, group 2 family protein
OG_02503	LG3211_3751	LG3211_3751; ribosomal L5 family protein	OG_02503	LEC3_3778	ribosomal protein L5
OG_02504	LG3211_5189	LG3211_5189; HAD super, subIIIB family protein	OG_02504	LEC3_0098	5'-nucleotidase, lipoprotein e(P4) family
OG_02505	LG3211_3181	thiD; phosphomethylpyrimidine kinase	OG_02505	LEC3_3189	phosphomethylpyrimidine kinase
OG_02506	LG3211_1369	serA; phosphoglycerate dehydrogenase	OG_02506	LEC3_1332	phosphoglycerate dehydrogenase
OG_02507	LG3211_2771	LG3211_2771; F5/8 type C domain protein	OG_02507	LEC3_2778	F5/8 type C domain
OG_02508	LG3211_2313	carA; carbamoyl-phosphate synthase, small subunit	OG_02508	LEC3_2237	carbamoyl-phosphate synthase, small subunit
OG_02509	LG3211_3950	mrcB; penicillin-binding protein 1B	OG_02509	LEC3_3972	penicillin-binding protein 1B
OG_02510	LG3211_904	LG3211_904; conserved hypothetical protein	OG_02510	LEC3_0851	conserved hypothetical protein
OG_02511	LG3211_4554	LG3211_4554; FAD binding domain protein	OG_02511	LEC3_4609	pyridine nucleotide-disulphide oxidoreductase
OG_02512	LG3211_5047	LG3211_5047; conserved hypothetical protein	OG_02512	LEC3_4966	lipoprotein
OG_02513	LG3211_720	LG3211_720; gtrA-like family protein	OG_02513	LEC3_0691	conserved hypothetical protein
OG_02514	LG3211_2176	LG3211_2176; conserved hypothetical protein	OG_02514	LEC3_2136	lipoprotein
OG_02515	LG3211_1446	LG3211_1446; conserved hypothetical protein	OG_02515	LEC3_1414	conserved hypothetical protein
OG_02516	LG3211_1211	LG3211_1211; twitching motility family protein	OG_02516	LEC3_1163	Type IV pilus assembly protein
OG_02517	LG3211_1719	dnaX; DNA polymerase III, subunit gamma and tau	OG_02517	LEC3_1675	DNA polymerase III subunit gamma and tau
OG_02518	LG3211_3770	rpoB; DNA-directed RNA polymerase, beta subunit	OG_02518	LEC3_3797	DNA-directed RNA polymerase, beta subunit
OG_02519	LG3211_5071	LG3211_5071; rrf2 family protein	OG_02519	LEC3_4985	transcriptional regulator, Rrf2 family
OG_02520	LG3211_4489	LG3211_4489; patatin-like phospholipase family protein	OG_02520	LEC3_4541	patatin-like phospholipase
OG_02521	LG3211_5162	LG3211_5162; hypothetical protein	OG_02521	LEC3_0126	membrane protein
OG_02522	LG3211_1401	msrA; peptide methionine sulfoxide reductase msrA	OG_02522	LEC3_3889	methionine-S-sulfoxide reductase
OG_02523	LG3211_5306	LG3211_5306; conserved hypothetical protein	OG_02523	LEC3_5434	inner membrane protein
OG_02524	LG3211_1083	LG3211_1083; conserved hypothetical protein	OG_02524	LEC3_1057	integral membrane protein
OG_02525	LG3211_4193	LG3211_4193; conserved hypothetical protein	OG_02525	LEC3_4252	UptF protein
OG_02526	LG3211_3285	pstB; phosphate ABC transporter, ATP-binding protein	OG_02526	LEC3_3317	phosphate ABC transporter, ATP-binding protein
OG_02527	LG3211_647	LG3211_647; glycosyl transferase 2 family protein	OG_02527	LEC3_4700	rhamnosyltransferase
OG_02528	LG3211_630	LG3211_630; amino acid permease family protein	OG_02528	LEC3_4721	proline-specific permease proY
OG_02529	LG3211_3118	LG3211_3118; bacterial regulatory helix-turn-helix, AraC family protei	OG_02529	LEC3_3118	transcriptional regulator, AraC family
OG_02530	LG3211_2263	LG3211_2263; conserved hypothetical protein	OG_02530	LEC3_2216	conserved hypothetical protein
OG_02531	LG3211_2654	LG3211_2654; conserved hypothetical protein	OG_02531	LEC3_2658	conserved hypothetical protein
OG_02532	LG3211_1971	LG3211_1971; acetyltransferase family protein	OG_02532	LEC3_1925	N-acetyltransferase
OG_02533	LG3211_246	LG3211_246; RNA polymerase sigma factor, sigma-70 family protein	OG_02533	LEC3_5084	RNA polymerase sigma factor, sigma-70 family
OG_02534	LG3211_1603	LG3211_1603; histidine kinase-, DNA gyrase B-, and HSP90-like ATF	OG_02534	LEC3_1578	histidine kinase

OG_02535	LG3211_3753	rplN; ribosomal protein L14	OG_02535	LEC3_3780	ribosomal protein L14
OG_02536	LG3211_627	LG3211_627; ABC transporter family protein	OG_02536	LEC3_4723	ABC transporter, ATP-binding protein
OG_02537	LG3211_5197	LG3211_5197; uvrD/REP helicase N-terminal domain protein	OG_02537	LEC3_0090	ATP-dependent DNA helicase
OG_02538	LG3211_1731	LG3211_1731; conserved hypothetical protein	OG_02538	LEC3_1687	conserved hypothetical protein
OG_02539	LG3211_3740	rpsD; ribosomal protein S4	OG_02539	LEC3_3767	ribosomal protein S4
OG_02540	LG3211_508	LG3211_508; conserved hypothetical protein	OG_02540	LEC3_0324	conserved hypothetical protein SEQUENCING GAP
OG_02541	LG3211_2700	LG3211_2700; conserved hypothetical protein	OG_02541	LEC3_2701	nucleoprotein/polynucleotide-associated enzyme
OG_02542	LG3211_3914	LG3211_3914; conserved hypothetical protein	OG_02542	LEC3_3936	conserved hypothetical protein
OG_02543	LG3211_3762	rplD; 50S ribosomal protein L4	OG_02543	LEC3_3789	ribosomal protein L4
OG_02544	LG3211_4075	murC; UDP-N-acetylmuramate--alanine ligase	OG_02544	LEC3_4130	UDP-N-acetylmuramate--alanine ligase
OG_02545	LG3211_3596	sodB; sodB	OG_02545	LEC3_3588	superoxide dismutase [Fe]
OG_02546	LG3211_355	LG3211_355; conserved hypothetical protein	OG_02546	LEC3_5018	conserved hypothetical protein
OG_02547	LG3211_1119	LG3211_1119; conserved hypothetical protein	OG_02547	LEC3_1094	hypothetical protein
OG_02548	LG3211_1440	LG3211_1440; eamA-like transporter family protein	OG_02548	LEC3_1410	integral membrane protein DUF6
OG_02549	LG3211_3809	LG3211_3809; conserved hypothetical protein	OG_02549	LEC3_3832	conserved hypothetical protein
OG_02550	LG3211_4209	speD; S-adenosylmethionine decarboxylase proenzyme	OG_02550	LEC3_4264	S-adenosylmethionine decarboxylase proenzyme
OG_02551	LG3211_4553	LG3211_4553; dihydrodipicolinate synthetase family protein	OG_02551	LEC3_4608	dihydrodipicolinate synthetase family
OG_02553	LG3211_4475	LG3211_4475; acetyltransferase family protein	OG_02553	LEC3_4527	acetyltransferase (GNAT) family
OG_02554	LG3211_1537	hflK; hflK protein	OG_02554	LEC3_1512	HflK protein
OG_02555	LG3211_3705	murD; UDP-N-acetylmuramoylalanine--D-glutamate ligase	OG_02555	LEC3_3730	UDP-N-acetylmuramoylalanine--D-glutamate ligase
OG_02556	LG3211_1241	LG3211_1241; phosphatase, YrbI family	OG_02556	LEC3_1196	3-deoxy-D-manno-octulosonate 8-phosphate phosphatase, YrbI famil
OG_02557	LG3211_896	ppa; ppa	OG_02557	LEC3_0842	inorganic diphosphatase
OG_02558	LG3211_4983	LG3211_4983; bacterial regulatory, tetR family protein	OG_02558	LEC3_4892	transcriptional regulator, TetR family
OG_02559	LG3211_4966	rpoH; alternative sigma factor RpoH	OG_02559	LEC3_4879	RNA polymerase sigma-32 factor
OG_02560	LG3211_706	aceF; dihydrolipoyllysine-residue acetyltransferase	OG_02560	LEC3_0675	dihydrolipoyllysine-residue acetyltransferase
OG_02561	LG3211_4017	cobQ; cobyric acid synthase CobQ	OG_02561	LEC3_4085	cobyric acid synthase CobQ
OG_02562	LG3211_2148	LG3211_2148; rhodanese-like domain protein	OG_02562	LEC3_2089	rhodanese domain protein
OG_02563	LG3211_3955	LG3211_3955; relA/SpoT family protein	OG_02563	LEC3_3978	RelA/SpoT family protein
OG_02564	LG3211_3920	cysQ; 3'(2'),5'-bisphosphate nucleotidase	OG_02564	LEC3_3944	3'(2'),5'-bisphosphate nucleotidase
OG_02565	LG3211_3049	LG3211_3049; flavinator of succinate dehydrogenase family protein	OG_02565	LEC3_3041	conserved domain protein
OG_02566	LG3211_1435	LG3211_1435; tat (twin-arginine translocation) pathway signal sequer	OG_02566	LEC3_1403	glucanase B
OG_02567	LG3211_564	LG3211_564; DSBA-like thioredoxin domain protein	OG_02567	LEC3_4808	dsba oxidoreductase
OG_02568	LG3211_1531	hflB; ftsH HflB	OG_02568	LEC3_1507	ATP-dependent metallopeptidase HflB
OG_02569	LG3211_3527	LG3211_3527; hypothetical protein	OG_02569	LEC3_3559	conserved hypothetical protein
OG_02570	LG3211_3655	rfbC; dTDP-4-dehydrorhamnose 3,5-epimerase	OG_02570	LEC3_3641	dTDP-4-dehydrorhamnose 3,5-epimerase
OG_02571	LG3211_3617	LG3211_3617; ABC-2 type transporter family protein	OG_02571	LEC3_3608	ABC-2 type transporter
OG_02572	LG3211_4126	gspE; type II secretion system protein E	OG_02572	LEC3_4183	general secretory pathway protein E
OG_02573	LG3211_1347	LG3211_1347; GDSL-like Lipase/Acylhydrolase family protein	OG_02573	LEC3_1303	lipolytic enzyme, G-D-S-L family
OG_02574	LG3211_2822	ccmB; heme exporter protein CcmB	OG_02574	LEC3_2827	heme exporter protein CcmB
OG_02575	LG3211_1450	LG3211_1450; molydopterin dinucleotide binding domain protein	OG_02575	LEC3_1420	oxidoreductase alpha (molybdopterin) subunit
OG_02576	LG3211_2773	LG3211_2773; binding-protein-dependent transport system inner mer	OG_02576	LEC3_2779	ABC transporter, permease protein
OG_02577	LG3211_3908	LG3211_3908; hypothetical protein	OG_02577	LEC3_3930	conserved hypothetical protein
OG_02578	LG3211_82	yigN; recombination limiting protein	OG_02578	LEC3_5241	DNA recombination protein RmuC homolog
OG_02579	LG3211_2905	LG3211_2905; hypothetical protein	OG_02579	LEC3_2918	hypothetical protein
OG_02580	LG3211_2456	LG3211_2456; cheW-like domain protein	OG_02580	LEC3_2452	purine-binding chemotaxis protein Chew
OG_02581	LG3211_1512	LG3211_1512; hypothetical protein	OG_02581	LEC3_1493	lipoprotein
OG_02582	LG3211_1558	LG3211_1558; peptidase M20/M25/M40 family protein	OG_02582	LEC3_1524	peptidase, M28 family
OG_02583	LG3211_1511	LG3211_1511; tRNA pseudouridine synthase, TruD family protein	OG_02583	LEC3_1492	ribosomal large subunit pseudouridine synthase D
OG_02584	LG3211_5130	LG3211_5130; conserved hypothetical protein	OG_02584	LEC3_0155	conserved hypothetical protein
OG_02585	LG3211_5362	LG3211_5362; ABC transporter family protein	OG_02585	LEC3_5357	ABC transporter, ATP-binding protein
OG_02586	LG3211_5206	LG3211_5206; DEAD/DEAH box helicase family protein	OG_02586	LEC3_0083	ATP-dependent DNA helicase
OG_02587	LG3211_1015	LG3211_1015; 5'-nucleotidase	OG_02587	LEC3_0978	haloacid dehalogenase superfamily hydrolase, subfamily IA
OG_02588	LG3211_1011	LG3211_1011; conserved hypothetical protein	OG_02588	LEC3_0975	membrane protein
OG_02589	LG3211_1613	LG3211_1613; hypothetical protein	OG_02589	LEC3_1587	hypothetical protein
OG_02590	LG3211_3512	LG3211_3512; conserved hypothetical protein	OG_02590	LEC3_3543	conserved hypothetical protein
OG_02591	LG3211_3722	LG3211_3722; glutaredoxin family protein	OG_02591	LEC3_3746	glutaredoxin
OG_02592	LG3211_4950	LG3211_4950; short chain dehydrogenase family protein	OG_02592	LEC3_4858	tropinone reductase
OG_02593	LG3211_2917	LG3211_2917; conserved hypothetical protein	OG_02593	LEC3_2929	agmatine deiminase
OG_02594	LG3211_2927	LG3211_2927; efflux transporter, RND family, MFP subunit	OG_02594	LEC3_2938	efflux transporter, RND family, MFP subunit
OG_02595	LG3211_113	LG3211_113; glucan biosynthesis protein D	OG_02595	LEC3_5210	periplasmic glucan biosynthesis protein, MdoG
OG_02596	LG3211_774	LG3211_774; interferon-induced transmembrane family protein	OG_02596	LEC3_0735	transmembrane protein
OG_02597	LG3211_1007	LG3211_1007; acyl-CoA hydrolase	OG_02597	LEC3_0970	thioesterase superfamily
OG_02598	LG3211_723	glmU; UDP-N-acetylglucosamine diphosphorylase/glucosamine-1-phc	OG_02598	LEC3_0692	UDP-N-acetylglucosamine diphosphorylase/glucosamine-1-phosphat
OG_02599	LG3211_4990	LG3211_4990; response regulator	OG_02599	LEC3_4902	response regulator receiver domain protein
OG_02600	LG3211_4954	LG3211_4954; MATE efflux family protein	OG_02600	LEC3_4860	MATE efflux family protein
OG_02601	LG3211_4407	LG3211_4407; conserved hypothetical protein	OG_02601	LEC3_4454	conserved hypothetical protein
OG_02602	LG3211_1327	LG3211_1327; small GTP-binding domain protein	OG_02602	LEC3_1282	translation elongation factor G-like protein
OG_02603	LG3211_2689	gdhB; NAD-specific glutamate dehydrogenase	OG_02603	LEC3_2691	bacterial NAD-glutamate dehydrogenase family protein
OG_02604	LG3211_3793	hemA; glutamyl-tRNA reductase	OG_02604	LEC3_3814	glutamyl-tRNA reductase
OG_02605	LG3211_1767	LG3211_1767; L,D-transpeptidase catalytic domain protein	OG_02605	LEC3_1723	ErfK/YbiS/YcfS/YnhG family protein
OG_02606	LG3211_1406	LG3211_1406; hypothetical protein	OG_02606	LEC3_1371	hypothetical protein
OG_02607	LG3211_3437	LG3211_3437; phosphate transporter family protein	OG_02607	LEC3_3500	phosphate transporter family
OG_02608	LG3211_1160	LG3211_1160; molybdopterin-binding domain of aldehyde dehydroge	OG_02608	LEC3_1132	aldehyde oxidase and xanthine dehydrogenase, molybdopterin bindir
OG_02609	LG3211_3220	LG3211_3220; sigma-54 interaction domain protein	OG_02609	LEC3_3239	ATPase family associated with various cellular activities (AAA)
OG_02610	LG3211_2455	LG3211_2455; methyl-accepting chemotaxis (MCP) signalling domair	OG_02610	LEC3_2453	Methyl-accepting chemotaxis protein (MCP) signaling domain
OG_02611	LG3211_1340	LG3211_1340; NADH:flavin oxidoreductase / NADH oxidase family pi	OG_02611	LEC3_1293	FAD dependent oxidoreductase
OG_02612	LG3211_445	LG3211_445; conserved hypothetical protein	OG_02612	LEC3_0256	conserved hypothetical protein
OG_02613	LG3211_5353	LG3211_5353; his Kinase A domain protein	OG_02613	LEC3_5368	sensor histidine kinase
OG_02614	LG3211_1304	LG3211_1304; SET domain protein	OG_02614	LEC3_1262	nuclear protein SET
OG_02615	LG3211_5193	LG3211_5193; ribonuclease UK114 domain protein	OG_02615	LEC3_0096	endoribonuclease L-PSP family protein
OG_02616	LG3211_2684	LG3211_2684; amino acid permease family protein	OG_02616	LEC3_2688	amino acid permease

OG_02617	LG3211_3186	kdpB; K+-transporting ATPase, B subunit	OG_02617	LEC3_3193	K+-transporting ATPase, B subunit
OG_02618	LG3211_3113	LG3211_3113; endonuclease/Exonuclease/phosphatase family protei	OG_02618	LEC3_3115	endonuclease/exonuclease/phosphatase family protein
OG_02619	LG3211_4293	LG3211_4293; acetyltransferase family protein	OG_02619	LEC3_4354	acetyltransferase, GNAT family
OG_02620	LG3211_2302	recN; DNA repair protein RecN	OG_02620	LEC3_2229	DNA repair protein
OG_02621	LG3211_3626	LG3211_3626; putative protein with nucleoside triphosphate hydrolas	OG_02621	LEC3_3618	ATP-binding protein
OG_02623	LG3211_3086	LG3211_3086; lytTr DNA-binding domain protein	OG_02623	LEC3_3080	transcriptional regulator, LytR/AlgR family
OG_02624	LG3211_705	LG3211_705; conserved hypothetical protein	OG_02624	LEC3_0673	conserved hypothetical protein
OG_02625	LG3211_974	LG3211_974; type IV pilus assembly PilM family protein	OG_02625	LEC3_0932	Type IV pilus assembly protein
OG_02626	LG3211_1709	LG3211_1709; thiS family protein	OG_02626	LEC3_1671	molybdopterin converting factor, subunit 1
OG_02627	LG3211_2548	LG3211_2548; tonB dependent receptor family protein	OG_02627	LEC3_2532	TonB-dependent receptor
OG_02628	LG3211_4290	LG3211_4290; hypothetical protein	OG_02628	LEC3_4351	beta-lactamase OXA-5
OG_02629	LG3211_129	LG3211_129; glutamine-fructose-6-phosphate transaminase	OG_02629	LEC3_5196	SIS domain protein
OG_02630	LG3211_759	LG3211_759; NUDIX domain protein	OG_02630	LEC3_0721	nudix hydrolase
OG_02631	LG3211_2269	mmsB; 3-hydroxyisobutyrate dehydrogenase	OG_02631	LEC3_0579	3-hydroxyisobutyrate dehydrogenase
OG_02632	LG3211_4349	LG3211_4349; fatty acid desaturase family protein	OG_02632	LEC3_4391	fatty acid desaturase
OG_02633	LG3211_221	LG3211_221; conserved hypothetical protein	OG_02633	LEC3_5109	conserved hypothetical protein
OG_02634	LG3211_966	LG3211_966; inosine-uridine preferring nucleoside hydrolase family p	OG_02634	LEC3_0924	inosine-uridine preferring nucleoside hydrolase
OG_02635	LG3211_3268	LG3211_3268; conserved hypothetical protein	OG_02635	LEC3_3300	conserved hypothetical protein
OG_02636	LG3211_3010	arsC; arsenate reductase	OG_02636	LEC3_3002	arsenate reductase
OG_02637	LG3211_1206	lipL; lactonizing lipase	OG_02637	LEC3_1157	lipase (Triacylglycerol lipase)
OG_02638	LG3211_2912	rpsA; ribosomal protein S1	OG_02638	LEC3_2925	ribosomal protein S1
OG_02639	LG3211_336	LG3211_336; uroporphyrinogen-III synthase HemD family protein	OG_02639	LEC3_5035	uroporphyrinogen-III synthase
OG_02640	LG3211_3037	pgsA; CDP-diacylglycerol--glycerol-3-phosphate 3-phosphatidyltransf	OG_02640	LEC3_3027	CDP-diacylglycerol--glycerol-3-phosphate 3-phosphatidyltransferase
OG_02642	LG3211_1408	LG3211_1408; glyoxalase/Bleomycin resistance /Dioxygenase superf	OG_02642	LEC3_1373	glyoxalase/bleomycin resistance protein/dioxygenase superfamily pro
OG_02643	LG3211_838	LG3211_838; alpha/beta hydrolase family protein	OG_02643	LEC3_0786	lipoprotein
OG_02644	LG3211_4629	LG3211_4629; putative acid phosphatase Wzb	OG_02644	LEC3_2259	Low molecular weight phosphotyrosine protein phosphatase
OG_02645	LG3211_1412	LG3211_1412; zinc-binding dehydrogenase family protein	OG_02645	LEC3_1377	oxidoreductase, zinc-binding dehydrogenase family protein
OG_02646	LG3211_4198	LG3211_4198; fumarylacetoacetate (FAA) hydrolase family protein	OG_02646	LEC3_4257	fumarylacetoacetate hydrolase
OG_02647	LG3211_467	LG3211_467; conserved hypothetical protein	OG_02647	LEC3_0277	conserved hypothetical protein
OG_02648	LG3211_1234	LG3211_1234; HPr Serine kinase N terminus family protein	OG_02648	LEC3_1189	HPr(Ser) kinase/phosphatase
OG_02649	LG3211_88	yrbE; mlaE	OG_02649	LEC3_5234	toluene ABC transport system permease protein
OG_02650	LG3211_4703	LG3211_4703; ompA family protein	OG_02650	LEC3_0523	OmpA/MotB family protein
OG_02651	LG3211_4523	LG3211_4523; histidine kinase-, DNA gyrase B-, and HSP90-like ATF	OG_02651	LEC3_4582	sensor histidine kinase
OG_02652	LG3211_4305	LG3211_4305; glucose / Sorbosone dehydrogenase family protein	OG_02652	LEC3_4364	glucose/sorbosone dehydrogenase
OG_02653	LG3211_5344	LG3211_5344; isocitrate dehydrogenase kinase/phosphatase family p	OG_02653	LEC3_5377	isocitrate dehydrogenase kinase/phosphatase
OG_02654	LG3211_5119	LG3211_5119; trypsin-like peptidase domain protein	OG_02654	LEC3_0165	FOG: PKD repeat family protein
OG_02655	LG3211_4429	LG3211_4429; C-terminal processing peptidase family protein	OG_02655	LEC3_4472	tail-specific protease precursor
OG_02656	LG3211_4162	LG3211_4162; 3-oxoacid CoA-transferase, A subunit	OG_02656	LEC3_4228	3-oxoacid CoA-transferase, A subunit family
OG_02657	LG3211_2870	LG3211_2870; conserved hypothetical protein	OG_02657	LEC3_2881	conserved hypothetical protein
OG_02658	LG3211_3287	LG3211_3287; RNA polymerase sigma factor, sigma-70 family protei	OG_02658	LEC3_3319	RNA polymerase sigma-E factor
OG_02659	LG3211_3750	rpsN; 30S ribosomal subunit protein S14	OG_02659	LEC3_3777	ribosomal protein S14
OG_02660	LG3211_5227	bioB; biotin synthase	OG_02660	LEC3_0061	biotin synthase
OG_02661	LG3211_2551	LG3211_2551; ROK family protein	OG_02661	LEC3_2535	transcriptional regulatory protein
OG_02662	LG3211_934	LG3211_934; 5-formyltetrahydrofolate cyclo-ligase	OG_02662	LEC3_0884	5,10-methenyltetrahydrofolate synthetase
OG_02663	LG3211_2185	truA; tRNA pseudouridine(38-40) synthase	OG_02663	LEC3_2145	tRNA pseudouridine synthase A
OG_02664	LG3211_3987	tolR; protein TolR	OG_02664	LEC3_4055	protein TolR
OG_02665	LG3211_3328	lpxD; UDP-3-O-[3-hydroxymyristoyl] glucosamine N-acyltransferase	OG_02665	LEC3_3380	UDP-3-O-[3-hydroxymyristoyl] glucosamine N-acyltransferase
OG_02666	LG3211_5070	LG3211_5070; binding-protein-dependent transport system inner mer	OG_02666	LEC3_4984	binding-protein-dependent transport system inner membrane compor
OG_02667	LG3211_1376	LG3211_1376; conserved hypothetical protein	OG_02667	LEC3_1340	conserved hypothetical protein
OG_02668	LG3211_3153	LG3211_3153; conserved hypothetical protein	OG_02668	LEC3_3160	conserved hypothetical protein
OG_02669	LG3211_4430	lipA; lipoyl synthase	OG_02669	LEC3_4475	lipoic acid synthetase
OG_02670	LG3211_1470	Akp3; alkaline phosphatase 3, intestine, not Mn requiring	OG_02670	LEC3_1439	intestinal alkaline phosphatase
OG_02671	LG3211_4635	LG3211_4635; matE family protein	OG_02671	LEC3_2253	polysaccharide biosynthesis protein
OG_02672	LG3211_5016	LG3211_5016; ankyrin repeat family protein	OG_02672	LEC3_4930	ankyrin repeat protein
OG_02673	LG3211_1184	pdxH; pyridoxamine 5'-phosphate oxidase	OG_02673	LEC3_1154	pyridoxamine 5'-phosphate oxidase
OG_02674	LG3211_5006	LG3211_5006; ftsX-like permease family protein	OG_02674	LEC3_4914	efflux ABC transporter, permease protein
OG_02675	LG3211_4962	LG3211_4962; conserved hypothetical protein	OG_02675	LEC3_4872	conserved hypothetical protein
OG_02676	LG3211_3738	rplQ; ribosomal protein L17	OG_02676	LEC3_3765	ribosomal protein L17
OG_02677	LG3211_1269	LG3211_1269; macro domain protein	OG_02677	LEC3_1225	UPF0189 protein
OG_02678	LG3211_1955	LG3211_1955; calcineurin-like phosphoesterase family protein	OG_02678	LEC3_1905	Ser/Thr protein phosphatase family protein
OG_02679	LG3211_2487	LG3211_2487; conserved hypothetical protein	OG_02679	LEC3_1797	conserved hypothetical protein
OG_02680	LG3211_3534	pheS; phenylalanine--tRNA ligase, alpha subunit	OG_02680	LEC3_3565	phenylalanyl-tRNA synthetase, alpha subunit
OG_02681	LG3211_227	LG3211_227; conserved hypothetical protein	OG_02681	LEC3_5103	hypothetical protein
OG_02682	LG3211_4123	gspG; type II secretion system protein G	OG_02682	LEC3_4180	general secretion pathway protein G
OG_02683	LG3211_2136	LG3211_2136; conserved hypothetical protein	OG_02683	LEC3_2079	conserved hypothetical protein
OG_02684	LG3211_1159	LG3211_1159; 2Fe-2S iron-sulfur cluster binding domain protein	OG_02684	LEC3_1131	xanthine dehydrogenase
OG_02685	LG3211_1127	LG3211_1127; aspartyl/Asparaginyl beta-hydroxylase family protein	OG_02685	LEC3_1103	aspartyl/Asparaginyl beta-hydroxylase family
OG_02686	LG3211_2723	lpdA; dihydrolipoyl dehydrogenase	OG_02686	LEC3_2725	dihydrolipoyl dehydrogenase
OG_02687	LG3211_3838	LG3211_3838; conserved hypothetical protein	OG_02687	LEC3_3861	conserved hypothetical protein
OG_02688	LG3211_3131	LG3211_3131; conserved hypothetical protein	OG_02688	LEC3_3135	YaeQ family protein
OG_02689	LG3211_239	LG3211_239; putative transcriptional regulator, ArsR family protein	OG_02689	LEC3_5092	transcriptional regulator, ArsR family
OG_02690	LG3211_3082	yliI; aldose sugar dehydrogenase	OG_02690	LEC3_3075	soluble aldose sugar dehydrogenase YliI precusor
OG_02691	LG3211_1074	LG3211_1074; conserved hypothetical protein	OG_02691	LEC3_1046	integral membrane protein
OG_02692	LG3211_2403	LG3211_2403; bacterial regulatory helix-turn-helix , lysR family protei	OG_02692	LEC3_2345	transcriptional regulator, LysR family
OG_02693	LG3211_773	LG3211_773; conserved hypothetical protein	OG_02693	LEC3_0734	conserved hypothetical protein
OG_02694	LG3211_4306	LG3211_4306; transglycosylase SLT domain protein	OG_02694	LEC3_4365	transglycosylase SLT domain
OG_02695	LG3211_3313	map; methionine aminopeptidase, type I	OG_02695	LEC3_3360	methionine aminopeptidase, type I
OG_02696	LG3211_124	LG3211_124; thiamine pyrophosphate enzyme, C-terminal TPP bindi	OG_02696	LEC3_5200	2-oxoacid:ferredoxin/flavodoxin oxidoreductases, gamma subunit dor
OG_02697	LG3211_2713	LG3211_2713; hypothetical protein	OG_02697	LEC3_2714	hypothetical protein
OG_02698	LG3211_1259	LG3211_1259; conserved hypothetical protein	OG_02698	LEC3_1215	hypothetical protein
OG_02699	LG3211_2832	LG3211_2832; conserved hypothetical protein	OG_02699	LEC3_2838	conserved hypothetical protein

OG_02700	LG3211_1691	LG3211_1691; lysM domain protein	OG_02700	LEC3_1657	transglycosylase SLT domain/LysM domain
OG_02701	LG3211_1436	LG3211_1436; peptidase M1 family protein	OG_02701	LEC3_1405	peptidase, family M1
OG_02702	LG3211_3410	LG3211_3410; peptidase M1 family protein	OG_02702	LEC3_3470	membrane protein
OG_02703	LG3211_4030	LG3211_4030; putative transmembrane protein	OG_02703	LEC3_4100	conserved hypothetical protein
OG_02704	LG3211_2690	LG3211_2690; acyl-CoA dehydrogenase, N-terminal domain protein	OG_02704	LEC3_2692	acyl-CoA dehydrogenase
OG_02705	LG3211_566	LG3211_566; amidohydrolase family protein	OG_02705	LEC3_4805	amidohydrolase family protein
OG_02706	LG3211_3222	LG3211_3222; conserved hypothetical protein	OG_02706	LEC3_3245	conserved hypothetical protein
OG_02707	LG3211_811	LG3211_811; conserved hypothetical protein	OG_02707	LEC3_0761	conserved hypothetical protein
OG_02708	LG3211_3647	LG3211_3647; conserved hypothetical protein	OG_02708	LEC3_3634	inner membrane protein
OG_02709	LG3211_1020	pyk; pyruvate kinase	OG_02709	LEC3_0983	pyruvate kinase
OG_02710	LG3211_4957	LG3211_4957; conserved hypothetical protein	OG_02710	LEC3_4863	conserved hypothetical protein
OG_02711	LG3211_3045	LG3211_3045; DNA internalization-related competence protein ComE	OG_02711	LEC3_3036	DNA internalization-related competence protein ComEC/Rec2
OG_02712	LG3211_3709	LG3211_3709; sodium Bile acid symporter family protein	OG_02712	LEC3_3735	sodium bile acid symporter family
OG_02713	LG3211_459	LG3211_459; biofilm formation and stress response factor family prot	OG_02713	LEC3_0268	conserved hypothetical protein
OG_02714	LG3211_3044	LG3211_3044; motA/TolQ/ExbB proton channel family protein	OG_02714	LEC3_3035	transporter, MotA/TolQ/ExbB proton channel family
OG_02715	LG3211_2808	LG3211_2808; type IV secretion/conjugal transfer ATPase, VirB4 fam	OG_02715	LEC3_2815	virB4 protein
OG_02716	LG3211_2216	nusA; transcription termination/antitermination L factor	OG_02716	LEC3_2175	transcription termination factor NusA
OG_02717	LG3211_643	LG3211_643; HEAT repeats family protein	OG_02717	LEC3_4704	hypothetical protein
OG_02718	LG3211_1872	ubiG; 3-demethylubiquinone-9 3-O-methyltransferase	OG_02718	LEC3_1817	3-demethylubiquinone-9 3-O-methyltransferase
OG_02719	LG3211_3785	pth; peptidyl-tRNA hydrolase	OG_02719	LEC3_3807	peptidyl-tRNA hydrolase
OG_02720	LG3211_2163	LG3211_2163; hypothetical protein	OG_02720	LEC3_2117	hypothetical protein
OG_02721	LG3211_663	LG3211_663; eamA-like transporter family protein	OG_02721	LEC3_4684	integral membrane protein DUF6
OG_02722	LG3211_4497	LG3211_4497; conserved hypothetical protein	OG_02722	LEC3_4550	hypothetical protein
OG_02723	LG3211_136	eda; 2-dehydro-3-deoxyphosphogluconate aldolase/4-hydroxy-2-oxog	OG_02723	LEC3_5189	2-dehydro-3-deoxyphosphogluconate aldolase/4-hydroxy-2-oxoglutar
OG_02724	LG3211_3344	LG3211_3344; conserved hypothetical protein	OG_02724	LEC3_3401	conserved hypothetical protein
OG_02725	LG3211_3755	rpmC; ribosomal protein L29	OG_02725	LEC3_3782	ribosomal protein L29
OG_02726	LG3211_392	LG3211_392; RF-1 domain protein	OG_02726	LEC3_0214	peptidyl-tRNA hydrolase domain
OG_02727	LG3211_4927	dtd; D-tyrosyl-tRNA(Tyr) deacylase	OG_02727	LEC3_0367	D-tyrosyl-tRNA(Tyr) deacylase
OG_02728	LG3211_409	LG3211_409; bacterial regulatory helix-turn-helix, AraC family protein	OG_02728	LEC3_0235	transcriptional regulator, AraC family
OG_02729	LG3211_536	LG3211_536; eamA-like transporter family protein	OG_02729	LEC3_4849	integral membrane protein DUF6
OG_02730	LG3211_1181	LG3211_1181; shikimate kinase family protein	OG_02730	LEC3_1151	shikimate kinase
OG_02731	LG3211_3456	tgt; queuine tRNA-ribosyltransferase	OG_02731	LEC3_3515	queuine tRNA-ribosyltransferase
OG_02732	LG3211_3256	ribF; riboflavin biosynthesis protein RibF	OG_02732	LEC3_3287	riboflavin biosynthesis protein RibF
OG_02733	LG3211_2699	LG3211_2699; alpha/beta hydrolase family protein	OG_02733	LEC3_2700	hydrolase, alpha/beta fold family
OG_02734	LG3211_1612	LG3211_1612; conserved hypothetical protein	OG_02734	LEC3_1586	conserved hypothetical protein
OG_02735	LG3211_2361	LG3211_2361; hypothetical protein	OG_02735	LEC3_2306	lipoprotein
OG_02736	LG3211_4018	LG3211_4018; threonine-phosphate decarboxylase	OG_02736	LEC3_4086	L-threonine-O-3-phosphate decarboxylase
OG_02737	LG3211_3516	LG3211_3516; TPR repeat family protein	OG_02737	LEC3_3548	serine/threonine-protein kinase Pkn2
OG_02738	LG3211_3238	LG3211_3238; H+ antiporter-2 family protein	OG_02738	LEC3_3266	multidrug resistance protein B
OG_02739	LG3211_5010	trxA; thioredoxin	OG_02739	LEC3_4917	thioredoxin
OG_02740	LG3211_3178	LG3211_3178; ACT domain protein	OG_02740	LEC3_3184	glycine cleavage system transcriptional repressor
OG_02741	LG3211_594	LG3211_594; bacterial regulatory helix-turn-helix , lysR family protein	OG_02741	LEC3_4773	transcriptional regulator, LysR family
OG_02742	LG3211_2342	LG3211_2342; conserved hypothetical protein	OG_02742	LEC3_2286	conserved hypothetical protein
OG_02743	LG3211_1604	LG3211_1604; bacterial regulatory, luxR family protein	OG_02743	LEC3_1579	two-component system regulatory protein
OG_02744	LG3211_3116	LG3211_3116; 2'-5' RNA ligase	OG_02744	LEC3_3116	2'-5' RNA ligase
OG_02745	LG3211_5023	LG3211_5023; bacterial Cytochrome Ubiquinol Oxidase family protei	OG_02745	LEC3_4934	cytochrome d ubiquinol oxidase, subunit I
OG_02746	LG3211_222	LG3211_222; type III secretion apparatus lipoprotein, YscJ/HrcJ famil	OG_02746	LEC3_5108	type III secretion apparatus lipoprotein
OG_02747	LG3211_1965	LG3211_1965; NUDIX domain protein	OG_02747	LEC3_1917	nudix hydrolase family protein
OG_02748	LG3211_880	thrB; homoserine kinase	OG_02748	LEC3_0828	homoserine kinase
OG_02749	LG3211_2293	LG3211_2293; hypothetical protein	OG_02749	LEC3_2220	conserved hypothetical protein
OG_02750	LG3211_543	LG3211_543; efflux transporter, RND family, MFP subunit	OG_02750	LEC3_4839	efflux transporter, RND family, MFP subunit
OG_02751	LG3211_997	LG3211_997; ompW family protein	OG_02751	LEC3_0961	outer membrane protein, OmpW family
OG_02752	LG3211_3607	LG3211_3607; cytochrome b(C-terminal)/b6/petD family protein	OG_02752	LEC3_3600	cytochrome B family protein
OG_02753	LG3211_5326	LG3211_5326; aldo/keto reductase family protein	OG_02753	LEC3_5405	oxidoreductase, aldo/keto reductase family protein
OG_02754	LG3211_4294	LG3211_4294; conserved hypothetical protein	OG_02754	LEC3_4355	conserved hypothetical protein
OG_02755	LG3211_4108	map; methionine aminopeptidase, type I	OG_02755	LEC3_4165	methionine aminopeptidase, type I
OG_02756	LG3211_123	LG3211_123; putative transmembrane regulator PrtR	OG_02756	LEC3_5202	transmembrane regulator PrtR
OG_02757	LG3211_1894	argH; argininosuccinate lyase	OG_02757	LEC3_1843	argininosuccinate lyase
OG_02758	LG3211_215	LG3211_215; his Kinase A domain protein	OG_02758	LEC3_5115	His Kinase A (phosphoacceptor) domain
OG_02759	LG3211_4082	LG3211_4082; penicillin binding transpeptidase domain protein	OG_02759	LEC3_4136	penicillin binding protein
OG_02760	LG3211_2373	lolA; outer membrane lipocarrier protein LolA	OG_02760	LEC3_2320	outer membrane lipoprotein carrier protein LolA
OG_02761	LG3211_3137	LG3211_3137; conserved hypothetical protein	OG_02761	LEC3_3141	allophanate hydrolase, subunit 2
OG_02762	LG3211_3154	metG; methionyl-tRNA synthetase	OG_02762	LEC3_3161	methionyl-tRNA synthetase
OG_02763	LG3211_2947	LG3211_2947; EF hand family protein	OG_02763	LEC3_2966	EF hand domain protein
OG_02764	LG3211_4770	chiD; chitinase D	OG_02764	LEC3_0401	chitinase
OG_02765	LG3211_846	LG3211_846; glnD PII-uridylyltransferase family protein	OG_02765	LEC3_0793	glutamate-ammonia ligase adenylyltransferase
OG_02766	LG3211_3019	LG3211_3019; 7, 8-dihydro-6-hydroxymethylpterin-pyrophosphokinas	OG_02766	LEC3_3012	putative 2-amino-4-hydroxy-6-hydroxymethyldihydropteridine pyropho
OG_02767	LG3211_3937	LG3211_3937; response regulator	OG_02767	LEC3_3961	Type IV pilus assembly protein
OG_02768	LG3211_2144	LG3211_2144; phage Tail Collar domain protein	OG_02768	LEC3_2085	microcystin dependent protein
OG_02769	LG3211_4072	ftsA; cell division protein FtsA	OG_02769	LEC3_4127	cell division protein FtsA
OG_02770	LG3211_4114	LG3211_4114; glycosyltransferase like 2 family protein	OG_02770	LEC3_4171	conserved hypothetical protein
OG_02771	LG3211_2667	LG3211_2667; carbonic anhydrase family protein	OG_02771	LEC3_2673	carbonate dehydratase
OG_02772	LG3211_451	anmK; anhydro-N-acetylmuramic acid kinase	OG_02772	LEC3_0262	anhydro-N-acetylmuramic acid kinase
OG_02773	LG3211_4309	LG3211_4309; NADPH-dependent FMN reductase family protein	OG_02773	LEC3_4369	NADPH-dependent fmn reductase
OG_02774	LG3211_2087	LG3211_2087; lipase family protein	OG_02774	LEC3_2027	lipase family
OG_02775	LG3211_139	LG3211_139; hypothetical protein	OG_02775	LEC3_5186	hypothetical protein
OG_02776	LG3211_4978	LG3211_4978; alpha/beta hydrolase fold family protein	OG_02776	LEC3_0231	2-hydroxymuconic semialdehyde hydrolase
OG_02777	LG3211_2955	LG3211_2955; transcriptional regulatory , C terminal family protein	OG_02777	LEC3_2972	transcriptional regulatory protein
OG_02778	LG3211_910	mpl; L-alanyl-gamma-D-glutamyl-meso-diaminopimelate ligase	OG_02778	LEC3_0859	UDP-N-acetylmuramate:L-alanyl-gamma-D-glutamyl- meso-diaminop
OG_02779	LG3211_2932	LG3211_2932; pterin 4 alpha carbinolamine dehydratase family prote	OG_02779	LEC3_2945	pterin-4-alpha-carbinolamine dehydratase
OG_02780	LG3211_692	LG3211_692; conserved hypothetical protein	OG_02780	LEC3_0657	conserved hypothetical protein

OG_02781	LG3211_913	LG3211_913; putative serine/threonine protein kinase	OG_02781	LEC3_0865	serine/threonine protein kinase protein family
OG_02782	LG3211_14	LG3211_14; hypothetical protein	OG_02782	LEC3_5322	hypothetical protein
OG_02783	LG3211_3134	LG3211_3134; dienelactone hydrolase family protein	OG_02783	LEC3_3138	carboxymethylenebutenolidase
OG_02784	LG3211_4999	LG3211_4999; bacterial regulatory helix-turn-helix , lysR family protei	OG_02784	LEC3_4909	transcriptional regulator, LysR-family
OG_02785	LG3211_2138	LG3211_2138; hypothetical protein	OG_02785	LEC3_2080	conserved hypothetical protein
OG_02786	LG3211_662	LG3211_662; bacterial regulatory, gntR family protein	OG_02786	LEC3_4686	bacterial regulatory proteins, gntR family/aminotransferase class I anc
OG_02787	LG3211_3403	LG3211_3403; diguanylate cyclase domain protein	OG_02787	LEC3_3458	cyclic diguanylate phosphodiesterase/diguanylate cyclase
OG_02788	LG3211_2029	LG3211_2029; polymer-forming cytoskeletal family protein	OG_02788	LEC3_1961	conserved hypothetical protein
OG_02789	LG3211_2404	LG3211_2404; acyl-CoA dehydrogenase, N-terminal domain protein	OG_02789	LEC3_2346	acyl-CoA dehydrogenase
OG_02790	LG3211_1750	lepA; GTP-binding protein LepA	OG_02790	LEC3_1707	GTP-binding protein LepA
OG_02791	LG3211_665	rtcB; conserved protein	OG_02791	LEC3_4681	conserved hypothetical protein
OG_02792	LG3211_969	gltA; citrate (Si)-synthase	OG_02792	LEC3_0927	citrate (Si)-synthase
OG_02793	LG3211_970	LG3211_970; conserved hypothetical protein	OG_02793	LEC3_0928	hypothetical protein
OG_02794	LG3211_3145	pcp; pyroglutamyl-peptidase I	OG_02794	LEC3_3153	pyrrolidone-carboxylate peptidase
OG_02795	LG3211_4151	LG3211_4151; ABC transporter family protein	OG_02795	LEC3_4217	ABC transporter, ATP-binding protein
OG_02796	LG3211_3848	LG3211_3848; putative NAD	OG_02796	LEC3_3869	alanine dehydrogenase/pyridine nucleotide transhydrogenase
OG_02797	LG3211_3594	LG3211_3594; oxidoreductase, short chain dehydrogenase/reductase	OG_02797	LEC3_3586	oxidoreductase, short chain dehydrogenase/reductase family protein
OG_02798	LG3211_3833	LG3211_3833; conserved hypothetical protein	OG_02798	LEC3_3856	FeS assembly SUF system protein SufT
OG_02799	LG3211_3302	LG3211_3302; proton antiporter-2 family protein	OG_02799	LEC3_3345	transporter, monovalent cation:proton antiporter-2 (CPA2) family
OG_02800	LG3211_3237	LG3211_3237; efflux transporter, RND family, MFP subunit	OG_02800	LEC3_3264	auxiliary transport protein, membrane fusion protein (MFP) family
OG_02801	LG3211_326	LG3211_326; conserved hypothetical protein	OG_02801	LEC3_5044	conserved hypothetical protein
OG_02802	LG3211_3245	radA; DNA repair protein RadA	OG_02802	LEC3_3274	DNA repair protein
OG_02803	LG3211_2666	Aldh8a1; aldehyde dehydrogenase family 8 member A1	OG_02803	LEC3_2672	aldehyde dehydrogenase (NAD) family protein
OG_02804	LG3211_3318	LG3211_3318; blaR1 peptidase M56 family protein	OG_02804	LEC3_3370	peptidase, M56 family
OG_02805	LG3211_5309	LG3211_5309; pspA/IM30 family protein	OG_02805	LEC3_5429	PspA/IM30 family protein
OG_02806	LG3211_2848	phaE; poly(R)-hydroxyalkanoic acid synthase, class III, PhaE subunit	OG_02806	LEC3_2859	PHA synthase subunit
OG_02807	LG3211_684	LG3211_684; hypothetical protein	OG_02807	LEC3_4666	hypothetical protein
OG_02808	LG3211_5172	LG3211_5172; conserved hypothetical protein	OG_02808	LEC3_0116	conserved hypothetical protein
OG_02809	LG3211_3882	LG3211_3882; sugar (and other) transporter family protein	OG_02809	LEC3_3901	transporter, major facilitator superfamily
OG_02810	LG3211_4468	LG3211_4468; alpha/beta hydrolase fold family protein	OG_02810	LEC3_4518	PGAP1-like protein
OG_02811	LG3211_5182	LG3211_5182; conserved hypothetical protein	OG_02811	LEC3_0104	conserved hypothetical protein
OG_02812	LG3211_3083	LG3211_3083; conserved hypothetical protein	OG_02812	LEC3_3076	transcription-related protein
OG_02813	LG3211_367	LG3211_367; conserved hypothetical protein	OG_02813	LEC3_0184	conserved hypothetical protein
OG_02814	LG3211_41	LG3211_41; hypothetical protein	OG_02814	LEC3_5283	hypothetical protein
OG_02815	LG3211_3000	LG3211_3000; poly(hydroxyalkanoate) granule-associated domain pr	OG_02815	LEC3_2992	possibly something SEQUENCING GAP
OG_02816	LG3211_1302	sufB; feS assembly protein SufB	OG_02816	LEC3_1260	FeS assembly protein SufB
OG_02817	LG3211_363	fabH; 3-oxoacyl-synthase III	OG_02817	LEC3_5012	3-oxoacyl-[acyl-carrier-protein (ACP)] synthase III C terminal
OG_02818	LG3211_1501	LG3211_1501; hypothetical protein	OG_02818	LEC3_1478	hypothetical protein
OG_02819	LG3211_3160	LG3211_3160; N-acetylmuramoyl-L-alanine amidase family protein	OG_02819	LEC3_3167	N-acetylmuramoyl-L-alanine amidase
OG_02820	LG3211_949	LG3211_949; radical SAM superfamily protein	OG_02820	LEC3_0904	oxygen-independent coproporphyrinogen III oxidase
OG_02821	LG3211_2201	nuoC; NADH (or F420H2) dehydrogenase, subunit C family protein	OG_02821	LEC3_2161	NADH (or F420H2) dehydrogenase, subunit C family protein
OG_02822	LG3211_1167	LG3211_1167; ycel-like domain protein	OG_02822	LEC3_1141	Ycel family protein
OG_02823	LG3211_2431	LG3211_2431; SURF1 family protein	OG_02823	LEC3_2373	exported SurF1-family protein
OG_02824	LG3211_5039	LG3211_5039; disulfide bond reductase	OG_02824	LEC3_4949	glutathione S-transferase
OG_02825	LG3211_2174	LG3211_2174; lysM domain protein	OG_02825	LEC3_2132	transglycosylase SLT domain/LysM domain
OG_02826	LG3211_4484	LG3211_4484; conserved hypothetical protein	OG_02826	LEC3_4536	conserved hypothetical protein
OG_02827	LG3211_3907	dgkA; dgkA	OG_02827	LEC3_3928	prokaryotic diacylglycerol kinase
OG_02828	LG3211_271	LG3211_271; putative trans-aconitate 2-methyltransferase	OG_02828	LEC3_5055	trans-aconitate 2-methyltransferase
OG_02829	LG3211_5113	LG3211_5113; polysaccharide biosynthesis family protein	OG_02829	LEC3_0173	NAD-dependent epimerase/dehydratase family protein
OG_02830	LG3211_2028	LG3211_2028; zinc-ribbon domain protein	OG_02830	LEC3_1960	conserved hypothetical protein
OG_02831	LG3211_70	LG3211_70; M42 glutamyl aminopeptidase family protein	OG_02831	LEC3_5275	peptidase, M28 family
OG_02832	LG3211_1725	LG3211_1725; transglutaminase-like superfamily protein	OG_02832	LEC3_1681	transglutaminase domain protein
OG_02833	LG3211_3923	LG3211_3923; RNA methyltransferase, RsmE family protein	OG_02833	LEC3_3947	conserved hypothetical protein
OG_02834	LG3211_822	cutA; copper binding protein CutA	OG_02834	LEC3_0772	periplasmic divalent cation tolerance protein CutA
OG_02835	LG3211_1397	LG3211_1397; phosphodiesterase-nucleotide pyrophosphatase	OG_02835	LEC3_1363	type I phosphodiesterase / nucleotide pyrophosphatase family protein
OG_02836	LG3211_1480	LG3211_1480; NADPH-dependent FMN reductase family protein	OG_02836	LEC3_1450	NADPH-dependent FMN reductase domain protein
OG_02837	LG3211_4974	LG3211_4974; cupin domain protein	OG_02837	LEC3_4885	cupin domain protein
OG_02838	LG3211_1280	LG3211_1280; sulfite reductase [NADPH] flavoprotein, alpha-compor	OG_02838	LEC3_1236	sulfite reductase [NADPH] flavoprotein, alpha-component
OG_02839	LG3211_1246	LG3211_1246; conserved hypothetical protein	OG_02839	LEC3_1201	conserved hypothetical protein
OG_02840	LG3211_1516	pcm; protein-L-isoaspartate O-methyltransferase	OG_02840	LEC3_1497	protein-L-isoaspartate O-methyltransferase
OG_02841	LG3211_1388	LG3211_1388; outer membrane beta-barrel domain protein	OG_02841	LEC3_1356	conserved hypothetical protein
OG_02842	LG3211_4541	LG3211_4541; D-ala-D-ala dipeptidase family protein	OG_02842	LEC3_4596	D-alanyl-D-alanine dipeptidase
OG_02843	LG3211_2140	LG3211_2140; conserved hypothetical protein	OG_02843	LEC3_2081	conserved hypothetical protein
OG_02844	LG3211_106	LG3211_106; hlyD secretion family protein	OG_02844	LEC3_5218	lipoprotein
OG_02845	LG3211_1349	LG3211_1349; sugar (and other) transporter family protein	OG_02845	LEC3_1304	transporter, major facilitator family
OG_02846	LG3211_2064	LG3211_2064; methyltransferase domain protein	OG_02846	LEC3_2001	conserved hypothetical protein
OG_02847	LG3211_2868	orn; oligoribonuclease	OG_02847	LEC3_2879	oligoribonuclease
OG_02848	LG3211_2256	LG3211_2256; conserved hypothetical protein	OG_02848	LEC3_2209	thiol-disulphide oxidoreductase DCC
OG_02849	LG3211_3343	LG3211_3343; conserved hypothetical protein	OG_02849	LEC3_3400	conserved hypothetical protein
OG_02850	LG3211_4934	LG3211_4934; O-Antigen ligase family protein	OG_02850	LEC3_0360	O-Antigen polymerase
OG_02851	LG3211_1918	pgaC; poly-beta-1,6 N-acetyl-D-glucosamine synthase	OG_02851	LEC3_1869	glycosyl transferase, group 2 family protein
OG_02852	LG3211_1941	LG3211_1941; aromatic amino acid lyase family protein	OG_02852	LEC3_1893	histidine ammonia-lyase
OG_02853	LG3211_801	LG3211_801; putative hydrolase	OG_02853	LEC3_0752	lysophospholipase
OG_02854	LG3211_2433	cyoC; cytochrome o ubiquinol oxidase, subunit III	OG_02854	LEC3_2375	cytochrome o ubiquinol oxidase, subunit III
OG_02855	LG3211_3266	uvrA; excinuclease ABC subunit A	OG_02855	LEC3_3297	excinuclease ABC, A subunit
OG_02856	LG3211_2673	kmo; kynurenine 3-monooxygenase	OG_02856	LEC3_2679	kynurenine 3-monooxygenase
OG_02857	LG3211_540	LG3211_540; metal-sensitive transcriptional repressor family protein	OG_02857	LEC3_4843	conserved hypothetical protein
OG_02858	LG3211_4083	ftsL; cell division protein FtsL	OG_02858	LEC3_4137	cell division protein FtsL
OG_02859	LG3211_3441	LG3211_3441; exopolysaccharide synthesis, ExoD family protein	OG_02859	LEC3_3504	exopolysaccharide synthesis, ExoD
OG_02860	LG3211_5320	LG3211_5320; efflux transporter, outer membrane factor (OMF) lipo,	OG_02860	LEC3_5414	efflux transporter, outer membrane factor (OMF) lipoprotein, NodT far
OG_02861	LG3211_2643	rplI; ribosomal protein L9	OG_02861	LEC3_2648	ribosomal protein L9

OG_02862	LG3211_5069	nikE; nickel import ATP-binding protein NikE	OG_02862	LEC3_4983	ABC transporter, ATP-binding protein
OG_02863	LG3211_1695	dnaQ; DNA polymerase III, epsilon subunit	OG_02863	LEC3_1661	DNA polymerase III, epsilon subunit
OG_02864	LG3211_3476	LG3211_3476; RNA methyltransferase, RsmD family	OG_02864	LEC3_3533	methyltransferase
OG_02865	LG3211_1128	LG3211_1128; tonB dependent receptor family protein	OG_02865	LEC3_1104	TonB-dependent receptor
OG_02866	LG3211_3259	cgtA; obg family GTPase CgtA	OG_02866	LEC3_3291	GTP-binding protein Obg/CgtA
OG_02867	LG3211_3917	LG3211_3917; hypothetical protein	OG_02867	LEC3_3941	hypothetical protein
OG_02868	LG3211_5042	LG3211_5042; conserved hypothetical protein	OG_02868	LEC3_4952	conserved hypothetical protein
OG_02869	LG3211_541	LG3211_541; S-(hydroxymethyl)glutathione dehydrogenase/class III a	OG_02869	LEC3_4842	S-(hydroxymethyl)glutathione dehydrogenase/class III alcohol dehydr
OG_02870	LG3211_3003	DBI; acyl-CoA-binding protein	OG_02870	LEC3_2996	peroxisomal 3,2-trans-enoyl-CoA isomerase
OG_02871	LG3211_1668	LG3211_1668; HAMP domain protein	OG_02871	LEC3_1629	sensor histidine kinase
OG_02872	LG3211_2424	LG3211_2424; bacterial regulatory helix-turn-helix, AraC family protei	OG_02872	LEC3_2368	transcriptional regulator, AraC family
OG_02873	LG3211_2748	LG3211_2748; conserved hypothetical protein	OG_02873	LEC3_2753	conserved hypothetical protein
OG_02874	LG3211_5159	glyS; glycyl-tRNA synthetase, &beta subunit	OG_02874	LEC3_0129	glycyl-tRNA synthetase, beta subunit
OG_02875	LG3211_3017	LG3211_3017; hypothetical protein	OG_02875	LEC3_3009	conserved hypothetical protein
OG_02876	LG3211_4092	LG3211_4092; FAD linked oxidase, C-terminal domain protein	OG_02876	LEC3_4146	D-lactate dehydrogenase
OG_02877	LG3211_522	ysxC; ribosome biogenesis GTP-binding protein YsxC	OG_02877	LEC3_0335	GTP-binding protein
OG_02878	LG3211_218	LG3211_218; type III secretion , HrcV family protein	OG_02878	LEC3_5112	type III secretion protein
OG_02879	LG3211_2244	LG3211_2244; conserved hypothetical protein	OG_02879	LEC3_2196	conserved hypothetical protein
OG_02880	LG3211_2152	LG3211_2152; beta-lactamase family protein	OG_02880	LEC3_2093	beta-lactamase
OG_02881	LG3211_1520	LG3211_1520; hypothetical protein	OG_02881	LEC3_1501	hypothetical protein
OG_02882	LG3211_755	bioD; dethiobiotin synthase	OG_02882	LEC3_0718	dethiobiotin synthase
OG_02883	LG3211_1443	LG3211_1443; bacterial regulatory helix-turn-helix , lysR family protei	OG_02883	LEC3_1412	transcriptional regulator, LysR family
OG_02884	LG3211_1419	LG3211_1419; bifunctional NMN adenylyltransferase/Nudix hydrolase	OG_02884	LEC3_1384	mutT/nudix family protein
OG_02885	LG3211_4633	LG3211_4633; UDP-N-acetylglucosamine 2-epimerase	OG_02885	LEC3_2255	UDP-N-acetylglucosamine 2-epimerase
OG_02886	LG3211_5091	moxR2; magnesium chelatase, putative	OG_02886	LEC3_4999	protein containing ATPase domain associated with various cellular ac
OG_02887	LG3211_1927	LG3211_1927; polysaccharide deacetylase family protein	OG_02887	LEC3_1877	Polysaccharide deacetylase
OG_02888	LG3211_1766	LG3211_1766; acyl-CoA dehydrogenase, C-terminal domain protein	OG_02888	LEC3_1722	protein AidB
OG_02889	LG3211_2418	LG3211_2418; putative osmotically inducible lipoprotein B	OG_02889	LEC3_2360	conserved domain protein
OG_02890	LG3211_1121	LG3211_1121; acyl-CoA dehydrogenase, N-terminal domain protein	OG_02890	LEC3_1096	acyl-CoA dehydrogenase, C-terminal domain
OG_02891	LG3211_2124	LG3211_2124; putative oxidoreductase	OG_02891	LEC3_2069	oxidoreductase family protein
OG_02892	LG3211_5117	rpmB; ribosomal protein L28	OG_02892	LEC3_0169	ribosomal protein L28
OG_02893	LG3211_2407	LG3211_2407; histidine phosphatase super family protein	OG_02893	LEC3_2349	phosphoglycerate mutase family protein
OG_02894	LG3211_172	LG3211_172; glucokinase family protein	OG_02894	LEC3_5159	glucokinase
OG_02895	LG3211_1745	LG3211_1745; enoyl-CoA hydratase/isomerase family protein	OG_02895	LEC3_1702	fatty acid oxidation complex subunit alpha
OG_02896	LG3211_2133	LG3211_2133; glycosyl transferases group 1 family protein	OG_02896	LEC3_2075	glycosyl transferase
OG_02897	LG3211_4467	LG3211_4467; conserved hypothetical protein	OG_02897	LEC3_4517	lipoprotein
OG_02898	LG3211_3477	ftsY; signal recognition particle-docking protein FtsY	OG_02898	LEC3_3534	signal recognition particle-docking protein FtsY
OG_02899	LG3211_4428	LG3211_4428; methylated-DNA-[]-cysteine S-methyltransferase famil	OG_02899	LEC3_4471	ada regulatory protein
OG_02900	LG3211_3727	LG3211_3727; conserved hypothetical protein	OG_02900	LEC3_3755	conserved hypothetical protein
OG_02901	LG3211_882	LG3211_882; phosphatidylethanolamine-binding family protein	OG_02901	LEC3_0831	conserved hypothetical protein
OG_02902	LG3211_3323	uppS; di-trans,poly-cis-decaprenylcistransferase	OG_02902	LEC3_3375	di-trans,poly-cis-decaprenylcistransferase
OG_02903	LG3211_1236	raiA; ribosomal subunit interface protein	OG_02903	LEC3_1191	ribosomal subunit interface protein
OG_02904	LG3211_3965	LG3211_3965; POTRA domain, ShlB-type family protein	OG_02904	LEC3_3988	POTRA domain, ShlB-type
OG_02905	LG3211_2946	LG3211_2946; doxX family protein	OG_02905	LEC3_2965	DoxX
OG_02906	LG3211_528	LG3211_528; tonB-dependent Receptor Plug domain protein	OG_02906	LEC3_0340	TonB-dependent receptor
OG_02907	LG3211_4509	LG3211_4509; tat (twin-arginine translocation) pathway signal sequer	OG_02907	LEC3_4564	aldehyde oxidase and xanthine dehydrogenase, molybdopterin bindir
OG_02908	LG3211_1539	LG3211_1539; conserved hypothetical protein	OG_02908	LEC3_1514	conserved hypothetical protein
OG_02909	LG3211_362	LG3211_362; conserved hypothetical protein	OG_02909	LEC3_5013	lipoprotein
OG_02910	LG3211_1779	LG3211_1779; aldehyde dehydrogenase family protein	OG_02910	LEC3_1742	succinate-semialdehyde dehydrogenase
OG_02911	LG3211_1124	LG3211_1124; RNA polymerase sigma factor, sigma-70 family proteir	OG_02911	LEC3_1100	RNA polymerase sigma-E factor
OG_02912	LG3211_1398	LG3211_1398; snoaL-like domain protein	OG_02912	LEC3_1364	conserved hypothetical protein
OG_02913	LG3211_214	LG3211_214; bacterial regulatory, luxR family protein	OG_02913	LEC3_5118	response regulator receiver domain
OG_02914	LG3211_1773	LG3211_1773; ABC transporter family protein	OG_02914	LEC3_1735	ABC transporter, permease/ATP-binding protein
OG_02915	LG3211_237	LG3211_237; glycosyl transferases group 1 family protein	OG_02915	LEC3_5093	glycosyl transferase, group 1 family protein
OG_02916	LG3211_1942	LG3211_1942; 2OG-Fe(II) oxygenase superfamily protein	OG_02916	LEC3_1894	oxidoreductase, 2OG-Fe(II) oxygenase family
OG_02917	LG3211_322	ntrC; nitrogen regulation protein NR	OG_02917	LEC3_5048	nitrogen regulation protein NR(I)
OG_02918	LG3211_3468	upp; uracil phosphoribosyltransferase	OG_02918	LEC3_3525	uracil phosphoribosyltransferase
OG_02919	LG3211_3851	LG3211_3851; 5'-3' exonuclease, C-terminal SAM fold family protein	OG_02919	LEC3_3872	DNA polymerase I
OG_02920	LG3211_4373	blaCTX-M-27; beta-lactamase CTX-M-27	OG_02920	LEC3_4415	beta-lactamase
OG_02921	LG3211_4347	LG3211_4347; bacterial regulatory helix-turn-helix , lysR family protei	OG_02921	LEC3_4389	transcriptional regulator, LysR family
OG_02922	LG3211_3106	LG3211_3106; rhomboid family protein	OG_02922	LEC3_3109	peptidase, S54 (rhomboid) family
OG_02923	LG3211_3351	LG3211_3351; conserved hypothetical protein	OG_02923	LEC3_3409	outer membrane protein
OG_02924	LG3211_4188	LG3211_4188; ABC-2 type transporter family protein	OG_02924	LEC3_4246	ABC-2 type transporter
OG_02925	LG3211_963	LG3211_963; tonB dependent receptor family protein	OG_02925	LEC3_0919	TonB-dependent receptor
OG_02926	LG3211_1680	ppk1; polyphosphate kinase 1	OG_02926	LEC3_1647	polyphosphate kinase
OG_02927	LG3211_4513	LG3211_4513; hypothetical protein	OG_02927	LEC3_4569	hypothetical protein
OG_02928	LG3211_156	LG3211_156; conserved hypothetical protein	OG_02928	LEC3_5170	conserved hypothetical protein
OG_02929	LG3211_4444	LG3211_4444; NAD(P)H binding domain of trans-2-enoyl-CoA reduct	OG_02929	LEC3_0533	short-chain alcohol dehydrogenase
OG_02930	LG3211_3342	LG3211_3342; tic20-like family protein	OG_02930	LEC3_3399	conserved hypothetical protein
OG_02931	LG3211_3642	LG3211_3642; conserved hypothetical protein	OG_02931	LEC3_3630	membrane protein
OG_02932	LG3211_1509	ispD; 4-diphosphocytidyl-2C-methyl-D-erythritol synthetase	OG_02932	LEC3_1490	2-C-methyl-D-erythritol 4-phosphate cytidylyltransferase
OG_02933	LG3211_4920	LG3211_4920; N-acylglucosamine 2-epimerase family protein	OG_02933	LEC3_0374	N-acylglucosamine 2-epimerase family protein
OG_02934	LG3211_5103	xth; exodeoxyribonuclease III	OG_02934	LEC3_5009	exodeoxyribonuclease III
OG_02935	LG3211_4079	mraY; phospho-N-acetylmuramoyl-pentapeptide- transferase	OG_02935	LEC3_4133	phospho-N-acetylmuramoyl-pentapeptide-transferas e
OG_02936	LG3211_5316	LG3211_5316; bacterial DNA-binding family protein	OG_02936	LEC3_5418	DNA-binding protein
OG_02937	LG3211_2758	pgmB; beta-phosphoglucomutase	OG_02937	LEC3_2764	beta-phosphoglucomutase
OG_02938	LG3211_3196	recA; protein RecA	OG_02938	LEC3_3203	protein RecA
OG_02939	LG3211_5211	rnaSA3; guanyl-specific ribonuclease Sa3	OG_02939	LEC3_0078	guanyl-specific ribonuclease Sa3 (RNase Sa3)
OG_02940	LG3211_5342	LG3211_5342; conserved hypothetical protein	OG_02940	LEC3_5379	conserved hypothetical protein
OG_02941	LG3211_267	LG3211_267; peptidase S51 family protein	OG_02941	LEC3_5060	putative cyanophycinase
OG_02942	LG3211_2913	cmk; cytidylate kinase	OG_02942	LEC3_2926	cytidylate kinase

OG_02943	LG3211_3621	LG3211_3621; metallo-beta-lactamase superfamily protein	OG_02943	LEC3_3612	metallo-beta-lactamase domain protein
OG_02944	LG3211_3940	LG3211_3940; tonB family C-terminal domain protein	OG_02944	LEC3_3965	TonB family C-terminal domain
OG_02945	LG3211_1744	pilZ; type IV fimbriae assembly protein	OG_02945	LEC3_1699	type IV pilus assembly protein
OG_02946	LG3211_5200	LG3211_5200; thymidine kinase family protein	OG_02946	LEC3_0089	thymidine kinase
OG_02947	LG3211_3609	LG3211_3609; conserved hypothetical protein	OG_02947	LEC3_3603	lytic murein transglycosylase
OG_02948	LG3211_5323	LG3211_5323; amino acid permease family protein	OG_02948	LEC3_5407	amino acid permease
OG_02949	LG3211_2356	LG3211_2356; NUDIX hydrolase	OG_02949	LEC3_2302	hydrolase, NUDIX family
OG_02950	LG3211_2330	LG3211_2330; alpha-1,2-mannosidase family protein	OG_02950	LEC3_2270	alpha-1,2-mannosidase, putative
OG_02951	LG3211_694	LG3211_694; methyltransferase small domain protein	OG_02951	LEC3_0660	ribosomal RNA small subunit methyltransferase C
OG_02952	LG3211_2694	metH; methionine synthase	OG_02952	LEC3_2695	methionine synthase
OG_02953	LG3211_3399	LG3211_3399; conserved hypothetical protein	OG_02953	LEC3_3455	ounterparts found in phytopathogens
OG_02954	LG3211_2777	LG3211_2777; carboxypeptidase regulatory-like domain protein	OG_02954	LEC3_2784	TonB-dependent outer membrane receptor
OG_02955	LG3211_805	LG3211_805; CDP-alcohol phosphatidyltransferase family protein	OG_02955	LEC3_0756	conserved hypothetical protein
OG_02956	LG3211_5334	LG3211_5334; MG2 domain protein	OG_02956	LEC3_5394	lipoprotein
OG_02957	LG3211_3315	tsf; translation elongation factor Ts	OG_02957	LEC3_3363	translation elongation factor Ts
OG_02958	LG3211_1644	LG3211_1644; hypothetical protein	OG_02958	LEC3_1616	hypothetical protein
OG_02959	LG3211_131	zwf; glucose-6-phosphate dehydrogenase	OG_02959	LEC3_5193	glucose-6-phosphate 1-dehydrogenase
OG_02960	LG3211_4259	trpS; tryptophan--tRNA ligase	OG_02960	LEC3_4329	tryptophanyl-tRNA synthetase
OG_02961	LG3211_2790	LG3211_2790; hydrolase	OG_02961	LEC3_2796	hydrolase
OG_02962	LG3211_1065	LG3211_1065; prolyl oligopeptidase family protein	OG_02962	LEC3_1034	peptidase, S9A/B/C families, catalytic domain
OG_02963	LG3211_377	LG3211_377; bacterial regulatory helix-turn-helix , lysR family protein	OG_02963	LEC3_0198	transcriptional regulator, LysR family
OG_02964	LG3211_3311	dapD; 2,3,4,5-tetrahydropyridine-2,6-dicarboxylate N-succinyltransfer	OG_02964	LEC3_3358	2,3,4,5-tetrahydropyridine-2,6-dicarboxylate N-succinyltransferase
OG_02965	LG3211_43	LG3211_43; impB/mucB/samB family protein	OG_02965	LEC3_5281	DNA polymerase IV
OG_02966	LG3211_2311	LG3211_2311; transglutaminase-like superfamily protein	OG_02966	LEC3_2235	conserved hypothetical protein
OG_02967	LG3211_4167	LG3211_4167; electron transfer flavodomain protein	OG_02967	LEC3_4234	electron transfer flavoprotein subunit alpha
OG_02968	LG3211_3951	LG3211_3951; glycosyl transferase 2 family protein	OG_02968	LEC3_3976	glycosyl transferase
OG_02969	LG3211_5304	LG3211_5304; glutathionylspermidine synthase preATP-grasp family	OG_02969	LEC3_5436	glutathionylspermidine synthase
OG_02970	LG3211_4228	rpe; ribulose-phosphate 3-epimerase	OG_02970	LEC3_4288	ribulose-phosphate 3-epimerase
OG_02971	LG3211_3148	dcd; deoxycytidine triphosphate deaminase	OG_02971	LEC3_3156	deoxycytidine triphosphate deaminase
OG_02972	LG3211_4959	priA; primosomal protein N'	OG_02972	LEC3_4865	primosomal protein N'
OG_02973	LG3211_1057	ilvE; branched-chain amino acid aminotransferase	OG_02973	LEC3_1025	branched-chain amino acid aminotransferase
OG_02974	LG3211_2847	phaC; poly(R)-hydroxyalkanoic acid synthase, class III, PhaC subunit	OG_02974	LEC3_2858	poly(R)-hydroxyalkanoic acid synthase, class III, PhaC subunit
OG_02975	LG3211_3330	lpxA; acyl-[acyl-carrier-protein]-UDP-N- acetylglucosamine O-acyltrar	OG_02975	LEC3_3382	acyl-[acyl-carrier-protein]-UDP-N- acetylglucosamine O-acyltransfera
OG_02976	LG3211_1974	LG3211_1974; acetyltransferase family protein	OG_02976	LEC3_1929	acetyltransferase (GNAT) family
OG_02977	LG3211_1247	purN; phosphoribosylglycinamide formyltransferase	OG_02977	LEC3_1202	phosphoribosylglycinamide formyltransferase SEQUENCING GAP
OG_02978	LG3211_4230	purC; phosphoribosylaminoimidazolesuccinocarboxamide synthase	OG_02978	LEC3_4290	phosphoribosylaminoimidazole-succinocarboxamide synthase
OG_02979	LG3211_4704	LG3211_4704; conserved hypothetical protein	OG_02979	LEC3_0522	conserved hypothetical protein
OG_02980	LG3211_2339	rpfC; sensory/regulatory protein rpfC	OG_02980	LEC3_2282	sensory/regulatory protein RpfC
OG_02981	LG3211_523	LG3211_523; tetratricopeptide repeat family protein	OG_02981	LEC3_0336	tetratricopeptide repeat domain protein
OG_02982	LG3211_1761	LG3211_1761; CYTH domain protein	OG_02982	LEC3_1717	adenylate cyclase
OG_02983	LG3211_1166	LG3211_1166; prokaryotic cytochrome b561 family protein	OG_02983	LEC3_1140	cytochrome B561
OG_02984	LG3211_2416	ligD; DNA ligase D	OG_02984	LEC3_3698	DNA ligase D
OG_02985	LG3211_45	LG3211_45; acetyltransferase family protein	OG_02985	LEC3_5246	N-acetyltransferase
OG_02986	LG3211_1860	LG3211_1860; peptidase M48 family protein	OG_02986	LEC3_1804	peptidase, M48 family
OG_02987	LG3211_1116	wrbA; quinone oxidoreductase, type IV	OG_02987	LEC3_1091	NAD(P)H:quinone oxidoreductase, type IV
OG_02988	LG3211_1035	LG3211_1035; fumarylacetoacetate (FAA) hydrolase family protein	OG_02988	LEC3_1001	fumarylacetoacetate hydrolase domain-containing protein 1
OG_02989	LG3211_3144	LG3211_3144; conserved hypothetical protein	OG_02989	LEC3_3152	membrane protein
OG_02990	LG3211_5024	LG3211_5024; prolyl oligopeptidase family protein	OG_02990	LEC3_4937	dipeptidyl peptidase IV/S9A/B/C peptidase domain protein
OG_02992	LG3211_3800	ppk2; polyphosphate kinase 2	OG_02992	LEC3_3822	Polyphosphate kinase 2
OG_02993	LG3211_676	LG3211_676; polysaccharide deacetylase family protein	OG_02993	LEC3_4673	polysaccharide deacetylase
OG_02994	LG3211_2661	LG3211_2661; conserved hypothetical protein	OG_02994	LEC3_2667	conserved hypothetical protein
OG_02995	LG3211_4615	LG3211_4615; hypothetical protein	OG_02995	LEC3_2847	hypothetical protein
OG_02996	LG3211_1444	LG3211_1444; NAD-dependent epimerase/dehydratase	OG_02996	LEC3_1413	NAD dependent epimerase/dehydratase family
OG_02997	LG3211_2674	LG3211_2674; exonuclease family protein	OG_02997	LEC3_2680	exodeoxyribonuclease I
OG_02998	LG3211_2097	ampR; HTH-type transcriptional activator AmpR	OG_02998	LEC3_2035	HTH-type transcriptional activator AmpR
OG_02999	LG3211_4258	LG3211_4258; putative transmembrane protein	OG_02999	LEC3_4328	hypothetical protein
OG_03000	LG3211_3314	rpsB; ribosomal protein S2	OG_03000	LEC3_3362	ribosomal protein S2
OG_03001	LG3211_5171	LG3211_5171; alpha/beta hydrolase fold family protein	OG_03001	LEC3_0117	hydrolase, alpha/beta fold family
OG_03002	LG3211_4021	cobO; cob(I)yrinic acid a,c-diamide adenosyltransferase	OG_03002	LEC3_4089	cob(I)yrinic acid a,c-diamide adenosyltransferase
OG_03003	LG3211_3117	LG3211_3117; hypothetical protein	OG_03003	LEC3_3117	hypothetical protein
OG_03004	LG3211_3438	LG3211_3438; conserved hypothetical protein	OG_03004	LEC3_3501	conserved hypothetical protein
OG_03005	LG3211_4642	LG3211_4642; glycosyl transferases group 1 family protein	OG_03005	LEC3_2246	glycosyl transferase, group 1 family protein
OG_03006	LG3211_5115	LG3211_5115; lipid A Biosynthesis N-terminal domain protein	OG_03006	LEC3_0171	lipid A Biosynthesis N-terminal domain
OG_03007	LG3211_504	LG3211_504; S-adenosyl-L-methionine-dependent methyltransferase	OG_03007	LEC3_0316	conserved hypothetical protein
OG_03008	LG3211_1751	lepB; signal peptidase I	OG_03008	LEC3_1708	signal peptidase I
OG_03009	LG3211_4274	LG3211_4274; HAMP domain protein	OG_03009	LEC3_4341	sensor histidine kinase
OG_03010	LG3211_1692	gloB; hydroxyacylglutathione hydrolase	OG_03010	LEC3_1658	hydroxyacylglutathione hydrolase
OG_03011	LG3211_544	LG3211_544; acrB/AcrD/AcrF family protein	OG_03011	LEC3_4838	acriflavin resistance protein
OG_03012	LG3211_3255	ileS; isoleucine--tRNA ligase	OG_03012	LEC3_3286	isoleucyl-tRNA synthetase
OG_03013	LG3211_796	purD; phosphoribosylamine--glycine ligase	OG_03013	LEC3_0745	phosphoribosylamine--glycine ligase
OG_03014	LG3211_3067	LG3211_3067; cytochrome C' family protein	OG_03014	LEC3_3060	cytochrome C precusor
OG_03015	LG3211_4270	plsY; acyl-phosphate glycerol 3-phosphate acyltransferase	OG_03015	LEC3_4337	UPF0078 membrane protein SEQUENCING GAP
OG_03016	LG3211_1264	purM; phosphoribosylformylglycinamidine cyclo-ligase	OG_03016	LEC3_1220	phosphoribosylformylglycinamidine cyclo-ligase
OG_03017	LG3211_873	LG3211_873; ACT domain protein	OG_03017	LEC3_0822	conserved hypothetical protein
OG_03018	LG3211_373	LG3211_373; conserved hypothetical protein	OG_03018	LEC3_0191	conserved hypothetical protein
OG_03019	LG3211_2860	LG3211_2860; hypothetical protein	OG_03019	LEC3_2872	hypothetical protein
OG_03020	LG3211_1742	tmk; thymidylate kinase	OG_03020	LEC3_1697	thymidylate kinase
OG_03021	LG3211_3910	LG3211_3910; ABC transporter family protein	OG_03021	LEC3_3932	ABC transporter, ATP-binding protein
OG_03022	LG3211_1053	LG3211_1053; alpha-L-glutamate ligases, RimK family protein	OG_03022	LEC3_1022	ribosomal protein S6 modification protein
OG_03023	LG3211_833	groES; 10 kDa chaperonin	OG_03023	LEC3_0783	chaperonin GroS
OG_03024	LG3211_105	LG3211_105; ftsX-like permease family protein	OG_03024	LEC3_5219	efflux ABC transporter, permease protein

OG_03025	LG3211_2660	LG3211_2660; AAA domain family protein
OG_03026	LG3211_2705	LG3211_2705; glutathione peroxidase family protein
OG_03027	LG3211_3185	kdpA; K+-transporting ATPase, A subunit
OG_03028	LG3211_1708	moaC; molybdenum cofactor biosynthesis protein C
OG_03029	LG3211_4158	ETFDH; electron transfer flavoprotein-ubiquinone oxidoreductase, mit
OG_03030	LG3211_3393	LG3211_3393; putative translation elongation factor P
OG_03031	LG3211_1078	LG3211_1078; pepSY-associated TM helix family protein
OG_03032	LG3211_1760	rumA; 23S rRNA (uracil-5-)-methyltransferase RumA
OG_03033	LG3211_339	LG3211_339; rhodanese-like domain protein
OG_03034	LG3211_4156	LG3211_4156; 2OG-Fe(II) oxygenase superfamily protein
OG_03035	LG3211_2879	guaB; inosine-5'-monophosphate dehydrogenase
OG_03036	LG3211_1681	phoR; phosphate regulon sensor kinase PhoR
OG_03037	LG3211_1606	LG3211_1606; conserved hypothetical protein
OG_03038	LG3211_1356	LG3211_1356; pseudouridine synthase family protein
OG_03039	LG3211_3194	alaS; alanine--tRNA ligase
OG_03040	LG3211_1413	LG3211_1413; acetyltransferase family protein
OG_03041	LG3211_5061	LG3211_5061; conserved hypothetical protein
OG_03042	LG3211_908	LG3211_908; pyrophosphate-dependent phosphofructokinase
OG_03043	LG3211_3020	LG3211_3020; polysaccharide biosynthesis family protein
OG_03044	LG3211_4557	LG3211_4557; 4-hydroxyproline epimerase
OG_03045	LG3211_1916	LG3211_1916; hydrolase CocE/NonD family protein
OG_03046	LG3211_3533	pheT; phenylalanine--tRNA ligase, beta subunit
OG_03047	LG3211_5014	LG3211_5014; tonB family C-terminal domain protein
OG_03048	LG3211_5167	LG3211_5167; conserved hypothetical protein
OG_03049	LG3211_2114	chiG; secreted chitinase domain protein
OG_03050	LG3211_719	atpC; ATP synthase F1, epsilon subunit
OG_03051	LG3211_185	LG3211_185; META domain protein
OG_03052	LG3211_2197	secG; preprotein translocase, SecG subunit
OG_03053	LG3211_681	LG3211_681; killing trait family protein
OG_03054	LG3211_2846	LG3211_2846; GDSL-like Lipase/Acylhydrolase family protein
OG_03056	LG3211_2043	LG3211_2043; YXWGXW repeat family protein
OG_03057	LG3211_1212	LG3211_1212; twitching motility family protein
OG_03058	LG3211_164	LG3211_164; ankyrin repeat family protein
OG_03059	LG3211_3270	LG3211_3270; enoyl-CoA hydratase/isomerase family protein
OG_03060	LG3211_1335	LG3211_1335; conserved hypothetical protein
OG_03061	LG3211_3842	LG3211_3842; putative NAD
OG_03062	LG3211_1182	LG3211_1182; dodecin family protein
OG_03063	LG3211_2366	aat; leucyl/phenylalanyl-tRNA--protein transferase
OG_03064	LG3211_1393	LG3211_1393; methylated-DNA-[]-cysteine S-methyltransferase famil
OG_03065	LG3211_3531	LG3211_3531; merR regulatory family protein
OG_03066	LG3211_1283	LG3211_1283; putative transglycosylase-associated protein
OG_03067	LG3211_166	LG3211_166; response regulator
OG_03068	LG3211_2722	sucB; dihydrolipoyllysine-residue succinyltransferase, E2 component
OG_03069	LG3211_3370	LG3211_3370; conserved hypothetical protein
OG_03070	LG3211_4514	LG3211_4514; bacterial regulatory helix-turn-helix , lysR family protei
OG_03071	LG3211_2268	LG3211_2268; enoyl-CoA hydratase/isomerase family protein
OG_03072	LG3211_4194	uptE; outer membrane domain protein
OG_03073	LG3211_3337	accA; acetyl-CoA carboxylase, carboxyl transferase, alpha subunit
OG_03074	LG3211_5359	LG3211_5359; conserved hypothetical protein
OG_03075	LG3211_4195	LG3211_4195; conserved hypothetical protein
OG_03076	LG3211_2115	LG3211_2115; hypothetical protein
OG_03077	LG3211_3243	LG3211_3243; glutaredoxin family protein
OG_03078	LG3211_3203	LG3211_3203; tonB dependent receptor family protein
OG_03079	LG3211_4190	cysA; cystathionine gamma-lyase
OG_03080	LG3211_1801	ALDH7A1; alpha-aminoadipic semialdehyde dehydrogenase
OG_03081	LG3211_3135	LG3211_3135; histidine triad (HIT) protein
OG_03082	LG3211_1727	LG3211_1727; AAA domain family protein
OG_03083	LG3211_3377	nadD; nicotinate (nicotinamide) nucleotide adenylyltransferase
OG_03084	LG3211_1135	LG3211_1135; asmA family protein
OG_03085	LG3211_3763	rplC; 50S ribosomal protein L3
OG_03086	LG3211_3264	LG3211_3264; ompA family protein

OG_03025	LEC3_2666	methanol dehydrogenase regulator
OG_03026	LEC3_2706	glutathione peroxidase/vitamin B12 transport periplasmic protein btuE
OG_03027	LEC3_3192	K+-transporting ATPase, A subunit
OG_03028	LEC3_1670	molybdenum cofactor biosynthesis protein C
OG_03029	LEC3_4223	flavoprotein-ubiquinone oxidoreductase
OG_03030	LEC3_3448	conserved hypothetical protein
OG_03031	LEC3_1051	PepSY-associated TM helix
OG_03032	LEC3_1716	23S rRNA (uracil-5-)-methyltransferase RumA
OG_03033	LEC3_5032	rhodanese domain protein
OG_03034	LEC3_4221	oxidoreductase, 2OG-Fe(II) oxygenase family
OG_03035	LEC3_2894	inosine-5'-monophosphate dehydrogenase
OG_03036	LEC3_1648	phosphate regulon sensor kinase PhoR
OG_03037	LEC3_1581	conserved hypothetical protein
OG_03038	LEC3_1314	RNA pseudouridine synthase family
OG_03039	LEC3_3200	alanyl-tRNA synthetase
OG_03040	LEC3_1378	acetyltransferase, GNAT family
OG_03041	LEC3_4977	conserved hypothetical protein
OG_03042	LEC3_0856	6-phosphofructokinase
OG_03043	LEC3_3013	NAD dependent epimerase/dehydratase family protein
OG_03044	LEC3_4612	proline racemase
OG_03045	LEC3_1859	hydrolase, CocE/NonD family
OG_03046	LEC3_3564	phenylalanyl-tRNA synthetase, beta subunit
OG_03047	LEC3_4928	TonB family C-terminal domain
OG_03048	LEC3_0121	conserved hypothetical protein
OG_03049	LEC3_2053	endochitinase A
OG_03050	LEC3_0690	ATP synthase F1, epsilon subunit
OG_03051	LEC3_5147	conserved hypothetical protein
OG_03052	LEC3_2158	preprotein translocase, SecG subunit
OG_03053	LEC3_4668	conserved domain protein
OG_03054	LEC3_2857	lipolytic enzyme, G-D-S-L family
OG_03056	LEC3_1978	conserved hypothetical protein
OG_03057	LEC3_1164	Type IV pilus assembly protein
OG_03058	LEC3_5166	phospholipase accessory protein
OG_03059	LEC3_3302	enoyl-CoA hydratase/isomerase family protein
OG_03060	LEC3_1289	2-methylcitrate synthase
OG_03061	LEC3_3865	NAD(P) transhydrogenase, alpha subunit
OG_03062	LEC3_1152	conserved domain protein
OG_03063	LEC3_2312	leucyl/phenylalanyl-tRNA--protein transferase
OG_03064	LEC3_1361	methylated-DNA--protein-cysteine methyltransferase
OG_03065	LEC3_3562	transcriptional regulator, MerR family
OG_03066	LEC3_1239	conserved hypothetical protein
OG_03067	LEC3_5164	response regulator protein
OG_03068	LEC3_2724	dihydrolipoyllysine-residue succinyltransferase, E2 component
OG_03069	LEC3_4424	hypothetical protein
OG_03070	LEC3_4570	transcriptional regulator, LysR family
OG_03071	LEC3_0578	enoyl-CoA hydratase
OG_03072	LEC3_4253	outer membrane protein
OG_03073	LEC3_3390	acetyl-CoA carboxylase, carboxyl transferase, alpha subunit
OG_03074	LEC3_5361	membrane protein
OG_03075	LEC3_4254	outer membrane protein
OG_03076	LEC3_2054	lipoprotein
OG_03077	LEC3_3273	glutaredoxin
OG_03078	LEC3_3209	TonB-dependent receptor
OG_03079	LEC3_4248	methionine gamma-lyase
OG_03080	LEC3_1765	piperideine-6-carboxylate dehydrogenase
OG_03081	LEC3_3139	HIT family hydrolase
OG_03082	LEC3_1683	methanol dehydrogenase regulator
OG_03083	LEC3_3434	nicotinate nucleotide adenylyltransferase
OG_03084	LEC3_1114	membrane protein, AsmA family
OG_03085	LEC3_3790	ribosomal protein L3
OG_03086	LEC3_3296	OmpA/MotB family protein

Table S2: CDSs unique to each *Lysobacter strain*.

Orthologous group	*L. ant* ATCC29479<br>GeneID (#491)<br>9.5% of genome	Gene description
OG_00019	LA29479_1325	hypothetical protein
OG_00019	LA29479_3184	hypothetical protein
OG_00019	LA29479_769	hypothetical protein
OG_00019	LA29479_3800	hypothetical protein
OG_00019	LA29479_127	hypothetical protein
OG_00019	LA29479_2899	hypothetical protein
OG_00019	LA29479_1082	hypothetical protein
OG_00019	LA29479_1439	hypothetical protein
OG_00019	LA29479_4974	hypothetical protein
OG_00019	LA29479_4616	hypothetical protein
OG_00019	LA29479_4352	hypothetical protein
OG_00019	LA29479_1007	hypothetical protein
OG_00019	LA29479_1959	hypothetical protein
OG_00019	LA29479_5037	hypothetical protein
OG_00019	LA29479_5147	hypothetical protein
OG_03983	LA29479_557	hypothetical protein
OG_03983	LA29479_3008	hypothetical protein
OG_03983	LA29479_1081	hypothetical protein
OG_04630	LA29479_3767	addiction module antidote protein, HigA family
OG_04630	LA29479_5177	addiction module antidote protein, HigA family
OG_04631	LA29479_5178	plasmid maintenance system killer family protein
OG_04631	LA29479_3768	plasmid maintenance system killer family protein
OG_04632	LA29479_5176	putative uncharacterized protein
OG_04632	LA29479_3766	putative uncharacterized protein
OG_04633	LA29479_5077	hypothetical protein
OG_04633	LA29479_5114	hypothetical protein
OG_04634	LA29479_1531	hypothetical protein
OG_04634	LA29479_882	hypothetical protein
OG_04635	LA29479_5121	protein
OG_04635	LA29479_2357	protein
OG_04636	LA29479_1145	hypothetical protein
OG_04636	LA29479_1125	hypothetical protein
OG_04637	LA29479_4664	putative transposase
OG_04637	LA29479_4657	putative isxal5 transposase protein
OG_04638	LA29479_4901	hypothetical protein
OG_04638	LA29479_3556	hypothetical protein
OG_04639	LA29479_5174	putative membrane protein
OG_04639	LA29479_3764	putative membrane protein
OG_04640	LA29479_2208	putative uncharacterized domain protein
OG_04640	LA29479_5160	putative uncharacterized domain protein
OG_04641	LA29479_1015	ompA family protein
OG_04641	LA29479_5230	ompA family protein
OG_04642	LA29479_5078	putative hydrolase domain protein
OG_04642	LA29479_5113	putative hydrolase domain protein
OG_04643	LA29479_579	hypothetical protein
OG_04643	LA29479_4318	hypothetical protein
OG_04644	LA29479_162	hypothetical protein
OG_04644	LA29479_160	hypothetical protein
OG_04645	LA29479_2127	hypothetical protein
OG_04645	LA29479_4795	hypothetical protein
OG_04646	LA29479_4649	conserved repeat domain protein
OG_04646	LA29479_2209	conserved repeat domain protein
OG_04647	LA29479_3695	hypothetical protein
OG_04647	LA29479_5090	hypothetical protein
OG_06110	LA29479_1454	hypothetical protein
OG_06111	LA29479_2	putative lipoprotein
OG_06112	LA29479_16	hypothetical protein
OG_06113	LA29479_17	helicase domain protein
OG_06114	LA29479_18	hypothetical protein
OG_06115	LA29479_21	hypothetical protein
OG_06116	LA29479_25	transposase
OG_06117	LA29479_26	nucleoside-diphosphate-sugar epimerase domain protein
OG_06118	LA29479_31	hypothetical protein
OG_06119	LA29479_51	hypothetical protein
OG_06120	LA29479_122	hypothetical protein
OG_06121	LA29479_126	hypothetical protein
OG_06122	LA29479_140	hypothetical protein

Orthologous group	*L. ant* 76<br>GeneID (#3214)<br>7.5% of genome	Gene description
OG_04629	LA76x_4975	LA76x_4975; transposase IS116/IS110/IS902 family protein
OG_06106	LA76x_3217	LA76x_3217; hypothetical protein
OG_06107	LA76x_4200	LA76x_4200; hypothetical protein
OG_06108	LA76x_3728	LA76x_3728; hypothetical protein
OG_08091	LA76x_5	LA76x_5; CAAX protease self-immunity family protein
OG_08092	LA76x_6	LA76x_6; peptidase M48 family protein
OG_08093	LA76x_34	LA76x_34; hypothetical protein
OG_08094	LA76x_98	LA76x_98; hypothetical protein
OG_08095	LA76x_107	LA76x_107; hypothetical protein
OG_08096	LA76x_116	LA76x_116; hypothetical protein
OG_08097	LA76x_128	LA76x_128; hypothetical protein
OG_08098	LA76x_168	LA76x_168; hypothetical protein
OG_08099	LA76x_204	LA76x_204; hypothetical protein
OG_08100	LA76x_210	LA76x_210; hypothetical protein
OG_08101	LA76x_240	dltA; D-alanine--poly(phosphoribitol) ligase, subunit 1
OG_08102	LA76x_280	LA76x_280; hypothetical protein
OG_08103	LA76x_284	LA76x_284; hypothetical protein
OG_08104	LA76x_293	LA76x_293; hypothetical protein
OG_08105	LA76x_294	LA76x_294; hypothetical protein
OG_08106	LA76x_319	dhbF; dimodular nonribosomal peptide synthase
OG_08107	LA76x_320	LA76x_320; hypothetical protein
OG_08108	LA76x_377	LA76x_377; hypothetical protein
OG_08109	LA76x_384	LA76x_384; AAA ATPase domain protein
OG_08110	LA76x_385	LA76x_385; uvrD/REP helicase N-terminal domain protein
OG_08111	LA76x_386	LA76x_386; hypothetical protein
OG_08112	LA76x_387	LA76x_387; hypothetical protein
OG_08113	LA76x_389	LA76x_389; lacI family regulatory protein
OG_08114	LA76x_390	LA76x_390; hypothetical protein
OG_08115	LA76x_397	LA76x_397; conserved hypothetical protein
OG_08116	LA76x_407	LA76x_407; hypothetical protein
OG_08117	LA76x_409	LA76x_409; hypothetical protein
OG_08118	LA76x_411	LA76x_411; site-specific DNA methylase domain protein
OG_08119	LA76x_417	LA76x_417; hypothetical protein
OG_08120	LA76x_418	LA76x_418; hypothetical protein
OG_08121	LA76x_419	LA76x_419; hypothetical protein
OG_08122	LA76x_420	LA76x_420; hypothetical protein
OG_08123	LA76x_433	LA76x_433; hypothetical protein
OG_08124	LA76x_454	LA76x_454; hypothetical protein
OG_08125	LA76x_456	LA76x_456; hypothetical protein
OG_08126	LA76x_458	LA76x_458; autotransporter-associated beta strand repeat family pro
OG_08127	LA76x_460	LA76x_460; hypothetical protein
OG_08128	LA76x_465	LA76x_465; hypothetical protein
OG_08129	LA76x_468	LA76x_468; hypothetical protein
OG_08130	LA76x_469	LA76x_469; hypothetical protein
OG_08131	LA76x_470	LA76x_470; RHS Repeat family protein
OG_08132	LA76x_471	LA76x_471; hypothetical protein
OG_08133	LA76x_473	LA76x_473; hypothetical protein
OG_08134	LA76x_484	LA76x_484; hypothetical protein
OG_08135	LA76x_487	LA76x_487; hypothetical protein
OG_08136	LA76x_492	LA76x_492; hypothetical protein
OG_08137	LA76x_493	LA76x_493; hypothetical protein
OG_08138	LA76x_515	LA76x_515; hypothetical protein
OG_08139	LA76x_516	LA76x_516; bacterial pre-peptidase C-terminal domain protein
OG_08140	LA76x_522	LA76x_522; hypothetical protein
OG_08141	LA76x_523	LA76x_523; hypothetical protein
OG_08142	LA76x_542	LA76x_542; hypothetical protein
OG_08143	LA76x_543	LA76x_543; hypothetical protein
OG_08144	LA76x_544	LA76x_544; hypothetical protein
OG_08145	LA76x_576	LA76x_576; hypothetical protein
OG_08146	LA76x_590	LA76x_590; hypothetical protein
OG_08147	LA76x_597	LA76x_597; hypothetical protein
OG_08148	LA76x_606	LA76x_606; NAD dependent epimerase/dehydratase family protein
OG_08149	LA76x_609	LA76x_609; hypothetical protein
OG_08150	LA76x_632	LA76x_632; hypothetical protein
OG_08151	LA76x_634	LA76x_634; hypothetical protein
OG_08152	LA76x_701	LA76x_701; hypothetical protein
OG_08153	LA76x_708	LA76x_708; hypothetical protein

OG_06123	LA29479_156	hypothetical protein
OG_06124	LA29479_161	hypothetical protein
OG_06125	LA29479_187	hypothetical protein
OG_06126	LA29479_210	hypothetical protein
OG_06127	LA29479_267	hypothetical protein
OG_06128	LA29479_284	hypothetical protein
OG_06129	LA29479_295	ferrichrome-iron receptor domain protein
OG_06130	LA29479_308	hypothetical protein
OG_06131	LA29479_372	hypothetical protein
OG_06132	LA29479_391	hypothetical protein
OG_06133	LA29479_395	hypothetical protein
OG_06134	LA29479_399	hypothetical protein
OG_06135	LA29479_402	hypothetical protein
OG_06136	LA29479_403	hypothetical protein
OG_06137	LA29479_408	hypothetical protein
OG_06138	LA29479_411	hypothetical protein
OG_06139	LA29479_415	hypothetical protein
OG_06140	LA29479_417	hypothetical protein
OG_06141	LA29479_422	hypothetical protein
OG_06142	LA29479_423	hypothetical protein
OG_06143	LA29479_445	hypothetical protein
OG_06144	LA29479_447	putative membrane protein
OG_06145	LA29479_482	hypothetical protein
OG_06146	LA29479_490	hypothetical protein
OG_06147	LA29479_499	putative uncharacterized protein
OG_06148	LA29479_511	hypothetical protein
OG_06149	LA29479_519	hypothetical protein
OG_06150	LA29479_571	hypothetical protein
OG_06151	LA29479_576	hypothetical protein
OG_06152	LA29479_589	hypothetical protein
OG_06153	LA29479_608	putative uncharacterized protein
OG_06154	LA29479_609	helix-turn-helix family protein
OG_06155	LA29479_610	hypothetical protein
OG_06156	LA29479_611	hypothetical protein
OG_06157	LA29479_612	putative uncharacterized domain protein
OG_06158	LA29479_613	hypothetical protein
OG_06159	LA29479_614	hypothetical protein
OG_06160	LA29479_615	hypothetical protein
OG_06161	LA29479_624	hypothetical protein
OG_06162	LA29479_650	hypothetical protein
OG_06163	LA29479_654	hypothetical protein
OG_06164	LA29479_687	hypothetical protein
OG_06165	LA29479_694	hypothetical protein
OG_06166	LA29479_696	hypothetical protein
OG_06167	LA29479_720	hypothetical protein
OG_06168	LA29479_728	hypothetical protein
OG_06169	LA29479_730	type II secretion system protein E
OG_06170	LA29479_752	hypothetical protein
OG_06171	LA29479_799	hypothetical protein
OG_06172	LA29479_806	hypothetical protein
OG_06173	LA29479_814	hypothetical protein
OG_06174	LA29479_832	hypothetical protein
OG_06175	LA29479_834	hypothetical protein
OG_06176	LA29479_843	hypothetical protein
OG_06177	LA29479_844	hypothetical protein
OG_06178	LA29479_888	hypothetical protein
OG_06179	LA29479_889	hypothetical protein
OG_06180	LA29479_963	hypothetical protein
OG_06181	LA29479_964	putative membrane protein
OG_06182	LA29479_973	putative membrane protein
OG_06183	LA29479_986	hypothetical protein
OG_06184	LA29479_996	hypothetical protein
OG_06185	LA29479_997	hypothetical protein
OG_06186	LA29479_1014	hypothetical protein
OG_06187	LA29479_1042	hypothetical protein
OG_06188	LA29479_1080	hypothetical protein
OG_06189	LA29479_1091	hypothetical protein
OG_06190	LA29479_1106	hypothetical protein
OG_06191	LA29479_1114	hypothetical protein
OG_06192	LA29479_1170	hypothetical protein
OG_06193	LA29479_1172	hypothetical protein
OG_08154	LA76x_729	LA76x_729; hypothetical protein
OG_08155	LA76x_741	LA76x_741; hypothetical protein
OG_08156	LA76x_755	LA76x_755; hypothetical protein
OG_08157	LA76x_759	LA76x_759; hypothetical protein
OG_08158	LA76x_775	LA76x_775; hypothetical protein
OG_08159	LA76x_813	LA76x_813; hypothetical protein
OG_08160	LA76x_910	LA76x_910; hypothetical protein
OG_08161	LA76x_943	LA76x_943; hypothetical protein
OG_08162	LA76x_948	LA76x_948; hypothetical protein
OG_08163	LA76x_956	LA76x_956; hypothetical protein
OG_08164	LA76x_972	LA76x_972; hypothetical protein
OG_08165	LA76x_1015	LA76x_1015; hypothetical protein
OG_08166	LA76x_1033	LA76x_1033; hypothetical protein
OG_08167	LA76x_1034	LA76x_1034; hypothetical protein
OG_08168	LA76x_1075	LA76x_1075; glycosyl transferases group 1 family protein
OG_08169	LA76x_1084	LA76x_1084; hypothetical protein
OG_08170	LA76x_1110	LA76x_1110; hypothetical protein
OG_08171	LA76x_1120	LA76x_1120; hypothetical protein
OG_08172	LA76x_1190	LA76x_1190; glycosyl transferases group 1 family protein
OG_08173	LA76x_1198	LA76x_1198; hypothetical protein
OG_08174	LA76x_1216	LA76x_1216; hypothetical protein
OG_08175	LA76x_1232	LA76x_1232; hypothetical protein
OG_08176	LA76x_1252	LA76x_1252; hypothetical protein
OG_08177	LA76x_1267	LA76x_1267; hypothetical protein
OG_08178	LA76x_1278	LA76x_1278; conserved hypothetical protein
OG_08179	LA76x_1282	LA76x_1282; hypothetical protein
OG_08180	LA76x_1283	LA76x_1283; hypothetical protein
OG_08181	LA76x_1284	LA76x_1284; hypothetical protein
OG_08182	LA76x_1285	LA76x_1285; conserved hypothetical protein
OG_08183	LA76x_1299	LA76x_1299; conserved hypothetical protein
OG_08184	LA76x_1300	LA76x_1300; Reverse
OG_08185	LA76x_1301	LA76x_1301; hypothetical protein
OG_08186	LA76x_1304	LA76x_1304; conserved hypothetical protein
OG_08187	LA76x_1305	LA76x_1305; conserved hypothetical protein
OG_08188	LA76x_1306	LA76x_1306; hypothetical protein
OG_08189	LA76x_1307	LA76x_1307; hypothetical protein
OG_08190	LA76x_1308	LA76x_1308; hypothetical protein
OG_08191	LA76x_1309	LA76x_1309; hypothetical protein
OG_08192	LA76x_1310	LA76x_1310; hypothetical protein
OG_08193	LA76x_1311	LA76x_1311; yqaJ-like viral recombinase domain protein
OG_08194	LA76x_1312	LA76x_1312; conserved hypothetical protein
OG_08195	LA76x_1313	LA76x_1313; ninB family protein
OG_08196	LA76x_1314	LA76x_1314; hypothetical protein
OG_08197	LA76x_1315	LA76x_1315; hypothetical protein
OG_08198	LA76x_1316	LA76x_1316; hypothetical protein
OG_08199	LA76x_1317	LA76x_1317; hypothetical protein
OG_08200	LA76x_1318	LA76x_1318; hypothetical protein
OG_08201	LA76x_1319	LA76x_1319; hypothetical protein
OG_08202	LA76x_1320	LA76x_1320; conserved hypothetical protein
OG_08203	LA76x_1321	LA76x_1321; hypothetical protein
OG_08204	LA76x_1323	LA76x_1323; hypothetical protein
OG_08205	LA76x_1324	LA76x_1324; hypothetical protein
OG_08206	LA76x_1325	LA76x_1325; hypothetical protein
OG_08207	LA76x_1326	LA76x_1326; hypothetical protein
OG_08208	LA76x_1327	LA76x_1327; hypothetical protein
OG_08209	LA76x_1328	LA76x_1328; conserved hypothetical protein
OG_08210	LA76x_1329	dnaB; replicative DNA helicase
OG_08211	LA76x_1330	LA76x_1330; hypothetical protein
OG_08212	LA76x_1331	LA76x_1331; hypothetical protein
OG_08213	LA76x_1332	LA76x_1332; hypothetical protein
OG_08214	LA76x_1333	LA76x_1333; hypothetical protein
OG_08215	LA76x_1334	LA76x_1334; hypothetical protein
OG_08216	LA76x_1335	LA76x_1335; hypothetical protein
OG_08217	LA76x_1336	LA76x_1336; D-alanyl-D-alanine carboxypeptidase family protein
OG_08218	LA76x_1337	LA76x_1337; hypothetical protein
OG_08219	LA76x_1338	LA76x_1338; hypothetical protein
OG_08220	LA76x_1339	LA76x_1339; hypothetical protein
OG_08221	LA76x_1340	LA76x_1340; putative gp12
OG_08222	LA76x_1341	LA76x_1341; hypothetical protein
OG_08223	LA76x_1342	LA76x_1342; conserved hypothetical protein
OG_08224	LA76x_1343	LA76x_1343; putative bacteriophage TerL protein

OG_06194	LA29479_1173	hypothetical protein
OG_06195	LA29479_1194	hypothetical protein
OG_06196	LA29479_1209	hypothetical protein
OG_06197	LA29479_1214	hypothetical protein
OG_06198	LA29479_1228	hypothetical protein
OG_06199	LA29479_1239	hypothetical protein
OG_06200	LA29479_1241	hypothetical protein
OG_06201	LA29479_1242	putative membrane protein
OG_06202	LA29479_1260	hypothetical protein
OG_06203	LA29479_1265	GTPase subunit of restriction endonuclease-like protein
OG_06204	LA29479_1270	hypothetical protein
OG_06205	LA29479_1292	putative membrane protein
OG_06206	LA29479_1307	hypothetical protein
OG_06207	LA29479_1321	hypothetical protein
OG_06208	LA29479_1331	hypothetical protein
OG_06209	LA29479_1356	hypothetical protein
OG_06210	LA29479_1370	hypothetical protein
OG_06211	LA29479_1373	hypothetical protein
OG_06212	LA29479_1391	putative membrane protein
OG_06213	LA29479_1400	hypothetical protein
OG_06214	LA29479_1402	hypothetical protein
OG_06215	LA29479_1403	hypothetical protein
OG_06216	LA29479_1405	primase C terminal 2 family protein
OG_06217	LA29479_1497	hypothetical protein
OG_06218	LA29479_1503	hypothetical protein
OG_06219	LA29479_1506	hypothetical protein
OG_06220	LA29479_1507	hypothetical protein
OG_06221	LA29479_1553	hypothetical protein
OG_06222	LA29479_1559	hypothetical protein
OG_06223	LA29479_1562	hypothetical protein
OG_06224	LA29479_1572	hypothetical protein
OG_06225	LA29479_1585	hypothetical protein
OG_06226	LA29479_1586	hypothetical protein
OG_06227	LA29479_1588	short chain dehydrogenase domain protein
OG_06228	LA29479_1608	hypothetical protein
OG_06229	LA29479_1616	hypothetical protein
OG_06230	LA29479_1617	putative phage protein
OG_06231	LA29479_1624	hypothetical protein
OG_06232	LA29479_1683	hypothetical protein
OG_06233	LA29479_1706	amino acid adenylation domain protein
OG_06234	LA29479_1709	amino acid adenylation domain protein
OG_06235	LA29479_1710	amino acid adenylation domain protein
OG_06236	LA29479_1718	hypothetical protein
OG_06237	LA29479_1717	hypothetical protein
OG_06238	LA29479_1740	hypothetical protein
OG_06239	LA29479_1741	hypothetical protein
OG_06240	LA29479_1744	hypothetical protein
OG_06241	LA29479_1748	hypothetical protein
OG_06242	LA29479_1752	hypothetical protein
OG_06243	LA29479_1765	hypothetical protein
OG_06244	LA29479_1784	hypothetical protein
OG_06245	LA29479_1819	hypothetical protein
OG_06246	LA29479_1826	hypothetical protein
OG_06247	LA29479_1831	hypothetical protein
OG_06248	LA29479_1833	hypothetical protein
OG_06249	LA29479_1840	hypothetical protein
OG_06250	LA29479_1871	hypothetical protein
OG_06251	LA29479_1878	hypothetical protein
OG_06252	LA29479_1896	hypothetical protein
OG_06253	LA29479_1899	hypothetical protein
OG_06254	LA29479_1933	hypothetical protein
OG_06255	LA29479_1948	hypothetical protein
OG_06256	LA29479_1970	hypothetical protein
OG_06257	LA29479_2002	hypothetical protein
OG_06258	LA29479_2011	hypothetical protein
OG_06259	LA29479_2041	hypothetical protein
OG_06260	LA29479_2074	putative membrane protein
OG_06261	LA29479_2077	glycosyl transferases group 1 family protein
OG_06262	LA29479_2118	hypothetical protein
OG_06263	LA29479_2137	hypothetical protein
OG_06264	LA29479_2166	hypothetical protein

OG_08225	LA76x_1344	LA76x_1344; conserved hypothetical protein
OG_08226	LA76x_1345	LA76x_1345; hypothetical protein
OG_08227	LA76x_1346	LA76x_1346; P22 coat - gene 5 family protein
OG_08228	LA76x_1347	LA76x_1347; hypothetical protein
OG_08229	LA76x_1348	LA76x_1348; P22 tail accessory factor family protein
OG_08230	LA76x_1349	LA76x_1349; phage stabilisation family protein
OG_08231	LA76x_1350	LA76x_1350; hypothetical protein
OG_08232	LA76x_1351	LA76x_1351; acetyltransferase family protein
OG_08233	LA76x_1352	LA76x_1352; conserved hypothetical protein
OG_08234	LA76x_1353	LA76x_1353; peptidase M15 family protein
OG_08235	LA76x_1354	LA76x_1354; hypothetical protein
OG_08236	LA76x_1355	LA76x_1355; hypothetical protein
OG_08237	LA76x_1356	LA76x_1356; hypothetical protein
OG_08238	LA76x_1357	LA76x_1357; hypothetical protein
OG_08239	LA76x_1358	LA76x_1358; conserved hypothetical protein
OG_08240	LA76x_1359	LA76x_1359; hypothetical protein
OG_08241	LA76x_1377	LA76x_1377; hypothetical protein
OG_08242	LA76x_1386	LA76x_1386; hypothetical protein
OG_08243	LA76x_1395	LA76x_1395; RHS repeat-associated core domain protein
OG_08244	LA76x_1398	LA76x_1398; hypothetical protein
OG_08245	LA76x_1415	LA76x_1415; hypothetical protein
OG_08246	LA76x_1419	LA76x_1419; hypothetical protein
OG_08247	LA76x_1422	LA76x_1422; hypothetical protein
OG_08248	LA76x_1455	LA76x_1455; hypothetical protein
OG_08249	LA76x_1458	LA76x_1458; hypothetical protein
OG_08250	LA76x_1508	LA76x_1508; hypothetical protein
OG_08251	LA76x_1537	LA76x_1537; hypothetical protein
OG_08252	LA76x_1547	LA76x_1547; hypothetical protein
OG_08253	LA76x_1580	LA76x_1580; hypothetical protein
OG_08254	LA76x_1594	LA76x_1594; hypothetical protein
OG_08255	LA76x_1602	LA76x_1602; hypothetical protein
OG_08256	LA76x_1625	LA76x_1625; hypothetical protein
OG_08257	LA76x_1659	LA76x_1659; helix-turn-helix family protein
OG_08258	LA76x_1708	LA76x_1708; hypothetical protein
OG_08259	LA76x_1716	LA76x_1716; hypothetical protein
OG_08260	LA76x_1732	LA76x_1732; hypothetical protein
OG_08261	LA76x_1754	LA76x_1754; periplasmic binding protein-like domain protein
OG_08262	LA76x_1775	LA76x_1775; hypothetical protein
OG_08263	LA76x_1839	LA76x_1839; hypothetical protein
OG_08264	LA76x_1858	LA76x_1858; hypothetical protein
OG_08265	LA76x_1869	LA76x_1869; hypothetical protein
OG_08266	LA76x_1883	LA76x_1883; hypothetical protein
OG_08267	LA76x_1891	LA76x_1891; hypothetical protein
OG_08268	LA76x_1913	LA76x_1913; hypothetical protein
OG_08269	LA76x_1947	LA76x_1947; hypothetical protein
OG_08270	LA76x_1988	LA76x_1988; hypothetical protein
OG_08271	LA76x_2006	LA76x_2006; hypothetical protein
OG_08272	LA76x_2018	LA76x_2018; hypothetical protein
OG_08273	LA76x_2039	LA76x_2039; hypothetical protein
OG_08274	LA76x_2043	LA76x_2043; hypothetical protein
OG_08275	LA76x_2051	LA76x_2051; hypothetical protein
OG_08276	LA76x_2054	LA76x_2054; hypothetical protein
OG_08277	LA76x_2099	LA76x_2099; AIPR family protein
OG_08278	LA76x_2120	LA76x_2120; amino acid adenylation domain protein
OG_08279	LA76x_2122	dltA; D-alanine--poly(phosphoribitol) ligase, subunit 1
OG_08280	LA76x_2123	dltA; D-alanine--poly(phosphoribitol) ligase, subunit 1
OG_08281	LA76x_2139	LA76x_2139; hypothetical protein
OG_08282	LA76x_2151	LA76x_2151; hypothetical protein
OG_08283	LA76x_2216	LA76x_2216; hypothetical protein
OG_08284	LA76x_2217	LA76x_2217; hypothetical protein
OG_08285	LA76x_2219	LA76x_2219; hypothetical protein
OG_08286	LA76x_2241	LA76x_2241; hypothetical protein
OG_08287	LA76x_2264	LA76x_2264; hypothetical protein
OG_08288	LA76x_2268	LA76x_2268; ftsK/SpoIIIE family protein
OG_08289	LA76x_2269	LA76x_2269; conserved hypothethical domain protein
OG_08290	LA76x_2270	LA76x_2270; hypothetical protein
OG_08291	LA76x_2273	LA76x_2273; hypothetical protein
OG_08292	LA76x_2280	LA76x_2280; hypothetical protein
OG_08293	LA76x_2300	LA76x_2300; hypothetical protein
OG_08294	LA76x_2316	LA76x_2316; hypothetical protein
OG_08295	LA76x_2350	LA76x_2350; hypothetical protein

OG_06265	LA29479_2167	hypothetical protein
OG_06266	LA29479_2188	hypothetical protein
OG_06267	LA29479_2193	hypothetical protein
OG_06268	LA29479_2204	hypothetical protein
OG_06269	LA29479_2256	putative lipoprotein
OG_06270	LA29479_2263	hypothetical protein
OG_06271	LA29479_2278	hypothetical protein
OG_06272	LA29479_2289	hypothetical protein
OG_06273	LA29479_2296	hypothetical protein
OG_06274	LA29479_2301	hypothetical protein
OG_06275	LA29479_2338	hypothetical protein
OG_06276	LA29479_2394	hypothetical protein
OG_06277	LA29479_2415	hypothetical protein
OG_06278	LA29479_2417	hypothetical protein
OG_06279	LA29479_2433	hypothetical protein
OG_06280	LA29479_2440	hypothetical protein
OG_06281	LA29479_2445	hypothetical protein
OG_06282	LA29479_2450	hypothetical protein
OG_06283	LA29479_2466	hypothetical protein
OG_06284	LA29479_2481	hypothetical protein
OG_06285	LA29479_2530	putative uncharacterized protein
OG_06286	LA29479_2544	hypothetical protein
OG_06287	LA29479_2547	hypothetical protein
OG_06288	LA29479_2550	hypothetical protein
OG_06289	LA29479_2551	putative uncharacterized domain protein
OG_06290	LA29479_2552	hypothetical protein
OG_06291	LA29479_2569	hypothetical protein
OG_06292	LA29479_2592	hypothetical protein
OG_06293	LA29479_2593	hypothetical protein
OG_06294	LA29479_2595	ompA family protein
OG_06295	LA29479_2597	hypothetical protein
OG_06296	LA29479_2600	hypothetical protein
OG_06297	LA29479_2615	hypothetical protein
OG_06298	LA29479_2620	hypothetical protein
OG_06299	LA29479_2642	hypothetical protein
OG_06300	LA29479_2647	hypothetical protein
OG_06301	LA29479_2657	hypothetical protein
OG_06302	LA29479_2666	hypothetical protein
OG_06303	LA29479_2680	NAD dependent epimerase/dehydratase family protein
OG_06304	LA29479_2704	hypothetical protein
OG_06305	LA29479_2715	hypothetical protein
OG_06306	LA29479_2725	hypothetical protein
OG_06307	LA29479_2787	hypothetical protein
OG_06308	LA29479_2808	hypothetical protein
OG_06309	LA29479_2835	putative uncharacterized protein
OG_06310	LA29479_2853	hypothetical protein
OG_06311	LA29479_2854	hypothetical protein
OG_06312	LA29479_2880	hypothetical protein
OG_06313	LA29479_2896	hypothetical protein
OG_06314	LA29479_2898	hypothetical protein
OG_06315	LA29479_2906	hypothetical protein
OG_06316	LA29479_2918	ricin-type beta-trefoil lectin domain protein
OG_06317	LA29479_2921	hypothetical protein
OG_06318	LA29479_2922	tonB dependent receptor family protein
OG_06319	LA29479_2923	bacterial regulatory s, gntR family protein
OG_06320	LA29479_2928	hypothetical protein
OG_06321	LA29479_2950	hypothetical protein
OG_06322	LA29479_2956	amino acid adenylation domain protein
OG_06323	LA29479_2960	hypothetical protein
OG_06324	LA29479_2990	hypothetical protein
OG_06325	LA29479_3013	hypothetical protein
OG_06326	LA29479_3018	hypothetical protein
OG_06327	LA29479_3021	hypothetical protein
OG_06328	LA29479_3025	hypothetical protein
OG_06329	LA29479_3026	hemolysin-type calcium-binding repeat family protein
OG_06330	LA29479_3055	nrdJb domain protein
OG_06331	LA29479_3061	hypothetical protein
OG_06332	LA29479_3076	hypothetical protein
OG_06333	LA29479_3086	hypothetical protein
OG_06334	LA29479_3135	hypothetical protein
OG_06335	LA29479_3143	hypothetical protein

OG_08296	LA76x_2365	LA76x_2365; hypothetical protein
OG_08297	LA76x_2431	LA76x_2431; hypothetical protein
OG_08298	LA76x_2437	LA76x_2437; hypothetical protein
OG_08299	LA76x_2456	LA76x_2456; hypothetical protein
OG_08300	LA76x_2460	LA76x_2460; hypothetical protein
OG_08301	LA76x_2463	LA76x_2463; hypothetical protein
OG_08302	LA76x_2464	LA76x_2464; hypothetical protein
OG_08303	LA76x_2474	LA76x_2474; hypothetical protein
OG_08304	LA76x_2499	LA76x_2499; hypothetical protein
OG_08305	LA76x_2563	LA76x_2563; hypothetical protein
OG_08306	LA76x_2572	LA76x_2572; RHS repeat-associated core domain protein
OG_08307	LA76x_2573	LA76x_2573; conserved hypothetical protein
OG_08308	LA76x_2574	LA76x_2574; hypothetical protein
OG_08309	LA76x_2600	LA76x_2600; hypothetical protein
OG_08310	LA76x_2601	LA76x_2601; hypothetical protein
OG_08311	LA76x_2628	LA76x_2628; hypothetical protein
OG_08312	LA76x_2670	LA76x_2670; hypothetical protein
OG_08313	LA76x_2688	LA76x_2688; hypothetical protein
OG_08314	LA76x_2693	LA76x_2693; hypothetical protein
OG_08315	LA76x_2727	LA76x_2727; GAD-like domain protein
OG_08316	LA76x_2763	LA76x_2763; hypothetical protein
OG_08317	LA76x_2790	LA76x_2790; hypothetical protein
OG_08318	LA76x_2838	LA76x_2838; hypothetical protein
OG_08319	LA76x_2843	LA76x_2843; conserved hypothetical protein
OG_08320	LA76x_2844	LA76x_2844; hypothetical protein
OG_08321	LA76x_2878	LA76x_2878; hypothetical protein
OG_08322	LA76x_2881	LA76x_2881; hypothetical protein
OG_08323	LA76x_2889	LA76x_2889; L,D-transpeptidase catalytic domain protein
OG_08324	LA76x_2892	LA76x_2892; acetyltransferase family protein
OG_08325	LA76x_2909	LA76x_2909; hypothetical protein
OG_08326	LA76x_2935	LA76x_2935; hypothetical protein
OG_08327	LA76x_2956	LA76x_2956; hypothetical protein
OG_08328	LA76x_2972	LA76x_2972; hypothetical protein
OG_08329	LA76x_3006	LA76x_3006; hypothetical protein
OG_08330	LA76x_3040	LA76x_3040; hypothetical protein
OG_08331	LA76x_3060	LA76x_3060; hypothetical protein
OG_08332	LA76x_3064	LA76x_3064; hypothetical protein
OG_08333	LA76x_3078	LA76x_3078; hypothetical protein
OG_08334	LA76x_3087	LA76x_3087; hypothetical protein
OG_08335	LA76x_3136	LA76x_3136; hypothetical protein
OG_08336	LA76x_3165	LA76x_3165; putative nucleoside-diphosphate-sugar epimerase
OG_08337	LA76x_3166	LA76x_3166; divergent AAA domain protein
OG_08338	LA76x_3167	LA76x_3167; zeta toxin family protein
OG_08339	LA76x_3172	LA76x_3172; hypothetical protein
OG_08340	LA76x_3182	LA76x_3182; hypothetical protein
OG_08341	LA76x_3188	LA76x_3188; hypothetical protein
OG_08342	LA76x_3199	LA76x_3199; hypothetical protein
OG_08343	LA76x_3231	LA76x_3231; hypothetical protein
OG_08344	LA76x_3242	LA76x_3242; hypothetical protein
OG_08345	LA76x_3246	LA76x_3246; hypothetical protein
OG_08346	LA76x_3248	LA76x_3248; hypothetical protein
OG_08347	LA76x_3253	LA76x_3253; restriction endonuclease family protein
OG_08348	LA76x_3254	LA76x_3254; conserved hypothetical protein
OG_08349	LA76x_3258	LA76x_3258; hypothetical protein
OG_08350	LA76x_3263	LA76x_3263; amino acid adenylation domain protein
OG_08351	LA76x_3324	LA76x_3324; autotransporter-associated beta strand repeat family pr
OG_08352	LA76x_3345	LA76x_3345; hypothetical protein
OG_08353	LA76x_3355	LA76x_3355; hypothetical protein
OG_08354	LA76x_3385	LA76x_3385; hypothetical protein
OG_08355	LA76x_3418	LA76x_3418; hypothetical protein
OG_08356	LA76x_3442	LA76x_3442; hypothetical protein
OG_08357	LA76x_3452	LA76x_3452; hypothetical protein
OG_08358	LA76x_3502	LA76x_3502; hypothetical protein
OG_08359	LA76x_3552	LA76x_3552; hypothetical protein
OG_08360	LA76x_3574	LA76x_3574; hypothetical protein
OG_08361	LA76x_3586	LA76x_3586; hypothetical protein
OG_08362	LA76x_3620	LA76x_3620; hypothetical protein
OG_08363	LA76x_3650	LA76x_3650; hypothetical protein
OG_08364	LA76x_3661	LA76x_3661; hypothetical protein
OG_08365	LA76x_3673	LA76x_3673; hypothetical protein
OG_08366	LA76x_3683	LA76x_3683; hypothetical protein

OG_06336	LA29479_3146	hypothetical protein
OG_06337	LA29479_3151	hypothetical protein
OG_06338	LA29479_3161	hypothetical protein
OG_06339	LA29479_3164	hypothetical protein
OG_06340	LA29479_3182	hypothetical protein
OG_06341	LA29479_3189	hypothetical protein
OG_06342	LA29479_3209	hypothetical protein
OG_06343	LA29479_3218	hypothetical protein
OG_06344	LA29479_3239	amino acid adenylation domain protein
OG_06345	LA29479_3244	hypothetical protein
OG_06346	LA29479_3269	hypothetical protein
OG_06347	LA29479_3284	glyoxalase/Bleomycin resistance /Dioxygenase superfamily protein
OG_06348	LA29479_3285	sigma-70, region 4 family protein
OG_06349	LA29479_3286	putative membrane protein
OG_06350	LA29479_3297	hypothetical protein
OG_06351	LA29479_3311	hypothetical protein
OG_06352	LA29479_3314	hypothetical protein
OG_06353	LA29479_3316	hypothetical protein
OG_06354	LA29479_3322	hypothetical protein
OG_06355	LA29479_3329	hypothetical protein
OG_06356	LA29479_3332	outer membrane domain protein
OG_06357	LA29479_3375	hypothetical protein
OG_06358	LA29479_3427	hypothetical protein
OG_06359	LA29479_3450	hypothetical protein
OG_06360	LA29479_3453	hypothetical protein
OG_06361	LA29479_3462	hypothetical protein
OG_06362	LA29479_3479	hypothetical protein
OG_06363	LA29479_3493	putative uncharacterized domain protein
OG_06364	LA29479_3494	DNA polymerase LigD, polymerase domain
OG_06365	LA29479_3505	hypothetical protein
OG_06366	LA29479_3540	hypothetical protein
OG_06367	LA29479_3542	hypothetical protein
OG_06368	LA29479_3569	hypothetical protein
OG_06369	LA29479_3580	hypothetical protein
OG_06370	LA29479_3598	putative uncharacterized protein
OG_06371	LA29479_3599	hypothetical protein
OG_06372	LA29479_3624	hypothetical protein
OG_06373	LA29479_3634	hypothetical protein
OG_06374	LA29479_3636	putative membrane protein
OG_06375	LA29479_3637	napD family protein
OG_06376	LA29479_3638	periplasmic nitrate reductase, large subunit
OG_06377	LA29479_3639	nitrate reductase cytochrome c-type subunit family protein
OG_06378	LA29479_3640	periplasmic nitrate (or nitrite) reductase c-type cytochrome, NapC/Nir
OG_06379	LA29479_3666	hypothetical protein
OG_06380	LA29479_3685	hypothetical protein
OG_06381	LA29479_3686	hypothetical protein
OG_06382	LA29479_3694	hypothetical protein
OG_06383	LA29479_3697	trbL/VirB6 plasmid conjugal transfer family protein
OG_06384	LA29479_3699	putative membrane fusion transmembrane protein
OG_06385	LA29479_3700	hypothetical protein
OG_06386	LA29479_3701	hypothetical protein
OG_06387	LA29479_3702	hypothetical protein
OG_06388	LA29479_3714	hypothetical protein
OG_06389	LA29479_3716	putative membrane protein
OG_06390	LA29479_3726	hypothetical protein
OG_06391	LA29479_3731	hypothetical protein
OG_06392	LA29479_3737	hypothetical protein
OG_06393	LA29479_3738	hypothetical protein
OG_06394	LA29479_3740	hypothetical protein
OG_06395	LA29479_3741	hypothetical protein
OG_06396	LA29479_3745	hypothetical protein
OG_06397	LA29479_3747	hypothetical protein
OG_06398	LA29479_3760	hypothetical protein
OG_06399	LA29479_3780	hypothetical protein
OG_06400	LA29479_3785	hypothetical protein
OG_06401	LA29479_3789	hypothetical protein
OG_06402	LA29479_3791	hypothetical protein
OG_06403	LA29479_3797	hypothetical protein
OG_06404	LA29479_3801	hypothetical protein
OG_06405	LA29479_3804	hypothetical protein
OG_06406	LA29479_3806	hypothetical protein

OG_08367	LA76x_3721	LA76x_3721; hypothetical protein
OG_08368	LA76x_3722	LA76x_3722; thermolysin metallopeptidase, catalytic domain protein
OG_08369	LA76x_3735	LA76x_3735; hypothetical protein
OG_08370	LA76x_3778	LA76x_3778; hypothetical protein
OG_08371	LA76x_3784	LA76x_3784; hypothetical protein
OG_08372	LA76x_3806	LA76x_3806; hypothetical protein
OG_08373	LA76x_3816	LA76x_3816; hypothetical protein
OG_08374	LA76x_3817	LA76x_3817; hypothetical protein
OG_08375	LA76x_3839	LA76x_3839; hypothetical protein
OG_08376	LA76x_3845	LA76x_3845; hypothetical protein
OG_08377	LA76x_3850	LA76x_3850; hypothetical protein
OG_08378	LA76x_3931	LA76x_3931; hypothetical protein
OG_08379	LA76x_3988	LA76x_3988; hypothetical protein
OG_08380	LA76x_3993	LA76x_3993; helix-turn-helix domain protein
OG_08381	LA76x_4007	LA76x_4007; hypothetical protein
OG_08382	LA76x_4026	LA76x_4026; hypothetical protein
OG_08383	LA76x_4032	LA76x_4032; hypothetical protein
OG_08384	LA76x_4036	LA76x_4036; hypothetical protein
OG_08385	LA76x_4103	LA76x_4103; hypothetical protein
OG_08386	LA76x_4107	LA76x_4107; hypothetical protein
OG_08387	LA76x_4112	LA76x_4112; hypothetical protein
OG_08388	LA76x_4129	LA76x_4129; hypothetical protein
OG_08389	LA76x_4141	LA76x_4141; hypothetical protein
OG_08390	LA76x_4151	LA76x_4151; hypothetical protein
OG_08391	LA76x_4187	LA76x_4187; hypothetical protein
OG_08392	LA76x_4234	LA76x_4234; hypothetical protein
OG_08393	LA76x_4238	LA76x_4238; hypothetical protein
OG_08394	LA76x_4243	LA76x_4243; hypothetical protein
OG_08395	LA76x_4284	LA76x_4284; hypothetical protein
OG_08396	LA76x_4360	LA76x_4360; hypothetical protein
OG_08397	LA76x_4364	LA76x_4364; hypothetical protein
OG_08398	LA76x_4391	LA76x_4391; hypothetical protein
OG_08399	LA76x_4398	LA76x_4398; hypothetical protein
OG_08400	LA76x_4449	LA76x_4449; hypothetical protein
OG_08401	LA76x_4455	LA76x_4455; hypothetical protein
OG_08402	LA76x_4502	LA76x_4502; conserved hypothetical protein
OG_08403	LA76x_4542	LA76x_4542; hypothetical protein
OG_08404	LA76x_4547	LA76x_4547; hypothetical protein
OG_08405	LA76x_4548	LA76x_4548; hypothetical protein
OG_08406	LA76x_4650	LA76x_4650; hypothetical protein
OG_08407	LA76x_4651	LA76x_4651; hypothetical protein
OG_08408	LA76x_4660	LA76x_4660; hypothetical protein
OG_08409	LA76x_4663	LA76x_4663; hypothetical protein
OG_08410	LA76x_4684	LA76x_4684; hypothetical protein
OG_08411	LA76x_4718	LA76x_4718; hypothetical protein
OG_08412	LA76x_4768	LA76x_4768; ankyrin repeat family protein
OG_08413	LA76x_4774	LA76x_4774; hypothetical protein
OG_08414	LA76x_4849	LA76x_4849; hypothetical protein
OG_08415	LA76x_4876	LA76x_4876; hypothetical protein
OG_08416	LA76x_4920	LA76x_4920; hypothetical protein
OG_08417	LA76x_4921	LA76x_4921; histidine kinase-, DNA gyrase B-, and HSP90-like ATPa
OG_08418	LA76x_4924	LA76x_4924; hypothetical protein
OG_08419	LA76x_4927	LA76x_4927; resolvase, N terminal domain protein
OG_08420	LA76x_4930	LA76x_4930; hypothetical protein
OG_08421	LA76x_4934	LA76x_4934; hypothetical protein
OG_08422	LA76x_4935	LA76x_4935; PD-(D/E)XK nuclease superfamily protein
OG_08423	LA76x_4937	LA76x_4937; conserved hypothetical protein
OG_08424	LA76x_4938	LA76x_4938; conserved hypothetical protein
OG_08425	LA76x_4940	LA76x_4940; hypothetical protein
OG_08426	LA76x_4941	LA76x_4941; hypothetical protein
OG_08427	LA76x_4943	LA76x_4943; DNA methylase family protein
OG_08428	LA76x_4950	LA76x_4950; hypothetical protein
OG_08429	LA76x_4952	LA76x_4952; phage terminase large subunit family protein
OG_08430	LA76x_4953	LA76x_4953; hypothetical protein
OG_08431	LA76x_4958	LA76x_4958; hypothetical protein
OG_08432	LA76x_4961	LA76x_4961; conserved hypothetical protein
OG_08433	LA76x_4962	LA76x_4962; conserved hypothetical protein
OG_08434	LA76x_4963	LA76x_4963; hypothetical protein
OG_08435	LA76x_4964	LA76x_4964; conserved hypothetical protein
OG_08436	LA76x_4965	LA76x_4965; phage lysozyme family protein
OG_08437	LA76x_4966	LA76x_4966; hypothetical protein

OG_06407	LA29479_3816	hypothetical protein
OG_06408	LA29479_3840	hypothetical protein
OG_06409	LA29479_3844	hypothetical protein
OG_06410	LA29479_3857	hypothetical protein
OG_06411	LA29479_3885	hypothetical protein
OG_06412	LA29479_3911	hypothetical protein
OG_06413	LA29479_3928	hypothetical protein
OG_06414	LA29479_3944	hypothetical protein
OG_06415	LA29479_3987	hypothetical protein
OG_06416	LA29479_3990	hypothetical protein
OG_06417	LA29479_4037	hypothetical protein
OG_06418	LA29479_4044	putative membrane protein
OG_06419	LA29479_4055	hypothetical protein
OG_06420	LA29479_4061	hypothetical protein
OG_06421	LA29479_4064	hypothetical protein
OG_06422	LA29479_4091	condensation domain protein
OG_06423	LA29479_4114	hypothetical protein
OG_06424	LA29479_4134	hypothetical protein
OG_06425	LA29479_4135	dimodular nonribosomal peptide synthase
OG_06426	LA29479_4155	hypothetical protein
OG_06427	LA29479_4156	conserved hypothetical protein
OG_06428	LA29479_4187	hypothetical protein
OG_06429	LA29479_4193	hypothetical protein
OG_06430	LA29479_4199	hypothetical protein
OG_06431	LA29479_4209	hypothetical protein
OG_06432	LA29479_4215	hypothetical protein
OG_06433	LA29479_4248	transcriptional regulator, LacI family
OG_06434	LA29479_4267	hypothetical protein
OG_06435	LA29479_4273	lppC family lipoprotein
OG_06436	LA29479_4315	hypothetical protein
OG_06437	LA29479_4328	hypothetical protein
OG_06438	LA29479_4353	hypothetical protein
OG_06439	LA29479_4363	hypothetical protein
OG_06440	LA29479_4377	hypothetical protein
OG_06441	LA29479_4381	hypothetical protein
OG_06442	LA29479_4394	hypothetical protein
OG_06443	LA29479_4398	hypothetical protein
OG_06444	LA29479_4428	mg chelatase-like domain protein
OG_06445	LA29479_4433	hypothetical protein
OG_06446	LA29479_4434	hypothetical protein
OG_06447	LA29479_4437	peptidase C39 family protein
OG_06448	LA29479_4438	hypothetical protein
OG_06449	LA29479_4475	hypothetical protein
OG_06450	LA29479_4496	hypothetical protein
OG_06451	LA29479_4506	hypothetical protein
OG_06452	LA29479_4508	hypothetical protein
OG_06453	LA29479_4527	hypothetical protein
OG_06454	LA29479_4548	hypothetical protein
OG_06455	LA29479_4560	hypothetical protein
OG_06456	LA29479_4586	hypothetical protein
OG_06457	LA29479_4596	hypothetical protein
OG_06458	LA29479_4603	hypothetical protein
OG_06459	LA29479_4634	hypothetical protein
OG_06460	LA29479_4650	putative uncharacterized domain protein
OG_06461	LA29479_4652	hypothetical protein
OG_06462	LA29479_4653	transposase B
OG_06463	LA29479_4654	glycosyl hydrolase 85 family protein
OG_06464	LA29479_4655	astacin family protein
OG_06465	LA29479_4656	hypothetical protein
OG_06466	LA29479_4658	hypothetical protein
OG_06467	LA29479_4659	lysR substrate binding domain protein
OG_06468	LA29479_4660	hypothetical protein
OG_06469	LA29479_4663	hypothetical protein
OG_06470	LA29479_4665	subtilase family protein
OG_06471	LA29479_4666	caspase domain protein
OG_06472	LA29479_4667	HEAT repeat family protein
OG_06473	LA29479_4668	phage lysozyme family protein
OG_06474	LA29479_4700	hypothetical protein
OG_06475	LA29479_4702	hypothetical protein
OG_06476	LA29479_4724	hypothetical protein
OG_06477	LA29479_4733	hypothetical protein

OG_08438	LA76x_4967	LA76x_4967; putative integron gene cassette protein
OG_08439	LA76x_4968	LA76x_4968; hypothetical protein
OG_08440	LA76x_4970	LA76x_4970; hypothetical protein
OG_08441	LA76x_4984	LA76x_4984; hypothetical protein
OG_08442	LA76x_4985	LA76x_4985; trbL/VirB6 plasmid conjugal transfer family protein
OG_08443	LA76x_4987	LA76x_4987; hypothetical protein
OG_08444	LA76x_4988	LA76x_4988; bacterial regulatory, luxR family protein
OG_08445	LA76x_4990	LA76x_4990; hypothetical protein
OG_08446	LA76x_4993	LA76x_4993; putative transmembrane protein
OG_08447	LA76x_4994	LA76x_4994; hypothetical protein
OG_08448	LA76x_4995	LA76x_4995; conserved hypothetical protein
OG_08449	LA76x_4996	LA76x_4996; conserved hypothetical protein
OG_08450	LA76x_4997	LA76x_4997; hypothetical protein
OG_08451	LA76x_4998	LA76x_4998; transposase family protein
OG_08452	LA76x_4999	LA76x_4999; conserved hypothetical protein
OG_08453	LA76x_5000	LA76x_5000; HTH-like domain protein
OG_08454	LA76x_5003	LA76x_5003; hypothetical protein
OG_08455	LA76x_5004	LA76x_5004; autotransporter-associated beta strand repeat family pr
OG_08456	LA76x_5005	LA76x_5005; sulfotransferase domain protein
OG_08457	LA76x_5006	LA76x_5006; bacterial regulatory, luxR family protein
OG_08458	LA76x_5007	LA76x_5007; hypothetical protein
OG_08459	LA76x_5008	LA76x_5008; sugar (and other) transporter family protein
OG_08460	LA76x_5011	LA76x_5011; hypothetical protein
OG_08461	LA76x_5037	LA76x_5037; hypothetical protein
OG_08462	LA76x_5038	LA76x_5038; conserved hypothetical protein
OG_08463	LA76x_5056	LA76x_5056; hypothetical protein
OG_08464	LA76x_5149	LA76x_5149; hypothetical protein
OG_08465	LA76x_5167	LA76x_5167; hypothetical protein
OG_08466	LA76x_5178	LA76x_5178; hypothetical protein
OG_08467	LA76x_5187	LA76x_5187; hypothetical protein
OG_04629	LA76x_423	LA76x_423; transposase IS116/IS110/IS902 family protein
OG_06106	LA76x_3219	LA76x_3219; hypothetical protein
OG_06107	LA76x_4616	LA76x_4616; hypothetical protein
OG_06108	LA76x_2424	LA76x_2424; hypothetical protein
OG_04629	LA76x_414	LA76x_414; transposase IS116/IS110/IS902 family protein

OG_06478	LA29479_4744	hypothetical protein
OG_06479	LA29479_4747	hypothetical protein
OG_06480	LA29479_4755	putative uncharacterized protein
OG_06481	LA29479_4760	hypothetical protein
OG_06482	LA29479_4762	hypothetical protein
OG_06483	LA29479_4769	hypothetical protein
OG_06484	LA29479_4768	hypothetical protein
OG_06485	LA29479_4799	hypothetical protein
OG_06486	LA29479_4801	hypothetical protein
OG_06487	LA29479_4802	hypothetical protein
OG_06488	LA29479_4856	outer membrane autotransporter barrel domain protein
OG_06489	LA29479_4862	hypothetical protein
OG_06490	LA29479_4889	hypothetical protein
OG_06491	LA29479_4891	hypothetical protein
OG_06492	LA29479_4902	putative membrane protein
OG_06493	LA29479_4904	hypothetical protein
OG_06494	LA29479_4918	DNA polymerase III tau and gamma subunits domain protein
OG_06495	LA29479_4919	hypothetical protein
OG_06496	LA29479_4943	putative 2-octaprenyl-6-methoxyphenol hydroxylase domain protein
OG_06497	LA29479_4948	hypothetical protein
OG_06498	LA29479_4969	efflux transporter, RND family, MFP subunit domain protein
OG_06499	LA29479_4976	hypothetical protein
OG_06500	LA29479_4992	DNA processing chain A
OG_06501	LA29479_5000	hypothetical protein
OG_06502	LA29479_5015	putative uncharacterized orf5 domain protein
OG_06503	LA29479_5019	hypothetical protein
OG_06504	LA29479_5025	hypothetical protein
OG_06505	LA29479_5033	peptidase M16 inactive domain protein
OG_06506	LA29479_5036	hypothetical protein
OG_06507	LA29479_5053	hypothetical protein
OG_06508	LA29479_5055	hypothetical protein
OG_06509	LA29479_5059	putative TonB dependent receptor domain protein
OG_06510	LA29479_5065	hypothetical protein
OG_06511	LA29479_5093	putative uncharacterized protein
OG_06512	LA29479_5097	hypothetical protein
OG_06513	LA29479_5109	transposase domain protein
OG_06514	LA29479_5111	hypothetical protein
OG_06515	LA29479_5122	hypothetical protein
OG_06516	LA29479_5123	hypothetical protein
OG_06517	LA29479_5124	hypothetical protein
OG_06518	LA29479_5126	hypothetical protein
OG_06519	LA29479_5145	hypothetical protein
OG_06520	LA29479_5155	hypothetical protein
OG_06521	LA29479_5157	hypothetical protein
OG_06522	LA29479_5158	outer membrane autotransporter barrel domain protein
OG_06523	LA29479_5159	conserved repeat domain protein
OG_06524	LA29479_5167	hypothetical protein
OG_06525	LA29479_5179	hypothetical protein
OG_06526	LA29479_5180	hypothetical protein
OG_06527	LA29479_5183	yop virulence translocation R domain protein
OG_06528	LA29479_5191	outer membrane autotransporter barrel domain
OG_06529	LA29479_5197	glutamyl-Q tRNA(Asp) synthetase domain protein
OG_06530	LA29479_5198	elongation factor Tu GTP binding domain protein
OG_06531	LA29479_5208	hypothetical protein
OG_06532	LA29479_5213	outer membrane autotransporter barrel domain
OG_06533	LA29479_5219	hypothetical protein
OG_06534	LA29479_5221	ribosomal S6 modification domain protein
OG_06535	LA29479_5224	hypothetical protein
OG_06536	LA29479_5229	hemY N-terminus family protein
OG_06537	LA29479_5231	putative lipoprotein
OG_06538	LA29479_5233	hypothetical protein
OG_06539	LA29479_5236	isochorismatase hydrolase domain protein
OG_06540	LA29479_5235	hypothetical protein
OG_06541	LA29479_5237	hypothetical protein
OG_06542	LA29479_5238	tonB-dependent Receptor Plug domain protein
OG_06543	LA29479_5239	hypothetical protein
OG_06544	LA29479_5240	putative uncharacterized domain protein
OG_06545	LA29479_5241	pseudouridine synthase domain protein
OG_06546	LA29479_5242	hypothetical protein

Orthologous group	*L. cap* 55<br>GeneID (#710)<br>12.5% of genome	Gene description
OG_00025	LC55x_4111	LC55x_4111; hypothetical protein
OG_00025	LC55x_3872	LC55x_3872; hypothetical protein
OG_00025	LC55x_2029	LC55x_2029; hypothetical protein
OG_00025	LC55x_5043	LC55x_5043; hypothetical protein
OG_00025	LC55x_4163	LC55x_4163; hypothetical protein
OG_00025	LC55x_2057	LC55x_2057; hypothetical protein
OG_00025	LC55x_5734	LC55x_5734; hypothetical protein
OG_00025	LC55x_1176	LC55x_1176; hypothetical protein
OG_00025	LC55x_160	LC55x_160; hypothetical protein
OG_00025	LC55x_4775	LC55x_4775; hypothetical protein
OG_00025	LC55x_176	LC55x_176; hypothetical protein
OG_00025	LC55x_1766	LC55x_1766; hypothetical protein
OG_00025	LC55x_1236	LC55x_1236; hypothetical protein
OG_00025	LC55x_3896	LC55x_3896; hypothetical protein
OG_05559	LC55x_5257	LC55x_5257; hypothetical protein
OG_05560	LC55x_3783	LC55x_3783; hypothetical protein
OG_05561	LC55x_5679	LC55x_5679; hypothetical protein
OG_05562	LC55x_3107	LC55x_3107; hypothetical protein
OG_05563	LC55x_5314	LC55x_5314; hypothetical protein
OG_08468	LC55x_3	recF; DNA replication and repair RecF family protein
OG_08469	LC55x_4	gyrB; DNA gyrase, B subunit
OG_08470	LC55x_11	LC55x_11; biopolymer transport ExbD/TolR family protein
OG_08471	LC55x_18	LC55x_18; hypothetical protein
OG_08472	LC55x_21	LC55x_21; hypothetical protein
OG_08473	LC55x_27	LC55x_27; hypothetical protein
OG_08474	LC55x_31	LC55x_31; hypothetical protein
OG_08475	LC55x_33	LC55x_33; hypothetical protein
OG_08476	LC55x_41	LC55x_41; hypothetical protein
OG_08477	LC55x_45	LC55x_45; hypothetical protein
OG_08478	LC55x_49	LC55x_49; hypothetical protein
OG_08479	LC55x_61	LC55x_61; hypothetical protein
OG_08480	LC55x_66	LC55x_66; hypothetical protein
OG_08481	LC55x_77	LC55x_77; hypothetical protein
OG_08482	LC55x_99	LC55x_99; hypothetical protein
OG_08483	LC55x_103	LC55x_103; type I restriction enzyme R N terminus family protein
OG_08484	LC55x_121	LC55x_121; hypothetical protein
OG_08485	LC55x_123	LC55x_123; hypothetical protein
OG_08486	LC55x_125	LC55x_125; hypothetical protein
OG_08487	LC55x_128	LC55x_128; hypothetical protein
OG_08488	LC55x_137	LC55x_137; hypothetical protein
OG_08489	LC55x_140	LC55x_140; hypothetical protein
OG_08490	LC55x_147	LC55x_147; hypothetical protein
OG_08491	LC55x_177	LC55x_177; hypothetical protein
OG_08492	LC55x_178	LC55x_178; hypothetical protein
OG_08493	LC55x_181	LC55x_181; hypothetical protein
OG_08494	LC55x_204	LC55x_204; hypothetical protein
OG_08495	LC55x_212	LC55x_212; hypothetical protein
OG_08496	LC55x_215	LC55x_215; hypothetical protein
OG_08497	LC55x_218	LC55x_218; hypothetical protein
OG_08498	LC55x_221	LC55x_221; hypothetical protein
OG_08499	LC55x_224	LC55x_224; hypothetical protein
OG_08500	LC55x_232	LC55x_232; hypothetical protein
OG_08501	LC55x_234	LC55x_234; hypothetical protein
OG_08502	LC55x_235	LC55x_235; hypothetical protein
OG_08503	LC55x_251	LC55x_251; hypothetical protein
OG_08504	LC55x_257	LC55x_257; hypothetical protein
OG_08505	LC55x_260	LC55x_260; hypothetical protein
OG_08506	LC55x_271	LC55x_271; hypothetical protein
OG_08507	LC55x_275	LC55x_275; hypothetical protein
OG_08508	LC55x_280	LC55x_280; hypothetical protein
OG_08509	LC55x_292	LC55x_292; hypothetical protein
OG_08510	LC55x_296	LC55x_296; hypothetical protein
OG_08511	LC55x_298	LC55x_298; hypothetical protein
OG_08512	LC55x_301	LC55x_301; hypothetical protein
OG_08513	LC55x_304	LC55x_304; hypothetical protein
OG_08514	LC55x_326	LC55x_326; hypothetical protein
OG_08515	LC55x_355	LC55x_355; hypothetical protein

Orthologous group	*L. cap* AZ78<br>Acc. no. (#451)<br>8.8% of genome	Gene description
OG_04628	EYR65335.1	hypothetical protein AZ78_27000 [Lysobacter capsici AZ78]
OG_04628	EYR67207.1	hypothetical protein AZ78_16675 [Lysobacter capsici AZ78]
OG_04628	EYR67209.1	hypothetical protein AZ78_16685 [Lysobacter capsici AZ78]
OG_06100	EYR70141.1	hypothetical protein AZ78_02785 [Lysobacter capsici AZ78]
OG_06100	EYR66491.1	hypothetical protein AZ78_21105 [Lysobacter capsici AZ78]
OG_06101	EYR67208.1	hypothetical protein AZ78_16680 [Lysobacter capsici AZ78]
OG_06101	EYR65334.1	hypothetical protein AZ78_26995 [Lysobacter capsici AZ78]
OG_06102	EYR67302.1	N-acetylmuramoyl-L-alanine amidase [Lysobacter capsici AZ78]
OG_06102	EYR67292.1	lysozyme [Lysobacter capsici AZ78]
OG_06103	EYR65336.1	hypothetical protein AZ78_26980 [Lysobacter capsici AZ78]
OG_06103	EYR65340.1	hypothetical protein AZ78_26955 [Lysobacter capsici AZ78]
OG_06104	EYR66151.1	hypothetical protein AZ78_22545 [Lysobacter capsici AZ78]
OG_06104	EYR66150.1	hypothetical protein AZ78_22540 [Lysobacter capsici AZ78]
OG_06105	EYR65883.1	hypothetical protein AZ78_24105 [Lysobacter capsici AZ78]
OG_06105	EYR65882.1	hypothetical protein AZ78_24100 [Lysobacter capsici AZ78]
OG_06547	EYR70460.1	hypothetical protein AZ78_01560 [Lysobacter capsici AZ78]
OG_06548	EYR70449.1	hypothetical protein AZ78_01320 [Lysobacter capsici AZ78]
OG_06549	EYR70434.1	hypothetical protein AZ78_00880 [Lysobacter capsici AZ78]
OG_06550	EYR70428.1	hypothetical protein AZ78_00535 [Lysobacter capsici AZ78]
OG_06551	EYR70427.1	hypothetical protein AZ78_00525 [Lysobacter capsici AZ78]
OG_06552	EYR70426.1	hypothetical protein AZ78_00510 [Lysobacter capsici AZ78]
OG_06553	EYR70425.1	hypothetical protein AZ78_00500 [Lysobacter capsici AZ78]
OG_06554	EYR70424.1	hypothetical protein AZ78_00495 [Lysobacter capsici AZ78]
OG_06555	EYR70423.1	hypothetical protein AZ78_00490 [Lysobacter capsici AZ78]
OG_06556	EYR70422.1	hypothetical protein AZ78_00480 [Lysobacter capsici AZ78]
OG_06557	EYR70412.1	hypothetical protein AZ78_00325 [Lysobacter capsici AZ78]
OG_06558	EYR70411.1	hypothetical protein AZ78_00320 [Lysobacter capsici AZ78]
OG_06559	EYR70408.1	hypothetical protein AZ78_00160 [Lysobacter capsici AZ78]
OG_06560	EYR70407.1	hypothetical protein AZ78_00155 [Lysobacter capsici AZ78]
OG_06561	EYR70406.1	hypothetical protein AZ78_00150 [Lysobacter capsici AZ78]
OG_06562	EYR70405.1	hypothetical protein AZ78_00140 [Lysobacter capsici AZ78]
OG_06563	EYR70404.1	hypothetical protein AZ78_00135 [Lysobacter capsici AZ78]
OG_06564	EYR70398.1	hypothetical protein AZ78_00100 [Lysobacter capsici AZ78]
OG_06565	EYR70393.1	hypothetical protein AZ78_00050 [Lysobacter capsici AZ78]
OG_06566	EYR70219.1	enhanced serine sensitivity protein SseB [Lysobacter capsici AZ78]
OG_06567	EYR70218.1	hypothetical protein AZ78_00540 [Lysobacter capsici AZ78]
OG_06568	EYR70217.1	hypothetical protein AZ78_00530 [Lysobacter capsici AZ78]
OG_06569	EYR70216.1	NTPase KAP [Lysobacter capsici AZ78]
OG_06570	EYR70215.1	hypothetical protein AZ78_00515 [Lysobacter capsici AZ78]
OG_06571	EYR70213.1	recombinase [Lysobacter capsici AZ78]
OG_06572	EYR70164.1	molecular chaperone Tir [Lysobacter capsici AZ78]
OG_06573	EYR70152.1	hypothetical protein AZ78_02950 [Lysobacter capsici AZ78]
OG_06574	EYR70150.1	hypothetical protein AZ78_02940 [Lysobacter capsici AZ78]
OG_06575	EYR70142.1	hypothetical protein AZ78_02795 [Lysobacter capsici AZ78]
OG_06576	EYR70132.1	hypothetical protein AZ78_02500 [Lysobacter capsici AZ78]
OG_06577	EYR70131.1	hypothetical protein AZ78_02415 [Lysobacter capsici AZ78]
OG_06578	EYR70130.1	hypothetical protein AZ78_02410 [Lysobacter capsici AZ78]
OG_06579	EYR70129.1	hypothetical protein AZ78_02405 [Lysobacter capsici AZ78]
OG_06580	EYR70128.1	hypothetical protein AZ78_02395 [Lysobacter capsici AZ78]
OG_06581	EYR70118.1	hypothetical protein AZ78_02260 [Lysobacter capsici AZ78]
OG_06582	EYR70112.1	hypothetical protein AZ78_02170 [Lysobacter capsici AZ78]
OG_06583	EYR70110.1	hypothetical protein AZ78_02135 [Lysobacter capsici AZ78]
OG_06584	EYR70107.1	hypothetical protein AZ78_02095 [Lysobacter capsici AZ78]
OG_06585	EYR70095.1	hypothetical protein AZ78_01910 [Lysobacter capsici AZ78]
OG_06586	EYR70093.1	hypothetical protein AZ78_01880 [Lysobacter capsici AZ78]
OG_06587	EYR70071.1	hypothetical protein AZ78_02915 [Lysobacter capsici AZ78]
OG_06588	EYR70031.1	hypothetical protein AZ78_02625 [Lysobacter capsici AZ78]
OG_06589	EYR69978.1	hypothetical protein AZ78_02270 [Lysobacter capsici AZ78]
OG_06590	EYR69977.1	hypothetical protein AZ78_02265 [Lysobacter capsici AZ78]
OG_06591	EYR69922.1	hypothetical protein AZ78_01825 [Lysobacter capsici AZ78]
OG_06592	EYR69832.1	hypothetical protein AZ78_03805 [Lysobacter capsici AZ78]
OG_06593	EYR69826.1	hypothetical protein AZ78_03705 [Lysobacter capsici AZ78]
OG_06594	EYR69825.1	hypothetical protein AZ78_03700 [Lysobacter capsici AZ78]
OG_06595	EYR69822.1	hypothetical protein AZ78_03565 [Lysobacter capsici AZ78]
OG_06596	EYR69811.1	hypothetical protein AZ78_03285 [Lysobacter capsici AZ78]
OG_06597	EYR69810.1	hypothetical protein AZ78_03175 [Lysobacter capsici AZ78]
OG_06598	EYR69804.1	hypothetical protein AZ78_03110 [Lysobacter capsici AZ78]

OG_08516	LC55x_388	LC55x_388; hypothetical protein
OG_08517	LC55x_392	LC55x_392; nucleoside 2-deoxyribosyltransferase family protein
OG_08518	LC55x_393	LC55x_393; conserved hypothetical protein
OG_08519	LC55x_394	LC55x_394; hypothetical protein
OG_08520	LC55x_395	LC55x_395; penicillin-Binding Protein family protein
OG_08521	LC55x_397	LC55x_397; uvrD/REP helicase N-terminal domain protein
OG_08522	LC55x_398	LC55x_398; AAA ATPase domain protein
OG_08523	LC55x_399	LC55x_399; conserved hypothetical protein
OG_08524	LC55x_401	LC55x_401; conserved hypothetical protein
OG_08525	LC55x_402	LC55x_402; bacterial regulatory helix-turn-helix , lysR family protein
OG_08526	LC55x_404	LC55x_404; transcriptional regulator, XRE family
OG_08527	LC55x_403	LC55x_403; conserved hypothetical protein
OG_08528	LC55x_405	LC55x_405; conserved hypothetical protein
OG_08529	LC55x_407	LC55x_407; hypothetical protein
OG_08530	LC55x_408	LC55x_408; flavodoxin family protein
OG_08531	LC55x_409	LC55x_409; aldo/keto reductase family protein
OG_08532	LC55x_410	LC55x_410; hypothetical protein
OG_08533	LC55x_411	LC55x_411; hypothetical protein
OG_08534	LC55x_412	LC55x_412; hypothetical protein
OG_08535	LC55x_414	LC55x_414; hypothetical protein
OG_08536	LC55x_427	LC55x_427; hypothetical protein
OG_08537	LC55x_437	LC55x_437; hypothetical protein
OG_08538	LC55x_453	LC55x_453; hypothetical protein
OG_08539	LC55x_464	LC55x_464; hypothetical protein
OG_08540	LC55x_500	LC55x_500; SEC-C motif family protein
OG_08541	LC55x_516	LC55x_516; hypothetical protein
OG_08542	LC55x_551	LC55x_551; hypothetical protein
OG_08543	LC55x_557	LC55x_557; hypothetical protein
OG_08544	LC55x_559	LC55x_559; hypothetical protein
OG_08545	LC55x_560	LC55x_560; hypothetical protein
OG_08546	LC55x_591	LC55x_591; hypothetical protein
OG_08547	LC55x_593	LC55x_593; hypothetical protein
OG_08548	LC55x_600	LC55x_600; hypothetical protein
OG_08549	LC55x_611	LC55x_611; subtilase family protein
OG_08550	LC55x_616	LC55x_616; hypothetical protein
OG_08551	LC55x_619	LC55x_619; hypothetical protein
OG_08552	LC55x_626	LC55x_626; FAD binding domain protein
OG_08553	LC55x_628	LC55x_628; hypothetical protein
OG_08554	LC55x_641	LC55x_641; hypothetical protein
OG_08555	LC55x_643	LC55x_643; hypothetical protein
OG_08556	LC55x_644	LC55x_644; hypothetical protein
OG_08557	LC55x_649	LC55x_649; hypothetical protein
OG_08558	LC55x_663	LC55x_663; hypothetical protein
OG_08559	LC55x_664	LC55x_664; hypothetical protein
OG_08560	LC55x_667	LC55x_667; hypothetical protein
OG_08561	LC55x_669	LC55x_669; hypothetical protein
OG_08562	LC55x_670	LC55x_670; helix-turn-helix family protein
OG_08563	LC55x_694	LC55x_694; hypothetical protein
OG_08564	LC55x_697	LC55x_697; hypothetical protein
OG_08565	LC55x_699	LC55x_699; hypothetical protein
OG_08566	LC55x_704	LC55x_704; hypothetical protein
OG_08567	LC55x_714	LC55x_714; hypothetical protein
OG_08568	LC55x_717	LC55x_717; hypothetical protein
OG_08569	LC55x_754	LC55x_754; hypothetical protein
OG_08570	LC55x_767	LC55x_767; hypothetical protein
OG_08571	LC55x_800	LC55x_800; radical SAM superfamily protein
OG_08572	LC55x_801	LC55x_801; HEXXH motif domain protein
OG_08573	LC55x_802	LC55x_802; ABC transporter family protein
OG_08574	LC55x_803	LC55x_803; hypothetical protein
OG_08575	LC55x_807	LC55x_807; hypothetical protein
OG_08576	LC55x_821	LC55x_821; hypothetical protein
OG_08577	LC55x_826	LC55x_826; hypothetical protein
OG_08578	LC55x_831	LC55x_831; hypothetical protein
OG_08579	LC55x_833	LC55x_833; hypothetical protein
OG_08580	LC55x_852	LC55x_852; hypothetical protein
OG_08581	LC55x_858	LC55x_858; hypothetical protein
OG_08582	LC55x_884	LC55x_884; hypothetical protein
OG_08583	LC55x_886	LC55x_886; hypothetical protein
OG_08584	LC55x_896	LC55x_896; hypothetical protein
OG_08585	LC55x_905	LC55x_905; hypothetical protein
OG_08586	LC55x_916	LC55x_916; hypothetical protein

OG_06599	EYR69682.1	kinase [Lysobacter capsici AZ78]
OG_06600	EYR69681.1	guanylyltransferase [Lysobacter capsici AZ78]
OG_06601	EYR69451.1	hypothetical protein AZ78_06020 [Lysobacter capsici AZ78]
OG_06602	EYR69441.1	hypothetical protein AZ78_05970 [Lysobacter capsici AZ78]
OG_06603	EYR69427.1	hypothetical protein AZ78_05900 [Lysobacter capsici AZ78]
OG_06604	EYR69388.1	hypothetical protein AZ78_05705 [Lysobacter capsici AZ78]
OG_06605	EYR69387.1	hypothetical protein AZ78_05695 [Lysobacter capsici AZ78]
OG_06606	EYR69377.1	hypothetical protein AZ78_05645 [Lysobacter capsici AZ78]
OG_06607	EYR69352.1	hypothetical protein AZ78_05520 [Lysobacter capsici AZ78]
OG_06608	EYR69333.1	hypothetical protein AZ78_05425 [Lysobacter capsici AZ78]
OG_06609	EYR69294.1	hypothetical protein AZ78_05210 [Lysobacter capsici AZ78]
OG_06610	EYR69293.1	hypothetical protein AZ78_05205 [Lysobacter capsici AZ78]
OG_06611	EYR69287.1	hypothetical protein AZ78_05175 [Lysobacter capsici AZ78]
OG_06612	EYR69283.1	hypothetical protein AZ78_05155 [Lysobacter capsici AZ78]
OG_06613	EYR69281.1	hypothetical protein AZ78_07120 [Lysobacter capsici AZ78]
OG_06614	EYR69278.1	hypothetical protein AZ78_07025 [Lysobacter capsici AZ78]
OG_06615	EYR69277.1	hypothetical protein AZ78_07020 [Lysobacter capsici AZ78]
OG_06616	EYR69276.1	hypothetical protein AZ78_07015 [Lysobacter capsici AZ78]
OG_06617	EYR69268.1	hypothetical protein AZ78_06770 [Lysobacter capsici AZ78]
OG_06618	EYR69264.1	hypothetical protein AZ78_06665 [Lysobacter capsici AZ78]
OG_06619	EYR69262.1	hypothetical protein AZ78_06645 [Lysobacter capsici AZ78]
OG_06620	EYR69248.1	hypothetical protein AZ78_06280 [Lysobacter capsici AZ78]
OG_06621	EYR69247.1	hypothetical protein AZ78_06180 [Lysobacter capsici AZ78]
OG_06622	EYR69212.1	glycosyl transferase family 1 [Lysobacter capsici AZ78]
OG_06623	EYR69067.1	hypothetical protein AZ78_08260 [Lysobacter capsici AZ78]
OG_06624	EYR69052.1	hypothetical protein AZ78_08040 [Lysobacter capsici AZ78]
OG_06625	EYR69044.1	hypothetical protein AZ78_07970 [Lysobacter capsici AZ78]
OG_06626	EYR69043.1	hypothetical protein AZ78_07955 [Lysobacter capsici AZ78]
OG_06627	EYR69035.1	hypothetical protein AZ78_07885 [Lysobacter capsici AZ78]
OG_06628	EYR69024.1	hypothetical protein AZ78_07785 [Lysobacter capsici AZ78]
OG_06629	EYR69019.1	hypothetical protein AZ78_07725 [Lysobacter capsici AZ78]
OG_06630	EYR69018.1	hypothetical protein AZ78_07700 [Lysobacter capsici AZ78]
OG_06631	EYR69012.1	hypothetical protein AZ78_07660 [Lysobacter capsici AZ78]
OG_06632	EYR69010.1	hypothetical protein AZ78_07630 [Lysobacter capsici AZ78]
OG_06633	EYR69008.1	hypothetical protein AZ78_07620 [Lysobacter capsici AZ78]
OG_06634	EYR69007.1	hypothetical protein AZ78_07615 [Lysobacter capsici AZ78]
OG_06635	EYR69006.1	hypothetical protein AZ78_07610 [Lysobacter capsici AZ78]
OG_06636	EYR69005.1	hypothetical protein AZ78_07605 [Lysobacter capsici AZ78]
OG_06637	EYR69004.1	hypothetical protein AZ78_07600 [Lysobacter capsici AZ78]
OG_06638	EYR69003.1	hypothetical protein AZ78_07595 [Lysobacter capsici AZ78]
OG_06639	EYR68996.1	hypothetical protein AZ78_07390 [Lysobacter capsici AZ78]
OG_06640	EYR68929.1	hypothetical protein AZ78_07845 [Lysobacter capsici AZ78]
OG_06641	EYR68843.1	hypothetical protein AZ78_09335 [Lysobacter capsici AZ78]
OG_06642	EYR68842.1	hypothetical protein AZ78_09330 [Lysobacter capsici AZ78]
OG_06643	EYR68833.1	hypothetical protein AZ78_09235 [Lysobacter capsici AZ78]
OG_06644	EYR68828.1	hypothetical protein AZ78_09185 [Lysobacter capsici AZ78]
OG_06645	EYR68824.1	hypothetical protein AZ78_09070 [Lysobacter capsici AZ78]
OG_06646	EYR68821.1	hypothetical protein AZ78_09005 [Lysobacter capsici AZ78]
OG_06647	EYR68816.1	hypothetical protein AZ78_08915 [Lysobacter capsici AZ78]
OG_06648	EYR68814.1	hypothetical protein AZ78_08865 [Lysobacter capsici AZ78]
OG_06649	EYR68813.1	hypothetical protein AZ78_08860 [Lysobacter capsici AZ78]
OG_06650	EYR68804.1	hypothetical protein AZ78_08590 [Lysobacter capsici AZ78]
OG_06651	EYR68800.1	hypothetical protein AZ78_08555 [Lysobacter capsici AZ78]
OG_06652	EYR68794.1	hypothetical protein AZ78_08390 [Lysobacter capsici AZ78]
OG_06653	EYR68718.1	hypothetical protein AZ78_08900 [Lysobacter capsici AZ78]
OG_06654	EYR68717.1	hypothetical protein AZ78_08895 [Lysobacter capsici AZ78]
OG_06655	EYR68716.1	DNA mismatch repair protein [Lysobacter capsici AZ78]
OG_06656	EYR68715.1	DNA-cytosine methyltransferase [Lysobacter capsici AZ78]
OG_06657	EYR68640.1	hypothetical protein AZ78_11035 [Lysobacter capsici AZ78]
OG_06658	EYR68639.1	hypothetical protein AZ78_10975 [Lysobacter capsici AZ78]
OG_06659	EYR68637.1	hypothetical protein AZ78_10955 [Lysobacter capsici AZ78]
OG_06660	EYR68636.1	hypothetical protein AZ78_10935 [Lysobacter capsici AZ78]
OG_06661	EYR68635.1	hypothetical protein AZ78_10930 [Lysobacter capsici AZ78]
OG_06662	EYR68632.1	hypothetical protein AZ78_10855 [Lysobacter capsici AZ78]
OG_06663	EYR68630.1	hypothetical protein AZ78_10835 [Lysobacter capsici AZ78]
OG_06664	EYR68629.1	hypothetical protein AZ78_10830 [Lysobacter capsici AZ78]
OG_06665	EYR68628.1	hypothetical protein AZ78_10820 [Lysobacter capsici AZ78]
OG_06666	EYR68627.1	hypothetical protein AZ78_10815 [Lysobacter capsici AZ78]
OG_06667	EYR68626.1	hypothetical protein AZ78_10810 [Lysobacter capsici AZ78]
OG_06668	EYR68624.1	hypothetical protein AZ78_10790 [Lysobacter capsici AZ78]
OG_06669	EYR68622.1	hypothetical protein AZ78_10770 [Lysobacter capsici AZ78]

OG_08587	LC55x_938	LC55x_938; hypothetical protein
OG_08588	LC55x_943	LC55x_943; hypothetical protein
OG_08589	LC55x_951	LC55x_951; hypothetical protein
OG_08590	LC55x_952	LC55x_952; hypothetical protein
OG_08591	LC55x_958	LC55x_958; hypothetical protein
OG_08592	LC55x_995	LC55x_995; hypothetical protein
OG_08593	LC55x_1012	LC55x_1012; hypothetical protein
OG_08594	LC55x_1018	LC55x_1018; hypothetical protein
OG_08595	LC55x_1036	LC55x_1036; hypothetical protein
OG_08596	LC55x_1048	LC55x_1048; hypothetical protein
OG_08597	LC55x_1050	LC55x_1050; hypothetical protein
OG_08598	LC55x_1062	LC55x_1062; hypothetical protein
OG_08599	LC55x_1064	LC55x_1064; hypothetical protein
OG_08600	LC55x_1074	aprA; alkaline metalloase domain protein
OG_08601	LC55x_1078	LC55x_1078; hypothetical protein
OG_08602	LC55x_1101	LC55x_1101; hypothetical protein
OG_08603	LC55x_1120	LC55x_1120; hypothetical protein
OG_08604	LC55x_1124	LC55x_1124; hypothetical protein
OG_08605	LC55x_1128	LC55x_1128; conserved hypothetical protein
OG_08606	LC55x_1132	LC55x_1132; Reverse
OG_08607	LC55x_1134	LC55x_1134; hypothetical protein
OG_08608	LC55x_1146	LC55x_1146; hypothetical protein
OG_08609	LC55x_1147	LC55x_1147; hypothetical protein
OG_08610	LC55x_1152	LC55x_1152; hypothetical protein
OG_08611	LC55x_1157	LC55x_1157; hypothetical protein
OG_08612	LC55x_1170	LC55x_1170; hypothetical protein
OG_08613	LC55x_1183	LC55x_1183; hypothetical protein
OG_08614	LC55x_1192	LC55x_1192; hypothetical protein
OG_08615	LC55x_1194	LC55x_1194; hypothetical protein
OG_08616	LC55x_1195	LC55x_1195; hypothetical protein
OG_08617	LC55x_1196	LC55x_1196; hypothetical protein
OG_08618	LC55x_1212	LC55x_1212; hypothetical protein
OG_08619	LC55x_1215	LC55x_1215; hypothetical protein
OG_08620	LC55x_1229	LC55x_1229; hypothetical protein
OG_08621	LC55x_1237	LC55x_1237; hypothetical protein
OG_08622	LC55x_1243	LC55x_1243; hypothetical protein
OG_08623	LC55x_1245	LC55x_1245; hypothetical protein
OG_08624	LC55x_1268	LC55x_1268; hypothetical protein
OG_08625	LC55x_1288	LC55x_1288; hypothetical protein
OG_08626	LC55x_1329	LC55x_1329; hypothetical protein
OG_08627	LC55x_1336	LC55x_1336; hypothetical protein
OG_08628	LC55x_1343	LC55x_1343; hypothetical protein
OG_08629	LC55x_1342	LC55x_1342; hypothetical protein
OG_08630	LC55x_1355	LC55x_1355; hypothetical protein
OG_08631	LC55x_1385	LC55x_1385; putative transposase
OG_08632	LC55x_1387	LC55x_1387; hypothetical protein
OG_08633	LC55x_1410	LC55x_1410; hypothetical protein
OG_08634	LC55x_1412	LC55x_1412; hypothetical protein
OG_08635	LC55x_1419	LC55x_1419; hypothetical protein
OG_08636	LC55x_1421	LC55x_1421; hypothetical protein
OG_08637	LC55x_1425	LC55x_1425; hypothetical protein
OG_08638	LC55x_1426	LC55x_1426; hypothetical protein
OG_08639	LC55x_1436	LC55x_1436; hypothetical protein
OG_08640	LC55x_1447	LC55x_1447; hypothetical protein
OG_08641	LC55x_1448	LC55x_1448; hypothetical protein
OG_08642	LC55x_1449	LC55x_1449; hypothetical protein
OG_08643	LC55x_1452	LC55x_1452; hypothetical protein
OG_08644	LC55x_1453	LC55x_1453; hypothetical protein
OG_08645	LC55x_1454	LC55x_1454; intein C-terminal splicing region domain protein
OG_08646	LC55x_1502	LC55x_1502; hypothetical protein
OG_08647	LC55x_1513	LC55x_1513; hypothetical protein
OG_08648	LC55x_1517	LC55x_1517; hypothetical protein
OG_08649	LC55x_1519	LC55x_1519; hypothetical protein
OG_08650	LC55x_1525	LC55x_1525; hypothetical protein
OG_08651	LC55x_1540	LC55x_1540; hypothetical protein
OG_08652	LC55x_1539	xth; exodeoxyribonuclease III
OG_08653	LC55x_1543	LC55x_1543; hypothetical protein
OG_08654	LC55x_1556	LC55x_1556; hypothetical protein
OG_08655	LC55x_1566	LC55x_1566; hypothetical protein
OG_08656	LC55x_1577	LC55x_1577; hypothetical protein
OG_08657	LC55x_1586	LC55x_1586; hypothetical protein

OG_06670	EYR68621.1	hypothetical protein AZ78_10760 [Lysobacter capsici AZ78]
OG_06671	EYR68620.1	hypothetical protein AZ78_10750 [Lysobacter capsici AZ78]
OG_06672	EYR68619.1	hypothetical protein AZ78_10745 [Lysobacter capsici AZ78]
OG_06673	EYR68618.1	hypothetical protein AZ78_10730 [Lysobacter capsici AZ78]
OG_06674	EYR68617.1	hypothetical protein AZ78_10725 [Lysobacter capsici AZ78]
OG_06675	EYR68616.1	hypothetical protein AZ78_10705 [Lysobacter capsici AZ78]
OG_06676	EYR68615.1	hypothetical protein AZ78_10695 [Lysobacter capsici AZ78]
OG_06677	EYR68614.1	hypothetical protein AZ78_10690 [Lysobacter capsici AZ78]
OG_06678	EYR68613.1	hypothetical protein AZ78_10675 [Lysobacter capsici AZ78]
OG_06679	EYR68612.1	hypothetical protein AZ78_10670 [Lysobacter capsici AZ78]
OG_06680	EYR68611.1	hypothetical protein AZ78_10665 [Lysobacter capsici AZ78]
OG_06681	EYR68610.1	hypothetical protein AZ78_10660 [Lysobacter capsici AZ78]
OG_06682	EYR68609.1	hypothetical protein AZ78_10640 [Lysobacter capsici AZ78]
OG_06683	EYR68608.1	hypothetical protein AZ78_10630 [Lysobacter capsici AZ78]
OG_06684	EYR68607.1	hypothetical protein AZ78_10605 [Lysobacter capsici AZ78]
OG_06685	EYR68606.1	hypothetical protein AZ78_10600 [Lysobacter capsici AZ78]
OG_06686	EYR68605.1	hypothetical protein AZ78_10595 [Lysobacter capsici AZ78]
OG_06687	EYR68604.1	hypothetical protein AZ78_10590 [Lysobacter capsici AZ78]
OG_06688	EYR68595.1	hypothetical protein AZ78_10400 [Lysobacter capsici AZ78]
OG_06689	EYR68587.1	hypothetical protein AZ78_10130 [Lysobacter capsici AZ78]
OG_06690	EYR68584.1	hypothetical protein AZ78_10095 [Lysobacter capsici AZ78]
OG_06691	EYR68581.1	hypothetical protein AZ78_10060 [Lysobacter capsici AZ78]
OG_06692	EYR68576.1	hypothetical protein AZ78_09930 [Lysobacter capsici AZ78]
OG_06693	EYR68561.1	hypothetical protein AZ78_09820 [Lysobacter capsici AZ78]
OG_06694	EYR68560.1	hypothetical protein AZ78_09810 [Lysobacter capsici AZ78]
OG_06695	EYR68549.1	hypothetical protein AZ78_09650 [Lysobacter capsici AZ78]
OG_06696	EYR68542.1	hypothetical protein AZ78_09475 [Lysobacter capsici AZ78]
OG_06697	EYR68506.1	hypothetical protein AZ78_10800 [Lysobacter capsici AZ78]
OG_06698	EYR68505.1	hypothetical protein AZ78_10795 [Lysobacter capsici AZ78]
OG_06699	EYR68502.1	hypothetical protein AZ78_10765 [Lysobacter capsici AZ78]
OG_06700	EYR68499.1	hypothetical protein AZ78_10735 [Lysobacter capsici AZ78]
OG_06701	EYR68497.1	integrase [Lysobacter capsici AZ78]
OG_06702	EYR68496.1	Appr-1-p processing enzyme [Lysobacter capsici AZ78]
OG_06703	EYR68495.1	hypothetical protein AZ78_10700 [Lysobacter capsici AZ78]
OG_06704	EYR68494.1	serine/threonine phosphatase [Lysobacter capsici AZ78]
OG_06705	EYR68493.1	hypothetical protein AZ78_10680 [Lysobacter capsici AZ78]
OG_06706	EYR68492.1	RadC family protein [Lysobacter capsici AZ78]
OG_06707	EYR68491.1	membrane protein [Lysobacter capsici AZ78]
OG_06708	EYR68487.1	hypothetical protein AZ78_10620 [Lysobacter capsici AZ78]
OG_06709	EYR68486.1	hypothetical protein AZ78_10615 [Lysobacter capsici AZ78]
OG_06710	EYR68482.1	hypothetical protein AZ78_10575 [Lysobacter capsici AZ78]
OG_06711	EYR68298.1	hypothetical protein AZ78_11575 [Lysobacter capsici AZ78]
OG_06712	EYR68297.1	hypothetical protein AZ78_11570 [Lysobacter capsici AZ78]
OG_06713	EYR68296.1	hypothetical protein AZ78_11565 [Lysobacter capsici AZ78]
OG_06714	EYR68279.1	hypothetical protein AZ78_11475 [Lysobacter capsici AZ78]
OG_06715	EYR68277.1	hypothetical protein AZ78_11465 [Lysobacter capsici AZ78]
OG_06716	EYR68262.1	hypothetical protein AZ78_11390 [Lysobacter capsici AZ78]
OG_06717	EYR68239.1	hypothetical protein AZ78_11275 [Lysobacter capsici AZ78]
OG_06718	EYR68225.1	hypothetical protein AZ78_11205 [Lysobacter capsici AZ78]
OG_06719	EYR68212.1	hypothetical protein AZ78_11140 [Lysobacter capsici AZ78]
OG_06720	EYR68210.1	hypothetical protein AZ78_11120 [Lysobacter capsici AZ78]
OG_06721	EYR68209.1	hypothetical protein AZ78_11115 [Lysobacter capsici AZ78]
OG_06722	EYR68208.1	hypothetical protein AZ78_11110 [Lysobacter capsici AZ78]
OG_06723	EYR68207.1	hypothetical protein AZ78_11100 [Lysobacter capsici AZ78]
OG_06724	EYR68206.1	hypothetical protein AZ78_11090 [Lysobacter capsici AZ78]
OG_06725	EYR68201.1	hypothetical protein AZ78_12260 [Lysobacter capsici AZ78]
OG_06726	EYR68194.1	hypothetical protein AZ78_12190 [Lysobacter capsici AZ78]
OG_06727	EYR68193.1	hypothetical protein AZ78_12180 [Lysobacter capsici AZ78]
OG_06728	EYR68192.1	hypothetical protein AZ78_12175 [Lysobacter capsici AZ78]
OG_06729	EYR68190.1	hypothetical protein AZ78_12165 [Lysobacter capsici AZ78]
OG_06730	EYR68189.1	hypothetical protein AZ78_12160 [Lysobacter capsici AZ78]
OG_06731	EYR68164.1	hypothetical protein AZ78_12185 [Lysobacter capsici AZ78]
OG_06732	EYR68087.1	hypothetical protein AZ78_12605 [Lysobacter capsici AZ78]
OG_06733	EYR68084.1	hypothetical protein AZ78_12520 [Lysobacter capsici AZ78]
OG_06734	EYR68078.1	hypothetical protein AZ78_12410 [Lysobacter capsici AZ78]
OG_06735	EYR68073.1	hypothetical protein AZ78_12305 [Lysobacter capsici AZ78]
OG_06736	EYR68068.1	hypothetical protein AZ78_12800 [Lysobacter capsici AZ78]
OG_06737	EYR67993.1	membrane protein, partial [Lysobacter capsici AZ78]
OG_06738	EYR67987.1	hypothetical protein AZ78_13345 [Lysobacter capsici AZ78]
OG_06739	EYR67977.1	hypothetical protein AZ78_13295 [Lysobacter capsici AZ78]
OG_06740	EYR67971.1	hypothetical protein AZ78_13265 [Lysobacter capsici AZ78]

OG_08658	LC55x_1591	LC55x_1591; hypothetical protein
OG_08659	LC55x_1590	LC55x_1590; hypothetical protein
OG_08660	LC55x_1601	LC55x_1601; hypothetical protein
OG_08661	LC55x_1607	LC55x_1607; hypothetical protein
OG_08662	LC55x_1629	LC55x_1629; hypothetical protein
OG_08663	LC55x_1630	LC55x_1630; hypothetical protein
OG_08664	LC55x_1631	LC55x_1631; conserved hypothetical protein
OG_08665	LC55x_1632	LC55x_1632; hypothetical protein
OG_08666	LC55x_1633	LC55x_1633; hypothetical protein
OG_08667	LC55x_1634	LC55x_1634; hypothetical protein
OG_08668	LC55x_1635	LC55x_1635; hypothetical protein
OG_08669	LC55x_1636	LC55x_1636; hypothetical protein
OG_08670	LC55x_1637	LC55x_1637; hypothetical protein
OG_08671	LC55x_1638	LC55x_1638; hypothetical protein
OG_08672	LC55x_1639	LC55x_1639; MASE1 family protein
OG_08673	LC55x_1640	LC55x_1640; hypothetical protein
OG_08674	LC55x_1641	LC55x_1641; conserved hypothetical protein
OG_08675	LC55x_1642	LC55x_1642; dnaB-like helicase N terminal domain protein
OG_08676	LC55x_1643	LC55x_1643; phage holin 6 family protein
OG_08677	LC55x_1644	R; lysozyme
OG_08678	LC55x_1645	LC55x_1645; hypothetical protein
OG_08679	LC55x_1646	LC55x_1646; HNH endonuclease family protein
OG_08680	LC55x_1647	LC55x_1647; hypothetical protein
OG_08681	LC55x_1648	LC55x_1648; hypothetical protein
OG_08682	LC55x_1649	LC55x_1649; hypothetical protein
OG_08683	LC55x_1658	LC55x_1658; hypothetical protein
OG_08684	LC55x_1665	LC55x_1665; hypothetical protein
OG_08685	LC55x_1666	LC55x_1666; hypothetical protein
OG_08686	LC55x_1686	LC55x_1686; hypothetical protein
OG_08687	LC55x_1694	LC55x_1694; hypothetical protein
OG_08688	LC55x_1696	LC55x_1696; hypothetical protein
OG_08689	LC55x_1699	LC55x_1699; hypothetical protein
OG_08690	LC55x_1719	LC55x_1719; hypothetical protein
OG_08691	LC55x_1722	LC55x_1722; hypothetical protein
OG_08692	LC55x_1728	LC55x_1728; hypothetical protein
OG_08693	LC55x_1732	LC55x_1732; hypothetical protein
OG_08694	LC55x_1753	LC55x_1753; hypothetical protein
OG_08695	LC55x_1769	LC55x_1769; transposase DDE domain protein
OG_08696	LC55x_1770	LC55x_1770; hypothetical protein
OG_08697	LC55x_1773	LC55x_1773; hypothetical protein
OG_08698	LC55x_1786	LC55x_1786; hypothetical protein
OG_08699	LC55x_1792	LC55x_1792; hypothetical protein
OG_08700	LC55x_1794	LC55x_1794; hypothetical protein
OG_08701	LC55x_1806	LC55x_1806; putative dNA methylase
OG_08702	LC55x_1807	LC55x_1807; conserved hypothetical protein
OG_08703	LC55x_1808	LC55x_1808; hypothetical protein
OG_08704	LC55x_1809	LC55x_1809; hypothetical protein
OG_08705	LC55x_1813	LC55x_1813; hypothetical protein
OG_08706	LC55x_1833	LC55x_1833; hypothetical protein
OG_08707	LC55x_1848	LC55x_1848; hypothetical protein
OG_08708	LC55x_1849	LC55x_1849; hypothetical protein
OG_08709	LC55x_1878	LC55x_1878; hypothetical protein
OG_08710	LC55x_1879	LC55x_1879; hypothetical protein
OG_08711	LC55x_1888	LC55x_1888; hypothetical protein
OG_08712	LC55x_1910	LC55x_1910; hypothetical protein
OG_08713	LC55x_1916	LC55x_1916; hypothetical protein
OG_08714	LC55x_1918	LC55x_1918; hypothetical protein
OG_08715	LC55x_1920	LC55x_1920; hypothetical protein
OG_08716	LC55x_1938	LC55x_1938; hypothetical protein
OG_08717	LC55x_1944	LC55x_1944; hypothetical protein
OG_08718	LC55x_1959	LC55x_1959; hypothetical protein
OG_08719	LC55x_1966	LC55x_1966; hypothetical protein
OG_08720	LC55x_1979	LC55x_1979; hypothetical protein
OG_08721	LC55x_1981	LC55x_1981; hypothetical protein
OG_08722	LC55x_1986	LC55x_1986; hypothetical protein
OG_08723	LC55x_1991	LC55x_1991; conserved hypothetical protein
OG_08724	LC55x_1997	LC55x_1997; hypothetical protein
OG_08725	LC55x_2005	LC55x_2005; amino acid adenylation domain protein
OG_08726	LC55x_2009	LC55x_2009; hypothetical protein
OG_08727	LC55x_2022	LC55x_2022; hypothetical protein
OG_08728	LC55x_2047	LC55x_2047; hypothetical protein

OG_06741	EYR67960.1	hypothetical protein AZ78_13210 [Lysobacter capsici AZ78]
OG_06742	EYR67958.1	hypothetical protein AZ78_13200 [Lysobacter capsici AZ78]
OG_06743	EYR67957.1	hypothetical protein AZ78_13195 [Lysobacter capsici AZ78]
OG_06744	EYR67956.1	hypothetical protein AZ78_13190 [Lysobacter capsici AZ78]
OG_06745	EYR67955.1	hypothetical protein AZ78_13185 [Lysobacter capsici AZ78]
OG_06746	EYR67953.1	hypothetical protein AZ78_13175 [Lysobacter capsici AZ78]
OG_06747	EYR67952.1	5-aminolevulinate synthase [Lysobacter capsici AZ78]
OG_06748	EYR67951.1	taurine dioxygenase [Lysobacter capsici AZ78]
OG_06749	EYR67950.1	3-oxoacyl-ACP synthase [Lysobacter capsici AZ78]
OG_06750	EYR67949.1	hypothetical protein AZ78_13155 [Lysobacter capsici AZ78]
OG_06751	EYR67948.1	hypothetical protein AZ78_13150 [Lysobacter capsici AZ78]
OG_06752	EYR67947.1	MFS transporter permease [Lysobacter capsici AZ78]
OG_06753	EYR67946.1	NADP-dependent oxidoreductase [Lysobacter capsici AZ78]
OG_06754	EYR67945.1	hypothetical protein AZ78_13130 [Lysobacter capsici AZ78]
OG_06755	EYR67931.1	hypothetical protein AZ78_13060 [Lysobacter capsici AZ78]
OG_06756	EYR67891.1	hypothetical protein AZ78_12855 [Lysobacter capsici AZ78]
OG_06757	EYR67890.1	hypothetical protein AZ78_12850 [Lysobacter capsici AZ78]
OG_06758	EYR67884.1	hypothetical protein AZ78_13860 [Lysobacter capsici AZ78]
OG_06759	EYR67879.1	hypothetical protein AZ78_13750 [Lysobacter capsici AZ78]
OG_06760	EYR67873.1	hypothetical protein AZ78_13570 [Lysobacter capsici AZ78]
OG_06761	EYR67872.1	hypothetical protein AZ78_13430 [Lysobacter capsici AZ78]
OG_06762	EYR67842.1	hypothetical protein AZ78_13715 [Lysobacter capsici AZ78]
OG_06763	EYR67704.1	hypothetical protein AZ78_14905 [Lysobacter capsici AZ78]
OG_06764	EYR67700.1	hypothetical protein AZ78_14865 [Lysobacter capsici AZ78]
OG_06765	EYR67696.1	hypothetical protein AZ78_14780 [Lysobacter capsici AZ78]
OG_06766	EYR67695.1	hypothetical protein AZ78_14720 [Lysobacter capsici AZ78]
OG_06767	EYR67683.1	hypothetical protein AZ78_14370 [Lysobacter capsici AZ78]
OG_06768	EYR67556.1	hypothetical protein AZ78_15530 [Lysobacter capsici AZ78]
OG_06769	EYR67549.1	hypothetical protein AZ78_15495 [Lysobacter capsici AZ78]
OG_06770	EYR67547.1	hypothetical protein AZ78_15485 [Lysobacter capsici AZ78]
OG_06771	EYR67500.1	hypothetical protein AZ78_15250 [Lysobacter capsici AZ78]
OG_06772	EYR67482.1	hypothetical protein AZ78_15155 [Lysobacter capsici AZ78]
OG_06773	EYR67477.1	hypothetical protein AZ78_15130 [Lysobacter capsici AZ78]
OG_06774	EYR67473.1	hypothetical protein AZ78_15110 [Lysobacter capsici AZ78]
OG_06775	EYR67469.1	hypothetical protein AZ78_16125 [Lysobacter capsici AZ78]
OG_06776	EYR67461.1	hypothetical protein AZ78_15910 [Lysobacter capsici AZ78]
OG_06777	EYR67458.1	hypothetical protein AZ78_15885 [Lysobacter capsici AZ78]
OG_06778	EYR67457.1	hypothetical protein AZ78_15865 [Lysobacter capsici AZ78]
OG_06779	EYR67454.1	hypothetical protein AZ78_15770 [Lysobacter capsici AZ78]
OG_06780	EYR67452.1	hypothetical protein AZ78_15620 [Lysobacter capsici AZ78]
OG_06781	EYR67366.1	hypothetical protein AZ78_16615 [Lysobacter capsici AZ78]
OG_06782	EYR67365.1	hypothetical protein AZ78_16610 [Lysobacter capsici AZ78]
OG_06783	EYR67364.1	hypothetical protein AZ78_16605 [Lysobacter capsici AZ78]
OG_06784	EYR67363.1	hypothetical protein AZ78_16600 [Lysobacter capsici AZ78]
OG_06785	EYR67362.1	hypothetical protein AZ78_16590 [Lysobacter capsici AZ78]
OG_06786	EYR67361.1	hypothetical protein AZ78_16580 [Lysobacter capsici AZ78]
OG_06787	EYR67360.1	hypothetical protein AZ78_16575 [Lysobacter capsici AZ78]
OG_06788	EYR67359.1	hypothetical protein AZ78_16565 [Lysobacter capsici AZ78]
OG_06789	EYR67358.1	hypothetical protein AZ78_16560 [Lysobacter capsici AZ78]
OG_06790	EYR67357.1	hypothetical protein AZ78_16550 [Lysobacter capsici AZ78]
OG_06791	EYR67356.1	hypothetical protein AZ78_16545 [Lysobacter capsici AZ78]
OG_06792	EYR67355.1	hypothetical protein AZ78_16540 [Lysobacter capsici AZ78]
OG_06793	EYR67354.1	hypothetical protein AZ78_16535 [Lysobacter capsici AZ78]
OG_06794	EYR67353.1	hypothetical protein AZ78_16525 [Lysobacter capsici AZ78]
OG_06795	EYR67352.1	hypothetical protein AZ78_16520 [Lysobacter capsici AZ78]
OG_06796	EYR67351.1	hypothetical protein AZ78_16510 [Lysobacter capsici AZ78]
OG_06797	EYR67350.1	hypothetical protein AZ78_16505 [Lysobacter capsici AZ78]
OG_06798	EYR67349.1	hypothetical protein AZ78_16495 [Lysobacter capsici AZ78]
OG_06799	EYR67348.1	hypothetical protein AZ78_16490 [Lysobacter capsici AZ78]
OG_06800	EYR67347.1	hypothetical protein AZ78_16475 [Lysobacter capsici AZ78]
OG_06801	EYR67346.1	hypothetical protein AZ78_16470 [Lysobacter capsici AZ78]
OG_06802	EYR67345.1	hypothetical protein AZ78_16465 [Lysobacter capsici AZ78]
OG_06803	EYR67344.1	hypothetical protein AZ78_16460 [Lysobacter capsici AZ78]
OG_06804	EYR67343.1	hypothetical protein AZ78_16455 [Lysobacter capsici AZ78]
OG_06805	EYR67342.1	hypothetical protein AZ78_16450 [Lysobacter capsici AZ78]
OG_06806	EYR67341.1	hypothetical protein AZ78_16440 [Lysobacter capsici AZ78]
OG_06807	EYR67340.1	hypothetical protein AZ78_16435 [Lysobacter capsici AZ78]
OG_06808	EYR67339.1	hypothetical protein AZ78_16430 [Lysobacter capsici AZ78]
OG_06809	EYR67338.1	hypothetical protein AZ78_16420 [Lysobacter capsici AZ78]
OG_06810	EYR67337.1	hypothetical protein AZ78_16410 [Lysobacter capsici AZ78]
OG_06811	EYR67336.1	hypothetical protein AZ78_16400 [Lysobacter capsici AZ78]

OG_08729	LC55x_2064	LC55x_2064; hypothetical protein
OG_08730	LC55x_2074	LC55x_2074; hypothetical protein
OG_08731	LC55x_2086	LC55x_2086; hypothetical protein
OG_08732	LC55x_2090	LC55x_2090; hypothetical protein
OG_08733	LC55x_2103	LC55x_2103; hypothetical protein
OG_08734	LC55x_2113	LC55x_2113; hypothetical protein
OG_08735	LC55x_2126	LC55x_2126; hypothetical protein
OG_08736	LC55x_2130	LC55x_2130; hypothetical protein
OG_08737	LC55x_2135	LC55x_2135; hypothetical protein
OG_08738	LC55x_2153	LC55x_2153; hypothetical protein
OG_08739	LC55x_2188	LC55x_2188; hypothetical protein
OG_08740	LC55x_2197	LC55x_2197; hypothetical protein
OG_08741	LC55x_2198	LC55x_2198; hypothetical protein
OG_08742	LC55x_2199	LC55x_2199; hypothetical protein
OG_08743	LC55x_2200	LC55x_2200; hypothetical protein
OG_08744	LC55x_2204	LC55x_2204; hypothetical protein
OG_08745	LC55x_2208	LC55x_2208; NLPA lipofamily protein
OG_08746	LC55x_2213	LC55x_2213; hypothetical protein
OG_08747	LC55x_2216	LC55x_2216; hypothetical protein
OG_08748	LC55x_2218	LC55x_2218; hypothetical protein
OG_08749	LC55x_2219	LC55x_2219; hypothetical protein
OG_08750	LC55x_2244	LC55x_2244; hypothetical protein
OG_08751	LC55x_2255	LC55x_2255; hypothetical protein
OG_08752	LC55x_2272	LC55x_2272; RNA ligase
OG_08753	LC55x_2275	LC55x_2275; hypothetical protein
OG_08754	LC55x_2305	LC55x_2305; hypothetical protein
OG_08755	LC55x_2327	LC55x_2327; hypothetical protein
OG_08756	LC55x_2338	engA; GTP-binding engA domain protein
OG_08757	LC55x_2339	engA; ribosome-associated GTPase EngA
OG_08758	LC55x_2349	LC55x_2349; hypothetical protein
OG_08759	LC55x_2353	LC55x_2353; hypothetical protein
OG_08760	LC55x_2356	LC55x_2356; hypothetical protein
OG_08761	LC55x_2383	LC55x_2383; hypothetical protein
OG_08762	LC55x_2387	LC55x_2387; hypothetical protein
OG_08763	LC55x_2395	LC55x_2395; hypothetical protein
OG_08764	LC55x_2396	LC55x_2396; hypothetical protein
OG_08765	LC55x_2412	LC55x_2412; hypothetical protein
OG_08766	LC55x_2429	LC55x_2429; conserved hypothetical protein
OG_08767	LC55x_2444	LC55x_2444; hypothetical protein
OG_08768	LC55x_2454	LC55x_2454; hypothetical protein
OG_08769	LC55x_2469	LC55x_2469; hypothetical protein
OG_08770	LC55x_2490	LC55x_2490; hypothetical protein
OG_08771	LC55x_2491	LC55x_2491; hypothetical protein
OG_08772	LC55x_2496	LC55x_2496; hypothetical protein
OG_08773	LC55x_2529	LC55x_2529; hypothetical protein
OG_08774	LC55x_2542	LC55x_2542; hypothetical protein
OG_08775	LC55x_2550	LC55x_2550; hypothetical protein
OG_08776	LC55x_2552	LC55x_2552; hypothetical protein
OG_08777	LC55x_2553	LC55x_2553; hypothetical protein
OG_08778	LC55x_2577	LC55x_2577; hypothetical protein
OG_08779	LC55x_2585	LC55x_2585; alpha/beta hydrolase family protein
OG_08780	LC55x_2601	LC55x_2601; hypothetical protein
OG_08781	LC55x_2612	LC55x_2612; hypothetical protein
OG_08782	LC55x_2615	LC55x_2615; putative peptidoglycan binding domain protein
OG_08783	LC55x_2616	LC55x_2616; hypothetical protein
OG_08784	LC55x_2633	LC55x_2633; hypothetical protein
OG_08785	LC55x_2634	LC55x_2634; hypothetical protein
OG_08786	LC55x_2636	LC55x_2636; hypothetical protein
OG_08787	LC55x_2645	LC55x_2645; hypothetical protein
OG_08788	LC55x_2657	LC55x_2657; hypothetical protein
OG_08789	LC55x_2668	LC55x_2668; hypothetical protein
OG_08790	LC55x_2673	LC55x_2673; hypothetical protein
OG_08791	LC55x_2679	LC55x_2679; hypothetical protein
OG_08792	LC55x_2680	LC55x_2680; hypothetical protein
OG_08793	LC55x_2689	LC55x_2689; amino acid adenylation domain protein
OG_08794	LC55x_2691	dltA; D-alanine--poly(phosphoribitol) ligase, subunit 1
OG_08795	LC55x_2698	LC55x_2698; hypothetical protein
OG_08796	LC55x_2709	LC55x_2709; hypothetical protein
OG_08797	LC55x_2710	LC55x_2710; hypothetical protein
OG_08798	LC55x_2711	LC55x_2711; hypothetical protein
OG_08799	LC55x_2712	LC55x_2712; zonular occludens toxin family protein

OG_06812	EYR67335.1	hypothetical protein AZ78_16395 [Lysobacter capsici AZ78]
OG_06813	EYR67334.1	hypothetical protein AZ78_16385 [Lysobacter capsici AZ78]
OG_06814	EYR67333.1	hypothetical protein AZ78_16380 [Lysobacter capsici AZ78]
OG_06815	EYR67332.1	hypothetical protein AZ78_16375 [Lysobacter capsici AZ78]
OG_06816	EYR67331.1	hypothetical protein AZ78_16370 [Lysobacter capsici AZ78]
OG_06817	EYR67330.1	hypothetical protein AZ78_16365 [Lysobacter capsici AZ78]
OG_06818	EYR67329.1	hypothetical protein AZ78_16360 [Lysobacter capsici AZ78]
OG_06819	EYR67328.1	hypothetical protein AZ78_16355 [Lysobacter capsici AZ78]
OG_06820	EYR67327.1	hypothetical protein AZ78_16350 [Lysobacter capsici AZ78]
OG_06821	EYR67326.1	hypothetical protein AZ78_16345 [Lysobacter capsici AZ78]
OG_06822	EYR67325.1	hypothetical protein AZ78_16340 [Lysobacter capsici AZ78]
OG_06823	EYR67324.1	hypothetical protein AZ78_16335 [Lysobacter capsici AZ78]
OG_06824	EYR67323.1	hypothetical protein AZ78_16330 [Lysobacter capsici AZ78]
OG_06825	EYR67322.1	hypothetical protein AZ78_16325 [Lysobacter capsici AZ78]
OG_06826	EYR67318.1	hypothetical protein AZ78_16270 [Lysobacter capsici AZ78]
OG_06827	EYR67316.1	hypothetical protein AZ78_16220 [Lysobacter capsici AZ78]
OG_06828	EYR67303.1	single-stranded DNA-binding protein [Lysobacter capsici AZ78]
OG_06829	EYR67301.1	DNA primase [Lysobacter capsici AZ78]
OG_06830	EYR67300.1	hypothetical protein AZ78_16555 [Lysobacter capsici AZ78]
OG_06831	EYR67299.1	hypothetical protein AZ78_16530 [Lysobacter capsici AZ78]
OG_06832	EYR67297.1	head-tail joining protein [Lysobacter capsici AZ78]
OG_06833	EYR67296.1	hypothetical protein AZ78_16485 [Lysobacter capsici AZ78]
OG_06834	EYR67295.1	hypothetical protein AZ78_16445 [Lysobacter capsici AZ78]
OG_06835	EYR67294.1	holin [Lysobacter capsici AZ78]
OG_06836	EYR67293.1	hypothetical protein AZ78_16415 [Lysobacter capsici AZ78]
OG_06837	EYR67254.1	hypothetical protein AZ78_16920 [Lysobacter capsici AZ78]
OG_06838	EYR67252.1	hypothetical protein AZ78_16910 [Lysobacter capsici AZ78]
OG_06839	EYR67250.1	hypothetical protein AZ78_16900 [Lysobacter capsici AZ78]
OG_06840	EYR67237.1	hypothetical protein AZ78_16835 [Lysobacter capsici AZ78]
OG_06841	EYR67230.1	hypothetical protein AZ78_16800 [Lysobacter capsici AZ78]
OG_06842	EYR67225.1	hypothetical protein AZ78_16775 [Lysobacter capsici AZ78]
OG_06843	EYR67197.1	hypothetical protein AZ78_17390 [Lysobacter capsici AZ78]
OG_06844	EYR67132.1	hypothetical protein AZ78_17055 [Lysobacter capsici AZ78]
OG_06845	EYR67126.1	hypothetical protein AZ78_17850 [Lysobacter capsici AZ78]
OG_06846	EYR67105.1	hypothetical protein AZ78_17745 [Lysobacter capsici AZ78]
OG_06847	EYR67088.1	hypothetical protein AZ78_17660 [Lysobacter capsici AZ78]
OG_06848	EYR67067.1	hypothetical protein AZ78_17550 [Lysobacter capsici AZ78]
OG_06849	EYR67051.1	hypothetical protein AZ78_17470 [Lysobacter capsici AZ78]
OG_06850	EYR67037.1	hypothetical protein AZ78_18150 [Lysobacter capsici AZ78]
OG_06851	EYR67030.1	hypothetical protein AZ78_18115 [Lysobacter capsici AZ78]
OG_06852	EYR67029.1	hypothetical protein AZ78_18110 [Lysobacter capsici AZ78]
OG_06853	EYR67027.1	hypothetical protein AZ78_18100 [Lysobacter capsici AZ78]
OG_06854	EYR67026.1	hypothetical protein AZ78_18095 [Lysobacter capsici AZ78]
OG_06855	EYR67025.1	hypothetical protein AZ78_18090 [Lysobacter capsici AZ78]
OG_06856	EYR67024.1	hypothetical protein AZ78_18085 [Lysobacter capsici AZ78]
OG_06857	EYR67023.1	hypothetical protein AZ78_18080 [Lysobacter capsici AZ78]
OG_06858	EYR67022.1	hypothetical protein AZ78_18075 [Lysobacter capsici AZ78]
OG_06859	EYR67021.1	hypothetical protein AZ78_18070 [Lysobacter capsici AZ78]
OG_06860	EYR67020.1	hypothetical protein AZ78_18065 [Lysobacter capsici AZ78]
OG_06861	EYR67008.1	hypothetical protein AZ78_18005 [Lysobacter capsici AZ78]
OG_06862	EYR67006.1	hypothetical protein AZ78_17995 [Lysobacter capsici AZ78]
OG_06863	EYR67003.1	hypothetical protein AZ78_17980 [Lysobacter capsici AZ78]
OG_06864	EYR66975.1	hypothetical protein AZ78_18565 [Lysobacter capsici AZ78]
OG_06865	EYR66952.1	hypothetical protein AZ78_18440 [Lysobacter capsici AZ78]
OG_06866	EYR66949.1	hypothetical protein AZ78_18425 [Lysobacter capsici AZ78]
OG_06867	EYR66924.1	hypothetical protein AZ78_18300 [Lysobacter capsici AZ78]
OG_06868	EYR66922.1	hypothetical protein AZ78_18290 [Lysobacter capsici AZ78]
OG_06869	EYR66921.1	hypothetical protein AZ78_18285 [Lysobacter capsici AZ78]
OG_06870	EYR66919.1	hypothetical protein AZ78_18275 [Lysobacter capsici AZ78]
OG_06871	EYR66917.1	hypothetical protein AZ78_18265 [Lysobacter capsici AZ78]
OG_06872	EYR66916.1	hypothetical protein AZ78_18260 [Lysobacter capsici AZ78]
OG_06873	EYR66915.1	hypothetical protein AZ78_18250 [Lysobacter capsici AZ78]
OG_06874	EYR66904.1	membrane protein [Lysobacter capsici AZ78]
OG_06875	EYR66903.1	hypothetical protein AZ78_18190 [Lysobacter capsici AZ78]
OG_06876	EYR66873.1	acid virulence protein B [Lysobacter capsici AZ78]
OG_06877	EYR66839.1	hypothetical protein AZ78_18845 [Lysobacter capsici AZ78]
OG_06878	EYR66811.1	hypothetical protein AZ78_18700 [Lysobacter capsici AZ78]
OG_06879	EYR66803.1	hypothetical protein AZ78_18660 [Lysobacter capsici AZ78]
OG_06880	EYR66789.1	hypothetical protein AZ78_19410 [Lysobacter capsici AZ78]
OG_06881	EYR66782.1	hypothetical protein AZ78_19320 [Lysobacter capsici AZ78]
OG_06882	EYR66771.1	hypothetical protein AZ78_19395 [Lysobacter capsici AZ78]

OG_08800	LC55x_2713	LC55x_2713; conserved hypothetical protein
OG_08801	LC55x_2714	LC55x_2714; conserved hypothetical protein
OG_08802	LC55x_2715	LC55x_2715; hypothetical protein
OG_08803	LC55x_2716	LC55x_2716; hypothetical protein
OG_08804	LC55x_2717	LC55x_2717; conserved hypothetical protein
OG_08805	LC55x_2718	LC55x_2718; hypothetical protein
OG_08806	LC55x_2719	LC55x_2719; hypothetical protein
OG_08807	LC55x_2724	LC55x_2724; hypothetical protein
OG_08808	LC55x_2728	LC55x_2728; hypothetical protein
OG_08809	LC55x_2731	LC55x_2731; hypothetical protein
OG_08810	LC55x_2749	LC55x_2749; hypothetical protein
OG_08811	LC55x_2751	LC55x_2751; hypothetical protein
OG_08812	LC55x_2757	LC55x_2757; hypothetical protein
OG_08813	LC55x_2768	LC55x_2768; hypothetical protein
OG_08814	LC55x_2769	LC55x_2769; hypothetical protein
OG_08815	LC55x_2774	LC55x_2774; hypothetical protein
OG_08816	LC55x_2776	LC55x_2776; hypothetical protein
OG_08817	LC55x_2784	LC55x_2784; hypothetical protein
OG_08818	LC55x_2804	LC55x_2804; hypothetical protein
OG_08819	LC55x_2807	LC55x_2807; hypothetical protein
OG_08820	LC55x_2821	LC55x_2821; hypothetical protein
OG_08821	LC55x_2832	LC55x_2832; hypothetical protein
OG_08822	LC55x_2845	LC55x_2845; yiaA/B two helix domain protein
OG_08823	LC55x_2851	LC55x_2851; hypothetical protein
OG_08824	LC55x_2863	LC55x_2863; hypothetical protein
OG_08825	LC55x_2882	LC55x_2882; hypothetical protein
OG_08826	LC55x_2886	LC55x_2886; hypothetical protein
OG_08827	LC55x_2888	LC55x_2888; hypothetical protein
OG_08828	LC55x_2895	LC55x_2895; hypothetical protein
OG_08829	LC55x_2916	LC55x_2916; hypothetical protein
OG_08830	LC55x_2931	LC55x_2931; hypothetical protein
OG_08831	LC55x_2949	LC55x_2949; hypothetical protein
OG_08832	LC55x_2958	LC55x_2958; hypothetical protein
OG_08833	LC55x_2962	LC55x_2962; hypothetical protein
OG_08834	LC55x_2975	LC55x_2975; hypothetical protein
OG_08835	LC55x_2992	LC55x_2992; hypothetical protein
OG_08836	LC55x_3021	LC55x_3021; hypothetical protein
OG_08837	LC55x_3030	LC55x_3030; hypothetical protein
OG_08838	LC55x_3034	LC55x_3034; hypothetical protein
OG_08839	LC55x_3042	LC55x_3042; hypothetical protein
OG_08840	LC55x_3072	LC55x_3072; hypothetical protein
OG_08841	LC55x_3078	LC55x_3078; hypothetical protein
OG_08842	LC55x_3087	LC55x_3087; hypothetical protein
OG_08843	LC55x_3091	LC55x_3091; hypothetical protein
OG_08844	LC55x_3093	LC55x_3093; hypothetical protein
OG_08845	LC55x_3108	LC55x_3108; hypothetical protein
OG_08846	LC55x_3115	LC55x_3115; hypothetical protein
OG_08847	LC55x_3119	LC55x_3119; hypothetical protein
OG_08848	LC55x_3141	LC55x_3141; hypothetical protein
OG_08849	LC55x_3144	LC55x_3144; hypothetical protein
OG_08850	LC55x_3143	LC55x_3143; hypothetical protein
OG_08851	LC55x_3146	LC55x_3146; hypothetical protein
OG_08852	LC55x_3149	LC55x_3149; hypothetical protein
OG_08853	LC55x_3155	LC55x_3155; hypothetical protein
OG_08854	LC55x_3164	LC55x_3164; uvrD/REP helicase N-terminal domain protein
OG_08855	LC55x_3165	LC55x_3165; hypothetical protein
OG_08856	LC55x_3171	LC55x_3171; hypothetical protein
OG_08857	LC55x_3176	LC55x_3176; hypothetical protein
OG_08858	LC55x_3180	LC55x_3180; hypothetical protein
OG_08859	LC55x_3185	LC55x_3185; conserved hypothetical protein
OG_08860	LC55x_3188	LC55x_3188; hypothetical protein
OG_08861	LC55x_3193	LC55x_3193; hypothetical protein
OG_08862	LC55x_3195	LC55x_3195; hypothetical protein
OG_08863	LC55x_3196	LC55x_3196; hypothetical protein
OG_08864	LC55x_3216	LC55x_3216; hypothetical protein
OG_08865	LC55x_3229	LC55x_3229; putative integron gene cassette protein
OG_08866	LC55x_3230	LC55x_3230; hypothetical protein
OG_08867	LC55x_3232	LC55x_3232; divergent AAA domain protein
OG_08868	LC55x_3233	LC55x_3233; hypothetical protein
OG_08869	LC55x_3235	LC55x_3235; hypothetical protein
OG_08870	LC55x_3236	LC55x_3236; hypothetical protein

OG_06883	EYR66752.1	polyketide synthase [Lysobacter capsici AZ78]
OG_06884	EYR66747.1	hypothetical protein AZ78_19230 [Lysobacter capsici AZ78]
OG_06885	EYR66736.1	hypothetical protein AZ78_19860 [Lysobacter capsici AZ78]
OG_06886	EYR66669.1	coenzyme PQQ biosynthesis protein A [Lysobacter capsici AZ78]
OG_06887	EYR66656.1	hypothetical protein AZ78_20225 [Lysobacter capsici AZ78]
OG_06888	EYR66651.1	hypothetical protein AZ78_20055 [Lysobacter capsici AZ78]
OG_06889	EYR66650.1	hypothetical protein AZ78_20050 [Lysobacter capsici AZ78]
OG_06890	EYR66649.1	hypothetical protein AZ78_20045 [Lysobacter capsici AZ78]
OG_06891	EYR66648.1	hypothetical protein AZ78_20040 [Lysobacter capsici AZ78]
OG_06892	EYR66647.1	hypothetical protein AZ78_20035 [Lysobacter capsici AZ78]
OG_06893	EYR66646.1	hypothetical protein AZ78_20030 [Lysobacter capsici AZ78]
OG_06894	EYR66585.1	hypothetical protein AZ78_20710 [Lysobacter capsici AZ78]
OG_06895	EYR66574.1	hypothetical protein AZ78_20555 [Lysobacter capsici AZ78]
OG_06896	EYR66486.1	hypothetical protein AZ78_20995 [Lysobacter capsici AZ78]
OG_06897	EYR66485.1	hypothetical protein AZ78_20940 [Lysobacter capsici AZ78]
OG_06898	EYR66426.1	hypothetical protein AZ78_21440, partial [Lysobacter capsici AZ78]
OG_06899	EYR66408.1	hypothetical protein AZ78_21345 [Lysobacter capsici AZ78]
OG_06900	EYR66393.1	hypothetical protein AZ78_21270 [Lysobacter capsici AZ78]
OG_06901	EYR66392.1	hypothetical protein AZ78_21260 [Lysobacter capsici AZ78]
OG_06902	EYR66356.1	hypothetical protein AZ78_21635 [Lysobacter capsici AZ78]
OG_06903	EYR66344.1	hypothetical protein AZ78_21575 [Lysobacter capsici AZ78]
OG_06904	EYR66313.1	hypothetical protein AZ78_22345 [Lysobacter capsici AZ78]
OG_06905	EYR66307.1	hypothetical protein AZ78_22315 [Lysobacter capsici AZ78]
OG_06906	EYR66306.1	hypothetical protein AZ78_22310 [Lysobacter capsici AZ78]
OG_06907	EYR66240.1	hypothetical protein AZ78_21975 [Lysobacter capsici AZ78]
OG_06908	EYR66201.1	hypothetical protein AZ78_21770 [Lysobacter capsici AZ78]
OG_06909	EYR66197.1	hypothetical protein AZ78_21750 [Lysobacter capsici AZ78]
OG_06910	EYR66170.1	hypothetical protein AZ78_22640 [Lysobacter capsici AZ78]
OG_06911	EYR66154.1	hypothetical protein AZ78_22560 [Lysobacter capsici AZ78]
OG_06912	EYR66153.1	hypothetical protein AZ78_22555 [Lysobacter capsici AZ78]
OG_06913	EYR66144.1	hypothetical protein AZ78_22510 [Lysobacter capsici AZ78]
OG_06914	EYR66113.1	hypothetical protein AZ78_22915 [Lysobacter capsici AZ78]
OG_06915	EYR66090.1	hypothetical protein AZ78_22800 [Lysobacter capsici AZ78]
OG_06916	EYR66059.1	hypothetical protein AZ78_23150 [Lysobacter capsici AZ78]
OG_06917	EYR66036.1	hypothetical protein AZ78_23035 [Lysobacter capsici AZ78]
OG_06918	EYR66032.1	hypothetical protein AZ78_23015 [Lysobacter capsici AZ78]
OG_06919	EYR65939.1	hypothetical protein AZ78_23815 [Lysobacter capsici AZ78]
OG_06920	EYR65927.1	hypothetical protein AZ78_23755 [Lysobacter capsici AZ78]
OG_06921	EYR65918.1	hypothetical protein AZ78_23705 [Lysobacter capsici AZ78]
OG_06922	EYR65893.1	hypothetical protein AZ78_23580 [Lysobacter capsici AZ78]
OG_06923	EYR65888.1	hypothetical protein AZ78_24130 [Lysobacter capsici AZ78]
OG_06924	EYR65866.1	hypothetical protein AZ78_24020 [Lysobacter capsici AZ78]
OG_06925	EYR65849.1	hypothetical protein AZ78_23930 [Lysobacter capsici AZ78]
OG_06926	EYR65836.1	hypothetical protein AZ78_24305 [Lysobacter capsici AZ78]
OG_06927	EYR65835.1	hypothetical protein AZ78_24300 [Lysobacter capsici AZ78]
OG_06928	EYR65834.1	hypothetical protein AZ78_24295 [Lysobacter capsici AZ78]
OG_06929	EYR65828.1	hypothetical protein AZ78_24265 [Lysobacter capsici AZ78]
OG_06930	EYR65815.1	hypothetical protein AZ78_24195 [Lysobacter capsici AZ78]
OG_06931	EYR65807.1	hypothetical protein AZ78_24155 [Lysobacter capsici AZ78]
OG_06932	EYR65790.1	hypothetical protein AZ78_24535 [Lysobacter capsici AZ78]
OG_06933	EYR65788.1	hypothetical protein AZ78_24525 [Lysobacter capsici AZ78]
OG_06934	EYR65772.1	hypothetical protein AZ78_24430 [Lysobacter capsici AZ78]
OG_06935	EYR65771.1	hypothetical protein AZ78_24425 [Lysobacter capsici AZ78]
OG_06936	EYR65770.1	hypothetical protein AZ78_24420 [Lysobacter capsici AZ78]
OG_06937	EYR65769.1	hypothetical protein AZ78_24415 [Lysobacter capsici AZ78]
OG_06938	EYR65768.1	hypothetical protein AZ78_24410 [Lysobacter capsici AZ78]
OG_06939	EYR65767.1	hypothetical protein AZ78_24405 [Lysobacter capsici AZ78]
OG_06940	EYR65766.1	hypothetical protein AZ78_24400 [Lysobacter capsici AZ78]
OG_06941	EYR65765.1	hypothetical protein AZ78_24395 [Lysobacter capsici AZ78]
OG_06942	EYR65735.1	hypothetical protein AZ78_24650 [Lysobacter capsici AZ78]
OG_06943	EYR65698.1	hypothetical protein AZ78_24925 [Lysobacter capsici AZ78]
OG_06944	EYR65679.1	hypothetical protein AZ78_25090 [Lysobacter capsici AZ78]
OG_06945	EYR65617.1	hypothetical protein AZ78_25265 [Lysobacter capsici AZ78]
OG_06946	EYR65608.1	hypothetical protein AZ78_25365 [Lysobacter capsici AZ78]
OG_06947	EYR65560.1	hypothetical protein AZ78_25590 [Lysobacter capsici AZ78]
OG_06948	EYR65559.1	hypothetical protein AZ78_25580 [Lysobacter capsici AZ78]
OG_06949	EYR65558.1	hypothetical protein AZ78_25575 [Lysobacter capsici AZ78]
OG_06950	EYR65546.1	hypothetical protein AZ78_25700 [Lysobacter capsici AZ78]
OG_06951	EYR65509.1	hypothetical protein AZ78_25985 [Lysobacter capsici AZ78]
OG_06952	EYR65480.1	elongation factor Tu, partial [Lysobacter capsici AZ78]
OG_06953	EYR65474.1	hypothetical protein AZ78_26130 [Lysobacter capsici AZ78]

OG_08871	LC55x_3238	LC55x_3238; hypothetical protein
OG_08872	LC55x_3239	LC55x_3239; conserved hypothetical protein
OG_08873	LC55x_3247	LC55x_3247; hypothetical protein
OG_08874	LC55x_3248	LC55x_3248; hypothetical protein
OG_08875	LC55x_3252	LC55x_3252; hypothetical protein
OG_08876	LC55x_3292	LC55x_3292; hypothetical protein
OG_08877	LC55x_3296	LC55x_3296; hypothetical protein
OG_08878	LC55x_3327	LC55x_3327; hypothetical protein
OG_08879	LC55x_3363	LC55x_3363; hypothetical protein
OG_08880	LC55x_3365	LC55x_3365; hypothetical protein
OG_08881	LC55x_3377	LC55x_3377; hypothetical protein
OG_08882	LC55x_3380	LC55x_3380; hypothetical protein
OG_08883	LC55x_3384	LC55x_3384; hypothetical protein
OG_08884	LC55x_3391	LC55x_3391; hypothetical protein
OG_08885	LC55x_3408	LC55x_3408; hypothetical protein
OG_08886	LC55x_3407	LC55x_3407; hypothetical protein
OG_08887	LC55x_3411	LC55x_3411; conserved hypothetical protein
OG_08888	LC55x_3462	LC55x_3462; hypothetical protein
OG_08889	LC55x_3470	LC55x_3470; hypothetical protein
OG_08890	LC55x_3472	LC55x_3472; phage integrase family protein
OG_08891	LC55x_3473	LC55x_3473; hypothetical protein
OG_08892	LC55x_3475	LC55x_3475; hypothetical protein
OG_08893	LC55x_3476	LC55x_3476; hypothetical protein
OG_08894	LC55x_3502	LC55x_3502; hypothetical protein
OG_08895	LC55x_3512	LC55x_3512; hypothetical protein
OG_08896	LC55x_3513	LC55x_3513; hypothetical protein
OG_08897	LC55x_3551	LC55x_3551; hypothetical protein
OG_08898	LC55x_3554	LC55x_3554; hypothetical protein
OG_08899	LC55x_3558	LC55x_3558; hypothetical protein
OG_08900	LC55x_3572	LC55x_3572; hypothetical protein
OG_08901	LC55x_3581	LC55x_3581; hypothetical protein
OG_08902	LC55x_3587	LC55x_3587; hypothetical protein
OG_08903	LC55x_3590	LC55x_3590; hypothetical protein
OG_08904	LC55x_3592	LC55x_3592; hypothetical protein
OG_08905	LC55x_3598	LC55x_3598; hypothetical protein
OG_08906	LC55x_3601	LC55x_3601; hypothetical protein
OG_08907	LC55x_3608	LC55x_3608; hypothetical protein
OG_08908	LC55x_3610	LC55x_3610; hypothetical protein
OG_08909	LC55x_3613	LC55x_3613; hypothetical protein
OG_08910	LC55x_3620	LC55x_3620; hypothetical protein
OG_08911	LC55x_3622	LC55x_3622; hypothetical protein
OG_08912	LC55x_3624	LC55x_3624; hypothetical protein
OG_08913	LC55x_3630	LC55x_3630; hypothetical protein
OG_08914	LC55x_3652	LC55x_3652; hypothetical protein
OG_08915	LC55x_3656	LC55x_3656; hypothetical protein
OG_08916	LC55x_3665	LC55x_3665; hypothetical protein
OG_08917	LC55x_3672	LC55x_3672; hypothetical protein
OG_08918	LC55x_3681	LC55x_3681; hypothetical protein
OG_08919	LC55x_3683	LC55x_3683; hypothetical protein
OG_08920	LC55x_3701	LC55x_3701; hypothetical protein
OG_08921	LC55x_3706	LC55x_3706; hypothetical protein
OG_08922	LC55x_3708	LC55x_3708; hypothetical protein
OG_08923	LC55x_3709	LC55x_3709; cellulase family protein
OG_08924	LC55x_3717	LC55x_3717; hypothetical protein
OG_08925	LC55x_3719	LC55x_3719; thermolysin metallopeptidase, alpha-helical domain protein
OG_08926	LC55x_3722	LC55x_3722; hypothetical protein
OG_08927	LC55x_3743	LC55x_3743; hypothetical protein
OG_08928	LC55x_3755	LC55x_3755; hypothetical protein
OG_08929	LC55x_3757	LC55x_3757; hypothetical protein
OG_08930	LC55x_3760	LC55x_3760; hypothetical protein
OG_08931	LC55x_3781	LC55x_3781; hypothetical protein
OG_08932	LC55x_3798	LC55x_3798; hypothetical protein
OG_08933	LC55x_3806	LC55x_3806; hypothetical protein
OG_08934	LC55x_3810	LC55x_3810; hypothetical protein
OG_08935	LC55x_3824	LC55x_3824; hypothetical protein
OG_08936	LC55x_3854	LC55x_3854; hypothetical protein
OG_08937	LC55x_3863	LC55x_3863; hypothetical protein
OG_08938	LC55x_3869	LC55x_3869; hypothetical protein
OG_08939	LC55x_3873	LC55x_3873; hypothetical protein
OG_08940	LC55x_3882	LC55x_3882; hypothetical protein
OG_08941	LC55x_3883	LC55x_3883; hypothetical protein

OG_06954	EYR65463.1	hypothetical protein AZ78_26200 [Lysobacter capsici AZ78]
OG_06955	EYR65462.1	hypothetical protein AZ78_26195 [Lysobacter capsici AZ78]
OG_06956	EYR65458.1	hypothetical protein AZ78_26165, partial [Lysobacter capsici AZ78]
OG_06957	EYR65446.1	hypothetical protein AZ78_26230 [Lysobacter capsici AZ78]
OG_06958	EYR65423.1	hypothetical protein AZ78_26400 [Lysobacter capsici AZ78]
OG_06959	EYR65374.1	potassium-transporting system small peptide KdpF [Lysobacter capsici AZ7
OG_06960	EYR65362.1	hypothetical protein AZ78_26805 [Lysobacter capsici AZ78]
OG_06961	EYR65361.1	hypothetical protein AZ78_26800 [Lysobacter capsici AZ78]
OG_06962	EYR65356.1	MFS transporter permease [Lysobacter capsici AZ78]
OG_06963	EYR65355.1	hypothetical protein AZ78_26855 [Lysobacter capsici AZ78]
OG_06964	EYR65354.1	hypothetical protein AZ78_26845 [Lysobacter capsici AZ78]
OG_06965	EYR65349.1	hypothetical protein AZ78_26880 [Lysobacter capsici AZ78]
OG_06966	EYR65343.1	hypothetical protein AZ78_26940 [Lysobacter capsici AZ78]
OG_06967	EYR65342.1	hypothetical protein AZ78_26950 [Lysobacter capsici AZ78]
OG_06968	EYR65341.1	hypothetical protein AZ78_26945 [Lysobacter capsici AZ78]
OG_06969	EYR65339.1	hypothetical protein AZ78_26960 [Lysobacter capsici AZ78]
OG_06970	EYR65338.1	hypothetical protein AZ78_26965 [Lysobacter capsici AZ78]
OG_06971	EYR65337.1	hypothetical protein AZ78_26985 [Lysobacter capsici AZ78]
OG_06972	EYR65333.1	hypothetical protein AZ78_27010 [Lysobacter capsici AZ78]
OG_06973	EYR65332.1	hypothetical protein AZ78_27015 [Lysobacter capsici AZ78]
OG_06974	EYR65331.1	hypothetical protein AZ78_27025 [Lysobacter capsici AZ78]
OG_06975	EYR65329.1	hypothetical protein AZ78_27050 [Lysobacter capsici AZ78]
OG_06976	EYR65328.1	hypothetical protein AZ78_27045 [Lysobacter capsici AZ78]
OG_06977	EYR65327.1	hypothetical protein AZ78_27060 [Lysobacter capsici AZ78]
OG_06978	EYR65326.1	hypothetical protein AZ78_27055 [Lysobacter capsici AZ78]
OG_06979	EYR65325.1	membrane protein, partial [Lysobacter capsici AZ78]
OG_06980	EYR65324.1	hypothetical protein AZ78_27105 [Lysobacter capsici AZ78]
OG_06981	EYR65323.1	hypothetical protein AZ78_27110 [Lysobacter capsici AZ78]
OG_06982	EYR65322.1	hypothetical protein AZ78_27135, partial [Lysobacter capsici AZ78]

OG_08942	LC55x_3889	LC55x_3889; hypothetical protein
OG_08943	LC55x_3899	LC55x_3899; hypothetical protein
OG_08944	LC55x_3903	LC55x_3903; hypothetical protein
OG_08945	LC55x_3909	LC55x_3909; hypothetical protein
OG_08946	LC55x_3917	pqqA; coenzyme PQQ peptide PqqA
OG_08947	LC55x_3923	LC55x_3923; hypothetical protein
OG_08948	LC55x_3981	LC55x_3981; hypothetical protein
OG_08949	LC55x_3998	LC55x_3998; conserved hypothetical protein
OG_08950	LC55x_4004	LC55x_4004; hypothetical protein
OG_08951	LC55x_4011	LC55x_4011; hypothetical protein
OG_08952	LC55x_4013	LC55x_4013; hypothetical protein
OG_08953	LC55x_4014	LC55x_4014; hypothetical protein
OG_08954	LC55x_4016	LC55x_4016; hypothetical protein
OG_08955	LC55x_4022	LC55x_4022; hypothetical protein
OG_08956	LC55x_4024	LC55x_4024; hypothetical protein
OG_08957	LC55x_4026	LC55x_4026; hypothetical protein
OG_08958	LC55x_4027	LC55x_4027; hypothetical protein
OG_08959	LC55x_4029	LC55x_4029; hypothetical protein
OG_08960	LC55x_4055	LC55x_4055; polysaccharide pyruvyl transferase family protein
OG_08961	LC55x_4056	LC55x_4056; hypothetical protein
OG_08962	LC55x_4057	LC55x_4057; glycosyl transferase 2 family protein
OG_08963	LC55x_4058	LC55x_4058; glycosyltransferase WbsX family protein
OG_08964	LC55x_4064	LC55x_4064; hypothetical protein
OG_08965	LC55x_4065	LC55x_4065; hypothetical protein
OG_08966	LC55x_4072	LC55x_4072; hypothetical protein
OG_08967	LC55x_4077	LC55x_4077; hypothetical protein
OG_08968	LC55x_4090	LC55x_4090; hypothetical protein
OG_08969	LC55x_4112	LC55x_4112; hypothetical protein
OG_08970	LC55x_4116	LC55x_4116; hypothetical protein
OG_08971	LC55x_4137	LC55x_4137; hypothetical protein
OG_08972	LC55x_4167	LC55x_4167; hypothetical protein
OG_08973	LC55x_4171	LC55x_4171; hypothetical protein
OG_08974	LC55x_4182	LC55x_4182; hypothetical protein
OG_08975	LC55x_4213	LC55x_4213; outer membrane lipoSlp family protein
OG_08976	LC55x_4215	LC55x_4215; hypothetical protein
OG_08977	LC55x_4218	LC55x_4218; hypothetical protein
OG_08978	LC55x_4234	LC55x_4234; hypothetical protein
OG_08979	LC55x_4237	LC55x_4237; hypothetical protein
OG_08980	LC55x_4244	LC55x_4244; hypothetical protein
OG_08981	LC55x_4280	LC55x_4280; hypothetical protein
OG_08982	LC55x_4282	LC55x_4282; hypothetical protein
OG_08983	LC55x_4304	LC55x_4304; hypothetical protein
OG_08984	LC55x_4324	LC55x_4324; hypothetical protein
OG_08985	LC55x_4335	LC55x_4335; hypothetical protein
OG_08986	LC55x_4366	LC55x_4366; putative electron transfer flavoprotein beta-subunit
OG_08987	LC55x_4367	LC55x_4367; electron transfer flavodomain protein
OG_08988	LC55x_4368	LC55x_4368; pectate lyase superfamily protein
OG_08989	LC55x_4371	LC55x_4371; NAD dependent epimerase/dehydratase family protein
OG_08990	LC55x_4372	LC55x_4372; glycosyl transferase 2 family protein
OG_08991	LC55x_4373	LC55x_4373; gtrA-like family protein
OG_08992	LC55x_4375	LC55x_4375; hypothetical protein
OG_08993	LC55x_4376	LC55x_4376; hypothetical protein
OG_08994	LC55x_4378	LC55x_4378; glycosyl transferases group 1 family protein
OG_08995	LC55x_4414	LC55x_4414; hypothetical protein
OG_08996	LC55x_4418	LC55x_4418; hypothetical protein
OG_08997	LC55x_4458	LC55x_4458; hypothetical protein
OG_08998	LC55x_4478	LC55x_4478; hypothetical protein
OG_08999	LC55x_4484	LC55x_4484; hypothetical protein
OG_09000	LC55x_4488	LC55x_4488; hypothetical protein
OG_09001	LC55x_4498	LC55x_4498; hypothetical protein
OG_09002	LC55x_4499	LC55x_4499; hypothetical protein
OG_09003	LC55x_4506	LC55x_4506; hypothetical protein
OG_09004	LC55x_4509	LC55x_4509; hypothetical protein
OG_09005	LC55x_4542	LC55x_4542; hypothetical protein
OG_09006	LC55x_4561	LC55x_4561; hypothetical protein
OG_09007	LC55x_4575	LC55x_4575; hypothetical protein
OG_09008	LC55x_4587	LC55x_4587; hypothetical protein
OG_09009	LC55x_4596	LC55x_4596; peptidase M23 family protein
OG_09010	LC55x_4601	LC55x_4601; hypothetical protein
OG_09011	LC55x_4613	LC55x_4613; hypothetical protein
OG_09012	LC55x_4622	LC55x_4622; hypothetical protein

OG_09013	LC55x_4625	LC55x_4625; hypothetical protein
OG_09014	LC55x_4628	LC55x_4628; hypothetical protein
OG_09015	LC55x_4635	LC55x_4635; hypothetical protein
OG_09016	LC55x_4650	LC55x_4650; hypothetical protein
OG_09017	LC55x_4662	LC55x_4662; hypothetical protein
OG_09018	LC55x_4663	LC55x_4663; diguanylate cyclase domain protein
OG_09019	LC55x_4692	LC55x_4692; hypothetical protein
OG_09020	LC55x_4718	LC55x_4718; hypothetical protein
OG_09021	LC55x_4731	LC55x_4731; hypothetical protein
OG_09022	LC55x_4739	LC55x_4739; hypothetical protein
OG_09023	LC55x_4743	LC55x_4743; hypothetical protein
OG_09024	LC55x_4756	LC55x_4756; hypothetical protein
OG_09025	LC55x_4770	LC55x_4770; hypothetical protein
OG_09026	LC55x_4781	LC55x_4781; hypothetical protein
OG_09027	LC55x_4784	LC55x_4784; hypothetical protein
OG_09028	LC55x_4787	LC55x_4787; hypothetical protein
OG_09029	LC55x_4789	LC55x_4789; hypothetical protein
OG_09030	LC55x_4806	LC55x_4806; hypothetical protein
OG_09031	LC55x_4820	LC55x_4820; hypothetical protein
OG_09032	LC55x_4823	LC55x_4823; hypothetical protein
OG_09033	LC55x_4824	LC55x_4824; hypothetical protein
OG_09034	LC55x_4856	LC55x_4856; hypothetical protein
OG_09035	LC55x_4860	LC55x_4860; hypothetical protein
OG_09036	LC55x_4867	LC55x_4867; hypothetical protein
OG_09037	LC55x_4869	LC55x_4869; hypothetical protein
OG_09038	LC55x_4872	LC55x_4872; hypothetical protein
OG_09039	LC55x_4889	LC55x_4889; hypothetical protein
OG_09040	LC55x_4894	LC55x_4894; hypothetical protein
OG_09041	LC55x_4926	LC55x_4926; hypothetical protein
OG_09042	LC55x_4930	LC55x_4930; hypothetical protein
OG_09043	LC55x_4950	LC55x_4950; hypothetical protein
OG_09044	LC55x_4956	LC55x_4956; conserved hypothetical protein
OG_09045	LC55x_4957	LC55x_4957; conserved hypothetical protein
OG_09046	LC55x_4958	LC55x_4958; AAA domain protein
OG_09047	LC55x_4959	LC55x_4959; SEC-C motif family protein
OG_09048	LC55x_4961	LC55x_4961; hypothetical protein
OG_09049	LC55x_4969	LC55x_4969; hypothetical protein
OG_09050	LC55x_4975	LC55x_4975; amino acid adenylation domain protein
OG_09051	LC55x_4977	lgrD; linear gramicidin synthase subunit D
OG_09052	LC55x_4979	LC55x_4979; hypothetical protein
OG_09053	LC55x_4992	LC55x_4992; conserved hypothetical protein
OG_09054	LC55x_4999	LC55x_4999; hypothetical protein
OG_09055	LC55x_5004	dhbF; dimodular nonribosomal peptide synthase
OG_09056	LC55x_5006	LC55x_5006; hypothetical protein
OG_09057	LC55x_5025	LC55x_5025; hypothetical protein
OG_09058	LC55x_5033	LC55x_5033; hypothetical protein
OG_09059	LC55x_5041	LC55x_5041; hypothetical protein
OG_09060	LC55x_5050	LC55x_5050; D-alanyl-D-alanine carboxypeptidase family protein
OG_09061	LC55x_5061	LC55x_5061; hypothetical protein
OG_09062	LC55x_5064	LC55x_5064; hypothetical protein
OG_09063	LC55x_5072	LC55x_5072; hypothetical protein
OG_09064	LC55x_5076	LC55x_5076; conserved hypothetical protein
OG_09065	LC55x_5079	LC55x_5079; resolvase, N terminal domain protein
OG_09066	LC55x_5080	LC55x_5080; putative transposase
OG_09067	LC55x_5097	LC55x_5097; hypothetical protein
OG_09068	LC55x_5109	LC55x_5109; hypothetical protein
OG_09069	LC55x_5129	LC55x_5129; hypothetical protein
OG_09070	LC55x_5148	LC55x_5148; hypothetical protein
OG_09071	LC55x_5149	LC55x_5149; hypothetical protein
OG_09072	LC55x_5171	LC55x_5171; hypothetical protein
OG_09073	LC55x_5177	LC55x_5177; hypothetical protein
OG_09074	LC55x_5180	LC55x_5180; hypothetical protein
OG_09075	LC55x_5200	LC55x_5200; hypothetical protein
OG_09076	LC55x_5202	LC55x_5202; hypothetical protein
OG_09077	LC55x_5210	LC55x_5210; tRNA_anti-like family protein
OG_09078	LC55x_5216	LC55x_5216; hypothetical protein
OG_09079	LC55x_5234	LC55x_5234; hypothetical protein
OG_09080	LC55x_5246	comF; competence F domain protein
OG_09081	LC55x_5249	LC55x_5249; hypothetical protein
OG_09082	LC55x_5250	LC55x_5250; hypothetical protein
OG_09083	LC55x_5258	LC55x_5258; conserved hypothetical protein

OG_09084	LC55x_5259	LC55x_5259; conserved hypothetical protein
OG_09085	LC55x_5260	LC55x_5260; Reverse
OG_09086	LC55x_5278	LC55x_5278; hypothetical protein
OG_09087	LC55x_5279	LC55x_5279; hypothetical protein
OG_09088	LC55x_5326	LC55x_5326; hypothetical protein
OG_09089	LC55x_5327	LC55x_5327; outer membrane autotransporter barrel domain protein
OG_09090	LC55x_5329	LC55x_5329; bacterial regulatory, luxR family protein
OG_09091	LC55x_5330	LC55x_5330; hypothetical protein
OG_09092	LC55x_5336	LC55x_5336; histidine kinase-, DNA gyrase B-, and HSP90-like ATPase family
OG_09093	LC55x_5343	LC55x_5343; AAA domain protein
OG_09094	LC55x_5347	LC55x_5347; histidine kinase-, DNA gyrase B-, and HSP90-like ATPase family
OG_09095	LC55x_5348	LC55x_5348; hypothetical protein
OG_09096	LC55x_5364	LC55x_5364; pentapeptide repeats family protein
OG_09097	LC55x_5365	LC55x_5365; his Kinase A domain protein
OG_09098	LC55x_5366	LC55x_5366; qseB transcriptional activator
OG_09099	LC55x_5367	LC55x_5367; TPR repeat family protein
OG_09100	LC55x_5368	LC55x_5368; polysaccharide biosynthesis family protein
OG_09101	LC55x_5369	LC55x_5369; TENA/THI-4/PQQC family protein
OG_09102	LC55x_5370	LC55x_5370; AMP-binding enzyme family protein
OG_09103	LC55x_5371	LC55x_5371; thermostable hemolysin family protein
OG_09104	LC55x_5372	LC55x_5372; hemerythrin HHE cation binding domain protein
OG_09105	LC55x_5374	LC55x_5374; BON domain protein
OG_09106	LC55x_5385	LC55x_5385; hypothetical protein
OG_09107	LC55x_5386	LC55x_5386; hypothetical protein
OG_09108	LC55x_5388	LC55x_5388; major intrinsic domain protein
OG_09109	LC55x_5390	LC55x_5390; hypothetical protein
OG_09110	LC55x_5392	LC55x_5392; fecR family protein
OG_09111	LC55x_5393	LC55x_5393; tonB-dependent receptor family protein
OG_09112	LC55x_5394	LC55x_5394; conserved hypothetical protein
OG_09113	LC55x_5395	LC55x_5395; putative DNA metabolism protein
OG_09114	LC55x_5396	LC55x_5396; radical SAM superfamily protein
OG_09115	LC55x_5397	LC55x_5397; hypothetical protein
OG_09116	LC55x_5398	LC55x_5398; hypothetical protein
OG_09117	LC55x_5404	LC55x_5404; hypothetical protein
OG_09118	LC55x_5405	LC55x_5405; conserved hypothetical protein
OG_09119	LC55x_5421	LC55x_5421; hypothetical protein
OG_09120	LC55x_5423	LC55x_5423; hypothetical protein
OG_09121	LC55x_5435	LC55x_5435; hypothetical protein
OG_09122	LC55x_5436	LC55x_5436; hypothetical protein
OG_09123	LC55x_5444	LC55x_5444; short chain dehydrogenase family protein
OG_09124	LC55x_5453	LC55x_5453; hypothetical protein
OG_09125	LC55x_5456	LC55x_5456; hypothetical protein
OG_09126	LC55x_5462	LC55x_5462; hypothetical protein
OG_09127	LC55x_5477	LC55x_5477; barstar family protein
OG_09128	LC55x_5479	LC55x_5479; hypothetical protein
OG_09129	LC55x_5498	LC55x_5498; hypothetical protein
OG_09130	LC55x_5500	LC55x_5500; hypothetical protein
OG_09131	LC55x_5504	LC55x_5504; hypothetical protein
OG_09132	LC55x_5511	LC55x_5511; conserved hypothetical protein
OG_09133	LC55x_5515	LC55x_5515; hypothetical protein
OG_09134	LC55x_5527	LC55x_5527; hypothetical protein
OG_09135	LC55x_5564	LC55x_5564; hypothetical protein
OG_09136	LC55x_5572	LC55x_5572; hypothetical protein
OG_09137	LC55x_5587	LC55x_5587; hypothetical protein
OG_09138	LC55x_5590	LC55x_5590; hypothetical protein
OG_09139	LC55x_5595	LC55x_5595; hypothetical protein
OG_09140	LC55x_5601	LC55x_5601; hypothetical protein
OG_09141	LC55x_5600	LC55x_5600; hypothetical protein
OG_09142	LC55x_5608	LC55x_5608; hypothetical protein
OG_09143	LC55x_5628	LC55x_5628; hypothetical protein
OG_09144	LC55x_5655	LC55x_5655; hypothetical protein
OG_09145	LC55x_5659	LC55x_5659; hypothetical protein
OG_09146	LC55x_5671	LC55x_5671; hypothetical protein
OG_09147	LC55x_5675	LC55x_5675; hypothetical protein
OG_09148	LC55x_5680	LC55x_5680; hypothetical protein
OG_09149	LC55x_5682	LC55x_5682; hypothetical protein
OG_09150	LC55x_5692	LC55x_5692; hypothetical protein
OG_09151	LC55x_5720	LC55x_5720; hypothetical protein
OG_09152	LC55x_5727	LC55x_5727; hypothetical protein
OG_09153	LC55x_5735	LC55x_5735; hypothetical protein
OG_05559	LC55x_406	LC55x_406; hypothetical protein

OG_05560	LC55x_3782	LC55x_3782; hypothetical protein
OG_05561	LC55x_5681	LC55x_5681; hypothetical protein
OG_05562	LC55x_3106	LC55x_3106; hypothetical protein
OG_05563	LC55x_5354	LC55x_5354; hypothetical protein

Orthologous group	*L. gum* 3.2.11<br>GeneID (#914)<br>17.2% of genome	Gene description
OG_03487	LG3211_5086	LG3211_5086; NHL repeat family protein
OG_03487	LG3211_5088	LG3211_5088; NHL repeat family protein
OG_03487	LG3211_5087	LG3211_5087; SMP-30/Gluconolaconase/LRE-like region family protein
OG_03487	LG3211_4285	LG3211_4285; insecticide toxin TcdB middle/N-terminal region family prote
OG_03989	LG3211_4408	LG3211_4408; hypothetical protein
OG_03989	LG3211_1433	LG3211_1433; hypothetical protein
OG_03989	LG3211_552	LG3211_552; hypothetical protein
OG_03990	LG3211_2239	LG3211_2239; bacterial regulatory helix-turn-helix , lysR family protein
OG_03990	LG3211_2570	LG3211_2570; bacterial regulatory helix-turn-helix , lysR family protein
OG_03990	LG3211_2562	LG3211_2562; bacterial regulatory helix-turn-helix , lysR family protein
OG_03991	LG3211_2557	agaA; alpha-galactosidase A
OG_03991	LG3211_296	agaA; alpha-galactosidase A
OG_03991	LG3211_1843	LG3211_1843; BH1870 protein
OG_03992	LG3211_4841	sldA; glycerol dehydrogenase large subunit
OG_03992	LG3211_4673	LG3211_4673; membrane-bound PQQ-dependent dehydrogenase, glucose/
OG_03992	LG3211_4849	quiA; quinate/shikimate dehydrogenase [Pyrroloquinoline-quinone]
OG_03996	LG3211_4135	LG3211_4135; hypothetical protein
OG_03996	LG3211_3284	LG3211_3284; hypothetical protein
OG_03996	LG3211_2627	LG3211_2627; hypothetical protein
OG_04648	LG3211_1497	LG3211_1497; conserved hypothetical protein
OG_04648	LG3211_342	LG3211_342; putative lytic enzyme
OG_04649	LG3211_4807	xylS; alpha-xylosidase
OG_04649	LG3211_1835	LG3211_1835; glycosyl hydrolases 31 family protein
OG_04650	LG3211_1201	LG3211_1201; conserved hypothetical protein
OG_04650	LG3211_1199	LG3211_1199; hypothetical protein
OG_04651	LG3211_3421	LG3211_3421; hypothetical protein
OG_04651	LG3211_3419	LG3211_3419; hypothetical protein
OG_04652	LG3211_2981	LG3211_2981; phage portal family protein
OG_04652	LG3211_3977	LG3211_3977; phage portal family protein
OG_04653	LG3211_2233	CHI9; basic 30 kDa endochitinase
OG_04653	LG3211_2232	chiB; endochitinase domain protein
OG_04654	LG3211_4851	LG3211_4851; transposase family protein
OG_04654	LG3211_2983	LG3211_2983; transposase family protein
OG_04655	LG3211_1318	LG3211_1318; ferric iron reductase FhuF-like transporter family protein
OG_04655	LG3211_1316	LG3211_1316; ferric iron reductase FhuF-like transporter family protein
OG_04656	LG3211_2504	LG3211_2504; PAAR motif family protein
OG_04656	LG3211_4792	LG3211_4792; PAAR motif family protein
OG_04657	LG3211_4923	LG3211_4923; hypothetical protein
OG_04657	LG3211_4922	LG3211_4922; hypothetical protein
OG_09154	LG3211_1	dnaA; chromosomal replication initiator protein DnaA
OG_09155	LG3211_2	dnaN; DNA polymerase III, beta subunit
OG_09156	LG3211_3	recF; DNA replication and repair RecF family protein
OG_09157	LG3211_4	gyrB; DNA gyrase, B subunit
OG_09158	LG3211_5	LG3211_5; CAAX protease self-immunity family protein
OG_09159	LG3211_6	LG3211_6; peptidase M48 family protein
OG_09160	LG3211_11	LG3211_11; biopolymer transport ExbD/TolR family protein
OG_09161	LG3211_16	LG3211_16; isochorismatase family protein
OG_09162	LG3211_36	LG3211_36; hypothetical protein
OG_09163	LG3211_38	LG3211_38; conserved hypothetical protein
OG_09164	LG3211_39	LG3211_39; hypothetical protein
OG_09165	LG3211_56	LG3211_56; hypothetical protein
OG_09166	LG3211_63	LG3211_63; hypothetical protein
OG_09167	LG3211_71	LG3211_71; hypothetical protein
OG_09168	LG3211_81	LG3211_81; hypothetical protein
OG_09169	LG3211_83	LG3211_83; hypothetical protein
OG_09170	LG3211_90	LG3211_90; hypothetical protein
OG_09171	LG3211_122	LG3211_122; hypothetical protein
OG_09172	LG3211_125	LG3211_125; conserved hypothetical protein
OG_09173	LG3211_141	LG3211_141; hypothetical protein
OG_09174	LG3211_147	LG3211_147; membrane transport family protein
OG_09175	LG3211_162	LG3211_162; hypothetical protein
OG_09176	LG3211_163	LG3211_163; hypothetical protein
OG_09177	LG3211_167	LG3211_167; hypothetical protein
OG_09178	LG3211_173	LG3211_173; hypothetical protein
OG_09179	LG3211_181	LG3211_181; hypothetical protein
OG_09180	LG3211_194	LG3211_194; hypothetical protein
OG_09181	LG3211_195	LG3211_195; hypothetical protein

Orthologous group	*L. enz* C3<br>GeneID (#1242)<br>22.4% of genome	Gene description
OG_00015	LEC3_5272	hypothetical protein
OG_00015	LEC3_5359	hypothetical protein
OG_00015	LEC3_4793	hypothetical protein
OG_00015	LEC3_1551	hypothetical protein
OG_00015	LEC3_5251	hypothetical protein
OG_00015	LEC3_5011	hypothetical protein
OG_00015	LEC3_1952	hypothetical protein
OG_00015	LEC3_1334	hypothetical protein
OG_00015	LEC3_2352	hypothetical protein
OG_00015	LEC3_4562	hypothetical protein
OG_00015	LEC3_1783	hypothetical protein
OG_00015	LEC3_1700	hypothetical protein
OG_00015	LEC3_5367	hypothetical protein
OG_00015	LEC3_0299	hypothetical protein
OG_00015	LEC3_4954	hypothetical protein
OG_00015	LEC3_0861	hypothetical protein
OG_00015	LEC3_2971	hypothetical protein
OG_00027	LEC3_5431	hypothetical protein
OG_00027	LEC3_2293	hypothetical protein
OG_00027	LEC3_4850	hypothetical protein
OG_00027	LEC3_5056	hypothetical protein
OG_00027	LEC3_3244	hypothetical protein
OG_00027	LEC3_0216	hypothetical protein
OG_00027	LEC3_2351	hypothetical protein
OG_00027	LEC3_1224	hypothetical protein
OG_00027	LEC3_0766	conserved hypothetical protein
OG_00027	LEC3_0914	hypothetical protein
OG_00027	LEC3_4791	hypothetical protein
OG_00027	LEC3_2129	hypothetical protein
OG_00027	LEC3_0208	hypothetical protein
OG_00167	LEC3_5465	peptidase, families S8 and S53/proprotein convertase P-domain protein
OG_00167	LEC3_0421	peptidase, families S8 and S53/proprotein convertase P-domain protein
OG_00167	LEC3_0484	peptidase, families S8 and S53/proprotein convertase P-domain protein
OG_00167	LEC3_5460	peptidase, families S8 and S53/proprotein convertase P-domain protein
OG_00167	LEC3_5462	peptidase, families S8 and S53/proprotein convertase P-domain protein
OG_00167	LEC3_4434	peptidase, families S8 and S53
OG_00167	LEC3_5475	peptidase, families S8 and S53/proprotein convertase P-domain protein
OG_00168	LEC3_4435	hypothetical protein
OG_00168	LEC3_5476	conserved hypothetical protein
OG_00168	LEC3_0485	conserved hypothetical protein
OG_00168	LEC3_5461	conserved hypothetical protein
OG_00168	LEC3_5466	conserved hypothetical protein
OG_00168	LEC3_0422	conserved hypothetical protein
OG_00168	LEC3_5463	conserved hypothetical protein
OG_03613	LEC3_2014	conserved domain protein
OG_03613	LEC3_2016	conserved domain protein
OG_03613	LEC3_2015	conserved domain protein
OG_03613	LEC3_4506	conserved domain protein
OG_03976	LEC3_4433	fimbrial protein
OG_03976	LEC3_0483	fimbrial protein
OG_03976	LEC3_5464	fimbrial protein
OG_03976	LEC3_5459	fimbrial protein
OG_04425	LEC3_1175	hypothetical protein
OG_04425	LEC3_4804	hypothetical protein
OG_04425	LEC3_4203	amelogenin (Fragment)
OG_04427	LEC3_4291	hypothetical protein
OG_04427	LEC3_0964	hypothetical protein
OG_04427	LEC3_3208	hypothetical protein
OG_04430	LEC3_0003	conserved hypothetical protein
OG_04430	LEC3_5541	wall associated protein
OG_04430	LEC3_4036	wall associated protein
OG_04431	LEC3_3818	hypothetical protein
OG_04431	LEC3_2425	heme oxygenase
OG_04431	LEC3_2544	heme oxygenase
OG_04432	LEC3_0436	type VI secretion system Vgr family protein
OG_04432	LEC3_0442	type VI secretion system Vgr family protein
OG_04432	LEC3_0455	type VI secretion system Vgr family protein

OG_09182	LG3211_197	LG3211_197; hypothetical protein
OG_09183	LG3211_217	LG3211_217; hypothetical protein
OG_09184	LG3211_232	LG3211_232; hypothetical protein
OG_09185	LG3211_235	LG3211_235; hypothetical protein
OG_09186	LG3211_238	LG3211_238; hypothetical protein
OG_09187	LG3211_240	LG3211_240; hypothetical protein
OG_09188	LG3211_242	LG3211_242; hypothetical protein
OG_09189	LG3211_250	LG3211_250; hypothetical protein
OG_09190	LG3211_253	LG3211_253; hypothetical protein
OG_09191	LG3211_256	LG3211_256; hypothetical protein
OG_09192	LG3211_259	LG3211_259; hypothetical protein
OG_09193	LG3211_272	LG3211_272; TPR repeat family protein
OG_09194	LG3211_273	LG3211_273; hypothetical protein
OG_09195	LG3211_275	LG3211_275; conserved hypothetical protein
OG_09196	LG3211_283	LG3211_283; hypothetical protein
OG_09197	LG3211_306	LG3211_306; hypothetical protein
OG_09198	LG3211_314	LG3211_314; hypothetical protein
OG_09199	LG3211_319	LG3211_319; hypothetical protein
OG_09200	LG3211_320	LG3211_320; alpha/beta hydrolase fold family protein
OG_09201	LG3211_327	LG3211_327; hypothetical protein
OG_09202	LG3211_346	LG3211_346; hypothetical protein
OG_09203	LG3211_357	LG3211_357; bacterial regulatory helix-turn-helix , lysR family protein
OG_09204	LG3211_358	LG3211_358; putative glyoxalase/extradiol ring-cleavage dioxygenase prote
OG_09205	LG3211_359	LG3211_359; major royal jelly family protein
OG_09206	LG3211_378	LG3211_378; hypothetical protein
OG_09207	LG3211_379	LG3211_379; phytanoyl-CoA dioxygenase family protein
OG_09208	LG3211_382	LG3211_382; hypothetical protein
OG_09209	LG3211_397	LG3211_397; hypothetical protein
OG_09210	LG3211_403	LG3211_403; hypothetical protein
OG_09211	LG3211_405	LG3211_405; hypothetical protein
OG_09212	LG3211_410	LG3211_410; hypothetical protein
OG_09213	LG3211_414	LG3211_414; conserved hypothetical protein
OG_09214	LG3211_416	LG3211_416; mazG nucleotide pyrophosphohydrolase domain protein
OG_09215	LG3211_426	LG3211_426; hypothetical protein
OG_09216	LG3211_428	LG3211_428; hypothetical protein
OG_09217	LG3211_429	LG3211_429; hypothetical protein
OG_09218	LG3211_432	LG3211_432; hypothetical protein
OG_09219	LG3211_435	LG3211_435; hypothetical protein
OG_09220	LG3211_443	LG3211_443; hypothetical protein
OG_09221	LG3211_473	LG3211_473; hypothetical protein
OG_09222	LG3211_474	LG3211_474; hypothetical protein
OG_09223	LG3211_476	LG3211_476; lysM domain protein
OG_09224	LG3211_478	LG3211_478; hypothetical protein
OG_09225	LG3211_495	LG3211_495; hypothetical protein
OG_09226	LG3211_511	LG3211_511; hypothetical protein
OG_09227	LG3211_513	LG3211_513; hypothetical protein
OG_09228	LG3211_514	LG3211_514; hypothetical protein
OG_09229	LG3211_527	LG3211_527; hypothetical protein
OG_09230	LG3211_546	LG3211_546; hypothetical protein
OG_09231	LG3211_562	LG3211_562; hypothetical protein
OG_09232	LG3211_571	LG3211_571; hypothetical protein
OG_09233	LG3211_578	LG3211_578; hypothetical protein
OG_09234	LG3211_581	LG3211_581; hypothetical protein
OG_09235	LG3211_582	LG3211_582; hypothetical protein
OG_09236	LG3211_591	LG3211_591; hypothetical protein
OG_09237	LG3211_598	LG3211_598; hypothetical protein
OG_09238	LG3211_599	LG3211_599; hypothetical protein
OG_09239	LG3211_608	LG3211_608; hypothetical protein
OG_09240	LG3211_615	LG3211_615; hypothetical protein
OG_09241	LG3211_624	LG3211_624; hypothetical protein
OG_09242	LG3211_629	LG3211_629; hypothetical protein
OG_09243	LG3211_632	LG3211_632; hypothetical protein
OG_09244	LG3211_644	LG3211_644; hypothetical protein
OG_09245	LG3211_653	LG3211_653; hypothetical protein
OG_09246	LG3211_657	LG3211_657; conserved hypothetical protein
OG_09247	LG3211_668	LG3211_668; hypothetical protein
OG_09248	LG3211_670	LG3211_670; hypothetical protein
OG_09249	LG3211_704	LG3211_704; hypothetical protein
OG_09250	LG3211_725	LG3211_725; hypothetical protein
OG_09251	LG3211_731	LG3211_731; hypothetical protein
OG_09252	LG3211_741	LG3211_741; hypothetical protein

OG_04433	LEC3_1953	hypothetical protein
OG_04433	LEC3_1701	hypothetical protein
OG_04433	LEC3_5252	hypothetical protein
OG_04773	LEC3_5499	transcriptional regulator, XRE family
OG_04773	LEC3_0020	transcriptional regulator, XRE family
OG_05528	LEC3_4763	hypothetical protein
OG_05528	LEC3_4762	hypothetical protein
OG_05529	LEC3_0618	sigma factor regulatory protein, FecR/PupR family
OG_05529	LEC3_2547	sigma factor regulatory protein, FecR/PupR family
OG_05530	LEC3_2564	Transcriptional regulatory protein, C terminal - DNA-binding response regul
OG_05530	LEC3_4323	Transcriptional regulatory proteins, C terminal - transcriptional regulator
OG_05531	LEC3_0443	type IV / VI secretion system protein, DotU family
OG_05531	LEC3_2585	type VI secretion system OmpA/MotB family protein
OG_05532	LEC3_0210	hypothetical protein
OG_05532	LEC3_5271	hypothetical protein
OG_05533	LEC3_0384	hypothetical protein
OG_05533	LEC3_4041	hypothetical protein
OG_05534	LEC3_0581	UP0157 protein YqkA
OG_05534	LEC3_4446	conserved hypothetical protein
OG_05535	LEC3_2539	ABC transporter, ATP-binding protein
OG_05535	LEC3_2517	ABC transporter, ATP-binding protein
OG_05536	LEC3_0431	type VI secretion protein IcmF
OG_05536	LEC3_2586	type VI secretion protein IcmF
OG_05537	LEC3_5498	hypothetical protein
OG_05537	LEC3_0019	hypothetical protein
OG_05538	LEC3_1366	hypothetical protein
OG_05538	LEC3_3762	hypothetical protein
OG_05539	LEC3_0002	conserved hypothetical protein
OG_05539	LEC3_5540	hypothetical protein
OG_05540	LEC3_3714	conserved hypothetical protein
OG_05540	LEC3_5535	site-specific recombinase, resolvase family
OG_05541	LEC3_2543	membrane protein
OG_05541	LEC3_2424	conserved hypothetical protein
OG_05542	LEC3_4322	sensor histidine kinase
OG_05542	LEC3_2565	histidine kinase
OG_05543	LEC3_4023	hypothetical protein
OG_05543	LEC3_0392	conserved hypothetical protein
OG_05544	LEC3_0014	hypothetical protein
OG_05544	LEC3_4031	hypothetical protein
OG_05545	LEC3_1856	hypothetical protein
OG_05545	LEC3_2440	hypothetical protein
OG_05546	LEC3_0010	hypothetical protein
OG_05546	LEC3_5547	hypothetical protein
OG_05547	LEC3_2567	efflux transporter, RND family, MFP subunit
OG_05547	LEC3_2109	efflux transporter, RND family, MFP subunit
OG_05548	LEC3_5296	amino acid permease
OG_05548	LEC3_5307	amino acid permease
OG_05549	LEC3_4032	sensory box-containing diguanylate cyclase/cyclic diguanylate phosphodies
OG_05549	LEC3_0012	diguanylate cyclase
OG_05550	LEC3_4436	sulfotransferase
OG_05550	LEC3_3583	sulfotransferase
OG_05551	LEC3_1204	hypothetical protein
OG_05551	LEC3_4719	hypothetical protein
OG_05552	LEC3_0393	calcium-binding EF-hand
OG_05552	LEC3_4022	calcium-binding EF-hand
OG_05553	LEC3_3469	hypothetical protein
OG_05553	LEC3_3742	hypothetical protein
OG_05554	LEC3_2545	TonB-dependent receptor
OG_05554	LEC3_0617	TonB-dependent receptor
OG_05555	LEC3_2515	cyclic beta 1-2 glucan synthase
OG_05555	LEC3_2513	conserved hypothetical protein
OG_05556	LEC3_2540	TonB family C-terminal domain
OG_05556	LEC3_0614	TonB family C-terminal domain
OG_05557	LEC3_4792	hypothetical protein
OG_05557	LEC3_1782	hypothetical protein
OG_05558	LEC3_0477	hypothetical protein
OG_05558	LEC3_0478	hypothetical protein
OG_06983	LEC3_0008	conserved hypothetical protein
OG_06984	LEC3_0011	hypothetical protein
OG_06985	LEC3_0013	hypothetical protein
OG_06986	LEC3_0015	hypothetical protein

OG_09253	LG3211_752	LG3211_752; hypothetical protein
OG_09254	LG3211_769	LG3211_769; hypothetical protein
OG_09255	LG3211_776	LG3211_776; hypothetical protein
OG_09256	LG3211_779	LG3211_779; hypothetical protein
OG_09257	LG3211_781	LG3211_781; hypothetical protein
OG_09258	LG3211_786	LG3211_786; hypothetical protein
OG_09259	LG3211_787	LG3211_787; outer membrane autotransporter barrel domain protein
OG_09260	LG3211_790	LG3211_790; hypothetical protein
OG_09261	LG3211_791	LG3211_791; receptor ligand binding region family protein
OG_09262	LG3211_792	LG3211_792; hypothetical protein
OG_09263	LG3211_793	LG3211_793; conserved hypothetical protein
OG_09264	LG3211_799	LG3211_799; hypothetical protein
OG_09265	LG3211_819	LG3211_819; hypothetical protein
OG_09266	LG3211_823	LG3211_823; aldo/keto reductase domain protein
OG_09267	LG3211_824	LG3211_824; bacterial SH3 domain protein
OG_09268	LG3211_832	LG3211_832; bacterial regulatory helix-turn-helix, AraC family protein
OG_09269	LG3211_841	LG3211_841; hypothetical protein
OG_09270	LG3211_862	LG3211_862; conserved hypothetical protein
OG_09271	LG3211_870	LG3211_870; hypothetical protein
OG_09272	LG3211_877	LG3211_877; hypothetical protein
OG_09273	LG3211_878	LG3211_878; hypothetical protein
OG_09274	LG3211_889	LG3211_889; hypothetical protein
OG_09275	LG3211_895	LG3211_895; hypothetical protein
OG_09276	LG3211_916	LG3211_916; hypothetical protein
OG_09277	LG3211_938	LG3211_938; hypothetical protein
OG_09278	LG3211_943	LG3211_943; snoaL-like domain protein
OG_09279	LG3211_957	LG3211_957; hypothetical protein
OG_09280	LG3211_968	LG3211_968; hypothetical protein
OG_09281	LG3211_973	LG3211_973; hypothetical protein
OG_09282	LG3211_989	LG3211_989; V4R domain protein
OG_09283	LG3211_994	LG3211_994; hypothetical protein
OG_09284	LG3211_995	LG3211_995; hypothetical protein
OG_09285	LG3211_1016	LG3211_1016; hypothetical protein
OG_09286	LG3211_1046	LG3211_1046; prolyl oligopeptidase family protein
OG_09287	LG3211_1048	LG3211_1048; xamI restriction endonuclease family protein
OG_09288	LG3211_1050	LG3211_1050; hypothetical protein
OG_09289	LG3211_1064	LG3211_1064; hypothetical protein
OG_09290	LG3211_1067	LG3211_1067; hypothetical protein
OG_09291	LG3211_1084	LG3211_1084; hypothetical protein
OG_09292	LG3211_1088	LG3211_1088; hypothetical protein
OG_09293	LG3211_1097	LG3211_1097; hypothetical protein
OG_09294	LG3211_1101	LG3211_1101; hypothetical protein
OG_09295	LG3211_1103	LG3211_1103; hypothetical protein
OG_09296	LG3211_1104	LG3211_1104; hypothetical protein
OG_09297	LG3211_1105	LG3211_1105; hypothetical protein
OG_09298	LG3211_1112	LG3211_1112; hypothetical protein
OG_09299	LG3211_1117	LG3211_1117; hypothetical protein
OG_09300	LG3211_1138	LG3211_1138; hypothetical protein
OG_09301	LG3211_1142	LG3211_1142; hypothetical protein
OG_09302	LG3211_1149	LG3211_1149; NADPH-dependent FMN reductase family protein
OG_09303	LG3211_1150	LG3211_1150; doxX-like family protein
OG_09304	LG3211_1151	LG3211_1151; bacterial regulatory helix-turn-helix , lysR family protein
OG_09305	LG3211_1153	LG3211_1153; hypothetical protein
OG_09306	LG3211_1154	LG3211_1154; hypothetical protein
OG_09307	LG3211_1155	LG3211_1155; hypothetical protein
OG_09308	LG3211_1172	LG3211_1172; polysaccharide lyase family protein
OG_09309	LG3211_1187	LG3211_1187; putative substrate-binding domain protein
OG_09310	LG3211_1188	LG3211_1188; putative substrate-binding domain protein
OG_09311	LG3211_1190	LG3211_1190; hypothetical protein
OG_09312	LG3211_1191	LG3211_1191; hypothetical protein
OG_09313	LG3211_1192	LG3211_1192; hypothetical protein
OG_09314	LG3211_1195	LG3211_1195; hypothetical protein
OG_09315	LG3211_1196	LG3211_1196; hypothetical protein
OG_09316	LG3211_1197	LG3211_1197; hypothetical protein
OG_09317	LG3211_1202	LG3211_1202; conserved hypothetical protein
OG_09318	LG3211_1205	LG3211_1205; periplasmic binding family protein
OG_09319	LG3211_1213	LG3211_1213; hypothetical protein
OG_09320	LG3211_1214	LG3211_1214; RHS repeat-associated core domain protein
OG_09321	LG3211_1216	LG3211_1216; hypothetical protein
OG_09322	LG3211_1254	LG3211_1254; hypothetical protein
OG_09323	LG3211_1261	LG3211_1261; hypothetical protein

OG_06987	LEC3_0022	hypothetical protein
OG_06988	LEC3_0025	hypothetical protein
OG_06989	LEC3_0029	tail length tape measure protein
OG_06990	LEC3_0030	hypothetical protein
OG_06991	LEC3_0039	hypothetical protein
OG_06992	LEC3_0041	hypothetical protein
OG_06993	LEC3_0042	hypothetical protein
OG_06994	LEC3_0045	hypothetical protein
OG_06995	LEC3_0050	hypothetical protein
OG_06996	LEC3_0051	hypothetical protein
OG_06997	LEC3_0052	hypothetical protein
OG_06998	LEC3_0056	hypothetical protein
OG_06999	LEC3_0064	hypothetical protein
OG_07000	LEC3_0071	hypothetical protein
OG_07001	LEC3_0073	hypothetical protein
OG_07002	LEC3_0074	hypothetical protein
OG_07003	LEC3_0079	hypothetical protein
OG_07004	LEC3_0093	hypothetical protein
OG_07005	LEC3_0094	hypothetical protein
OG_07006	LEC3_0097	conserved hypothetical protein
OG_07007	LEC3_0107	hypothetical protein
OG_07008	LEC3_0118	hypothetical protein
OG_07009	LEC3_0120	hypothetical protein
OG_07010	LEC3_0131	hypothetical protein
OG_07011	LEC3_0135	hypothetical protein
OG_07012	LEC3_0137	conserved hypothetical protein
OG_07013	LEC3_0138	hypothetical protein
OG_07014	LEC3_0139	conserved hypothetical protein
OG_07015	LEC3_0145	hypothetical protein
OG_07016	LEC3_0150	hypothetical protein
OG_07017	LEC3_0153	hypothetical protein
OG_07018	LEC3_0156	hypothetical protein
OG_07019	LEC3_0161	lipoprotein
OG_07020	LEC3_0162	hypothetical protein
OG_07021	LEC3_0167	hypothetical protein
OG_07022	LEC3_0186	hypothetical protein
OG_07023	LEC3_0195	hypothetical protein
OG_07024	LEC3_0207	hypothetical protein
OG_07025	LEC3_0217	hypothetical protein
OG_07026	LEC3_0219	hypothetical protein
OG_07027	LEC3_0228	hypothetical protein
OG_07028	LEC3_0234	conserved hypothetical protein
OG_07029	LEC3_0239	hypothetical protein
OG_07030	LEC3_0255	hypothetical protein
OG_07031	LEC3_0266	hypothetical protein
OG_07032	LEC3_0280	transcriptional regulator, MarR family
OG_07033	LEC3_0281	conserved domain protein
OG_07034	LEC3_0296	amidohydrolase family protein
OG_07035	LEC3_0298	hypothetical protein
OG_07036	LEC3_0300	conserved hypothetical protein
OG_07037	LEC3_0313	lipoprotein
OG_07038	LEC3_0315	hypothetical protein
OG_07039	LEC3_0318	hypothetical protein
OG_07040	LEC3_0320	PAAR motif family protein
OG_07041	LEC3_0330	hypothetical protein
OG_07042	LEC3_0348	hypothetical protein
OG_07043	LEC3_0355	hypothetical protein
OG_07044	LEC3_0370	hypothetical protein
OG_07045	LEC3_0371	hypothetical protein
OG_07046	LEC3_0372	hypothetical protein
OG_07047	LEC3_0380	hypothetical protein
OG_07048	LEC3_0381	KAP family P-loop domain protein
OG_07049	LEC3_0382	hypothetical protein
OG_07050	LEC3_0383	conserved hypothetical protein
OG_07051	LEC3_0391	acetyltransferase (GNAT) family
OG_07052	LEC3_0394	conserved hypothetical protein
OG_07053	LEC3_0400	glutaredoxin domain protein
OG_07054	LEC3_0402	conserved hypothetical protein
OG_07055	LEC3_0403	conserved hypothetical protein
OG_07056	LEC3_0404	RNA polymerase sigma factor, ECF family
OG_07057	LEC3_0405	serine/threonine-protein kinase Pkn2

OG_09324	LG3211_1268	LG3211_1268; hypothetical protein
OG_09325	LG3211_1275	LG3211_1275; hypothetical protein
OG_09326	LG3211_1279	LG3211_1279; hypothetical protein
OG_09327	LG3211_1297	LG3211_1297; cupin domain protein
OG_09328	LG3211_1300	LG3211_1300; conserved hypothetical protein
OG_09329	LG3211_1305	LG3211_1305; hypothetical protein
OG_09330	LG3211_1313	LG3211_1313; hypothetical protein
OG_09331	LG3211_1314	LG3211_1314; hpcH/HpaI aldolase/citrate lyase family protein
OG_09332	LG3211_1315	LG3211_1315; ATP-grasp domain protein
OG_09333	LG3211_1317	LG3211_1317; major Facilitator Superfamily protein
OG_09334	LG3211_1320	LG3211_1320; pyridoxal-dependent decarboxylase, pyridoxal binding doma
OG_09335	LG3211_1334	LG3211_1334; hypothetical protein
OG_09336	LG3211_1339	LG3211_1339; hypothetical protein
OG_09337	LG3211_1357	LG3211_1357; hypothetical protein
OG_09338	LG3211_1368	LG3211_1368; hypothetical protein
OG_09339	LG3211_1400	LG3211_1400; conserved hypothetical protein
OG_09340	LG3211_1432	LG3211_1432; GDSL-like Lipase/Acylhydrolase family protein
OG_09341	LG3211_1455	LG3211_1455; hypothetical protein
OG_09342	LG3211_1456	LG3211_1456; hypothetical protein
OG_09343	LG3211_1473	LG3211_1473; hypothetical protein
OG_09344	LG3211_1487	LG3211_1487; hypothetical protein
OG_09345	LG3211_1489	LG3211_1489; hypothetical protein
OG_09346	LG3211_1491	LG3211_1491; hypothetical protein
OG_09347	LG3211_1498	LG3211_1498; hypothetical protein
OG_09348	LG3211_1504	LG3211_1504; hypothetical protein
OG_09349	LG3211_1521	LG3211_1521; hypothetical protein
OG_09350	LG3211_1523	LG3211_1523; hypothetical protein
OG_09351	LG3211_1524	LG3211_1524; hypothetical protein
OG_09352	LG3211_1525	LG3211_1525; hypothetical protein
OG_09353	LG3211_1526	LG3211_1526; hypothetical protein
OG_09354	LG3211_1533	LG3211_1533; hypothetical protein
OG_09355	LG3211_1540	LG3211_1540; hypothetical protein
OG_09356	LG3211_1541	LG3211_1541; hypothetical protein
OG_09357	LG3211_1545	LG3211_1545; hypothetical protein
OG_09358	LG3211_1547	LG3211_1547; TPR repeat family protein
OG_09359	LG3211_1564	LG3211_1564; hypothetical protein
OG_09360	LG3211_1566	LG3211_1566; YCII-related domain protein
OG_09361	LG3211_1568	LG3211_1568; conserved hypothetical protein
OG_09362	LG3211_1579	LG3211_1579; hypothetical protein
OG_09363	LG3211_1582	LG3211_1582; hypothetical protein
OG_09364	LG3211_1595	LG3211_1595; hypothetical protein
OG_09365	LG3211_1597	LG3211_1597; hypothetical protein
OG_09366	LG3211_1626	LG3211_1626; hypothetical protein
OG_09367	LG3211_1627	LG3211_1627; hypothetical protein
OG_09368	LG3211_1630	LG3211_1630; hypothetical protein
OG_09369	LG3211_1639	LG3211_1639; hypothetical protein
OG_09370	LG3211_1650	LG3211_1650; hypothetical protein
OG_09371	LG3211_1651	LG3211_1651; hypothetical protein
OG_09372	LG3211_1653	LG3211_1653; hypothetical protein
OG_09373	LG3211_1654	LG3211_1654; hypothetical protein
OG_09374	LG3211_1655	LG3211_1655; sodium:solute symporter family protein
OG_09375	LG3211_1656	LG3211_1656; tonB dependent receptor family protein
OG_09376	LG3211_1657	LG3211_1657; mandelate racemase / muconate lactonizing enzyme, C-term
OG_09377	LG3211_1658	LG3211_1658; bacterial transcriptional regulator family protein
OG_09378	LG3211_1659	LG3211_1659; short chain dehydrogenase family protein
OG_09379	LG3211_1660	LG3211_1660; 2-keto-3-deoxy-galactonokinase family protein
OG_09380	LG3211_1661	LG3211_1661; DEHYDDEOXPHOSGALACT
OG_09381	LG3211_1666	LG3211_1666; hypothetical protein
OG_09382	LG3211_1684	LG3211_1684; hypothetical protein
OG_09383	LG3211_1697	LG3211_1697; hypothetical protein
OG_09384	LG3211_1700	LG3211_1700; conserved hypothetical protein
OG_09385	LG3211_1713	LG3211_1713; hypothetical protein
OG_09386	LG3211_1716	LG3211_1716; conserved hypothetical protein
OG_09387	LG3211_1746	LG3211_1746; hypothetical protein
OG_09388	LG3211_1775	LG3211_1775; putative peptidoglycan-binding protein
OG_09389	LG3211_1788	LG3211_1788; hypothetical protein
OG_09390	LG3211_1797	LG3211_1797; hypothetical protein
OG_09391	LG3211_1803	LG3211_1803; hypothetical protein
OG_09392	LG3211_1804	LG3211_1804; conserved hypothetical protein
OG_09393	LG3211_1808	LG3211_1808; hypothetical protein
OG_09394	LG3211_1809	LG3211_1809; hypothetical protein

OG_07058	LEC3_0406	hypothetical protein
OG_07059	LEC3_0407	hypothetical protein
OG_07060	LEC3_0408	para-aminobenzoate synthase
OG_07061	LEC3_0409	conserved hypothetical protein
OG_07062	LEC3_0410	alpha-amino acid ester hydrolase
OG_07063	LEC3_0411	hypothetical protein
OG_07064	LEC3_0417	hypothetical protein
OG_07065	LEC3_0419	hypothetical protein
OG_07066	LEC3_0420	conserved hypothetical protein
OG_07067	LEC3_0423	conserved hypothetical protein
OG_07068	LEC3_0427	conserved hypothetical protein
OG_07069	LEC3_0428	L-alanyl-D-glutamate peptidase
OG_07070	LEC3_0429	hypothetical protein
OG_07071	LEC3_0430	conserved hypothetical protein
OG_07072	LEC3_0432	type VI secretion-associated protein, BMA_A0400 family
OG_07073	LEC3_0437	conserved hypothetical protein
OG_07074	LEC3_0438	conserved hypothetical protein
OG_07075	LEC3_0439	hypothetical protein
OG_07076	LEC3_0440	hypothetical protein
OG_07077	LEC3_0441	arginine deiminase
OG_07078	LEC3_0445	type VI secretion lipoprotein, VC_A0113 family
OG_07079	LEC3_0446	tetratricopeptide repeat protein
OG_07080	LEC3_0450	lysine-specific histone demethylase 1
OG_07081	LEC3_0456	conserved hypothetical protein
OG_07082	LEC3_0457	hypothetical protein
OG_07083	LEC3_0458	hypothetical protein
OG_07084	LEC3_0459	hypothetical protein
OG_07085	LEC3_0460	hypothetical protein
OG_07086	LEC3_0461	hypothetical protein
OG_07087	LEC3_0468	hypothetical protein
OG_07088	LEC3_0470	hypothetical protein
OG_07089	LEC3_0471	hypothetical protein
OG_07090	LEC3_0472	hypothetical protein
OG_07091	LEC3_0475	hypothetical protein
OG_07092	LEC3_0476	lipoprotein
OG_07093	LEC3_0479	hypothetical protein
OG_07094	LEC3_0488	hypothetical protein
OG_07095	LEC3_0491	hypothetical protein
OG_07096	LEC3_0494	Arabinosidase
OG_07097	LEC3_0496	transcriptional regulator, LysR family
OG_07098	LEC3_0497	membrane protein
OG_07099	LEC3_0499	acriflavine resistance protein A
OG_07100	LEC3_0500	conserved hypothetical protein
OG_07101	LEC3_0502	chromosome replication initiation inhibitor
OG_07102	LEC3_0503	translocator protein, LysE family
OG_07103	LEC3_0505	rare lipoprotein A
OG_07104	LEC3_0506	hypothetical protein
OG_07105	LEC3_0509	hypothetical protein
OG_07106	LEC3_0517	hypothetical protein
OG_07107	LEC3_0518	hypothetical protein
OG_07108	LEC3_0520	hypothetical protein
OG_07109	LEC3_0530	hypothetical protein
OG_07110	LEC3_0531	hypothetical protein
OG_07111	LEC3_0532	hypothetical protein
OG_07112	LEC3_0535	hypothetical protein
OG_07113	LEC3_0544	linear gramicidin synthetase subunit C
OG_07114	LEC3_0546	linear gramicidin synthetase subunit D
OG_07115	LEC3_0550	hypothetical protein
OG_07116	LEC3_0552	transcriptional regulator, AraC family
OG_07117	LEC3_0555	hypothetical protein
OG_07118	LEC3_0556	hypothetical protein
OG_07119	LEC3_0557	hypothetical protein
OG_07120	LEC3_0560	conserved hypothetical protein
OG_07121	LEC3_0562	probable acetyltransferase
OG_07122	LEC3_0567	hypothetical protein
OG_07123	LEC3_0569	two-component system response regulator, LuxR family
OG_07124	LEC3_0570	hypothetical protein
OG_07125	LEC3_0571	hypothetical protein
OG_07126	LEC3_0573	hypothetical protein
OG_07127	LEC3_0583	hypothetical protein
OG_07128	LEC3_0590	hypothetical protein

OG_09395	LG3211_1812	LG3211_1812; hypothetical protein
OG_09396	LG3211_1813	LG3211_1813; autotransporter beta-domain protein
OG_09397	LG3211_1820	LG3211_1820; hypothetical protein
OG_09398	LG3211_1825	LG3211_1825; hypothetical protein
OG_09399	LG3211_1826	LG3211_1826; anti-sigma-K factor rskA family protein
OG_09400	LG3211_1829	LG3211_1829; bacterial regulatory helix-turn-helix , lysR family protein
OG_09401	LG3211_1830	LG3211_1830; tonB-dependent receptor family protein
OG_09402	LG3211_1831	xghA; xyloglucanase Xgh74A
OG_09403	LG3211_1832	LG3211_1832; glycosyl hydrolases family 2, sugar binding domain protein
OG_09404	LG3211_1833	LG3211_1833; glycosyl hydrolases family 2, sugar binding domain protein
OG_09405	LG3211_1834	LG3211_1834; beta-galactosidase family protein
OG_09406	LG3211_1836	LG3211_1836; putative alpha-L-fucosidase
OG_09407	LG3211_1837	xylB; xylulokinase
OG_09408	LG3211_1838	xylA; xylose isomerase
OG_09409	LG3211_1841	LG3211_1841; hypothetical protein
OG_09410	LG3211_1842	celA; endoglucanase A
OG_09411	LG3211_1844	AbjA; alpha-L-arabinofuranosidase
OG_09412	LG3211_1845	LG3211_1845; asparagine synthase domain protein
OG_09413	LG3211_1852	LG3211_1852; hypothetical protein
OG_09414	LG3211_1853	LG3211_1853; hypothetical protein
OG_09415	LG3211_1856	dhaK; dihydroxyacetone kinase
OG_09416	LG3211_1868	LG3211_1868; hypothetical protein
OG_09417	LG3211_1870	LG3211_1870; hypothetical protein
OG_09418	LG3211_1900	LG3211_1900; isoprenylcysteine carboxyl methyltransferase family protein
OG_09419	LG3211_1902	LG3211_1902; conserved hypothetical protein
OG_09420	LG3211_1903	LG3211_1903; bacterial regulatory, tetR family protein
OG_09421	LG3211_1906	LG3211_1906; conserved hypothetical protein
OG_09422	LG3211_1907	LG3211_1907; pyridoxamine 5'-phosphate oxidase family protein
OG_09423	LG3211_1908	LG3211_1908; serine hydrolase family protein
OG_09424	LG3211_1913	LG3211_1913; short chain dehydrogenase family protein
OG_09425	LG3211_1914	LG3211_1914; bacterial regulatory helix-turn-helix , lysR family protein
OG_09426	LG3211_1921	LG3211_1921; hypothetical protein
OG_09427	LG3211_1928	LG3211_1928; hypothetical protein
OG_09428	LG3211_1929	LG3211_1929; ompA family protein
OG_09429	LG3211_1930	LG3211_1930; hypothetical protein
OG_09430	LG3211_1931	LG3211_1931; conserved hypothetical protein
OG_09431	LG3211_1932	LG3211_1932; conserved hypothetical protein
OG_09432	LG3211_1933	LG3211_1933; conserved hypothetical protein
OG_09433	LG3211_1934	LG3211_1934; hypothetical protein
OG_09434	LG3211_1953	LG3211_1953; hypothetical protein
OG_09435	LG3211_1954	LG3211_1954; hypothetical protein
OG_09436	LG3211_1968	LG3211_1968; bacterial regulatory helix-turn-helix, AraC family protein
OG_09437	LG3211_1970	LG3211_1970; hypothetical protein
OG_09438	LG3211_1979	LG3211_1979; hypothetical protein
OG_09439	LG3211_1982	LG3211_1982; hypothetical protein
OG_09440	LG3211_1983	LG3211_1983; conserved hypothetical protein
OG_09441	LG3211_1985	LG3211_1985; hypothetical protein
OG_09442	LG3211_1986	LG3211_1986; hypothetical protein
OG_09443	LG3211_1987	LG3211_1987; tonB family C-terminal domain protein
OG_09444	LG3211_1989	LG3211_1989; hypothetical protein
OG_09445	LG3211_1996	LG3211_1996; hypothetical protein
OG_09446	LG3211_2000	LG3211_2000; sigma-70, region 4 family protein
OG_09447	LG3211_2004	LG3211_2004; TM1410 hypothetical-related family protein
OG_09448	LG3211_2005	LG3211_2005; hypothetical protein
OG_09449	LG3211_2009	LG3211_2009; conserved hypothetical protein
OG_09450	LG3211_2014	LG3211_2014; hypothetical protein
OG_09451	LG3211_2021	LG3211_2021; hypothetical protein
OG_09452	LG3211_2034	LG3211_2034; hypothetical protein
OG_09453	LG3211_2035	LG3211_2035; hypothetical protein
OG_09454	LG3211_2037	LG3211_2037; hypothetical protein
OG_09455	LG3211_2047	LG3211_2047; hypothetical protein
OG_09456	LG3211_2048	LG3211_2048; zeta toxin family protein
OG_09457	LG3211_2050	LG3211_2050; conserved hypothetical protein
OG_09458	LG3211_2052	LG3211_2052; zeta toxin family protein
OG_09459	LG3211_2055	LG3211_2055; hypothetical protein
OG_09460	LG3211_2057	LG3211_2057; hypothetical protein
OG_09461	LG3211_2059	LG3211_2059; hypothetical protein
OG_09462	LG3211_2062	LG3211_2062; hypothetical protein
OG_09463	LG3211_2065	LG3211_2065; hypothetical protein
OG_09464	LG3211_2073	LG3211_2073; hypothetical protein
OG_09465	LG3211_2074	LG3211_2074; hypothetical protein

OG_07129	LEC3_0592	hypothetical protein
OG_07130	LEC3_0593	histidine kinase family protein
OG_07131	LEC3_0594	hypothetical protein
OG_07132	LEC3_0596	hypothetical protein
OG_07133	LEC3_0597	lipoprotein
OG_07134	LEC3_0611	hypothetical protein
OG_07135	LEC3_0615	conserved hypothetical protein
OG_07136	LEC3_0616	conserved hypothetical protein
OG_07137	LEC3_0623	hypothetical protein
OG_07138	LEC3_0626	conserved hypothetical protein
OG_07139	LEC3_0627	peptidase, M16 family
OG_07140	LEC3_0628	cystathionine beta-lyase
OG_07141	LEC3_0630	ribbon-helix-helix protein, CopG family
OG_07142	LEC3_0637	hypothetical protein
OG_07143	LEC3_0643	translocator protein, LysE family
OG_07144	LEC3_0644	hypothetical protein SEQUENCING GAP
OG_07145	LEC3_0645	beta-lactamase
OG_07146	LEC3_0646	histidine kinase
OG_07147	LEC3_0647	response regulAtor containing a LytTR DNA-binding domain
OG_07148	LEC3_0650	hypothetical protein
OG_07149	LEC3_0664	hypothetical protein
OG_07150	LEC3_0670	glyoxalase/bleomycin resistance protein/dioxygenase
OG_07151	LEC3_0672	hypothetical protein
OG_07152	LEC3_0674	hypothetical protein
OG_07153	LEC3_0680	hypothetical protein
OG_07154	LEC3_0695	hypothetical protein
OG_07155	LEC3_0703	conserved hypothetical protein
OG_07156	LEC3_0705	hypothetical protein
OG_07157	LEC3_0706	lipoprotein
OG_07158	LEC3_0712	conserved hypothetical protein
OG_07159	LEC3_0739	conserved hypothetical protein
OG_07160	LEC3_0740	hypothetical protein
OG_07161	LEC3_0742	conserved hypothetical protein
OG_07162	LEC3_0750	hypothetical protein
OG_07163	LEC3_0773	cell wall surface anchor family protein
OG_07164	LEC3_0787	conserved hypothetical protein
OG_07165	LEC3_0789	DNA polymerase III, subunits gamma and tau-like protein
OG_07166	LEC3_0791	conserved hypothetical protein
OG_07167	LEC3_0792	hypothetical protein
OG_07168	LEC3_0802	hypothetical protein
OG_07169	LEC3_0804	hypothetical protein
OG_07170	LEC3_0806	hypothetical protein
OG_07171	LEC3_0814	hypothetical protein
OG_07172	LEC3_0830	hypothetical protein
OG_07173	LEC3_0832	hypothetical protein
OG_07174	LEC3_0839	hypothetical protein
OG_07175	LEC3_0840	hypothetical protein
OG_07176	LEC3_0846	hypothetical protein
OG_07177	LEC3_0855	lipoprotein
OG_07178	LEC3_0858	conserved hypothetical protein
OG_07179	LEC3_0860	hypothetical protein
OG_07180	LEC3_0862	hypothetical protein
OG_07181	LEC3_0878	hypothetical protein
OG_07182	LEC3_0903	hypothetical protein
OG_07183	LEC3_0920	hypothetical protein
OG_07184	LEC3_0926	hypothetical protein
OG_07185	LEC3_0938	hypothetical protein
OG_07186	LEC3_0945	hypothetical protein
OG_07187	LEC3_0947	hypothetical protein
OG_07188	LEC3_0959	hypothetical protein
OG_07189	LEC3_0963	diguanylate cyclase
OG_07190	LEC3_0966	hypothetical protein
OG_07191	LEC3_0967	hypothetical protein
OG_07192	LEC3_0972	hypothetical protein
OG_07193	LEC3_0979	hypothetical protein
OG_07194	LEC3_0984	hypothetical protein
OG_07195	LEC3_0991	hypothetical protein
OG_07196	LEC3_1007	hypothetical protein
OG_07197	LEC3_1013	hypothetical protein
OG_07198	LEC3_1014	hypothetical protein
OG_07199	LEC3_1015	hypothetical protein

OG_09466	LG3211_2081	LG3211_2081; hypothetical protein
OG_09467	LG3211_2082	LG3211_2082; hypothetical protein
OG_09468	LG3211_2092	LG3211_2092; hypothetical protein
OG_09469	LG3211_2093	LG3211_2093; hypothetical protein
OG_09470	LG3211_2117	LG3211_2117; hypothetical protein
OG_09471	LG3211_2121	LG3211_2121; transcriptional regulator, LysR family domain protein
OG_09472	LG3211_2123	LG3211_2123; hypothetical protein
OG_09473	LG3211_2125	LG3211_2125; hypothetical protein
OG_09474	LG3211_2127	LG3211_2127; hypothetical protein
OG_09475	LG3211_2130	LG3211_2130; hypothetical protein
OG_09476	LG3211_2137	LG3211_2137; hypothetical protein
OG_09477	LG3211_2139	LG3211_2139; hypothetical protein
OG_09478	LG3211_2151	LG3211_2151; hypothetical protein
OG_09479	LG3211_2160	LG3211_2160; hypothetical protein
OG_09480	LG3211_2162	LG3211_2162; hypothetical protein
OG_09481	LG3211_2169	LG3211_2169; transcriptional regulatory , C terminal family protein
OG_09482	LG3211_2173	LG3211_2173; hypothetical protein
OG_09483	LG3211_2180	LG3211_2180; hypothetical protein
OG_09484	LG3211_2213	LG3211_2213; hypothetical protein
OG_09485	LG3211_2222	LG3211_2222; hypothetical protein
OG_09486	LG3211_2224	LG3211_2224; hypothetical protein
OG_09487	LG3211_2228	LG3211_2228; hypothetical protein
OG_09488	LG3211_2229	LG3211_2229; hypothetical protein
OG_09489	LG3211_2234	LG3211_2234; conserved hypothetical protein
OG_09490	LG3211_2235	LG3211_2235; conserved hypothetical protein
OG_09491	LG3211_2240	LG3211_2240; hypothetical protein
OG_09492	LG3211_2241	LG3211_2241; type I phosphodiesterase / nucleotide pyrophosphatase fam
OG_09493	LG3211_2245	LG3211_2245; hypothetical protein
OG_09494	LG3211_2246	LG3211_2246; hypothetical protein
OG_09495	LG3211_2262	LG3211_2262; hypothetical protein
OG_09496	LG3211_2271	LG3211_2271; hypothetical protein
OG_09497	LG3211_2283	LG3211_2283; hypothetical protein
OG_09498	LG3211_2285	LG3211_2285; hypothetical protein
OG_09499	LG3211_2303	LG3211_2303; hypothetical protein
OG_09500	LG3211_2309	LG3211_2309; hypothetical protein
OG_09501	LG3211_2320	LG3211_2320; hypothetical protein
OG_09502	LG3211_2322	LG3211_2322; hypothetical protein
OG_09503	LG3211_2326	LG3211_2326; hypothetical protein
OG_09504	LG3211_2332	LG3211_2332; hypothetical protein
OG_09505	LG3211_2334	LG3211_2334; hypothetical protein
OG_09506	LG3211_2360	LG3211_2360; hypothetical protein
OG_09507	LG3211_2362	LG3211_2362; hypothetical protein
OG_09508	LG3211_2363	LG3211_2363; hypothetical protein
OG_09509	LG3211_2369	LG3211_2369; hypothetical protein
OG_09510	LG3211_2380	LG3211_2380; hypothetical protein
OG_09511	LG3211_2381	LG3211_2381; hypothetical protein
OG_09512	LG3211_2390	LG3211_2390; hypothetical protein
OG_09513	LG3211_2392	LG3211_2392; hypothetical protein
OG_09514	LG3211_2400	LG3211_2400; transcriptional regulator, LysR family domain protein
OG_09515	LG3211_2401	LG3211_2401; hypothetical protein
OG_09516	LG3211_2412	LG3211_2412; hypothetical protein
OG_09517	LG3211_2428	LG3211_2428; hypothetical protein
OG_09518	LG3211_2438	LG3211_2438; hypothetical protein
OG_09519	LG3211_2440	LG3211_2440; sugar (and other) transporter family protein
OG_09520	LG3211_2441	LG3211_2441; hypothetical protein
OG_09521	LG3211_2446	bglF; beta-1,3-glucanase
OG_09522	LG3211_2450	LG3211_2450; hypothetical protein
OG_09523	LG3211_2462	LG3211_2462; subtilase family protein
OG_09524	LG3211_2463	LG3211_2463; hypothetical protein
OG_09525	LG3211_2468	LG3211_2468; hypothetical protein
OG_09526	LG3211_2469	LG3211_2469; hypothetical protein
OG_09527	LG3211_2472	LG3211_2472; hypothetical protein
OG_09528	LG3211_2473	LG3211_2473; hypothetical protein
OG_09529	LG3211_2474	LG3211_2474; hypothetical protein
OG_09530	LG3211_2475	dltA; D-alanine--poly(phosphoribitol) ligase, subunit 1
OG_09531	LG3211_2480	LG3211_2480; efflux transporter, RND family, MFP subunit
OG_09532	LG3211_2483	LG3211_2483; conserved hypothetical protein
OG_09533	LG3211_2484	LG3211_2484; conserved hypothetical protein
OG_09534	LG3211_2501	LG3211_2501; 3D domain protein
OG_09535	LG3211_2502	LG3211_2502; ompA family protein
OG_09536	LG3211_2503	LG3211_2503; hypothetical protein

OG_07200	LEC3_1020	hypothetical protein
OG_07201	LEC3_1031	response regulator receiver domain protein
OG_07202	LEC3_1042	conserved hypothetical protein
OG_07203	LEC3_1053	hypothetical protein
OG_07204	LEC3_1060	hypothetical protein
OG_07205	LEC3_1064	hypothetical protein
OG_07206	LEC3_1076	hypothetical protein
OG_07207	LEC3_1077	hypothetical protein
OG_07208	LEC3_1078	hypothetical protein
OG_07209	LEC3_1087	hypothetical protein
OG_07210	LEC3_1093	hypothetical protein
OG_07211	LEC3_1097	hypothetical protein
OG_07212	LEC3_1106	hypothetical protein
OG_07213	LEC3_1112	hypothetical protein
OG_07214	LEC3_1136	hypothetical protein
OG_07215	LEC3_1138	hypothetical protein
OG_07216	LEC3_1145	hypothetical protein
OG_07217	LEC3_1161	haloacid dehalogenase-like hydrolase
OG_07218	LEC3_1166	hypothetical protein
OG_07219	LEC3_1168	hypothetical protein
OG_07220	LEC3_1170	hypothetical protein
OG_07221	LEC3_1181	conserved hypothetical protein
OG_07222	LEC3_1186	lipoprotein
OG_07223	LEC3_1205	hypothetical protein
OG_07224	LEC3_1211	hypothetical protein
OG_07225	LEC3_1219	hypothetical protein
OG_07226	LEC3_1231	hypothetical protein
OG_07227	LEC3_1235	hypothetical protein
OG_07228	LEC3_1250	hypothetical protein
OG_07229	LEC3_1265	hypothetical protein
OG_07230	LEC3_1269	hypothetical protein
OG_07231	LEC3_1298	lipoprotein
OG_07232	LEC3_1302	hypothetical protein
OG_07233	LEC3_1306	hypothetical protein
OG_07234	LEC3_1310	hypothetical protein
OG_07235	LEC3_1315	hypothetical protein
OG_07236	LEC3_1321	hypothetical protein
OG_07237	LEC3_1322	Rhs element Vgr protein
OG_07238	LEC3_1323	hypothetical protein
OG_07239	LEC3_1328	hypothetical protein
OG_07240	LEC3_1329	hypothetical protein
OG_07241	LEC3_1330	hypothetical protein
OG_07242	LEC3_1331	hypothetical protein
OG_07243	LEC3_1335	hypothetical protein
OG_07244	LEC3_1341	hypothetical protein
OG_07245	LEC3_1344	hypothetical protein
OG_07246	LEC3_1347	hypothetical protein
OG_07247	LEC3_1350	hypothetical protein
OG_07248	LEC3_1386	hypothetical protein
OG_07249	LEC3_1397	conserved hypothetical protein
OG_07250	LEC3_1398	hypothetical protein
OG_07251	LEC3_1401	hypothetical protein
OG_07252	LEC3_1404	conserved hypothetical protein
OG_07253	LEC3_1417	hypothetical protein
OG_07254	LEC3_1425	conserved hypothetical protein
OG_07255	LEC3_1433	hypothetical protein SEQUENCING GAP
OG_07256	LEC3_1438	hypothetical protein
OG_07257	LEC3_1440	hypothetical protein
OG_07258	LEC3_1441	hypothetical protein
OG_07259	LEC3_1442	hypothetical protein
OG_07260	LEC3_1452	RepB plasmid partitioning protein
OG_07261	LEC3_1453	ParB-like partition proteins
OG_07262	LEC3_1454	site-specific recombinase, integrase family
OG_07263	LEC3_1455	conserved hypothetical protein
OG_07264	LEC3_1458	transposase
OG_07265	LEC3_1465	hypothetical protein
OG_07266	LEC3_1466	conserved hypothetical protein
OG_07267	LEC3_1474	hypothetical protein
OG_07268	LEC3_1476	hypothetical protein
OG_07269	LEC3_1480	conserved hypothetical protein
OG_07270	LEC3_1482	hypothetical protein

OG_09537	LG3211_2505	LG3211_2505; hypothetical protein
OG_09538	LG3211_2506	LG3211_2506; putative peptidoglycan binding domain protein
OG_09539	LG3211_2511	phnD; phosphate/phosphite/phosphonate ABC transporters, periplasmic bi
OG_09540	LG3211_2512	LG3211_2512; bacterial regulatory, luxR family protein
OG_09541	LG3211_2513	LG3211_2513; traB family protein
OG_09542	LG3211_2515	LG3211_2515; conserved hypothetical protein
OG_09543	LG3211_2516	LG3211_2516; imcF-related N-terminal domain protein
OG_09544	LG3211_2521	LG3211_2521; conserved hypothetical protein
OG_09545	LG3211_2522	LG3211_2522; hypothetical protein
OG_09546	LG3211_2524	LG3211_2524; HAMP domain protein
OG_09547	LG3211_2525	LG3211_2525; conserved hypothetical protein
OG_09548	LG3211_2526	LG3211_2526; hypothetical protein
OG_09549	LG3211_2527	LG3211_2527; TPR repeat family protein
OG_09550	LG3211_2529	LG3211_2529; filamentous hemagglutinin family N-terminal domain protei
OG_09551	LG3211_2531	hex; B-N-acetylhexosaminidase
OG_09552	LG3211_2532	LG3211_2532; tonB-dependent receptor family protein
OG_09553	LG3211_2535	LG3211_2535; hypothetical protein
OG_09554	LG3211_2537	LG3211_2537; hypothetical protein
OG_09555	LG3211_2541	LG3211_2541; conserved hypothetical protein
OG_09556	LG3211_2542	LG3211_2542; conserved hypothetical protein
OG_09557	LG3211_2547	LG3211_2547; cupin domain protein
OG_09558	LG3211_2555	LG3211_2555; type I phosphodiesterase / nucleotide pyrophosphatase fam
OG_09559	LG3211_2558	LG3211_2558; gram-negative porin family protein
OG_09560	LG3211_2559	LG3211_2559; putative phosphonoacetaldehyde dehydrogenase
OG_09561	LG3211_2560	LG3211_2560; major Facilitator Superfamily protein
OG_09562	LG3211_2561	LG3211_2561; iron-containing alcohol dehydrogenase family protein
OG_09563	LG3211_2563	phnA; phosphonoacetate hydrolase
OG_09564	LG3211_2564	LG3211_2564; bacterial regulatory helix-turn-helix , lysR family protein
OG_09565	LG3211_2567	LG3211_2567; hypothetical protein
OG_09566	LG3211_2568	LG3211_2568; hypothetical protein
OG_09567	LG3211_2569	LG3211_2569; calx-beta domain protein
OG_09568	LG3211_2573	LG3211_2573; hypothetical protein
OG_09569	LG3211_2575	LG3211_2575; helix-turn-helix family protein
OG_09570	LG3211_2576	LG3211_2576; hypothetical protein
OG_09571	LG3211_2580	LG3211_2580; osmC-like family protein
OG_09572	LG3211_2584	LG3211_2584; conserved hypothetical protein
OG_09573	LG3211_2585	LG3211_2585; gluconate 2-dehydrogenase subunit 3 family protein
OG_09574	LG3211_2586	LG3211_2586; GMC oxidoreductase family protein
OG_09575	LG3211_2587	LG3211_2587; xylose isomerase-like TIM barrel family protein
OG_09576	LG3211_2588	LG3211_2588; hypothetical protein
OG_09577	LG3211_2589	LG3211_2589; hypothetical protein
OG_09578	LG3211_2590	LG3211_2590; formylglycine-generating sulfatase enzyme family protein
OG_09579	LG3211_2591	LG3211_2591; oxidoreductase , NAD-binding Rossmann fold family protein
OG_09580	LG3211_2592	LG3211_2592; apbE family protein
OG_09581	LG3211_2594	LG3211_2594; major Facilitator Superfamily protein
OG_09582	LG3211_2595	LG3211_2595; hypothetical protein
OG_09583	LG3211_2596	LG3211_2596; xylose isomerase-like TIM barrel family protein
OG_09584	LG3211_2597	LG3211_2597; xylose isomerase-like TIM barrel family protein
OG_09585	LG3211_2598	LG3211_2598; endoribonuclease L-PSP family protein
OG_09586	LG3211_2602	LG3211_2602; conserved hypothetical protein
OG_09587	LG3211_2603	LG3211_2603; hypothetical protein
OG_09588	LG3211_2605	serA; serA
OG_09589	LG3211_2610	LG3211_2610; hypothetical protein
OG_09590	LG3211_2625	LG3211_2625; hypothetical protein
OG_09591	LG3211_2630	LG3211_2630; hypothetical protein
OG_09592	LG3211_2633	LG3211_2633; hypothetical protein
OG_09593	LG3211_2634	LG3211_2634; hypothetical protein
OG_09594	LG3211_2638	LG3211_2638; hypothetical protein
OG_09595	LG3211_2642	LG3211_2642; hypothetical protein
OG_09596	LG3211_2647	LG3211_2647; hypothetical protein
OG_09597	LG3211_2665	LG3211_2665; hypothetical protein
OG_09598	LG3211_2676	LG3211_2676; hypothetical protein
OG_09599	LG3211_2685	LG3211_2685; NAD binding domain of 6-phosphogluconate dehydrogenase
OG_09600	LG3211_2686	LG3211_2686; bacterial regulatory helix-turn-helix , lysR family protein
OG_09601	LG3211_2696	LG3211_2696; hypothetical protein
OG_09602	LG3211_2716	LG3211_2716; conserved hypothetical protein
OG_09603	LG3211_2737	LG3211_2737; hypothetical protein
OG_09604	LG3211_2738	LG3211_2738; hypothetical protein
OG_09605	LG3211_2740	LG3211_2740; hypothetical protein
OG_09606	LG3211_2746	LG3211_2746; hypothetical protein
OG_09607	LG3211_2754	LG3211_2754; hypothetical protein

OG_07271	LEC3_1486	hypothetical protein
OG_07272	LEC3_1487	conserved hypothetical protein
OG_07273	LEC3_1519	hypothetical protein
OG_07274	LEC3_1526	hypothetical protein
OG_07275	LEC3_1534	conserved hypothetical protein
OG_07276	LEC3_1535	glutathione-dependent formaldehyde-activating protein
OG_07277	LEC3_1536	hypothetical protein
OG_07278	LEC3_1541	hypothetical protein
OG_07279	LEC3_1545	hypothetical protein
OG_07280	LEC3_1550	siderophore-interacting protein
OG_07281	LEC3_1553	hypothetical protein
OG_07282	LEC3_1559	hypothetical protein
OG_07283	LEC3_1564	hypothetical protein
OG_07284	LEC3_1567	hypothetical protein
OG_07285	LEC3_1568	conserved hypothetical protein
OG_07286	LEC3_1607	hypothetical protein
OG_07287	LEC3_1613	hypothetical protein
OG_07288	LEC3_1620	hypothetical protein
OG_07289	LEC3_1626	lipoprotein
OG_07290	LEC3_1630	hypothetical protein
OG_07291	LEC3_1633	hypothetical protein
OG_07292	LEC3_1638	hypothetical protein
OG_07293	LEC3_1650	hypothetical protein
OG_07294	LEC3_1690	conserved hypothetical protein
OG_07295	LEC3_1706	hypothetical protein
OG_07296	LEC3_1725	hypothetical protein
OG_07297	LEC3_1728	hypothetical protein
OG_07298	LEC3_1729	hypothetical protein
OG_07299	LEC3_1731	hypothetical protein
OG_07300	LEC3_1732	hypothetical protein
OG_07301	LEC3_1737	conserved hypothetical protein
OG_07302	LEC3_1741	conserved hypothetical protein
OG_07303	LEC3_1754	hypothetical protein
OG_07304	LEC3_1755	peptidase, S41 family
OG_07305	LEC3_1763	hypothetical protein
OG_07306	LEC3_1766	hypothetical protein
OG_07307	LEC3_1770	hypothetical protein
OG_07308	LEC3_1774	hypothetical protein
OG_07309	LEC3_1779	hypothetical protein
OG_07310	LEC3_1787	hypothetical protein
OG_07311	LEC3_1790	OmpA/MotB family protein
OG_07312	LEC3_1792	conserved hypothetical protein
OG_07313	LEC3_1793	hypothetical protein
OG_07314	LEC3_1796	hypothetical protein
OG_07315	LEC3_1798	3-demethylubiquinone-9 3-methyltransferase domain protein
OG_07316	LEC3_1800	hypothetical protein
OG_07317	LEC3_1801	hypothetical protein
OG_07318	LEC3_1805	hypothetical protein
OG_07319	LEC3_1808	hypothetical protein
OG_07320	LEC3_1815	hypothetical protein
OG_07321	LEC3_1826	hypothetical protein
OG_07322	LEC3_1828	cytidine and deoxycytidylate deaminase zinc-binding region
OG_07323	LEC3_1833	hypothetical protein
OG_07324	LEC3_1848	condensation/amino acid adenylation/thioesterase domain protein
OG_07325	LEC3_1853	ATP-dependent DNA helicase
OG_07326	LEC3_1855	hypothetical protein
OG_07327	LEC3_1861	protein Ypar24
OG_07328	LEC3_1862	hypothetical protein
OG_07329	LEC3_1864	hypothetical protein
OG_07330	LEC3_1880	conserved hypothetical protein
OG_07331	LEC3_1881	hypothetical protein
OG_07332	LEC3_1884	hypothetical protein
OG_07333	LEC3_1885	malonyl CoA-acyl carrier protein transacylase
OG_07334	LEC3_1886	hypothetical protein
OG_07335	LEC3_1888	hypothetical protein
OG_07336	LEC3_1889	hypothetical protein
OG_07337	LEC3_1890	hypothetical protein
OG_07338	LEC3_1892	hypothetical protein
OG_07339	LEC3_1916	lipoprotein
OG_07340	LEC3_1919	ubiquinone biosynthesis protein COQ7
OG_07341	LEC3_1920	hypothetical protein

OG_09608	LG3211_2756	LG3211_2756; hypothetical protein
OG_09609	LG3211_2757	LG3211_2757; hypothetical protein
OG_09610	LG3211_2761	LG3211_2761; nudix-type nucleoside diphosphatase, YffH/AdpP family prot
OG_09611	LG3211_2798	LG3211_2798; hypothetical protein
OG_09612	LG3211_2804	LG3211_2804; conserved hypothetical protein
OG_09613	LG3211_2813	LG3211_2813; hypothetical protein
OG_09614	LG3211_2843	LG3211_2843; hypothetical protein
OG_09615	LG3211_2876	LG3211_2876; hypothetical protein
OG_09616	LG3211_2884	LG3211_2884; hypothetical protein
OG_09617	LG3211_2914	LG3211_2914; hypothetical protein
OG_09618	LG3211_2918	LG3211_2918; hypothetical protein
OG_09619	LG3211_2923	LG3211_2923; hypothetical protein
OG_09620	LG3211_2936	LG3211_2936; hypothetical protein
OG_09621	LG3211_2945	LG3211_2945; conserved hypothetical protein
OG_09622	LG3211_2961	LG3211_2961; bacterial regulatory helix-turn-helix, AraC family protein
OG_09623	LG3211_2962	fadD; long-chain fatty-acid-CoA ligase domain protein
OG_09624	LG3211_2966	LG3211_2966; transcriptional regulator, PadR family
OG_09625	LG3211_2967	LG3211_2967; conserved hypothetical protein
OG_09626	LG3211_2968	LG3211_2968; ABC transporter family protein
OG_09627	LG3211_2969	LG3211_2969; ABC-2 type transporter family protein
OG_09628	LG3211_2971	LG3211_2971; hypothetical protein
OG_09629	LG3211_2972	LG3211_2972; bacterial regulatory , arsR family protein
OG_09630	LG3211_2974	LG3211_2974; hypothetical protein
OG_09631	LG3211_2977	LG3211_2977; conserved hypothetical protein
OG_09632	LG3211_2982	LG3211_2982; HTH-like domain protein
OG_09633	LG3211_3016	LG3211_3016; subtilase family protein
OG_09634	LG3211_3021	LG3211_3021; bacterial regulatory helix-turn-helix , lysR family protein
OG_09635	LG3211_3022	LG3211_3022; snoaL-like polyketide cyclase family protein
OG_09636	LG3211_3028	LG3211_3028; proteobacterial lipase chaperone family protein
OG_09637	LG3211_3029	lipL; lactonizing lipase
OG_09638	LG3211_3030	LG3211_3030; hypothetical protein
OG_09639	LG3211_3031	LG3211_3031; bacterial regulatory helix-turn-helix, AraC family protein
OG_09640	LG3211_3050	LG3211_3050; hypothetical protein
OG_09641	LG3211_3063	LG3211_3063; hypothetical protein
OG_09642	LG3211_3070	LG3211_3070; hypothetical protein
OG_09643	LG3211_3071	LG3211_3071; hypothetical protein
OG_09644	LG3211_3079	LG3211_3079; WD40-like Beta Propeller Repeat family protein
OG_09645	LG3211_3080	LG3211_3080; hypothetical protein
OG_09646	LG3211_3104	LG3211_3104; histone H1-like nucleoHC2 family protein
OG_09647	LG3211_3149	LG3211_3149; hypothetical protein
OG_09648	LG3211_3151	LG3211_3151; hypothetical protein
OG_09649	LG3211_3164	LG3211_3164; biphenyl-2,3-diol 1,2-dioxygenase III-related protein
OG_09650	LG3211_3172	LG3211_3172; hypothetical protein
OG_09651	LG3211_3174	LG3211_3174; hypothetical protein
OG_09652	LG3211_3183	LG3211_3183; hypothetical protein
OG_09653	LG3211_3202	LG3211_3202; hypothetical protein
OG_09654	LG3211_3204	LG3211_3204; hypothetical protein
OG_09655	LG3211_3207	LG3211_3207; hypothetical protein
OG_09656	LG3211_3209	LG3211_3209; amino acid adenylation domain protein
OG_09657	LG3211_3216	LG3211_3216; hypothetical protein
OG_09658	LG3211_3215	LG3211_3215; hypothetical protein
OG_09659	LG3211_3217	LG3211_3217; hypothetical protein
OG_09660	LG3211_3225	LG3211_3225; hypothetical protein
OG_09661	LG3211_3231	LG3211_3231; hypothetical protein
OG_09662	LG3211_3234	LG3211_3234; CAAX protease self-immunity family protein
OG_09663	LG3211_3241	LG3211_3241; hypothetical protein
OG_09664	LG3211_3247	LG3211_3247; hypothetical protein
OG_09665	LG3211_3262	LG3211_3262; hypothetical protein
OG_09666	LG3211_3265	LG3211_3265; hypothetical protein
OG_09667	LG3211_3298	LG3211_3298; hypothetical protein
OG_09668	LG3211_3299	LG3211_3299; hypothetical protein
OG_09669	LG3211_3306	LG3211_3306; hypothetical protein
OG_09670	LG3211_3320	LG3211_3320; hypothetical protein
OG_09671	LG3211_3353	LG3211_3353; hypothetical protein
OG_09672	LG3211_3366	LG3211_3366; hypothetical protein
OG_09673	LG3211_3367	LG3211_3367; hypothetical protein
OG_09674	LG3211_3383	LG3211_3383; hypothetical protein
OG_09675	LG3211_3385	LG3211_3385; hypothetical protein
OG_09676	LG3211_3387	LG3211_3387; hypothetical protein
OG_09677	LG3211_3388	LG3211_3388; hypothetical protein
OG_09678	LG3211_3389	LG3211_3389; hypothetical protein

OG_07342	LEC3_1922	hypothetical protein
OG_07343	LEC3_1931	hypothetical protein
OG_07344	LEC3_1933	hypothetical protein
OG_07345	LEC3_1934	hypothetical protein
OG_07346	LEC3_1935	hypothetical protein
OG_07347	LEC3_1937	conserved hypothetical protein
OG_07348	LEC3_1939	lipoprotein
OG_07349	LEC3_1941	hypothetical protein
OG_07350	LEC3_1943	hypothetical protein
OG_07351	LEC3_1944	hypothetical protein
OG_07352	LEC3_1946	hypothetical protein
OG_07353	LEC3_1951	hypothetical protein
OG_07354	LEC3_1958	hypothetical protein
OG_07355	LEC3_1962	hypothetical protein
OG_07356	LEC3_1963	dnaJ domain
OG_07357	LEC3_1968	hypothetical protein
OG_07358	LEC3_1969	possibly something SEQUENCING GAP
OG_07359	LEC3_1970	lipoprotein
OG_07360	LEC3_1977	oxidoreductase, 2OG-Fe(II) oxygenase family protein
OG_07361	LEC3_1985	ATP-dependent DNA helicase
OG_07362	LEC3_1988	lipase family
OG_07363	LEC3_1990	hypothetical protein
OG_07364	LEC3_1994	hypothetical protein
OG_07365	LEC3_1997	methyl-accepting chemotaxis protein
OG_07366	LEC3_1998	hypothetical protein
OG_07367	LEC3_1999	hypothetical protein
OG_07368	LEC3_2004	conserved hypothetical protein
OG_07369	LEC3_2005	conserved hypothetical protein
OG_07370	LEC3_2007	hypothetical protein
OG_07371	LEC3_2010	hypothetical protein
OG_07372	LEC3_2012	conserved hypothetical protein
OG_07373	LEC3_2013	hypothetical protein
OG_07374	LEC3_2020	YcII domain protein
OG_07375	LEC3_2021	hypothetical protein
OG_07376	LEC3_2022	hypothetical protein
OG_07377	LEC3_2024	hypothetical protein
OG_07378	LEC3_2031	conserved hypothetical protein
OG_07379	LEC3_2041	hypothetical protein
OG_07380	LEC3_2045	hypothetical protein
OG_07381	LEC3_2047	hypothetical protein
OG_07382	LEC3_2050	hypothetical protein
OG_07383	LEC3_2056	putative peptidoglycan binding domain
OG_07384	LEC3_2057	conserved hypothetical protein
OG_07385	LEC3_2061	hypothetical protein
OG_07386	LEC3_2062	hypothetical protein
OG_07387	LEC3_2074	hypothetical protein
OG_07388	LEC3_2095	lipoprotein
OG_07389	LEC3_2098	4'-phosphopantetheinyl transferase
OG_07390	LEC3_2100	NAD-dependent epimerase
OG_07391	LEC3_2101	aminotransferase, classes I, II, and III/beta-eliminating lyase
OG_07392	LEC3_2102	acyl carrier protein
OG_07393	LEC3_2105	ABC transporter, ATP-binding protein
OG_07394	LEC3_2106	ABC transporter, permease protein, ABC-2 family
OG_07395	LEC3_2107	diaminobutyrate--2-oxoglutarate aminotransferase
OG_07396	LEC3_2108	conserved hypothetical protein
OG_07397	LEC3_2112	transcriptional regulator, LuxR family
OG_07398	LEC3_2113	hypothetical protein
OG_07399	LEC3_2116	hypothetical protein
OG_07400	LEC3_2125	hypothetical protein
OG_07401	LEC3_2131	hypothetical protein
OG_07402	LEC3_2133	hypothetical protein
OG_07403	LEC3_2135	conserved hypothetical protein
OG_07404	LEC3_2151	hypothetical protein
OG_07405	LEC3_2155	hypothetical protein
OG_07406	LEC3_2173	hypothetical protein
OG_07407	LEC3_2181	conserved domain protein
OG_07408	LEC3_2184	conserved hypothetical protein
OG_07409	LEC3_2185	conserved hypothetical protein
OG_07410	LEC3_2189	hypothetical protein
OG_07411	LEC3_2197	hypothetical protein
OG_07412	LEC3_2198	hypothetical protein

OG_09679	LG3211_3395	LG3211_3395; hypothetical protein	OG_07413	LEC3_2215	hypothetical protein
OG_09680	LG3211_3400	LG3211_3400; hypothetical protein	OG_07414	LEC3_2217	hypothetical protein
OG_09681	LG3211_3401	LG3211_3401; hypothetical protein	OG_07415	LEC3_2243	hypothetical protein
OG_09682	LG3211_3404	LG3211_3404; hypothetical protein	OG_07416	LEC3_2256	hypothetical protein
OG_09683	LG3211_3408	LG3211_3408; hypothetical protein	OG_07417	LEC3_2257	hypothetical protein
OG_09684	LG3211_3409	LG3211_3409; hypothetical protein	OG_07418	LEC3_2260	hypothetical protein
OG_09685	LG3211_3420	LG3211_3420; hypothetical protein	OG_07419	LEC3_2275	hypothetical protein
OG_09686	LG3211_3422	LG3211_3422; hypothetical protein	OG_07420	LEC3_2277	hypothetical protein
OG_09687	LG3211_3423	LG3211_3423; hypothetical protein	OG_07421	LEC3_2279	hypothetical protein
OG_09688	LG3211_3447	LG3211_3447; putative methyltransferase domain protein	OG_07422	LEC3_2280	hypothetical protein
OG_09689	LG3211_3448	LG3211_3448; hypothetical protein	OG_07423	LEC3_2285	hypothetical protein
OG_09690	LG3211_3449	LG3211_3449; hypothetical protein	OG_07424	LEC3_2287	SWIM zinc finger
OG_09691	LG3211_3450	LG3211_3450; hypothetical protein	OG_07425	LEC3_2288	conserved hypothetical protein
OG_09692	LG3211_3460	LG3211_3460; hypothetical protein	OG_07426	LEC3_2295	hypothetical protein
OG_09693	LG3211_3461	LG3211_3461; hypothetical protein	OG_07427	LEC3_2307	hypothetical protein
OG_09694	LG3211_3462	LG3211_3462; hypothetical protein	OG_07428	LEC3_2308	hypothetical protein
OG_09695	LG3211_3463	LG3211_3463; hypothetical protein	OG_07429	LEC3_2316	hypothetical protein
OG_09696	LG3211_3481	LG3211_3481; hypothetical protein	OG_07430	LEC3_2318	conserved hypothetical protein
OG_09697	LG3211_3485	LG3211_3485; hypothetical protein	OG_07431	LEC3_2322	hypothetical protein
OG_09698	LG3211_3492	LG3211_3492; hypothetical protein	OG_07432	LEC3_2325	hypothetical protein
OG_09699	LG3211_3493	LG3211_3493; hypothetical protein	OG_07433	LEC3_2327	hypothetical protein
OG_09700	LG3211_3494	LG3211_3494; hypothetical protein	OG_07434	LEC3_2336	hypothetical protein
OG_09701	LG3211_3495	LG3211_3495; hypothetical protein	OG_07435	LEC3_2337	hypothetical protein
OG_09702	LG3211_3498	LG3211_3498; hypothetical protein	OG_07436	LEC3_2355	hypothetical protein
OG_09703	LG3211_3500	LG3211_3500; hypothetical protein	OG_07437	LEC3_2357	hypothetical protein
OG_09704	LG3211_3499	LG3211_3499; hypothetical protein	OG_07438	LEC3_2380	hypothetical protein
OG_09705	LG3211_3504	secF; protein-export membrane protein SecF	OG_07439	LEC3_2383	hypothetical protein
OG_09706	LG3211_3505	LG3211_3505; hypothetical protein	OG_07440	LEC3_2384	lipoprotein
OG_09707	LG3211_3507	LG3211_3507; hypothetical protein	OG_07441	LEC3_2390	hypothetical protein
OG_09708	LG3211_3521	LG3211_3521; hypothetical protein	OG_07442	LEC3_2394	transcriptional regulator, Rrf2 family
OG_09709	LG3211_3536	LG3211_3536; hypothetical protein	OG_07443	LEC3_2396	flavohemoprotein
OG_09710	LG3211_3539	LG3211_3539; hypothetical protein	OG_07444	LEC3_2398	conserved hypothetical protein
OG_09711	LG3211_3542	LG3211_3542; hypothetical protein	OG_07445	LEC3_2402	hypothetical protein
OG_09712	LG3211_3545	LG3211_3545; hypothetical protein	OG_07446	LEC3_2408	flavin reductase like domain protein
OG_09713	LG3211_3557	LG3211_3557; hypothetical protein	OG_07447	LEC3_2416	hypothetical protein
OG_09714	LG3211_3558	LG3211_3558; AAA domain protein	OG_07448	LEC3_2417	TonB-dependent siderophore receptor
OG_09715	LG3211_3560	LG3211_3560; hypothetical protein	OG_07449	LEC3_2419	TSPO protein
OG_09716	LG3211_3573	LG3211_3573; phage virion morphogenesis protein	OG_07450	LEC3_2420	conserved hypothetical protein
OG_09717	LG3211_3574	LG3211_3574; restriction endonuclease family protein	OG_07451	LEC3_2421	PepSY-associated TM helix
OG_09718	LG3211_3577	LG3211_3577; hypothetical protein	OG_07452	LEC3_2422	esterase
OG_09719	LG3211_3597	LG3211_3597; alpha/beta hydrolase family protein	OG_07453	LEC3_2426	nipsnap family protein
OG_09720	LG3211_3610	LG3211_3610; hypothetical protein	OG_07454	LEC3_2434	transcriptional regulator, AraC family
OG_09721	LG3211_3613	LG3211_3613; hypothetical protein	OG_07455	LEC3_2441	hypothetical protein
OG_09722	LG3211_3630	LG3211_3630; hypothetical protein	OG_07456	LEC3_2446	hypothetical protein
OG_09723	LG3211_3636	LG3211_3636; hypothetical protein	OG_07457	LEC3_2454	hypothetical protein
OG_09724	LG3211_3638	LG3211_3638; prepilin-type N-terminal cleavage/methylation domain prote	OG_07458	LEC3_2456	hypothetical protein
OG_09725	LG3211_3639	LG3211_3639; hypothetical protein	OG_07459	LEC3_2474	hypothetical protein
OG_09726	LG3211_3644	LG3211_3644; hypothetical protein	OG_07460	LEC3_2477	conserved hypothetical protein
OG_09727	LG3211_3650	LG3211_3650; hypothetical protein	OG_07461	LEC3_2478	conserved hypothetical protein
OG_09728	LG3211_3666	LG3211_3666; hypothetical protein	OG_07462	LEC3_2480	hypothetical protein
OG_09729	LG3211_3680	LG3211_3680; ankyrin repeat family protein	OG_07463	LEC3_2488	methyltransferase
OG_09730	LG3211_3681	LG3211_3681; HEAT repeats family protein	OG_07464	LEC3_2489	hemolysin activator protein
OG_09731	LG3211_3682	LG3211_3682; anti-sigma-K factor rskA family protein	OG_07465	LEC3_2490	conserved hypothetical protein
OG_09732	LG3211_3683	LG3211_3683; RNA polymerase sigma factor, sigma-70 family protein	OG_07466	LEC3_2491	hypothetical protein
OG_09733	LG3211_3685	LG3211_3685; hypothetical protein	OG_07467	LEC3_2492	sulphotransferase
OG_09734	LG3211_3686	Gatm; glycine amidinotransferase, mitochondrial	OG_07468	LEC3_2494	polyketide synthase pksM
OG_09735	LG3211_3689	dltA; D-alanine--poly(phosphoribitol) ligase, subunit 1	OG_07469	LEC3_2495	beta-ketoacyl synthase/short chain dehydrogenase/methyltransferase dom
OG_09736	LG3211_3690	dltA; D-alanine--poly(phosphoribitol) ligase, subunit 1	OG_07470	LEC3_2496	hypothetical protein
OG_09737	LG3211_3691	dltA; D-alanine--poly(phosphoribitol) ligase, subunit 1	OG_07471	LEC3_2497	mbtH family protein
OG_09738	LG3211_3692	dltA; D-alanine--poly(phosphoribitol) ligase, subunit 1	OG_07472	LEC3_2499	conserved hypothetical protein
OG_09739	LG3211_3700	LG3211_3700; integrase core domain protein	OG_07473	LEC3_2501	polysaccharide deacetylase
OG_09740	LG3211_3702	LG3211_3702; conserved repeat domain protein	OG_07474	LEC3_2502	ABC transporter, ATP-binding protein
OG_09741	LG3211_3713	LG3211_3713; hypothetical protein	OG_07475	LEC3_2503	aryl-alcohol dehydrogenase
OG_09742	LG3211_3719	LG3211_3719; conserved hypothetical protein	OG_07476	LEC3_2504	efflux ABC transporter, permease protein
OG_09743	LG3211_3724	LG3211_3724; hypothetical protein	OG_07477	LEC3_2505	efflux ABC transporter, permease protein
OG_09744	LG3211_3771	LG3211_3771; hypothetical protein	OG_07478	LEC3_2506	conserved hypothetical protein
OG_09745	LG3211_3781	LG3211_3781; hypothetical protein	OG_07479	LEC3_2507	DinB family protein
OG_09746	LG3211_3792	LG3211_3792; hypothetical protein	OG_07480	LEC3_2509	flavin-dependent oxidoreductase
OG_09747	LG3211_3797	LG3211_3797; hypothetical protein	OG_07481	LEC3_2510	amidohydrolase family protein
OG_09748	LG3211_3807	LG3211_3807; hypothetical protein	OG_07482	LEC3_2512	Nodulation protein nolO
OG_09749	LG3211_3814	LG3211_3814; hypothetical protein	OG_07483	LEC3_2514	hypothetical protein

OG_09750	LG3211_3818	LG3211_3818; hypothetical protein
OG_09751	LG3211_3820	LG3211_3820; hypothetical protein
OG_09752	LG3211_3825	LG3211_3825; short chain dehydrogenase family protein
OG_09753	LG3211_3828	LG3211_3828; hypothetical protein
OG_09754	LG3211_3830	LG3211_3830; hypothetical protein
OG_09755	LG3211_3831	LG3211_3831; hypothetical protein
OG_09756	LG3211_3846	LG3211_3846; conserved hypothetical protein
OG_09757	LG3211_3854	LG3211_3854; hypothetical protein
OG_09758	LG3211_3855	LG3211_3855; tellurite resistance TehB family protein
OG_09759	LG3211_3860	LG3211_3860; hypothetical protein
OG_09760	LG3211_3861	LG3211_3861; hypothetical protein
OG_09761	LG3211_3864	LG3211_3864; hypothetical protein
OG_09762	LG3211_3865	LG3211_3865; hypothetical protein
OG_09763	LG3211_3866	LG3211_3866; conserved hypothetical protein
OG_09764	LG3211_3869	LG3211_3869; hypothetical protein
OG_09765	LG3211_3873	LG3211_3873; antibiotic biosynthesis monooxygenase family protein
OG_09766	LG3211_3884	LG3211_3884; hypothetical protein
OG_09767	LG3211_3898	LG3211_3898; AMP-binding enzyme family protein
OG_09768	LG3211_3924	LG3211_3924; histidine kinase-, DNA gyrase B-, and HSP90-like ATPase fam
OG_09769	LG3211_3929	LG3211_3929; hypothetical protein
OG_09770	LG3211_3930	LG3211_3930; hypothetical protein
OG_09771	LG3211_3943	LG3211_3943; por secretion system C-terminal sorting domain protein
OG_09772	LG3211_3944	LG3211_3944; conserved hypothetical protein
OG_09773	LG3211_3945	LG3211_3945; hypothetical protein
OG_09774	LG3211_3952	LG3211_3952; hypothetical protein
OG_09775	LG3211_3953	LG3211_3953; hypothetical protein
OG_09776	LG3211_3956	LG3211_3956; hypothetical protein
OG_09777	LG3211_3968	LG3211_3968; peptidase M23 family protein
OG_09778	LG3211_3969	LG3211_3969; hypothetical protein
OG_09779	LG3211_3998	LG3211_3998; hypothetical protein
OG_09780	LG3211_4009	LG3211_4009; hypothetical protein
OG_09781	LG3211_4031	LG3211_4031; hypothetical protein
OG_09782	LG3211_4032	LG3211_4032; hypothetical protein
OG_09783	LG3211_4043	LG3211_4043; hypothetical protein
OG_09784	LG3211_4050	LG3211_4050; hypothetical protein
OG_09785	LG3211_4051	LG3211_4051; gtrA-like family protein
OG_09786	LG3211_4053	LG3211_4053; polysaccharide biosynthesis family protein
OG_09787	LG3211_4054	LG3211_4054; short chain dehydrogenase family protein
OG_09788	LG3211_4055	LG3211_4055; FAD binding domain protein
OG_09789	LG3211_4056	LG3211_4056; ubiA prenyltransferase family protein
OG_09790	LG3211_4057	LG3211_4057; glycosyl transferases group 1 family protein
OG_09791	LG3211_4058	LG3211_4058; conserved hypothetical protein
OG_09792	LG3211_4069	LG3211_4069; hypothetical protein
OG_09793	LG3211_4125	LG3211_4125; conserved hypothetical protein
OG_09794	LG3211_4140	LG3211_4140; hypothetical protein
OG_09795	LG3211_4150	LG3211_4150; hypothetical protein
OG_09796	LG3211_4157	LG3211_4157; hypothetical protein
OG_09797	LG3211_4160	LG3211_4160; hypothetical protein
OG_09798	LG3211_4172	LG3211_4172; hypothetical protein
OG_09799	LG3211_4174	LG3211_4174; NUDIX domain protein
OG_09800	LG3211_4176	LG3211_4176; methyltransferase domain protein
OG_09801	LG3211_4177	LG3211_4177; UDP-N-acetylglucosamine 4,6-dehydratase
OG_09802	LG3211_4178	LG3211_4178; glycosyl transferase 2 family protein
OG_09803	LG3211_4179	LG3211_4179; NAD dependent epimerase/dehydratase family protein
OG_09804	LG3211_4180	LG3211_4180; mobA-like NTP transferase domain protein
OG_09805	LG3211_4181	LG3211_4181; bacterial membrane YfhO family protein
OG_09806	LG3211_4192	LG3211_4192; hypothetical protein
OG_09807	LG3211_4200	LG3211_4200; hypothetical protein
OG_09808	LG3211_4223	LG3211_4223; hypothetical protein
OG_09809	LG3211_4243	LG3211_4243; hypothetical protein
OG_09810	LG3211_4279	LG3211_4279; hypothetical protein
OG_09811	LG3211_4280	LG3211_4280; hypothetical protein
OG_09812	LG3211_4282	LG3211_4282; hypothetical protein
OG_09813	LG3211_4292	LG3211_4292; hypothetical protein
OG_09814	LG3211_4296	LG3211_4296; hypothetical protein
OG_09815	LG3211_4300	LG3211_4300; hypothetical protein
OG_09816	LG3211_4302	LG3211_4302; hypothetical protein
OG_09817	LG3211_4313	cel8A; endoglucanase domain protein
OG_09818	LG3211_4314	LG3211_4314; hypothetical protein
OG_09819	LG3211_4316	LG3211_4316; phospholipase domain protein
OG_09820	LG3211_4317	LG3211_4317; putative phospholipase A1

OG_07484	LEC3_2516	methyltransferase
OG_07485	LEC3_2519	luciferase-like monooxygenase
OG_07486	LEC3_2520	conserved hypothetical protein
OG_07487	LEC3_2521	hypothetical protein
OG_07488	LEC3_2522	acyl-CoA ligase
OG_07489	LEC3_2523	beta-glucosidase
OG_07490	LEC3_2524	hypothetical protein
OG_07491	LEC3_2525	oxidoreductase
OG_07492	LEC3_2526	hypothetical protein
OG_07493	LEC3_2527	membrane protein
OG_07494	LEC3_2528	membrane protein
OG_07495	LEC3_2529	ABC transporter, ATP-binding protein
OG_07496	LEC3_2530	radical SAM domain protein
OG_07497	LEC3_2531	conserved hypothetical protein
OG_07498	LEC3_2538	hypothetical protein
OG_07499	LEC3_2541	TonB system transport protein exbD
OG_07500	LEC3_2542	TonB-system energizer exbB
OG_07501	LEC3_2546	conserved hypothetical protein
OG_07502	LEC3_2549	TonB-dependent receptor
OG_07503	LEC3_2552	TonB-dependent siderophore receptor
OG_07504	LEC3_2553	hypothetical protein
OG_07505	LEC3_2554	PepSY-associated TM helix domain protein
OG_07506	LEC3_2555	thioesterase domain
OG_07507	LEC3_2557	amino acid adenylation domain protein
OG_07508	LEC3_2558	amino acid adenylation domain protein
OG_07509	LEC3_2559	amino acid adenylation domain protein
OG_07510	LEC3_2560	amino acid adenylation domain protein
OG_07511	LEC3_2563	hypothetical protein
OG_07512	LEC3_2568	outer membrane efflux protein
OG_07513	LEC3_2569	hypothetical protein
OG_07514	LEC3_2570	conserved hypothetical protein
OG_07515	LEC3_2571	hypothetical protein
OG_07516	LEC3_2572	type VI secretion protein, VC_A0107 family
OG_07517	LEC3_2573	type VI secretion protein, EvpB/VC_A0108 family
OG_07518	LEC3_2574	conserved hypothetical protein
OG_07519	LEC3_2575	ImpE/SciE family protein
OG_07520	LEC3_2576	type VI secretion system lysozyme-related protein
OG_07521	LEC3_2577	type VI secretion protein, VC_A0110 family
OG_07522	LEC3_2578	type VI secretion protein, VC_A0111 family
OG_07523	LEC3_2580	hypothetical protein
OG_07524	LEC3_2581	type VI secretion system Vgr family protein
OG_07525	LEC3_2582	conserved hypothetical protein
OG_07526	LEC3_2583	type VI secretion system FHA domain protein
OG_07527	LEC3_2587	type VI secretion-associated protein, BMA_A0400 family
OG_07528	LEC3_2588	phosphoprotein phosphatase
OG_07529	LEC3_2589	serine/threonine kinase family protein
OG_07530	LEC3_2590	conserved hypothetical protein
OG_07531	LEC3_2593	His Kinase A
OG_07532	LEC3_2594	regulatory protein virG
OG_07533	LEC3_2595	hypothetical protein
OG_07534	LEC3_2596	conserved domain protein
OG_07535	LEC3_2602	hypothetical protein
OG_07536	LEC3_2615	hypothetical protein
OG_07537	LEC3_2616	hypothetical protein
OG_07538	LEC3_2626	hypothetical protein
OG_07539	LEC3_2651	hypothetical protein
OG_07540	LEC3_2671	hypothetical protein
OG_07541	LEC3_2682	hypothetical protein
OG_07542	LEC3_2683	hypothetical protein
OG_07543	LEC3_2687	hypothetical protein
OG_07544	LEC3_2699	hypothetical protein
OG_07545	LEC3_2721	hypothetical protein
OG_07546	LEC3_2736	hypothetical protein
OG_07547	LEC3_2748	hypothetical protein
OG_07548	LEC3_2750	hypothetical protein
OG_07549	LEC3_2751	conserved hypothetical protein
OG_07550	LEC3_2763	hypothetical protein
OG_07551	LEC3_2772	transcriptional regulator, TetR family
OG_07552	LEC3_2774	hypothetical protein
OG_07553	LEC3_2783	hypothetical protein
OG_07554	LEC3_2805	hypothetical protein

OG_09821	LG3211_4318	LG3211_4318; conserved hypothetical protein
OG_09822	LG3211_4320	LG3211_4320; hypothetical protein
OG_09823	LG3211_4329	LG3211_4329; hypothetical protein
OG_09824	LG3211_4333	LG3211_4333; pentapeptide repeats family protein
OG_09825	LG3211_4353	LG3211_4353; conserved hypothetical protein
OG_09826	LG3211_4355	LG3211_4355; hypothetical protein
OG_09827	LG3211_4356	LG3211_4356; hypothetical protein
OG_09828	LG3211_4359	LG3211_4359; hypothetical protein
OG_09829	LG3211_4364	LG3211_4364; hypothetical protein
OG_09830	LG3211_4365	LG3211_4365; hypothetical protein
OG_09831	LG3211_4368	LG3211_4368; AAA domain family protein
OG_09832	LG3211_4370	LG3211_4370; hypothetical protein
OG_09833	LG3211_4380	LG3211_4380; hypothetical protein
OG_09834	LG3211_4383	acrA; acrA Membrane Fusion Protein
OG_09835	LG3211_4384	LG3211_4384; acyltransferase family protein
OG_09836	LG3211_4385	LG3211_4385; hypothetical protein
OG_09837	LG3211_4387	LG3211_4387; hypothetical protein
OG_09838	LG3211_4391	dltA; D-alanine--poly(phosphoribitol) ligase, subunit 1
OG_09839	LG3211_4393	LG3211_4393; hypothetical protein
OG_09840	LG3211_4394	LG3211_4394; hypothetical protein
OG_09841	LG3211_4396	LG3211_4396; conserved hypothetical protein
OG_09842	LG3211_4397	ubiG; 3-demethylubiquinone-9 3-O-methyltransferase
OG_09843	LG3211_4403	LG3211_4403; hypothetical protein
OG_09844	LG3211_4410	LG3211_4410; hypothetical protein
OG_09845	LG3211_4414	LG3211_4414; hypothetical protein
OG_09846	LG3211_4416	LG3211_4416; hypothetical protein
OG_09847	LG3211_4422	LG3211_4422; hypothetical protein
OG_09848	LG3211_4423	LG3211_4423; hypothetical protein
OG_09849	LG3211_4435	LG3211_4435; hypothetical protein
OG_09850	LG3211_4441	LG3211_4441; hypothetical protein
OG_09851	LG3211_4446	LG3211_4446; hypothetical protein
OG_09852	LG3211_4448	LG3211_4448; hypothetical protein
OG_09853	LG3211_4453	LG3211_4453; hypothetical protein
OG_09854	LG3211_4458	LG3211_4458; hypothetical protein
OG_09855	LG3211_4464	LG3211_4464; hypothetical protein
OG_09856	LG3211_4470	LG3211_4470; hypothetical protein
OG_09857	LG3211_4488	LG3211_4488; hypothetical protein
OG_09858	LG3211_4525	LG3211_4525; hypothetical protein
OG_09859	LG3211_4533	LG3211_4533; hypothetical protein
OG_09860	LG3211_4536	LG3211_4536; hypothetical protein
OG_09861	LG3211_4539	LG3211_4539; hypothetical protein
OG_09862	LG3211_4561	LG3211_4561; hypothetical protein
OG_09863	LG3211_4563	LG3211_4563; hypothetical protein
OG_09864	LG3211_4577	LG3211_4577; hypothetical protein
OG_09865	LG3211_4612	LG3211_4612; hypothetical protein
OG_09866	LG3211_4619	LG3211_4619; hypothetical protein
OG_09867	LG3211_4625	LG3211_4625; hypothetical protein
OG_09868	LG3211_4631	LG3211_4631; hypothetical protein
OG_09869	LG3211_4632	LG3211_4632; hypothetical protein
OG_09870	LG3211_4637	LG3211_4637; bacterial transferase hexapeptide family protein
OG_09871	LG3211_4649	LG3211_4649; hypothetical protein
OG_09872	LG3211_4656	LG3211_4656; hypothetical protein
OG_09873	LG3211_4657	LG3211_4657; secretory lipase family protein
OG_09874	LG3211_4658	LG3211_4658; hypothetical protein
OG_09875	LG3211_4659	LG3211_4659; hypothetical protein
OG_09876	LG3211_4660	LG3211_4660; short chain dehydrogenase family protein
OG_09877	LG3211_4665	LG3211_4665; bacterial regulatory, tetR family protein
OG_09878	LG3211_4670	LG3211_4670; hypothetical protein
OG_09879	LG3211_4671	LG3211_4671; hypothetical protein
OG_09880	LG3211_4685	LG3211_4685; short chain dehydrogenase family protein
OG_09881	LG3211_4686	LG3211_4686; lumazine-binding family protein
OG_09882	LG3211_4692	LG3211_4692; hypothetical protein
OG_09883	LG3211_4693	LG3211_4693; hypothetical protein
OG_09884	LG3211_4701	LG3211_4701; hypothetical protein
OG_09885	LG3211_4713	LG3211_4713; hypothetical protein
OG_09886	LG3211_4714	LG3211_4714; hypothetical protein
OG_09887	LG3211_4719	LG3211_4719; cupin domain protein
OG_09888	LG3211_4721	LG3211_4721; hypothetical protein
OG_09889	LG3211_4722	LG3211_4722; hypothetical protein
OG_09890	LG3211_4724	LG3211_4724; snoaL-like domain protein
OG_09891	LG3211_4727	LG3211_4727; hypothetical protein

OG_07555	LEC3_2810	hypothetical protein
OG_07556	LEC3_2839	conserved hypothetical protein
OG_07557	LEC3_2841	CAAX amino protease family
OG_07558	LEC3_2843	hypothetical protein
OG_07559	LEC3_2844	hypothetical protein
OG_07560	LEC3_2845	hypothetical protein
OG_07561	LEC3_2846	hypothetical protein
OG_07562	LEC3_2849	conserved hypothetical protein
OG_07563	LEC3_2853	hypothetical protein
OG_07564	LEC3_2863	lipoprotein
OG_07565	LEC3_2873	hypothetical protein
OG_07566	LEC3_2874	'Cold-shock' DNA-binding domain
OG_07567	LEC3_2882	hypothetical protein
OG_07568	LEC3_2888	hypothetical protein
OG_07569	LEC3_2890	hypothetical protein
OG_07570	LEC3_2891	ATP-binding protein
OG_07571	LEC3_2892	hypothetical protein
OG_07572	LEC3_2902	hypothetical protein
OG_07573	LEC3_2930	hypothetical protein
OG_07574	LEC3_2942	conserved hypothetical protein
OG_07575	LEC3_2943	hypothetical protein
OG_07576	LEC3_2952	hypothetical protein
OG_07577	LEC3_2954	hydrolase, alpha/beta fold family
OG_07578	LEC3_2955	hypothetical protein
OG_07579	LEC3_2956	hypothetical protein
OG_07580	LEC3_2957	conserved hypothetical protein
OG_07581	LEC3_2958	linear gramicidin synthetase subunit C
OG_07582	LEC3_2959	linear gramicidin synthetase subunit D
OG_07583	LEC3_2964	hypothetical protein
OG_07584	LEC3_2974	transcription factor
OG_07585	LEC3_2989	hypothetical protein
OG_07586	LEC3_2993	hypothetical protein
OG_07587	LEC3_2998	hypothetical protein
OG_07588	LEC3_3008	hypothetical protein
OG_07589	LEC3_3014	metal-dependent phosphohydrolase
OG_07590	LEC3_3020	hypothetical protein
OG_07591	LEC3_3021	conserved hypothetical protein
OG_07592	LEC3_3022	carbonate dehydratase
OG_07593	LEC3_3026	conserved hypothetical protein
OG_07594	LEC3_3029	hypothetical protein
OG_07595	LEC3_3037	hypothetical protein
OG_07596	LEC3_3049	conserved hypothetical protein
OG_07597	LEC3_3050	trypsin
OG_07598	LEC3_3067	oxidoreductase
OG_07599	LEC3_3070	hypothetical protein
OG_07600	LEC3_3072	hypothetical protein
OG_07601	LEC3_3074	hypothetical protein
OG_07602	LEC3_3086	hypothetical protein
OG_07603	LEC3_3089	hypothetical protein
OG_07604	LEC3_3092	hypothetical protein
OG_07605	LEC3_3096	conserved hypothetical protein
OG_07606	LEC3_3098	conserved hypothetical protein
OG_07607	LEC3_3102	hypothetical protein
OG_07608	LEC3_3107	hypothetical protein
OG_07609	LEC3_3119	hypothetical protein
OG_07610	LEC3_3123	hypothetical protein
OG_07611	LEC3_3124	hypothetical protein
OG_07612	LEC3_3126	conserved hypothetical protein
OG_07613	LEC3_3127	conserved hypothetical protein
OG_07614	LEC3_3146	hypothetical protein
OG_07615	LEC3_3148	possibly something SEQUENCING GAP
OG_07616	LEC3_3158	hypothetical protein
OG_07617	LEC3_3168	hypothetical protein
OG_07618	LEC3_3175	hypothetical protein
OG_07619	LEC3_3178	hypothetical protein
OG_07620	LEC3_3186	hypothetical protein
OG_07621	LEC3_3195	hypothetical protein
OG_07622	LEC3_3201	hypothetical protein
OG_07623	LEC3_3210	hypothetical protein
OG_07624	LEC3_3214	hypothetical protein
OG_07625	LEC3_3215	HSAF non-ribosomal peptide synthetase/polyketide synthase

OG_09892	LG3211_4728	LG3211_4728; hypothetical protein
OG_09893	LG3211_4732	LG3211_4732; hypothetical protein
OG_09894	LG3211_4740	LG3211_4740; hypothetical protein
OG_09895	LG3211_4744	LG3211_4744; conserved hypothetical protein
OG_09896	LG3211_4745	LG3211_4745; hypothetical protein
OG_09897	LG3211_4747	LG3211_4747; bacterial regulatory helix-turn-helix , lysR family protein
OG_09898	LG3211_4749	LG3211_4749; hypothetical protein
OG_09899	LG3211_4750	LG3211_4750; salicylate synthase
OG_09900	LG3211_4752	LG3211_4752; ompA family protein
OG_09901	LG3211_4753	LG3211_4753; hypothetical protein
OG_09902	LG3211_4754	LG3211_4754; hypothetical protein
OG_09903	LG3211_4769	LG3211_4769; hypothetical protein
OG_09904	LG3211_4771	LG3211_4771; hypothetical protein
OG_09905	LG3211_4781	LG3211_4781; hypothetical protein
OG_09906	LG3211_4782	LG3211_4782; ATPase associated with various cellular activities family protein
OG_09907	LG3211_4783	LG3211_4783; conserved hypothetical protein
OG_09908	LG3211_4784	LG3211_4784; hypothetical protein
OG_09909	LG3211_4785	LG3211_4785; F5/8 type C -containing domain protein
OG_09910	LG3211_4786	LG3211_4786; hypothetical protein
OG_09911	LG3211_4787	LG3211_4787; hypothetical protein
OG_09912	LG3211_4788	LG3211_4788; hypothetical protein
OG_09913	LG3211_4789	LG3211_4789; chaperone of endosialidase family protein
OG_09914	LG3211_4790	LG3211_4790; hypothetical protein
OG_09915	LG3211_4793	LG3211_4793; rhs element Vgr family protein
OG_09916	LG3211_4794	LG3211_4794; conserved hypothetical protein
OG_09917	LG3211_4795	LG3211_4795; hypothetical protein
OG_09918	LG3211_4796	LG3211_4796; conserved hypothetical phage tail region family protein
OG_09919	LG3211_4800	LG3211_4800; hypothetical protein
OG_09920	LG3211_4801	LG3211_4801; conserved hypothetical protein
OG_09921	LG3211_4803	LG3211_4803; hypothetical protein
OG_09922	LG3211_4804	mopR; mopR domain protein
OG_09923	LG3211_4805	LG3211_4805; cellulase family protein
OG_09924	LG3211_4808	LG3211_4808; alpha-L-fucosidase family protein
OG_09925	LG3211_4813	ybaR; sulfate permease domain protein
OG_09926	LG3211_4814	LG3211_4814; magnesium chelatase, subunit ChlI family protein
OG_09927	LG3211_4818	LG3211_4818; conserved hypothetical protein
OG_09928	LG3211_4819	LG3211_4819; conserved hypothetical protein
OG_09929	LG3211_4820	LG3211_4820; hypothetical protein
OG_09930	LG3211_4821	LG3211_4821; hypothetical protein
OG_09931	LG3211_4822	LG3211_4822; hypothetical protein
OG_09932	LG3211_4823	LG3211_4823; conserved hypothetical protein
OG_09933	LG3211_4825	LG3211_4825; tonB dependent receptor family protein
OG_09934	LG3211_4826	LG3211_4826; conserved hypothetical protein
OG_09935	LG3211_4828	LG3211_4828; fecR family protein
OG_09936	LG3211_4829	LG3211_4829; RNA polymerase sigma factor, sigma-70 family protein
OG_09937	LG3211_4830	cyoD; cytochrome o ubiquinol oxidase subunit IV
OG_09938	LG3211_4831	LG3211_4831; cytochrome c oxidase subunit III family protein
OG_09939	LG3211_4833	LG3211_4833; COX Aromatic Rich Motif family protein
OG_09940	LG3211_4834	LG3211_4834; outer membrane efflux family protein
OG_09941	LG3211_4835	LG3211_4835; hlyD secretion family protein
OG_09942	LG3211_4836	LG3211_4836; ABC-2 transporter family protein
OG_09943	LG3211_4837	LG3211_4837; ABC-2 type transporter family protein
OG_09944	LG3211_4838	LG3211_4838; tonB dependent receptor family protein
OG_09945	LG3211_4839	LG3211_4839; alpha/beta hydrolase family protein
OG_09946	LG3211_4840	cydB; cytochrome d ubiquinol oxidase, subunit II
OG_09947	LG3211_4842	marC; inner membrane involved in multiple antibiotic resistance domain protei
OG_09948	LG3211_4843	LG3211_4843; hypothetical protein
OG_09949	LG3211_4844	LG3211_4844; putative pqqE protein
OG_09950	LG3211_4845	pqqD; coenzyme PQQ biosynthesis protein PqqD
OG_09951	LG3211_4846	LG3211_4846; hypothetical protein
OG_09952	LG3211_4848	LG3211_4848; outer membrane autotransporter barrel domain protein
OG_09953	LG3211_4852	LG3211_4852; hypothetical protein
OG_09954	LG3211_4855	LG3211_4855; putative secreted domain protein
OG_09955	LG3211_4857	LG3211_4857; global regulator family protein
OG_09956	LG3211_4868	LG3211_4868; hypothetical protein
OG_09957	LG3211_4885	LG3211_4885; hypothetical protein
OG_09958	LG3211_4895	LG3211_4895; hypothetical protein
OG_09959	LG3211_4912	LG3211_4912; hypothetical protein
OG_09960	LG3211_4913	LG3211_4913; conserved hypothetical protein
OG_09961	LG3211_4953	LG3211_4953; hypothetical protein
OG_09962	LG3211_4961	LG3211_4961; hypothetical protein

OG_07626	LEC3_3220	hypothetical protein
OG_07627	LEC3_3223	hypothetical protein
OG_07628	LEC3_3224	lipoprotein
OG_07629	LEC3_3225	hypothetical protein
OG_07630	LEC3_3227	hypothetical protein
OG_07631	LEC3_3231	hypothetical protein
OG_07632	LEC3_3232	membrane protein
OG_07633	LEC3_3234	hypothetical protein
OG_07634	LEC3_3235	hypothetical protein
OG_07635	LEC3_3242	hypothetical protein
OG_07636	LEC3_3247	hypothetical protein
OG_07637	LEC3_3250	hypothetical protein
OG_07638	LEC3_3251	hypothetical protein
OG_07639	LEC3_3258	hypothetical protein
OG_07640	LEC3_3261	hypothetical protein
OG_07641	LEC3_3265	serine/arginine repetitive matrix 2
OG_07642	LEC3_3269	hypothetical protein
OG_07643	LEC3_3270	lipoprotein
OG_07644	LEC3_3271	hypothetical protein
OG_07645	LEC3_3279	hypothetical protein
OG_07646	LEC3_3280	conserved hypothetical protein
OG_07647	LEC3_3282	hypothetical protein
OG_07648	LEC3_3295	hypothetical protein
OG_07649	LEC3_3329	hypothetical protein
OG_07650	LEC3_3330	ankyrin repeat-containing exported protein
OG_07651	LEC3_3332	conserved hypothetical protein
OG_07652	LEC3_3333	conserved hypothetical protein
OG_07653	LEC3_3334	conserved hypothetical protein
OG_07654	LEC3_3335	conserved hypothetical protein
OG_07655	LEC3_3336	conserved hypothetical protein
OG_07656	LEC3_3337	conserved hypothetical protein
OG_07657	LEC3_3339	hypothetical protein
OG_07658	LEC3_3340	hypothetical protein
OG_07659	LEC3_3346	conserved hypothetical protein
OG_07660	LEC3_3351	hypothetical protein
OG_07661	LEC3_3353	hypothetical protein
OG_07662	LEC3_3372	hypothetical protein
OG_07663	LEC3_3387	hypothetical protein
OG_07664	LEC3_3392	transglycosylase family protein
OG_07665	LEC3_3395	hypothetical protein
OG_07666	LEC3_3398	bordetella uptake gene product family
OG_07667	LEC3_3404	UPF0307 protein
OG_07668	LEC3_3414	hypothetical protein
OG_07669	LEC3_3417	hypothetical protein
OG_07670	LEC3_3420	hypothetical protein
OG_07671	LEC3_3422	hypothetical protein
OG_07672	LEC3_3424	conserved hypothetical protein
OG_07673	LEC3_3426	hypothetical protein
OG_07674	LEC3_3427	hypothetical protein
OG_07675	LEC3_3428	hypothetical protein
OG_07676	LEC3_3429	hypothetical protein
OG_07677	LEC3_3441	hypothetical protein
OG_07678	LEC3_3442	hypothetical protein
OG_07679	LEC3_3447	hypothetical protein SEQUENCING GAP
OG_07680	LEC3_3454	hypothetical protein
OG_07681	LEC3_3456	hypothetical protein
OG_07682	LEC3_3459	conserved hypothetical protein
OG_07683	LEC3_3460	hypothetical protein
OG_07684	LEC3_3462	hypothetical protein
OG_07685	LEC3_3463	hypothetical protein
OG_07686	LEC3_3464	wall-associated protein
OG_07687	LEC3_3465	hypothetical protein
OG_07688	LEC3_3477	hypothetical protein
OG_07689	LEC3_3479	peptidase, families S8 and S53
OG_07690	LEC3_3480	hypothetical protein
OG_07691	LEC3_3481	hypothetical protein
OG_07692	LEC3_3484	conserved hypothetical protein
OG_07693	LEC3_3493	hypothetical protein
OG_07694	LEC3_3510	hypothetical protein
OG_07695	LEC3_3511	hypothetical protein
OG_07696	LEC3_3519	hypothetical protein

OG_09963	LG3211_4979	LG3211_4979; hypothetical protein
OG_09964	LG3211_4992	LG3211_4992; hypothetical protein
OG_09965	LG3211_5013	LG3211_5013; hypothetical protein
OG_09966	LG3211_5018	LG3211_5018; hypothetical protein
OG_09967	LG3211_5019	LG3211_5019; hypothetical protein
OG_09968	LG3211_5021	LG3211_5021; hypothetical protein
OG_09969	LG3211_5027	LG3211_5027; hypothetical protein
OG_09970	LG3211_5029	LG3211_5029; hypothetical protein
OG_09971	LG3211_5043	LG3211_5043; alpha-2-macroglobulin N-terminal region family protein
OG_09972	LG3211_5046	LG3211_5046; polysaccharide lyase family protein
OG_09973	LG3211_5048	LG3211_5048; putative lipoprotein
OG_09974	LG3211_5065	LG3211_5065; hep_Hag family protein
OG_09975	LG3211_5079	LG3211_5079; conserved hypothetical protein
OG_09976	LG3211_5089	LG3211_5089; hypothetical protein
OG_09977	LG3211_5098	LG3211_5098; HEAT repeats family protein
OG_09978	LG3211_5099	LG3211_5099; hypothetical protein
OG_09979	LG3211_5101	LG3211_5101; hypothetical protein
OG_09980	LG3211_5102	LG3211_5102; hypothetical protein
OG_09981	LG3211_5105	LG3211_5105; hypothetical protein
OG_09982	LG3211_5120	LG3211_5120; hypothetical protein
OG_09983	LG3211_5121	LG3211_5121; hypothetical protein
OG_09984	LG3211_5129	LG3211_5129; hypothetical protein
OG_09985	LG3211_5138	LG3211_5138; hypothetical protein
OG_09986	LG3211_5147	LG3211_5147; hypothetical protein
OG_09987	LG3211_5157	LG3211_5157; hypothetical protein
OG_09988	LG3211_5168	LG3211_5168; hypothetical protein
OG_09989	LG3211_5169	LG3211_5169; hypothetical protein
OG_09990	LG3211_5175	LG3211_5175; hypothetical protein
OG_09991	LG3211_5183	LG3211_5183; hypothetical protein
OG_09992	LG3211_5188	LG3211_5188; hypothetical protein
OG_09993	LG3211_5190	LG3211_5190; bacterial regulatory , arsR family protein
OG_09994	LG3211_5191	LG3211_5191; conserved hypothetical protein
OG_09995	LG3211_5192	LG3211_5192; alpha/beta hydrolase fold family protein
OG_09996	LG3211_5210	LG3211_5210; hypothetical protein
OG_09997	LG3211_5217	LG3211_5217; hypothetical protein
OG_09998	LG3211_5230	LG3211_5230; hypothetical protein
OG_09999	LG3211_5236	LG3211_5236; hypothetical protein
OG_10000	LG3211_5237	LG3211_5237; restriction endonuclease family protein
OG_10001	LG3211_5242	LG3211_5242; ubiquitin domain protein
OG_10002	LG3211_5243	LG3211_5243; hypothetical protein
OG_10003	LG3211_5248	hspA; low molecular weight heat shock protein
OG_10004	LG3211_5250	LG3211_5250; hypothetical protein
OG_10005	LG3211_5251	LG3211_5251; putative glyoxalase/bleomycin resistance protein/dioxygenase
OG_10006	LG3211_5258	LG3211_5258; histone family nucleoid-structuring H-NS domain protein
OG_10007	LG3211_5259	LG3211_5259; putative amine oxydase, deshydrogenase; Phytoene dehydrogen
OG_10008	LG3211_5263	LG3211_5263; terpene synthase family, metal binding domain protein
OG_10009	LG3211_5266	LG3211_5266; conserved hypothetical protein
OG_10010	LG3211_5271	LG3211_5271; hypothetical protein
OG_10011	LG3211_5273	LG3211_5273; metallo-beta-lactamase superfamily protein
OG_10012	LG3211_5279	LG3211_5279; hypothetical protein
OG_10013	LG3211_5282	LG3211_5282; hypothetical protein
OG_10014	LG3211_5283	LG3211_5283; hypothetical protein
OG_10015	LG3211_5284	LG3211_5284; hypothetical protein
OG_10016	LG3211_5287	LG3211_5287; hypothetical protein
OG_10017	LG3211_5289	LG3211_5289; hypothetical protein
OG_10018	LG3211_5293	LG3211_5293; hypothetical protein
OG_10019	LG3211_5296	LG3211_5296; hypothetical protein
OG_10020	LG3211_5317	LG3211_5317; hypothetical protein
OG_10021	LG3211_5324	LG3211_5324; hypothetical protein
OG_10022	LG3211_5327	LG3211_5327; hypothetical protein
OG_10023	LG3211_5330	LG3211_5330; hypothetical protein
OG_10024	LG3211_5341	LG3211_5341; hypothetical protein
OG_10025	LG3211_5343	LG3211_5343; hypothetical protein
OG_10026	LG3211_5349	LG3211_5349; hypothetical protein
OG_10027	LG3211_5374	LG3211_5374; hypothetical protein
OG_10028	LG3211_5377	LG3211_5377; hypothetical protein

OG_07697	LEC3_3520	hypothetical protein
OG_07698	LEC3_3521	conserved hypothetical protein
OG_07699	LEC3_3544	hypothetical protein
OG_07700	LEC3_3553	lipoprotein
OG_07701	LEC3_3575	type IV pilus assembly protein
OG_07702	LEC3_3579	type IV pilus assembly protein
OG_07703	LEC3_3597	hypothetical protein
OG_07704	LEC3_3602	hypothetical protein
OG_07705	LEC3_3606	hypothetical protein
OG_07706	LEC3_3616	hypothetical protein
OG_07707	LEC3_3627	hypothetical protein
OG_07708	LEC3_3635	hypothetical protein
OG_07709	LEC3_3644	hypothetical protein
OG_07710	LEC3_3647	conserved hypothetical protein
OG_07711	LEC3_3648	hypothetical protein
OG_07712	LEC3_3650	hypothetical protein
OG_07713	LEC3_3651	hypothetical protein
OG_07714	LEC3_3652	hypothetical protein
OG_07715	LEC3_3655	hypothetical protein
OG_07716	LEC3_3667	hypothetical protein
OG_07717	LEC3_3668	alpha-lytic protease prodomain
OG_07718	LEC3_3670	hypothetical protein
OG_07719	LEC3_3671	conserved hypothetical protein
OG_07720	LEC3_3672	hypothetical protein
OG_07721	LEC3_3678	TonB-dependent siderophore receptor
OG_07722	LEC3_3680	hypothetical protein
OG_07723	LEC3_3681	hypothetical protein
OG_07724	LEC3_3683	dihydrodipicolinate synthetase family
OG_07725	LEC3_3685	DNA-binding protein
OG_07726	LEC3_3686	hypothetical protein
OG_07727	LEC3_3690	hypothetical protein
OG_07728	LEC3_3691	hypothetical protein
OG_07729	LEC3_3694	conserved hypothetical protein
OG_07730	LEC3_3699	transporter, small conductance mechanosensitive ion channel (MscS) famil
OG_07731	LEC3_3701	sigma-54 dependent transcriptional regulator
OG_07732	LEC3_3702	hypothetical protein
OG_07733	LEC3_3704	phospholipid-binding domain protein
OG_07734	LEC3_3705	short chain dehydrogenase/reductase family protein
OG_07735	LEC3_3706	uracil-DNA glycosylase, family 4
OG_07736	LEC3_3708	trehalose-phosphatase
OG_07737	LEC3_3709	alpha,alpha-trehalose-phosphate synthase
OG_07738	LEC3_3710	glycosyl transferase, group 1 family protein
OG_07739	LEC3_3711	amylo-alpha-1,6-glucosidase
OG_07740	LEC3_3712	hypothetical protein
OG_07741	LEC3_3713	gp06 protein
OG_07742	LEC3_3719	hypothetical protein
OG_07743	LEC3_3720	Rhs family protein
OG_07744	LEC3_3721	Rhs family protein
OG_07745	LEC3_3722	hypothetical protein
OG_07746	LEC3_3723	conserved hypothetical protein
OG_07747	LEC3_3725	hypothetical protein
OG_07748	LEC3_3734	N-acetylmuramoyl-L-alanine amidase
OG_07749	LEC3_3753	hypothetical protein
OG_07750	LEC3_3760	hypothetical protein
OG_07751	LEC3_3805	hypothetical protein
OG_07752	LEC3_3819	PAS/GAF domain protein
OG_07753	LEC3_3835	peptidase, M28 family
OG_07754	LEC3_3836	hypothetical protein
OG_07755	LEC3_3838	hypothetical protein
OG_07756	LEC3_3842	hypothetical protein
OG_07757	LEC3_3844	conserved hypothetical protein
OG_07758	LEC3_3848	hypothetical protein
OG_07759	LEC3_3849	subtilase family
OG_07760	LEC3_3851	hypothetical protein
OG_07761	LEC3_3853	hypothetical protein
OG_07762	LEC3_3862	conserved hypothetical protein
OG_07763	LEC3_3875	conserved hypothetical protein
OG_07764	LEC3_3880	SptB protein
OG_07765	LEC3_3883	hypothetical protein
OG_07766	LEC3_3891	hypothetical protein
OG_07767	LEC3_3899	hypothetical protein

OG_07768	LEC3_3929	hypothetical protein
OG_07769	LEC3_3948	signal transduction histidine kinase
OG_07770	LEC3_3949	possibly something SEQUENCING GAP
OG_07771	LEC3_3954	hypothetical protein
OG_07772	LEC3_3963	hypothetical protein
OG_07773	LEC3_3973	hypothetical protein
OG_07774	LEC3_3975	hypothetical protein
OG_07775	LEC3_3984	hypothetical protein
OG_07776	LEC3_3987	hypothetical protein
OG_07777	LEC3_3989	hypothetical protein
OG_07778	LEC3_3992	transcriptional regulatory protein
OG_07779	LEC3_3993	hypothetical protein
OG_07780	LEC3_3994	hypothetical protein
OG_07781	LEC3_3996	hypothetical protein
OG_07782	LEC3_3997	linear gramicidin synthetase subunit D
OG_07783	LEC3_3998	hypothetical protein
OG_07784	LEC3_4001	hypothetical protein
OG_07785	LEC3_4003	hypothetical protein
OG_07786	LEC3_4006	conserved hypothetical protein
OG_07787	LEC3_4010	arsenical resistance operon repressor
OG_07788	LEC3_4014	arsenate reductase
OG_07789	LEC3_4015	arsenical resistance protein ArsH
OG_07790	LEC3_4019	hypothetical protein
OG_07791	LEC3_4020	hypothetical protein
OG_07792	LEC3_4021	hypothetical protein
OG_07793	LEC3_4024	hypothetical protein
OG_07794	LEC3_4025	hypothetical protein
OG_07795	LEC3_4026	acetyltransferase (GNAT) family
OG_07796	LEC3_4030	hypothetical protein
OG_07797	LEC3_4033	signal transduction protein
OG_07798	LEC3_4043	conserved hypothetical protein
OG_07799	LEC3_4044	hypothetical protein
OG_07800	LEC3_4046	hypothetical protein
OG_07801	LEC3_4047	cytochrome c
OG_07802	LEC3_4048	catalase/peroxidase HPI
OG_07803	LEC3_4073	hypothetical protein
OG_07804	LEC3_4093	hypothetical protein
OG_07805	LEC3_4097	hypothetical protein
OG_07806	LEC3_4110	hypothetical protein
OG_07807	LEC3_4115	sulfatase
OG_07808	LEC3_4121	hypothetical protein
OG_07809	LEC3_4164	membrane protein
OG_07810	LEC3_4167	hydrolase
OG_07811	LEC3_4182	conserved hypothetical protein
OG_07812	LEC3_4184	hypothetical protein
OG_07813	LEC3_4185	hypothetical protein
OG_07814	LEC3_4188	conserved hypothetical protein
OG_07815	LEC3_4195	hypothetical protein
OG_07816	LEC3_4206	alginate biosynthesis transcriptional regulatory protein AlgB
OG_07817	LEC3_4207	sensory box histidine kinase
OG_07818	LEC3_4211	hypothetical protein
OG_07819	LEC3_4213	ABC transporter, ATP-binding protein
OG_07820	LEC3_4214	hypothetical protein
OG_07821	LEC3_4215	hypothetical protein
OG_07822	LEC3_4222	hypothetical protein
OG_07823	LEC3_4224	hypothetical protein
OG_07824	LEC3_4226	hypothetical protein
OG_07825	LEC3_4238	membrane protein
OG_07826	LEC3_4239	hypothetical protein
OG_07827	LEC3_4249	hypothetical protein
OG_07828	LEC3_4261	hypothetical protein
OG_07829	LEC3_4265	hypothetical protein
OG_07830	LEC3_4274	conserved hypothetical protein
OG_07831	LEC3_4275	hypothetical protein
OG_07832	LEC3_4276	conserved hypothetical protein
OG_07833	LEC3_4277	conserved hypothetical protein
OG_07834	LEC3_4278	conserved hypothetical protein
OG_07835	LEC3_4279	conserved hypothetical protein
OG_07836	LEC3_4280	conserved hypothetical protein
OG_07837	LEC3_4283	hypothetical protein
OG_07838	LEC3_4284	hypothetical protein

OG_07839	LEC3_4294	hypothetical protein
OG_07840	LEC3_4303	hypothetical protein
OG_07841	LEC3_4307	hypothetical protein
OG_07842	LEC3_4312	hypothetical protein
OG_07843	LEC3_4315	conserved hypothetical protein
OG_07844	LEC3_4320	hypothetical protein
OG_07845	LEC3_4324	conserved hypothetical protein
OG_07846	LEC3_4327	hypothetical protein
OG_07847	LEC3_4349	hypothetical protein
OG_07848	LEC3_4352	hypothetical protein
OG_07849	LEC3_4361	hypothetical protein
OG_07850	LEC3_4367	hypothetical protein
OG_07851	LEC3_4383	hypothetical protein
OG_07852	LEC3_4400	conserved hypothetical protein
OG_07853	LEC3_4404	hypothetical protein
OG_07854	LEC3_4405	hypothetical protein
OG_07855	LEC3_4406	hypothetical protein
OG_07856	LEC3_4411	hypothetical protein
OG_07857	LEC3_4416	hypothetical protein
OG_07858	LEC3_4418	hypothetical protein
OG_07859	LEC3_4425	signal peptidase I
OG_07860	LEC3_4426	conserved hypothetical protein
OG_07861	LEC3_4427	hypothetical protein
OG_07862	LEC3_4428	hypothetical protein
OG_07863	LEC3_4432	hypothetical protein
OG_07864	LEC3_4438	conserved hypothetical protein
OG_07865	LEC3_4439	Oxygen-independent coproporphyrinogen III oxidase 1
OG_07866	LEC3_4440	acyl carrier protein
OG_07867	LEC3_4441	AMP-binding enzyme
OG_07868	LEC3_4442	hypothetical protein
OG_07869	LEC3_4443	hypothetical protein
OG_07870	LEC3_4444	tRNA nucleotidyltransferase
OG_07871	LEC3_4445	ABC-2 type transporter
OG_07872	LEC3_4450	hypothetical protein
OG_07873	LEC3_4461	hypothetical protein
OG_07874	LEC3_4473	conserved hypothetical protein
OG_07875	LEC3_4474	lytic enzyme
OG_07876	LEC3_4485	cytosolic protein
OG_07877	LEC3_4487	hypothetical protein
OG_07878	LEC3_4492	hypothetical protein
OG_07879	LEC3_4493	conserved hypothetical protein
OG_07880	LEC3_4497	hypothetical protein
OG_07881	LEC3_4498	conserved hypothetical protein
OG_07882	LEC3_4499	histidine kinase family protein
OG_07883	LEC3_4500	hypothetical protein
OG_07884	LEC3_4501	transcriptional regulator, AraC family
OG_07885	LEC3_4502	methyltransferase
OG_07886	LEC3_4503	hypothetical protein
OG_07887	LEC3_4504	hypothetical protein
OG_07888	LEC3_4507	hypothetical protein
OG_07889	LEC3_4508	conserved hypothetical protein
OG_07890	LEC3_4520	hypothetical protein
OG_07891	LEC3_4526	hypothetical protein
OG_07892	LEC3_4538	hypothetical protein
OG_07893	LEC3_4549	hypothetical protein
OG_07894	LEC3_4577	hypothetical protein
OG_07895	LEC3_4580	hypothetical protein
OG_07896	LEC3_4590	hypothetical protein
OG_07897	LEC3_4591	hypothetical protein
OG_07898	LEC3_4594	hypothetical protein
OG_07899	LEC3_4615	hypothetical protein
OG_07900	LEC3_4622	conserved hypothetical protein
OG_07901	LEC3_4630	hypothetical protein
OG_07902	LEC3_4655	hypothetical protein
OG_07903	LEC3_4656	hypothetical protein
OG_07904	LEC3_4659	hypothetical protein
OG_07905	LEC3_4660	conserved hypothetical protein
OG_07906	LEC3_4662	hypothetical protein
OG_07907	LEC3_4676	hypothetical protein
OG_07908	LEC3_4677	lipoprotein
OG_07909	LEC3_4678	hypothetical protein

OG_07910	LEC3_4683	hypothetical protein
OG_07911	LEC3_4691	hypothetical protein
OG_07912	LEC3_4693	hypothetical protein
OG_07913	LEC3_4696	hypothetical protein
OG_07914	LEC3_4703	hypothetical protein
OG_07915	LEC3_4709	hypothetical protein
OG_07916	LEC3_4716	hypothetical protein
OG_07917	LEC3_4718	hypothetical protein
OG_07918	LEC3_4720	hypothetical protein
OG_07919	LEC3_4733	hypothetical protein
OG_07920	LEC3_4736	apical junction molecule protein
OG_07921	LEC3_4742	hypothetical protein
OG_07922	LEC3_4743	hypothetical protein
OG_07923	LEC3_4744	serine protease, subtilase family
OG_07924	LEC3_4745	Trypsin
OG_07925	LEC3_4746	hypothetical protein
OG_07926	LEC3_4747	conserved hypothetical protein
OG_07927	LEC3_4750	hypothetical protein
OG_07928	LEC3_4751	metal dependent amidohydrolase
OG_07929	LEC3_4757	hypothetical protein
OG_07930	LEC3_4758	tetratricopeptide repeat protein
OG_07931	LEC3_4764	conserved hypothetical protein
OG_07932	LEC3_4766	conserved hypothetical protein
OG_07933	LEC3_4768	DNA mismatch repair protein
OG_07934	LEC3_4784	hypothetical protein
OG_07935	LEC3_4787	hypothetical protein
OG_07936	LEC3_4797	conserved hypothetical protein
OG_07937	LEC3_4798	hypothetical protein
OG_07938	LEC3_4806	hypothetical protein
OG_07939	LEC3_4812	taurine catabolism dioxygenase TauD, TfdA family
OG_07940	LEC3_4813	pyoverdine/dityrosine biosynthesis protein
OG_07941	LEC3_4814	hypothetical protein
OG_07942	LEC3_4815	hypothetical protein
OG_07943	LEC3_4816	aminotransferase, class I and II
OG_07944	LEC3_4817	pyridoxal-dependent decarboxylase domain protein
OG_07945	LEC3_4818	integral membrane protein DUF6
OG_07946	LEC3_4825	glutathione-dependent formaldehyde-activating protein
OG_07947	LEC3_4829	peptidase, families S8 and S53
OG_07948	LEC3_4830	hypothetical protein
OG_07949	LEC3_4834	hypothetical protein
OG_07950	LEC3_4837	hypothetical protein
OG_07951	LEC3_4844	hypothetical protein
OG_07952	LEC3_4846	hypothetical protein
OG_07953	LEC3_4867	hypothetical protein
OG_07954	LEC3_4869	hypothetical protein
OG_07955	LEC3_4870	hypothetical protein
OG_07956	LEC3_4871	hypothetical protein
OG_07957	LEC3_4875	hypothetical protein
OG_07958	LEC3_4876	hypothetical protein
OG_07959	LEC3_4878	hypothetical protein
OG_07960	LEC3_4888	hypothetical protein
OG_07961	LEC3_4894	amino acid adenylation domain protein
OG_07962	LEC3_4897	hypothetical protein
OG_07963	LEC3_4901	hypothetical protein
OG_07964	LEC3_4906	hypothetical protein
OG_07965	LEC3_4919	hypothetical protein
OG_07966	LEC3_4927	lipoprotein
OG_07967	LEC3_4932	hypothetical protein
OG_07968	LEC3_4935	hypothetical protein
OG_07969	LEC3_4936	hypothetical protein
OG_07970	LEC3_4938	hypothetical protein
OG_07971	LEC3_4953	hypothetical protein
OG_07972	LEC3_4955	hypothetical protein
OG_07973	LEC3_4957	hypothetical protein
OG_07974	LEC3_4958	possibly something SEQUENCING GAP
OG_07975	LEC3_4965	hypothetical protein
OG_07976	LEC3_4982	hypothetical protein
OG_07977	LEC3_4993	hypothetical protein
OG_07978	LEC3_5008	hypothetical protein
OG_07979	LEC3_5015	hypothetical protein
OG_07980	LEC3_5039	hypothetical protein

OG_07981	LEC3_5047	hypothetical protein
OG_07982	LEC3_5052	hypothetical protein
OG_07983	LEC3_5064	hypothetical protein
OG_07984	LEC3_5066	hypothetical protein
OG_07985	LEC3_5078	hypothetical protein
OG_07986	LEC3_5080	hypothetical protein
OG_07987	LEC3_5089	hypothetical protein
OG_07988	LEC3_5090	hypothetical protein
OG_07989	LEC3_5096	hypothetical protein
OG_07990	LEC3_5098	hypothetical protein
OG_07991	LEC3_5113	hypothetical protein
OG_07992	LEC3_5130	hypothetical protein
OG_07993	LEC3_5134	conserved hypothetical protein
OG_07994	LEC3_5136	hypothetical protein
OG_07995	LEC3_5151	hypothetical protein
OG_07996	LEC3_5155	conserved hypothetical protein
OG_07997	LEC3_5157	hypothetical protein
OG_07998	LEC3_5158	conserved hypothetical protein
OG_07999	LEC3_5172	tetracycline resistance protein, class A (TetA(A))
OG_08000	LEC3_5173	Tetracyclin repressor, C-terminal all-alpha domain
OG_08001	LEC3_5174	hypothetical protein
OG_08002	LEC3_5178	hypothetical protein
OG_08003	LEC3_5194	conserved hypothetical protein
OG_08004	LEC3_5198	conserved hypothetical protein
OG_08005	LEC3_5201	conserved hypothetical protein
OG_08006	LEC3_5212	hypothetical protein
OG_08007	LEC3_5214	lipoprotein
OG_08008	LEC3_5239	hypothetical protein
OG_08009	LEC3_5243	cytosolic protein
OG_08010	LEC3_5285	hypothetical protein
OG_08011	LEC3_5297	fumarylacetoacetase
OG_08012	LEC3_5299	conserved hypothetical protein
OG_08013	LEC3_5300	FAD binding domain/FAD dependent oxidoreductase
OG_08014	LEC3_5302	transcriptional regulator, LuxR family
OG_08015	LEC3_5303	FAD dependent oxidoreductase
OG_08016	LEC3_5304	conserved hypothetical protein
OG_08017	LEC3_5305	phenylacetaldehyde dehydrogenase (PAD)
OG_08018	LEC3_5308	conserved hypothetical protein
OG_08019	LEC3_5313	hypothetical protein
OG_08020	LEC3_5318	major facilitator superfamily MFS_1, putative
OG_08021	LEC3_5319	radical SAM domain protein
OG_08022	LEC3_5324	hypothetical protein
OG_08023	LEC3_5325	hypothetical protein
OG_08024	LEC3_5326	hypothetical protein
OG_08025	LEC3_5341	hypothetical protein
OG_08026	LEC3_5354	hypothetical protein
OG_08027	LEC3_5362	hypothetical protein
OG_08028	LEC3_5380	conserved hypothetical protein
OG_08029	LEC3_5381	hypothetical protein
OG_08030	LEC3_5382	hypothetical protein
OG_08031	LEC3_5384	N-acetylmuramoyl-L-alanine amidase
OG_08032	LEC3_5385	hypothetical protein
OG_08033	LEC3_5387	hypothetical protein
OG_08034	LEC3_5388	ATP-dependent helicase
OG_08035	LEC3_5410	hypothetical protein
OG_08036	LEC3_5411	conserved hypothetical protein
OG_08037	LEC3_5417	hypothetical protein
OG_08038	LEC3_5419	hypothetical protein
OG_08039	LEC3_5420	hypothetical protein
OG_08040	LEC3_5424	hypothetical protein
OG_08041	LEC3_5426	hypothetical protein
OG_08042	LEC3_5430	lipoprotein
OG_08043	LEC3_5438	hypothetical protein
OG_08044	LEC3_5443	inorganic anion transporter, sulfate permease family
OG_08045	LEC3_5444	hypothetical protein
OG_08046	LEC3_5448	hypothetical protein
OG_08047	LEC3_5449	adenosine/AMP deaminase
OG_08048	LEC3_5450	purine nucleoside permease (NUP)
OG_08049	LEC3_5452	hypothetical protein
OG_08050	LEC3_5454	lipoprotein
OG_08051	LEC3_5455	gram-negative pili assembly chaperone, N-terminal domain

OG_08052	LEC3_5456	outer membrane usher protein
OG_08053	LEC3_5457	fimbrial protein
OG_08054	LEC3_5458	hypothetical protein
OG_08055	LEC3_5470	hypothetical protein
OG_08056	LEC3_5472	hypothetical protein
OG_08057	LEC3_5474	hypothetical protein
OG_08058	LEC3_5477	hypothetical protein
OG_08059	LEC3_5479	hypothetical protein
OG_08060	LEC3_5480	prolyl 4-hydroxylase, alpha subunit
OG_08061	LEC3_5482	hypothetical protein
OG_08062	LEC3_5484	cysteine desulfurase
OG_08063	LEC3_5485	conserved hypothetical protein
OG_08064	LEC3_5486	hypothetical protein
OG_08065	LEC3_5487	phosphoadenosine phosphosulfate reductase
OG_08066	LEC3_5488	histidine kinase family protein
OG_08067	LEC3_5489	hypothetical protein
OG_08068	LEC3_5493	hypothetical protein
OG_08069	LEC3_5496	hypothetical protein
OG_08070	LEC3_5497	conserved hypothetical protein
OG_08071	LEC3_5502	hypothetical protein
OG_08072	LEC3_5503	plasmid/phage primase, P4 family
OG_08073	LEC3_5505	conserved hypothetical protein
OG_08074	LEC3_5509	hypothetical protein
OG_08075	LEC3_5510	hypothetical protein
OG_08076	LEC3_5511	hypothetical protein
OG_08077	LEC3_5513	hypothetical protein
OG_08078	LEC3_5514	hypothetical protein
OG_08079	LEC3_5518	hypothetical protein
OG_08080	LEC3_5520	hypothetical protein
OG_08081	LEC3_5526	conserved hypothetical protein
OG_08082	LEC3_5528	hypothetical protein
OG_08083	LEC3_5529	hypothetical protein
OG_08084	LEC3_5530	hypothetical protein
OG_08085	LEC3_5531	hypothetical protein
OG_08086	LEC3_5533	hypothetical protein
OG_08087	LEC3_5534	myosin, heavy polypeptide 7B, cardiac muscle, beta isoform 1
OG_08088	LEC3_5536	hypothetical protein
OG_08089	LEC3_5537	hypothetical protein
OG_08090	LEC3_5538	conserved hypothetical protein

Table S3: CDSs unique to either *Lysobacter antibioticus or L. capsici* species.

Orthologous group	*L. ant* ATCC29479 GeneID (#667) 12.9% of genome	Gene description	Orthologous group	*L. ant* 76 GeneID (#670) 13.0% of genome	Gene description
OG_03094	LA29479_592	hypothetical protein	OG_03094	LA76x_1675	LA76x_1675; metallo-peptidase M12B Reprolysin-like family protein
OG_03094	LA29479_591	hypothetical protein	OG_03094	LA76x_455	LA76x_455; metallo-peptidase M12B Reprolysin-like family protein
			OG_03094	LA76x_1676	LA76x_1676; metallo-peptidase M12B Reprolysin-like family protein
OG_03095	LA29479_4600	toxin RTX-I translocation ATP-binding protein	OG_03095	LA76x_4095	hlyB; alpha-hemolysin translocation ATP-binding protein hlyB
OG_03095	LA29479_1509	toxin RTX-I translocation ATP-binding protein	OG_03095	LA76x_1995	hlyB; alpha-hemolysin translocation ATP-binding protein hlyB
			OG_03095	LA76x_461	hlyB; alpha-hemolysin translocation ATP-binding protein hlyB
OG_03482	LA29479_3655	hypothetical protein	OG_03482	LA76x_5160	LA76x_5160; hypothetical protein
OG_03482	LA29479_2436	hypothetical protein			
OG_03482	LA29479_1102	hypothetical protein			
OG_03484	LA29479_349	putative uncharacterized domain protein	OG_03484	LA76x_1776	LA76x_1776; conserved hypothetical protein
OG_03484	LA29479_4227	putative uncharacterized protein	OG_03484	LA76x_5095	LA76x_5095; conserved hypothetical protein
OG_03485	LA29479_1159	hypothetical protein	OG_03485	LA76x_47	LA76x_47; hypothetical protein
OG_03485	LA29479_819	hypothetical protein	OG_03485	LA76x_4784	LA76x_4784; hypothetical protein
OG_03521	LA29479_2610	hypothetical protein	OG_03521	LA76x_4992	LA76x_4992; hypothetical protein
OG_03521	LA29479_5125	hypothetical protein	OG_03521	LA76x_537	LA76x_537; hypothetical protein
OG_03581	LA29479_2447	hypothetical protein	OG_03581	LA76x_4783	LA76x_4783; hypothetical protein
OG_03581	LA29479_807	hypothetical protein	OG_03581	LA76x_4785	LA76x_4785; hypothetical protein
OG_03985	LA29479_1243	hypothetical protein	OG_03985	LA76x_1701	LA76x_1701; hypothetical protein
OG_03985	LA29479_5038	hypothetical protein			
OG_03986	LA29479_2984	filamentous haemagglutinin family N-terminal domain protein	OG_03986	LA76x_2663	LA76x_2663; filamentous hemagglutinin family N-terminal domain pro
OG_03986	LA29479_4436	filamentous haemagglutinin family N-terminal domain protein			
OG_03988	LA29479_406	sensory box protein	OG_03988	LA76x_5045	LA76x_5045; diguanylate cyclase domain protein
OG_03988	LA29479_2621	sensory box protein			
OG_04001	LA29479_3024	hypothetical protein	OG_04001	LA76x_2629	LA76x_2629; hypothetical protein
			OG_04001	LA76x_459	LA76x_459; hypothetical protein
OG_04021	LA29479_4597	WGS CADB00000000 data, contig 91	OG_04021	LA76x_464	LA76x_464; HNH/ENDO VII supernuclease with conserved GHE resi
			OG_04021	LA76x_4092	LA76x_4092; HNH/ENDO VII supernuclease with conserved GHE res
OG_04061	LA29479_4599	type I secretion membrane fusion , HlyD family protein	OG_04061	LA76x_462	LA76x_462; type I secretion membrane fusion , HlyD family protein
			OG_04061	LA76x_4094	LA76x_4094; type I secretion membrane fusion , HlyD family protein
OG_04066	LA29479_4670	conserved hypothetical protein	OG_04066	LA76x_475	LA76x_475; conserved hypothetical protein
OG_04066	LA29479_5096	conserved hypothetical protein			
OG_04111	LA29479_1293	hypothetical protein	OG_04111	LA76x_66	LA76x_66; hypothetical protein
			OG_04111	LA76x_2519	LA76x_2519; hypothetical protein
OG_04128	LA29479_2541	nuclease-related domain protein	OG_04128	LA76x_4499	LA76x_4499; nuclease-related domain protein
			OG_04128	LA76x_4496	LA76x_4496; nuclease-related domain protein
OG_04184	LA29479_5102	hypothetical protein	OG_04184	LA76x_472	LA76x_472; hypothetical protein
			OG_04184	LA76x_480	LA76x_480; hypothetical protein
OG_04201	LA29479_4598	alpha-hemolysin translocation ATP-binding protein HlyB	OG_04201	LA76x_463	hlyB; alpha-hemolysin translocation ATP-binding protein hlyB
			OG_04201	LA76x_4093	hlyB; alpha-hemolysin translocation ATP-binding protein hlyB
OG_04658	LA29479_2702	hypothetical protein	OG_04658	LA76x_629	LA76x_629; hypothetical protein
OG_04659	LA29479_4504	hypothetical protein	OG_04659	LA76x_4654	LA76x_4654; hypothetical protein
OG_04660	LA29479_3610	hypothetical protein	OG_04660	LA76x_4018	LA76x_4018; hypothetical protein
OG_04661	LA29479_2179	fatty acid hydroxylase superfamily protein	OG_04661	LA76x_977	LA76x_977; fatty acid hydroxylase superfamily protein
OG_04662	LA29479_343	hypothetical protein	OG_04662	LA76x_5101	LA76x_5101; hypothetical protein
OG_04663	LA29479_4228	hypothetical protein	OG_04663	LA76x_1774	LA76x_1774; hypothetical protein
OG_04664	LA29479_2184	hypothetical protein	OG_04664	LA76x_971	LA76x_971; hypothetical protein
OG_04665	LA29479_2548	hypothetical protein	OG_04665	LA76x_4500	LA76x_4500; hypothetical protein
OG_04666	LA29479_71	hypothetical protein	OG_04666	LA76x_3117	LA76x_3117; hypothetical protein
OG_04667	LA29479_1656	hypothetical protein	OG_04667	LA76x_2179	LA76x_2179; hypothetical protein
OG_04668	LA29479_5050	bacterial regulatory helix-turn-helix s, AraC family protein	OG_04668	LA76x_4824	LA76x_4824; bacterial regulatory helix-turn-helix, AraC family protein
OG_04669	LA29479_3506	putative uncharacterized domain protein	OG_04669	LA76x_2731	LA76x_2731; hypothetical protein
OG_04670	LA29479_3005	helix-turn-helix family protein	OG_04670	LA76x_2643	LA76x_2643; helix-turn-helix family protein
OG_04671	LA29479_3631	putative uncharacterized protein	OG_04671	LA76x_3238	LA76x_3238; conserved hypothetical protein
OG_04672	LA29479_2449	hypothetical protein	OG_04672	LA76x_3413	LA76x_3413; hypothetical protein
OG_04673	LA29479_4323	putative membrane protein	OG_04673	LA76x_1113	LA76x_1113; putative membrane protein
OG_04674	LA29479_412	hypothetical protein	OG_04674	LA76x_5041	LA76x_5041; hypothetical protein
OG_04675	LA29479_4133	hypothetical protein	OG_04675	LA76x_321	LA76x_321; hypothetical protein
OG_04676	LA29479_3607	putative uncharacterized domain protein	OG_04676	LA76x_4015	LA76x_4015; conserved hypothetical protein
OG_04678	LA29479_3296	putative uncharacterized protein	OG_04678	LA76x_187	LA76x_187; snoaL-like domain protein
OG_04679	LA29479_738	hypothetical protein	OG_04679	LA76x_1538	LA76x_1538; hypothetical protein
OG_04680	LA29479_5168	hypothetical protein	OG_04680	LA76x_837	LA76x_837; hypothetical protein
OG_04681	LA29479_1040	IS3/IS911 family transposase	OG_04681	LA76x_2603	LA76x_2603; snoaL-like domain protein
OG_04682	LA29479_2412	putative uncharacterized protein yjbG	OG_04682	LA76x_3382	LA76x_3382; capsule biosynthesis GfcC family protein
OG_04683	LA29479_4591	hypothetical protein	OG_04683	LA76x_4087	LA76x_4087; hypothetical protein
OG_04684	LA29479_2983	hypothetical protein	OG_04684	LA76x_2664	LA76x_2664; hypothetical protein
OG_04686	LA29479_2206	hypothetical protein	OG_04686	LA76x_951	LA76x_951; hypothetical protein
OG_04687	LA29479_3001	bacterial regulatory s, tetR family protein	OG_04687	LA76x_2647	LA76x_2647; bacterial regulatory, tetR family protein
OG_04688	LA29479_1928	cytochrome C biogenesis transmembrane region family protein	OG_04688	LA76x_1364	LA76x_1364; cytochrome C biogenesis transmembrane region family
OG_04689	LA29479_2861	bacterial regulatory helix-turn-helix s, AraC family protein	OG_04689	LA76x_666	LA76x_666; bacterial regulatory helix-turn-helix, AraC family protein
OG_04690	LA29479_3924	hypothetical protein	OG_04690	LA76x_754	LA76x_754; hypothetical protein
OG_04691	LA29479_340	hypothetical protein	OG_04691	LA76x_5104	LA76x_5104; hypothetical protein
OG_04692	LA29479_3178	hypothetical protein	OG_04692	LA76x_1844	LA76x_1844; hypothetical protein
OG_04693	LA29479_178	putative uncharacterized protein	OG_04693	LA76x_3012	LA76x_3012; conserved hypothetical protein
OG_04694	LA29479_1818	sigma-54 interaction domain protein	OG_04694	LA76x_2808	LA76x_2808; AAA domain family protein
OG_04695	LA29479_2397	CBS domain pair family protein	OG_04695	LA76x_3365	LA76x_3365; CBS domain protein
OG_04696	LA29479_1457	hypothetical protein	OG_04696	LA76x_2048	LA76x_2048; hypothetical protein
OG_04697	LA29479_4344	hypothetical protein	OG_04697	LA76x_1491	LA76x_1491; hypothetical protein

OG_04698	LA29479_1035	hypothetical protein	OG_04698	LA76x_2608	LA76x_2608; hypothetical protein
OG_04699	LA29479_2687	DJ-1/PfpI family protein	OG_04699	LA76x_614	LA76x_614; N-terminal domain of DJ-1_PfpI family protein
OG_04700	LA29479_4853	hypothetical protein	OG_04700	LA76x_3321	LA76x_3321; hypothetical protein
OG_04701	LA29479_626	hypothetical protein	OG_04701	LA76x_1649	LA76x_1649; hypothetical protein
OG_04702	LA29479_4861	hypothetical protein	OG_04702	LA76x_3521	LA76x_3521; hypothetical protein
OG_04703	LA29479_2385	putative uncharacterized protein	OG_04703	LA76x_3353	LA76x_3353; conserved hypothetical protein
OG_04704	LA29479_4882	hypothetical protein	OG_04704	LA76x_3540	LA76x_3540; hypothetical protein
OG_04705	LA29479_2202	hypothetical protein	OG_04705	LA76x_954	LA76x_954; hypothetical protein
OG_04707	LA29479_4756	hypothetical protein	OG_04707	LA76x_2276	LA76x_2276; hypothetical protein
OG_04708	LA29479_2382	hypothetical protein	OG_04708	LA76x_3350	LA76x_3350; serine hydrolase family protein
OG_04709	LA29479_1808	hypothetical protein	OG_04709	LA76x_2818	LA76x_2818; hypothetical protein
OG_04710	LA29479_3938	hypothetical protein	OG_04710	LA76x_769	LA76x_769; hypothetical protein
OG_04711	LA29479_382	hypothetical protein	OG_04711	LA76x_5065	LA76x_5065; hypothetical protein
OG_04712	LA29479_4529	hypothetical protein	OG_04712	LA76x_1905	LA76x_1905; hypothetical protein
OG_04713	LA29479_3959	hypothetical protein	OG_04713	LA76x_790	LA76x_790; hypothetical protein
OG_04714	LA29479_5107	hypothetical protein	OG_04714	LA76x_485	LA76x_485; hypothetical protein
OG_04715	LA29479_3437	hypothetical protein	OG_04715	LA76x_4221	LA76x_4221; hypothetical protein
OG_04716	LA29479_3603	hypothetical protein	OG_04716	LA76x_4011	LA76x_4011; hypothetical protein
OG_04717	LA29479_5099	putative helicase	OG_04717	LA76x_477	LA76x_477; viral (Super1) RNA helicase family protein
OG_04718	LA29479_3975	hypothetical protein	OG_04718	LA76x_4564	LA76x_4564; hypothetical protein
OG_04719	LA29479_2870	putative membrane protein	OG_04719	LA76x_675	LA76x_675; hypothetical protein
OG_04720	LA29479_2752	hypothetical protein	OG_04720	LA76x_2382	LA76x_2382; hypothetical protein
OG_04721	LA29479_3065	hypothetical protein	OG_04721	LA76x_879	LA76x_879; hypothetical protein
OG_04722	LA29479_1438	hypothetical protein	OG_04722	LA76x_296	LA76x_296; hypothetical protein
OG_04723	LA29479_2728	hypothetical protein	OG_04723	LA76x_2407	LA76x_2407; hypothetical protein
OG_04724	LA29479_2648	hypothetical protein	OG_04724	LA76x_572	LA76x_572; hypothetical protein
OG_04725	LA29479_1955	hypothetical protein	OG_04725	LA76x_1244	LA76x_1244; hypothetical protein
OG_04726	LA29479_2419	hypothetical protein	OG_04726	LA76x_3388	LA76x_3388; hypothetical protein
OG_04727	LA29479_598	hypothetical protein	OG_04727	LA76x_1669	LA76x_1669; hypothetical protein
OG_04728	LA29479_2959	hypothetical protein	OG_04728	LA76x_2689	LA76x_2689; hypothetical protein
OG_04729	LA29479_1961	hypothetical protein	OG_04729	LA76x_1239	LA76x_1239; hypothetical protein
OG_04730	LA29479_4399	hypothetical protein	OG_04730	LA76x_518	LA76x_518; hypothetical protein
OG_04731	LA29479_1157	hypothetical protein	OG_04731	LA76x_2487	LA76x_2487; hypothetical protein
OG_04733	LA29479_3106	putative uncharacterized protein	OG_04733	LA76x_918	LA76x_918; conserved hypothetical protein
OG_04734	LA29479_2579	hypothetical protein	OG_04734	LA76x_4530	LA76x_4530; hypothetical protein
OG_04735	LA29479_2379	hypothetical protein	OG_04735	LA76x_3347	LA76x_3347; hypothetical protein
OG_04736	LA29479_2705	ricin-type beta-trefoil lectin domain protein	OG_04736	LA76x_2430	LA76x_2430; ricin-type beta-trefoil lectin domain protein
OG_04737	LA29479_1858	hypothetical protein	OG_04737	LA76x_2772	LA76x_2772; hypothetical protein
OG_04738	LA29479_5089	hypothetical protein	OG_04738	LA76x_3255	LA76x_3255; hypothetical protein
OG_04739	LA29479_5104	hypothetical protein	OG_04739	LA76x_482	LA76x_482; hypothetical protein
OG_04740	LA29479_2404	putative uncharacterized domain protein	OG_04740	LA76x_3372	LA76x_3372; conserved hypothetical protein
OG_04741	LA29479_150	hypothetical protein	OG_04741	LA76x_3037	LA76x_3037; hypothetical protein
OG_04742	LA29479_2305	hypothetical protein	OG_04742	LA76x_4368	LA76x_4368; hypothetical protein
OG_04743	LA29479_4863	hypothetical protein	OG_04743	LA76x_3522	LA76x_3522; hypothetical protein
OG_04744	LA29479_226	hypothetical protein	OG_04744	LA76x_2964	LA76x_2964; hypothetical protein
OG_04745	LA29479_1019	hypothetical protein	OG_04745	LA76x_2624	LA76x_2624; hypothetical protein
OG_04746	LA29479_168	hypothetical protein	OG_04746	LA76x_3022	LA76x_3022; hypothetical protein
OG_04748	LA29479_1508	type I secretion membrane fusion , HlyD family protein	OG_04748	LA76x_1996	LA76x_1996; type I secretion membrane fusion , HlyD family protein
OG_04749	LA29479_385	hypothetical protein	OG_04749	LA76x_5062	LA76x_5062; hypothetical protein
OG_04750	LA29479_5166	hypothetical protein	OG_04750	LA76x_836	LA76x_836; hypothetical protein
OG_04751	LA29479_2254	hypothetical protein	OG_04751	LA76x_4321	LA76x_4321; hypothetical protein
OG_04752	LA29479_174	hypothetical protein	OG_04752	LA76x_3016	LA76x_3016; hypothetical protein
OG_04753	LA29479_4333	hypothetical protein	OG_04753	LA76x_1501	LA76x_1501; hypothetical protein
OG_04754	LA29479_3979	hypothetical protein	OG_04754	LA76x_4568	LA76x_4568; hypothetical protein
OG_04755	LA29479_1881	hypothetical protein	OG_04755	LA76x_2750	LA76x_2750; hypothetical protein
OG_04756	LA29479_2919	beta-galactosidase	OG_04756	LA76x_720	bga; beta-galactosidase
OG_04757	LA29479_880	hypothetical protein	OG_04757	LA76x_3719	LA76x_3719; hypothetical protein
OG_04758	LA29479_4565	hypothetical protein	OG_04758	LA76x_796	LA76x_796; hypothetical protein
OG_04759	LA29479_836	hypothetical protein	OG_04759	LA76x_3676	LA76x_3676; hypothetical protein
OG_04760	LA29479_94	putative membrane protein	OG_04760	LA76x_3094	LA76x_3094; hypothetical protein
OG_04761	LA29479_4102	astacin family protein	OG_04761	LA76x_351	LA76x_351; astacin family protein
OG_04762	LA29479_2386	hypothetical protein	OG_04762	LA76x_3354	LA76x_3354; hypothetical protein
OG_04763	LA29479_3133	hypothetical protein	OG_04763	LA76x_946	LA76x_946; hypothetical protein
OG_04764	LA29479_2378	hypothetical protein	OG_04764	LA76x_3346	LA76x_3346; hypothetical protein
OG_04765	LA29479_2431	NIPSNAP family protein	OG_04765	LA76x_3400	LA76x_3400; NIPSNAP family protein
OG_04766	LA29479_2031	hypothetical protein	OG_04766	LA76x_1174	LA76x_1174; hypothetical protein
OG_04767	LA29479_4669	type III restriction enzyme, res subunit	OG_04767	LA76x_474	LA76x_474; DEAD/DEAH box helicase family protein
OG_04768	LA29479_1563	bacterial regulatory helix-turn-helix , lysR family protein	OG_04768	LA76x_1944	LA76x_1944; bacterial regulatory helix-turn-helix , lysR family protein
OG_04769	LA29479_2672	TENA/THI-4/PQQC family protein	OG_04769	LA76x_598	LA76x_598; TENA/THI-4/PQQC family protein
OG_04770	LA29479_4110	antibiotic biosynthesis monooxygenase family protein	OG_04770	LA76x_343	LA76x_343; antibiotic biosynthesis monooxygenase family protein
OG_04771	LA29479_4636	putative exported protein	OG_04771	LA76x_3446	LA76x_3446; RHS repeat-associated core domain protein
OG_04772	LA29479_2839	marR family protein	OG_04772	LA76x_646	LA76x_646; marR family protein
OG_04774	LA29479_2114	antibiotic biosynthesis monooxygenase family protein	OG_04774	LA76x_1038	LA76x_1038; antibiotic biosynthesis monooxygenase family protein
OG_04775	LA29479_2295	hypothetical protein	OG_04775	LA76x_4358	LA76x_4358; hypothetical protein
OG_04776	LA29479_3970	hypothetical protein	OG_04776	LA76x_802	LA76x_802; hypothetical protein
OG_04777	LA29479_5101	hypothetical protein	OG_04777	LA76x_479	LA76x_479; hypothetical protein
OG_04778	LA29479_3291	demethylmenaquinone methyltransferase	OG_04778	LA76x_192	LA76x_192; demethylmenaquinone methyltransferase family protein
OG_04779	LA29479_2128	hypothetical protein	OG_04779	LA76x_1024	LA76x_1024; hypothetical protein
OG_04780	LA29479_4245	glucokinase	OG_04780	LA76x_1757	glk; glucokinase
OG_04782	LA29479_2314	hypothetical protein	OG_04782	LA76x_4377	LA76x_4377; hypothetical protein
OG_04783	LA29479_4242	PEP-utilising enzyme, mobile domain protein	OG_04783	LA76x_1760	LA76x_1760; pyruvate phosphate dikinase, PEP/pyruvate binding do
OG_04784	LA29479_2367	phenazine biosynthesis protein PhzD	OG_04784	LA76x_3334	phzD1; phenazine biosynthesis protein PhzD

OG_04785	LA29479_4238	hypothetical protein
OG_04786	LA29479_2110	putative uncharacterized domain protein
OG_04787	LA29479_3876	hypothetical protein
OG_04788	LA29479_4397	hypothetical protein
OG_04789	LA29479_3829	tetratricopeptide repeat family protein
OG_04790	LA29479_4499	hypothetical protein
OG_04791	LA29479_3821	hypothetical protein
OG_04792	LA29479_331	ATP dependent DNA ligase domain protein
OG_04794	LA29479_45	putative uncharacterized protein
OG_04795	LA29479_335	putative uncharacterized protein
OG_04796	LA29479_2059	hypothetical protein
OG_04797	LA29479_2963	hypothetical protein
OG_04798	LA29479_2696	putative membrane protein
OG_04799	LA29479_862	hypothetical protein
OG_04800	LA29479_2852	hypothetical protein
OG_04801	LA29479_348	sensory box protein
OG_04802	LA29479_33	hypothetical protein
OG_04803	LA29479_2108	hypothetical protein
OG_04804	LA29479_72	hypothetical protein
OG_04805	LA29479_1813	hypothetical protein
OG_04806	LA29479_2364	phenazine biosynthesis protein phzG
OG_04807	LA29479_1800	gene 25-like lysozyme family protein
OG_04808	LA29479_2879	ricin-type beta-trefoil lectin domain protein
OG_04809	LA29479_2444	hypothetical protein
OG_04810	LA29479_1103	hypothetical protein
OG_04811	LA29479_2617	dioxygenase family protein
OG_04813	LA29479_2782	hypothetical protein
OG_04814	LA29479_38	putative membrane protein
OG_04816	LA29479_4481	hypothetical protein
OG_04817	LA29479_1713	homoserine dehydrogenase family protein
OG_04818	LA29479_5100	prophage CP4-57 regulatory family protein
OG_04819	LA29479_389	smpA / OmlA family protein
OG_04820	LA29479_5070	hypothetical protein
OG_04821	LA29479_596	putative uncharacterized protein
OG_04822	LA29479_1382	hypothetical protein
OG_04823	LA29479_2802	hypothetical protein
OG_04824	LA29479_1727	hypothetical protein
OG_04825	LA29479_1148	proteophosphoglycan ppg4 domain protein
OG_04826	LA29479_86	hypothetical protein
OG_04827	LA29479_1107	CHRD domain containing protein
OG_04829	LA29479_1305	hypothetical protein
OG_04830	LA29479_1810	hypothetical protein
OG_04831	LA29479_3778	hypothetical protein
OG_04832	LA29479_4321	hypothetical protein
OG_04833	LA29479_4412	putative uncharacterized domain protein
OG_04834	LA29479_1	putative lipoprotein
OG_04836	LA29479_10	hypothetical protein
OG_04837	LA29479_386	hypothetical protein
OG_04838	LA29479_337	hypothetical protein
OG_04839	LA29479_3513	hypothetical protein
OG_04841	LA29479_3485	hypothetical protein
OG_04842	LA29479_3070	BNR/Asp-box repeat family protein
OG_04843	LA29479_617	hypothetical protein
OG_04844	LA29479_1285	hypothetical protein
OG_04845	LA29479_4468	hypothetical protein
OG_04846	LA29479_2740	hypothetical protein
OG_04847	LA29479_1607	hypothetical protein
OG_04848	LA29479_2494	hypothetical protein
OG_04849	LA29479_3307	putative uncharacterized protein
OG_04850	LA29479_2623	putative uncharacterized protein
OG_04851	LA29479_2369	hypothetical protein
OG_04852	LA29479_3262	hypothetical protein
OG_04853	LA29479_2391	hypothetical protein
OG_04854	LA29479_5164	hypothetical protein
OG_04855	LA29479_2187	hypothetical protein
OG_04856	LA29479_428	beta-lactamase family protein
OG_04857	LA29479_1803	putative uncharacterized protein
OG_04858	LA29479_4341	hypothetical protein
OG_04859	LA29479_1313	hypothetical protein
OG_04861	LA29479_4595	putative ATP-dependent RNA helicase HrpA
OG_04862	LA29479_414	hypothetical protein
OG_04863	LA29479_1897	ABC transporter, permease/ATP-binding protein
OG_04864	LA29479_1026	hypothetical protein
OG_04865	LA29479_2460	hypothetical protein
OG_04866	LA29479_869	hypothetical protein
OG_04867	LA29479_4761	hypothetical protein
OG_04868	LA29479_2673	aminotransferase class IV family protein
OG_04869	LA29479_4208	hypothetical protein
OG_04870	LA29479_3079	hypothetical protein
OG_04871	LA29479_2524	mll3908 protein
OG_04872	LA29479_479	hypothetical protein
OG_04873	LA29479_1797	putative uncharacterized protein

OG_04785	LA76x_1765	LA76x_1765; hypothetical protein
OG_04786	LA76x_1042	LA76x_1042; glycosyl hydrolase 71 family protein
OG_04787	LA76x_3577	LA76x_3577; hypothetical protein
OG_04788	LA76x_519	LA76x_519; hypothetical protein
OG_04789	LA76x_4741	LA76x_4741; phosphotransferase enzyme family protein
OG_04790	LA76x_4659	LA76x_4659; hypothetical protein
OG_04791	LA76x_4749	LA76x_4749; hypothetical protein
OG_04792	LA76x_5113	LA76x_5113; ATP dependent DNA ligase domain protein
OG_04794	LA76x_3142	LA76x_3142; hypothetical protein
OG_04795	LA76x_5109	LA76x_5109; conserved hypothetical protein
OG_04796	LA76x_1093	LA76x_1093; hypothetical protein
OG_04797	LA76x_2685	LA76x_2685; hypothetical protein
OG_04798	LA76x_623	LA76x_623; conserved hypothetical protein
OG_04799	LA76x_3701	LA76x_3701; hypothetical protein
OG_04800	LA76x_659	LA76x_659; hypothetical protein
OG_04801	LA76x_5096	LA76x_5096; diguanylate cyclase domain protein
OG_04802	LA76x_3154	LA76x_3154; hypothetical protein
OG_04803	LA76x_1044	LA76x_1044; hypothetical protein
OG_04804	LA76x_3116	LA76x_3116; hypothetical protein
OG_04805	LA76x_2813	LA76x_2813; hypothetical protein
OG_04806	LA76x_3331	phzG; phzG
OG_04807	LA76x_2826	LA76x_2826; gene 25-like lysozyme family protein
OG_04808	LA76x_684	LA76x_684; ricin-type beta-trefoil lectin domain protein
OG_04809	LA76x_3410	LA76x_3410; hypothetical protein
OG_04810	LA76x_2539	LA76x_2539; hypothetical protein
OG_04811	LA76x_546	LA76x_546; dioxygenase family protein
OG_04813	LA76x_2351	LA76x_2351; hypothetical protein
OG_04814	LA76x_3149	LA76x_3149; conserved hypothetical protein
OG_04816	LA76x_4678	LA76x_4678; hypothetical protein
OG_04817	LA76x_2119	LA76x_2119; homoserine dehydrogenase family protein
OG_04818	LA76x_478	LA76x_478; prophage CP4-57 regulatory family protein
OG_04819	LA76x_5058	LA76x_5058; smpA / OmlA family protein
OG_04820	LA76x_4808	LA76x_4808; hypothetical protein
OG_04821	LA76x_1671	LA76x_1671; glycosyltransferase family protein
OG_04822	LA76x_5196	LA76x_5196; hypothetical protein
OG_04823	LA76x_2331	LA76x_2331; hypothetical protein
OG_04824	LA76x_2107	LA76x_2107; hypothetical protein
OG_04825	LA76x_2496	LA76x_2496; proteophosphoglycan ppg4 domain protein
OG_04826	LA76x_3102	LA76x_3102; hypothetical protein
OG_04827	LA76x_2536	LA76x_2536; CHRD domain protein
OG_04829	LA76x_68	LA76x_68; hypothetical protein
OG_04830	LA76x_2816	LA76x_2816; hypothetical protein
OG_04831	LA76x_4787	LA76x_4787; hypothetical protein
OG_04832	LA76x_1115	LA76x_1115; hypothetical protein
OG_04833	LA76x_505	LA76x_505; conserved hypothetical protein
OG_04834	LA76x_3183	LA76x_3183; hypothetical protein
OG_04836	LA76x_3174	LA76x_3174; hypothetical protein
OG_04837	LA76x_5061	LA76x_5061; hypothetical protein
OG_04838	LA76x_5107	LA76x_5107; hypothetical protein
OG_04839	LA76x_2723	LA76x_2723; von Willebrand factor type A domain protein
OG_04841	LA76x_4268	LA76x_4268; hypothetical protein
OG_04842	LA76x_884	LA76x_884; BNR repeat-like domain protein
OG_04843	LA76x_1657	LA76x_1657; hypothetical protein
OG_04844	LA76x_86	LA76x_86; hypothetical protein
OG_04845	LA76x_3028	LA76x_3028; hypothetical protein
OG_04846	LA76x_2394	LA76x_2394; hypothetical protein
OG_04847	LA76x_2227	LA76x_2227; hypothetical protein
OG_04848	LA76x_4447	LA76x_4447; hypothetical protein
OG_04849	LA76x_177	LA76x_177; conserved hypothetical protein
OG_04850	LA76x_550	LA76x_550; conserved hypothetical protein
OG_04851	LA76x_3336	LA76x_3336; hypothetical protein
OG_04852	LA76x_218	LA76x_218; hypothetical protein
OG_04853	LA76x_3360	LA76x_3360; hypothetical protein
OG_04854	LA76x_834	LA76x_834; hypothetical protein
OG_04855	LA76x_968	LA76x_968; hypothetical protein
OG_04856	LA76x_5024	LA76x_5024; beta-lactamase family protein
OG_04857	LA76x_2823	LA76x_2823; conserved hypothetical protein
OG_04858	LA76x_3373	LA76x_3373; hypothetical protein
OG_04859	LA76x_59	LA76x_59; hypothetical protein
OG_04861	LA76x_4091	LA76x_4091; conserved hypothetical protein
OG_04862	LA76x_5039	LA76x_5039; hypothetical protein
OG_04863	LA76x_1397	LA76x_1397; putative aBC transporter, permease/ATP-binding protei
OG_04864	LA76x_2611	LA76x_2611; trypsin-like peptidase domain protein
OG_04865	LA76x_3424	LA76x_3424; hypothetical protein
OG_04866	LA76x_3708	LA76x_3708; hypothetical protein
OG_04867	LA76x_2271	LA76x_2271; conserved hypothetical protein
OG_04868	LA76x_599	LA76x_599; aminotransferase class IV family protein
OG_04869	LA76x_4199	LA76x_4199; hypothetical protein
OG_04870	LA76x_892	LA76x_892; hypothetical protein
OG_04871	LA76x_4479	LA76x_4479; conserved hypothetical protein
OG_04872	LA76x_4879	LA76x_4879; hypothetical protein
OG_04873	LA76x_2829	LA76x_2829; conserved hypothetical protein

OG_04874	LA29479_2727	hypothetical protein
OG_04875	LA29479_2977	hypothetical protein
OG_04876	LA29479_2374	antibiotic biosynthesis monooxygenase family protein
OG_04877	LA29479_63	hypothetical protein
OG_04878	LA29479_4637	putative uncharacterized protein
OG_04879	LA29479_5080	hydrolase CocE/NonD family domain protein
OG_04880	LA29479_2370	hypothetical protein
OG_04881	LA29479_338	hypothetical protein
OG_04882	LA29479_103	hypothetical protein
OG_04883	LA29479_4042	hypothetical protein
OG_04884	LA29479_2663	putative membrane protein
OG_04885	LA29479_2085	hypothetical protein
OG_04886	LA29479_193	hypothetical protein
OG_04887	LA29479_4178	hypothetical protein
OG_04888	LA29479_1021	hypothetical protein
OG_04889	LA29479_506	hypothetical protein
OG_04890	LA29479_4053	putative membrane protein
OG_04891	LA29479_2674	hypothetical protein
OG_04892	LA29479_3793	hypothetical protein
OG_04893	LA29479_4149	putative membrane protein
OG_04894	LA29479_4103	rimK-like ATP-grasp domain protein
OG_04895	LA29479_1855	hypothetical protein
OG_04896	LA29479_3576	hypothetical protein
OG_04897	LA29479_5103	hypothetical protein
OG_04898	LA29479_353	merR regulatory family protein
OG_04899	LA29479_2377	hypothetical protein
OG_04900	LA29479_3126	hypothetical protein
OG_04901	LA29479_2633	hypothetical protein
OG_04902	LA29479_3873	tonB-dependent siderophore receptor family protein
OG_04903	LA29479_3777	hypothetical protein
OG_04905	LA29479_2200	putative Insecticial toxin domain protein
OG_04906	LA29479_330	hypothetical protein
OG_04907	LA29479_356	putative uncharacterized protein
OG_04908	LA29479_677	hypothetical protein
OG_04909	LA29479_4778	hypothetical protein
OG_04910	LA29479_2388	hypothetical protein
OG_04911	LA29479_1806	hypothetical protein
OG_04912	LA29479_3294	asnC family protein
OG_04913	LA29479_3346	hypothetical protein
OG_04914	LA29479_2706	bacterial regulatory s, gntR family protein
OG_04915	LA29479_52	hypothetical protein
OG_04916	LA29479_145	hypothetical protein
OG_04917	LA29479_3589	hypothetical protein
OG_04918	LA29479_1783	hypothetical protein
OG_04919	LA29479_1805	putative uncharacterized protein
OG_04920	LA29479_1968	hypothetical protein
OG_04921	LA29479_1027	putative uncharacterized domain protein
OG_04923	LA29479_3847	hypothetical protein
OG_04924	LA29479_438	hypothetical protein
OG_04925	LA29479_4400	hypothetical protein
OG_04927	LA29479_2863	putative uncharacterized protein
OG_04929	LA29479_4639	hypothetical protein
OG_04930	LA29479_2855	putative membrane protein
OG_04931	LA29479_4633	hypothetical protein
OG_04932	LA29479_169	acetyltransferase family protein
OG_04933	LA29479_3068	glutaminase 1
OG_04934	LA29479_1860	hypothetical protein
OG_04937	LA29479_146	hypothetical protein
OG_04938	LA29479_1811	ATPase associated with various cellular activities family protein
OG_04939	LA29479_1246	hypothetical protein
OG_04940	LA29479_1930	putative uncharacterized protein
OG_04941	LA29479_271	hypothetical protein
OG_04942	LA29479_350	hypothetical protein
OG_04944	LA29479_344	metallo-beta-lactamase superfamily protein
OG_04945	LA29479_2258	hypothetical protein
OG_04946	LA29479_36	hypothetical protein
OG_04947	LA29479_1801	putative uncharacterized protein
OG_04948	LA29479_35	hypothetical protein
OG_04949	LA29479_387	hypothetical protein
OG_04950	LA29479_470	hypothetical protein
OG_04952	LA29479_1604	hypothetical protein
OG_04953	LA29479_464	hypothetical protein
OG_04954	LA29479_5188	hypothetical protein
OG_04955	LA29479_4803	exported serine endopeptidase
OG_04956	LA29479_4380	hypothetical protein
OG_04957	LA29479_138	hypothetical protein
OG_04958	LA29479_2668	hypothetical protein
OG_04959	LA29479_241	hypothetical protein
OG_04960	LA29479_2881	ricin-type beta-trefoil lectin domain protein
OG_04961	LA29479_1050	hypothetical protein
OG_04962	LA29479_175	hypothetical protein
OG_04963	LA29479_339	lysM domain protein

OG_04874	LA76x_2408	LA76x_2408; hypothetical protein
OG_04875	LA76x_2671	LA76x_2671; hypothetical protein
OG_04876	LA76x_3341	LA76x_3341; antibiotic biosynthesis monooxygenase family protein
OG_04877	LA76x_3125	LA76x_3125; hypothetical protein
OG_04878	LA76x_3447	LA76x_3447; chitinase A, N-terminal domain protein
OG_04879	LA76x_3257	LA76x_3257; hydrolase CocE/NonD family protein
OG_04880	LA76x_3337	LA76x_3337; hypothetical protein
OG_04881	LA76x_5106	LA76x_5106; hypothetical protein
OG_04882	LA76x_3084	LA76x_3084; hypothetical protein
OG_04883	LA76x_286	LA76x_286; hypothetical protein
OG_04884	LA76x_587	LA76x_587; conserved hypothetical protein
OG_04885	LA76x_1067	LA76x_1067; hypothetical protein
OG_04886	LA76x_2997	LA76x_2997; hypothetical protein
OG_04887	LA76x_4172	LA76x_4172; hypothetical protein
OG_04888	LA76x_2622	LA76x_2622; hypothetical protein
OG_04889	LA76x_4853	LA76x_4853; hypothetical protein
OG_04890	LA76x_274	LA76x_274; hypothetical protein
OG_04891	LA76x_600	LA76x_600; hypothetical protein
OG_04892	LA76x_4772	LA76x_4772; hypothetical protein
OG_04893	LA76x_305	LA76x_305; hypothetical protein
OG_04894	LA76x_350	LA76x_350; ATP-grasp domain protein
OG_04895	LA76x_2775	LA76x_2775; hypothetical protein
OG_04896	LA76x_3984	LA76x_3984; hypothetical protein
OG_04897	LA76x_481	LA76x_481; hypothetical protein
OG_04898	LA76x_5091	LA76x_5091; merR regulatory family protein
OG_04899	LA76x_3344	LA76x_3344; hypothetical protein
OG_04900	LA76x_938	LA76x_938; hypothetical protein
OG_04901	LA76x_560	LA76x_560; hypothetical protein
OG_04902	LA76x_3573	LA76x_3573; tonB-dependent siderophore receptor family protein
OG_04903	LA76x_4788	LA76x_4788; hypothetical protein
OG_04905	LA76x_957	LA76x_957; putative Insecticial toxin domain protein
OG_04906	LA76x_5114	LA76x_5114; hypothetical protein
OG_04907	LA76x_5088	LA76x_5088; conserved hypothetical protein
OG_04908	LA76x_1597	LA76x_1597; hypothetical protein
OG_04909	LA76x_4123	LA76x_4123; hypothetical protein
OG_04910	LA76x_3357	LA76x_3357; hypothetical protein
OG_04911	LA76x_2820	LA76x_2820; hypothetical protein
OG_04912	LA76x_189	LA76x_189; asnC family protein
OG_04913	LA76x_3871	LA76x_3871; hypothetical protein
OG_04914	LA76x_2429	LA76x_2429; deoR C terminal sensor domain protein
OG_04915	LA76x_3135	LA76x_3135; hypothetical protein
OG_04916	LA76x_3043	LA76x_3043; hypothetical protein
OG_04917	LA76x_3998	LA76x_3998; hypothetical protein
OG_04918	LA76x_2846	LA76x_2846; hypothetical protein
OG_04919	LA76x_2821	LA76x_2821; conserved hypothetical protein
OG_04920	LA76x_1233	LA76x_1233; hypothetical protein
OG_04921	LA76x_2612	LA76x_2612; PGAP1-like family protein
OG_04923	LA76x_3547	LA76x_3547; hypothetical protein
OG_04924	LA76x_5015	LA76x_5015; hypothetical protein
OG_04925	LA76x_517	LA76x_517; hypothetical protein
OG_04927	LA76x_668	LA76x_668; conserved hypothetical protein
OG_04929	LA76x_3448	LA76x_3448; hypothetical protein
OG_04930	LA76x_660	LA76x_660; conserved hypothetical protein
OG_04931	LA76x_3444	LA76x_3444; hypothetical protein
OG_04932	LA76x_3021	LA76x_3021; acetyltransferase family protein
OG_04933	LA76x_882	glsA; glutaminase A
OG_04934	LA76x_2770	LA76x_2770; hypothetical protein
OG_04937	LA76x_3042	LA76x_3042; hypothetical protein
OG_04938	LA76x_2815	LA76x_2815; ATPase associated with various cellular activities family
OG_04939	LA76x_125	LA76x_125; hypothetical protein
OG_04940	LA76x_1362	LA76x_1362; conserved hypothetical protein
OG_04941	LA76x_5174	LA76x_5174; hypothetical protein
OG_04942	LA76x_5094	LA76x_5094; hypothetical protein
OG_04944	LA76x_5100	LA76x_5100; metallo-beta-lactamase superfamily protein
OG_04945	LA76x_4324	LA76x_4324; hypothetical protein
OG_04946	LA76x_3151	LA76x_3151; hypothetical protein
OG_04947	LA76x_2825	LA76x_2825; conserved hypothetical protein
OG_04948	LA76x_3152	LA76x_3152; hypothetical protein
OG_04949	LA76x_5060	LA76x_5060; hypothetical protein
OG_04950	LA76x_4888	LA76x_4888; hypothetical protein
OG_04952	LA76x_2230	LA76x_2230; hypothetical protein
OG_04953	LA76x_4895	LA76x_4895; hypothetical protein
OG_04954	LA76x_4799	LA76x_4799; hypothetical protein
OG_04955	LA76x_3265	LA76x_3265; trypsin family protein
OG_04956	LA76x_1457	LA76x_1457; hypothetical protein
OG_04957	LA76x_3049	LA76x_3049; hypothetical protein
OG_04958	LA76x_592	LA76x_592; hypothetical protein
OG_04959	LA76x_2948	LA76x_2948; hypothetical protein
OG_04960	LA76x_685	LA76x_685; ricin-type beta-trefoil lectin domain protein
OG_04961	LA76x_2592	LA76x_2592; hypothetical protein
OG_04962	LA76x_3015	LA76x_3015; hypothetical protein
OG_04963	LA76x_5105	LA76x_5105; lysM domain protein

OG_04964	LA29479_3483	hypothetical protein
OG_04965	LA29479_1807	hypothetical protein
OG_04966	LA29479_2442	FKBP-type peptidyl-prolyl cis-trans isomerase family protein
OG_04967	LA29479_46	hypothetical protein
OG_04968	LA29479_97	alpha/beta hydrolase fold family protein
OG_04969	LA29479_2840	conserved hypothetical protein
OG_04970	LA29479_4575	hypothetical protein
OG_04971	LA29479_901	oxidoreductase, short-chain dehydrogenase/reductase family
OG_04972	LA29479_5194	flagellar domain protein
OG_04973	LA29479_4842	putative lipoprotein
OG_04974	LA29479_3217	hypothetical protein
OG_04975	LA29479_2182	putative uncharacterized protein
OG_04976	LA29479_3828	RNA polymerase sigma factor, TIGR02999 family protein
OG_04977	LA29479_1143	hypothetical protein
OG_04978	LA29479_3860	hypothetical protein
OG_04979	LA29479_2505	hypothetical protein
OG_04980	LA29479_233	putative uncharacterized protein
OG_04981	LA29479_22	hypothetical protein
OG_04982	LA29479_4758	hypothetical protein
OG_04983	LA29479_4998	hypothetical protein
OG_04984	LA29479_329	response regulator
OG_04985	LA29479_1927	putative uncharacterized protein
OG_04986	LA29479_3058	putative membrane protein
OG_04987	LA29479_2676	putative membrane protein
OG_04988	LA29479_177	hypothetical protein
OG_04990	LA29479_268	hypothetical protein
OG_04991	LA29479_2180	fatty acid desaturase family protein
OG_04992	LA29479_3116	hypothetical protein
OG_04994	LA29479_1762	hypothetical protein
OG_04995	LA29479_1902	hypothetical protein
OG_04996	LA29479_816	bacterial regulatory helix-turn-helix s, AraC family protein
OG_04997	LA29479_2365	putative uncharacterized protein
OG_04998	LA29479_3822	hypothetical protein
OG_04999	LA29479_4013	hypothetical protein
OG_05000	LA29479_2613	hypothetical protein
OG_05001	LA29479_3587	hypothetical protein
OG_05002	LA29479_2008	degT/DnrJ/EryC1/StrS aminotransferase family protein
OG_05003	LA29479_2448	putative lipoprotein
OG_05004	LA29479_498	putative membrane protein
OG_05005	LA29479_2828	general secretion pathway protein A
OG_05006	LA29479_1024	hypothetical protein
OG_05007	LA29479_2665	hypothetical protein
OG_05009	LA29479_2871	glutathione-dependent formaldehyde-activating enzyme family protein
OG_05010	LA29479_3007	alpha/beta hydrolase fold family protein
OG_05011	LA29479_5057	hypothetical protein
OG_05012	LA29479_369	peptidase M23 family protein
OG_05014	LA29479_2856	hypothetical protein
OG_05015	LA29479_347	marR family protein
OG_05016	LA29479_887	hypothetical protein
OG_05017	LA29479_5056	hypothetical protein
OG_05018	LA29479_5098	putative phage-type endonuclease domain protein
OG_05019	LA29479_2175	putative uncharacterized domain protein
OG_05020	LA29479_2078	methyltransferase, FkbM family domain protein
OG_05021	LA29479_2688	hypothetical protein
OG_05022	LA29479_65	putative uncharacterized protein
OG_05023	LA29479_2895	trbL/VirB6 plasmid conjugal transfer family protein
OG_05024	LA29479_2632	hypothetical protein
OG_05026	LA29479_3225	hypothetical protein
OG_05027	LA29479_2537	hypothetical protein
OG_05028	LA29479_2373	hypothetical protein
OG_05029	LA29479_2675	hypothetical protein
OG_05030	LA29479_2157	hypothetical protein
OG_05032	LA29479_3228	amidohydrolase family protein
OG_05033	LA29479_2827	hypothetical protein
OG_05034	LA29479_2955	hypothetical protein
OG_05035	LA29479_2438	RNA polymerase sigma factor, sigma-70 family protein
OG_05036	LA29479_3293	lysE type translocator family protein
OG_05037	LA29479_2437	fecR family protein
OG_05038	LA29479_637	hypothetical protein
OG_05041	LA29479_595	bacterial regulatory s, tetR family protein
OG_05042	LA29479_400	hypothetical protein
OG_05043	LA29479_618	hypothetical protein
OG_05044	LA29479_3412	hypothetical protein
OG_05046	LA29479_2703	hypothetical protein
OG_05047	LA29479_116	bacterial regulatory s, tetR family protein
OG_05048	LA29479_1065	penicillinase repressor family protein
OG_05049	LA29479_2411	conserved hypothetical protein
OG_05050	LA29479_247	hypothetical protein
OG_05051	LA29479_246	glycoprotein endo-alpha-1,2-mannosidase
OG_05052	LA29479_1480	hypothetical protein
OG_05053	LA29479_788	hypothetical protein
OG_05054	LA29479_3787	hypothetical protein

OG_04964	LA76x_4266	LA76x_4266; hypothetical protein
OG_04965	LA76x_2819	LA76x_2819; hypothetical protein
OG_04966	LA76x_3408	LA76x_3408; FKBP-type peptidyl-prolyl cis-trans isomerase family pr
OG_04967	LA76x_3141	LA76x_3141; helix-turn-helix family protein
OG_04968	LA76x_3091	LA76x_3091; alpha/beta hydrolase fold family protein
OG_04969	LA76x_647	LA76x_647; eamA-like transporter family protein
OG_04970	LA76x_1425	LA76x_1425; hypothetical protein
OG_04971	LA76x_3741	LA76x_3741; oxidoreductase, short-chain dehydrogenase/reductase
OG_04972	LA76x_1702	LA76x_1702; 6-phosphofructokinase domain protein
OG_04973	LA76x_3310	LA76x_3310; hypothetical protein
OG_04974	LA76x_1808	LA76x_1808; hypothetical protein
OG_04975	LA76x_974	LA76x_974; conserved hypothetical protein
OG_04976	LA76x_4742	LA76x_4742; RNA polymerase sigma factor, TIGR02999 family prote
OG_04977	LA76x_2501	LA76x_2501; hypothetical protein
OG_04978	LA76x_3560	LA76x_3560; hypothetical protein
OG_04979	LA76x_4460	LA76x_4460; hypothetical protein
OG_04980	LA76x_2957	LA76x_2957; conserved hypothetical protein
OG_04981	LA76x_3162	LA76x_3162; hypothetical protein
OG_04982	LA76x_2274	LA76x_2274; hypothetical protein
OG_04983	LA76x_4720	LA76x_4720; hypothetical protein
OG_04984	LA76x_5115	LA76x_5115; bacterial regulatory, luxR family protein
OG_04985	LA76x_1365	LA76x_1365; catalase family protein
OG_04986	LA76x_873	LA76x_873; hypothetical protein
OG_04987	LA76x_602	LA76x_602; sterol-sensing domain of SREBP cleavage-activation fan
OG_04988	LA76x_3013	LA76x_3013; hypothetical protein
OG_04990	LA76x_5177	LA76x_5177; hypothetical protein
OG_04991	LA76x_976	LA76x_976; fatty acid desaturase family protein
OG_04992	LA76x_928	LA76x_928; hypothetical protein
OG_04994	LA76x_2867	LA76x_2867; conserved hypothetical protein
OG_04995	LA76x_1392	LA76x_1392; hypothetical protein
OG_04996	LA76x_3658	LA76x_3658; bacterial regulatory helix-turn-helix, AraC family protein
OG_04997	LA76x_3332	LA76x_3332; conserved hypothetical protein
OG_04998	LA76x_4748	LA76x_4748; hypothetical protein
OG_04999	LA76x_4600	LA76x_4600; hypothetical protein
OG_05000	LA76x_540	LA76x_540; hypothetical protein
OG_05001	LA76x_3996	LA76x_3996; hypothetical protein
OG_05002	LA76x_1194	LA76x_1194; degT/DnrJ/EryC1/StrS aminotransferase family protein
OG_05003	LA76x_3412	LA76x_3412; hypothetical protein
OG_05004	LA76x_4861	LA76x_4861; conserved hypothetical protein
OG_05005	LA76x_2305	LA76x_2305; putative peptidoglycan binding domain protein
OG_05006	LA76x_2609	LA76x_2609; hypothetical protein
OG_05007	LA76x_589	LA76x_589; calpain cysteine protease family protein
OG_05009	LA76x_676	LA76x_676; glutathione-dependent formaldehyde-activating enzyme
OG_05010	LA76x_2641	LA76x_2641; alpha/beta hydrolase fold family protein
OG_05011	LA76x_4819	LA76x_4819; hypothetical protein
OG_05012	LA76x_5075	LA76x_5075; peptidase M23 family protein
OG_05014	LA76x_661	LA76x_661; hypothetical protein
OG_05015	LA76x_5097	LA76x_5097; marR family protein
OG_05016	LA76x_3727	LA76x_3727; hypothetical protein
OG_05017	LA76x_4820	LA76x_4820; hypothetical protein
OG_05018	LA76x_476	LA76x_476; putative phage-type endonuclease domain protein
OG_05019	LA76x_981	LA76x_981; conserved hypothetical protein
OG_05020	LA76x_1074	LA76x_1074; methyltransferase, FkbM family domain protein
OG_05021	LA76x_615	LA76x_615; hypothetical protein
OG_05022	LA76x_3123	LA76x_3123; conserved hypothetical protein
OG_05023	LA76x_699	LA76x_699; trbL/VirB6 plasmid conjugal transfer family protein
OG_05024	LA76x_559	LA76x_559; hypothetical protein
OG_05026	LA76x_1801	LA76x_1801; hypothetical protein
OG_05027	LA76x_4492	LA76x_4492; hypothetical protein
OG_05028	LA76x_3340	LA76x_3340; hypothetical protein
OG_05029	LA76x_601	LA76x_601; conserved hypothetical protein
OG_05030	LA76x_995	LA76x_995; hypothetical protein
OG_05032	LA76x_1798	LA76x_1798; amidohydrolase family protein
OG_05033	LA76x_2306	LA76x_2306; hypothetical protein
OG_05034	LA76x_746	LA76x_746; hypothetical protein
OG_05035	LA76x_3405	LA76x_3405; RNA polymerase sigma factor, sigma-70 family protein
OG_05036	LA76x_190	LA76x_190; lysE type translocator family protein
OG_05037	LA76x_3404	LA76x_3404; fecR family protein
OG_05038	LA76x_1638	LA76x_1638; hypothetical protein
OG_05041	LA76x_1672	LA76x_1672; bacterial regulatory, tetR family protein
OG_05042	LA76x_5049	LA76x_5049; hypothetical protein
OG_05043	LA76x_1656	LA76x_1656; hypothetical protein
OG_05044	LA76x_3938	LA76x_3938; hypothetical protein
OG_05046	LA76x_630	LA76x_630; hypothetical protein
OG_05047	LA76x_3070	LA76x_3070; bacterial regulatory, tetR family protein
OG_05048	LA76x_2577	LA76x_2577; penicillinase repressor family protein
OG_05049	LA76x_3381	LA76x_3381; conserved hypothetical protein
OG_05050	LA76x_2942	LA76x_2942; hypothetical protein
OG_05051	LA76x_2943	MANEA; glycoprotein endo-alpha-1,2-mannosidase
OG_05052	LA76x_2023	LA76x_2023; hypothetical protein
OG_05053	LA76x_3633	LA76x_3633; hypothetical protein
OG_05054	LA76x_4777	LA76x_4777; hypothetical protein

OG_05055	LA29479_4581	hypothetical protein
OG_05056	LA29479_101	mauG protein
OG_05058	LA29479_1379	hypothetical protein
OG_05059	LA29479_2362	phenazine biosynthesis protein phzB 2
OG_05060	LA29479_1804	putative uncharacterized protein
OG_05061	LA29479_2874	hypothetical protein
OG_05062	LA29479_83	hypothetical protein
OG_05063	LA29479_427	hypothetical protein
OG_05064	LA29479_2177	putative uncharacterized domain protein
OG_05065	LA29479_2683	hypothetical protein
OG_05066	LA29479_2464	hypothetical protein
OG_05067	LA29479_4878	hypothetical protein
OG_05068	LA29479_2573	NIPSNAP family protein
OG_05069	LA29479_1337	hypothetical protein
OG_05070	LA29479_87	putative uncharacterized protein
OG_05071	LA29479_1522	hypothetical protein
OG_05072	LA29479_3148	hypothetical protein
OG_05075	LA29479_2109	hypothetical protein
OG_05076	LA29479_4850	hypothetical protein
OG_05077	LA29479_2664	hypothetical protein
OG_05078	LA29479_4601	hypothetical protein
OG_05079	LA29479_5153	putative uncharacterized protein
OG_05081	LA29479_5051	hypothetical protein
OG_05082	LA29479_5079	putative uncharacterized domain protein
OG_05083	LA29479_569	hypothetical protein
OG_05084	LA29479_4526	hypothetical protein
OG_05085	LA29479_873	hypothetical protein
OG_05086	LA29479_2363	phenazine biosynthesis protein phzF
OG_05087	LA29479_1569	hypothetical protein
OG_05089	LA29479_2608	hypothetical protein
OG_05090	LA29479_567	hypothetical protein
OG_05091	LA29479_3463	hypothetical protein
OG_05092	LA29479_529	putative lipoprotein
OG_05093	LA29479_47	hypothetical protein
OG_05094	LA29479_1064	hypothetical protein
OG_05095	LA29479_599	hypothetical protein
OG_05096	LA29479_4336	hypothetical protein
OG_05097	LA29479_2846	hypothetical protein
OG_05098	LA29479_2288	hypothetical protein
OG_05099	LA29479_3342	hypothetical protein
OG_05100	LA29479_2682	hypothetical protein
OG_05101	LA29479_1815	conserved hypothetical protein
OG_05102	LA29479_1274	putative uncharacterized protein
OG_05104	LA29479_341	putative membrane protein
OG_05105	LA29479_3020	hypothetical protein
OG_05106	LA29479_1666	hypothetical protein
OG_05107	LA29479_3309	hypothetical protein
OG_05108	LA29479_444	hypothetical protein
OG_05109	LA29479_1536	hypothetical protein
OG_05110	LA29479_1407	hypothetical protein
OG_05111	LA29479_1798	peptidoglycan-binding lysin domain protein
OG_05112	LA29479_1108	hypothetical protein
OG_05113	LA29479_1922	putative uncharacterized protein
OG_05114	LA29479_4779	hypothetical protein
OG_05115	LA29479_2361	hypothetical protein
OG_05117	LA29479_3030	hypothetical protein
OG_05118	LA29479_3618	hypothetical protein
OG_05119	LA29479_2678	phenylacetic acid catabolic family protein
OG_05120	LA29479_64	leupeptin-inactivating enzyme 1
OG_05121	LA29479_1662	hypothetical protein
OG_05122	LA29479_2381	hypothetical protein
OG_05123	LA29479_4743	putative lipoprotein
OG_05124	LA29479_2181	3-Oxoacyl-[acyl-carrier-(ACP)] synthase III C terminal family protein
OG_05125	LA29479_1802	putative uncharacterized protein
OG_05127	LA29479_3278	hypothetical protein
OG_05128	LA29479_2669	hypothetical protein
OG_05129	LA29479_1814	putative uncharacterized protein
OG_05130	LA29479_2414	hypothetical protein
OG_05131	LA29479_1429	putative uncharacterized protein
OG_05132	LA29479_3181	avidin family protein
OG_05133	LA29479_774	hypothetical protein
OG_05134	LA29479_1809	putative uncharacterized protein
OG_05136	LA29479_2859	conserved hypothetical protein
OG_05137	LA29479_4635	hypothetical protein
OG_05138	LA29479_2376	cyclohexanone monooxygenase
OG_05140	LA29479_4101	hypothetical protein
OG_05141	LA29479_5108	bacterial extracellular solute-binding family protein
OG_05142	LA29479_2677	hypothetical protein
OG_05143	LA29479_5156	hypothetical protein
OG_05144	LA29479_2694	putative uncharacterized domain protein
OG_05145	LA29479_81	hypothetical protein
OG_05146	LA29479_810	methyltransferase domain protein

OG_05055	LA76x_1417	LA76x_1417; hypothetical protein
OG_05056	LA76x_3086	LA76x_3086; putative mauG protein
OG_05058	LA76x_5199	LA76x_5199; hypothetical protein
OG_05059	LA76x_3329	LA76x_3329; phenazine biosynthesis protein A/B
OG_05060	LA76x_2822	LA76x_2822; conserved hypothetical protein
OG_05061	LA76x_679	LA76x_679; conserved hypothetical protein
OG_05062	LA76x_3105	LA76x_3105; hypothetical protein
OG_05063	LA76x_5025	LA76x_5025; hypothetical protein
OG_05064	LA76x_979	LA76x_979; conserved hypothetical protein
OG_05065	LA76x_610	LA76x_610; hypothetical protein
OG_05066	LA76x_3428	LA76x_3428; hypothetical protein
OG_05067	LA76x_3536	LA76x_3536; hypothetical protein
OG_05068	LA76x_4524	LA76x_4524; NIPSNAP family protein
OG_05069	LA76x_35	LA76x_35; hypothetical protein
OG_05070	LA76x_3101	LA76x_3101; conserved hypothetical protein
OG_05071	LA76x_1981	LA76x_1981; hypothetical protein
OG_05072	LA76x_1872	LA76x_1872; hypothetical protein
OG_05075	LA76x_1043	LA76x_1043; hypothetical protein
OG_05076	LA76x_3318	LA76x_3318; hypothetical protein
OG_05077	LA76x_588	LA76x_588; hypothetical protein
OG_05078	LA76x_4096	LA76x_4096; hypothetical protein
OG_05079	LA76x_640	LA76x_640; conserved hypothetical protein
OG_05081	LA76x_4823	LA76x_4823; hypothetical protein
OG_05082	LA76x_3256	LA76x_3256; conserved hypothetical protein
OG_05083	LA76x_1694	LA76x_1694; hypothetical protein
OG_05084	LA76x_1907	LA76x_1907; conserved hypothetical protein
OG_05085	LA76x_3712	LA76x_3712; hypothetical protein
OG_05086	LA76x_3330	LA76x_3330; phenazine biosynthesis , PhzF family protein
OG_05087	LA76x_1938	LA76x_1938; hypothetical protein
OG_05089	LA76x_535	LA76x_535; hypothetical protein
OG_05090	LA76x_1696	LA76x_1696; hypothetical protein
OG_05091	LA76x_4247	LA76x_4247; hypothetical protein
OG_05092	LA76x_4832	LA76x_4832; META domain protein
OG_05093	LA76x_3140	LA76x_3140; hypothetical protein
OG_05094	LA76x_2578	LA76x_2578; hypothetical protein
OG_05095	LA76x_1668	LA76x_1668; hypothetical protein
OG_05096	LA76x_1498	LA76x_1498; hypothetical protein
OG_05097	LA76x_653	LA76x_653; hypothetical protein
OG_05098	LA76x_4352	LA76x_4352; hypothetical protein
OG_05099	LA76x_3867	LA76x_3867; hypothetical protein
OG_05100	LA76x_608	LA76x_608; hypothetical protein
OG_05101	LA76x_2811	LA76x_2811; conserved hypothetical phage tail region family protein
OG_05102	LA76x_99	LA76x_99; KTSC domain protein
OG_05104	LA76x_5103	LA76x_5103; conserved hypothetical protein
OG_05105	LA76x_2632	LA76x_2632; hpt domain protein
OG_05106	LA76x_2169	LA76x_2169; hypothetical protein
OG_05107	LA76x_175	LA76x_175; hypothetical protein
OG_05108	LA76x_4913	LA76x_4913; hypothetical protein
OG_05109	LA76x_1968	LA76x_1968; hypothetical protein
OG_05110	LA76x_2097	LA76x_2097; hypothetical protein
OG_05111	LA76x_2828	LA76x_2828; putative peptidoglycan-binding lysin domain protein
OG_05112	LA76x_2535	LA76x_2535; hypothetical protein
OG_05113	LA76x_1370	LA76x_1370; ribosomal L7/L12 C-terminal domain protein
OG_05114	LA76x_4124	LA76x_4124; hypothetical protein
OG_05115	LA76x_3328	LA76x_3328; hypothetical protein
OG_05117	LA76x_846	LA76x_846; hypothetical protein
OG_05118	LA76x_4027	LA76x_4027; hypothetical protein
OG_05119	LA76x_604	LA76x_604; phenylacetic acid catabolic family protein
OG_05120	LA76x_3124	lieA; leupeptin-inactivating enzyme 1
OG_05121	LA76x_2173	LA76x_2173; hypothetical protein
OG_05122	LA76x_3349	LA76x_3349; hypothetical protein
OG_05123	LA76x_2288	LA76x_2288; conserved hypothetical protein
OG_05124	LA76x_975	LA76x_975; 3-Oxoacyl-[acyl-carrier-(ACP)] synthase III C terminal fa
OG_05125	LA76x_2824	LA76x_2824; phage-related baseplate assembly family protein
OG_05127	LA76x_202	LA76x_202; hypothetical protein
OG_05128	LA76x_593	LA76x_593; hypothetical protein
OG_05129	LA76x_2812	LA76x_2812; conserved hypothetical protein
OG_05130	LA76x_3384	LA76x_3384; hypothetical protein
OG_05131	LA76x_2075	LA76x_2075; conserved hypothetical protein
OG_05132	LA76x_1841	LA76x_1841; avidin family protein
OG_05133	LA76x_3618	LA76x_3618; hypothetical protein
OG_05134	LA76x_2817	LA76x_2817; conserved hypothetical protein
OG_05136	LA76x_664	LA76x_664; glycine rich family protein
OG_05137	LA76x_3445	LA76x_3445; hypothetical protein
OG_05138	LA76x_3343	chnB; cyclohexanone monooxygenase
OG_05140	LA76x_352	LA76x_352; hypothetical protein
OG_05141	LA76x_486	LA76x_486; bacterial extracellular solute-binding family protein
OG_05142	LA76x_603	LA76x_603; condensation domain protein
OG_05143	LA76x_642	LA76x_642; hypothetical protein
OG_05144	LA76x_621	LA76x_621; conserved hypothetical protein
OG_05145	LA76x_3107	LA76x_3107; hypothetical protein
OG_05146	LA76x_3653	LA76x_3653; methyltransferase domain protein

OG_05147	LA29479_301	hypothetical protein
OG_05148	LA29479_435	ISEch10 OrfB
OG_05149	LA29479_783	hypothetical protein
OG_05150	LA29479_1191	hypothetical protein
OG_05151	LA29479_1799	putative uncharacterized protein
OG_05152	LA29479_3739	hypothetical protein
OG_05153	LA29479_3208	hypothetical protein
OG_05154	LA29479_1141	hypothetical protein
OG_05156	LA29479_2603	hypothetical protein
OG_05158	LA29479_2598	hypothetical protein
OG_05159	LA29479_3355	hypothetical protein
OG_05160	LA29479_5106	hypothetical protein
OG_05161	LA29479_2366	glutamine amidotransferase class-I family protein
OG_05162	LA29479_4859	exonuclease SbcC
OG_05163	LA29479_4231	hypothetical protein
OG_05164	LA29479_1665	endoribonuclease L-PSP family protein
OG_05165	LA29479_3514	IPT/TIG domain protein
OG_05167	LA29479_4936	hypothetical protein
OG_05168	LA29479_3229	hypothetical protein
OG_05169	LA29479_911	hypothetical protein
OG_05170	LA29479_27	hypothetical protein
OG_05171	LA29479_1017	hypothetical protein
OG_05172	LA29479_3016	hypothetical protein
OG_05173	LA29479_346	aldehyde dehydrogenase family protein
OG_05174	LA29479_4471	hypothetical protein
OG_05175	LA29479_2862	dienelactone hydrolase family protein
OG_05176	LA29479_4868	nmrA-like family protein
OG_05177	LA29479_4291	hypothetical protein
OG_05178	LA29479_4254	hypothetical protein
OG_05179	LA29479_2113	hypothetical protein
OG_05182	LA29479_1066	peptidase M48 family protein
OG_05183	LA29479_590	tonB-dependent Receptor Plug domain protein
OG_05184	LA29479_2892	putative uncharacterized protein
OG_05185	LA29479_287	putative uncharacterized protein
OG_05186	LA29479_2213	hypothetical protein
OG_05187	LA29479_2526	glutathione S-transferase, N-terminal domain protein
OG_05188	LA29479_920	hypothetical protein
OG_05190	LA29479_59	hypothetical protein
OG_05191	LA29479_82	hypothetical protein
OG_05192	LA29479_1689	putative uncharacterized protein
OG_05193	LA29479_3110	hypothetical protein
OG_05194	LA29479_39	anti-anti-sigma factor family protein
OG_05195	LA29479_1016	outer membrane autotransporter barrel domain protein
OG_05196	LA29479_3808	hypothetical protein
OG_05197	LA29479_2380	bacterial regulatory s, luxR family protein
OG_05198	LA29479_4183	hypothetical protein
OG_05199	LA29479_2216	hypothetical protein
OG_05201	LA29479_4645	hypothetical protein
OG_05202	LA29479_3630	putative membrane protein
OG_05203	LA29479_690	hypothetical protein
OG_05204	LA29479_4224	putative uncharacterized protein
OG_05205	LA29479_324	hypothetical protein
OG_05206	LA29479_4081	hypothetical protein
OG_05207	LA29479_1008	hypothetical protein
OG_05208	LA29479_40	HAMP domain protein
OG_05209	LA29479_4395	hypothetical protein
OG_05210	LA29479_593	hypothetical protein
OG_05211	LA29479_5137	hypothetical protein
OG_05212	LA29479_2700	putative uncharacterized domain protein
OG_05213	LA29479_149	hypothetical protein
OG_05214	LA29479_3321	hypothetical protein
OG_05215	LA29479_1267	hypothetical protein
OG_05216	LA29479_351	putative transmembrane protein
OG_05217	LA29479_3632	hypothetical protein
OG_05218	LA29479_2652	hypothetical protein
OG_05221	LA29479_4439	tetratricopeptide repeat family protein
OG_05222	LA29479_4841	hypothetical protein
OG_05223	LA29479_4342	hypothetical protein
OG_05224	LA29479_4396	hypothetical protein
OG_05225	LA29479_600	putative uncharacterized domain protein
OG_05226	LA29479_425	hypothetical protein
OG_05227	LA29479_354	GAF domain protein
OG_05228	LA29479_2606	putative uncharacterized protein
OG_05230	LA29479_555	hypothetical protein
OG_05231	LA29479_2531	hypothetical protein
OG_05232	LA29479_4816	hypothetical protein
OG_05233	LA29479_1390	hypothetical protein
OG_05234	LA29479_917	hypothetical protein
OG_05235	LA29479_1346	hypothetical protein
OG_05236	LA29479_708	hypothetical protein
OG_05237	LA29479_1023	phospholipase C, PlcB
OG_05238	LA29479_2622	hypothetical protein

OG_05147	LA76x_5142	LA76x_5142; hypothetical protein
OG_05148	LA76x_5018	LA76x_5018; putative transposase
OG_05149	LA76x_3628	LA76x_3628; hypothetical protein
OG_05150	LA76x_2452	LA76x_2452; hypothetical protein
OG_05151	LA76x_2827	LA76x_2827; conserved hypothetical protein
OG_05152	LA76x_4948	LA76x_4948; hypothetical protein
OG_05153	LA76x_1816	LA76x_1816; hypothetical protein
OG_05154	LA76x_2503	LA76x_2503; hypothetical protein
OG_05156	LA76x_530	LA76x_530; hypothetical protein
OG_05158	LA76x_526	LA76x_526; conserved hypothetical protein
OG_05159	LA76x_3880	LA76x_3880; hypothetical protein
OG_05160	LA76x_483	LA76x_483; hypothetical protein
OG_05161	LA76x_3333	LA76x_3333; bacterial extracellular solute-binding, 5 Middle family pr
OG_05162	LA76x_3519	LA76x_3519; putative exonuclease SbcC
OG_05163	LA76x_1771	LA76x_1771; hypothetical protein
OG_05164	LA76x_2170	LA76x_2170; endoribonuclease L-PSP family protein
OG_05165	LA76x_2722	LA76x_2722; IPT/TIG domain protein
OG_05167	LA76x_823	LA76x_823; hypothetical protein
OG_05168	LA76x_1797	LA76x_1797; hypothetical protein
OG_05169	LA76x_3751	LA76x_3751; hypothetical protein
OG_05170	LA76x_3159	LA76x_3159; ser-Thr-rich glycosyl-phosphatidyl-inositol-anchored me
OG_05171	LA76x_2626	LA76x_2626; hypothetical protein
OG_05172	LA76x_2635	LA76x_2635; hypothetical protein
OG_05173	LA76x_5098	LA76x_5098; aldehyde dehydrogenase family protein
OG_05174	LA76x_4688	LA76x_4688; hypothetical protein
OG_05175	LA76x_667	LA76x_667; dienelactone hydrolase family protein
OG_05176	LA76x_3527	LA76x_3527; NAD dependent epimerase/dehydratase family protein
OG_05177	LA76x_1144	LA76x_1144; hypothetical protein
OG_05178	LA76x_1748	LA76x_1748; hypothetical protein
OG_05179	LA76x_1039	LA76x_1039; hypothetical protein
OG_05182	LA76x_2576	LA76x_2576; peptidase M48 family protein
OG_05183	LA76x_1677	LA76x_1677; tonB-dependent Receptor Plug domain protein
OG_05184	LA76x_696	LA76x_696; lipase family protein
OG_05185	LA76x_5157	LA76x_5157; conserved hypothetical protein
OG_05186	LA76x_4279	LA76x_4279; hypothetical protein
OG_05187	LA76x_4481	LA76x_4481; glutathione S-transferase, C-terminal domain protein
OG_05188	LA76x_3760	LA76x_3760; hypothetical protein
OG_05190	LA76x_3128	LA76x_3128; hypothetical protein
OG_05191	LA76x_3106	LA76x_3106; hypothetical protein
OG_05192	LA76x_2144	LA76x_2144; histone H1-like nucleoHC2 family protein
OG_05193	LA76x_922	LA76x_922; hypothetical protein
OG_05194	LA76x_3148	LA76x_3148; anti-anti-sigma factor family protein
OG_05195	LA76x_2627	LA76x_2627; autotransporter-associated beta strand repeat family pr
OG_05196	LA76x_4761	LA76x_4761; hypothetical protein
OG_05197	LA76x_3348	LA76x_3348; sensory box protein
OG_05198	LA76x_4177	LA76x_4177; hypothetical protein
OG_05199	LA76x_4282	LA76x_4282; hypothetical protein
OG_05201	LA76x_3455	LA76x_3455; hypothetical protein
OG_05202	LA76x_3239	LA76x_3239; hypothetical protein
OG_05203	LA76x_1584	LA76x_1584; hypothetical protein
OG_05204	LA76x_1779	LA76x_1779; conserved hypothetical protein
OG_05205	LA76x_5120	LA76x_5120; hypothetical protein
OG_05206	LA76x_250	LA76x_250; hypothetical protein
OG_05207	LA76x_2558	LA76x_2558; hypothetical protein
OG_05208	LA76x_3147	LA76x_3147; HAMP domain protein
OG_05209	LA76x_521	LA76x_521; hypothetical protein
OG_05210	LA76x_1674	LA76x_1674; hypothetical protein
OG_05211	LA76x_2254	LA76x_2254; hypothetical protein
OG_05212	LA76x_627	LA76x_627; hypothetical protein
OG_05213	LA76x_3038	LA76x_3038; hypothetical protein
OG_05214	LA76x_165	LA76x_165; hypothetical protein
OG_05215	LA76x_105	LA76x_105; hypothetical protein
OG_05216	LA76x_5093	LA76x_5093; conserved hypothetical protein
OG_05217	LA76x_3237	LA76x_3237; hypothetical protein
OG_05218	LA76x_577	LA76x_577; hypothetical protein
OG_05221	LA76x_4643	LA76x_4643; TPR repeat family protein
OG_05222	LA76x_3309	LA76x_3309; hypothetical protein
OG_05223	LA76x_1493	LA76x_1493; hypothetical protein
OG_05224	LA76x_520	LA76x_520; hypothetical protein
OG_05225	LA76x_1667	LA76x_1667; conserved hypothetical protein
OG_05226	LA76x_5027	LA76x_5027; hypothetical protein
OG_05227	LA76x_5090	LA76x_5090; GAF domain protein
OG_05228	LA76x_533	LA76x_533; conserved hypothetical family protein
OG_05230	LA76x_1710	LA76x_1710; hypothetical protein
OG_05231	LA76x_4486	LA76x_4486; hypothetical protein
OG_05232	LA76x_3279	LA76x_3279; hypothetical protein
OG_05233	LA76x_5188	LA76x_5188; hypothetical protein
OG_05234	LA76x_3757	LA76x_3757; hypothetical protein
OG_05235	LA76x_25	LA76x_25; hypothetical protein
OG_05236	LA76x_1567	LA76x_1567; hypothetical protein
OG_05237	LA76x_2620	LA76x_2620; putative phospholipase C, PlcB
OG_05238	LA76x_549	LA76x_549; hypothetical protein

OG_05239	LA29479_465	putative lipoprotein
OG_05240	LA29479_4406	hypothetical protein
OG_05241	LA29479_4407	hypothetical protein
OG_05242	LA29479_4405	hypothetical protein
OG_05243	LA29479_4551	tat (twin-arginine translocation) pathway signal sequence domain pro
OG_05244	LA29479_5170	hypothetical protein
OG_05245	LA29479_619	hypothetical protein
OG_05246	LA29479_3458	hypothetical protein
OG_05247	LA29479_1785	glutathione S-transferase, N-terminal domain protein
OG_05248	LA29479_2535	hypothetical protein
OG_05249	LA29479_325	hypothetical protein
OG_05250	LA29479_443	hypothetical protein
OG_05251	LA29479_1816	phage tail sheath family protein
OG_05252	LA29479_1743	hypothetical protein
OG_05253	LA29479_1747	hypothetical protein
OG_05254	LA29479_4371	hypothetical protein
OG_05255	LA29479_2838	hypothetical protein
OG_05256	LA29479_2679	AMP-binding enzyme family protein
OG_05257	LA29479_3292	thioesterase family protein
OG_05258	LA29479_2985	hypothetical protein
OG_05259	LA29479_1692	hypothetical protein
OG_05260	LA29479_390	putative uncharacterized protein XOO1433
OG_05261	LA29479_817	hypothetical protein
OG_05262	LA29479_2604	fels-1 Prophage Protein-like family protein
OG_05263	LA29479_2173	hypothetical protein
OG_05264	LA29479_54	putative uncharacterized protein
OG_05265	LA29479_345	putative uncharacterized protein
OG_05266	LA29479_2384	hypothetical protein
OG_05267	LA29479_407	hypothetical protein
OG_05268	LA29479_2667	RHS Repeat family protein
OG_05269	LA29479_4035	putative lipoprotein
OG_05270	LA29479_1268	N-acetylmuramoyl-L-alanine amidase family protein
OG_05271	LA29479_28	hypothetical protein
OG_05272	LA29479_5165	hypothetical protein
OG_05273	LA29479_79	putative uncharacterized protein
OG_05274	LA29479_1348	hypothetical protein
OG_05275	LA29479_1139	hypothetical protein
OG_05276	LA29479_2543	hypothetical protein
OG_05277	LA29479_4244	conserved hypothetical protein
OG_05278	LA29479_4367	hypothetical protein
OG_05279	LA29479_37	putative transcriptional regualtor
OG_05280	LA29479_2686	hypothetical protein
OG_05281	LA29479_2601	bacterial regulatory helix-turn-helix , lysR family protein
OG_05282	LA29479_918	hypothetical protein
OG_05283	LA29479_334	acetyltransferase, CysE/LacA/LpxA/NodL family
OG_05284	LA29479_4556	hypothetical protein
OG_05285	LA29479_352	methyltransferase family protein
OG_05286	LA29479_4708	hypothetical protein
OG_05287	LA29479_3295	lipase/esterase
OG_05288	LA29479_3230	hypothetical protein
OG_05289	LA29479_3101	putative membrane protein
OG_05290	LA29479_1138	hypothetical protein
OG_05291	LA29479_2396	thioredoxin reductase
OG_05292	LA29479_2351	rebB protein
OG_05293	LA29479_1788	putative membrane protein
OG_05294	LA29479_4411	putative uncharacterized protein
OG_05295	LA29479_2982	putative membrane protein
OG_05296	LA29479_2506	hypothetical protein
OG_05297	LA29479_106	hypothetical protein
OG_05298	LA29479_2199	relaxation protein
OG_05299	LA29479_4864	hypothetical protein
OG_05300	LA29479_3830	TMP repeat family protein
OG_05301	LA29479_2869	hypothetical protein
OG_05302	LA29479_118	darcynin homolog
OG_05303	LA29479_2013	hypothetical protein
OG_05304	LA29479_1777	putative uncharacterized protein
OG_05305	LA29479_1789	hypothetical protein
OG_05306	LA29479_1229	putative uncharacterized protein
OG_05307	LA29479_1880	hypothetical protein
OG_05308	LA29479_2841	nitroreductase family protein
OG_05309	LA29479_809	hypothetical protein
OG_05310	LA29479_2383	hypothetical protein
OG_05311	LA29479_365	hypothetical protein
OG_05312	LA29479_4568	tonB-dependent siderophore receptor family protein
OG_05313	LA29479_4632	putative uncharacterized domain protein
OG_05314	LA29479_3259	acetyltransferase
OG_05315	LA29479_182	hypothetical protein
OG_05318	LA29479_3193	hypothetical protein
OG_05319	LA29479_77	putative uncharacterized protein
OG_05320	LA29479_1812	putative uncharacterized protein
OG_05321	LA29479_75	subtilase family protein
OG_05322	LA29479_1022	hypothetical protein

OG_05239	LA76x_4894	LA76x_4894; hypothetical protein
OG_05240	LA76x_510	LA76x_510; hypothetical protein
OG_05241	LA76x_509	LA76x_509; alpha/beta hydrolase family protein
OG_05242	LA76x_511	LA76x_511; hypothetical protein
OG_05243	LA76x_1882	LA76x_1882; conserved hypothetical protein
OG_05244	LA76x_839	LA76x_839; hypothetical protein
OG_05245	LA76x_1655	LA76x_1655; hypothetical protein
OG_05246	LA76x_4242	LA76x_4242; hypothetical protein
OG_05247	LA76x_2845	LA76x_2845; glutathione S-transferase, N-terminal domain protein
OG_05248	LA76x_4490	LA76x_4490; hypothetical protein
OG_05249	LA76x_5119	LA76x_5119; hypothetical protein
OG_05250	LA76x_4914	LA76x_4914; hypothetical protein
OG_05251	LA76x_2810	LA76x_2810; phage tail sheath family protein
OG_05252	LA76x_2885	LA76x_2885; hypothetical protein
OG_05253	LA76x_2882	LA76x_2882; hypothetical protein
OG_05254	LA76x_1466	LA76x_1466; hypothetical protein
OG_05255	LA76x_645	LA76x_645; hypothetical protein
OG_05256	LA76x_605	LA76x_605; AMP-binding enzyme family protein
OG_05257	LA76x_191	LA76x_191; thioesterase family protein
OG_05258	LA76x_2662	LA76x_2662; hypothetical protein
OG_05259	LA76x_2141	LA76x_2141; hypothetical protein
OG_05260	LA76x_5057	LA76x_5057; conserved hypothetical protein
OG_05261	LA76x_3659	LA76x_3659; YCII-related domain protein
OG_05262	LA76x_531	LA76x_531; fels-1 Prophage Protein-like family protein
OG_05263	LA76x_982	LA76x_982; hypothetical protein
OG_05264	LA76x_3133	LA76x_3133; conserved hypothetical protein
OG_05265	LA76x_5099	LA76x_5099; conserved hypothetical protein
OG_05266	LA76x_3352	LA76x_3352; CHAT domain protein
OG_05267	LA76x_5044	LA76x_5044; hypothetical protein
OG_05268	LA76x_591	LA76x_591; RHS repeat-associated core domain protein
OG_05269	LA76x_292	LA76x_292; hypothetical protein
OG_05270	LA76x_104	LA76x_104; N-acetylmuramoyl-L-alanine amidase family protein
OG_05271	LA76x_3158	LA76x_3158; trypsin family protein
OG_05272	LA76x_835	LA76x_835; hypothetical protein
OG_05273	LA76x_3109	LA76x_3109; conserved hypothetical protein
OG_05274	LA76x_23	LA76x_23; hypothetical protein
OG_05275	LA76x_2505	LA76x_2505; hypothetical protein
OG_05276	LA76x_4498	LA76x_4498; hypothetical protein
OG_05277	LA76x_1758	LA76x_1758; conserved hypothetical protein
OG_05278	LA76x_1470	LA76x_1470; hypothetical protein
OG_05279	LA76x_3150	LA76x_3150; cro/C1-type HTH DNA-binding domain protein
OG_05280	LA76x_613	LA76x_613; hypothetical protein
OG_05281	LA76x_528	LA76x_528; bacterial regulatory helix-turn-helix , lysR family protein
OG_05282	LA76x_3758	LA76x_3758; hypothetical protein
OG_05283	LA76x_5110	LA76x_5110; bacterial transferase hexapeptide family protein
OG_05284	LA76x_1442	LA76x_1442; hypothetical protein
OG_05285	LA76x_5092	LA76x_5092; methyltransferase domain protein
OG_05286	LA76x_3464	LA76x_3464; hypothetical protein
OG_05287	LA76x_188	LA76x_188; prolyl oligopeptidase family protein
OG_05288	LA76x_1796	LA76x_1796; hypothetical protein
OG_05289	LA76x_913	LA76x_913; hypothetical protein
OG_05290	LA76x_2506	LA76x_2506; hypothetical protein
OG_05291	LA76x_3364	LA76x_3364; pyridine nucleotide-disulfide oxidoreductase family prote
OG_05292	LA76x_4415	LA76x_4415; killing trait family protein
OG_05293	LA76x_2839	LA76x_2839; hypothetical protein
OG_05294	LA76x_506	LA76x_506; conserved hypothetical protein
OG_05295	LA76x_2665	LA76x_2665; sulfite exporter TauE/SafE family protein
OG_05296	LA76x_4461	LA76x_4461; hypothetical protein
OG_05297	LA76x_3081	LA76x_3081; hypothetical protein
OG_05298	LA76x_958	LA76x_958; putative relaxation protein
OG_05299	LA76x_3523	LA76x_3523; hypothetical protein
OG_05300	LA76x_4740	LA76x_4740; hypothetical protein
OG_05301	LA76x_674	LA76x_674; hypothetical protein
OG_05302	LA76x_3068	LA76x_3068; conserved hypothetical protein
OG_05303	LA76x_1191	LA76x_1191; hypothetical protein
OG_05304	LA76x_2852	LA76x_2852; conserved hypothetical protein
OG_05305	LA76x_2837	LA76x_2837; hypothetical protein
OG_05306	LA76x_139	LA76x_139; conserved hypothetical protein
OG_05307	LA76x_2751	LA76x_2751; hypothetical protein
OG_05308	LA76x_648	LA76x_648; nitroreductase family protein
OG_05309	LA76x_3652	LA76x_3652; D-mannose binding lectin family protein
OG_05310	LA76x_3351	LA76x_3351; hypothetical protein
OG_05311	LA76x_5079	LA76x_5079; hypothetical protein
OG_05312	LA76x_1432	LA76x_1432; tonB-dependent siderophore receptor family protein
OG_05313	LA76x_3443	LA76x_3443; conserved hypothetical protein
OG_05314	LA76x_221	LA76x_221; hypothetical protein
OG_05315	LA76x_3008	LA76x_3008; hypothetical protein
OG_05318	LA76x_1831	LA76x_1831; hypothetical protein
OG_05319	LA76x_3111	LA76x_3111; alpha/beta hydrolase fold family protein
OG_05320	LA76x_2814	LA76x_2814; conserved hypothetical protein
OG_05321	LA76x_3113	LA76x_3113; subtilase family protein
OG_05322	LA76x_2621	LA76x_2621; PPIC-type PPIASE domain protein

OG_05323	LA29479_3066	putative lipoprotein
OG_05324	LA29479_2631	putative membrane protein
OG_05325	LA29479_2646	putative lipoprotein
OG_05326	LA29479_2375	flavin-containing monooxygenase
OG_05327	LA29479_4366	hypothetical protein
OG_05328	LA29479_228	hypothetical protein
OG_05329	LA29479_2372	hypothetical protein
OG_05330	LA29479_41	tetratricopeptide repeat family protein
OG_05331	LA29479_1623	hypothetical protein
OG_05333	LA29479_2010	hypothetical protein
OG_05334	LA29479_2453	hypothetical protein
OG_05335	LA29479_1796	putative uncharacterized protein
OG_05336	LA29479_2605	superoxide dismutase [Cu-Zn]
OG_05337	LA29479_2829	hypothetical protein
OG_05338	LA29479_1425	hypothetical protein
OG_05339	LA29479_1396	hypothetical protein
OG_05340	LA29479_5152	putative uncharacterized protein
OG_05342	LA29479_2618	putative membrane protein
OG_05343	LA29479_1714	homoserine O-acetyltransferase
OG_05344	LA29479_2432	antibiotic biosynthesis monooxygenase family protein
OG_05345	LA29479_4280	hypothetical protein
OG_05346	LA29479_1000	hypothetical protein
OG_05347	LA29479_558	putative membrane protein
OG_05348	LA29479_2390	hypothetical protein
OG_05349	LA29479_484	conserved hypothetical protein

OG_05323	LA76x_880	LA76x_880; hypothetical protein
OG_05324	LA76x_558	LA76x_558; hypothetical protein
OG_05325	LA76x_571	LA76x_571; hypothetical protein
OG_05326	LA76x_3342	phzS; flavin-containing monooxygenase
OG_05327	LA76x_1471	LA76x_1471; hypothetical protein
OG_05328	LA76x_2962	LA76x_2962; hypothetical protein
OG_05329	LA76x_3339	LA76x_3339; hypothetical protein
OG_05330	LA76x_3146	LA76x_3146; TPR repeat family protein
OG_05331	LA76x_2211	LA76x_2211; hypothetical protein
OG_05333	LA76x_1192	LA76x_1192; hypothetical protein
OG_05334	LA76x_3416	LA76x_3416; hypothetical protein
OG_05335	LA76x_2830	LA76x_2830; conserved hypothetical protein
OG_05336	LA76x_532	SOD1; superoxide dismutase [Cu-Zn]
OG_05337	LA76x_2304	LA76x_2304; hypothetical protein
OG_05338	LA76x_2079	LA76x_2079; hypothetical protein
OG_05339	LA76x_5182	LA76x_5182; hypothetical protein
OG_05340	LA76x_639	LA76x_639; conserved hypothetical protein
OG_05342	LA76x_547	LA76x_547; conserved hypothetical protein
OG_05343	LA76x_2118	metX; homoserine O-acetyltransferase
OG_05344	LA76x_3401	LA76x_3401; antibiotic biosynthesis monooxygenase family protein
OG_05345	LA76x_1155	LA76x_1155; hypothetical protein
OG_05346	LA76x_3842	LA76x_3842; hypothetical protein
OG_05347	LA76x_1707	LA76x_1707; hypothetical protein
OG_05348	LA76x_3359	LA76x_3359; hypothetical protein
OG_05349	LA76x_4874	LA76x_4874; eamA-like transporter family protein

Orthologous group	*L. cap* 55<br><br>GeneID (#490)<br>8.6% of genome	Gene description
OG_03981	LC55x_1127	LC55x_1127; conserved hypothetical protein
OG_03981		
OG_03981		
OG_04434	LC55x_1508	LC55x_1508; major Facilitator Superfamily protein
OG_04434	LC55x_400	LC55x_400; major Facilitator Superfamily protein
OG_04677	LC55x_2614	LC55x_2614; hypothetical protein
OG_04812	LC55x_1056	pcaH; protocatechuate 3,4-dioxygenase, beta subunit
OG_04835	LC55x_2399	LC55x_2399; hypothetical protein
OG_04840	LC55x_2592	LC55x_2592; von Willebrand factor type A domain protein
OG_04922	LC55x_3161	LC55x_3161; putative v8-like Glu-specific endopeptidase
OG_04951	LC55x_1960	LC55x_1960; hypothetical protein
OG_05008	LC55x_4758	LC55x_4758; calpain cysteine protease family protein
OG_05157	LC55x_3175	LC55x_3175; hypothetical protein
OG_05180	LC55x_4417	LC55x_4417; hypothetical protein
OG_05200	LC55x_2688	LC55x_2688; glycosyl transferases group 1 family protein
OG_05424	LC55x_939	LC55x_939; conserved repeat domain protein
OG_05475	LC55x_1214	LC55x_1214; RHS Repeat family protein
OG_05524	LC55x_1316	LC55x_1316; hypothetical protein
OG_05526	LC55x_5434	LC55x_5434; hypothetical protein
OG_05587	LC55x_3858	LC55x_3858; ricin-type beta-trefoil lectin domain protein
OG_05611	LC55x_2988	LC55x_2988; hypothetical protein
OG_05612	LC55x_2672	pld; phospholipase D domain protein
OG_05613	LC55x_2696	cysK; cysteine synthase A
OG_05615	LC55x_5482	LC55x_5482; hypothetical protein
OG_05616	LC55x_2966	LC55x_2966; putative peptidoglycan binding domain protein
OG_05617	LC55x_1122	LC55x_1122; phage integrase family protein
OG_05618	LC55x_3735	LC55x_3735; bacterial regulatory, tetR family protein
OG_05619	LC55x_4690	LC55x_4690; tautomerase enzyme family protein
OG_05620	LC55x_3043	LC55x_3043; diguanylate cyclase domain protein
OG_05621	LC55x_5438	LC55x_5438; aldo/keto reductase family protein
OG_05622	LC55x_1373	LC55x_1373; methyltransferase domain protein
OG_05623	LC55x_988	LC55x_988; hypothetical protein
OG_05624	LC55x_4982	LC55x_4982; hypothetical protein
OG_05625	LC55x_5485	LC55x_5485; beta-lactamase family protein
OG_05626	LC55x_3402	LC55x_3402; putative peptidoglycan-binding protein
OG_05627	LC55x_5573	LC55x_5573; hypothetical protein
OG_05629	LC55x_4117	LC55x_4117; hypothetical protein
OG_05630	LC55x_4550	LC55x_4550; hypothetical protein
OG_05631	LC55x_2206	LC55x_2206; conserved hypothetical protein
OG_05632	LC55x_5513	LC55x_5513; conserved hypothetical protein
OG_05633	LC55x_2690	LC55x_2690; bacterial lipid A biosynthesis acyltransferase family prot
OG_05634	LC55x_1509	LC55x_1509; conserved hypothetical protein
OG_05635	LC55x_3362	LC55x_3362; bacterial regulatory helix-turn-helix, AraC family protein
OG_05636	LC55x_2477	LC55x_2477; conserved hypothetical protein
OG_05637	LC55x_4864	LC55x_4864; hypothetical protein
OG_05638	LC55x_4301	LC55x_4301; gram-negative bacterial tonB family protein
OG_05639	LC55x_1581	LC55x_1581; pilX N-terminal family protein
OG_05640	LC55x_5739	LC55x_5739; DNA methylase family protein
OG_05641	LC55x_3843	LC55x_3843; conserved hypothetical protein
OG_05642	LC55x_2674	LC55x_2674; hypothetical protein
OG_05643	LC55x_4655	LC55x_4655; hypothetical protein
OG_05644	LC55x_3220	LC55x_3220; conserved hypothetical protein
OG_05645	LC55x_1757	LC55x_1757; conserved hypothetical protein
OG_05646	LC55x_5251	LC55x_5251; hypothetical protein
OG_05647	LC55x_1151	LC55x_1151; response regulator
OG_05648	LC55x_92	LC55x_92; hypothetical protein
OG_05649	LC55x_3169	LC55x_3169; hypothetical protein
OG_05650	LC55x_640	LC55x_640; histidine kinase family protein
OG_05651	LC55x_1123	LC55x_1123; phage integrase family protein
OG_05652	LC55x_5583	LC55x_5583; pepSY-associated TM helix family protein
OG_05653	LC55x_1599	LC55x_1599; hypothetical protein
OG_05654	LC55x_2695	LC55x_2695; disulfide oxidoreductase domain protein
OG_05655	LC55x_4963	LC55x_4963; conserved hypothetical protein
OG_05656	LC55x_1467	LC55x_1467; hypothetical protein
OG_05657	LC55x_2354	LC55x_2354; hypothetical protein
OG_05658	LC55x_5443	LC55x_5443; hypothetical protein
OG_05659	LC55x_1381	LC55x_1381; outer membrane beta-barrel domain protein
OG_05660	LC55x_1272	LC55x_1272; ABC transporter family protein
OG_05661	LC55x_2524	LC55x_2524; hypothetical protein
OG_05662	LC55x_3711	LC55x_3711; hypothetical protein
OG_05663	LC55x_2687	LC55x_2687; alpha amylase, catalytic domain protein
OG_05664	LC55x_3076	LC55x_3076; nucleoside-specific channel-forming , Tsx family protein
OG_05665	LC55x_1762	LC55x_1762; hypothetical protein
OG_05666	LC55x_2990	LC55x_2990; hypothetical protein
OG_05667	LC55x_4584	LC55x_4584; FAD binding domain protein
OG_05668	LC55x_4782	LC55x_4782; CAAX protease self-immunity family protein
OG_05669	LC55x_4568	LC55x_4568; hypothetical protein

Orthologous group	*L. cap* AZ78<br><br>Acc. no. (#491)<br>9.6% of genome	Gene description
OG_03981	EYR68498.1	transcriptional regulator [Lysobacter capsici AZ78]
OG_03981	EYR68507.1	XRE family transcriptional regulator [Lysobacter capsici AZ78]
OG_03981	EYR70214.1	transcriptional regulator [Lysobacter capsici AZ78]
OG_04434	EYR70238.1	transcriptional regulator [Lysobacter capsici AZ78]
OG_04434		
OG_04677	EYR68282.1	hypothetical protein AZ78_11490 [Lysobacter capsici AZ78]
OG_04812	EYR67731.1	protocatechuate 3,4-dioxygenase subunit beta [Lysobacter capsici AZ
OG_04835	EYR69830.1	hypothetical protein AZ78_03740 [Lysobacter capsici AZ78]
OG_04840	EYR68303.1	hypothetical protein AZ78_11600 [Lysobacter capsici AZ78]
OG_04922	EYR66938.1	hypothetical protein AZ78_18370 [Lysobacter capsici AZ78]
OG_04951	EYR66004.1	hypothetical protein AZ78_23235 [Lysobacter capsici AZ78]
OG_05008	EYR66089.1	hypothetical protein AZ78_22795 [Lysobacter capsici AZ78]
OG_05157	EYR66927.1	hypothetical protein AZ78_18315 [Lysobacter capsici AZ78]
OG_05180	EYR69244.1	signal peptide protein [Lysobacter capsici AZ78]
OG_05200	EYR68213.1	hypothetical protein AZ78_11145 [Lysobacter capsici AZ78]
OG_05424	EYR66822.1	hypothetical protein AZ78_18760 [Lysobacter capsici AZ78]
OG_05475	EYR66322.1	hypothetical protein AZ78_21465 [Lysobacter capsici AZ78]
OG_05524	EYR70089.1	hypothetical protein AZ78_01800 [Lysobacter capsici AZ78]
OG_05526	EYR68918.1	hypothetical protein AZ78_07760 [Lysobacter capsici AZ78]
OG_05587	EYR65550.1	hypothetical protein AZ78_25720 [Lysobacter capsici AZ78]
OG_05611	EYR67868.1	hypothetical protein AZ78_13405 [Lysobacter capsici AZ78]
OG_05612	EYR68227.1	hypothetical protein AZ78_11215 [Lysobacter capsici AZ78]
OG_05613	EYR68204.1	cysteine synthase [Lysobacter capsici AZ78]
OG_05615	EYR69046.1	hypothetical protein AZ78_07980 [Lysobacter capsici AZ78]
OG_05616	EYR66196.1	peptidoglycan-binding protein [Lysobacter capsici AZ78]
OG_05617	EYR68501.1	integrase [Lysobacter capsici AZ78]
OG_05618	EYR67047.1	TetR family transcriptional regulator [Lysobacter capsici AZ78]
OG_05619	EYR70188.1	tautomerase [Lysobacter capsici AZ78]
OG_05620	EYR67830.1	membrane protein [Lysobacter capsici AZ78]
OG_05621	EYR68920.1	aldehyde oxidase [Lysobacter capsici AZ78]
OG_05622	EYR67191.1	cyclopropane-fatty-acyl-phospholipid synthase [Lysobacter capsici AZ
OG_05623	EYR67790.1	hypothetical protein AZ78_14310 [Lysobacter capsici AZ78]
OG_05624	EYR67246.1	hypothetical protein AZ78_16880 [Lysobacter capsici AZ78]
OG_05625	EYR69048.1	hypothetical protein AZ78_07995 [Lysobacter capsici AZ78]
OG_05626	EYR66563.1	hypothetical protein AZ78_20420 [Lysobacter capsici AZ78]
OG_05627	EYR68668.1	hypothetical protein AZ78_08560 [Lysobacter capsici AZ78]
OG_05629	EYR66363.1	hypothetical protein AZ78_21670 [Lysobacter capsici AZ78]
OG_05630	EYR68572.1	hypothetical protein AZ78_09880 [Lysobacter capsici AZ78]
OG_05631	EYR67410.1	hypothetical protein AZ78_15855 [Lysobacter capsici AZ78]
OG_05632	EYR68953.1	hypothetical protein AZ78_08130 [Lysobacter capsici AZ78]
OG_05633	EYR68211.1	hypothetical protein AZ78_11125 [Lysobacter capsici AZ78]
OG_05634	EYR70239.1	membrane protein [Lysobacter capsici AZ78]
OG_05635	EYR66535.1	AraC family transcriptional regulator [Lysobacter capsici AZ78]
OG_05636	EYR69535.1	hypothetical protein AZ78_03350 [Lysobacter capsici AZ78]
OG_05637	EYR68822.1	hypothetical protein AZ78_09040 [Lysobacter capsici AZ78]
OG_05638	EYR69261.1	hypothetical protein AZ78_06640 [Lysobacter capsici AZ78]
OG_05639	EYR70297.1	PilX protein [Lysobacter capsici AZ78]
OG_05640	EYR69701.1	DNA methyltransferase [Lysobacter capsici AZ78]
OG_05641	EYR65736.1	hypothetical protein AZ78_24655 [Lysobacter capsici AZ78]
OG_05642	EYR68226.1	hypothetical protein AZ78_11210 [Lysobacter capsici AZ78]
OG_05643	EYR70397.1	hypothetical protein AZ78_00095 [Lysobacter capsici AZ78]
OG_05644	EYR67969.1	hypothetical protein AZ78_13255 [Lysobacter capsici AZ78]
OG_05645	EYR65693.1	hypothetical protein AZ78_24890 [Lysobacter capsici AZ78]
OG_05646	EYR69001.1	hypothetical protein AZ78_07565 [Lysobacter capsici AZ78]
OG_05647	EYR68522.1	transcriptional regulator [Lysobacter capsici AZ78]
OG_05648	EYR69756.1	hypothetical protein AZ78_04865 [Lysobacter capsici AZ78]
OG_05649	EYR66932.1	hypothetical protein AZ78_18340 [Lysobacter capsici AZ78]
OG_05650	EYR70119.1	hypothetical protein AZ78_02280 [Lysobacter capsici AZ78]
OG_05651	EYR68504.1	integrase [Lysobacter capsici AZ78]
OG_05652	EYR68672.1	hypothetical protein AZ78_08610 [Lysobacter capsici AZ78]
OG_05653	EYR70439.1	hypothetical protein AZ78_01060 [Lysobacter capsici AZ78]
OG_05654	EYR68205.1	hypothetical protein AZ78_11085 [Lysobacter capsici AZ78]
OG_05655	EYR67263.1	hypothetical protein AZ78_16965 [Lysobacter capsici AZ78]
OG_05656	EYR70415.1	hypothetical protein AZ78_00395 [Lysobacter capsici AZ78]
OG_05657	EYR69840.1	hypothetical protein AZ78_03945 [Lysobacter capsici AZ78]
OG_05658	EYR69026.1	hypothetical protein AZ78_07800 [Lysobacter capsici AZ78]
OG_05659	EYR67183.1	membrane protein [Lysobacter capsici AZ78]
OG_05660	EYR67675.1	ABC transporter ATP-binding protein [Lysobacter capsici AZ78]
OG_05661	EYR69806.1	hypothetical protein AZ78_03120 [Lysobacter capsici AZ78]
OG_05662	EYR65911.1	hypothetical protein AZ78_23670 [Lysobacter capsici AZ78]
OG_05663	EYR68214.1	hypothetical protein AZ78_11150 [Lysobacter capsici AZ78]
OG_05664	EYR67853.1	nucleoside-binding outer membrane protein [Lysobacter capsici AZ78
OG_05665	EYR65688.1	hypothetical protein AZ78_24865 [Lysobacter capsici AZ78]
OG_05666	EYR67870.1	hypothetical protein AZ78_13415 [Lysobacter capsici AZ78]
OG_05667	EYR68364.1	FAD-dependent pyridine nucleotide-disulfide oxidoreductase [Lysoba
OG_05668	EYR68787.1	CAAX amino terminal protease [Lysobacter capsici AZ78]
OG_05669	EYR68558.1	hypothetical protein AZ78_09775 [Lysobacter capsici AZ78]

OG_05670	LC55x_2358	LC55x_2358; hypothetical protein
OG_05671	LC55x_1504	LC55x_1504; bacterial regulatory helix-turn-helix , lysR family protein
OG_05672	LC55x_1131	LC55x_1131; hypothetical protein
OG_05673	LC55x_3053	LC55x_3053; hypothetical protein
OG_05674	LC55x_2793	LC55x_2793; hypothetical protein
OG_05675	LC55x_2844	LC55x_2844; conserved hypothetical protein
OG_05676	LC55x_2302	LC55x_2302; hypothetical protein
OG_05677	LC55x_1478	LC55x_1478; surface presentation of antigens family protein
OG_05678	LC55x_278	LC55x_278; ankyrin repeat family protein
OG_05679	LC55x_3379	LC55x_3379; hypothetical protein
OG_05680	LC55x_198	LC55x_198; RDD family protein
OG_05683	LC55x_88	LC55x_88; hypothetical protein
OG_05684	LC55x_2817	LC55x_2817; conserved hypothetical protein
OG_05685	LC55x_5406	LC55x_5406; hypothetical protein
OG_05686	LC55x_2476	LC55x_2476; conserved hypothetical protein
OG_05687	LC55x_3118	LC55x_3118; hypothetical protein
OG_05688	LC55x_2849	LC55x_2849; hypothetical protein
OG_05689	LC55x_1209	LC55x_1209; SWIM zinc finger family protein
OG_05690	LC55x_5627	LC55x_5627; hypothetical protein
OG_05691	LC55x_4855	LC55x_4855; hypothetical protein
OG_05692	LC55x_1254	LC55x_1254; conserved hypothetical protein
OG_05693	LC55x_1057	pcaG; protocatechuate 3,4-dioxygenase, alpha subunit
OG_05694	LC55x_5673	LC55x_5673; hypothetical protein
OG_05695	LC55x_255	LC55x_255; hypothetical protein
OG_05696	LC55x_2903	LC55x_2903; pfkB carbohydrate kinase family protein
OG_05697	LC55x_3045	LC55x_3045; hypothetical protein
OG_05698	LC55x_1456	LC55x_1456; hypothetical protein
OG_05699	LC55x_5484	LC55x_5484; bacterial regulatory , arsR family protein
OG_05700	LC55x_5586	hutC; histidine utilization repressor
OG_05701	LC55x_3415	LC55x_3415; hypothetical protein
OG_05702	LC55x_1130	LC55x_1130; mobA/MobL family protein
OG_05703	LC55x_808	LC55x_808; papain cysteine protease family protein
OG_05704	LC55x_1155	LC55x_1155; hypothetical protein
OG_05705	LC55x_4964	LC55x_4964; conserved hypothetical protein
OG_05707	LC55x_3259	LC55x_3259; hypothetical protein
OG_05708	LC55x_3460	LC55x_3460; hypothetical protein
OG_05709	LC55x_4554	LC55x_4554; hypothetical protein
OG_05710	LC55x_3779	LC55x_3779; hypothetical protein
OG_05711	LC55x_5454	LC55x_5454; hypothetical protein
OG_05712	LC55x_2986	LC55x_2986; hypothetical protein
OG_05714	LC55x_4938	LC55x_4938; hypothetical protein
OG_05715	LC55x_5261	LC55x_5261; hypothetical protein
OG_05716	LC55x_1810	LC55x_1810; hypothetical protein
OG_05717	LC55x_4645	LC55x_4645; hypothetical protein
OG_05718	LC55x_638	LC55x_638; hypothetical protein
OG_05719	LC55x_1059	pcaC; 4-carboxymuconolactone decarboxylase
OG_05720	LC55x_4156	LC55x_4156; glycosyl transferase 2 family protein
OG_05721	LC55x_4653	LC55x_4653; glyoxalase/Bleomycin resistance /Dioxygenase superfa
OG_05722	LC55x_2727	LC55x_2727; conserved hypothetical protein
OG_05723	LC55x_2285	LC55x_2285; hypothetical protein
OG_05724	LC55x_474	LC55x_474; YHS domain protein
OG_05725	LC55x_3336	LC55x_3336; hypothetical protein
OG_05726	LC55x_4658	LC55x_4658; hypothetical protein
OG_05727	LC55x_5464	LC55x_5464; hypothetical protein
OG_05728	LC55x_627	LC55x_627; putative xylan 1,4-beta-xylosidase
OG_05729	LC55x_1150	LC55x_1150; histidine kinase-, DNA gyrase B-, and HSP90-like ATPa
OG_05730	LC55x_1612	LC55x_1612; hypothetical protein
OG_05731	LC55x_4994	LC55x_4994; IPT/TIG domain protein
OG_05732	LC55x_3588	LC55x_3588; short chain dehydrogenase family protein
OG_05733	LC55x_2901	LC55x_2901; xylose isomerase-like TIM barrel family protein
OG_05734	LC55x_1472	LC55x_1472; flagellar assembly FliH family protein
OG_05735	LC55x_1250	LC55x_1250; hypothetical protein
OG_05736	LC55x_4878	LC55x_4878; transglutaminase-like superfamily protein
OG_05737	LC55x_4657	LC55x_4657; hypothetical protein
OG_05738	LC55x_5512	LC55x_5512; hypothetical protein
OG_05739	LC55x_1785	LC55x_1785; hypothetical protein
OG_05740	LC55x_1308	LC55x_1308; hypothetical protein
OG_05741	LC55x_2481	LC55x_2481; hypothetical protein
OG_05742	LC55x_5570	LC55x_5570; putative copL
OG_05743	LC55x_5674	LC55x_5674; hypothetical protein
OG_05744	LC55x_679	LC55x_679; putative integron gene cassette protein
OG_05745	LC55x_617	LC55x_617; hypothetical protein
OG_05746	LC55x_5440	LC55x_5440; pentapeptide repeats family protein
OG_05747	LC55x_1464	flgA; flagella basal body P-ring formation protein FlgA
OG_05748	LC55x_3168	LC55x_3168; nuclease-related domain protein
OG_05749	LC55x_4041	LC55x_4041; hypothetical protein
OG_05750	LC55x_4686	LC55x_4686; tetratricopeptide repeat family protein
OG_05751	LC55x_3636	LC55x_3636; hypothetical protein
OG_05752	LC55x_3697	LC55x_3697; hypothetical protein
OG_05753	LC55x_1133	LC55x_1133; conserved hypothetical protein
OG_05754	LC55x_91	LC55x_91; conserved hypothetical protein
OG_05755	LC55x_2675	LC55x_2675; hypothetical protein

OG_05670	EYR69839.1	hypothetical protein AZ78_03930 [Lysobacter capsici AZ78]
OG_05671	EYR70234.1	LysR family transcriptional regulator [Lysobacter capsici AZ78]
OG_05672	EYR68631.1	hypothetical protein AZ78_10850 [Lysobacter capsici AZ78]
OG_05673	EYR67876.1	hypothetical protein AZ78_13695 [Lysobacter capsici AZ78]
OG_05674	EYR69357.1	hypothetical protein AZ78_05545 [Lysobacter capsici AZ78]
OG_05675	EYR69405.1	hypothetical protein AZ78_05790 [Lysobacter capsici AZ78]
OG_05676	EYR69846.1	hypothetical protein AZ78_04190 [Lysobacter capsici AZ78]
OG_05677	EYR70420.1	hypothetical protein AZ78_00450 [Lysobacter capsici AZ78]
OG_05678	EYR67004.1	ATP dependent DNA ligase [Lysobacter capsici AZ78]
OG_05679	EYR66568.1	hypothetical protein AZ78_20520 [Lysobacter capsici AZ78]
OG_05680	EYR66138.1	hypothetical protein AZ78_22480 [Lysobacter capsici AZ78]
OG_05683	EYR69877.1	hypothetical protein AZ78_04845 [Lysobacter capsici AZ78]
OG_05684	EYR69379.1	hypothetical protein AZ78_05655 [Lysobacter capsici AZ78]
OG_05685	EYR69009.1	hypothetical protein AZ78_07625 [Lysobacter capsici AZ78]
OG_05686	EYR69536.1	hypothetical protein AZ78_03355 [Lysobacter capsici AZ78]
OG_05687	EYR66976.1	hypothetical protein AZ78_18570 [Lysobacter capsici AZ78]
OG_05688	EYR69410.1	hypothetical protein AZ78_05815 [Lysobacter capsici AZ78]
OG_05689	EYR65776.1	hypothetical protein AZ78_24460 [Lysobacter capsici AZ78]
OG_05690	EYR66267.1	hypothetical protein AZ78_22110 [Lysobacter capsici AZ78]
OG_05691	EYR68825.1	hypothetical protein AZ78_09075 [Lysobacter capsici AZ78]
OG_05692	EYR65644.1	hypothetical protein AZ78_25190 [Lysobacter capsici AZ78]
OG_05693	EYR67730.1	protocatechuate 3,4-dioxygenase subunit alpha [Lysobacter capsici A
OG_05694	EYR65880.1	hypothetical protein AZ78_24090 [Lysobacter capsici AZ78]
OG_05695	EYR66986.1	hypothetical protein AZ78_17890 [Lysobacter capsici AZ78]
OG_05696	EYR69459.1	5-dehydro-2-deoxygluconokinase [Lysobacter capsici AZ78]
OG_05697	EYR67875.1	hypothetical protein AZ78_13655 [Lysobacter capsici AZ78]
OG_05698	EYR70194.1	hypothetical protein AZ78_00340 [Lysobacter capsici AZ78]
OG_05699	EYR69047.1	hypothetical protein AZ78_07990 [Lysobacter capsici AZ78]
OG_05700	EYR65418.1	hypothetical protein AZ78_26375 [Lysobacter capsici AZ78]
OG_05701	EYR66559.1	hypothetical protein AZ78_20370 [Lysobacter capsici AZ78]
OG_05702	EYR68509.1	MobA/MobL family protein [Lysobacter capsici AZ78]
OG_05703	EYR67692.1	hypothetical protein AZ78_14520 [Lysobacter capsici AZ78]
OG_05704	EYR68638.1	hypothetical protein AZ78_10970 [Lysobacter capsici AZ78]
OG_05705	EYR67262.1	hypothetical protein AZ78_16960 [Lysobacter capsici AZ78]
OG_05707	EYR67924.1	hypothetical protein AZ78_13025 [Lysobacter capsici AZ78]
OG_05708	EYR66645.1	hypothetical protein AZ78_19980 [Lysobacter capsici AZ78]
OG_05709	EYR68568.1	hypothetical protein AZ78_09860 [Lysobacter capsici AZ78]
OG_05710	EYR67089.1	hypothetical protein AZ78_17665 [Lysobacter capsici AZ78]
OG_05711	EYR69029.1	hypothetical protein AZ78_07850 [Lysobacter capsici AZ78]
OG_05712	EYR67867.1	hypothetical protein AZ78_13395 [Lysobacter capsici AZ78]
OG_05714	EYR68808.1	hypothetical protein AZ78_08665 [Lysobacter capsici AZ78]
OG_05715	EYR68906.1	hypothetical protein AZ78_07635 [Lysobacter capsici AZ78]
OG_05716	EYR67319.1	hypothetical protein AZ78_16295 [Lysobacter capsici AZ78]
OG_05717	EYR70157.1	hypothetical protein AZ78_00045 [Lysobacter capsici AZ78]
OG_05718	EYR70120.1	hypothetical protein AZ78_02290 [Lysobacter capsici AZ78]
OG_05719	EYR67728.1	3-oxoadipate enol-lactonase [Lysobacter capsici AZ78]
OG_05720	EYR66325.1	hypothetical protein AZ78_21480 [Lysobacter capsici AZ78]
OG_05721	EYR70161.1	glyoxalase [Lysobacter capsici AZ78]
OG_05722	EYR69300.1	hypothetical protein AZ78_05250 [Lysobacter capsici AZ78]
OG_05723	EYR69850.1	hypothetical protein AZ78_04275 [Lysobacter capsici AZ78]
OG_05724	EYR66487.1	hypothetical protein AZ78_21005 [Lysobacter capsici AZ78]
OG_05725	EYR66586.1	hypothetical protein AZ78_20730 [Lysobacter capsici AZ78]
OG_05726	EYR70401.1	hypothetical protein AZ78_00115 [Lysobacter capsici AZ78]
OG_05727	EYR68932.1	hypothetical protein AZ78_07895 [Lysobacter capsici AZ78]
OG_05728	EYR69989.1	lectin [Lysobacter capsici AZ78]
OG_05729	EYR68521.1	membrane protein [Lysobacter capsici AZ78]
OG_05730	EYR70441.1	hypothetical protein AZ78_01115 [Lysobacter capsici AZ78]
OG_05731	EYR67235.1	hypothetical protein AZ78_16825 [Lysobacter capsici AZ78]
OG_05732	EYR67534.1	oxidoreductase [Lysobacter capsici AZ78]
OG_05733	EYR69457.1	xylose isomerase [Lysobacter capsici AZ78]
OG_05734	EYR70417.1	hypothetical protein AZ78_00420 [Lysobacter capsici AZ78]
OG_05735	EYR65655.1	hypothetical protein AZ78_25175 [Lysobacter capsici AZ78]
OG_05736	EYR68817.1	hypothetical protein AZ78_08980 [Lysobacter capsici AZ78]
OG_05737	EYR70400.1	hypothetical protein AZ78_00110 [Lysobacter capsici AZ78]
OG_05738	EYR69060.1	hypothetical protein AZ78_08125 [Lysobacter capsici AZ78]
OG_05739	EYR67312.1	hypothetical protein AZ78_16195 [Lysobacter capsici AZ78]
OG_05740	EYR70092.1	hypothetical protein AZ78_01845 [Lysobacter capsici AZ78]
OG_05741	EYR69813.1	hypothetical protein AZ78_03330 [Lysobacter capsici AZ78]
OG_05742	EYR68667.1	transcriptional regulator [Lysobacter capsici AZ78]
OG_05743	EYR65879.1	hypothetical protein AZ78_24085 [Lysobacter capsici AZ78]
OG_05744	EYR70108.1	hypothetical protein AZ78_02100 [Lysobacter capsici AZ78]
OG_05745	EYR70127.1	hypothetical protein AZ78_02390 [Lysobacter capsici AZ78]
OG_05746	EYR69023.1	hypothetical protein AZ78_07780 [Lysobacter capsici AZ78]
OG_05747	EYR70414.1	hypothetical protein AZ78_00380 [Lysobacter capsici AZ78]
OG_05748	EYR66933.1	hypothetical protein AZ78_18345 [Lysobacter capsici AZ78]
OG_05749	EYR65820.1	hypothetical protein AZ78_24220 [Lysobacter capsici AZ78]
OG_05750	EYR70184.1	hypothetical protein AZ78_00275 [Lysobacter capsici AZ78]
OG_05751	EYR68797.1	hypothetical protein AZ78_08485 [Lysobacter capsici AZ78]
OG_05752	EYR65922.1	hypothetical protein AZ78_23725 [Lysobacter capsici AZ78]
OG_05753	EYR68485.1	hypothetical protein AZ78_10610 [Lysobacter capsici AZ78]
OG_05754	EYR69879.1	hypothetical protein AZ78_04860 [Lysobacter capsici AZ78]
OG_05755	EYR68224.1	hypothetical protein AZ78_11200 [Lysobacter capsici AZ78]

OG_05756	LC55x_570	LC55x_570; hypothetical protein
OG_05757	LC55x_143	LC55x_143; macro domain protein
OG_05758	LC55x_2700	LC55x_2700; alpha/beta hydrolase family protein
OG_05759	LC55x_2381	LC55x_2381; hypothetical protein
OG_05760	LC55x_2290	LC55x_2290; tonB family C-terminal domain protein
OG_05761	LC55x_4837	LC55x_4837; merR HTH regulatory family protein
OG_05762	LC55x_1580	LC55x_1580; conserved hypothetical protein
OG_05763	LC55x_1052	LC55x_1052; pcaR
OG_05764	LC55x_3154	LC55x_3154; hypothetical protein
OG_05765	LC55x_5524	LC55x_5524; response regulator
OG_05766	LC55x_1053	catI; 3-oxoadipate CoA-transferase subunit A
OG_05767	LC55x_2685	LC55x_2685; cupin superfamily protein
OG_05768	LC55x_2221	LC55x_2221; hemerythrin HHE cation binding domain protein
OG_05769	LC55x_677	LC55x_677; pirin family protein
OG_05770	LC55x_1205	LC55x_1205; putative molybdate metabolism regulator
OG_05771	LC55x_1514	LC55x_1514; doxX-like family protein
OG_05772	LC55x_4597	LC55x_4597; sensory box protein
OG_05773	LC55x_812	LC55x_812; hypothetical protein
OG_05774	LC55x_4833	LC55x_4833; hypothetical protein
OG_05775	LC55x_902	LC55x_902; hypothetical protein
OG_05776	LC55x_2459	LC55x_2459; hypothetical protein
OG_05777	LC55x_2967	LC55x_2967; conserved hypothetical protein
OG_05778	LC55x_1379	LC55x_1379; fatty acid desaturase family protein
OG_05779	LC55x_3733	LC55x_3733; efflux transporter, RND family, MFP subunit
OG_05780	LC55x_1718	LC55x_1718; hypothetical protein
OG_05781	LC55x_2268	LC55x_2268; hypothetical protein
OG_05782	LC55x_5728	LC55x_5728; SMI1 / KNR4 family protein
OG_05783	LC55x_4019	LC55x_4019; putative dehydrogenase
OG_05784	LC55x_5548	LC55x_5548; hypothetical protein
OG_05785	LC55x_5115	LC55x_5115; hypothetical protein
OG_05786	LC55x_809	LC55x_809; hypothetical protein
OG_05787	LC55x_1376	LC55x_1376; mycolic acid cyclopropane synthetase family protein
OG_05788	LC55x_3095	LC55x_3095; hypothetical protein
OG_05789	LC55x_4047	LC55x_4047; hypothetical protein
OG_05790	LC55x_2907	LC55x_2907; helix-turn-helix domain, rpiR family protein
OG_05791	LC55x_4670	LC55x_4670; M61 glycyl aminopeptidase family protein
OG_05792	LC55x_4962	LC55x_4962; thiazole biosynthesis ThiG family protein
OG_05793	LC55x_849	LC55x_849; putative integron gene cassette protein
OG_05794	LC55x_1455	LC55x_1455; tetratricopeptide repeat family protein
OG_05795	LC55x_3515	LC55x_3515; conserved hypothetical protein
OG_05796	LC55x_5449	LC55x_5449; outer membrane beta-barrel domain protein
OG_05797	LC55x_1149	LC55x_1149; esterase family protein
OG_05798	LC55x_4204	LC55x_4204; hypothetical protein
OG_05799	LC55x_2993	LC55x_2993; hypothetical protein
OG_05800	LC55x_4945	LC55x_4945; putative endoproteinase Arg-C
OG_05801	LC55x_3172	LC55x_3172; hypothetical protein
OG_05802	LC55x_4954	LC55x_4954; hypothetical protein
OG_05803	LC55x_2174	LC55x_2174; hypothetical protein
OG_05804	LC55x_1274	LC55x_1274; prolyl oligopeptidase family protein
OG_05805	LC55x_3747	LC55x_3747; hypothetical protein
OG_05806	LC55x_5520	LC55x_5520; putative bifunctional deaminase-reductase-like protein
OG_05807	LC55x_3560	LC55x_3560; conserved hypothetical protein
OG_05808	LC55x_1446	LC55x_1446; hypothetical protein
OG_05809	LC55x_3506	LC55x_3506; hypothetical protein
OG_05810	LC55x_4174	LC55x_4174; hypothetical protein
OG_05811	LC55x_3359	LC55x_3359; conserved hypothetical protein
OG_05812	LC55x_4179	LC55x_4179; hypothetical protein
OG_05813	LC55x_3160	LC55x_3160; hypothetical protein
OG_05814	LC55x_1578	LC55x_1578; type II transport GspH family protein
OG_05815	LC55x_579	LC55x_579; conserved hypothetical protein
OG_05816	LC55x_52	LC55x_52; hypothetical protein
OG_05817	LC55x_2902	LC55x_2902; thiamine pyrophosphate enzyme, C-terminal TPP bindi
OG_05818	LC55x_1377	LC55x_1377; conserved hypothetical protein
OG_05819	LC55x_2123	LC55x_2123; conserved hypothetical protein
OG_05820	LC55x_4579	LC55x_4579; response regulator
OG_05821	LC55x_5530	LC55x_5530; beta-lactamase family protein
OG_05822	LC55x_5579	LC55x_5579; hypothetical protein
OG_05823	LC55x_5422	LC55x_5422; gram-negative bacterial tonB family protein
OG_05824	LC55x_1872	LC55x_1872; metallo-beta-lactamase superfamily protein
OG_05825	LC55x_1054	catJ; 3-oxoadipate CoA-transferase subunit B
OG_05826	LC55x_4158	LC55x_4158; hypothetical protein
OG_05827	LC55x_494	LC55x_494; hypothetical protein
OG_05828	LC55x_1075	LC55x_1075; ABC transporter family protein
OG_05829	LC55x_3173	LC55x_3173; SMI1 / KNR4 family protein
OG_05830	LC55x_1675	LC55x_1675; hypothetical protein
OG_05831	LC55x_4054	LC55x_4054; ubiE/COQ5 methyltransferase family protein
OG_05832	LC55x_4327	LC55x_4327; hypothetical protein
OG_05833	LC55x_2904	LC55x_2904; ABC transporter family protein
OG_05834	LC55x_2898	LC55x_2898; carbohydrate-selective porin, OprB family protein
OG_05835	LC55x_3174	LC55x_3174; tonB-dependent siderophore receptor family protein
OG_05836	LC55x_1682	LC55x_1682; hypothetical protein
OG_05837	LC55x_5529	LC55x_5529; response regulator

OG_05756	EYR70135.1	hypothetical protein AZ78_02605 [Lysobacter capsici AZ78]
OG_05757	EYR69789.1	Appr-1-p processing protein [Lysobacter capsici AZ78]
OG_05758	EYR69285.1	hypothetical protein AZ78_05165 [Lysobacter capsici AZ78]
OG_05759	EYR69833.1	hypothetical protein AZ78_03815 [Lysobacter capsici AZ78]
OG_05760	EYR69667.1	cell envelope biogenesis protein TonB [Lysobacter capsici AZ78]
OG_05761	EYR68757.1	hypothetical protein AZ78_09165 [Lysobacter capsici AZ78]
OG_05762	EYR70437.1	hypothetical protein AZ78_00985 [Lysobacter capsici AZ78]
OG_05763	EYR67735.1	IclR family transcriptional regulator [Lysobacter capsici AZ78]
OG_05764	EYR66944.1	hypothetical protein AZ78_18400 [Lysobacter capsici AZ78]
OG_05765	EYR68959.1	response regulator [Lysobacter capsici AZ78]
OG_05766	EYR67734.1	3-oxoadipate:succinyl-CoA transferase subunit A [Lysobacter capsici
OG_05767	EYR68216.1	hypothetical protein AZ78_11160 [Lysobacter capsici AZ78]
OG_05768	EYR67417.1	hemerythrin [Lysobacter capsici AZ78]
OG_05769	EYR69958.1	quercetin 2,3-dioxygenase [Lysobacter capsici AZ78]
OG_05770	EYR65780.1	WGR domain-containing protein [Lysobacter capsici AZ78]
OG_05771	EYR70243.1	membrane protein [Lysobacter capsici AZ78]
OG_05772	EYR68548.1	hypothetical protein AZ78_09635 [Lysobacter capsici AZ78]
OG_05773	EYR67690.1	hypothetical protein AZ78_14500 [Lysobacter capsici AZ78]
OG_05774	EYR68829.1	hypothetical protein AZ78_09190 [Lysobacter capsici AZ78]
OG_05775	EYR66856.1	hypothetical protein AZ78_18930 [Lysobacter capsici AZ78]
OG_05776	EYR69817.1	hypothetical protein AZ78_03435 [Lysobacter capsici AZ78]
OG_05777	EYR66195.1	hypothetical protein AZ78_21740 [Lysobacter capsici AZ78]
OG_05778	EYR67185.1	delta 9 acyl-lipid fatty acid desaturase [Lysobacter capsici AZ78]
OG_05779	EYR67045.1	transporter [Lysobacter capsici AZ78]
OG_05780	EYR70459.1	hypothetical protein AZ78_01515 [Lysobacter capsici AZ78]
OG_05781	EYR69853.1	hypothetical protein AZ78_04360 [Lysobacter capsici AZ78]
OG_05782	EYR69857.1	hypothetical protein AZ78_04430 [Lysobacter capsici AZ78]
OG_05783	EYR65570.1	hypothetical protein AZ78_25645 [Lysobacter capsici AZ78]
OG_05784	EYR69069.1	hypothetical protein AZ78_08300 [Lysobacter capsici AZ78]
OG_05785	EYR68086.1	hypothetical protein AZ78_12600 [Lysobacter capsici AZ78]
OG_05786	EYR67691.1	hypothetical protein AZ78_14515 [Lysobacter capsici AZ78]
OG_05787	EYR67188.1	cyclopropane-fatty-acyl-phospholipid synthase [Lysobacter capsici A
OG_05788	EYR65444.1	hypothetical protein AZ78_26220 [Lysobacter capsici AZ78]
OG_05789	EYR65826.1	hypothetical protein AZ78_24250 [Lysobacter capsici AZ78]
OG_05790	EYR69463.1	RpiR family transcriptional regulator [Lysobacter capsici AZ78]
OG_05791	EYR70409.1	hypothetical protein AZ78_00195 [Lysobacter capsici AZ78]
OG_05792	EYR67264.1	N-acetylmannosamine-6-phosphate 2-epimerase [Lysobacter capsici
OG_05793	EYR67566.1	hypothetical protein AZ78_14330 [Lysobacter capsici AZ78]
OG_05794	EYR70193.1	hypothetical protein AZ78_00335 [Lysobacter capsici AZ78]
OG_05795	EYR66639.1	hypothetical protein AZ78_20230 [Lysobacter capsici AZ78]
OG_05796	EYR68927.1	membrane protein [Lysobacter capsici AZ78]
OG_05797	EYR68520.1	alpha/beta hydrolase [Lysobacter capsici AZ78]
OG_05798	EYR68186.1	hypothetical protein AZ78_12055 [Lysobacter capsici AZ78]
OG_05799	EYR67871.1	hypothetical protein AZ78_13425 [Lysobacter capsici AZ78]
OG_05800	EYR68676.1	endoproteinase ArgC [Lysobacter capsici AZ78]
OG_05801	EYR66930.1	hypothetical protein AZ78_18330 [Lysobacter capsici AZ78]
OG_05802	EYR67267.1	hypothetical protein AZ78_16985 [Lysobacter capsici AZ78]
OG_05803	EYR67392.1	hypothetical protein AZ78_15715 [Lysobacter capsici AZ78]
OG_05804	EYR67707.1	hypothetical protein AZ78_15050 [Lysobacter capsici AZ78]
OG_05805	EYR67059.1	hypothetical protein AZ78_17510 [Lysobacter capsici AZ78]
OG_05806	EYR68956.1	dihydrofolate reductase [Lysobacter capsici AZ78]
OG_05807	EYR67510.1	hypothetical protein AZ78_15300 [Lysobacter capsici AZ78]
OG_05808	EYR67130.1	hypothetical protein AZ78_17030 [Lysobacter capsici AZ78]
OG_05809	EYR66655.1	hypothetical protein AZ78_20190 [Lysobacter capsici AZ78]
OG_05810	EYR68199.1	hypothetical protein AZ78_12230 [Lysobacter capsici AZ78]
OG_05811	EYR66537.1	nucleotide pyrophosphohydrolase [Lysobacter capsici AZ78]
OG_05812	EYR68197.1	hypothetical protein AZ78_12205 [Lysobacter capsici AZ78]
OG_05813	EYR66939.1	hypothetical protein AZ78_18375 [Lysobacter capsici AZ78]
OG_05814	EYR70295.1	prepilin [Lysobacter capsici AZ78]
OG_05815	EYR70020.1	hypothetical protein AZ78_02560 [Lysobacter capsici AZ78]
OG_05816	EYR69868.1	hypothetical protein AZ78_04680 [Lysobacter capsici AZ78]
OG_05817	EYR69458.1	3D-(3,5/4)-trihydroxycyclohexane-1,2-dione hydrolase [Lysobacter ca
OG_05818	EYR67187.1	chromosome partitioning protein ParA [Lysobacter capsici AZ78]
OG_05819	EYR66383.1	hypothetical protein AZ78_21215 [Lysobacter capsici AZ78]
OG_05820	EYR68553.1	hypothetical protein AZ78_09725 [Lysobacter capsici AZ78]
OG_05821	EYR68962.1	D-Ala-D-Ala carboxypeptidase [Lysobacter capsici AZ78]
OG_05822	EYR68805.1	hypothetical protein AZ78_08595 [Lysobacter capsici AZ78]
OG_05823	EYR69017.1	hypothetical protein AZ78_07695 [Lysobacter capsici AZ78]
OG_05824	EYR66065.1	hypothetical protein AZ78_23180 [Lysobacter capsici AZ78]
OG_05825	EYR67733.1	3-oxoadipate:succinyl-CoA transferase subunit B [Lysobacter capsici
OG_05826	EYR66323.1	hypothetical protein AZ78_21470 [Lysobacter capsici AZ78]
OG_05827	EYR66482.1	hypothetical protein AZ78_20895 [Lysobacter capsici AZ78]
OG_05828	EYR67715.1	ABC transporter ATP-binding protein [Lysobacter capsici AZ78]
OG_05829	EYR66929.1	hypothetical protein AZ78_18325 [Lysobacter capsici AZ78]
OG_05830	EYR70448.1	hypothetical protein AZ78_01315 [Lysobacter capsici AZ78]
OG_05831	EYR65832.1	hypothetical protein AZ78_24285 [Lysobacter capsici AZ78]
OG_05832	EYR69269.1	hypothetical protein AZ78_06780 [Lysobacter capsici AZ78]
OG_05833	EYR69460.1	ABC transporter ATP-binding protein [Lysobacter capsici AZ78]
OG_05834	EYR69454.1	porin [Lysobacter capsici AZ78]
OG_05835	EYR66928.1	TonB-denpendent receptor [Lysobacter capsici AZ78]
OG_05836	EYR70451.1	hypothetical protein AZ78_01355 [Lysobacter capsici AZ78]
OG_05837	EYR68963.1	transcriptional regulator [Lysobacter capsici AZ78]

OG_05838	LC55x_2908	idhA; inositol 2-dehydrogenase
OG_05839	LC55x_1901	LC55x_1901; conserved hypothetical protein
OG_05840	LC55x_5691	LC55x_5691; putative integron gene cassette protein
OG_05841	LC55x_5617	LC55x_5617; hypothetical protein
OG_05842	LC55x_5740	LC55x_5740; hypothetical protein
OG_05843	LC55x_3297	LC55x_3297; hypothetical protein
OG_05844	LC55x_2202	LC55x_2202; conserved hypothetical protein
OG_05845	LC55x_1378	LC55x_1378; flavin containing amine oxidoreductase family protein
OG_05846	LC55x_4556	LC55x_4556; hypothetical protein
OG_05847	LC55x_5473	LC55x_5473; hypothetical protein
OG_05848	LC55x_3574	LC55x_3574; hypothetical protein
OG_05849	LC55x_5481	LC55x_5481; hypothetical protein
OG_05850	LC55x_1475	LC55x_1475; hypothetical protein
OG_05851	LC55x_3745	LC55x_3745; hypothetical protein
OG_05852	LC55x_5420	LC55x_5420; beta-ketoacyl synthase, C-terminal domain protein
OG_05853	LC55x_1600	LC55x_1600; hypothetical protein
OG_05854	LC55x_4251	LC55x_4251; conserved hypothetical protein
OG_05855	LC55x_2873	LC55x_2873; hypothetical protein
OG_05856	LC55x_3736	LC55x_3736; H+ antiporter-2 family protein
OG_05857	LC55x_3385	LC55x_3385; hypothetical protein
OG_05858	LC55x_1251	LC55x_1251; hypothetical protein
OG_05859	LC55x_1976	LC55x_1976; hypothetical protein
OG_05860	LC55x_4572	LC55x_4572; hypothetical protein
OG_05861	LC55x_2203	LC55x_2203; ankyrin repeat family protein
OG_05862	LC55x_2401	LC55x_2401; hypothetical protein
OG_05863	LC55x_5493	LC55x_5493; putative barstar
OG_05864	LC55x_2972	LC55x_2972; hypothetical protein
OG_05865	LC55x_5450	LC55x_5450; hypothetical protein
OG_05866	LC55x_622	LC55x_622; phytanoyl-CoA dioxygenase family protein
OG_05867	LC55x_2730	LC55x_2730; conserved hypothetical protein
OG_05868	LC55x_1273	LC55x_1273; hypothetical protein
OG_05869	LC55x_879	LC55x_879; hypothetical protein
OG_05870	LC55x_5428	LC55x_5428; hypothetical protein
OG_05871	LC55x_293	LC55x_293; RHS Repeat family protein
OG_05872	LC55x_5478	LC55x_5478; cupin domain protein
OG_05873	LC55x_4558	LC55x_4558; hypothetical protein
OG_05874	LC55x_1884	LC55x_1884; hypothetical protein
OG_05876	LC55x_5445	LC55x_5445; efflux transporter, outer membrane factor (OMF) lipo, N
OG_05877	LC55x_2900	LC55x_2900; kdul/IolB family protein
OG_05878	LC55x_4523	LC55x_4523; acetyltransferase family protein
OG_05879	LC55x_436	LC55x_436; hypothetical protein
OG_05880	LC55x_5588	LC55x_5588; glyoxalase-like domain protein
OG_05881	LC55x_1503	LC55x_1503; short chain dehydrogenase family protein
OG_05883	LC55x_1135	LC55x_1135; conserved hypothetical protein
OG_05885	LC55x_3922	LC55x_3922; hypothetical protein
OG_05886	LC55x_701	LC55x_701; hypothetical protein
OG_05887	LC55x_5738	LC55x_5738; DEAD/DEAH box helicase family protein
OG_05888	LC55x_4815	LC55x_4815; KWG Leptospira family protein
OG_05889	LC55x_1374	LC55x_1374; conserved hypothetical protein
OG_05890	LC55x_4012	LC55x_4012; subtilase family protein
OG_05891	LC55x_2671	LC55x_2671; hypothetical protein
OG_05892	LC55x_3819	LC55x_3819; aspartyl protease family protein
OG_05893	LC55x_4828	LC55x_4828; hypothetical protein
OG_05894	LC55x_4581	LC55x_4581; bacteriophytochrome domain protein
OG_05895	LC55x_3298	LC55x_3298; hypothetical protein
OG_05896	LC55x_3315	LC55x_3315; hypothetical protein
OG_05897	LC55x_297	LC55x_297; hypothetical protein
OG_05898	LC55x_2276	LC55x_2276; hypothetical protein
OG_05899	LC55x_3737	LC55x_3737; hypothetical protein
OG_05900	LC55x_5533	LC55x_5533; hypothetical protein
OG_05901	LC55x_1051	LC55x_1051; metA-pathway of phenol degradation family protein
OG_05902	LC55x_3597	LC55x_3597; hypothetical protein
OG_05903	LC55x_1058	pcaB; 3-carboxy-cis,cis-muconate cycloisomerase
OG_05904	LC55x_3813	LC55x_3813; conserved hypothetical protein
OG_05905	LC55x_4797	LC55x_4797; major Facilitator Superfamily protein
OG_05907	LC55x_4836	LC55x_4836; dnaJ C terminal domain protein
OG_05909	LC55x_4293	LC55x_4293; phytanoyl-CoA dioxygenase family protein
OG_05911	LC55x_2766	LC55x_2766; rhodanese-like domain protein
OG_05912	LC55x_1462	LC55x_1462; flagellar hook-basal body family protein
OG_05913	LC55x_4190	LC55x_4190; hypothetical protein
OG_05914	LC55x_2726	LC55x_2726; hypothetical protein
OG_05915	LC55x_4031	LC55x_4031; proconvertase P-domain protein
OG_05916	LC55x_5403	LC55x_5403; lexA DNA binding domain protein
OG_05917	LC55x_5437	LC55x_5437; bacterial regulatory helix-turn-helix, AraC family protein
OG_05918	LC55x_5427	LC55x_5427; conserved hypothetical protein
OG_05919	LC55x_4988	LC55x_4988; hypothetical protein
OG_05920	LC55x_1271	LC55x_1271; bacterial regulatory, gntR family protein
OG_05921	LC55x_3474	LC55x_3474; mobA/MobL family protein
OG_05922	LC55x_5429	LC55x_5429; hypothetical protein
OG_05923	LC55x_883	LC55x_883; lysR substrate binding domain protein
OG_05924	LC55x_1032	LC55x_1032; hypothetical protein
OG_05925	LC55x_5472	LC55x_5472; hypothetical protein

OG_05838	EYR69464.1	myo-inositol 2-dehydrogenase [Lysobacter capsici AZ78]
OG_05839	EYR65993.1	hypothetical protein AZ78_23490 [Lysobacter capsici AZ78]
OG_05840	EYR65867.1	hypothetical protein AZ78_24025 [Lysobacter capsici AZ78]
OG_05841	EYR66257.1	hypothetical protein AZ78_22060 [Lysobacter capsici AZ78]
OG_05842	EYR69858.1	hypothetical protein AZ78_04475 [Lysobacter capsici AZ78]
OG_05843	EYR70145.1	hypothetical protein AZ78_02815 [Lysobacter capsici AZ78]
OG_05844	EYR67408.1	phospholipase A [Lysobacter capsici AZ78]
OG_05845	EYR67186.1	dehydrogenase [Lysobacter capsici AZ78]
OG_05846	EYR68566.1	hypothetical protein AZ78_09850 [Lysobacter capsici AZ78]
OG_05847	EYR69041.1	hypothetical protein AZ78_07945 [Lysobacter capsici AZ78]
OG_05848	EYR67522.1	hypothetical protein AZ78_15360 [Lysobacter capsici AZ78]
OG_05849	EYR69045.1	hypothetical protein AZ78_07975 [Lysobacter capsici AZ78]
OG_05850	EYR70419.1	hypothetical protein AZ78_00435 [Lysobacter capsici AZ78]
OG_05851	EYR67057.1	hypothetical protein AZ78_17500 [Lysobacter capsici AZ78]
OG_05852	EYR68910.1	beta-ketoacyl synthase [Lysobacter capsici AZ78]
OG_05853	EYR70440.1	hypothetical protein AZ78_01065 [Lysobacter capsici AZ78]
OG_05854	EYR69106.1	DNA methyltransferase [Lysobacter capsici AZ78]
OG_05855	EYR69432.1	hypothetical protein AZ78_05925 [Lysobacter capsici AZ78]
OG_05856	EYR67048.1	DSBA oxidoreductase [Lysobacter capsici AZ78]
OG_05857	EYR66567.1	hypothetical protein AZ78_20500 [Lysobacter capsici AZ78]
OG_05858	EYR65656.1	hypothetical protein AZ78_25180 [Lysobacter capsici AZ78]
OG_05859	EYR66788.1	hypothetical protein AZ78_19380 [Lysobacter capsici AZ78]
OG_05860	EYR68554.1	hypothetical protein AZ78_09755 [Lysobacter capsici AZ78]
OG_05861	EYR67409.1	phospholipase A [Lysobacter capsici AZ78]
OG_05862	EYR69829.1	hypothetical protein AZ78_03730 [Lysobacter capsici AZ78]
OG_05863	EYR68943.1	hypothetical protein AZ78_08035 [Lysobacter capsici AZ78]
OG_05864	EYR66190.1	hypothetical protein AZ78_21715 [Lysobacter capsici AZ78]
OG_05865	EYR69027.1	hypothetical protein AZ78_07830 [Lysobacter capsici AZ78]
OG_05866	EYR69992.1	hypothetical protein AZ78_02360 [Lysobacter capsici AZ78]
OG_05867	EYR69302.1	hypothetical protein AZ78_05260 [Lysobacter capsici AZ78]
OG_05868	EYR67708.1	hypothetical protein AZ78_15055 [Lysobacter capsici AZ78]
OG_05869	EYR66878.1	hypothetical protein AZ78_19040 [Lysobacter capsici AZ78]
OG_05870	EYR69020.1	hypothetical protein AZ78_07730 [Lysobacter capsici AZ78]
OG_05871	EYR70413.1	hypothetical protein AZ78_00330 [Lysobacter capsici AZ78]
OG_05872	EYR68937.1	cupin [Lysobacter capsici AZ78]
OG_05873	EYR68564.1	hypothetical protein AZ78_09840 [Lysobacter capsici AZ78]
OG_05874	EYR66020.1	hypothetical protein AZ78_23565 [Lysobacter capsici AZ78]
OG_05876	EYR68923.1	RND transporter [Lysobacter capsici AZ78]
OG_05877	EYR69456.1	5-deoxyglucuronate isomerase [Lysobacter capsici AZ78]
OG_05878	EYR68398.1	GCN5 family acetyltransferase [Lysobacter capsici AZ78]
OG_05879	EYR65522.1	hypothetical protein AZ78_25850 [Lysobacter capsici AZ78]
OG_05880	EYR65419.1	hypothetical protein AZ78_26380 [Lysobacter capsici AZ78]
OG_05881	EYR70233.1	NmrA family transcriptional regulator [Lysobacter capsici AZ78]
OG_05883	EYR68510.1	hypothetical protein AZ78_10860 [Lysobacter capsici AZ78]
OG_05885	EYR66674.1	hypothetical protein AZ78_19555 [Lysobacter capsici AZ78]
OG_05886	EYR70102.1	hypothetical protein AZ78_01995 [Lysobacter capsici AZ78]
OG_05887	EYR69700.1	type III restriction enzyme, res subunit [Lysobacter capsici AZ78]
OG_05888	EYR68838.1	hypothetical protein AZ78_09275 [Lysobacter capsici AZ78]
OG_05889	EYR67190.1	membrane protein [Lysobacter capsici AZ78]
OG_05890	EYR65566.1	hypothetical protein AZ78_25625 [Lysobacter capsici AZ78]
OG_05891	EYR68228.1	hypothetical protein AZ78_11220 [Lysobacter capsici AZ78]
OG_05892	EYR67123.1	hypothetical protein AZ78_17835 [Lysobacter capsici AZ78]
OG_05893	EYR68830.1	hypothetical protein AZ78_09215 [Lysobacter capsici AZ78]
OG_05894	EYR68367.1	sensor histidine kinase [Lysobacter capsici AZ78]
OG_05895	EYR70146.1	hypothetical protein AZ78_02820 [Lysobacter capsici AZ78]
OG_05896	EYR70149.1	hypothetical protein AZ78_02905 [Lysobacter capsici AZ78]
OG_05897	EYR67028.1	hypothetical protein AZ78_18105 [Lysobacter capsici AZ78]
OG_05898	EYR69852.1	hypothetical protein AZ78_04320 [Lysobacter capsici AZ78]
OG_05899	EYR67049.1	hypothetical protein AZ78_17460 [Lysobacter capsici AZ78]
OG_05900	EYR69066.1	hypothetical protein AZ78_08220 [Lysobacter capsici AZ78]
OG_05901	EYR67736.1	hypothetical protein AZ78_14040 [Lysobacter capsici AZ78]
OG_05902	EYR67541.1	hypothetical protein AZ78_15455 [Lysobacter capsici AZ78]
OG_05903	EYR67729.1	3-carboxy-cis,cis-muconate cycloisomerase [Lysobacter capsici AZ7
OG_05904	EYR67117.1	hypothetical protein AZ78_17805 [Lysobacter capsici AZ78]
OG_05905	EYR68780.1	MFS transporter [Lysobacter capsici AZ78]
OG_05907	EYR68758.1	molecular chaperone DnaJ [Lysobacter capsici AZ78]
OG_05909	EYR69147.1	phytanoyl-CoA dioxygenase [Lysobacter capsici AZ78]
OG_05911	EYR69334.1	hypothetical protein AZ78_05430 [Lysobacter capsici AZ78]
OG_05912	EYR70200.1	flagellar hook-basal body protein [Lysobacter capsici AZ78]
OG_05913	EYR68187.1	hypothetical protein AZ78_12120 [Lysobacter capsici AZ78]
OG_05914	EYR69299.1	hypothetical protein AZ78_05245 [Lysobacter capsici AZ78]
OG_05915	EYR65810.1	hypothetical protein AZ78_24170 [Lysobacter capsici AZ78]
OG_05916	EYR68625.1	hypothetical protein AZ78_10805 [Lysobacter capsici AZ78]
OG_05917	EYR68919.1	AraC family transcriptional regulator [Lysobacter capsici AZ78]
OG_05918	EYR68914.1	antibiotic biosynthesis monooxygenase [Lysobacter capsici AZ78]
OG_05919	EYR67240.1	hypothetical protein AZ78_16850 [Lysobacter capsici AZ78]
OG_05920	EYR67676.1	GntR family transcriptional regulator [Lysobacter capsici AZ78]
OG_05921	EYR68500.1	plasmid mobilization protein [Lysobacter capsici AZ78]
OG_05922	EYR69021.1	hypothetical protein AZ78_07735 [Lysobacter capsici AZ78]
OG_05923	EYR66874.1	hypothetical protein AZ78_19020 [Lysobacter capsici AZ78]
OG_05924	EYR67750.1	hypothetical protein AZ78_14120 [Lysobacter capsici AZ78]
OG_05925	EYR69039.1	hypothetical protein AZ78_07935 [Lysobacter capsici AZ78]

OG_05926	LC55x_4659	LC55x_4659; ribosomal L7/L12 C-terminal domain protein	OG_05926	EYR70402.1	hypothetical protein AZ78_00120 [Lysobacter capsici AZ78]
OG_05927	LC55x_2815	LC55x_2815; hypothetical protein	OG_05927	EYR69376.1	hypothetical protein AZ78_05640 [Lysobacter capsici AZ78]
OG_05928	LC55x_4569	LC55x_4569; subtilase family protein	OG_05928	EYR68557.1	hypothetical protein AZ78_09770 [Lysobacter capsici AZ78]
OG_05929	LC55x_1787	LC55x_1787; hypothetical protein	OG_05929	EYR67314.1	hypothetical protein AZ78_16205 [Lysobacter capsici AZ78]
OG_05930	LC55x_4053	LC55x_4053; methyltransferase domain protein	OG_05930	EYR65833.1	hypothetical protein AZ78_24290 [Lysobacter capsici AZ78]
OG_05931	LC55x_5452	LC55x_5452; putative integron gene cassette domain protein	OG_05931	EYR69028.1	hypothetical protein AZ78_07840 [Lysobacter capsici AZ78]
OG_05932	LC55x_3181	LC55x_3181; hypothetical protein	OG_05932	EYR66923.1	hypothetical protein AZ78_18295 [Lysobacter capsici AZ78]
OG_05933	LC55x_2905	LC55x_2905; branched-chain amino acid transport system / permeas	OG_05933	EYR69461.1	ATPase [Lysobacter capsici AZ78]
OG_05934	LC55x_654	LC55x_654; peptidase M13 family protein	OG_05934	EYR69969.1	peptidase [Lysobacter capsici AZ78]
OG_05935	LC55x_4996	LC55x_4996; hypothetical protein	OG_05935	EYR67233.1	hypothetical protein AZ78_16815 [Lysobacter capsici AZ78]
OG_05936	LC55x_5417	LC55x_5417; phosphopantetheine attachment site family protein	OG_05936	EYR69015.1	hypothetical protein AZ78_07675 [Lysobacter capsici AZ78]
OG_05937	LC55x_4656	LC55x_4656; conserved hypothetical protein	OG_05937	EYR70399.1	hypothetical protein AZ78_00105 [Lysobacter capsici AZ78]
OG_05938	LC55x_1812	LC55x_1812; hypothetical protein	OG_05938	EYR67320.1	hypothetical protein AZ78_16305 [Lysobacter capsici AZ78]
OG_05939	LC55x_1515	LC55x_1515; hxlR-like helix-turn-helix family protein	OG_05939	EYR70244.1	MarR family transcriptional regulator [Lysobacter capsici AZ78]
OG_05940	LC55x_2896	LC55x_2896; subtilase family protein	OG_05940	EYR69452.1	hypothetical protein AZ78_06025 [Lysobacter capsici AZ78]
OG_05941	LC55x_1559	LC55x_1559; hypothetical protein	OG_05941	EYR70435.1	hypothetical protein AZ78_00890 [Lysobacter capsici AZ78]
OG_05942	LC55x_4507	LC55x_4507; hypothetical protein	OG_05942	EYR68583.1	hypothetical protein AZ78_10090 [Lysobacter capsici AZ78]
OG_05943	LC55x_5599	LC55x_5599; hypothetical protein	OG_05943	EYR66238.1	hypothetical protein AZ78_21965 [Lysobacter capsici AZ78]
OG_05944	LC55x_4636	LC55x_4636; hypothetical protein	OG_05944	EYR70390.1	hypothetical protein AZ78_00005 [Lysobacter capsici AZ78]
OG_05945	LC55x_81	LC55x_81; hypothetical protein	OG_05945	EYR69874.1	hypothetical protein AZ78_04810 [Lysobacter capsici AZ78]
OG_05946	LC55x_585	LC55x_585; hypothetical protein	OG_05946	EYR69863.1	hypothetical protein AZ78_04620 [Lysobacter capsici AZ78]
OG_05947	LC55x_2991	LC55x_2991; CAAX protease self-immunity family protein	OG_05947	EYR67793.1	abortive infection protein [Lysobacter capsici AZ78]
OG_05948	LC55x_4794	LC55x_4794; transcriptional regulatory , C terminal family protein	OG_05948	EYR68781.1	transcriptional regulator [Lysobacter capsici AZ78]
OG_05949	LC55x_2701	LC55x_2701; cupin-like domain protein	OG_05949	EYR69286.1	hypothetical protein AZ78_05170 [Lysobacter capsici AZ78]
OG_05950	LC55x_4841	LC55x_4841; hypothetical protein	OG_05950	EYR68827.1	hypothetical protein AZ78_09145 [Lysobacter capsici AZ78]
OG_05951	LC55x_5439	LC55x_5439; short chain dehydrogenase family protein	OG_05951	EYR68921.1	short-chain dehydrogenase [Lysobacter capsici AZ78]
OG_05952	LC55x_2352	LC55x_2352; hypothetical protein	OG_05952	EYR69842.1	hypothetical protein AZ78_03955 [Lysobacter capsici AZ78]
OG_05953	LC55x_4528	LC55x_4528; hypothetical protein	OG_05953	EYR68394.1	hypothetical protein AZ78_09990 [Lysobacter capsici AZ78]
OG_05955	LC55x_2278	LC55x_2278; ABC transporter family protein	OG_05955	EYR69678.1	ABC transporter ATP-binding protein [Lysobacter capsici AZ78]
OG_05956	LC55x_1380	LC55x_1380; conserved hypothetical protein	OG_05956	EYR67184.1	hypothetical protein AZ78_17325 [Lysobacter capsici AZ78]
OG_05957	LC55x_4516	LC55x_4516; conserved hypothetical protein	OG_05957	EYR68642.1	RNA signal recognition particle 4.5S RNA [Lysobacter capsici AZ78]
OG_05958	LC55x_1347	LC55x_1347; hypothetical protein	OG_05958	EYR70085.1	hypothetical protein AZ78_01670 [Lysobacter capsici AZ78]
OG_05959	LC55x_1953	LC55x_1953; hypothetical protein	OG_05959	EYR66008.1	hypothetical protein AZ78_23265 [Lysobacter capsici AZ78]
OG_05960	LC55x_4877	LC55x_4877; hypothetical protein	OG_05960	EYR68818.1	hypothetical protein AZ78_08985 [Lysobacter capsici AZ78]
OG_05961	LC55x_5474	LC55x_5474; hypothetical protein	OG_05961	EYR69040.1	hypothetical protein AZ78_07940 [Lysobacter capsici AZ78]
OG_05962	LC55x_5082	LC55x_5082; hypothetical protein	OG_05962	EYR68094.1	hypothetical protein AZ78_12755 [Lysobacter capsici AZ78]
OG_05963	LC55x_1471	LC55x_1471; hypothetical protein	OG_05963	EYR70416.1	hypothetical protein AZ78_00415 [Lysobacter capsici AZ78]
OG_05964	LC55x_1996	LC55x_1996; beta-lactamase family protein	OG_05964	EYR66759.1	beta-lactamase [Lysobacter capsici AZ78]
OG_05965	LC55x_1512	LC55x_1512; NAD dependent epimerase/dehydratase family protein	OG_05965	EYR70242.1	flavin reductase [Lysobacter capsici AZ78]
OG_05966	LC55x_4817	LC55x_4817; hypothetical protein	OG_05966	EYR68836.1	hypothetical protein AZ78_09265 [Lysobacter capsici AZ78]
OG_05967	LC55x_5087	LC55x_5087; outer membrane autotransporter barrel domain protein	OG_05967	EYR69267.1	hypothetical protein AZ78_06765 [Lysobacter capsici AZ78]
OG_05968	LC55x_1480	LC55x_1480; hypothetical protein	OG_05968	EYR70421.1	hypothetical protein AZ78_00460 [Lysobacter capsici AZ78]
OG_05969	LC55x_1060	pobA; 4-hydroxybenzoate 3-monooxygenase	OG_05969	EYR67727.1	4-hydroxybenzoate 3-monooxygenase [Lysobacter capsici AZ78]
OG_05970	LC55x_3430	LC55x_3430; hypothetical protein	OG_05970	EYR66556.1	hypothetical protein AZ78_20300 [Lysobacter capsici AZ78]
OG_05971	LC55x_5561	LC55x_5561; marR family protein	OG_05971	EYR69072.1	hypothetical protein AZ78_08365 [Lysobacter capsici AZ78]
OG_05972	LC55x_1474	LC55x_1474; hypothetical protein	OG_05972	EYR70418.1	hypothetical protein AZ78_00430 [Lysobacter capsici AZ78]
OG_05973	LC55x_3253	LC55x_3253; conserved hypothetical protein	OG_05973	EYR67930.1	hypothetical protein AZ78_13055 [Lysobacter capsici AZ78]
OG_05974	LC55x_2231	LC55x_2231; macro domain protein	OG_05974	EYR67426.1	hypothetical protein AZ78_15970 [Lysobacter capsici AZ78]
OG_05975	LC55x_5063	LC55x_5063; hypothetical protein	OG_05975	EYR68097.1	hypothetical protein AZ78_12820 [Lysobacter capsici AZ78]
OG_05976	LC55x_1375	LC55x_1375; conserved hypothetical protein	OG_05976	EYR67189.1	hypothetical protein AZ78_17350 [Lysobacter capsici AZ78]
OG_05977	LC55x_1677	LC55x_1677; acyltransferase family protein	OG_05977	EYR70344.1	acyltransferase [Lysobacter capsici AZ78]
OG_05978	LC55x_2593	LC55x_2593; hypothetical protein	OG_05978	EYR68302.1	hypothetical protein AZ78_11595 [Lysobacter capsici AZ78]
OG_05979	LC55x_1076	LC55x_1076; hypothetical protein	OG_05979	EYR67784.1	hypothetical protein AZ78_13925 [Lysobacter capsici AZ78]
OG_05980	LC55x_1676	LC55x_1676; hypothetical protein	OG_05980	EYR70450.1	hypothetical protein AZ78_01325 [Lysobacter capsici AZ78]
OG_05981	LC55x_3406	alpha-LP; alpha-lytic protease	OG_05981	EYR66515.1	serine protease [Lysobacter capsici AZ78]
OG_05982	LC55x_2217	LC55x_2217; small Multidrug Resistance family protein	OG_05982	EYR67415.1	multidrug transporter [Lysobacter capsici AZ78]
OG_05983	LC55x_5514	LC55x_5514; putative whole genome shotgun sequence assembly, c	OG_05983	EYR68954.1	hypothetical protein AZ78_08135 [Lysobacter capsici AZ78]
OG_05984	LC55x_1985	LC55x_1985; hypothetical protein	OG_05984	EYR66786.1	hypothetical protein AZ78_19350 [Lysobacter capsici AZ78]
OG_05985	LC55x_2987	LC55x_2987; hypothetical protein	OG_05985	EYR67792.1	hypothetical protein AZ78_13400 [Lysobacter capsici AZ78]
OG_05986	LC55x_3116	LC55x_3116; hypothetical protein	OG_05986	EYR66978.1	hypothetical protein AZ78_18580 [Lysobacter capsici AZ78]
OG_05987	LC55x_2729	LC55x_2729; lipase family protein	OG_05987	EYR69301.1	hypothetical protein AZ78_05255 [Lysobacter capsici AZ78]
OG_05988	LC55x_3378	LC55x_3378; hypothetical protein	OG_05988	EYR66569.1	hypothetical protein AZ78_20525 [Lysobacter capsici AZ78]
OG_05989	LC55x_1442	LC55x_1442; hypothetical protein	OG_05989	EYR67134.1	hypothetical protein AZ78_17075 [Lysobacter capsici AZ78]
OG_05990	LC55x_3151	LC55x_3151; hypothetical protein	OG_05990	EYR66947.1	hypothetical protein AZ78_18415 [Lysobacter capsici AZ78]
OG_05991	LC55x_4771	LC55x_4771; peptidase M23 family protein	OG_05991	EYR66077.1	hypothetical protein AZ78_22735 [Lysobacter capsici AZ78]
OG_05992	LC55x_4854	LC55x_4854; short chain dehydrogenase family protein	OG_05992	EYR68742.1	AraC family transcriptional regulator [Lysobacter capsici AZ78]
OG_05993	LC55x_5163	LC55x_5163; conserved hypothetical protein	OG_05993	EYR68076.1	hypothetical protein AZ78_12360 [Lysobacter capsici AZ78]
OG_05995	LC55x_3299	LC55x_3299; hypothetical protein	OG_05995	EYR70147.1	hypothetical protein AZ78_02825 [Lysobacter capsici AZ78]
OG_05996	LC55x_4570	LC55x_4570; trypsin-like peptidase domain protein	OG_05996	EYR68556.1	hypothetical protein AZ78_09765 [Lysobacter capsici AZ78]
OG_05997	LC55x_1579	pilV; type IV pilus modification protein PilV	OG_05997	EYR70296.1	prepilin [Lysobacter capsici AZ78]
OG_05998	LC55x_1372	LC55x_1372; lipocalin-like domain protein	OG_05998	EYR67192.1	membrane protein [Lysobacter capsici AZ78]
OG_06000	LC55x_5663	LC55x_5663; hypothetical protein	OG_06000	EYR66297.1	hypothetical protein AZ78_22265 [Lysobacter capsici AZ78]
OG_06001	LC55x_2764	LC55x_2764; hypothetical protein	OG_06001	EYR69331.1	hypothetical protein AZ78_05415 [Lysobacter capsici AZ78]
OG_06002	LC55x_1999	LC55x_1999; hypothetical protein	OG_06002	EYR66780.1	hypothetical protein AZ78_19285 [Lysobacter capsici AZ78]
OG_06003	LC55x_1951	LC55x_1951; conserved hypothetical protein	OG_06003	EYR65959.1	hypothetical protein AZ78_23275 [Lysobacter capsici AZ78]
OG_06004	LC55x_192	LC55x_192; hypothetical protein	OG_06004	EYR66132.1	hypothetical protein AZ78_22450 [Lysobacter capsici AZ78]
OG_06005	LC55x_4759	LC55x_4759; hypothetical protein	OG_06005	EYR66088.1	hypothetical protein AZ78_22790 [Lysobacter capsici AZ78]
OG_06006	LC55x_3228	LC55x_3228; hypothetical protein	OG_06006	EYR67961.1	hypothetical protein AZ78_13215 [Lysobacter capsici AZ78]
OG_06007	LC55x_1750	LC55x_1750; conserved hypothetical protein	OG_06007	EYR65699.1	hypothetical protein AZ78_24930 [Lysobacter capsici AZ78]
OG_06008	LC55x_4816	LC55x_4816; hypothetical protein	OG_06008	EYR68837.1	hypothetical protein AZ78_09270 [Lysobacter capsici AZ78]
OG_06009	LC55x_671	LC55x_671; conserved hypothetical protein	OG_06009	EYR69961.1	hypothetical protein AZ78_02145 [Lysobacter capsici AZ78]
OG_06010	LC55x_3111	LC55x_3111; hypothetical protein	OG_06010	EYR65456.1	hypothetical protein AZ78_26280 [Lysobacter capsici AZ78]

OG_06011	LC55x_3347	LC55x_3347; hypothetical protein
OG_06012	LC55x_1952	LC55x_1952; killing trait family protein
OG_06013	LC55x_5416	LC55x_5416; hypothetical protein
OG_06014	LC55x_50	LC55x_50; hypothetical protein
OG_06015	LC55x_5702	LC55x_5702; hypothetical protein
OG_06016	LC55x_4515	LC55x_4515; conserved hypothetical protein
OG_06017	LC55x_2876	LC55x_2876; hypothetical protein
OG_06018	LC55x_4178	LC55x_4178; hypothetical protein
OG_06019	LC55x_5526	LC55x_5526; response regulator
OG_06020	LC55x_3112	LC55x_3112; conserved hypothetical protein
OG_06021	LC55x_4624	LC55x_4624; hypothetical protein
OG_06022	LC55x_4652	LC55x_4652; ankyrin repeat family protein
OG_06023	LC55x_4649	LC55x_4649; putative predicted protein
OG_06024	LC55x_5690	LC55x_5690; conserved hypothetical protein
OG_06025	LC55x_2205	LC55x_2205; DNA directed RNA polymerase, 7 kDa subunit
OG_06026	LC55x_2899	mmsA; methylmalonate-semialdehyde dehydrogenase
OG_06027	LC55x_5626	LC55x_5626; hypothetical protein
OG_06028	LC55x_26	LC55x_26; hypothetical protein
OG_06029	LC55x_3734	LC55x_3734; conserved hypothetical protein
OG_06030	LC55x_3227	LC55x_3227; conserved hypothetical protein
OG_06031	LC55x_1990	LC55x_1990; conserved hypothetical protein
OG_06032	LC55x_5459	LC55x_5459; hypothetical protein
OG_06033	LC55x_2892	LC55x_2892; hypothetical protein
OG_06034	LC55x_678	LC55x_678; osmC-like family protein
OG_06035	LC55x_2260	LC55x_2260; hypothetical protein
OG_06036	LC55x_4006	LC55x_4006; hypothetical protein
OG_06037	LC55x_3241	LC55x_3241; conserved hypothetical protein
OG_06038	LC55x_132	LC55x_132; peptidase S41 family protein
OG_06039	LC55x_3527	LC55x_3527; hypothetical protein
OG_06040	LC55x_2372	LC55x_2372; hypothetical protein
OG_06041	LC55x_1583	LC55x_1583; putative prepilin like protein
OG_06042	LC55x_1558	LC55x_1558; short chain dehydrogenase family protein
OG_06043	LC55x_4571	LC55x_4571; trypsin-like peptidase domain protein
OG_06044	LC55x_5585	LC55x_5585; hypothetical protein
OG_06045	LC55x_811	LC55x_811; peptidase S41 family protein
OG_06046	LC55x_5531	LC55x_5531; histidine kinase family protein
OG_06047	LC55x_2300	LC55x_2300; DSBA-like thioredoxin domain protein
OG_06048	LC55x_1891	LC55x_1891; hypothetical protein
OG_06049	LC55x_267	LC55x_267; hypothetical protein
OG_06050	LC55x_1055	pcaF; 3-oxoadipyl-CoA thiolase
OG_06051	LC55x_2635	LC55x_2635; conserved hypothetical protein
OG_06052	LC55x_2686	LC55x_2686; hypothetical protein
OG_06053	LC55x_4997	LC55x_4997; hypothetical protein
OG_06054	LC55x_5563	LC55x_5563; beta-lactamase family protein
OG_06055	LC55x_556	LC55x_556; helix-turn-helix family protein
OG_06056	LC55x_3699	LC55x_3699; hypothetical protein
OG_06057	LC55x_3723	LC55x_3723; right handed beta helix region family protein
OG_06059	LC55x_1876	LC55x_1876; hypothetical protein
OG_06060	LC55x_3098	LC55x_3098; hypothetical protein
OG_06061	LC55x_5534	LC55x_5534; AAA domain protein
OG_06062	LC55x_53	LC55x_53; hypothetical protein
OG_06063	LC55x_5603	LC55x_5603; hypothetical protein
OG_06064	LC55x_1211	LC55x_1211; hypothetical protein
OG_06065	LC55x_3084	LC55x_3084; hypothetical protein
OG_06066	LC55x_5542	LC55x_5542; WD40-like Beta Propeller Repeat family protein
OG_06067	LC55x_1389	LC55x_1389; immunity Imm1 family protein
OG_06068	LC55x_1293	LC55x_1293; conserved hypothetical protein
OG_06069	LC55x_4512	LC55x_4512; hypothetical protein
OG_06070	LC55x_3744	LC55x_3744; hypothetical protein
OG_06071	LC55x_4329	LC55x_4329; hypothetical protein
OG_06072	LC55x_2906	LC55x_2906; periplasmic binding domain protein
OG_06073	LC55x_2303	LC55x_2303; hypothetical protein
OG_06074	LC55x_3125	LC55x_3125; beta-lactamase family protein
OG_06075	LC55x_5132	LC55x_5132; hypothetical protein
OG_06076	LC55x_5525	LC55x_5525; istB-like ATP binding family protein
OG_06077	LC55x_2146	LC55x_2146; hypothetical protein
OG_06078	LC55x_4634	LC55x_4634; hypothetical protein
OG_06079	LC55x_5582	LC55x_5582; 2Fe-2S iron-sulfur cluster binding domain protein
OG_06080	LC55x_5451	LC55x_5451; beta-lactamase family protein
OG_06081	LC55x_1886	LC55x_1886; hypothetical protein
OG_06082	LC55x_5461	LC55x_5461; hypothetical protein
OG_06083	LC55x_639	LC55x_639; response regulator
OG_06084	LC55x_1129	LC55x_1129; hypothetical protein
OG_06085	LC55x_2301	LC55x_2301; hypothetical protein
OG_06086	LC55x_5039	LC55x_5039; hypothetical protein
OG_06087	LC55x_1350	LC55x_1350; hypothetical protein
OG_06088	LC55x_4148	LC55x_4148; conserved hypothetical protein
OG_06089	LC55x_145	LC55x_145; hypothetical protein
OG_06090	LC55x_4595	LC55x_4595; putative peptidoglycan binding domain protein
OG_06091	LC55x_3293	LC55x_3293; hypothetical protein
OG_06092	LC55x_2909	LC55x_2909; oxidoreductase , NAD-binding Rossmann fold family pr
OG_06094	LC55x_3440	LC55x_3440; hypothetical protein

OG_06011	EYR66581.1	hypothetical protein AZ78_20670 [Lysobacter capsici AZ78]
OG_06012	EYR66009.1	hypothetical protein AZ78_23270 [Lysobacter capsici AZ78]
OG_06013	EYR69014.1	hypothetical protein AZ78_07670 [Lysobacter capsici AZ78]
OG_06014	EYR69866.1	hypothetical protein AZ78_04670 [Lysobacter capsici AZ78]
OG_06015	EYR65856.1	hypothetical protein AZ78_23970 [Lysobacter capsici AZ78]
OG_06016	EYR68405.1	hypothetical protein AZ78_10055 [Lysobacter capsici AZ78]
OG_06017	EYR69435.1	hypothetical protein AZ78_05940 [Lysobacter capsici AZ78]
OG_06018	EYR68198.1	hypothetical protein AZ78_12210 [Lysobacter capsici AZ78]
OG_06019	EYR68961.1	histidine kinase [Lysobacter capsici AZ78]
OG_06020	EYR65457.1	hypothetical protein AZ78_26285 [Lysobacter capsici AZ78]
OG_06021	EYR68544.1	hypothetical protein AZ78_09515 [Lysobacter capsici AZ78]
OG_06022	EYR70160.1	ankyrin [Lysobacter capsici AZ78]
OG_06023	EYR70395.1	hypothetical protein AZ78_00070 [Lysobacter capsici AZ78]
OG_06024	EYR65868.1	hypothetical protein AZ78_24030 [Lysobacter capsici AZ78]
OG_06025	EYR67455.1	hypothetical protein AZ78_15850 [Lysobacter capsici AZ78]
OG_06026	EYR69455.1	methylmalonate-semialdehyde dehydrogenase [Lysobacter capsici A
OG_06027	EYR66266.1	hypothetical protein AZ78_22105 [Lysobacter capsici AZ78]
OG_06028	EYR69862.1	hypothetical protein AZ78_04610 [Lysobacter capsici AZ78]
OG_06029	EYR67046.1	thioesterase [Lysobacter capsici AZ78]
OG_06030	EYR67962.1	hypothetical protein AZ78_13220 [Lysobacter capsici AZ78]
OG_06031	EYR66763.1	hypothetical protein AZ78_19325 [Lysobacter capsici AZ78]
OG_06032	EYR69032.1	hypothetical protein AZ78_07870 [Lysobacter capsici AZ78]
OG_06033	EYR69448.1	hypothetical protein AZ78_06005 [Lysobacter capsici AZ78]
OG_06034	EYR69957.1	osmotically inducible protein C [Lysobacter capsici AZ78]
OG_06035	EYR67468.1	hypothetical protein AZ78_16105 [Lysobacter capsici AZ78]
OG_06036	EYR65561.1	hypothetical protein AZ78_25600 [Lysobacter capsici AZ78]
OG_06037	EYR67940.1	hypothetical protein AZ78_13105 [Lysobacter capsici AZ78]
OG_06038	EYR69885.1	hypothetical protein AZ78_05035 [Lysobacter capsici AZ78]
OG_06039	EYR67479.1	hypothetical protein AZ78_15140 [Lysobacter capsici AZ78]
OG_06040	EYR69836.1	hypothetical protein AZ78_03860 [Lysobacter capsici AZ78]
OG_06041	EYR70299.1	pilus assembly protein PilE [Lysobacter capsici AZ78]
OG_06042	EYR70279.1	DltE [Lysobacter capsici AZ78]
OG_06043	EYR68555.1	hypothetical protein AZ78_09760 [Lysobacter capsici AZ78]
OG_06044	EYR65417.1	hypothetical protein AZ78_26370 [Lysobacter capsici AZ78]
OG_06045	EYR67588.1	peptidase S41 [Lysobacter capsici AZ78]
OG_06046	EYR68964.1	histidine kinase [Lysobacter capsici AZ78]
OG_06047	EYR69848.1	hypothetical protein AZ78_04200 [Lysobacter capsici AZ78]
OG_06048	EYR66017.1	hypothetical protein AZ78_23535 [Lysobacter capsici AZ78]
OG_06049	EYR66994.1	hypothetical protein AZ78_17930 [Lysobacter capsici AZ78]
OG_06050	EYR67732.1	acetyl-CoA acetyltransferase [Lysobacter capsici AZ78]
OG_06051	EYR68261.1	hypothetical protein AZ78_11385 [Lysobacter capsici AZ78]
OG_06052	EYR68215.1	hypothetical protein AZ78_11155 [Lysobacter capsici AZ78]
OG_06053	EYR67232.1	hypothetical protein AZ78_16810 [Lysobacter capsici AZ78]
OG_06054	EYR69073.1	hypothetical protein AZ78_08375 [Lysobacter capsici AZ78]
OG_06055	EYR67916.1	hypothetical protein AZ78_12985 [Lysobacter capsici AZ78]
OG_06056	EYR65920.1	hypothetical protein AZ78_23715 [Lysobacter capsici AZ78]
OG_06057	EYR65902.1	hypothetical protein AZ78_23625 [Lysobacter capsici AZ78]
OG_06059	EYR66069.1	hypothetical protein AZ78_23200 [Lysobacter capsici AZ78]
OG_06060	EYR65447.1	hypothetical protein AZ78_26235 [Lysobacter capsici AZ78]
OG_06061	EYR68966.1	ATPase [Lysobacter capsici AZ78]
OG_06062	EYR69869.1	hypothetical protein AZ78_04685 [Lysobacter capsici AZ78]
OG_06063	EYR66243.1	hypothetical protein AZ78_21990 [Lysobacter capsici AZ78]
OG_06064	EYR65774.1	hypothetical protein AZ78_24450 [Lysobacter capsici AZ78]
OG_06065	EYR67883.1	hypothetical protein AZ78_13850 [Lysobacter capsici AZ78]
OG_06066	EYR68973.1	hypothetical protein AZ78_08270 [Lysobacter capsici AZ78]
OG_06067	EYR67179.1	hypothetical protein AZ78_17300 [Lysobacter capsici AZ78]
OG_06068	EYR67661.1	hypothetical protein AZ78_14965 [Lysobacter capsici AZ78]
OG_06069	EYR68582.1	hypothetical protein AZ78_10070 [Lysobacter capsici AZ78]
OG_06070	EYR67056.1	hypothetical protein AZ78_17495 [Lysobacter capsici AZ78]
OG_06071	EYR69270.1	hypothetical protein AZ78_06790 [Lysobacter capsici AZ78]
OG_06072	EYR69462.1	sugar ABC transporter substrate-binding protein [Lysobacter capsici /
OG_06073	EYR69845.1	hypothetical protein AZ78_04185 [Lysobacter capsici AZ78]
OG_06074	EYR66969.1	hypothetical protein AZ78_18535 [Lysobacter capsici AZ78]
OG_06075	EYR68082.1	hypothetical protein AZ78_12510 [Lysobacter capsici AZ78]
OG_06076	EYR68960.1	circadian clock protein KaiC [Lysobacter capsici AZ78]
OG_06077	EYR67450.1	hypothetical protein AZ78_15580 [Lysobacter capsici AZ78]
OG_06078	EYR68541.1	hypothetical protein AZ78_09470 [Lysobacter capsici AZ78]
OG_06079	EYR68806.1	hypothetical protein AZ78_08605 [Lysobacter capsici AZ78]
OG_06080	EYR68928.1	beta-lactamase [Lysobacter capsici AZ78]
OG_06081	EYR66019.1	hypothetical protein AZ78_23555 [Lysobacter capsici AZ78]
OG_06082	EYR69034.1	hypothetical protein AZ78_07880 [Lysobacter capsici AZ78]
OG_06083	EYR69980.1	LytTR family transcriptional regulator [Lysobacter capsici AZ78]
OG_06084	EYR68508.1	hypothetical protein AZ78_10840 [Lysobacter capsici AZ78]
OG_06085	EYR69847.1	hypothetical protein AZ78_04195 [Lysobacter capsici AZ78]
OG_06086	EYR66300.1	hypothetical protein AZ78_22280 [Lysobacter capsici AZ78]
OG_06087	EYR70084.1	hypothetical protein AZ78_01655 [Lysobacter capsici AZ78]
OG_06088	EYR66333.1	hypothetical protein AZ78_21520 [Lysobacter capsici AZ78]
OG_06089	EYR69889.1	hypothetical protein AZ78_05090 [Lysobacter capsici AZ78]
OG_06090	EYR68357.1	peptidoglycan-binding protein [Lysobacter capsici AZ78]
OG_06091	EYR70143.1	hypothetical protein AZ78_02800 [Lysobacter capsici AZ78]
OG_06092	EYR69465.1	oxidoreductase [Lysobacter capsici AZ78]
OG_06094	EYR66643.1	hypothetical protein AZ78_19885 [Lysobacter capsici AZ78]

OG_06095	LC55x_1510	LC55x_1510; putative oxidoreductase, Zn-dependent and NAD(P)-bir
OG_06096	LC55x_5584	LC55x_5584; tonB dependent receptor family protein
OG_06097	LC55x_3170	LC55x_3170; polysaccharide lyase family 8, super-sandwich domain
OG_06098	LC55x_4172	LC55x_4172; hypothetical protein
OG_06099	LC55x_4339	LC55x_4339; hypothetical protein

OG_06095	EYR70240.1	zinc-binding dehydrogenase [Lysobacter capsici AZ78]
OG_06096	EYR65416.1	TonB-dependent receptor [Lysobacter capsici AZ78]
OG_06097	EYR66931.1	hypothetical protein AZ78_18335 [Lysobacter capsici AZ78]
OG_06098	EYR68200.1	hypothetical protein AZ78_12245 [Lysobacter capsici AZ78]
OG_06099	EYR69271.1	hypothetical protein AZ78_06835 [Lysobacter capsici AZ78]
