# Supplementary material for: Comparative genomics and metabolic profiling of the genus Lysobacter
Source: BMC Genomics. 2015 Nov 23;16:991. doi: 10.1186/s12864-015-2191-z (PMC4657364; doi:10.1186/s12864-015-2191-z)
Supplement: Additional file 3: Tables S4 to S15. — (PDF 877 kb) [file 12864_2015_2191_MOESM3_ESM.pdf]

Supplementary tables S12

Comparative genomics and metabolic profiling of the genus *Lysobacter*

Irene de Bruijn*, Xu Cheng, Victor de Jager, Ruth Gómez Expósito, Jeramie Watrous, Nrupali Patel, Joeke Postma, Pieter C. Dorrestein, Donald Kobayashi and Jos M. Raaijmakers.

*Correspondence: **Dr. Irene de Bruijn:** i.debruijn@nioo.knaw.nl

Table S4. pp 2. Antimicrobial and extracellular enzyme activity of the *Lysobacter* strains.

Table S5. pp 3. Number of CDSs encoding peptidases containing a glycosyl hydrolase catalytic domain with chitinase activity in *Lysobacter*, *Stenotrophomonas* and *Xanthomonas* spp.

Table S6. pp. 4. CDSs encoding peptidases containing a glycoside hydrolase domain with chitinase activity.

Table S7. pp. 5. Number of CDSs encoding peptidases containing a glycosyl hydrolase catalytic domain with glucanase activity in *Lysobacter*, *Stenotrophomonas* and *Xanthomonas* spp.

Table S8. pp. 6. CDSs encoding peptidases containing glycoside hydrolase domain with glucanase activity.

Table S9. pp. 7. Number of CDSs encoding peptidase identified by BLASTp analysis in MEROPS database.

Table S10. pp. 10. Blastp analysis of the predicted protein sequences of the peptidase identified in *Lysobacter gummosus* DSMZ60980 (Gokcen et al, 2014).

Table S11. pp. 12. Overview of the antimicrobial compounds produced by *Lysobacter*.

Table S12. pp. 14. Overview of reference protein sequences used for blastp analysis in the Lysobacter genomes. '+' indicates a hit in the genome sequence with cut-off E value of e-05 and >60 % identity (>70% for NRPS). Not detected is indicated with '-'.

Table S13. pp 23. NRPS/PKS clusters identified in the *Lysobacter* genome sequences using AntiSmash and subsequent domain and prediction analysis. Per cluster, the top row describes the GeneID number, the middle row describes the domains identified by PFAM analysis and bottom row describes the amino acid predicted to be incorporated based on NRPS/PKS, NRPS predictor2 and phylogenetic analysis.

Table S14. pp 27. Genetic organization of flagellar apparatus and type IV pilus in *Lysobacter* genomes.

Table S15. pp. 29. Genetic organization of the General Secretory Pathway and twin-arginine translocation pathway in the *Lysobacter* genomes.

Table S4. Antimicrobial and extracellular enzyme activity of the *Lysobacter* strains.

Activity assays		*L. ant* ATCC 29479	*L. ant* 76	*L. cap* 55	*L. gum* 3.2.11	*L. enz* C3
Enzymatic activity						
Protease		+	+/-	+	-	+
Chitinase		+	+	+	+	+
Glucanase		+	+	+	+	+
Antagonistic activity	***Medium***					
<u>Fungi</u>						
Rhizoctonia solani AG2-2 IIIb	PDA	-	-	+	+	+
	1/5 PDA	+	-	+	+	+
	R2A	+	+/-	+	+	+
R. solani AG2-2 IIIb, 2 day head start	1/5 PDA	-	-	+/-	+	+/-
	R2A	+	+	+	+	+
R. solani	volatile	-	-	-	-	-
Verticillium dahliae JR2	PDA	+	+	+	+	+
	1/5 PDA	+	+	+	+	+
	R2A	+	+	+	+	+
Verticillium dahliae spores	PDA	-	-	+	+	+
	1/5 PDA	-	-	+	+	+
	R2A	+	+	+	+	+
Fusarium solani	PDA	NT	NT	+	+	+
	1/5 PDA	-	-	+	+	+
	R2A	-	-	+	+	+
Fusarium oxysporum (IRS) spores	PDA	-	-	+	+	+
	1/5 PDA	-	+	+	+	+
	R2A	+	+	+	+	+
Fusarium oxysporum (PRI) spores	PDA	+	-	+	+	+
	1/5 PDA	-	-	+	+	+
	R2A	+	+	+	+	+
Aspergillus niger spores	PDA	-	-	+/-	+/-	+/-
	1/5 PDA	-	+	+	+	+
	R2A	+	+	+	+	+
Cercospora spores	PDA	-	-/+	+	+	+
	1/5 PDA	-	-	+	+	+
	R2A	+	+	+	+	+
Stemphylium spores	PDA	-	-	+	+	+
	1/5 PDA	-/+	-/+	+	+	+
	R2A	+	+	+	+	+
<u>Oomycetes</u>						
Aphanomyces cochloides	PDA	+	+	+	+	+
	1/5 PDA	-	+	+	+	+
	R2A	+	+	+	+	+
Phytophthora infestans 88069	PDA	+	+	+	+	+
	1/5 PDA	-	+	+	+	+
	R2A	+	+	+	+	+
Pythium ultimum	PDA	-	-	+	+	+
	1/5 PDA	-	-/+	+	+	+
	R2A	+	+	+	+	+
Saprolegnia parasitica	PDA	-	+/-	+	+	-/+
	1/5 PDA	-/+	+	+	+	+
	R2A	-	+	+	+	+
<u>Bacteria</u>						
Xanthomonas campestris pv *campestris*	1/5 PDA	+	+	-	+	-
	R2A	-	+	-	-	-
Pectobacterium atrosepticum	1/5 PDA	-	-	-	-	-
	R2A	-	-	-	-	-
	LB	-	-	-	-	-

Note: + indicates inhibition of the plant pathogen was observed; - indicates no inhibition was observed. +/- indicates an inhibition was observed after 2-3 days of incubation, but the inhibition disappeared upon longer incubation. NT indicates not tested.
L. ant: *Lysobacter antibioticus*; L. cap: *L. capsic*i; L. gum: *L. gummosus*; L. enz: *L. enzymogenes*.

Table S5. Number of CDSs encoding peptidases containing a glycosyl hydrolase catalytic domain with chitinase activity in *Lysobacter*, *Stenotrophomonas* and *Xanthomonas* spp.

	Taxonomy ID	# GH18	# GH19	# GH23	# GH48
L. ant ATCC29479		2	3	6	0
L. ant 55		2	0	5	0
L. cap 76		2	0	6	0
L. gum 3.2.11		3	0	6	0
L. enz C3		2	2	6	0
S. mal D457	1163399	2	3	8	0
S. mal K279a	522373	2	2	7	0
S. mal R551-3	391008	2	1	7	0
X. alb GPE PC73	29447	2	0	9	0
X. axo_cit 306	190486	1	2	8	0
X. axo_cit F1	981368	1	1	5	0
X. axo Xac29-1	1304892	1	2	10	0
X. cam_cam B100	340	1	0	8	0
X. cam_cam 8004	314565	1	1	7	0
X. cam_cam ATCC33913	190485	1	0	6	0
X. cam_rap 756C	990315	1	0	7	0
X. cam_ves 85-10	316273	1	1	7	0
X. cit_cit Aw12879	1137651	1	4	8	0
X. fus_fus 4824R	366649	1	1	5	0
X. ory_ory KACC10331	291331	1	1	5	0
X. ory_ory MAFF311018	342109	1	1	5	0
X. ory_ory PXO99A	360094	1	2	5	0
X. ory_ory BLS256	383407	1	1	5	0

Note: Taxonomy ID refers to ID number in NCBI. The values for the *Stenotrophomonas* and *Xanthomonas* strains were obtained from CAZy database.

L. ant: *Lysobacter antibioticus*; L. cap: *L. capsici*; L. gum: *L. gummosus*; L. enz: *L. enzymogenes*. S. mal: *Stenotrophomonas maltophilia*; X. alb: *Xanthomonas albilineans*; X. axo_cit_306: X. axonoponis pv. citri str. 306; X. axo_cit F1: X. axonoponis pv. citrumelo F1; X. cam_cam: X. *campestris* pv. *campestris*; X.cam_rap: *X. campestris* pv. *raphani* X. cam_ves: *X. campestris* pv. *vesicatoria*; X. cit_cit Aw12879: *X. citri* subsp. *citri* Aw12879; X. fus_fus: *X. fuscans* subsp. *fuscans*; X. ory_ory: *X. oryzae* pv. *oryzae*; X. ory_ory BLS256: *X. oryzae* pv. *oryzicola* BLS256

Table S6. CDSs encoding peptidases containing a glycoside hydrolase domain with chitinase activity.

ID	PFAM domains		
L. ant ATCC29479			
LA29479_768	Glyco_hydro_18	CBM_5_12	Fn3-like
LA29479_4426	Glyco_hydro_18	CBM_5_12	Fn3-like
LA29479_108	Glyco_hydro_19		
LA29479_1555	Glyco_hydro_19		
LA29479_2721	Glyco_hydro_19	PG_binding_1	
LA29479_173	SLT	LysM	
LA29479_550	SLT		
LA29479_2732	SLT		
LA29479_3128	SLT		
LA29479_3846	SLT	LysM	
LA29479_3980	SLT_L	SLT	
L. ant 76			
LA76x_490	Glyco_hydro_18	CBM_5_12	fn3
LA76x_1507	Glyco_hydro_18	CBM_5_12	fn3
LA76x_1715	SLT		
LA76x_2402	SLT		
LA76x_3017	SLT	LysM	
LA76x_3546	SLT	LysM	
LA76x_4569	SLT_L	SLT	
L. cap 55			
LC55x_1252	Glyco_hydro_18	CBM_5_12	fn3
LC55x_4660	Glyco_hydro_18	CBM_5_12	fn3
LC55x_515	SLT_L	SLT	
LC55x_1606	SLT		
LC55x_2425	SLT		
LC55x_3046	SLT	LysM	
LC55x_3500	SLT	LysM	
LC55x_4497	SLT		
L. gum 3.2.11			
LG3211_1152	Glyco_hydro_18	CBM_5_12	fn3
LG3211_1156	Glyco_hydro_18	CBM_5_12	fn3
LG3211_4770	Glyco_hydro_18	CBM_5_12	fn3
LG3211_1691	GH23	SLT	LysM
LG3211_2174	GH23	SLT	LysM
LG3211_2812	GH23	SLT	
LG3211_3609	GH23	SLT	
LG3211_4306	GH23	SLT	
LG3211_510	GH23	SLT_L	SLT
L. enz C3			
LEC3_0401	Glyco_hydro_18	CBM_5_12	
LEC3_1126	Glyco_hydro_18	CBM_5_12	
LEC3_2053	Glyco_hydro_19		
LEC3_3325	Glyco_hydro_19		
LEC3_0326	SLT_L	SLT	
LEC3_1657	SLT	LysM	
LEC3_2132	SLT	LysM	Fn3-like
LEC3_2818	SLT		Fn3-like
LEC3_3603	SLT		
LEC3_4365	SLT		

Note: BLASTp analysis was performed with the GH domain of reference sequences using a cut-off of e-value < e-5. Subsequently, blast hits were subjected to PFAM analysis and only those that contained a significant hit for a GH domain were considered as a CDS containing a GH domain with chitinase activity.
L. ant: *Lysobacter antibioticus*; L. cap: *L. capsic*i; L. gum: *L. gummosus*; L. enz: *L. enzymogenes*.

Table S7. Number of CDSs encoding peptidases containing a glycosyl hydrolase catalytic domain with glucanase activity in *Lysobacter*, *Stenotrophomonas* and *Xanthomonas* spp.

	Taxonomy ID	GH1	GH3	GH5	GH6	GH9	GH12	GH16	GH30	GH51	GH71	GH74
L. ant 29479		1	2	0	0	0	0	2	0	0	1	0
L. ant 55		0	2	0	0	0	0	1	0	0	0	0
L. cap 76		0	3	0	0	0	0	3	0	0	0	0
L. gum 3.2.11		0	4	0	0	0	0	2	0	0	0	0
L. enz. C3		0	3	1	0	0	0	2	0	0	0	0
S. mal D457	1163399	0	5	0	0	0	0	0	0	0	0	0
S. mal K279a	522373	0	4	0	0	0	0	1	0	0	0	0
S. mal R551-3	391008	0	3	0	0	0	0	0	0	0	0	0
X. alb GPE PC73	29447	0	6	2	1	1	1	0	0	1	0	0
X. axo_cit 306	190486	0	6	6	0	1	1	1	3	1	0	1
X. axo_cit F1	981368	0	6	6	0	1	1	0	2	1	0	1
X. axo Xac29-1	1304892	0	6	6	0	1	0	1	1	1	0	1
X. cam_cam B100	340	0	8	6	2	1	2	1	2	1	0	1
X. cam_cam 8004	314565	0	8	6	2	1	2	1	2	1	0	1
X. cam_cam ATCC33913	190485	0	8	6	2	1	2	1	2	1	0	1
X. cam_rap 756C	990315	0	8	6	1	1	2	1	2	1	0	1
X. cam_ves 85-10	316273	0	6	6	0	1	1	0	2	1	0	1
X. cit_cit Aq12879	1137651	0	6	6	0	1	2	1	3	1	0	1
X. fus_fus 4824R	366649	0	6	6	0	1	1	0	1	1	0	1
X. ory_ory KACC10331	291331	0	6	5	1	0	2	0	1	1	0	0
X. ory_ory MAFF311018	342109	0	6	5	1	0	2	0	1	1	0	0
X. ory_ory PXO99A	360094	0	6	5	1	0	2	0	1	1	0	0
X. ory_ory BLS256	383407	0	6	7	3	0	0	0	2	1	0	0

Note: other domains with glucanase activity according to CAZy database include GH7, GH26, GH44, GH45, GH48, GH55, GH64, GH81, GH87, GH124, GH128, GH131 were not detected or not present in the strains listed above.

L. ant: *Lysobacter antibioticus*; L. cap: *L. capsici*; L. gum: *L. gummosus*; L. enz: *L. enzymogenes; S. mal: Stenotrophomonas maltophilia; X. alb: Xanthomonas albilineans; X. axo_cit_306: X. axonoponis pv. citri str. 306; X. axo_cit F1: X. axonoponis pv. citrumelo F1; X. cam_cam: X. campestris pv. campestris; X.cam_rap: X. campestris pv. raphani X. cam_ves: X. campestris pv. vesicatoria; X. cit_cit Aw12879: X. citri subsp. citri Aw12879; X. fus_fus: X. fuscans subsp. fuscans; X. ory_ory: X. oryzae pv. oryzae; X. ory_ory BLS256: X. oryzae pv. oryzicola BLS256.*

Table S8. CDSs encoding peptidases containing glycoside hydrolase domain with glucanase activity.

ID	PFAM domain		
L. ant ATCC29479			
LA29479_1193	Glyco_hydro_1		
LA29479_4718	Glyco_hydro_3		
LA29479_5063	Glyco_hydro_3	Glyco_hydro_3_C	
LA29479_1051	Glyco_hydro_16		
LA29479_2056	Glyco_hydro_16		
LA29479_2110	Glyco_hydro_71		
L. ant 76			
LA76x_3474	GH3	Glyco_hydro_3	
LA76x_4814	GH3	Glyco_hydro_3	Glyco_hydro_3_C
LA76x_2591	GH16	Glyco_hydro_16	
L. cap 55			
LC55x_216	Glyco_hydro_3	Glyco_hydro_3_C	
LC55x_3422	Glyco_hydro_3		
LC55x_4890	Glyco_hydro_3	Glyco_hydro_3_C	
LC55x_1557	Glyco_hydro_16	CBM_6	
LC55x_2740	Glyco_hydro_16		
LC55x_4859	Glyco_hydro_16		
L. gum 3.2.11			
LG3211_1762	Glyco_hydro_3		
LG3211_1840	Glyco_hydro_3	Glyco_hydro_3_C	
LG3211_203	Glyco_hydro_3	Glyco_hydro_3_C	
LG3211_4758	Glyco_hydro_3	Glyco_hydro_3_C	
LG3211_2492	Glyco_hydro_16		
LG3211_2614	Glyco_hydro_16		
L. enz C3			
LEC3_1718	Glyco_hydro_3		
LEC3_2523	Glyco_hydro_3	Glyco_hydro_3_C	Fn3-like
LEC3_5129	Glyco_hydro_3	Glyco_hydro_3_C	
LEC3_3663	Cellulase		
LEC3_0490	Glyco_hydro_16	CBM_6	
LEC3_2518	Glyco_hydro_16		

L. ant: *Lysobacter antibioticus*; L. cap: *L. capsic*i; L. gum: *L. gummosus*; L. enz: *L. enzymogenes*.

Table S9. Number of CDSs encoding peptidase identified by BLASTp analysis in the MEROPS database.

	L. ant ATCC29479	*L. ant* 76	*L. cap* 55	*L. gum* 3.2.11	*L. enz* C3
Total no. CDS	372	330	325	298	324
% per genome	7.2	6.4	5.7	5.6	5.8
Family					
Aspartic Peptidases					
A08	1	1	1	1	1
A24A	1	1	1	1	1
Cysteine Peptidases					
C01A	2	1	0	1	0
C02A	1	1	1	0	1
C11	1	1	0	1	0
C14B	3	0	0	0	0
C15	1	1	1	1	1
C19	0	1	0	0	0
C26	10	11	9	9	7
C39	3	3	3	3	1
C40	3	3	2	2	3
C44	5	5	4	6	7
C55	1	1	0	0	0
C56	8	8	7	3	2
C59	0	0	1	1	1
C80	1	2	0	0	0
C82	0	2	3	1	0
Inhibitors					
I29	0	0	0	1	0
I39	0	2	2	2	0
I51	1	1	1	1	1
I78	2	2	2	2	1
I87	0	3	3	3	0
Metallopeptidases					
M01	3	3	3	3	3
M02	2	2	2	2	2
M03A	1	1	1	1	1
M04	5	6	9	7	6
M06	1	0	0	0	0
M09A	2	2	1	1	1
M09B	0	11	8	5	0
M10A	0	0	1	1	0
M10B	0	1	1	0	0
M12A	2	1	0	0	0
M12B	1	1	0	0	0
M13	5	6	5	5	6
M14A	1	3	5	3	1
M14B	0	0	0	1	0
M14X	2	0	0	0	2
M15A	0	0	0	0	1
M15B	1	1	1	1	1
M15C	1	2	1	0	2
M15D	1	1	1	1	1

M16B	8	8	6	6	10
M17	2	2	2	2	2
M19	2	2	3	2	3
M20A	6	2	2	2	6
M20B	1	0	0	0	0
M20D	2	2	2	2	2
M20F	0	1	1	1	0
M20X	0	2	2	2	0
M22	4	0	0	0	4
M23A	1	1	1	1	1
M23B	16	16	18	17	12
M24A	2	2	2	2	2
M24B	3	3	3	3	3
M28A	1	1	1	0	0
M28B	1	0	0	0	1
M28D	0	1	1	1	0
M28E	4	4	5	5	6
M28X	0	0	1	0	1
M35	2	2	2	2	2
M36	0	0	0	1	0
M38	10	10	12	10	9
M41	1	1	1	1	1
M48A	0	1	0	0	0
M48B	1	1	1	1	1
M48C	0	3	4	3	0
M48X	4	0	0	0	4
M50B	1	1	3	2	2
M56	2	2	2	1	1
M61	1	1	1	1	1
M66	1	1	2	1	1
M72	4	3	3	4	4
M79	0	2	1	2	0
M90	0	1	1	1	0
M97	0	0	0	2	0
Asparagine peptide lyases					
N06	0	1	2	2	0
Serine Peptidases					
S01A	6	1	1	1	8
S01B	3	3	1	0	6
S01C	0	3	4	4	0
S01D	3	3	6	6	5
S01E	0	5	4	5	0
S01X	4	1	1	1	4
S08A	85	29	25	22	46
S08B	9	0	0	0	6
S09A	2	2	2	3	2
S09B	3	4	4	5	3
S09C	9	12	12	7	10
S09D	1	1	1	1	1
S09X	7	5	8	7	5
S11	2	2	2	2	2
S12	11	10	20	13	15

S14	1	1	1	1	1
S15	3	0	0	0	7
S16	4	2	2	2	3
S24	2	2	2	2	2
S26A	1	2	2	2	3
S28	0	0	1	0	0
S33	30	32	30	31	28
S41A	5	3	4	3	8
S45	3	4	1	2	1
S46	4	4	3	3	4
S49	3	0	0	0	2
S49A	0	1	1	1	0
S49B	0	2	1	1	0
S51	1	1	2	2	1
S53	1	0	1	0	0
S54	3	4	4	4	3
S73	0	1	0	1	0
Threonine Peptidases					
T01B	1	1	1	1	1
T02	3	2	2	2	3
T03	2	2	2	2	2
Peptidases of Unknown Catalytic Type					
U48	1	0	0	0	2
U62	5	6	5	5	5
U68	1	0	0	0	2
U69	1	7	0	2	0
U73	0	4	4	4	0

L. ant: *Lysobacter antibioticus*; L. cap: *L. capsic*i; L. gum: *L. gummosus*; L. enz: *L. enzymogenes*.

Table S10. Blastp analysis of the predicted protein sequences of the peptidase identified in *Lysobacter gummosus* DSMZ60980 (Gokcen et al, 2014).

			L. ant 29479		*L. ant* 76		*L. cap* 55		*L. gum* 3.2.11		*L. enz* C3	
query	Accession number	MEROPS family	subject	% id	subject	% id	subject	% id	subject	% id	subject	% id
Serine peptidases												
α-lytic protease 1	KF738067	S01E	LA29479_4711	79.0	LA76x_3467	79.2	LC55x_3409	78.5	LG3211_1771	99.7	LEC3_1730	85.7
α-lytic protease 2	KF738068	S01E							LG3211_1772	99.2	LEC3_1733	69.4
α-lytic protease 3	KF738069	S01E	LA29479_30	86.9	LA76x_3156	87.4	LC55x_3177	94.1	LG3211_2056	99.8	LEC3_1989	87.4
			LA29479_29	75.0	LA76x_3157	75.0			LG3211_2058	81.5	LEC3_1992	78.0
α-lytic protease 4	KF738070	S01E	LA29479_30	82.9	LA76x_3156	83.4	LC55x_3177	81.9	LG3211_2058	99.5	LEC3_1992	82.4
			LA29479_29	78.0	LA76x_3157	78.0			LG3211_2056	81.4	LEC3_1989	79.0
α-lytic protease 5	KF738071	S01E	LA29479_2300	67.8	LA76x_4363	67.8			LG3211_2833	100.0		
Lysyl peptidase 1	KF738073	S01D	LA29479_3571	78.1	LA76x_3979	78.1	LC55x_2732	88.3	LG3211_2609	98.2	LEC3_3841	76.5
			LA29479_3572	67.0	LA76x_3980	67.0	LC55x_4007	80.3	LG3211_3817	81.3	LEC3_3843	61.3
							LC55x_4008	61.1	LG3211_3819	66.7		
Lysyl peptidase 2	KF738074	S01D	LA29479_3571	85.4	LA76x_3979	85.4	LC55x_4007	88.5	LG3211_3817	100.0	LEC3_3841	85.7
			LA29479_413	76.6	LA76x_5040	77.1	LC55x_2732	83.3	LG3211_2609	82.4	LEC3_3839	78.8
			LA29479_3602	75.0	LA76x_4010	75.0	LC55x_4005	79.9	LG3211_3815	81.3	LEC3_2743	73.0
			LA29479_3570	73.9	LA76x_3978	73.9	LC55x_4003	77.0	LG3211_4445	79.2	LEC3_3845	71.5
			LA29479_3575	72.7	LA76x_3983	72.7	LC55x_4002	74.1	LG3211_3813	76.8	LEC3_3884	68.8
			LA29479_3568	71.3	LA76x_3976	71.3	LC55x_4031	73.3	LG3211_3868	72.5	LEC3_0220	68.5
			LA29479_3572	70.9	LA76x_3980	70.7	LC55x_4009	72.5	LG3211_3819	71.7	LEC3_3843	65.7
			LA29479_1174	70.3	LA76x_2473	70.3	LC55x_4066	71.3	LG3211_3821	71.0	LEC3_3837	65.6
			LA29479_3573	69.0	LA76x_3981	69.0	LC55x_2891	71.2	LG3211_2741	69.1	LEC3_3850	65.0
			LA29479_4489	67.1	LA76x_4670	68.2	LC55x_2887	69.9	LG3211_395	64.7	LEC3_3854	64.4
			LA29479_2359	64.2	LA76x_3326	64.2	LC55x_4008	69.1	LG3211_3832	61.2	LEC3_3852	62.4
			LA29479_3577	64.1	LA76x_3985	64.1	LC55x_4023	65.2				
			LA29479_3277	63.3	LA76x_516	63.1	LC55x_4025	64.7				
			LA29479_4409	61.2	LA76x_203	62.4	LC55x_4015	60.7				
					LA76x_507	61.2						
Lysyl peptidase 3	KF738075	S01D	LA29479_3572	85.1	LA76x_3980	84.9	LC55x_4005	79.4	LG3211_3819	99.7	LEC3_3845	74.2
			LA29479_3570	73.9	LA76x_3978	73.9	LC55x_4008	78.6	LG3211_4445	77.4	LEC3_3843	73.4
			LA29479_3571	70.8	LA76x_5040	71.2	LC55x_4003	77.9	LG3211_3815	75.9	LEC3_3841	70.8
			LA29479_413	70.8	LA76x_3979	70.8	LC55x_2887	75.0	LG3211_3813	74.1	LEC3_3837	68.8
			LA29479_3573	70.4	LA76x_3981	70.4	LC55x_4009	74.2	LG3211_3821	72.2	LEC3_3839	68.7
			LA29479_3575	70.1	LA76x_3983	70.1	LC55x_4007	71.5	LG3211_3817	71.4	LEC3_3850	68.3
			LA29479_2446	70.0	LA76x_3411	70.0	LC55x_4031	70.0	LG3211_2741	68.2	LEC3_0220	65.2

			LA29479_1174	68.5	LA76x_2473	68.5	LC55x_2891	69.4	LG3211_2609	67.2	LEC3_3854	65.2
			LA29479_4489	67.4	LA76x_3411	68.3	LC55x_4002	68.8	LG3211_3829	62.7	LEC3_2743	63.8
			LA29479_2359	67.0	LA76x_4670	67.4	LC55x_4025	68.6	LG3211_3868	60.4	LEC3_3852	63.7
			LA29479_3277	67.0	LA76x_3326	67.0	LC55x_4023	67.3				
			LA29479_2446	65.9	LA76x_203	66.1	LC55x_4021	66.4				
			LA29479_3568	65.6	LA76x_3976	65.6	LC55x_2732	66.3				
			LA29479_3602	64.4	LA76x_4010	64.4	LC55x_4015	61.6				
			LA29479_3577	61.2	LA76x_4670	62.4	LC55x_4066	61.4				
			LA29479_4489	61.2	LA76x_3985	61.2						
					LA76x_516	60.5						
Lysyl peptidase 4	KF738081	S01D	LA29479_3277	75.3	LA76x_203	75.3	LC55x_5147	93.4	LG3211_5119	99.5	LEC3_0165	87.0
Serine peptidase 1, family S8	KF738082	S08A	LA29479_5054	74.7	LA76x_4821	74.8	LC55x_211	81.9	LG3211_196	99.8	LEC3_5135	72.3
Serine peptidase 2, family S8	KF738083	S08A	LA29479_4	77.5	LA76x_3180	77.4	LC55x_3192	85.1	LG3211_2038	99.7	LEC3_1973	81.9
Metallopeptidases												
Hemagglutinin/ proteinase	KF738078	M04	LA29479_3568	91.8	LA76x_3976	91.8	LC55x_2891	91.5	LG3211_2741	100.0	LEC3_2743	86.7
			LA29479_1174	89.7	LA76x_2473	90.0	LC55x_4002	82.7	LG3211_3813	87.3	LEC3_3837	74.6
			LA29479_413	81.8	LA76x_5040	80.9	LC55x_4031	81.7	LG3211_4445	83.4	LEC3_3841	71.8
			LA29479_3570	75.5	LA76x_3980	76.4	LC55x_2887	76.4	LG3211_2739	75.9	LEC3_2741	71.3
			LA29479_3572	75.5	LA76x_2475	75.5	LC55x_2889	76.1	LG3211_3832	75.9	LEC3_3839	70.0
			LA29479_3575	75.5	LA76x_3978	75.5	LC55x_4007	74.6	LG3211_3815	72.7	LEC3_3845	66.1
			LA29479_1171	75.1	LA76x_3983	75.5	LC55x_4030	70.5	LG3211_3821	71.4	LEC3_3854	66.1
			LA29479_3571	74.6	LA76x_3979	74.6	LC55x_4003	69.1	LG3211_3817	69.1	LEC3_3852	65.2
			LA29479_3577	74.1	LA76x_3985	74.1	LC55x_4005	69.1	LG3211_4445	69.1	LEC3_3843	63.4
			LA29479_3573	69.6	LA76x_3981	69.6	LC55x_4008	69.1	LG3211_3819	68.2	LEC3_3850	60.7
			LA29479_2359	64.0	LA76x_3326	64.0	LC55x_4015	68.8	LG3211_3829	68.2		
			LA29479_4409	63.1	LA76x_507	63.1	LC55x_4009	67.9				
			LA29479_3277	61.3	LA76x_203	60.4	LC55x_4021	64.3				
							LC55x_4023	63.4				
							LC55x_4025	63.4				
							LC55x_4028	60.7				
β-lytic-metallo-endopeptidase	KF738072	M23A	LA29479_74	74.2	LA76x_3114	74.2	LC55x_945	82.5	LG3211_883	99.2	LEC3_0833	81.2
Metallopeptidase, family M72	KF738080	M72	LA29479_4589	87.0	LA76x_1409	86.7	LC55x_1143	89.4	LG3211_1059	99.8	LEC3_1028	79.7

Note: cut-off E-value for BLASTp analysis was set at 10^{-5}. L. ant: *Lysobacter antibioticus*; L. cap: *L. capsici*; L. gum: *L. gummosus*; L. enz: *L. enzymogenes*.

Table S11. Overview of the antimicrobial compounds produced by *Lysobacter*.

Species	Compound	Activity	Gene	References
L. antibioticus	1-hydroxy-6-methoxyphenazine	antioomycete		[1]
	1-Hydroxy-6-methoxyphenazine 5,10-dioxide (myxin)			[2]
	4-hydroxyphenylacetic acid	antioomycete		[3]
L. capsici	cyclo(L-Pro-L-Tyr	antioomycete		[4]
L. enzymogenes	dihydromaltophilin (HSAF)	antifungal/antioomycete	PKS/NRPS	[5,6,7]
	biosurfactant	antifungal/antioomycete		[8]
	cyclic lipodepsipeptide (WAP-8294A2)	antibacterial	*WAPS1,2*; NRPS	[9]
L. gummosus	2,4-diacetylphloroglucinol	antifungal		[10]
L. lactamgenus	cephabacin	unknown	*pcnABC*; PKS/NRPS	[11,12]
Lysobacter sp.	tripropeptin C (cyclic lipodepsipeptide)	antibacterial		[13,14]
	lysobactin (macrocyclic depsipeptide)	antibacterial	*lybAB*; NRPS	[15]
	xanthobaccin A, B & C	antibacterial		[16,17]
	endopeptidase L1,4,5	antibacterial - lysis	*alpAB*	[18,19,20]
	N-acetylmuramoyl-L-alanine amidase	antibacterial - lysis		[18,19,20]
	muramidase	antibacterial - lysis		[18,19,20]
Lysobacter spp.	β-1,3-glucanases	antifungal	*gluABC*	[21]
	chitinases	antifungal	*chiA*	[2,2,22]

1. Cook FD, Edwards OE, Gillespie DC, Peterson ER (1971) 1-Hydroxy-6-methoxy phenazines. United States Patent 3609153 pp. 9.

2. Christensen P, Cook FD (1978) *Lysobacter*, a new genus of nonfruiting, gliding bacteria with a high base ratio. International Journal of Systematic Bacteriology 28: 367-393.

3. Ko HS, Jin RD, Krishnan HB, Lee SB, Kim KY (2009) Biocontrol ability of *Lysobacter antibioticus* HS124 against *Phytophthora* blight is mediated by the production of 4-hydroxyphenylacetic acid and several lytic enzymes. Current Microbiology 59: 608-615.

4. Puopolo G, Cimmino A, Palmieri MC, Giovannini O, Evidente A, et al. (2014) *Lysobacter capsici* AZ78 produces cyclo(l-Pro-l-Tyr), a 2,5-diketopiperazine with toxic activity against sporangia of *Phytophthora infestans* and *Plasmopara viticola*. J Appl Microbiol 117: 1168-1180.

5. Li S, Calvo AM, Yuen GY, Du L, Harris SD (2009) Induction of cell wall thickening by the antifungal compound dihydromaltophilin disrupts fungal growth and is mediated by sphingolipid biosynthesis. J Eukaryot Microbiol 56: 182-187.

6. Lou L, Qian G, Xie Y, Hang J, Chen H, et al. (2011) Biosynthesis of HSAF, a tetramic acid-containing macrolactam from *Lysobacter enzymogenes*. J Am Chem Soc 133: 643-645.
7. Yu F, Zaleta-Rivera K, Zhu X, Huffman J, Millet JC, et al. (2007) Structure and biosynthesis of heat-stable antifungal factor (HSAF), a broad-spectrum antimycotic with a novel mode of action. Antimicrobial Agents and Chemotherapy 51: 64-72.
8. Folman LB (2003) Biological control of *Pythium aphanidermatum* in soilless systems: Selection of biocontrol agents and modes of action. PhD Thesis, University of Leiden, The Netherlands: 123-143.
9. Zhang W, Li Y, Qian G, Wang Y, Chen H, et al. (2011) Identification and characterization of the anti-methicillin-resistant Staphylococcus aureus WAP-8294A2 biosynthetic gene cluster from Lysobacter enzymogenes OH11. Antimicrob Agents Chemother 55: 5581-5589.
10. Brucker RM, Baylor CM, Walters RL, Lauer A, Harris RN, et al. (2008) The identification of 2,4-diacetylphloroglucinol as an antifungal metabolite produced by cutaneous bacteria of the salamander *Plethodon cinereus*. Journal of Chemical Ecology 34: 39-43.
11. Demirev AV, Lee CH, Jaishy BP, Nam DH, Ryu DDY (2006) Substrate specificity of nonribosomal peptide synthetase modules responsible for the biosynthesis of the oligopeptide moiety of cephabacin in *Lysobacter lactamgenus*. FEMS Microbiology Letters 255: 121-128.
12. Lee JS, Vladimirova MG, Demirev AV, Kim BG, Lim SK, et al. (2008) Expression and characterization of polyketide synthase module involved in the late step of cephabacin biosynthesis from *Lysobacter lactamgenus*. Journal of Microbiology and Biotechnology 18: 427-433.
13. Hashizume H, Igarashi M, Hattori S, Hori M, Hamada M, et al. (2001) Tripropeptins, novel antimicrobial agents produced by *Lysobacter* sp. I. Taxonomy, isolation and biological activities. Journal of Antibiotics 54: 1054-1059.
14. Hashizume H, Sawa R, Harada S, Igarashi M, Adachi H, et al. (2011) Tripropeptin C blocks the lipid cycle of cell wall biosynthesis by complex formation with undecaprenyl pyrophosphate. Antimicrob Agents Chemother 55: 3821-3828.
15. Hou J, Robbel L, Marahiel MA (2011) Identification and characterization of the lysobactin biosynthetic gene cluster reveals mechanistic insights into an unusual termination module architecture. Chem Biol 18: 655-664.
16. Hashidoko Y, Nakayama T, Homma Y, Tahara S (1999) Structure elucidation of xanthobaccin A, a new antibiotic produced from *Stenotrophomonas* sp. strain SB-K88. Tetrahedron Letters 40: 2957-2960.
17. Nakayama T, Homma Y, Hashidoko Y, Mizutani J, Tahara S (1999) Possible role of xanthobaccins produced by *Stenotrophomonas* sp. strain SB-K88 in suppression of sugar beet damping-off disease. Applied and Environmental Microbiology 65: 4334-4339.
18. Lapteva YS, Zolova OE, Shlyapnikov MG, Tsfasman IM, Muranova TA, et al. (2012) Cloning and expression analysis of genes encoding lytic endopeptidases L1 and L5 from Lysobacter sp. strain XL1. Appl Environ Microbiol 78: 7082-7089.
19. Tsfasman IM, Sitkin BV, Lysanskaya VY, Stepnaya OA, Kulaev IS (2007) Substrate specificity and some physicochemical properties of autolytic enzymes of the bacterium *Lysobacter* sp. XL 1. Biochemistry (Moscow) 72: 760-765.
20. Vasilyeva NV, Tsfasman IM, Suzina NE, Stepnaya OA, Kulaev IS (2008) Secretion of bacteriolytic endopeptidase L5 of *Lysobacter* sp. XL1 into the medium by means of outer membrane vesicles. FEBS Journal 275: 3827-3835.
21. Palumbo JD, Yuen GY, Jochum CC, Tatum K, Kobayashi DY (2005) Mutagenesis of β-1,3-glucanase genes in *Lysobacter enzymogenes* strain C3 results in reduced biological control activity toward *Bipolaris* leaf spot of tall fescue and *Pythium* damping-off of sugar beet. Phytopathology 95: 701-707.
22. Zhang Z, Yuen GY, Sarath G, Penheiter AR (2001) Chitinases from the plant disease biocontrol agent, *Stenotrophomonas maltophilia* C3. Phytopathology 91: 204-211.

Table S12. Overview of reference protein sequences used for blastp analysis in the *Lysobacter* genomes. '+' indicates a hit in the genome sequence with cut-off E value of e-05 and >60 % identity (>70% for NRPS). Not detected is indicated with '-'.

Compound	Derivative/alternate na	Protein name	*L. ant* ATCC29479	*L. ant* 76	*L. cap* 55	*L. gum* 3.2.11	*L. enz* C3	Reference sequence obtained from:	Accession number	Reference
Exoenzymes										
chitinase		ChiA	+	+	+	+	+	*Lysobacter enzymogenes* OH11	DQ888611	Qian et al, 2012
		ChiA	-	-	-	-	-	*Serratia marcescens* 2170	AB015996	Watanabe et al, 1997
		ChiB	-	-	-	-	-	*Serratia marcescens* 2170	BAA31568	Watanabe et al, 1997
		ChiC	-	-	-	-	-	*Serratia marcescens* 2170	-	Watanabe et al, 1997
		ChiA	+	+	+	+	+	*Stenotrophomonas maltophilia* 34S1	AAB70917	Kobayashi et al, 2002
		ChiA	-	-	-	-	-	*Bacillus circulans* WL-12	AAA81528	Watanabe et al, 1990
		ChiC	-	-	-	-	-	*Bacillus circulans* WL-12	BAA13974	Watanabe et al, 1990
		ChiD	-	-	-	-	-	*Bacillus circulans* WL-12	BAA34114	Watanabe et al, 1990
endopeptidase		AlpA	+	+	+	+	+	*Lysobacter* spp. XL1	ACZ72924	Lapteva et al, 2012
		AlpB	+	+	+	-	-	*Lysobacter* spp. XL1	ACZ72925	Lapteva et al, 2012
		GH16	+	+	+	+	-	*Lysobacter gummosus* UASM 402	AHE78434	Goekcen et al, 2014
		M04_1	+	+	+	+	+	*Lysobacter gummosus* UASM 402	AHE78433	Goekcen et al, 2014
		M23A	+	+	+	+	+	*Lysobacter gummosus* UASM 402	AHE78427	Goekcen et al, 2014
		M72	+	+	+	+	+	*Lysobacter gummosus* UASM 402	AHE78435	Goekcen et al, 2014
		OmpA-like_1	+	+	+	+	+	*Lysobacter gummosus* UASM 402	AHE78431	Goekcen et al, 2014
		OmpA-like_2	+	+	+	+	+	*Lysobacter gummosus* UASM 402	AHE78432	Goekcen et al, 2014
		S01D_1	+	+	+	+	+	*Lysobacter gummosus* UASM 402	AHE78428	Goekcen et al, 2014
		S01D_2	+	+	+	+	+	*Lysobacter gummosus* UASM 402	AHE78429	Goekcen et al, 2014
		S01D_3	+	+	+	+	+	*Lysobacter gummosus* UASM 402	AHE78430	Goekcen et al, 2014
		S01D_4	+	+	+	+	+	*Lysobacter gummosus* UASM 402	AHE78436	Goekcen et al, 2014
		S01E_1	+	+	+	+	+	*Lysobacter gummosus* UASM 402	AHE78422	Goekcen et al, 2014
		S01E_2	-	-	+	+	+	*Lysobacter gummosus* UASM 402	AHE78423	Goekcen et al, 2014
		S01E_3	+	+	+	+	+	*Lysobacter gummosus* UASM 402	AHE78424	Goekcen et al, 2014
		S01E_4	+	+	+	+	+	*Lysobacter gummosus* UASM 402	AHE78425	Goekcen et al, 2014
		S01E_5	+	+	-	+	-	*Lysobacter gummosus* UASM 402	AHE78426	Goekcen et al, 2014
		S08A_1	+	+	+	+	+	*Lysobacter gummosus* UASM 402	AHE78437	Goekcen et al, 2014
		S08A_2	+	+	+	+	+	*Lysobacter gummosus* UASM 402	AHE78438	Goekcen et al, 2014
exoprotease		AprA	-	-	-	-	-	*Pseudomonas protegens* Pf-5	CP000076 (PFL_3210)	Paulsen et al, 2005; Loper et al, 2012
glucanase		GluA	-	-	+	+	+	*Lysobacter enzymogenes* C3	AAN77503	Palumbo et al, 2003
		GluB	+	+	+	+	+	*Lysobacter enzymogenes* C3	AAN77504	Palumbo et al, 2003
		GluC	-	-	+	+	+	*Lysobacter enzymogenes* C3	AAN77505	Palumbo et al, 2003
pectate lyase		PFLU3229	-	-	-	-	-	*Pseudomonas fluorescens* SBW25	NC_012660 (PFLU3229)	Silby et al, 2009
Antibiotics										
cephabacin	cephalosporin	PcnAB	-	-	-	-	-	*Lysobacter lactamgenus* YK90	BAA08846	Kimura et al 1996
		PcnC	-	-	-	-	-	*Lysobacter lactamgenus* YK90	Q48739	Kimura et al 1996
		CpbI	-	-	-	-	-	*Lysobacter lactamgenus* IFO14288	ABB80392	Demirev et al 2006
		CpbK	-	-	-	-	-	*Lysobacter lactamgenus* IFO14288	ABB80393	Demirev et al 2006
DAPG		PhlA	-	-	-	-	-	*Pseudomonas fluorescens* Q2-87	AAB48109	Bangera and Thomashow 1999
		PhlB	-	-	-	-	-	*Pseudomonas fluorescens* Q2-87	AAB48107	Bangera and Thomashow 1999
		PhlC	-	-	-	-	-	*Pseudomonas fluorescens* Q2-87	AAB48108	Bangera and Thomashow 1999
		PhlD	-	-	-	-	-	*Pseudomonas fluorescens* Q2-87	AAB48106	Bangera and Thomashow 1999
		PhlE	-	-	-	-	-	*Pseudomonas fluorescens* Q2-87	AAB48105	Bangera and Thomashow 1999
		PhlF	-	-	-	-	-	*Pseudomonas fluorescens* Q2-87	AAB48110	Bangera and Thomashow 1999
HCN	hydrogen cyanide	HcnA	-	-	-	-	-	*Pseudomonas protegens* CHAO	CP000076 (PFL_2577)	Gross and Loper 2009; Paulsen et al, 2005; Loper et al, 2012
		HcnB	-	-	-	-	-	*Pseudomonas protegens* CHAO	CP000076 (PFL_2578)	Gross and Loper 2009; Paulsen et al, 2005; Loper et al, 2012
		HcnC	-	-	-	-	-	*Pseudomonas protegens* CHAO	CP000076 (PFL_2579)	Gross and Loper 2009; Paulsen et al, 2005; Loper et al, 2012
HRP	dialkylrecorsinol	DarA	-	-	-	-	-	*Pseudomonas chlororaphis* subsp. *aureofaciens* 30-84	EJL05820	Loper et al, 2012
		DarB	-	-	-	-	-	*Pseudomonas chlororaphis* subsp. *aureofaciens* 30-84	EJL05977	Loper et al, 2012
		DarC	-	-	-	-	-	*Pseudomonas chlororaphis* subsp. *aureofaciens* 30-84	EJL06701	Loper et al, 2012
		DarR	-	-	-	-	-	*Pseudomonas chlororaphis* subsp. *aureofaciens* 30-84	EJL07355	Loper et al, 2012
		DarS	-	-	-	-	-	*Pseudomonas chlororaphis* subsp. *aureofaciens* 30-84	EJL07090	Loper et al, 2012
Phenazine		PhzA1	+	+	-	-	-	*Pseudomonas aeruginosa* PA01	NC_002516 (PA4210)	Winsor et al, 2009
		PhzB1	+	+	-	-	-	*Pseudomonas aeruginosa* PA01	NC_002516 (PA4211)	Winsor et al, 2009
		PhzC1	-	-	-	-	-	*Pseudomonas aeruginosa* PA01	NC_002516 (PA4212)	Winsor et al, 2009
		PhzD1	+	+	-	-	-	*Pseudomonas aeruginosa* PA01	NC_002516 (PA4213)	Winsor et al, 2009
		PhzE1	-	-	-	-	-	*Pseudomonas aeruginosa* PA01	NC_002516 (PA4214)	Winsor et al, 2009
		PhzF1	+	+	-	-	-	*Pseudomonas aeruginosa* PA01	NC_002516 (PA4215)	Winsor et al, 2009
		PhzG1	-	-	-	-	-	*Pseudomonas aeruginosa* PA01	NC_002516 (PA4216)	Winsor et al, 2009
		PhzH	-	-	-	-	-	*Pseudomonas aeruginosa* PA01	NC_002516 (PA0051)	Winsor et al, 2009
		PhzM	-	-	-	-	-	*Pseudomonas aeruginosa* PA01	NC_002516 (PA4209)	Winsor et al, 2009
		PhzS	+	+	-	-	-	*Pseudomonas aeruginosa* PA01	NC_002516 (PA4217)	Winsor et al, 2009
		MexG	-	-	-	-	-	*Pseudomonas aeruginosa* PA01	NC_002516 (PA4205)	Winsor et al, 2009
		MexH	-	-	-	-	-	*Pseudomonas aeruginosa* PA01	NC_002516 (PA4206)	Winsor et al, 2009
		MexI	-	-	-	-	-	*Pseudomonas aeruginosa* PA01	NC_002516 (PA4207)	Winsor et al, 2009
		OmpD	-	-	-	-	-	*Pseudomonas aeruginosa* PA01	NC_002516 (PA4208)	Winsor et al, 2009
		PhzA2	+	+	-	-	-	*Pseudomonas aeruginosa* PA01	NC_002516 (PA1899)	Winsor et al, 2009
		PhzB2	+	+	-	-	-	*Pseudomonas aeruginosa* PA01	NC_002516 (PA1900)	Winsor et al, 2009
		PhzC2	-	-	-	-	-	*Pseudomonas aeruginosa* PA01	NC_002516 (PA1901)	Winsor et al, 2009
		PhzD2	+	+	-	-	-	*Pseudomonas aeruginosa* PA01	NC_002516 (PA1902)	Winsor et al, 2009
		PhzE2	-	-	-	-	-	*Pseudomonas aeruginosa* PA01	NC_002516 (PA1903)	Winsor et al, 2009
		PhzF2	+	+	-	-	-	*Pseudomonas aeruginosa* PA01	NC_002516 (PA1904)	Winsor et al, 2009

		PhzG2	-	-	-	-	-	*Pseudomonas aeruginosa* PA01	NC_002516 (PA1905)	Winsor et al, 2009
pyoluteorin		PltA	-	-	-	-	-	*Pseudomonas protegens* Pf-5	CP000076 (PFL_2787)	Gross and Loper 2009; Paulsen et al, 2005
		PltB	-	-	-	-	-	*Pseudomonas protegens* Pf-5	CP000076 (PFL_2788)	Gross and Loper 2009; Paulsen et al, 2005
		PltC	-	-	-	-	-	*Pseudomonas protegens* Pf-5	CP000076 (PFL_2789)	Gross and Loper 2009; Paulsen et al, 2005
		PltD	-	-	-	-	-	*Pseudomonas protegens* Pf-5	CP000076 (PFL_2790)	Gross and Loper 2009; Paulsen et al, 2005
		PltE	-	-	-	-	-	*Pseudomonas protegens* Pf-5	CP000076 (PFL_2791)	Gross and Loper 2009; Paulsen et al, 2005
		PltF	-	-	-	-	-	*Pseudomonas protegens* Pf-5	CP000076 (PFL_2792)	Gross and Loper 2009; Paulsen et al, 2005
		PltG	-	-	-	-	-	*Pseudomonas protegens* Pf-5	CP000076 (PFL_2793)	Gross and Loper 2009; Paulsen et al, 2005
		PltM	-	-	-	-	-	*Pseudomonas protegens* Pf-5	CP000076 (PFL_2784)	Gross and Loper 2009; Paulsen et al, 2005
		PltR	-	-	-	-	-	*Pseudomonas protegens* Pf-5	CP000076 (PFL_2785)	Gross and Loper 2009; Paulsen et al, 2005
pyrrolnitrin		PrnA	-	-	-	-	-	*Pseudomonas protegens* Pf-5	CP000076 (PFL_3604)	Gross and Loper 2009; Paulsen et al, 2005
		PrnB	-	-	-	-	-	*Pseudomonas protegens* Pf-5	CP000076 (PFL_3605)	Gross and Loper 2009; Paulsen et al, 2005
		PrnC	-	-	-	-	-	*Pseudomonas protegens* Pf-5	CP000076 (PFL_3606)	Gross and Loper 2009; Paulsen et al, 2005
		PrnD	-	-	-	-	-	*Pseudomonas protegens* Pf-5	CP000076 (PFL_3607)	Gross and Loper 2009; Paulsen et al, 2005
rhizoxin		RzxB	-	-	-	-	-	*Pseudomonas protegens* Pf-5	CP000076 (PFL_2989)	Loper et al, 2008; Loper et al, 2012
		RzxC	-	-	-	-	-	*Pseudomonas protegens* Pf-5	CP000076 (PFL_2990)	Loper et al, 2008; Loper et al, 2012
		RzxD	-	-	-	-	-	*Pseudomonas protegens* Pf-5	CP000076 (PFL_2991)	Loper et al, 2008; Loper et al, 2012
		RzxH	-	-	-	-	-	*Pseudomonas protegens* Pf-5	CP000076 (PFL_2992)	Loper et al, 2008; Loper et al, 2012
		RzxE	-	-	-	-	-	*Pseudomonas protegens* Pf-5	CP000076 (PFL_2993)	Loper et al, 2008; Loper et al, 2012
		RzxF	-	-	-	-	-	*Pseudomonas protegens* Pf-5	CP000076 (PFL_2994)	Loper et al, 2008; Loper et al, 2012
		RzxI	-	-	-	-	-	*Pseudomonas protegens* Pf-5	CP000076 (PFL_2995)	Loper et al, 2008; Loper et al, 2012
		RzxG	-	-	-	-	-	*Pseudomonas protegens* Pf-5	CP000076 (PFL_2996)	Loper et al, 2008; Loper et al, 2012
		RzxA	-	-	-	-	-	*Pseudomonas protegens* Pf-5	CP000076 (PFL_2997)	Loper et al, 2008; Loper et al, 2012
cyclo(L-Pro-L-Tyr)	2,5-diketopiperazine	AZ78_PKS1	-	-	+	+	+	*Lysobacter capsici* AZ78	AHG93908	Puopolo et al, 2014
NRPS/PKS clusters										
HSAF	dihydromaltophillin	HSAF - NRPS	-	-	+	+	+	*Lysobacter enzymogenes* C3	ABL86391	Lou et al, 2011
lysobactin		LybA	-	-	-	+	-	*Lysobacter* sp. ATCC 53042	AEH59099	Hou et al, 2011
		LybB	-	-	-	+	-	*Lysobacter* sp. ATCC 53042	AEH59100	Hou et al, 2011
WAPS	WAP8294A2	WAPS1	-	-	+	-	+	*Lysobacter enzymogenes* OH11	AEP18656	Zhang et al 2011
		WAPS2	-	-	+	-	+	*Lysobacter enzymogenes* OH11	AEP18655	Zhang et al 2011
tripropeptin	cyclic depsipeptide	TrpA	-	-	-	-	-	*Collimonas fungivorans* Ter331	NIOO	
		TrpB	-	-	-	-	-	*Collimonas fungivorans* Ter331	YP_004752836	Mela et al, 2011
		TrpC	-	-	-	-	-	*Collimonas fungivorans* Ter331	YP_004752837	Mela et al, 2011
arthrofactin	cyclic lipopeptide	ArfA	-	-	-	-	-	*Pseudomonas spp.* MIS38	BAC67534	Roongsawang et al, 2003
		ArfB	-	-	-	-	-	*Pseudomonas spp.* MIS38	BAC67535	Roongsawang et al, 2003
		ArfC	-	-	-	-	-	*Pseudomonas spp.* MIS38	BAC67536	Roongsawang et al, 2003
brabantamide	dipeptide		-	-	-	-	-	*Pseudomonas chlororaphis* CH-C52		Gross?
collimomycin		cfu_1083	-	-	-	-	-	*Collimonas fungivorans* Ter331	NIOO	
		cfu_1084	-	-	-	-	-	*Collimonas fungivorans* Ter331	NIOO	
		cfu_1085	-	-	-	-	-	*Collimonas fungivorans* Ter331	NIOO	
gramicidin	cyclic peptide	LgrA	-	-	-	-	-	*Bacillus brevis* ATCC 8185	CAD92849	Kessler et al, 2004
		LgrB	-	-	-	-	-	*Bacillus brevis* ATCC 8185	CAD92850	Kessler et al, 2004
		LgrC	-	-	-	-	-	*Bacillus brevis* ATCC 8185	CAD92851	Kessler et al, 2004
		LgrD	-	-	-	-	-	*Bacillus brevis* ATCC 8185	CAD92852	Kessler et al, 2004
entolysin	cyclic lipopeptide	EtlA	-	-	-	-	-	*Pseudomonas enthomophila* L48	NC_008027 (PSEEN3332)	Vodovar et al, 2006; Vallet-Gely et al, 2010
		EtlB	-	-	-	-	-	*Pseudomonas enthomophila* L48	NC_008027 (PSEEN3045)	Vodovar et al, 2006; Vallet-Gely et al, 2010
		EtlC	-	-	-	-	-	*Pseudomonas enthomophila* L48	NC_008027 (PSEEN3044)	Vodovar et al, 2006; Vallet-Gely et al, 2010
massetolide	cyclic lipopeptide	MassA	-	-	-	-	-	*Pseudomonas fluoresens* SS101	ABH06367	De Bruijn et al, 2007; Loper et al, 2012
		MassB	-	-	-	-	-	*Pseudomonas fluoresens* SS101	ABH06368	De Bruijn et al, 2007; Loper et al, 2012
		MassC	-	-	-	-	-	*Pseudomonas fluoresens* SS101	ABH06369	De Bruijn et al, 2007; Loper et al, 2012
orfamide	cyclic lipopeptide	OfaA	-	-	-	-	-	*Pseudomonas protegens* Pf-5	CP000076 (PFL_2145)	Paulsen et al, 2005; Gross et al, 2007
		OfaB	-	-	-	-	-	*Pseudomonas protegens* Pf-5	CP000076 (PFL_2146)	Paulsen et al, 2005; Gross et al, 2007
		OfaC	-	-	-	-	-	*Pseudomonas protegens* Pf-5	CP000076 (PFL_2147)	Paulsen et al, 2005; Gross et al, 2007
putisolvin	cyclic lipopeptide	PsoA	-	-	-	-	-	*Pseudomonas putida* PCL1445	DQ151887	Dubern et al, 2008
		PsoB	-	-	-	-	-	*Pseudomonas putida* PCL1445	DQ151887	Dubern et al, 2008
		PsoC	-	-	-	-	-	*Pseudomonas putida* PCL1445	DQ151887	Dubern et al, 2008
syringopeptin	cyclic lipopeptide	SypA	-	-	-	-	-	*Pseudomonas syringae* pv *syringae* B301D	AAF99707	Scholz-Schreuder et al, 2001
		SypB	-	-	-	-	-	*Pseudomonas syringae* pv *syringae* B301D	AAO72424	Scholz-Schreuder et al, 2001
		SypC	-	-	-	-	-	*Pseudomonas syringae* pv *syringae* B301D	AAO72425	Scholz-Schreuder et al, 2001
syringomycin	cyclic lipopeptide	SyrE	-	-	-	-	-	*Pseudomonas syringae* pv *syringae* B301D	AF047828	Guenzi et al, 1998
		SyrB1	-	-	-	-	-	*Pseudomonas syringae* pv *syringae* B301D	AAA85160	Guenzi et al, 1998
syringafactin	cyclic lipopeptide	SyfA	-	-	-	-	-	*Pseudomonas syringae* pv *tomato* DC3000	NC_004578 (PSPTO_2829)	Buell et al, 2003; Berti et al, 2007
		SyfB	-	-	-	-	-	*Pseudomonas syringae* pv *tomato* DC3000	NC_004578 (PSPTO_2830)	Buell et al, 2003; Berti et al, 2007
thanamycin/cormycin	cyclic lipopeptide	ThaA	-	-	-	-	-	*Pseudomonas chlororaphis* CH-C52		Mendes et al, 2011; Watrous et al, 2012
		ThaB	-	-	-	-	-	*Pseudomonas chlororaphis* CH-C52		Mendes et al, 2011; Watrous et al, 2012
thanapeptin/corpeptin	cyclic lipopeptide	Peptin1	-	-	-	-	-	*Pseudomonas chlororaphis* CH-C52		?
		Peptin2	-	-	-	-	-	*Pseudomonas chlororaphis* CH-C52		?
		Peptin3	-	-	-	-	-	*Pseudomonas chlororaphis* CH-C52		?
viscosin	cyclic lipopeptide	ViscA	-	-	-	-	-	*Pseudomonas fluorescens* SBW25	NC_012660 (PFLU4007)	Silby et al, 2009; De Bruijn et al, 2007
		ViscB	-	-	-	-	-	*Pseudomonas fluorescens* SBW25	NC_012660 (PFLU2552)	Silby et al, 2009; De Bruijn et al, 2007
		ViscC	-	-	-	-	-	*Pseudomonas fluorescens* SBW25	NC_012660 (PFLU2553)	Silby et al, 2009; De Bruijn et al, 2007
unknown	(cyclic) lipopeptide	Pfl01_2211	-	-	-	-	-	*Pseudomonas fluorescens* Pf0-1	NC_007492 (Pfl01_2211)	Silby et al, 2009; De Bruijn et al, 2007
		Pfl01_2212	-	-	-	-	-	*Pseudomonas fluorescens* Pf0-1	NC_007492 (Pfl01_2212)	Silby et al, 2009; De Bruijn et al, 2007
		Pfl01_2213	-	-	-	-	-	*Pseudomonas fluorescens* Pf0-1	NC_007492 (Pfl01_2213)	Silby et al, 2009; De Bruijn et al, 2007

Siderophores									
achromobactin	AcsA	-	-	-	-	-	*Pseudomonas syringae* pv *syringae* B7281		Berti et al, 2009
	AcsA	-	-	-	-	-	*Erwinia chrysanthemi* 3937	AF416739	Franza et al, 2005
	AcsB	-	-	-	-	-	*Erwinia chrysanthemi* 3937	AF416739	Franza et al, 2005
	AcsC	-	-	-	-	-	*Erwinia chrysanthemi* 3937	AF416739	Franza et al, 2005
	AcsD	-	-	-	-	-	*Erwinia chrysanthemi* 3937	AF416739	Franza et al, 2005
aerobactin	IucA	-	-	-	-	-	*Yersinia pseudotuberculosis* IP 31758	YP_001399703	Eppinger et al, 2007
	IucB	-	-	-	-	-	*Yersinia pseudotuberculosis* IP 31758	YP_001399702	Eppinger et al, 2007
	IucC	-	-	-	-	-	*Yersinia pseudotuberculosis* IP 31758	YP_001399701	Eppinger et al, 2007
	IucD	-	-	-	-	-	*Yersinia pseudotuberculosis* IP 31758	YP_001399700	Eppinger et al, 2007
enterobactin	EntA	-	-	-	-	-	*Escherichi coli* O104:H4 str. C227-11	AFST01000002.2	Rasko et al, 2011
	EntB	-	-	-	-	-	*Escherichi coli* O104:H4 str. C227-11	AFST01000002.2	Rasko et al, 2011
	EntC	-	-	-	-	-	*Escherichi coli* O104:H4 str. C227-11	AFST01000002.2	Rasko et al, 2011
	EntD	-	-	-	-	-	*Escherichi coli* O104:H4 str. C227-11	AFST01000002.2	Rasko et al, 2011
	EntE	-	-	-	-	-	*Escherichi coli* O104:H4 str. C227-11	AFST01000002.2	Rasko et al, 2011
	EntF	-	-	-	-	-	*Escherichi coli* O104:H4 str. C227-11	AFST01000002.2	Rasko et al, 2011
ornibactin	OrbS	-	-	-	-	-	*Burkholderia cenocepacia* strain 715j	DQ279459	Agnoli et al, 2006
	OrbB	-	-	-	-	-	*Burkholderia cenocepacia* strain 715j	DQ279460	Agnoli et al, 2006
	OrbE	-	-	-	-	-	*Burkholderia cenocepacia* strain 715j	DQ279460	Agnoli et al, 2006
	OrbI	-	-	-	-	-	*Burkholderia cenocepacia* strain 715j	DQ279460	Agnoli et al, 2006
	OrbS	-	-	-	-	-	Burkholderia cenocepacia J2315	AM747720 (BCAL1688)	Holden et al, 2009
	OrbH	-	-	-	-	-	Burkholderia cenocepacia J2315	AM747720 (BCAL1689)	Holden et al, 2009
	OrbG	-	-	-	-	-	Burkholderia cenocepacia J2315	AM747720 (BCAL1690)	Holden et al, 2009
	OrbC	-	-	-	-	-	Burkholderia cenocepacia J2315	AM747720 (BCAL1691)	Holden et al, 2009
	OrbD	-	-	-	-	-	Burkholderia cenocepacia J2315	AM747720 (BCAL1692)	Holden et al, 2009
	OrbF2	-	-	-	-	-	Burkholderia cenocepacia J2315	AM747720 (BCAL1693)	Holden et al, 2009
	OrbB	-	-	-	-	-	Burkholderia cenocepacia J2315	AM747720 (BCAL1694)	Holden et al, 2009
	OrbE	-	-	-	-	-	Burkholderia cenocepacia J2315	AM747720 (BCAL1695)	Holden et al, 2009
	OrbI	-	-	-	-	-	Burkholderia cenocepacia J2315	AM747720 (BCAL1696)	Holden et al, 2009
	OrbJ	-	-	-	-	-	Burkholderia cenocepacia J2315	AM747720 (BCAL1697)	Holden et al, 2009
	OrbK	-	-	-	-	-	Burkholderia cenocepacia J2315	AM747720 (BCAL1698)	Holden et al, 2009
	PvdA	-	-	-	-	-	Burkholderia cenocepacia J2315	AM747720 (BCAL1699)	Holden et al, 2009
	OrbA	-	-	-	-	-	Burkholderia cenocepacia J2315	AM747720 (BCAL1700)	Holden et al, 2009
	OrbF	-	-	-	-	-	Burkholderia cenocepacia J2315	AM747720 (BCAL1701)	Holden et al, 2009
	OrbL	-	-	-	-	-	Burkholderia cenocepacia J2315	AM747720 (BCAL1702)	Holden et al, 2009
pseudomonin	PmsC	-	-	-	-	-	*Pseudomonas fluorescens* WCS374	CAA70528	Mercado-Blanco et al, 2001
	PmsE	-	-	-	-	-	*Pseudomonas fluorescens* WCS374	CAA70529	Mercado-Blanco et al, 2001
	PmsA	-	-	-	-	-	*Pseudomonas fluorescens* WCS374	CAA70530	Mercado-Blanco et al, 2001
	PmsB	-	-	-	-	-	*Pseudomonas fluorescens* WCS374	CAA70531	Mercado-Blanco et al, 2001
	PmsG/BasB	-	-	-	-	-	*Pseudomonas enthomophila* L48	NC_008027 (PSEEN2500)	Vodovar et al, 2006; Gross and Loper 2009; Loper et al, 2012
	PmsF/BasC	-	-	-	-	-	*Pseudomonas enthomophila* L48	NC_008027 (PSEEN2502)	Vodovar et al, 2006; Gross and Loper 2009; Loper et al, 2012
	PmsD/BasA/D	-	-	-	-	-	*Pseudomonas enthomophila* L48	NC_008027 (PSEEN2503)	Vodovar et al, 2006; Gross and Loper 2009; Loper et al, 2012
	PmsC	-	-	-	-	-	*Pseudomonas enthomophila* L48	NC_008027 (PSEEN2504)	Vodovar et al, 2006; Gross and Loper 2009; Loper et al, 2012
	PmsE	-	-	-	-	-	*Pseudomonas enthomophila* L48	NC_008027 (PSEEN2505)	Vodovar et al, 2006; Gross and Loper 2009; Loper et al, 2012
	PmsA	-	-	-	-	-	*Pseudomonas enthomophila* L48	NC_008027 (PSEEN2506)	Vodovar et al, 2006; Gross and Loper 2009; Loper et al, 2012
	PmsB	-	-	-	-	-	*Pseudomonas enthomophila* L48	NC_008027 (PSEEN2507)	Vodovar et al, 2006; Gross and Loper 2009; Loper et al, 2012
pyochelin	PchF	-	-	-	-	-	*Pseudomonas aeruginosa* PA01	NC_002516 (PA4225)	Winsor et al, 2009; Loper et al, 2012
	PchG	-	-	-	-	-	*Pseudomonas aeruginosa* PA01	NC_002516 (PA4224)	Winsor et al, 2009; Loper et al, 2012
	PchE	-	-	-	-	-	*Pseudomonas aeruginosa* PA01	NC_002516 (PA4226)	Winsor et al, 2009; Loper et al, 2012
	PchR	-	-	-	-	-	*Pseudomonas aeruginosa* PA01	NC_002516 (PA4227)	Winsor et al, 2009; Loper et al, 2012
	PchD	-	-	-	-	-	*Pseudomonas aeruginosa* PA01	NC_002516 (PA4228)	Winsor et al, 2009; Loper et al, 2012
	PchC	-	-	-	-	-	*Pseudomonas aeruginosa* PA01	NC_002516 (PA4229)	Winsor et al, 2009; Loper et al, 2012
	PchB	-	-	-	-	-	*Pseudomonas aeruginosa* PA01	NC_002516 (PA4230)	Winsor et al, 2009; Loper et al, 2012
	PchA	-	-	-	-	-	*Pseudomonas aeruginosa* PA01	NC_002516 (PA4231)	Winsor et al, 2009; Loper et al, 2012
pyoverdine	PvdQ	-	-	-	-	-	*Pseudomonas aeruginosa* PA01	NC_002516 (PA2385)	Winsor et al, 2009; Gross and Loper 2009; Loper et al, 2012
	PvdA	-	-	-	-	-	*Pseudomonas aeruginosa* PA01	NC_002516 (PA2386)	Winsor et al, 2009; Gross and Loper 2009; Loper et al, 2012
	FpvI	-	-	-	-	-	*Pseudomonas aeruginosa* PA01	NC_002516 (PA2387)	Winsor et al, 2009; Gross and Loper 2009; Loper et al, 2012
	FpvR	-	-	-	-	-	*Pseudomonas aeruginosa* PA01	NC_002516 (PA2388)	Winsor et al, 2009; Gross and Loper 2009; Loper et al, 2012
	PvdR	-	-	-	-	-	*Pseudomonas aeruginosa* PA01	NC_002516 (PA2389)	Winsor et al, 2009; Gross and Loper 2009; Loper et al, 2012
	PvdT	-	-	-	-	-	*Pseudomonas aeruginosa* PA01	NC_002516 (PA2390)	Winsor et al, 2009; Gross and Loper 2009; Loper et al, 2012
	PA2391	-	-	-	-	-	*Pseudomonas aeruginosa* PA01	NC_002516 (PA2391)	Winsor et al, 2009; Gross and Loper 2009; Loper et al, 2012
	PvdP	-	-	-	-	-	*Pseudomonas aeruginosa* PA01	NC_002516 (PA2392)	Winsor et al, 2009; Gross and Loper 2009; Loper et al, 2012
	PvdM	-	-	-	-	-	*Pseudomonas aeruginosa* PA01	NC_002516 (PA2393)	Winsor et al, 2009; Gross and Loper 2009; Loper et al, 2012
	PvdN	-	-	-	-	-	*Pseudomonas aeruginosa* PA01	NC_002516 (PA2394)	Winsor et al, 2009; Gross and Loper 2009; Loper et al, 2012
	PvdO	-	-	-	-	-	*Pseudomonas aeruginosa* PA01	NC_002516 (PA2395)	Winsor et al, 2009; Gross and Loper 2009; Loper et al, 2012
	PvdF	-	-	-	-	-	*Pseudomonas aeruginosa* PA01	NC_002516 (PA2396)	Winsor et al, 2009; Gross and Loper 2009; Loper et al, 2012
	PvdE	-	-	-	-	-	*Pseudomonas aeruginosa* PA01	NC_002516 (PA2397)	Winsor et al, 2009; Gross and Loper 2009; Loper et al, 2012
	PA2398	-	-	-	-	-	*Pseudomonas aeruginosa* PA01	NC_002516 (PA2398)	Winsor et al, 2009; Gross and Loper 2009; Loper et al, 2012
	PvdD	-	-	-	-	-	*Pseudomonas aeruginosa* PA01	NC_002516 (PA2399)	Winsor et al, 2009; Gross and Loper 2009; Loper et al, 2012
	PvdJ	-	-	-	-	-	*Pseudomonas aeruginosa* PA01	NC_002516 (PA2400)	Winsor et al, 2009; Gross and Loper 2009; Loper et al, 2012
	PA2402	-	-	-	-	-	*Pseudomonas aeruginosa* PA01	NC_002516 (PA2402)	Winsor et al, 2009; Gross and Loper 2009; Loper et al, 2012
	PA2403	-	-	-	-	-	*Pseudomonas aeruginosa* PA01	NC_002516 (PA2403)	Winsor et al, 2009; Gross and Loper 2009; Loper et al, 2012
	PA2404	-	-	-	-	-	*Pseudomonas aeruginosa* PA01	NC_002516 (PA2404)	Winsor et al, 2009; Gross and Loper 2009; Loper et al, 2012
	PA2405	-	-	-	-	-	*Pseudomonas aeruginosa* PA01	NC_002516 (PA2405)	Winsor et al, 2009; Gross and Loper 2009; Loper et al, 2012
	PA2406	-	-	-	-	-	*Pseudomonas aeruginosa* PA01	NC_002516 (PA2406)	Winsor et al, 2009; Gross and Loper 2009; Loper et al, 2012
	PA2407	-	-	-	-	-	*Pseudomonas aeruginosa* PA01	NC_002516 (PA2407)	Winsor et al, 2009; Gross and Loper 2009; Loper et al, 2012

		PA2408	-	-	+	-	-	*Pseudomonas aeruginosa* PA01	NC_002516 (PA2408)	Winsor et al, 2009; Gross and Loper 2009; Loper et al, 2012
		PA2409	-	-	-	-	-	*Pseudomonas aeruginosa* PA01	NC_002516 (PA2409)	Winsor et al, 2009; Gross and Loper 2009; Loper et al, 2012
		PA2410	-	-	-	-	-	*Pseudomonas aeruginosa* PA01	NC_002516 (PA2410)	Winsor et al, 2009; Gross and Loper 2009; Loper et al, 2012
		PA2411	-	-	-	-	-	*Pseudomonas aeruginosa* PA01	NC_002516 (PA2411)	Winsor et al, 2009; Gross and Loper 2009; Loper et al, 2012
		PA2412	-	-	-	-	-	*Pseudomonas aeruginosa* PA01	NC_002516 (PA2412)	Winsor et al, 2009; Gross and Loper 2009; Loper et al, 2012
		PvdH	-	-	-	-	-	*Pseudomonas aeruginosa* PA01	NC_002516 (PA2413)	Winsor et al, 2009; Gross and Loper 2009; Loper et al, 2012
		PvdL	-	-	-	-	-	*Pseudomonas aeruginosa* PA01	NC_002516 (PA2424)	Winsor et al, 2009; Gross and Loper 2009; Loper et al, 2012
		PvdG	-	-	-	-	-	*Pseudomonas aeruginosa* PA01	NC_002516 (PA2425)	Winsor et al, 2009; Gross and Loper 2009; Loper et al, 2012
		PvdS	-	-	-	-	-	*Pseudomonas aeruginosa* PA01	NC_002516 (PA2426)	Winsor et al, 2009; Gross and Loper 2009; Loper et al, 2012
		PA2427	-	-	-	-	-	*Pseudomonas aeruginosa* PA01	NC_002516 (PA2427)	Winsor et al, 2009; Gross and Loper 2009; Loper et al, 2012
rhizobactin		RhbA	-	-	-	-	-	*Sinorhizobium meliloti* strain 2011	AF110737	Lynch et al, 2001
		RhbB	-	-	-	-	-	*Sinorhizobium meliloti* strain 2011	AF110737	Lynch et al, 2001
		RhbC	-	-	-	-	-	*Sinorhizobium meliloti* strain 2011	AF110737	Lynch et al, 2001
		RhbD	-	-	-	-	-	*Sinorhizobium meliloti* strain 2011	AF110737	Lynch et al, 2001
		RhbE	-	-	-	-	-	*Sinorhizobium meliloti* strain 2011	AF110737	Lynch et al, 2001
		RhbF	-	-	-	-	-	*Sinorhizobium meliloti* strain 2011	AF110737	Lynch et al, 2001
		RhRA	-	-	-	-	-	*Sinorhizobium meliloti* strain 2011	AF110737	Lynch et al, 2001
		RhtA	-	-	-	-	-	*Sinorhizobium meliloti* strain 2011	AF110737	Lynch et al, 2001
staphyloferrin		SbnA	-	-	-	-	-	*Staphylococcus aureus*	AY251022	Dale et al, 2004
		SbnB	-	-	-	-	-	*Staphylococcus aureus*	AY251022	Dale et al, 2004
		SbnC	-	-	-	-	-	*Staphylococcus aureus*	AY251022	Dale et al, 2004
		SbnD	-	-	-	-	-	*Staphylococcus aureus*	AY251022	Dale et al, 2004
		SbnE	-	-	-	-	-	*Staphylococcus aureus*	AY251022	Dale et al, 2004
		SbnF	-	-	-	-	-	*Staphylococcus aureus*	AY251022	Dale et al, 2004
		SbnG	-	-	-	-	-	*Staphylococcus aureus*	AY251022	Dale et al, 2004
		SbnH	-	-	-	-	-	*Staphylococcus aureus*	AY251022	Dale et al, 2004
		SbnI	-	-	-	-	-	*Staphylococcus aureus*	AY251022	Dale et al, 2004
vibroferrin		PvsA	-	-	-	+	-	*Xanthomonas campestris pv. raphani* 756C	AEL08250	Bogdanova et al, 2011
		PvsB	-	-	-	-	-	*Xanthomonas campestris pv. raphani* 756C	AEL08251	Bogdanova et al, 2011
		PvsA	-	-	-	-	-	*Vibrio parahaemolyticus* WP1	BAC16544	Tanabe et al, 2003
		PvsB	-	-	-	-	-	*Vibrio parahaemolyticus* WP1	BAC16545	Tanabe et al, 2003
		PvsC	-	-	-	-	-	*Vibrio parahaemolyticus* WP1	BAC16546	Tanabe et al, 2003
		PvsD	-	-	-	-	-	*Vibrio parahaemolyticus* WP1	BAC16547	Tanabe et al, 2003
		PvsE	-	-	-	-	-	*Vibrio parahaemolyticus* WP1	BAC16548	Tanabe et al, 2003
Xss		MphE	+	+	+	+	+	*Xanthomonas oryzae* pv *oryzae* KACC10331	NC_006834 (XOO1360)	Lee et al, 2005
		XsuA	-	-	-	-	-	*Xanthomonas oryzae* pv *oryzae* KACC10331	NC_006834 (XOO1369)	Lee et al, 2005
		XssA	-	-	-	-	-	*Xanthomonas oryzae* pv *oryzae* KACC10331	NC_006834 (XOO1358)	Lee et al, 2005
		XssB	-	-	-	-	-	*Xanthomonas oryzae* pv *oryzae* KACC10331	NC_006834 (XOO1357)	Lee et al, 2005
		XssC	-	-	-	-	-	*Xanthomonas oryzae* pv *oryzae* KACC10331	NC_006834 (XOO1356)	Lee et al, 2005
		XssD	-	-	-	-	-	*Xanthomonas oryzae* pv *oryzae* KACC10331	NC_006834 (XOO1355)	Lee et al, 2005
		XssE	-	-	-	-	-	*Xanthomonas oryzae* pv *oryzae* KACC10331	NC_006834 (XOO1354)	Lee et al, 2005
yersiniabactin		Pns	-	-	-	-	-	*Yersinia pestis* KIM10	NC_004088	Deng et al, 2002; Carniel 2001
		YbtE	-	-	-	-	-	*Yersinia pestis* KIM10	NC_004088	Deng et al, 2002; Carniel 2001
		YbtT	-	-	-	-	-	*Yersinia pestis* KIM10	NC_004088	Deng et al, 2002; Carniel 2001
		YbtU	-	-	-	-	-	*Yersinia pestis* KIM10	NC_004088	Deng et al, 2002; Carniel 2001
		Irp1	-	-	-	-	-	*Yersinia pestis* KIM10	NC_004088	Deng et al, 2002; Carniel 2001
		Irp2	-	-	-	-	-	*Yersinia pestis* KIM10	NC_004088	Deng et al, 2002; Carniel 2001
		YbtA	-	-	-	-	-	*Yersinia pestis* KIM10	NC_004088	Deng et al, 2002; Carniel 2001
		YbtP	-	-	-	-	-	*Yersinia pestis* KIM10	NC_004088	Deng et al, 2002; Carniel 2001
		YbtQ	-	-	-	-	-	*Yersinia pestis* KIM10	NC_004088	Deng et al, 2002; Carniel 2001
		YbtX	-	-	-	-	-	*Yersinia pestis* KIM10	NC_004088	Deng et al, 2002; Carniel 2001
		YbtS	-	-	-	-	-	*Yersinia pestis* KIM10	NC_004088	Deng et al, 2002; Carniel 2001
Toxins										
mangotoxin		MboA	-	-	-	-	-	*Pseudomonas syringae* pv. *syringae* UMAF0167	JX878400	Carrión et al, 2013
		MboB	-	-	-	-	-	*Pseudomonas syringae* pv. *syringae* UMAF0167	JX878400	Carrión et al, 2013
		MboC	-	-	-	-	-	*Pseudomonas syringae* pv. *syringae* UMAF0167	JX878400	Carrión et al, 2013
		MboD	-	-	-	-	-	*Pseudomonas syringae* pv. *syringae* UMAF0167	JX878400	Carrión et al, 2013
		MboE	-	-	-	-	-	*Pseudomonas syringae* pv. *syringae* UMAF0167	JX878400	Carrión et al, 2013
		MboF	-	-	-	-	-	*Pseudomonas syringae* pv. *syringae* UMAF0167	JX878400	Carrión et al, 2013
fit	insect toxin	FitA	+	+	-	-	-	*Pseudomonas protegens* Pf-5	CP000076 (PFL_2980)	Paulsen et al, 2005; Loper et al, 2012
		FitB	-	-	-	-	-	*Pseudomonas protegens* Pf-5	CP000076 (PFL_2981)	Paulsen et al, 2005; Loper et al, 2012
		FitC	+	+	-	-	-	*Pseudomonas protegens* Pf-5	CP000076 (PFL_2982)	Paulsen et al, 2005; Loper et al, 2012
		FitD	-	-	-	-	-	*Pseudomonas protegens* Pf-5	CP000076 (PFL_2983)	Paulsen et al, 2005; Loper et al, 2012
		FitE	-	-	-	-	-	*Pseudomonas protegens* Pf-5	CP000076 (PFL_2984)	Paulsen et al, 2005; Loper et al, 2012
		FitF	-	-	-	-	-	*Pseudomonas protegens* Pf-5	CP000076 (PFL_2985)	Paulsen et al, 2005; Loper et al, 2012
		FitG	-	-	-	-	-	*Pseudomonas protegens* Pf-5	CP000076 (PFL_2986)	Paulsen et al, 2005; Loper et al, 2012
		FitH	-	-	-	-	-	*Pseudomonas protegens* Pf-5	CP000076 (PFL_2987)	Paulsen et al, 2005; Loper et al, 2012
Tc complex	insecticidin	PSF113_0731	-	-	-	-	-	*Pseudomonas fluorescens* F113	NC_016830 (PSF113_0731)	Redondo-Nieto et al, 2012; Loper et al, 2012
		PSF113_0732	-	-	-	-	-	*Pseudomonas fluorescens* F113	NC_016830 (PSF113_0732)	Redondo-Nieto et al, 2012; Loper et al, 2012
		PSF113_0733	-	-	-	-	-	*Pseudomonas fluorescens* F113	NC_016830 (PSF113_0733)	Redondo-Nieto et al, 2012; Loper et al, 2012
		PSF113_0734	-	-	-	-	-	*Pseudomonas fluorescens* F113	NC_016830 (PSF113_0734)	Redondo-Nieto et al, 2012; Loper et al, 2012

Secretion systems									
General system	SecY	+	+	+	+	+	*Xanthomonas campestris* pv. *vesicatoria* str. 85-10	YP_362750	Thieme et al, 2005
	SecE	-	-	-	-	-	*Xanthomonas campestris* pv. *vesicatoria* str. 85-10	YP_362716	Thieme et al, 2005
	SecG	+	+	-	-	+	*Xanthomonas campestris* pv. *vesicatoria* str. 85-10	YP_364585	Thieme et al, 2005
	TatA	-	+	+	+	-	*Xanthomonas campestris* pv. *vesicatoria* str. 85-10	Q3BMG1	Thieme et al, 2005
	TatB	-	-	+	-	+	*Xanthomonas campestris* pv. *vesicatoria* str. 85-10	Q3BMG2	Thieme et al, 2005
	TatC	+	+	+	+	+	*Xanthomonas campestris* pv. *vesicatoria* str. 85-10	YP_366050	Thieme et al, 2005
T1SS	TolC	+	-	+	-	+	*Escherichia coli* K1-12	P02930	Hacket et al, 1983
	MsbA	-	-	-	-	-	*Vibrio cholerae* O1 biovar El Tor str. N16961	Q9KQW9	Heidelberg et al, 2000
	AcrA	-	-	-	-	-	*Escherichia coli* OH111:H- str. 11128	YP_003233025	Ogura et al, 2009
	SsmD	-	-	-	-	-	*Stenotrophomonas maltophilia* K279a	NC_010943 (Smlt2432)	Crossman et al, 2008
	TolC	-	-	-	-	-	*Stenotrophomonas maltophilia* K279a	NC_010943 (Smlt3928)	Crossman et al, 2008
	SsmJ	-	-	-	-	-	*Stenotrophomonas maltophilia* K279a	NC_010943 (Smlt0038)	Crossman et al, 2008
T2SS	ExeA	-	-	-	-	-	*Aeromonas hydrophila* Ah65	CAA57225	Howard et al, 1993
	ExeB	-	-	-	-	-	*Aeromonas hydrophila* Ah65	CAA57226	Howard et al, 1993
	ExeC	-	-	-	-	-	*Aeromonas hydrophila* Ah65	CAA47125	Howard et al, 1993
	ExeD	-	-	-	-	-	*Aeromonas hydrophila* Ah65	CAA47124	Howard et al, 1993
	ExeE	-	-	-	-	-	*Aeromonas hydrophila* Ah65	CAA47126	Howard et al, 1993
	ExeF	-	-	-	-	-	*Aeromonas hydrophila* Ah65	CAA47127	Howard et al, 1993
	ExeG	-	-	-	-	-	*Aeromonas hydrophila* Ah65	CAA47128	Howard et al, 1993
	ExeH	-	-	-	-	-	*Aeromonas hydrophila* Ah65	CAA47129	Howard et al, 1993
	ExeI	-	-	-	-	-	*Aeromonas hydrophila* Ah65	CAA47130	Howard et al, 1993
	ExeJ	-	-	-	-	-	*Aeromonas hydrophila* Ah65	CAA47131	Howard et al, 1993
	ExeK	-	-	-	-	-	*Aeromonas hydrophila* Ah65	CAA47132	Howard et al, 1993
	ExeL	-	-	-	-	-	*Aeromonas hydrophila* Ah65	CAA47133	Howard et al, 1993
	ExeM	-	-	-	-	-	*Aeromonas hydrophila* Ah65	CAA47134	Howard et al, 1993
	ExeN	-	-	-	-	-	*Aeromonas hydrophila* Ah65	CAA47135	Howard et al, 1993
	TapD	-	-	-	-	-	*Aeromonas hydrophila* Ah65	AAC43998	Howard et al, 1993
	Smlt2729	-	-	-	-	-	*Stenotrophomonas maltophilia* K279a	NC_010943 (Smlt2729)	Karaba et al, 2013; Crossman et al, 2008
	Smlt2730	-	-	-	-	-	*Stenotrophomonas maltophilia* K279a	NC_010943 (Smlt2730)	Karaba et al, 2013; Crossman et al, 2008
	GspH	-	-	-	-	-	*Stenotrophomonas maltophilia* K279a	NC_010943 (Smlt2731)	Karaba et al, 2013; Crossman et al, 2008
	GspI	-	-	-	-	-	*Stenotrophomonas maltophilia* K279a	NC_010943 (Smlt2732)	Karaba et al, 2013; Crossman et al, 2008
	GspJ	-	-	-	-	-	*Stenotrophomonas maltophilia* K279a	NC_010943 (Smlt2733)	Karaba et al, 2013; Crossman et al, 2008
	Smlt2735	-	-	-	-	-	*Stenotrophomonas maltophilia* K279a	NC_010943 (Smlt2735)	Karaba et al, 2013; Crossman et al, 2008
	Smlt2736	-	-	-	-	-	*Stenotrophomonas maltophilia* K279a	NC_010943 (Smlt2736)	Karaba et al, 2013; Crossman et al, 2008
	Smlt2737	-	-	-	-	-	*Stenotrophomonas maltophilia* K279a	NC_010943 (Smlt2737)	Karaba et al, 2013; Crossman et al, 2008
	GspF	-	-	-	-	-	*Stenotrophomonas maltophilia* K279a	NC_010943 (Smlt2740)	Karaba et al, 2013; Crossman et al, 2008
	GspE	-	-	-	-	-	*Stenotrophomonas maltophilia* K279a	NC_010943 (Smlt2741)	Karaba et al, 2013; Crossman et al, 2008
	GspD	-	-	-	-	-	*Stenotrophomonas maltophilia* K279a	NC_010943 (Smlt2742)	Karaba et al, 2013; Crossman et al, 2008
	GspM	-	-	-	-	-	*Stenotrophomonas maltophilia* K279a	NC_010943 (Smlt2743)	Karaba et al, 2013; Crossman et al, 2008
	GspL	-	-	-	-	-	*Stenotrophomonas maltophilia* K279a	NC_010943 (Smlt2744)	Karaba et al, 2013; Crossman et al, 2008
	GspK	-	-	-	-	-	*Stenotrophomonas maltophilia* K279a	NC_010943 (Smlt2745)	Karaba et al, 2013; Crossman et al, 2008
	GspG	-	-	-	-	-	*Stenotrophomonas maltophilia* K279a	NC_010943 (Smlt2746)	Karaba et al, 2013; Crossman et al, 2008
	Smlt2747	-	-	-	-	-	*Stenotrophomonas maltophilia* K279a	NC_010943 (Smlt2747)	Karaba et al, 2013; Crossman et al, 2008
	XspE	+	+	+	+	+	*Stenotrophomonas maltophilia* K279a	NC_010943 (Smlt0687)	Karaba et al, 2013; Crossman et al, 2008
	XspF	+	+	+	+	+	*Stenotrophomonas maltophilia* K279a	NC_010943 (Smlt0688)	Karaba et al, 2013; Crossman et al, 2008
	XspG	+	+	+	+	+	*Stenotrophomonas maltophilia* K279a	NC_010943 (Smlt0689)	Karaba et al, 2013; Crossman et al, 2008
	XspH	-	-	-	-	-	*Stenotrophomonas maltophilia* K279a	NC_010943 (Smlt0690)	Karaba et al, 2013; Crossman et al, 2008
	XspI	-	-	-	-	-	*Stenotrophomonas maltophilia* K279a	NC_010943 (Smlt0691)	Karaba et al, 2013; Crossman et al, 2008
	XspJ	-	-	-	-	-	*Stenotrophomonas maltophilia* K279a	NC_010943 (Smlt0692)	Karaba et al, 2013; Crossman et al, 2008
	XspK	-	-	-	-	-	*Stenotrophomonas maltophilia* K279a	NC_010943 (Smlt0693)	Karaba et al, 2013; Crossman et al, 2008
	XspL	-	-	-	-	-	*Stenotrophomonas maltophilia* K279a	NC_010943 (Smlt0694)	Karaba et al, 2013; Crossman et al, 2008
	XspM	-	-	-	-	-	*Stenotrophomonas maltophilia* K279a	NC_010943 (Smlt0695)	Karaba et al, 2013; Crossman et al, 2008
	XspN	-	-	-	-	-	*Stenotrophomonas maltophilia* K279a	NC_010943 (Smlt0696)	Karaba et al, 2013; Crossman et al, 2008
	XspD	+	+	+	+	+	*Stenotrophomonas maltophilia* K279a	NC_010943 (Smlt0697)	Karaba et al, 2013; Crossman et al, 2008
T3SS	HrcC	-	-	-	-	-	*Pseudomonas syringae* pv *tomato* DC3000	NC_004578 (PSPTO_1389)	Buell et al, 2003
	HrcJ	-	-	-	-	-	*Pseudomonas syringae* pv *tomato* DC3000	NC_004578 (PSPTO_1384)	Buell et al, 2003
	HrcN	-	-	-	-	-	*Pseudomonas syringae* pv *tomato* DC3000	NC_004578 (PSPTO_1400)	Buell et al, 2003
	HrcQa	-	-	-	-	-	*Pseudomonas syringae* pv *tomato* DC3000	NC_004578 (PSPTO_1397)	Buell et al, 2003
	HrcQb	-	-	-	-	-	*Pseudomonas syringae* pv *tomato* DC3000	NC_004578 (PSPTO_1396)	Buell et al, 2003
	HrcR	-	-	-	-	-	*Pseudomonas syringae* pv *tomato* DC3000	NC_004578 (PSPTO_1395)	Buell et al, 2003
	HrcS	-	-	-	-	-	*Pseudomonas syringae* pv *tomato* DC3000	NC_004578 (PSPTO_1394)	Buell et al, 2003
	HrcT	-	-	-	-	-	*Pseudomonas syringae* pv *tomato* DC3000	NC_004578 (PSPTO_1393)	Buell et al, 2003
	HrcU	-	-	-	-	-	*Pseudomonas syringae* pv *tomato* DC3000	NC_004578 (PSPTO_1392)	Buell et al, 2003
	HrcV	-	-	-	-	-	*Pseudomonas syringae* pv *tomato* DC3000	NC_004578 (PSPTO_1402)	Buell et al, 2003
	FliC	-	-	-	-	-	*Xanthomonas campestris* pv. *campestris* str. ATCC 33913	NC_003902	Da Silva et al, 2002
	FliD	-	-	-	-	-	*Xanthomonas campestris* pv. *campestris* str. ATCC 33913	NC_003902	Da Silva et al, 2002
	FlgL	-	-	-	-	-	*Xanthomonas campestris* pv. *campestris* str. ATCC 33913	NC_003902	Da Silva et al, 2002
	FglK	-	-	-	-	-	*Xanthomonas campestris* pv. *campestris* str. ATCC 33913	NC_003902	Da Silva et al, 2002
	FlgD	-	-	-	-	-	*Xanthomonas campestris* pv. *campestris* str. ATCC 33913	NC_003902	Da Silva et al, 2002
	FlgE	-	-	-	-	-	*Xanthomonas campestris* pv. *campestris* str. ATCC 33913	NC_003902	Da Silva et al, 2002
	FlgF	-	-	-	-	-	*Xanthomonas campestris* pv. *campestris* str. ATCC 33913	NC_003902	Da Silva et al, 2002
	FLgG	-	-	-	-	-	*Xanthomonas campestris* pv. *campestris* str. ATCC 33913	NC_003902	Da Silva et al, 2002
	MotB	-	-	-	-	-	*Xanthomonas campestris* pv. *campestris* str. ATCC 33913	NC_003902	Da Silva et al, 2002
	MotA	-	-	-	-	-	*Xanthomonas campestris* pv. *campestris* str. ATCC 33913	NC_003902	Da Silva et al, 2002
	FliE	-	-	-	-	-	*Xanthomonas campestris* pv. *campestris* str. ATCC 33913	NC_003902	Da Silva et al, 2002

	FlgB	-	-	-	-	-	*Xanthomonas campestris* pv. *campestris* str. ATCC 33913	NC_003902	Da Silva et al, 2002
	FlgC	-	-	-	-	-	*Xanthomonas campestris* pv. *campestris* str. ATCC 33913	NC_003902	Da Silva et al, 2002
	FlgG	-	-	-	-	-	*Xanthomonas campestris* pv. *campestris* str. ATCC 33913	NC_003902	Da Silva et al, 2002
	FliM	-	-	-	-	-	*Xanthomonas campestris* pv. *campestris* str. ATCC 33913	NC_003902	Da Silva et al, 2002
	FliN	-	-	-	-	-	*Xanthomonas campestris* pv. *campestris* str. ATCC 33913	NC_003902	Da Silva et al, 2002
	FlhA	-	-	-	-	-	*Xanthomonas campestris* pv. *campestris* str. ATCC 33913	NC_003902	Da Silva et al, 2002
	FlhB	-	-	-	-	-	*Xanthomonas campestris* pv. *campestris* str. ATCC 33913	NC_003902	Da Silva et al, 2002
	FliH	-	-	-	-	-	*Xanthomonas campestris* pv. *campestris* str. ATCC 33913	NC_003902	Da Silva et al, 2002
	FliL	-	-	-	-	-	*Xanthomonas campestris* pv. *campestris* str. ATCC 33913	NC_003902	Da Silva et al, 2002
	FliQ	-	-	-	-	-	*Xanthomonas campestris* pv. *campestris* str. ATCC 33913	NC_003902	Da Silva et al, 2002
	FliP	-	-	-	-	-	*Xanthomonas campestris* pv. *campestris* str. ATCC 33913	NC_003902	Da Silva et al, 2002
	FliR	-	-	-	-	-	*Xanthomonas campestris* pv. *campestris* str. ATCC 33913	NC_003902	Da Silva et al, 2002
	FliK	-	-	-	-	-	*Xanthomonas campestris* pv. *campestris* str. ATCC 33913	NC_003902	Da Silva et al, 2002
	FlgH	-	-	-	-	-	*Xanthomonas campestris* pv. *campestris* str. ATCC 33913	NC_003902	Da Silva et al, 2002
	FlgI	-	-	-	-	-	*Xanthomonas campestris* pv. *campestris* str. ATCC 33913	NC_003902	Da Silva et al, 2002
	FliO	-	-	-	-	-	*Xanthomonas campestris* pv. *campestris* str. ATCC 33913	NC_003902	Da Silva et al, 2002
	FlgA	-	-	-	-	-	*Xanthomonas campestris* pv. *campestris* str. ATCC 33913	NC_003902	Da Silva et al, 2002
	FlgJ	-	-	-	-	-	*Xanthomonas campestris* pv. *campestris* str. ATCC 33913	NC_003902	Da Silva et al, 2002
	FlhF	-	-	-	-	-	*Xanthomonas campestris* pv. *campestris* str. ATCC 33913	NC_003902	Da Silva et al, 2002
	FliA	-	-	-	-	-	*Xanthomonas campestris* pv. *campestris* str. ATCC 33913	NC_003902	Da Silva et al, 2002
	FliI	-	-	-	-	-	*Xanthomonas campestris* pv. *campestris* str. ATCC 33913	NC_003902	Da Silva et al, 2002
	FliS	-	-	-	-	-	*Xanthomonas campestris* pv. *campestris* str. ATCC 33913	NC_003902	Da Silva et al, 2002
	FlgM	-	-	-	-	-	*Xanthomonas campestris* pv. *campestris* str. ATCC 33913	NC_003902	Da Silva et al, 2002
	FlgB	-	-	-	-	-	*Xanthomonas campestris* pv. *campestris* str. ATCC 33913	NC_003902	Da Silva et al, 2002
	FlgC	-	-	-	-	-	*Xanthomonas campestris* pv. *campestris* str. ATCC 33913	NC_003902	Da Silva et al, 2002
	FliJ	-	-	-	-	-	*Xanthomonas campestris* pv. *campestris* str. ATCC 33913	NC_003902	Da Silva et al, 2002
	FliG	-	-	-	-	-	*Xanthomonas campestris* pv. *campestris* str. ATCC 33913	NC_003902	Da Silva et al, 2002
	FleN	-	-	-	-	-	*Xanthomonas campestris* pv. *campestris* str. ATCC 33913	NC_003902	Da Silva et al, 2002
ATPase FliI/YscN family	LEC3_5105	+	+	+	+	+	*Lysobacter enzymogenes* C3	-	-
type III secretion appara	LEC3_5106	+	+	+	+	+	*Lysobacter enzymogenes* C3	-	-
hypothetical protein	LEC3_5107	+	+	+	+	+	*Lysobacter enzymogenes* C3	-	-
type III secretion appara	LEC3_5108	+	+	+	+	+	*Lysobacter enzymogenes* C3	-	-
conserved hypothetical	LEC3_5109	+	+	+	+	+	*Lysobacter enzymogenes* C3	-	-
conserved hypothetical	LEC3_5110	+	+	+	+	+	*Lysobacter enzymogenes* C3	-	-
type III secretion appara	LEC3_5111	+	+	+	+	+	*Lysobacter enzymogenes* C3	-	-
type III secretion protein	LEC3_5112	+	+	+	+	+	*Lysobacter enzymogenes* C3	-	-
hypothetical protein	LEC3_5113	-	-	-	-	+	*Lysobacter enzymogenes* C3	-	-
type III secretion protein	LEC3_5114	+	+	+	+	+	*Lysobacter enzymogenes* C3	-	-
His Kinase A (phosphoa	LEC3_5115	+	+	+	+	+	*Lysobacter enzymogenes* C3	-	-
hypothetical protein	LEC3_5116	-	-	-	-	+	*Lysobacter enzymogenes* C3	-	-
conserved hypothetical	LEC3_5117	-	-	-	-	+	*Lysobacter enzymogenes* C3	-	-
response regulator rece	LEC3_5118	+	+	+	+	+	*Lysobacter enzymogenes* C3	-	-
type III secretion appara	LEC3_5119	+	+	+	+	+	*Lysobacter enzymogenes* C3	-	-
type III secretion appara	LEC3_5120	+	+	+	+	+	*Lysobacter enzymogenes* C3	-	-
type III secretion family	LEC3_5121	+	+	+	+	+	*Lysobacter enzymogenes* C3	-	-
type III secretion appara	LEC3_5122	+	+	+	+	+	*Lysobacter enzymogenes* C3	-	-
type III secretion protein	LEC3_5123	+	+	+	+	+	*Lysobacter enzymogenes* C3	-	-
T3SS-I	HrpR	-	-	-	-	-	Pseudomonads	-	Victor
T3SS-I	HrpS	-	-	-	-	-	Pseudomonads	-	Victor
T3SS-I	HrpA	-	-	-	-	-	Pseudomonads	-	Victor
T3SS-I	HrpZ	-	-	-	-	-	Pseudomonads	-	Victor
T3SS-I	HrpB	-	-	-	-	-	Pseudomonads	-	Victor
T3SS-I	HrcJ	-	-	-	-	-	Pseudomonads	-	Victor
T3SS-I	HrpD	-	-	-	-	-	Pseudomonads	-	Victor
T3SS-I	YscL	-	-	-	-	-	Pseudomonads	-	Victor
T3SS-I	HrpF	-	-	-	-	-	Pseudomonads	-	Victor
T3SS-I	HrpG	-	-	-	-	-	Pseudomonads	-	Victor
T3SS-I	YscC	-	-	-	-	-	Pseudomonads	-	Victor
T3SS-I	HrpT	-	-	-	-	-	Pseudomonads	-	Victor
T3SS-I	HrpV	-	-	-	-	-	Pseudomonads	-	Victor
T3SS-I	YscU	-	-	-	-	-	Pseudomonads	-	Victor
T3SS-I	cell_division	-	-	-	-	-	Pseudomonads	-	Victor
T3SS-I	HrcT	-	-	-	-	-	Pseudomonads	-	Victor
T3SS-I	YscS	-	-	-	-	-	Pseudomonads	-	Victor
T3SS-I	YscR	-	-	-	-	-	Pseudomonads	-	Victor
T3SS-I	HrcQa	-	-	-	-	-	Pseudomonads	-	Victor
T3SS-I	HrcQb	-	-	-	-	-	Pseudomonads	-	Victor
T3SS-I	HrpP	-	-	-	-	-	Pseudomonads	-	Victor
T3SS-I	YscO	-	-	-	-	-	Pseudomonads	-	Victor
T3SS-I	FliI	-	-	-	-	-	Pseudomonads	-	Victor
T3SS-I	HrpQ	-	-	-	-	-	Pseudomonads	-	Victor
T3SS-I	HrcV	-	-	-	-	-	Pseudomonads	-	Victor
T3SS-I	HrpJ	-	-	-	-	-	Pseudomonads	-	Victor
T3SS-I	HrpL	-	-	-	-	-	Pseudomonads	-	Victor
T3SS-I	exoprotein	-	-	-	-	-	Pseudomonads	-	Victor
T3SS-I	HopA1	-	-	-	-	-	Pseudomonads	-	Victor

	T3SS-I	ShcA	-	-	-	-	-	Pseudomonads	-	Victor
	T3SS-II	Hyp.1	-	-	-	-	-	Pseudomonads	-	Victor
	T3SS-II	Hyp.2	-	-	-	-	-	Pseudomonads	-	Victor
	T3SS-II	Hyp.3	-	-	-	-	-	Pseudomonads	-	Victor
	T3SS-II	Hyp.4	-	-	-	-	-	Pseudomonads	-	Victor
	T3SS-II	Hyp.5	-	-	-	-	-	Pseudomonads	-	Victor
	T3SS-II	Hyp.6	-	-	-	-	-	Pseudomonads	-	Victor
	T3SS-II	RhcV	-	-	-	-	-	Pseudomonads	-	Victor
	T3SS-II	RhcC2	-	-	-	-	-	Pseudomonads	-	Victor
	T3SS-II	Hyp.7	-	-	-	-	-	Pseudomonads	-	Victor
	T3SS-II	gene8	-	-	-	-	-	Pseudomonads	-	Victor
	T3SS-II	RhcC1	-	-	-	-	-	Pseudomonads	-	Victor
	T3SS-II	Hyp.9	-	-	-	-	-	Pseudomonads	-	Victor
	T3SS-II	Hyp. 10	-	-	-	-	-	Pseudomonads	-	Victor
	T3SS-II	RhcJ	-	-	-	-	-	Pseudomonads	-	Victor
	T3SS-II	Hyp. 11	-	-	-	-	-	Pseudomonads	-	Victor
	T3SS-II	RhcL	-	-	-	-	-	Pseudomonads	-	Victor
	T3SS-II	RhcN	-	-	-	-	-	Pseudomonads	-	Victor
	T3SS-II	MhcB	-	-	-	-	-	Pseudomonads	-	Victor
	T3SS-II	RhcQ	-	-	-	-	-	Pseudomonads	-	Victor
	T3SS-II	RhcR	-	-	-	-	-	Pseudomonads	-	Victor
	T3SS-II	RhcS	-	-	-	-	-	Pseudomonads	-	Victor
	T3SS-II	RhcU	-	-	-	-	-	Pseudomonads	-	Victor
	T3SS-II	gene12	-	-	-	-	-	Pseudomonads	-	Victor
	T3SS-II	gene13	-	-	-	-	-	Pseudomonads	-	Victor
	T3SS-II	cds	-	-	-	-	-	Pseudomonads	-	Victor
	T3SS-II	cds14	-	-	-	-	-	Pseudomonads	-	Victor
	T3SS-II	cds15	-	-	-	-	-	Pseudomonads	-	Victor
T4SS		VirB1	-	-	-	-	-	*Agrobacterium tumefaciens* plasmid pTiAB2/73	AF329849	Schmidt et al, unsubmitted; Warren et al, 2013
		VirB2	-	-	-	-	-	*Agrobacterium tumefaciens* plasmid pTiAB2/73	AF329849	Schmidt et al, unsubmitted; Warren et al, 2013
		VirB3	-	-	-	-	-	*Agrobacterium tumefaciens* plasmid pTiAB2/73	AF329849	Schmidt et al, unsubmitted; Warren et al, 2013
		VirB4	-	-	-	-	-	*Agrobacterium tumefaciens* plasmid pTiAB2/73	AF329849	Schmidt et al, unsubmitted; Warren et al, 2013
		VirB5	-	-	-	-	-	*Agrobacterium tumefaciens* plasmid pTiAB2/73	AF329849	Schmidt et al, unsubmitted; Warren et al, 2013
		VirB6	-	-	-	-	-	*Agrobacterium tumefaciens* plasmid pTiAB2/73	AF329849	Schmidt et al, unsubmitted; Warren et al, 2013
		VirB7	-	-	-	-	-	*Agrobacterium tumefaciens* plasmid pTiAB2/73	AF329849	Schmidt et al, unsubmitted; Warren et al, 2013
		VirB8	-	-	-	-	-	*Agrobacterium tumefaciens* plasmid pTiAB2/73	AF329849	Schmidt et al, unsubmitted; Warren et al, 2013
		VirB9	-	-	-	-	-	*Agrobacterium tumefaciens* plasmid pTiAB2/73	AF329849	Schmidt et al, unsubmitted; Warren et al, 2013
		VirB10	-	-	-	-	-	*Agrobacterium tumefaciens* plasmid pTiAB2/73	AF329849	Schmidt et al, unsubmitted; Warren et al, 2013
		VirB11	-	-	-	-	-	*Agrobacterium tumefaciens* plasmid pTiAB2/73	AF329849	Schmidt et al, unsubmitted; Warren et al, 2013
		VirD4	-	-	-	-	-	*Agrobacterium tumefaciens* plasmid pTiAB2/73	AF329849	Schmidt et al, unsubmitted; Warren et al, 2013
		TrbI/VirB10	-	-	-	-	-	*Stenotrophomonas maltophilia* K279a	NC_010943 (Smlt1283)	Crossman et al, 2008
		TrbG	-	-	-	-	-	*Stenotrophomonas maltophilia* K279a	NC_010943 (Smlt1284)	Crossman et al, 2008
		TrbF	-	-	-	-	-	*Stenotrophomonas maltophilia* K279a	NC_010943 (Smlt1285)	Crossman et al, 2008
		TrbL/VirB6	-	-	-	-	-	*Stenotrophomonas maltophilia* K279a	NC_010943 (Smlt1286)	Crossman et al, 2008
		TrbJ	-	-	-	-	-	*Stenotrophomonas maltophilia* K279a	NC_010943 (Smlt1287)	Crossman et al, 2008
		TrbE/VirB4	-	-	-	-	-	*Stenotrophomonas maltophilia* K279a	NC_010943 (Smlt1288)	Crossman et al, 2008
		TrbD	-	-	-	-	-	*Stenotrophomonas maltophilia* K279a	NC_010943 (Smlt1289)	Crossman et al, 2008
		TrbC/VirB2	-	-	-	-	-	*Stenotrophomonas maltophilia* K279a	NC_010943 (Smlt1290)	Crossman et al, 2008
		TrbB/VirB11	-	-	-	-	-	*Stenotrophomonas maltophilia* K279a	NC_010943 (Smlt1291)	Crossman et al, 2008
		TraG/VirD4	-	-	-	-	-	*Stenotrophomonas maltophilia* K279a	NC_010943 (Smlt1293)	Crossman et al, 2008
T5SS		TspB	-	-	-	-	-	*Neisseria meningitidis* 69166	EJU62795	no info; Tseng et al, 2012
		Omp85	-	-	-	-	-	*Escherichia coli* str. K-12 substr. MG1655	AAC73288	Blattner et al, 1997
		Smlt1001	-	-	-	-	-	*Stenotrophomonas maltophilia* K279a	NC_010943 (Smlt1001)	Karaba et al, 2013
		AlpA	-	-	-	-	-	*Helicobacter pylori* OK310	YP_007538498	Yahara et al, 2013
		SphB	-	-	-	-	-	*Bordetella pertussis* CS	YP_005588486	Zhang et al, 2011
		AspA/NalP	-	-	-	-	-	*Neisseria meningitidis* H44/76	AAN71715	Van Ulsen et al, 2001
		IgA1	-	-	-	-	-	*Neisseria gonorrhoeae*	P09790	Pohlner et al, 1987
T6SS		VasA	-	-	-	-	-	*Aeromonas hydrophila* SSU	ABG57138	Suarez et al, 2008
		VasF	-	-	-	-	-	*Aeromonas hydrophila* SSU	ABG57143	Suarez et al, 2008
		VasH	-	-	-	-	-	*Aeromonas hydrophila* SSU	ABG57145	Suarez et al, 2008
		VasK	-	-	-	-	-	*Aeromonas hydrophila* SSU	ABG57148	Suarez et al, 2008
		VasA	-	-	-	-	+	*Xanthomonas campestris* pv. *vesicatoria* str. 85-10	YP_365969	Thieme et al, 2005
		ClpB	-	-	-	+	+	*Xanthomonas campestris* pv. *vesicatoria* str. 85-10	YP_365967	Thieme et al, 2005
	type VI secretion protei	LEC3_0431	-	-	-	-	+	*Lysobacter enzymogenes* C3	-	-
	type VI secretion-associ	LEC3_0432	-	-	-	-	+	*Lysobacter enzymogenes* C3	-	-
	OmpA/MotB family prot	LEC3_0433	-	-	-	-	+	*Lysobacter enzymogenes* C3	-	-
	hypothetical protein	LEC3_0434	-	-	-	-	+	*Lysobacter enzymogenes* C3	-	-
	type VI secretion-associ	LEC3_0435	-	-	-	-	+	*Lysobacter enzymogenes* C3	-	-
	type VI secretion systen	LEC3_0436	-	-	-	-	+	*Lysobacter enzymogenes* C3	-	-
	conserved hypothetical	LEC3_0437	-	-	-	-	+	*Lysobacter enzymogenes* C3	-	-
	conserved hypothetical	LEC3_0438	-	-	-	-	+	*Lysobacter enzymogenes* C3	-	-
	hypothetical protein	LEC3_0439	-	-	-	-	+	*Lysobacter enzymogenes* C3	-	-
	hypothetical protein	LEC3_0440	-	-	-	-	+	*Lysobacter enzymogenes* C3	-	-
	arginine deiminase	LEC3_0441	-	-	-	-	+	*Lysobacter enzymogenes* C3	-	-
	type VI secretion systen	LEC3_0442	-	-	-	-	+	*Lysobacter enzymogenes* C3	-	-
	type IV / VI secretion sy	LEC3_0443	-	-	-	-	+	*Lysobacter enzymogenes* C3	-	-

	type VI secretion protei	LEC3_0444	-	-	-	-	+	*Lysobacter enzymogenes* C3	-	-
	type VI secretion lipopro	LEC3_0445	-	-	-	-	+	*Lysobacter enzymogenes* C3	-	-
	tetratricopeptide repeat	LEC3_0446	-	-	-	-	+	*Lysobacter enzymogenes* C3	-	-
	type VI secretion protei	LEC3_0447	-	-	-	+	+	*Lysobacter enzymogenes* C3	-	-
	type VI secretion protei	LEC3_0448	-	-	-	+	+	*Lysobacter enzymogenes* C3	-	-
	conserved hypothetical	LEC3_0449	-	-	-	-	+	*Lysobacter enzymogenes* C3	-	-
	lysine-specific histone d	LEC3_0450	-	-	-	-	+	*Lysobacter enzymogenes* C3	-	-
	type VI secretion systen	LEC3_0451	-	-	-	-	+	*Lysobacter enzymogenes* C3	-	-
	type VI secretion protei	LEC3_0452	-	-	-	-	+	*Lysobacter enzymogenes* C3	-	-
	type VI secretion protei	LEC3_0453	-	-	-	-	+	*Lysobacter enzymogenes* C3	-	-
	type VI secretion ATPas	LEC3_0454	-	-	-	+	+	*Lysobacter enzymogenes* C3	-	-
	type VI secretion systen	LEC3_0455	-	-	-	-	+	*Lysobacter enzymogenes* C3	-	-
	type VI secretion protei	LEC3_2572	-	-	-	-	+	*Lysobacter enzymogenes* C3	-	-
	type VI secretion protei	LEC3_2573	-	-	-	-	+	*Lysobacter enzymogenes* C3	-	-
	conserved hypothetical	LEC3_2574	-	-	-	-	+	*Lysobacter enzymogenes* C3	-	-
	ImpE/SciE family protei	LEC3_2575	-	-	-	-	+	*Lysobacter enzymogenes* C3	-	-
	type VI secretion systen	LEC3_2576	-	-	-	-	+	*Lysobacter enzymogenes* C3	-	-
	type VI secretion protei	LEC3_2577	-	-	-	-	+	*Lysobacter enzymogenes* C3	-	-
	type VI secretion protei	LEC3_2578	-	-	-	-	+	*Lysobacter enzymogenes* C3	-	-
	type VI secretion ATPas	LEC3_2579	-	-	-	+	+	*Lysobacter enzymogenes* C3	-	-
	hypothetical protein	LEC3_2580	-	-	-	-	+	*Lysobacter enzymogenes* C3	-	-
	type VI secretion systen	LEC3_2581	-	-	-	-	+	*Lysobacter enzymogenes* C3	-	-
	conserved hypothetical	LEC3_2582	-	-	-	-	+	*Lysobacter enzymogenes* C3	-	-
	type VI secretion systen	LEC3_2583	-	-	-	-	+	*Lysobacter enzymogenes* C3	-	-
	type VI secretion protei	LEC3_2584	-	-	-	-	+	*Lysobacter enzymogenes* C3	-	-
	type VI secretion systen	LEC3_2585	-	-	-	-	+	*Lysobacter enzymogenes* C3	-	-
	type VI secretion protei	LEC3_2586	-	-	-	-	+	*Lysobacter enzymogenes* C3	-	-
	type VI secretion-assoc	LEC3_2587	-	-	-	-	+	*Lysobacter enzymogenes* C3	-	-
	type VI secretion-assoc	LEC3_2591	-	-	-	-	+	*Lysobacter enzymogenes* C3	-	-
	type VI secretion systen	LEC3_3331	-	-	-	-	+	*Lysobacter enzymogenes* C3		
T7SS		Rv3864	-	-	-	-	-	*Mycobacterium tuberculosis* H37Rv	NC_000962 (Rv3864)	Cole et al, 1998; Abdallah et al, 207
		Rv3865	-	-	-	-	-	*Mycobacterium tuberculosis* H37Rv	NC_000962 (Rv3865)	Cole et al, 1998; Abdallah et al, 207
		Rv3866	-	-	-	-	-	*Mycobacterium tuberculosis* H37Rv	NC_000962 (Rv3866)	Cole et al, 1998; Abdallah et al, 207
		Rv3867	-	-	-	-	-	*Mycobacterium tuberculosis* H37Rv	NC_000962 (Rv3867)	Cole et al, 1998; Abdallah et al, 207
		Rv3868	-	-	-	-	-	*Mycobacterium tuberculosis* H37Rv	NC_000962 (Rv3868)	Cole et al, 1998; Abdallah et al, 207
		Rv3869	-	-	-	-	-	*Mycobacterium tuberculosis* H37Rv	NC_000962 (Rv3869)	Cole et al, 1998; Abdallah et al, 207
		Rv3870	-	-	-	-	-	*Mycobacterium tuberculosis* H37Rv	NC_000962 (Rv3870)	Cole et al, 1998; Abdallah et al, 207
		Rv3871	-	-	-	-	-	*Mycobacterium tuberculosis* H37Rv	NC_000962 (Rv3871)	Cole et al, 1998; Abdallah et al, 207
		Rv3872	-	-	-	-	-	*Mycobacterium tuberculosis* H37Rv	NC_000962 (Rv3872)	Cole et al, 1998; Abdallah et al, 207
		Rv3873	-	-	-	-	-	*Mycobacterium tuberculosis* H37Rv	NC_000962 (Rv3873)	Cole et al, 1998; Abdallah et al, 207
		Rv3874	-	-	-	-	-	*Mycobacterium tuberculosis* H37Rv	NC_000962 (Rv3874)	Cole et al, 1998; Abdallah et al, 207
		Rv3875	-	-	-	-	-	*Mycobacterium tuberculosis* H37Rv	NC_000962 (Rv3875)	Cole et al, 1998; Abdallah et al, 207
		Rv3876	-	-	-	-	-	*Mycobacterium tuberculosis* H37Rv	NC_000962 (Rv3876)	Cole et al, 1998; Abdallah et al, 207
		Rv3877	-	-	-	-	-	*Mycobacterium tuberculosis* H37Rv	NC_000962 (Rv3877)	Cole et al, 1998; Abdallah et al, 207
		Rv3878	-	-	-	-	-	*Mycobacterium tuberculosis* H37Rv	NC_000962 (Rv3878)	Cole et al, 1998; Abdallah et al, 207
		MycP1	-	-	-	-	-	*Mycobacterium tuberculosis* H37Rv	NC_000962 (Rv3883)	Cole et al, 1998; Abdallah et al, 207
<u>Attachment/motility</u>										
exopolysaccharides/xanthan		GumP	-	-	-	-	-	*Xanthomonas campestris* pv. *campestris* str. ATCC 33913	NP_637792	Da Silva et al, 2002
		GumO	-	-	-	-	-	*Xanthomonas campestris* pv. *campestris* str. ATCC 33913	NP_637793	Da Silva et al, 2002
		GumN	-	-	-	-	-	*Xanthomonas campestris* pv. *campestris* str. ATCC 33913	NP_637794	Da Silva et al, 2002
		Hypothetical	-	-	-	-	-	*Xanthomonas campestris* pv. *campestris* str. ATCC 33913	NP_637795	Da Silva et al, 2002
		GumM	-	-	-	-	-	*Xanthomonas campestris* pv. *campestris* str. ATCC 33913	NP_637796	Da Silva et al, 2002
		GumL	-	-	-	-	-	*Xanthomonas campestris* pv. *campestris* str. ATCC 33913	NP_637797	Da Silva et al, 2002
		GumK	-	-	-	-	-	*Xanthomonas campestris* pv. *campestris* str. ATCC 33913	NP_637798	Da Silva et al, 2002
		GumJ	-	-	-	-	-	*Xanthomonas campestris* pv. *campestris* str. ATCC 33913	NP_637799	Da Silva et al, 2002
		GumI	-	-	-	-	-	*Xanthomonas campestris* pv. *campestris* str. ATCC 33913	NP_637800	Da Silva et al, 2002
		GumH	-	-	-	-	-	*Xanthomonas campestris* pv. *campestris* str. ATCC 33913	NP_637801	Da Silva et al, 2002
		GumG	-	-	-	-	-	*Xanthomonas campestris* pv. *campestris* str. ATCC 33913	NP_637802	Da Silva et al, 2002
		GumF	-	-	-	-	-	*Xanthomonas campestris* pv. *campestris* str. ATCC 33913	NP_637803	Da Silva et al, 2002
		GumE	-	-	-	-	-	*Xanthomonas campestris* pv. *campestris* str. ATCC 33913	NP_637804	Da Silva et al, 2002
		GumD	-	-	-	-	-	*Xanthomonas campestris* pv. *campestris* str. ATCC 33913	NP_637805	Da Silva et al, 2002
		GumC	-	-	-	-	-	*Xanthomonas campestris* pv. *campestris* str. ATCC 33913	NP_637806	Da Silva et al, 2002
		GumB	-	-	-	-	-	*Xanthomonas campestris* pv. *campestris* str. ATCC 33913	NP_637807	Da Silva et al, 2002
		EpsA	-	-	-	-	-	*Bacillus amyloliquefaciens* subsp. *plantarum* str. FZB42	ABS75498	Chen et al, 2007
		EpsB	-	-	-	-	-	*Bacillus amyloliquefaciens* subsp. *plantarum* str. FZB42	ABS75497	Chen et al, 2007
		EpsC	-	-	-	-	-	*Bacillus amyloliquefaciens* subsp. *plantarum* str. FZB42	ABS75496	Chen et al, 2007
		EpsD	-	-	-	-	-	*Bacillus amyloliquefaciens* subsp. *plantarum* str. FZB42	ABS75495	Chen et al, 2007
		EpsE	-	-	-	-	-	*Bacillus amyloliquefaciens* subsp. *plantarum* str. FZB42	ABS75494	Chen et al, 2007
		EpsF	-	-	-	-	-	*Bacillus amyloliquefaciens* subsp. *plantarum* str. FZB42	ABS75493	Chen et al, 2007
		EpsG	-	-	-	-	-	*Bacillus amyloliquefaciens* subsp. *plantarum* str. FZB42	ABS75492	Chen et al, 2007
		EpsH	-	-	-	-	-	*Bacillus amyloliquefaciens* subsp. *plantarum* str. FZB42	ABS75491	Chen et al, 2007
		EpsI	-	-	-	-	-	*Bacillus amyloliquefaciens* subsp. *plantarum* str. FZB42	ABS75490	Chen et al, 2007
		EpsJ	-	-	-	-	-	*Bacillus amyloliquefaciens* subsp. *plantarum* str. FZB42	ABS75489	Chen et al, 2007
		EpsK	-	-	-	-	-	*Bacillus amyloliquefaciens* subsp. *plantarum* str. FZB42	ABS75488	Chen et al, 2007
		EpsL	-	-	-	-	-	*Bacillus amyloliquefaciens* subsp. *plantarum* str. FZB42	ABS75487	Chen et al, 2007

		EpsM	-	-	-	-	-	*Bacillus amyloliquefaciens* subsp. *plantarum* str. FZB42	ABS75486	Chen et al, 2007
		EpsN	-	-	-	-	-	*Bacillus amyloliquefaciens* subsp. *plantarum* str. FZB42	ABS75485	Chen et al, 2007
		EpsO	-	-	-	-	-	*Bacillus amyloliquefaciens* subsp. *plantarum* str. FZB42	ABS75484	Chen et al, 2007
fimbrae/pili		Smlt0706	-	-	-	-	-	*Stenotrophomonas maltophilia* K279a	NC_010943 (Smlt1290)	Crossman et al, 2008
		Smlt0707	-	-	-	-	-	*Stenotrophomonas maltophilia* K279a	NC_010943 (Smlt1290)	Crossman et al, 2008
		Smlt0708	-	-	-	-	-	*Stenotrophomonas maltophilia* K279a	NC_010943 (Smlt1290)	Crossman et al, 2008
		Smlt0709	-	-	-	-	-	*Stenotrophomonas maltophilia* K279a	NC_010943 (Smlt1290)	Crossman et al, 2008
		Smlt0732	-	-	-	-	-	*Stenotrophomonas maltophilia* K279a	NC_010943 (Smlt1290)	Crossman et al, 2008
		Smlt0733	-	-	-	-	-	*Stenotrophomonas maltophilia* K279a	NC_010943 (Smlt1290)	Crossman et al, 2008
		Smlt0734	-	-	-	-	-	*Stenotrophomonas maltophilia* K279a	NC_010943 (Smlt1290)	Crossman et al, 2008
		Smlt0735	-	-	-	-	-	*Stenotrophomonas maltophilia* K279a	NC_010943 (Smlt1290)	Crossman et al, 2008
		Smlt0736	-	-	-	-	-	*Stenotrophomonas maltophilia* K279a	NC_010943 (Smlt1290)	Crossman et al, 2008
		Smlt1508	-	-	-	-	-	*Stenotrophomonas maltophilia* K279a	NC_010943 (Smlt1290)	Crossman et al, 2008
		Smlt1509	-	-	-	-	-	*Stenotrophomonas maltophilia* K279a	NC_010943 (Smlt1290)	Crossman et al, 2008
		Smlt1510	-	-	-	-	-	*Stenotrophomonas maltophilia* K279a	NC_010943 (Smlt1290)	Crossman et al, 2008
		Smlt1511	-	-	-	-	-	*Stenotrophomonas maltophilia* K279a	NC_010943 (Smlt1290)	Crossman et al, 2008
		Smlt1512	-	-	-	-	-	*Stenotrophomonas maltophilia* K279a	NC_010943 (Smlt1290)	Crossman et al, 2008
		Smlt2867	-	-	-	-	-	*Stenotrophomonas maltophilia* K279a	NC_010943 (Smlt1290)	Crossman et al, 2008
		Smlt2868	-	-	-	-	-	*Stenotrophomonas maltophilia* K279a	NC_010943 (Smlt1290)	Crossman et al, 2008
		Smlt2869	-	-	-	-	-	*Stenotrophomonas maltophilia* K279a	NC_010943 (Smlt1290)	Crossman et al, 2008
		Smlt2870	-	-	-	-	-	*Stenotrophomonas maltophilia* K279a	NC_010943 (Smlt1290)	Crossman et al, 2008
		Smlt2871	-	-	-	-	-	*Stenotrophomonas maltophilia* K279a	NC_010943 (Smlt1290)	Crossman et al, 2008
		Smlt2872	-	-	-	-	-	*Stenotrophomonas maltophilia* K279a	NC_010943 (Smlt1290)	Crossman et al, 2008
		Smlt2873	-	-	-	-	-	*Stenotrophomonas maltophilia* K279a	NC_010943 (Smlt1290)	Crossman et al, 2008
		Smlt2874	-	-	-	-	-	*Stenotrophomonas maltophilia* K279a	NC_010943 (Smlt1290)	Crossman et al, 2008
		Smlt2875	-	-	-	-	-	*Stenotrophomonas maltophilia* K279a	NC_010943 (Smlt1290)	Crossman et al, 2008
flagella		FleN	-	-	-	-	-	*Xanthomonas campestris* pv. *campestris* str. ATCC 33913	NP_637272	Da Silva et al, 2002
		FlgA	-	-	-	-	-	*Xanthomonas campestris* pv. *campestris* str. ATCC 33913	NP_637319	Da Silva et al, 2002
		FlgB	-	-	-	-	-	*Xanthomonas campestris* pv. *campestris* str. ATCC 33913	NP_637317	Da Silva et al, 2002
		FlgB	-	-	-	-	-	*Xanthomonas campestris* pv. *campestris* str. ATCC 33913	NP_637317	Da Silva et al, 2002
		FlgC	-	-	-	-	-	*Xanthomonas campestris* pv. *campestris* str. ATCC 33913	NP_637316	Da Silva et al, 2002
		FlgC	-	-	-	-	-	*Xanthomonas campestris* pv. *campestris* str. ATCC 33913	NP_637316	Da Silva et al, 2002
		FlgD	-	-	-	-	-	*Xanthomonas campestris* pv. *campestris* str. ATCC 33913	NP_637315	Da Silva et al, 2002
		FlgE	-	-	-	-	-	*Xanthomonas campestris* pv. *campestris* str. ATCC 33913	NP_637314	Da Silva et al, 2002
		FlgF	-	-	-	-	-	*Xanthomonas campestris* pv. *campestris* str. ATCC 33913	NP_637313	Da Silva et al, 2002
		FlgG	-	-	-	-	-	*Xanthomonas campestris* pv. *campestris* str. ATCC 33913	NP_637312	Da Silva et al, 2002
		FlgG	-	-	-	-	-	*Xanthomonas campestris* pv. *campestris* str. ATCC 33913	NP_637312	Da Silva et al, 2002
		FlgH	-	-	-	-	-	*Xanthomonas campestris* pv. *campestris* str. ATCC 33913	NP_637311	Da Silva et al, 2002
		FlgI	-	-	-	-	-	*Xanthomonas campestris* pv. *campestris* str. ATCC 33913	NP_637310	Da Silva et al, 2002
		FlgJ	-	-	-	-	-	*Xanthomonas campestris* pv. *campestris* str. ATCC 33913	NP_637309	Da Silva et al, 2002
		FlgK	-	-	-	-	-	*Xanthomonas campestris* pv. *campestris* str. ATCC 33913	NP_637308	Da Silva et al, 2002
		FlgL	-	-	-	-	-	*Xanthomonas campestris* pv. *campestris* str. ATCC 33913	NP_637307	Da Silva et al, 2002
		FlgM	-	-	-	-	-	*Xanthomonas campestris* pv. *campestris* str. ATCC 33913	NP_637320	Da Silva et al, 2002
		FlhA	-	-	-	-	-	*Xanthomonas campestris* pv. *campestris* str. ATCC 33913	NP_637274	Da Silva et al, 2002
		FlhB	-	-	-	-	-	*Xanthomonas campestris* pv. *campestris* str. ATCC 33913	NP_637275	Da Silva et al, 2002
		FlhF	-	-	-	-	-	*Xanthomonas campestris* pv. *campestris* str. ATCC 33913	NP_637273	Da Silva et al, 2002
		FliA	-	-	-	-	-	*Xanthomonas campestris* pv. *campestris* str. ATCC 33913	NP_637271	Da Silva et al, 2002
		FliC	-	-	-	-	-	*Xanthomonas campestris* pv. *campestris* str. ATCC 33913	NP_637306	Da Silva et al, 2002
		FliD	-	-	-	-	-	*Xanthomonas campestris* pv. *campestris* str. ATCC 33913	NP_637305	Da Silva et al, 2002
		FliE	-	-	-	-	-	*Xanthomonas campestris* pv. *campestris* str. ATCC 33913	NP_637292	Da Silva et al, 2002
		FliF	-	-	-	-	-	*Xanthomonas campestris* pv. *campestris* str. ATCC 33913	NP_637291	Da Silva et al, 2002
		FliG	-	-	-	-	-	*Xanthomonas campestris* pv. *campestris* str. ATCC 33913	NP_637290	Da Silva et al, 2002
		FliH	-	-	-	-	-	*Xanthomonas campestris* pv. *campestris* str. ATCC 33913	NP_637289	Da Silva et al, 2002
		FliJ	-	-	-	-	-	*Xanthomonas campestris* pv. *campestris* str. ATCC 33913	NP_637288	Da Silva et al, 2002
		FliJ	-	-	-	-	-	*Xanthomonas campestris* pv. *campestris* str. ATCC 33913	NP_637287	Da Silva et al, 2002
		FliK	-	-	-	-	-	*Xanthomonas campestris* pv. *campestris* str. ATCC 33913	NP_637286	Da Silva et al, 2002
		FliL	-	-	-	-	-	*Xanthomonas campestris* pv. *campestris* str. ATCC 33913	NP_637285	Da Silva et al, 2002
		FliM	-	-	-	-	-	*Xanthomonas campestris* pv. *campestris* str. ATCC 33913	NP_637284	Da Silva et al, 2002
		FliN	-	-	-	-	-	*Xanthomonas campestris* pv. *campestris* str. ATCC 33913	NP_637283	Da Silva et al, 2002
		FliO	-	-	-	-	-	*Xanthomonas campestris* pv. *campestris* str. ATCC 33913	NP_637282	Da Silva et al, 2002
		FliP	-	-	-	-	-	*Xanthomonas campestris* pv. *campestris* str. ATCC 33913	NP_637281	Da Silva et al, 2002
		FliQ	-	-	-	-	-	*Xanthomonas campestris* pv. *campestris* str. ATCC 33913	NP_637280	Da Silva et al, 2002
		FliR	-	-	-	-	-	*Xanthomonas campestris* pv. *campestris* str. ATCC 33913	NP_637279	Da Silva et al, 2002
		FliS	-	-	-	-	-	*Xanthomonas campestris* pv. *campestris* str. ATCC 33913	NP_637304	Da Silva et al, 2002
		MotA	-	-	-	-	-	*Xanthomonas campestris* pv. *campestris* str. ATCC 33913	NP_638999	Da Silva et al, 2002
		MotB	-	-	-	-	-	*Xanthomonas campestris* pv. *campestris* str. ATCC 33913	NP_639000	Da Silva et al, 2002
Regulators										
Clp	CAP like protein	Clp	+	+	+	+	+	*Lysobacter enzymogenes* C3	AAP83141	Kobayashi et al 2005

Quorum sensing									
Rpf/DSF signalling	RpfG	+	+	+	+	+	*Stenotrophomonas maltophilia* K279a	NC_010943 (Smlt2233)	Crossman et al, 2008
	RpfC	-	-	-	+	-	*Stenotrophomonas maltophilia* K279a	NC_010943 (Smlt2234)	Crossman et al, 2008
	RpfF	-	-	-	+	-	*Stenotrophomonas maltophilia* K279a	NC_010943 (Smlt2235)	Crossman et al, 2008
	RpfB	+	+	+	+	+	*Stenotrophomonas maltophilia* K279a	NC_010943 (Smlt2236a)	Crossman et al, 2008
DF signalling	xcc-b100_4202	-	+	+	+	-	*Xanthomonas campestris* pv. *campestris* B100	AM920689 (xcc-b100_4202)	Vorholter et al, 2008
	xcc-b100_4203	-	-	-	-	-	*Xanthomonas campestris* pv. *campestris* B101	AM920689 (xcc-b100_4203)	Vorholter et al, 2008
	xcc-b100_4204	-	-	-	-	-	*Xanthomonas campestris* pv. *campestris* B102	AM920689 (xcc-b100_4204)	Vorholter et al, 2008
	xcc-b100_4205	+	+	+	+	+	*Xanthomonas campestris* pv. *campestris* B103	AM920689 (xcc-b100_4205)	Vorholter et al, 2008
	xcc-b100_4206	+	+	-	+	-	*Xanthomonas campestris* pv. *campestris* B104	AM920689 (xcc-b100_4206)	Vorholter et al, 2008
	XanB2	+	+	+	+	+	*Xanthomonas campestris* pv. *campestris* B105	AM920689 (xcc-b100_4207)	Vorholter et al, 2008
Other									
Argonaut RNAi silencing	P0025	-	-	-	-	-	*Thermus thermophilis* HB27	AAS82356	Swarts et al, 2014
	P0026	-	-	-	-	-	*Thermus thermophilis* HB27	AAS82355	Swarts et al, 2014

L. ant: *Lysobacter antibioticus*; L. cap: *L. capsici*; L. gum: *L. gummosus*; L. enz: *L. enzymogenes*.

Table S13. NRPS/PKS clusters identified in the *Lysobacter* genome sequences using AntiSmash and subsequent domain and prediction analysis. Per cluster, the top row describes the GeneID number, the middle row describes the domains identified by PFAM analysis and bottom row describes the amino acid predicted to be incorporated based on NRPS/PKS, NRPS predictor2 and phylogenetic analysis.

L. ant ATCC29479

cluster 1	1048	1047	1046
	C1	T	A
			Dhb

cluster 2	1716	1715	1712		1710			1709		1708	1707		1706			
#14 aa			1	2	3	4	5	6	7	8	9	10	11	12	13	14
	A	T	CAT	CAT	CAT	CAT	CAT	CAT	CAT	CAT	CAT	CAT	CAT	CAT	CAT	CAT
	?		Ala	Ala	?	Arg	Asp	Asp	Asp	Phe/Asn	Thr	Asp	Pro	?	Ala	Ala

cluster 3	2680	2678	2677
	C	A	A
		?	?

cluster 4	2957							2956
#8 aa	1	2	3	4	5	6	7	8
	C1AT	CAT	CCAT	CAT	CAmT	CCAT	CAT	CAT
	Val	Orn	Ser	Gly	mPhe	Leu	Orn	Gly

cluster 5	3239			
#3 aa	1	2	3	
	CAT	CAT	CAT	TE
	Val	Phe	Thr	

cluster 6	3545				
# 4 aa	1	2	3	4	
	CAT	CAT	CAT	CAT	TE
	Gly	Val	Trp	?	

cluster 7	4135	
#1 aa	1	
	C1AT	TE
	Gly	

L. ant 76

cluster 1	240				
#4 aa	1	2	3	4	
	CAT	CAT	CAT	CAT	TE
	Ser	Val	?	Thr	

cluster 2	319	
#1 aa	1	
	C1AT	TE
	Gly	

cluster 3	606		605	603
	A	A	A	C
	?	?	?	

cluster 4	2116	2117	2120		2122			2123		2124	2125		2126				
#14 aa			1	2	3	4	5	6	7	8	9	10	11	12	13	14	
	A	T	CAT	CAT	CAT	CCAT	CAmT	CAT	CAT	CAT	CAT	CAT	CAT	CAT	CAT	CAT	TE
	?		Ala	Ala	?	Arg	Asp	Asp	Asp	Asn	Thr	Asp	Pro	?	Ala	Asp	

cluster 5	2594	2595	2596
	A	T	C1
	?		

cluster 6	2691							2692				
#11 aa	1	2	3	4	5	6	7	8	9	10	11	
	C1AT	CAT	CCAT	CAT	CAmT	CCAT	CAT C	CCAT	CAT	CCAT	CCAT	TE
	Val	Orn	Ser	Gly	mPhe	Leu	Orn	Ser	Val	Trp	?	

cluster 7	3260	3263	
#1 aa		1	
	A	CAT	TE
	?	Cys	

L. cap 55

cluster 1				2005			
#1 aa						1	
	KS	AT	PS	K	T	CAT	TE
dihydromaltophilin						Orn	

cluster 2	2689	2691						2694						2697		2699			
#14 aa		1	2	3	4	5	6					7	8	9	10	11	12	13	14
	ATC	AT	CAT	CAmT	CCAT	CAT	CAT	KS	AT	KR	T	CAT	CAmT	CAT	CAmT	CAT	CAT	CAT	CAT C
	?	Orn	?	?	Thr	Cys	?					Thr	Cys	?	mSer	Ser	Cys	Ser	Orn

cluster 3				2722						2723			
# 12 aa	1	2	3	4	5	6	7	8	9	10	11	12	
	C1AT	CAT	CCAT	CAT	CAmT	CCAT	CAT C	CAT	CAT	CCAT	CCAT	CCAmT	TE
WAP-8294	Ser	Asn	Ser	Gly	mPhe	Leu	Orn	Gly	Asn	Trp	Orn	mVal	

cluster 4	2738	2737	2736
	A	T	C
	Val		

cluster 5	4975	4976	4977
#3 aa	1	2	3
	CAT	CAT	CAT
	Gly	Gly	?

cluster 6	5004	
#1 aa	1	
	C1AT	TE
	Gly	

L. gum 3.2.11

cluster 1	2475				2476								
#11 aa	AT	CAT	CAT	CAT	CAT	CAT	CAT	CCAT	CAT	CAT	CAT	TE	TE
lysobactin	Leu	Leu	Phe	Leu	Leu	Arg	Ile	Thr	Gly	Asn	Ser		

cluster 2				3209			
#1 aa						1	
	KS	AT	PS	K	T	C1AT	TE
dihydromaltophilin						Orn	

cluster 3	3694	3693	3692		3691			3690			3689				
# 12 aa			1	2	3	4	5	6	7	8	9	10	11	12	
	A	T	CAT	CAT	CAT	CAT	CAT	CAT	CAT	CAT	CAT	CAT	CAT	CAT	TE
	Leu		Arg	Ser	Asp	Asp	Thr	Ser	Asp	Asp	Arg	Ser	Thr	Pro	

cluster 4	4391				
#5 aa	1	2	3	4	5
	AT	CAT	CAT	CAT	CAT
	Ala	Ala	Arg	?	Ala

cluster 5	4985	
#1 aa	1	
	C1AT	TE
	Gly	

L. enz C3

cluster 1	0544	0545	0547
#3 aa	1	2	3
	CAT	CAT	CAT
	Gly	Gly	Phe

cluster 2	1848		1851
#2 aa	1		2
	CAT	TE	A
	Pro?		Phe?

cluster 3	2099											2100	2101	2102	2103			2104				
#6 aa																		1	2	3	4	5	6
	KS	PS	KR	T	KS	PS	T	KS	PS	KR	T	E	At	T	At	At	At	CAT	CAT	CAT	CAT	CAT	CATC
																		Tyr	Ser	Phe	Phe/Ser	Ser	Phe/Ser

cluster 4	2493												2494														2495						
#4 aa?		1	2	3									4																				
	A	mAT	CAT	CAT	KS	KR	T	KS	KR	T	KS	T	CAT	KS	PS	KR	mTT	KS	PS	KR	T	KS	TT	KS	KR	T	KS	KR	T	KS	PS	KR	mTTT
	Thr	?	Tyr	Gly									?																				

cluster 5	2577		2558			2559			2560		
#10 aa	1	2	3	4	5	6	7	8	9	10	
	CATT	CAT	CCAT	CCAT	CCAT	CAT	CAT	CAT	CAT	CCAT	TE
	Asp	Phe?	Arg	Asp	Thr	?	Pro	?	Asp	Thr	

cluster 6	2621	2623
	C	A
		?

cluster 7	2957				2958		2959	
#6 aa	1	2	3		4	5	6	
	AT	CAT	CAT	NAD	AT	CAT	CAT	NAD
	Ala/Arg	Pro	Ala/Arg		Pro	Ala/Arg	Pro	

cluster 8				3215			
#1 aa						1	
	KS	AT	PS	K	T	C1AT	TE
dihydromaltophilin						Orn	

cluster 9				3675						3676			
#12 aa	1	2	3	4	5	6	7	8	9	10	11	12	
	C1AT	CAT	CCAT	CAT	CAmT	CCAT	CATC	CAT	CAT	CCAT	CCAT	CCAmT	TE
WAP-8294	Ser	Asp	Ser	Gly	mPhe	Leu	Orn	Ser	Asn	Trp	Arg	mVal	

cluster 10	4984	
#1 aa	1	
	CAT	NAD
	Phe	

cluster 11	3997	
#1 aa	1	
	C1AT	TE
	Gly	

Note: C: condensation domain; C1: Condensation domain predicted to be the start of the peptide synthesis, based on phylogenetic analysis; A: adenylation domain; T: thiolation domain; E: epimerase domain; TE: thioesterase domain; M: methyltransferase domain; KS: ketoacyl synthase domain; KR: KR domain; PS: polyketide synthase dehydratase domain; At: acyltransferase domain; NAD: NAD-binding domain. ? indicates prediction was inconclusive.

Table S14. Genetic organization of flagellar apparatus and type IV pilus in *Lysobacter* genomes.

		L. ant ATCC29479	*L. ant* 76	*L. cap* 55	*L. gum* 3.2.11	*L. enz* C3
Flagellar apparatus						
	TCP fam protein	-	-	1457	4578	4623
	fliQ	-	-	1458	4579	4624
	fliR	-	-	1459	4580	4625
	FlhB	-	-	1460	4581	4626
	FlhA	-	-	1461	4582	4627
	flgF	-	-	1462	4583	4628
	flgG	-	-	1463	4584	4629
	flgA-like	-	-	1464	4585	4631
	flgH	-	-	1465	4586	4632
	flgI	-	-	1466	4587	4633
	flgB	-	-	1467	4588	4634
	flgC	-	-	1468	4589	4635
	fliE	-	-	1469	4590	4636
	fliF	-	-	1470	4591	4637
	fliG	-	-	1471	4592	4638
	fliH-like	-	-	1472	4593	4639
	fliI	-	-	1473	4594	4640
	flgD	-	-	1476	4597	4643
	flgE	-	-	1477	4598	4644
	fliM	-	-	1478	4599	4645
	fliN	-	-	1479	4600	4646
	fliO	-	-	1480	4601	4647
	fliP	-	-	1481	4602	4648
	sigma-70	-	-	1482	4603	4649
	TCP fam pro	-	-	-	4604	4650
Type IV pilus						
	pilS	2003	1483	4254	4044	4112
	pilR	2004	2551	4255	4045	4113
	pilA	2005/2006	1197	4256	4046	4114
cluster I	*pilB*	2017	1188	4257	4061	4116
	pilC	2018	1187	4258	4062	4117
	pilD	2019	1186	4259	4063	4118
	pilG	4690	4075	4146	3938	3962
	pilH	4689	4074	4145	3937	3961
cluster II	*pilI*	4688	4073	4144	3936	3960
	pilJ	4687	4072	4143	3935	3959
	pilL	4686	4071	4142	3934	3958
	pilK	4685	4070	4140	3933	3957
	pilM	4162	4156	1037	0974	932
	pilN	4161	4155	1038	0975	933
cluster III	*pilO*	4160	4154	1039	0976	934
	pilP	4159	4153	1040	0977	935
	pilQ	4158	4152	1041	0978	936
	pilE	4261	1741	1583	3629	3575
	pilY1	4262	1740	1582	3630	3576
	pilX	4263	1739	1581	3632	3577

cluster IVa	*pilW*	4264	1738	1580	3633	3578
	pilV	4265	1737	1579	3634	3579
	pre-pilin	4266	1736	1578	3637	3580
	pilE			1656	3548	3621
	pilY1			1655	3549	3622
	pilX			1654	3550	3623
cluster IVb	*pilW*			1653	3551	3624
	pilV			1652	3552	3625
	pre-pilin			1651	3553	3626
cluster V	*pilT*	0732	1544	1295	1211	1163
	pilU	0731	1545	1296	1212	1164
	pilZ	4190	4183	1006	0947	0901
	pilZ_2	4892	3493	3441	1744	1619

Note: Genes are indicated by genome identification number. For Type IV pilus: entire pathways are located in five different gene clusters (I-V), with homologues of pilZ distributed throughout all five genomes.
L. ant: *Lysobacter antibioticus*; L. cap: *L. capsic*i; L. gum: *L. gummosus*; L. enz: *L. enzymogenes*.

Table S15. Genetic organization of the General Secretory Pathway and twin-arginine translocation pathway in the *Lysobacter* genomes.

		L. ant ATCC29479	*L. ant* 76	*L. cap* 55	*L. gum* 3.2.11	*L. enz* C3
General Secretory Pathway						
cluster I	*gspD*	4300	1135	4315	4115	4172
	gspN	4301	1134	4316	4116	4173
	gspM	4302	1133	4317	4117	4174
	gspL	4303	1132	4318	4118	4175
	gspK	4304	1131	4319	4119	4176
	gspJ	4305	1130	4320	4120	4177
	gspI	4306	1129	4321	4121	4178
	gspH	4307	1128	4322	4122	4179
	gspG	4308	1127	4323	4123	4180
	gspF	4309	1126	4324	4124	4181
	gspE	4310	1125	4325	4125	4183
	yajC	3222	1804	1725	3455	3514
cluster II	*secD*	3221	1805	1726	3454	3513
	secF	3220	1806	1727	3453	3512
	secG	202	2988	3018	2197	2158
	secY	3383	3908	3934	3743	3770
others	*secE*	3417	3943	3966	3777	3803
	secA	2202	1183	4262	4066	4122
	secB	4996	4722	321	340	5031
Twin-arginine translocation pathway						
	tatA/E	1226	141	5186	5166	122
	tatB	1225	142	5185	5166	123
	tatC	1224	143	5184	5166	124
	RDD fam	1223	144	5183	5166	125
	tatD_1	1651	2184	2161	5166	3058
	tatD_2	1395	5183	5713	5166	5364

Note: All genes are indicated by locus ID number. Gene positions and transcriptional directions within the genome are presented from top to bottom in the table.
L. ant: *Lysobacter antibioticus*; L. cap: *L. capsic*i; L. gum: *L. gummosus*; L. enz: *L. enzymogenes*.
